# Supplementary material for: Influence of Selected Hypromellose Functionality-Related Characteristics and Soluble/Insoluble Filler Ratio on Carvedilol Release from Matrix Tablets
Source: Pharmaceutics. 2025 Oct 21;17(10):1358. doi: 10.3390/pharmaceutics17101358 (PMC12566823; doi:10.3390/pharmaceutics17101358)
Supplement: Supplementary file 1 [file pharmaceutics-17-01358-s001.zip › Supplementary Materials_Tables & Figures.pdf]

Article: **Influence of Selected Hypromellose Functionality-related Characteristics and Soluble/Insoluble Filler Ratio on Carvedilol Release from Matrix Tablets**

Supplementary Materials: Tables & Figures

Table S1. HPMC Producer's (Shin-Etsu) analyses results of QbD samples in the QbD sample kit.

Table S2. Theoretical factor levels in the DoE.

Table S3. Theoretical factor levels' values in the DoE (based on the most extreme values of HPMC FRCs in the QbD samples – see Table S1).

Table S4. Actual factor levels' values in the DoE (based on theoretical weighed amounts of both fillers and up to 3 HPMC QbD samples per experiment).

Table S5. Actual factor levels in the DoE (based on theoretical weighed amounts of both fillers and up to 3 HPMC QbD samples per experiment).

Table S6. Experimental carvedilol release results shown as mean release (%) ± 1 SD. Data is shown for experiments 1 to 9[1].

Table S7. Experimental carvedilol release results shown as mean release (%) ± 1 SD. Data is shown for experiments 10 to 18[1].

Table S8. Experimental carvedilol release results shown as mean release (%) ± 1 SD. Data is shown for experiments 19 to 27[1].

Table S9. R^2 and R^2_{pred} for full RSM MLR models and MLR models generated via stepwise regression procedures for predicting mean % of carvedilol release. Range of models used in interpretation of main effects and interaction effects of factors in terms of their influence on carvedilol release is bolded.

Table S10. R^2 and R^2_{pred} of full RSM MLR models, Basic MLR models and selected Optimized MLR models generated via stepwise regression procedures for predicting mean % of carvedilol release. Range of models used in interpretation of main effects and interaction effects of factors in terms of their influence on carvedilol release is bolded.

Table S11. Basic MLR models for prediction of mean % of carvedilol release, summary of model terms (Lac, HPMC_Visc, HPMC_HP, HPMC_PS): intercepts, regression coefficients, p-values of regression coefficients, and contribution %. Range of models used in interpretation of main effects and interaction effects of factors in terms of their influence on carvedilol release is bolded.

Table S12. Optimized MLR models for prediction of mean % of carvedilol release, summary of model terms: intercepts and regression coefficients. Range of models used in interpretation of main effects and interaction effects of factors in terms of their influence on carvedilol release is bolded.

Table S13. Optimized MLR models for prediction of mean % of carvedilol release, summary of model terms: p-values of model terms (p-values ≤ 0.05 are coloured dark red). Range of models

used in interpretation of main effects and interaction effects of factors in terms of their influence on carvedilol release is bolded.

Table S14. Optimized MLR models for prediction of mean % of carvedilol release, summary of model terms: contribution % of model terms. Range of models used in interpretation of main effects and interaction effects of factors in terms of their influence on carvedilol release is bolded.

Table S15. R^2 and R^2_{pred} for full RSM MLR models and MLR models generated via stepwise regression procedures for predicting SD of carvedilol release. Range of models used in interpretation of main effects and interaction effects of factors in terms of their influence on carvedilol release is bolded. In cases where no stepwise MLR model could be created by Minitab®, "N/A" is shown in the table.

Table S16. R^2 and R^2_{pred} of full RSM MLR models and selected Optimized MLR models generated via stepwise regression procedures for predicting SD of carvedilol release. Range of models used in interpretation of main effects and interaction effects of factors in terms of their influence on carvedilol release is bolded.

Table S17. Optimized MLR models for prediction of SD of carvedilol release, summary of model terms: intercepts and regression coefficients. Range of models used in interpretation of main effects and interaction effects of factors in terms of their influence on carvedilol release is bolded.

Table S18. Optimized MLR models for prediction of SD of carvedilol release, summary of model terms: p-values of model terms (p-values ≤ 0.05 are coloured dark red). Range of models used in interpretation of main effects and interaction effects of factors in terms of their influence on carvedilol release is bolded.

Table S19. Optimized MLR models for prediction of SD of carvedilol release, summary of model terms: contribution % of model terms. Range of models used in interpretation of main effects and interaction effects of factors in terms of their influence on carvedilol release is bolded.

Figure S1. Main effects plot for mean % of carvedilol release using a Basic MLR model at t = 0.17 h (10 min).

Figure S2. Main effects plot for mean % of carvedilol release using a Basic MLR model at t = 0.33 h (20 min).

Figure S3. Main effects plot for mean % of carvedilol release using a Basic MLR model at t = 0.5 h (30 min).

Figure S4. Main effects plot for mean % of carvedilol release using a Basic MLR model at t = 0.75 h (45 min).

Figure S5. Main effects plot for mean % of carvedilol release using a Basic MLR model at t = 1 h (60 min).

Figure S6. Main effects plot for mean % of carvedilol release using a Basic MLR model at t = 1.5 h (90 min).

Figure S7. Main effects plot for mean % of carvedilol release using a Basic MLR model at t = 2 h (120 min).

Figure S8. Main effects plot for mean % of carvedilol release using a Basic MLR model at t = 2.5 h (150 min).

Figure S9. Main effects plot for mean % of carvedilol release using a Basic MLR model at t = 3 h (180 min).

Figure S10. Main effects plot for mean % of carvedilol release using a Basic MLR model at t = 3.5 h (210 min).

Figure S11. Main effects plot for mean % of carvedilol release using a Basic MLR model at t = 4 h (240 min).

Figure S12. Main effects plot for mean % of carvedilol release using a Basic MLR model at t = 4.5 h (270 min).

Figure S13. Main effects plot for mean % of carvedilol release using a Basic MLR model at t = 5 h (300 min).

Figure S14. Main effects plot for mean % of carvedilol release using a Basic MLR model at t = 5.5 h (330 min).

Figure S15. Main effects plot for mean % of carvedilol release using a Basic MLR model at t = 6 h (360 min).

Figure S16. Main effects plot for mean % of carvedilol release using a Basic MLR model at t = 7 h (420 min).

Figure S17. Main effects plot for mean % of carvedilol release using a Basic MLR model at t = 8 h (480 min).

Figure S18. Main effects plot for mean % of carvedilol release using a Basic MLR model at t = 9 h (540 min).

Figure S19. Main effects plot for mean % of carvedilol release using a Basic MLR model at t = 10 h (600 min).

Figure S20. Main effects plot for mean % of carvedilol release using a Basic MLR model at t = 11 h (660 min).

Figure S21. Main effects plot for mean % of carvedilol release using a Basic MLR model at t = 12 h (720 min).

Figure S22. Main effects plot for mean % of carvedilol release using a Basic MLR model at t = 13 h (780 min).

Figure S23. Main effects plot for mean % of carvedilol release using a Basic MLR model at t = 14 h (840 min).

Figure S24. Main effects plot for mean % of carvedilol release using a Basic MLR model at t = 15 h (900 min).

Figure S25. Main effects plot for mean % of carvedilol release using a Basic MLR model at t = 16 h (960 min).

Figure S26. Main effects plot for mean % of carvedilol release using a Basic MLR model at t = 17 h (1020 min).

Figure S27. Main effects plot for mean % of carvedilol release using a Basic MLR model at t = 18 h (1080 min).

Figure S28. Main effects plot for mean % of carvedilol release using a Basic MLR model at t = 19 h (1140 min).

Figure S29. Main effects plot for mean % of carvedilol release using a Basic MLR model at t = 20 h (1200 min).

Figure S30. Main effects plot for mean % of carvedilol release using a Basic MLR model at t = 21 h (1260 min).

Figure S31. Main effects plot for mean % of carvedilol release using a Basic MLR model at t = 22 h (1320 min).

Figure S32. Main effects plot for mean % of carvedilol release using a Basic MLR model at t = 23 h (1380 min).

Figure S33. Main effects plot for mean % of carvedilol release using a Basic MLR model at t = 24 h (1440 min).

Figure S34. Main effects plot (a) and Interaction plot for mean % of carvedilol release using an Optimized MLR model at t = 0.17 h (10 min).

Figure S35. Main effects plot (a) and Interaction plot for mean % of carvedilol release using an Optimized MLR model at t = 0.33 h (20 min).

Figure S36. Main effects plot (a) and Interaction plot for mean % of carvedilol release using an Optimized MLR model at t = 0.5 h (30 min).

Figure S37. Main effects plot (a) and Interaction plot for mean % of carvedilol release using an Optimized MLR model at t = 0.75 h (45 min).

Figure S38. Main effects plot (a) and Interaction plot for mean % of carvedilol release using an Optimized MLR model at t = 1 h (60 min).

Figure S39. Main effects plot (a) and Interaction plot for mean % of carvedilol release using an Optimized MLR model at t = 1.5 h (90 min).

Figure S40. Main effects plot (a) and Interaction plot for mean % of carvedilol release using an Optimized MLR model at t = 2 h (120 min).

Figure S41. Main effects plot (a) and Interaction plot for mean % of carvedilol release using an Optimized MLR model at t = 2.5 h (150 min).

Figure S42. Main effects plot (a) and Interaction plot for mean % of carvedilol release using an Optimized MLR model at t = 3 h (180 min).

Figure S43. Main effects plot (a) and Interaction plot for mean % of carvedilol release using an Optimized MLR model at t = 3.5 h (210 min).

Figure S44. Main effects plot (a) and Interaction plot for mean % of carvedilol release using an Optimized MLR model at t = 4 h (240 min).

Figure S45. Main effects plot (a) and Interaction plot for mean % of carvedilol release using an Optimized MLR model at t = 4.5 h (270 min).

Figure S46. Main effects plot (a) and Interaction plot for mean % of carvedilol release using an Optimized MLR model at t = 5 h (300 min).

Figure S47. Main effects plot (a) and Interaction plot for mean % of carvedilol release using an Optimized MLR model at t = 5.5 h (330 min).

Figure S48. Main effects plot (a) and Interaction plot for mean % of carvedilol release using an Optimized MLR model at t = 6 h (360 min).

Figure S49. Main effects plot (a) and Interaction plot for mean % of carvedilol release using an Optimized MLR model at t = 7 h (420 min).

Figure S50. Main effects plot (a) and Interaction plot for mean % of carvedilol release using an Optimized MLR model at t = 8 h (480 min).

Figure S51. Main effects plot (a) and Interaction plot for mean % of carvedilol release using an Optimized MLR model at t = 9 h (540 min).

Figure S52. Main effects plot (a) and Interaction plot for mean % of carvedilol release using an Optimized MLR model at t = 10 h (600 min).

Figure S53. Main effects plot (a) and Interaction plot for mean % of carvedilol release using an Optimized MLR model at t = 11 h (660 min).

Figure S54. Main effects plot (a) and Interaction plot for mean % of carvedilol release using an Optimized MLR model at t = 12 h (720 min).

Figure S55. Main effects plot (a) and Interaction plot for mean % of carvedilol release using an Optimized MLR model at t = 13 h (780 min).

Figure S56. Main effects plot (a) and Interaction plot for mean % of carvedilol release using an Optimized MLR model at t = 14 h (840 min).

Figure S57. Main effects plot (a) and Interaction plot for mean % of carvedilol release using an Optimized MLR model at t = 15 h (900 min).

Figure S58. Main effects plot (a) and Interaction plot for mean % of carvedilol release using an Optimized MLR model at t = 16 h (960 min).

Figure S59. Main effects plot (a) and Interaction plot for mean % of carvedilol release using an Optimized MLR model at t = 17 h (1020 min).

Figure S60. Main effects plot (a) and Interaction plot for mean % of carvedilol release using an Optimized MLR model at t = 18 h (1080 min).

Figure S61. Main effects plot (a) and Interaction plot for mean % of carvedilol release using an Optimized MLR model at t = 19 h (1140 min).

Figure S62. Main effects plot (a) and Interaction plot for mean % of carvedilol release using an Optimized MLR model at t = 20 h (1200 min).

Figure S63. Main effects plot (a) and Interaction plot for mean % of carvedilol release using an Optimized MLR model at t = 21 h (1260 min).

Figure S64. Main effects plot (a) and Interaction plot for mean % of carvedilol release using an Optimized MLR model at t = 22 h (1320 min).

Figure S65. Main effects plot (a) and Interaction plot for mean % of carvedilol release using an Optimized MLR model at t = 23 h (1380 min).

Figure S66. Main effects plot (a) and Interaction plot for mean % of carvedilol release using an Optimized MLR model at t = 24 h (1440 min).

Figure S67. Main effects plot (a) and Interaction plot for SD of carvedilol release using an Optimized MLR model at t = 0.17 h (10 min).

Figure S68. Main effects plot (a) and Interaction plot for SD of carvedilol release using an Optimized MLR model at t = 0.33 h (20 min).

Figure S69. Main effects plot (a) and Interaction plot for SD of carvedilol release using an Optimized MLR model at t = 0.5 h (30 min).

Figure S70. Main effects plot (a) and Interaction plot for SD of carvedilol release using an Optimized MLR model at t = 0.75 h (45 min).

Figure S71. Main effects plot (a) and Interaction plot for SD of carvedilol release using an Optimized MLR model at t = 1 h (60 min).

Figure S72. Main effects plot (a) and Interaction plot for SD of carvedilol release using an Optimized MLR model at t = 1.5 h (90 min).

Figure S73. Main effects plot for SD of carvedilol release using an Optimized MLR model at t = 2 h (120 min).

Figure S74. Main effects plot for SD of carvedilol release using an Optimized MLR model at t = 2.5 h (150 min).

Figure S75. Main effects plot for SD of carvedilol release using an Optimized MLR model at t = 3 h (180 min).

Figure S76. Main effects plot (a) and Interaction plot for SD of carvedilol release using an Optimized MLR model at t = 3.5 h (210 min).

Figure S77. Main effects plot (a) and Interaction plot for SD of carvedilol release using an Optimized MLR model at t = 4 h (240 min).

Figure S78. Main effects plot (a) and Interaction plot for SD of carvedilol release using an Optimized MLR model at t = 4.5 h (270 min).

Figure S79. Main effects plot (a) and Interaction plot for SD of carvedilol release using an Optimized MLR model at t = 5 h (300 min).

Figure S80. Main effects plot (a) and Interaction plot for SD of carvedilol release using an Optimized MLR model at t = 5.5 h (330 min).

Figure S81. Main effects plot (a) and Interaction plot for SD of carvedilol release using an Optimized MLR model at t = 6 h (360 min).

Figure S82. Main effects plot (a) and Interaction plot for SD of carvedilol release using an Optimized MLR model at t = 7 h (420 min).

Figure S83. Main effects plot (a) and Interaction plot for SD of carvedilol release using an Optimized MLR model at t = 8 h (480 min).

Figure S84. Main effects plot (a) and Interaction plot for SD of carvedilol release using an Optimized MLR model at t = 9 h (540 min).

Figure S85. Main effects plot (a) and Interaction plot for SD of carvedilol release using an Optimized MLR model at t = 10 h (600 min).

Figure S86. Main effects plot (a) and Interaction plot for SD of carvedilol release using an Optimized MLR model at t = 11 h (660 min).

Figure S87. Main effects plot (a) and Interaction plot for SD of carvedilol release using an Optimized MLR model at t = 12 h (720 min).

Figure S88. Main effects plot (a) and Interaction plot for SD of carvedilol release using an Optimized MLR model at t = 13 h (780 min).

Figure S89. Main effects plot (a) and Interaction plot for SD of carvedilol release using an Optimized MLR model at t = 14 h (840 min).

Figure S90. Main effects plot for SD of carvedilol release using an Optimized MLR model at t = 15 h (900 min).

Figure S91. Main effects plot for SD of carvedilol release using an Optimized MLR model at t = 16 h (960 min).

Figure S92. Main effects plot (a) and Interaction plot for SD of carvedilol release using an Optimized MLR model at t = 17 h (1020 min).

Figure S93. Main effects plot for SD of carvedilol release using an Optimized MLR model at t = 18 h (1080 min).

Figure S94. Main effects plot for SD of carvedilol release using an Optimized MLR model at t = 19 h (1140 min).

Figure S95. Main effects plot for SD of carvedilol release using an Optimized MLR model at t = 20 h (1200 min).

Figure S96. Main effects plot for SD of carvedilol release using an Optimized MLR model at t = 21 h (1260 min).

Figure S97. Main effects plot (a) and Interaction plot for SD of carvedilol release using an Optimized MLR model at t = 22 h (1320 min).

Figure S98. Main effects plot for SD of carvedilol release using an Optimized MLR model at t = 23 h (1380 min).

Figure S99. Main effects plot for SD of carvedilol release using an Optimized MLR model at t = 24 h (1440 min).

Table S20. In-process control (IPC) results for the tablet mass, measured on 20 tablets per experiment using a representative sample. Results are shown for the mean, minimum, and maximum tablet mass, where a more reddish colour tone represents a higher mass, a more bluish tone represents a lower mass, and a white tone indicates approximately the mean of all results. The range, standard deviation, and relative standard deviation of tablet mass are also shown, where longer bars indicate larger values and shorter bars indicate smaller ones. “DoE Id” data in the legend is comprised of theoretical levels (-2, -1, 0, 1, 2) of factors A (Lac), B (HPMC_Visc), C (HPMC_HP), and D (HPMC_PS) used in each experiment, respectively.

Table S21. In-process control (IPC) results for the tablet hardness, measured on 10 tablets per experiment using a representative sample. Results are shown for the mean, minimum, and maximum tablet hardness, where a more reddish colour tone represents a higher hardness, a more bluish tone represents a lower hardness, and a white tone indicates approximately the mean of all results. The range, standard deviation, and relative standard deviation of tablet hardness are also shown, where longer bars indicate larger values and shorter bars indicate smaller ones. “DoE Id” data in the legend is comprised of theoretical levels (-2, -1, 0, 1, 2) of factors A (Lac), B (HPMC_Visc), C (HPMC_HP), and D (HPMC_PS) used in each experiment, respectively.

Table S22. In-process control (IPC) results for the tablet thickness, measured on 10 tablets per experiment using a representative sample. Results are shown for the mean, minimum, and maximum tablet thickness, where a more reddish colour tone represents a higher thickness, a more bluish tone represents a lower thickness, and a white tone indicates approximately the mean of all results. The range, standard deviation, and relative standard deviation of tablet thickness are also shown, where longer bars indicate larger values and shorter bars indicate smaller ones. “DoE Id” data in the legend is comprised of theoretical levels (-2, -1, 0, 1, 2) of factors A (Lac), B (HPMC_Visc), C (HPMC_HP), and D (HPMC_PS) used in each experiment, respectively.

Table S23. In-process control (IPC) results for tablet friability, measured on approximately 6.5-gram samples of tablets per experiment using a representative sample. Longer bars indicate larger values, while shorter bars indicate smaller ones. The “DoE ID” data in the legend comprise the theoretical levels (−2, −1, 0, 1, 2) of factors A (Lac), B (HPMC_Visc), C (HPMC_HP), and D (HPMC_PS) used in each experiment, respectively.

Table S1. HPMC Producer's (Shin-Etsu) analyses results of QbD samples in the QbD sample kit.

HPMC type: 90SH-15000SR (2208, K15M)

Id	Id - short	Producer QbD Batch	QbD Batch Procucer's Comment	Viscosity (mPas) (specification: 11,250 - 21,000)	Methoxy content (%) (specification: 19.0 - 24.0)	Hydroxypropoxy content (%) (specification: 8.5 - 10.5)	Mean particle size* (µm) (specification: 50 - 80)
Center Point	C	9015ST01	Standard	13,000	23.2	9.8	70.4
↓ Viscosity	↓V	9015LV01	Low Viscosity	7,700	23.2	9.4	75.7
↑ Viscosity	↑V	9015HV01	High Viscosity	19,300	23.1	9.3	65.4
↓ HP Substitution	↓ HP	9015LS01	Low Substitution	11,900	22.9	7.9	65.4
↑HP Substitution	↑HP	9015HS01	High Substitution	13,300	22.8	10.7	68.6
↓ Particle Size	↓PS	9015FP01	Fine Particle	12,500	23.2	9.4	62.3
↑ Particle Size	↑PS	9015CP01	Coarse Particle	11,700	22.9	9.3	78.3

*Laser diffraction method

Table S2. Theoretical factor levels in the DoE.

Standard Order (as provided by the software)	Run Order (randomized)	Std Order (centre points positioned at start, middle and end of DoE)	Blocks	**Factor A (Lac):** Fraction of FlowLac® 100 in filler composition (FlowLac® 100 + Avicel® PH-102)	**Factor B (HPMC_Visc):** HPMC K15M Viscosity (m·Pas)	**Factor C (HPMC_HP):** HPMc K15M Hydroxypropoxy content (%)	**Factor D (HPMC_PS):** HPMC K15M Mean particle size (µm)
25	1	1	1	0	0	0	0
1	24	2	1	-1	-1	-1	-1
2	6	3	1	1	-1	-1	-1
3	3	4	1	-1	1	-1	-1
4	12	5	1	1	1	-1	-1
5	25	6	1	-1	-1	1	-1
6	17	7	1	1	-1	1	-1
7	23	8	1	-1	1	1	-1
8	26	9	1	1	1	1	-1
9	11	10	1	-1	-1	-1	1
10	9	11	1	1	-1	-1	1
11	19	12	1	-1	1	-1	1
12	7	13	1	1	1	-1	1
26	14	14	1	0	0	0	0
13	15	15	1	-1	-1	1	1
14	16	16	1	1	-1	1	1
15	4	17	1	-1	1	1	1
16	10	18	1	1	1	1	1
17	22	19	1	-2	0	0	0
18	8	20	1	2	0	0	0
19	2	21	1	0	-2	0	0
20	5	22	1	0	2	0	0
21	20	23	1	0	0	-2	0
22	21	24	1	0	0	2	0
23	18	25	1	0	0	0	-2
24	13	26	1	0	0	0	2
27	27	27	1	0	0	0	0

Table S3. Theoretical factor levels' values in the DoE (based on the most extreme values of HPMC FRCs in the QbD samples – see Table S1).

Standard Order (as provided by the software)	Run Order (randomized)	Std Order (centre points positioned at start, middle and end of DoE)	Blocks	**Factor A (Lac):** Fraction of FlowLac® 100 in filler composition (FlowLac® 100 + Avicel® PH-102)	**Factor B (HPMC_Visc):** HPMC K15M Viscosity (m·Pas)	**Factor C (HPMC_HP):** HPMc K15M Hydroxypropoxy content (%)	**Factor D (HPMC_PS):** HPMC K15M Mean particle size (µm)
25	1	1	1	0.500	13500	9.3	70.3
1	24	2	1	0.375	10600	8.6	66.3
2	6	3	1	0.625	10600	8.6	66.3
3	3	4	1	0.375	16400	8.6	66.3
4	12	5	1	0.625	16400	8.6	66.3
5	25	6	1	0.375	10600	10	66.3
6	17	7	1	0.625	10600	10	66.3
7	23	8	1	0.375	16400	10	66.3
8	26	9	1	0.625	16400	10	66.3
9	11	10	1	0.375	10600	8.6	74.3
10	9	11	1	0.625	10600	8.6	74.3
11	19	12	1	0.375	16400	8.6	74.3
12	7	13	1	0.625	16400	8.6	74.3
26	14	14	1	0.500	13500	9.3	70.3
13	15	15	1	0.375	10600	10	74.3
14	16	16	1	0.625	10600	10	74.3
15	4	17	1	0.375	16400	10	74.3
16	10	18	1	0.625	16400	10	74.3
17	22	19	1	0.250	13500	9.3	70.3
18	8	20	1	0.750	13500	9.3	70.3
19	2	21	1	0.500	7700	9.3	70.3
20	5	22	1	0.500	19300	9.3	70.3
21	20	23	1	0.500	13500	7.9	70.3
22	21	24	1	0.500	13500	10.7	70.3
23	18	25	1	0.500	13500	9.3	62.3
24	13	26	1	0.500	13500	9.3	78.3
27	27	27	1	0.500	13500	9.3	70.3

Table S4. Actual factor levels' values in the DoE (based on theoretical weighed amounts of both fillers and up to 3 HPMC QbD samples per experiment).

Standard Order (as provided by the software)	Run Order (randomized)	Std Order (centre points positioned at start, middle and end of DoE)	Blocks	**Factor A (Lac):** Fraction of FlowLac® 100 in filler composition (FlowLac® 100 + Avicel® PH-102)	**Factor B (HPMC_Visc):** HPMC K15M Viscosity (m·Pas)	**Factor C (HPMC_HP):** HPMc K15M Hydroxypropoxy content (%)	**Factor D (HPMC_PS):** HPMC K15M Mean particle size (µm)
25	1	1	1	0.500	12780	9.4	69.4
1	24	2	1	0.375	10741	8.7	67.8
2	6	3	1	0.625	10741	8.7	67.8
3	3	4	1	0.375	16192	8.7	65.4
4	12	5	1	0.625	16192	8.7	65.4
5	25	6	1	0.375	10984	9.7	68.4
6	17	7	1	0.625	10984	9.7	68.4
7	23	8	1	0.375	16273	10.0	66.8
8	26	9	1	0.625	16273	10.0	66.8
9	11	10	1	0.375	10817	8.8	72.7
10	9	11	1	0.625	10817	8.8	72.7
11	19	12	1	0.375	14252	9.0	71.2
12	7	13	1	0.625	14252	9.0	71.2
26	14	14	1	0.500	12780	9.4	69.4
13	15	15	1	0.375	10783	9.9	73.5
14	16	16	1	0.625	10783	9.9	73.5
15	4	17	1	0.375	14608	9.7	71.6
16	10	18	1	0.625	14608	9.7	71.6
17	22	19	1	0.25	12780	9.4	69.4
18	8	20	1	0.75	12780	9.4	69.4
19	2	21	1	0.500	9620	9.4	70.3
20	5	22	1	0.500	17400	9.3	68.6
21	20	23	1	0.500	12808	8.5	68.9
22	21	24	1	0.500	13060	10.5	70.1
23	18	25	1	0.500	12500	9.4	62.3
24	13	26	1	0.500	12460	9.3	77.0
27	27	27	1	0.500	12780	9.4	69.4

Table S5. Actual factor levels in the DoE (based on theoretical weighed amounts of both fillers and up to 3 HPMC QbD samples per experiment).

Standard Order (as provided by the software)	Run Order (randomized)	Std Order (centre points positioned at start, middle and end of DoE)	Blocks	**Factor A (Lac):** Fraction of FlowLac® 100 in filler composition (FlowLac® 100 + Avicel® PH-102)	**Factor B (HPMC_Visc):** HPMC K15M Viscosity (m·Pas)	**Factor C (HPMC_HP):** HPMc K15M Hydroxypropoxy content (%)	**Factor D (HPMC_PS):** HPMC K15M Mean particle size (µm)
25	1	1	1	0	-0.053	0.013	-0.013
1	24	2	1	-1	-0.987	-0.984	-0.977
2	6	3	1	1	-0.987	-0.984	-0.977
3	3	4	1	-1	0.987	-0.987	-1.014
4	12	5	1	1	0.987	-0.987	-1.014
5	25	6	1	-1	-0.965	0.968	-0.969
6	17	7	1	1	-0.965	0.968	-0.969
7	23	8	1	-1	0.992	0.996	-0.993
8	26	9	1	1	0.992	0.996	-0.993
9	11	10	1	-1	-0.980	-0.979	0.979
10	9	11	1	1	-0.980	-0.979	0.979
11	19	12	1	-1	0.869	-0.956	0.958
12	7	13	1	1	0.869	-0.956	0.958
26	14	14	1	0	-0.053	0.013	-0.013
13	15	15	1	-1	-0.983	0.989	0.989
14	16	16	1	1	-0.983	0.989	0.989
15	4	17	1	-1	0.891	0.965	0.964
16	10	18	1	1	0.891	0.965	0.964
17	22	19	1	-2	-0.053	0.013	-0.013
18	8	20	1	2	-0.053	0.013	-0.013
19	2	21	1	0	-1.601	0.011	0.001
20	5	22	1	0	1.803	0.000	-0.024
21	20	23	1	0	-0.051	-1.868	-0.020
22	21	24	1	0	-0.033	1.961	-0.003
23	18	25	1	0	-0.074	0.011	-2.000
24	13	26	1	0	-0.077	0.000	1.967
27	27	27	1	0	-0.053	0.013	-0.013

Table S6. Experimental carvedilol release results shown as mean release (%) ± 1 SD. Data is shown for experiments 1 to 9[1].

t (min)	t (h)	Mean release ± SD (%), DoE Id -1_-1_-1_-1 (Stamdard order 1)[1]	Mean release ± SD (%), DoE Id 1_-1_-1_-1 (Stamdard order 2)[1]	Mean release ± SD (%), DoE Id -1_1_-1_-1 (Stamdard order 3)[1]	Mean release ± SD (%), DoE Id 1_1_-1_-1 (Stamdard order 4)[1]	Mean release ± SD (%), DoE Id -1_-1_1_-1 (Stamdard order 5)[1]	Mean release ± SD (%), DoE Id 1_-1_1_-1 (Stamdard order 6)[1]	Mean release ± SD (%), DoE Id -1_1_1_-1 (Stamdard order 7)[1]	Mean release ± SD (%), DoE Id 1_1_1_-1 (Stamdard order 8)[1]	Mean release ± SD (%), DoE Id -1_-1_-1_1 (Stamdard order 9)[1]
10	0.167	9.1 ± 1.1	11.8 ± 2.3	7.3 ± 0.7	8.1 ± 0.8	7.1 ± 0.9	12.8 ± 5.1	7.5 ± 0.9	9.8 ± 1.3	8.6 ± 1.1
20	0.333	12.4 ± 1.3	16.3 ± 2.6	10 ± 1	11.4 ± 0.9	10.1 ± 1.3	17.1 ± 5.3	10.5 ± 1.1	13.4 ± 1.2	12.1 ± 1.6
30	0.5	14.6 ± 1.4	18.9 ± 2.7	11.9 ± 1.1	13.7 ± 0.9	12.1 ± 1.5	19.6 ± 5.4	12.6 ± 1.2	15.8 ± 1.2	14.3 ± 1.7
45	0.75	17.4 ± 1.5	22 ± 2.9	14.3 ± 1.2	16.5 ± 0.9	14.7 ± 1.8	22.7 ± 5.5	15.2 ± 1.2	18.6 ± 1.3	16.9 ± 1.9
60	1	19.8 ± 1.6	24.7 ± 3	16.4 ± 1.3	18.9 ± 1	16.9 ± 1.9	25.4 ± 5.6	17.4 ± 1.3	21 ± 1.4	19.1 ± 1.9
90	1.5	24 ± 1.8	29.2 ± 3.2	20 ± 1.4	23.3 ± 1.1	20.8 ± 2.2	30 ± 5.7	21.4 ± 1.4	25.2 ± 1.6	23.2 ± 1.9
120	2	27.6 ± 2	33.3 ± 3.3	23.3 ± 1.5	27 ± 1	24.3 ± 2.4	33.9 ± 5.7	24.9 ± 1.5	29 ± 1.7	26.8 ± 1.8
150	2.5	31 ± 2.2	36.9 ± 3.3	26.4 ± 1.5	30.4 ± 1	27.5 ± 2.5	37.6 ± 5.7	28.1 ± 1.6	32.3 ± 1.9	30 ± 1.8
180	3	34 ± 2.4	40.2 ± 3.3	29.2 ± 1.6	33.6 ± 1	30.5 ± 2.6	40.9 ± 5.7	31.1 ± 1.7	35.4 ± 2.1	33 ± 1.7
210	3.5	36.9 ± 2.5	43.4 ± 3.3	31.9 ± 1.7	36.6 ± 0.9	33.3 ± 2.7	44.1 ± 5.8	34 ± 1.7	38.4 ± 2.3	35.8 ± 1.7
240	4	39 ± 3.6	46.5 ± 3.3	34.4 ± 1.7	39.5 ± 1	36 ± 2.8	47 ± 5.7	36.7 ± 1.8	41.3 ± 2.4	38.4 ± 1.7
270	4.5	41.5 ± 3.7	49.3 ± 3.3	36.8 ± 1.8	42.2 ± 0.9	38.6 ± 2.9	49.8 ± 5.7	39.3 ± 2.1	44 ± 2.6	40.9 ± 1.6
300	5	44 ± 3.4	52.1 ± 3.3	39.1 ± 1.8	44.9 ± 1	41 ± 2.9	52.5 ± 5.7	41.9 ± 2.1	46.7 ± 2.7	43.3 ± 1.6
330	5.5	46.4 ± 3.5	54.8 ± 3.3	41.4 ± 1.8	47.5 ± 0.9	43.4 ± 3	55.1 ± 5.7	44.3 ± 2.2	49.2 ± 2.8	45.6 ± 1.6
360	6	48.9 ± 4	57.3 ± 3.3	43.5 ± 1.8	49.9 ± 0.9	45.7 ± 3	57.7 ± 5.6	46.7 ± 2.3	51.7 ± 2.9	47.8 ± 1.5
420	7	52.7 ± 4.5	62.1 ± 3.3	47.7 ± 1.8	54.6 ± 0.8	50.1 ± 3.2	62.6 ± 5.5	51.5 ± 2.5	56.5 ± 3	52 ± 1.5
480	8	57 ± 4.5	66.6 ± 3.3	51.6 ± 1.8	58.9 ± 0.7	54.3 ± 3.4	67.1 ± 5.5	55.6 ± 2.9	61 ± 3.2	55.8 ± 1.5
540	9	61.8 ± 3.7	70.7 ± 3.3	55.3 ± 1.8	63 ± 0.7	58.3 ± 3.5	71.3 ± 5.5	59.8 ± 2.8	65.4 ± 3.2	59.4 ± 1.6
600	10	65.7 ± 4.2	74.6 ± 3.4	58.8 ± 1.7	66.9 ± 0.7	62.1 ± 3.5	75.1 ± 5.4	63.6 ± 2.8	69.5 ± 3.2	62.8 ± 1.6
660	11	68.9 ± 4.2	78.5 ± 3.4	62.2 ± 1.8	70.6 ± 0.7	65.6 ± 3.6	78.5 ± 5.3	67.3 ± 2.6	73.4 ± 3.3	65.9 ± 1.7
720	12	72.1 ± 4.3	81.9 ± 3.4	65.4 ± 1.8	74.1 ± 0.8	68.9 ± 3.6	81.7 ± 5.2	70.7 ± 2.5	77 ± 3.3	68.9 ± 1.8
780	13	75.2 ± 4.2	85.1 ± 3.3	68.3 ± 1.8	77.5 ± 1.2	72 ± 3.6	84.6 ± 5.1	73.8 ± 2.2	80.5 ± 3.5	71.7 ± 1.7
840	14	77.7 ± 3.9	87.9 ± 3.2	71.1 ± 1.8	80.9 ± 2	75 ± 3.6	87.4 ± 5.1	77.1 ± 2.3	84 ± 4.4	74.3 ± 1.8
900	15	80.6 ± 4.5	90.4 ± 3.1	73.7 ± 1.8	84 ± 3	77.6 ± 3.5	89.9 ± 4.9	80.2 ± 2.5	87.2 ± 5.3	76.9 ± 1.7
960	16	82.8 ± 4.1	93 ± 3.3	76.2 ± 1.8	86.6 ± 3.5	80.1 ± 3.4	92.4 ± 4.5	83.1 ± 2.8	89.6 ± 5.4	79.3 ± 1.5
1020	17	86.3 ± 5	95 ± 3.1	78.5 ± 1.8	88.7 ± 3.4	82.5 ± 3.4	94 ± 4.1	85.5 ± 2.4	91.5 ± 5.1	81.5 ± 1.3
1080	18	88.3 ± 4.5	96.8 ± 2.9	80.7 ± 1.6	90.4 ± 2.9	84.6 ± 3.3	95.2 ± 3.7	87.8 ± 2.2	92.8 ± 4.4	83.5 ± 0.9
1140	19	89.7 ± 3.5	97.8 ± 2.3	82.7 ± 1.4	91.7 ± 2.3	86.1 ± 2.6	96 ± 3.2	89.5 ± 2.5	93.9 ± 3.7	85.3 ± 0.7
1200	20	91.1 ± 3.1	98.6 ± 1.5	84.6 ± 1.2	92.7 ± 1.8	87.8 ± 2.6	96.4 ± 2.8	91.1 ± 2.5	94.8 ± 3	87 ± 0.5
1260	21	92.8 ± 2.7	99.1 ± 1	86.4 ± 1	93.4 ± 1.4	89.1 ± 2.3	96.6 ± 2.5	92.6 ± 2.2	95.8 ± 2.5	88.4 ± 0.2
1320	22	94.1 ± 2.3	99.1 ± 1	88 ± 1	93.9 ± 1.1	90.3 ± 2.1	96.9 ± 2.4	93.9 ± 2.1	96.3 ± 2	89.6 ± 0.1
1380	23	94.9 ± 2	99.1 ± 1	89.5 ± 1.3	94.1 ± 1.1	91.6 ± 2.1	97 ± 2.4	94.9 ± 2	96.7 ± 1.4	90.5 ± 0.2
1440	24	95.5 ± 1.8	99 ± 1	90.8 ± 1.5	94.2 ± 1	92.5 ± 1.9	97.1 ± 2.4	95.9 ± 1.8	96.7 ± 1	91.3 ± 0.3

[1] As provided by the Minitab® software,corresponds to data in the first column of Tables S2, S3, S4, and S5.

Note: "DoE Id" data in the first row is comprised of theoretical levels (-2, -1, 0, 1, 2) of factors A (Lac), B (HPMC_Visc), C (HPMC_HP), and D (HPMC_PS) used in each experiment, respectively.

Table S7. Experimental carvedilol release results shown as mean release (%) ± 1 SD. Data is shown for experiments 10 to 18[1].

t(min)	t(h)	Mean release ± SD (%), DoE Id 1_-1_-1_1 (Stamdard order 10)[1]	Mean release ± SD (%), DoE Id -1_1_-1_1 (Stamdard order 11)[1]	Mean release ± SD (%), DoE Id 1_1_-1_1 (Stamdard order 12)[1]	Mean release ± SD (%), DoE Id -1_-1_1_1 (Stamdard order 13)[1]	Mean release ± SD (%), DoE Id 1_-1_1_1 (Stamdard order 14)[1]	Mean release ± SD (%), DoE Id -1_1_1_1 (Stamdard order 15)[1]	Mean release ± SD (%), DoE Id 1_1_1_1 (Stamdard order 16)[1]	Mean release ± SD (%), DoE Id -2_0_0_0 (Stamdard order 17)[1]	Mean release ± SD (%), DoE Id 2_0_0_0 (Stamdard order 18)[1]
10	0.167	11.3 ± 2.5	9.4 ± 0.2	12.5 ± 1.9	7.9 ± 0.8	12.4 ± 2.6	10.5 ± 1.4	10.2 ± 1.7	8.2 ± 1.8	13.1 ± 3.6
20	0.333	15.4 ± 2.6	13 ± 0.4	16.5 ± 2.1	11.3 ± 1.2	16.9 ± 3	14.4 ± 2	13.9 ± 1.7	11 ± 2.1	17.4 ± 3.9
30	0.5	18 ± 2.6	15.3 ± 0.4	19 ± 2.1	13.5 ± 1.4	19.9 ± 3.2	16.8 ± 2.2	16.3 ± 1.9	12.9 ± 2.2	20.3 ± 4.1
45	0.75	21.1 ± 2.5	18.1 ± 0.3	22.1 ± 2.2	16.3 ± 1.3	23.4 ± 3.6	19.6 ± 2.4	19.2 ± 2.2	15.3 ± 2.3	23.7 ± 4.4
60	1	23.9 ± 2.4	20.4 ± 0.3	24.7 ± 2.2	18.6 ± 1.3	26.3 ± 3.9	22 ± 2.5	21.6 ± 2.5	17.4 ± 2.4	26.7 ± 4.7
90	1.5	28.7 ± 2.4	24.5 ± 0.3	29.2 ± 2.1	22.6 ± 1.3	31.2 ± 4.4	26.1 ± 2.6	25.9 ± 3	21.1 ± 2.7	31.7 ± 5
120	2	32.8 ± 2.5	28.1 ± 0.5	33.2 ± 2	26.2 ± 1.3	35.4 ± 4.6	29.7 ± 2.7	29.7 ± 3.5	24.3 ± 2.9	36.1 ± 5.4
150	2.5	36.5 ± 2.5	31.5 ± 0.7	36.7 ± 1.9	29.4 ± 1.3	39.1 ± 4.9	33 ± 2.7	33.2 ± 3.9	27.3 ± 3	40 ± 5.6
180	3	40 ± 2.5	34.5 ± 0.8	40 ± 1.9	32.4 ± 1.4	42.5 ± 5	36 ± 2.7	36.4 ± 4.3	30.1 ± 3.1	43.6 ± 5.8
210	3.5	43.2 ± 2.5	37.4 ± 0.9	43 ± 1.8	35.3 ± 1.5	45.7 ± 5.2	38.8 ± 2.6	39.5 ± 4.6	32.7 ± 3.1	47 ± 6
240	4	46.2 ± 2.5	40 ± 1.1	45.9 ± 1.8	38 ± 1.6	48.8 ± 5.4	41.4 ± 2.5	42.5 ± 4.7	35.2 ± 3.2	50.2 ± 6.1
270	4.5	49.1 ± 2.5	42.7 ± 1.2	48.6 ± 1.9	40.5 ± 1.6	51.6 ± 5.5	44 ± 2.5	45.3 ± 4.9	37.5 ± 3.3	53.3 ± 6.3
300	5	51.9 ± 2.5	45.2 ± 1.3	51.2 ± 1.9	43 ± 1.7	54.5 ± 5.6	46.3 ± 2.2	48 ± 4.9	39.8 ± 3.4	56.3 ± 6.4
330	5.5	54.5 ± 2.5	47.6 ± 1.3	53.7 ± 2	45.4 ± 1.8	57.2 ± 5.8	48.6 ± 2.1	50.7 ± 5	42.1 ± 3.5	59.1 ± 6.5
360	6	57.1 ± 2.4	50 ± 1.4	56.1 ± 2	47.8 ± 1.9	59.8 ± 5.9	50.9 ± 2.1	53.4 ± 5	44.2 ± 3.6	61.9 ± 6.7
420	7	62 ± 2.3	54.5 ± 1.6	60.7 ± 2	52.3 ± 2.2	64.7 ± 6.1	54.7 ± 1.8	58.4 ± 5.1	48.5 ± 3.8	67 ± 6.8
480	8	66.5 ± 2.2	59 ± 1.6	64.9 ± 2	56.6 ± 2.4	69.3 ± 6.3	58.9 ± 1.8	63 ± 5.1	52.5 ± 4	71.8 ± 6.9
540	9	70.7 ± 2.1	63.2 ± 1.6	68.9 ± 1.9	60.6 ± 2.8	73.6 ± 6.4	62.6 ± 1.9	67.3 ± 4.9	56.4 ± 4.3	77.1 ± 7.6
600	10	74.8 ± 2.2	67.1 ± 1.6	72.6 ± 1.8	64.4 ± 3	77.5 ± 6.6	66.4 ± 2	71.3 ± 4.9	60.1 ± 4.5	84.2 ± 10.5
660	11	78.6 ± 2.4	70.9 ± 1.5	76.2 ± 1.9	68.1 ± 3.3	82.1 ± 7.8	70.1 ± 2	75.1 ± 4.8	63.7 ± 4.7	88.5 ± 10.6
720	12	82.6 ± 2.6	74.4 ± 1.4	79.6 ± 1.8	71.6 ± 3.6	86.1 ± 8.4	73.5 ± 2.1	78.6 ± 4.6	67 ± 4.8	91 ± 9.1
780	13	86.3 ± 3.1	77.7 ± 1.4	82.6 ± 1.7	74.7 ± 3.8	88.8 ± 7.9	76.5 ± 2.2	81.9 ± 4.2	70.2 ± 4.9	93.3 ± 7.5
840	14	89.5 ± 3.5	80.8 ± 1.3	85.5 ± 1.5	77.6 ± 3.9	90.4 ± 6.6	79.2 ± 2.3	84.9 ± 3.9	73.2 ± 5.1	94.9 ± 6.1
900	15	92.3 ± 3.6	83.6 ± 1.3	88 ± 1.3	80.3 ± 4	91.6 ± 5.1	81.6 ± 2.3	87.6 ± 3.6	75.9 ± 5.2	95.8 ± 4.9
960	16	94.5 ± 3.2	86.2 ± 1.2	90.3 ± 1.2	82.7 ± 3.9	92.6 ± 3.7	83.9 ± 2.4	89.9 ± 3.4	78.6 ± 5.1	96.3 ± 3.8
1020	17	96.3 ± 2.7	88.5 ± 1.2	92.4 ± 1.3	85 ± 3.7	93.7 ± 2.4	86 ± 2.3	92 ± 3	80.9 ± 4.9	96.7 ± 2.8
1080	18	97.8 ± 2.3	90.5 ± 1.1	94.3 ± 1.7	86.9 ± 3.4	94.6 ± 1.5	87.9 ± 2.2	93.8 ± 2.6	83.2 ± 4.7	97 ± 1.9
1140	19	98.8 ± 1.9	92.5 ± 1.1	95.8 ± 1.5	88.7 ± 3.3	94.9 ± 0.9	89.4 ± 2.1	95.5 ± 2.1	85.2 ± 4.5	97.3 ± 1.3
1200	20	99.6 ± 1.7	94.1 ± 1.1	96.7 ± 0.9	90.1 ± 3.1	95.1 ± 0.8	90.7 ± 2	96.9 ± 1.5	87.1 ± 4.3	97.7 ± 0.5
1260	21	100.2 ± 1.5	95.7 ± 1.1	97.3 ± 0.4	91.3 ± 2.9	95 ± 0.9	91.8 ± 2.2	98 ± 0.8	88.7 ± 4	97.7 ± 0.5
1320	22	100.5 ± 1.8	97 ± 1.1	97.6 ± 0.5	92.4 ± 2.8	94.7 ± 1	92.9 ± 2.2	98.7 ± 0.8	90.1 ± 3.8	97.6 ± 0.5
1380	23	100.5 ± 2.1	98.2 ± 1.2	97.6 ± 0.8	93.2 ± 2.6	94.5 ± 1	93.9 ± 1.9	99.1 ± 1.1	91.4 ± 3.6	97.4 ± 0.5
1440	24	100.5 ± 2.2	99.1 ± 1.2	97.6 ± 1	93.9 ± 2.4	94.3 ± 1.1	94.9 ± 1.6	99.3 ± 1.5	92.4 ± 3.5	97.3 ± 0.5

[1] As provided by the Minitab® software,corresponds to data in the first column of Tables S2, S3, S4, and S5.

Note: "DoE Id" data in the first row is comprised of theoretical levels (-2, -1, 0, 1, 2) of factors A (Lac), B (HPMC_Visc), C (HPMC_HP), and D (HPMC_PS) used in each experiment, respectively.

Table S8. Experimental carvedilol release results shown as mean release (%) ± 1 SD. Data is shown for experiments 19 to 27[1].

t(min)	t(h)	Mean release ± SD (%), DoE Id 0_-2_0_0 (Stamdard order 19)[1]	Mean release ± SD (%), DoE Id 0_2_0_0 (Stamdard order 20)[1]	Mean release ± SD (%), DoE Id 0_0_-2_0 (Stamdard order 21)[1]	Mean release ± SD (%), DoE Id 0_0_2_0 (Stamdard order 22)[1]	Mean release ± SD (%), DoE Id 0_0_0_-2 (Stamdard order 23)[1]	Mean release ± SD (%), DoE Id 0_0_0_2 (Stamdard order 24)[1]	Mean release ± SD (%), DoE Id 0_0_0_0 (1 of 3) (Stamdard order 25)[1]	Mean release ± SD (%), DoE Id 0_0_0_0 (2 of 3) (Stamdard order 26)[1]	Mean release ± SD (%), DoE Id 0_0_0_0 (3 of 3) (Stamdard order 27)[1]
10	0.167	9.6 ± 1	13.1 ± 2	10.5 ± 1.4	12.7 ± 1.2	9.3 ± 1.3	10.3 ± 1	8.1 ± 0.7	8.5 ± 1.8	12.9 ± 3
20	0.333	13.4 ± 1.4	17.6 ± 2.4	14.1 ± 1.5	17.1 ± 1.5	12.8 ± 1.4	14.8 ± 1.1	11.7 ± 0.9	11.9 ± 2.1	16.9 ± 3.1
30	0.5	15.9 ± 1.4	20.1 ± 2.4	16.4 ± 1.5	19.9 ± 1.5	15 ± 1.4	17.4 ± 1.1	14.1 ± 1	14.2 ± 2.4	19.5 ± 3.1
45	0.75	19 ± 1.4	23.1 ± 2.6	19.3 ± 1.6	23.3 ± 1.4	17.8 ± 1.4	20.3 ± 1.1	16.9 ± 0.9	17 ± 2.8	22.6 ± 3.2
60	1	21.7 ± 1.3	25.5 ± 2.7	21.8 ± 1.6	26.1 ± 1.5	20.3 ± 1.4	22.8 ± 1.1	19.4 ± 0.9	19.5 ± 3	25.1 ± 3.2
90	1.5	26.4 ± 1.1	29.7 ± 3	26.1 ± 1.7	30.8 ± 1.5	24.6 ± 1.3	27.2 ± 1.1	23.6 ± 0.6	23.8 ± 3.5	29.5 ± 3.2
120	2	30.5 ± 1.1	33.5 ± 3.2	29.8 ± 1.9	34.9 ± 1.6	28.6 ± 1.4	31.1 ± 1.2	27.5 ± 0.4	27.6 ± 4	33.3 ± 3.1
150	2.5	34.3 ± 1.1	36.8 ± 3.4	33.2 ± 2	38.6 ± 1.7	32.1 ± 1.4	34.5 ± 1.2	30.9 ± 0.2	31.1 ± 4.3	36.8 ± 3.1
180	3	37.7 ± 1.1	39.8 ± 3.5	36.2 ± 2.1	42 ± 1.8	35.4 ± 1.4	37.8 ± 1.3	34.2 ± 0.1	34.4 ± 4.5	40 ± 3
210	3.5	40.8 ± 1.1	42.7 ± 3.6	39.1 ± 2.1	45.2 ± 1.9	38.7 ± 1.4	40.9 ± 1.5	37.2 ± 0.2	37.4 ± 4.7	43 ± 2.9
240	4	43.9 ± 1.2	45.4 ± 3.7	41.9 ± 2.2	48.2 ± 2	41.7 ± 1.5	43.8 ± 1.6	40 ± 0.4	40.4 ± 4.9	45.8 ± 2.8
270	4.5	46.8 ± 1.2	48 ± 3.7	44.4 ± 2.2	51 ± 2.1	44.7 ± 1.5	46.5 ± 1.7	42.7 ± 0.5	43.2 ± 5	48.5 ± 2.8
300	5	49.6 ± 1.3	50.5 ± 3.8	46.9 ± 2.2	53.7 ± 2.2	47.6 ± 1.5	49.2 ± 1.8	45.3 ± 0.7	45.9 ± 5.1	51.1 ± 2.7
330	5.5	52.3 ± 1.4	52.9 ± 3.8	49.3 ± 2.3	56.4 ± 2.3	50.3 ± 1.4	51.7 ± 2	47.9 ± 0.8	48.5 ± 5.2	53.6 ± 2.7
360	6	55 ± 1.5	55.2 ± 3.9	51.6 ± 2.3	58.9 ± 2.4	52.9 ± 1.4	54.1 ± 2.1	50.3 ± 1	51.1 ± 5.3	56 ± 2.6
420	7	59.9 ± 1.6	59.5 ± 3.9	56 ± 2.4	63.8 ± 2.5	58 ± 1.3	58.7 ± 2.2	54.9 ± 1.3	55.9 ± 5.6	60.6 ± 2.5
480	8	64.6 ± 1.6	63.6 ± 3.9	60 ± 2.5	68.4 ± 2.7	62.7 ± 1.2	63 ± 2.5	59.3 ± 1.4	60.5 ± 5.9	64.9 ± 2.3
540	9	68.9 ± 1.5	67.4 ± 3.8	63.9 ± 2.6	72.5 ± 3	67.1 ± 1	67 ± 2.6	63.3 ± 1.5	64.8 ± 6	69 ± 2.2
600	10	72.9 ± 1.6	70.9 ± 3.6	67.6 ± 2.7	76.2 ± 3.1	71.2 ± 1	70.8 ± 2.6	67.1 ± 1.5	68.9 ± 6.1	72.7 ± 2.2
660	11	76.7 ± 1.7	74.3 ± 3.4	71 ± 2.7	79.5 ± 3.2	74.8 ± 0.8	74.2 ± 2.5	70.7 ± 1.4	72.6 ± 6.3	76.1 ± 2.1
720	12	80.2 ± 1.7	77.3 ± 3.3	74.1 ± 2.7	82.5 ± 3.3	78.1 ± 0.8	77.4 ± 2.5	74 ± 1.4	76.1 ± 6.3	79.3 ± 2
780	13	83.4 ± 1.7	80.1 ± 3.1	77.2 ± 2.6	85.2 ± 3.4	81 ± 0.9	80.5 ± 2.5	77.1 ± 1.5	79.2 ± 6	82.3 ± 1.9
840	14	86.4 ± 1.8	82.7 ± 2.8	80.1 ± 2.6	87.7 ± 3.5	83.6 ± 1	83.4 ± 2.4	80 ± 1.5	82.1 ± 5.7	84.9 ± 1.8
900	15	89.1 ± 1.8	85.1 ± 2.6	82.7 ± 2.5	90 ± 3.7	85.9 ± 1.2	86 ± 2.3	82.7 ± 1.5	84.7 ± 5.2	87.3 ± 1.7
960	16	91.5 ± 2	87.2 ± 2.4	85.3 ± 2.4	92.1 ± 4.1	87.8 ± 1.3	88.4 ± 2.2	85.2 ± 1.4	87.2 ± 4.8	89.5 ± 1.6
1020	17	93.6 ± 2.1	89 ± 2.2	87.6 ± 2.3	93.8 ± 4.1	89.6 ± 1.4	90.6 ± 2	87.3 ± 1.4	89.5 ± 4.5	91.4 ± 1.4
1080	18	95.4 ± 2.1	90.6 ± 2.1	89.7 ± 2.3	95 ± 3.7	91 ± 1.6	92.5 ± 1.9	89.2 ± 1.3	91.6 ± 4.2	93.2 ± 1.3
1140	19	96.8 ± 2.1	92 ± 2	91.3 ± 2.2	95.8 ± 3.2	92.3 ± 1.8	94.1 ± 1.9	90.8 ± 1.3	93.5 ± 3.8	94.6 ± 1.2
1200	20	97.9 ± 1.7	93.2 ± 1.9	92.8 ± 2.2	96.3 ± 2.7	93.3 ± 2	95.6 ± 1.9	92.3 ± 1.2	95.2 ± 3.6	96 ± 1.1
1260	21	98.8 ± 1.1	94.2 ± 1.8	94.1 ± 2.2	96.7 ± 2.2	94.1 ± 2.2	96.8 ± 1.9	93.6 ± 1	96.6 ± 3.4	97 ± 0.9
1320	22	99.4 ± 0.7	95.1 ± 1.6	95.2 ± 2.2	96.9 ± 1.9	94.8 ± 2.4	97.8 ± 1.9	94.8 ± 0.9	97.8 ± 3.1	97.8 ± 0.7
1380	23	99.7 ± 0.4	95.8 ± 1.4	96.2 ± 2.1	97.1 ± 1.6	95.2 ± 2.6	98.7 ± 1.9	95.8 ± 0.8	98.7 ± 2.7	98.5 ± 0.5
1440	24	100 ± 0.3	96.3 ± 1.2	97 ± 2.1	97.3 ± 1.4	95.4 ± 2.7	99.4 ± 1.9	96.9 ± 0.6	99.5 ± 2.3	99.1 ± 0.5

[1] As provided by the Minitab® software,corresponds to data in the first column of Tables S2, S3, S4, and S5.

Note: "DoE Id" data in the first row is comprised of theoretical levels (-2, -1, 0, 1, 2) of factors A (Lac), B (HPMC_Visc), C (HPMC_HP), and D (HPMC_PS) used in each experiment, respectively.

Table S9. R^2 and R^2_{pred} for full RSM MLR models and MLR models generated via stepwise regression procedures for predicting mean % of carvedilol release. Range of models used in interpretation of main effects and interaction effects of factors in terms of their influence on carvedilol release is bolded.

t (min)	t (h)	Full RSM (CCD) MLR model, R^2	Full RSM (CCD) MLR model, R^2_{pred}	Stepwise Regression, Forward, Information Criterion (AIC), R^2	Stepwise Regression, Forward, Information Criterion (AIC), R^2_{pred}	Stepwise Regression, Forward, Information Criterion (BIC), R^2	Stepwise Regression, Forward, Information Criterion (BIC), R^2_{pred}	Stepwise Regression, Stepwise, R^2	Stepwise Regression, Stepwise, R^2_{pred}	Stepwise Regression, Forward Selection, R^2	Stepwise Regression, Forward Selection, R^2_{pred}	Stepwise Regression, Backward Elimination, R^2	Stepwise Regression, Backward Elimination, R^2predicted
10	0.167	71.08%	0.00%	40.13%	32.16%	40.13%	32.16%	45.35%	34.89%	45.35%	34.89%	55.81%	37.66%
**20**	**0.333**	**74.48%**	**0.00%**	**49.14%**	**39.71%**	**49.14%**	**39.71%**	**49.14%**	**39.71%**	**49.14%**	**39.71%**	**63.84%**	**47.25%**
**30**	**0.5**	**76.18%**	**0.00%**	**52.09%**	**43.19%**	**52.09%**	**43.19%**	**52.09%**	**43.19%**	**52.09%**	**43.19%**	**65.75%**	**49.97%**
**45**	**0.75**	**77.56%**	**0.00%**	**54.56%**	**45.87%**	**54.56%**	**45.87%**	**54.56%**	**45.87%**	**54.56%**	**45.87%**	**67.31%**	**52.11%**
**60**	**1**	**78.62%**	**0.00%**	**56.40%**	**47.83%**	**56.40%**	**47.83%**	**56.40%**	**47.83%**	**56.40%**	**47.83%**	**68.68%**	**53.95%**
**90**	**1.5**	**80.03%**	**0.00%**	**59.18%**	**50.86%**	**59.18%**	**50.86%**	**59.18%**	**50.86%**	**59.18%**	**50.86%**	**70.77%**	**57.03%**
**120**	**2**	**81.24%**	**0.03%**	**61.27%**	**53.14%**	**61.27%**	**53.14%**	**61.27%**	**53.14%**	**61.27%**	**53.14%**	**72.59%**	**59.57%**
**150**	**2.5**	**82.28%**	**2.39%**	**62.60%**	**54.51%**	**62.60%**	**54.51%**	**62.60%**	**54.51%**	**62.60%**	**54.51%**	**74.00%**	**61.63%**
**180**	**3**	**83.14%**	**4.41%**	**64.06%**	**56.13%**	**64.06%**	**56.13%**	**64.06%**	**56.13%**	**64.06%**	**56.13%**	**75.30%**	**63.58%**
**210**	**3.5**	**83.98%**	**7.58%**	**65.35%**	**57.55%**	**65.35%**	**57.55%**	**65.35%**	**57.55%**	**65.35%**	**57.55%**	**76.53%**	**65.41%**
**240**	**4**	**85.07%**	**11.82%**	**67.14%**	**59.56%**	**67.14%**	**59.56%**	**67.14%**	**59.56%**	**67.14%**	**59.56%**	**78.00%**	**67.50%**
**270**	**4.5**	**85.80%**	**13.99%**	**68.05%**	**60.46%**	**80.87%**	**67.75%**	**68.05%**	**60.46%**	**84.09%**	**44.33%**	**78.97%**	**68.82%**
**300**	**5**	**86.27%**	**14.81%**	**81.73%**	**69.09%**	**81.73%**	**69.09%**	**68.94%**	**61.40%**	**84.67%**	**45.46%**	**79.64%**	**69.70%**
**330**	**5.5**	**86.89%**	**17.01%**	**82.69%**	**70.63%**	**82.69%**	**70.63%**	**69.67%**	**62.12%**	**85.39%**	**47.42%**	**80.44%**	**70.86%**
**360**	**6**	**87.40%**	**18.68%**	**83.46%**	**71.86%**	**83.46%**	**71.86%**	**70.23%**	**62.66%**	**86.00%**	**49.25%**	**81.04%**	**71.69%**
**420**	**7**	**88.54%**	**24.06%**	**80.39%**	**67.24%**	**85.11%**	**74.52%**	**80.39%**	**67.24%**	**85.11%**	**74.52%**	**85.11%**	**74.52%**
**480**	**8**	**89.11%**	**26.25%**	**81.57%**	**69.15%**	**86.05%**	**75.99%**	**81.57%**	**69.15%**	**86.05%**	**75.99%**	**86.05%**	**75.99%**
**540**	**9**	**88.72%**	**23.92%**	**82.07%**	**70.13%**	**86.24%**	**76.37%**	**82.07%**	**70.13%**	**82.07%**	**70.13%**	**86.24%**	**76.37%**
**600**	**10**	**87.98%**	**19.40%**	**82.30%**	**70.70%**	**86.22%**	**76.62%**	**82.30%**	**70.70%**	**82.30%**	**70.70%**	**86.22%**	**76.62%**
**660**	**11**	**88.40%**	**23.07%**	**83.42%**	**72.75%**	**83.42%**	**72.75%**	**83.42%**	**72.75%**	**83.42%**	**72.75%**	**86.86%**	**77.61%**
**720**	**12**	**88.99%**	**26.75%**	**84.72%**	**75.00%**	**84.72%**	**75.00%**	**84.72%**	**75.00%**	**84.72%**	**75.00%**	**87.82%**	**78.91%**
**780**	**13**	**89.44%**	**30.92%**	**85.33%**	**76.01%**	**85.33%**	**76.01%**	**85.33%**	**76.01%**	**85.33%**	**76.01%**	**88.42%**	**79.78%**
**840**	**14**	**90.07%**	**38.36%**	**86.01%**	**77.17%**	**86.01%**	**77.17%**	**86.01%**	**77.17%**	**86.01%**	**77.17%**	**88.97%**	**80.32%**
**900**	**15**	**90.07%**	**41.07%**	**85.48%**	**76.17%**	**85.48%**	**76.17%**	**85.48%**	**76.17%**	**86.48%**	**74.02%**	**88.38%**	**78.50%**
**960**	**16**	**90.41%**	**46.74%**	**84.32%**	**73.98%**	**84.32%**	**73.98%**	**84.32%**	**73.98%**	**85.81%**	**72.61%**	**87.50%**	**76.02%**
1020	17	88.98%	40.50%	79.02%	70.11%	79.02%	70.11%	83.30%	67.89%	83.30%	67.89%	80.67%	71.94%
1080	18	88.14%	39.35%	73.34%	65.69%	73.34%	65.69%	80.82%	63.21%	82.41%	63.15%	79.01%	69.27%
1140	19	86.26%	32.71%	69.85%	61.02%	69.85%	61.02%	69.85%	61.02%	80.85%	55.27%	76.83%	65.94%
1200	20	84.26%	26.09%	77.18%	63.26%	77.18%	63.26%	69.16%	56.43%	69.16%	56.43%	74.09%	61.76%
1260	21	80.70%	11.44%	59.65%	48.70%	54.85%	47.39%	63.93%	49.27%	63.93%	49.27%	73.02%	56.37%
1320	22	76.83%	0.00%	57.43%	40.50%	46.25%	37.30%	57.43%	40.50%	57.43%	40.50%	72.61%	46.24%
1380	23	72.89%	0.00%	44.42%	29.46%	44.42%	29.46%	50.28%	30.75%	50.28%	30.75%	63.00%	40.66%
1440	24	69.74%	0.00%	36.02%	19.07%	36.02%	19.07%	42.54%	20.11%	42.54%	20.11%	57.38%	31.85%

Table S10. R^2 and R^2_{pred} of full RSM MLR models, Basic MLR models and selected Optimized MLR models generated via stepwise regression procedures for predicting mean % of carvedilol release. Range of models used in interpretation of main effects and interaction effects of factors in terms of their influence on carvedilol release is bolded.

t (min)	t (h)	Full RSM (CCD) MLR model, R^2	Full RSM (CCD) MLR model, R^2_{pred}	Basic MLR model, R^2	Basic MLR model, R^2_{pred}	Stepwise regression procedure used in the Optimized MLR model	Optimized MLR model, R^2	Optimized MLR model, R^2_{pred}
10	0.167	71.08%	0.00%	46.12%	20.66%	Stepwise Regression, Backward Elimination	55.81%	37.66%
**20**	**0.333**	**74.48%**	**0.00%**	**50.10%**	**25.81%**	**Stepwise Regression, Backward Elimination**	**63.84%**	**47.25%**
**30**	**0.5**	**76.18%**	**0.00%**	**53.29%**	**30.46%**	**Stepwise Regression, Backward Elimination**	**65.75%**	**49.97%**
**45**	**0.75**	**77.56%**	**0.00%**	**56.04%**	**34.41%**	**Stepwise Regression, Backward Elimination**	**67.31%**	**52.11%**
**60**	**1**	**78.62%**	**0.00%**	**58.08%**	**37.28%**	**Stepwise Regression, Backward Elimination**	**68.68%**	**53.95%**
**90**	**1.5**	**80.03%**	**0.00%**	**61.21%**	**41.79%**	**Stepwise Regression, Backward Elimination**	**70.77%**	**57.03%**
**120**	**2**	**81.24%**	**0.03%**	**63.59%**	**45.10%**	**Stepwise Regression, Backward Elimination**	**72.59%**	**59.57%**
**150**	**2.5**	**82.28%**	**2.39%**	**65.30%**	**47.46%**	**Stepwise Regression, Backward Elimination**	**74.00%**	**61.63%**
**180**	**3**	**83.14%**	**4.41%**	**67.13%**	**50.09%**	**Stepwise Regression, Backward Elimination**	**75.30%**	**63.58%**
**210**	**3.5**	**83.98%**	**7.58%**	**68.71%**	**52.37%**	**Stepwise Regression, Backward Elimination**	**76.53%**	**65.41%**
**240**	**4**	**85.07%**	**11.82%**	**70.83%**	**55.49%**	**Stepwise Regression, Backward Elimination**	**78.00%**	**67.50%**
**270**	**4.5**	**85.80%**	**13.99%**	**72.00%**	**57.12%**	**Stepwise Regression, Backward Elimination**	**78.97%**	**68.82%**
**300**	**5**	**86.27%**	**14.81%**	**73.26%**	**58.92%**	**Stepwise Regression, Backward Elimination**	**79.64%**	**69.70%**
**330**	**5.5**	**86.89%**	**17.01%**	**74.40%**	**60.57%**	**Stepwise Regression, Backward Elimination**	**80.44%**	**70.86%**
**360**	**6**	**87.40%**	**18.68%**	**75.32%**	**61.87%**	**Stepwise Regression, Backward Elimination**	**81.04%**	**71.69%**
**420**	**7**	**88.54%**	**24.06%**	**77.56%**	**65.19%**	**Stepwise Regression, Backward Elimination**	**85.11%**	**74.52%**
**480**	**8**	**89.11%**	**26.25%**	**78.70%**	**66.86%**	**Stepwise Regression, Backward Elimination**	**86.05%**	**75.99%**
**540**	**9**	**88.72%**	**23.92%**	**79.24%**	**67.74%**	**Stepwise Regression, Backward Elimination**	**86.24%**	**76.37%**
**600**	**10**	**87.98%**	**19.40%**	**79.54%**	**68.28%**	**Stepwise Regression, Backward Elimination**	**86.22%**	**76.62%**
**660**	**11**	**88.40%**	**23.07%**	**80.42%**	**69.79%**	**Stepwise Regression, Backward Elimination**	**86.86%**	**77.61%**
**720**	**12**	**88.99%**	**26.75%**	**81.57%**	**71.73%**	**Stepwise Regression, Backward Elimination**	**87.82%**	**78.91%**
**780**	**13**	**89.44%**	**30.92%**	**82.50%**	**73.26%**	**Stepwise Regression, Backward Elimination**	**88.42%**	**79.78%**
**840**	**14**	**90.07%**	**38.36%**	**83.56%**	**75.11%**	**Stepwise Regression, Backward Elimination**	**88.97%**	**80.32%**
**900**	**15**	**90.07%**	**41.07%**	**83.61%**	**75.29%**	**Stepwise Regression, Backward Elimination**	**88.38%**	**78.50%**
**960**	**16**	**90.41%**	**46.74%**	**82.36%**	**73.41%**	**Stepwise Regression, Backward Elimination**	**87.50%**	**76.02%**
1020	17	88.98%	40.50%	79.69%	69.21%	Stepwise Regression, Backward Elimination	80.67%	71.94%
1080	18	88.14%	39.35%	76.70%	64.54%	Stepwise Regression, Backward Elimination	79.01%	69.27%
1140	19	86.26%	32.71%	72.96%	58.91%	Stepwise Regression, Backward Elimination	76.83%	65.94%
1200	20	84.26%	26.09%	68.46%	52.21%	Stepwise Regression, Forward Information Criteria (AIC/BIC)	77.18%	63.26%
1260	21	80.70%	11.44%	61.12%	41.18%	Stepwise Regression, Backward Elimination	73.02%	56.37%
1320	22	76.83%	0.00%	52.24%	28.02%	Stepwise Regression, Backward Elimination	72.61%	46.24%
1380	23	72.89%	0.00%	42.70%	14.13%	Stepwise Regression, Backward Elimination	63.00%	40.66%
1440	24	69.74%	0.00%	32.77%	0.00%	Stepwise Regression, Backward Elimination	57.38%	31.85%

Table S11. Basic MLR models for prediction of mean % of carvedilol release, summary of model terms (Lac, HPMC_Visc, HPMC_HP, HPMC_PS): intercepts, regression coefficients, p-values of regression coefficients, and contribution %. Range of models used in interpretation of main effects and interaction effects of factors in terms of their influence on carvedilol release is bolded.

t (min)	t (h)	Intercept	Lac, Reg. Coef.	HPMC_Visc, Reg. Coef.	HPMC_HP, Reg. Coef.	HPMC_PS, Reg. Coef.	Lac, p-value	HPMC_Visc, p-value	HPMC_HP, p-value	HPMC_PS, p-value	Lac, Contribution %	HPMC_Visc, Contribution %	HPMC_HP, Contribution %	HPMC_PS, Contribution %
10	0.167	-8.78	10.49	0.000009	0.353	0.147	0.001	0.957	0.587	0.203	40.13%	0.43%	1.36%	4.21%
**20**	**0.333**	**-12.6**	**13.29**	**-0.000005**	**0.497**	**0.218**	**0**	**0.978**	**0.524**	**0.119**	**41.47%**	**0.88%**	**1.78%**	**5.97%**
**30**	**0.5**	**-13.7**	**14.96**	**-0.000016**	**0.61**	**0.244**	**0**	**0.936**	**0.461**	**0.101**	**43.87%**	**1.07%**	**2.13%**	**6.21%**
**45**	**0.75**	**-14**	**16.72**	**-0.000047**	**0.732**	**0.265**	**0**	**0.825**	**0.404**	**0.093**	**45.88%**	**1.54%**	**2.46%**	**6.17%**
**60**	**1**	**-12.9**	**18.16**	**-0.000081**	**0.801**	**0.272**	**0**	**0.716**	**0.381**	**0.097**	**47.84%**	**1.99%**	**2.53%**	**5.73%**
**90**	**1.5**	**-10.4**	**20.41**	**-0.000138**	**0.883**	**0.283**	**0**	**0.552**	**0.358**	**0.1**	**50.67%**	**2.83%**	**2.52%**	**5.20%**
**120**	**2**	**-8.1**	**22.29**	**-0.000178**	**0.95**	**0.289**	**0**	**0.46**	**0.339**	**0.105**	**53.03%**	**3.31%**	**2.50%**	**4.74%**
**150**	**2.5**	**-5.7**	**23.83**	**-0.000217**	**1.036**	**0.288**	**0**	**0.383**	**0.311**	**0.114**	**54.68%**	**3.75%**	**2.60%**	**4.27%**
**180**	**3**	**-3.3**	**25.17**	**-0.00025**	**1.11**	**0.286**	**0**	**0.321**	**0.282**	**0.121**	**56.41%**	**4.13%**	**2.70%**	**3.90%**
**210**	**3.5**	**-0.9**	**26.47**	**-0.000281**	**1.17**	**0.283**	**0**	**0.272**	**0.264**	**0.13**	**58.04%**	**4.42%**	**2.72%**	**3.53%**
**240**	**4**	**0.2**	**27.87**	**-0.000297**	**1.3**	**0.283**	**0**	**0.247**	**0.216**	**0.129**	**60.07%**	**4.42%**	**3.03%**	**3.30%**
**270**	**4.5**	**2.3**	**29.1**	**-0.000319**	**1.38**	**0.277**	**0**	**0.219**	**0.195**	**0.142**	**61.38%**	**4.52%**	**3.13%**	**2.96%**
**300**	**5**	**4.6**	**30.3**	**-0.000346**	**1.48**	**0.264**	**0**	**0.187**	**0.168**	**0.162**	**62.78%**	**4.65%**	**3.29%**	**2.55%**
**330**	**5.5**	**6.9**	**31.36**	**-0.000376**	**1.57**	**0.252**	**0**	**0.154**	**0.147**	**0.182**	**63.89%**	**4.88%**	**3.43%**	**2.21%**
**360**	**6**	**8.9**	**32.31**	**-0.0004**	**1.67**	**0.245**	**0**	**0.132**	**0.127**	**0.198**	**64.71%**	**5.03%**	**3.60%**	**1.98%**
**420**	**7**	**11.8**	**34.36**	**-0.000434**	**1.94**	**0.223**	**0**	**0.104**	**0.077**	**0.238**	**66.82%**	**4.97%**	**4.27%**	**1.50%**
**480**	**8**	**15.4**	**35.74**	**-0.000478**	**2.13**	**0.207**	**0**	**0.077**	**0.056**	**0.276**	**67.54%**	**5.26%**	**4.69%**	**1.21%**
**540**	**9**	**20.7**	**37.04**	**-0.000528**	**2.26**	**0.174**	**0**	**0.057**	**0.047**	**0.368**	**68.12%**	**5.52%**	**4.80%**	**0.80%**
**600**	**10**	**23.8**	**39.9**	**-0.000556**	**2.39**	**0.152**	**0**	**0.058**	**0.048**	**0.455**	**69.27%**	**5.11%**	**4.62%**	**0.54%**
**660**	**11**	**24.9**	**41.15**	**-0.000566**	**2.55**	**0.161**	**0**	**0.055**	**0.037**	**0.431**	**69.85%**	**5.05%**	**4.94%**	**0.57%**
**720**	**12**	**26.9**	**41.38**	**-0.000575**	**2.57**	**0.176**	**0**	**0.046**	**0.03**	**0.376**	**70.49%**	**5.31%**	**5.09%**	**0.68%**
**780**	**13**	**30.9**	**41.24**	**-0.000562**	**2.43**	**0.18**	**0**	**0.043**	**0.033**	**0.349**	**71.80%**	**5.28%**	**4.70%**	**0.73%**
**840**	**14**	**35**	**40.37**	**-0.000499**	**2.31**	**0.173**	**0**	**0.053**	**0.031**	**0.336**	**73.74%**	**4.55%**	**4.55%**	**0.72%**
**900**	**15**	**40.4**	**38.72**	**-0.000455**	**2.112**	**0.162**	**0**	**0.064**	**0.038**	**0.345**	**74.54%**	**4.19%**	**4.19%**	**0.69%**
**960**	**16**	**45.6**	**36.67**	**-0.000415**	**1.943**	**0.15**	**0**	**0.085**	**0.051**	**0.373**	**73.91%**	**3.86%**	**3.92%**	**0.66%**
1020	17	52.9	33.77	-0.000415	1.6	0.141	0	0.086	0.105	0.405	71.54%	4.40%	3.09%	0.67%
1080	18	57.3	31.06	-0.00038	1.301	0.156	0	0.116	0.185	0.359	68.86%	4.48%	2.43%	0.93%
1140	19	60.9	28.23	-0.000316	0.992	0.174	0	0.188	0.309	0.308	65.81%	4.05%	1.77%	1.34%
1200	20	64.1	25.14	-0.000254	0.714	0.193	0	0.284	0.459	0.257	61.64%	3.64%	1.24%	1.94%
1260	21	68.3	21.74	-0.000196	0.425	0.2	0	0.412	0.663	0.25	54.85%	3.13%	0.67%	2.47%
1320	22	71.9	18.11	-0.000139	0.21	0.203	0	0.561	0.83	0.244	46.25%	2.55%	0.33%	3.11%
1380	23	74.2	14.65	-0.000083	0.063	0.214	0.001	0.727	0.949	0.221	36.47%	1.93%	0.16%	4.13%
1440	24	76.3	11.51	-0.00004	-0.067	0.223	0.008	0.869	0.946	0.206	26.11%	1.43%	0.04%	5.20%

Table S12. Optimized MLR models for prediction of mean % of carvedilol release, summary of model terms: intercepts and regression coefficients. Range of models used in interpretation of main effects and interaction effects of factors in terms of their influence on carvedilol release is bolded.

t (min)	t (h)	Intercept	Lac, Reg. Coef.	HPMC_Visc, Reg. Coef.	HPMC_HP, Reg. Coef.	HPMC_PS, Reg. Coef.	Lac², Reg. Coef.	Lac·HPMC_Visc, Reg. Coef.	HPMC_Visc·HPMC_HP, Reg. Coef.	HPMC_Visc·HPMC_PS, Reg. Coef.
10	0.167	105	10.49	-0.00868		-1.46				0.000127
**20**	**0.333**	**107.4**	**45.2**	**-0.00911**		**-1.692**		**-0.00245**		**0.000151**
**30**	**0.5**	**110.8**	**49.5**	**-0.00941**		**-1.737**		**-0.00266**		**0.000156**
**45**	**0.75**	**115.8**	**53.9**	**-0.00978**		**-1.794**		**-0.00286**		**0.000163**
**60**	**1**	**118.4**	**58.1**	**-0.00989**		**-1.821**		**-0.00307**		**0.000166**
**90**	**1.5**	**123.9**	**63.2**	**-0.01015**		**-1.87**		**-0.0033**		**0.00017**
**120**	**2**	**130.2**	**67.4**	**-0.01045**		**-1.92**		**-0.00348**		**0.000175**
**150**	**2.5**	**137.1**	**71.5**	**-0.01079**		**-2**		**-0.00367**		**0.000181**
**180**	**3**	**141.9**	**73.9**	**-0.01097**		**-2.03**		**-0.00375**		**0.000184**
**210**	**3.5**	**146.3**	**76.7**	**-0.01112**		**-2.07**		**-0.00387**		**0.000187**
**240**	**4**	**146.8**	**80**	**-0.01101**		**-2.05**		**-0.00401**		**0.000186**
**270**	**4.5**	**152.8**	**82.8**	**-0.0113**		**-2.12**		**-0.00413**		**0.000191**
**300**	**5**	**154.3**	**85.1**	**-0.0112**		**-2.12**		**-0.00422**		**0.000189**
**330**	**5.5**	**159.4**	**87**	**-0.0114**		**-2.16**		**-0.00429**		**0.000192**
**360**	**6**	**166.4**	**88.3**	**-0.01176**		**-2.23**		**-0.00431**		**0.000197**
**420**	**7**	**140.1**	**93.6**	**-0.01043**	**1.83**	**-2.06**		**-0.00456**		**0.000179**
**480**	**8**	**150.3**	**95.2**	**-0.01099**	**2.017**	**-2.17**		**-0.00458**		**0.000187**
**540**	**9**	**164.5**	**94.9**	**-0.01174**	**2.139**	**-2.32**		**-0.00445**		**0.000196**
**600**	**10**	**178.8**	**98.9**	**-0.01264**	**2.263**	**-2.51**		**-0.00454**		**0.000209**
**660**	**11**	**168**	**104.2**	**-0.01172**	**2.42**	**-2.36**		**-0.00486**		**0.000198**
**720**	**12**	**154.6**	**106.7**	**-0.01053**	**2.46**	**-2.14**		**-0.00503**		**0.000182**
**780**	**13**	**156.7**	**103.4**	**-0.01036**	**2.321**	**-2.08**		**-0.00479**		**0.000178**
**840**	**14**	**153.9**	**97.1**	**-0.00977**	**2.206**	**-1.95**		**-0.00437**		**0.000167**
**900**	**15**	**155.7**	**87.4**	**-0.00944**	**2.012**	**-1.85**		**-0.00375**		**0.000158**
**960**	**16**	**164**	**83.4**	**-0.00964**	**1.842**	**-1.9**		**-0.0036**		**0.000161**
1020	17	222.4	33.77	-0.01272		-2.11				0.00018
1080	18	227.9	31.06	-0.01295		-2.15				0.000184
1140	19	233.2	28.23	-0.0132		-2.2				0.000188
1200	20	228.4	67.9	-0.01346		-2.25	-42.7			0.000193
1260	21	232.4	68.1	-0.01362		-2.29	-46.4			0.000196
1320	22	289.9	68.2	-0.01788	-8.8	-1.92	-50		0.000678	0.000166
1380	23	240	66.5	-0.01396		-2.365	-51.9			0.000202
1440	24	243.2	65.3	-0.01411		-2.395	-53.8			0.000205

Table S13. Optimized MLR models for prediction of mean % of carvedilol release, summary of model terms: p-values of model terms (p-values ≤ 0.05 are coloured dark red). Range of models used in interpretation of main effects and interaction effects of factors in terms of their influence on carvedilol release is bolded.

t (min)	t (h)	Lac, p-value	HPMC_Visc, p-value	HPMC_HP, p-value	HPMC_PS, p-value	Lac², p-value	Lac·HPMC_Visc, p-value	HPMC_Visc·HPMC_HP, p-value	HPMC_Visc·HPMC_PS, p-value
10	0.17	0	0.348		0.031				0.033
**20**	**0.333**	0	0.373		0.012		0.097		0.029
**30**	**0.5**	0	0.41		0.01		0.091		0.033
**45**	**0.75**	0	0.505		0.01		0.089		0.037
**60**	**1**	0	0.615		0.011		0.08		0.041
**90**	**1.5**	0	0.806		0.012		0.076		0.046
**120**	**2**	0	0.925		0.012		0.07		0.046
**150**	**2.5**	0	0.968		0.013		0.063		0.045
**180**	**3**	0	0.872		0.014		0.061		0.045
**210**	**3.5**	0	0.787		0.015		0.056		0.044
**240**	**4**	0	0.745		0.015		0.049		0.045
**270**	**4.5**	0	0.698		0.016		0.045		0.042
**300**	**5**	0	0.628		0.019		0.044		0.046
**330**	**5.5**	0	0.56		0.022		0.042		0.044
**360**	**6**	0	0.518		0.023		0.042		0.04
**420**	**7**	0	0.303	0.055	0.047		0.026		0.051
**480**	**8**	0	0.241	0.036	0.053		0.025		0.043
**540**	**9**	0	0.193	0.031	0.079		0.033		0.038
**600**	**10**	0	0.203	0.032	0.11		0.041		0.039
**660**	**11**	0	0.179	0.024	0.111		0.03		0.05
**720**	**12**	0	0.137	0.018	0.096		0.021		0.06
**780**	**13**	0	0.131	0.02	0.084		0.022		0.057
**840**	**14**	0	0.165	0.019	0.081		0.026		0.058
**900**	**15**	0	0.203	0.027	0.093		0.049		0.065
**960**	**16**	0	0.269	0.039	0.1		0.054		0.058
1020	17	0	0.425		0.083				0.055
1080	18	0	0.495		0.068				0.046
1140	19	0	0.662		0.052				0.039
1200	20	0	0.835		0.033	0.106			0.024
1260	21	0	0.97		0.032	0.081			0.022
1320	22	0	0.533	0.66	0.041	0.053		0.093	0.046
1380	23	0	0.573		0.024	0.046			0.015
1440	24	0.002	0.441		0.021	0.039			0.014

Table S14. Optimized MLR models for prediction of mean % of carvedilol release, summary of model terms: contribution % of model terms. Range of models used in interpretation of main effects and interaction effects of factors in terms of their influence on carvedilol release is bolded.

t (min)	t (h)	Lac, Contribution %	HPMC_Visc, Contribution %	HPMC_HP, Contribution %	HPMC_PS, Contribution %	Lac^2, Contribution %	Lac·HPMC_Visc, Contribution %	HPMC_Visc·HPMC_HP, Contribution %	HPMC_Visc·HPMC_PS, Contribution %
10	0.167	40.13%	0.43%		4.82%				10.44%
**20**	**0.333**	**41.47%**	**0.88%**		**6.79%**		**5.19%**		**9.50%**
**30**	**0.5**	**43.87%**	**1.07%**		**7.15%**		**5.11%**		**8.55%**
**45**	**0.75**	**45.88%**	**1.54%**		**7.19%**		**4.95%**		**7.76%**
**60**	**1**	**47.84%**	**1.99%**		**6.73%**		**5.03%**		**7.09%**
**90**	**1.5**	**50.67%**	**2.83%**		**6.15%**		**4.85%**		**6.27%**
**120**	**2**	**53.03%**	**3.31%**		**5.66%**		**4.74%**		**5.85%**
**150**	**2.5**	**54.68%**	**3.75%**		**5.17%**		**4.77%**		**5.63%**
**180**	**3**	**56.41%**	**4.13%**		**4.78%**		**4.61%**		**5.37%**
**210**	**3.5**	**58.04%**	**4.42%**		**4.38%**		**4.55%**		**5.14%**
**240**	**4**	**60.07%**	**4.42%**		**4.18%**		**4.57%**		**4.75%**
**270**	**4.5**	**61.38%**	**4.52%**		**3.82%**		**4.55%**		**4.69%**
**300**	**5**	**62.78%**	**4.65%**		**3.37%**		**4.47%**		**4.37%**
**330**	**5.5**	**63.89%**	**4.88%**		**3.00%**		**4.39%**		**4.28%**
**360**	**6**	**64.71%**	**5.03%**		**2.76%**		**4.23%**		**4.31%**
**420**	**7**	**66.82%**	**4.97%**	**4.27%**	**1.50%**		**4.33%**		**3.22%**
**480**	**8**	**67.54%**	**5.26%**	**4.69%**	**1.21%**		**4.08%**		**3.27%**
**540**	**9**	**68.12%**	**5.52%**	**4.80%**	**0.80%**		**3.62%**		**3.38%**
**600**	**10**	**69.27%**	**5.11%**	**4.62%**	**0.54%**		**3.30%**		**3.38%**
**660**	**11**	**69.85%**	**5.05%**	**4.94%**	**0.57%**		**3.58%**		**2.87%**
**720**	**12**	**70.49%**	**5.31%**	**5.09%**	**0.68%**		**3.83%**		**2.42%**
**780**	**13**	**71.80%**	**5.28%**	**4.70%**	**0.73%**		**3.56%**		**2.37%**
**840**	**14**	**73.74%**	**4.55%**	**4.55%**	**0.72%**		**3.17%**		**2.24%**
**900**	**15**	**74.54%**	**4.19%**	**4.19%**	**0.69%**		**2.56%**		**2.21%**
**960**	**16**	**73.91%**	**3.86%**	**3.92%**	**0.66%**		**2.62%**		**2.52%**
1020	17	71.54%	4.40%		1.12%				3.61%
1080	18	68.86%	4.48%		1.38%				4.28%
1140	19	65.81%	4.05%		1.78%				5.20%
1200	20	61.64%	3.64%		2.36%	3.09%			6.45%
1260	21	54.85%	3.13%		2.79%	4.33%			7.92%
1320	22	46.25%	2.55%	0.33%	3.11%	6.04%		7.78%	6.55%
1380	23	36.47%	1.93%		4.28%	7.94%			12.37%
1440	24	26.11%	1.43%		5.23%	9.89%			14.73%

Table S15. R^2 and R^2_{pred} for full RSM MLR models and MLR models generated via stepwise regression procedures for predicting SD of carvedilol release. Range of models used in interpretation of main effects and interaction effects of factors in terms of their influence on carvedilol release is bolded. In cases where no stepwise MLR model could be created by Minitab®, “N/A” is shown in the table.

t (min)	t (h)	Full RSM (CCD) MLR model, R^2	Full RSM (CCD) MLR model, R^2_{pred}	Stepwise Regression, Forward, Information Criterion (AIC), R^2	Stepwise Regression, Forward, Information Criterion (AIC), R^2_{pred}	Stepwise Regression, Forward, Information Criterion (BIC), R^2	Stepwise Regression, Forward, Information Criterion (BIC), R^2_{pred}	Stepwise Regression, Stepwise, R^2	Stepwise Regression, Stepwise, R^2_{pred}	Stepwise Regression, Forward Selection, R^2	Stepwise Regression, Forward Selection, R^2_{pred}	Stepwise Regression, Backward Elimination, R^2	Stepwise Regression, Backward Elimination, R^2predicted
10	0.167	65.86%	0.00%	54.69%	32.21%	54.69%	32.21%	54.69%	32.21%	54.69%	32.21%	54.69%	32.21%
**20**	**0.333**	**66.18%**	**0.00%**	**52.49%**	**30.28%**	**52.49%**	**30.28%**	**52.49%**	**30.28%**	**52.49%**	**30.28%**	**52.49%**	**30.28%**
**30**	**0.5**	**66.22%**	**0.00%**	**51.82%**	**30.88%**	**51.82%**	**30.88%**	**51.82%**	**30.88%**	**51.82%**	**30.88%**	**58.56%**	**34.43%**
**45**	**0.75**	**66.02%**	**0.00%**	**50.82%**	**30.58%**	**50.82%**	**30.58%**	**50.82%**	**30.58%**	**50.82%**	**30.58%**	**58.14%**	**33.73%**
**60**	**1**	**65.40%**	**0.00%**	**49.72%**	**29.27%**	**49.72%**	**29.27%**	**49.72%**	**29.27%**	**49.72%**	**29.27%**	**49.72%**	**29.27%**
**90**	**1.5**	**63.79%**	**0.00%**	**36.21%**	**25.00%**	**36.21%**	**25.00%**	**36.21%**	**25.00%**	**47.40%**	**26.55%**	**36.21%**	**25.00%**
**120**	**2**	**63.19%**	**0.00%**	**35.81%**	**24.79%**	**35.81%**	**24.79%**	**35.81%**	**24.79%**	**35.81%**	**24.79%**	**35.81%**	**24.79%**
**150**	**2.5**	**62.88%**	**0.00%**	**35.21%**	**24.17%**	**35.21%**	**24.17%**	**35.21%**	**24.17%**	**46.69%**	**21.98%**	**35.21%**	**24.17%**
**180**	**3**	**63.03%**	**0.00%**	**34.76%**	**23.71%**	**34.76%**	**23.71%**	**34.76%**	**23.71%**	**43.51%**	**23.51%**	**34.76%**	**23.71%**
**210**	**3.5**	**63.75%**	**0.00%**	**34.71%**	**23.58%**	**34.71%**	**23.58%**	**34.71%**	**23.58%**	**45.39%**	**26.17%**	**34.71%**	**23.58%**
**240**	**4**	**65.26%**	**0.00%**	**32.85%**	**21.20%**	**32.85%**	**21.20%**	**32.85%**	**21.20%**	**45.03%**	**25.00%**	**45.03%**	**25.00%**
**270**	**4.5**	**66.58%**	**0.00%**	**33.08%**	**21.31%**	**33.08%**	**21.31%**	**33.08%**	**21.31%**	**46.30%**	**26.45%**	**46.30%**	**26.45%**
**300**	**5**	**67.39%**	**0.00%**	**48.81%**	**29.91%**	**34.85%**	**23.29%**	**34.85%**	**23.29%**	**48.81%**	**29.91%**	**48.81%**	**29.91%**
**330**	**5.5**	**68.22%**	**0.00%**	**49.51%**	**30.50%**	**49.51%**	**30.50%**	**49.51%**	**30.50%**	**49.51%**	**30.50%**	**49.51%**	**30.50%**
**360**	**6**	**68.65%**	**0.00%**	**49.11%**	**29.49%**	**49.11%**	**29.49%**	**49.11%**	**29.49%**	**49.11%**	**29.49%**	**49.11%**	**29.49%**
**420**	**7**	**68.68%**	**0.00%**	**48.02%**	**27.08%**	**48.02%**	**27.08%**	**48.02%**	**27.08%**	**48.02%**	**27.08%**	**48.02%**	**27.08%**
**480**	**8**	**66.16%**	**0.00%**	**46.72%**	**25.54%**	**46.72%**	**25.54%**	**46.72%**	**25.54%**	**46.72%**	**25.54%**	**46.72%**	**25.54%**
**540**	**9**	**66.96%**	**0.00%**	**51.76%**	**32.53%**	**51.76%**	**32.53%**	**51.76%**	**32.53%**	**51.76%**	**32.53%**	**51.76%**	**32.53%**
**600**	**10**	**70.74%**	**0.00%**	**58.85%**	**30.83%**	**58.85%**	**30.83%**	**58.85%**	**30.83%**	**61.92%**	**31.39%**	**58.85%**	**30.83%**
**660**	**11**	**73.48%**	**0.00%**	**59.62%**	**35.29%**	**59.62%**	**35.29%**	**64.12%**	**38.86%**	**64.12%**	**38.86%**	**59.62%**	**35.29%**
**720**	**12**	**72.50%**	**0.00%**	**60.60%**	**40.21%**	**60.60%**	**40.21%**	**60.60%**	**40.21%**	**60.60%**	**40.21%**	**60.60%**	**40.21%**
**780**	**13**	**70.92%**	**0.00%**	**52.83%**	**35.96%**	**58.80%**	**41.09%**	**58.80%**	**41.09%**	**58.80%**	**41.09%**	**58.80%**	**41.09%**
**840**	**14**	**65.98%**	**0.00%**	**53.10%**	**36.25%**	**53.10%**	**36.25%**	**53.10%**	**36.25%**	**57.01%**	**37.83%**	**53.10%**	**36.25%**
900	15	58.37%	0.00%	39.17%	14.13%	39.17%	14.13%	12.63%	1.03%	12.63%	1.03%	39.17%	14.13%
960	16	55.07%	0.00%	39.60%	0.00%	14.90%	3.61%	14.90%	3.61%	14.90%	3.61%	33.43%	0.00%
1020	17	51.74%	0.00%	N/A	N/A	N/A	N/A	8.83%	0.00%	8.83%	0.00%	47.67%	0.00%
1080	18	48.00%	0.00%	N/A	N/A	N/A	N/A	N/A	N/A	7.32%	0.00%	13.10%	0.00%
1140	19	42.17%	0.00%	N/A	N/A	N/A	N/A	8.84%	0.00%	19.65%	0.00%	N/A	N/A
1200	20	44.54%	0.00%	26.44%	4.69%	26.44%	4.69%	26.44%	4.69%	31.43%	5.06%	26.44%	4.69%
1260	21	41.82%	0.00%	29.81%	10.18%	20.82%	5.25%	29.81%	10.18%	29.81%	10.18%	29.81%	10.18%
1320	22	39.67%	0.00%	18.83%	3.00%	18.83%	3.00%	18.83%	3.00%	29.80%	4.51%	18.83%	3.00%
1380	23	41.89%	0.00%	18.10%	2.44%	18.10%	2.44%	18.10%	2.44%	18.10%	2.44%	18.10%	2.44%
1440	24	44.30%	0.00%	13.89%	0.00%	13.89%	0.00%	13.89%	0.00%	13.89%	0.00%	13.89%	0.00%

Table S16. R^2 and R^2_{pred} of full RSM MLR models and selected Optimized MLR models generated via stepwise regression procedures for predicting SD of carvedilol release. Range of models used in interpretation of main effects and interaction effects of factors in terms of their influence on carvedilol release is bolded.

t (min)	t (h)	RSM (CCD) full model, R^2	RSM (CCD) full model, R^2_{pred}	Stepwise regression procedure used in the Optimized MLR model	Optimized MLR model, R^2	Optimized MLR model, R^2_{pred}
10	0.167	65.86%	0.00%	Stepwise Regression, Backward Elimination	54.69%	32.21%
**20**	**0.333**	**66.18%**	**0.00%**	**Stepwise Regression, Backward Elimination**	**52.49%**	**30.28%**
**30**	**0.5**	**66.22%**	**0.00%**	**Stepwise Regression, Backward Elimination**	**58.56%**	**34.43%**
**45**	**0.75**	**66.02%**	**0.00%**	**Stepwise Regression, Backward Elimination**	**58.14%**	**33.73%**
**60**	**1**	**65.40%**	**0.00%**	**Stepwise Regression, Backward Elimination**	**49.72%**	**29.27%**
**90**	**1.5**	**63.79%**	**0.00%**	**Stepwise Regression, Forward Selection**	**47.40%**	**26.55%**
**120**	**2**	**63.19%**	**0.00%**	**Stepwise Regression, Backward Elimination**	**35.81%**	**24.79%**
**150**	**2.5**	**62.88%**	**0.00%**	**Stepwise Regression, Backward Elimination**	**35.21%**	**24.17%**
**180**	**3**	**63.03%**	**0.00%**	**Stepwise Regression, Backward Elimination**	**34.76%**	**23.71%**
**210**	**3.5**	**63.75%**	**0.00%**	**Stepwise Regression, Forward Selection**	**45.39%**	**26.17%**
**240**	**4**	**65.26%**	**0.00%**	**Stepwise Regression, Backward Elimination**	**45.03%**	**25.00%**
**270**	**4.5**	**66.58%**	**0.00%**	**Stepwise Regression, Backward Elimination**	**46.30%**	**26.45%**
**300**	**5**	**67.39%**	**0.00%**	**Stepwise Regression, Backward Elimination**	**48.81%**	**29.91%**
**330**	**5.5**	**68.22%**	**0.00%**	**Stepwise Regression, Backward Elimination**	**49.51%**	**30.50%**
**360**	**6**	**68.65%**	**0.00%**	**Stepwise Regression, Backward Elimination**	**49.11%**	**29.49%**
**420**	**7**	**68.68%**	**0.00%**	**Stepwise Regression, Backward Elimination**	**48.02%**	**27.08%**
**480**	**8**	**66.16%**	**0.00%**	**Stepwise Regression, Backward Elimination**	**46.72%**	**25.54%**
**540**	**9**	**66.96%**	**0.00%**	**Stepwise Regression, Backward Elimination**	**51.76%**	**32.53%**
**600**	**10**	**70.74%**	**0.00%**	**Stepwise Regression, Forward Selection**	**61.92%**	**31.39%**
**660**	**11**	**73.48%**	**0.00%**	**Stepwise Regression, Forward Selection**	**64.12%**	**38.86%**
**720**	**12**	**72.50%**	**0.00%**	**Stepwise Regression, Forward Selection**	**60.60%**	**40.21%**
**780**	**13**	**70.92%**	**0.00%**	**Stepwise Regression, Forward Selection**	**58.80%**	**41.09%**
**840**	**14**	**65.98%**	**0.00%**	**Stepwise Regression, Forward Selection**	**57.01%**	**37.83%**
900	15	58.37%	0.00%	Stepwise Regression, Backward Elimination	39.17%	14.13%
960	16	55.07%	0.00%	Stepwise Regression, Forward Selection	14.90%	3.61%
1020	17	51.74%	0.00%	Stepwise Regression, Backward Elimination	47.67%	0.00%
1080	18	48.00%	0.00%	Stepwise Regression, Forward Selection	7.32%	0.00%
1140	19	42.17%	0.00%	Stepwise Regression, Forward Selection	19.65%	0.00%
1200	20	44.54%	0.00%	Stepwise Regression, Forward Selection	31.43%	5.06%
1260	21	41.82%	0.00%	Stepwise Regression, Forward Selection	29.81%	10.18%
1320	22	39.67%	0.00%	Stepwise Regression, Forward Selection	29.80%	4.51%
1380	23	41.89%	0.00%	Stepwise Regression, Forward Selection	18.10%	2.44%
1440	24	44.30%	0.00%	Stepwise Regression, Forward Selection	13.89%	0.00%

Table S17. Optimized MLR models for prediction of SD of carvedilol release, summary of model terms: intercepts and regression coefficients. Range of models used in interpretation of main effects and interaction effects of factors in terms of their influence on carvedilol release is bolded.

t (min)	t (h)	Intercept	Lac, Reg. Coef.	HPMC_Visc, Reg. Coef.	HPMC_HP, Reg. Coef.	HPMC_PS, Reg. Coef.	Lac², Reg. Coef.	HPMC_HP², Reg. Coef.	HPMC_PS², Reg. Coef.	Lac·HPMC_Visc, Reg. Coef.	Lac·HPMC_HP, Reg. Coef.	HPMC_Visc·HPMC_HP, Reg. Coef.	HPMC_HP·HPMC_PS, Reg. Coef.
10	0.167	-3.81	4.1	-0.000571			18.66			-0.001378			
**20**	**0.333**	**-3.42**	**4**	**0.00059**			**19.41**			**-0.001464**			
**30**	**0.5**	**-203.6**	**23.46**	**0.000576**	**18.9**	**3.08**		**-0.993**	**-0.0223**	**-0.001472**			
**45**	**0.75**	**-225.8**	**24.3**	**0.000603**	**21.1**	**3.4**		**-1.107**	**-0.0246**	**-0.001517**			
**60**	**1**	**-1.9**	**-2.4**	**0.000608**			**26.5**			**-0.001489**			
**90**	**1.5**	**-1.05**	**-6.4**	**0.000621**			**30.9**			**-0.001493**			
**120**	**2**	**7.67**	**-28.3**				**33.6**						
**150**	**2.5**	**8.19**	**-30.2**				**35.5**						
**180**	**3**	**8.52**	**-31.4**				**36.9**						
**210**	**3.5**	**36**	**-99.5**		**-2.92**		**37.6**				**7.21**		
**240**	**4**	**43.5**	**-113.6**		**-3.68**		**38.4**				**8.63**		
**270**	**4.5**	**44.4**	**-117.1**		**-3.74**		**39.4**				**8.87**		
**300**	**5**	**44.1**	**-117.6**		**-3.71**		**39.8**				**8.92**		
**330**	**5.5**	**44.8**	**-120**		**-3.77**		**40.4**				**9.12**		
**360**	**6**	**47.8**	**-126.8**		**-4.06**		**40.9**				**9.77**		
**420**	**7**	**51.9**	**-136.4**		**-4.44**		**42.3**				**10.61**		
**480**	**8**	**48.7**	**-133.4**		**-4.03**		**43.8**				**10.1**		
**540**	**9**	**44.5**	**-131.4**		**-3.43**		**50.1**				**9.24**		
**600**	**10**	**56.4**	**-163.4**	**-0.000172**	**-3.95**		**73.2**				**10.36**		
**660**	**11**	**60**	**-173.4**	**-0.000218**	**-4.21**		**76.1**				**11.15**		
**720**	**12**	**56.7**	**-161.4**	**-0.000245**	**-4.02**		**66.1**				**10.82**		
**780**	**13**	**46.4**	**-133.6**	**-0.000242**	**-3.13**		**55.8**				**8.84**		
**840**	**14**	**32.3**	**-101.5**	**-0.000186**	**-1.84**		**48.2**				**6.11**		
900	15	2.1	-38.6		1.013		41.1						
960	16	-6.27			0.989								
1020	17	368	-45.8	-0.00588	-36.5	-4.19	23.7			0.001694		0.000529	0.439
1080	18	-3.11			0.604								
1140	19	2.52	-1.86		0.645	-0.0773							
1200	20	2.47	-3.27		0.655	-0.0719							
1260	21	-1.86	-3.6		0.578								
1320	22	-18.3	31.1		2.31						-3.68		
1380	23	3.026	-2.92										
1440	24	2.716	-2.42										

Table S18. Optimized MLR models for prediction of SD of carvedilol release, summary of model terms: p-values of model terms (p-values ≤ 0.05 are coloured dark red). Range of models used in interpretation of main effects and interaction effects of factors in terms of their influence on carvedilol release is bolded.

t (min)	t (h)	Lac, p-value	HPMC_Visc, p-value	HPMC_HP, p-value	HPMC_PS, p-value	Lac², p-value	HPMC_HP², p-value	HPMC_PS², p-value	Lac·HPMC_Visc, p-value	Lac·HPMC_HP, p-value	HPMC_Visc·HPMC_HP, p-value	HPMC_HP·HPMC_PS, p-value
10	0.167	0.004	0.107			0.061			0.045			
20	0.333	0.013	0.064			0.059			0.04			
30	0.5	0.017	0.06	0.745	0.641		0.092	0.062	0.046			
45	0.75	0.017	0.082	0.739	0.66		0.078	0.054	0.053			
60	1	0.018	0.121			0.03			0.072			
90	1.5	0.021	0.195			0.024			0.103			
120	2	0.01				0.027						
150	2.5	0.012				0.025						
180	3	0.014				0.025						
210	3.5	0.005		0.171		0.021				0.143		
240	4	0.006		0.21		0.02				0.087		
270	4.5	0.006		0.172		0.017				0.078		
300	5	0.004		0.134		0.014				0.072		
330	5.5	0.004		0.113		0.013				0.065		
360	6	0.005		0.106		0.014				0.054		
420	7	0.007		0.105		0.014				0.046		
480	8	0.014		0.067		0.014				0.063		
540	9	0.01		0.033		0.005				0.084		
600	10	0.004	0.207	0.044		0.001				0.079		
660	11	0.003	0.119	0.029		0				0.064		
720	12	0.008	0.078	0.025		0.001				0.069		
780	13	0.015	0.055	0.021		0.002				0.096		
840	14	0.028	0.087	0.014		0.002				0.182		
900	15	0.193		0.038		0.009						
960	16			0.047								
1020	17	0.646	0.853	0.019	0.735	0.086			0.076		0.071	0.077
1080	18			0.172								
1140	19	0.232		0.098	0.22							
1200	20	0.026		0.065	0.208							
1260	21	0.013		0.092								
1320	22	0.012		0.152						0.248		
1380	23	0.027										
1440	24	0.056										

Table S19. Optimized MLR models for prediction of SD of carvedilol release, summary of model terms: contribution % of model terms. Range of models used in interpretation of main effects and interaction effects of factors in terms of their influence on carvedilol release is bolded.

t (min)	t (h)	Lac, Contribution %	HPMC_Visc, Contribution %	HPMC_HP, Contribution %	HPMC_PS, Contribution %	Lac^2, Contribution %	HPMC_HP^2, Contribution %	HPMC_PS^2, Contribution %	Lac·HPMC_Visc, Contribution %	Lac·HPMC_HP, Contribution %	HPMC_Visc·HPMC_HP, Contribution %	HPMC_HP·HPMC_PS, Contribution %
10	0.167	31.44%	5.95%			8.03%			9.27%			
**20**	**0.333**	**25.22%**	**8.34%**			**8.59%**			**10.34%**			
**30**	**0.5**	**23.61%**	**8.90%**	**2.25%**	**0.21%**		**5.12%**	**8.55%**	**9.92%**			
**45**	**0.75**	**23.56%**	**7.54%**	**2.48%**	**0.17%**		**5.69%**	**9.32%**	**9.38%**			
**60**	**1**	**23.18%**	**6.11%**			**12.24%**			**8.19%**			
**90**	**1.5**	**22.09%**	**4.40%**			**13.98%**			**6.92%**			
**120**	**2**	**21.04%**				**14.77%**						
**150**	**2.5**	**19.87%**				**15.35%**						
**180**	**3**	**19.27%**				**15.48%**						
**210**	**3.5**	**19.37%**		**4.89%**		**15.42%**				**5.71%**		
**240**	**4**	**17.23%**		**4.09%**		**15.70%**				**8.01%**		
**270**	**4.5**	**16.81%**		**4.79%**		**16.35%**				**8.35%**		
**300**	**5**	**18.50%**		**5.53%**		**16.44%**				**8.34%**		
**330**	**5.5**	**17.89%**		**6.16%**		**16.81%**				**8.65%**		
**360**	**6**	**16.41%**		**6.50%**		**16.62%**				**9.59%**		
**420**	**7**	**14.05%**		**6.67%**		**16.70%**				**10.60%**		
**480**	**8**	**11.33%**		**8.91%**		**17.23%**				**9.25%**		
**540**	**9**	**12.24%**		**11.26%**		**21.05%**				**7.20%**		
**600**	**10**	**14.23%**	**2.81%**	**8.20%**		**30.52%**				**6.17%**		
**660**	**11**	**14.14%**	**4.15%**	**9.24%**		**30.08%**				**6.51%**		
**720**	**12**	**11.49%**	**5.93%**	**10.76%**		**25.52%**				**6.90%**		
**780**	**13**	**9.75%**	**7.52%**	**12.04%**		**23.52%**				**5.97%**		
**840**	**14**	**8.51%**	**5.96%**	**14.50%**		**14.50%**				**3.91%**		
900	15	4.77%		12.63%		21.77%						
960	16			14.90%								
1020	17	0.00%	0.68%	9.06%	5.42%	9.75%			10.32%		2.18%	10.26%
1080	18			7.32%								
1140	19	5.25%		8.84%	5.56%							
1200	20	16.79%		9.65%	5.00%							
1260	21	20.82%		8.99%								
1320	22	18.83%		6.69%						4.29%		
1380	23	18.10%										
1440	24	13.89%										

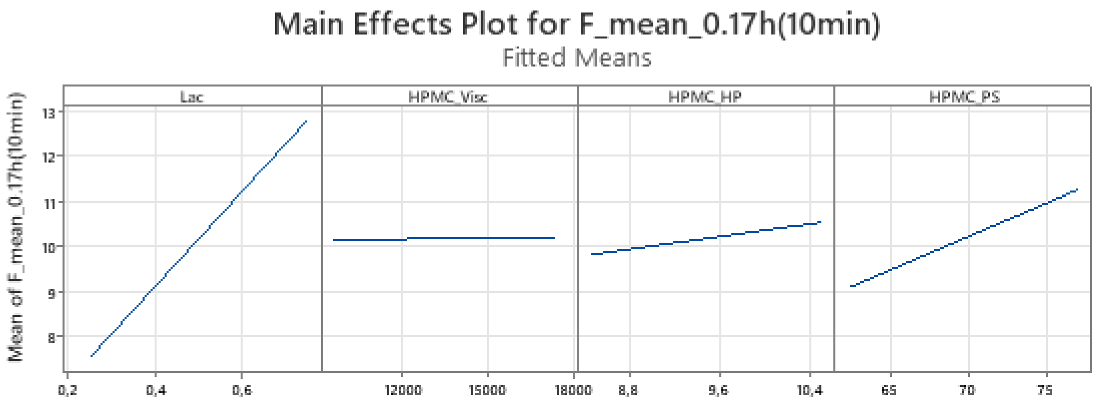

Figure S1. Main effects plot for mean % of carvedilol release using a Basic MLR model at t = 0.17 h (10 min).

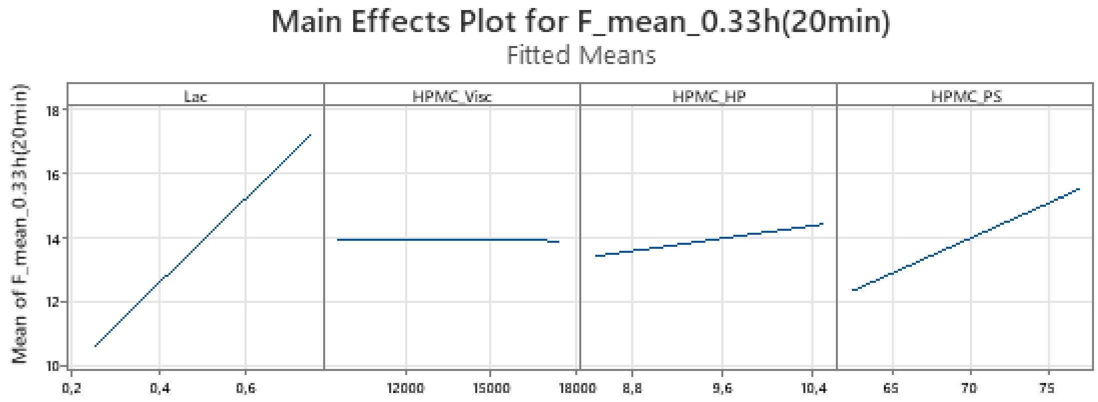

Figure S2. Main effects plot for mean % of carvedilol release using a Basic MLR model at t = 0.33 h (20 min).

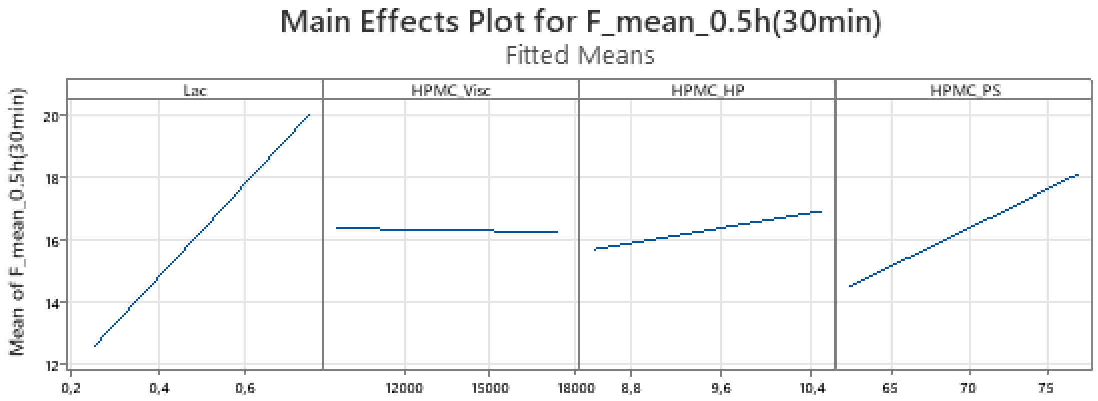

Figure S3. Main effects plot for mean % of carvedilol release using a Basic MLR model at t = 0.5 h (30 min).

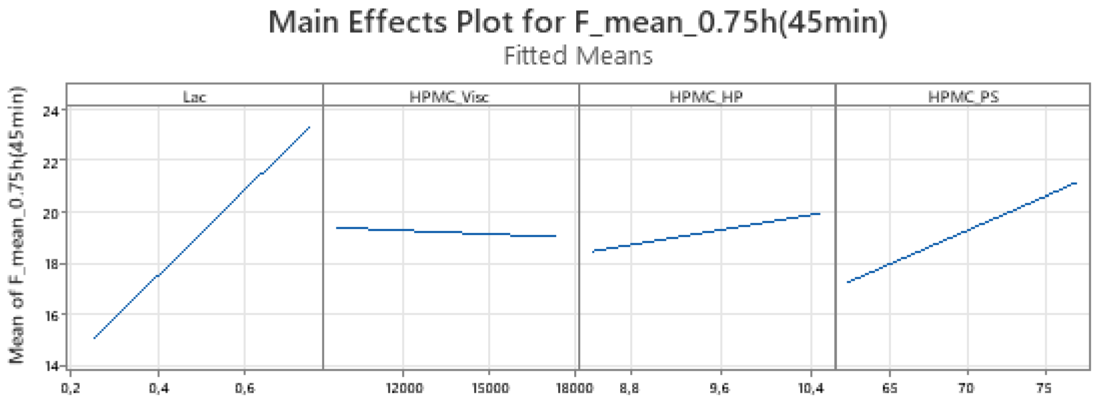

Figure S4. Main effects plot for mean % of carvedilol release using a Basic MLR model at t = 0.75 h (45 min).

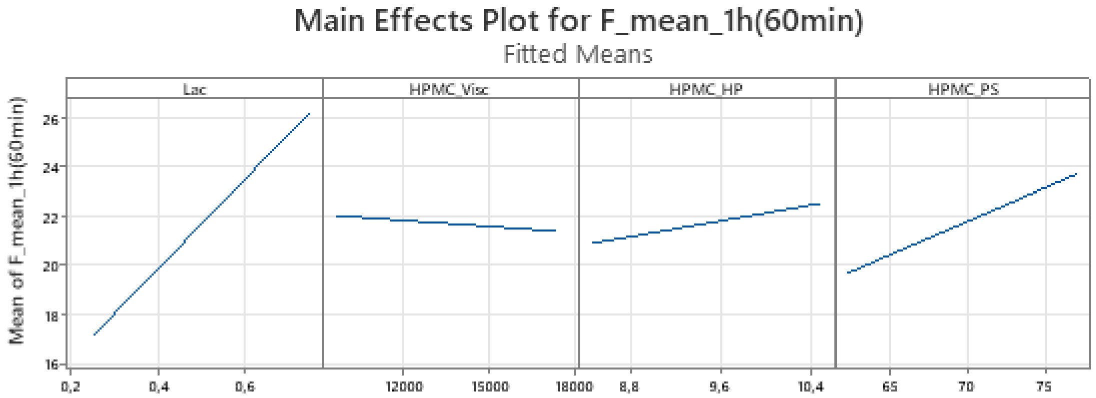

Figure S5. Main effects plot for mean % of carvedilol release using a Basic MLR model at t = 1 h (60 min).

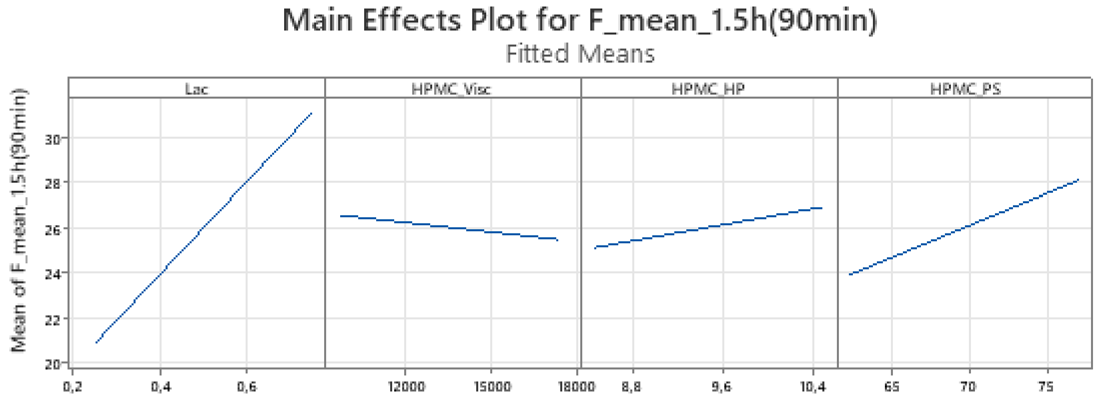

Figure S6. Main effects plot for mean % of carvedilol release using a Basic MLR model at t = 1.5 h (90 min).

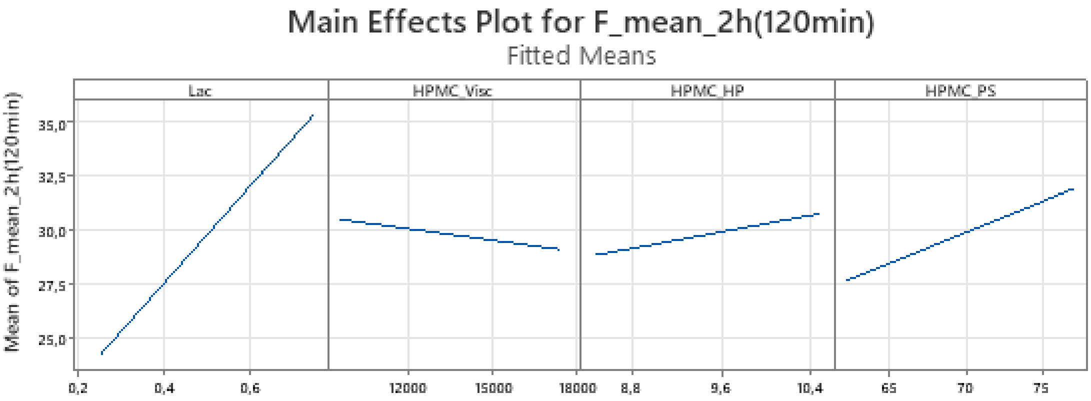

Figure S7. Main effects plot for mean % of carvedilol release using a Basic MLR model at t = 2 h (120 min).

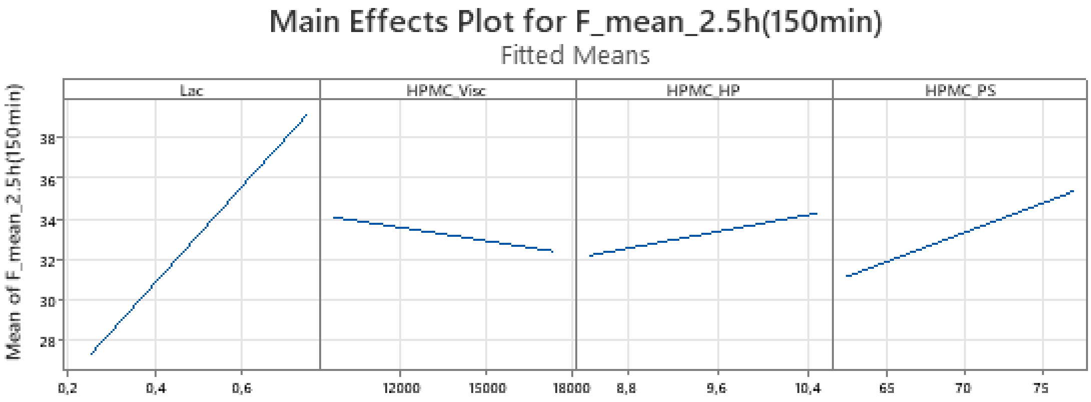

Figure S8. Main effects plot for mean % of carvedilol release using a Basic MLR model at t = 2.5 h (150 min).

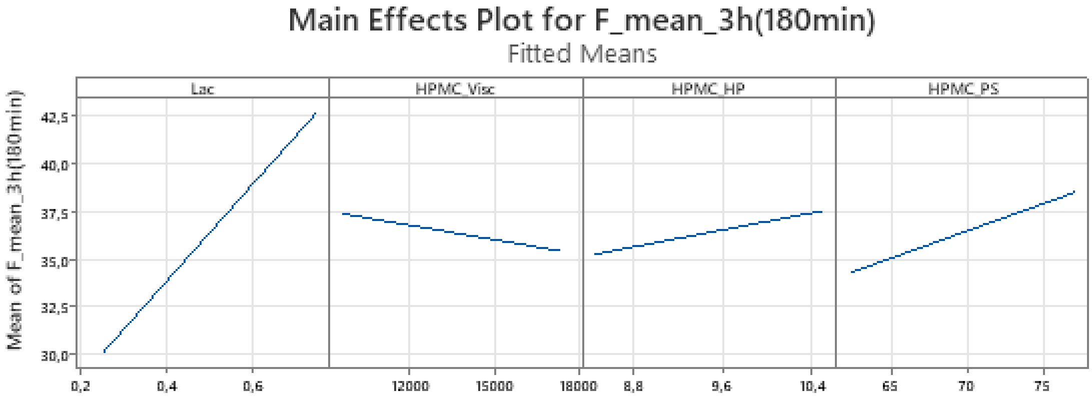

Figure S9. Main effects plot for mean % of carvedilol release using a Basic MLR model at t = 3 h (180 min).

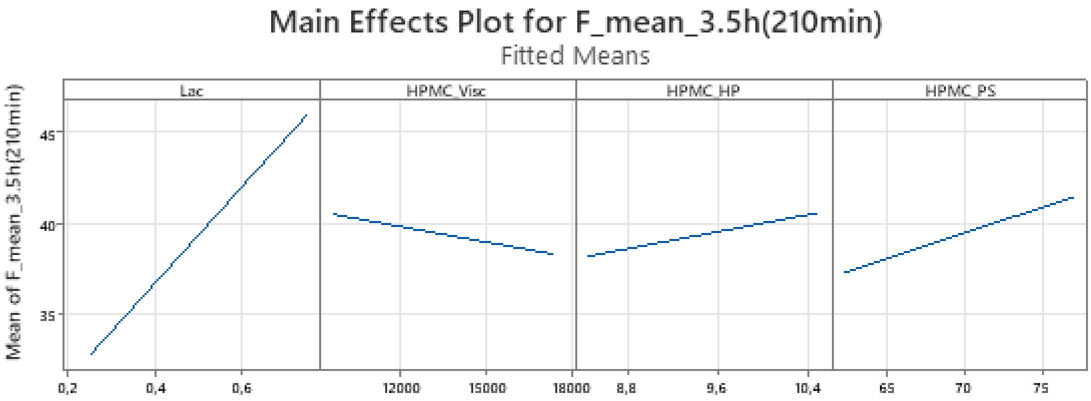

Figure S10. Main effects plot for mean % of carvedilol release using a Basic MLR model at t = 3.5 h (210 min).

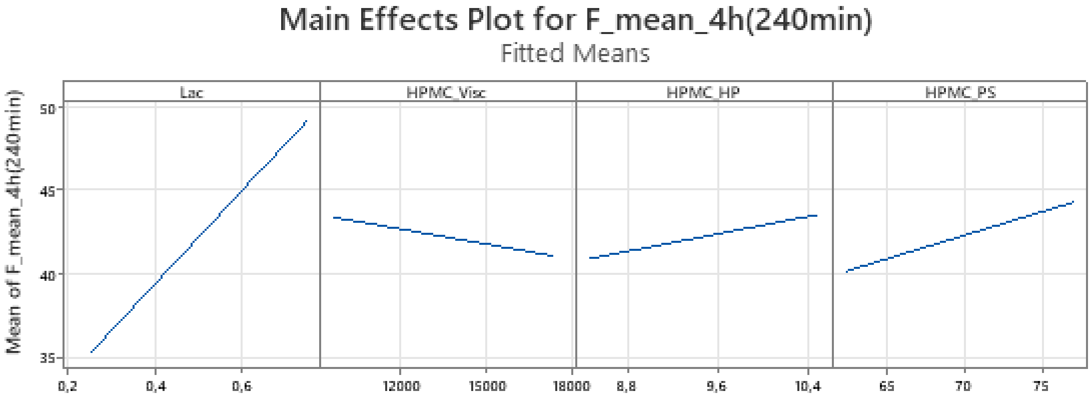

Figure S11. Main effects plot for mean % of carvedilol release using a Basic MLR model at t = 4 h (240 min).

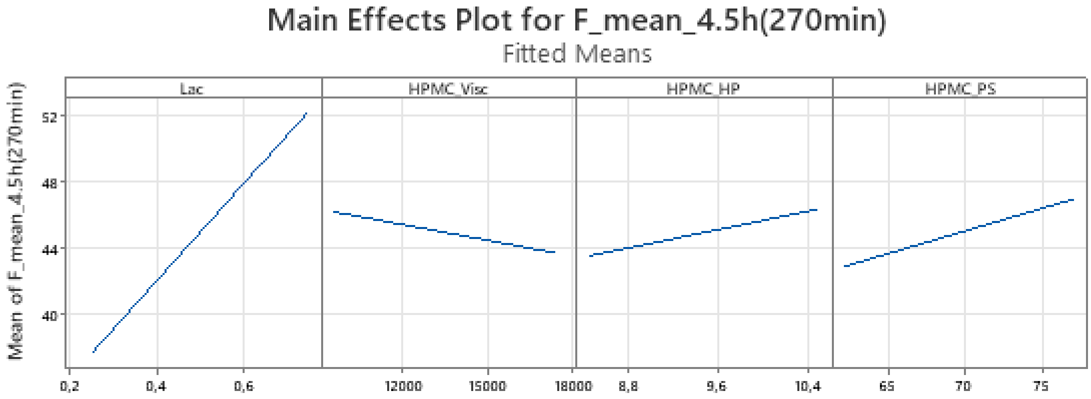

Figure S12. Main effects plot for mean % of carvedilol release using a Basic MLR model at t = 4.5 h (270 min).

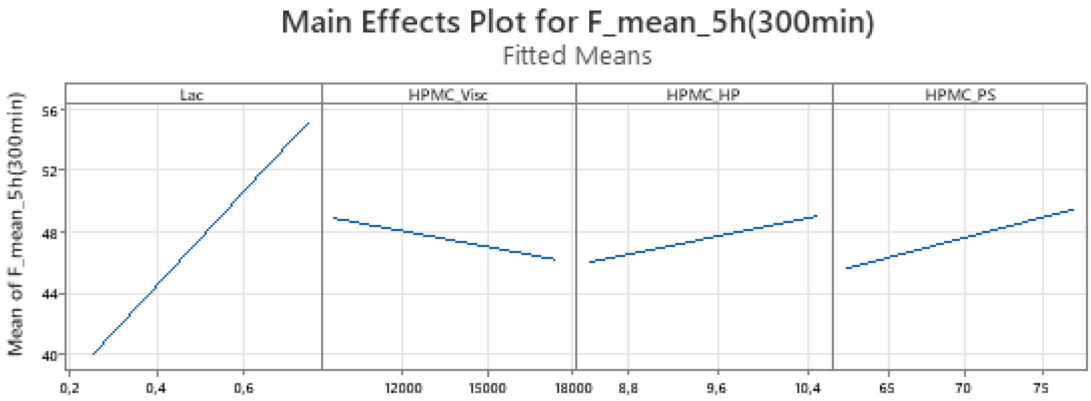

Figure S13. Main effects plot for mean % of carvedilol release using a Basic MLR model at t = 5 h (300 min).

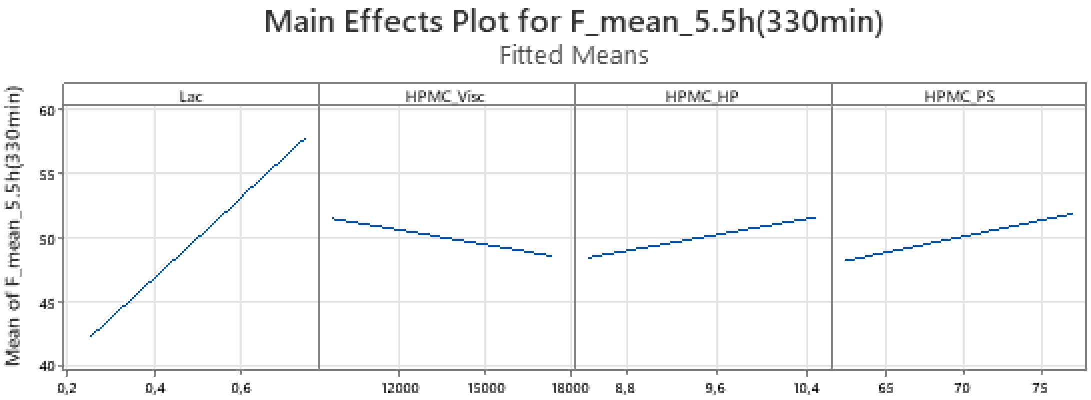

Figure S14. Main effects plot for mean % of carvedilol release using a Basic MLR model at t = 5.5 h (330 min).

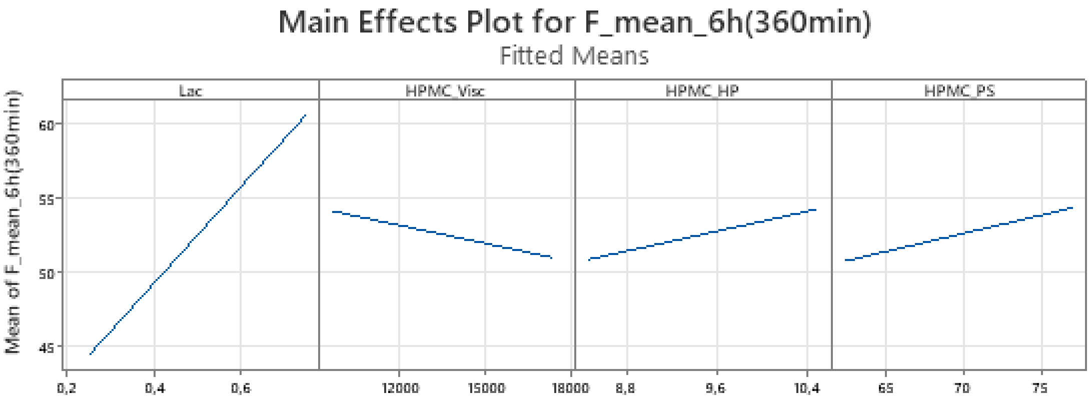

Figure S15. Main effects plot for mean % of carvedilol release using a Basic MLR model at t = 6 h (360 min).

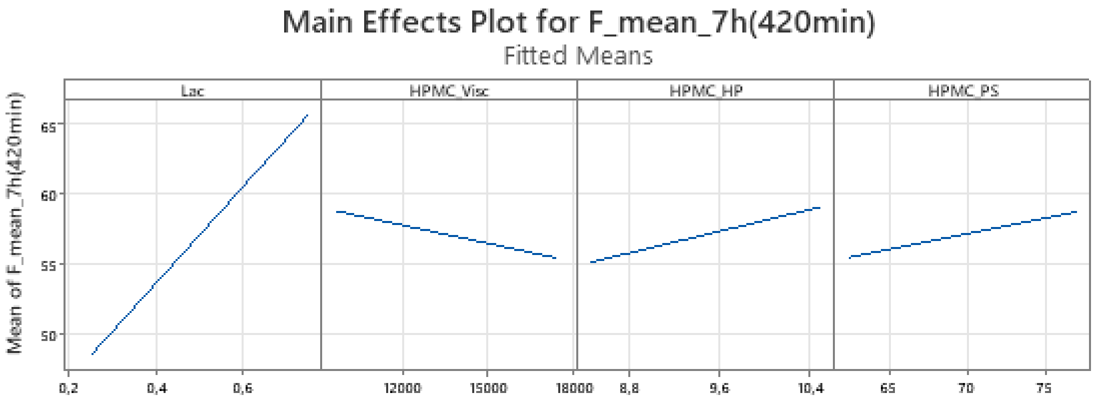

Figure S16. Main effects plot for mean % of carvedilol release using a Basic MLR model at t = 7 h (420 min).

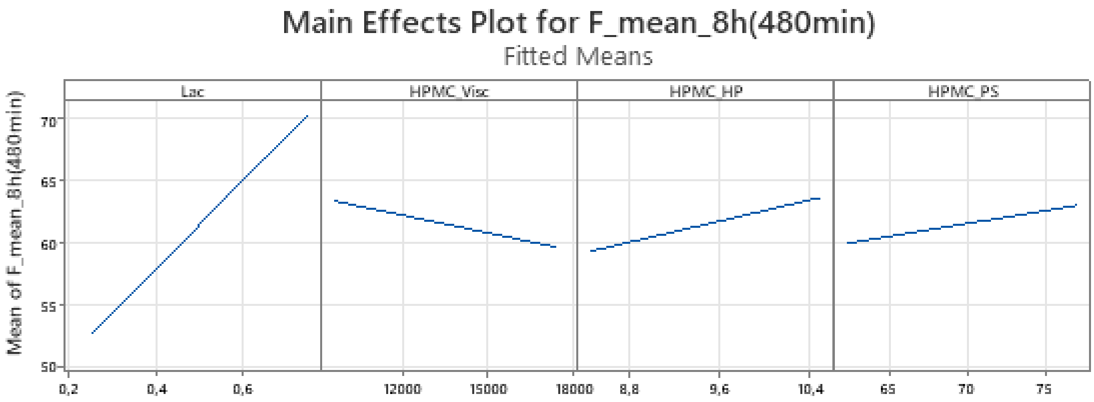

Figure S17. Main effects plot for mean % of carvedilol release using a Basic MLR model at t = 8 h (480 min).

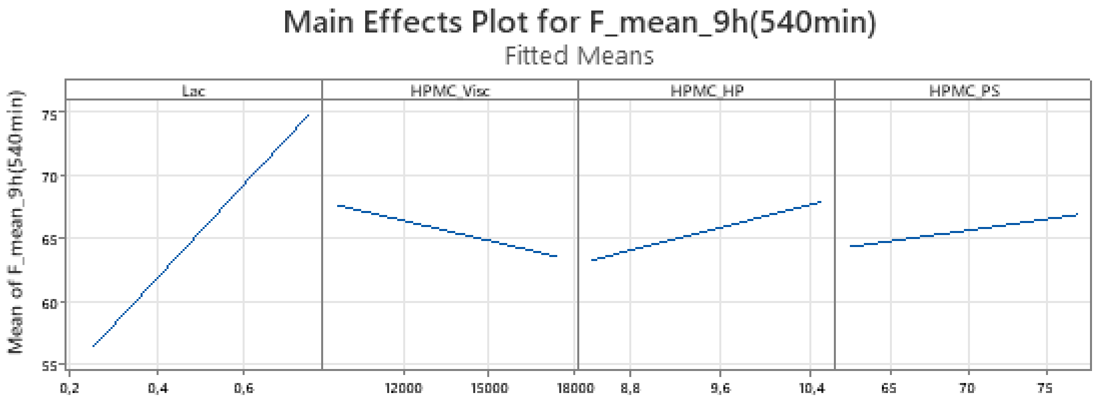

Figure S18. Main effects plot for mean % of carvedilol release using a Basic MLR model at t = 9 h (540 min).

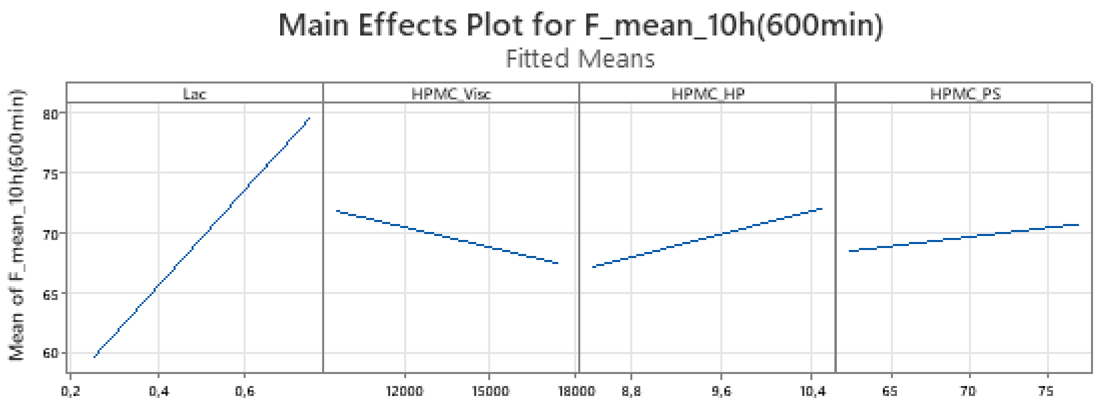

Figure S19. Main effects plot for mean % of carvedilol release using a Basic MLR model at t = 10 h (600 min).

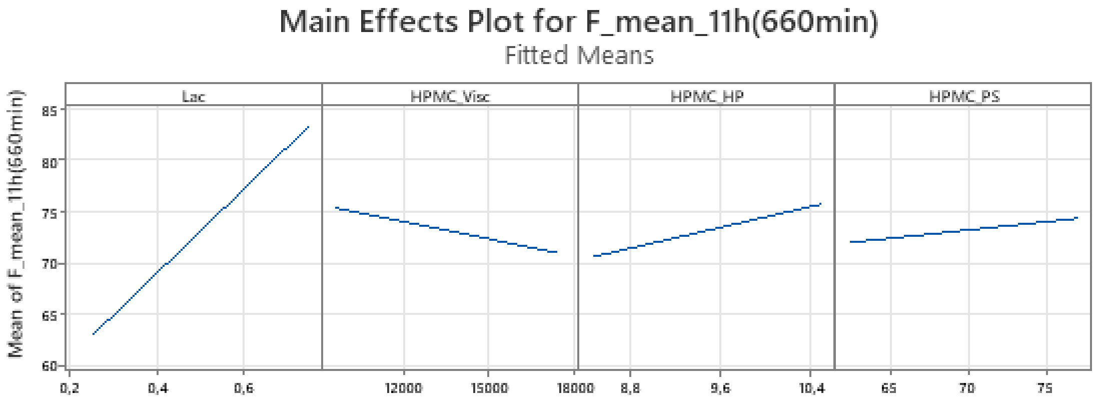

Figure S20. Main effects plot for mean % of carvedilol release using a Basic MLR model at t = 11 h (660 min).

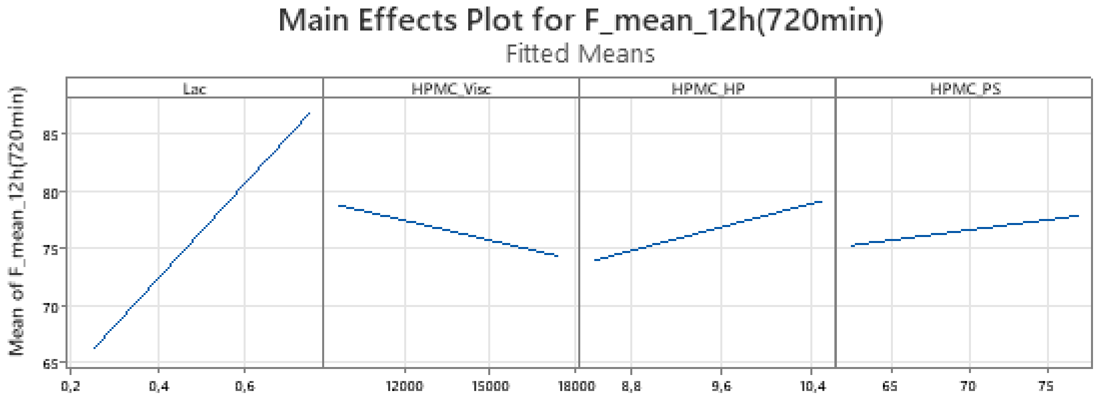

Figure S21. Main effects plot for mean % of carvedilol release using a Basic MLR model at t = 12 h (720 min).

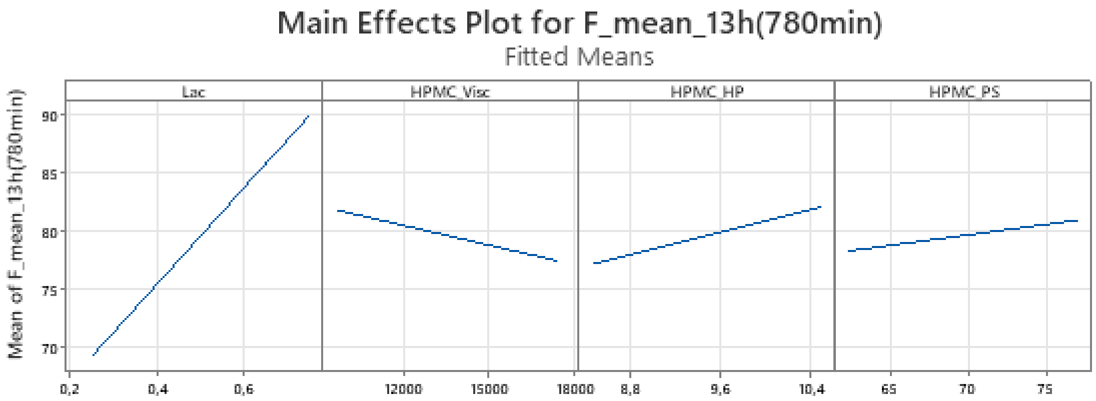

Figure S22. Main effects plot for mean % of carvedilol release using a Basic MLR model at t = 13 h (780 min).

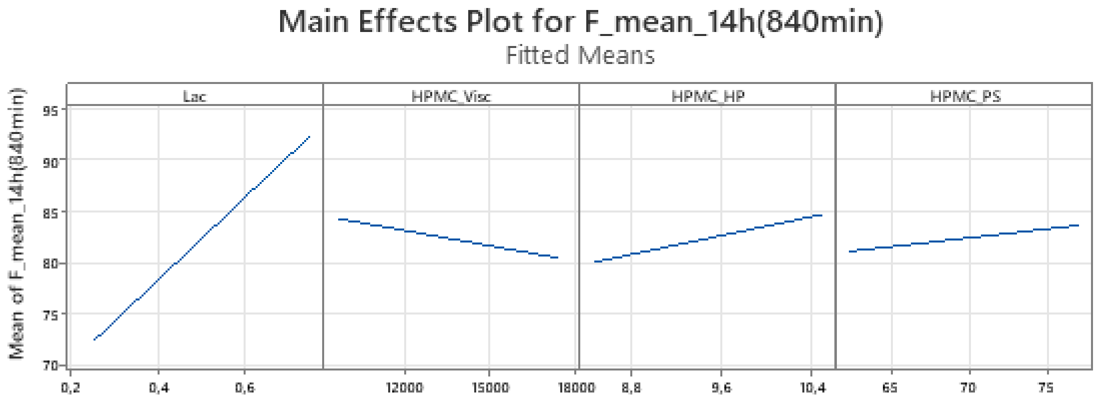

Figure S23. Main effects plot for mean % of carvedilol release using a Basic MLR model at t = 14 h (840 min).

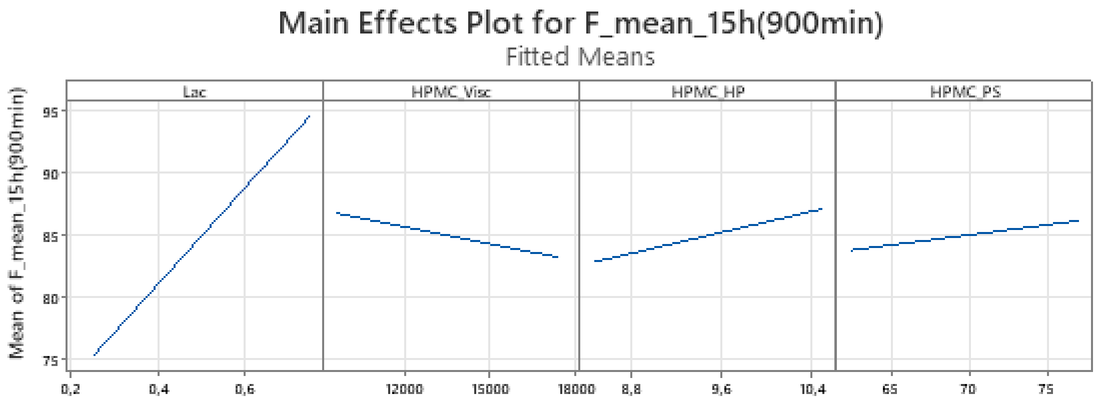

Figure S24. Main effects plot for mean % of carvedilol release using a Basic MLR model at t = 15 h (900 min).

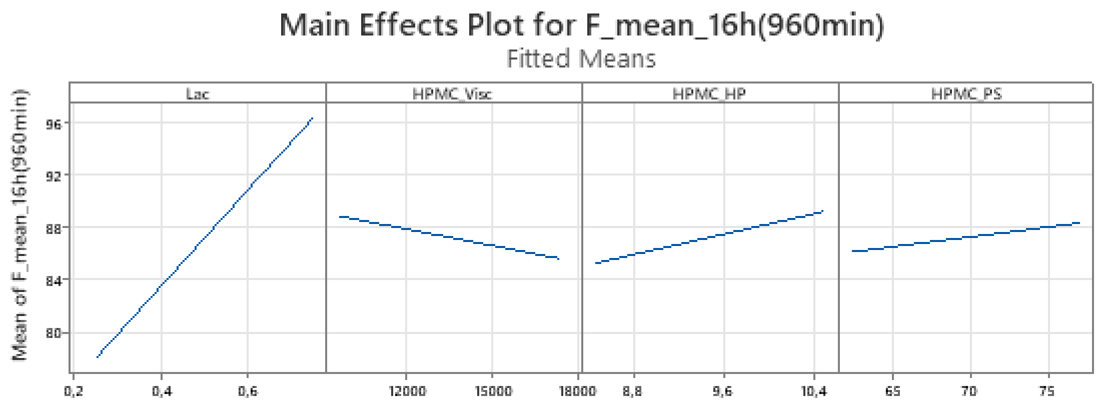

Figure S25. Main effects plot for mean % of carvedilol release using a Basic MLR model at t = 16 h (960 min).

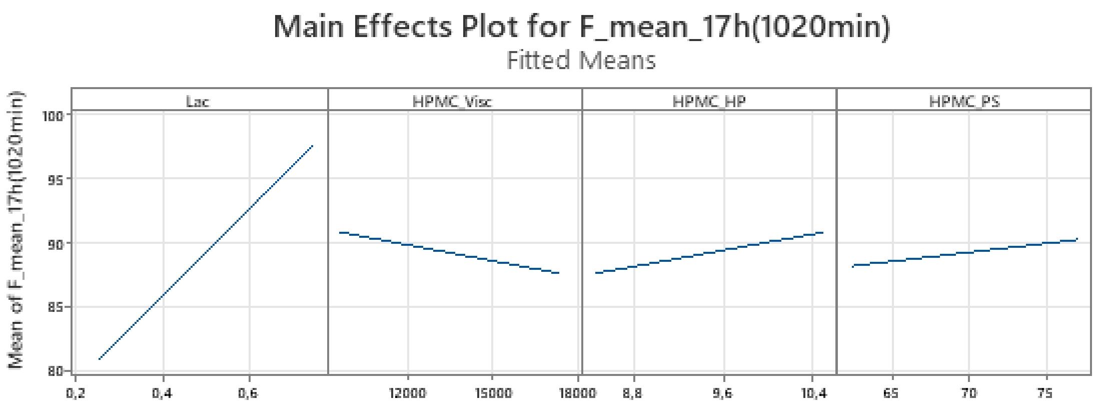

Figure S26. Main effects plot for mean % of carvedilol release using a Basic MLR model at t = 17 h (1020 min).

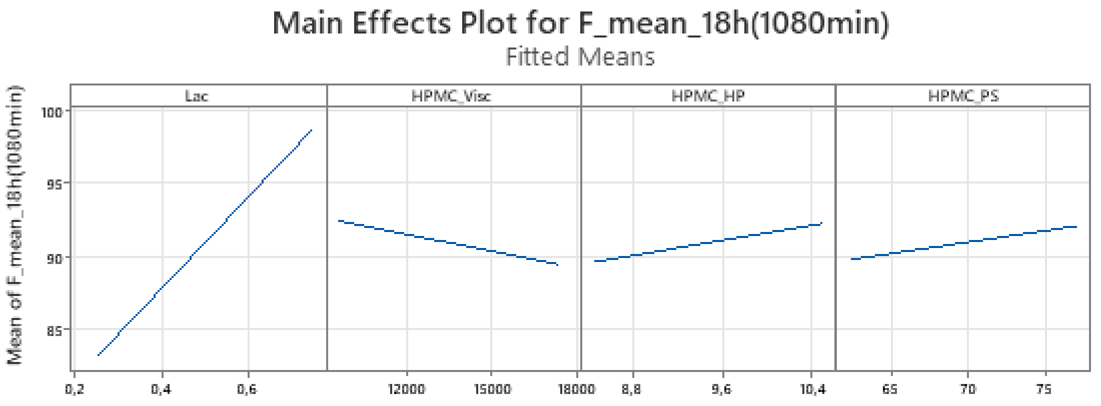

Figure S27. Main effects plot for mean % of carvedilol release using a Basic MLR model at t = 18 h (1080 min).

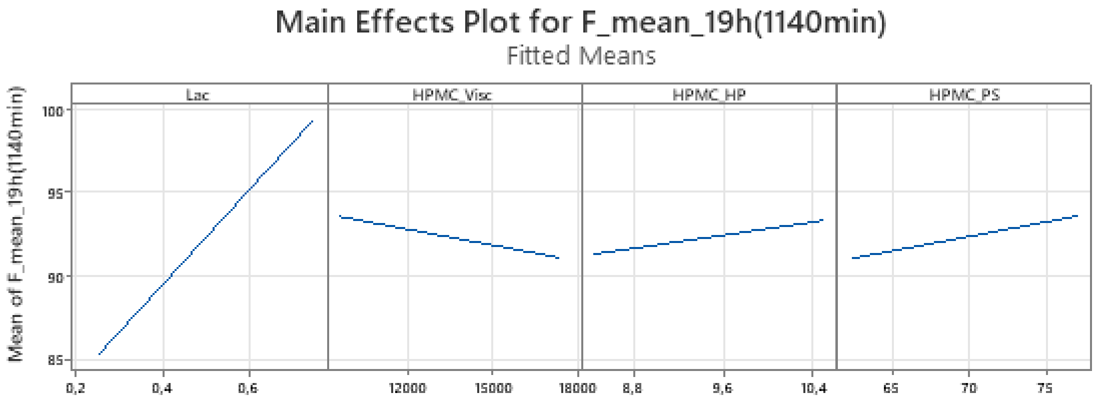

Figure S28. Main effects plot for mean % of carvedilol release using a Basic MLR model at t = 19 h (1140 min).

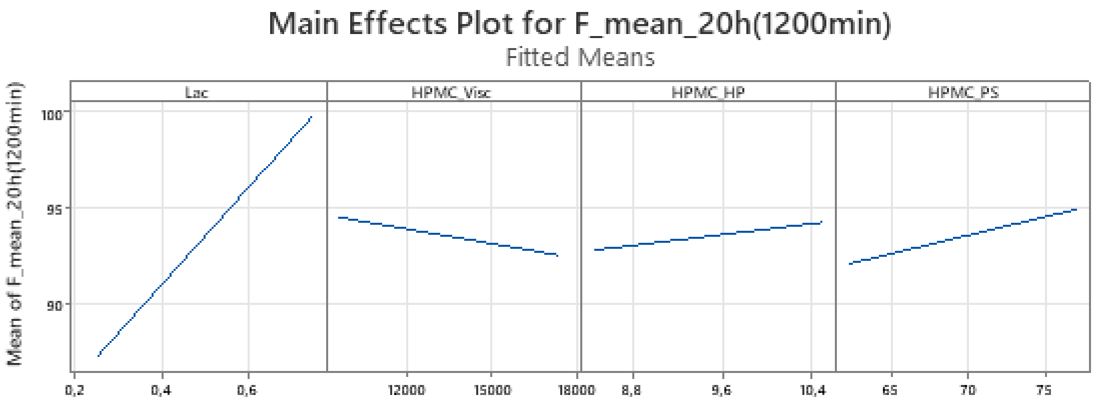

Figure S29. Main effects plot for mean % of carvedilol release using a Basic MLR model at t = 20 h (1200 min).

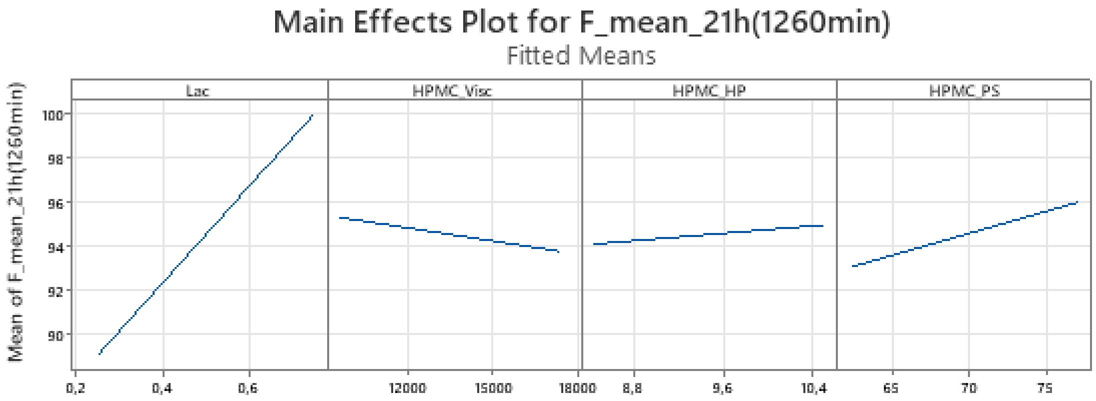

Figure S30. Main effects plot for mean % of carvedilol release using a Basic MLR model at t = 21 h (1260 min).

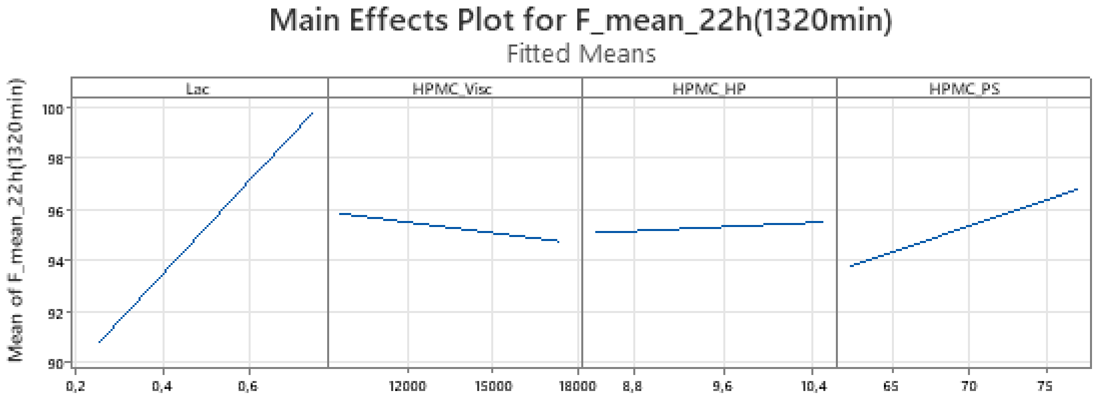

Figure S31. Main effects plot for mean % of carvedilol release using a Basic MLR model at t = 22 h (1320 min).

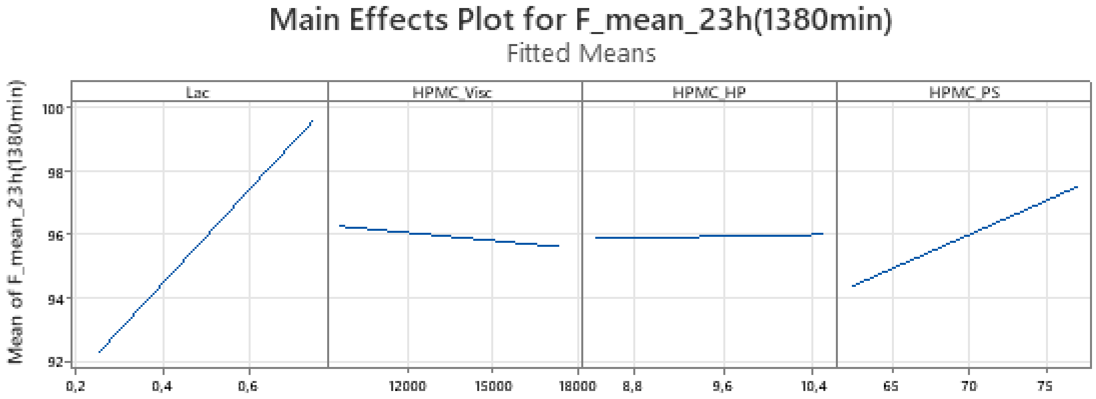

Figure S32. Main effects plot for mean % of carvedilol release using a Basic MLR model at t = 23 h (1380 min).

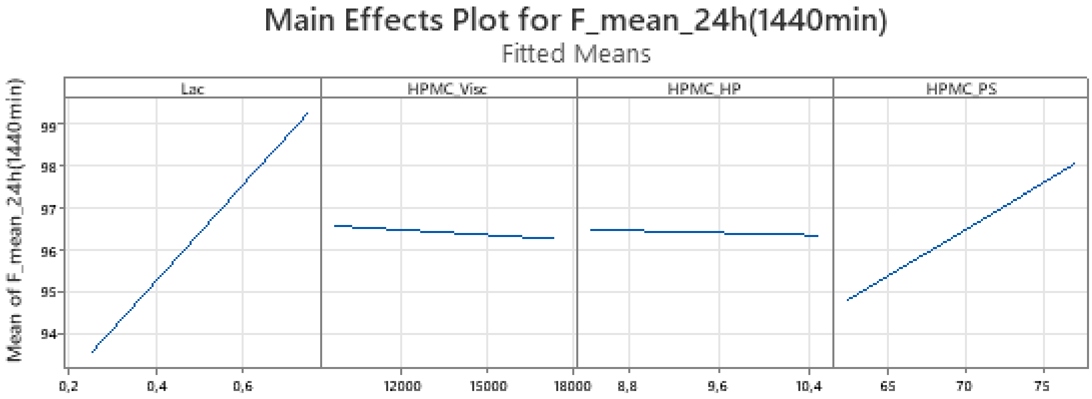

Figure S33. Main effects plot for mean % of carvedilol release using a Basic MLR model at t = 24 h (1440 min).

a)

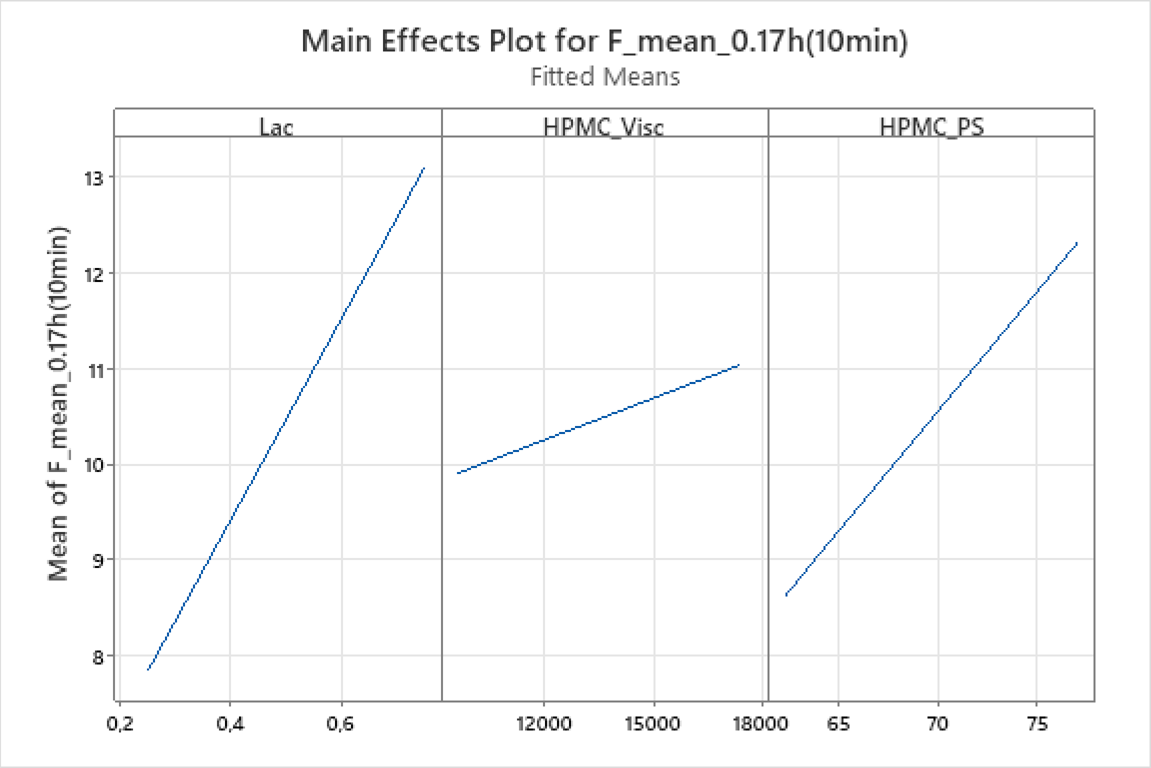

b)

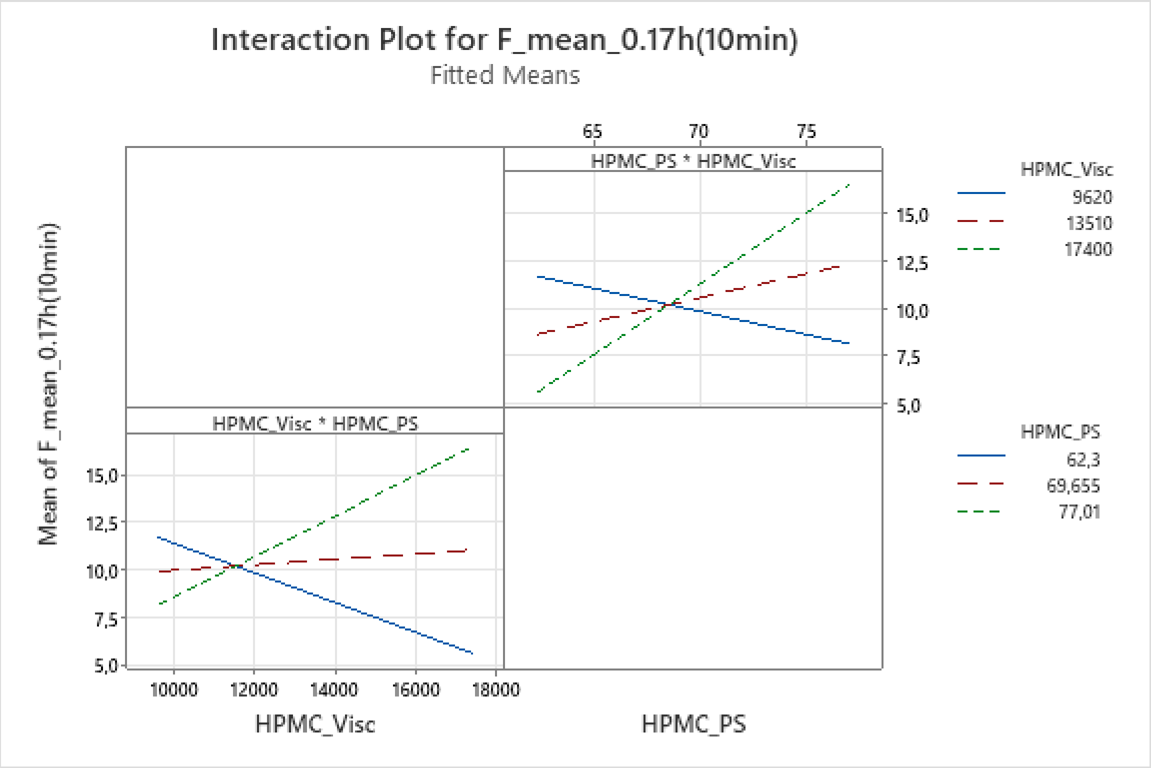

Figure S34. Main effects plot (a) and Interaction plot for mean % of carvedilol release using an Optimized MLR model at t = 0.17 h (10 min).

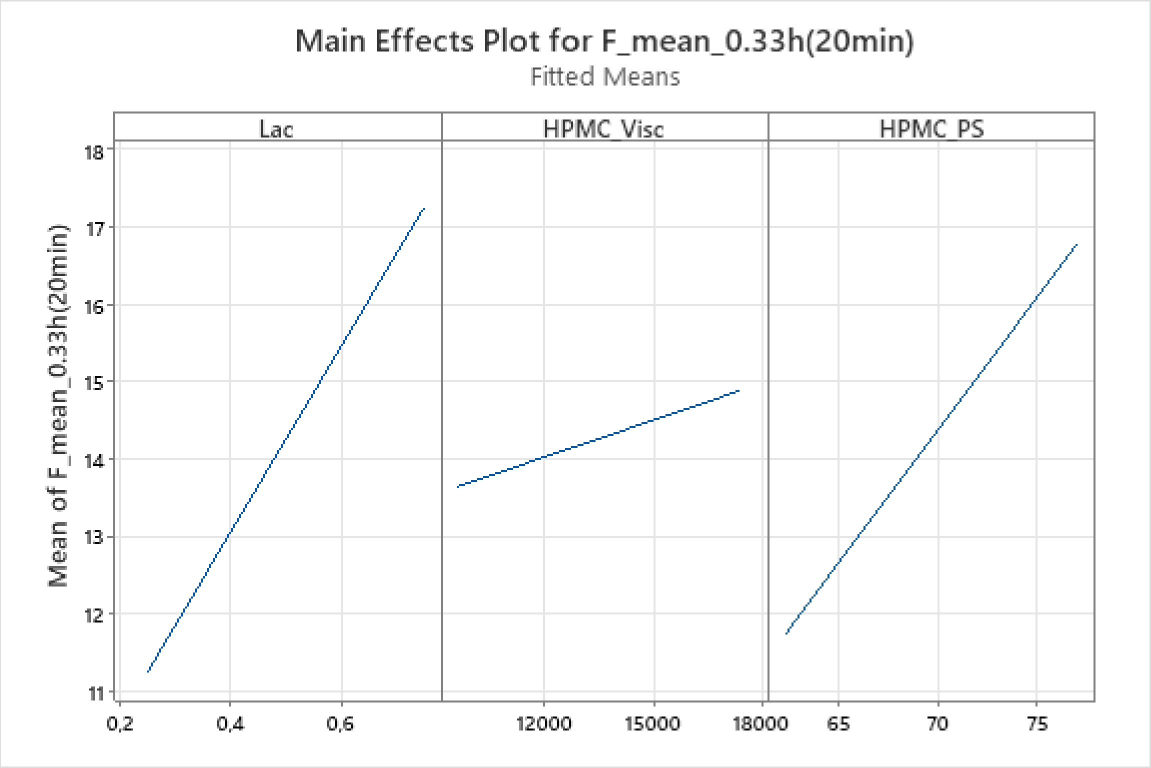

a)

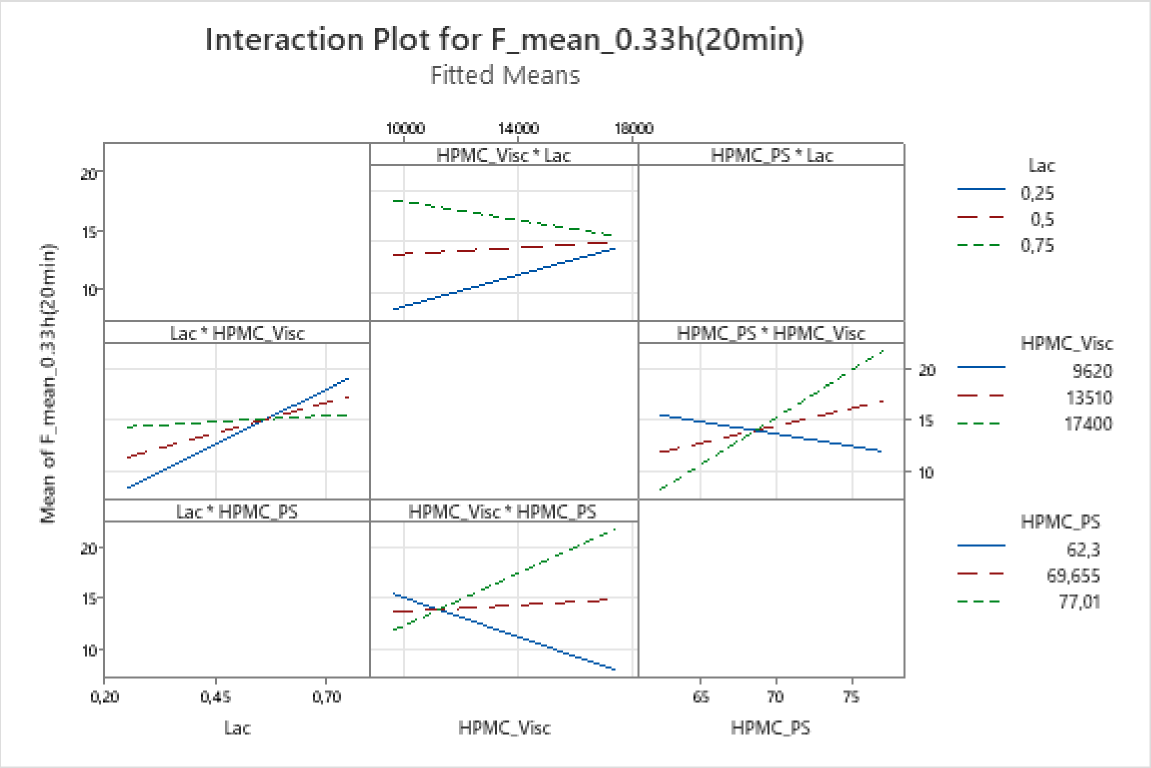

b)

Figure S35. Main effects plot (a) and Interaction plot for mean % of carvedilol release using an Optimized MLR model at t = 0.33 h (20 min).

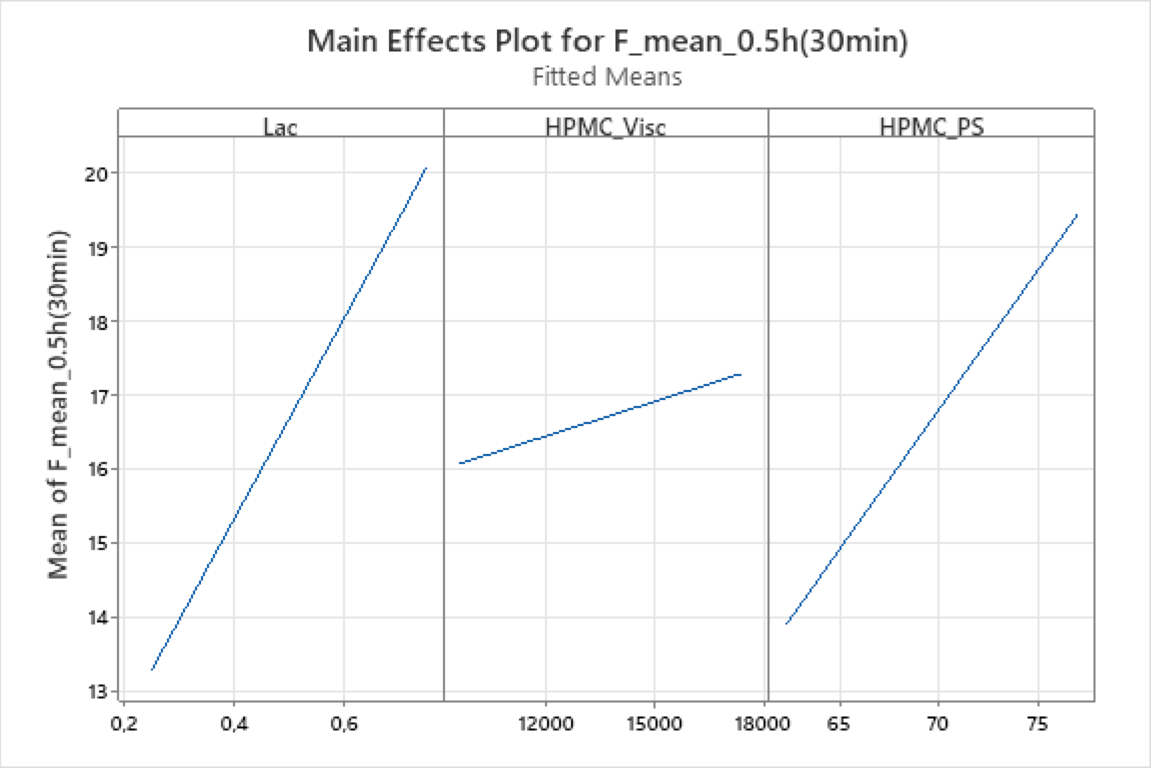

a)

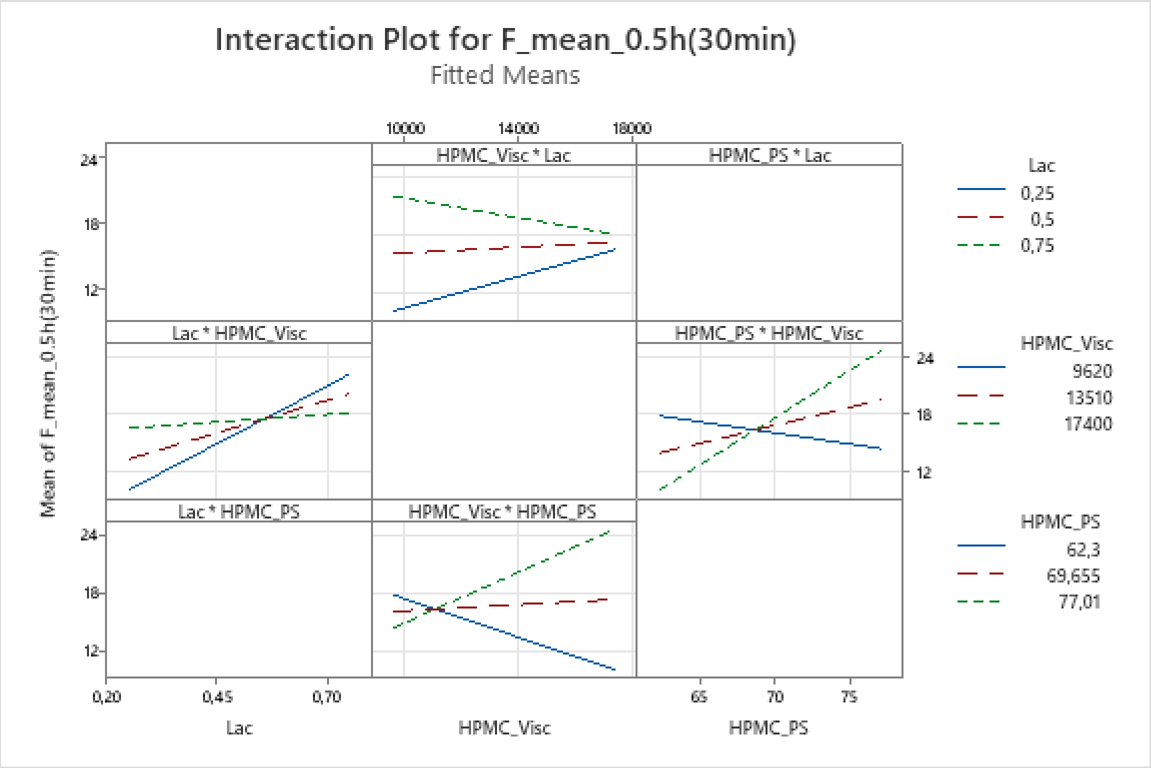

b)

Figure S36. Main effects plot (a) and Interaction plot for mean % of carvedilol release using an Optimized MLR model at t = 0.5 h (30 min).

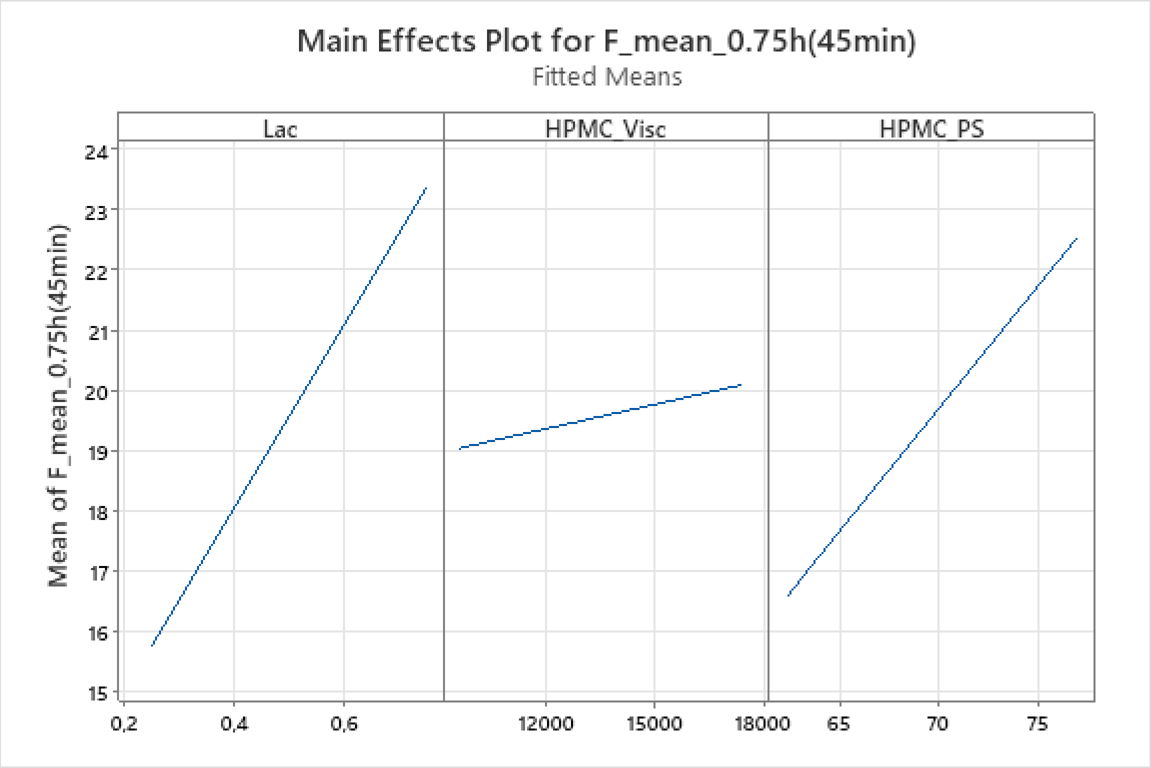

a)

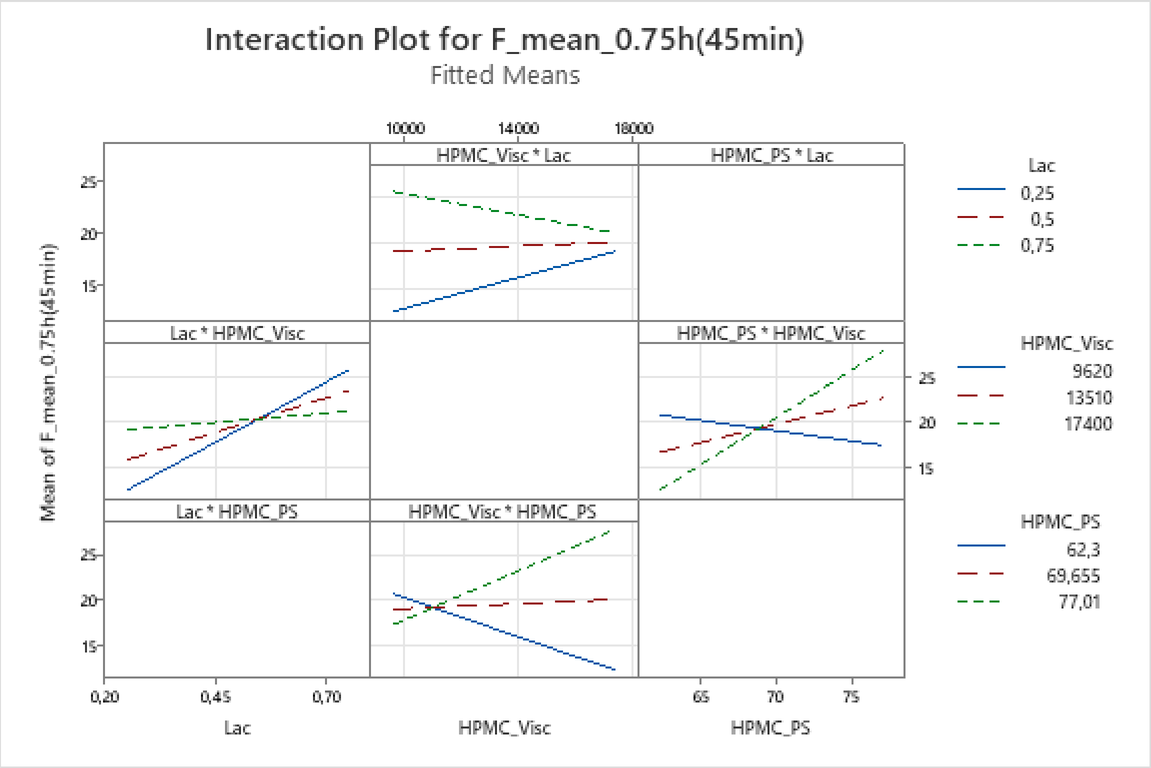

b)

Figure S37. Main effects plot (a) and Interaction plot for mean % of carvedilol release using an Optimized MLR model at t = 0.75 h (45 min).

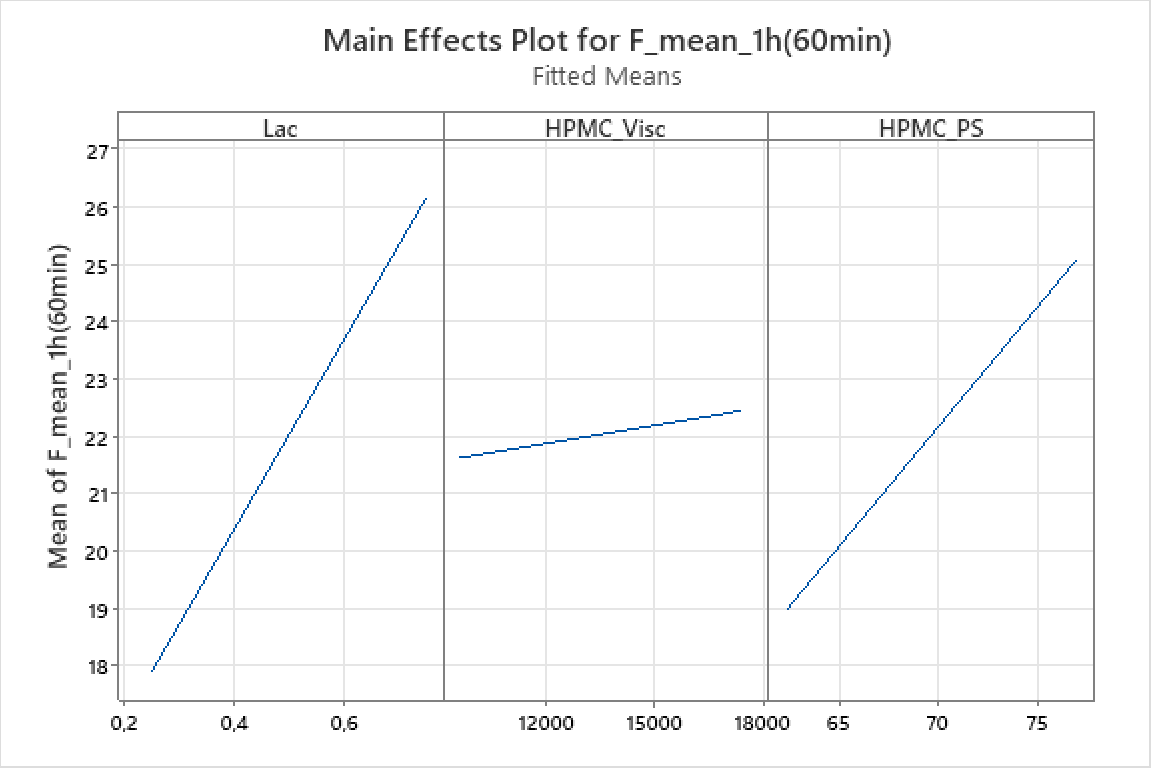

a)

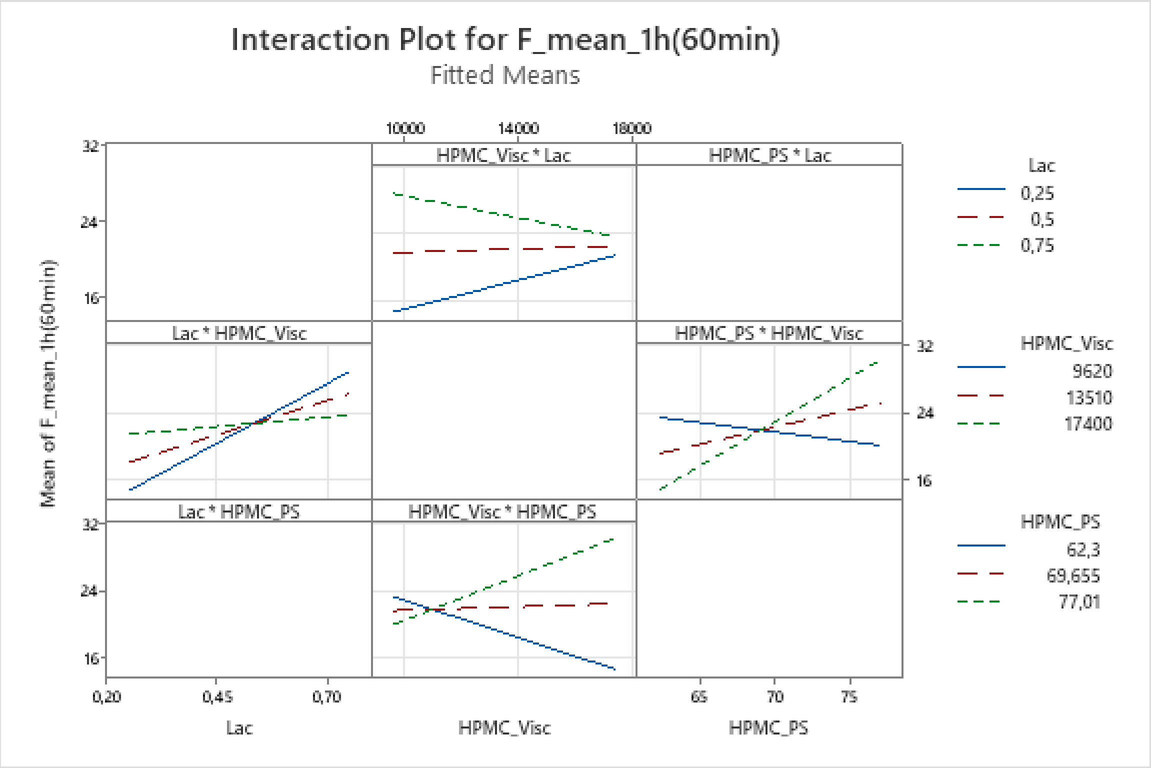

b)

Figure S38. Main effects plot (a) and Interaction plot for mean % of carvedilol release using an Optimized MLR model at t = 1 h (60 min).

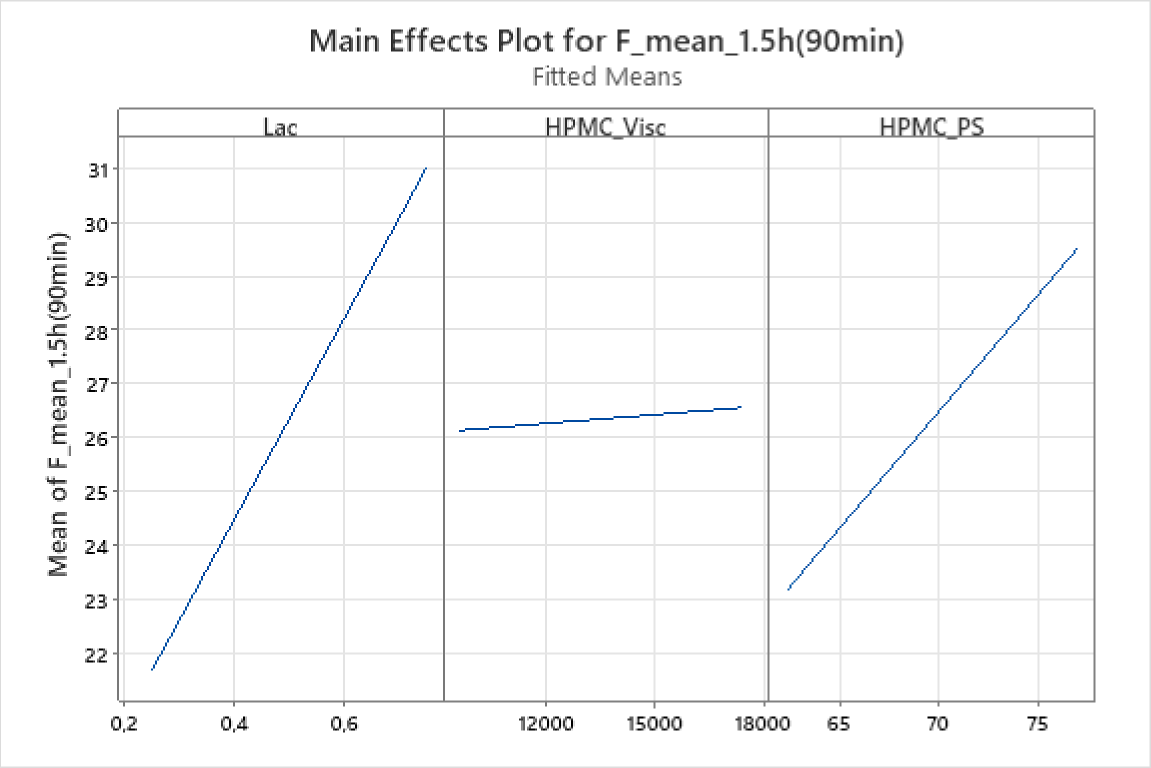

a)

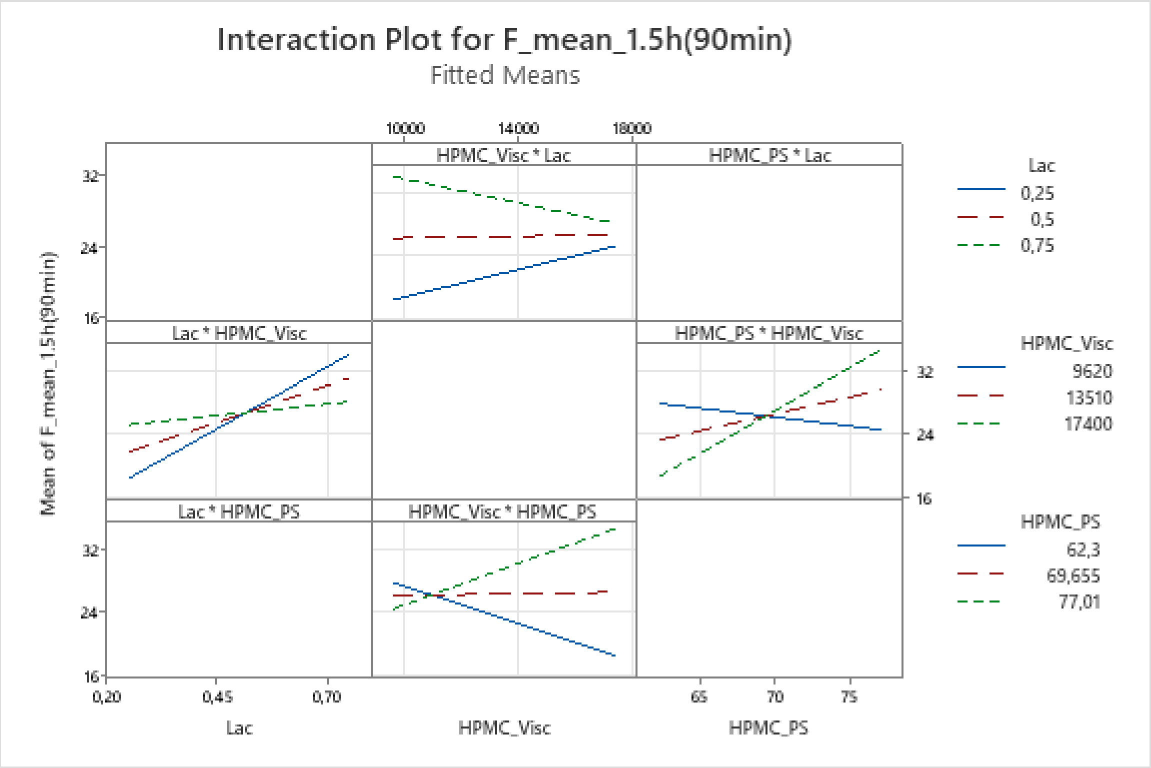

b)

Figure S39. Main effects plot (a) and Interaction plot for mean % of carvedilol release using an Optimized MLR model at t = 1.5 h (90 min).

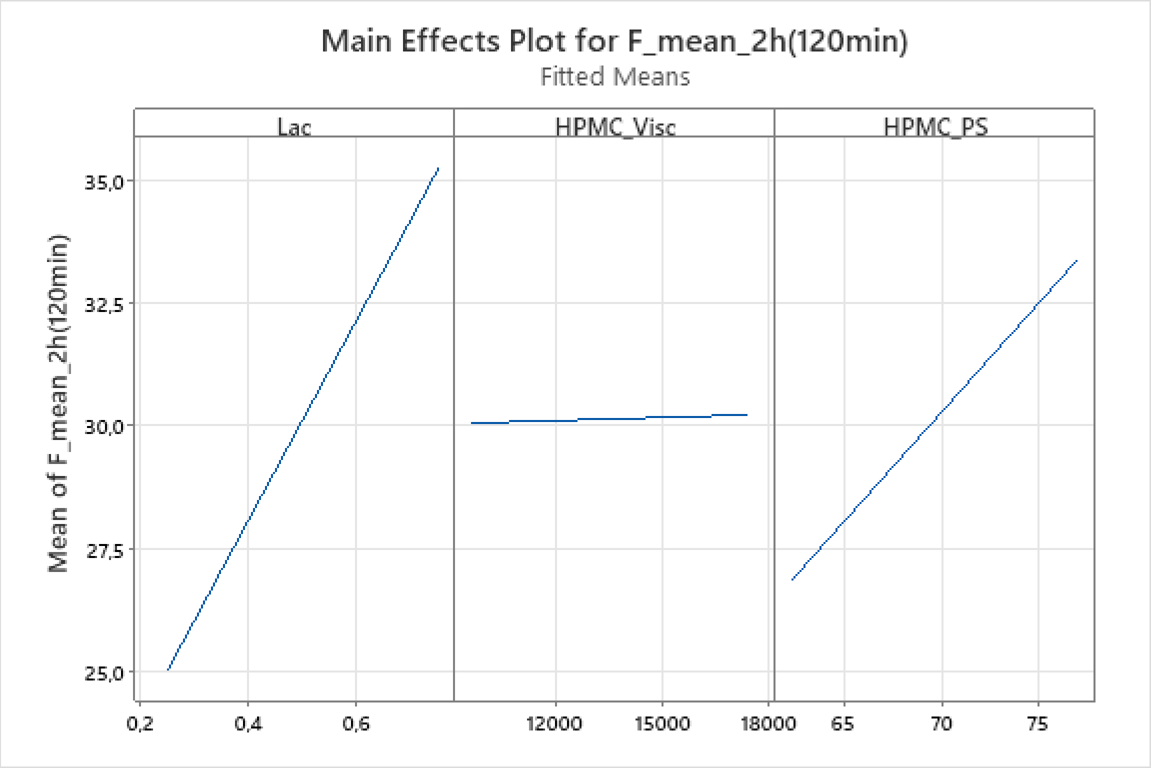

a)

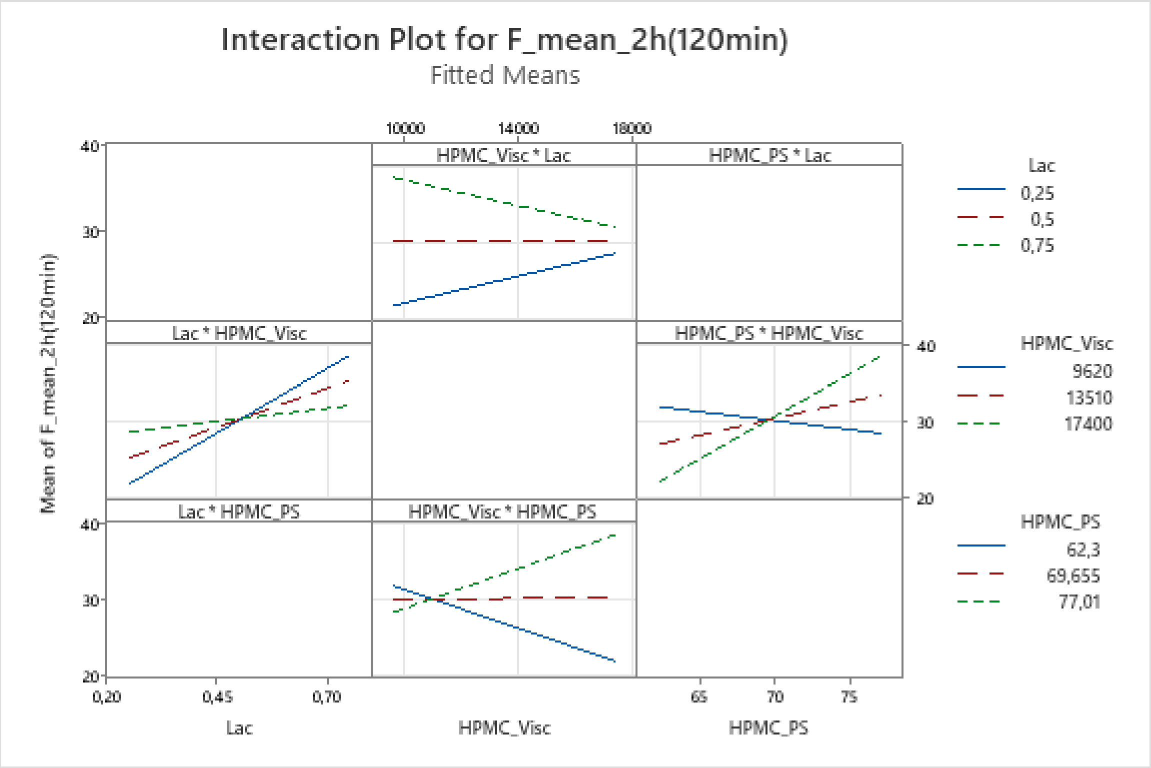

b)

Figure S40. Main effects plot (a) and Interaction plot for mean % of carvedilol release using an Optimized MLR model at t = 2 h (120 min).

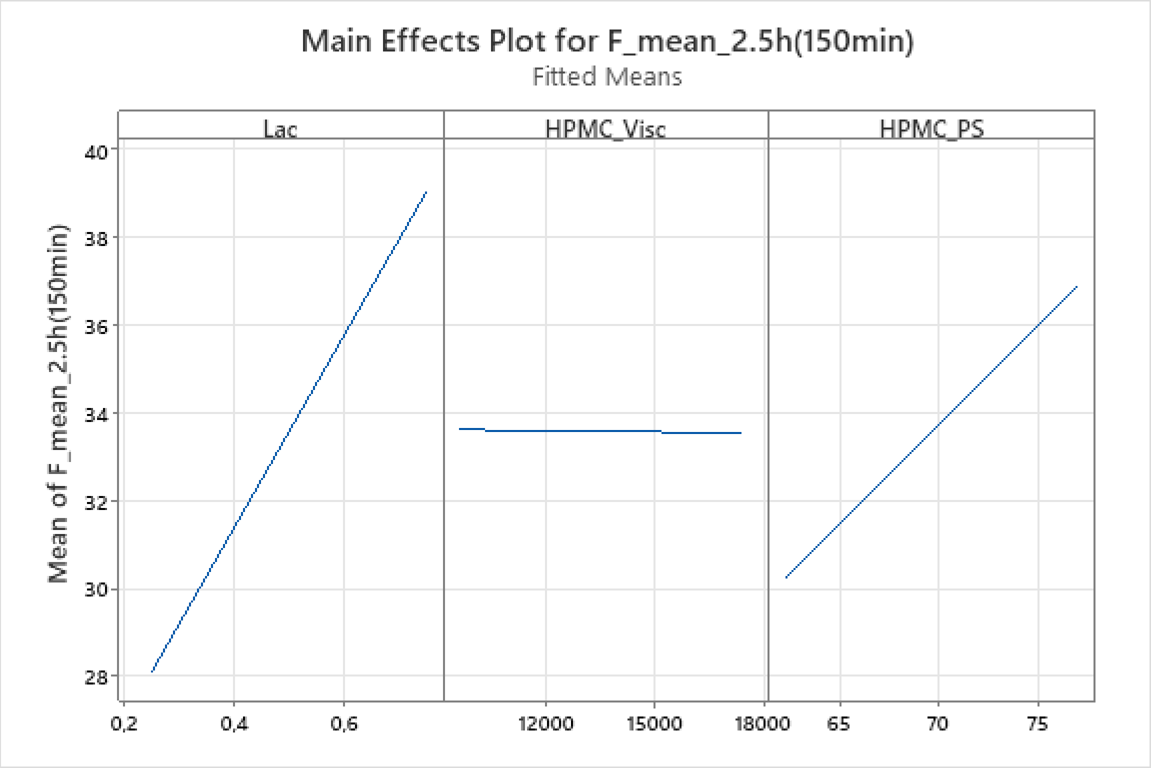

a)

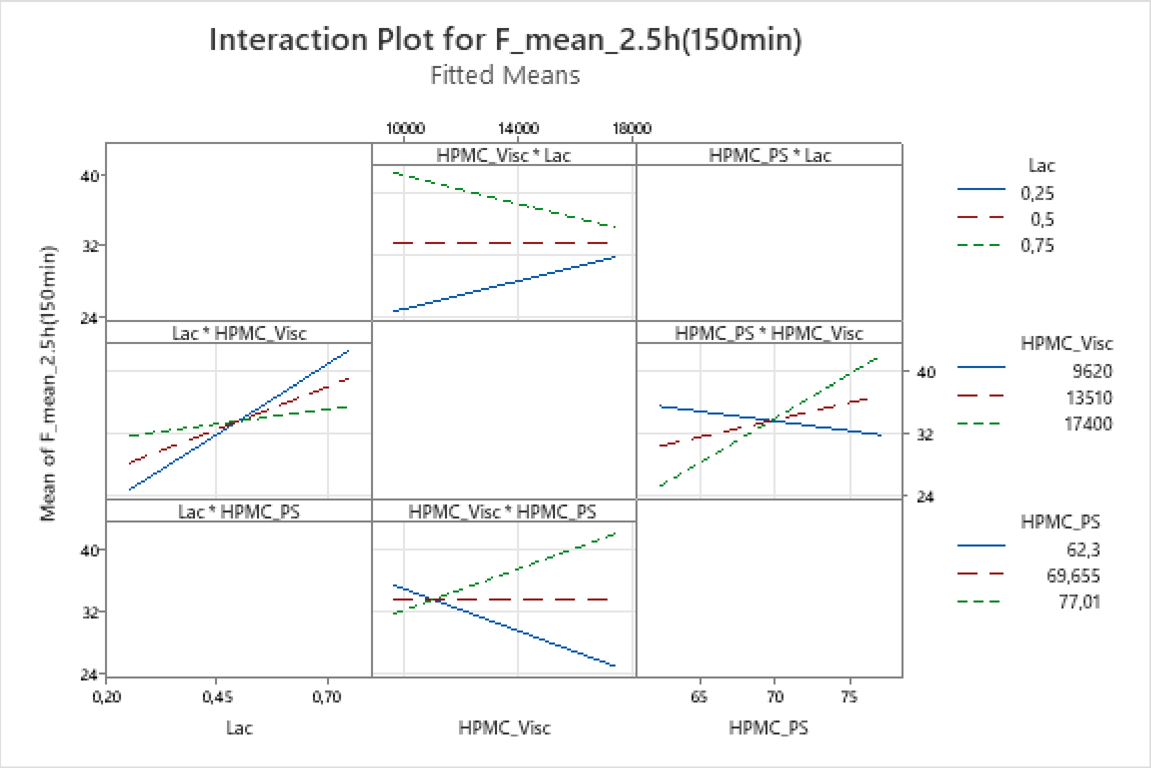

b)

Figure S41. Main effects plot (a) and Interaction plot for mean % of carvedilol release using an Optimized MLR model at t = 2.5 h (150 min).

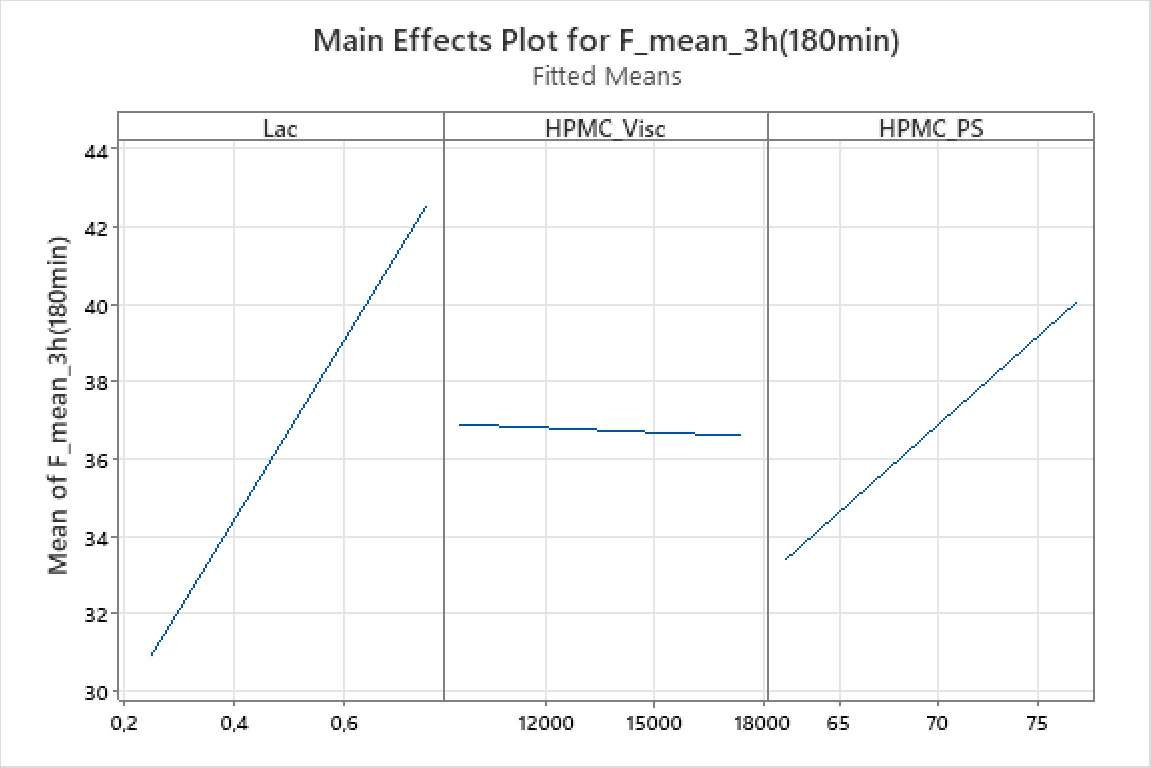

a)

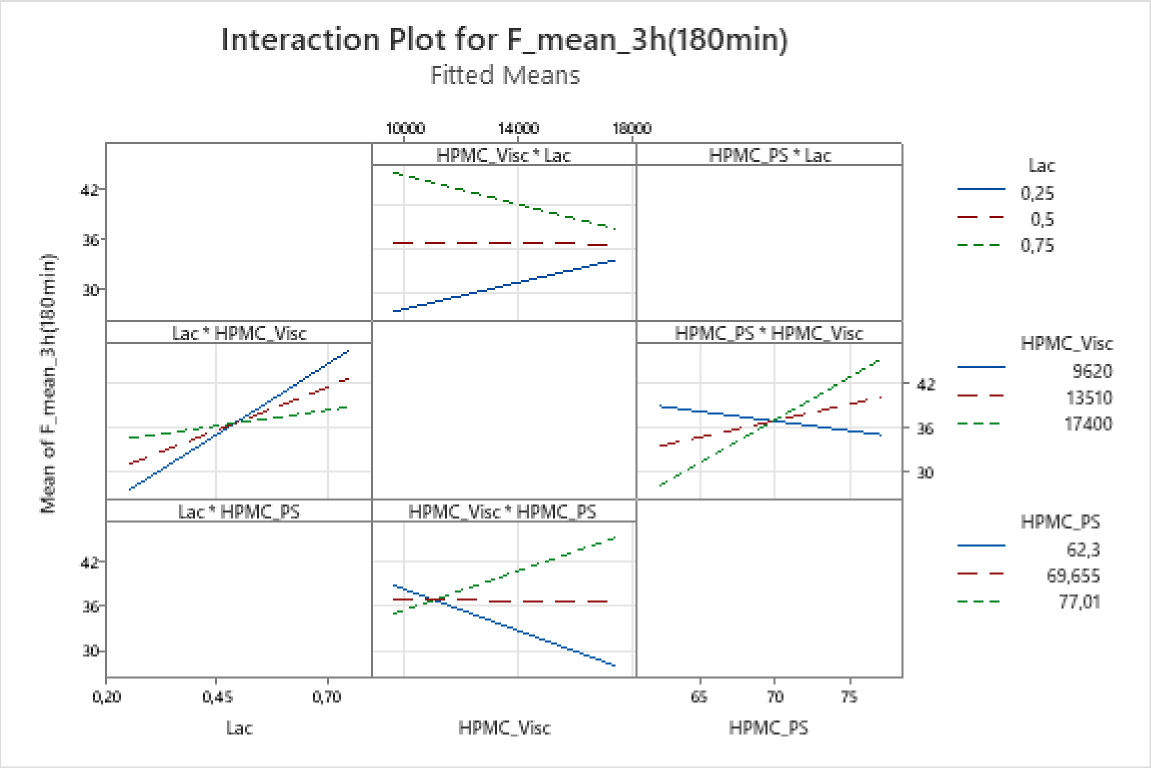

b)

Figure S42. Main effects plot (a) and Interaction plot for mean % of carvedilol release using an Optimized MLR model at t = 3 h (180 min).

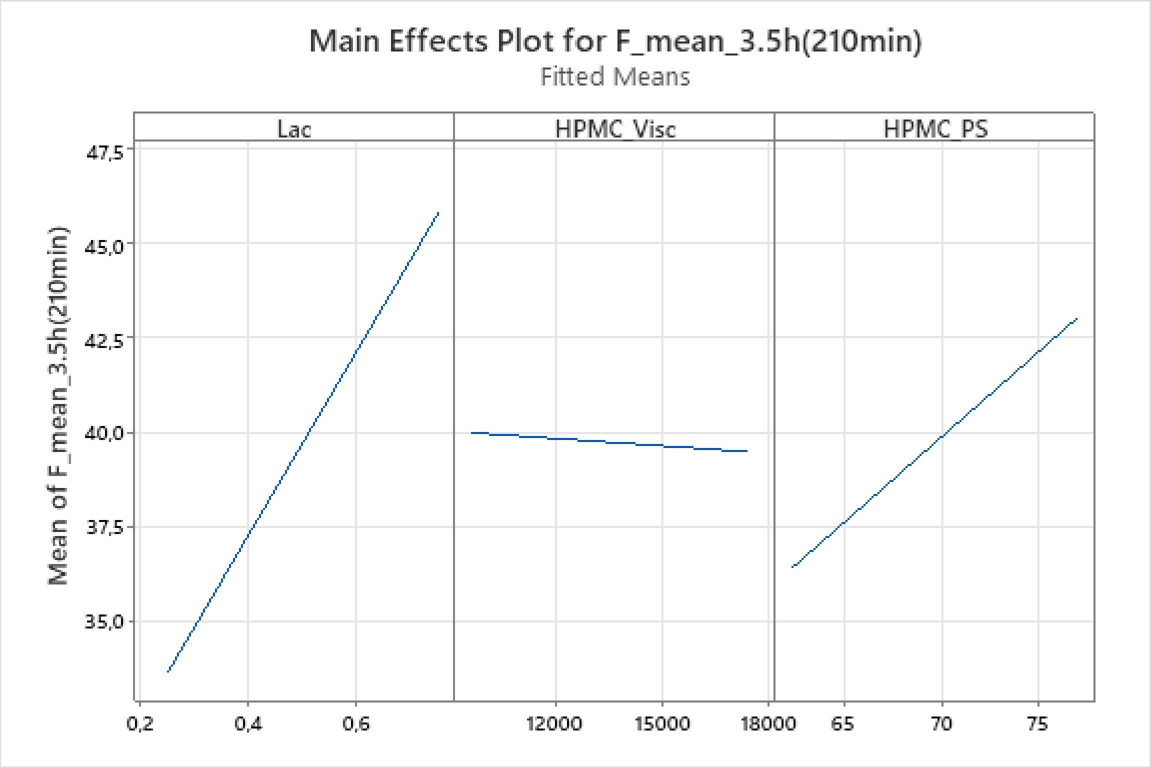

a)

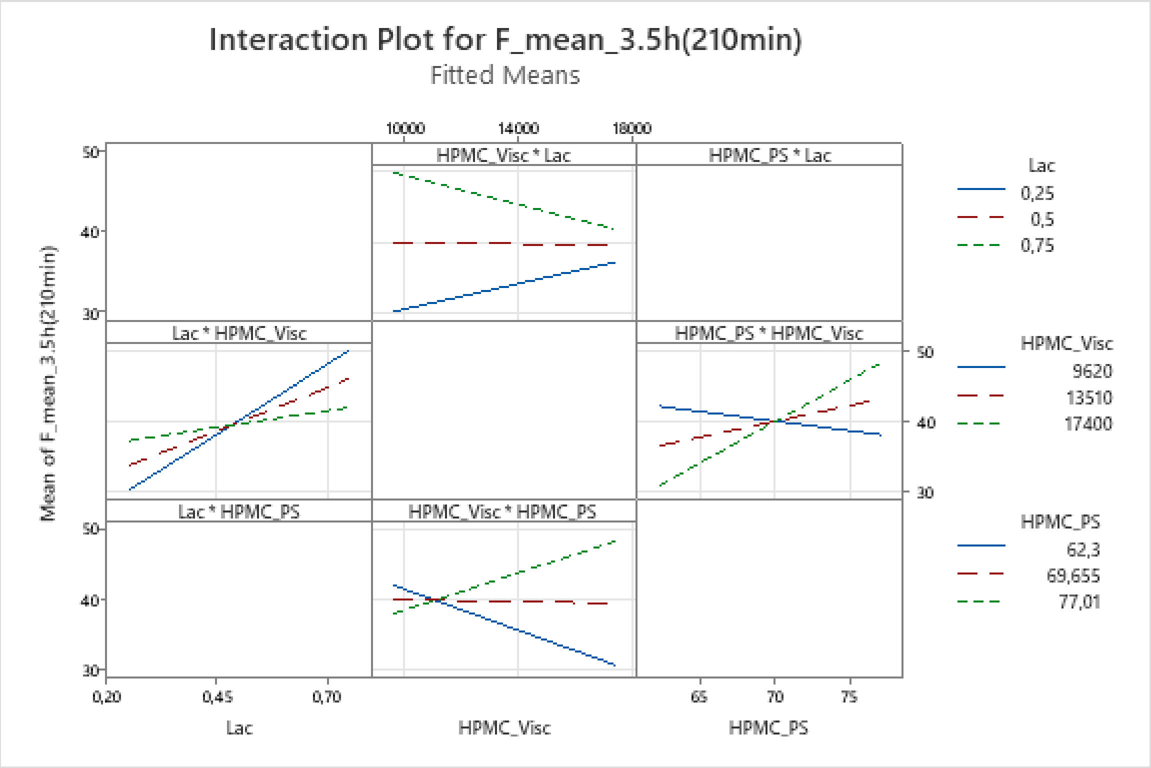

b)

Figure S43. Main effects plot (a) and Interaction plot for mean % of carvedilol release using an Optimized MLR model at t = 3.5 h (210 min).

a)
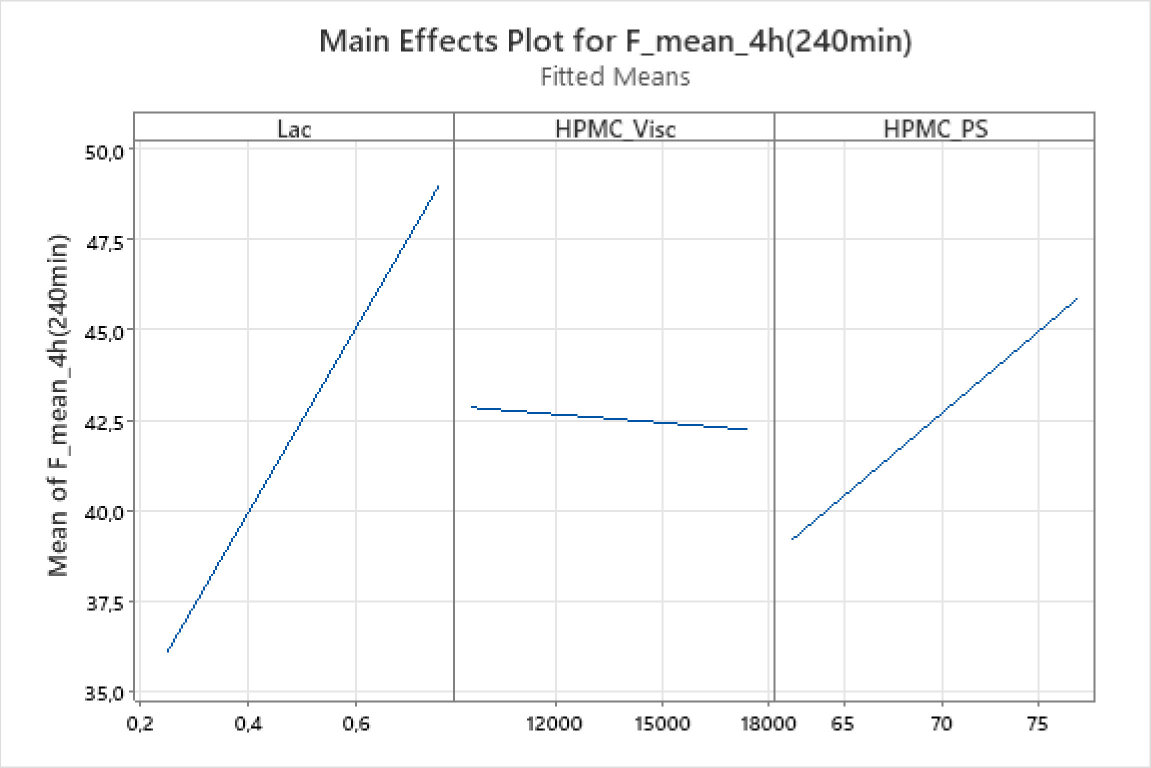

b)
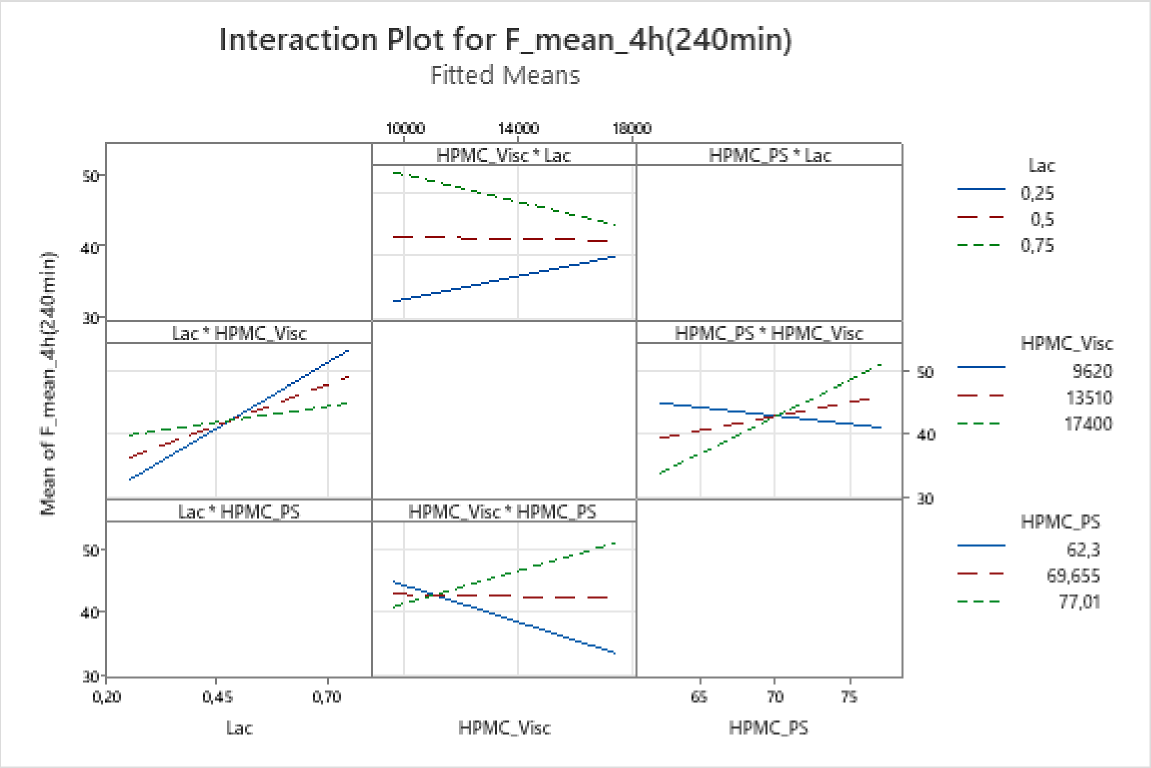

Figure S44. Main effects plot (a) and Interaction plot for mean % of carvedilol release using an Optimized MLR model at t = 4 h (240 min).

a)
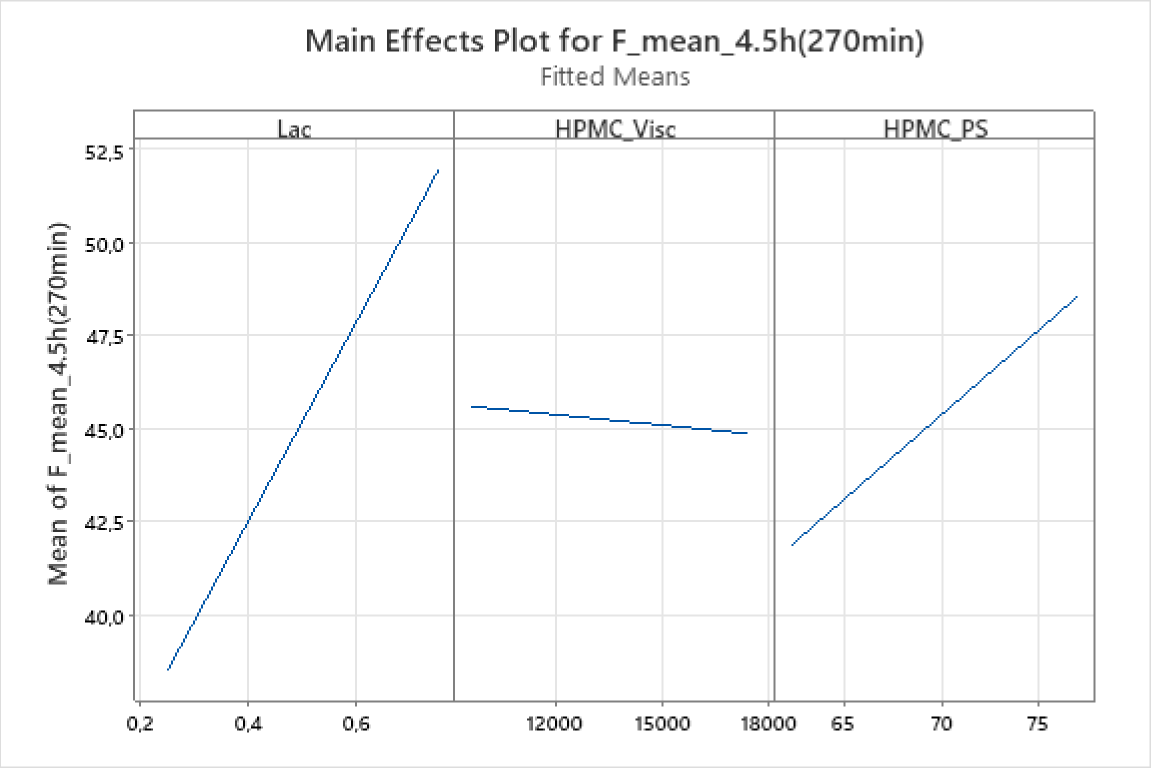

b)
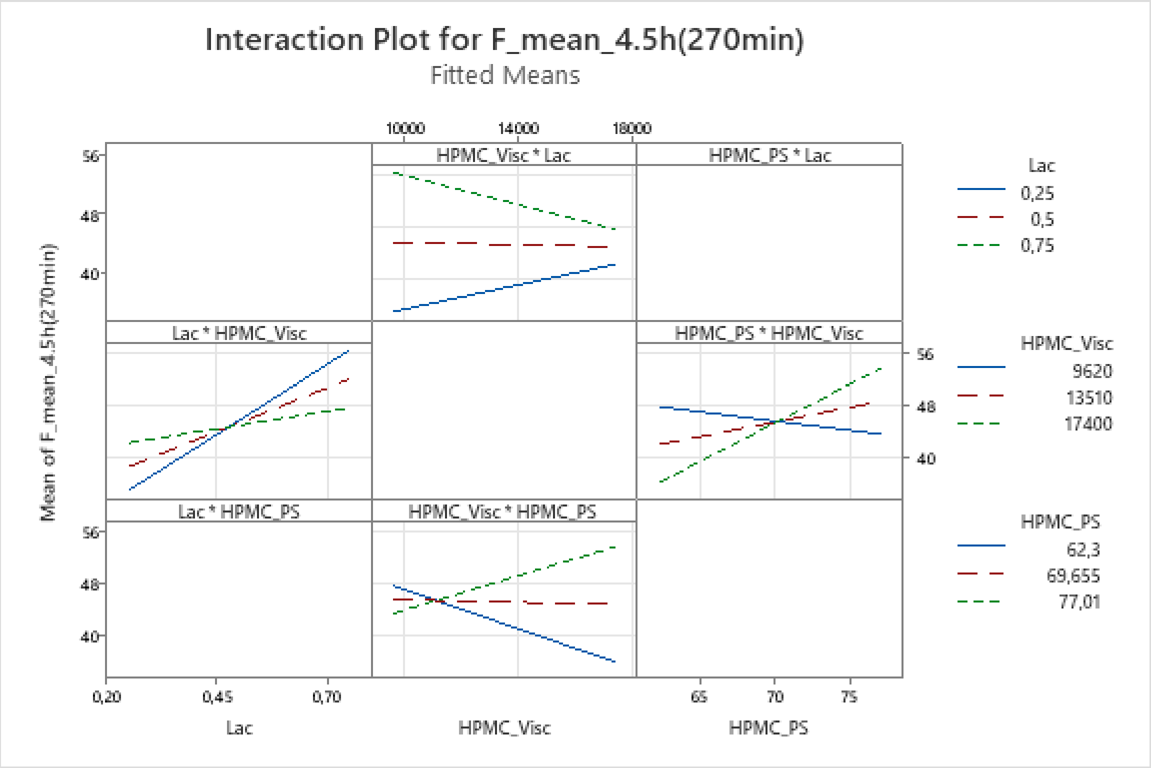

Figure S45. Main effects plot (a) and Interaction plot for mean % of carvedilol release using an Optimized MLR model at t = 4.5 h (270 min).

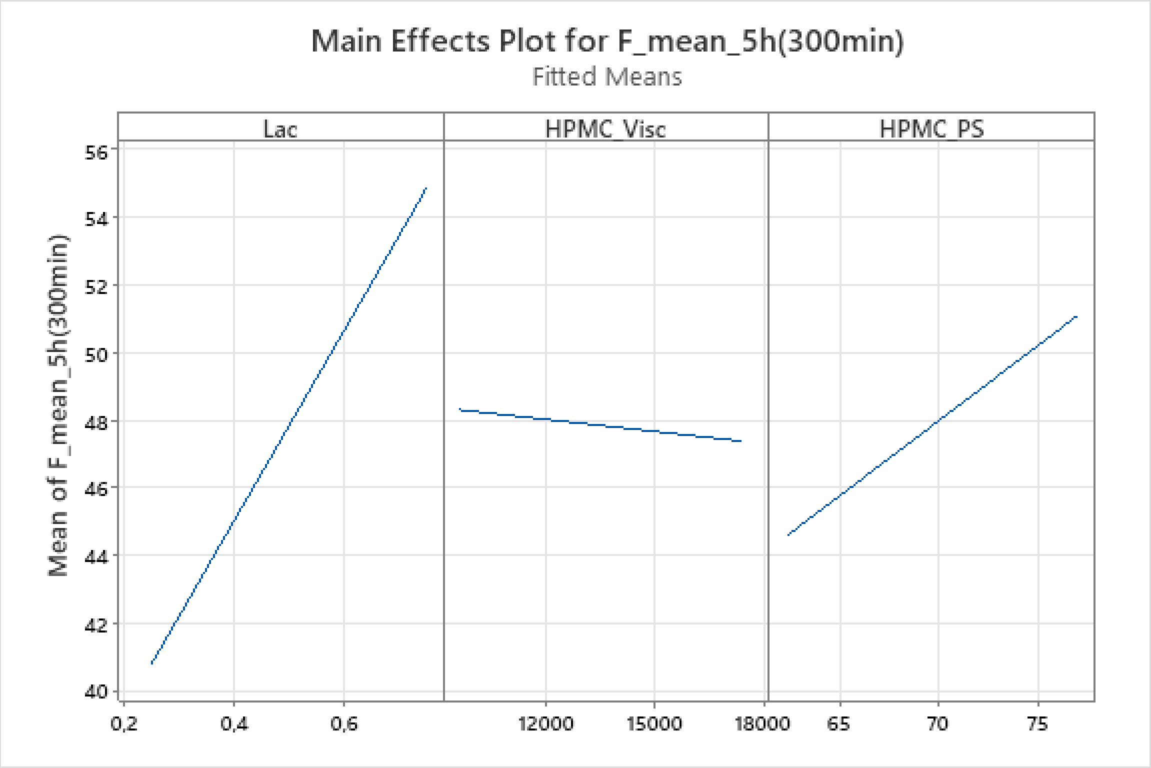

a)

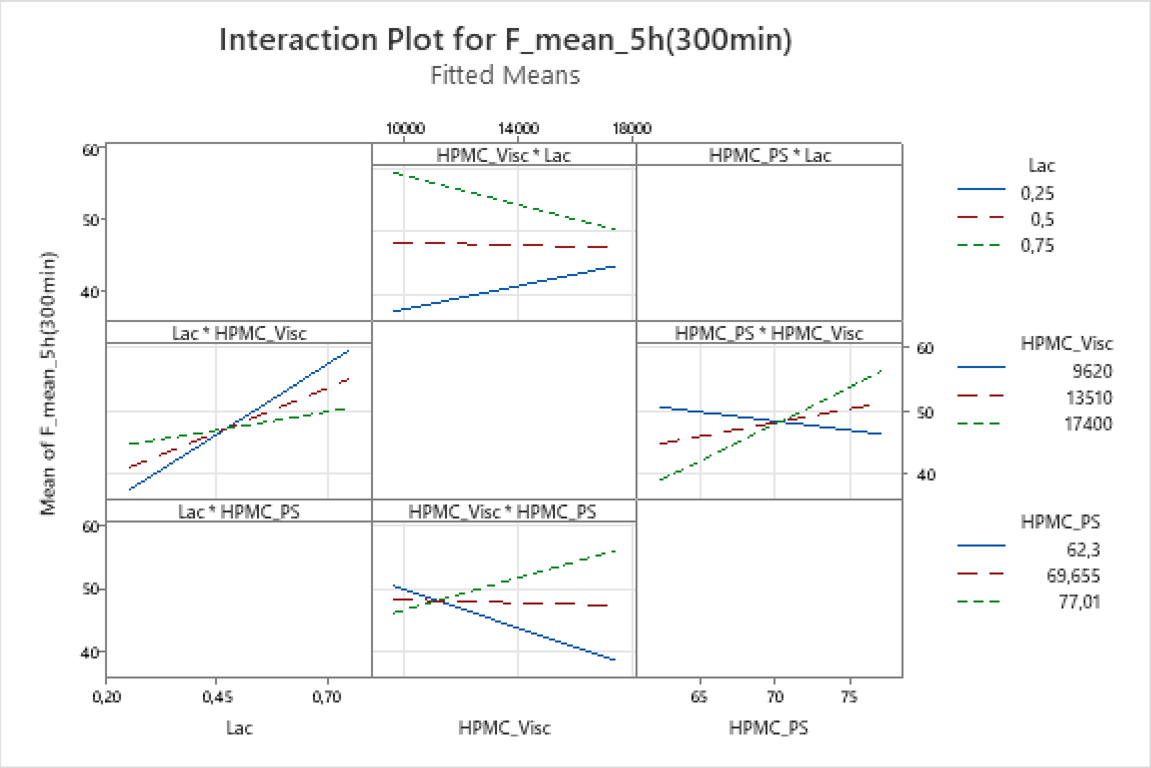

b)

Figure S46. Main effects plot (a) and Interaction plot for mean % of carvedilol release using an Optimized MLR model at t = 5 h (300 min).

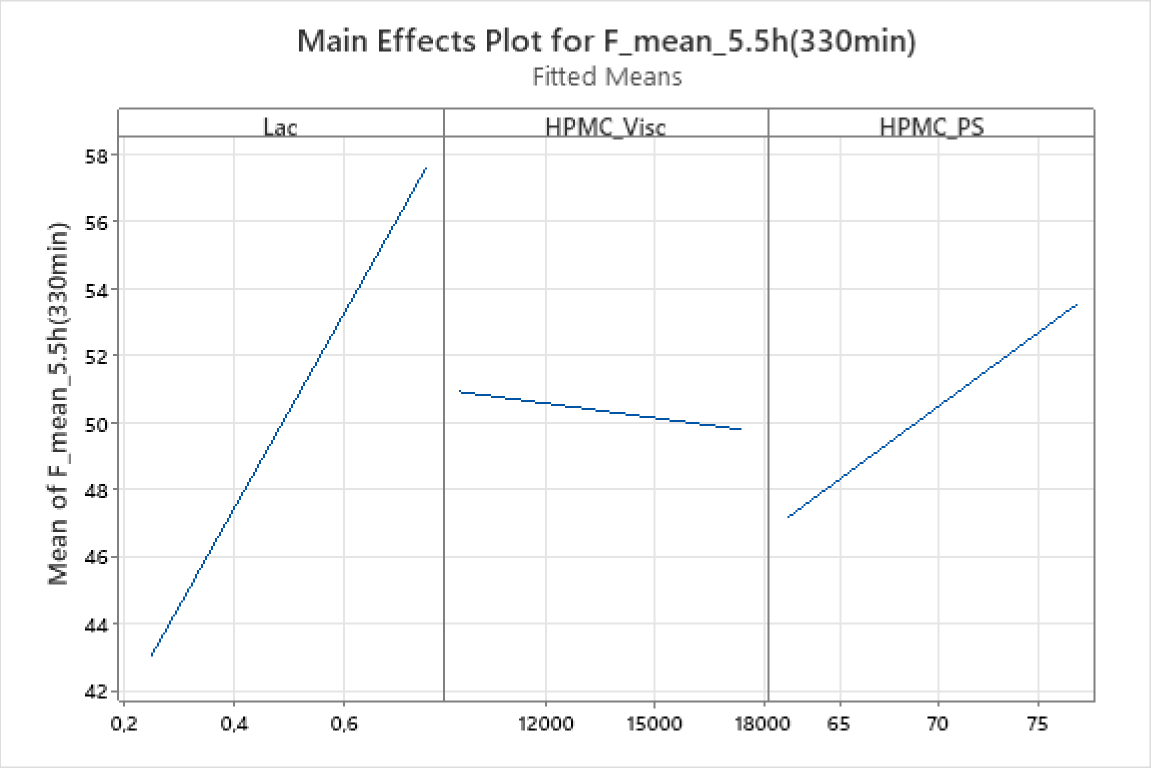

a)

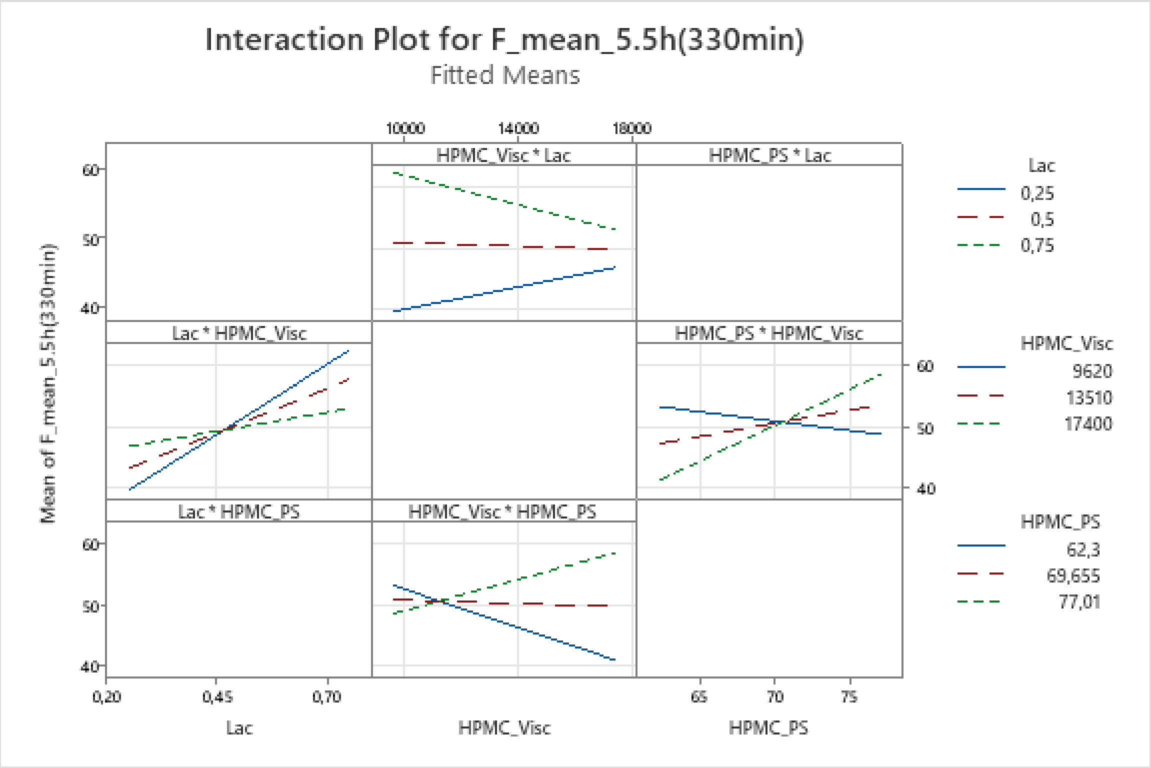

b)

Figure S47. Main effects plot (a) and Interaction plot for mean % of carvedilol release using an Optimized MLR model at t = 5.5 h (330 min).

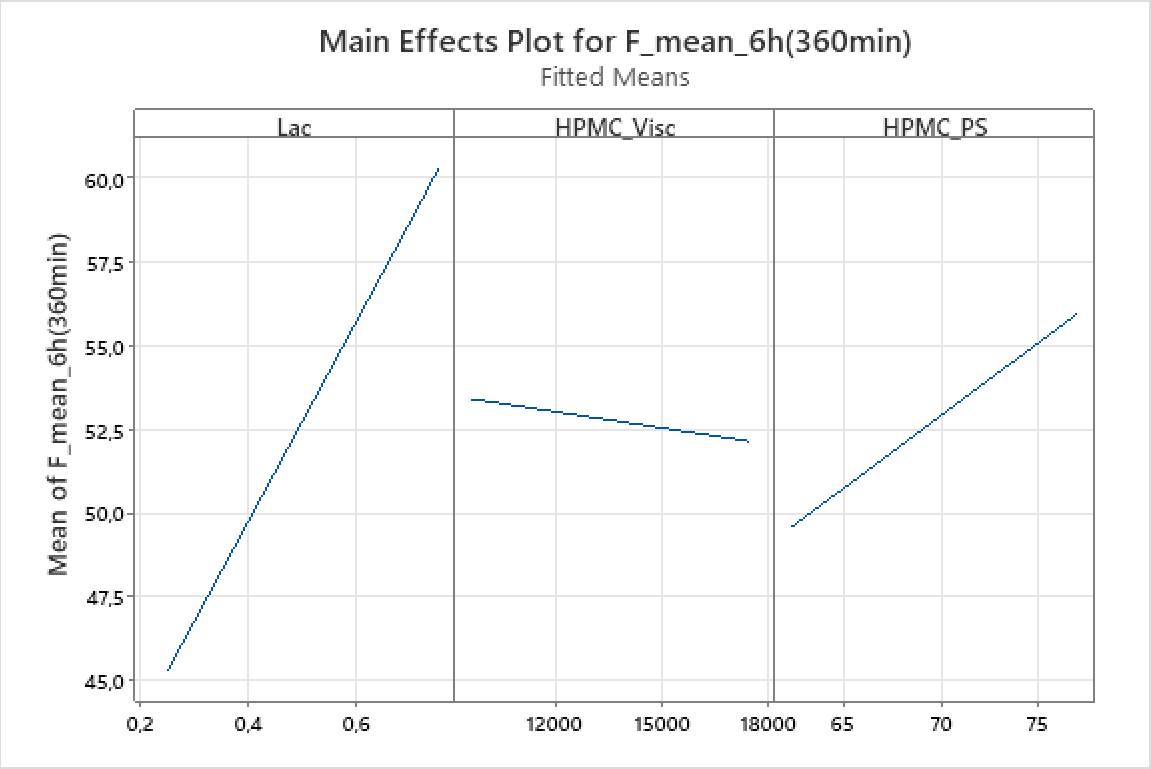

a)

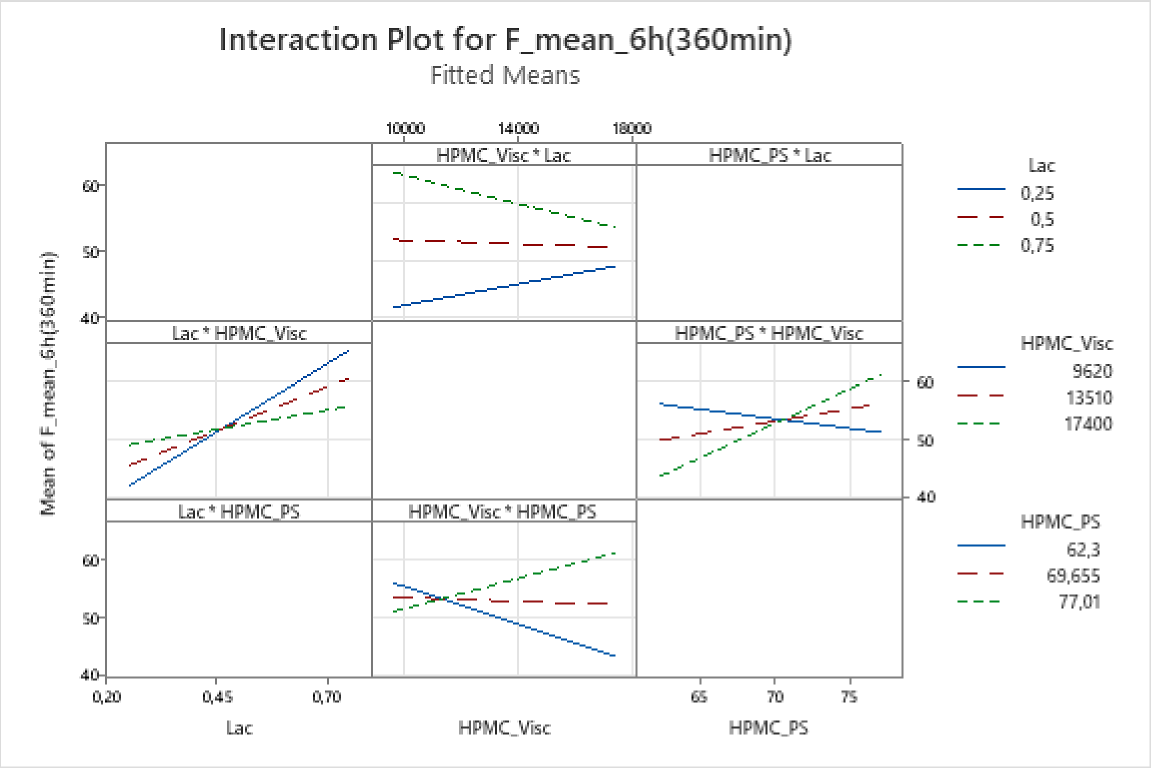

b)

Figure S48. Main effects plot (a) and Interaction plot for mean % of carvedilol release using an Optimized MLR model at t = 6 h (360 min).

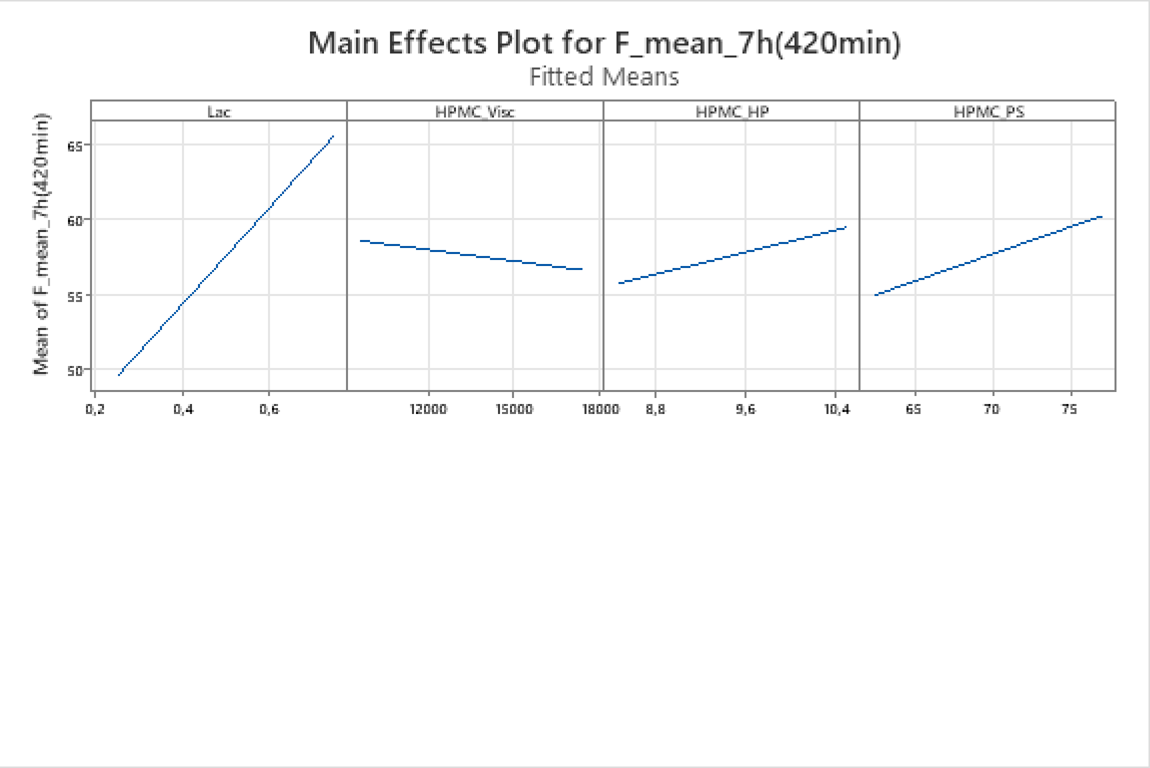

a)

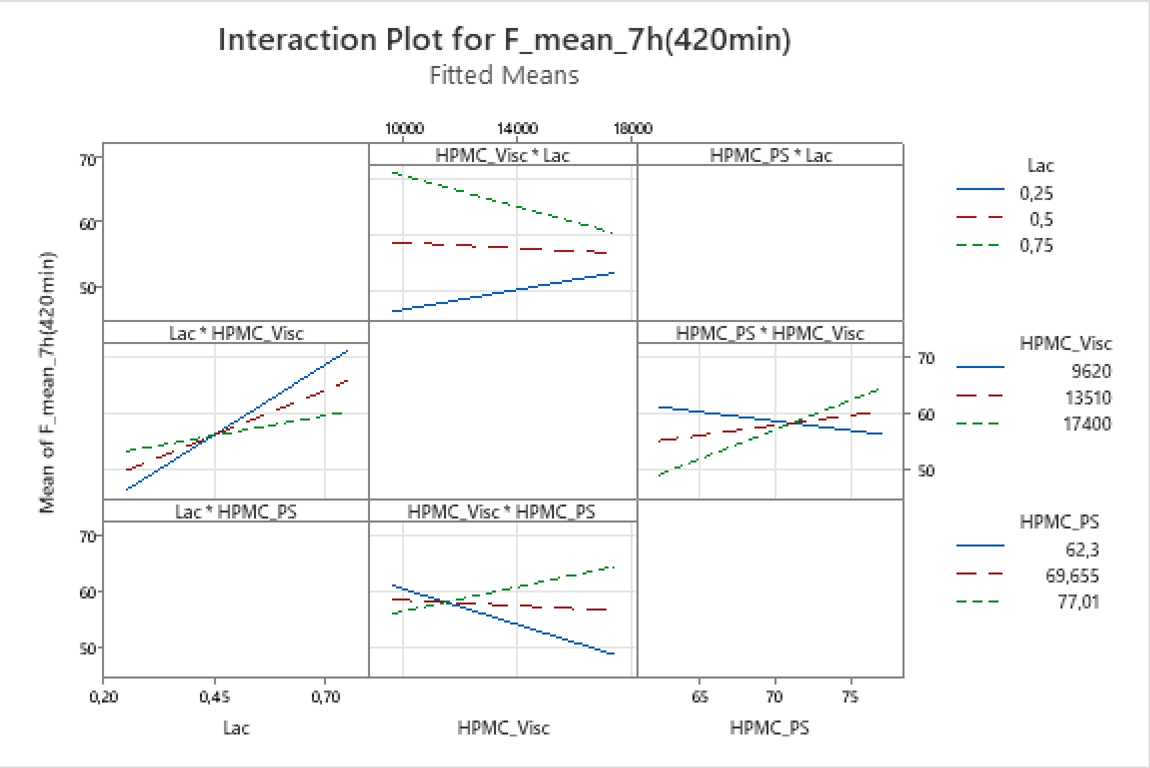

b)

Figure S49. Main effects plot (a) and Interaction plot for mean % of carvedilol release using an Optimized MLR model at t = 7 h (420 min).

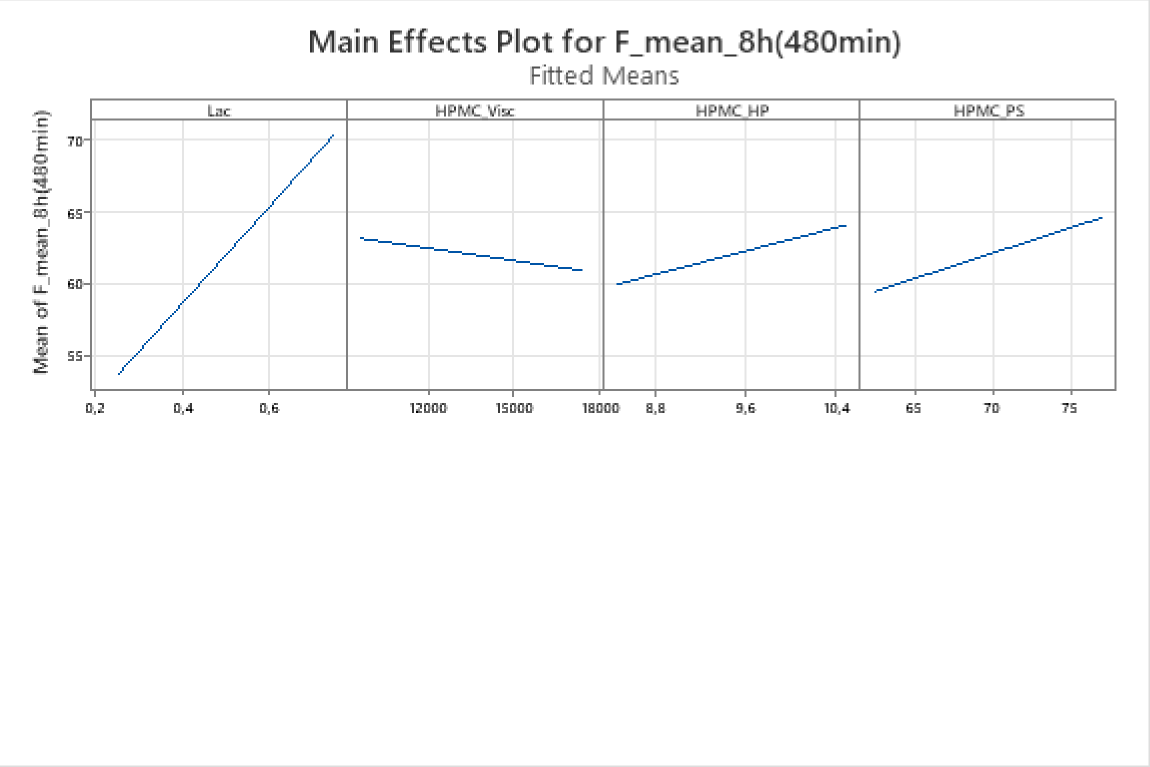

a)

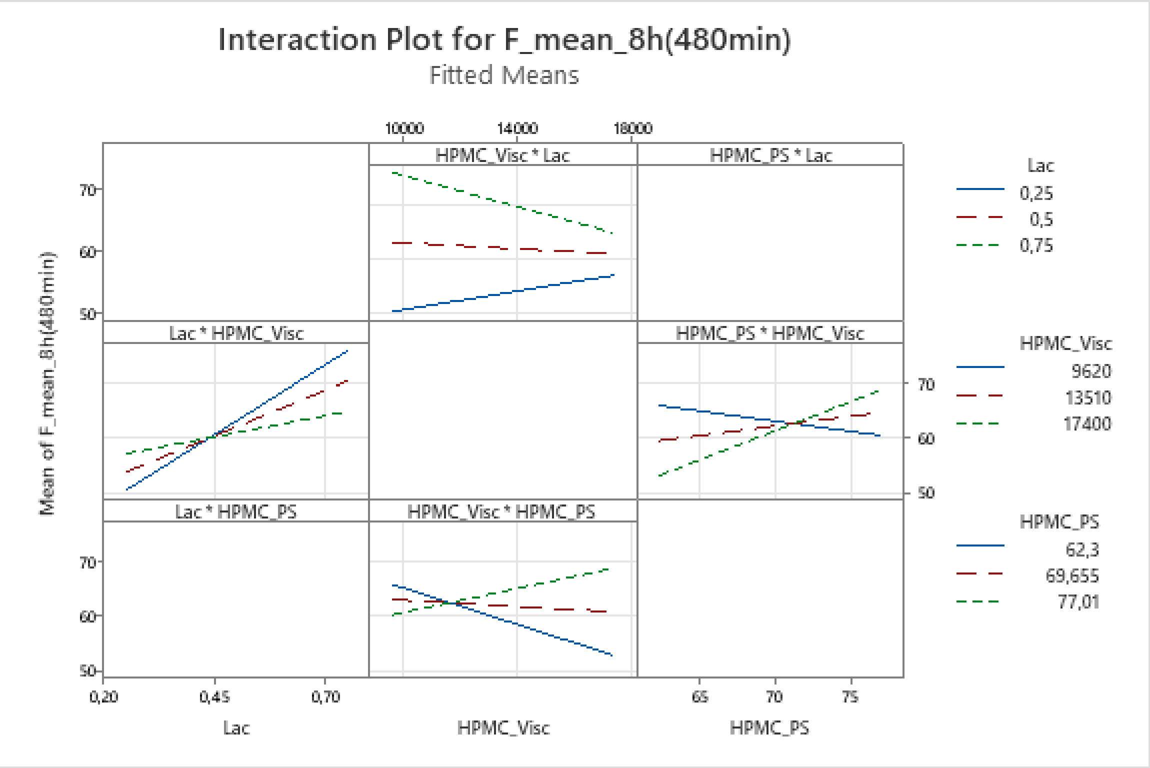

b)

Figure S50. Main effects plot (a) and Interaction plot for mean % of carvedilol release using an Optimized MLR model at t = 8 h (480 min).

a) 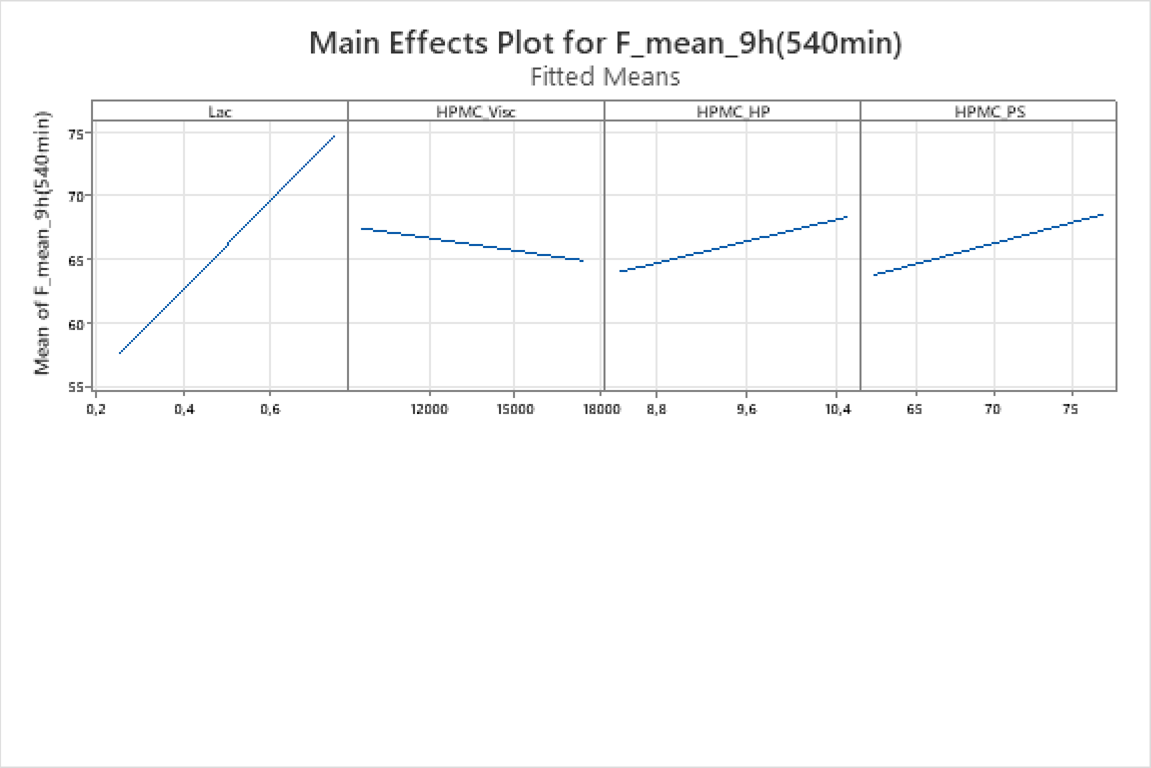

b) 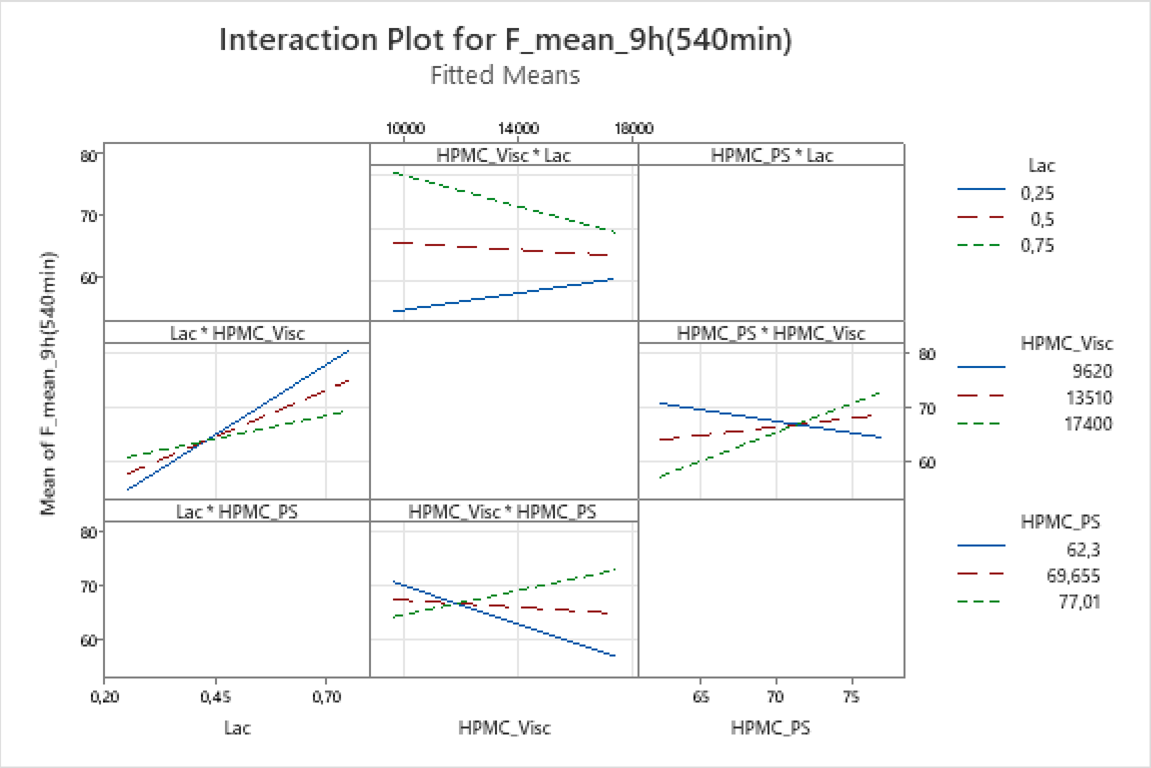

Figure S51. Main effects plot (a) and Interaction plot for mean % of carvedilol release using an Optimized MLR model at t = 9 h (540 min).

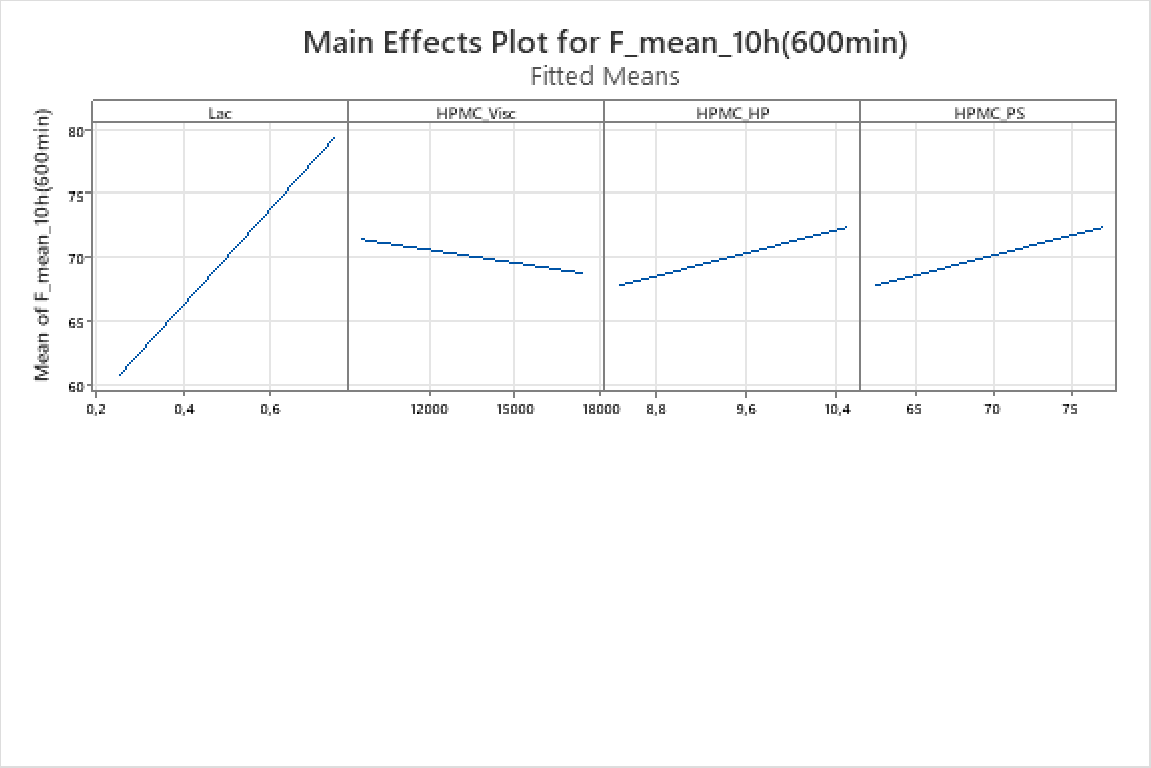

a)

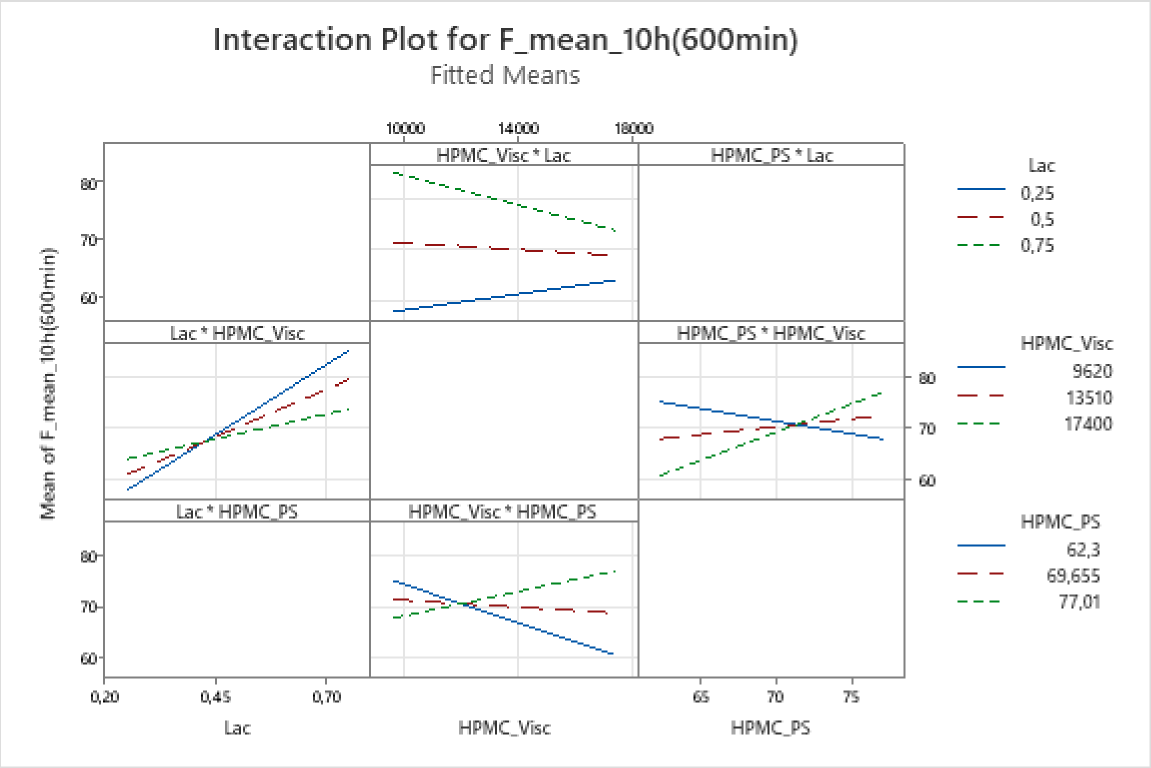

b)

Figure S52. Main effects plot (a) and Interaction plot for mean % of carvedilol release using an Optimized MLR model at t = 10 h (600 min).

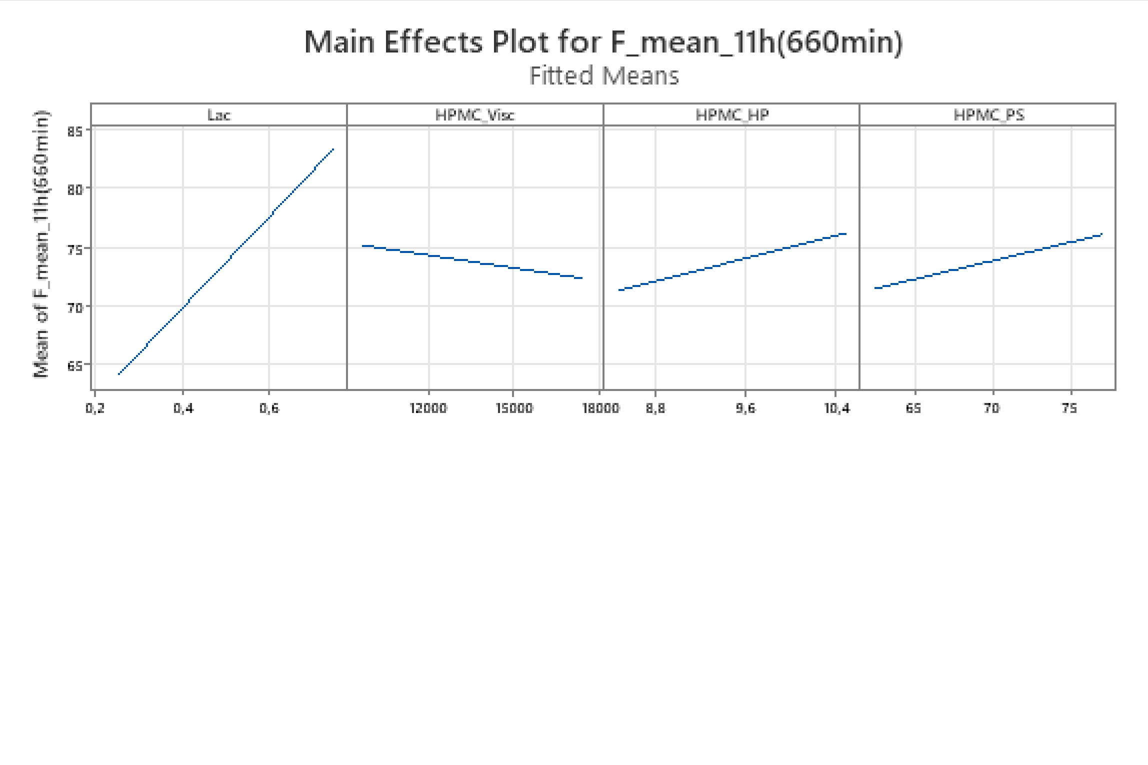

a)

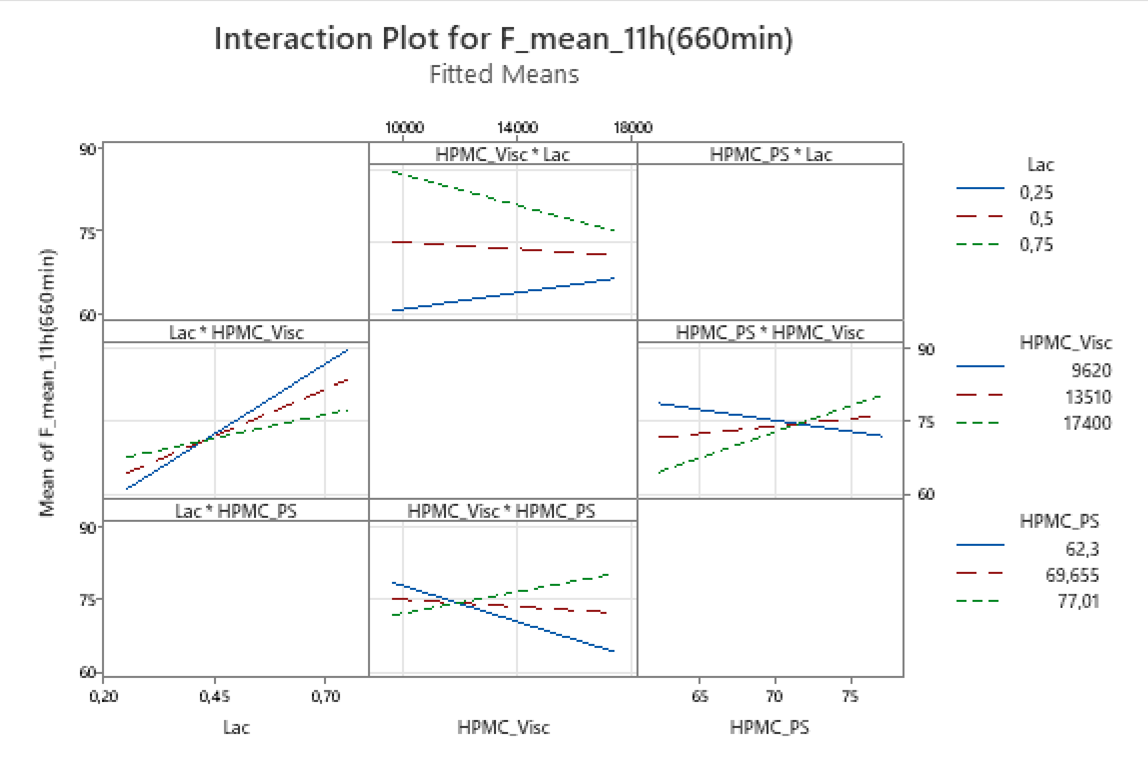

b)

Figure S53. Main effects plot (a) and Interaction plot for mean % of carvedilol release using an Optimized MLR model at t = 11 h (660 min).

a) 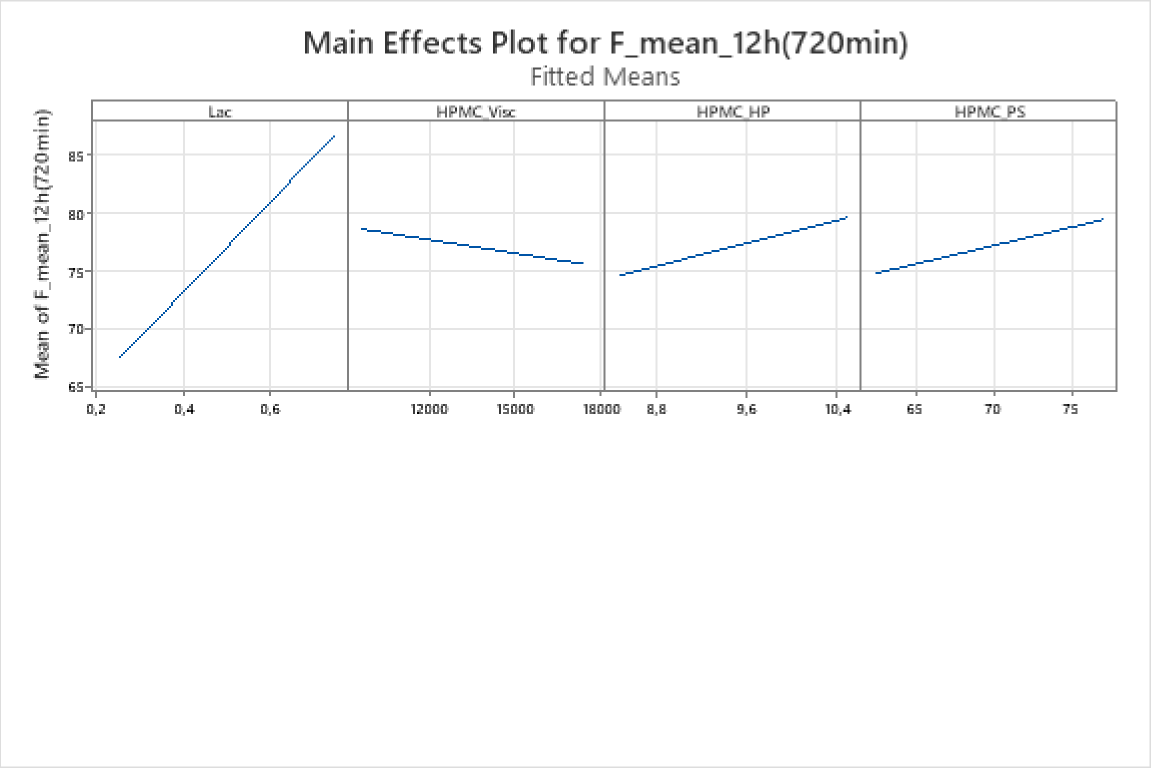

b) 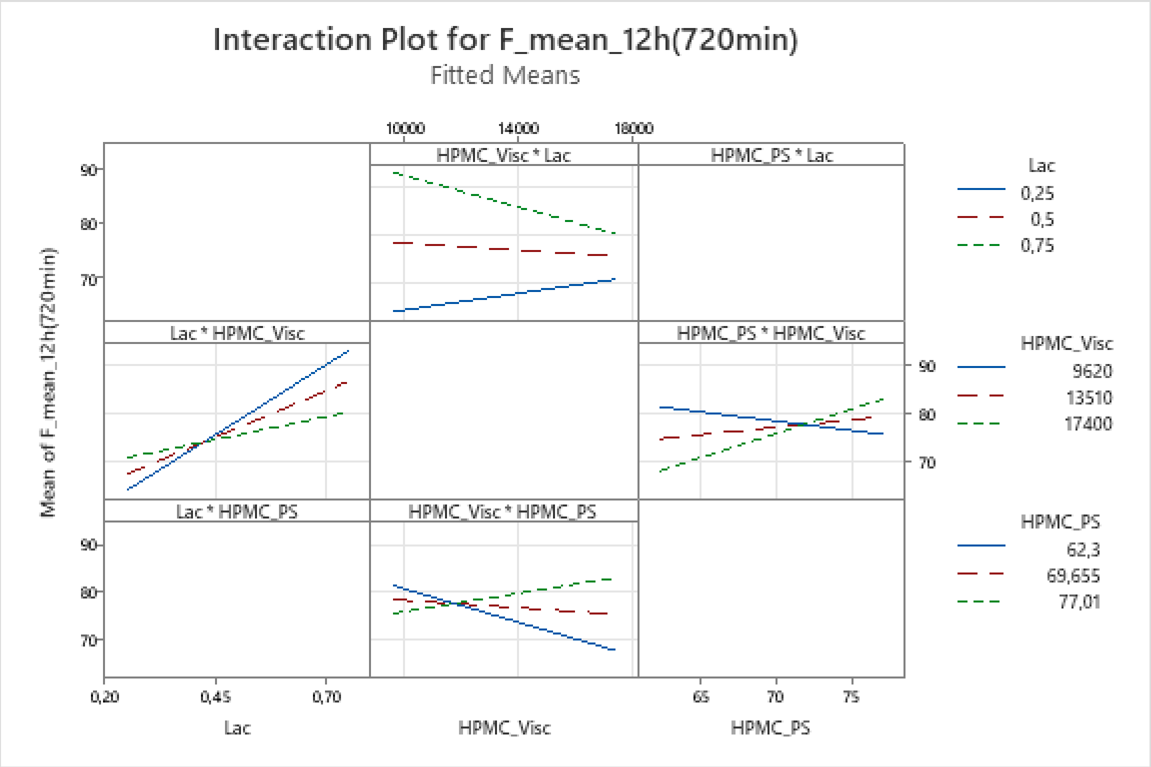

Figure S54. Main effects plot (a) and Interaction plot for mean % of carvedilol release using an Optimized MLR model at t = 12 h (720 min).

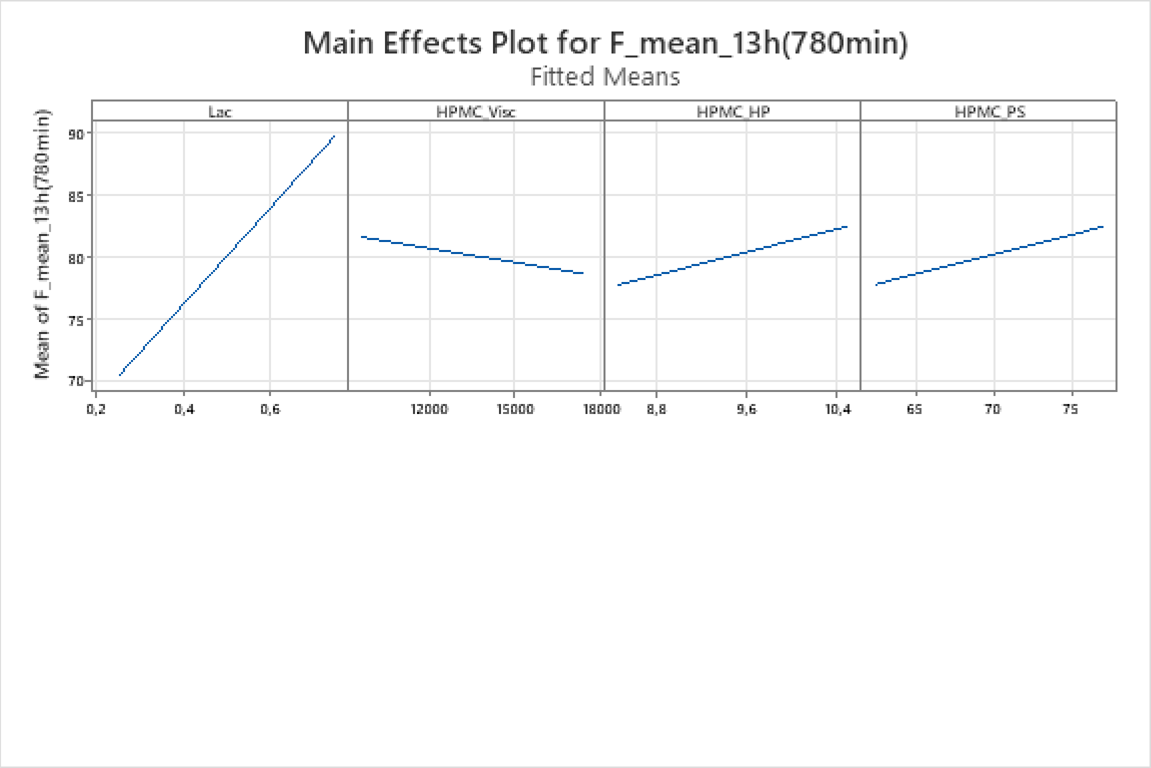

a)

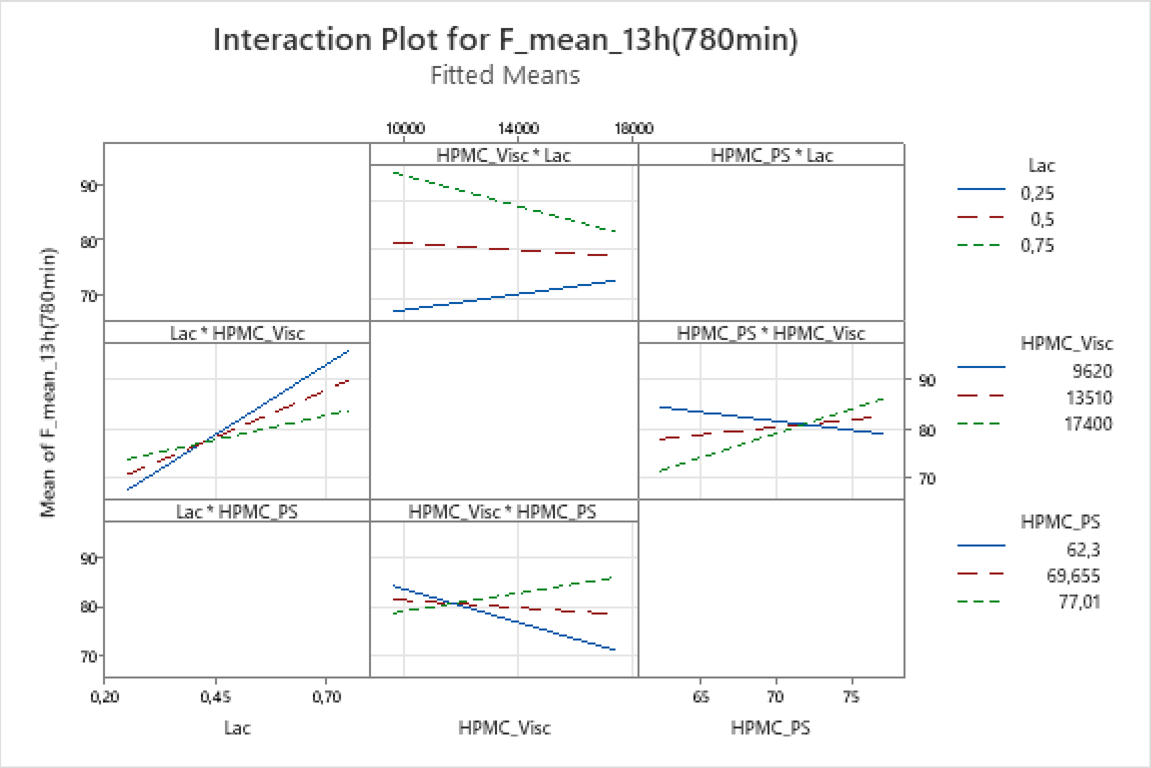

b)

Figure S55. Main effects plot (a) and Interaction plot for mean % of carvedilol release using an Optimized MLR model at t = 13 h (780 min).

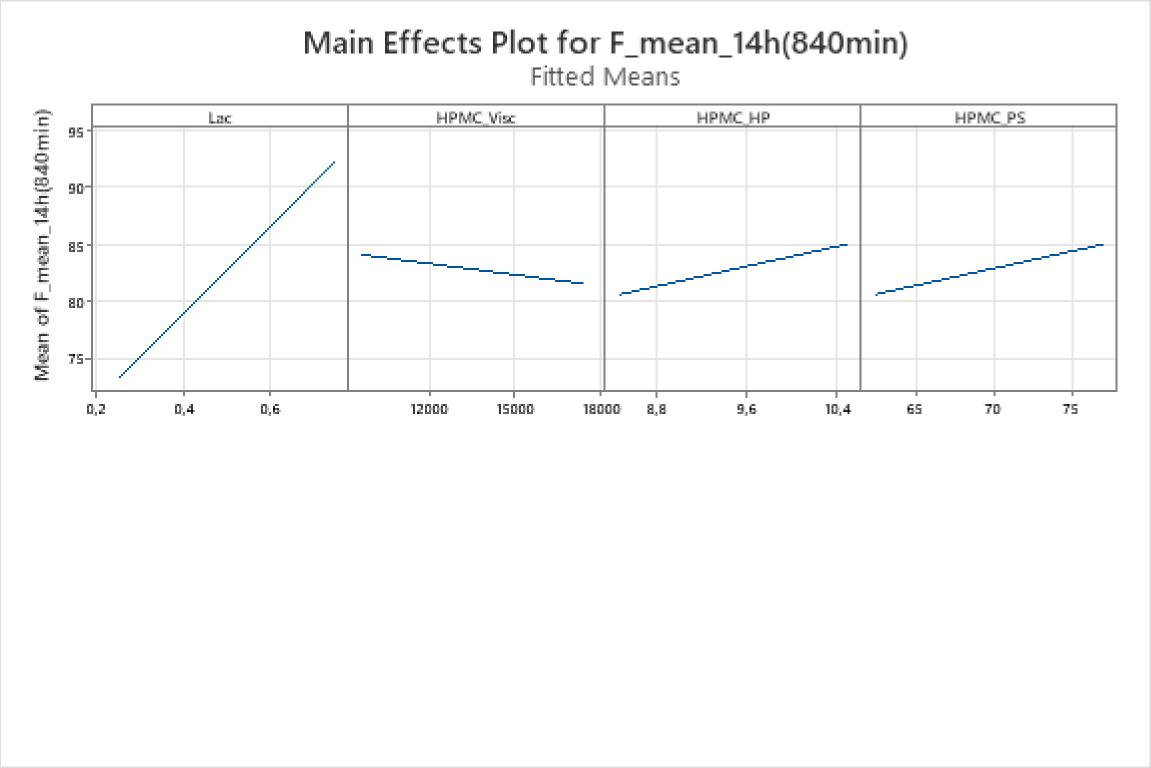

a)

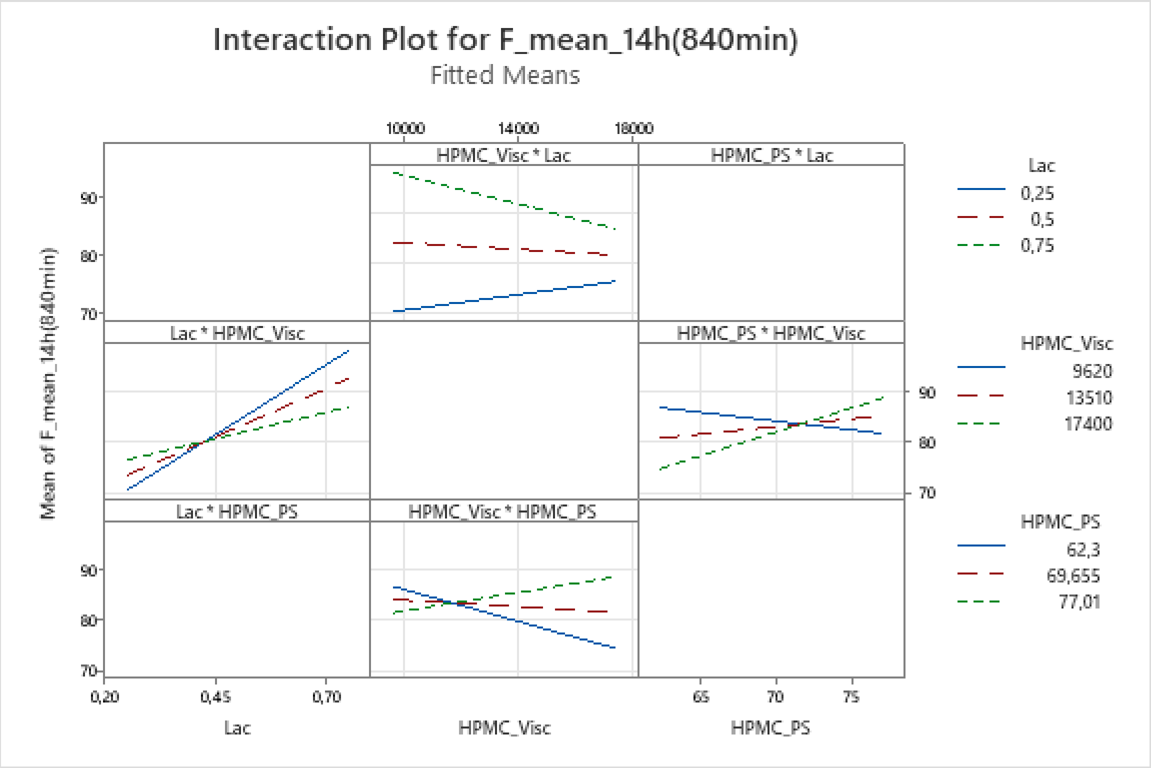

b)

Figure S56. Main effects plot (a) and Interaction plot for mean % of carvedilol release using an Optimized MLR model at t = 14 h (840 min).

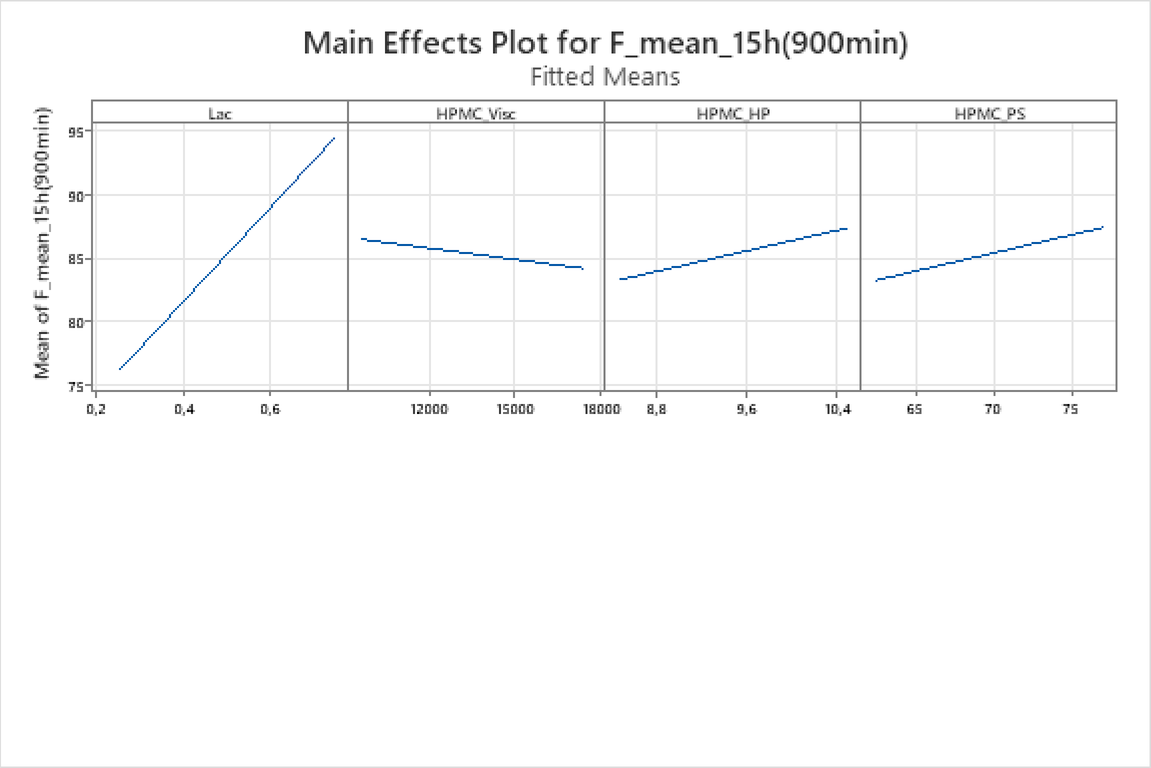

a)

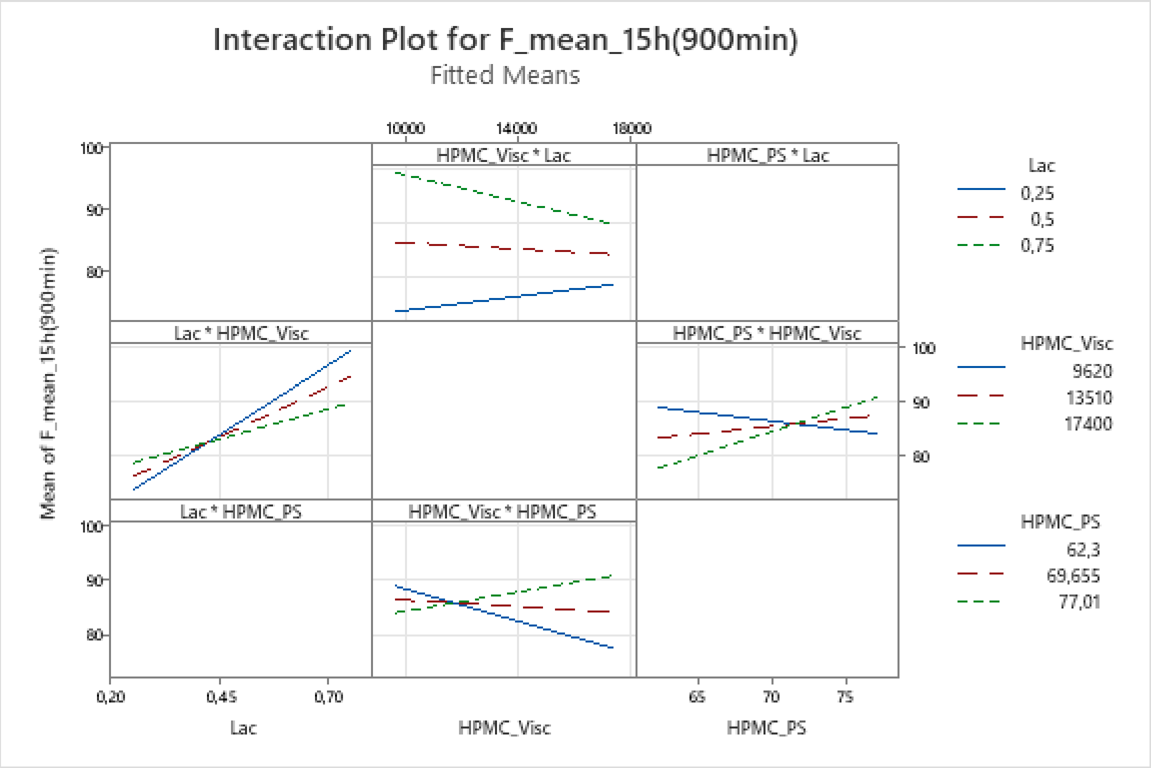

b)

Figure S57. Main effects plot (a) and Interaction plot for mean % of carvedilol release using an Optimized MLR model at t = 15 h (900 min).

a)

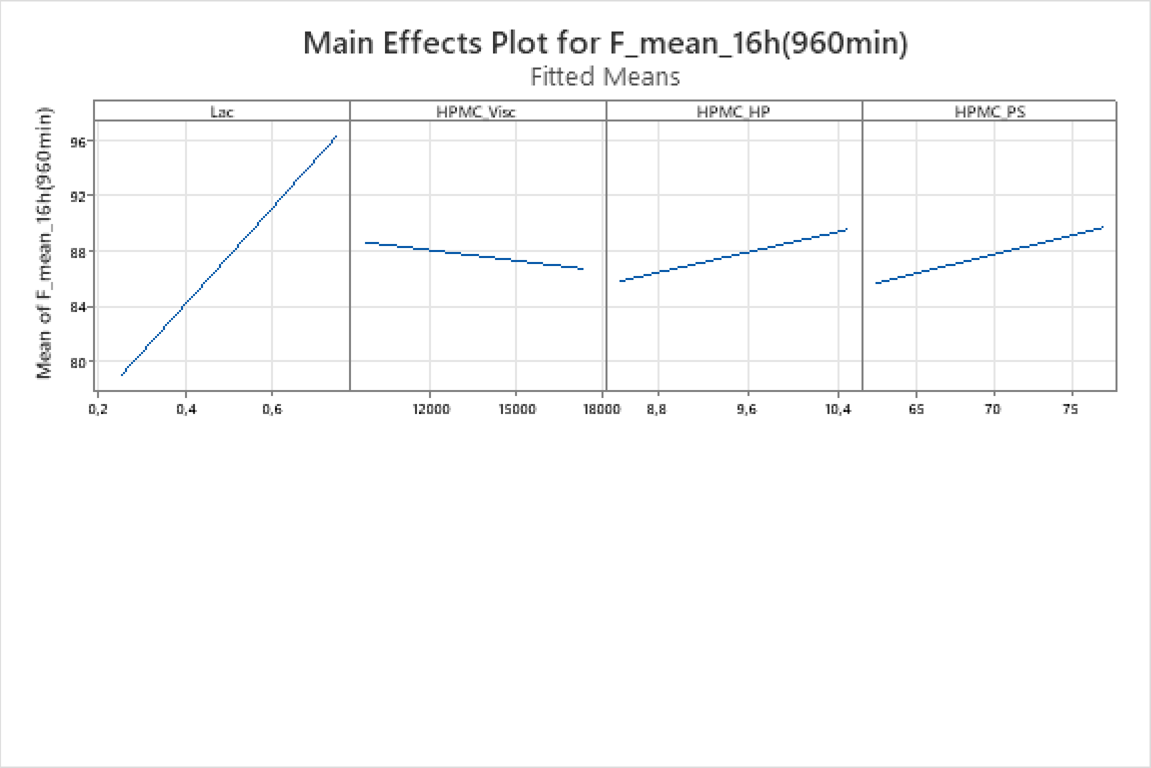

b)

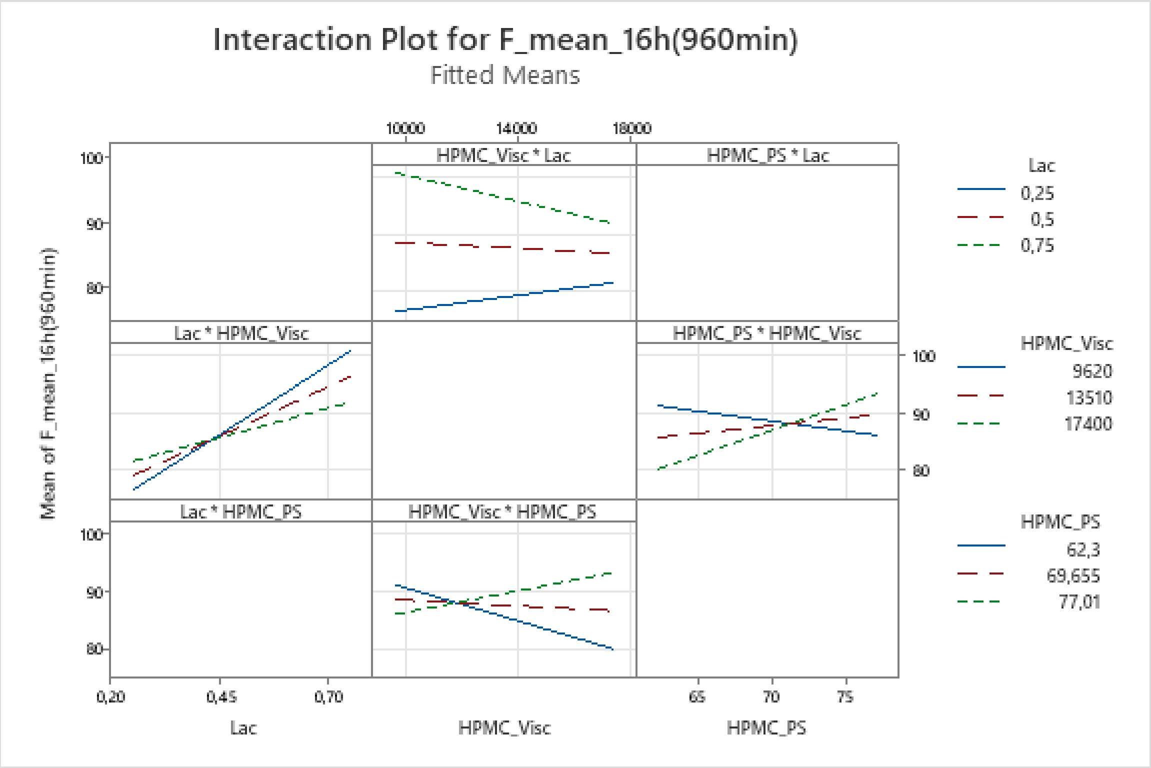

Figure S58. Main effects plot (a) and Interaction plot for mean % of carvedilol release using an Optimized MLR model at t = 16 h (960 min).

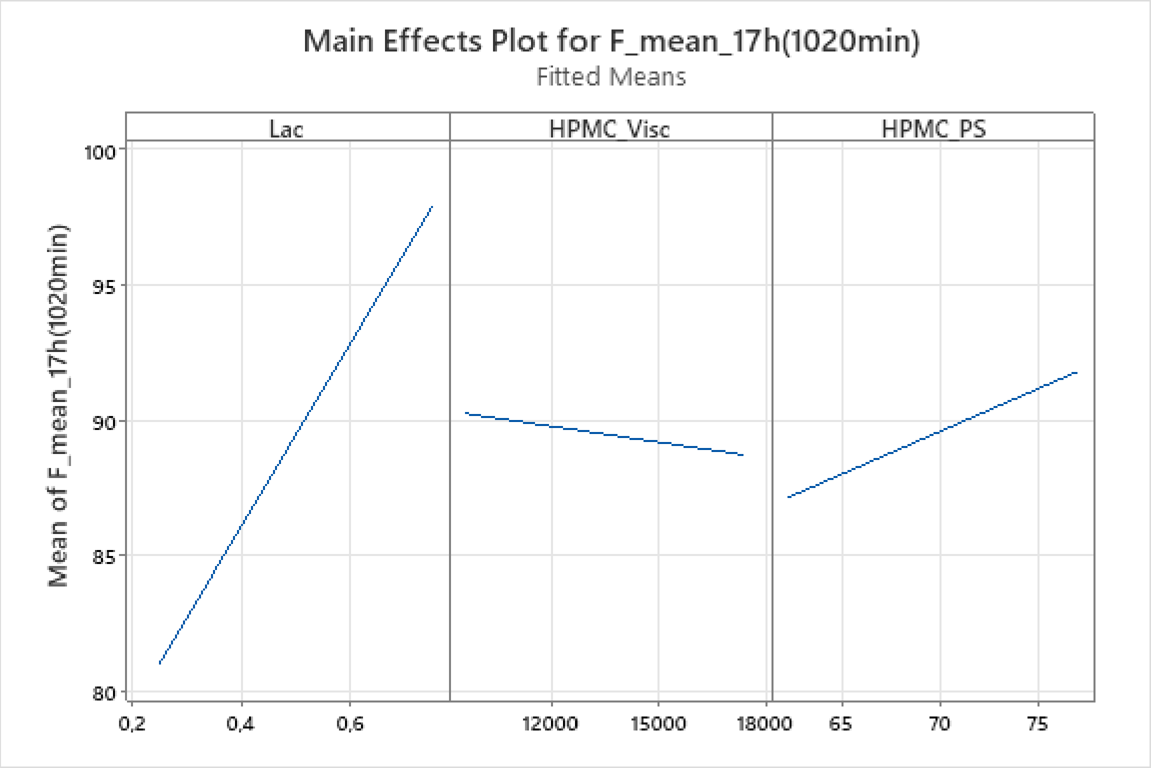

a)

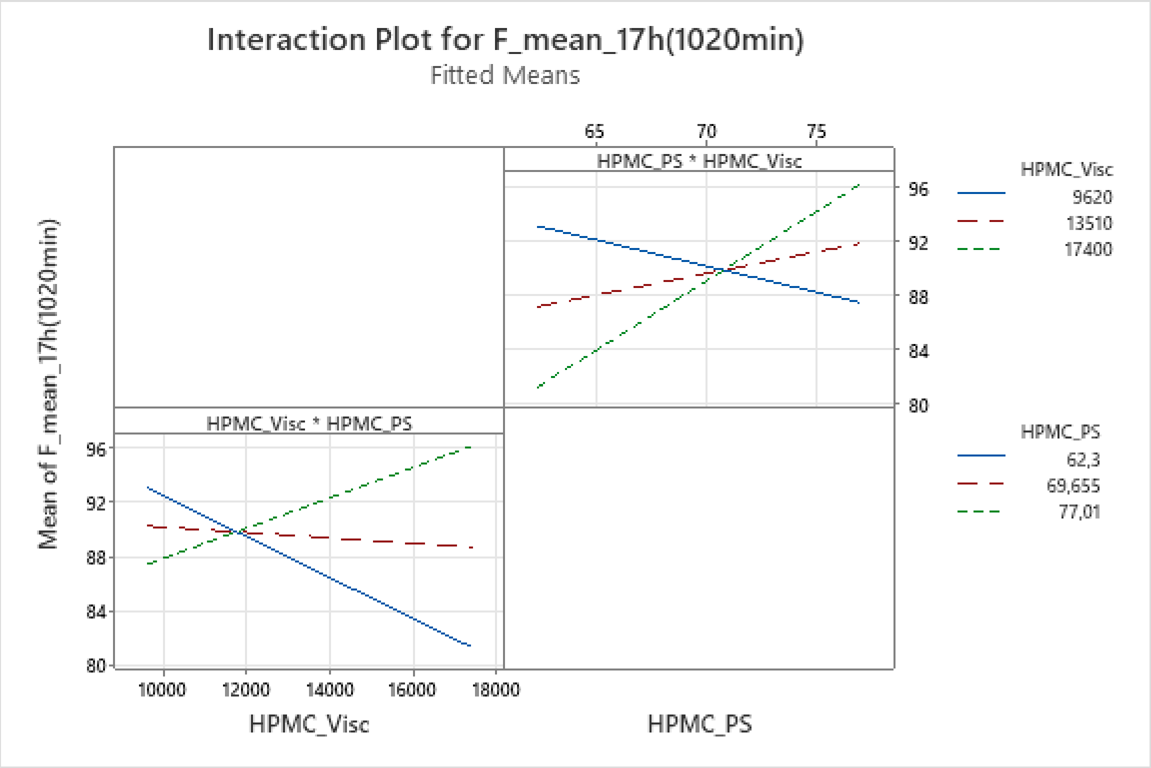

b)

Figure S59. Main effects plot (a) and Interaction plot for mean % of carvedilol release using an Optimized MLR model at t = 17 h (1020 min).

a)

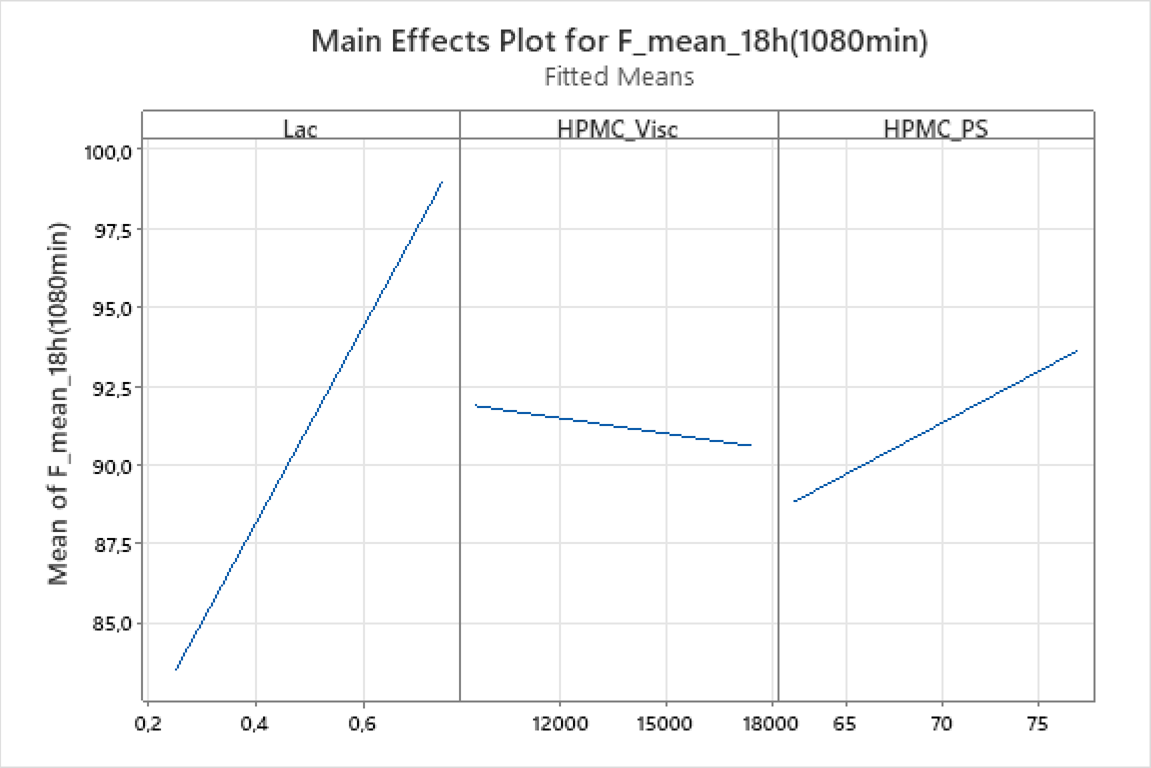

b)

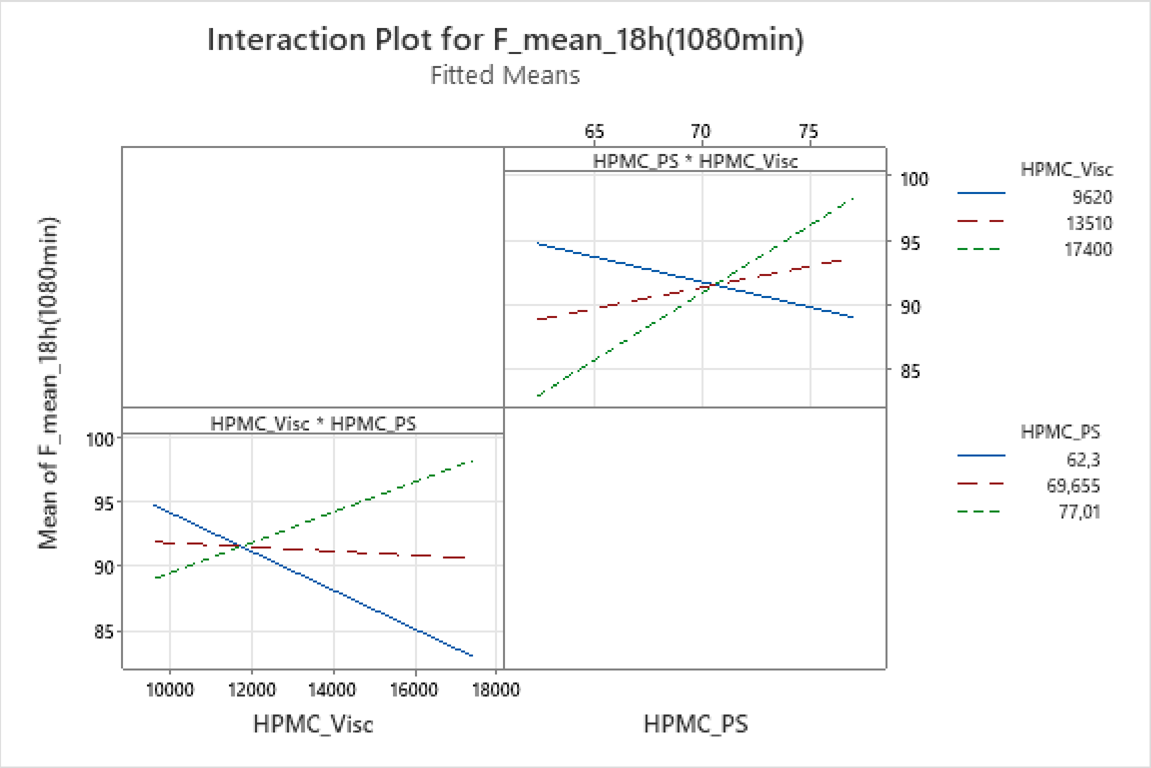

Figure S60. Main effects plot (a) and Interaction plot for mean % of carvedilol release using an Optimized MLR model at t = 18 h (1080 min).

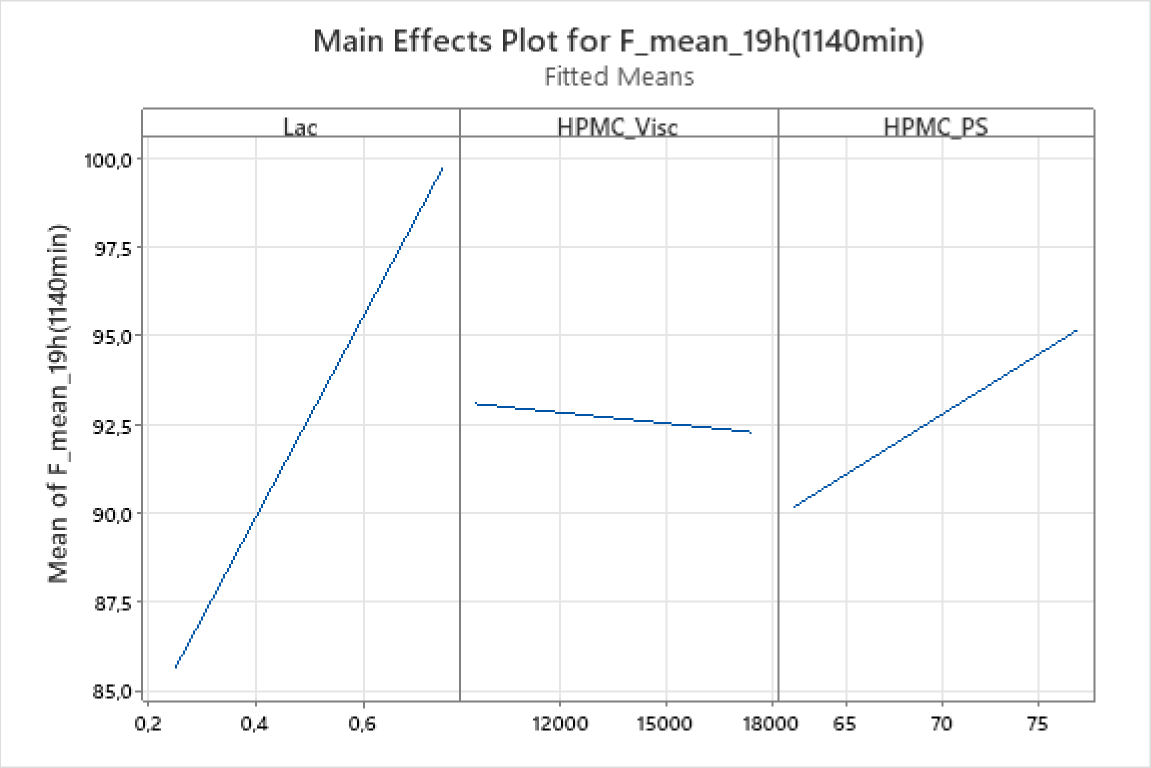

a)

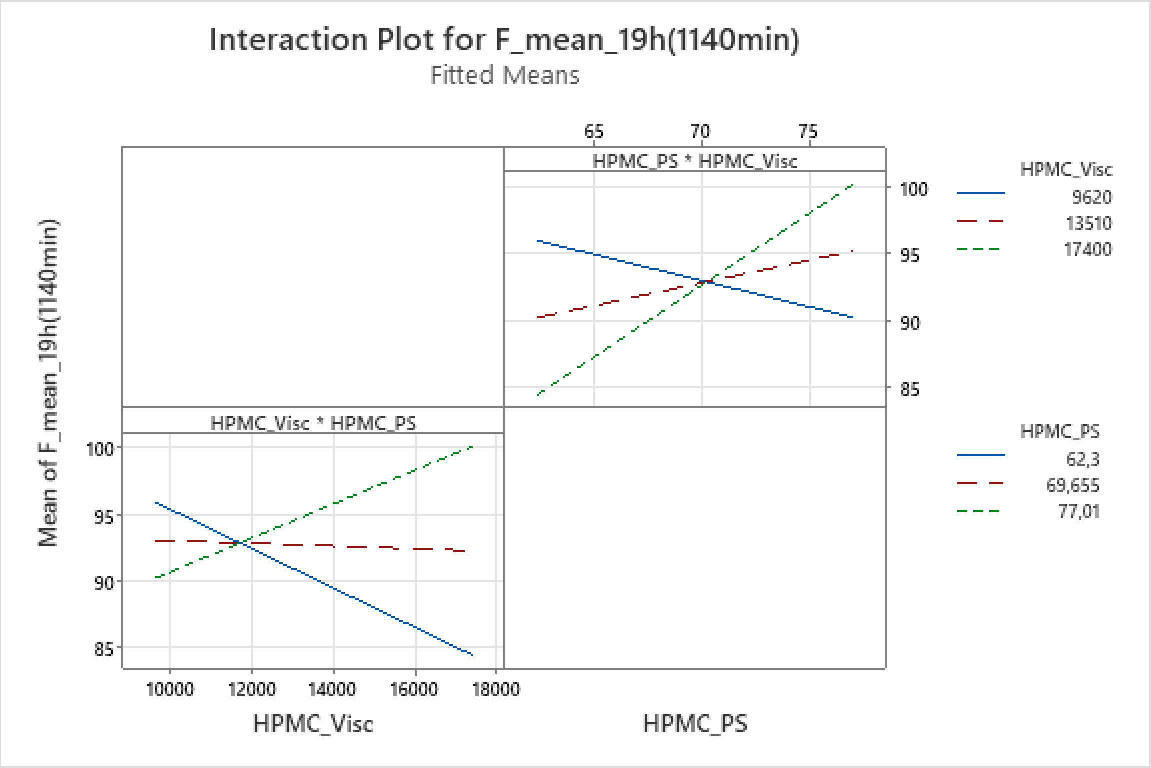

b)

Figure S61. Main effects plot (a) and Interaction plot for mean % of carvedilol release using an Optimized MLR model at t = 19 h (1140 min).

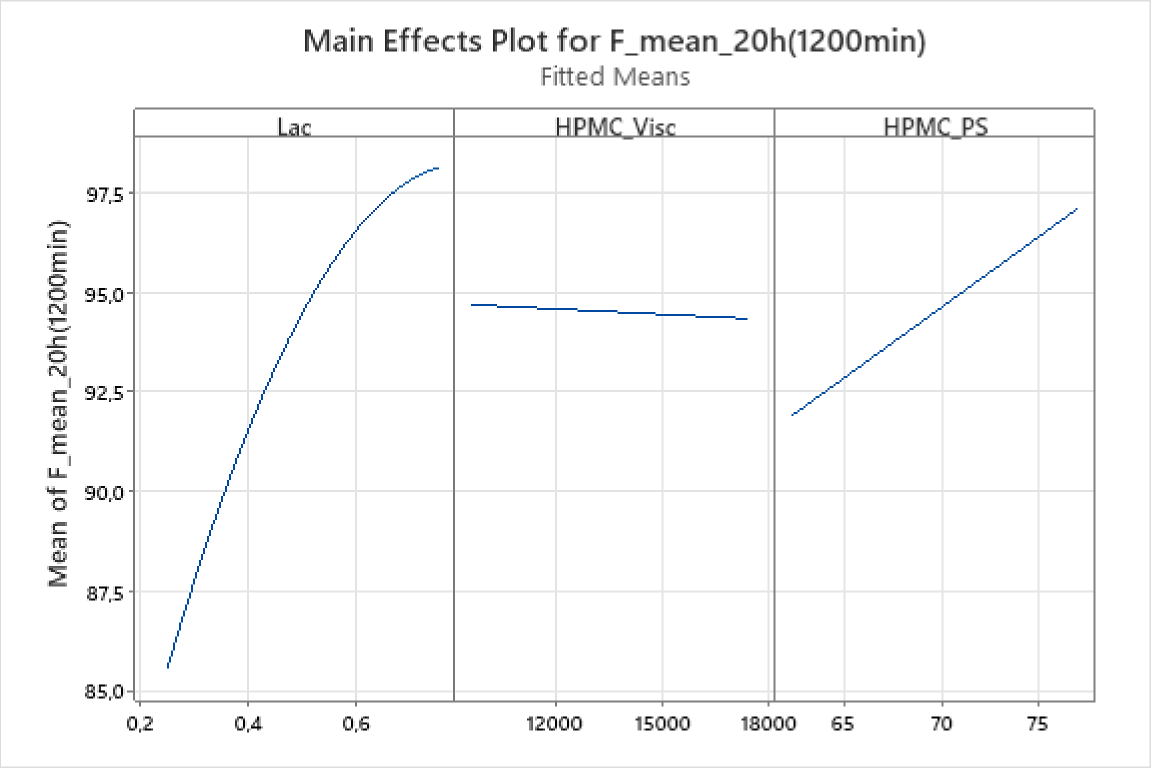

a)

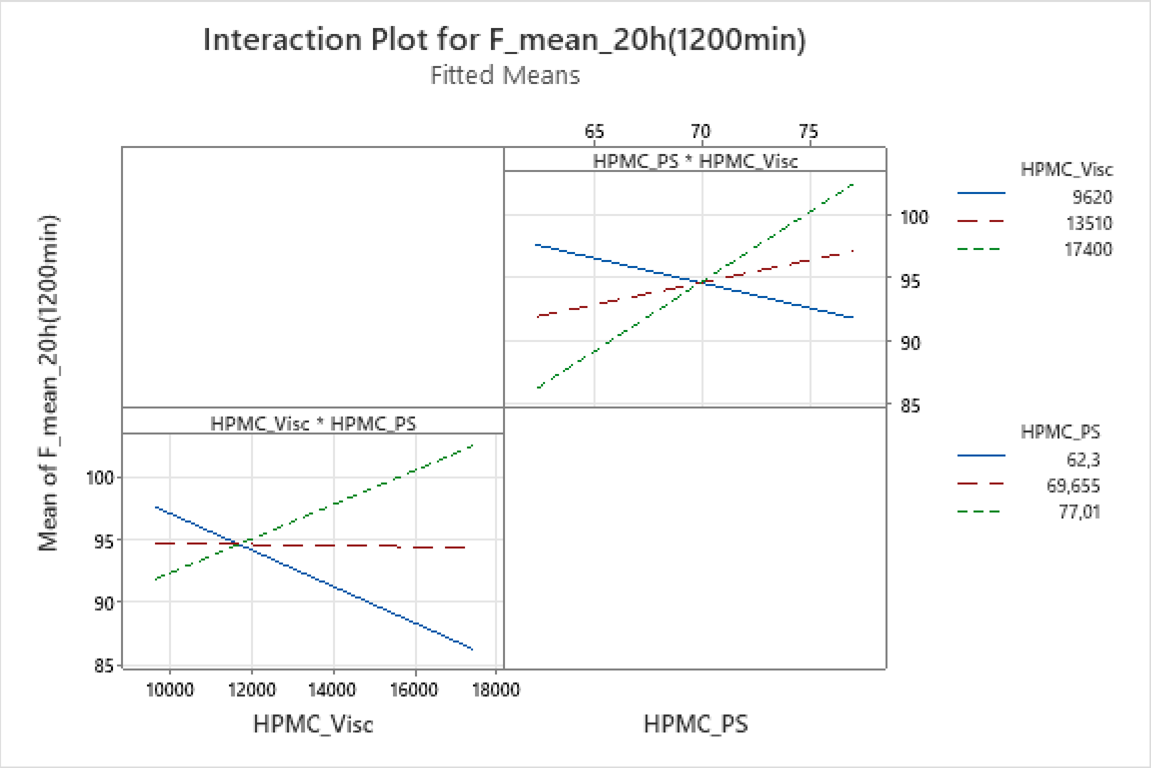

b)

Figure S62. Main effects plot (a) and Interaction plot for mean % of carvedilol release using an Optimized MLR model at t = 20 h (1200 min).

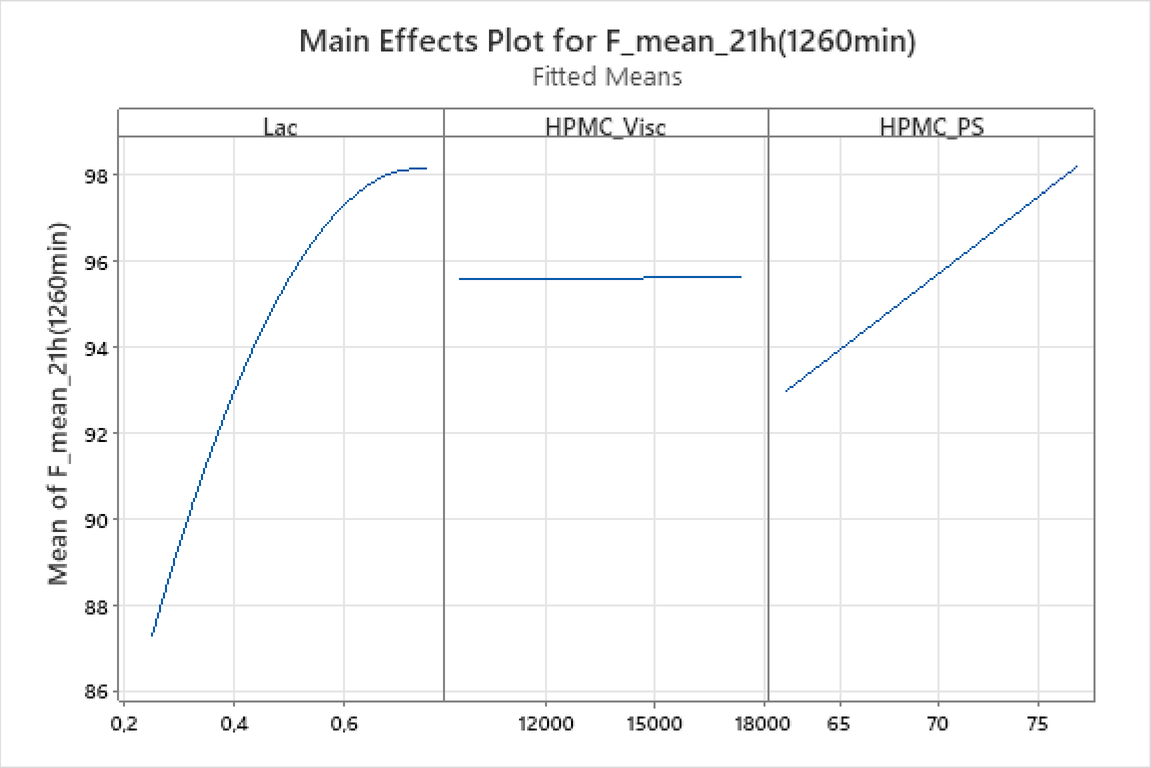

a)

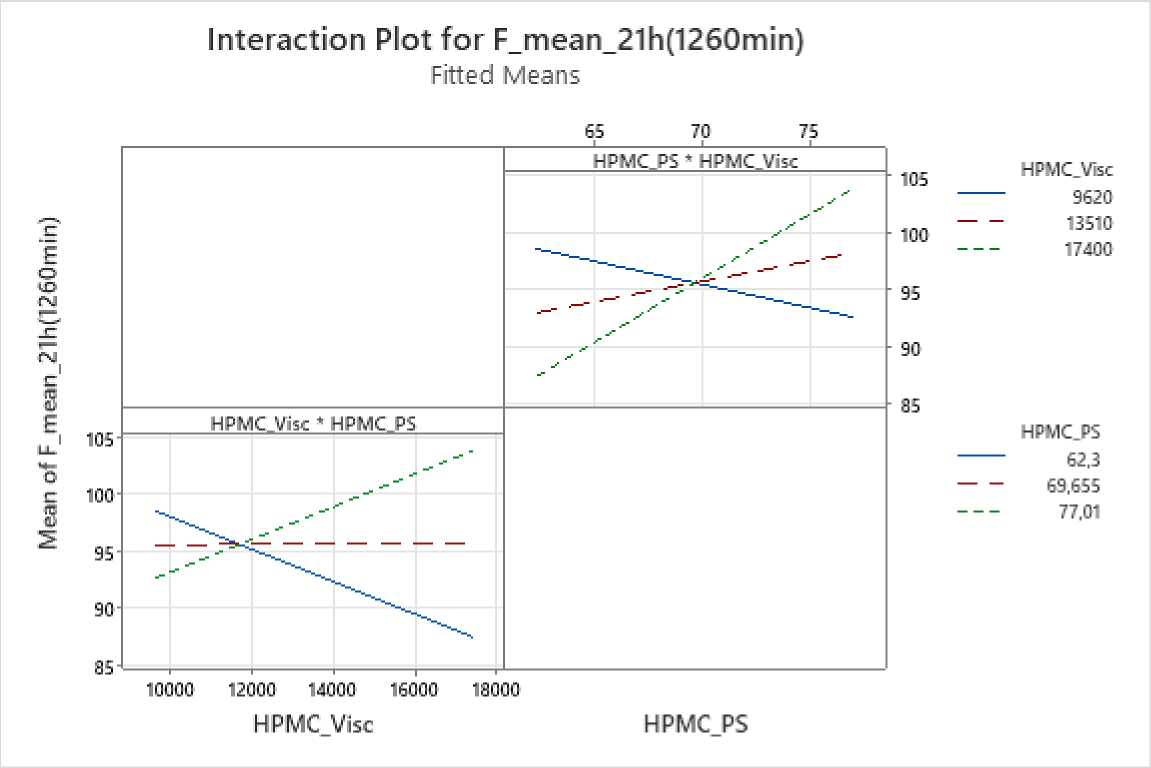

b)

Figure S63. Main effects plot (a) and Interaction plot for mean % of carvedilol release using an Optimized MLR model at t = 21 h (1260 min).

a)

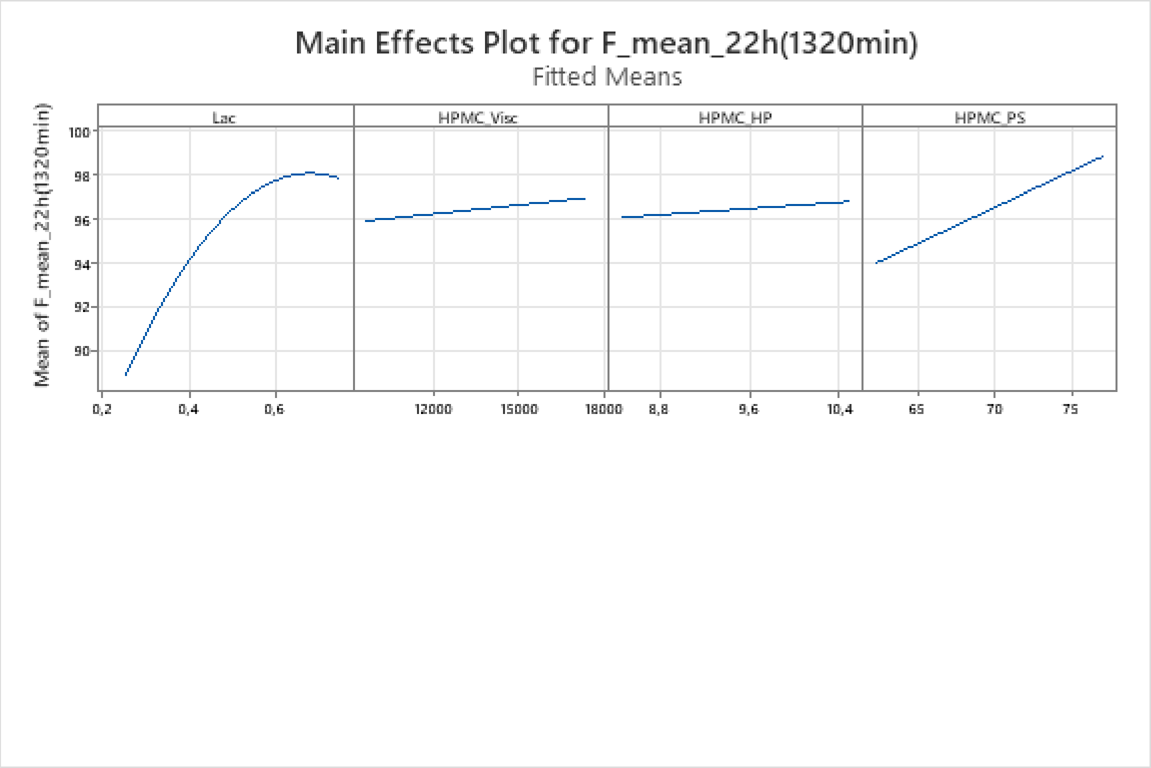

b)

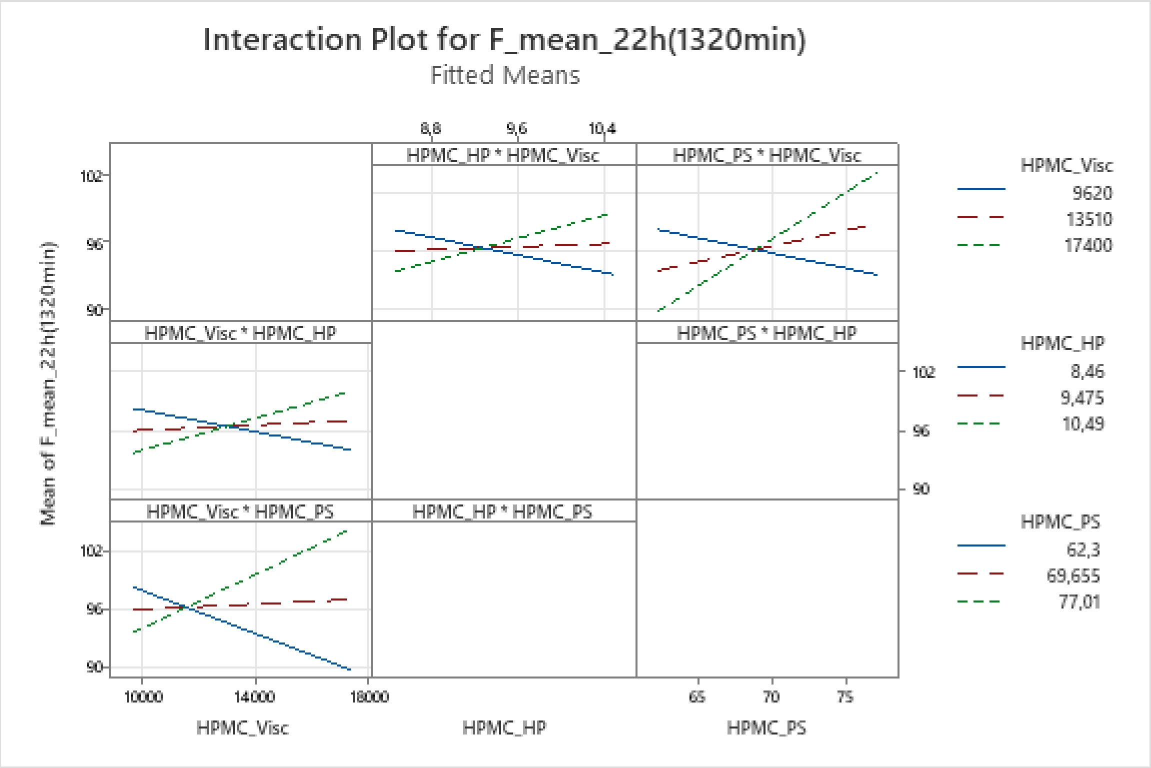

Figure S64. Main effects plot (a) and Interaction plot for mean % of carvedilol release using an Optimized MLR model at t = 22 h (1320 min).

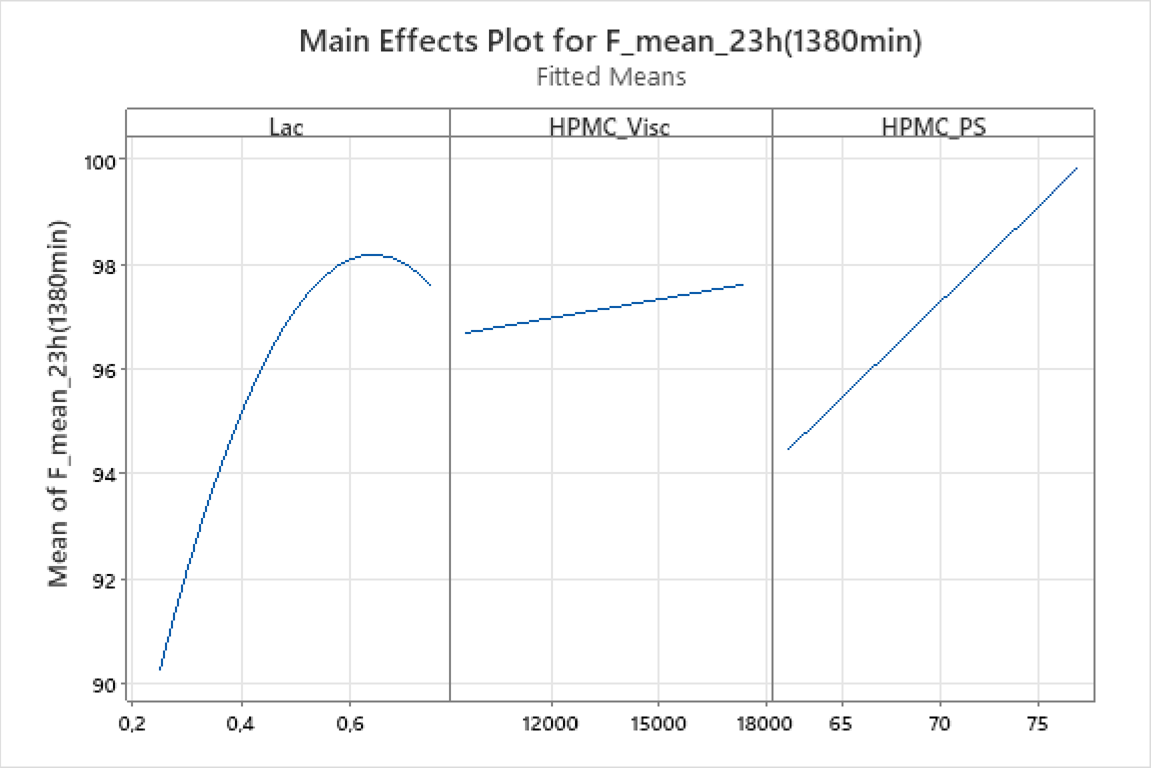

a)

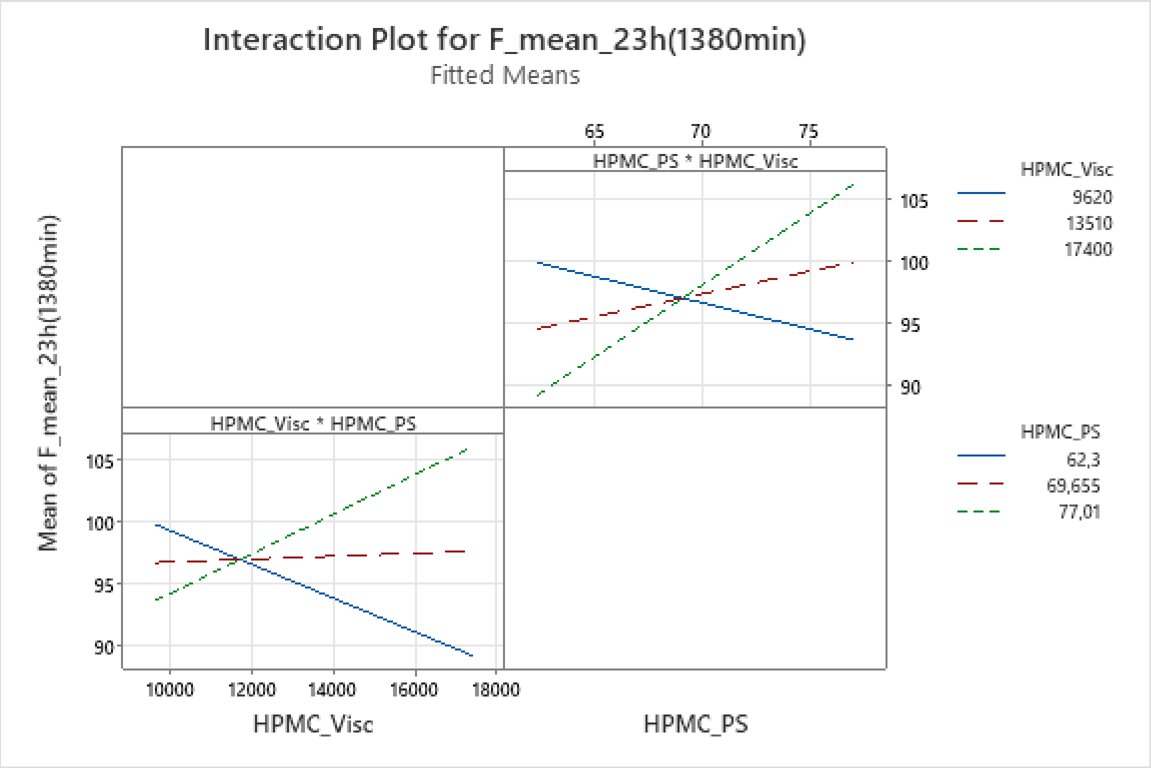

b)

Figure S65. Main effects plot (a) and Interaction plot for mean % of carvedilol release using an Optimized MLR model at t = 23 h (1380 min).

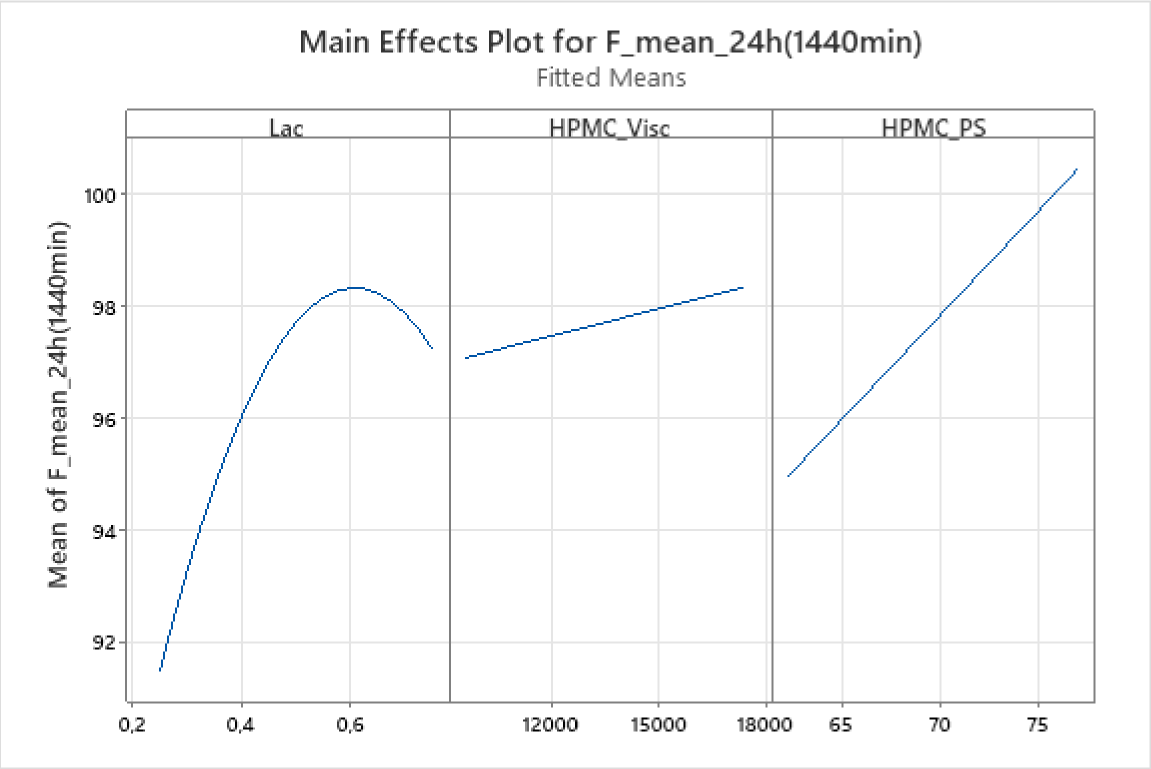

a)

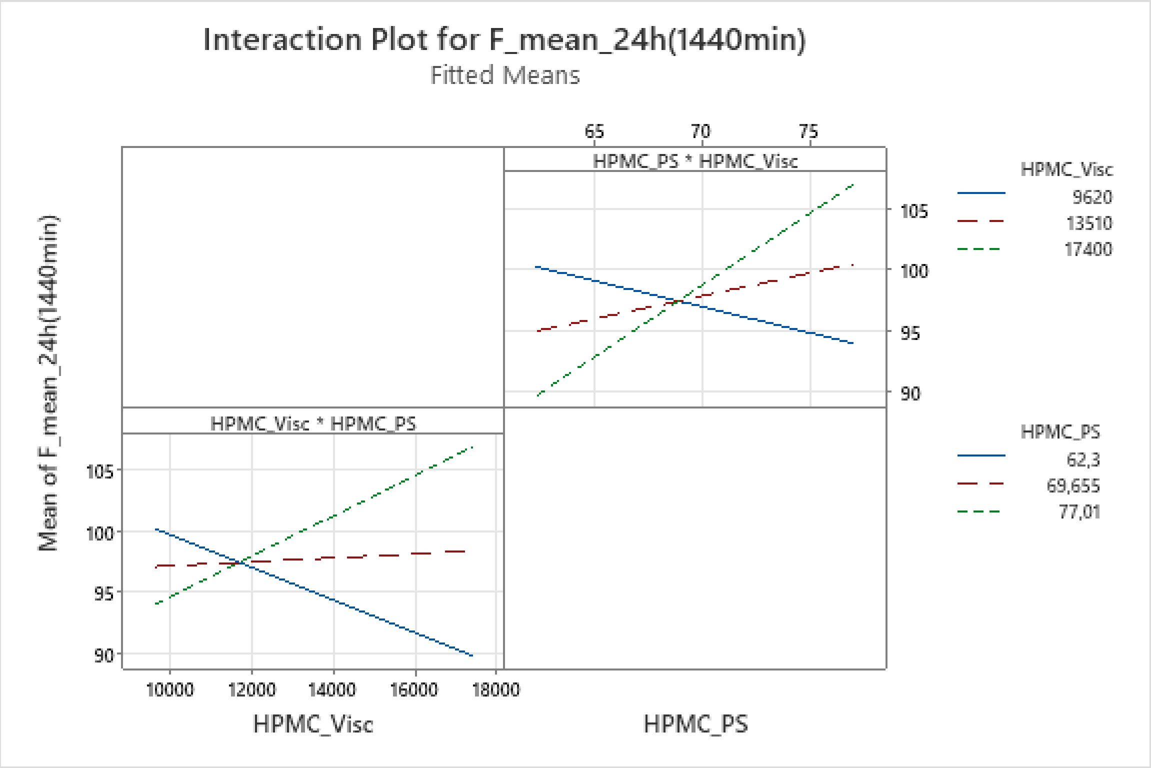

b)

Figure S66. Main effects plot (a) and Interaction plot for mean % of carvedilol release using an Optimized MLR model at t = 24 h (1440 min).

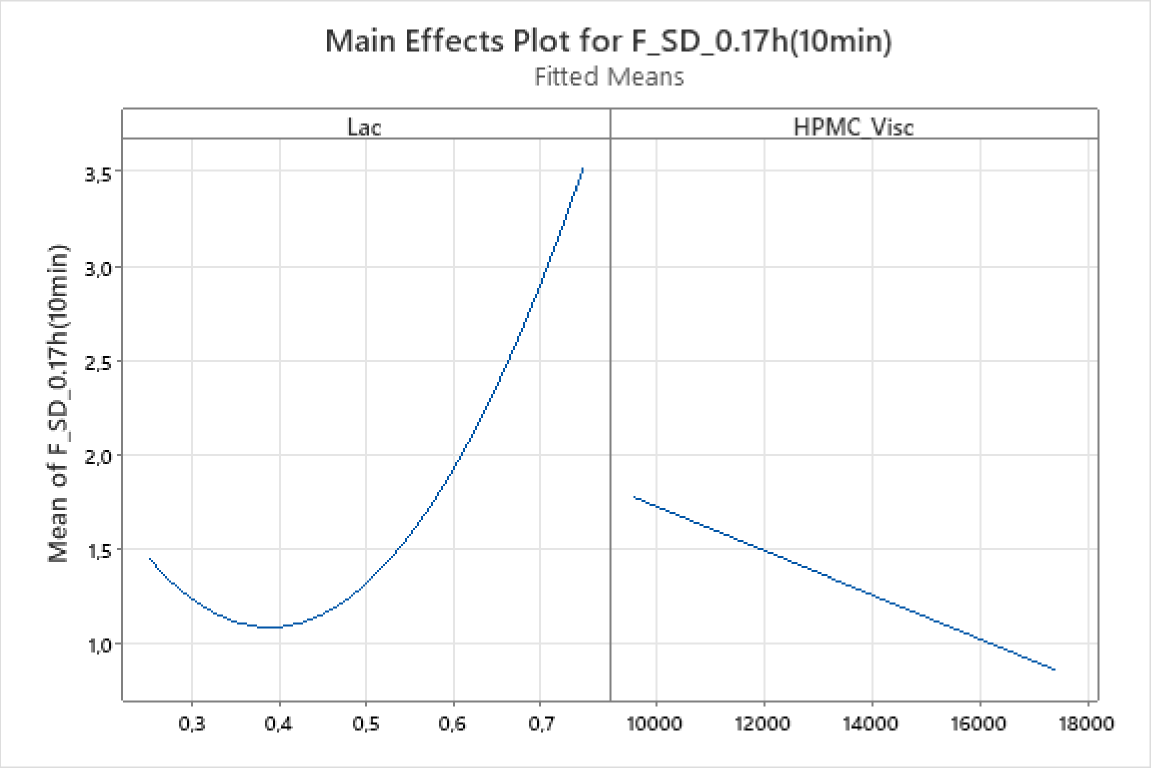

a)

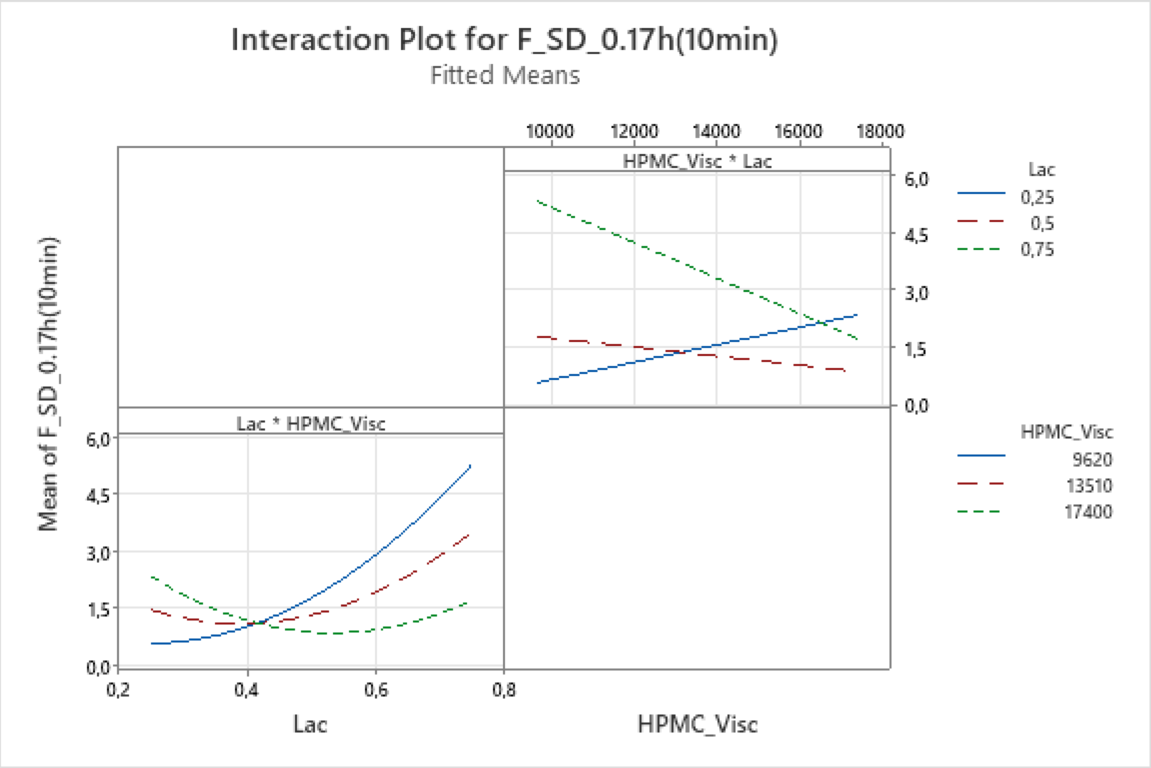

b)

Figure S67. Main effects plot (a) and Interaction plot for SD of carvedilol release using an Optimized MLR model at t = 0.17 h (10 min).

a)

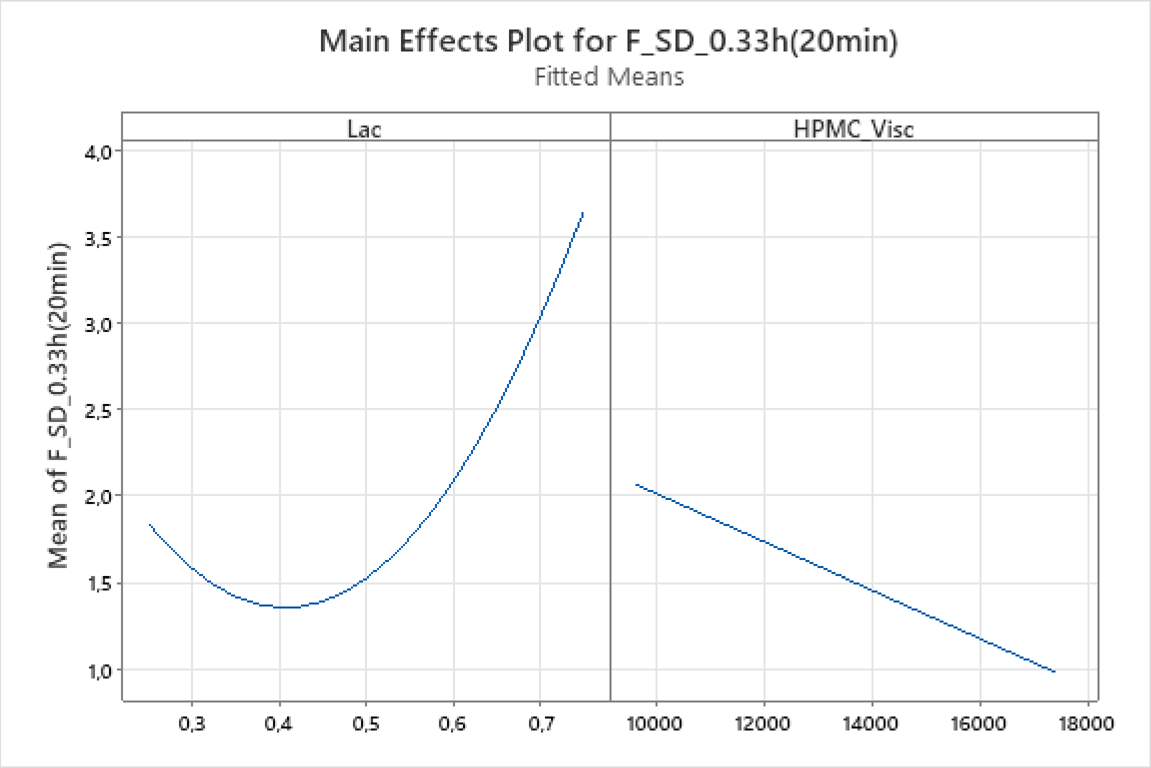

b)

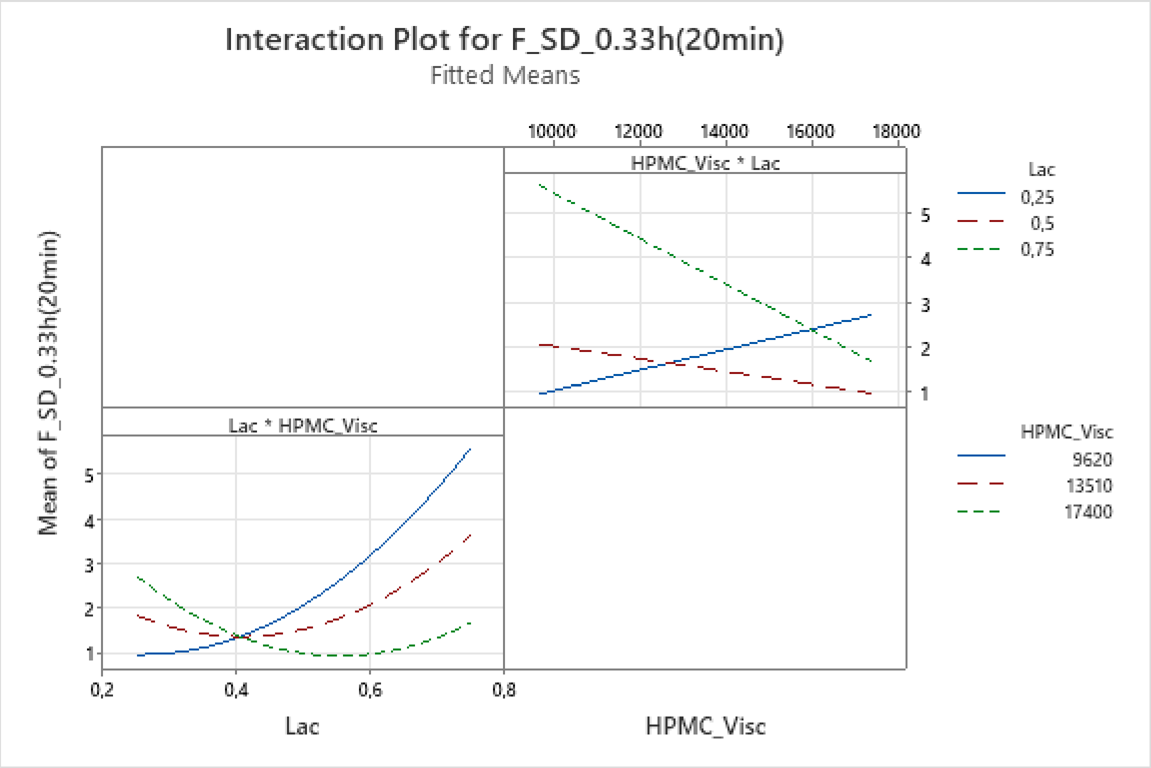

Figure S68. Main effects plot (a) and Interaction plot for SD of carvedilol release using an Optimized MLR model at t = 0.33 h (20 min).

a) 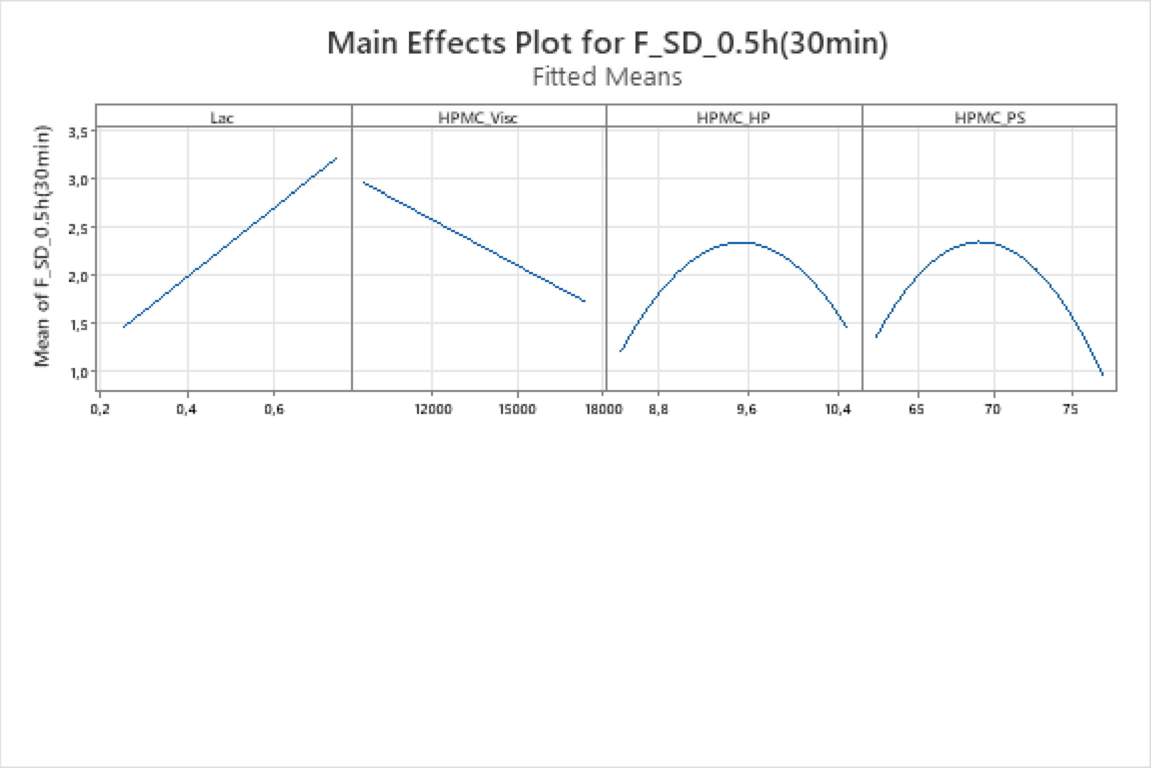

b) 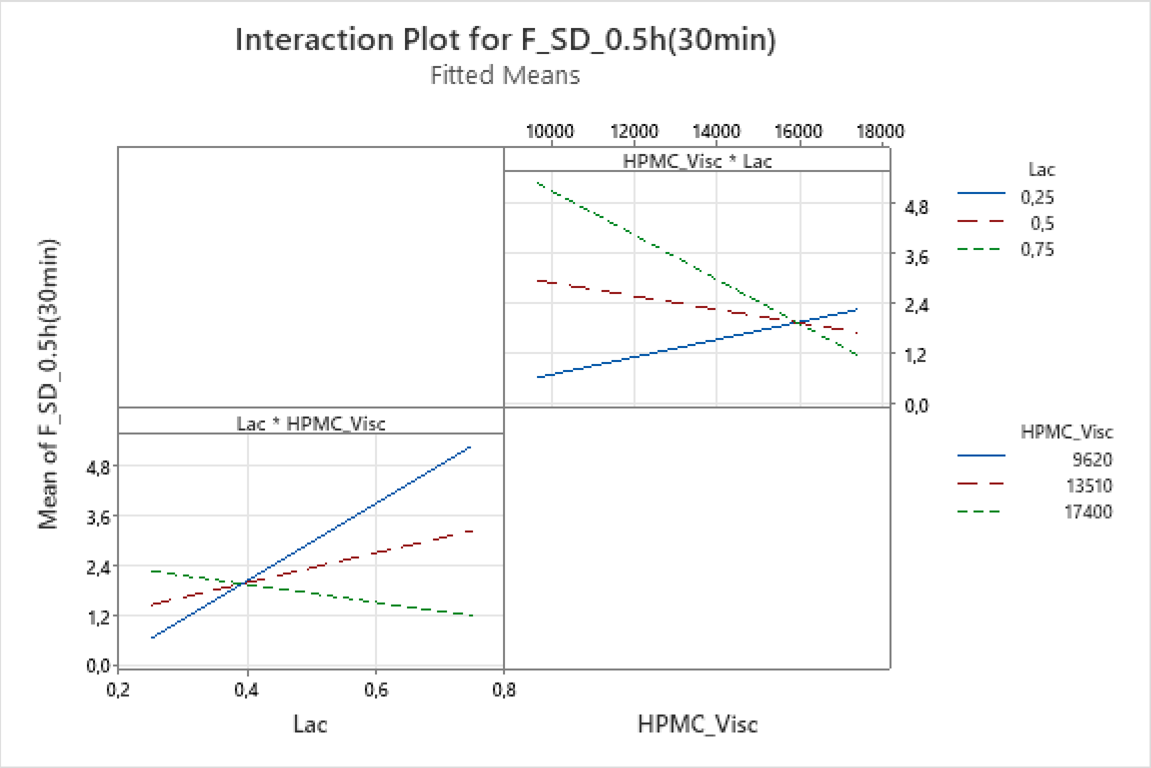

Figure S69. Main effects plot (a) and Interaction plot for SD of carvedilol release using an Optimized MLR model at t = 0.5 h (30 min).

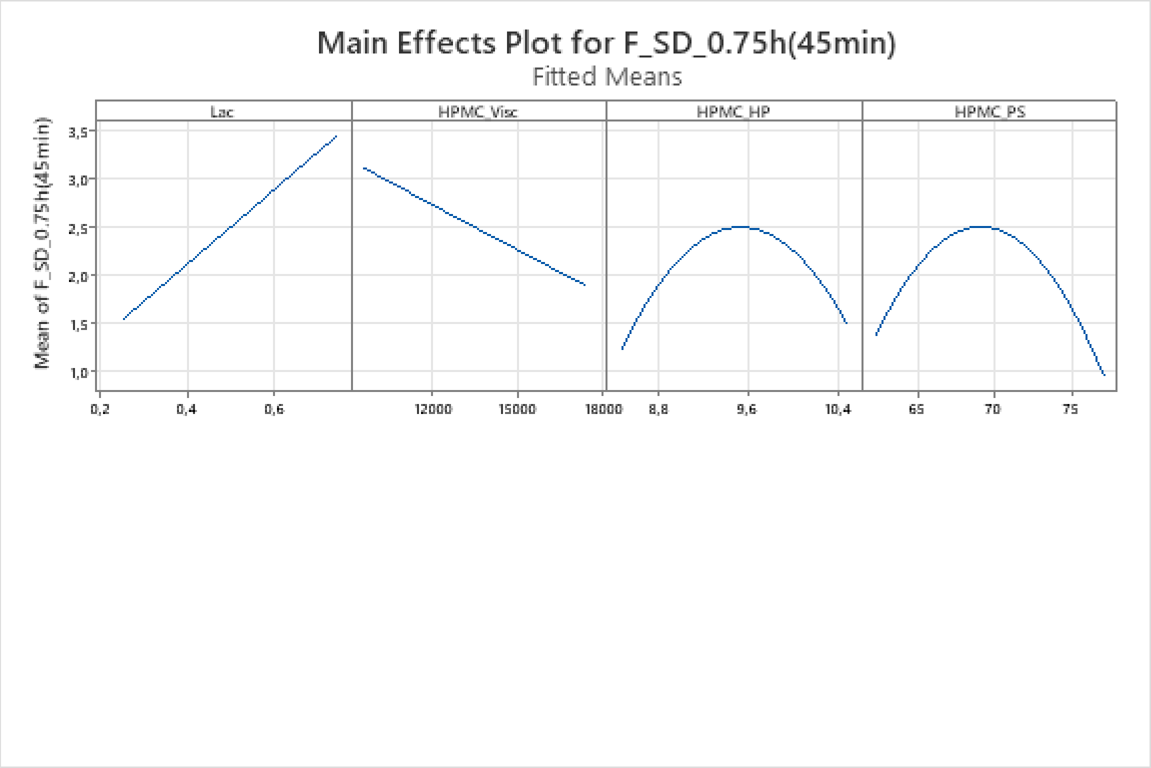

a)

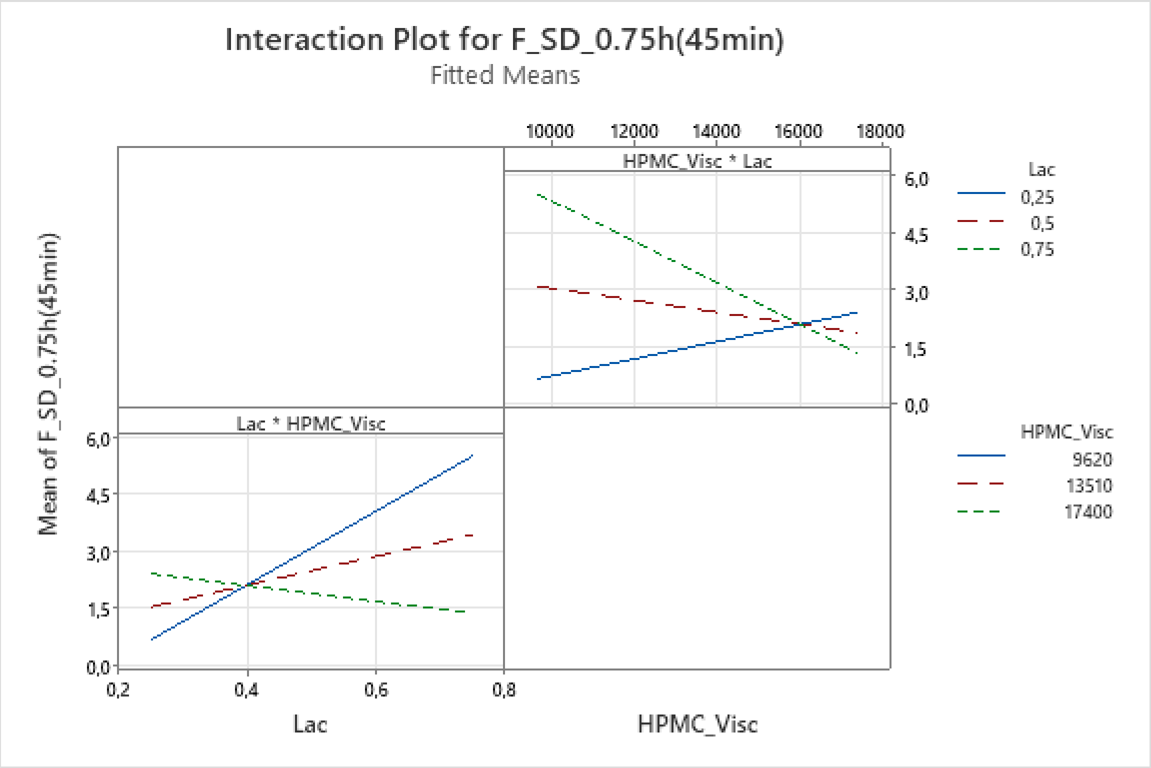

b)

Figure S70. Main effects plot (a) and Interaction plot for SD of carvedilol release using an Optimized MLR model at t = 0.75 h (45 min).

a)

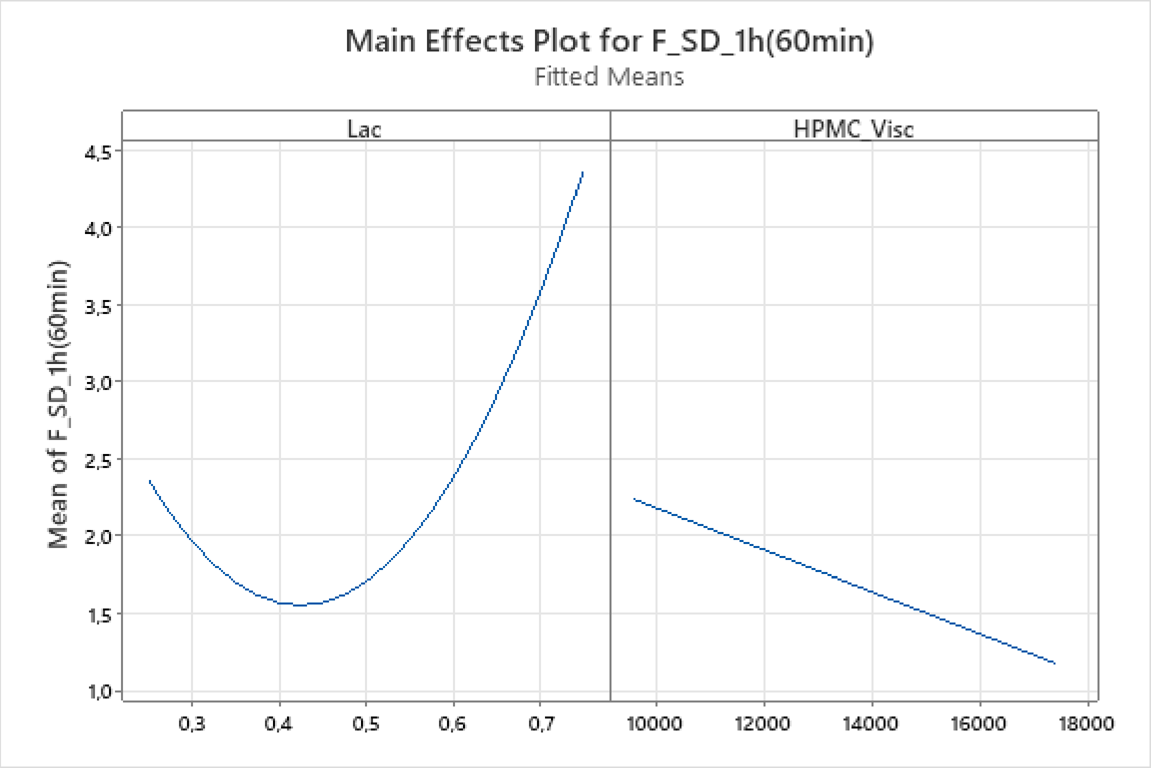

b)

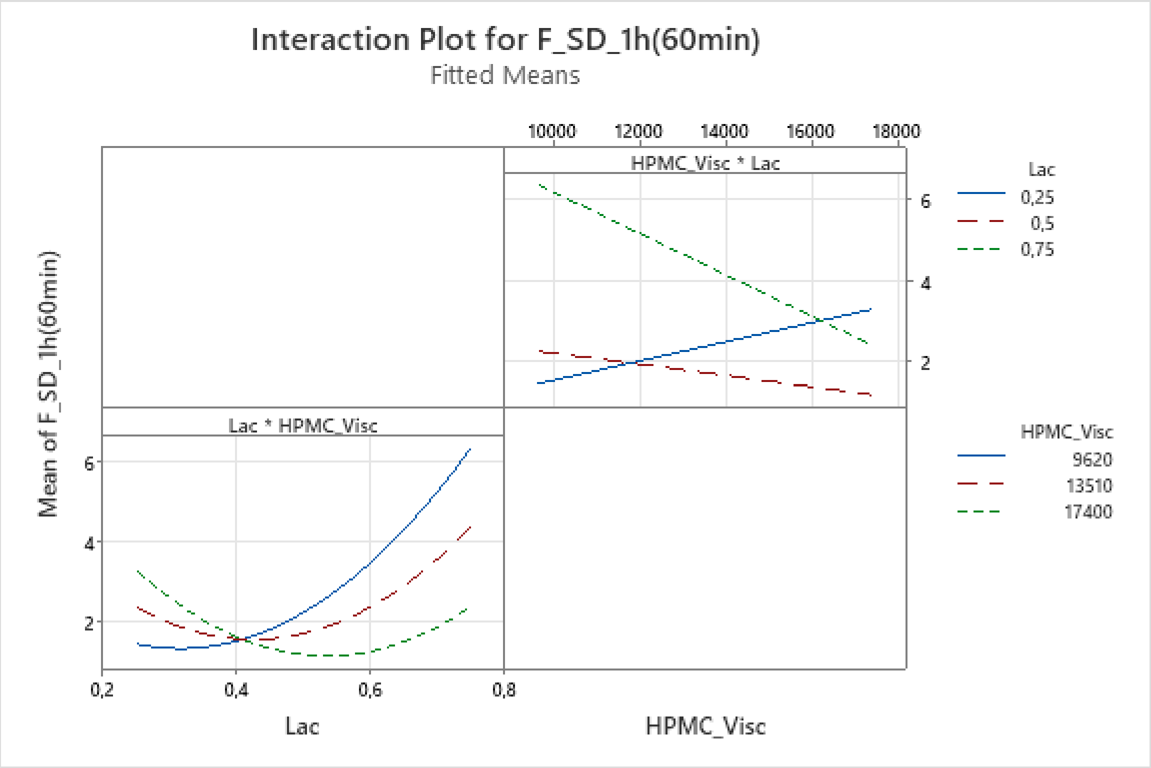

Figure S71. Main effects plot (a) and Interaction plot for SD of carvedilol release using an Optimized MLR model at t = 1 h (60 min).

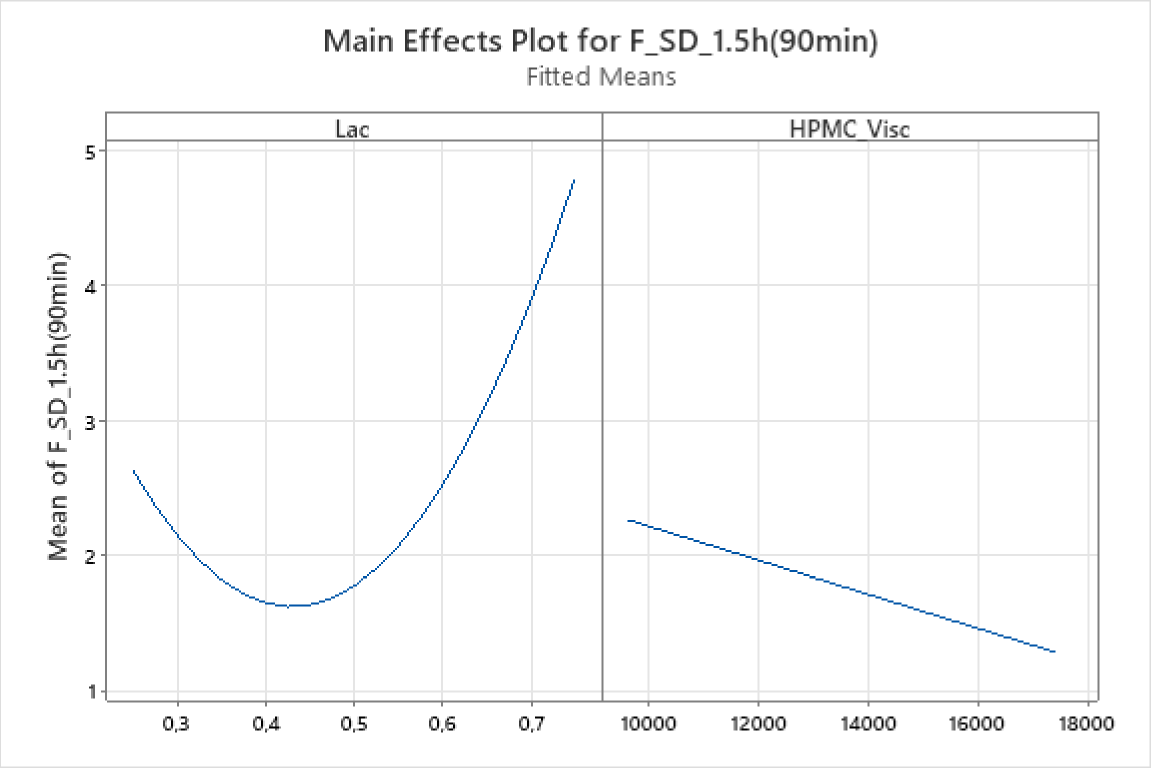

a)

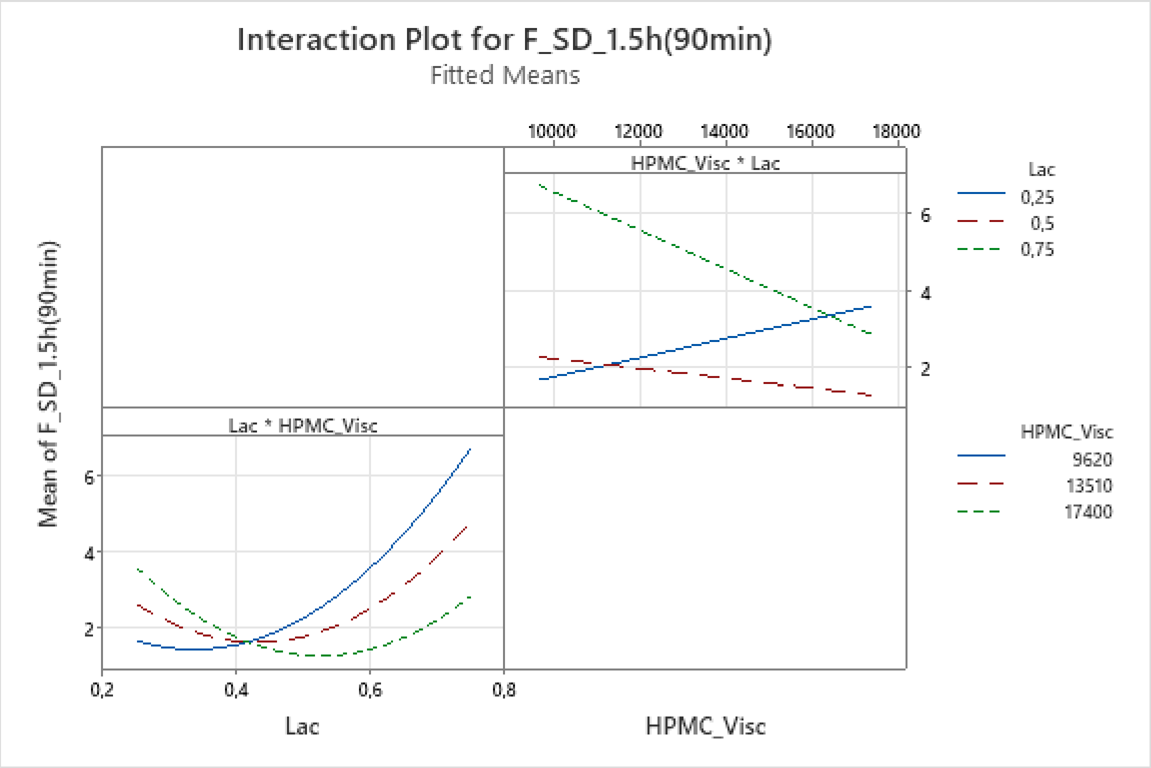

b)

Figure S72. Main effects plot (a) and Interaction plot for SD of carvedilol release using an Optimized MLR model at t = 1.5 h (90 min).

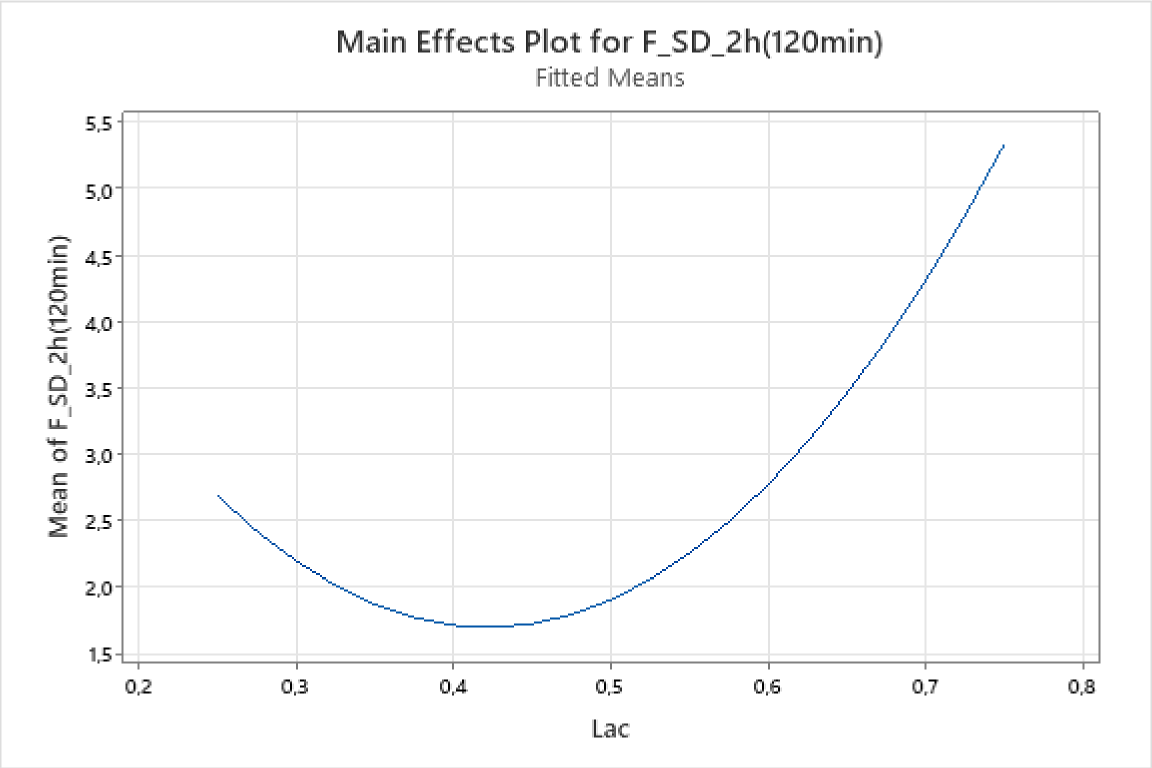

Figure S73. Main effects plot for SD of carvedilol release using an Optimized MLR model at t = 2 h (120 min).

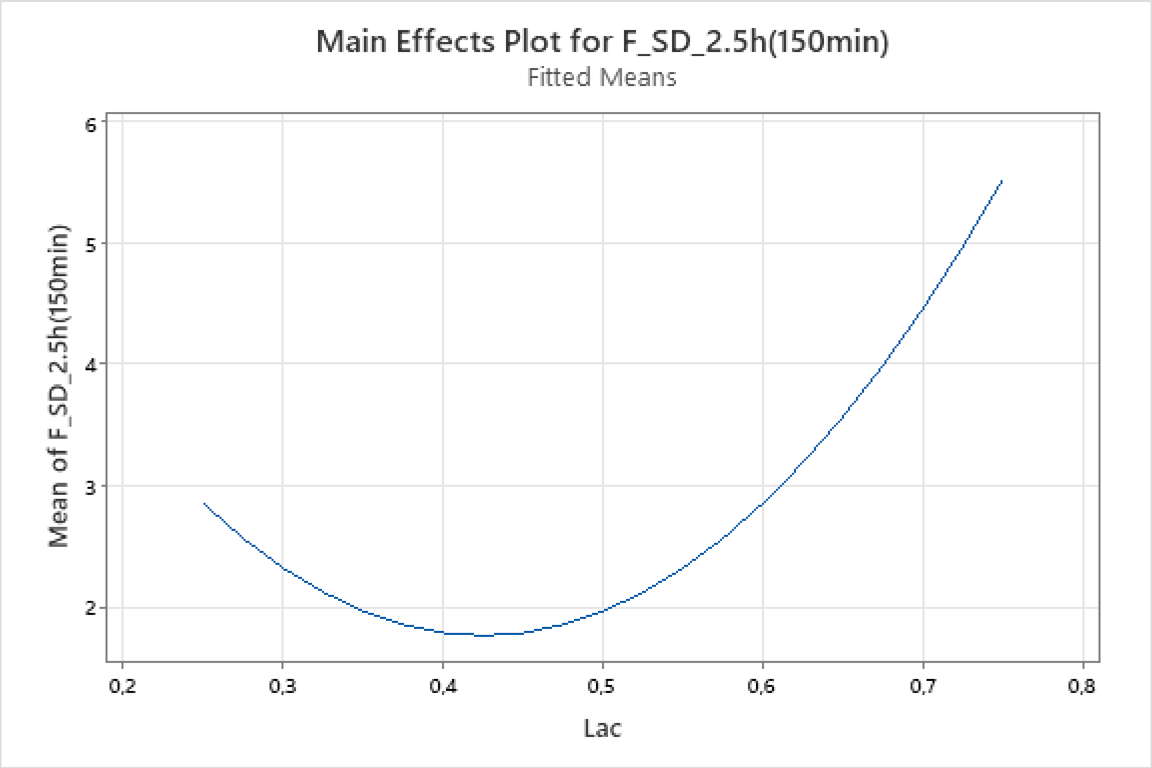

Figure S74. Main effects plot for SD of carvedilol release using an Optimized MLR model at t = 2.5 h (150 min).

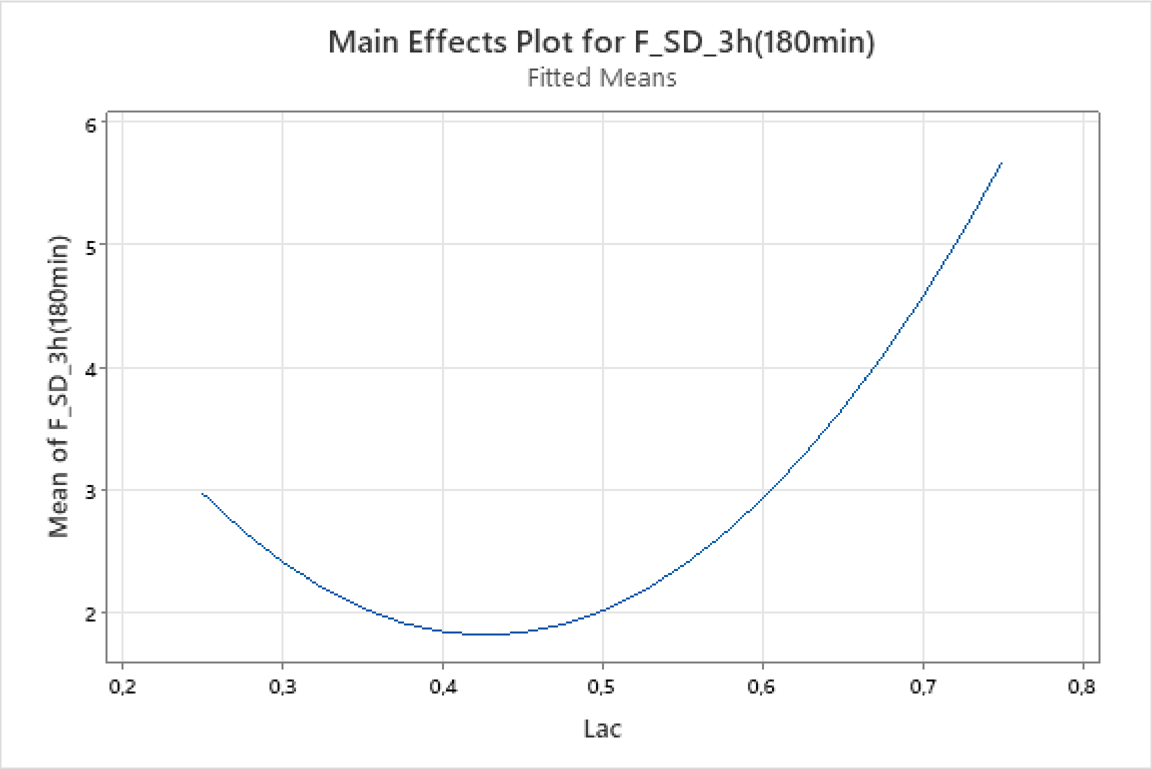

Figure S75. Main effects plot for SD of carvedilol release using an Optimized MLR model at t = 3 h (180 min).

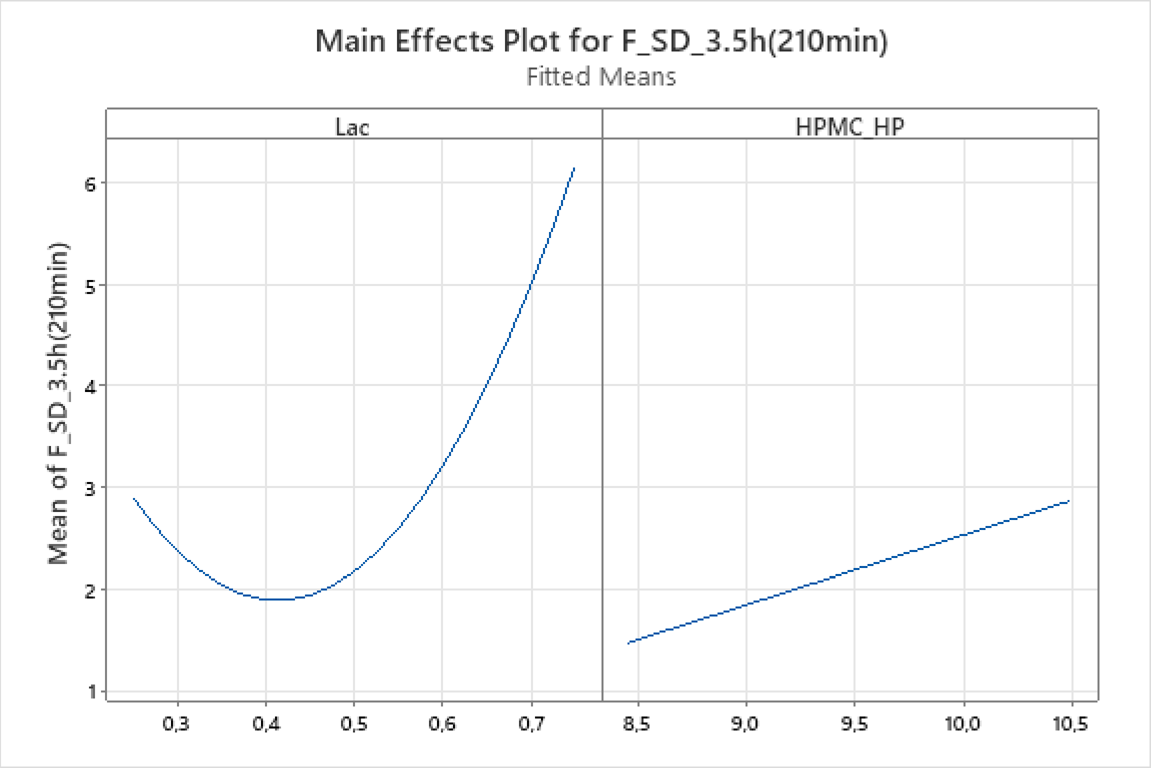

a)

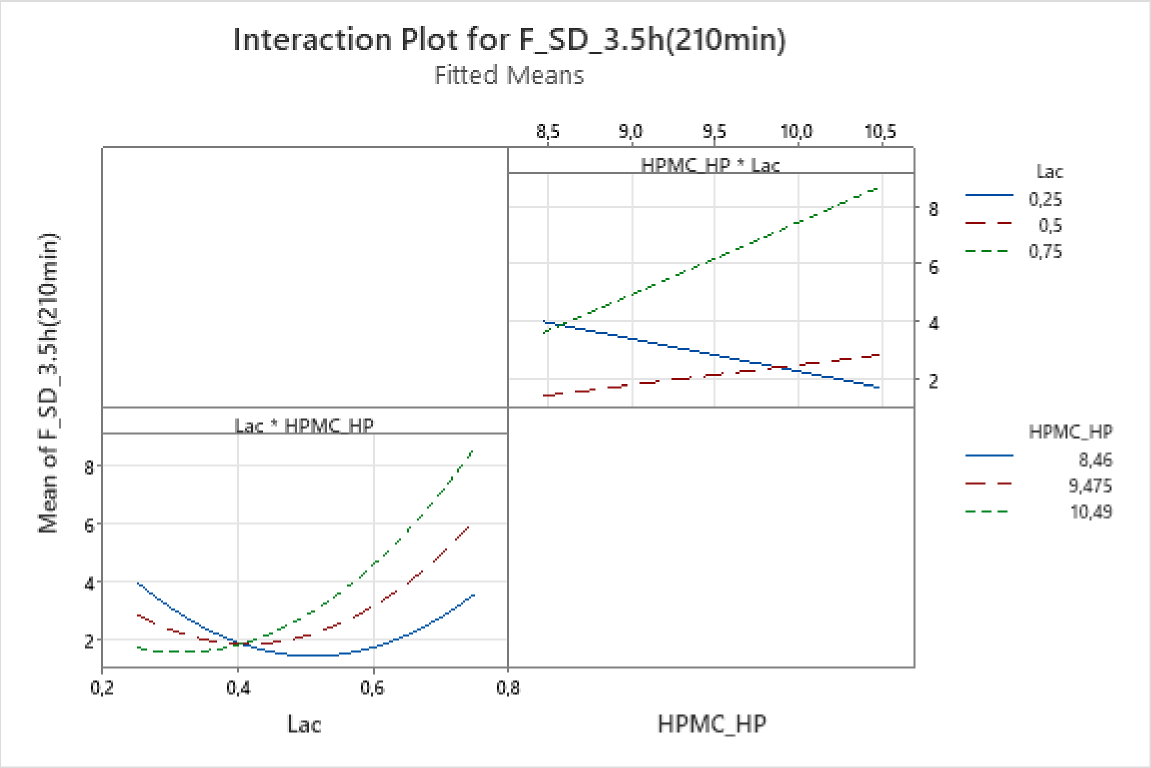

b)

Figure S76. Main effects plot (a) and Interaction plot for SD of carvedilol release using an Optimized MLR model at t = 3.5 h (210 min).

a)

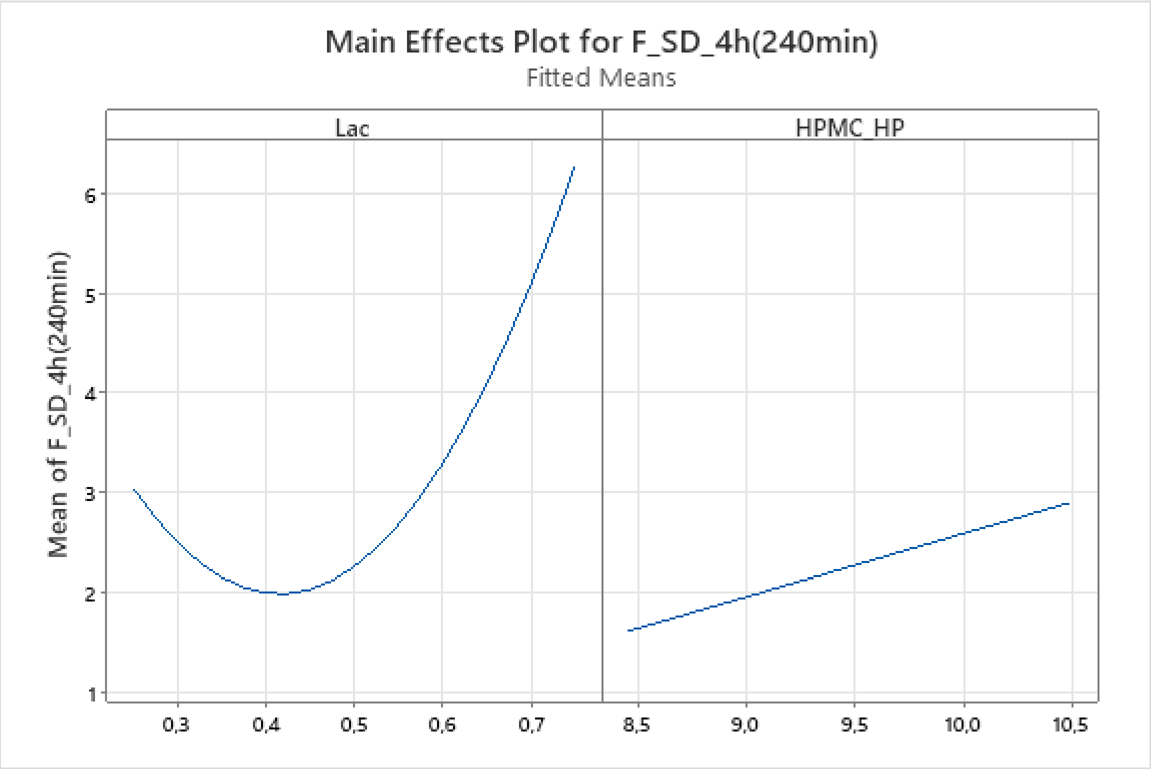

b)

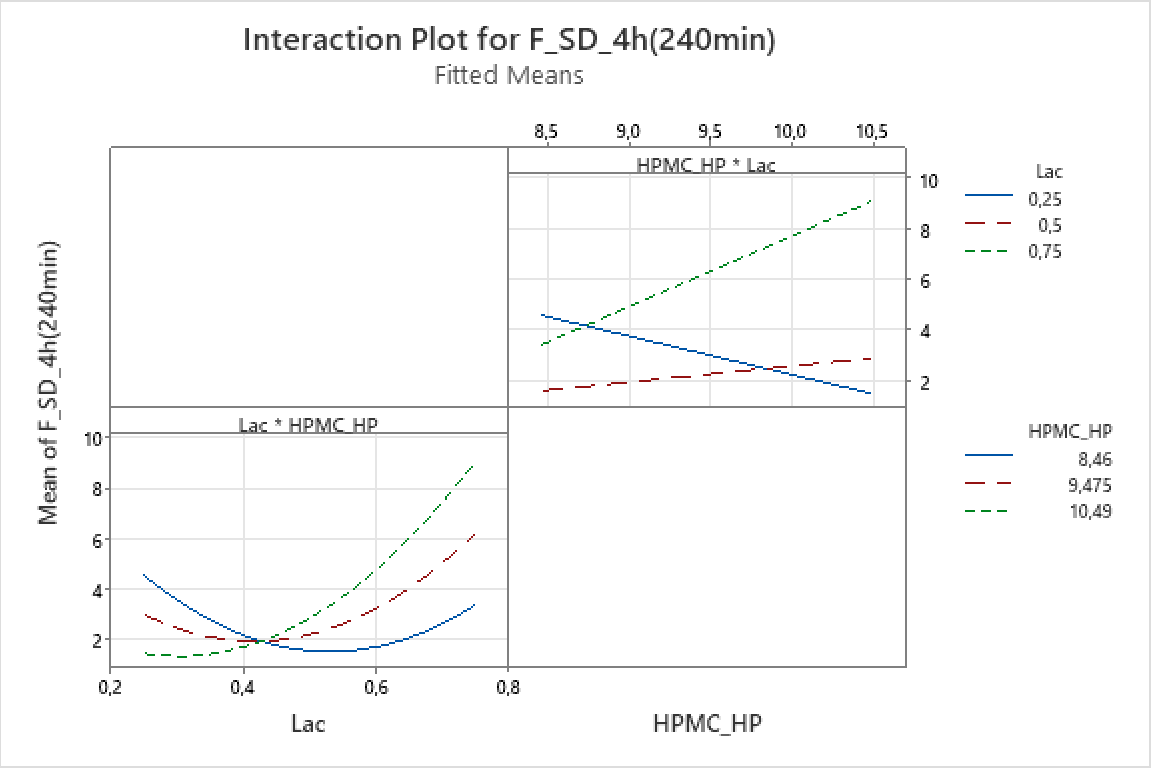

Figure S77. Main effects plot (a) and Interaction plot for SD of carvedilol release using an Optimized MLR model at t = 4 h (240 min).

a)

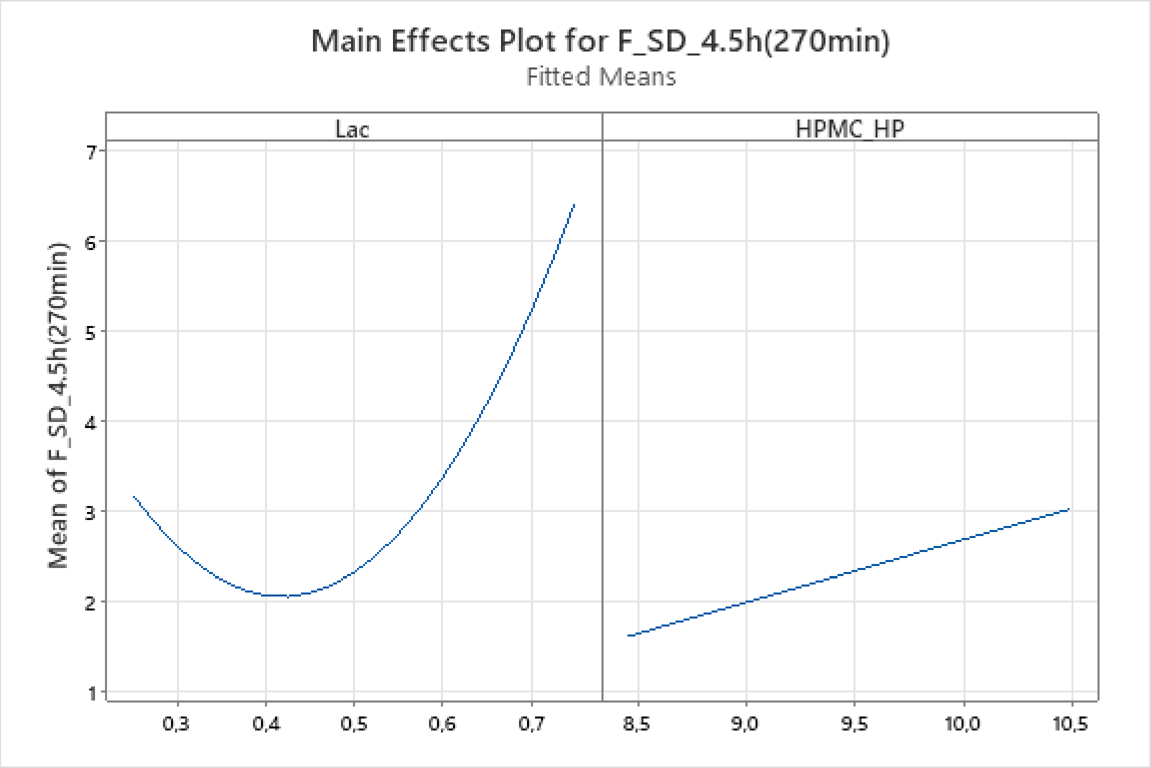

b)

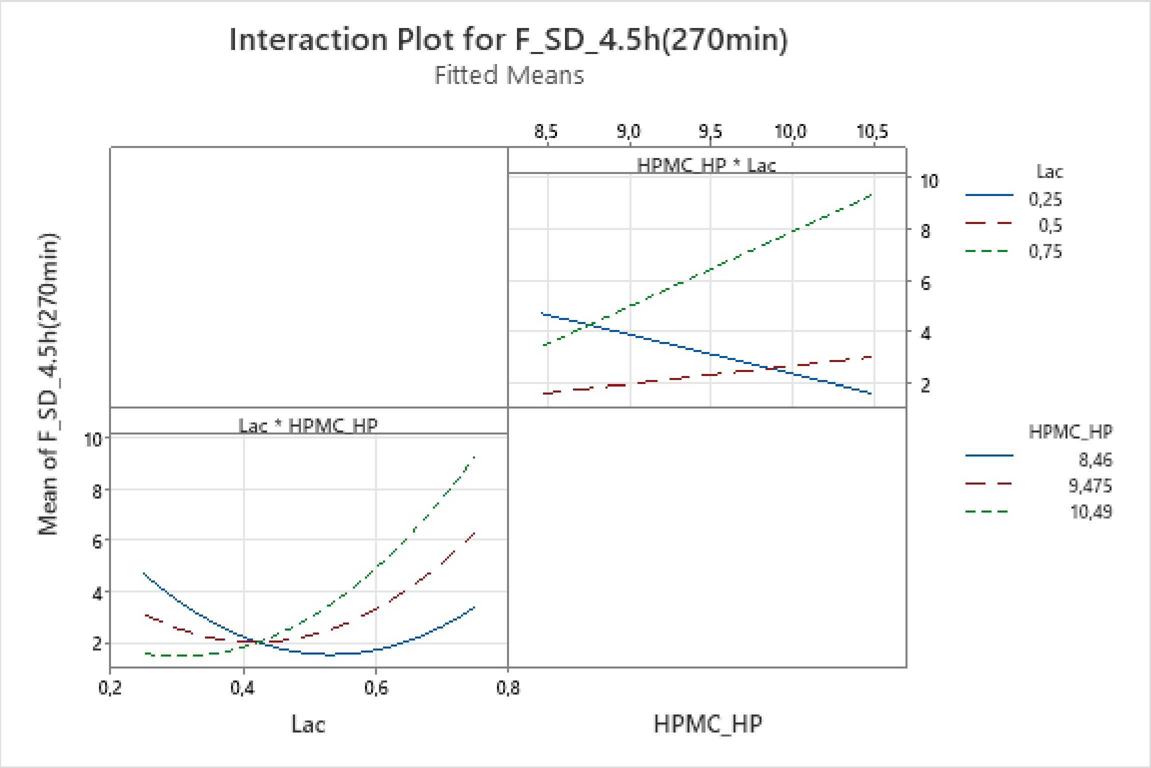

Figure S78. Main effects plot (a) and Interaction plot for SD of carvedilol release using an Optimized MLR model at t = 4.5 h (270 min).

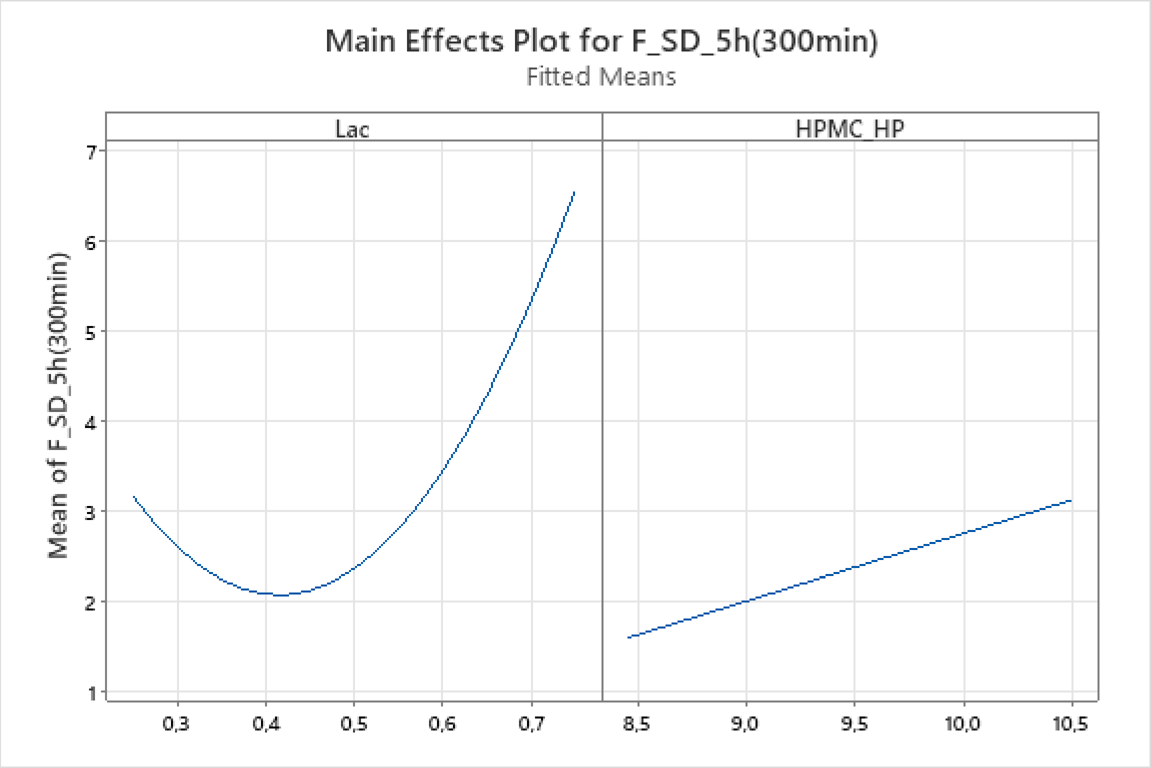

a)

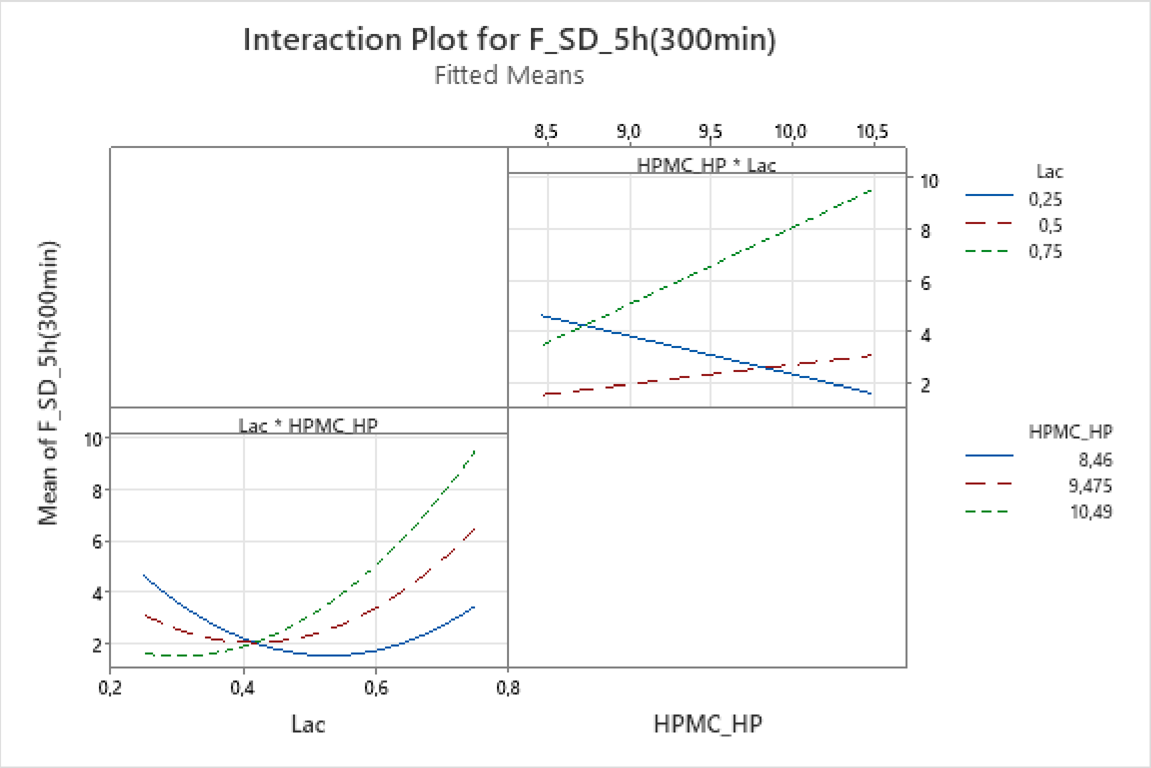

b)

Figure S79. Main effects plot (a) and Interaction plot for SD of carvedilol release using an Optimized MLR model at t = 5 h (300 min).

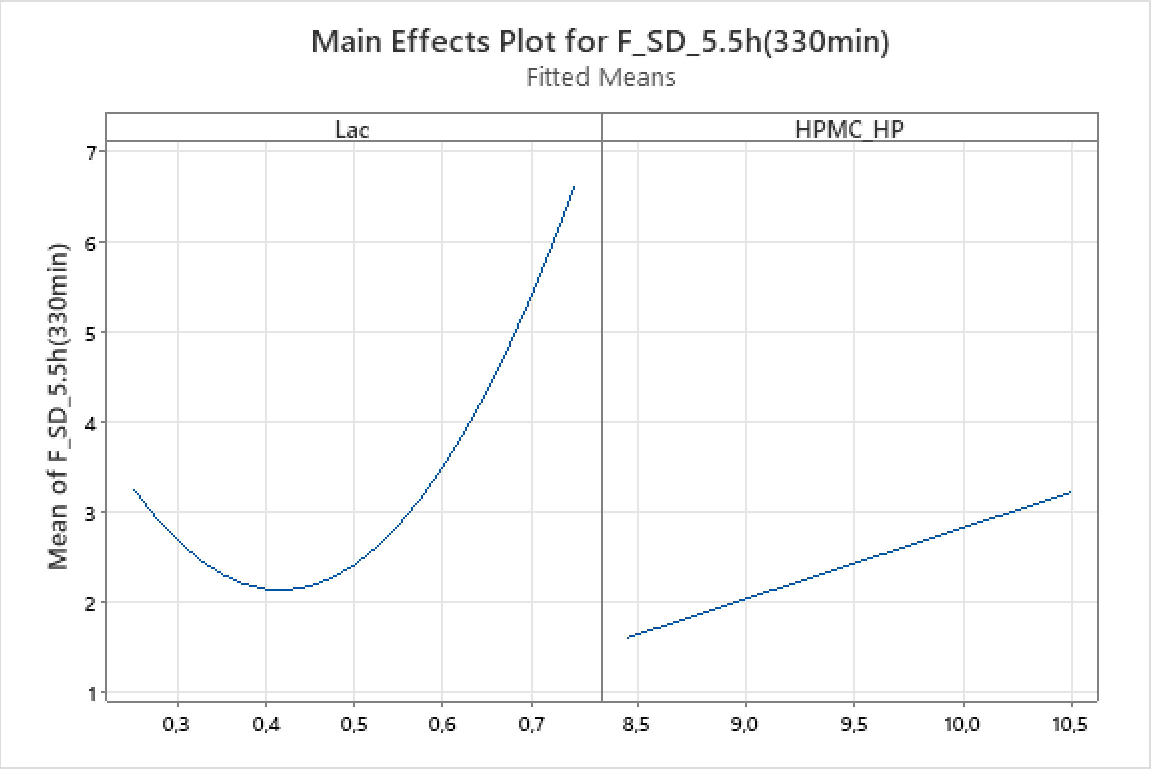

a)

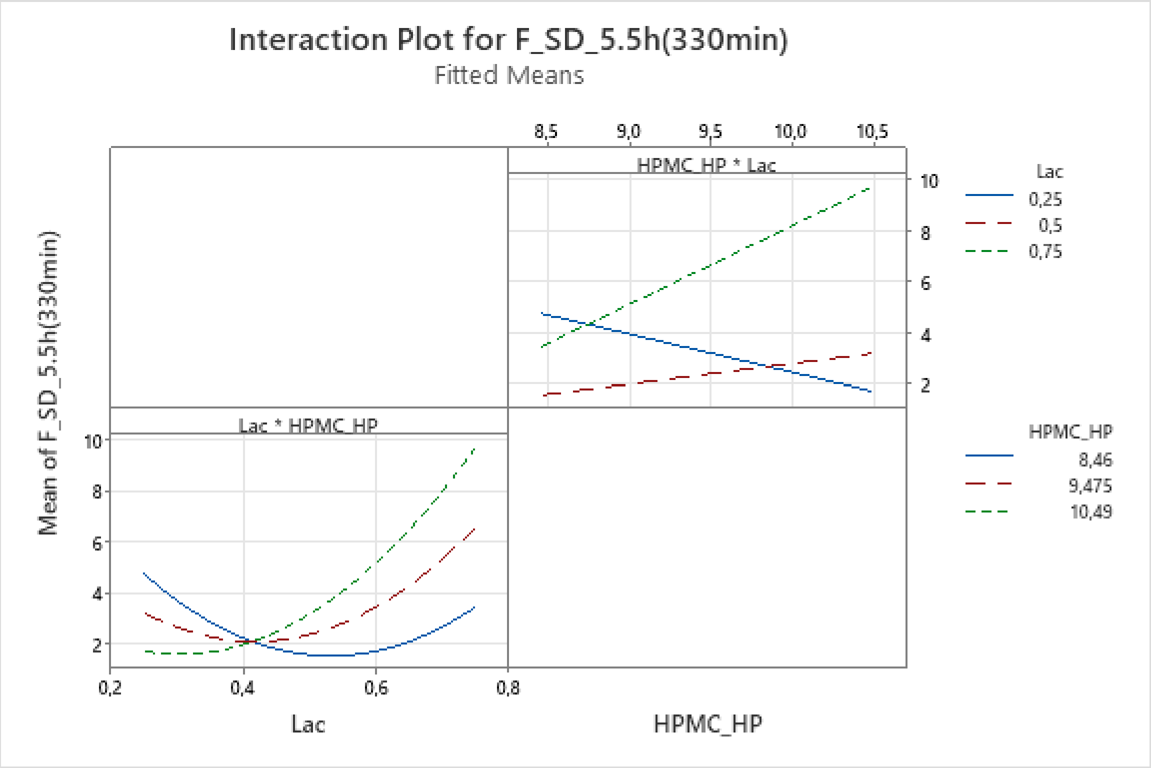

b)

Figure S80. Main effects plot (a) and Interaction plot for SD of carvedilol release using an Optimized MLR model at t = 5.5 h (330 min).

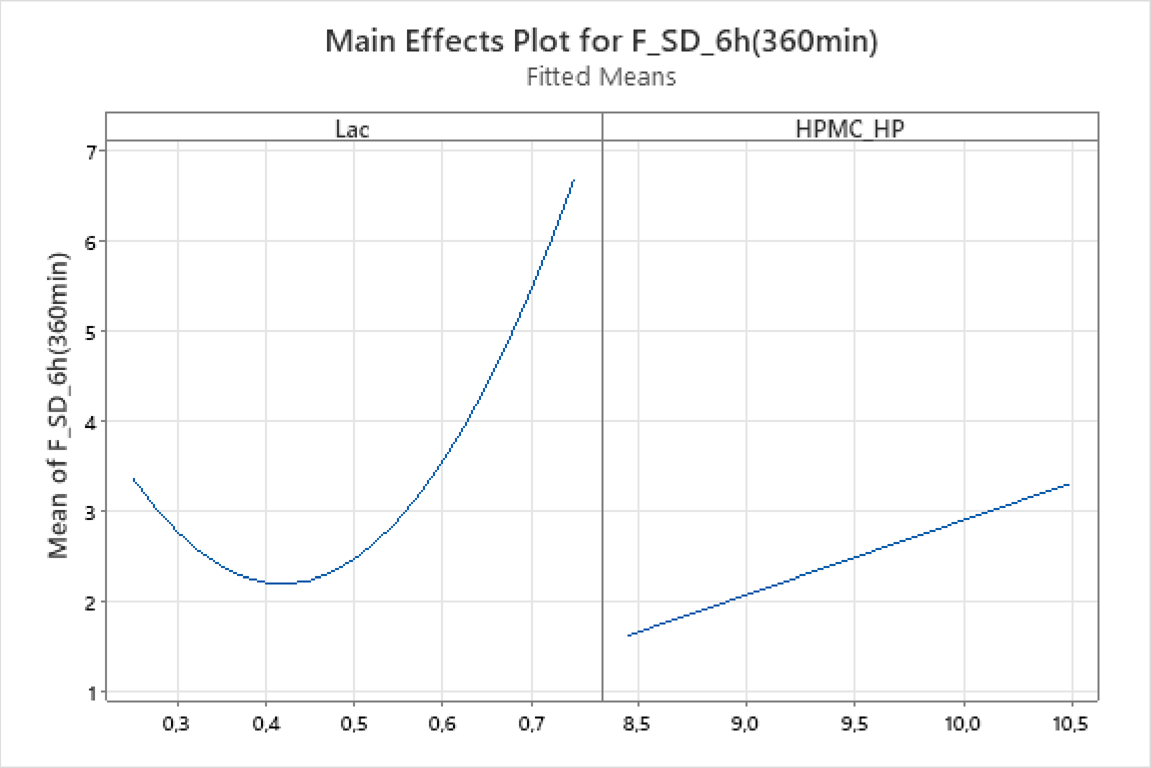

a)

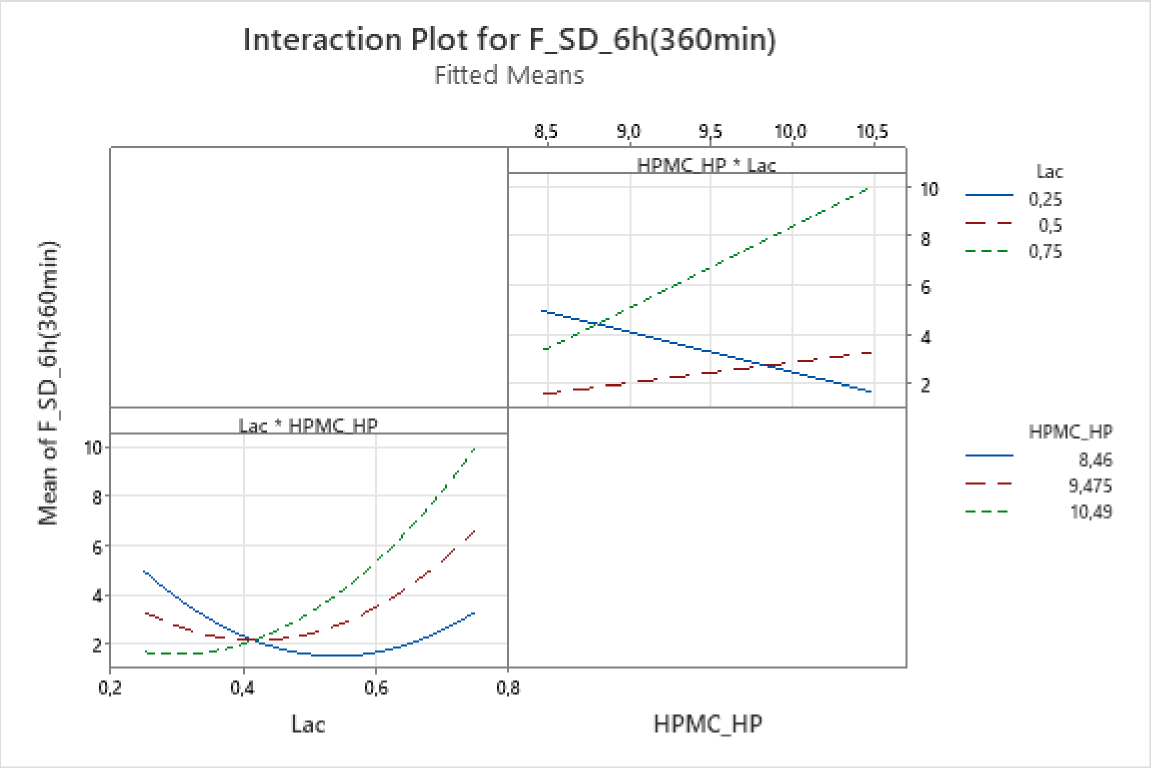

b)

Figure S81. Main effects plot (a) and Interaction plot for SD of carvedilol release using an Optimized MLR model at t = 6 h (360 min).

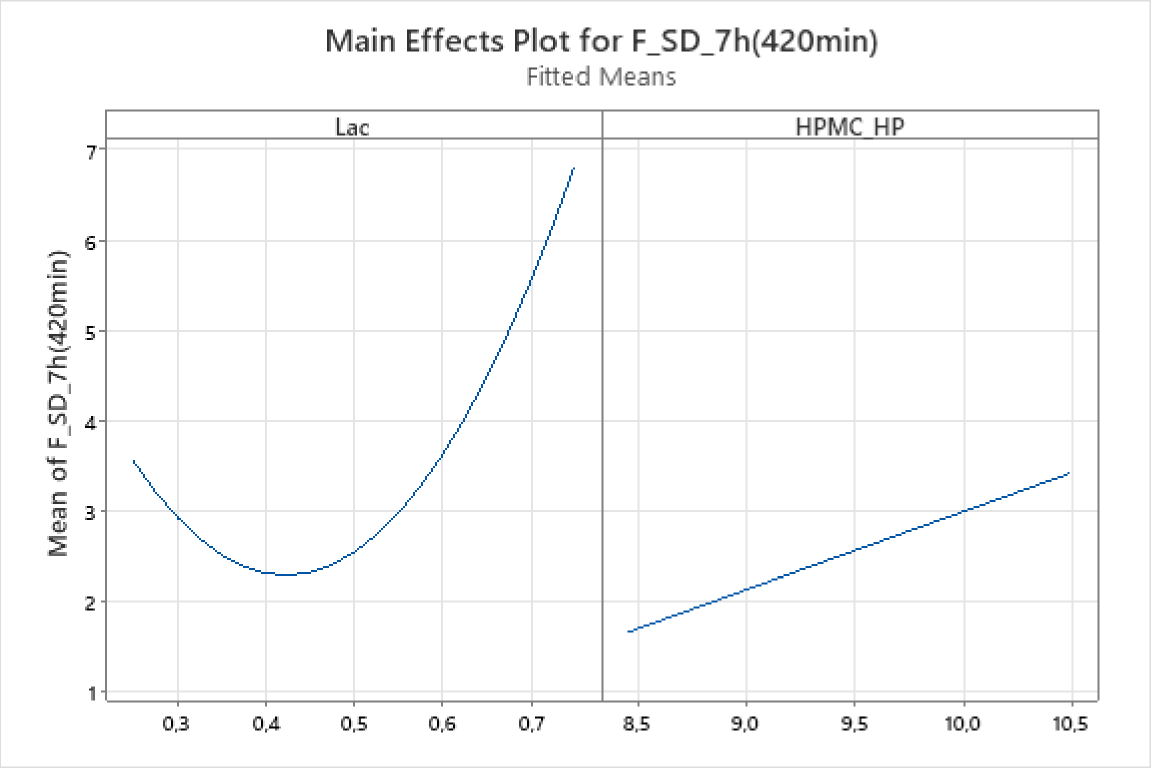

a)

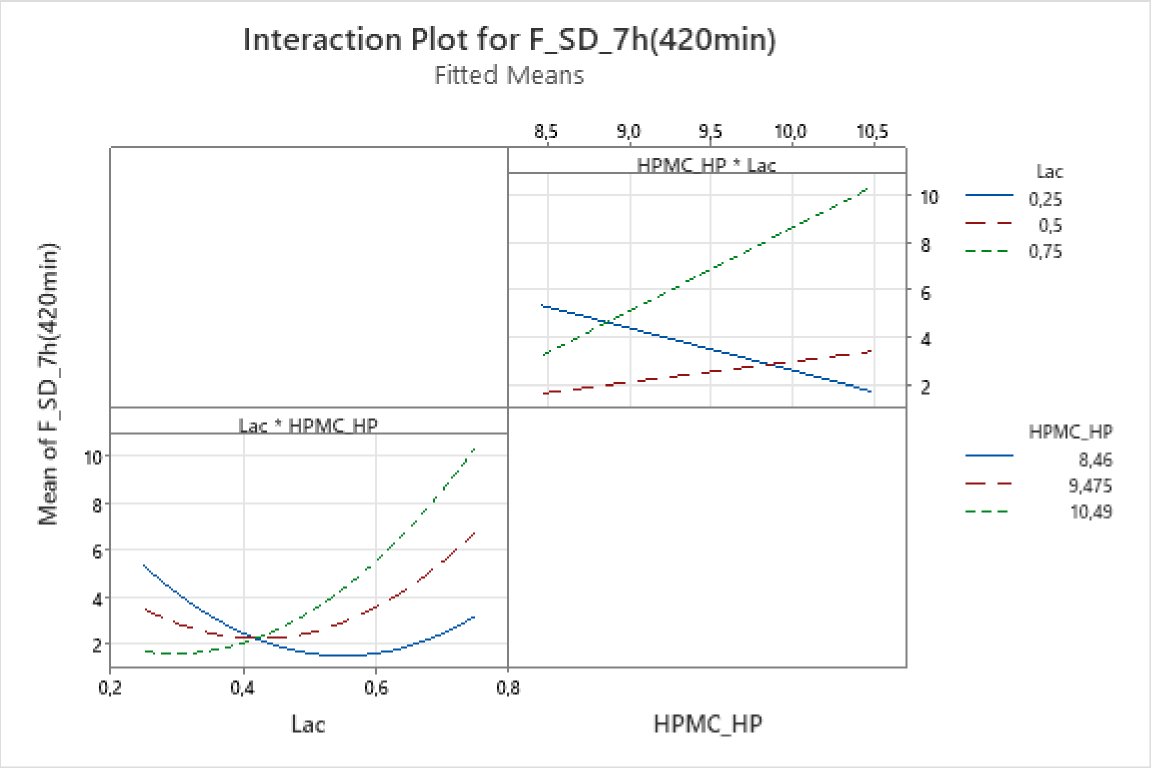

b)

Figure S82. Main effects plot (a) and Interaction plot for SD of carvedilol release using an Optimized MLR model at t = 7 h (420 min).

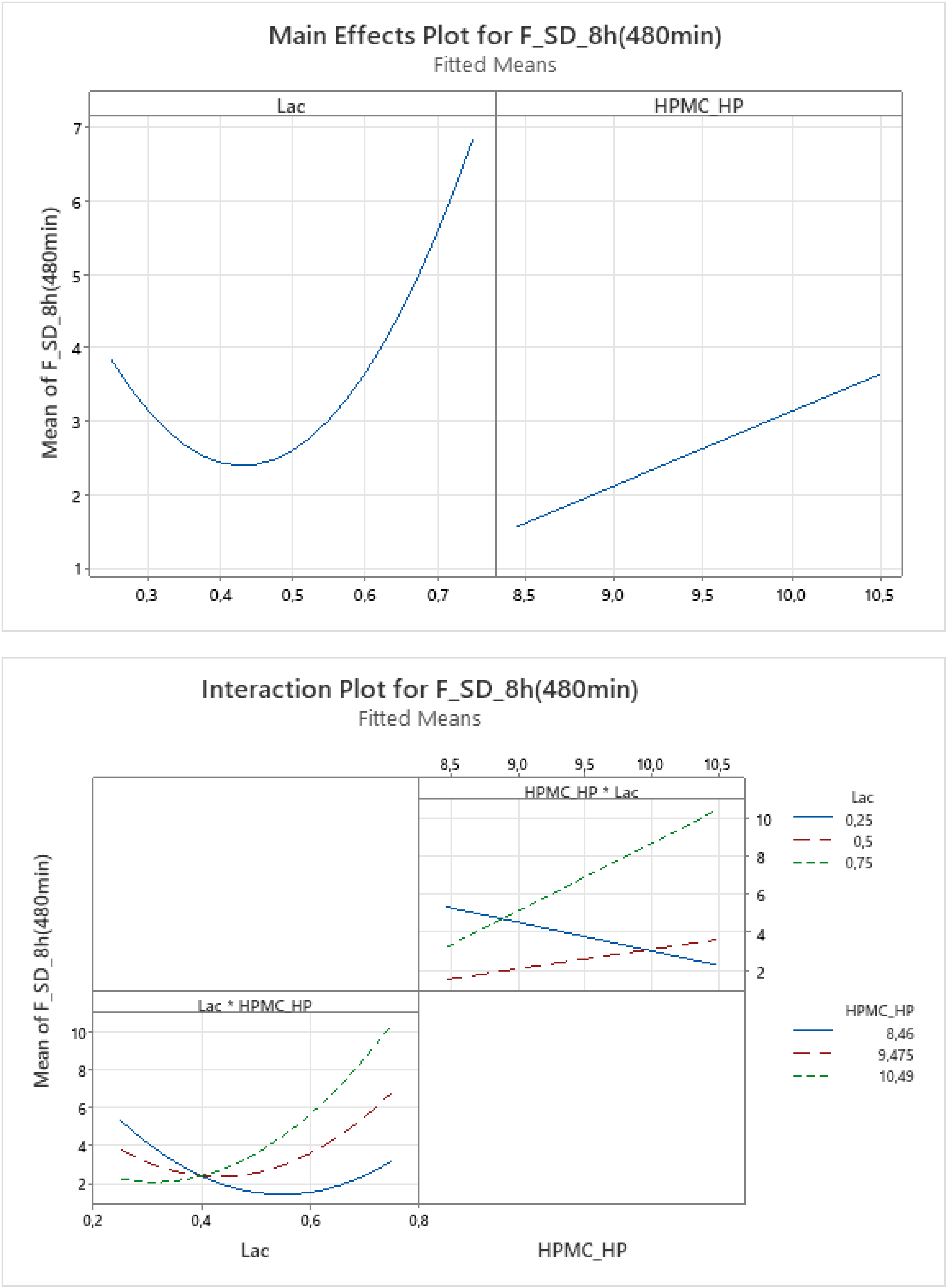

a)

b)

Figure S83. Main effects plot (a) and Interaction plot for SD of carvedilol release using an Optimized MLR model at t = 8 h (480 min).

a)

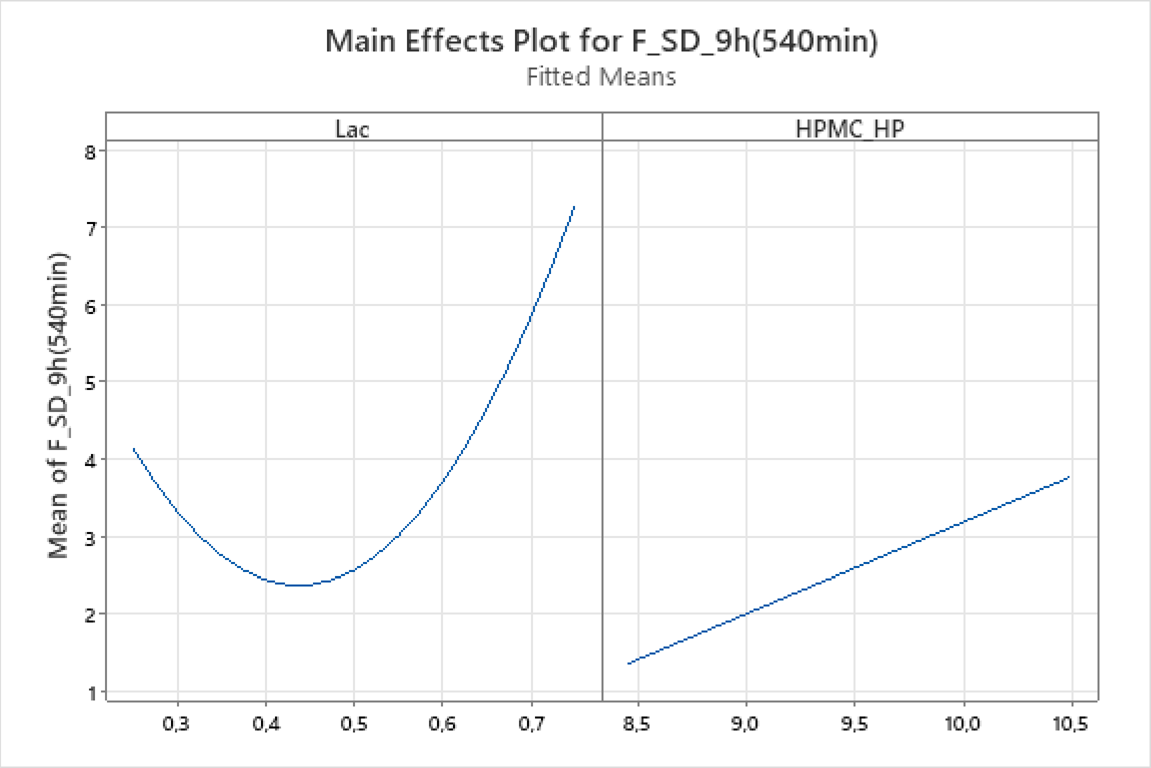

b)

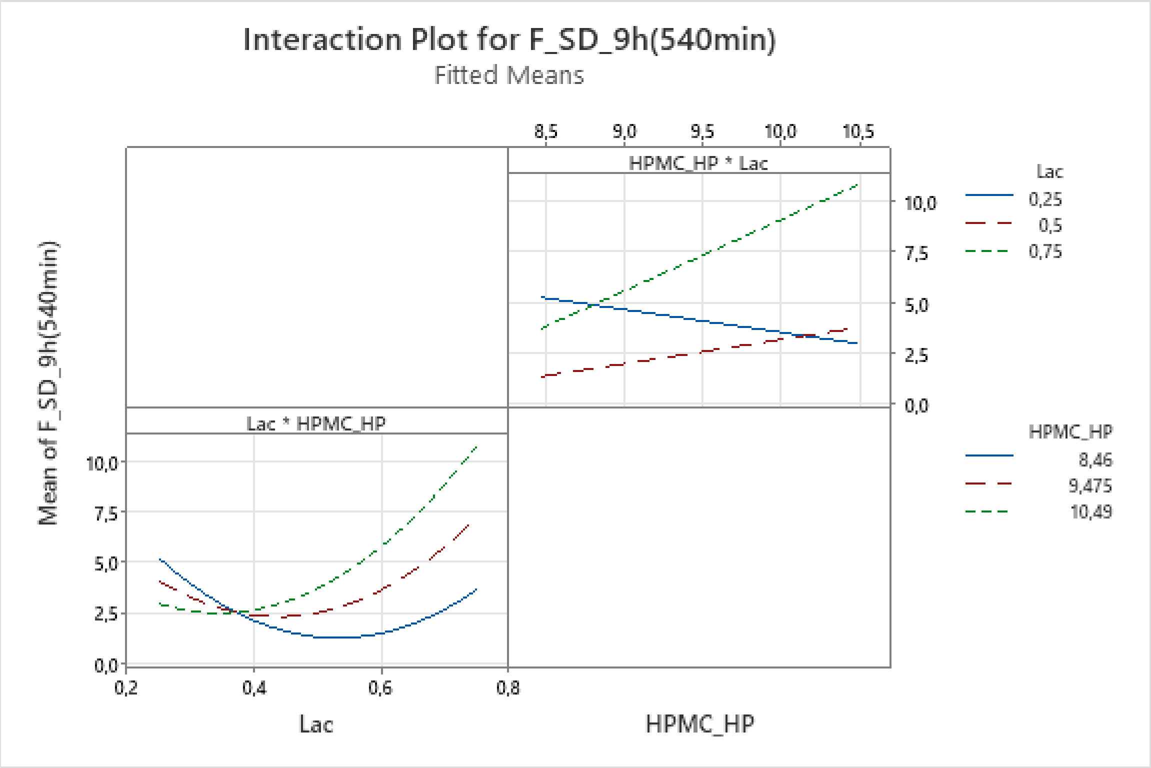

Figure S84. Main effects plot (a) and Interaction plot for SD of carvedilol release using an Optimized MLR model at t = 9 h (540 min).

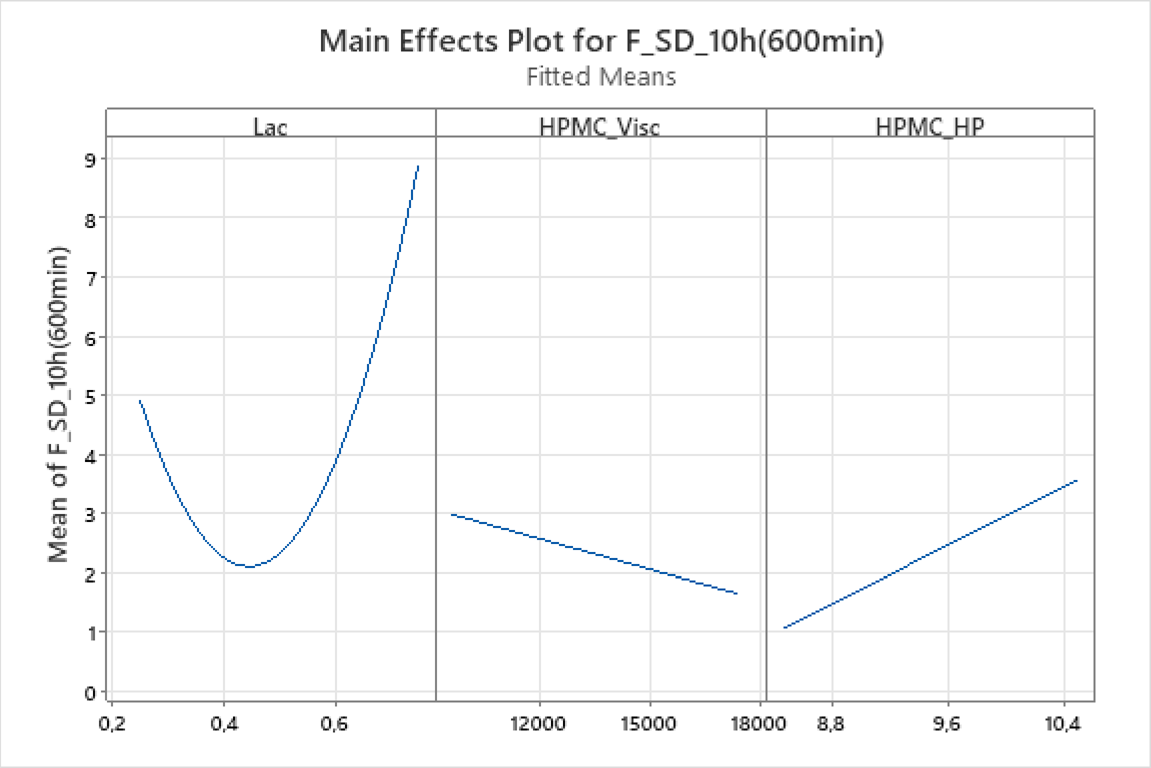

a)

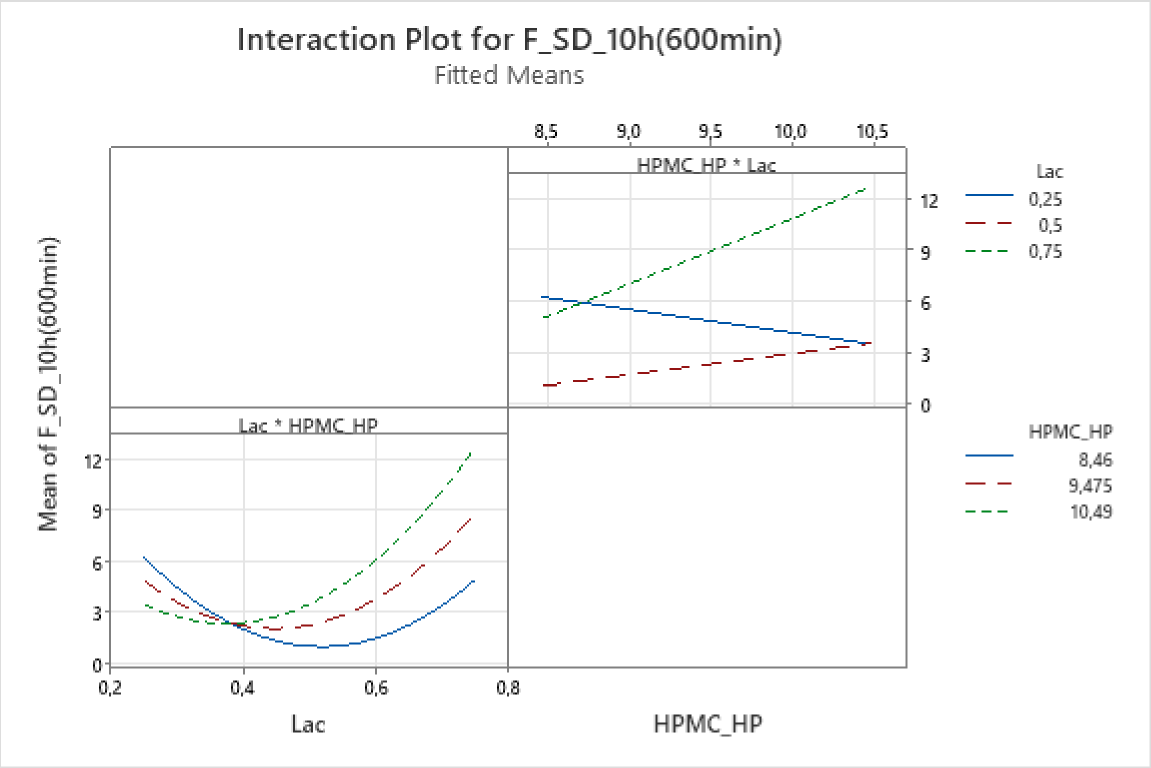

b)

Figure S85. Main effects plot (a) and Interaction plot for SD of carvedilol release using an Optimized MLR model at t = 10 h (600 min).

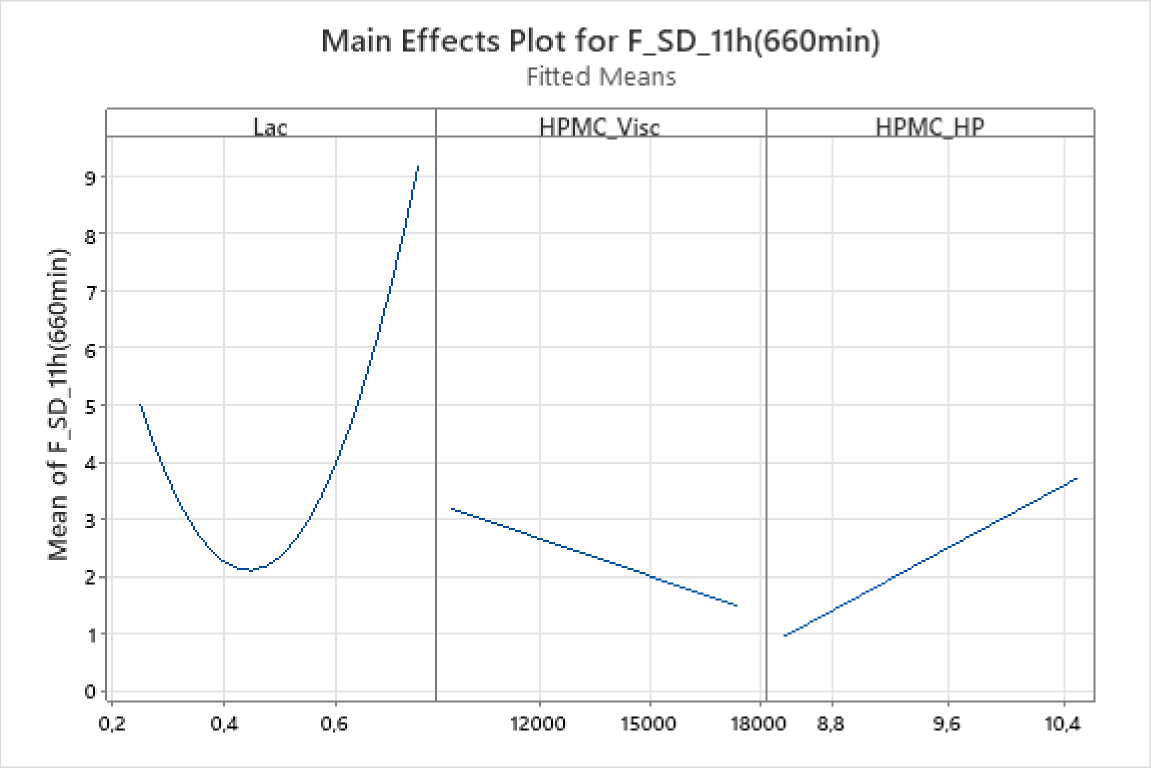

a)

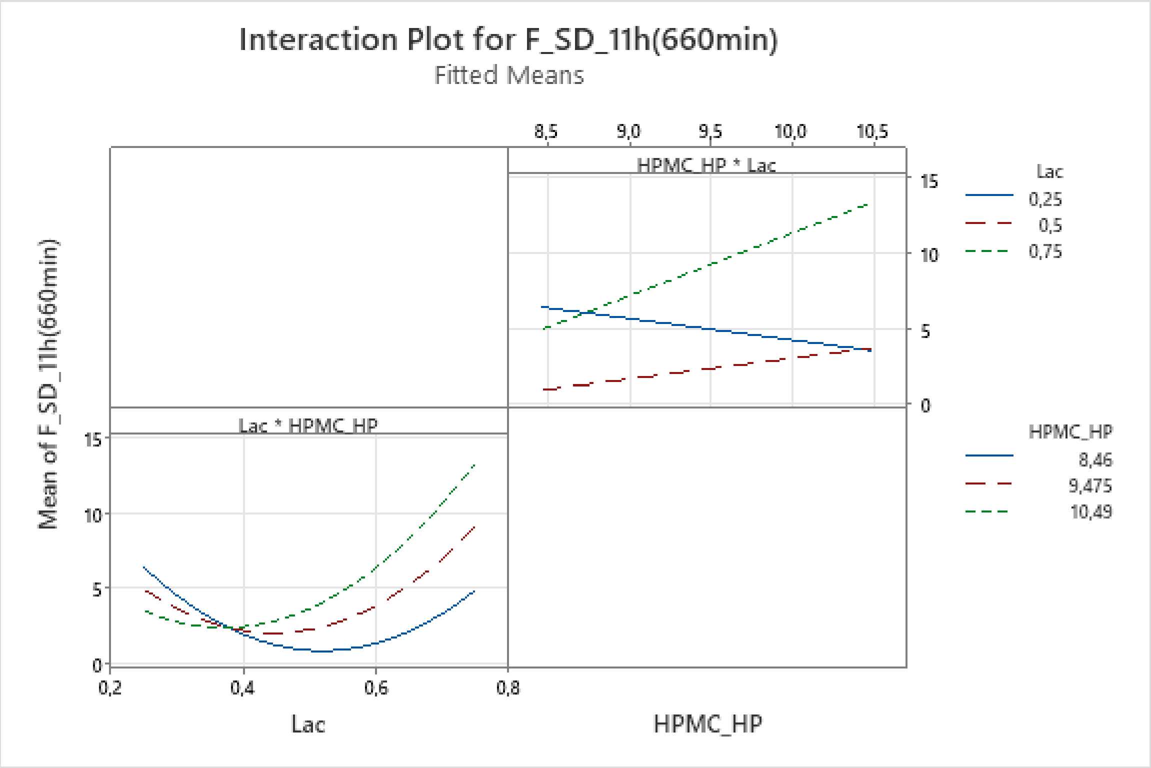

b)

Figure S86. Main effects plot (a) and Interaction plot for SD of carvedilol release using an Optimized MLR model at t = 11 h (660 min).

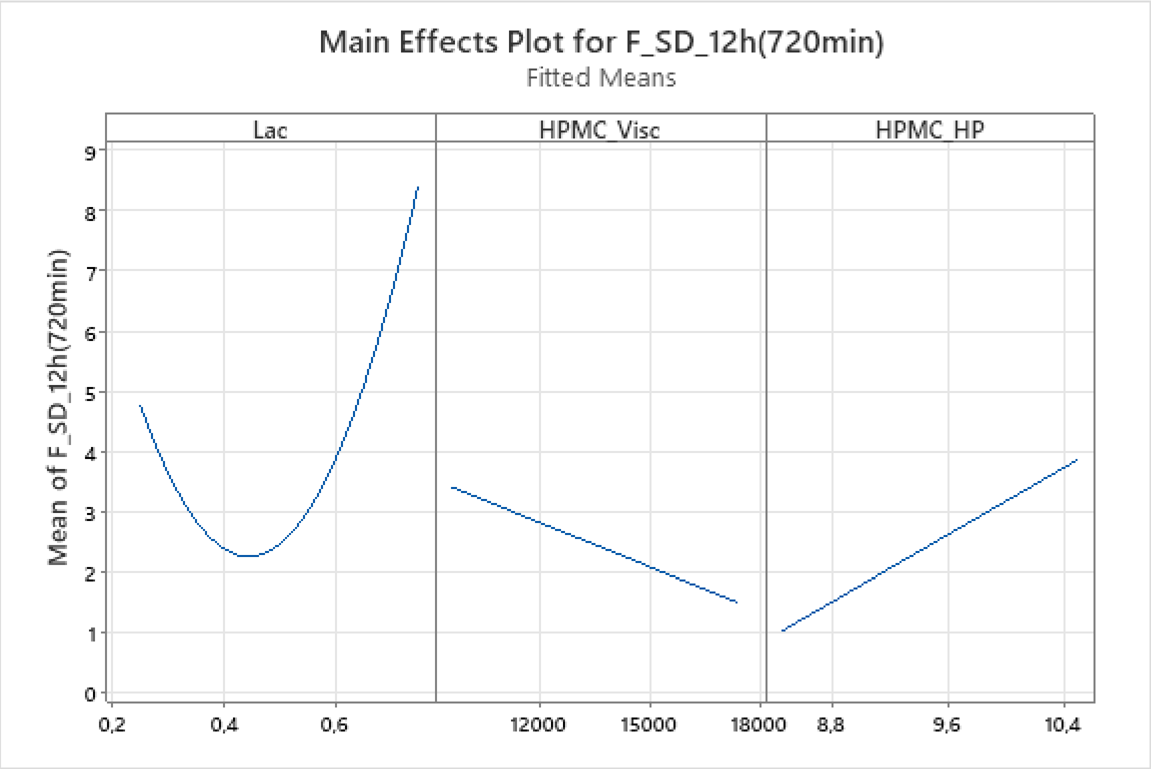

a)

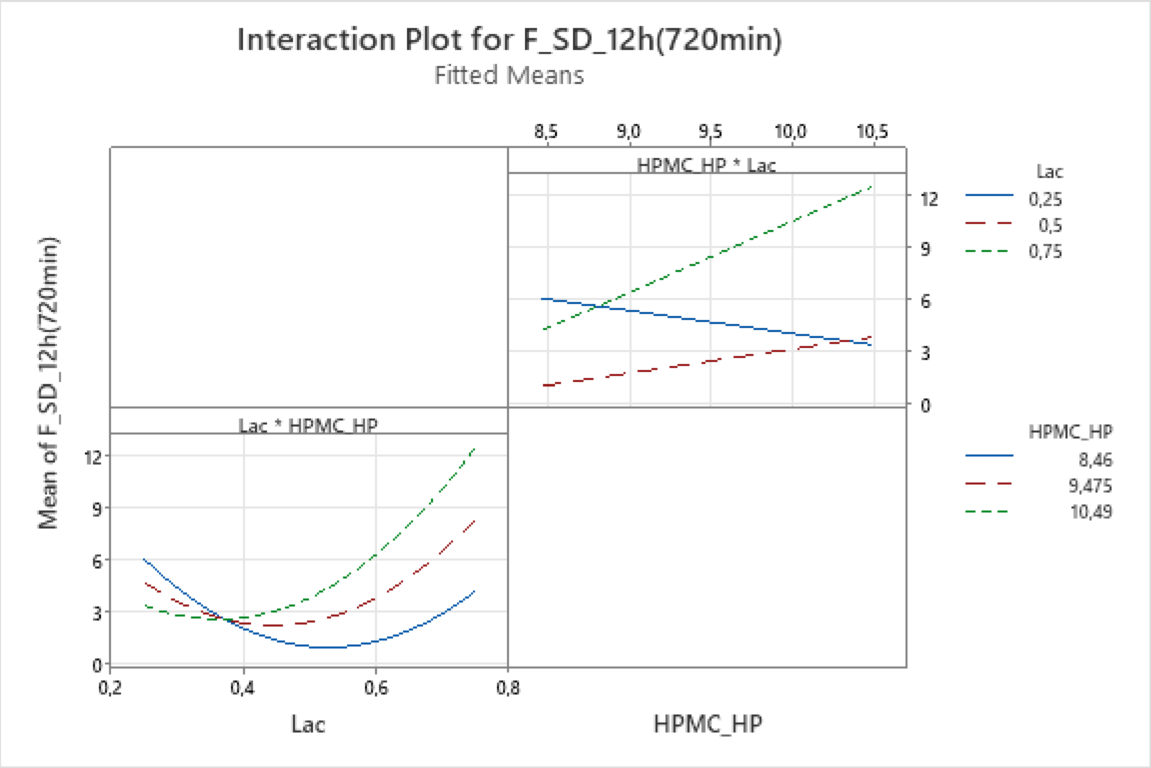

b)

Figure S87. Main effects plot (a) and Interaction plot for SD of carvedilol release using an Optimized MLR model at t = 12 h (720 min).

a)

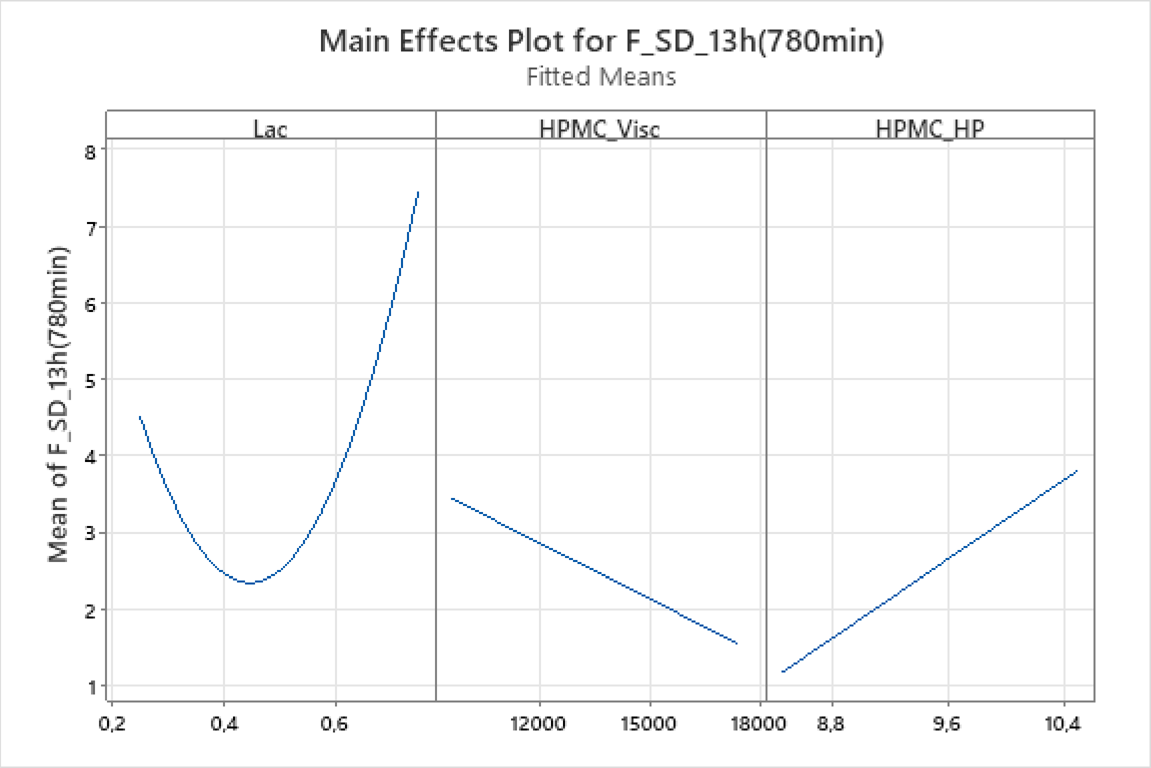

b)

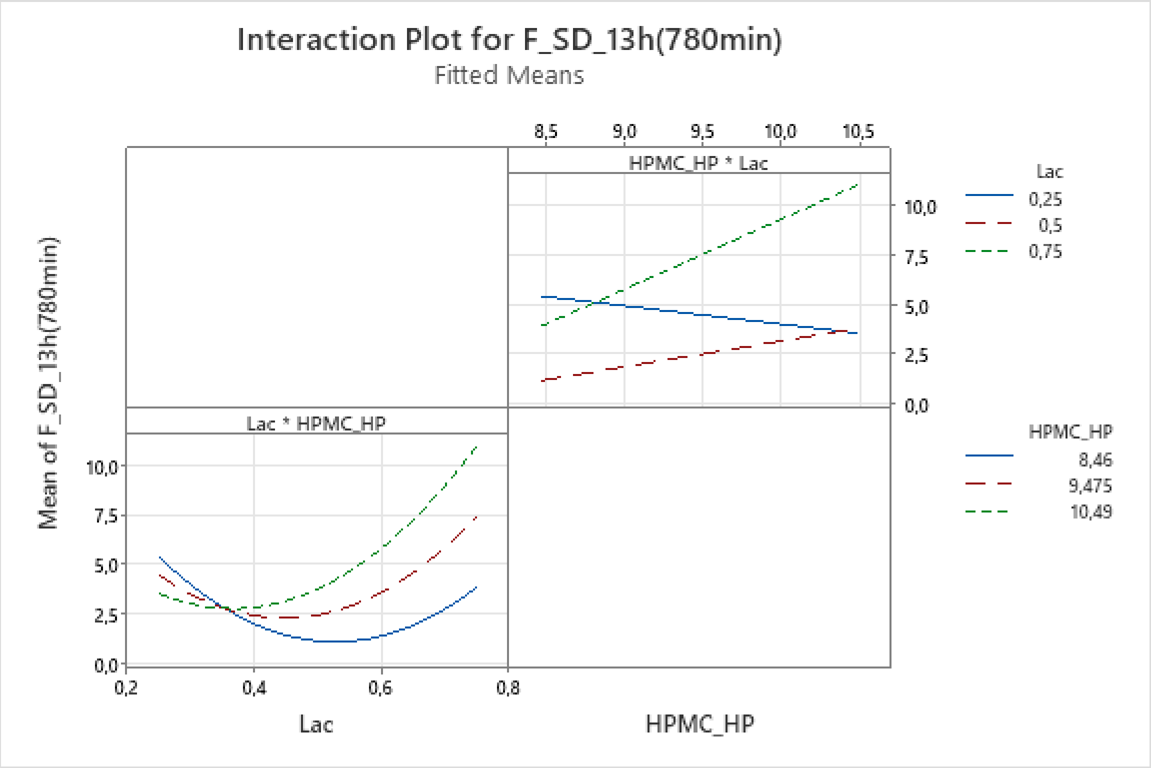

Figure S88. Main effects plot (a) and Interaction plot for SD of carvedilol release using an Optimized MLR model at t = 13 h (780 min).

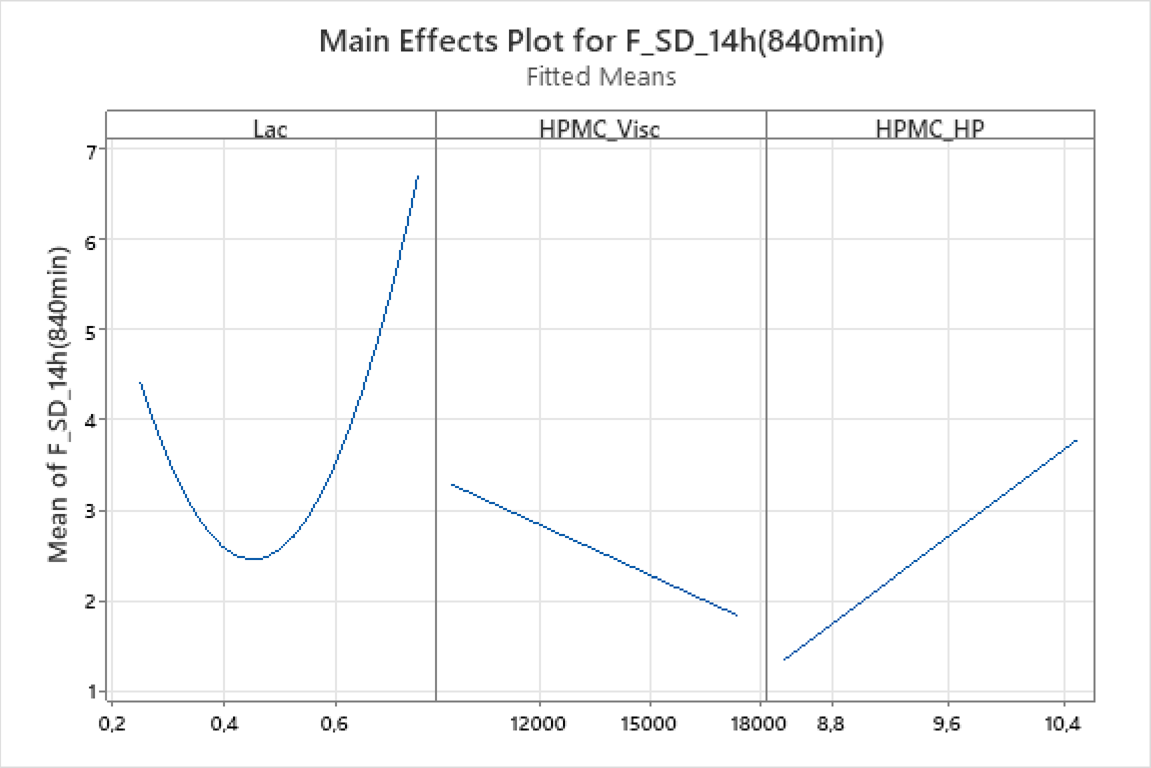

a)

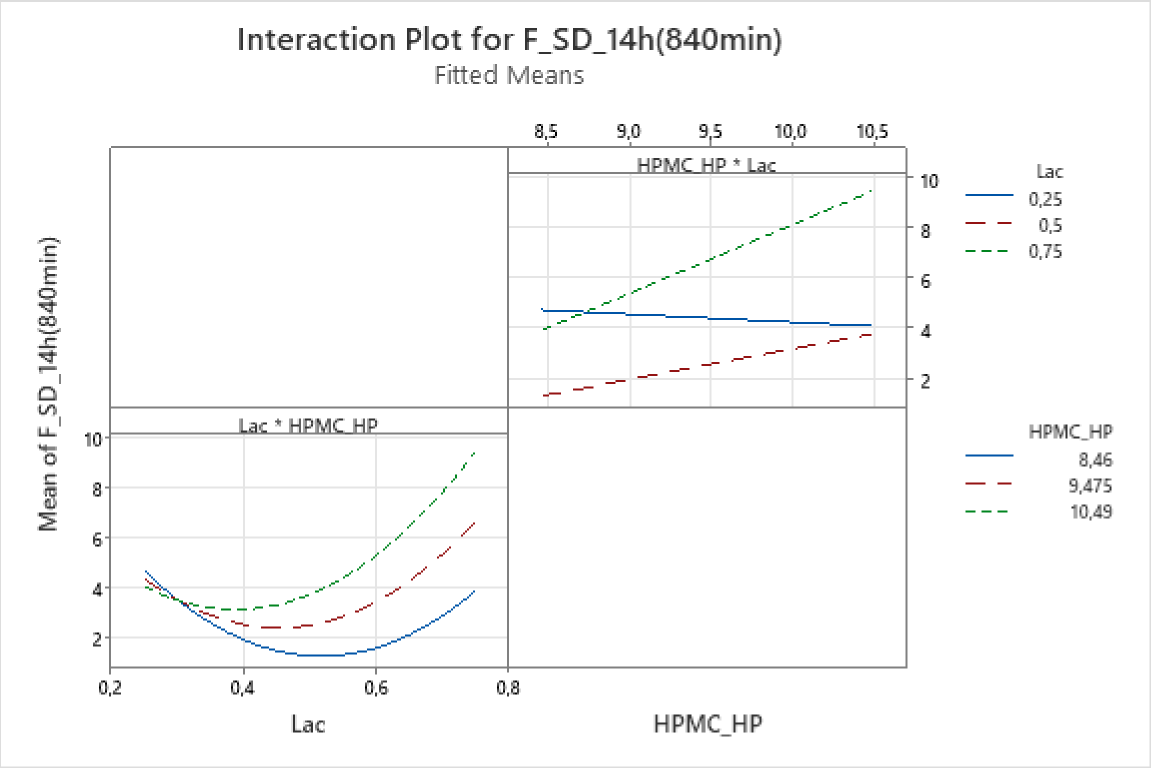

b)

Figure S89. Main effects plot (a) and Interaction plot for SD of carvedilol release using an Optimized MLR model at t = 14 h (840 min).

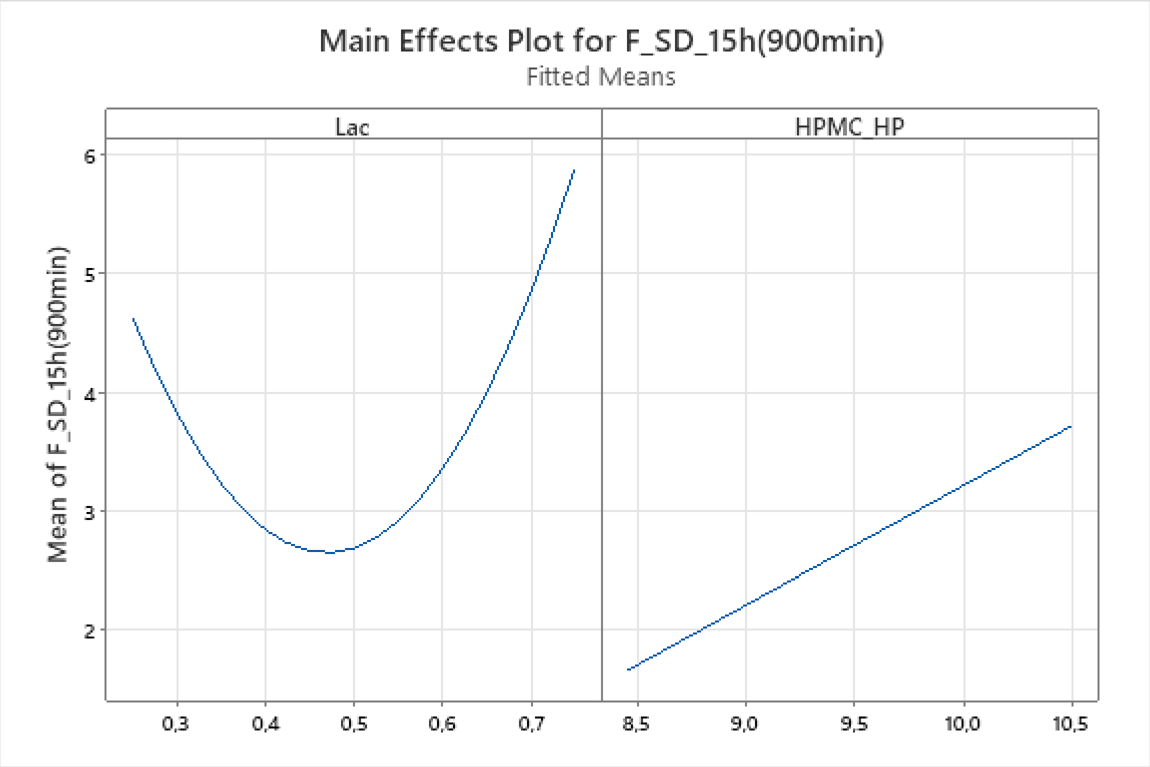

Figure S90. Main effects plot for SD of carvedilol release using an Optimized MLR model at t = 15 h (900 min).

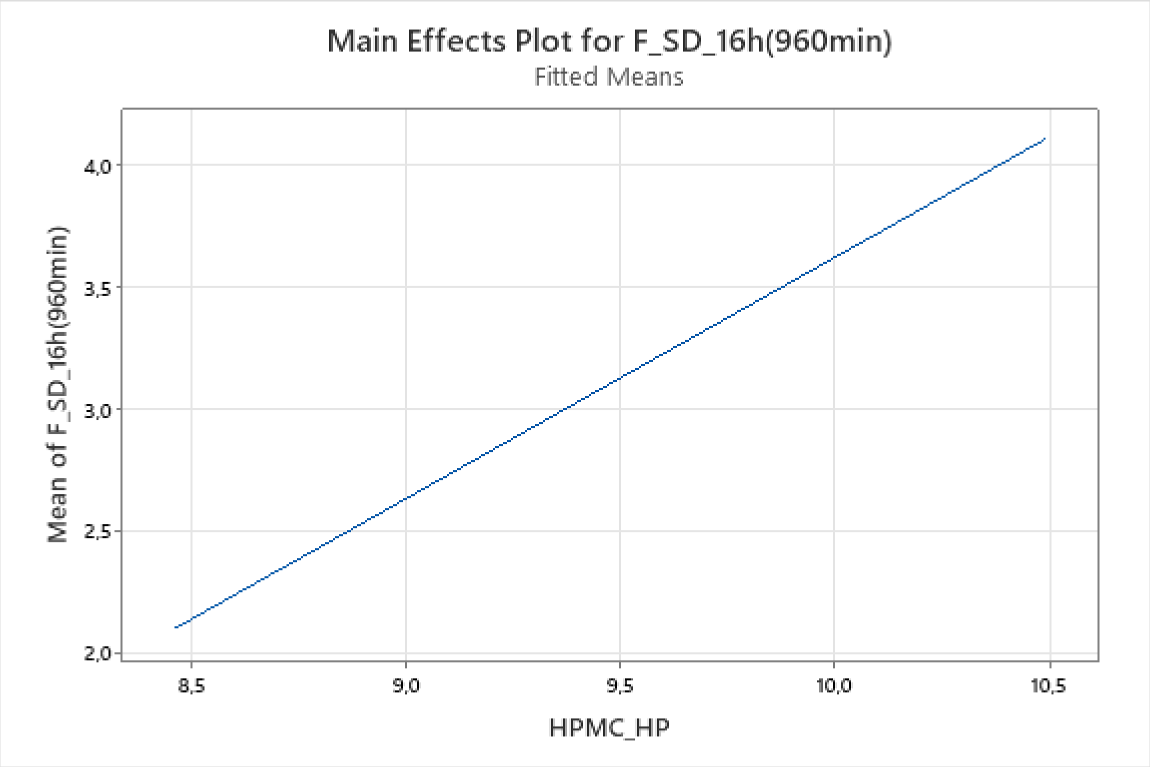

Figure S91. Main effects plot for SD of carvedilol release using an Optimized MLR model at t = 16 h (960 min).

a) 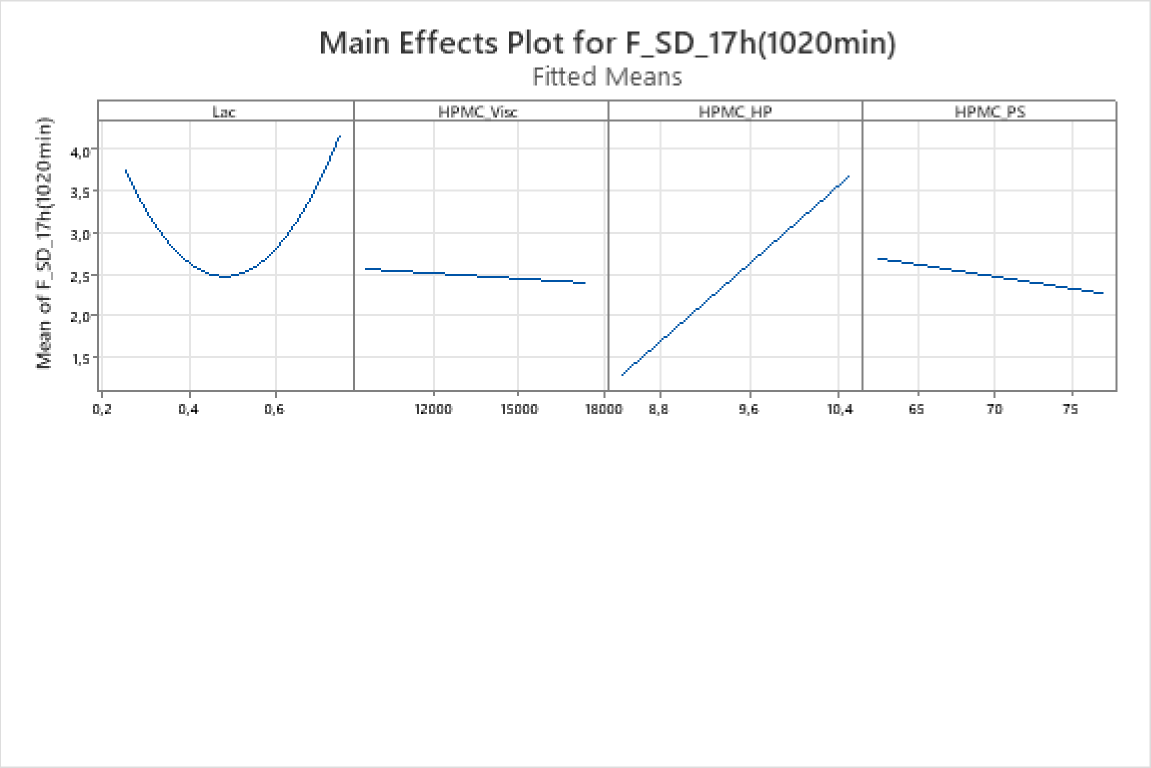

b) 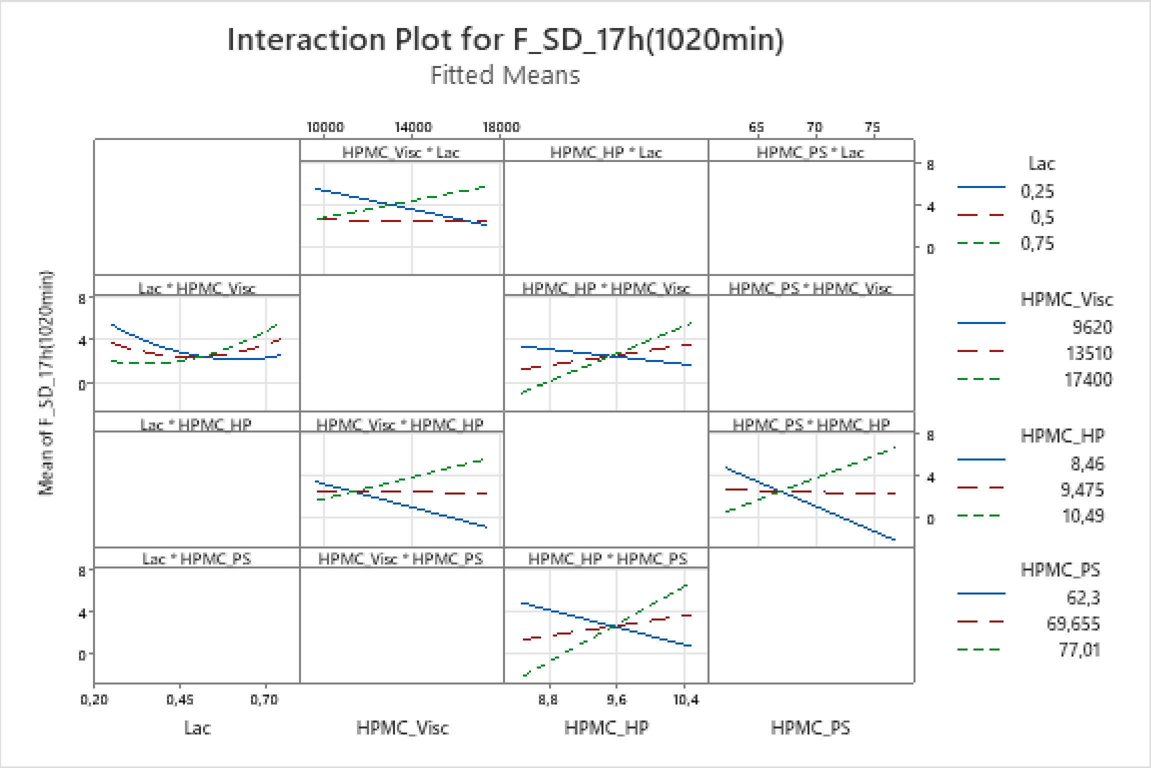

Figure S92. Main effects plot (a) and Interaction plot for SD of carvedilol release using an Optimized MLR model at t = 17 h (1020 min).

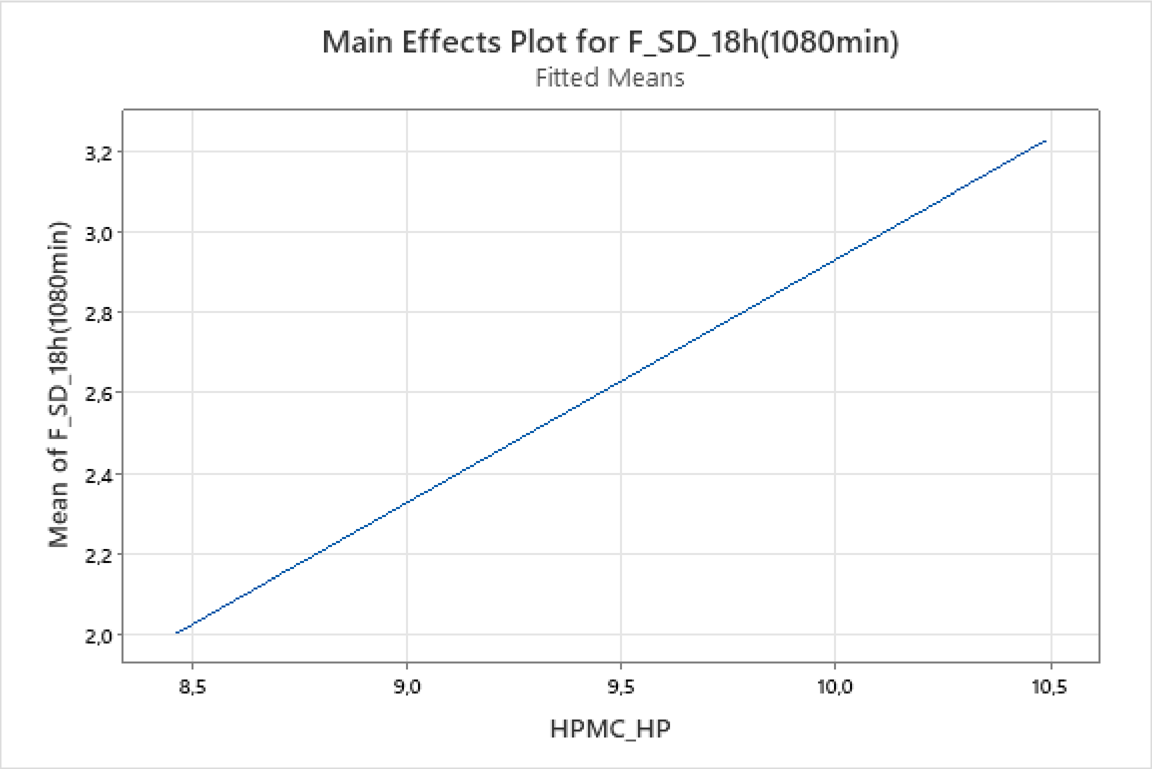

Figure S93. Main effects plot for SD of carvedilol release using an Optimized MLR model at t = 18 h (1080 min).

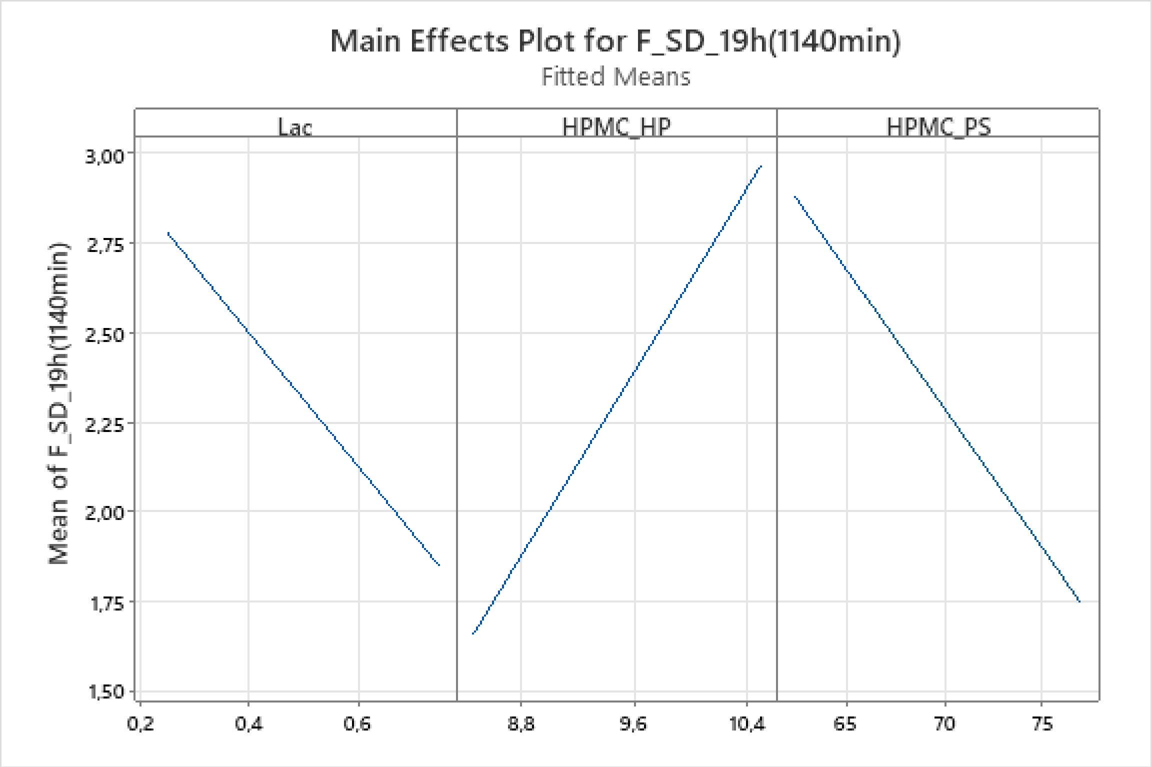

Figure S94. Main effects plot for SD of carvedilol release using an Optimized MLR model at t = 19 h (1140 min).

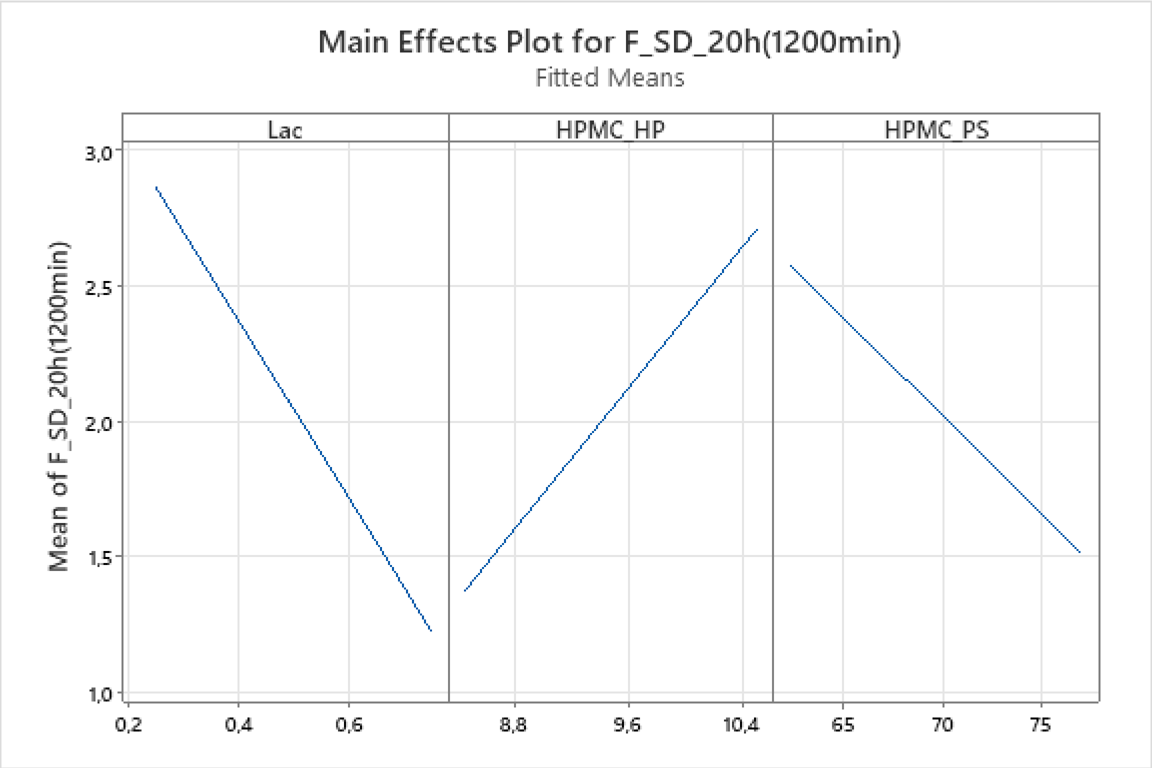

Figure S95. Main effects plot for SD of carvedilol release using an Optimized MLR model at t = 20 h (1200 min).

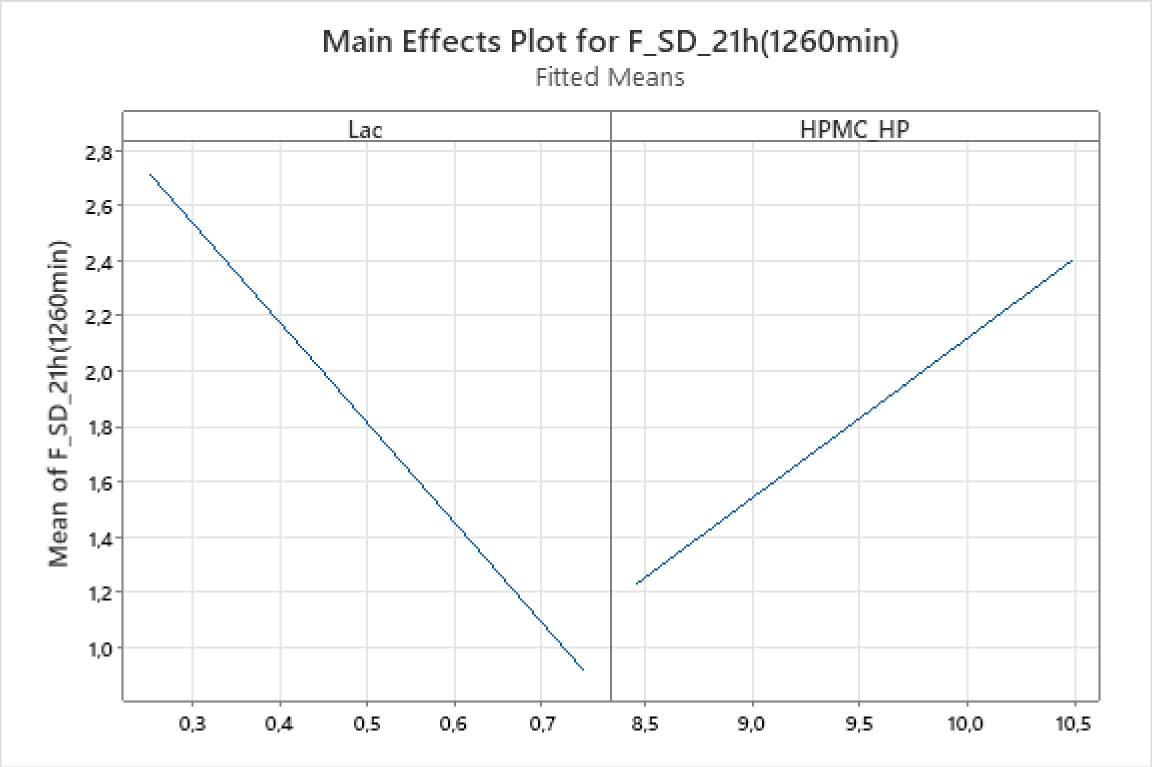

Figure S96. Main effects plot for SD of carvedilol release using an Optimized MLR model at t = 21 h (1260 min).

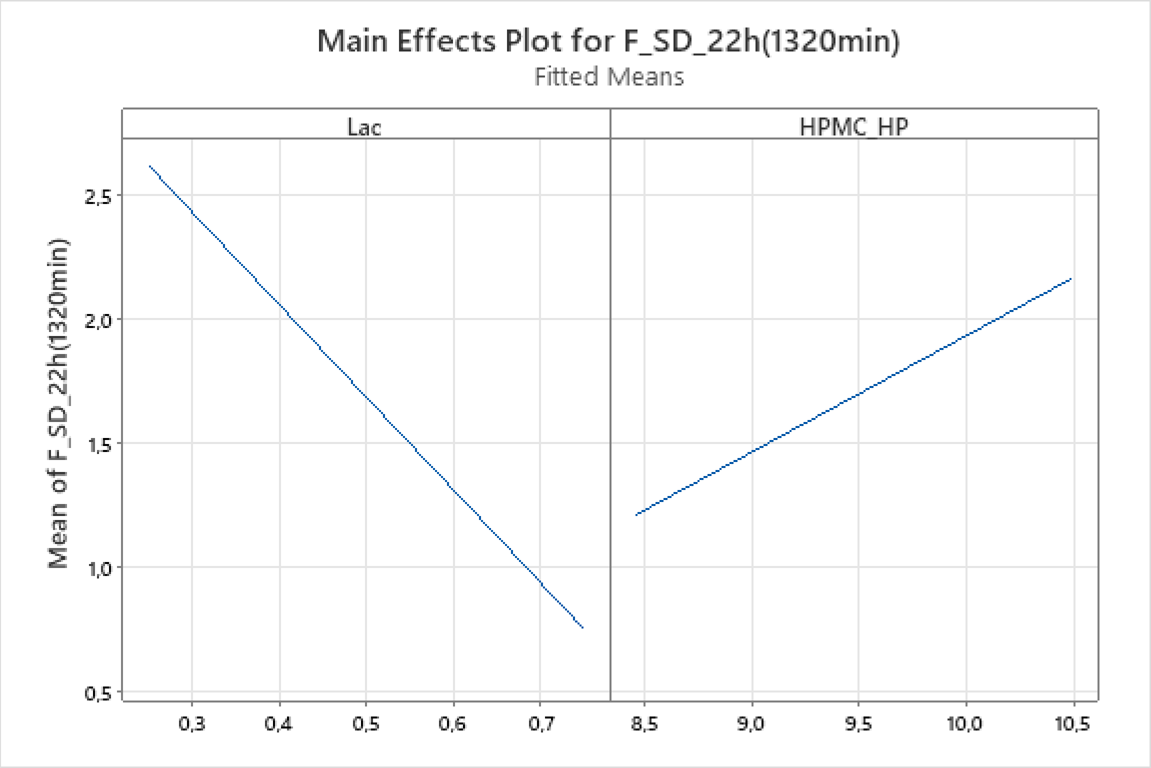

a)

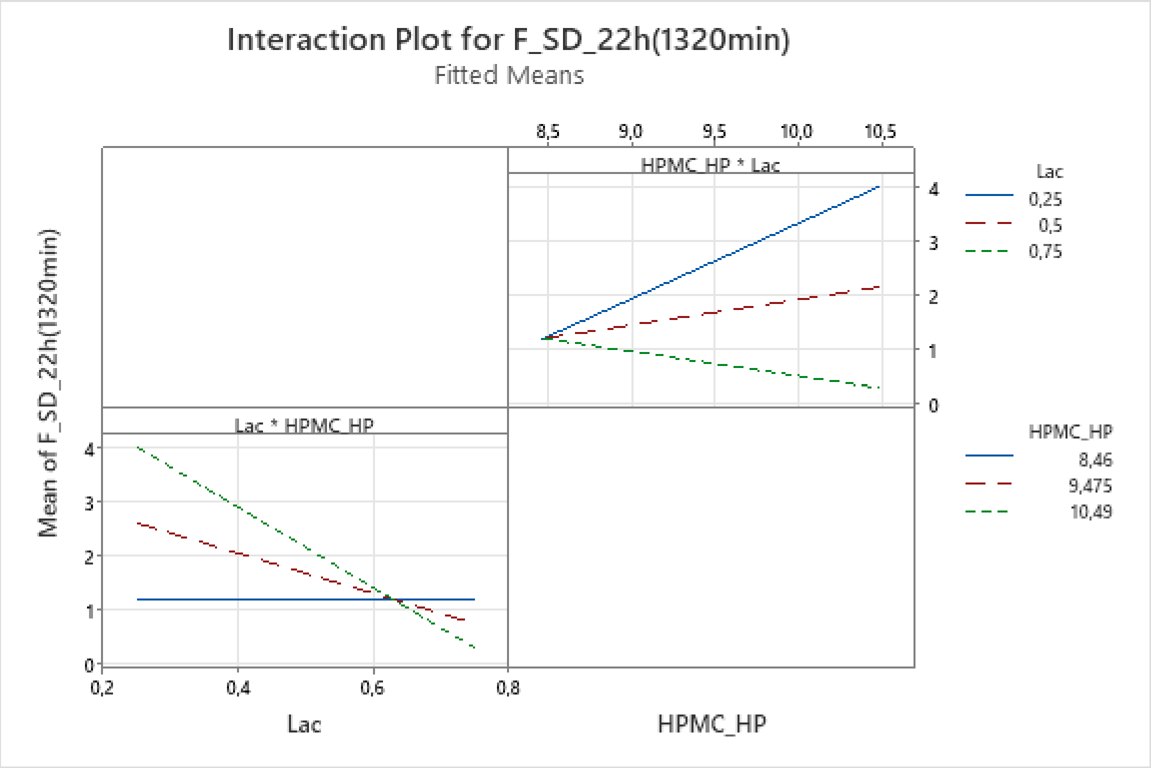

b)

Figure S97. Main effects plot (a) and Interaction plot for SD of carvedilol release using an Optimized MLR model at t = 22 h (1320 min).

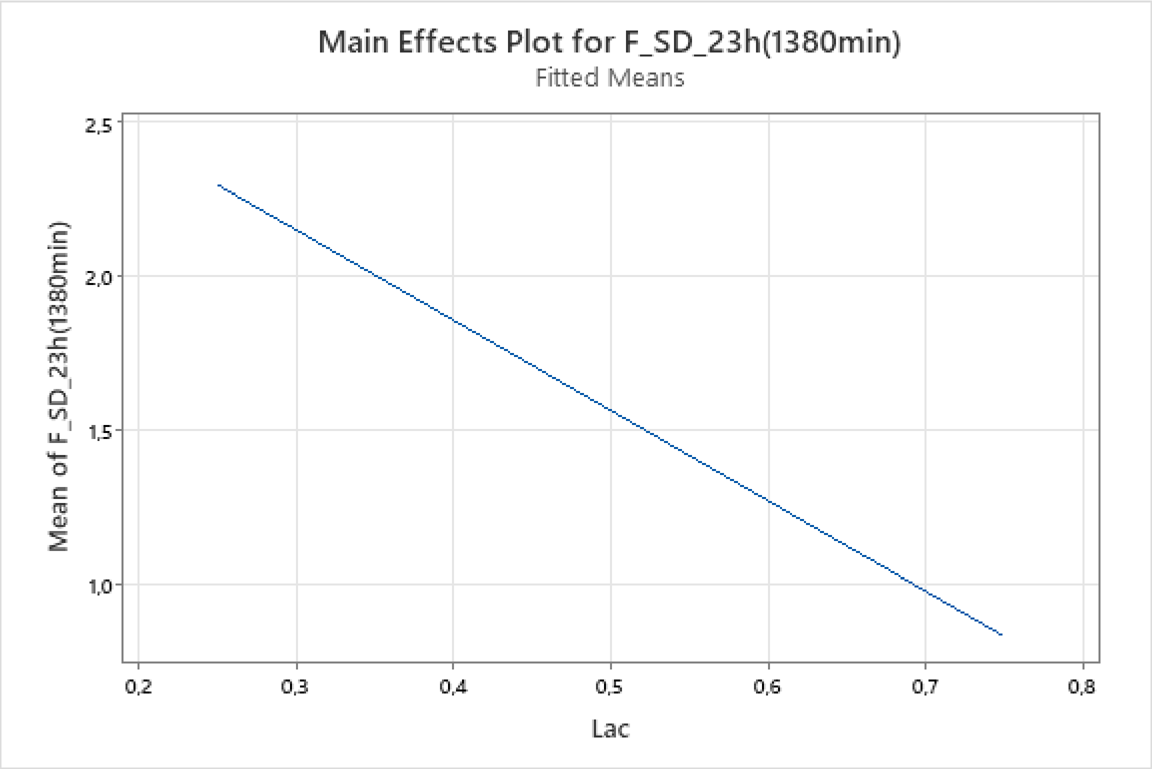

Figure S98. Main effects plot for SD of carvedilol release using an Optimized MLR model at t = 23 h (1380 min).

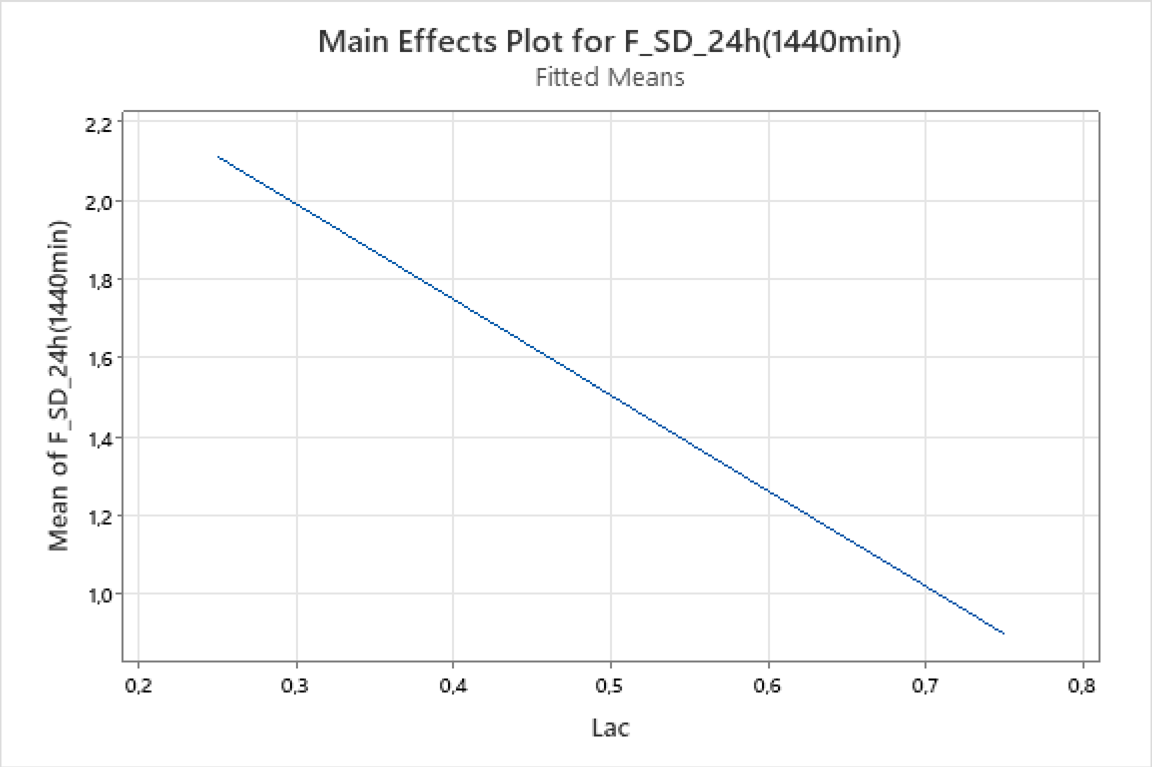

Figure S99. Main effects plot for SD of carvedilol release using an Optimized MLR model at t = 24 h (1440 min).

Table S20. In-process control (IPC) results for the tablet mass, measured on 20 tablets per experiment using a representative sample. Results are shown for the mean, minimum, and maximum tablet mass, where a more reddish colour tone represents a higher mass, a more bluish tone represents a lower mass, and a white tone indicates approximately the mean of all results. The range, standard deviation, and relative standard deviation of tablet mass are also shown, where longer bars indicate larger values and shorter bars indicate smaller ones. “DoE Id” data in the legend is comprised of theoretical levels (-2, -1, 0, 1, 2) of factors A (Lac), B (HPMC_Visc), C (HPMC_HP), and D (HPMC_PS) used in each experiment, respectively.

DoE Id	Mean mass of tablets (mg) (N = 20)	Minimal mass of tablets (mg) (N = 20)	Maximal mass of tablets (mg) (N = 20)	Range of mass of tablets (mg) (N = 20)	Standard deviation of mass of tablets (mg) (N = 20)	Relative standard deviation of mass of tablets i.e. RSD% (%) (N = 20)
0_0_0_0	647.6	641.7	653.5	11.8	3.3	0.51
0_-2_0_0	649.0	644.9	657.7	12.8	2.9	0.45
-1_1_-1_-1	648.0	636.4	656.0	19.6	5.4	0.83
-1_1_1_1	648.9	638.4	655.5	17.1	5.6	0.86
0_2_0_0	647.8	638.9	653.2	14.3	3.8	0.58
1_-1_-1_-1	648.9	642.9	656.2	13.3	3.8	0.59
1_1_-1_1	648.5	644.7	652.9	8.2	1.9	0.29
2_0_0_0	649.3	643.8	656.1	12.3	3.7	0.57
1_-1_-1_1	651.8	647.2	657.7	10.5	2.7	0.42
1_1_1_1	648.4	641.2	656.9	15.7	4.3	0.67
-1_-1_-1_1	648.2	641.8	658.5	16.7	3.6	0.55
1_1_-1_-1	648.5	642.5	654.9	12.4	3.8	0.59
0_0_0_2	648.2	642.6	656.0	13.4	3.3	0.51
0_0_0_0	647.4	641.3	657.4	16.1	4.7	0.72
-1_-1_1_1	647.8	642.3	653.0	10.7	2.9	0.44
1_-1_1_1	648.6	643.3	657.9	14.6	3.7	0.57
1_-1_1_-1	647.1	642.6	652.1	9.5	3.3	0.50
0_0_0_-2	647.6	641.8	653.6	11.8	3.3	0.50
-1_1_-1_1	647.1	642.9	654.2	11.3	3.6	0.56
0_0_-2_0	647.8	644.2	654.5	10.3	3.2	0.50
0_0_2_0	648.2	643.7	652.9	9.2	2.6	0.40
-2_0_0_0	645.9	641.6	650.8	9.2	2.8	0.44
-1_1_1_-1	647.6	643.5	651.6	8.1	2.0	0.31
-1_-1_-1_-1	649.3	643.7	654.2	10.5	3.0	0.46
-1_-1_1_-1	649.0	642.8	656.1	13.3	3.9	0.59
1_1_1_-1	648.4	642.4	656.6	14.2	4.0	0.61
0_0_0_0	647.8	643.0	656.0	13.0	3.5	0.54

Table S21. In-process control (IPC) results for the tablet hardness, measured on 10 tablets per experiment using a representative sample. Results are shown for the mean, minimum, and maximum tablet hardness, where a more reddish colour tone represents a higher hardness, a more bluish tone represents a lower hardness, and a white tone indicates approximately the mean of all results. The range, standard deviation, and relative standard deviation of tablet hardness are also shown, where longer bars indicate larger values and shorter bars indicate smaller ones. "DoE Id" data in the legend is comprised of theoretical levels (-2, -1, 0, 1, 2) of factors A (Lac), B (HPMC_Visc), C (HPMC_HP), and D (HPMC_PS) used in each experiment, respectively.

DoE Id	Mean hardness of tablets (N) (N = 10)	Minimal hardness of tablets (N) (N = 10)	Maximal hardness of tablets (N) (N = 10)	Range of hardness of tablets (N) (N = 10)	Standard deviation of hardness of tablets (N) (N = 10)	Relative standard deviation of hardness of tablets i.e. RSD% (%) (N = 10)
0_0_0_0	388	373	402	29	9	2.34
0_-2_0_0	392	383	402	19	6	1.63
-1_1_-1_-1	399	385	403	18	5	1.20
-1_1_1_1	398	377	405	28	7	1.79
0_2_0_0	393	370	403	33	8	2.13
1_-1_-1_-1	377	357	392	35	9	2.39
1_1_-1_1	378	359	393	34	8	2.19
2_0_0_0	344	326	360	34	10	2.79
1_-1_-1_1	368	357	379	22	7	1.85
1_1_1_1	358	342	379	37	9	2.55
-1_-1_-1_1	400	388	402	14	4	0.95
1_1_-1_-1	369	358	386	28	8	2.15
0_0_0_2	379	367	396	29	9	2.43
0_0_0_0	387	370	405	35	10	2.53
-1_-1_1_1	398	383	404	21	7	1.64
1_-1_1_1	359	345	379	34	8	2.18
1_-1_1_-1	361	340	373	33	9	2.43
0_0_0_-2	381	358	395	37	9	2.35
-1_1_-1_1	399	392	405	13	4	1.03
0_0_-2_0	391	376	402	26	8	1.96
0_0_2_0	375	362	387	25	6	1.64
-2_0_0_0	402	400	405	5	2	0.39
-1_1_1_-1	401	397	403	6	1	0.35
-1_-1_-1_-1	402	398	406	8	2	0.52
-1_-1_1_-1	401	393	407	14	3	0.86
1_1_1_-1	361	343	378	35	11	2.93
0_0_0_0	386	375	402	27	8	2.02

Table S22. In-process control (IPC) results for the tablet thickness, measured on 10 tablets per experiment using a representative sample. Results are shown for the mean, minimum, and maximum tablet thickness, where a more reddish colour tone represents a higher thickness, a more bluish tone represents a lower thickness, and a white tone indicates approximately the mean of all results. The range, standard deviation, and relative standard deviation of tablet thickness are also shown, where longer bars indicate larger values and shorter bars indicate smaller ones. “DoE Id” data in the legend is comprised of theoretical levels (-2, -1, 0, 1, 2) of factors A (Lac), B (HPMC_Visc), C (HPMC_HP), and D (HPMC_PS) used in each experiment, respectively.

DoE Id	Mean thickness of tablets (mm) (N = 10)	Minimal thickness of tablets (mm) (N = 10)	Maximal thickness of tablets (mm) (N = 10)	Range of thickness of tablets (mm) (N = 10)	Standard deviation of thickness of tablets (mm) (N = 10)	Relative standard deviation of thickness of tablets i.e. RSD% (%) (N = 10)
0_0_0_0	4.91	4.88	4.94	0.06	0.01	0.30
0_-2_0_0	4.92	4.90	4.95	0.05	0.01	0.24
-1_1_-1_-1	4.91	4.87	4.95	0.08	0.02	0.43
-1_1_1_1	4.93	4.89	4.96	0.07	0.02	0.39
0_2_0_0	4.92	4.89	4.95	0.06	0.02	0.37
1_-1_-1_-1	4.92	4.90	4.94	0.04	0.01	0.25
1_1_-1_1	4.91	4.89	4.94	0.05	0.01	0.26
2_0_0_0	4.93	4.90	4.95	0.05	0.02	0.35
1_-1_-1_1	4.94	4.92	4.96	0.04	0.01	0.25
1_1_1_1	4.93	4.90	4.96	0.06	0.02	0.38
-1_-1_-1_1	4.92	4.89	4.96	0.07	0.02	0.38
1_1_-1_-1	4.91	4.89	4.94	0.05	0.02	0.41
0_0_0_2	4.91	4.88	4.94	0.06	0.02	0.33
0_0_0_0	4.90	4.86	4.94	0.08	0.02	0.41
-1_-1_1_1	4.92	4.90	4.94	0.04	0.01	0.28
1_-1_1_1	4.93	4.91	4.96	0.05	0.02	0.34
1_-1_1_-1	4.91	4.89	4.94	0.05	0.02	0.31
0_0_0_-2	4.93	4.90	4.96	0.06	0.02	0.34
-1_1_-1_1	4.93	4.90	4.95	0.05	0.01	0.30
0_0_-2_0	4.93	4.90	4.96	0.06	0.02	0.36
0_0_2_0	4.93	4.90	4.96	0.06	0.02	0.35
-2_0_0_0	4.91	4.89	4.93	0.04	0.01	0.23
-1_1_1_-1	4.93	4.90	4.95	0.05	0.01	0.30
-1_-1_-1_-1	4.94	4.90	4.96	0.06	0.01	0.29
-1_-1_1_-1	4.93	4.90	4.96	0.06	0.02	0.34
1_1_1_-1	4.94	4.91	4.97	0.06	0.02	0.34
0_0_0_0	4.91	4.88	4.96	0.08	0.02	0.41

Table S23. In-process control (IPC) results for tablet friability, measured on approximately 6.5-gram samples of tablets per experiment using a representative sample. Longer bars indicate larger values, while shorter bars indicate smaller ones. The “DoE ID” data in the legend comprise the theoretical levels (−2, −1, 0, 1, 2) of factors A (Lac), B (HPMC_Visc), C (HPMC_HP), and D (HPMC_PS) used in each experiment, respectively.

DoE Id	Friability of tablets (%)
0_0_0_0	0.02
0_-2_0_0	0.02
-1_1_-1_-1	0.00
-1_1_1_1	0.03
0_2_0_0	0.05
1_-1_-1_-1	0.00
1_1_-1_1	0.02
2_0_0_0	0.02
1_-1_-1_1	0.00
1_1_1_1	0.00
-1_-1_-1_1	0.00
1_1_-1_-1	0.06
0_0_0_2	0.03
0_0_0_0	0.00
-1_-1_1_1	0.03
1_-1_1_1	0.00
1_-1_1_-1	0.05
0_0_0_-2	0.05
-1_1_-1_1	0.02
0_0_-2_0	0.02
0_0_2_0	0.05
-2_0_0_0	0.00
-1_1_1_-1	0.05
-1_-1_-1_-1	0.05
-1_-1_1_-1	0.03
1_1_1_-1	0.05
0_0_0_0	0.02
