# Supplementary material for: Influence of Selected Hypromellose Functionality-Related Characteristics and Soluble/Insoluble Filler Ratio on Carvedilol Release from Matrix Tablets
Source: Pharmaceutics. 2025 Oct 21;17(10):1358. doi: 10.3390/pharmaceutics17101358 (PMC12566823; doi:10.3390/pharmaceutics17101358)
Supplement: Supplementary file 1 [file pharmaceutics-17-01358-s001.zip › Report_Mean Release Analysis_RSM(CCD)_Full model.htm]

# Mean Release Analysis, Response Surface Design (Central Composite Design), Full model

## Coded Coefficients

| Term | Coef | SE Coef | 95% CI | T-Value | P-Value | VIF |
| --- | --- | --- | --- | --- | --- | --- |
| Constant | 9,541 | 0,845 | (7,699; 11,383) | 11,29 | 0,000 |  |
| Lac | 2,450 | 0,699 | (0,927; 3,973) | 3,51 | 0,004 | 1,18 |
| HPMC\_Visc | 0,789 | 0,730 | (-0,803; 2,380) | 1,08 | 0,301 | 1,70 |
| HPMC\_HP | 0,464 | 0,715 | (-1,093; 2,021) | 0,65 | 0,528 | 1,26 |
| HPMC\_PS | 2,40 | 1,10 | (0,02; 4,79) | 2,19 | 0,049 | 2,09 |
| Lac\*Lac | 0,72 | 1,39 | (-2,31; 3,74) | 0,52 | 0,615 | 1,30 |
| HPMC\_Visc\*HPMC\_Visc | 2,04 | 1,48 | (-1,18; 5,25) | 1,38 | 0,193 | 1,96 |
| HPMC\_HP\*HPMC\_HP | 1,98 | 1,40 | (-1,07; 5,04) | 1,42 | 0,182 | 1,81 |
| HPMC\_PS\*HPMC\_PS | 0,18 | 1,39 | (-2,86; 3,21) | 0,13 | 0,902 | 1,42 |
| Lac\*HPMC\_Visc | -2,39 | 1,53 | (-5,72; 0,95) | -1,56 | 0,145 | 1,49 |
| Lac\*HPMC\_HP | 1,26 | 1,61 | (-2,24; 4,76) | 0,78 | 0,449 | 1,17 |
| Lac\*HPMC\_PS | -1,35 | 2,48 | (-6,76; 4,05) | -0,55 | 0,595 | 1,42 |
| HPMC\_Visc\*HPMC\_HP | -1,39 | 1,83 | (-5,37; 2,60) | -0,76 | 0,463 | 2,73 |
| HPMC\_Visc\*HPMC\_PS | 6,25 | 2,58 | (0,62; 11,88) | 2,42 | 0,032 | 2,74 |
| HPMC\_HP\*HPMC\_PS | -1,53 | 2,85 | (-7,75; 4,68) | -0,54 | 0,600 | 2,70 |

## Model Summary

| S | R-sq | R-sq(adj) | PRESS | R-sq(pred) | AICc | BIC |
| --- | --- | --- | --- | --- | --- | --- |
| 1,57374 | 71,08% | 37,33% | 127,477 | 0,00% | 165,61 | 131,95 |

## Analysis of Variance

| Source | DF | Seq SS | Contribution | Adj SS | Adj MS | F-Value | P-Value |
| --- | --- | --- | --- | --- | --- | --- | --- |
| Model | 14 | 73,029 | 71,08% | 73,0290 | 5,2164 | 2,11 | 0,102 |
| Linear | 4 | 47,392 | 46,12% | 43,1674 | 10,7918 | 4,36 | 0,021 |
| Lac | 1 | 41,230 | 40,13% | 30,4353 | 30,4353 | 12,29 | 0,004 |
| HPMC\_Visc | 1 | 0,444 | 0,43% | 2,8883 | 2,8883 | 1,17 | 0,301 |
| HPMC\_HP | 1 | 1,395 | 1,36% | 1,0449 | 1,0449 | 0,42 | 0,528 |
| HPMC\_PS | 1 | 4,323 | 4,21% | 11,9150 | 11,9150 | 4,81 | 0,049 |
| Square | 4 | 3,867 | 3,76% | 8,1068 | 2,0267 | 0,82 | 0,538 |
| Lac\*Lac | 1 | 0,013 | 0,01% | 0,6601 | 0,6601 | 0,27 | 0,615 |
| HPMC\_Visc\*HPMC\_Visc | 1 | 0,108 | 0,10% | 4,7153 | 4,7153 | 1,90 | 0,193 |
| HPMC\_HP\*HPMC\_HP | 1 | 0,668 | 0,65% | 4,9609 | 4,9609 | 2,00 | 0,182 |
| HPMC\_PS\*HPMC\_PS | 1 | 3,078 | 3,00% | 0,0394 | 0,0394 | 0,02 | 0,902 |
| 2-Way Interaction | 6 | 21,770 | 21,19% | 21,7696 | 3,6283 | 1,46 | 0,270 |
| Lac\*HPMC\_Visc | 1 | 5,203 | 5,06% | 6,0081 | 6,0081 | 2,43 | 0,145 |
| Lac\*HPMC\_HP | 1 | 1,121 | 1,09% | 1,5205 | 1,5205 | 0,61 | 0,449 |
| Lac\*HPMC\_PS | 1 | 0,738 | 0,72% | 0,7379 | 0,7379 | 0,30 | 0,595 |
| HPMC\_Visc\*HPMC\_HP | 1 | 0,118 | 0,11% | 1,4225 | 1,4225 | 0,57 | 0,463 |
| HPMC\_Visc\*HPMC\_PS | 1 | 13,872 | 13,50% | 14,5001 | 14,5001 | 5,85 | 0,032 |
| HPMC\_HP\*HPMC\_PS | 1 | 0,718 | 0,70% | 0,7175 | 0,7175 | 0,29 | 0,600 |
| Error | 12 | 29,720 | 28,92% | 29,7199 | 2,4767 |  |  |
| Lack-of-Fit | 10 | 15,690 | 15,27% | 15,6905 | 1,5690 | 0,22 | 0,959 |
| Pure Error | 2 | 14,029 | 13,65% | 14,0295 | 7,0147 |  |  |
| Total | 26 | 102,749 | 100,00% |  |  |  |  |

## Regression Equation in Uncoded Units

|  |  |  |
| --- | --- | --- |
| F\_mean\_0.17h(10min) | = | 197 + 36 Lac - 0,01410 HPMC\_Visc - 19,4 HPMC\_HP - 0,76 HPMC\_PS + 11,5 Lac\*Lac + 0,000000 HPMC\_Visc\*HPMC\_Visc + 1,92 HPMC\_HP\*HPMC\_HP + 0,0032 HPMC\_PS\*HPMC\_PS - 0,00245 Lac\*HPMC\_Visc + 4,97 Lac\*HPMC\_HP - 0,74 Lac\*HPMC\_PS - 0,000351 HPMC\_Visc\*HPMC\_HP + 0,000218 HPMC\_Visc\*HPMC\_PS - 0,206 HPMC\_HP\*HPMC\_PS |

## Fits and Diagnostics for All Observations

| Obs | F\_mean\_0.17h(10min) | Fit | SE Fit | 95% CI | Resid | Std Resid | Del Resid |
| --- | --- | --- | --- | --- | --- | --- | --- |
| 1 | 9,10 | 8,63 | 1,27 | (5,85; 11,41) | 0,46 | 0,50 | 0,49 |
| 2 | 11,82 | 12,21 | 1,27 | (9,43; 14,98) | -0,39 | -0,42 | -0,40 |
| 3 | 7,32 | 7,91 | 1,34 | (4,98; 10,84) | -0,59 | -0,72 | -0,70 |
| 4 | 8,15 | 8,55 | 1,34 | (5,62; 11,48) | -0,40 | -0,49 | -0,48 |
| 5 | 7,10 | 8,53 | 1,09 | (6,16; 10,90) | -1,43 | -1,26 | -1,29 |
| 6 | 12,82 | 13,02 | 1,09 | (10,65; 15,39) | -0,20 | -0,17 | -0,17 |
| 7 | 7,48 | 8,21 | 1,31 | (5,37; 11,06) | -0,73 | -0,83 | -0,82 |
| 8 | 9,85 | 10,09 | 1,31 | (7,25; 12,94) | -0,25 | -0,28 | -0,27 |
| 9 | 8,62 | 8,42 | 1,20 | (5,81; 11,04) | 0,20 | 0,20 | 0,19 |
| 10 | 11,32 | 11,11 | 1,20 | (8,49; 13,72) | 0,21 | 0,21 | 0,20 |
| 11 | 9,40 | 10,66 | 1,03 | (8,43; 12,90) | -1,27 | -1,06 | -1,07 |
| 12 | 12,52 | 11,77 | 1,03 | (9,54; 14,01) | 0,75 | 0,63 | 0,61 |
| 13 | 7,87 | 7,81 | 1,19 | (5,22; 10,40) | 0,06 | 0,06 | 0,06 |
| 14 | 12,42 | 11,75 | 1,19 | (9,16; 14,33) | 0,67 | 0,65 | 0,64 |
| 15 | 10,50 | 10,41 | 1,04 | (8,16; 12,67) | 0,08 | 0,07 | 0,07 |
| 16 | 10,20 | 12,05 | 1,04 | (9,79; 14,30) | -1,85 | -1,56 | -1,68 |
| 17 | 8,25 | 7,23 | 1,22 | (4,58; 9,88) | 1,02 | 1,02 | 1,02 |
| 18 | 13,12 | 12,98 | 1,22 | (10,33; 15,63) | 0,14 | 0,14 | 0,14 |
| 19 | 9,65 | 10,32 | 1,00 | (8,13; 12,51) | -0,67 | -0,55 | -0,54 |
| 20 | 13,09 | 11,34 | 1,27 | (8,58; 14,10) | 1,76 | 1,88 | 2,14 |
| 21 | 10,47 | 10,44 | 1,01 | (8,25; 12,63) | 0,03 | 0,03 | 0,02 |
| 22 | 12,67 | 12,09 | 1,42 | (8,99; 15,19) | 0,58 | 0,86 | 0,85 |
| 23 | 9,30 | 8,70 | 1,43 | (5,59; 11,82) | 0,59 | 0,90 | 0,89 |
| 24 | 10,32 | 10,55 | 1,33 | (7,65; 13,44) | -0,22 | -0,27 | -0,26 |
| 25 | 8,15 | 9,39 | 0,85 | (7,54; 11,23) | -1,24 | -0,93 | -0,93 |
| 26 | 8,52 | 9,39 | 0,85 | (7,54; 11,23) | -0,86 | -0,65 | -0,63 |
| 27 | 12,91 | 9,39 | 0,85 | (7,54; 11,23) | 3,53 | 2,66 | 3,96 |

| Obs | HI | Cook’s D | DFITS |  |
| --- | --- | --- | --- | --- |
| 1 | 0,655994 | 0,03 | 0,67165 |  |
| 2 | 0,655994 | 0,02 | -0,55688 |  |
| 3 | 0,729945 | 0,09 | -1,15321 |  |
| 4 | 0,729945 | 0,04 | -0,78479 |  |
| 5 | 0,476813 | 0,10 | -1,23195 |  |
| 6 | 0,476813 | 0,00 | -0,15832 |  |
| 7 | 0,688750 | 0,10 | -1,21619 |  |
| 8 | 0,688750 | 0,01 | -0,40168 |  |
| 9 | 0,580949 | 0,00 | 0,22093 |  |
| 10 | 0,580949 | 0,00 | 0,23826 |  |
| 11 | 0,425781 | 0,06 | -0,91917 |  |
| 12 | 0,425781 | 0,02 | 0,52621 |  |
| 13 | 0,569258 | 0,00 | 0,06584 |  |
| 14 | 0,569258 | 0,04 | 0,73233 |  |
| 15 | 0,433227 | 0,00 | 0,05784 |  |
| 16 | 0,433227 | 0,12 | -1,46515 |  |
| 17 | 0,597775 | 0,10 | 1,24876 |  |
| 18 | 0,597775 | 0,00 | 0,16602 |  |
| 19 | 0,407038 | 0,01 | -0,44489 |  |
| 20 | 0,647412 | 0,43 | 2,90068 |  |
| 21 | 0,407933 | 0,00 | 0,02049 |  |
| 22 | 0,817044 | 0,22 | 1,79330 |  |
| 23 | 0,823434 | 0,25 | 1,91558 |  |
| 24 | 0,713775 | 0,01 | -0,40443 |  |
| 25 | 0,288793 | 0,02 | -0,59098 |  |
| 26 | 0,288793 | 0,01 | -0,40353 |  |
| 27 | 0,288793 | 0,19 | 2,52526 | R |

R  Large residual

## Coded Coefficients

| Term | Coef | SE Coef | 95% CI | T-Value | P-Value | VIF |
| --- | --- | --- | --- | --- | --- | --- |
| Constant | 13,090 | 0,990 | (10,932; 15,247) | 13,22 | 0,000 |  |
| Lac | 3,060 | 0,819 | (1,276; 4,845) | 3,74 | 0,003 | 1,18 |
| HPMC\_Visc | 0,970 | 0,856 | (-0,894; 2,834) | 1,13 | 0,279 | 1,70 |
| HPMC\_HP | 0,690 | 0,837 | (-1,134; 2,514) | 0,82 | 0,426 | 1,26 |
| HPMC\_PS | 3,34 | 1,28 | (0,54; 6,14) | 2,60 | 0,023 | 2,09 |
| Lac\*Lac | 0,61 | 1,63 | (-2,93; 4,16) | 0,38 | 0,713 | 1,30 |
| HPMC\_Visc\*HPMC\_Visc | 2,70 | 1,73 | (-1,07; 6,47) | 1,56 | 0,145 | 1,96 |
| HPMC\_HP\*HPMC\_HP | 2,44 | 1,64 | (-1,13; 6,02) | 1,49 | 0,163 | 1,81 |
| HPMC\_PS\*HPMC\_PS | 0,56 | 1,63 | (-2,99; 4,12) | 0,34 | 0,736 | 1,42 |
| Lac\*HPMC\_Visc | -3,16 | 1,79 | (-7,07; 0,75) | -1,76 | 0,104 | 1,49 |
| Lac\*HPMC\_HP | 1,42 | 1,88 | (-2,68; 5,53) | 0,76 | 0,464 | 1,17 |
| Lac\*HPMC\_PS | -2,21 | 2,91 | (-8,55; 4,12) | -0,76 | 0,461 | 1,42 |
| HPMC\_Visc\*HPMC\_HP | -1,46 | 2,14 | (-6,13; 3,21) | -0,68 | 0,509 | 2,73 |
| HPMC\_Visc\*HPMC\_PS | 7,81 | 3,03 | (1,22; 14,40) | 2,58 | 0,024 | 2,74 |
| HPMC\_HP\*HPMC\_PS | -1,50 | 3,34 | (-8,78; 5,78) | -0,45 | 0,662 | 2,70 |

## Model Summary

| S | R-sq | R-sq(adj) | PRESS | R-sq(pred) | AICc | BIC |
| --- | --- | --- | --- | --- | --- | --- |
| 1,84364 | 74,48% | 44,70% | 183,620 | 0,00% | 174,16 | 140,49 |

## Analysis of Variance

| Source | DF | Seq SS | Contribution | Adj SS | Adj MS | F-Value | P-Value |
| --- | --- | --- | --- | --- | --- | --- | --- |
| Model | 14 | 119,024 | 74,48% | 119,024 | 8,5017 | 2,50 | 0,060 |
| Linear | 4 | 80,062 | 50,10% | 72,662 | 18,1655 | 5,34 | 0,010 |
| Lac | 1 | 66,271 | 41,47% | 47,477 | 47,4768 | 13,97 | 0,003 |
| HPMC\_Visc | 1 | 1,411 | 0,88% | 4,367 | 4,3673 | 1,28 | 0,279 |
| HPMC\_HP | 1 | 2,846 | 1,78% | 2,311 | 2,3105 | 0,68 | 0,426 |
| HPMC\_PS | 1 | 9,533 | 5,97% | 22,982 | 22,9820 | 6,76 | 0,023 |
| Square | 4 | 4,551 | 2,85% | 12,647 | 3,1617 | 0,93 | 0,479 |
| Lac\*Lac | 1 | 0,404 | 0,25% | 0,482 | 0,4821 | 0,14 | 0,713 |
| HPMC\_Visc\*HPMC\_Visc | 1 | 0,047 | 0,03% | 8,258 | 8,2577 | 2,43 | 0,145 |
| HPMC\_HP\*HPMC\_HP | 1 | 0,987 | 0,62% | 7,524 | 7,5244 | 2,21 | 0,163 |
| HPMC\_PS\*HPMC\_PS | 1 | 3,114 | 1,95% | 0,404 | 0,4040 | 0,12 | 0,736 |
| 2-Way Interaction | 6 | 34,411 | 21,53% | 34,411 | 5,7352 | 1,69 | 0,207 |
| Lac\*HPMC\_Visc | 1 | 8,300 | 5,19% | 10,547 | 10,5468 | 3,10 | 0,104 |
| Lac\*HPMC\_HP | 1 | 1,189 | 0,74% | 1,941 | 1,9413 | 0,57 | 0,464 |
| Lac\*HPMC\_PS | 1 | 1,969 | 1,23% | 1,969 | 1,9690 | 0,58 | 0,461 |
| HPMC\_Visc\*HPMC\_HP | 1 | 0,295 | 0,18% | 1,576 | 1,5757 | 0,46 | 0,509 |
| HPMC\_Visc\*HPMC\_PS | 1 | 21,975 | 13,75% | 22,639 | 22,6393 | 6,66 | 0,024 |
| HPMC\_HP\*HPMC\_PS | 1 | 0,684 | 0,43% | 0,684 | 0,6839 | 0,20 | 0,662 |
| Error | 12 | 40,788 | 25,52% | 40,788 | 3,3990 |  |  |
| Lack-of-Fit | 10 | 23,163 | 14,49% | 23,163 | 2,3163 | 0,26 | 0,941 |
| Pure Error | 2 | 17,625 | 11,03% | 17,625 | 8,8124 |  |  |
| Total | 26 | 159,812 | 100,00% |  |  |  |  |

## Regression Equation in Uncoded Units

|  |  |  |
| --- | --- | --- |
| F\_mean\_0.33h(20min) | = | 304 + 77 Lac - 0,01845 HPMC\_Visc - 28,1 HPMC\_HP - 2,18 HPMC\_PS + 9,8 Lac\*Lac + 0,000000 HPMC\_Visc\*HPMC\_Visc + 2,37 HPMC\_HP\*HPMC\_HP + 0,0104 HPMC\_PS\*HPMC\_PS - 0,00325 Lac\*HPMC\_Visc + 5,61 Lac\*HPMC\_HP - 1,20 Lac\*HPMC\_PS - 0,000369 HPMC\_Visc\*HPMC\_HP + 0,000273 HPMC\_Visc\*HPMC\_PS - 0,201 HPMC\_HP\*HPMC\_PS |

## Fits and Diagnostics for All Observations

| Obs | F\_mean\_0.33h(20min) | Fit | SE Fit | 95% CI | Resid | Std Resid | Del Resid |
| --- | --- | --- | --- | --- | --- | --- | --- |
| 1 | 12,42 | 11,84 | 1,49 | (8,59; 15,10) | 0,58 | 0,53 | 0,52 |
| 2 | 16,29 | 16,67 | 1,49 | (13,42; 19,93) | -0,39 | -0,36 | -0,34 |
| 3 | 10,02 | 10,76 | 1,58 | (7,32; 14,19) | -0,74 | -0,77 | -0,75 |
| 4 | 11,39 | 11,85 | 1,58 | (8,41; 15,28) | -0,45 | -0,47 | -0,46 |
| 5 | 10,07 | 11,66 | 1,27 | (8,89; 14,44) | -1,59 | -1,19 | -1,22 |
| 6 | 17,07 | 17,45 | 1,27 | (14,67; 20,22) | -0,38 | -0,28 | -0,27 |
| 7 | 10,51 | 11,40 | 1,53 | (8,07; 14,74) | -0,89 | -0,87 | -0,86 |
| 8 | 13,41 | 13,75 | 1,53 | (10,42; 17,09) | -0,34 | -0,33 | -0,32 |
| 9 | 12,07 | 11,82 | 1,41 | (8,76; 14,88) | 0,25 | 0,21 | 0,20 |
| 10 | 15,42 | 15,18 | 1,41 | (12,12; 18,24) | 0,24 | 0,20 | 0,19 |
| 11 | 12,97 | 14,42 | 1,20 | (11,80; 17,04) | -1,45 | -1,04 | -1,04 |
| 12 | 16,49 | 15,73 | 1,20 | (13,11; 18,35) | 0,76 | 0,54 | 0,53 |
| 13 | 11,35 | 11,36 | 1,39 | (8,33; 14,39) | -0,02 | -0,01 | -0,01 |
| 14 | 16,91 | 16,07 | 1,39 | (13,04; 19,10) | 0,84 | 0,70 | 0,68 |
| 15 | 14,39 | 14,39 | 1,21 | (11,75; 17,03) | -0,00 | -0,00 | -0,00 |
| 16 | 13,89 | 16,21 | 1,21 | (13,57; 18,86) | -2,32 | -1,67 | -1,83 |
| 17 | 11,04 | 9,85 | 1,43 | (6,74; 12,96) | 1,19 | 1,02 | 1,02 |
| 18 | 17,44 | 17,16 | 1,43 | (14,05; 20,26) | 0,28 | 0,24 | 0,23 |
| 19 | 13,39 | 14,27 | 1,18 | (11,71; 16,83) | -0,88 | -0,62 | -0,60 |
| 20 | 17,59 | 15,37 | 1,48 | (12,14; 18,61) | 2,21 | 2,02 | 2,38 |
| 21 | 14,09 | 14,14 | 1,18 | (11,57; 16,70) | -0,05 | -0,03 | -0,03 |
| 22 | 17,14 | 16,37 | 1,67 | (12,74; 20,00) | 0,77 | 0,98 | 0,98 |
| 23 | 12,77 | 12,10 | 1,67 | (8,45; 15,74) | 0,67 | 0,87 | 0,86 |
| 24 | 14,77 | 14,96 | 1,56 | (11,57; 18,35) | -0,19 | -0,19 | -0,19 |
| 25 | 11,69 | 12,89 | 0,99 | (10,73; 15,05) | -1,20 | -0,77 | -0,76 |
| 26 | 11,92 | 12,89 | 0,99 | (10,73; 15,05) | -0,97 | -0,62 | -0,61 |
| 27 | 16,95 | 12,89 | 0,99 | (10,73; 15,05) | 4,05 | 2,61 | 3,79 |

| Obs | HI | Cook’s D | DFITS |  |
| --- | --- | --- | --- | --- |
| 1 | 0,655994 | 0,04 | 0,71561 |  |
| 2 | 0,655994 | 0,02 | -0,47352 |  |
| 3 | 0,729945 | 0,11 | -1,23886 |  |
| 4 | 0,729945 | 0,04 | -0,75164 |  |
| 5 | 0,476813 | 0,09 | -1,16212 |  |
| 6 | 0,476813 | 0,00 | -0,26105 |  |
| 7 | 0,688750 | 0,11 | -1,27600 |  |
| 8 | 0,688750 | 0,02 | -0,47363 |  |
| 9 | 0,580949 | 0,00 | 0,23820 |  |
| 10 | 0,580949 | 0,00 | 0,22347 |  |
| 11 | 0,425781 | 0,05 | -0,89715 |  |
| 12 | 0,425781 | 0,01 | 0,45380 |  |
| 13 | 0,569258 | 0,00 | -0,01555 |  |
| 14 | 0,569258 | 0,04 | 0,78101 |  |
| 15 | 0,433227 | 0,00 | -0,00017 |  |
| 16 | 0,433227 | 0,14 | -1,60092 |  |
| 17 | 0,597775 | 0,10 | 1,24349 |  |
| 18 | 0,597775 | 0,01 | 0,28518 |  |
| 19 | 0,407038 | 0,02 | -0,49899 |  |
| 20 | 0,647412 | 0,50 | 3,22706 | R |
| 21 | 0,407933 | 0,00 | -0,02587 |  |
| 22 | 0,817044 | 0,29 | 2,06371 |  |
| 23 | 0,823434 | 0,23 | 1,84884 |  |
| 24 | 0,713775 | 0,01 | -0,29396 |  |
| 25 | 0,288793 | 0,02 | -0,48165 |  |
| 26 | 0,288793 | 0,01 | -0,38661 |  |
| 27 | 0,288793 | 0,18 | 2,41760 | R |

R  Large residual

## Coded Coefficients

| Term | Coef | SE Coef | 95% CI | T-Value | P-Value | VIF |
| --- | --- | --- | --- | --- | --- | --- |
| Constant | 15,47 | 1,05 | (13,19; 17,75) | 14,78 | 0,000 |  |
| Lac | 3,453 | 0,865 | (1,567; 5,339) | 3,99 | 0,002 | 1,18 |
| HPMC\_Visc | 0,983 | 0,904 | (-0,987; 2,953) | 1,09 | 0,298 | 1,70 |
| HPMC\_HP | 0,840 | 0,885 | (-1,088; 2,767) | 0,95 | 0,361 | 1,26 |
| HPMC\_PS | 3,64 | 1,36 | (0,69; 6,60) | 2,68 | 0,020 | 2,09 |
| Lac\*Lac | 0,53 | 1,72 | (-3,22; 4,28) | 0,31 | 0,762 | 1,30 |
| HPMC\_Visc\*HPMC\_Visc | 2,81 | 1,83 | (-1,17; 6,80) | 1,54 | 0,150 | 1,96 |
| HPMC\_HP\*HPMC\_HP | 2,58 | 1,73 | (-1,20; 6,36) | 1,49 | 0,162 | 1,81 |
| HPMC\_PS\*HPMC\_PS | 0,61 | 1,72 | (-3,15; 4,37) | 0,35 | 0,730 | 1,42 |
| Lac\*HPMC\_Visc | -3,45 | 1,90 | (-7,58; 0,69) | -1,82 | 0,094 | 1,49 |
| Lac\*HPMC\_HP | 1,55 | 1,99 | (-2,78; 5,89) | 0,78 | 0,450 | 1,17 |
| Lac\*HPMC\_PS | -2,45 | 3,07 | (-9,14; 4,25) | -0,80 | 0,441 | 1,42 |
| HPMC\_Visc\*HPMC\_HP | -1,44 | 2,26 | (-6,37; 3,50) | -0,63 | 0,538 | 2,73 |
| HPMC\_Visc\*HPMC\_PS | 8,15 | 3,20 | (1,18; 15,12) | 2,55 | 0,026 | 2,74 |
| HPMC\_HP\*HPMC\_PS | -1,31 | 3,53 | (-9,01; 6,38) | -0,37 | 0,716 | 2,70 |

## Model Summary

| S | R-sq | R-sq(adj) | PRESS | R-sq(pred) | AICc | BIC |
| --- | --- | --- | --- | --- | --- | --- |
| 1,94845 | 76,18% | 48,40% | 209,919 | 0,00% | 177,15 | 143,48 |

## Analysis of Variance

| Source | DF | Seq SS | Contribution | Adj SS | Adj MS | F-Value | P-Value |
| --- | --- | --- | --- | --- | --- | --- | --- |
| Model | 14 | 145,722 | 76,18% | 145,722 | 10,4087 | 2,74 | 0,044 |
| Linear | 4 | 101,936 | 53,29% | 91,364 | 22,8410 | 6,02 | 0,007 |
| Lac | 1 | 83,920 | 43,87% | 60,440 | 60,4397 | 15,92 | 0,002 |
| HPMC\_Visc | 1 | 2,053 | 1,07% | 4,484 | 4,4839 | 1,18 | 0,298 |
| HPMC\_HP | 1 | 4,082 | 2,13% | 3,419 | 3,4186 | 0,90 | 0,361 |
| HPMC\_PS | 1 | 11,881 | 6,21% | 27,362 | 27,3625 | 7,21 | 0,020 |
| Square | 4 | 5,254 | 2,75% | 14,119 | 3,5296 | 0,93 | 0,479 |
| Lac\*Lac | 1 | 0,668 | 0,35% | 0,363 | 0,3635 | 0,10 | 0,762 |
| HPMC\_Visc\*HPMC\_Visc | 1 | 0,047 | 0,02% | 8,994 | 8,9944 | 2,37 | 0,150 |
| HPMC\_HP\*HPMC\_HP | 1 | 1,279 | 0,67% | 8,412 | 8,4117 | 2,22 | 0,162 |
| HPMC\_PS\*HPMC\_PS | 1 | 3,260 | 1,70% | 0,474 | 0,4744 | 0,12 | 0,730 |
| 2-Way Interaction | 6 | 38,532 | 20,14% | 38,532 | 6,4220 | 1,69 | 0,206 |
| Lac\*HPMC\_Visc | 1 | 9,777 | 5,11% | 12,535 | 12,5355 | 3,30 | 0,094 |
| Lac\*HPMC\_HP | 1 | 1,404 | 0,73% | 2,313 | 2,3132 | 0,61 | 0,450 |
| Lac\*HPMC\_PS | 1 | 2,412 | 1,26% | 2,412 | 2,4124 | 0,64 | 0,441 |
| HPMC\_Visc\*HPMC\_HP | 1 | 0,285 | 0,15% | 1,529 | 1,5294 | 0,40 | 0,538 |
| HPMC\_Visc\*HPMC\_PS | 1 | 24,128 | 12,61% | 24,654 | 24,6543 | 6,49 | 0,026 |
| HPMC\_HP\*HPMC\_PS | 1 | 0,527 | 0,28% | 0,527 | 0,5267 | 0,14 | 0,716 |
| Error | 12 | 45,558 | 23,82% | 45,558 | 3,7965 |  |  |
| Lack-of-Fit | 10 | 26,357 | 13,78% | 26,357 | 2,6357 | 0,27 | 0,935 |
| Pure Error | 2 | 19,201 | 10,04% | 19,201 | 9,6004 |  |  |
| Total | 26 | 191,280 | 100,00% |  |  |  |  |

## Regression Equation in Uncoded Units

|  |  |  |
| --- | --- | --- |
| F\_mean\_0.5h(30min) | = | 342 + 88 Lac - 0,01939 HPMC\_Visc - 32,5 HPMC\_HP - 2,59 HPMC\_PS + 8,5 Lac\*Lac + 0,000000 HPMC\_Visc\*HPMC\_Visc + 2,51 HPMC\_HP\*HPMC\_HP + 0,0113 HPMC\_PS\*HPMC\_PS - 0,00354 Lac\*HPMC\_Visc + 6,12 Lac\*HPMC\_HP - 1,33 Lac\*HPMC\_PS - 0,000364 HPMC\_Visc\*HPMC\_HP + 0,000285 HPMC\_Visc\*HPMC\_PS - 0,176 HPMC\_HP\*HPMC\_PS |

## Fits and Diagnostics for All Observations

| Obs | F\_mean\_0.5h(30min) | Fit | SE Fit | 95% CI | Resid | Std Resid | Del Resid | HI |
| --- | --- | --- | --- | --- | --- | --- | --- | --- |
| 1 | 14,63 | 13,97 | 1,58 | (10,53; 17,41) | 0,66 | 0,58 | 0,56 | 0,655994 |
| 2 | 18,93 | 19,36 | 1,58 | (15,93; 22,80) | -0,43 | -0,38 | -0,36 | 0,655994 |
| 3 | 11,94 | 12,80 | 1,66 | (9,18; 16,43) | -0,86 | -0,85 | -0,84 | 0,729945 |
| 4 | 13,74 | 14,13 | 1,66 | (10,50; 17,76) | -0,39 | -0,39 | -0,37 | 0,729945 |
| 5 | 12,09 | 13,76 | 1,35 | (10,83; 16,69) | -1,66 | -1,18 | -1,20 | 0,476813 |
| 6 | 19,64 | 20,19 | 1,35 | (17,26; 23,12) | -0,55 | -0,39 | -0,37 | 0,476813 |
| 7 | 12,63 | 13,53 | 1,62 | (10,01; 17,05) | -0,90 | -0,83 | -0,82 | 0,688750 |
| 8 | 15,81 | 16,23 | 1,62 | (12,70; 19,75) | -0,42 | -0,39 | -0,37 | 0,688750 |
| 9 | 14,26 | 13,98 | 1,49 | (10,75; 17,22) | 0,28 | 0,22 | 0,21 | 0,580949 |
| 10 | 17,96 | 17,75 | 1,49 | (14,51; 20,98) | 0,21 | 0,17 | 0,16 | 0,580949 |
| 11 | 15,26 | 16,69 | 1,27 | (13,92; 19,46) | -1,43 | -0,97 | -0,97 | 0,425781 |
| 12 | 18,99 | 18,23 | 1,27 | (15,46; 21,00) | 0,75 | 0,51 | 0,49 | 0,425781 |
| 13 | 13,54 | 13,68 | 1,47 | (10,47; 16,88) | -0,13 | -0,10 | -0,10 | 0,569258 |
| 14 | 19,88 | 18,91 | 1,47 | (15,70; 22,11) | 0,97 | 0,76 | 0,75 | 0,569258 |
| 15 | 16,78 | 16,78 | 1,28 | (13,99; 19,57) | 0,00 | 0,00 | 0,00 | 0,433227 |
| 16 | 16,33 | 18,87 | 1,28 | (16,08; 21,67) | -2,54 | -1,73 | -1,92 | 0,433227 |
| 17 | 12,91 | 11,69 | 1,51 | (8,41; 14,97) | 1,22 | 0,99 | 0,98 | 0,597775 |
| 18 | 20,29 | 19,89 | 1,51 | (16,61; 23,17) | 0,39 | 0,32 | 0,31 | 0,597775 |
| 19 | 15,88 | 16,74 | 1,24 | (14,03; 19,45) | -0,86 | -0,57 | -0,56 | 0,407038 |
| 20 | 20,13 | 17,78 | 1,57 | (14,36; 21,19) | 2,35 | 2,03 | 2,40 | 0,647412 |
| 21 | 16,41 | 16,51 | 1,24 | (13,80; 19,22) | -0,10 | -0,07 | -0,06 | 0,407933 |
| 22 | 19,93 | 19,06 | 1,76 | (15,22; 22,90) | 0,87 | 1,04 | 1,05 | 0,817044 |
| 23 | 15,03 | 14,32 | 1,77 | (10,46; 18,17) | 0,72 | 0,88 | 0,87 | 0,823434 |
| 24 | 17,37 | 17,55 | 1,65 | (13,97; 21,14) | -0,19 | -0,18 | -0,17 | 0,713775 |
| 25 | 14,09 | 15,26 | 1,05 | (12,98; 17,54) | -1,17 | -0,71 | -0,70 | 0,288793 |
| 26 | 14,22 | 15,26 | 1,05 | (12,98; 17,54) | -1,04 | -0,63 | -0,62 | 0,288793 |
| 27 | 19,52 | 15,26 | 1,05 | (12,98; 17,54) | 4,26 | 2,59 | 3,74 | 0,288793 |

| Obs | Cook’s D | DFITS |  |
| --- | --- | --- | --- |
| 1 | 0,04 | 0,77189 |  |
| 2 | 0,02 | -0,50200 |  |
| 3 | 0,13 | -1,38358 |  |
| 4 | 0,03 | -0,61393 |  |
| 5 | 0,08 | -1,14807 |  |
| 6 | 0,01 | -0,35798 |  |
| 7 | 0,10 | -1,21906 |  |
| 8 | 0,02 | -0,55531 |  |
| 9 | 0,00 | 0,25199 |  |
| 10 | 0,00 | 0,18862 |  |
| 11 | 0,05 | -0,83207 |  |
| 12 | 0,01 | 0,42444 |  |
| 13 | 0,00 | -0,11431 |  |
| 14 | 0,05 | 0,85650 |  |
| 15 | 0,00 | 0,00249 |  |
| 16 | 0,15 | -1,67476 |  |
| 17 | 0,10 | 1,20065 |  |
| 18 | 0,01 | 0,37396 |  |
| 19 | 0,02 | -0,46092 |  |
| 20 | 0,51 | 3,25294 | R |
| 21 | 0,00 | -0,05286 |  |
| 22 | 0,32 | 2,21508 |  |
| 23 | 0,24 | 1,87330 |  |
| 24 | 0,01 | -0,27588 |  |
| 25 | 0,01 | -0,44388 |  |
| 26 | 0,01 | -0,39283 |  |
| 27 | 0,18 | 2,38529 | R |

R  Large residual

## Coded Coefficients

| Term | Coef | SE Coef | 95% CI | T-Value | P-Value | VIF |
| --- | --- | --- | --- | --- | --- | --- |
| Constant | 18,31 | 1,11 | (15,89; 20,73) | 16,50 | 0,000 |  |
| Lac | 3,872 | 0,918 | (1,872; 5,872) | 4,22 | 0,001 | 1,18 |
| HPMC\_Visc | 0,901 | 0,959 | (-1,189; 2,991) | 0,94 | 0,366 | 1,70 |
| HPMC\_HP | 0,989 | 0,939 | (-1,056; 3,034) | 1,05 | 0,313 | 1,26 |
| HPMC\_PS | 3,90 | 1,44 | (0,76; 7,04) | 2,71 | 0,019 | 2,09 |
| Lac\*Lac | 0,51 | 1,82 | (-3,47; 4,48) | 0,28 | 0,786 | 1,30 |
| HPMC\_Visc\*HPMC\_Visc | 2,95 | 1,94 | (-1,28; 7,17) | 1,52 | 0,155 | 1,96 |
| HPMC\_HP\*HPMC\_HP | 2,82 | 1,84 | (-1,19; 6,83) | 1,53 | 0,152 | 1,81 |
| HPMC\_PS\*HPMC\_PS | 0,57 | 1,83 | (-3,41; 4,56) | 0,31 | 0,759 | 1,42 |
| Lac\*HPMC\_Visc | -3,66 | 2,01 | (-8,04; 0,73) | -1,82 | 0,094 | 1,49 |
| Lac\*HPMC\_HP | 1,61 | 2,11 | (-2,99; 6,22) | 0,76 | 0,460 | 1,17 |
| Lac\*HPMC\_PS | -2,48 | 3,26 | (-9,58; 4,62) | -0,76 | 0,462 | 1,42 |
| HPMC\_Visc\*HPMC\_HP | -1,57 | 2,40 | (-6,81; 3,66) | -0,65 | 0,525 | 2,73 |
| HPMC\_Visc\*HPMC\_PS | 8,54 | 3,39 | (1,14; 15,93) | 2,52 | 0,027 | 2,74 |
| HPMC\_HP\*HPMC\_PS | -1,35 | 3,74 | (-9,51; 6,81) | -0,36 | 0,724 | 2,70 |

## Model Summary

| S | R-sq | R-sq(adj) | PRESS | R-sq(pred) | AICc | BIC |
| --- | --- | --- | --- | --- | --- | --- |
| 2,06696 | 77,56% | 51,38% | 243,027 | 0,00% | 180,34 | 146,67 |

## Analysis of Variance

| Source | DF | Seq SS | Contribution | Adj SS | Adj MS | F-Value | P-Value |
| --- | --- | --- | --- | --- | --- | --- | --- |
| Model | 14 | 177,191 | 77,56% | 177,191 | 12,6565 | 2,96 | 0,033 |
| Linear | 4 | 128,036 | 56,04% | 113,189 | 28,2973 | 6,62 | 0,005 |
| Lac | 1 | 104,807 | 45,88% | 75,992 | 75,9918 | 17,79 | 0,001 |
| HPMC\_Visc | 1 | 3,514 | 1,54% | 3,772 | 3,7724 | 0,88 | 0,366 |
| HPMC\_HP | 1 | 5,623 | 2,46% | 4,743 | 4,7433 | 1,11 | 0,313 |
| HPMC\_PS | 1 | 14,092 | 6,17% | 31,378 | 31,3777 | 7,34 | 0,019 |
| Square | 4 | 6,586 | 2,88% | 16,405 | 4,1013 | 0,96 | 0,464 |
| Lac\*Lac | 1 | 0,867 | 0,38% | 0,330 | 0,3302 | 0,08 | 0,786 |
| HPMC\_Visc\*HPMC\_Visc | 1 | 0,053 | 0,02% | 9,854 | 9,8536 | 2,31 | 0,155 |
| HPMC\_HP\*HPMC\_HP | 1 | 1,829 | 0,80% | 10,007 | 10,0071 | 2,34 | 0,152 |
| HPMC\_PS\*HPMC\_PS | 1 | 3,837 | 1,68% | 0,422 | 0,4220 | 0,10 | 0,759 |
| 2-Way Interaction | 6 | 42,569 | 18,63% | 42,569 | 7,0949 | 1,66 | 0,214 |
| Lac\*HPMC\_Visc | 1 | 11,306 | 4,95% | 14,093 | 14,0933 | 3,30 | 0,094 |
| Lac\*HPMC\_HP | 1 | 1,537 | 0,67% | 2,493 | 2,4931 | 0,58 | 0,460 |
| Lac\*HPMC\_PS | 1 | 2,472 | 1,08% | 2,472 | 2,4715 | 0,58 | 0,462 |
| HPMC\_Visc\*HPMC\_HP | 1 | 0,208 | 0,09% | 1,830 | 1,8297 | 0,43 | 0,525 |
| HPMC\_Visc\*HPMC\_PS | 1 | 26,489 | 11,59% | 27,046 | 27,0459 | 6,33 | 0,027 |
| HPMC\_HP\*HPMC\_PS | 1 | 0,557 | 0,24% | 0,557 | 0,5567 | 0,13 | 0,724 |
| Error | 12 | 51,268 | 22,44% | 51,268 | 4,2723 |  |  |
| Lack-of-Fit | 10 | 30,397 | 13,31% | 30,397 | 3,0397 | 0,29 | 0,927 |
| Pure Error | 2 | 20,871 | 9,14% | 20,871 | 10,4357 |  |  |
| Total | 26 | 228,459 | 100,00% |  |  |  |  |

## Regression Equation in Uncoded Units

|  |  |  |
| --- | --- | --- |
| F\_mean\_0.75h(45min) | = | 364 + 92 Lac - 0,0202 HPMC\_Visc - 36,0 HPMC\_HP - 2,59 HPMC\_PS + 8,1 Lac\*Lac + 0,000000 HPMC\_Visc\*HPMC\_Visc + 2,73 HPMC\_HP\*HPMC\_HP + 0,0106 HPMC\_PS\*HPMC\_PS - 0,00376 Lac\*HPMC\_Visc + 6,36 Lac\*HPMC\_HP - 1,35 Lac\*HPMC\_PS - 0,000398 HPMC\_Visc\*HPMC\_HP + 0,000298 HPMC\_Visc\*HPMC\_PS - 0,181 HPMC\_HP\*HPMC\_PS |

## Fits and Diagnostics for All Observations

| Obs | F\_mean\_0.75h(45min) | Fit | SE Fit | 95% CI | Resid | Std Resid | Del Resid |
| --- | --- | --- | --- | --- | --- | --- | --- |
| 1 | 17,37 | 16,61 | 1,67 | (12,96; 20,25) | 0,76 | 0,63 | 0,61 |
| 2 | 21,99 | 22,53 | 1,67 | (18,88; 26,18) | -0,54 | -0,44 | -0,43 |
| 3 | 14,26 | 15,28 | 1,77 | (11,43; 19,13) | -1,02 | -0,95 | -0,95 |
| 4 | 16,50 | 16,85 | 1,77 | (13,01; 20,70) | -0,35 | -0,33 | -0,32 |
| 5 | 14,69 | 16,47 | 1,43 | (13,36; 19,58) | -1,79 | -1,19 | -1,22 |
| 6 | 22,71 | 23,48 | 1,43 | (20,37; 26,59) | -0,77 | -0,51 | -0,50 |
| 7 | 15,19 | 16,09 | 1,72 | (12,35; 19,83) | -0,90 | -0,78 | -0,77 |
| 8 | 18,62 | 19,10 | 1,72 | (15,36; 22,84) | -0,48 | -0,42 | -0,40 |
| 9 | 16,91 | 16,63 | 1,58 | (13,19; 20,06) | 0,28 | 0,21 | 0,20 |
| 10 | 21,10 | 20,90 | 1,58 | (17,47; 24,34) | 0,19 | 0,15 | 0,14 |
| 11 | 18,05 | 19,41 | 1,35 | (16,48; 22,35) | -1,36 | -0,87 | -0,86 |
| 12 | 22,10 | 21,30 | 1,35 | (18,36; 24,24) | 0,80 | 0,51 | 0,50 |
| 13 | 16,26 | 16,46 | 1,56 | (13,06; 19,86) | -0,20 | -0,15 | -0,14 |
| 14 | 23,41 | 22,27 | 1,56 | (18,87; 25,66) | 1,14 | 0,84 | 0,83 |
| 15 | 19,60 | 19,54 | 1,36 | (16,58; 22,51) | 0,05 | 0,04 | 0,03 |
| 16 | 19,19 | 22,00 | 1,36 | (19,03; 24,96) | -2,80 | -1,80 | -2,02 |
| 17 | 15,27 | 14,06 | 1,60 | (10,57; 17,54) | 1,21 | 0,93 | 0,92 |
| 18 | 23,70 | 23,17 | 1,60 | (19,69; 26,65) | 0,53 | 0,40 | 0,39 |
| 19 | 18,97 | 19,77 | 1,32 | (16,89; 22,64) | -0,80 | -0,50 | -0,48 |
| 20 | 23,06 | 20,58 | 1,66 | (16,96; 24,21) | 2,48 | 2,02 | 2,38 |
| 21 | 19,30 | 19,41 | 1,32 | (16,53; 22,28) | -0,11 | -0,07 | -0,07 |
| 22 | 23,27 | 22,32 | 1,87 | (18,25; 26,39) | 0,94 | 1,07 | 1,07 |
| 23 | 17,80 | 16,98 | 1,88 | (12,89; 21,07) | 0,82 | 0,94 | 0,93 |
| 24 | 20,28 | 20,53 | 1,75 | (16,72; 24,33) | -0,25 | -0,22 | -0,21 |
| 25 | 16,88 | 18,11 | 1,11 | (15,69; 20,53) | -1,23 | -0,70 | -0,69 |
| 26 | 17,04 | 18,11 | 1,11 | (15,69; 20,53) | -1,07 | -0,61 | -0,60 |
| 27 | 22,55 | 18,11 | 1,11 | (15,69; 20,53) | 4,45 | 2,55 | 3,61 |

| Obs | HI | Cook’s D | DFITS |  |
| --- | --- | --- | --- | --- |
| 1 | 0,655994 | 0,05 | 0,84549 |  |
| 2 | 0,655994 | 0,02 | -0,58991 |  |
| 3 | 0,729945 | 0,16 | -1,55801 |  |
| 4 | 0,729945 | 0,02 | -0,52108 |  |
| 5 | 0,476813 | 0,09 | -1,16348 |  |
| 6 | 0,476813 | 0,02 | -0,47507 |  |
| 7 | 0,688750 | 0,09 | -1,14428 |  |
| 8 | 0,688750 | 0,03 | -0,60178 |  |
| 9 | 0,580949 | 0,00 | 0,23502 |  |
| 10 | 0,580949 | 0,00 | 0,16417 |  |
| 11 | 0,425781 | 0,04 | -0,73893 |  |
| 12 | 0,425781 | 0,01 | 0,42685 |  |
| 13 | 0,569258 | 0,00 | -0,16145 |  |
| 14 | 0,569258 | 0,06 | 0,95770 |  |
| 15 | 0,433227 | 0,00 | 0,02934 |  |
| 16 | 0,433227 | 0,17 | -1,76692 |  |
| 17 | 0,597775 | 0,09 | 1,12211 |  |
| 18 | 0,597775 | 0,02 | 0,47565 |  |
| 19 | 0,407038 | 0,01 | -0,40133 |  |
| 20 | 0,647412 | 0,50 | 3,22280 | R |
| 21 | 0,407933 | 0,00 | -0,05467 |  |
| 22 | 0,817044 | 0,34 | 2,26757 |  |
| 23 | 0,823434 | 0,27 | 2,01637 |  |
| 24 | 0,713775 | 0,01 | -0,33851 |  |
| 25 | 0,288793 | 0,01 | -0,43839 |  |
| 26 | 0,288793 | 0,01 | -0,37970 |  |
| 27 | 0,288793 | 0,18 | 2,30023 | R |

R  Large residual

## Coded Coefficients

| Term | Coef | SE Coef | 95% CI | T-Value | P-Value | VIF |
| --- | --- | --- | --- | --- | --- | --- |
| Constant | 20,77 | 1,15 | (18,25; 23,28) | 18,01 | 0,000 |  |
| Lac | 4,204 | 0,953 | (2,127; 6,282) | 4,41 | 0,001 | 1,18 |
| HPMC\_Visc | 0,786 | 0,996 | (-1,384; 2,957) | 0,79 | 0,445 | 1,70 |
| HPMC\_HP | 1,080 | 0,975 | (-1,043; 3,204) | 1,11 | 0,289 | 1,26 |
| HPMC\_PS | 3,99 | 1,49 | (0,74; 7,25) | 2,67 | 0,020 | 2,09 |
| Lac\*Lac | 0,49 | 1,89 | (-3,64; 4,62) | 0,26 | 0,801 | 1,30 |
| HPMC\_Visc\*HPMC\_Visc | 2,98 | 2,01 | (-1,40; 7,37) | 1,48 | 0,164 | 1,96 |
| HPMC\_HP\*HPMC\_HP | 2,98 | 1,91 | (-1,18; 7,14) | 1,56 | 0,145 | 1,81 |
| HPMC\_PS\*HPMC\_PS | 0,56 | 1,90 | (-3,58; 4,70) | 0,30 | 0,772 | 1,42 |
| Lac\*HPMC\_Visc | -3,90 | 2,09 | (-8,45; 0,66) | -1,86 | 0,087 | 1,49 |
| Lac\*HPMC\_HP | 1,65 | 2,19 | (-3,13; 6,43) | 0,75 | 0,467 | 1,17 |
| Lac\*HPMC\_PS | -2,59 | 3,39 | (-9,97; 4,79) | -0,77 | 0,459 | 1,42 |
| HPMC\_Visc\*HPMC\_HP | -1,64 | 2,49 | (-7,07; 3,80) | -0,66 | 0,524 | 2,73 |
| HPMC\_Visc\*HPMC\_PS | 8,71 | 3,52 | (1,03; 16,38) | 2,47 | 0,029 | 2,74 |
| HPMC\_HP\*HPMC\_PS | -1,37 | 3,89 | (-9,85; 7,10) | -0,35 | 0,730 | 2,70 |

## Model Summary

| S | R-sq | R-sq(adj) | PRESS | R-sq(pred) | AICc | BIC |
| --- | --- | --- | --- | --- | --- | --- |
| 2,14646 | 78,62% | 53,69% | 270,961 | 0,00% | 182,37 | 148,71 |

## Analysis of Variance

| Source | DF | Seq SS | Contribution | Adj SS | Adj MS | F-Value | P-Value |
| --- | --- | --- | --- | --- | --- | --- | --- |
| Model | 14 | 203,363 | 78,62% | 203,363 | 14,5259 | 3,15 | 0,027 |
| Linear | 4 | 150,228 | 58,08% | 130,435 | 32,6087 | 7,08 | 0,004 |
| Lac | 1 | 123,731 | 47,84% | 89,601 | 89,6013 | 19,45 | 0,001 |
| HPMC\_Visc | 1 | 5,152 | 1,99% | 2,871 | 2,8709 | 0,62 | 0,445 |
| HPMC\_HP | 1 | 6,535 | 2,53% | 5,659 | 5,6589 | 1,23 | 0,289 |
| HPMC\_PS | 1 | 14,811 | 5,73% | 32,909 | 32,9086 | 7,14 | 0,020 |
| Square | 4 | 7,518 | 2,91% | 17,781 | 4,4452 | 0,96 | 0,462 |
| Lac\*Lac | 1 | 1,005 | 0,39% | 0,307 | 0,3075 | 0,07 | 0,801 |
| HPMC\_Visc\*HPMC\_Visc | 1 | 0,074 | 0,03% | 10,111 | 10,1112 | 2,19 | 0,164 |
| HPMC\_HP\*HPMC\_HP | 1 | 2,349 | 0,91% | 11,209 | 11,2093 | 2,43 | 0,145 |
| HPMC\_PS\*HPMC\_PS | 1 | 4,090 | 1,58% | 0,404 | 0,4044 | 0,09 | 0,772 |
| 2-Way Interaction | 6 | 45,617 | 17,64% | 45,617 | 7,6029 | 1,65 | 0,217 |
| Lac\*HPMC\_Visc | 1 | 13,019 | 5,03% | 16,006 | 16,0064 | 3,47 | 0,087 |
| Lac\*HPMC\_HP | 1 | 1,580 | 0,61% | 2,600 | 2,5996 | 0,56 | 0,467 |
| Lac\*HPMC\_PS | 1 | 2,697 | 1,04% | 2,697 | 2,6967 | 0,59 | 0,459 |
| HPMC\_Visc\*HPMC\_HP | 1 | 0,177 | 0,07% | 1,980 | 1,9801 | 0,43 | 0,524 |
| HPMC\_Visc\*HPMC\_PS | 1 | 27,570 | 10,66% | 28,144 | 28,1441 | 6,11 | 0,029 |
| HPMC\_HP\*HPMC\_PS | 1 | 0,574 | 0,22% | 0,574 | 0,5742 | 0,12 | 0,730 |
| Error | 12 | 55,287 | 21,38% | 55,287 | 4,6073 |  |  |
| Lack-of-Fit | 10 | 33,992 | 13,14% | 33,992 | 3,3992 | 0,32 | 0,912 |
| Pure Error | 2 | 21,296 | 8,23% | 21,296 | 10,6478 |  |  |
| Total | 26 | 258,650 | 100,00% |  |  |  |  |

## Regression Equation in Uncoded Units

|  |  |  |
| --- | --- | --- |
| F\_mean\_1h(60min) | = | 377 + 100 Lac - 0,0204 HPMC\_Visc - 38,6 HPMC\_HP - 2,57 HPMC\_PS + 7,8 Lac\*Lac + 0,000000 HPMC\_Visc\*HPMC\_Visc + 2,89 HPMC\_HP\*HPMC\_HP + 0,0104 HPMC\_PS\*HPMC\_PS - 0,00401 Lac\*HPMC\_Visc + 6,49 Lac\*HPMC\_HP - 1,41 Lac\*HPMC\_PS - 0,000414 HPMC\_Visc\*HPMC\_HP + 0,000304 HPMC\_Visc\*HPMC\_PS - 0,184 HPMC\_HP\*HPMC\_PS |

## Fits and Diagnostics for All Observations

| Obs | F\_mean\_1h(60min) | Fit | SE Fit | 95% CI | Resid | Std Resid | Del Resid | HI |
| --- | --- | --- | --- | --- | --- | --- | --- | --- |
| 1 | 19,76 | 18,89 | 1,74 | (15,10; 22,68) | 0,87 | 0,69 | 0,68 | 0,655994 |
| 2 | 24,65 | 25,32 | 1,74 | (21,53; 29,11) | -0,67 | -0,53 | -0,51 | 0,655994 |
| 3 | 16,35 | 17,49 | 1,83 | (13,49; 21,48) | -1,14 | -1,02 | -1,02 | 0,729945 |
| 4 | 18,94 | 19,26 | 1,83 | (15,27; 23,26) | -0,33 | -0,29 | -0,28 | 0,729945 |
| 5 | 16,86 | 18,79 | 1,48 | (15,56; 22,02) | -1,94 | -1,25 | -1,28 | 0,476813 |
| 6 | 25,37 | 26,31 | 1,48 | (23,08; 29,54) | -0,93 | -0,60 | -0,59 | 0,476813 |
| 7 | 17,43 | 18,34 | 1,78 | (14,46; 22,22) | -0,92 | -0,76 | -0,75 | 0,688750 |
| 8 | 21,05 | 21,57 | 1,78 | (17,69; 25,45) | -0,52 | -0,44 | -0,42 | 0,688750 |
| 9 | 19,15 | 18,94 | 1,64 | (15,37; 22,50) | 0,21 | 0,15 | 0,15 | 0,580949 |
| 10 | 23,86 | 23,64 | 1,64 | (20,08; 27,21) | 0,22 | 0,16 | 0,15 | 0,580949 |
| 11 | 20,44 | 21,74 | 1,40 | (18,69; 24,80) | -1,30 | -0,80 | -0,79 | 0,425781 |
| 12 | 24,69 | 23,88 | 1,40 | (20,82; 26,93) | 0,81 | 0,50 | 0,48 | 0,425781 |
| 13 | 18,61 | 18,84 | 1,62 | (15,31; 22,37) | -0,23 | -0,17 | -0,16 | 0,569258 |
| 14 | 26,32 | 25,10 | 1,62 | (21,57; 28,63) | 1,21 | 0,86 | 0,85 | 0,569258 |
| 15 | 21,98 | 21,89 | 1,41 | (18,81; 24,97) | 0,09 | 0,06 | 0,05 | 0,433227 |
| 16 | 21,58 | 24,59 | 1,41 | (21,51; 27,67) | -3,01 | -1,86 | -2,12 | 0,433227 |
| 17 | 17,36 | 16,13 | 1,66 | (12,51; 19,74) | 1,23 | 0,90 | 0,90 | 0,597775 |
| 18 | 26,66 | 26,00 | 1,66 | (22,38; 29,61) | 0,66 | 0,49 | 0,47 | 0,597775 |
| 19 | 21,68 | 22,35 | 1,37 | (19,37; 25,34) | -0,68 | -0,41 | -0,39 | 0,407038 |
| 20 | 25,49 | 22,92 | 1,73 | (19,16; 26,68) | 2,57 | 2,02 | 2,38 | 0,647412 |
| 21 | 21,83 | 21,93 | 1,37 | (18,95; 24,92) | -0,10 | -0,06 | -0,06 | 0,407933 |
| 22 | 26,07 | 25,05 | 1,94 | (20,83; 29,28) | 1,02 | 1,11 | 1,12 | 0,817044 |
| 23 | 20,28 | 19,39 | 1,95 | (15,15; 23,64) | 0,89 | 0,98 | 0,98 | 0,823434 |
| 24 | 22,77 | 23,04 | 1,81 | (19,09; 26,99) | -0,27 | -0,23 | -0,22 | 0,713775 |
| 25 | 19,37 | 20,57 | 1,15 | (18,06; 23,09) | -1,20 | -0,67 | -0,65 | 0,288793 |
| 26 | 19,51 | 20,57 | 1,15 | (18,06; 23,09) | -1,07 | -0,59 | -0,57 | 0,288793 |
| 27 | 25,09 | 20,57 | 1,15 | (18,06; 23,09) | 4,51 | 2,49 | 3,44 | 0,288793 |

| Obs | Cook’s D | DFITS |  |
| --- | --- | --- | --- |
| 1 | 0,06 | 0,93241 |  |
| 2 | 0,04 | -0,70989 |  |
| 3 | 0,19 | -1,67608 |  |
| 4 | 0,02 | -0,46256 |  |
| 5 | 0,09 | -1,22189 |  |
| 6 | 0,02 | -0,55879 |  |
| 7 | 0,09 | -1,11658 |  |
| 8 | 0,03 | -0,62735 |  |
| 9 | 0,00 | 0,17098 |  |
| 10 | 0,00 | 0,17729 |  |
| 11 | 0,03 | -0,67741 |  |
| 12 | 0,01 | 0,41579 |  |
| 13 | 0,00 | -0,18309 |  |
| 14 | 0,07 | 0,97925 |  |
| 15 | 0,00 | 0,04630 |  |
| 16 | 0,18 | -1,85153 |  |
| 17 | 0,08 | 1,09174 |  |
| 18 | 0,02 | 0,57501 |  |
| 19 | 0,01 | -0,32623 |  |
| 20 | 0,50 | 3,21971 | R |
| 21 | 0,00 | -0,04787 |  |
| 22 | 0,37 | 2,37153 |  |
| 23 | 0,30 | 2,11971 |  |
| 24 | 0,01 | -0,35195 |  |
| 25 | 0,01 | -0,41381 |  |
| 26 | 0,01 | -0,36474 |  |
| 27 | 0,17 | 2,19279 | R |

R  Large residual

## Coded Coefficients

| Term | Coef | SE Coef | 95% CI | T-Value | P-Value | VIF |
| --- | --- | --- | --- | --- | --- | --- |
| Constant | 25,01 | 1,22 | (22,36; 27,66) | 20,55 | 0,000 |  |
| Lac | 4,74 | 1,01 | (2,54; 6,93) | 4,71 | 0,001 | 1,18 |
| HPMC\_Visc | 0,59 | 1,05 | (-1,70; 2,89) | 0,57 | 0,582 | 1,70 |
| HPMC\_HP | 1,18 | 1,03 | (-1,06; 3,42) | 1,15 | 0,274 | 1,26 |
| HPMC\_PS | 4,15 | 1,58 | (0,71; 7,58) | 2,63 | 0,022 | 2,09 |
| Lac\*Lac | 0,50 | 2,00 | (-3,86; 4,86) | 0,25 | 0,807 | 1,30 |
| HPMC\_Visc\*HPMC\_Visc | 3,13 | 2,13 | (-1,50; 7,77) | 1,47 | 0,166 | 1,96 |
| HPMC\_HP\*HPMC\_HP | 3,17 | 2,02 | (-1,22; 7,57) | 1,57 | 0,142 | 1,81 |
| HPMC\_PS\*HPMC\_PS | 0,61 | 2,00 | (-3,76; 4,98) | 0,31 | 0,765 | 1,42 |
| Lac\*HPMC\_Visc | -4,11 | 2,21 | (-8,91; 0,70) | -1,86 | 0,087 | 1,49 |
| Lac\*HPMC\_HP | 1,62 | 2,32 | (-3,42; 6,67) | 0,70 | 0,496 | 1,17 |
| Lac\*HPMC\_PS | -2,58 | 3,57 | (-10,36; 5,21) | -0,72 | 0,485 | 1,42 |
| HPMC\_Visc\*HPMC\_HP | -1,77 | 2,63 | (-7,51; 3,96) | -0,67 | 0,513 | 2,73 |
| HPMC\_Visc\*HPMC\_PS | 9,05 | 3,72 | (0,95; 17,16) | 2,43 | 0,031 | 2,74 |
| HPMC\_HP\*HPMC\_PS | -1,52 | 4,11 | (-10,46; 7,42) | -0,37 | 0,717 | 2,70 |

## Model Summary

| S | R-sq | R-sq(adj) | PRESS | R-sq(pred) | AICc | BIC |
| --- | --- | --- | --- | --- | --- | --- |
| 2,26569 | 80,03% | 56,73% | 315,601 | 0,00% | 185,29 | 151,63 |

## Analysis of Variance

| Source | DF | Seq SS | Contribution | Adj SS | Adj MS | F-Value | P-Value |
| --- | --- | --- | --- | --- | --- | --- | --- |
| Model | 14 | 246,819 | 80,03% | 246,819 | 17,630 | 3,43 | 0,019 |
| Linear | 4 | 188,776 | 61,21% | 160,834 | 40,208 | 7,83 | 0,002 |
| Lac | 1 | 156,277 | 50,67% | 113,657 | 113,657 | 22,14 | 0,001 |
| HPMC\_Visc | 1 | 8,715 | 2,83% | 1,643 | 1,643 | 0,32 | 0,582 |
| HPMC\_HP | 1 | 7,760 | 2,52% | 6,747 | 6,747 | 1,31 | 0,274 |
| HPMC\_PS | 1 | 16,024 | 5,20% | 35,471 | 35,471 | 6,91 | 0,022 |
| Square | 4 | 8,293 | 2,69% | 19,919 | 4,980 | 0,97 | 0,459 |
| Lac\*Lac | 1 | 1,191 | 0,39% | 0,319 | 0,319 | 0,06 | 0,807 |
| HPMC\_Visc\*HPMC\_Visc | 1 | 0,064 | 0,02% | 11,154 | 11,154 | 2,17 | 0,166 |
| HPMC\_HP\*HPMC\_HP | 1 | 2,777 | 0,90% | 12,712 | 12,712 | 2,48 | 0,142 |
| HPMC\_PS\*HPMC\_PS | 1 | 4,260 | 1,38% | 0,479 | 0,479 | 0,09 | 0,765 |
| 2-Way Interaction | 6 | 49,750 | 16,13% | 49,750 | 8,292 | 1,62 | 0,226 |
| Lac\*HPMC\_Visc | 1 | 14,966 | 4,85% | 17,782 | 17,782 | 3,46 | 0,087 |
| Lac\*HPMC\_HP | 1 | 1,527 | 0,50% | 2,527 | 2,527 | 0,49 | 0,496 |
| Lac\*HPMC\_PS | 1 | 2,670 | 0,87% | 2,670 | 2,670 | 0,52 | 0,485 |
| HPMC\_Visc\*HPMC\_HP | 1 | 0,157 | 0,05% | 2,329 | 2,329 | 0,45 | 0,513 |
| HPMC\_Visc\*HPMC\_PS | 1 | 29,726 | 9,64% | 30,429 | 30,429 | 5,93 | 0,031 |
| HPMC\_HP\*HPMC\_PS | 1 | 0,705 | 0,23% | 0,705 | 0,705 | 0,14 | 0,717 |
| Error | 12 | 61,600 | 19,97% | 61,600 | 5,133 |  |  |
| Lack-of-Fit | 10 | 39,189 | 12,71% | 39,189 | 3,919 | 0,35 | 0,896 |
| Pure Error | 2 | 22,411 | 7,27% | 22,411 | 11,206 |  |  |
| Total | 26 | 308,419 | 100,00% |  |  |  |  |

## Regression Equation in Uncoded Units

|  |  |  |
| --- | --- | --- |
| F\_mean\_1.5h(90min) | = | 393 + 105 Lac - 0,0211 HPMC\_Visc - 40,2 HPMC\_HP - 2,66 HPMC\_PS + 8,0 Lac\*Lac + 0,000000 HPMC\_Visc\*HPMC\_Visc + 3,08 HPMC\_HP\*HPMC\_HP + 0,0113 HPMC\_PS\*HPMC\_PS - 0,00422 Lac\*HPMC\_Visc + 6,40 Lac\*HPMC\_HP - 1,40 Lac\*HPMC\_PS - 0,000449 HPMC\_Visc\*HPMC\_HP + 0,000316 HPMC\_Visc\*HPMC\_PS - 0,204 HPMC\_HP\*HPMC\_PS |

## Fits and Diagnostics for All Observations

| Obs | F\_mean\_1.5h(90min) | Fit | SE Fit | 95% CI | Resid | Std Resid | Del Resid | HI |
| --- | --- | --- | --- | --- | --- | --- | --- | --- |
| 1 | 23,96 | 22,96 | 1,84 | (18,96; 26,96) | 1,00 | 0,75 | 0,74 | 0,655994 |
| 2 | 29,24 | 30,09 | 1,84 | (26,09; 34,09) | -0,84 | -0,64 | -0,62 | 0,655994 |
| 3 | 20,03 | 21,31 | 1,94 | (17,09; 25,53) | -1,28 | -1,09 | -1,09 | 0,729945 |
| 4 | 23,26 | 23,48 | 1,94 | (19,26; 27,70) | -0,23 | -0,19 | -0,18 | 0,729945 |
| 5 | 20,81 | 22,96 | 1,56 | (19,55; 26,37) | -2,15 | -1,31 | -1,36 | 0,476813 |
| 6 | 29,98 | 31,14 | 1,56 | (27,73; 34,55) | -1,16 | -0,71 | -0,69 | 0,476813 |
| 7 | 21,35 | 22,27 | 1,88 | (18,18; 26,37) | -0,92 | -0,73 | -0,71 | 0,688750 |
| 8 | 25,21 | 25,87 | 1,88 | (21,77; 29,96) | -0,66 | -0,52 | -0,50 | 0,688750 |
| 9 | 23,22 | 23,01 | 1,73 | (19,25; 26,77) | 0,21 | 0,14 | 0,14 | 0,580949 |
| 10 | 28,65 | 28,41 | 1,73 | (24,65; 32,18) | 0,24 | 0,16 | 0,16 | 0,580949 |
| 11 | 24,52 | 25,78 | 1,48 | (22,56; 29,00) | -1,26 | -0,74 | -0,72 | 0,425781 |
| 12 | 29,21 | 28,42 | 1,48 | (25,20; 31,64) | 0,79 | 0,46 | 0,45 | 0,425781 |
| 13 | 22,64 | 23,01 | 1,71 | (19,28; 26,73) | -0,36 | -0,24 | -0,24 | 0,569258 |
| 14 | 31,22 | 29,94 | 1,71 | (26,22; 33,67) | 1,28 | 0,86 | 0,85 | 0,569258 |
| 15 | 26,10 | 25,94 | 1,49 | (22,69; 29,19) | 0,16 | 0,09 | 0,09 | 0,433227 |
| 16 | 25,89 | 29,11 | 1,49 | (25,86; 32,36) | -3,22 | -1,89 | -2,16 | 0,433227 |
| 17 | 21,09 | 19,84 | 1,75 | (16,03; 23,66) | 1,25 | 0,87 | 0,86 | 0,597775 |
| 18 | 31,71 | 30,86 | 1,75 | (27,04; 34,67) | 0,85 | 0,59 | 0,58 | 0,597775 |
| 19 | 26,44 | 26,91 | 1,45 | (23,76; 30,06) | -0,47 | -0,27 | -0,26 | 0,407038 |
| 20 | 29,74 | 27,06 | 1,82 | (23,09; 31,04) | 2,68 | 1,99 | 2,33 | 0,647412 |
| 21 | 26,10 | 26,26 | 1,45 | (23,11; 29,42) | -0,16 | -0,09 | -0,09 | 0,407933 |
| 22 | 30,81 | 29,63 | 2,05 | (25,17; 34,09) | 1,18 | 1,21 | 1,24 | 0,817044 |
| 23 | 24,65 | 23,67 | 2,06 | (19,19; 28,15) | 0,98 | 1,03 | 1,03 | 0,823434 |
| 24 | 27,18 | 27,47 | 1,91 | (23,29; 31,64) | -0,29 | -0,24 | -0,23 | 0,713775 |
| 25 | 23,64 | 24,85 | 1,22 | (22,20; 27,50) | -1,21 | -0,63 | -0,62 | 0,288793 |
| 26 | 23,79 | 24,85 | 1,22 | (22,20; 27,50) | -1,06 | -0,55 | -0,54 | 0,288793 |
| 27 | 29,51 | 24,85 | 1,22 | (22,20; 27,50) | 4,66 | 2,44 | 3,29 | 0,288793 |

| Obs | Cook’s D | DFITS |  |
| --- | --- | --- | --- |
| 1 | 0,07 | 1,01660 |  |
| 2 | 0,05 | -0,85520 |  |
| 3 | 0,21 | -1,79870 |  |
| 4 | 0,01 | -0,30243 |  |
| 5 | 0,10 | -1,29542 |  |
| 6 | 0,03 | -0,66223 |  |
| 7 | 0,08 | -1,05852 |  |
| 8 | 0,04 | -0,75075 |  |
| 9 | 0,00 | 0,16289 |  |
| 10 | 0,00 | 0,18334 |  |
| 11 | 0,03 | -0,62022 |  |
| 12 | 0,01 | 0,38330 |  |
| 13 | 0,01 | -0,27024 |  |
| 14 | 0,07 | 0,97610 |  |
| 15 | 0,00 | 0,07626 |  |
| 16 | 0,18 | -1,88594 |  |
| 17 | 0,08 | 1,05083 |  |
| 18 | 0,03 | 0,70197 |  |
| 19 | 0,00 | -0,21353 |  |
| 20 | 0,49 | 3,16082 |  |
| 21 | 0,00 | -0,07393 |  |
| 22 | 0,44 | 2,62443 |  |
| 23 | 0,33 | 2,22428 |  |
| 24 | 0,01 | -0,35955 |  |
| 25 | 0,01 | -0,39292 |  |
| 26 | 0,01 | -0,34280 |  |
| 27 | 0,16 | 2,09679 | R |

R  Large residual

## Coded Coefficients

| Term | Coef | SE Coef | 95% CI | T-Value | P-Value | VIF |
| --- | --- | --- | --- | --- | --- | --- |
| Constant | 28,78 | 1,26 | (26,03; 31,52) | 22,86 | 0,000 |  |
| Lac | 5,18 | 1,04 | (2,91; 7,45) | 4,97 | 0,000 | 1,18 |
| HPMC\_Visc | 0,47 | 1,09 | (-1,90; 2,84) | 0,44 | 0,670 | 1,70 |
| HPMC\_HP | 1,26 | 1,06 | (-1,06; 3,58) | 1,18 | 0,259 | 1,26 |
| HPMC\_PS | 4,24 | 1,63 | (0,68; 7,79) | 2,60 | 0,023 | 2,09 |
| Lac\*Lac | 0,45 | 2,07 | (-4,06; 4,96) | 0,22 | 0,832 | 1,30 |
| HPMC\_Visc\*HPMC\_Visc | 3,21 | 2,20 | (-1,58; 8,00) | 1,46 | 0,170 | 1,96 |
| HPMC\_HP\*HPMC\_HP | 3,29 | 2,09 | (-1,26; 7,84) | 1,58 | 0,141 | 1,81 |
| HPMC\_PS\*HPMC\_PS | 0,68 | 2,07 | (-3,84; 5,20) | 0,33 | 0,748 | 1,42 |
| Lac\*HPMC\_Visc | -4,27 | 2,28 | (-9,24; 0,70) | -1,87 | 0,086 | 1,49 |
| Lac\*HPMC\_HP | 1,60 | 2,40 | (-3,62; 6,82) | 0,67 | 0,517 | 1,17 |
| Lac\*HPMC\_PS | -2,54 | 3,70 | (-10,60; 5,51) | -0,69 | 0,504 | 1,42 |
| HPMC\_Visc\*HPMC\_HP | -1,81 | 2,72 | (-7,74; 4,13) | -0,66 | 0,520 | 2,73 |
| HPMC\_Visc\*HPMC\_PS | 9,32 | 3,85 | (0,93; 17,70) | 2,42 | 0,032 | 2,74 |
| HPMC\_HP\*HPMC\_PS | -1,63 | 4,25 | (-10,88; 7,63) | -0,38 | 0,708 | 2,70 |

## Model Summary

| S | R-sq | R-sq(adj) | PRESS | R-sq(pred) | AICc | BIC |
| --- | --- | --- | --- | --- | --- | --- |
| 2,34392 | 81,24% | 59,34% | 351,232 | 0,03% | 187,13 | 153,46 |

## Analysis of Variance

| Source | DF | Seq SS | Contribution | Adj SS | Adj MS | F-Value | P-Value |
| --- | --- | --- | --- | --- | --- | --- | --- |
| Model | 14 | 285,411 | 81,24% | 285,411 | 20,387 | 3,71 | 0,014 |
| Linear | 4 | 223,404 | 63,59% | 187,733 | 46,933 | 8,54 | 0,002 |
| Lac | 1 | 186,326 | 53,03% | 135,951 | 135,951 | 24,75 | 0,000 |
| HPMC\_Visc | 1 | 11,625 | 3,31% | 1,045 | 1,045 | 0,19 | 0,670 |
| HPMC\_HP | 1 | 8,789 | 2,50% | 7,701 | 7,701 | 1,40 | 0,259 |
| HPMC\_PS | 1 | 16,663 | 4,74% | 37,045 | 37,045 | 6,74 | 0,023 |
| Square | 4 | 8,844 | 2,52% | 21,269 | 5,317 | 0,97 | 0,460 |
| Lac\*Lac | 1 | 1,503 | 0,43% | 0,257 | 0,257 | 0,05 | 0,832 |
| HPMC\_Visc\*HPMC\_Visc | 1 | 0,084 | 0,02% | 11,724 | 11,724 | 2,13 | 0,170 |
| HPMC\_HP\*HPMC\_HP | 1 | 2,983 | 0,85% | 13,657 | 13,657 | 2,49 | 0,141 |
| HPMC\_PS\*HPMC\_PS | 1 | 4,273 | 1,22% | 0,592 | 0,592 | 0,11 | 0,748 |
| 2-Way Interaction | 6 | 53,163 | 15,13% | 53,163 | 8,861 | 1,61 | 0,226 |
| Lac\*HPMC\_Visc | 1 | 16,651 | 4,74% | 19,231 | 19,231 | 3,50 | 0,086 |
| Lac\*HPMC\_HP | 1 | 1,474 | 0,42% | 2,446 | 2,446 | 0,45 | 0,517 |
| Lac\*HPMC\_PS | 1 | 2,604 | 0,74% | 2,604 | 2,604 | 0,47 | 0,504 |
| HPMC\_Visc\*HPMC\_HP | 1 | 0,219 | 0,06% | 2,416 | 2,416 | 0,44 | 0,520 |
| HPMC\_Visc\*HPMC\_PS | 1 | 31,408 | 8,94% | 32,209 | 32,209 | 5,86 | 0,032 |
| HPMC\_HP\*HPMC\_PS | 1 | 0,807 | 0,23% | 0,807 | 0,807 | 0,15 | 0,708 |
| Error | 12 | 65,927 | 18,76% | 65,927 | 5,494 |  |  |
| Lack-of-Fit | 10 | 43,568 | 12,40% | 43,568 | 4,357 | 0,39 | 0,874 |
| Pure Error | 2 | 22,359 | 6,36% | 22,359 | 11,180 |  |  |
| Total | 26 | 351,339 | 100,00% |  |  |  |  |

## Regression Equation in Uncoded Units

|  |  |  |
| --- | --- | --- |
| F\_mean\_2h(120min) | = | 409 + 110 Lac - 0,0218 HPMC\_Visc - 41,1 HPMC\_HP - 2,82 HPMC\_PS + 7,2 Lac\*Lac + 0,000000 HPMC\_Visc\*HPMC\_Visc + 3,19 HPMC\_HP\*HPMC\_HP + 0,0126 HPMC\_PS\*HPMC\_PS - 0,00439 Lac\*HPMC\_Visc + 6,30 Lac\*HPMC\_HP - 1,38 Lac\*HPMC\_PS - 0,000458 HPMC\_Visc\*HPMC\_HP + 0,000326 HPMC\_Visc\*HPMC\_PS - 0,218 HPMC\_HP\*HPMC\_PS |

## Fits and Diagnostics for All Observations

| Obs | F\_mean\_2h(120min) | Fit | SE Fit | 95% CI | Resid | Std Resid | Del Resid | HI |
| --- | --- | --- | --- | --- | --- | --- | --- | --- |
| 1 | 27,65 | 26,55 | 1,90 | (22,41; 30,68) | 1,10 | 0,80 | 0,79 | 0,655994 |
| 2 | 33,27 | 34,24 | 1,90 | (30,11; 38,38) | -0,97 | -0,71 | -0,69 | 0,655994 |
| 3 | 23,33 | 24,69 | 2,00 | (20,33; 29,05) | -1,36 | -1,12 | -1,13 | 0,729945 |
| 4 | 27,02 | 27,20 | 2,00 | (22,83; 31,56) | -0,18 | -0,15 | -0,14 | 0,729945 |
| 5 | 24,32 | 26,60 | 1,62 | (23,08; 30,13) | -2,28 | -1,35 | -1,40 | 0,476813 |
| 6 | 33,93 | 35,32 | 1,62 | (31,79; 38,85) | -1,39 | -0,82 | -0,81 | 0,476813 |
| 7 | 24,87 | 25,80 | 1,95 | (21,56; 30,04) | -0,94 | -0,72 | -0,70 | 0,688750 |
| 8 | 28,96 | 29,70 | 1,95 | (25,46; 33,94) | -0,74 | -0,57 | -0,55 | 0,688750 |
| 9 | 26,77 | 26,58 | 1,79 | (22,68; 30,47) | 0,19 | 0,13 | 0,12 | 0,580949 |
| 10 | 32,84 | 32,57 | 1,79 | (28,68; 36,46) | 0,27 | 0,18 | 0,17 | 0,580949 |
| 11 | 28,14 | 29,33 | 1,53 | (26,00; 32,66) | -1,19 | -0,67 | -0,65 | 0,425781 |
| 12 | 33,17 | 32,40 | 1,53 | (29,07; 35,73) | 0,78 | 0,44 | 0,42 | 0,425781 |
| 13 | 26,18 | 26,61 | 1,77 | (22,76; 30,46) | -0,43 | -0,28 | -0,27 | 0,569258 |
| 14 | 35,42 | 34,11 | 1,77 | (30,26; 37,96) | 1,31 | 0,85 | 0,84 | 0,569258 |
| 15 | 29,73 | 29,53 | 1,54 | (26,17; 32,89) | 0,21 | 0,12 | 0,11 | 0,433227 |
| 16 | 29,70 | 33,10 | 1,54 | (29,74; 36,46) | -3,40 | -1,93 | -2,22 | 0,433227 |
| 17 | 24,33 | 23,10 | 1,81 | (19,15; 27,05) | 1,23 | 0,82 | 0,81 | 0,597775 |
| 18 | 36,10 | 35,06 | 1,81 | (31,12; 39,01) | 1,03 | 0,70 | 0,68 | 0,597775 |
| 19 | 30,54 | 30,85 | 1,50 | (27,59; 34,11) | -0,31 | -0,17 | -0,16 | 0,407038 |
| 20 | 33,49 | 30,73 | 1,89 | (26,62; 34,84) | 2,76 | 1,98 | 2,32 | 0,647412 |
| 21 | 29,84 | 30,07 | 1,50 | (26,81; 33,33) | -0,23 | -0,13 | -0,12 | 0,407933 |
| 22 | 34,91 | 33,61 | 2,12 | (28,99; 38,22) | 1,30 | 1,30 | 1,34 | 0,817044 |
| 23 | 28,55 | 27,50 | 2,13 | (22,87; 32,13) | 1,05 | 1,07 | 1,08 | 0,823434 |
| 24 | 31,05 | 31,36 | 1,98 | (27,05; 35,68) | -0,31 | -0,25 | -0,24 | 0,713775 |
| 25 | 27,48 | 28,63 | 1,26 | (25,89; 31,38) | -1,15 | -0,58 | -0,57 | 0,288793 |
| 26 | 27,60 | 28,63 | 1,26 | (25,89; 31,38) | -1,04 | -0,52 | -0,51 | 0,288793 |
| 27 | 33,33 | 28,63 | 1,26 | (25,89; 31,38) | 4,70 | 2,38 | 3,13 | 0,288793 |

| Obs | Cook’s D | DFITS |  |
| --- | --- | --- | --- |
| 1 | 0,08 | 1,08601 |  |
| 2 | 0,06 | -0,95266 |  |
| 3 | 0,22 | -1,85692 |  |
| 4 | 0,00 | -0,23008 |  |
| 5 | 0,11 | -1,33630 |  |
| 6 | 0,04 | -0,77069 |  |
| 7 | 0,08 | -1,04327 |  |
| 8 | 0,05 | -0,81815 |  |
| 9 | 0,00 | 0,14244 |  |
| 10 | 0,00 | 0,20052 |  |
| 11 | 0,02 | -0,56384 |  |
| 12 | 0,01 | 0,36269 |  |
| 13 | 0,01 | -0,31129 |  |
| 14 | 0,06 | 0,96535 |  |
| 15 | 0,00 | 0,09837 |  |
| 16 | 0,19 | -1,94159 |  |
| 17 | 0,07 | 0,99027 |  |
| 18 | 0,05 | 0,82851 |  |
| 19 | 0,00 | -0,13465 |  |
| 20 | 0,48 | 3,13755 |  |
| 21 | 0,00 | -0,10086 |  |
| 22 | 0,50 | 2,83588 |  |
| 23 | 0,36 | 2,32279 |  |
| 24 | 0,01 | -0,37548 |  |
| 25 | 0,01 | -0,36065 |  |
| 26 | 0,01 | -0,32394 |  |
| 27 | 0,15 | 1,99136 | R |

R  Large residual

## Coded Coefficients

| Term | Coef | SE Coef | 95% CI | T-Value | P-Value | VIF |
| --- | --- | --- | --- | --- | --- | --- |
| Constant | 32,21 | 1,29 | (29,40; 35,01) | 25,00 | 0,000 |  |
| Lac | 5,54 | 1,07 | (3,22; 7,86) | 5,20 | 0,000 | 1,18 |
| HPMC\_Visc | 0,34 | 1,11 | (-2,08; 2,77) | 0,31 | 0,763 | 1,70 |
| HPMC\_HP | 1,34 | 1,09 | (-1,03; 3,71) | 1,23 | 0,241 | 1,26 |
| HPMC\_PS | 4,27 | 1,67 | (0,63; 7,90) | 2,55 | 0,025 | 2,09 |
| Lac\*Lac | 0,39 | 2,12 | (-4,22; 5,00) | 0,18 | 0,857 | 1,30 |
| HPMC\_Visc\*HPMC\_Visc | 3,25 | 2,25 | (-1,65; 8,16) | 1,45 | 0,174 | 1,96 |
| HPMC\_HP\*HPMC\_HP | 3,36 | 2,13 | (-1,29; 8,01) | 1,58 | 0,141 | 1,81 |
| HPMC\_PS\*HPMC\_PS | 0,72 | 2,12 | (-3,90; 5,34) | 0,34 | 0,740 | 1,42 |
| Lac\*HPMC\_Visc | -4,48 | 2,33 | (-9,56; 0,61) | -1,92 | 0,079 | 1,49 |
| Lac\*HPMC\_HP | 1,62 | 2,45 | (-3,72; 6,96) | 0,66 | 0,520 | 1,17 |
| Lac\*HPMC\_PS | -2,60 | 3,78 | (-10,84; 5,64) | -0,69 | 0,505 | 1,42 |
| HPMC\_Visc\*HPMC\_HP | -1,90 | 2,79 | (-7,97; 4,17) | -0,68 | 0,508 | 2,73 |
| HPMC\_Visc\*HPMC\_PS | 9,55 | 3,94 | (0,97; 18,12) | 2,43 | 0,032 | 2,74 |
| HPMC\_HP\*HPMC\_PS | -1,78 | 4,34 | (-11,25; 7,69) | -0,41 | 0,689 | 2,70 |

## Model Summary

| S | R-sq | R-sq(adj) | PRESS | R-sq(pred) | AICc | BIC |
| --- | --- | --- | --- | --- | --- | --- |
| 2,39794 | 82,28% | 61,61% | 380,174 | 2,39% | 188,36 | 154,69 |

## Analysis of Variance

| Source | DF | Seq SS | Contribution | Adj SS | Adj MS | F-Value | P-Value |
| --- | --- | --- | --- | --- | --- | --- | --- |
| Model | 14 | 320,468 | 82,28% | 320,468 | 22,891 | 3,98 | 0,011 |
| Linear | 4 | 254,312 | 65,30% | 210,993 | 52,748 | 9,17 | 0,001 |
| Lac | 1 | 212,960 | 54,68% | 155,475 | 155,475 | 27,04 | 0,000 |
| HPMC\_Visc | 1 | 14,608 | 3,75% | 0,546 | 0,546 | 0,09 | 0,763 |
| HPMC\_HP | 1 | 10,109 | 2,60% | 8,734 | 8,734 | 1,52 | 0,241 |
| HPMC\_PS | 1 | 16,635 | 4,27% | 37,530 | 37,530 | 6,53 | 0,025 |
| Square | 4 | 9,284 | 2,38% | 22,195 | 5,549 | 0,96 | 0,462 |
| Lac\*Lac | 1 | 1,803 | 0,46% | 0,194 | 0,194 | 0,03 | 0,857 |
| HPMC\_Visc\*HPMC\_Visc | 1 | 0,132 | 0,03% | 12,027 | 12,027 | 2,09 | 0,174 |
| HPMC\_HP\*HPMC\_HP | 1 | 2,979 | 0,76% | 14,264 | 14,264 | 2,48 | 0,141 |
| HPMC\_PS\*HPMC\_PS | 1 | 4,369 | 1,12% | 0,663 | 0,663 | 0,12 | 0,740 |
| 2-Way Interaction | 6 | 56,873 | 14,60% | 56,873 | 9,479 | 1,65 | 0,217 |
| Lac\*HPMC\_Visc | 1 | 18,561 | 4,77% | 21,151 | 21,151 | 3,68 | 0,079 |
| Lac\*HPMC\_HP | 1 | 1,514 | 0,39% | 2,522 | 2,522 | 0,44 | 0,520 |
| Lac\*HPMC\_PS | 1 | 2,722 | 0,70% | 2,722 | 2,722 | 0,47 | 0,505 |
| HPMC\_Visc\*HPMC\_HP | 1 | 0,230 | 0,06% | 2,676 | 2,676 | 0,47 | 0,508 |
| HPMC\_Visc\*HPMC\_PS | 1 | 32,880 | 8,44% | 33,826 | 33,826 | 5,88 | 0,032 |
| HPMC\_HP\*HPMC\_PS | 1 | 0,966 | 0,25% | 0,966 | 0,966 | 0,17 | 0,689 |
| Error | 12 | 69,001 | 17,72% | 69,001 | 5,750 |  |  |
| Lack-of-Fit | 10 | 46,957 | 12,06% | 46,957 | 4,696 | 0,43 | 0,854 |
| Pure Error | 2 | 22,045 | 5,66% | 22,045 | 11,022 |  |  |
| Total | 26 | 389,470 | 100,00% |  |  |  |  |

## Regression Equation in Uncoded Units

|  |  |  |
| --- | --- | --- |
| F\_mean\_2.5h(150min) | = | 410 + 116 Lac - 0,0221 HPMC\_Visc - 40,6 HPMC\_HP - 2,82 HPMC\_PS + 6,2 Lac\*Lac + 0,000000 HPMC\_Visc\*HPMC\_Visc + 3,26 HPMC\_HP\*HPMC\_HP + 0,0133 HPMC\_PS\*HPMC\_PS - 0,00460 Lac\*HPMC\_Visc + 6,40 Lac\*HPMC\_HP - 1,42 Lac\*HPMC\_PS - 0,000482 HPMC\_Visc\*HPMC\_HP + 0,000334 HPMC\_Visc\*HPMC\_PS - 0,239 HPMC\_HP\*HPMC\_PS |

## Fits and Diagnostics for All Observations

| Obs | F\_mean\_2.5h(150min) | Fit | SE Fit | 95% CI | Resid | Std Resid | Del Resid |
| --- | --- | --- | --- | --- | --- | --- | --- |
| 1 | 30,97 | 29,77 | 1,94 | (25,53; 34,00) | 1,20 | 0,85 | 0,84 |
| 2 | 36,88 | 37,97 | 1,94 | (33,73; 42,20) | -1,09 | -0,77 | -0,76 |
| 3 | 26,38 | 27,79 | 2,05 | (23,32; 32,25) | -1,41 | -1,13 | -1,15 |
| 4 | 30,40 | 30,52 | 2,05 | (26,06; 34,99) | -0,12 | -0,10 | -0,10 |
| 5 | 27,47 | 29,93 | 1,66 | (26,32; 33,53) | -2,46 | -1,42 | -1,49 |
| 6 | 37,63 | 39,15 | 1,66 | (35,55; 42,76) | -1,53 | -0,88 | -0,87 |
| 7 | 28,09 | 29,01 | 1,99 | (24,67; 33,34) | -0,92 | -0,69 | -0,67 |
| 8 | 32,30 | 33,15 | 1,99 | (28,81; 37,48) | -0,85 | -0,63 | -0,62 |
| 9 | 29,99 | 29,80 | 1,83 | (25,82; 33,78) | 0,18 | 0,12 | 0,11 |
| 10 | 36,54 | 36,26 | 1,83 | (32,27; 40,24) | 0,29 | 0,19 | 0,18 |
| 11 | 31,46 | 32,60 | 1,56 | (29,19; 36,00) | -1,14 | -0,63 | -0,61 |
| 12 | 36,74 | 35,96 | 1,56 | (32,55; 39,37) | 0,78 | 0,43 | 0,41 |
| 13 | 29,41 | 29,88 | 1,81 | (25,94; 33,82) | -0,47 | -0,30 | -0,29 |
| 14 | 39,14 | 37,86 | 1,81 | (33,92; 41,81) | 1,27 | 0,81 | 0,80 |
| 15 | 33,01 | 32,80 | 1,58 | (29,36; 36,24) | 0,22 | 0,12 | 0,12 |
| 16 | 33,17 | 36,66 | 1,58 | (33,22; 40,09) | -3,49 | -1,93 | -2,23 |
| 17 | 27,30 | 26,09 | 1,85 | (22,06; 30,13) | 1,21 | 0,79 | 0,78 |
| 18 | 40,03 | 38,86 | 1,85 | (34,82; 42,90) | 1,18 | 0,77 | 0,76 |
| 19 | 34,29 | 34,42 | 1,53 | (31,09; 37,76) | -0,13 | -0,07 | -0,07 |
| 20 | 36,83 | 34,04 | 1,93 | (29,83; 38,24) | 2,79 | 1,96 | 2,28 |
| 21 | 33,17 | 33,48 | 1,53 | (30,15; 36,82) | -0,31 | -0,17 | -0,16 |
| 22 | 38,63 | 37,21 | 2,17 | (32,49; 41,94) | 1,42 | 1,39 | 1,45 |
| 23 | 32,13 | 31,02 | 2,18 | (26,28; 35,76) | 1,11 | 1,10 | 1,11 |
| 24 | 34,52 | 34,85 | 2,03 | (30,43; 39,26) | -0,32 | -0,25 | -0,24 |
| 25 | 30,94 | 32,09 | 1,29 | (29,28; 34,89) | -1,14 | -0,56 | -0,55 |
| 26 | 31,13 | 32,09 | 1,29 | (29,28; 34,89) | -0,96 | -0,48 | -0,46 |
| 27 | 36,78 | 32,09 | 1,29 | (29,28; 34,89) | 4,70 | 2,32 | 3,00 |

| Obs | HI | Cook’s D | DFITS |  |
| --- | --- | --- | --- | --- |
| 1 | 0,655994 | 0,09 | 1,16316 |  |
| 2 | 0,655994 | 0,08 | -1,05106 |  |
| 3 | 0,729945 | 0,23 | -1,88631 |  |
| 4 | 0,729945 | 0,00 | -0,15675 |  |
| 5 | 0,476813 | 0,12 | -1,41839 |  |
| 6 | 0,476813 | 0,05 | -0,83094 |  |
| 7 | 0,688750 | 0,07 | -0,99549 |  |
| 8 | 0,688750 | 0,06 | -0,91686 |  |
| 9 | 0,580949 | 0,00 | 0,13429 |  |
| 10 | 0,580949 | 0,00 | 0,21009 |  |
| 11 | 0,425781 | 0,02 | -0,52514 |  |
| 12 | 0,425781 | 0,01 | 0,35553 |  |
| 13 | 0,569258 | 0,01 | -0,33020 |  |
| 14 | 0,569258 | 0,06 | 0,91419 |  |
| 15 | 0,433227 | 0,00 | 0,10065 |  |
| 16 | 0,433227 | 0,19 | -1,94768 |  |
| 17 | 0,597775 | 0,06 | 0,95009 |  |
| 18 | 0,597775 | 0,06 | 0,92645 |  |
| 19 | 0,407038 | 0,00 | -0,05706 |  |
| 20 | 0,647412 | 0,47 | 3,08854 |  |
| 21 | 0,407933 | 0,00 | -0,13478 |  |
| 22 | 0,817044 | 0,57 | 3,05767 |  |
| 23 | 0,823434 | 0,38 | 2,39438 |  |
| 24 | 0,713775 | 0,01 | -0,38245 |  |
| 25 | 0,288793 | 0,01 | -0,34921 |  |
| 26 | 0,288793 | 0,01 | -0,29280 |  |
| 27 | 0,288793 | 0,15 | 1,90967 | R |

R  Large residual

## Coded Coefficients

| Term | Coef | SE Coef | 95% CI | T-Value | P-Value | VIF |
| --- | --- | --- | --- | --- | --- | --- |
| Constant | 35,38 | 1,31 | (32,54; 38,23) | 27,08 | 0,000 |  |
| Lac | 5,85 | 1,08 | (3,50; 8,21) | 5,42 | 0,000 | 1,18 |
| HPMC\_Visc | 0,22 | 1,13 | (-2,24; 2,68) | 0,19 | 0,850 | 1,70 |
| HPMC\_HP | 1,43 | 1,10 | (-0,98; 3,83) | 1,29 | 0,221 | 1,26 |
| HPMC\_PS | 4,25 | 1,69 | (0,56; 7,94) | 2,51 | 0,027 | 2,09 |
| Lac\*Lac | 0,34 | 2,15 | (-4,34; 5,02) | 0,16 | 0,877 | 1,30 |
| HPMC\_Visc\*HPMC\_Visc | 3,21 | 2,28 | (-1,76; 8,18) | 1,41 | 0,185 | 1,96 |
| HPMC\_HP\*HPMC\_HP | 3,40 | 2,17 | (-1,31; 8,12) | 1,57 | 0,142 | 1,81 |
| HPMC\_PS\*HPMC\_PS | 0,79 | 2,15 | (-3,90; 5,48) | 0,37 | 0,720 | 1,42 |
| Lac\*HPMC\_Visc | -4,55 | 2,37 | (-9,71; 0,61) | -1,92 | 0,079 | 1,49 |
| Lac\*HPMC\_HP | 1,55 | 2,49 | (-3,86; 6,97) | 0,62 | 0,544 | 1,17 |
| Lac\*HPMC\_PS | -2,58 | 3,84 | (-10,94; 5,78) | -0,67 | 0,514 | 1,42 |
| HPMC\_Visc\*HPMC\_HP | -1,90 | 2,83 | (-8,06; 4,26) | -0,67 | 0,515 | 2,73 |
| HPMC\_Visc\*HPMC\_PS | 9,60 | 3,99 | (0,90; 18,30) | 2,40 | 0,033 | 2,74 |
| HPMC\_HP\*HPMC\_PS | -1,84 | 4,41 | (-11,44; 7,76) | -0,42 | 0,684 | 2,70 |

## Model Summary

| S | R-sq | R-sq(adj) | PRESS | R-sq(pred) | AICc | BIC |
| --- | --- | --- | --- | --- | --- | --- |
| 2,43222 | 83,14% | 63,47% | 402,518 | 4,41% | 189,12 | 155,46 |

## Analysis of Variance

| Source | DF | Seq SS | Contribution | Adj SS | Adj MS | F-Value | P-Value |
| --- | --- | --- | --- | --- | --- | --- | --- |
| Model | 14 | 350,100 | 83,14% | 350,100 | 25,007 | 4,23 | 0,008 |
| Linear | 4 | 282,693 | 67,13% | 232,177 | 58,044 | 9,81 | 0,001 |
| Lac | 1 | 237,540 | 56,41% | 173,709 | 173,709 | 29,36 | 0,000 |
| HPMC\_Visc | 1 | 17,378 | 4,13% | 0,220 | 0,220 | 0,04 | 0,850 |
| HPMC\_HP | 1 | 11,373 | 2,70% | 9,873 | 9,873 | 1,67 | 0,221 |
| HPMC\_PS | 1 | 16,402 | 3,90% | 37,260 | 37,260 | 6,30 | 0,027 |
| Square | 4 | 9,425 | 2,24% | 22,289 | 5,572 | 0,94 | 0,473 |
| Lac\*Lac | 1 | 2,043 | 0,49% | 0,147 | 0,147 | 0,02 | 0,877 |
| HPMC\_Visc\*HPMC\_Visc | 1 | 0,222 | 0,05% | 11,705 | 11,705 | 1,98 | 0,185 |
| HPMC\_HP\*HPMC\_HP | 1 | 3,048 | 0,72% | 14,627 | 14,627 | 2,47 | 0,142 |
| HPMC\_PS\*HPMC\_PS | 1 | 4,112 | 0,98% | 0,794 | 0,794 | 0,13 | 0,720 |
| 2-Way Interaction | 6 | 57,982 | 13,77% | 57,982 | 9,664 | 1,63 | 0,221 |
| Lac\*HPMC\_Visc | 1 | 19,426 | 4,61% | 21,829 | 21,829 | 3,69 | 0,079 |
| Lac\*HPMC\_HP | 1 | 1,354 | 0,32% | 2,310 | 2,310 | 0,39 | 0,544 |
| Lac\*HPMC\_PS | 1 | 2,675 | 0,64% | 2,675 | 2,675 | 0,45 | 0,514 |
| HPMC\_Visc\*HPMC\_HP | 1 | 0,282 | 0,07% | 2,664 | 2,664 | 0,45 | 0,515 |
| HPMC\_Visc\*HPMC\_PS | 1 | 33,213 | 7,89% | 34,215 | 34,215 | 5,78 | 0,033 |
| HPMC\_HP\*HPMC\_PS | 1 | 1,032 | 0,25% | 1,032 | 1,032 | 0,17 | 0,684 |
| Error | 12 | 70,988 | 16,86% | 70,988 | 5,916 |  |  |
| Lack-of-Fit | 10 | 49,368 | 11,72% | 49,368 | 4,937 | 0,46 | 0,837 |
| Pure Error | 2 | 21,620 | 5,13% | 21,620 | 10,810 |  |  |
| Total | 26 | 421,088 | 100,00% |  |  |  |  |

## Regression Equation in Uncoded Units

|  |  |  |
| --- | --- | --- |
| F\_mean\_3h(180min) | = | 417 + 121 Lac - 0,0222 HPMC\_Visc - 40,6 HPMC\_HP - 2,95 HPMC\_PS + 5,4 Lac\*Lac + 0,000000 HPMC\_Visc\*HPMC\_Visc + 3,31 HPMC\_HP\*HPMC\_HP + 0,0146 HPMC\_PS\*HPMC\_PS - 0,00468 Lac\*HPMC\_Visc + 6,12 Lac\*HPMC\_HP - 1,40 Lac\*HPMC\_PS - 0,000480 HPMC\_Visc\*HPMC\_HP + 0,000336 HPMC\_Visc\*HPMC\_PS - 0,247 HPMC\_HP\*HPMC\_PS |

## Fits and Diagnostics for All Observations

| Obs | F\_mean\_3h(180min) | Fit | SE Fit | 95% CI | Resid | Std Resid | Del Resid | HI |
| --- | --- | --- | --- | --- | --- | --- | --- | --- |
| 1 | 34,03 | 32,76 | 1,97 | (28,47; 37,05) | 1,27 | 0,89 | 0,88 | 0,655994 |
| 2 | 40,22 | 41,37 | 1,97 | (37,08; 45,66) | -1,15 | -0,81 | -0,79 | 0,655994 |
| 3 | 29,17 | 30,63 | 2,08 | (26,10; 35,16) | -1,46 | -1,16 | -1,18 | 0,729945 |
| 4 | 33,62 | 33,67 | 2,08 | (29,15; 38,20) | -0,05 | -0,04 | -0,04 | 0,729945 |
| 5 | 30,47 | 33,01 | 1,68 | (29,35; 36,67) | -2,54 | -1,45 | -1,52 | 0,476813 |
| 6 | 40,92 | 42,58 | 1,68 | (38,92; 46,24) | -1,66 | -0,94 | -0,94 | 0,476813 |
| 7 | 31,11 | 32,01 | 2,02 | (27,61; 36,41) | -0,90 | -0,66 | -0,65 | 0,688750 |
| 8 | 35,44 | 36,38 | 2,02 | (31,98; 40,77) | -0,94 | -0,69 | -0,67 | 0,688750 |
| 9 | 32,97 | 32,79 | 1,85 | (28,75; 36,83) | 0,19 | 0,12 | 0,11 | 0,580949 |
| 10 | 39,97 | 39,67 | 1,85 | (35,63; 43,70) | 0,31 | 0,19 | 0,19 | 0,580949 |
| 11 | 34,49 | 35,54 | 1,59 | (32,08; 39,00) | -1,05 | -0,57 | -0,55 | 0,425781 |
| 12 | 39,97 | 39,24 | 1,59 | (35,79; 42,70) | 0,73 | 0,39 | 0,38 | 0,425781 |
| 13 | 32,41 | 32,95 | 1,84 | (28,95; 36,95) | -0,54 | -0,34 | -0,33 | 0,569258 |
| 14 | 42,55 | 41,29 | 1,84 | (37,29; 45,28) | 1,26 | 0,79 | 0,78 | 0,569258 |
| 15 | 35,99 | 35,79 | 1,60 | (32,31; 39,28) | 0,19 | 0,11 | 0,10 | 0,433227 |
| 16 | 36,41 | 39,94 | 1,60 | (36,46; 43,43) | -3,53 | -1,93 | -2,22 | 0,433227 |
| 17 | 30,09 | 28,91 | 1,88 | (24,81; 33,00) | 1,19 | 0,77 | 0,76 | 0,597775 |
| 18 | 43,61 | 42,33 | 1,88 | (38,23; 46,43) | 1,28 | 0,83 | 0,82 | 0,597775 |
| 19 | 37,66 | 37,67 | 1,55 | (34,29; 41,05) | -0,01 | -0,01 | -0,01 | 0,407038 |
| 20 | 39,84 | 37,02 | 1,96 | (32,76; 41,29) | 2,82 | 1,95 | 2,26 | 0,647412 |
| 21 | 36,22 | 36,63 | 1,55 | (33,25; 40,02) | -0,41 | -0,22 | -0,21 | 0,407933 |
| 22 | 42,05 | 40,52 | 2,20 | (35,73; 45,31) | 1,52 | 1,47 | 1,55 | 0,817044 |
| 23 | 35,45 | 34,31 | 2,21 | (29,51; 39,12) | 1,13 | 1,11 | 1,12 | 0,823434 |
| 24 | 37,77 | 38,09 | 2,05 | (33,61; 42,57) | -0,32 | -0,25 | -0,24 | 0,713775 |
| 25 | 34,15 | 35,28 | 1,31 | (32,43; 38,13) | -1,13 | -0,55 | -0,53 | 0,288793 |
| 26 | 34,40 | 35,28 | 1,31 | (32,43; 38,13) | -0,88 | -0,43 | -0,41 | 0,288793 |
| 27 | 39,97 | 35,28 | 1,31 | (32,43; 38,13) | 4,69 | 2,28 | 2,91 | 0,288793 |

| Obs | Cook’s D | DFITS |  |
| --- | --- | --- | --- |
| 1 | 0,10 | 1,21570 |  |
| 2 | 0,08 | -1,09447 |  |
| 3 | 0,24 | -1,93383 |  |
| 4 | 0,00 | -0,06132 |  |
| 5 | 0,13 | -1,45367 |  |
| 6 | 0,05 | -0,89569 |  |
| 7 | 0,06 | -0,96136 |  |
| 8 | 0,07 | -1,00360 |  |
| 9 | 0,00 | 0,13292 |  |
| 10 | 0,00 | 0,21931 |  |
| 11 | 0,02 | -0,47384 |  |
| 12 | 0,01 | 0,32673 |  |
| 13 | 0,01 | -0,37409 |  |
| 14 | 0,05 | 0,89197 |  |
| 15 | 0,00 | 0,08875 |  |
| 16 | 0,19 | -1,94359 |  |
| 17 | 0,06 | 0,92102 |  |
| 18 | 0,07 | 0,99962 |  |
| 19 | 0,00 | -0,00504 |  |
| 20 | 0,47 | 3,05973 |  |
| 21 | 0,00 | -0,17575 |  |
| 22 | 0,64 | 3,27330 |  |
| 23 | 0,38 | 2,42247 |  |
| 24 | 0,01 | -0,37386 |  |
| 25 | 0,01 | -0,33999 |  |
| 26 | 0,00 | -0,26356 |  |
| 27 | 0,14 | 1,85470 | R |

R  Large residual

## Coded Coefficients

| Term | Coef | SE Coef | 95% CI | T-Value | P-Value | VIF |
| --- | --- | --- | --- | --- | --- | --- |
| Constant | 38,37 | 1,32 | (35,49; 41,25) | 29,06 | 0,000 |  |
| Lac | 6,16 | 1,09 | (3,78; 8,54) | 5,64 | 0,000 | 1,18 |
| HPMC\_Visc | 0,11 | 1,14 | (-2,37; 2,60) | 0,10 | 0,924 | 1,70 |
| HPMC\_HP | 1,48 | 1,12 | (-0,95; 3,92) | 1,33 | 0,208 | 1,26 |
| HPMC\_PS | 4,23 | 1,71 | (0,50; 7,96) | 2,47 | 0,029 | 2,09 |
| Lac\*Lac | 0,26 | 2,17 | (-4,47; 4,99) | 0,12 | 0,907 | 1,30 |
| HPMC\_Visc\*HPMC\_Visc | 3,22 | 2,31 | (-1,81; 8,24) | 1,40 | 0,188 | 1,96 |
| HPMC\_HP\*HPMC\_HP | 3,42 | 2,19 | (-1,35; 8,19) | 1,56 | 0,144 | 1,81 |
| HPMC\_PS\*HPMC\_PS | 0,92 | 2,18 | (-3,82; 5,66) | 0,42 | 0,679 | 1,42 |
| Lac\*HPMC\_Visc | -4,65 | 2,39 | (-9,86; 0,57) | -1,94 | 0,076 | 1,49 |
| Lac\*HPMC\_HP | 1,49 | 2,51 | (-3,98; 6,97) | 0,59 | 0,563 | 1,17 |
| Lac\*HPMC\_PS | -2,56 | 3,88 | (-11,01; 5,89) | -0,66 | 0,521 | 1,42 |
| HPMC\_Visc\*HPMC\_HP | -1,91 | 2,86 | (-8,14; 4,31) | -0,67 | 0,516 | 2,73 |
| HPMC\_Visc\*HPMC\_PS | 9,71 | 4,04 | (0,92; 18,50) | 2,41 | 0,033 | 2,74 |
| HPMC\_HP\*HPMC\_PS | -1,94 | 4,45 | (-11,64; 7,76) | -0,44 | 0,671 | 2,70 |

## Model Summary

| S | R-sq | R-sq(adj) | PRESS | R-sq(pred) | AICc | BIC |
| --- | --- | --- | --- | --- | --- | --- |
| 2,45834 | 83,98% | 65,29% | 418,333 | 7,58% | 189,70 | 156,03 |

## Analysis of Variance

| Source | DF | Seq SS | Contribution | Adj SS | Adj MS | F-Value | P-Value |
| --- | --- | --- | --- | --- | --- | --- | --- |
| Model | 14 | 380,130 | 83,98% | 380,130 | 27,152 | 4,49 | 0,006 |
| Linear | 4 | 311,020 | 68,71% | 252,983 | 63,246 | 10,47 | 0,001 |
| Lac | 1 | 262,710 | 58,04% | 192,188 | 192,188 | 31,80 | 0,000 |
| HPMC\_Visc | 1 | 20,018 | 4,42% | 0,058 | 0,058 | 0,01 | 0,924 |
| HPMC\_HP | 1 | 12,328 | 2,72% | 10,679 | 10,679 | 1,77 | 0,208 |
| HPMC\_PS | 1 | 15,964 | 3,53% | 36,898 | 36,898 | 6,11 | 0,029 |
| Square | 4 | 9,232 | 2,04% | 22,532 | 5,633 | 0,93 | 0,478 |
| Lac\*Lac | 1 | 2,490 | 0,55% | 0,086 | 0,086 | 0,01 | 0,907 |
| HPMC\_Visc\*HPMC\_Visc | 1 | 0,291 | 0,06% | 11,767 | 11,767 | 1,95 | 0,188 |
| HPMC\_HP\*HPMC\_HP | 1 | 2,847 | 0,63% | 14,748 | 14,748 | 2,44 | 0,144 |
| HPMC\_PS\*HPMC\_PS | 1 | 3,604 | 0,80% | 1,089 | 1,089 | 0,18 | 0,679 |
| 2-Way Interaction | 6 | 59,879 | 13,23% | 59,879 | 9,980 | 1,65 | 0,216 |
| Lac\*HPMC\_Visc | 1 | 20,614 | 4,55% | 22,808 | 22,808 | 3,77 | 0,076 |
| Lac\*HPMC\_HP | 1 | 1,223 | 0,27% | 2,135 | 2,135 | 0,35 | 0,563 |
| Lac\*HPMC\_PS | 1 | 2,638 | 0,58% | 2,638 | 2,638 | 0,44 | 0,521 |
| HPMC\_Visc\*HPMC\_HP | 1 | 0,347 | 0,08% | 2,711 | 2,711 | 0,45 | 0,516 |
| HPMC\_Visc\*HPMC\_PS | 1 | 33,910 | 7,49% | 35,009 | 35,009 | 5,79 | 0,033 |
| HPMC\_HP\*HPMC\_PS | 1 | 1,146 | 0,25% | 1,146 | 1,146 | 0,19 | 0,671 |
| Error | 12 | 72,521 | 16,02% | 72,521 | 6,043 |  |  |
| Lack-of-Fit | 10 | 50,851 | 11,23% | 50,851 | 5,085 | 0,47 | 0,831 |
| Pure Error | 2 | 21,670 | 4,79% | 21,670 | 10,835 |  |  |
| Total | 26 | 452,652 | 100,00% |  |  |  |  |

## Regression Equation in Uncoded Units

|  |  |  |
| --- | --- | --- |
| F\_mean\_3.5h(210min) | = | 425 + 126 Lac - 0,0224 HPMC\_Visc - 39,7 HPMC\_HP - 3,23 HPMC\_PS + 4,1 Lac\*Lac + 0,000000 HPMC\_Visc\*HPMC\_Visc + 3,32 HPMC\_HP\*HPMC\_HP + 0,0171 HPMC\_PS\*HPMC\_PS - 0,00478 Lac\*HPMC\_Visc + 5,89 Lac\*HPMC\_HP - 1,39 Lac\*HPMC\_PS - 0,000485 HPMC\_Visc\*HPMC\_HP + 0,000339 HPMC\_Visc\*HPMC\_PS - 0,260 HPMC\_HP\*HPMC\_PS |

## Fits and Diagnostics for All Observations

| Obs | F\_mean\_3.5h(210min) | Fit | SE Fit | 95% CI | Resid | Std Resid | Del Resid |
| --- | --- | --- | --- | --- | --- | --- | --- |
| 1 | 36,87 | 35,57 | 1,99 | (31,24; 39,91) | 1,29 | 0,90 | 0,89 |
| 2 | 43,41 | 44,60 | 1,99 | (40,26; 48,94) | -1,19 | -0,82 | -0,81 |
| 3 | 31,86 | 33,34 | 2,10 | (28,76; 37,92) | -1,48 | -1,16 | -1,18 |
| 4 | 36,62 | 36,65 | 2,10 | (32,07; 41,23) | -0,03 | -0,02 | -0,02 |
| 5 | 33,31 | 35,91 | 1,70 | (32,22; 39,61) | -2,60 | -1,46 | -1,54 |
| 6 | 44,06 | 45,84 | 1,70 | (42,14; 49,54) | -1,77 | -1,00 | -1,00 |
| 7 | 33,96 | 34,85 | 2,04 | (30,41; 39,30) | -0,89 | -0,65 | -0,64 |
| 8 | 38,42 | 39,41 | 2,04 | (34,97; 43,86) | -0,99 | -0,72 | -0,71 |
| 9 | 35,76 | 35,59 | 1,87 | (31,51; 39,68) | 0,16 | 0,10 | 0,10 |
| 10 | 43,22 | 42,89 | 1,87 | (38,81; 46,98) | 0,33 | 0,20 | 0,20 |
| 11 | 37,35 | 38,32 | 1,60 | (34,83; 41,82) | -0,97 | -0,52 | -0,50 |
| 12 | 43,00 | 42,34 | 1,60 | (38,85; 45,84) | 0,65 | 0,35 | 0,34 |
| 13 | 35,26 | 35,81 | 1,85 | (31,77; 39,85) | -0,55 | -0,34 | -0,33 |
| 14 | 45,72 | 44,51 | 1,85 | (40,47; 48,55) | 1,22 | 0,75 | 0,74 |
| 15 | 38,76 | 38,62 | 1,62 | (35,09; 42,14) | 0,14 | 0,08 | 0,07 |
| 16 | 39,50 | 43,03 | 1,62 | (39,51; 46,56) | -3,54 | -1,91 | -2,19 |
| 17 | 32,68 | 31,51 | 1,90 | (27,37; 35,65) | 1,17 | 0,75 | 0,74 |
| 18 | 46,97 | 45,58 | 1,90 | (41,44; 49,73) | 1,38 | 0,89 | 0,88 |
| 19 | 40,84 | 40,76 | 1,57 | (37,34; 44,17) | 0,09 | 0,05 | 0,04 |
| 20 | 42,74 | 39,90 | 1,98 | (35,59; 44,20) | 2,85 | 1,95 | 2,26 |
| 21 | 39,14 | 39,59 | 1,57 | (36,17; 43,01) | -0,45 | -0,24 | -0,23 |
| 22 | 45,19 | 43,59 | 2,22 | (38,75; 48,43) | 1,59 | 1,52 | 1,61 |
| 23 | 38,66 | 37,50 | 2,23 | (32,64; 42,36) | 1,16 | 1,12 | 1,14 |
| 24 | 40,88 | 41,20 | 2,08 | (36,67; 45,72) | -0,31 | -0,24 | -0,23 |
| 25 | 37,16 | 38,29 | 1,32 | (35,41; 41,17) | -1,13 | -0,55 | -0,53 |
| 26 | 37,45 | 38,29 | 1,32 | (35,41; 41,17) | -0,84 | -0,40 | -0,39 |
| 27 | 43,00 | 38,29 | 1,32 | (35,41; 41,17) | 4,71 | 2,27 | 2,88 |

| Obs | HI | Cook’s D | DFITS |  |
| --- | --- | --- | --- | --- |
| 1 | 0,655994 | 0,10 | 1,22561 |  |
| 2 | 0,655994 | 0,09 | -1,11904 |  |
| 3 | 0,729945 | 0,24 | -1,93964 |  |
| 4 | 0,729945 | 0,00 | -0,03736 |  |
| 5 | 0,476813 | 0,13 | -1,47346 |  |
| 6 | 0,476813 | 0,06 | -0,95114 |  |
| 7 | 0,688750 | 0,06 | -0,94620 |  |
| 8 | 0,688750 | 0,08 | -1,05429 |  |
| 9 | 0,580949 | 0,00 | 0,11683 |  |
| 10 | 0,580949 | 0,00 | 0,23077 |  |
| 11 | 0,425781 | 0,01 | -0,43408 |  |
| 12 | 0,425781 | 0,01 | 0,29096 |  |
| 13 | 0,569258 | 0,01 | -0,37782 |  |
| 14 | 0,569258 | 0,05 | 0,85108 |  |
| 15 | 0,433227 | 0,00 | 0,06446 |  |
| 16 | 0,433227 | 0,19 | -1,91747 |  |
| 17 | 0,597775 | 0,06 | 0,89896 |  |
| 18 | 0,597775 | 0,08 | 1,07079 |  |
| 19 | 0,407038 | 0,00 | 0,03618 |  |
| 20 | 0,647412 | 0,47 | 3,06007 |  |
| 21 | 0,407933 | 0,00 | -0,18875 |  |
| 22 | 0,817044 | 0,68 | 3,41238 |  |
| 23 | 0,823434 | 0,39 | 2,45123 |  |
| 24 | 0,713775 | 0,01 | -0,36229 |  |
| 25 | 0,288793 | 0,01 | -0,33736 |  |
| 26 | 0,288793 | 0,00 | -0,24807 |  |
| 27 | 0,288793 | 0,14 | 1,83676 | R |

R  Large residual

## Coded Coefficients

| Term | Coef | SE Coef | 95% CI | T-Value | P-Value | VIF |
| --- | --- | --- | --- | --- | --- | --- |
| Constant | 41,25 | 1,32 | (38,38; 44,13) | 31,27 | 0,000 |  |
| Lac | 6,45 | 1,09 | (4,07; 8,83) | 5,91 | 0,000 | 1,18 |
| HPMC\_Visc | -0,00 | 1,14 | (-2,48; 2,48) | -0,00 | 0,999 | 1,70 |
| HPMC\_HP | 1,58 | 1,12 | (-0,85; 4,01) | 1,41 | 0,182 | 1,26 |
| HPMC\_PS | 4,14 | 1,71 | (0,41; 7,87) | 2,42 | 0,032 | 2,09 |
| Lac\*Lac | 0,19 | 2,17 | (-4,54; 4,91) | 0,09 | 0,933 | 1,30 |
| HPMC\_Visc\*HPMC\_Visc | 3,08 | 2,30 | (-1,94; 8,10) | 1,34 | 0,206 | 1,96 |
| HPMC\_HP\*HPMC\_HP | 3,36 | 2,19 | (-1,41; 8,12) | 1,54 | 0,151 | 1,81 |
| HPMC\_PS\*HPMC\_PS | 0,99 | 2,17 | (-3,75; 5,72) | 0,45 | 0,658 | 1,42 |
| Lac\*HPMC\_Visc | -4,86 | 2,39 | (-10,07; 0,35) | -2,03 | 0,065 | 1,49 |
| Lac\*HPMC\_HP | 1,29 | 2,51 | (-4,18; 6,76) | 0,51 | 0,616 | 1,17 |
| Lac\*HPMC\_PS | -2,81 | 3,87 | (-11,25; 5,63) | -0,73 | 0,482 | 1,42 |
| HPMC\_Visc\*HPMC\_HP | -2,06 | 2,85 | (-8,28; 4,16) | -0,72 | 0,484 | 2,73 |
| HPMC\_Visc\*HPMC\_PS | 9,55 | 4,03 | (0,77; 18,34) | 2,37 | 0,035 | 2,74 |
| HPMC\_HP\*HPMC\_PS | -2,20 | 4,45 | (-11,89; 7,50) | -0,49 | 0,631 | 2,70 |

## Model Summary

| S | R-sq | R-sq(adj) | PRESS | R-sq(pred) | AICc | BIC |
| --- | --- | --- | --- | --- | --- | --- |
| 2,45638 | 85,07% | 67,66% | 427,728 | 11,82% | 189,66 | 155,99 |

## Analysis of Variance

| Source | DF | Seq SS | Contribution | Adj SS | Adj MS | F-Value | P-Value |
| --- | --- | --- | --- | --- | --- | --- | --- |
| Model | 14 | 412,645 | 85,07% | 412,645 | 29,475 | 4,88 | 0,005 |
| Linear | 4 | 343,541 | 70,83% | 273,304 | 68,326 | 11,32 | 0,000 |
| Lac | 1 | 291,368 | 60,07% | 211,013 | 211,013 | 34,97 | 0,000 |
| HPMC\_Visc | 1 | 21,457 | 4,42% | 0,000 | 0,000 | 0,00 | 0,999 |
| HPMC\_HP | 1 | 14,721 | 3,03% | 12,081 | 12,081 | 2,00 | 0,182 |
| HPMC\_PS | 1 | 15,994 | 3,30% | 35,365 | 35,365 | 5,86 | 0,032 |
| Square | 4 | 8,726 | 1,80% | 21,442 | 5,361 | 0,89 | 0,500 |
| Lac\*Lac | 1 | 2,707 | 0,56% | 0,045 | 0,045 | 0,01 | 0,933 |
| HPMC\_Visc\*HPMC\_Visc | 1 | 0,471 | 0,10% | 10,784 | 10,784 | 1,79 | 0,206 |
| HPMC\_HP\*HPMC\_HP | 1 | 2,444 | 0,50% | 14,232 | 14,232 | 2,36 | 0,151 |
| HPMC\_PS\*HPMC\_PS | 1 | 3,104 | 0,64% | 1,244 | 1,244 | 0,21 | 0,658 |
| 2-Way Interaction | 6 | 60,379 | 12,45% | 60,379 | 10,063 | 1,67 | 0,212 |
| Lac\*HPMC\_Visc | 1 | 22,180 | 4,57% | 24,882 | 24,882 | 4,12 | 0,065 |
| Lac\*HPMC\_HP | 1 | 0,748 | 0,15% | 1,600 | 1,600 | 0,27 | 0,616 |
| Lac\*HPMC\_PS | 1 | 3,176 | 0,65% | 3,176 | 3,176 | 0,53 | 0,482 |
| HPMC\_Visc\*HPMC\_HP | 1 | 0,262 | 0,05% | 3,145 | 3,145 | 0,52 | 0,484 |
| HPMC\_Visc\*HPMC\_PS | 1 | 32,545 | 6,71% | 33,873 | 33,873 | 5,61 | 0,035 |
| HPMC\_HP\*HPMC\_PS | 1 | 1,468 | 0,30% | 1,468 | 1,468 | 0,24 | 0,631 |
| Error | 12 | 72,406 | 14,93% | 72,406 | 6,034 |  |  |
| Lack-of-Fit | 10 | 51,205 | 10,56% | 51,205 | 5,120 | 0,48 | 0,823 |
| Pure Error | 2 | 21,201 | 4,37% | 21,201 | 10,600 |  |  |
| Total | 26 | 485,051 | 100,00% |  |  |  |  |

## Regression Equation in Uncoded Units

|  |  |  |
| --- | --- | --- |
| F\_mean\_4h(240min) | = | 384 + 149 Lac - 0,0213 HPMC\_Visc - 35,2 HPMC\_HP - 2,94 HPMC\_PS + 3,0 Lac\*Lac + 0,000000 HPMC\_Visc\*HPMC\_Visc + 3,26 HPMC\_HP\*HPMC\_HP + 0,0182 HPMC\_PS\*HPMC\_PS - 0,00499 Lac\*HPMC\_Visc + 5,09 Lac\*HPMC\_HP - 1,53 Lac\*HPMC\_PS - 0,000522 HPMC\_Visc\*HPMC\_HP + 0,000334 HPMC\_Visc\*HPMC\_PS - 0,294 HPMC\_HP\*HPMC\_PS |

## Fits and Diagnostics for All Observations

| Obs | F\_mean\_4h(240min) | Fit | SE Fit | 95% CI | Resid | Std Resid | Del Resid | HI |
| --- | --- | --- | --- | --- | --- | --- | --- | --- |
| 1 | 39,02 | 37,90 | 1,99 | (33,57; 42,24) | 1,12 | 0,77 | 0,76 | 0,655994 |
| 2 | 46,45 | 47,57 | 1,99 | (43,24; 51,91) | -1,12 | -0,78 | -0,77 | 0,655994 |
| 3 | 34,37 | 35,83 | 2,10 | (31,26; 40,40) | -1,47 | -1,15 | -1,16 | 0,729945 |
| 4 | 39,54 | 39,59 | 2,10 | (35,02; 44,16) | -0,06 | -0,04 | -0,04 | 0,729945 |
| 5 | 36,01 | 38,62 | 1,70 | (34,93; 42,32) | -2,61 | -1,47 | -1,56 | 0,476813 |
| 6 | 46,98 | 48,98 | 1,70 | (45,28; 52,67) | -2,00 | -1,12 | -1,14 | 0,476813 |
| 7 | 36,68 | 37,61 | 2,04 | (33,16; 42,05) | -0,93 | -0,68 | -0,66 | 0,688750 |
| 8 | 41,29 | 42,32 | 2,04 | (37,87; 46,76) | -1,02 | -0,75 | -0,73 | 0,688750 |
| 9 | 38,42 | 38,16 | 1,87 | (34,09; 42,24) | 0,25 | 0,16 | 0,15 | 0,580949 |
| 10 | 46,21 | 45,93 | 1,87 | (41,85; 50,01) | 0,28 | 0,17 | 0,17 | 0,580949 |
| 11 | 40,03 | 40,97 | 1,60 | (37,48; 44,46) | -0,93 | -0,50 | -0,49 | 0,425781 |
| 12 | 45,90 | 45,29 | 1,60 | (41,79; 48,78) | 0,61 | 0,33 | 0,32 | 0,425781 |
| 13 | 38,01 | 38,62 | 1,85 | (34,58; 42,66) | -0,61 | -0,38 | -0,36 | 0,569258 |
| 14 | 48,77 | 47,54 | 1,85 | (43,50; 51,58) | 1,23 | 0,76 | 0,75 | 0,569258 |
| 15 | 41,45 | 41,33 | 1,62 | (37,81; 44,86) | 0,12 | 0,06 | 0,06 | 0,433227 |
| 16 | 42,45 | 45,89 | 1,62 | (42,36; 49,41) | -3,43 | -1,86 | -2,10 | 0,433227 |
| 17 | 35,19 | 33,98 | 1,90 | (29,84; 38,11) | 1,21 | 0,78 | 0,76 | 0,597775 |
| 18 | 50,19 | 48,76 | 1,90 | (44,62; 52,89) | 1,43 | 0,92 | 0,91 | 0,597775 |
| 19 | 43,87 | 43,60 | 1,57 | (40,19; 47,02) | 0,27 | 0,14 | 0,14 | 0,407038 |
| 20 | 45,41 | 42,56 | 1,98 | (38,26; 46,87) | 2,85 | 1,95 | 2,27 | 0,647412 |
| 21 | 41,86 | 42,29 | 1,57 | (38,87; 45,71) | -0,43 | -0,23 | -0,22 | 0,407933 |
| 22 | 48,17 | 46,52 | 2,22 | (41,68; 51,35) | 1,65 | 1,57 | 1,69 | 0,817044 |
| 23 | 41,72 | 40,49 | 2,23 | (35,63; 45,34) | 1,23 | 1,19 | 1,22 | 0,823434 |
| 24 | 43,77 | 44,14 | 2,08 | (39,61; 48,66) | -0,37 | -0,28 | -0,27 | 0,713775 |
| 25 | 40,00 | 41,18 | 1,32 | (38,30; 44,06) | -1,18 | -0,57 | -0,55 | 0,288793 |
| 26 | 40,42 | 41,18 | 1,32 | (38,30; 44,06) | -0,76 | -0,37 | -0,35 | 0,288793 |
| 27 | 45,84 | 41,18 | 1,32 | (38,30; 44,06) | 4,66 | 2,25 | 2,83 | 0,288793 |

| Obs | Cook’s D | DFITS |  |
| --- | --- | --- | --- |
| 1 | 0,08 | 1,05040 |  |
| 2 | 0,08 | -1,05895 |  |
| 3 | 0,24 | -1,91494 |  |
| 4 | 0,00 | -0,06788 |  |
| 5 | 0,13 | -1,48515 |  |
| 6 | 0,08 | -1,08537 |  |
| 7 | 0,07 | -0,98431 |  |
| 8 | 0,08 | -1,08969 |  |
| 9 | 0,00 | 0,17849 |  |
| 10 | 0,00 | 0,19677 |  |
| 11 | 0,01 | -0,41842 |  |
| 12 | 0,01 | 0,27135 |  |
| 13 | 0,01 | -0,41805 |  |
| 14 | 0,05 | 0,86250 |  |
| 15 | 0,00 | 0,05220 |  |
| 16 | 0,18 | -1,84018 |  |
| 17 | 0,06 | 0,93163 |  |
| 18 | 0,08 | 1,11403 |  |
| 19 | 0,00 | 0,11274 |  |
| 20 | 0,47 | 3,06934 |  |
| 21 | 0,00 | -0,17974 |  |
| 22 | 0,73 | 3,56368 |  |
| 23 | 0,44 | 2,63197 |  |
| 24 | 0,01 | -0,42136 |  |
| 25 | 0,01 | -0,35098 |  |
| 26 | 0,00 | -0,22470 |  |
| 27 | 0,14 | 1,80512 | R |

R  Large residual

## Coded Coefficients

| Term | Coef | SE Coef | 95% CI | T-Value | P-Value | VIF |
| --- | --- | --- | --- | --- | --- | --- |
| Constant | 43,95 | 1,33 | (41,06; 46,85) | 33,07 | 0,000 |  |
| Lac | 6,73 | 1,10 | (4,34; 9,13) | 6,13 | 0,000 | 1,18 |
| HPMC\_Visc | -0,07 | 1,15 | (-2,57; 2,43) | -0,06 | 0,951 | 1,70 |
| HPMC\_HP | 1,65 | 1,12 | (-0,80; 4,10) | 1,47 | 0,168 | 1,26 |
| HPMC\_PS | 4,11 | 1,72 | (0,35; 7,86) | 2,38 | 0,035 | 2,09 |
| Lac\*Lac | 0,16 | 2,18 | (-4,60; 4,92) | 0,07 | 0,942 | 1,30 |
| HPMC\_Visc\*HPMC\_Visc | 3,09 | 2,32 | (-1,97; 8,15) | 1,33 | 0,208 | 1,96 |
| HPMC\_HP\*HPMC\_HP | 3,34 | 2,20 | (-1,46; 8,14) | 1,52 | 0,156 | 1,81 |
| HPMC\_PS\*HPMC\_PS | 1,13 | 2,19 | (-3,64; 5,90) | 0,52 | 0,615 | 1,42 |
| Lac\*HPMC\_Visc | -4,99 | 2,41 | (-10,24; 0,26) | -2,07 | 0,060 | 1,49 |
| Lac\*HPMC\_HP | 1,24 | 2,53 | (-4,27; 6,75) | 0,49 | 0,632 | 1,17 |
| Lac\*HPMC\_PS | -2,87 | 3,90 | (-11,38; 5,63) | -0,74 | 0,476 | 1,42 |
| HPMC\_Visc\*HPMC\_HP | -2,09 | 2,88 | (-8,36; 4,17) | -0,73 | 0,481 | 2,73 |
| HPMC\_Visc\*HPMC\_PS | 9,72 | 4,06 | (0,87; 18,57) | 2,39 | 0,034 | 2,74 |
| HPMC\_HP\*HPMC\_PS | -2,32 | 4,48 | (-12,09; 7,45) | -0,52 | 0,614 | 2,70 |

## Model Summary

| S | R-sq | R-sq(adj) | PRESS | R-sq(pred) | AICc | BIC |
| --- | --- | --- | --- | --- | --- | --- |
| 2,47454 | 85,80% | 69,23% | 444,973 | 13,99% | 190,05 | 156,39 |

## Analysis of Variance

| Source | DF | Seq SS | Contribution | Adj SS | Adj MS | F-Value | P-Value |
| --- | --- | --- | --- | --- | --- | --- | --- |
| Model | 14 | 443,854 | 85,80% | 443,854 | 31,704 | 5,18 | 0,004 |
| Linear | 4 | 372,481 | 72,00% | 293,822 | 73,456 | 12,00 | 0,000 |
| Lac | 1 | 317,551 | 61,38% | 229,775 | 229,775 | 37,52 | 0,000 |
| HPMC\_Visc | 1 | 23,408 | 4,52% | 0,024 | 0,024 | 0,00 | 0,951 |
| HPMC\_HP | 1 | 16,213 | 3,13% | 13,168 | 13,168 | 2,15 | 0,168 |
| HPMC\_PS | 1 | 15,308 | 2,96% | 34,802 | 34,802 | 5,68 | 0,035 |
| Square | 4 | 8,301 | 1,60% | 21,329 | 5,332 | 0,87 | 0,509 |
| Lac\*Lac | 1 | 2,945 | 0,57% | 0,034 | 0,034 | 0,01 | 0,942 |
| HPMC\_Visc\*HPMC\_Visc | 1 | 0,606 | 0,12% | 10,853 | 10,853 | 1,77 | 0,208 |
| HPMC\_HP\*HPMC\_HP | 1 | 2,063 | 0,40% | 14,061 | 14,061 | 2,30 | 0,156 |
| HPMC\_PS\*HPMC\_PS | 1 | 2,687 | 0,52% | 1,630 | 1,630 | 0,27 | 0,615 |
| 2-Way Interaction | 6 | 63,073 | 12,19% | 63,073 | 10,512 | 1,72 | 0,200 |
| Lac\*HPMC\_Visc | 1 | 23,549 | 4,55% | 26,289 | 26,289 | 4,29 | 0,060 |
| Lac\*HPMC\_HP | 1 | 0,647 | 0,13% | 1,478 | 1,478 | 0,24 | 0,632 |
| Lac\*HPMC\_PS | 1 | 3,319 | 0,64% | 3,319 | 3,319 | 0,54 | 0,476 |
| HPMC\_Visc\*HPMC\_HP | 1 | 0,332 | 0,06% | 3,242 | 3,242 | 0,53 | 0,481 |
| HPMC\_Visc\*HPMC\_PS | 1 | 33,585 | 6,49% | 35,041 | 35,041 | 5,72 | 0,034 |
| HPMC\_HP\*HPMC\_PS | 1 | 1,639 | 0,32% | 1,639 | 1,639 | 0,27 | 0,614 |
| Error | 12 | 73,480 | 14,20% | 73,480 | 6,123 |  |  |
| Lack-of-Fit | 10 | 52,799 | 10,21% | 52,799 | 5,280 | 0,51 | 0,808 |
| Pure Error | 2 | 20,681 | 4,00% | 20,681 | 10,340 |  |  |
| Total | 26 | 517,334 | 100,00% |  |  |  |  |

## Regression Equation in Uncoded Units

|  |  |  |
| --- | --- | --- |
| F\_mean\_4.5h(270min) | = | 387 + 156 Lac - 0,0216 HPMC\_Visc - 33,4 HPMC\_HP - 3,21 HPMC\_PS + 2,6 Lac\*Lac + 0,000000 HPMC\_Visc\*HPMC\_Visc + 3,24 HPMC\_HP\*HPMC\_HP + 0,0209 HPMC\_PS\*HPMC\_PS - 0,00513 Lac\*HPMC\_Visc + 4,90 Lac\*HPMC\_HP - 1,56 Lac\*HPMC\_PS - 0,000530 HPMC\_Visc\*HPMC\_HP + 0,000340 HPMC\_Visc\*HPMC\_PS - 0,311 HPMC\_HP\*HPMC\_PS |

## Fits and Diagnostics for All Observations

| Obs | F\_mean\_4.5h(270min) | Fit | SE Fit | 95% CI | Resid | Std Resid | Del Resid |
| --- | --- | --- | --- | --- | --- | --- | --- |
| 1 | 41,49 | 40,39 | 2,00 | (36,02; 44,75) | 1,10 | 0,76 | 0,74 |
| 2 | 49,33 | 50,49 | 2,00 | (46,12; 54,85) | -1,16 | -0,80 | -0,78 |
| 3 | 36,77 | 38,25 | 2,11 | (33,65; 42,86) | -1,48 | -1,15 | -1,17 |
| 4 | 42,25 | 42,27 | 2,11 | (37,67; 46,88) | -0,03 | -0,02 | -0,02 |
| 5 | 38,57 | 41,23 | 1,71 | (37,51; 44,96) | -2,66 | -1,49 | -1,58 |
| 6 | 49,79 | 51,96 | 1,71 | (48,23; 55,68) | -2,16 | -1,21 | -1,24 |
| 7 | 39,26 | 40,20 | 2,05 | (35,72; 44,67) | -0,94 | -0,68 | -0,66 |
| 8 | 44,01 | 45,09 | 2,05 | (40,62; 49,56) | -1,08 | -0,78 | -0,77 |
| 9 | 40,92 | 40,65 | 1,89 | (36,54; 44,76) | 0,27 | 0,17 | 0,16 |
| 10 | 49,10 | 48,80 | 1,89 | (44,69; 52,91) | 0,30 | 0,19 | 0,18 |
| 11 | 42,66 | 43,50 | 1,61 | (39,98; 47,02) | -0,84 | -0,45 | -0,43 |
| 12 | 48,64 | 48,09 | 1,61 | (44,57; 51,60) | 0,56 | 0,30 | 0,29 |
| 13 | 40,53 | 41,18 | 1,87 | (37,11; 45,24) | -0,65 | -0,40 | -0,38 |
| 14 | 51,64 | 50,42 | 1,87 | (46,35; 54,49) | 1,22 | 0,75 | 0,74 |
| 15 | 44,03 | 43,92 | 1,63 | (40,37; 47,47) | 0,11 | 0,06 | 0,06 |
| 16 | 45,28 | 48,69 | 1,63 | (45,14; 52,24) | -3,41 | -1,83 | -2,06 |
| 17 | 37,54 | 36,35 | 1,91 | (32,19; 40,52) | 1,18 | 0,75 | 0,74 |
| 18 | 53,28 | 51,76 | 1,91 | (47,59; 55,93) | 1,53 | 0,97 | 0,97 |
| 19 | 46,77 | 46,36 | 1,58 | (42,92; 49,80) | 0,41 | 0,21 | 0,21 |
| 20 | 48,03 | 45,18 | 1,99 | (40,84; 49,52) | 2,85 | 1,94 | 2,24 |
| 21 | 44,42 | 44,90 | 1,58 | (41,46; 48,35) | -0,48 | -0,25 | -0,24 |
| 22 | 50,99 | 49,27 | 2,24 | (44,40; 54,14) | 1,72 | 1,63 | 1,76 |
| 23 | 44,70 | 43,41 | 2,25 | (38,52; 48,30) | 1,29 | 1,24 | 1,27 |
| 24 | 46,53 | 46,93 | 2,09 | (42,38; 51,49) | -0,40 | -0,30 | -0,29 |
| 25 | 42,72 | 43,89 | 1,33 | (41,00; 46,79) | -1,17 | -0,56 | -0,54 |
| 26 | 43,19 | 43,89 | 1,33 | (41,00; 46,79) | -0,70 | -0,34 | -0,32 |
| 27 | 48,51 | 43,89 | 1,33 | (41,00; 46,79) | 4,62 | 2,21 | 2,75 |

| Obs | HI | Cook’s D | DFITS |  |
| --- | --- | --- | --- | --- |
| 1 | 0,655994 | 0,07 | 1,02766 |  |
| 2 | 0,655994 | 0,08 | -1,08133 |  |
| 3 | 0,729945 | 0,24 | -1,92060 |  |
| 4 | 0,729945 | 0,00 | -0,03360 |  |
| 5 | 0,476813 | 0,13 | -1,50369 |  |
| 6 | 0,476813 | 0,09 | -1,17958 |  |
| 7 | 0,688750 | 0,07 | -0,98385 |  |
| 8 | 0,688750 | 0,09 | -1,14284 |  |
| 9 | 0,580949 | 0,00 | 0,18945 |  |
| 10 | 0,580949 | 0,00 | 0,20928 |  |
| 11 | 0,425781 | 0,01 | -0,37334 |  |
| 12 | 0,425781 | 0,00 | 0,24556 |  |
| 13 | 0,569258 | 0,01 | -0,44059 |  |
| 14 | 0,569258 | 0,05 | 0,84634 |  |
| 15 | 0,433227 | 0,00 | 0,05146 |  |
| 16 | 0,433227 | 0,17 | -1,80515 |  |
| 17 | 0,597775 | 0,06 | 0,90218 |  |
| 18 | 0,597775 | 0,09 | 1,18319 |  |
| 19 | 0,407038 | 0,00 | 0,17052 |  |
| 20 | 0,647412 | 0,46 | 3,03525 |  |
| 21 | 0,407933 | 0,00 | -0,19996 |  |
| 22 | 0,817044 | 0,79 | 3,72797 |  |
| 23 | 0,823434 | 0,48 | 2,73690 |  |
| 24 | 0,713775 | 0,02 | -0,45893 |  |
| 25 | 0,288793 | 0,01 | -0,34651 |  |
| 26 | 0,288793 | 0,00 | -0,20664 |  |
| 27 | 0,288793 | 0,13 | 1,75513 | R |

R  Large residual

## Coded Coefficients

| Term | Coef | SE Coef | 95% CI | T-Value | P-Value | VIF |
| --- | --- | --- | --- | --- | --- | --- |
| Constant | 46,56 | 1,35 | (43,63; 49,49) | 34,61 | 0,000 |  |
| Lac | 7,02 | 1,11 | (4,60; 9,45) | 6,31 | 0,000 | 1,18 |
| HPMC\_Visc | -0,17 | 1,16 | (-2,70; 2,36) | -0,15 | 0,885 | 1,70 |
| HPMC\_HP | 1,75 | 1,14 | (-0,73; 4,23) | 1,54 | 0,149 | 1,26 |
| HPMC\_PS | 4,00 | 1,74 | (0,20; 7,80) | 2,29 | 0,041 | 2,09 |
| Lac\*Lac | 0,14 | 2,21 | (-4,68; 4,95) | 0,06 | 0,952 | 1,30 |
| HPMC\_Visc\*HPMC\_Visc | 3,07 | 2,35 | (-2,05; 8,19) | 1,31 | 0,215 | 1,96 |
| HPMC\_HP\*HPMC\_HP | 3,30 | 2,23 | (-1,56; 8,16) | 1,48 | 0,165 | 1,81 |
| HPMC\_PS\*HPMC\_PS | 1,24 | 2,22 | (-3,58; 6,07) | 0,56 | 0,585 | 1,42 |
| Lac\*HPMC\_Visc | -5,04 | 2,44 | (-10,36; 0,27) | -2,07 | 0,061 | 1,49 |
| Lac\*HPMC\_HP | 1,21 | 2,56 | (-4,37; 6,79) | 0,47 | 0,645 | 1,17 |
| Lac\*HPMC\_PS | -2,78 | 3,95 | (-11,39; 5,83) | -0,70 | 0,495 | 1,42 |
| HPMC\_Visc\*HPMC\_HP | -2,01 | 2,91 | (-8,36; 4,33) | -0,69 | 0,502 | 2,73 |
| HPMC\_Visc\*HPMC\_PS | 9,65 | 4,11 | (0,70; 18,61) | 2,35 | 0,037 | 2,74 |
| HPMC\_HP\*HPMC\_PS | -2,32 | 4,54 | (-12,21; 7,57) | -0,51 | 0,619 | 2,70 |

## Model Summary

| S | R-sq | R-sq(adj) | PRESS | R-sq(pred) | AICc | BIC |
| --- | --- | --- | --- | --- | --- | --- |
| 2,50469 | 86,27% | 70,25% | 467,117 | 14,81% | 190,71 | 157,04 |

## Analysis of Variance

| Source | DF | Seq SS | Contribution | Adj SS | Adj MS | F-Value | P-Value |
| --- | --- | --- | --- | --- | --- | --- | --- |
| Model | 14 | 473,027 | 86,27% | 473,027 | 33,788 | 5,39 | 0,003 |
| Linear | 4 | 401,688 | 73,26% | 315,224 | 78,806 | 12,56 | 0,000 |
| Lac | 1 | 344,216 | 62,78% | 249,914 | 249,914 | 39,84 | 0,000 |
| HPMC\_Visc | 1 | 25,469 | 4,65% | 0,137 | 0,137 | 0,02 | 0,885 |
| HPMC\_HP | 1 | 18,040 | 3,29% | 14,883 | 14,883 | 2,37 | 0,149 |
| HPMC\_PS | 1 | 13,963 | 2,55% | 33,004 | 33,004 | 5,26 | 0,041 |
| Square | 4 | 7,867 | 1,43% | 21,027 | 5,257 | 0,84 | 0,527 |
| Lac\*Lac | 1 | 3,140 | 0,57% | 0,023 | 0,023 | 0,00 | 0,952 |
| HPMC\_Visc\*HPMC\_Visc | 1 | 0,616 | 0,11% | 10,737 | 10,737 | 1,71 | 0,215 |
| HPMC\_HP\*HPMC\_HP | 1 | 1,904 | 0,35% | 13,741 | 13,741 | 2,19 | 0,165 |
| HPMC\_PS\*HPMC\_PS | 1 | 2,207 | 0,40% | 1,977 | 1,977 | 0,32 | 0,585 |
| 2-Way Interaction | 6 | 63,472 | 11,58% | 63,472 | 10,579 | 1,69 | 0,208 |
| Lac\*HPMC\_Visc | 1 | 24,528 | 4,47% | 26,839 | 26,839 | 4,28 | 0,061 |
| Lac\*HPMC\_HP | 1 | 0,618 | 0,11% | 1,402 | 1,402 | 0,22 | 0,645 |
| Lac\*HPMC\_PS | 1 | 3,109 | 0,57% | 3,109 | 3,109 | 0,50 | 0,495 |
| HPMC\_Visc\*HPMC\_HP | 1 | 0,444 | 0,08% | 3,001 | 3,001 | 0,48 | 0,502 |
| HPMC\_Visc\*HPMC\_PS | 1 | 33,136 | 6,04% | 34,585 | 34,585 | 5,51 | 0,037 |
| HPMC\_HP\*HPMC\_PS | 1 | 1,637 | 0,30% | 1,637 | 1,637 | 0,26 | 0,619 |
| Error | 12 | 75,282 | 13,73% | 75,282 | 6,273 |  |  |
| Lack-of-Fit | 10 | 55,017 | 10,03% | 55,017 | 5,502 | 0,54 | 0,792 |
| Pure Error | 2 | 20,265 | 3,70% | 20,265 | 10,132 |  |  |
| Total | 26 | 548,309 | 100,00% |  |  |  |  |

## Regression Equation in Uncoded Units

|  |  |  |
| --- | --- | --- |
| F\_mean\_5h(300min) | = | 398 + 156 Lac - 0,0216 HPMC\_Visc - 32,8 HPMC\_HP - 3,52 HPMC\_PS + 2,2 Lac\*Lac + 0,000000 HPMC\_Visc\*HPMC\_Visc + 3,20 HPMC\_HP\*HPMC\_HP + 0,0230 HPMC\_PS\*HPMC\_PS - 0,00519 Lac\*HPMC\_Visc + 4,8 Lac\*HPMC\_HP - 1,51 Lac\*HPMC\_PS - 0,000510 HPMC\_Visc\*HPMC\_HP + 0,000337 HPMC\_Visc\*HPMC\_PS - 0,311 HPMC\_HP\*HPMC\_PS |

## Fits and Diagnostics for All Observations

| Obs | F\_mean\_5h(300min) | Fit | SE Fit | 95% CI | Resid | Std Resid | Del Resid | HI |
| --- | --- | --- | --- | --- | --- | --- | --- | --- |
| 1 | 43,97 | 42,85 | 2,03 | (38,43; 47,27) | 1,12 | 0,76 | 0,75 | 0,655994 |
| 2 | 52,11 | 53,28 | 2,03 | (48,86; 57,70) | -1,17 | -0,80 | -0,78 | 0,655994 |
| 3 | 39,10 | 40,65 | 2,14 | (35,99; 45,31) | -1,55 | -1,19 | -1,21 | 0,729945 |
| 4 | 44,90 | 44,89 | 2,14 | (40,23; 49,56) | 0,01 | 0,01 | 0,01 | 0,729945 |
| 5 | 40,99 | 43,76 | 1,73 | (40,00; 47,53) | -2,78 | -1,53 | -1,64 | 0,476813 |
| 6 | 52,48 | 54,79 | 1,73 | (51,02; 58,56) | -2,31 | -1,28 | -1,31 | 0,476813 |
| 7 | 41,89 | 42,76 | 2,08 | (38,23; 47,29) | -0,87 | -0,62 | -0,60 | 0,688750 |
| 8 | 46,67 | 47,85 | 2,08 | (43,32; 52,38) | -1,18 | -0,84 | -0,83 | 0,688750 |
| 9 | 43,32 | 43,06 | 1,91 | (38,90; 47,22) | 0,26 | 0,16 | 0,15 | 0,580949 |
| 10 | 51,86 | 51,60 | 1,91 | (47,44; 55,76) | 0,26 | 0,16 | 0,16 | 0,580949 |
| 11 | 45,16 | 45,84 | 1,63 | (42,28; 49,40) | -0,68 | -0,36 | -0,34 | 0,425781 |
| 12 | 51,24 | 50,74 | 1,63 | (47,18; 54,30) | 0,50 | 0,26 | 0,25 | 0,425781 |
| 13 | 43,03 | 43,67 | 1,89 | (39,55; 47,79) | -0,64 | -0,39 | -0,38 | 0,569258 |
| 14 | 54,48 | 53,27 | 1,89 | (49,16; 57,39) | 1,20 | 0,73 | 0,72 | 0,569258 |
| 15 | 46,29 | 46,34 | 1,65 | (42,74; 49,93) | -0,05 | -0,02 | -0,02 | 0,433227 |
| 16 | 48,02 | 51,40 | 1,65 | (47,81; 54,99) | -3,38 | -1,79 | -2,00 | 0,433227 |
| 17 | 39,83 | 38,65 | 1,94 | (34,43; 42,87) | 1,18 | 0,74 | 0,73 | 0,597775 |
| 18 | 56,28 | 54,65 | 1,94 | (50,43; 58,87) | 1,63 | 1,02 | 1,03 | 0,597775 |
| 19 | 49,59 | 49,05 | 1,60 | (45,56; 52,53) | 0,54 | 0,28 | 0,27 | 0,407038 |
| 20 | 50,54 | 47,66 | 2,02 | (43,27; 52,05) | 2,88 | 1,93 | 2,23 | 0,647412 |
| 21 | 46,88 | 47,41 | 1,60 | (43,92; 50,89) | -0,52 | -0,27 | -0,26 | 0,407933 |
| 22 | 53,74 | 51,94 | 2,26 | (47,01; 56,87) | 1,80 | 1,68 | 1,84 | 0,817044 |
| 23 | 47,57 | 46,24 | 2,27 | (41,29; 51,19) | 1,33 | 1,26 | 1,30 | 0,823434 |
| 24 | 49,16 | 49,57 | 2,12 | (44,96; 54,18) | -0,41 | -0,30 | -0,29 | 0,713775 |
| 25 | 45,31 | 46,52 | 1,35 | (43,58; 49,45) | -1,20 | -0,57 | -0,55 | 0,288793 |
| 26 | 45,93 | 46,52 | 1,35 | (43,58; 49,45) | -0,58 | -0,28 | -0,27 | 0,288793 |
| 27 | 51,11 | 46,52 | 1,35 | (43,58; 49,45) | 4,60 | 2,18 | 2,68 | 0,288793 |

| Obs | Cook’s D | DFITS |  |
| --- | --- | --- | --- |
| 1 | 0,07 | 1,03539 |  |
| 2 | 0,08 | -1,08299 |  |
| 3 | 0,25 | -1,99016 |  |
| 4 | 0,00 | 0,01131 |  |
| 5 | 0,14 | -1,56202 |  |
| 6 | 0,10 | -1,25346 |  |
| 7 | 0,06 | -0,89702 |  |
| 8 | 0,11 | -1,24042 |  |
| 9 | 0,00 | 0,18127 |  |
| 10 | 0,00 | 0,18294 |  |
| 11 | 0,01 | -0,29673 |  |
| 12 | 0,00 | 0,21768 |  |
| 13 | 0,01 | -0,43376 |  |
| 14 | 0,05 | 0,82475 |  |
| 15 | 0,00 | -0,02014 |  |
| 16 | 0,16 | -1,75082 |  |
| 17 | 0,05 | 0,88976 |  |
| 18 | 0,10 | 1,25094 |  |
| 19 | 0,00 | 0,22431 |  |
| 20 | 0,46 | 3,02527 |  |
| 21 | 0,00 | -0,21699 |  |
| 22 | 0,84 | 3,88705 |  |
| 23 | 0,50 | 2,80164 |  |
| 24 | 0,02 | -0,46129 |  |
| 25 | 0,01 | -0,35156 |  |
| 26 | 0,00 | -0,16922 |  |
| 27 | 0,13 | 1,70545 | R |

R  Large residual

## Coded Coefficients

| Term | Coef | SE Coef | 95% CI | T-Value | P-Value | VIF |
| --- | --- | --- | --- | --- | --- | --- |
| Constant | 49,08 | 1,35 | (46,14; 52,02) | 36,39 | 0,000 |  |
| Lac | 7,28 | 1,12 | (4,85; 9,71) | 6,53 | 0,000 | 1,18 |
| HPMC\_Visc | -0,28 | 1,17 | (-2,82; 2,26) | -0,24 | 0,816 | 1,70 |
| HPMC\_HP | 1,85 | 1,14 | (-0,64; 4,33) | 1,62 | 0,131 | 1,26 |
| HPMC\_PS | 3,93 | 1,75 | (0,12; 7,74) | 2,25 | 0,044 | 2,09 |
| Lac\*Lac | 0,12 | 2,22 | (-4,71; 4,95) | 0,06 | 0,956 | 1,30 |
| HPMC\_Visc\*HPMC\_Visc | 3,04 | 2,36 | (-2,10; 8,17) | 1,29 | 0,222 | 1,96 |
| HPMC\_HP\*HPMC\_HP | 3,27 | 2,24 | (-1,60; 8,14) | 1,46 | 0,169 | 1,81 |
| HPMC\_PS\*HPMC\_PS | 1,33 | 2,22 | (-3,52; 6,17) | 0,60 | 0,562 | 1,42 |
| Lac\*HPMC\_Visc | -5,08 | 2,44 | (-10,41; 0,25) | -2,08 | 0,060 | 1,49 |
| Lac\*HPMC\_HP | 1,19 | 2,57 | (-4,40; 6,79) | 0,47 | 0,650 | 1,17 |
| Lac\*HPMC\_PS | -2,68 | 3,96 | (-11,31; 5,95) | -0,68 | 0,511 | 1,42 |
| HPMC\_Visc\*HPMC\_HP | -1,95 | 2,92 | (-8,31; 4,41) | -0,67 | 0,516 | 2,73 |
| HPMC\_Visc\*HPMC\_PS | 9,70 | 4,12 | (0,72; 18,68) | 2,35 | 0,037 | 2,74 |
| HPMC\_HP\*HPMC\_PS | -2,25 | 4,55 | (-12,16; 7,66) | -0,49 | 0,630 | 2,70 |

## Model Summary

| S | R-sq | R-sq(adj) | PRESS | R-sq(pred) | AICc | BIC |
| --- | --- | --- | --- | --- | --- | --- |
| 2,51113 | 86,89% | 71,60% | 479,169 | 17,01% | 190,85 | 157,18 |

## Analysis of Variance

| Source | DF | Seq SS | Contribution | Adj SS | Adj MS | F-Value | P-Value |
| --- | --- | --- | --- | --- | --- | --- | --- |
| Model | 14 | 501,678 | 86,89% | 501,678 | 35,834 | 5,68 | 0,002 |
| Linear | 4 | 429,565 | 74,40% | 336,411 | 84,103 | 13,34 | 0,000 |
| Lac | 1 | 368,864 | 63,89% | 268,809 | 268,809 | 42,63 | 0,000 |
| HPMC\_Visc | 1 | 28,152 | 4,88% | 0,356 | 0,356 | 0,06 | 0,816 |
| HPMC\_HP | 1 | 19,803 | 3,43% | 16,537 | 16,537 | 2,62 | 0,131 |
| HPMC\_PS | 1 | 12,746 | 2,21% | 31,820 | 31,820 | 5,05 | 0,044 |
| Square | 4 | 7,686 | 1,33% | 20,633 | 5,158 | 0,82 | 0,538 |
| Lac\*Lac | 1 | 3,217 | 0,56% | 0,020 | 0,020 | 0,00 | 0,956 |
| HPMC\_Visc\*HPMC\_Visc | 1 | 0,751 | 0,13% | 10,476 | 10,476 | 1,66 | 0,222 |
| HPMC\_HP\*HPMC\_HP | 1 | 1,755 | 0,30% | 13,483 | 13,483 | 2,14 | 0,169 |
| HPMC\_PS\*HPMC\_PS | 1 | 1,963 | 0,34% | 2,244 | 2,244 | 0,36 | 0,562 |
| 2-Way Interaction | 6 | 64,427 | 11,16% | 64,427 | 10,738 | 1,70 | 0,204 |
| Lac\*HPMC\_Visc | 1 | 25,339 | 4,39% | 27,213 | 27,213 | 4,32 | 0,060 |
| Lac\*HPMC\_HP | 1 | 0,618 | 0,11% | 1,366 | 1,366 | 0,22 | 0,650 |
| Lac\*HPMC\_PS | 1 | 2,890 | 0,50% | 2,890 | 2,890 | 0,46 | 0,511 |
| HPMC\_Visc\*HPMC\_HP | 1 | 0,511 | 0,09% | 2,825 | 2,825 | 0,45 | 0,516 |
| HPMC\_Visc\*HPMC\_PS | 1 | 33,526 | 5,81% | 34,916 | 34,916 | 5,54 | 0,037 |
| HPMC\_HP\*HPMC\_PS | 1 | 1,543 | 0,27% | 1,543 | 1,543 | 0,24 | 0,630 |
| Error | 12 | 75,669 | 13,11% | 75,669 | 6,306 |  |  |
| Lack-of-Fit | 10 | 55,872 | 9,68% | 55,872 | 5,587 | 0,56 | 0,781 |
| Pure Error | 2 | 19,797 | 3,43% | 19,797 | 9,899 |  |  |
| Total | 26 | 577,347 | 100,00% |  |  |  |  |

## Regression Equation in Uncoded Units

|  |  |  |
| --- | --- | --- |
| F\_mean\_5.5h(330min) | = | 415 + 155 Lac - 0,0218 HPMC\_Visc - 33,0 HPMC\_HP - 3,87 HPMC\_PS + 2,0 Lac\*Lac + 0,000000 HPMC\_Visc\*HPMC\_Visc + 3,17 HPMC\_HP\*HPMC\_HP + 0,0245 HPMC\_PS\*HPMC\_PS - 0,00522 Lac\*HPMC\_Visc + 4,7 Lac\*HPMC\_HP - 1,46 Lac\*HPMC\_PS - 0,000495 HPMC\_Visc\*HPMC\_HP + 0,000339 HPMC\_Visc\*HPMC\_PS - 0,301 HPMC\_HP\*HPMC\_PS |

## Fits and Diagnostics for All Observations

| Obs | F\_mean\_5.5h(330min) | Fit | SE Fit | 95% CI | Resid | Std Resid | Del Resid |
| --- | --- | --- | --- | --- | --- | --- | --- |
| 1 | 46,43 | 45,28 | 2,03 | (40,85; 49,71) | 1,15 | 0,78 | 0,77 |
| 2 | 54,76 | 55,98 | 2,03 | (51,55; 60,41) | -1,23 | -0,83 | -0,82 |
| 3 | 41,35 | 42,95 | 2,15 | (38,27; 47,62) | -1,59 | -1,22 | -1,25 |
| 4 | 47,45 | 47,38 | 2,15 | (42,70; 52,05) | 0,07 | 0,06 | 0,05 |
| 5 | 43,40 | 46,23 | 1,73 | (42,46; 50,01) | -2,84 | -1,56 | -1,67 |
| 6 | 55,11 | 57,52 | 1,73 | (53,74; 61,30) | -2,41 | -1,33 | -1,38 |
| 7 | 44,34 | 45,16 | 2,08 | (40,62; 49,70) | -0,82 | -0,59 | -0,57 |
| 8 | 49,21 | 50,45 | 2,08 | (45,91; 54,99) | -1,24 | -0,89 | -0,88 |
| 9 | 45,59 | 45,37 | 1,91 | (41,20; 49,54) | 0,22 | 0,14 | 0,13 |
| 10 | 54,54 | 54,24 | 1,91 | (50,07; 58,41) | 0,30 | 0,18 | 0,18 |
| 11 | 47,60 | 48,12 | 1,64 | (44,55; 51,69) | -0,52 | -0,27 | -0,26 |
| 12 | 53,70 | 53,30 | 1,64 | (49,73; 56,87) | 0,40 | 0,21 | 0,20 |
| 13 | 45,42 | 46,08 | 1,89 | (41,95; 50,21) | -0,66 | -0,40 | -0,39 |
| 14 | 57,19 | 56,02 | 1,89 | (51,89; 60,14) | 1,17 | 0,71 | 0,70 |
| 15 | 48,59 | 48,70 | 1,65 | (45,10; 52,30) | -0,11 | -0,06 | -0,05 |
| 16 | 50,74 | 54,04 | 1,65 | (50,44; 57,64) | -3,30 | -1,75 | -1,93 |
| 17 | 42,07 | 40,92 | 1,94 | (36,69; 45,15) | 1,16 | 0,73 | 0,71 |
| 18 | 59,14 | 57,44 | 1,94 | (53,21; 61,67) | 1,69 | 1,06 | 1,07 |
| 19 | 52,30 | 51,62 | 1,60 | (48,13; 55,11) | 0,68 | 0,35 | 0,34 |
| 20 | 52,87 | 50,02 | 2,02 | (45,62; 54,42) | 2,85 | 1,91 | 2,19 |
| 21 | 49,29 | 49,85 | 1,60 | (46,36; 53,35) | -0,56 | -0,29 | -0,28 |
| 22 | 56,38 | 54,53 | 2,27 | (49,59; 59,48) | 1,85 | 1,72 | 1,90 |
| 23 | 50,31 | 48,95 | 2,28 | (43,99; 53,92) | 1,36 | 1,29 | 1,33 |
| 24 | 51,67 | 52,09 | 2,12 | (47,47; 56,71) | -0,42 | -0,31 | -0,30 |
| 25 | 47,85 | 49,06 | 1,35 | (46,12; 52,00) | -1,20 | -0,57 | -0,55 |
| 26 | 48,52 | 49,06 | 1,35 | (46,12; 52,00) | -0,54 | -0,25 | -0,24 |
| 27 | 53,60 | 49,06 | 1,35 | (46,12; 52,00) | 4,55 | 2,15 | 2,62 |

| Obs | HI | Cook’s D | DFITS |  |
| --- | --- | --- | --- | --- |
| 1 | 0,655994 | 0,08 | 1,05678 |  |
| 2 | 0,655994 | 0,09 | -1,13342 |  |
| 3 | 0,729945 | 0,27 | -2,05016 |  |
| 4 | 0,729945 | 0,00 | 0,08975 |  |
| 5 | 0,476813 | 0,15 | -1,59880 |  |
| 6 | 0,476813 | 0,11 | -1,31349 |  |
| 7 | 0,688750 | 0,05 | -0,85057 |  |
| 8 | 0,688750 | 0,12 | -1,30875 |  |
| 9 | 0,580949 | 0,00 | 0,15565 |  |
| 10 | 0,580949 | 0,00 | 0,20647 |  |
| 11 | 0,425781 | 0,00 | -0,22561 |  |
| 12 | 0,425781 | 0,00 | 0,17254 |  |
| 13 | 0,569258 | 0,01 | -0,44431 |  |
| 14 | 0,569258 | 0,04 | 0,79905 |  |
| 15 | 0,433227 | 0,00 | -0,04668 |  |
| 16 | 0,433227 | 0,16 | -1,69138 |  |
| 17 | 0,597775 | 0,05 | 0,86725 |  |
| 18 | 0,597775 | 0,11 | 1,30490 |  |
| 19 | 0,407038 | 0,01 | 0,27988 |  |
| 20 | 0,647412 | 0,45 | 2,96836 |  |
| 21 | 0,407933 | 0,00 | -0,23223 |  |
| 22 | 0,817044 | 0,88 | 4,01572 |  |
| 23 | 0,823434 | 0,51 | 2,86302 |  |
| 24 | 0,713775 | 0,02 | -0,47790 |  |
| 25 | 0,288793 | 0,01 | -0,35188 |  |
| 26 | 0,288793 | 0,00 | -0,15470 |  |
| 27 | 0,288793 | 0,12 | 1,67009 | R |

R  Large residual

## Coded Coefficients

| Term | Coef | SE Coef | 95% CI | T-Value | P-Value | VIF |
| --- | --- | --- | --- | --- | --- | --- |
| Constant | 51,54 | 1,35 | (48,59; 54,49) | 38,08 | 0,000 |  |
| Lac | 7,52 | 1,12 | (5,08; 9,96) | 6,72 | 0,000 | 1,18 |
| HPMC\_Visc | -0,34 | 1,17 | (-2,89; 2,21) | -0,29 | 0,778 | 1,70 |
| HPMC\_HP | 1,94 | 1,14 | (-0,55; 4,43) | 1,70 | 0,116 | 1,26 |
| HPMC\_PS | 3,91 | 1,75 | (0,09; 7,73) | 2,23 | 0,046 | 2,09 |
| Lac\*Lac | 0,09 | 2,22 | (-4,75; 4,94) | 0,04 | 0,967 | 1,30 |
| HPMC\_Visc\*HPMC\_Visc | 3,06 | 2,36 | (-2,09; 8,21) | 1,29 | 0,220 | 1,96 |
| HPMC\_HP\*HPMC\_HP | 3,20 | 2,24 | (-1,68; 8,09) | 1,43 | 0,179 | 1,81 |
| HPMC\_PS\*HPMC\_PS | 1,38 | 2,23 | (-3,48; 6,24) | 0,62 | 0,547 | 1,42 |
| Lac\*HPMC\_Visc | -5,06 | 2,45 | (-10,41; 0,28) | -2,06 | 0,061 | 1,49 |
| Lac\*HPMC\_HP | 1,19 | 2,58 | (-4,42; 6,80) | 0,46 | 0,653 | 1,17 |
| Lac\*HPMC\_PS | -2,56 | 3,97 | (-11,22; 6,09) | -0,65 | 0,531 | 1,42 |
| HPMC\_Visc\*HPMC\_HP | -1,86 | 2,93 | (-8,25; 4,52) | -0,64 | 0,536 | 2,73 |
| HPMC\_Visc\*HPMC\_PS | 9,84 | 4,14 | (0,83; 18,85) | 2,38 | 0,035 | 2,74 |
| HPMC\_HP\*HPMC\_PS | -2,15 | 4,57 | (-12,10; 7,80) | -0,47 | 0,646 | 2,70 |

## Model Summary

| S | R-sq | R-sq(adj) | PRESS | R-sq(pred) | AICc | BIC |
| --- | --- | --- | --- | --- | --- | --- |
| 2,51996 | 87,40% | 72,71% | 491,948 | 18,68% | 191,04 | 157,37 |

## Analysis of Variance

| Source | DF | Seq SS | Contribution | Adj SS | Adj MS | F-Value | P-Value |
| --- | --- | --- | --- | --- | --- | --- | --- |
| Model | 14 | 528,729 | 87,40% | 528,729 | 37,766 | 5,95 | 0,002 |
| Linear | 4 | 455,624 | 75,32% | 357,037 | 89,259 | 14,06 | 0,000 |
| Lac | 1 | 391,437 | 64,71% | 286,701 | 286,701 | 45,15 | 0,000 |
| HPMC\_Visc | 1 | 30,450 | 5,03% | 0,529 | 0,529 | 0,08 | 0,778 |
| HPMC\_HP | 1 | 21,785 | 3,60% | 18,270 | 18,270 | 2,88 | 0,116 |
| HPMC\_PS | 1 | 11,952 | 1,98% | 31,537 | 31,537 | 4,97 | 0,046 |
| Square | 4 | 7,562 | 1,25% | 20,438 | 5,110 | 0,80 | 0,545 |
| Lac\*Lac | 1 | 3,377 | 0,56% | 0,011 | 0,011 | 0,00 | 0,967 |
| HPMC\_Visc\*HPMC\_Visc | 1 | 0,803 | 0,13% | 10,632 | 10,632 | 1,67 | 0,220 |
| HPMC\_HP\*HPMC\_HP | 1 | 1,475 | 0,24% | 12,947 | 12,947 | 2,04 | 0,179 |
| HPMC\_PS\*HPMC\_PS | 1 | 1,907 | 0,32% | 2,441 | 2,441 | 0,38 | 0,547 |
| 2-Way Interaction | 6 | 65,542 | 10,83% | 65,542 | 10,924 | 1,72 | 0,200 |
| Lac\*HPMC\_Visc | 1 | 25,597 | 4,23% | 27,045 | 27,045 | 4,26 | 0,061 |
| Lac\*HPMC\_HP | 1 | 0,633 | 0,10% | 1,347 | 1,347 | 0,21 | 0,653 |
| Lac\*HPMC\_PS | 1 | 2,645 | 0,44% | 2,645 | 2,645 | 0,42 | 0,531 |
| HPMC\_Visc\*HPMC\_HP | 1 | 0,644 | 0,11% | 2,573 | 2,573 | 0,41 | 0,536 |
| HPMC\_Visc\*HPMC\_PS | 1 | 34,612 | 5,72% | 35,918 | 35,918 | 5,66 | 0,035 |
| HPMC\_HP\*HPMC\_PS | 1 | 1,411 | 0,23% | 1,411 | 1,411 | 0,22 | 0,646 |
| Error | 12 | 76,202 | 12,60% | 76,202 | 6,350 |  |  |
| Lack-of-Fit | 10 | 57,032 | 9,43% | 57,032 | 5,703 | 0,60 | 0,765 |
| Pure Error | 2 | 19,170 | 3,17% | 19,170 | 9,585 |  |  |
| Total | 26 | 604,932 | 100,00% |  |  |  |  |

## Regression Equation in Uncoded Units

|  |  |  |
| --- | --- | --- |
| F\_mean\_6h(360min) | = | 434 + 152 Lac - 0,0224 HPMC\_Visc - 32,9 HPMC\_HP - 4,25 HPMC\_PS + 1,5 Lac\*Lac + 0,000000 HPMC\_Visc\*HPMC\_Visc + 3,11 HPMC\_HP\*HPMC\_HP + 0,0256 HPMC\_PS\*HPMC\_PS - 0,00521 Lac\*HPMC\_Visc + 4,7 Lac\*HPMC\_HP - 1,39 Lac\*HPMC\_PS - 0,000472 HPMC\_Visc\*HPMC\_HP + 0,000344 HPMC\_Visc\*HPMC\_PS - 0,288 HPMC\_HP\*HPMC\_PS |

## Fits and Diagnostics for All Observations

| Obs | F\_mean\_6h(360min) | Fit | SE Fit | 95% CI | Resid | Std Resid | Del Resid | HI |
| --- | --- | --- | --- | --- | --- | --- | --- | --- |
| 1 | 48,90 | 47,68 | 2,04 | (43,23; 52,13) | 1,22 | 0,82 | 0,81 | 0,655994 |
| 2 | 57,28 | 58,58 | 2,04 | (54,14; 63,03) | -1,31 | -0,88 | -0,88 | 0,655994 |
| 3 | 43,50 | 45,13 | 2,15 | (40,44; 49,82) | -1,63 | -1,25 | -1,28 | 0,729945 |
| 4 | 49,90 | 49,76 | 2,15 | (45,07; 54,45) | 0,15 | 0,11 | 0,11 | 0,729945 |
| 5 | 45,73 | 48,65 | 1,74 | (44,86; 52,44) | -2,92 | -1,60 | -1,73 | 0,476813 |
| 6 | 57,66 | 60,14 | 1,74 | (56,35; 63,94) | -2,48 | -1,36 | -1,42 | 0,476813 |
| 7 | 46,73 | 47,50 | 2,09 | (42,95; 52,06) | -0,78 | -0,55 | -0,54 | 0,688750 |
| 8 | 51,68 | 52,99 | 2,09 | (48,43; 57,54) | -1,31 | -0,93 | -0,92 | 0,688750 |
| 9 | 47,79 | 47,61 | 1,92 | (43,43; 51,80) | 0,17 | 0,11 | 0,10 | 0,580949 |
| 10 | 57,12 | 56,77 | 1,92 | (52,58; 60,95) | 0,35 | 0,21 | 0,20 | 0,580949 |
| 11 | 50,00 | 50,36 | 1,64 | (46,77; 53,94) | -0,36 | -0,19 | -0,18 | 0,425781 |
| 12 | 56,14 | 55,81 | 1,64 | (52,22; 59,39) | 0,33 | 0,17 | 0,17 | 0,425781 |
| 13 | 47,76 | 48,42 | 1,90 | (44,28; 52,56) | -0,66 | -0,40 | -0,39 | 0,569258 |
| 14 | 59,76 | 58,64 | 1,90 | (54,50; 62,78) | 1,12 | 0,68 | 0,66 | 0,569258 |
| 15 | 50,89 | 51,03 | 1,66 | (47,42; 54,65) | -0,14 | -0,07 | -0,07 | 0,433227 |
| 16 | 53,40 | 56,64 | 1,66 | (53,03; 60,26) | -3,25 | -1,71 | -1,88 | 0,433227 |
| 17 | 44,23 | 43,12 | 1,95 | (38,88; 47,37) | 1,11 | 0,70 | 0,68 | 0,597775 |
| 18 | 61,87 | 60,11 | 1,95 | (55,87; 64,36) | 1,76 | 1,10 | 1,11 | 0,597775 |
| 19 | 54,96 | 54,15 | 1,61 | (50,65; 57,65) | 0,81 | 0,42 | 0,40 | 0,407038 |
| 20 | 55,19 | 52,40 | 2,03 | (47,98; 56,81) | 2,80 | 1,87 | 2,13 | 0,647412 |
| 21 | 51,57 | 52,19 | 1,61 | (48,69; 55,70) | -0,63 | -0,32 | -0,31 | 0,407933 |
| 22 | 58,92 | 57,02 | 2,28 | (52,06; 61,98) | 1,90 | 1,76 | 1,96 | 0,817044 |
| 23 | 52,92 | 51,54 | 2,29 | (46,56; 56,52) | 1,38 | 1,30 | 1,35 | 0,823434 |
| 24 | 54,10 | 54,54 | 2,13 | (49,90; 59,18) | -0,44 | -0,33 | -0,32 | 0,713775 |
| 25 | 50,29 | 51,52 | 1,35 | (48,57; 54,47) | -1,24 | -0,58 | -0,57 | 0,288793 |
| 26 | 51,08 | 51,52 | 1,35 | (48,57; 54,47) | -0,44 | -0,21 | -0,20 | 0,288793 |
| 27 | 56,00 | 51,52 | 1,35 | (48,57; 54,47) | 4,48 | 2,11 | 2,54 | 0,288793 |

| Obs | Cook’s D | DFITS |  |
| --- | --- | --- | --- |
| 1 | 0,09 | 1,12089 |  |
| 2 | 0,10 | -1,20969 |  |
| 3 | 0,28 | -2,10004 |  |
| 4 | 0,00 | 0,17482 |  |
| 5 | 0,16 | -1,65180 |  |
| 6 | 0,11 | -1,35255 |  |
| 7 | 0,04 | -0,79638 |  |
| 8 | 0,13 | -1,37381 |  |
| 9 | 0,00 | 0,12015 |  |
| 10 | 0,00 | 0,24052 |  |
| 11 | 0,00 | -0,15530 |  |
| 12 | 0,00 | 0,14365 |  |
| 13 | 0,01 | -0,44320 |  |
| 14 | 0,04 | 0,76249 |  |
| 15 | 0,00 | -0,06158 |  |
| 16 | 0,15 | -1,64675 |  |
| 17 | 0,05 | 0,82916 |  |
| 18 | 0,12 | 1,35539 |  |
| 19 | 0,01 | 0,33382 |  |
| 20 | 0,43 | 2,88088 |  |
| 21 | 0,00 | -0,25817 |  |
| 22 | 0,92 | 4,14153 |  |
| 23 | 0,53 | 2,91197 |  |
| 24 | 0,02 | -0,49909 |  |
| 25 | 0,01 | -0,36094 |  |
| 26 | 0,00 | -0,12692 |  |
| 27 | 0,12 | 1,61931 | R |

R  Large residual

## Coded Coefficients

| Term | Coef | SE Coef | 95% CI | T-Value | P-Value | VIF |
| --- | --- | --- | --- | --- | --- | --- |
| Constant | 56,19 | 1,35 | (53,24; 59,13) | 41,59 | 0,000 |  |
| Lac | 7,98 | 1,12 | (5,54; 10,41) | 7,14 | 0,000 | 1,18 |
| HPMC\_Visc | -0,57 | 1,17 | (-3,11; 1,98) | -0,49 | 0,635 | 1,70 |
| HPMC\_HP | 2,18 | 1,14 | (-0,31; 4,66) | 1,90 | 0,081 | 1,26 |
| HPMC\_PS | 3,56 | 1,75 | (-0,26; 7,37) | 2,03 | 0,065 | 2,09 |
| Lac\*Lac | 0,05 | 2,22 | (-4,79; 4,89) | 0,02 | 0,983 | 1,30 |
| HPMC\_Visc\*HPMC\_Visc | 2,90 | 2,36 | (-2,24; 8,04) | 1,23 | 0,243 | 1,96 |
| HPMC\_HP\*HPMC\_HP | 3,18 | 2,24 | (-1,70; 8,06) | 1,42 | 0,181 | 1,81 |
| HPMC\_PS\*HPMC\_PS | 1,50 | 2,23 | (-3,35; 6,35) | 0,67 | 0,513 | 1,42 |
| Lac\*HPMC\_Visc | -5,29 | 2,45 | (-10,63; 0,04) | -2,16 | 0,052 | 1,49 |
| Lac\*HPMC\_HP | 1,01 | 2,57 | (-4,59; 6,61) | 0,39 | 0,702 | 1,17 |
| Lac\*HPMC\_PS | -2,54 | 3,97 | (-11,19; 6,10) | -0,64 | 0,534 | 1,42 |
| HPMC\_Visc\*HPMC\_HP | -2,09 | 2,92 | (-8,46; 4,28) | -0,71 | 0,489 | 2,73 |
| HPMC\_Visc\*HPMC\_PS | 9,42 | 4,13 | (0,43; 18,42) | 2,28 | 0,042 | 2,74 |
| HPMC\_HP\*HPMC\_PS | -2,75 | 4,56 | (-12,68; 7,18) | -0,60 | 0,558 | 2,70 |

## Model Summary

| S | R-sq | R-sq(adj) | PRESS | R-sq(pred) | AICc | BIC |
| --- | --- | --- | --- | --- | --- | --- |
| 2,51540 | 88,54% | 75,17% | 503,014 | 24,06% | 190,94 | 157,27 |

## Analysis of Variance

| Source | DF | Seq SS | Contribution | Adj SS | Adj MS | F-Value | P-Value |
| --- | --- | --- | --- | --- | --- | --- | --- |
| Model | 14 | 586,480 | 88,54% | 586,480 | 41,891 | 6,62 | 0,001 |
| Linear | 4 | 513,762 | 77,56% | 394,860 | 98,715 | 15,60 | 0,000 |
| Lac | 1 | 442,639 | 66,82% | 322,648 | 322,648 | 50,99 | 0,000 |
| HPMC\_Visc | 1 | 32,892 | 4,97% | 1,500 | 1,500 | 0,24 | 0,635 |
| HPMC\_HP | 1 | 28,297 | 4,27% | 22,955 | 22,955 | 3,63 | 0,081 |
| HPMC\_PS | 1 | 9,934 | 1,50% | 26,078 | 26,078 | 4,12 | 0,065 |
| Square | 4 | 7,021 | 1,06% | 19,704 | 4,926 | 0,78 | 0,560 |
| Lac\*Lac | 1 | 3,575 | 0,54% | 0,003 | 0,003 | 0,00 | 0,983 |
| HPMC\_Visc\*HPMC\_Visc | 1 | 0,964 | 0,15% | 9,554 | 9,554 | 1,51 | 0,243 |
| HPMC\_HP\*HPMC\_HP | 1 | 1,255 | 0,19% | 12,752 | 12,752 | 2,02 | 0,181 |
| HPMC\_PS\*HPMC\_PS | 1 | 1,227 | 0,19% | 2,871 | 2,871 | 0,45 | 0,513 |
| 2-Way Interaction | 6 | 65,698 | 9,92% | 65,698 | 10,950 | 1,73 | 0,197 |
| Lac\*HPMC\_Visc | 1 | 28,693 | 4,33% | 29,548 | 29,548 | 4,67 | 0,052 |
| Lac\*HPMC\_HP | 1 | 0,384 | 0,06% | 0,972 | 0,972 | 0,15 | 0,702 |
| Lac\*HPMC\_PS | 1 | 2,599 | 0,39% | 2,599 | 2,599 | 0,41 | 0,534 |
| HPMC\_Visc\*HPMC\_HP | 1 | 0,596 | 0,09% | 3,225 | 3,225 | 0,51 | 0,489 |
| HPMC\_Visc\*HPMC\_PS | 1 | 31,126 | 4,70% | 32,945 | 32,945 | 5,21 | 0,042 |
| HPMC\_HP\*HPMC\_PS | 1 | 2,300 | 0,35% | 2,300 | 2,300 | 0,36 | 0,558 |
| Error | 12 | 75,927 | 11,46% | 75,927 | 6,327 |  |  |
| Lack-of-Fit | 10 | 57,390 | 8,66% | 57,390 | 5,739 | 0,62 | 0,753 |
| Pure Error | 2 | 18,537 | 2,80% | 18,537 | 9,268 |  |  |
| Total | 26 | 662,407 | 100,00% |  |  |  |  |

## Regression Equation in Uncoded Units

|  |  |  |
| --- | --- | --- |
| F\_mean\_7h(420min) | = | 368 + 163 Lac - 0,0205 HPMC\_Visc - 25,5 HPMC\_HP - 3,65 HPMC\_PS + 0,8 Lac\*Lac + 0,000000 HPMC\_Visc\*HPMC\_Visc + 3,09 HPMC\_HP\*HPMC\_HP + 0,0277 HPMC\_PS\*HPMC\_PS - 0,00544 Lac\*HPMC\_Visc + 4,0 Lac\*HPMC\_HP - 1,38 Lac\*HPMC\_PS - 0,000529 HPMC\_Visc\*HPMC\_HP + 0,000329 HPMC\_Visc\*HPMC\_PS - 0,368 HPMC\_HP\*HPMC\_PS |

## Fits and Diagnostics for All Observations

| Obs | F\_mean\_7h(420min) | Fit | SE Fit | 95% CI | Resid | Std Resid | Del Resid | HI |
| --- | --- | --- | --- | --- | --- | --- | --- | --- |
| 1 | 52,67 | 51,64 | 2,04 | (47,20; 56,08) | 1,03 | 0,70 | 0,68 | 0,655994 |
| 2 | 62,10 | 63,29 | 2,04 | (58,85; 67,73) | -1,19 | -0,81 | -0,79 | 0,655994 |
| 3 | 47,72 | 49,40 | 2,15 | (44,72; 54,08) | -1,68 | -1,29 | -1,33 | 0,729945 |
| 4 | 54,58 | 54,44 | 2,15 | (49,76; 59,12) | 0,14 | 0,11 | 0,10 | 0,729945 |
| 5 | 50,14 | 53,20 | 1,74 | (49,42; 56,99) | -3,06 | -1,68 | -1,84 | 0,476813 |
| 6 | 62,57 | 65,26 | 1,74 | (61,48; 69,04) | -2,69 | -1,48 | -1,57 | 0,476813 |
| 7 | 51,53 | 52,20 | 2,09 | (47,65; 56,75) | -0,68 | -0,48 | -0,47 | 0,688750 |
| 8 | 56,49 | 57,89 | 2,09 | (53,34; 62,44) | -1,40 | -1,00 | -1,00 | 0,688750 |
| 9 | 51,99 | 51,84 | 1,92 | (47,67; 56,02) | 0,15 | 0,09 | 0,09 | 0,580949 |
| 10 | 62,01 | 61,74 | 1,92 | (57,57; 65,92) | 0,27 | 0,16 | 0,16 | 0,580949 |
| 11 | 54,52 | 54,56 | 1,64 | (50,99; 58,14) | -0,04 | -0,02 | -0,02 | 0,425781 |
| 12 | 60,68 | 60,52 | 1,64 | (56,94; 64,09) | 0,16 | 0,08 | 0,08 | 0,425781 |
| 13 | 52,28 | 52,89 | 1,90 | (48,75; 57,02) | -0,60 | -0,37 | -0,35 | 0,569258 |
| 14 | 64,73 | 63,66 | 1,90 | (59,53; 67,80) | 1,07 | 0,65 | 0,63 | 0,569258 |
| 15 | 54,73 | 55,29 | 1,66 | (51,68; 58,89) | -0,55 | -0,29 | -0,28 | 0,433227 |
| 16 | 58,35 | 61,27 | 1,66 | (57,66; 64,87) | -2,91 | -1,54 | -1,64 | 0,433227 |
| 17 | 48,46 | 47,24 | 1,94 | (43,01; 51,48) | 1,22 | 0,76 | 0,75 | 0,597775 |
| 18 | 67,03 | 65,25 | 1,94 | (61,02; 69,49) | 1,78 | 1,12 | 1,13 | 0,597775 |
| 19 | 59,91 | 58,84 | 1,60 | (55,35; 62,34) | 1,07 | 0,55 | 0,54 | 0,407038 |
| 20 | 59,53 | 56,74 | 2,02 | (52,33; 61,15) | 2,78 | 1,86 | 2,12 | 0,647412 |
| 21 | 55,96 | 56,54 | 1,61 | (53,04; 60,05) | -0,58 | -0,30 | -0,29 | 0,407933 |
| 22 | 63,83 | 61,88 | 2,27 | (56,92; 66,83) | 1,95 | 1,82 | 2,04 | 0,817044 |
| 23 | 57,98 | 56,53 | 2,28 | (51,56; 61,51) | 1,45 | 1,37 | 1,43 | 0,823434 |
| 24 | 58,69 | 59,16 | 2,13 | (54,53; 63,79) | -0,47 | -0,35 | -0,33 | 0,713775 |
| 25 | 54,89 | 56,20 | 1,35 | (53,26; 59,15) | -1,31 | -0,62 | -0,60 | 0,288793 |
| 26 | 55,90 | 56,20 | 1,35 | (53,26; 59,15) | -0,30 | -0,14 | -0,13 | 0,288793 |
| 27 | 60,60 | 56,20 | 1,35 | (53,26; 59,15) | 4,40 | 2,07 | 2,48 | 0,288793 |

| Obs | Cook’s D | DFITS |  |
| --- | --- | --- | --- |
| 1 | 0,06 | 0,94077 |  |
| 2 | 0,08 | -1,09570 |  |
| 3 | 0,30 | -2,18232 |  |
| 4 | 0,00 | 0,17154 |  |
| 5 | 0,17 | -1,75778 |  |
| 6 | 0,13 | -1,49594 |  |
| 7 | 0,03 | -0,69303 |  |
| 8 | 0,15 | -1,48089 |  |
| 9 | 0,00 | 0,10512 |  |
| 10 | 0,00 | 0,18406 |  |
| 11 | 0,00 | -0,01851 |  |
| 12 | 0,00 | 0,06930 |  |
| 13 | 0,01 | -0,40440 |  |
| 14 | 0,04 | 0,72345 |  |
| 15 | 0,00 | -0,24610 |  |
| 16 | 0,12 | -1,43766 |  |
| 17 | 0,06 | 0,91489 |  |
| 18 | 0,12 | 1,37541 |  |
| 19 | 0,01 | 0,44409 |  |
| 20 | 0,43 | 2,86927 |  |
| 21 | 0,00 | -0,24100 |  |
| 22 | 0,98 | 4,31587 |  |
| 23 | 0,59 | 3,09059 |  |
| 24 | 0,02 | -0,52615 |  |
| 25 | 0,01 | -0,38250 |  |
| 26 | 0,00 | -0,08594 |  |
| 27 | 0,12 | 1,57798 | R |

R  Large residual

## Coded Coefficients

| Term | Coef | SE Coef | 95% CI | T-Value | P-Value | VIF |
| --- | --- | --- | --- | --- | --- | --- |
| Constant | 60,60 | 1,36 | (57,63; 63,57) | 44,48 | 0,000 |  |
| Lac | 8,33 | 1,13 | (5,88; 10,79) | 7,40 | 0,000 | 1,18 |
| HPMC\_Visc | -0,71 | 1,18 | (-3,28; 1,85) | -0,61 | 0,556 | 1,70 |
| HPMC\_HP | 2,36 | 1,15 | (-0,15; 4,87) | 2,05 | 0,063 | 1,26 |
| HPMC\_PS | 3,48 | 1,77 | (-0,37; 7,33) | 1,97 | 0,072 | 2,09 |
| Lac\*Lac | 0,03 | 2,24 | (-4,85; 4,91) | 0,01 | 0,990 | 1,30 |
| HPMC\_Visc\*HPMC\_Visc | 2,81 | 2,38 | (-2,38; 7,99) | 1,18 | 0,261 | 1,96 |
| HPMC\_HP\*HPMC\_HP | 3,04 | 2,26 | (-1,88; 7,96) | 1,35 | 0,203 | 1,81 |
| HPMC\_PS\*HPMC\_PS | 1,60 | 2,24 | (-3,29; 6,49) | 0,71 | 0,490 | 1,42 |
| Lac\*HPMC\_Visc | -5,33 | 2,47 | (-10,71; 0,05) | -2,16 | 0,052 | 1,49 |
| Lac\*HPMC\_HP | 1,11 | 2,59 | (-4,53; 6,76) | 0,43 | 0,675 | 1,17 |
| Lac\*HPMC\_PS | -2,59 | 4,00 | (-11,30; 6,13) | -0,65 | 0,530 | 1,42 |
| HPMC\_Visc\*HPMC\_HP | -2,01 | 2,95 | (-8,43; 4,41) | -0,68 | 0,509 | 2,73 |
| HPMC\_Visc\*HPMC\_PS | 9,56 | 4,16 | (0,49; 18,63) | 2,30 | 0,040 | 2,74 |
| HPMC\_HP\*HPMC\_PS | -2,56 | 4,59 | (-12,57; 7,45) | -0,56 | 0,588 | 2,70 |

## Model Summary

| S | R-sq | R-sq(adj) | PRESS | R-sq(pred) | AICc | BIC |
| --- | --- | --- | --- | --- | --- | --- |
| 2,53612 | 89,11% | 76,41% | 522,930 | 26,25% | 191,38 | 157,72 |

## Analysis of Variance

| Source | DF | Seq SS | Contribution | Adj SS | Adj MS | F-Value | P-Value |
| --- | --- | --- | --- | --- | --- | --- | --- |
| Model | 14 | 631,851 | 89,11% | 631,851 | 45,132 | 7,02 | 0,001 |
| Linear | 4 | 558,040 | 78,70% | 430,022 | 107,505 | 16,71 | 0,000 |
| Lac | 1 | 478,903 | 67,54% | 351,844 | 351,844 | 54,70 | 0,000 |
| HPMC\_Visc | 1 | 37,307 | 5,26% | 2,358 | 2,358 | 0,37 | 0,556 |
| HPMC\_HP | 1 | 33,265 | 4,69% | 26,936 | 26,936 | 4,19 | 0,063 |
| HPMC\_PS | 1 | 8,565 | 1,21% | 24,949 | 24,949 | 3,88 | 0,072 |
| Square | 4 | 6,823 | 0,96% | 18,484 | 4,621 | 0,72 | 0,595 |
| Lac\*Lac | 1 | 3,568 | 0,50% | 0,001 | 0,001 | 0,00 | 0,990 |
| HPMC\_Visc\*HPMC\_Visc | 1 | 1,375 | 0,19% | 8,963 | 8,963 | 1,39 | 0,261 |
| HPMC\_HP\*HPMC\_HP | 1 | 0,834 | 0,12% | 11,662 | 11,662 | 1,81 | 0,203 |
| HPMC\_PS\*HPMC\_PS | 1 | 1,046 | 0,15% | 3,266 | 3,266 | 0,51 | 0,490 |
| 2-Way Interaction | 6 | 66,988 | 9,45% | 66,988 | 11,165 | 1,74 | 0,196 |
| Lac\*HPMC\_Visc | 1 | 28,910 | 4,08% | 29,967 | 29,967 | 4,66 | 0,052 |
| Lac\*HPMC\_HP | 1 | 0,515 | 0,07% | 1,184 | 1,184 | 0,18 | 0,675 |
| Lac\*HPMC\_PS | 1 | 2,696 | 0,38% | 2,696 | 2,696 | 0,42 | 0,530 |
| HPMC\_Visc\*HPMC\_HP | 1 | 0,629 | 0,09% | 2,985 | 2,985 | 0,46 | 0,509 |
| HPMC\_Visc\*HPMC\_PS | 1 | 32,245 | 4,55% | 33,907 | 33,907 | 5,27 | 0,040 |
| HPMC\_HP\*HPMC\_PS | 1 | 1,992 | 0,28% | 1,992 | 1,992 | 0,31 | 0,588 |
| Error | 12 | 77,183 | 10,89% | 77,183 | 6,432 |  |  |
| Lack-of-Fit | 10 | 59,964 | 8,46% | 59,964 | 5,996 | 0,70 | 0,717 |
| Pure Error | 2 | 17,218 | 2,43% | 17,218 | 8,609 |  |  |
| Total | 26 | 709,033 | 100,00% |  |  |  |  |

## Regression Equation in Uncoded Units

|  |  |  |
| --- | --- | --- |
| F\_mean\_8h(480min) | = | 392 + 163 Lac - 0,0209 HPMC\_Visc - 25,1 HPMC\_HP - 4,21 HPMC\_PS + 0,5 Lac\*Lac + 0,000000 HPMC\_Visc\*HPMC\_Visc + 2,95 HPMC\_HP\*HPMC\_HP + 0,0296 HPMC\_PS\*HPMC\_PS - 0,00548 Lac\*HPMC\_Visc + 4,4 Lac\*HPMC\_HP - 1,41 Lac\*HPMC\_PS - 0,000509 HPMC\_Visc\*HPMC\_HP + 0,000334 HPMC\_Visc\*HPMC\_PS - 0,343 HPMC\_HP\*HPMC\_PS |

## Fits and Diagnostics for All Observations

| Obs | F\_mean\_8h(480min) | Fit | SE Fit | 95% CI | Resid | Std Resid | Del Resid | HI |
| --- | --- | --- | --- | --- | --- | --- | --- | --- |
| 1 | 56,98 | 55,87 | 2,05 | (51,40; 60,35) | 1,10 | 0,74 | 0,73 | 0,655994 |
| 2 | 66,57 | 67,84 | 2,05 | (63,36; 72,31) | -1,27 | -0,85 | -0,84 | 0,655994 |
| 3 | 51,65 | 53,38 | 2,17 | (48,66; 58,10) | -1,73 | -1,31 | -1,36 | 0,729945 |
| 4 | 58,93 | 58,70 | 2,17 | (53,97; 63,42) | 0,23 | 0,18 | 0,17 | 0,729945 |
| 5 | 54,34 | 57,51 | 1,75 | (53,69; 61,32) | -3,16 | -1,72 | -1,90 | 0,476813 |
| 6 | 67,11 | 69,97 | 1,75 | (66,16; 73,79) | -2,86 | -1,56 | -1,67 | 0,476813 |
| 7 | 55,61 | 56,32 | 2,10 | (51,73; 60,90) | -0,71 | -0,50 | -0,49 | 0,688750 |
| 8 | 61,03 | 62,40 | 2,10 | (57,81; 66,99) | -1,37 | -0,97 | -0,96 | 0,688750 |
| 9 | 55,84 | 55,90 | 1,93 | (51,69; 60,12) | -0,06 | -0,04 | -0,04 | 0,580949 |
| 10 | 66,54 | 66,09 | 1,93 | (61,88; 70,31) | 0,45 | 0,27 | 0,26 | 0,580949 |
| 11 | 58,96 | 58,65 | 1,65 | (55,04; 62,25) | 0,31 | 0,16 | 0,16 | 0,425781 |
| 12 | 64,88 | 64,89 | 1,65 | (61,28; 68,49) | -0,00 | -0,00 | -0,00 | 0,425781 |
| 13 | 56,55 | 57,16 | 1,91 | (52,99; 61,33) | -0,60 | -0,36 | -0,35 | 0,569258 |
| 14 | 69,33 | 68,33 | 1,91 | (64,16; 72,50) | 1,00 | 0,60 | 0,58 | 0,569258 |
| 15 | 58,88 | 59,51 | 1,67 | (55,87; 63,15) | -0,63 | -0,33 | -0,32 | 0,433227 |
| 16 | 62,99 | 65,84 | 1,67 | (62,20; 69,47) | -2,84 | -1,49 | -1,58 | 0,433227 |
| 17 | 52,52 | 51,30 | 1,96 | (47,03; 55,57) | 1,22 | 0,76 | 0,75 | 0,597775 |
| 18 | 71,83 | 70,02 | 1,96 | (65,75; 74,29) | 1,81 | 1,13 | 1,14 | 0,597775 |
| 19 | 64,58 | 63,28 | 1,62 | (59,75; 66,81) | 1,29 | 0,66 | 0,65 | 0,407038 |
| 20 | 63,57 | 60,87 | 2,04 | (56,42; 65,32) | 2,70 | 1,79 | 2,01 | 0,647412 |
| 21 | 60,02 | 60,71 | 1,62 | (57,18; 64,24) | -0,69 | -0,35 | -0,34 | 0,407933 |
| 22 | 68,38 | 66,34 | 2,29 | (61,35; 71,34) | 2,03 | 1,88 | 2,14 | 0,817044 |
| 23 | 62,68 | 61,19 | 2,30 | (56,18; 66,21) | 1,49 | 1,40 | 1,46 | 0,823434 |
| 24 | 63,03 | 63,53 | 2,14 | (58,86; 68,19) | -0,49 | -0,36 | -0,35 | 0,713775 |
| 25 | 59,29 | 60,63 | 1,36 | (57,66; 63,60) | -1,35 | -0,63 | -0,61 | 0,288793 |
| 26 | 60,51 | 60,63 | 1,36 | (57,66; 63,60) | -0,12 | -0,05 | -0,05 | 0,288793 |
| 27 | 64,87 | 60,63 | 1,36 | (57,66; 63,60) | 4,24 | 1,98 | 2,31 | 0,288793 |

| Obs | Cook’s D | DFITS |
| --- | --- | --- |
| 1 | 0,07 | 1,00505 |
| 2 | 0,09 | -1,16360 |
| 3 | 0,31 | -2,23453 |
| 4 | 0,01 | 0,27870 |
| 5 | 0,18 | -1,81592 |
| 6 | 0,15 | -1,59447 |
| 7 | 0,04 | -0,72502 |
| 8 | 0,14 | -1,43209 |
| 9 | 0,00 | -0,04216 |
| 10 | 0,01 | 0,30807 |
| 11 | 0,00 | 0,13364 |
| 12 | 0,00 | -0,00166 |
| 13 | 0,01 | -0,40211 |
| 14 | 0,03 | 0,67113 |
| 15 | 0,01 | -0,27754 |
| 16 | 0,11 | -1,38148 |
| 17 | 0,06 | 0,91083 |
| 18 | 0,13 | 1,39092 |
| 19 | 0,02 | 0,53590 |
| 20 | 0,39 | 2,71887 |
| 21 | 0,01 | -0,28247 |
| 22 | 1,05 | 4,51475 |
| 23 | 0,61 | 3,15258 |
| 24 | 0,02 | -0,55043 |
| 25 | 0,01 | -0,39032 |
| 26 | 0,00 | -0,03355 |
| 27 | 0,11 | 1,47384 |

## Coded Coefficients

| Term | Coef | SE Coef | 95% CI | T-Value | P-Value | VIF |
| --- | --- | --- | --- | --- | --- | --- |
| Constant | 64,70 | 1,43 | (61,58; 67,82) | 45,20 | 0,000 |  |
| Lac | 8,72 | 1,18 | (6,14; 11,29) | 7,36 | 0,000 | 1,18 |
| HPMC\_Visc | -0,82 | 1,24 | (-3,51; 1,88) | -0,66 | 0,521 | 1,70 |
| HPMC\_HP | 2,52 | 1,21 | (-0,11; 5,16) | 2,09 | 0,059 | 1,26 |
| HPMC\_PS | 3,33 | 1,86 | (-0,72; 7,37) | 1,79 | 0,098 | 2,09 |
| Lac\*Lac | 0,38 | 2,35 | (-4,74; 5,51) | 0,16 | 0,873 | 1,30 |
| HPMC\_Visc\*HPMC\_Visc | 2,73 | 2,50 | (-2,72; 8,18) | 1,09 | 0,297 | 1,96 |
| HPMC\_HP\*HPMC\_HP | 2,86 | 2,37 | (-2,31; 8,03) | 1,21 | 0,251 | 1,81 |
| HPMC\_PS\*HPMC\_PS | 1,70 | 2,36 | (-3,44; 6,83) | 0,72 | 0,485 | 1,42 |
| Lac\*HPMC\_Visc | -5,12 | 2,59 | (-10,77; 0,53) | -1,97 | 0,072 | 1,49 |
| Lac\*HPMC\_HP | 1,31 | 2,72 | (-4,62; 7,25) | 0,48 | 0,638 | 1,17 |
| Lac\*HPMC\_PS | -2,27 | 4,20 | (-11,43; 6,89) | -0,54 | 0,599 | 1,42 |
| HPMC\_Visc\*HPMC\_HP | -1,52 | 3,10 | (-8,27; 5,22) | -0,49 | 0,632 | 2,73 |
| HPMC\_Visc\*HPMC\_PS | 9,67 | 4,37 | (0,14; 19,20) | 2,21 | 0,047 | 2,74 |
| HPMC\_HP\*HPMC\_PS | -2,01 | 4,83 | (-12,53; 8,51) | -0,42 | 0,685 | 2,70 |

## Model Summary

| S | R-sq | R-sq(adj) | PRESS | R-sq(pred) | AICc | BIC |
| --- | --- | --- | --- | --- | --- | --- |
| 2,66483 | 88,72% | 75,55% | 574,523 | 23,92% | 194,06 | 160,39 |

## Analysis of Variance

| Source | DF | Seq SS | Contribution | Adj SS | Adj MS | F-Value | P-Value |
| --- | --- | --- | --- | --- | --- | --- | --- |
| Model | 14 | 669,967 | 88,72% | 669,967 | 47,855 | 6,74 | 0,001 |
| Linear | 4 | 598,428 | 79,24% | 466,462 | 116,616 | 16,42 | 0,000 |
| Lac | 1 | 514,461 | 68,12% | 385,015 | 385,015 | 54,22 | 0,000 |
| HPMC\_Visc | 1 | 41,697 | 5,52% | 3,112 | 3,112 | 0,44 | 0,521 |
| HPMC\_HP | 1 | 36,251 | 4,80% | 30,891 | 30,891 | 4,35 | 0,059 |
| HPMC\_PS | 1 | 6,019 | 0,80% | 22,800 | 22,800 | 3,21 | 0,098 |
| Square | 4 | 5,150 | 0,68% | 15,602 | 3,901 | 0,55 | 0,703 |
| Lac\*Lac | 1 | 1,933 | 0,26% | 0,189 | 0,189 | 0,03 | 0,873 |
| HPMC\_Visc\*HPMC\_Visc | 1 | 1,690 | 0,22% | 8,459 | 8,459 | 1,19 | 0,297 |
| HPMC\_HP\*HPMC\_HP | 1 | 0,589 | 0,08% | 10,328 | 10,328 | 1,45 | 0,251 |
| HPMC\_PS\*HPMC\_PS | 1 | 0,938 | 0,12% | 3,678 | 3,678 | 0,52 | 0,485 |
| 2-Way Interaction | 6 | 66,389 | 8,79% | 66,389 | 11,065 | 1,56 | 0,241 |
| Lac\*HPMC\_Visc | 1 | 27,345 | 3,62% | 27,645 | 27,645 | 3,89 | 0,072 |
| Lac\*HPMC\_HP | 1 | 0,939 | 0,12% | 1,650 | 1,650 | 0,23 | 0,638 |
| Lac\*HPMC\_PS | 1 | 2,074 | 0,27% | 2,074 | 2,074 | 0,29 | 0,599 |
| HPMC\_Visc\*HPMC\_HP | 1 | 1,279 | 0,17% | 1,717 | 1,717 | 0,24 | 0,632 |
| HPMC\_Visc\*HPMC\_PS | 1 | 33,526 | 4,44% | 34,686 | 34,686 | 4,88 | 0,047 |
| HPMC\_HP\*HPMC\_PS | 1 | 1,227 | 0,16% | 1,227 | 1,227 | 0,17 | 0,685 |
| Error | 12 | 85,216 | 11,28% | 85,216 | 7,101 |  |  |
| Lack-of-Fit | 10 | 68,258 | 9,04% | 68,258 | 6,826 | 0,81 | 0,670 |
| Pure Error | 2 | 16,958 | 2,25% | 16,958 | 8,479 |  |  |
| Total | 26 | 755,182 | 100,00% |  |  |  |  |

## Regression Equation in Uncoded Units

|  |  |  |
| --- | --- | --- |
| F\_mean\_9h(540min) | = | 468 + 137 Lac - 0,0223 HPMC\_Visc - 28,8 HPMC\_HP - 5,32 HPMC\_PS + 6,1 Lac\*Lac + 0,000000 HPMC\_Visc\*HPMC\_Visc + 2,78 HPMC\_HP\*HPMC\_HP + 0,0314 HPMC\_PS\*HPMC\_PS - 0,00526 Lac\*HPMC\_Visc + 5,2 Lac\*HPMC\_HP - 1,24 Lac\*HPMC\_PS - 0,000386 HPMC\_Visc\*HPMC\_HP + 0,000338 HPMC\_Visc\*HPMC\_PS - 0,269 HPMC\_HP\*HPMC\_PS |

## Fits and Diagnostics for All Observations

| Obs | F\_mean\_9h(540min) | Fit | SE Fit | 95% CI | Resid | Std Resid | Del Resid | HI |
| --- | --- | --- | --- | --- | --- | --- | --- | --- |
| 1 | 61,82 | 60,29 | 2,16 | (55,59; 64,99) | 1,53 | 0,98 | 0,98 | 0,655994 |
| 2 | 70,69 | 72,26 | 2,16 | (67,56; 76,97) | -1,57 | -1,01 | -1,01 | 0,655994 |
| 3 | 55,31 | 57,20 | 2,28 | (52,24; 62,16) | -1,88 | -1,36 | -1,42 | 0,729945 |
| 4 | 63,03 | 62,71 | 2,28 | (57,75; 67,67) | 0,32 | 0,23 | 0,22 | 0,729945 |
| 5 | 58,31 | 61,60 | 1,84 | (57,59; 65,61) | -3,29 | -1,71 | -1,88 | 0,476813 |
| 6 | 71,25 | 74,30 | 1,84 | (70,29; 78,31) | -3,05 | -1,58 | -1,70 | 0,476813 |
| 7 | 59,80 | 60,29 | 2,21 | (55,47; 65,11) | -0,48 | -0,33 | -0,31 | 0,688750 |
| 8 | 65,38 | 66,87 | 2,21 | (62,06; 71,69) | -1,49 | -1,00 | -1,01 | 0,688750 |
| 9 | 59,43 | 59,81 | 2,03 | (55,39; 64,24) | -0,38 | -0,22 | -0,21 | 0,580949 |
| 10 | 70,74 | 70,24 | 2,03 | (65,81; 74,66) | 0,50 | 0,29 | 0,28 | 0,580949 |
| 11 | 63,16 | 62,38 | 1,74 | (58,59; 66,16) | 0,78 | 0,39 | 0,37 | 0,425781 |
| 12 | 68,85 | 69,01 | 1,74 | (65,22; 72,80) | -0,16 | -0,08 | -0,07 | 0,425781 |
| 13 | 60,61 | 61,07 | 2,01 | (56,69; 65,45) | -0,45 | -0,26 | -0,25 | 0,569258 |
| 14 | 73,59 | 72,72 | 2,01 | (68,34; 77,11) | 0,87 | 0,50 | 0,48 | 0,569258 |
| 15 | 62,64 | 63,43 | 1,75 | (59,61; 67,25) | -0,79 | -0,39 | -0,38 | 0,433227 |
| 16 | 67,27 | 70,32 | 1,75 | (66,50; 74,14) | -3,05 | -1,52 | -1,62 | 0,433227 |
| 17 | 56,36 | 55,45 | 2,06 | (50,97; 59,94) | 0,91 | 0,54 | 0,52 | 0,597775 |
| 18 | 77,06 | 74,82 | 2,06 | (70,33; 79,31) | 2,24 | 1,33 | 1,37 | 0,597775 |
| 19 | 68,90 | 67,40 | 1,70 | (63,70; 71,11) | 1,49 | 0,73 | 0,71 | 0,407038 |
| 20 | 67,37 | 64,69 | 2,14 | (60,02; 69,36) | 2,68 | 1,69 | 1,86 | 0,647412 |
| 21 | 63,91 | 64,64 | 1,70 | (60,94; 68,35) | -0,74 | -0,36 | -0,35 | 0,407933 |
| 22 | 72,51 | 70,41 | 2,41 | (65,16; 75,66) | 2,10 | 1,84 | 2,09 | 0,817044 |
| 23 | 67,11 | 65,63 | 2,42 | (60,36; 70,90) | 1,47 | 1,32 | 1,36 | 0,823434 |
| 24 | 67,04 | 67,46 | 2,25 | (62,56; 72,37) | -0,42 | -0,29 | -0,28 | 0,713775 |
| 25 | 63,34 | 64,75 | 1,43 | (61,63; 67,87) | -1,41 | -0,63 | -0,61 | 0,288793 |
| 26 | 64,82 | 64,75 | 1,43 | (61,63; 67,87) | 0,06 | 0,03 | 0,03 | 0,288793 |
| 27 | 68,96 | 64,75 | 1,43 | (61,63; 67,87) | 4,21 | 1,87 | 2,13 | 0,288793 |

| Obs | Cook’s D | DFITS |
| --- | --- | --- |
| 1 | 0,12 | 1,34691 |
| 2 | 0,13 | -1,38930 |
| 3 | 0,33 | -2,32983 |
| 4 | 0,01 | 0,36524 |
| 5 | 0,18 | -1,79055 |
| 6 | 0,15 | -1,62361 |
| 7 | 0,02 | -0,46645 |
| 8 | 0,15 | -1,49554 |
| 9 | 0,00 | -0,24959 |
| 10 | 0,01 | 0,32802 |
| 11 | 0,01 | 0,32187 |
| 12 | 0,00 | -0,06449 |
| 13 | 0,01 | -0,28538 |
| 14 | 0,02 | 0,55220 |
| 15 | 0,01 | -0,33242 |
| 16 | 0,12 | -1,41872 |
| 17 | 0,03 | 0,63580 |
| 18 | 0,17 | 1,67612 |
| 19 | 0,02 | 0,58965 |
| 20 | 0,35 | 2,51957 |
| 21 | 0,01 | -0,28667 |
| 22 | 1,01 | 4,40719 |
| 23 | 0,54 | 2,94346 |
| 24 | 0,01 | -0,44542 |
| 25 | 0,01 | -0,38936 |
| 26 | 0,00 | 0,01756 |
| 27 | 0,09 | 1,35695 |

## Coded Coefficients

| Term | Coef | SE Coef | 95% CI | T-Value | P-Value | VIF |
| --- | --- | --- | --- | --- | --- | --- |
| Constant | 68,50 | 1,58 | (65,06; 71,94) | 43,40 | 0,000 |  |
| Lac | 9,42 | 1,31 | (6,58; 12,27) | 7,22 | 0,000 | 1,18 |
| HPMC\_Visc | -0,91 | 1,36 | (-3,89; 2,06) | -0,67 | 0,515 | 1,70 |
| HPMC\_HP | 2,61 | 1,33 | (-0,30; 5,51) | 1,95 | 0,074 | 1,26 |
| HPMC\_PS | 3,13 | 2,05 | (-1,32; 7,59) | 1,53 | 0,152 | 2,09 |
| Lac\*Lac | 1,79 | 2,59 | (-3,86; 7,45) | 0,69 | 0,502 | 1,30 |
| HPMC\_Visc\*HPMC\_Visc | 2,41 | 2,76 | (-3,60; 8,42) | 0,87 | 0,399 | 1,96 |
| HPMC\_HP\*HPMC\_HP | 2,51 | 2,62 | (-3,19; 8,21) | 0,96 | 0,356 | 1,81 |
| HPMC\_PS\*HPMC\_PS | 1,71 | 2,60 | (-3,96; 7,38) | 0,66 | 0,523 | 1,42 |
| Lac\*HPMC\_Visc | -5,32 | 2,86 | (-11,55; 0,91) | -1,86 | 0,088 | 1,49 |
| Lac\*HPMC\_HP | 1,49 | 3,00 | (-5,05; 8,03) | 0,50 | 0,629 | 1,17 |
| Lac\*HPMC\_PS | -2,61 | 4,63 | (-12,71; 7,49) | -0,56 | 0,584 | 1,42 |
| HPMC\_Visc\*HPMC\_HP | -1,34 | 3,42 | (-8,78; 6,10) | -0,39 | 0,701 | 2,73 |
| HPMC\_Visc\*HPMC\_PS | 9,62 | 4,82 | (-0,89; 20,13) | 2,00 | 0,069 | 2,74 |
| HPMC\_HP\*HPMC\_PS | -1,87 | 5,32 | (-13,47; 9,73) | -0,35 | 0,731 | 2,70 |

## Model Summary

| S | R-sq | R-sq(adj) | PRESS | R-sq(pred) | AICc | BIC |
| --- | --- | --- | --- | --- | --- | --- |
| 2,93868 | 87,98% | 73,95% | 694,648 | 19,40% | 199,34 | 165,67 |

## Analysis of Variance

| Source | DF | Seq SS | Contribution | Adj SS | Adj MS | F-Value | P-Value |
| --- | --- | --- | --- | --- | --- | --- | --- |
| Model | 14 | 758,199 | 87,98% | 758,199 | 54,157 | 6,27 | 0,001 |
| Linear | 4 | 685,518 | 79,54% | 531,959 | 132,990 | 15,40 | 0,000 |
| Lac | 1 | 597,032 | 69,27% | 450,150 | 450,150 | 52,13 | 0,000 |
| HPMC\_Visc | 1 | 44,050 | 5,11% | 3,878 | 3,878 | 0,45 | 0,515 |
| HPMC\_HP | 1 | 39,790 | 4,62% | 32,961 | 32,961 | 3,82 | 0,074 |
| HPMC\_PS | 1 | 4,646 | 0,54% | 20,256 | 20,256 | 2,35 | 0,152 |
| Square | 4 | 4,272 | 0,50% | 11,164 | 2,791 | 0,32 | 0,857 |
| Lac\*Lac | 1 | 0,350 | 0,04% | 4,131 | 4,131 | 0,48 | 0,502 |
| HPMC\_Visc\*HPMC\_Visc | 1 | 2,900 | 0,34% | 6,600 | 6,600 | 0,76 | 0,399 |
| HPMC\_HP\*HPMC\_HP | 1 | 0,109 | 0,01% | 7,945 | 7,945 | 0,92 | 0,356 |
| HPMC\_PS\*HPMC\_PS | 1 | 0,913 | 0,11% | 3,735 | 3,735 | 0,43 | 0,523 |
| 2-Way Interaction | 6 | 68,410 | 7,94% | 68,410 | 11,402 | 1,32 | 0,320 |
| Lac\*HPMC\_Visc | 1 | 28,431 | 3,30% | 29,860 | 29,860 | 3,46 | 0,088 |
| Lac\*HPMC\_HP | 1 | 1,201 | 0,14% | 2,127 | 2,127 | 0,25 | 0,629 |
| Lac\*HPMC\_PS | 1 | 2,735 | 0,32% | 2,735 | 2,735 | 0,32 | 0,584 |
| HPMC\_Visc\*HPMC\_HP | 1 | 1,634 | 0,19% | 1,337 | 1,337 | 0,15 | 0,701 |
| HPMC\_Visc\*HPMC\_PS | 1 | 33,342 | 3,87% | 34,374 | 34,374 | 3,98 | 0,069 |
| HPMC\_HP\*HPMC\_PS | 1 | 1,066 | 0,12% | 1,066 | 1,066 | 0,12 | 0,731 |
| Error | 12 | 103,630 | 12,02% | 103,630 | 8,636 |  |  |
| Lack-of-Fit | 10 | 87,329 | 10,13% | 87,329 | 8,733 | 1,07 | 0,575 |
| Pure Error | 2 | 16,301 | 1,89% | 16,301 | 8,151 |  |  |
| Total | 26 | 861,830 | 100,00% |  |  |  |  |

## Regression Equation in Uncoded Units

|  |  |  |
| --- | --- | --- |
| F\_mean\_10h(600min) | = | 456 + 126 Lac - 0,0220 HPMC\_Visc - 24,5 HPMC\_HP - 5,44 HPMC\_PS + 28,7 Lac\*Lac + 0,000000 HPMC\_Visc\*HPMC\_Visc + 2,44 HPMC\_HP\*HPMC\_HP + 0,0316 HPMC\_PS\*HPMC\_PS - 0,00547 Lac\*HPMC\_Visc + 5,9 Lac\*HPMC\_HP - 1,42 Lac\*HPMC\_PS - 0,000340 HPMC\_Visc\*HPMC\_HP + 0,000336 HPMC\_Visc\*HPMC\_PS - 0,251 HPMC\_HP\*HPMC\_PS |

## Fits and Diagnostics for All Observations

| Obs | F\_mean\_10h(600min) | Fit | SE Fit | 95% CI | Resid | Std Resid | Del Resid | HI |
| --- | --- | --- | --- | --- | --- | --- | --- | --- |
| 1 | 65,67 | 63,86 | 2,38 | (58,67; 69,05) | 1,81 | 1,05 | 1,05 | 0,655994 |
| 2 | 74,63 | 76,64 | 2,38 | (71,46; 81,83) | -2,01 | -1,17 | -1,19 | 0,655994 |
| 3 | 58,83 | 60,66 | 2,51 | (55,19; 66,13) | -1,83 | -1,20 | -1,22 | 0,729945 |
| 4 | 66,93 | 66,80 | 2,51 | (61,33; 72,27) | 0,12 | 0,08 | 0,08 | 0,729945 |
| 5 | 62,05 | 65,22 | 2,03 | (60,80; 69,64) | -3,17 | -1,49 | -1,58 | 0,476813 |
| 6 | 75,06 | 78,85 | 2,03 | (74,43; 83,27) | -3,79 | -1,78 | -1,99 | 0,476813 |
| 7 | 63,63 | 63,91 | 2,44 | (58,59; 69,22) | -0,28 | -0,17 | -0,16 | 0,688750 |
| 8 | 69,51 | 71,27 | 2,44 | (65,96; 76,59) | -1,77 | -1,08 | -1,09 | 0,688750 |
| 9 | 62,76 | 63,35 | 2,24 | (58,47; 68,23) | -0,59 | -0,31 | -0,30 | 0,580949 |
| 10 | 74,79 | 74,36 | 2,24 | (69,48; 79,24) | 0,44 | 0,23 | 0,22 | 0,580949 |
| 11 | 67,12 | 66,05 | 1,92 | (61,87; 70,23) | 1,07 | 0,48 | 0,47 | 0,425781 |
| 12 | 72,64 | 73,20 | 1,92 | (69,02; 77,38) | -0,56 | -0,25 | -0,24 | 0,425781 |
| 13 | 64,44 | 64,63 | 2,22 | (59,80; 69,46) | -0,18 | -0,10 | -0,09 | 0,569258 |
| 14 | 77,49 | 77,03 | 2,22 | (72,20; 81,87) | 0,46 | 0,24 | 0,23 | 0,569258 |
| 15 | 66,38 | 67,20 | 1,93 | (62,99; 71,42) | -0,82 | -0,37 | -0,36 | 0,433227 |
| 16 | 71,30 | 74,68 | 1,93 | (70,47; 78,90) | -3,38 | -1,53 | -1,63 | 0,433227 |
| 17 | 60,11 | 59,93 | 2,27 | (54,98; 64,88) | 0,18 | 0,10 | 0,09 | 0,597775 |
| 18 | 84,23 | 80,79 | 2,27 | (75,84; 85,74) | 3,44 | 1,84 | 2,09 | 0,597775 |
| 19 | 72,93 | 70,97 | 1,87 | (66,89; 75,06) | 1,96 | 0,86 | 0,85 | 0,407038 |
| 20 | 70,91 | 68,06 | 2,36 | (62,91; 73,21) | 2,85 | 1,63 | 1,77 | 0,647412 |
| 21 | 67,57 | 68,08 | 1,88 | (63,99; 72,17) | -0,52 | -0,23 | -0,22 | 0,407933 |
| 22 | 76,19 | 73,93 | 2,66 | (68,14; 79,71) | 2,26 | 1,80 | 2,02 | 0,817044 |
| 23 | 71,17 | 69,64 | 2,67 | (63,83; 75,45) | 1,54 | 1,24 | 1,28 | 0,823434 |
| 24 | 70,77 | 71,06 | 2,48 | (65,65; 76,47) | -0,29 | -0,18 | -0,18 | 0,713775 |
| 25 | 67,14 | 68,57 | 1,58 | (65,12; 72,01) | -1,42 | -0,57 | -0,56 | 0,288793 |
| 26 | 68,89 | 68,57 | 1,58 | (65,12; 72,01) | 0,33 | 0,13 | 0,13 | 0,288793 |
| 27 | 72,73 | 68,57 | 1,58 | (65,12; 72,01) | 4,16 | 1,68 | 1,84 | 0,288793 |

| Obs | Cook’s D | DFITS |
| --- | --- | --- |
| 1 | 0,14 | 1,45299 |
| 2 | 0,17 | -1,63733 |
| 3 | 0,26 | -2,01363 |
| 4 | 0,00 | 0,12734 |
| 5 | 0,13 | -1,50767 |
| 6 | 0,19 | -1,89909 |
| 7 | 0,00 | -0,24424 |
| 8 | 0,17 | -1,61450 |
| 9 | 0,01 | -0,34829 |
| 10 | 0,00 | 0,25836 |
| 11 | 0,01 | 0,40116 |
| 12 | 0,00 | -0,20911 |
| 13 | 0,00 | -0,10543 |
| 14 | 0,00 | 0,26183 |
| 15 | 0,01 | -0,31103 |
| 16 | 0,12 | -1,42602 |
| 17 | 0,00 | 0,11521 |
| 18 | 0,34 | 2,54199 |
| 19 | 0,03 | 0,70793 |
| 20 | 0,33 | 2,39989 |
| 21 | 0,00 | -0,18238 |
| 22 | 0,96 | 4,26180 |
| 23 | 0,48 | 2,75795 |
| 24 | 0,01 | -0,27911 |
| 25 | 0,01 | -0,35503 |
| 26 | 0,00 | 0,08088 |
| 27 | 0,08 | 1,17066 |

## Coded Coefficients

| Term | Coef | SE Coef | 95% CI | T-Value | P-Value | VIF |
| --- | --- | --- | --- | --- | --- | --- |
| Constant | 72,16 | 1,59 | (68,69; 75,63) | 45,31 | 0,000 |  |
| Lac | 9,69 | 1,32 | (6,82; 12,56) | 7,36 | 0,000 | 1,18 |
| HPMC\_Visc | -0,99 | 1,38 | (-3,98; 2,01) | -0,72 | 0,488 | 1,70 |
| HPMC\_HP | 2,75 | 1,35 | (-0,18; 5,69) | 2,05 | 0,063 | 1,26 |
| HPMC\_PS | 3,15 | 2,06 | (-1,34; 7,65) | 1,53 | 0,152 | 2,09 |
| Lac\*Lac | 2,09 | 2,62 | (-3,62; 7,79) | 0,80 | 0,441 | 1,30 |
| HPMC\_Visc\*HPMC\_Visc | 2,29 | 2,78 | (-3,77; 8,36) | 0,82 | 0,426 | 1,96 |
| HPMC\_HP\*HPMC\_HP | 2,14 | 2,64 | (-3,61; 7,89) | 0,81 | 0,433 | 1,81 |
| HPMC\_PS\*HPMC\_PS | 1,66 | 2,62 | (-4,06; 7,37) | 0,63 | 0,540 | 1,42 |
| Lac\*HPMC\_Visc | -5,62 | 2,89 | (-11,91; 0,67) | -1,95 | 0,075 | 1,49 |
| Lac\*HPMC\_HP | 1,43 | 3,03 | (-5,17; 8,03) | 0,47 | 0,645 | 1,17 |
| Lac\*HPMC\_PS | -2,60 | 4,68 | (-12,79; 7,59) | -0,56 | 0,589 | 1,42 |
| HPMC\_Visc\*HPMC\_HP | -1,04 | 3,45 | (-8,55; 6,47) | -0,30 | 0,767 | 2,73 |
| HPMC\_Visc\*HPMC\_PS | 9,07 | 4,87 | (-1,53; 19,67) | 1,86 | 0,087 | 2,74 |
| HPMC\_HP\*HPMC\_PS | -1,26 | 5,37 | (-12,96; 10,45) | -0,23 | 0,819 | 2,70 |

## Model Summary

| S | R-sq | R-sq(adj) | PRESS | R-sq(pred) | AICc | BIC |
| --- | --- | --- | --- | --- | --- | --- |
| 2,96495 | 88,40% | 74,86% | 699,512 | 23,07% | 199,82 | 166,15 |

## Analysis of Variance

| Source | DF | Seq SS | Contribution | Adj SS | Adj MS | F-Value | P-Value |
| --- | --- | --- | --- | --- | --- | --- | --- |
| Model | 14 | 803,735 | 88,40% | 803,735 | 57,410 | 6,53 | 0,001 |
| Linear | 4 | 731,154 | 80,42% | 564,028 | 141,007 | 16,04 | 0,000 |
| Lac | 1 | 635,132 | 69,85% | 475,840 | 475,840 | 54,13 | 0,000 |
| HPMC\_Visc | 1 | 45,887 | 5,05% | 4,507 | 4,507 | 0,51 | 0,488 |
| HPMC\_HP | 1 | 44,933 | 4,94% | 36,786 | 36,786 | 4,18 | 0,063 |
| HPMC\_PS | 1 | 5,202 | 0,57% | 20,519 | 20,519 | 2,33 | 0,152 |
| Square | 4 | 4,284 | 0,47% | 10,104 | 2,526 | 0,29 | 0,881 |
| Lac\*Lac | 1 | 1,230 | 0,14% | 5,583 | 5,583 | 0,64 | 0,441 |
| HPMC\_Visc\*HPMC\_Visc | 1 | 2,339 | 0,26% | 5,980 | 5,980 | 0,68 | 0,426 |
| HPMC\_HP\*HPMC\_HP | 1 | 0,010 | 0,00% | 5,774 | 5,774 | 0,66 | 0,433 |
| HPMC\_PS\*HPMC\_PS | 1 | 0,705 | 0,08% | 3,500 | 3,500 | 0,40 | 0,540 |
| 2-Way Interaction | 6 | 68,297 | 7,51% | 68,297 | 11,383 | 1,29 | 0,330 |
| Lac\*HPMC\_Visc | 1 | 32,505 | 3,58% | 33,313 | 33,313 | 3,79 | 0,075 |
| Lac\*HPMC\_HP | 1 | 1,081 | 0,12% | 1,966 | 1,966 | 0,22 | 0,645 |
| Lac\*HPMC\_PS | 1 | 2,712 | 0,30% | 2,712 | 2,712 | 0,31 | 0,589 |
| HPMC\_Visc\*HPMC\_HP | 1 | 1,451 | 0,16% | 0,804 | 0,804 | 0,09 | 0,767 |
| HPMC\_Visc\*HPMC\_PS | 1 | 30,067 | 3,31% | 30,537 | 30,537 | 3,47 | 0,087 |
| HPMC\_HP\*HPMC\_PS | 1 | 0,481 | 0,05% | 0,481 | 0,481 | 0,05 | 0,819 |
| Error | 12 | 105,491 | 11,60% | 105,491 | 8,791 |  |  |
| Lack-of-Fit | 10 | 90,258 | 9,93% | 90,258 | 9,026 | 1,19 | 0,541 |
| Pure Error | 2 | 15,233 | 1,68% | 15,233 | 7,617 |  |  |
| Total | 26 | 909,226 | 100,00% |  |  |  |  |

## Regression Equation in Uncoded Units

|  |  |  |
| --- | --- | --- |
| F\_mean\_11h(660min) | = | 463 + 128 Lac - 0,0210 HPMC\_Visc - 24,2 HPMC\_HP - 5,82 HPMC\_PS + 33,4 Lac\*Lac + 0,000000 HPMC\_Visc\*HPMC\_Visc + 2,08 HPMC\_HP\*HPMC\_HP + 0,0306 HPMC\_PS\*HPMC\_PS - 0,00578 Lac\*HPMC\_Visc + 5,6 Lac\*HPMC\_HP - 1,41 Lac\*HPMC\_PS - 0,000264 HPMC\_Visc\*HPMC\_HP + 0,000317 HPMC\_Visc\*HPMC\_PS - 0,168 HPMC\_HP\*HPMC\_PS |

## Fits and Diagnostics for All Observations

| Obs | F\_mean\_11h(660min) | Fit | SE Fit | 95% CI | Resid | Std Resid | Del Resid | HI |
| --- | --- | --- | --- | --- | --- | --- | --- | --- |
| 1 | 68,86 | 67,18 | 2,40 | (61,95; 72,41) | 1,68 | 0,97 | 0,96 | 0,655994 |
| 2 | 78,48 | 80,48 | 2,40 | (75,24; 85,71) | -1,99 | -1,15 | -1,16 | 0,655994 |
| 3 | 62,20 | 64,21 | 2,53 | (58,69; 69,73) | -2,01 | -1,30 | -1,35 | 0,729945 |
| 4 | 70,58 | 70,45 | 2,53 | (64,93; 75,97) | 0,13 | 0,08 | 0,08 | 0,729945 |
| 5 | 65,58 | 68,60 | 2,05 | (64,14; 73,06) | -3,02 | -1,41 | -1,47 | 0,476813 |
| 6 | 78,49 | 82,68 | 2,05 | (78,22; 87,14) | -4,19 | -1,95 | -2,26 | 0,476813 |
| 7 | 67,32 | 67,62 | 2,46 | (62,26; 72,98) | -0,30 | -0,18 | -0,17 | 0,688750 |
| 8 | 73,44 | 75,01 | 2,46 | (69,65; 80,37) | -1,57 | -0,95 | -0,94 | 0,688750 |
| 9 | 65,94 | 66,66 | 2,26 | (61,74; 71,59) | -0,73 | -0,38 | -0,36 | 0,580949 |
| 10 | 78,64 | 78,19 | 2,26 | (73,27; 83,11) | 0,45 | 0,24 | 0,23 | 0,580949 |
| 11 | 70,91 | 69,38 | 1,93 | (65,16; 73,59) | 1,53 | 0,68 | 0,66 | 0,425781 |
| 12 | 76,19 | 76,76 | 1,93 | (72,55; 80,98) | -0,57 | -0,26 | -0,24 | 0,425781 |
| 13 | 68,14 | 68,36 | 2,24 | (63,48; 73,23) | -0,21 | -0,11 | -0,10 | 0,569258 |
| 14 | 82,05 | 81,22 | 2,24 | (76,35; 86,09) | 0,83 | 0,43 | 0,41 | 0,569258 |
| 15 | 70,11 | 70,83 | 1,95 | (66,57; 75,08) | -0,71 | -0,32 | -0,31 | 0,433227 |
| 16 | 75,05 | 78,48 | 1,95 | (74,23; 82,73) | -3,43 | -1,54 | -1,64 | 0,433227 |
| 17 | 63,67 | 63,55 | 2,29 | (58,55; 68,54) | 0,12 | 0,06 | 0,06 | 0,597775 |
| 18 | 88,47 | 85,06 | 2,29 | (80,06; 90,05) | 3,41 | 1,81 | 2,04 | 0,597775 |
| 19 | 76,75 | 74,64 | 1,89 | (70,52; 78,76) | 2,11 | 0,92 | 0,92 | 0,407038 |
| 20 | 74,25 | 71,52 | 2,39 | (66,32; 76,72) | 2,73 | 1,55 | 1,66 | 0,647412 |
| 21 | 70,99 | 71,33 | 1,89 | (67,21; 75,46) | -0,34 | -0,15 | -0,14 | 0,407933 |
| 22 | 79,48 | 77,36 | 2,68 | (71,53; 83,20) | 2,12 | 1,67 | 1,83 | 0,817044 |
| 23 | 74,80 | 73,12 | 2,69 | (67,25; 78,98) | 1,69 | 1,35 | 1,41 | 0,823434 |
| 24 | 74,21 | 74,71 | 2,50 | (69,25; 80,16) | -0,49 | -0,31 | -0,30 | 0,713775 |
| 25 | 70,68 | 72,22 | 1,59 | (68,74; 75,69) | -1,53 | -0,61 | -0,60 | 0,288793 |
| 26 | 72,61 | 72,22 | 1,59 | (68,74; 75,69) | 0,40 | 0,16 | 0,15 | 0,288793 |
| 27 | 76,13 | 72,22 | 1,59 | (68,74; 75,69) | 3,91 | 1,56 | 1,68 | 0,288793 |

| Obs | Cook’s D | DFITS |
| --- | --- | --- |
| 1 | 0,12 | 1,32994 |
| 2 | 0,17 | -1,60704 |
| 3 | 0,31 | -2,21574 |
| 4 | 0,00 | 0,13323 |
| 5 | 0,12 | -1,40810 |
| 6 | 0,23 | -2,16062 |
| 7 | 0,00 | -0,25812 |
| 8 | 0,13 | -1,40268 |
| 9 | 0,01 | -0,42938 |
| 10 | 0,01 | 0,26573 |
| 11 | 0,02 | 0,57254 |
| 12 | 0,00 | -0,21096 |
| 13 | 0,00 | -0,12053 |
| 14 | 0,02 | 0,47361 |
| 15 | 0,01 | -0,26820 |
| 16 | 0,12 | -1,43627 |
| 17 | 0,00 | 0,07505 |
| 18 | 0,33 | 2,48115 |
| 19 | 0,04 | 0,76070 |
| 20 | 0,29 | 2,24887 |
| 21 | 0,00 | -0,12006 |
| 22 | 0,83 | 3,85807 |
| 23 | 0,57 | 3,04334 |
| 24 | 0,02 | -0,47123 |
| 25 | 0,01 | -0,38001 |
| 26 | 0,00 | 0,09698 |
| 27 | 0,07 | 1,06934 |

## Coded Coefficients

| Term | Coef | SE Coef | 95% CI | T-Value | P-Value | VIF |
| --- | --- | --- | --- | --- | --- | --- |
| Constant | 75,61 | 1,55 | (72,23; 79,00) | 48,69 | 0,000 |  |
| Lac | 9,71 | 1,28 | (6,91; 12,51) | 7,56 | 0,000 | 1,18 |
| HPMC\_Visc | -1,08 | 1,34 | (-4,00; 1,84) | -0,80 | 0,437 | 1,70 |
| HPMC\_HP | 2,78 | 1,31 | (-0,08; 5,64) | 2,12 | 0,056 | 1,26 |
| HPMC\_PS | 3,17 | 2,01 | (-1,22; 7,56) | 1,57 | 0,141 | 2,09 |
| Lac\*Lac | 1,76 | 2,55 | (-3,80; 7,32) | 0,69 | 0,504 | 1,30 |
| HPMC\_Visc\*HPMC\_Visc | 2,20 | 2,71 | (-3,71; 8,11) | 0,81 | 0,433 | 1,96 |
| HPMC\_HP\*HPMC\_HP | 1,80 | 2,57 | (-3,80; 7,41) | 0,70 | 0,497 | 1,81 |
| HPMC\_PS\*HPMC\_PS | 1,51 | 2,56 | (-4,06; 7,09) | 0,59 | 0,565 | 1,42 |
| Lac\*HPMC\_Visc | -5,60 | 2,81 | (-11,74; 0,53) | -1,99 | 0,070 | 1,49 |
| Lac\*HPMC\_HP | 1,13 | 2,95 | (-5,31; 7,57) | 0,38 | 0,709 | 1,17 |
| Lac\*HPMC\_PS | -2,05 | 4,56 | (-11,99; 7,88) | -0,45 | 0,661 | 1,42 |
| HPMC\_Visc\*HPMC\_HP | -0,74 | 3,36 | (-8,06; 6,58) | -0,22 | 0,830 | 2,73 |
| HPMC\_Visc\*HPMC\_PS | 8,35 | 4,75 | (-1,99; 18,69) | 1,76 | 0,104 | 2,74 |
| HPMC\_HP\*HPMC\_PS | -0,77 | 5,24 | (-12,19; 10,64) | -0,15 | 0,885 | 2,70 |

## Model Summary

| S | R-sq | R-sq(adj) | PRESS | R-sq(pred) | AICc | BIC |
| --- | --- | --- | --- | --- | --- | --- |
| 2,89104 | 88,99% | 76,14% | 667,173 | 26,75% | 198,45 | 164,79 |

## Analysis of Variance

| Source | DF | Seq SS | Contribution | Adj SS | Adj MS | F-Value | P-Value |
| --- | --- | --- | --- | --- | --- | --- | --- |
| Model | 14 | 810,475 | 88,99% | 810,475 | 57,891 | 6,93 | 0,001 |
| Linear | 4 | 742,961 | 81,57% | 570,251 | 142,563 | 17,06 | 0,000 |
| Lac | 1 | 642,010 | 70,49% | 478,104 | 478,104 | 57,20 | 0,000 |
| HPMC\_Visc | 1 | 48,372 | 5,31% | 5,413 | 5,413 | 0,65 | 0,437 |
| HPMC\_HP | 1 | 46,356 | 5,09% | 37,494 | 37,494 | 4,49 | 0,056 |
| HPMC\_PS | 1 | 6,223 | 0,68% | 20,714 | 20,714 | 2,48 | 0,141 |
| Square | 4 | 2,868 | 0,31% | 8,022 | 2,005 | 0,24 | 0,910 |
| Lac\*Lac | 1 | 0,706 | 0,08% | 3,967 | 3,967 | 0,47 | 0,504 |
| HPMC\_Visc\*HPMC\_Visc | 1 | 1,533 | 0,17% | 5,505 | 5,505 | 0,66 | 0,433 |
| HPMC\_HP\*HPMC\_HP | 1 | 0,002 | 0,00% | 4,107 | 4,107 | 0,49 | 0,497 |
| HPMC\_PS\*HPMC\_PS | 1 | 0,628 | 0,07% | 2,921 | 2,921 | 0,35 | 0,565 |
| 2-Way Interaction | 6 | 64,646 | 7,10% | 64,646 | 10,774 | 1,29 | 0,332 |
| Lac\*HPMC\_Visc | 1 | 34,905 | 3,83% | 33,111 | 33,111 | 3,96 | 0,070 |
| Lac\*HPMC\_HP | 1 | 0,671 | 0,07% | 1,223 | 1,223 | 0,15 | 0,709 |
| Lac\*HPMC\_PS | 1 | 1,693 | 0,19% | 1,693 | 1,693 | 0,20 | 0,661 |
| HPMC\_Visc\*HPMC\_HP | 1 | 1,383 | 0,15% | 0,404 | 0,404 | 0,05 | 0,830 |
| HPMC\_Visc\*HPMC\_PS | 1 | 25,811 | 2,83% | 25,896 | 25,896 | 3,10 | 0,104 |
| HPMC\_HP\*HPMC\_PS | 1 | 0,182 | 0,02% | 0,182 | 0,182 | 0,02 | 0,885 |
| Error | 12 | 100,297 | 11,01% | 100,297 | 8,358 |  |  |
| Lack-of-Fit | 10 | 85,881 | 9,43% | 85,881 | 8,588 | 1,19 | 0,540 |
| Pure Error | 2 | 14,416 | 1,58% | 14,416 | 7,208 |  |  |
| Total | 26 | 910,772 | 100,00% |  |  |  |  |

## Regression Equation in Uncoded Units

|  |  |  |
| --- | --- | --- |
| F\_mean\_12h(720min) | = | 456 + 124 Lac - 0,0199 HPMC\_Visc - 22,9 HPMC\_HP - 5,87 HPMC\_PS + 28,1 Lac\*Lac + 0,000000 HPMC\_Visc\*HPMC\_Visc + 1,75 HPMC\_HP\*HPMC\_HP + 0,0280 HPMC\_PS\*HPMC\_PS - 0,00576 Lac\*HPMC\_Visc + 4,5 Lac\*HPMC\_HP - 1,12 Lac\*HPMC\_PS - 0,000187 HPMC\_Visc\*HPMC\_HP + 0,000292 HPMC\_Visc\*HPMC\_PS - 0,104 HPMC\_HP\*HPMC\_PS |

## Fits and Diagnostics for All Observations

| Obs | F\_mean\_12h(720min) | Fit | SE Fit | 95% CI | Resid | Std Resid | Del Resid | HI |
| --- | --- | --- | --- | --- | --- | --- | --- | --- |
| 1 | 72,06 | 70,44 | 2,34 | (65,34; 75,54) | 1,62 | 0,96 | 0,95 | 0,655994 |
| 2 | 81,93 | 83,83 | 2,34 | (78,73; 88,93) | -1,91 | -1,12 | -1,14 | 0,655994 |
| 3 | 65,37 | 67,58 | 2,47 | (62,20; 72,96) | -2,21 | -1,47 | -1,55 | 0,729945 |
| 4 | 74,08 | 73,77 | 2,47 | (68,38; 79,15) | 0,31 | 0,21 | 0,20 | 0,729945 |
| 5 | 68,88 | 71,92 | 2,00 | (67,57; 76,27) | -3,04 | -1,45 | -1,53 | 0,476813 |
| 6 | 81,72 | 85,85 | 2,00 | (81,50; 90,20) | -4,14 | -1,98 | -2,31 | 0,476813 |
| 7 | 70,73 | 71,17 | 2,40 | (65,94; 76,39) | -0,44 | -0,27 | -0,26 | 0,688750 |
| 8 | 77,00 | 78,23 | 2,40 | (73,01; 83,46) | -1,24 | -0,77 | -0,75 | 0,688750 |
| 9 | 68,90 | 69,86 | 2,20 | (65,06; 74,66) | -0,96 | -0,51 | -0,50 | 0,580949 |
| 10 | 82,58 | 81,83 | 2,20 | (77,03; 86,63) | 0,74 | 0,40 | 0,38 | 0,580949 |
| 11 | 74,43 | 72,39 | 1,89 | (68,28; 76,50) | 2,04 | 0,93 | 0,92 | 0,425781 |
| 12 | 79,56 | 80,07 | 1,89 | (75,96; 84,18) | -0,51 | -0,23 | -0,22 | 0,425781 |
| 13 | 71,59 | 71,87 | 2,18 | (67,12; 76,62) | -0,28 | -0,15 | -0,14 | 0,569258 |
| 14 | 86,15 | 84,90 | 2,18 | (80,15; 89,66) | 1,24 | 0,65 | 0,64 | 0,569258 |
| 15 | 73,46 | 74,08 | 1,90 | (69,93; 78,23) | -0,62 | -0,28 | -0,27 | 0,433227 |
| 16 | 78,59 | 81,86 | 1,90 | (77,71; 86,00) | -3,27 | -1,50 | -1,60 | 0,433227 |
| 17 | 67,03 | 66,67 | 2,24 | (61,80; 71,54) | 0,36 | 0,20 | 0,19 | 0,597775 |
| 18 | 91,02 | 88,22 | 2,24 | (83,35; 93,09) | 2,80 | 1,53 | 1,63 | 0,597775 |
| 19 | 80,23 | 78,18 | 1,84 | (74,16; 82,20) | 2,05 | 0,92 | 0,92 | 0,407038 |
| 20 | 77,31 | 74,83 | 2,33 | (69,77; 79,90) | 2,48 | 1,44 | 1,52 | 0,647412 |
| 21 | 74,10 | 74,53 | 1,85 | (70,51; 78,56) | -0,43 | -0,19 | -0,19 | 0,407933 |
| 22 | 82,47 | 80,52 | 2,61 | (74,83; 86,21) | 1,95 | 1,58 | 1,70 | 0,817044 |
| 23 | 78,08 | 76,29 | 2,62 | (70,57; 82,00) | 1,79 | 1,48 | 1,56 | 0,823434 |
| 24 | 77,41 | 78,17 | 2,44 | (72,84; 83,49) | -0,76 | -0,49 | -0,48 | 0,713775 |
| 25 | 74,01 | 75,69 | 1,55 | (72,30; 79,07) | -1,67 | -0,69 | -0,67 | 0,288793 |
| 26 | 76,10 | 75,69 | 1,55 | (72,30; 79,07) | 0,42 | 0,17 | 0,16 | 0,288793 |
| 27 | 79,34 | 75,69 | 1,55 | (72,30; 79,07) | 3,66 | 1,50 | 1,59 | 0,288793 |

| Obs | Cook’s D | DFITS |
| --- | --- | --- |
| 1 | 0,12 | 1,31465 |
| 2 | 0,16 | -1,57052 |
| 3 | 0,39 | -2,55416 |
| 4 | 0,01 | 0,32468 |
| 5 | 0,13 | -1,46176 |
| 6 | 0,24 | -2,20249 |
| 7 | 0,01 | -0,39095 |
| 8 | 0,09 | -1,12170 |
| 9 | 0,02 | -0,58295 |
| 10 | 0,01 | 0,45060 |
| 11 | 0,04 | 0,79579 |
| 12 | 0,00 | -0,19095 |
| 13 | 0,00 | -0,16314 |
| 14 | 0,04 | 0,73372 |
| 15 | 0,00 | -0,23863 |
| 16 | 0,12 | -1,39561 |
| 17 | 0,00 | 0,23038 |
| 18 | 0,23 | 1,98603 |
| 19 | 0,04 | 0,75849 |
| 20 | 0,25 | 2,05961 |
| 21 | 0,00 | -0,15363 |
| 22 | 0,74 | 3,58267 |
| 23 | 0,68 | 3,37249 |
| 24 | 0,04 | -0,75172 |
| 25 | 0,01 | -0,42670 |
| 26 | 0,00 | 0,10471 |
| 27 | 0,06 | 1,01522 |

## Coded Coefficients

| Term | Coef | SE Coef | 95% CI | T-Value | P-Value | VIF |
| --- | --- | --- | --- | --- | --- | --- |
| Constant | 78,74 | 1,50 | (75,46; 82,01) | 52,42 | 0,000 |  |
| Lac | 9,66 | 1,24 | (6,96; 12,37) | 7,78 | 0,000 | 1,18 |
| HPMC\_Visc | -1,03 | 1,30 | (-3,85; 1,80) | -0,79 | 0,444 | 1,70 |
| HPMC\_HP | 2,65 | 1,27 | (-0,12; 5,42) | 2,09 | 0,059 | 1,26 |
| HPMC\_PS | 3,12 | 1,95 | (-1,12; 7,37) | 1,60 | 0,135 | 2,09 |
| Lac\*Lac | 1,49 | 2,47 | (-3,89; 6,87) | 0,60 | 0,557 | 1,30 |
| HPMC\_Visc\*HPMC\_Visc | 2,17 | 2,62 | (-3,55; 7,89) | 0,83 | 0,425 | 1,96 |
| HPMC\_HP\*HPMC\_HP | 1,69 | 2,49 | (-3,73; 7,12) | 0,68 | 0,510 | 1,81 |
| HPMC\_PS\*HPMC\_PS | 1,44 | 2,47 | (-3,95; 6,83) | 0,58 | 0,571 | 1,42 |
| Lac\*HPMC\_Visc | -5,26 | 2,72 | (-11,19; 0,68) | -1,93 | 0,077 | 1,49 |
| Lac\*HPMC\_HP | 0,64 | 2,86 | (-5,58; 6,87) | 0,23 | 0,825 | 1,17 |
| Lac\*HPMC\_PS | -1,80 | 4,41 | (-11,41; 7,81) | -0,41 | 0,691 | 1,42 |
| HPMC\_Visc\*HPMC\_HP | -0,42 | 3,25 | (-7,50; 6,66) | -0,13 | 0,899 | 2,73 |
| HPMC\_Visc\*HPMC\_PS | 8,04 | 4,59 | (-1,96; 18,04) | 1,75 | 0,105 | 2,74 |
| HPMC\_HP\*HPMC\_PS | -0,76 | 5,07 | (-11,80; 10,28) | -0,15 | 0,883 | 2,70 |

## Model Summary

| S | R-sq | R-sq(adj) | PRESS | R-sq(pred) | AICc | BIC |
| --- | --- | --- | --- | --- | --- | --- |
| 2,79661 | 89,44% | 77,11% | 613,762 | 30,92% | 196,66 | 162,99 |

## Analysis of Variance

| Source | DF | Seq SS | Contribution | Adj SS | Adj MS | F-Value | P-Value |
| --- | --- | --- | --- | --- | --- | --- | --- |
| Model | 14 | 794,593 | 89,44% | 794,593 | 56,757 | 7,26 | 0,001 |
| Linear | 4 | 732,966 | 82,50% | 559,378 | 139,845 | 17,88 | 0,000 |
| Lac | 1 | 637,882 | 71,80% | 473,382 | 473,382 | 60,53 | 0,000 |
| HPMC\_Visc | 1 | 46,874 | 5,28% | 4,890 | 4,890 | 0,63 | 0,444 |
| HPMC\_HP | 1 | 41,747 | 4,70% | 34,077 | 34,077 | 4,36 | 0,059 |
| HPMC\_PS | 1 | 6,463 | 0,73% | 20,113 | 20,113 | 2,57 | 0,135 |
| Square | 4 | 2,221 | 0,25% | 7,131 | 1,783 | 0,23 | 0,917 |
| Lac\*Lac | 1 | 0,310 | 0,03% | 2,855 | 2,855 | 0,37 | 0,557 |
| HPMC\_Visc\*HPMC\_Visc | 1 | 1,218 | 0,14% | 5,341 | 5,341 | 0,68 | 0,425 |
| HPMC\_HP\*HPMC\_HP | 1 | 0,005 | 0,00% | 3,615 | 3,615 | 0,46 | 0,510 |
| HPMC\_PS\*HPMC\_PS | 1 | 0,688 | 0,08% | 2,657 | 2,657 | 0,34 | 0,571 |
| 2-Way Interaction | 6 | 59,407 | 6,69% | 59,407 | 9,901 | 1,27 | 0,342 |
| Lac\*HPMC\_Visc | 1 | 31,593 | 3,56% | 29,155 | 29,155 | 3,73 | 0,077 |
| Lac\*HPMC\_HP | 1 | 0,136 | 0,02% | 0,398 | 0,398 | 0,05 | 0,825 |
| Lac\*HPMC\_PS | 1 | 1,300 | 0,15% | 1,300 | 1,300 | 0,17 | 0,691 |
| HPMC\_Visc\*HPMC\_HP | 1 | 2,314 | 0,26% | 0,131 | 0,131 | 0,02 | 0,899 |
| HPMC\_Visc\*HPMC\_PS | 1 | 23,887 | 2,69% | 23,979 | 23,979 | 3,07 | 0,105 |
| HPMC\_HP\*HPMC\_PS | 1 | 0,177 | 0,02% | 0,177 | 0,177 | 0,02 | 0,883 |
| Error | 12 | 93,852 | 10,56% | 93,852 | 7,821 |  |  |
| Lack-of-Fit | 10 | 80,496 | 9,06% | 80,496 | 8,050 | 1,21 | 0,536 |
| Pure Error | 2 | 13,357 | 1,50% | 13,357 | 6,678 |  |  |
| Total | 26 | 888,446 | 100,00% |  |  |  |  |

## Regression Equation in Uncoded Units

|  |  |  |
| --- | --- | --- |
| F\_mean\_13h(780min) | = | 442 + 132 Lac - 0,0200 HPMC\_Visc - 21,2 HPMC\_HP - 5,63 HPMC\_PS + 23,9 Lac\*Lac + 0,000000 HPMC\_Visc\*HPMC\_Visc + 1,64 HPMC\_HP\*HPMC\_HP + 0,0267 HPMC\_PS\*HPMC\_PS - 0,00541 Lac\*HPMC\_Visc + 2,5 Lac\*HPMC\_HP - 0,98 Lac\*HPMC\_PS - 0,000107 HPMC\_Visc\*HPMC\_HP + 0,000281 HPMC\_Visc\*HPMC\_PS - 0,102 HPMC\_HP\*HPMC\_PS |

## Fits and Diagnostics for All Observations

| Obs | F\_mean\_13h(780min) | Fit | SE Fit | 95% CI | Resid | Std Resid | Del Resid | HI |
| --- | --- | --- | --- | --- | --- | --- | --- | --- |
| 1 | 75,21 | 73,59 | 2,27 | (68,66; 78,53) | 1,61 | 0,98 | 0,98 | 0,655994 |
| 2 | 85,11 | 86,98 | 2,27 | (82,05; 91,92) | -1,87 | -1,14 | -1,15 | 0,655994 |
| 3 | 68,33 | 70,46 | 2,39 | (65,25; 75,66) | -2,12 | -1,46 | -1,54 | 0,729945 |
| 4 | 77,45 | 77,05 | 2,39 | (71,85; 82,26) | 0,40 | 0,28 | 0,27 | 0,729945 |
| 5 | 72,00 | 75,03 | 1,93 | (70,82; 79,24) | -3,03 | -1,50 | -1,59 | 0,476813 |
| 6 | 84,62 | 88,55 | 1,93 | (84,34; 92,76) | -3,93 | -1,95 | -2,25 | 0,476813 |
| 7 | 73,78 | 74,42 | 2,32 | (69,37; 79,48) | -0,64 | -0,41 | -0,39 | 0,688750 |
| 8 | 80,48 | 81,36 | 2,32 | (76,30; 86,42) | -0,88 | -0,56 | -0,55 | 0,688750 |
| 9 | 71,67 | 73,03 | 2,13 | (68,39; 77,68) | -1,37 | -0,75 | -0,74 | 0,580949 |
| 10 | 86,27 | 85,15 | 2,13 | (80,51; 89,80) | 1,12 | 0,62 | 0,60 | 0,580949 |
| 11 | 77,67 | 75,29 | 1,82 | (71,31; 79,26) | 2,39 | 1,13 | 1,14 | 0,425781 |
| 12 | 82,65 | 83,26 | 1,82 | (79,29; 87,24) | -0,61 | -0,29 | -0,28 | 0,425781 |
| 13 | 74,71 | 74,96 | 2,11 | (70,37; 79,56) | -0,26 | -0,14 | -0,13 | 0,569258 |
| 14 | 88,78 | 87,64 | 2,11 | (83,04; 92,24) | 1,14 | 0,62 | 0,60 | 0,569258 |
| 15 | 76,50 | 77,08 | 1,84 | (73,07; 81,09) | -0,58 | -0,27 | -0,26 | 0,433227 |
| 16 | 81,89 | 84,89 | 1,84 | (80,88; 88,90) | -3,00 | -1,43 | -1,50 | 0,433227 |
| 17 | 70,16 | 69,62 | 2,16 | (64,91; 74,33) | 0,54 | 0,30 | 0,29 | 0,597775 |
| 18 | 93,34 | 90,98 | 2,16 | (86,27; 95,69) | 2,36 | 1,33 | 1,38 | 0,597775 |
| 19 | 83,43 | 81,27 | 1,78 | (77,39; 85,16) | 2,16 | 1,00 | 1,00 | 0,407038 |
| 20 | 80,14 | 77,99 | 2,25 | (73,09; 82,89) | 2,14 | 1,29 | 1,33 | 0,647412 |
| 21 | 77,18 | 77,72 | 1,79 | (73,83; 81,61) | -0,54 | -0,25 | -0,24 | 0,407933 |
| 22 | 85,23 | 83,36 | 2,53 | (77,85; 88,87) | 1,87 | 1,56 | 1,67 | 0,817044 |
| 23 | 81,03 | 79,30 | 2,54 | (73,78; 84,83) | 1,72 | 1,46 | 1,55 | 0,823434 |
| 24 | 80,49 | 81,27 | 2,36 | (76,12; 86,42) | -0,78 | -0,52 | -0,51 | 0,713775 |
| 25 | 77,11 | 78,81 | 1,50 | (75,53; 82,08) | -1,69 | -0,72 | -0,70 | 0,288793 |
| 26 | 79,19 | 78,81 | 1,50 | (75,53; 82,08) | 0,39 | 0,16 | 0,16 | 0,288793 |
| 27 | 82,25 | 78,81 | 1,50 | (75,53; 82,08) | 3,44 | 1,46 | 1,54 | 0,288793 |

| Obs | Cook’s D | DFITS |
| --- | --- | --- |
| 1 | 0,12 | 1,35640 |
| 2 | 0,16 | -1,59463 |
| 3 | 0,38 | -2,53307 |
| 4 | 0,01 | 0,43850 |
| 5 | 0,14 | -1,51675 |
| 6 | 0,23 | -2,14861 |
| 7 | 0,02 | -0,58723 |
| 8 | 0,05 | -0,81129 |
| 9 | 0,05 | -0,87137 |
| 10 | 0,04 | 0,71145 |
| 11 | 0,06 | 0,98141 |
| 12 | 0,00 | -0,23982 |
| 13 | 0,00 | -0,15306 |
| 14 | 0,03 | 0,69262 |
| 15 | 0,00 | -0,23039 |
| 16 | 0,10 | -1,30928 |
| 17 | 0,01 | 0,35736 |
| 18 | 0,18 | 1,68510 |
| 19 | 0,05 | 0,83161 |
| 20 | 0,20 | 1,80513 |
| 21 | 0,00 | -0,19857 |
| 22 | 0,73 | 3,53810 |
| 23 | 0,67 | 3,34241 |
| 24 | 0,05 | -0,79961 |
| 25 | 0,01 | -0,44770 |
| 26 | 0,00 | 0,10022 |
| 27 | 0,06 | 0,98264 |

## Coded Coefficients

| Term | Coef | SE Coef | 95% CI | T-Value | P-Value | VIF |
| --- | --- | --- | --- | --- | --- | --- |
| Constant | 81,69 | 1,41 | (78,62; 84,75) | 58,06 | 0,000 |  |
| Lac | 9,39 | 1,16 | (6,86; 11,93) | 8,07 | 0,000 | 1,18 |
| HPMC\_Visc | -0,87 | 1,22 | (-3,52; 1,78) | -0,71 | 0,490 | 1,70 |
| HPMC\_HP | 2,49 | 1,19 | (-0,10; 5,08) | 2,09 | 0,058 | 1,26 |
| HPMC\_PS | 2,79 | 1,82 | (-1,19; 6,76) | 1,53 | 0,152 | 2,09 |
| Lac\*Lac | 1,03 | 2,31 | (-4,01; 6,07) | 0,45 | 0,663 | 1,30 |
| HPMC\_Visc\*HPMC\_Visc | 2,06 | 2,46 | (-3,29; 7,42) | 0,84 | 0,418 | 1,96 |
| HPMC\_HP\*HPMC\_HP | 1,58 | 2,33 | (-3,50; 6,66) | 0,68 | 0,510 | 1,81 |
| HPMC\_PS\*HPMC\_PS | 1,24 | 2,32 | (-3,81; 6,29) | 0,54 | 0,602 | 1,42 |
| Lac\*HPMC\_Visc | -4,88 | 2,55 | (-10,44; 0,68) | -1,91 | 0,080 | 1,49 |
| Lac\*HPMC\_HP | -0,13 | 2,68 | (-5,96; 5,71) | -0,05 | 0,963 | 1,17 |
| Lac\*HPMC\_PS | -2,03 | 4,13 | (-11,03; 6,97) | -0,49 | 0,632 | 1,42 |
| HPMC\_Visc\*HPMC\_HP | -0,28 | 3,04 | (-6,91; 6,35) | -0,09 | 0,928 | 2,73 |
| HPMC\_Visc\*HPMC\_PS | 7,32 | 4,30 | (-2,04; 16,69) | 1,70 | 0,114 | 2,74 |
| HPMC\_HP\*HPMC\_PS | -1,55 | 4,75 | (-11,89; 8,79) | -0,33 | 0,749 | 2,70 |

## Model Summary

| S | R-sq | R-sq(adj) | PRESS | R-sq(pred) | AICc | BIC |
| --- | --- | --- | --- | --- | --- | --- |
| 2,61946 | 90,07% | 78,48% | 510,966 | 38,36% | 193,13 | 159,46 |

## Analysis of Variance

| Source | DF | Seq SS | Contribution | Adj SS | Adj MS | F-Value | P-Value |
| --- | --- | --- | --- | --- | --- | --- | --- |
| Model | 14 | 746,590 | 90,07% | 746,590 | 53,328 | 7,77 | 0,001 |
| Linear | 4 | 692,649 | 83,56% | 517,325 | 129,331 | 18,85 | 0,000 |
| Lac | 1 | 611,236 | 73,74% | 447,281 | 447,281 | 65,19 | 0,000 |
| HPMC\_Visc | 1 | 37,705 | 4,55% | 3,486 | 3,486 | 0,51 | 0,490 |
| HPMC\_HP | 1 | 37,718 | 4,55% | 30,033 | 30,033 | 4,38 | 0,058 |
| HPMC\_PS | 1 | 5,990 | 0,72% | 16,016 | 16,016 | 2,33 | 0,152 |
| Square | 4 | 1,724 | 0,21% | 5,973 | 1,493 | 0,22 | 0,924 |
| Lac\*Lac | 1 | 0,006 | 0,00% | 1,367 | 1,367 | 0,20 | 0,663 |
| HPMC\_Visc\*HPMC\_Visc | 1 | 0,822 | 0,10% | 4,836 | 4,836 | 0,70 | 0,418 |
| HPMC\_HP\*HPMC\_HP | 1 | 0,016 | 0,00% | 3,156 | 3,156 | 0,46 | 0,510 |
| HPMC\_PS\*HPMC\_PS | 1 | 0,881 | 0,11% | 1,972 | 1,972 | 0,29 | 0,602 |
| 2-Way Interaction | 6 | 52,217 | 6,30% | 52,217 | 8,703 | 1,27 | 0,341 |
| Lac\*HPMC\_Visc | 1 | 26,311 | 3,17% | 25,117 | 25,117 | 3,66 | 0,080 |
| Lac\*HPMC\_HP | 1 | 0,196 | 0,02% | 0,015 | 0,015 | 0,00 | 0,963 |
| Lac\*HPMC\_PS | 1 | 1,660 | 0,20% | 1,660 | 1,660 | 0,24 | 0,632 |
| HPMC\_Visc\*HPMC\_HP | 1 | 4,092 | 0,49% | 0,058 | 0,058 | 0,01 | 0,928 |
| HPMC\_Visc\*HPMC\_PS | 1 | 19,222 | 2,32% | 19,912 | 19,912 | 2,90 | 0,114 |
| HPMC\_HP\*HPMC\_PS | 1 | 0,736 | 0,09% | 0,736 | 0,736 | 0,11 | 0,749 |
| Error | 12 | 82,339 | 9,93% | 82,339 | 6,862 |  |  |
| Lack-of-Fit | 10 | 70,139 | 8,46% | 70,139 | 7,014 | 1,15 | 0,552 |
| Pure Error | 2 | 12,200 | 1,47% | 12,200 | 6,100 |  |  |
| Total | 26 | 828,929 | 100,00% |  |  |  |  |

## Regression Equation in Uncoded Units

|  |  |  |
| --- | --- | --- |
| F\_mean\_14h(840min) | = | 314 + 171 Lac - 0,0186 HPMC\_Visc - 10,9 HPMC\_HP - 3,76 HPMC\_PS + 16,5 Lac\*Lac + 0,000000 HPMC\_Visc\*HPMC\_Visc + 1,54 HPMC\_HP\*HPMC\_HP + 0,0230 HPMC\_PS\*HPMC\_PS - 0,00502 Lac\*HPMC\_Visc - 0,5 Lac\*HPMC\_HP - 1,11 Lac\*HPMC\_PS - 0,000071 HPMC\_Visc\*HPMC\_HP + 0,000256 HPMC\_Visc\*HPMC\_PS - 0,208 HPMC\_HP\*HPMC\_PS |

## Fits and Diagnostics for All Observations

| Obs | F\_mean\_14h(840min) | Fit | SE Fit | 95% CI | Resid | Std Resid | Del Resid | HI |
| --- | --- | --- | --- | --- | --- | --- | --- | --- |
| 1 | 77,72 | 76,16 | 2,12 | (71,54; 80,79) | 1,56 | 1,01 | 1,01 | 0,655994 |
| 2 | 87,87 | 89,62 | 2,12 | (85,00; 94,25) | -1,75 | -1,14 | -1,16 | 0,655994 |
| 3 | 71,14 | 73,06 | 2,24 | (68,18; 77,93) | -1,91 | -1,41 | -1,47 | 0,729945 |
| 4 | 80,87 | 80,35 | 2,24 | (75,48; 85,23) | 0,51 | 0,38 | 0,36 | 0,729945 |
| 5 | 74,97 | 77,98 | 1,81 | (74,04; 81,92) | -3,01 | -1,59 | -1,71 | 0,476813 |
| 6 | 87,43 | 90,87 | 1,81 | (86,93; 94,81) | -3,43 | -1,81 | -2,04 | 0,476813 |
| 7 | 77,14 | 77,85 | 2,17 | (73,11; 82,59) | -0,71 | -0,48 | -0,47 | 0,688750 |
| 8 | 84,05 | 84,51 | 2,17 | (79,77; 89,25) | -0,46 | -0,32 | -0,30 | 0,688750 |
| 9 | 74,30 | 76,14 | 2,00 | (71,79; 80,49) | -1,84 | -1,09 | -1,10 | 0,580949 |
| 10 | 89,52 | 88,16 | 2,00 | (83,81; 92,51) | 1,37 | 0,81 | 0,79 | 0,580949 |
| 11 | 80,76 | 78,10 | 1,71 | (74,38; 81,83) | 2,66 | 1,34 | 1,39 | 0,425781 |
| 12 | 85,51 | 86,20 | 1,71 | (82,47; 89,92) | -0,69 | -0,35 | -0,33 | 0,425781 |
| 13 | 77,65 | 77,86 | 1,98 | (73,55; 82,17) | -0,21 | -0,12 | -0,12 | 0,569258 |
| 14 | 90,39 | 89,57 | 1,98 | (85,26; 93,87) | 0,82 | 0,48 | 0,46 | 0,569258 |
| 15 | 79,24 | 79,92 | 1,72 | (76,16; 83,67) | -0,68 | -0,34 | -0,33 | 0,433227 |
| 16 | 84,86 | 87,37 | 1,72 | (83,61; 91,12) | -2,51 | -1,27 | -1,31 | 0,433227 |
| 17 | 73,18 | 72,39 | 2,03 | (67,97; 76,80) | 0,79 | 0,48 | 0,46 | 0,597775 |
| 18 | 94,95 | 93,16 | 2,03 | (88,75; 97,57) | 1,79 | 1,07 | 1,08 | 0,597775 |
| 19 | 86,40 | 84,02 | 1,67 | (80,38; 87,66) | 2,38 | 1,18 | 1,20 | 0,407038 |
| 20 | 82,72 | 81,12 | 2,11 | (76,53; 85,72) | 1,60 | 1,03 | 1,03 | 0,647412 |
| 21 | 80,08 | 80,65 | 1,67 | (77,01; 84,30) | -0,57 | -0,28 | -0,27 | 0,407933 |
| 22 | 87,65 | 85,94 | 2,37 | (80,79; 91,10) | 1,71 | 1,53 | 1,63 | 0,817044 |
| 23 | 83,59 | 82,12 | 2,38 | (76,94; 87,29) | 1,48 | 1,34 | 1,40 | 0,823434 |
| 24 | 83,37 | 84,00 | 2,21 | (79,18; 88,82) | -0,63 | -0,45 | -0,43 | 0,713775 |
| 25 | 80,01 | 81,74 | 1,41 | (78,67; 84,81) | -1,73 | -0,79 | -0,77 | 0,288793 |
| 26 | 82,06 | 81,74 | 1,41 | (78,67; 84,81) | 0,31 | 0,14 | 0,14 | 0,288793 |
| 27 | 84,92 | 81,74 | 1,41 | (78,67; 84,81) | 3,18 | 1,44 | 1,52 | 0,288793 |

| Obs | Cook’s D | DFITS |
| --- | --- | --- |
| 1 | 0,13 | 1,40051 |
| 2 | 0,17 | -1,59747 |
| 3 | 0,36 | -2,42043 |
| 4 | 0,03 | 0,59433 |
| 5 | 0,15 | -1,63504 |
| 6 | 0,20 | -1,94343 |
| 7 | 0,03 | -0,69717 |
| 8 | 0,01 | -0,45345 |
| 9 | 0,11 | -1,29177 |
| 10 | 0,06 | 0,93369 |
| 11 | 0,09 | 1,19607 |
| 12 | 0,01 | -0,28782 |
| 13 | 0,00 | -0,13652 |
| 14 | 0,02 | 0,53027 |
| 15 | 0,01 | -0,28894 |
| 16 | 0,08 | -1,14332 |
| 17 | 0,02 | 0,56031 |
| 18 | 0,11 | 1,31987 |
| 19 | 0,06 | 0,99583 |
| 20 | 0,13 | 1,39371 |
| 21 | 0,00 | -0,22636 |
| 22 | 0,69 | 3,43674 |
| 23 | 0,56 | 3,01409 |
| 24 | 0,03 | -0,68298 |
| 25 | 0,02 | -0,49180 |
| 26 | 0,00 | 0,08699 |
| 27 | 0,06 | 0,96655 |

## Coded Coefficients

| Term | Coef | SE Coef | 95% CI | T-Value | P-Value | VIF |
| --- | --- | --- | --- | --- | --- | --- |
| Constant | 84,38 | 1,34 | (81,46; 87,31) | 62,88 | 0,000 |  |
| Lac | 8,99 | 1,11 | (6,57; 11,40) | 8,10 | 0,000 | 1,18 |
| HPMC\_Visc | -0,72 | 1,16 | (-3,24; 1,81) | -0,62 | 0,549 | 1,70 |
| HPMC\_HP | 2,31 | 1,13 | (-0,16; 4,78) | 2,04 | 0,064 | 1,26 |
| HPMC\_PS | 2,52 | 1,74 | (-1,27; 6,31) | 1,45 | 0,173 | 2,09 |
| Lac\*Lac | 0,30 | 2,21 | (-4,50; 5,11) | 0,14 | 0,893 | 1,30 |
| HPMC\_Visc\*HPMC\_Visc | 2,02 | 2,34 | (-3,08; 7,13) | 0,86 | 0,405 | 1,96 |
| HPMC\_HP\*HPMC\_HP | 1,56 | 2,22 | (-3,29; 6,40) | 0,70 | 0,497 | 1,81 |
| HPMC\_PS\*HPMC\_PS | 1,04 | 2,21 | (-3,78; 5,86) | 0,47 | 0,646 | 1,42 |
| Lac\*HPMC\_Visc | -4,26 | 2,43 | (-9,56; 1,03) | -1,75 | 0,105 | 1,49 |
| Lac\*HPMC\_HP | -0,70 | 2,55 | (-6,27; 4,86) | -0,28 | 0,787 | 1,17 |
| Lac\*HPMC\_PS | -2,11 | 3,94 | (-10,69; 6,48) | -0,54 | 0,602 | 1,42 |
| HPMC\_Visc\*HPMC\_HP | 0,14 | 2,90 | (-6,19; 6,47) | 0,05 | 0,962 | 2,73 |
| HPMC\_Visc\*HPMC\_PS | 6,75 | 4,10 | (-2,19; 15,68) | 1,64 | 0,126 | 2,74 |
| HPMC\_HP\*HPMC\_PS | -1,94 | 4,53 | (-11,80; 7,93) | -0,43 | 0,676 | 2,70 |

## Model Summary

| S | R-sq | R-sq(adj) | PRESS | R-sq(pred) | AICc | BIC |
| --- | --- | --- | --- | --- | --- | --- |
| 2,49832 | 90,07% | 78,49% | 444,525 | 41,07% | 190,57 | 156,90 |

## Analysis of Variance

| Source | DF | Seq SS | Contribution | Adj SS | Adj MS | F-Value | P-Value |
| --- | --- | --- | --- | --- | --- | --- | --- |
| Model | 14 | 679,409 | 90,07% | 679,409 | 48,529 | 7,78 | 0,001 |
| Linear | 4 | 630,641 | 83,61% | 466,415 | 116,604 | 18,68 | 0,000 |
| Lac | 1 | 562,249 | 74,54% | 409,402 | 409,402 | 65,59 | 0,000 |
| HPMC\_Visc | 1 | 31,578 | 4,19% | 2,374 | 2,374 | 0,38 | 0,549 |
| HPMC\_HP | 1 | 31,576 | 4,19% | 25,876 | 25,876 | 4,15 | 0,064 |
| HPMC\_PS | 1 | 5,239 | 0,69% | 13,115 | 13,115 | 2,10 | 0,173 |
| Square | 4 | 2,264 | 0,30% | 6,275 | 1,569 | 0,25 | 0,903 |
| Lac\*Lac | 1 | 0,609 | 0,08% | 0,117 | 0,117 | 0,02 | 0,893 |
| HPMC\_Visc\*HPMC\_Visc | 1 | 0,428 | 0,06% | 4,658 | 4,658 | 0,75 | 0,405 |
| HPMC\_HP\*HPMC\_HP | 1 | 0,000 | 0,00% | 3,060 | 3,060 | 0,49 | 0,497 |
| HPMC\_PS\*HPMC\_PS | 1 | 1,226 | 0,16% | 1,382 | 1,382 | 0,22 | 0,646 |
| 2-Way Interaction | 6 | 46,504 | 6,17% | 46,504 | 7,751 | 1,24 | 0,352 |
| Lac\*HPMC\_Visc | 1 | 19,347 | 2,56% | 19,190 | 19,190 | 3,07 | 0,105 |
| Lac\*HPMC\_HP | 1 | 1,078 | 0,14% | 0,475 | 0,475 | 0,08 | 0,787 |
| Lac\*HPMC\_PS | 1 | 1,789 | 0,24% | 1,789 | 1,789 | 0,29 | 0,602 |
| HPMC\_Visc\*HPMC\_HP | 1 | 7,170 | 0,95% | 0,015 | 0,015 | 0,00 | 0,962 |
| HPMC\_Visc\*HPMC\_PS | 1 | 15,978 | 2,12% | 16,890 | 16,890 | 2,71 | 0,126 |
| HPMC\_HP\*HPMC\_PS | 1 | 1,142 | 0,15% | 1,142 | 1,142 | 0,18 | 0,676 |
| Error | 12 | 74,900 | 9,93% | 74,900 | 6,242 |  |  |
| Lack-of-Fit | 10 | 64,250 | 8,52% | 64,250 | 6,425 | 1,21 | 0,536 |
| Pure Error | 2 | 10,650 | 1,41% | 10,650 | 5,325 |  |  |
| Total | 26 | 754,308 | 100,00% |  |  |  |  |

## Regression Equation in Uncoded Units

|  |  |  |
| --- | --- | --- |
| F\_mean\_15h(900min) | = | 251 + 197 Lac - 0,0184 HPMC\_Visc - 7,4 HPMC\_HP - 2,49 HPMC\_PS + 4,8 Lac\*Lac + 0,000000 HPMC\_Visc\*HPMC\_Visc + 1,51 HPMC\_HP\*HPMC\_HP + 0,0192 HPMC\_PS\*HPMC\_PS - 0,00439 Lac\*HPMC\_Visc - 2,8 Lac\*HPMC\_HP - 1,15 Lac\*HPMC\_PS + 0,000036 HPMC\_Visc\*HPMC\_HP + 0,000236 HPMC\_Visc\*HPMC\_PS - 0,259 HPMC\_HP\*HPMC\_PS |

## Fits and Diagnostics for All Observations

| Obs | F\_mean\_15h(900min) | Fit | SE Fit | 95% CI | Resid | Std Resid | Del Resid | HI |
| --- | --- | --- | --- | --- | --- | --- | --- | --- |
| 1 | 80,61 | 78,97 | 2,02 | (74,56; 83,37) | 1,64 | 1,12 | 1,13 | 0,655994 |
| 2 | 90,40 | 92,02 | 2,02 | (87,61; 96,43) | -1,62 | -1,10 | -1,12 | 0,655994 |
| 3 | 73,72 | 75,46 | 2,13 | (70,81; 80,11) | -1,74 | -1,34 | -1,39 | 0,729945 |
| 4 | 83,97 | 83,25 | 2,13 | (78,60; 87,90) | 0,72 | 0,55 | 0,53 | 0,729945 |
| 5 | 77,63 | 80,73 | 1,73 | (76,97; 84,49) | -3,09 | -1,71 | -1,89 | 0,476813 |
| 6 | 89,90 | 92,70 | 1,73 | (88,94; 96,46) | -2,80 | -1,55 | -1,66 | 0,476813 |
| 7 | 80,20 | 80,91 | 2,07 | (76,39; 85,43) | -0,71 | -0,51 | -0,50 | 0,688750 |
| 8 | 87,18 | 87,35 | 2,07 | (82,84; 91,87) | -0,17 | -0,12 | -0,12 | 0,688750 |
| 9 | 76,94 | 79,20 | 1,90 | (75,05; 83,35) | -2,26 | -1,40 | -1,46 | 0,580949 |
| 10 | 92,34 | 90,75 | 1,90 | (86,60; 94,89) | 1,60 | 0,99 | 0,99 | 0,580949 |
| 11 | 83,57 | 80,65 | 1,63 | (77,10; 84,20) | 2,92 | 1,54 | 1,65 | 0,425781 |
| 12 | 87,99 | 88,71 | 1,63 | (85,16; 92,26) | -0,72 | -0,38 | -0,37 | 0,425781 |
| 13 | 80,33 | 80,54 | 1,88 | (76,44; 84,65) | -0,21 | -0,13 | -0,12 | 0,569258 |
| 14 | 91,61 | 91,14 | 1,88 | (87,03; 95,24) | 0,47 | 0,29 | 0,28 | 0,569258 |
| 15 | 81,60 | 82,49 | 1,64 | (78,90; 86,07) | -0,89 | -0,47 | -0,46 | 0,433227 |
| 16 | 87,55 | 89,58 | 1,64 | (86,00; 93,17) | -2,03 | -1,08 | -1,09 | 0,433227 |
| 17 | 75,88 | 74,83 | 1,93 | (70,62; 79,04) | 1,06 | 0,67 | 0,65 | 0,597775 |
| 18 | 95,79 | 94,62 | 1,93 | (90,42; 98,83) | 1,17 | 0,74 | 0,72 | 0,597775 |
| 19 | 89,09 | 86,60 | 1,59 | (83,13; 90,07) | 2,49 | 1,29 | 1,34 | 0,407038 |
| 20 | 85,07 | 83,99 | 2,01 | (79,61; 88,37) | 1,08 | 0,73 | 0,71 | 0,647412 |
| 21 | 82,70 | 83,52 | 1,60 | (80,05; 87,00) | -0,82 | -0,43 | -0,41 | 0,407933 |
| 22 | 89,97 | 88,34 | 2,26 | (83,42; 93,26) | 1,64 | 1,53 | 1,63 | 0,817044 |
| 23 | 85,86 | 84,67 | 2,27 | (79,73; 89,61) | 1,19 | 1,13 | 1,15 | 0,823434 |
| 24 | 86,02 | 86,45 | 2,11 | (81,85; 91,05) | -0,43 | -0,32 | -0,31 | 0,713775 |
| 25 | 82,74 | 84,42 | 1,34 | (81,50; 87,35) | -1,68 | -0,80 | -0,78 | 0,288793 |
| 26 | 84,72 | 84,42 | 1,34 | (81,50; 87,35) | 0,29 | 0,14 | 0,13 | 0,288793 |
| 27 | 87,34 | 84,42 | 1,34 | (81,50; 87,35) | 2,92 | 1,39 | 1,45 | 0,288793 |

| Obs | Cook’s D | DFITS |
| --- | --- | --- |
| 1 | 0,16 | 1,56311 |
| 2 | 0,15 | -1,53992 |
| 3 | 0,32 | -2,28235 |
| 4 | 0,05 | 0,87903 |
| 5 | 0,18 | -1,80085 |
| 6 | 0,15 | -1,58538 |
| 7 | 0,04 | -0,73642 |
| 8 | 0,00 | -0,17775 |
| 9 | 0,18 | -1,71950 |
| 10 | 0,09 | 1,16086 |
| 11 | 0,12 | 1,41912 |
| 12 | 0,01 | -0,31589 |
| 13 | 0,00 | -0,14031 |
| 14 | 0,01 | 0,31786 |
| 15 | 0,01 | -0,39913 |
| 16 | 0,06 | -0,95075 |
| 17 | 0,04 | 0,79415 |
| 18 | 0,05 | 0,88038 |
| 19 | 0,08 | 1,10745 |
| 20 | 0,06 | 0,96682 |
| 21 | 0,01 | -0,34254 |
| 22 | 0,70 | 3,45424 |
| 23 | 0,40 | 2,47478 |
| 24 | 0,02 | -0,48707 |
| 25 | 0,02 | -0,49993 |
| 26 | 0,00 | 0,08474 |
| 27 | 0,05 | 0,92242 |

## Coded Coefficients

| Term | Coef | SE Coef | 95% CI | T-Value | P-Value | VIF |
| --- | --- | --- | --- | --- | --- | --- |
| Constant | 86,83 | 1,25 | (84,10; 89,56) | 69,24 | 0,000 |  |
| Lac | 8,39 | 1,04 | (6,13; 10,65) | 8,09 | 0,000 | 1,18 |
| HPMC\_Visc | -0,56 | 1,08 | (-2,92; 1,80) | -0,51 | 0,616 | 1,70 |
| HPMC\_HP | 2,14 | 1,06 | (-0,17; 4,45) | 2,02 | 0,067 | 1,26 |
| HPMC\_PS | 2,34 | 1,63 | (-1,20; 5,89) | 1,44 | 0,175 | 2,09 |
| Lac\*Lac | -0,35 | 2,06 | (-4,84; 4,14) | -0,17 | 0,868 | 1,30 |
| HPMC\_Visc\*HPMC\_Visc | 2,03 | 2,19 | (-2,75; 6,80) | 0,93 | 0,373 | 1,96 |
| HPMC\_HP\*HPMC\_HP | 1,63 | 2,08 | (-2,90; 6,16) | 0,79 | 0,447 | 1,81 |
| HPMC\_PS\*HPMC\_PS | 0,81 | 2,07 | (-3,69; 5,31) | 0,39 | 0,702 | 1,42 |
| Lac\*HPMC\_Visc | -4,24 | 2,27 | (-9,19; 0,72) | -1,86 | 0,087 | 1,49 |
| Lac\*HPMC\_HP | -1,36 | 2,39 | (-6,56; 3,84) | -0,57 | 0,580 | 1,17 |
| Lac\*HPMC\_PS | -2,60 | 3,68 | (-10,62; 5,43) | -0,71 | 0,494 | 1,42 |
| HPMC\_Visc\*HPMC\_HP | 0,26 | 2,71 | (-5,65; 6,17) | 0,10 | 0,925 | 2,73 |
| HPMC\_Visc\*HPMC\_PS | 6,63 | 3,83 | (-1,72; 14,98) | 1,73 | 0,109 | 2,74 |
| HPMC\_HP\*HPMC\_PS | -2,39 | 4,23 | (-11,61; 6,82) | -0,57 | 0,582 | 2,70 |

## Model Summary

| S | R-sq | R-sq(adj) | PRESS | R-sq(pred) | AICc | BIC |
| --- | --- | --- | --- | --- | --- | --- |
| 2,33472 | 90,41% | 79,23% | 363,391 | 46,74% | 186,91 | 153,25 |

## Analysis of Variance

| Source | DF | Seq SS | Contribution | Adj SS | Adj MS | F-Value | P-Value |
| --- | --- | --- | --- | --- | --- | --- | --- |
| Model | 14 | 616,854 | 90,41% | 616,854 | 44,061 | 8,08 | 0,000 |
| Linear | 4 | 561,941 | 82,36% | 403,461 | 100,865 | 18,50 | 0,000 |
| Lac | 1 | 504,288 | 73,91% | 356,588 | 356,588 | 65,42 | 0,000 |
| HPMC\_Visc | 1 | 26,365 | 3,86% | 1,444 | 1,444 | 0,26 | 0,616 |
| HPMC\_HP | 1 | 26,767 | 3,92% | 22,188 | 22,188 | 4,07 | 0,067 |
| HPMC\_PS | 1 | 4,521 | 0,66% | 11,330 | 11,330 | 2,08 | 0,175 |
| Square | 4 | 4,854 | 0,71% | 8,372 | 2,093 | 0,38 | 0,816 |
| Lac\*Lac | 1 | 2,469 | 0,36% | 0,158 | 0,158 | 0,03 | 0,868 |
| HPMC\_Visc\*HPMC\_Visc | 1 | 0,338 | 0,05% | 4,669 | 4,669 | 0,86 | 0,373 |
| HPMC\_HP\*HPMC\_HP | 1 | 0,018 | 0,00% | 3,364 | 3,364 | 0,62 | 0,447 |
| HPMC\_PS\*HPMC\_PS | 1 | 2,029 | 0,30% | 0,835 | 0,835 | 0,15 | 0,702 |
| 2-Way Interaction | 6 | 50,059 | 7,34% | 50,059 | 8,343 | 1,53 | 0,249 |
| Lac\*HPMC\_Visc | 1 | 17,858 | 2,62% | 18,941 | 18,941 | 3,47 | 0,087 |
| Lac\*HPMC\_HP | 1 | 3,142 | 0,46% | 1,766 | 1,766 | 0,32 | 0,580 |
| Lac\*HPMC\_PS | 1 | 2,710 | 0,40% | 2,710 | 2,710 | 0,50 | 0,494 |
| HPMC\_Visc\*HPMC\_HP | 1 | 9,474 | 1,39% | 0,050 | 0,050 | 0,01 | 0,925 |
| HPMC\_Visc\*HPMC\_PS | 1 | 15,128 | 2,22% | 16,320 | 16,320 | 2,99 | 0,109 |
| HPMC\_HP\*HPMC\_PS | 1 | 1,747 | 0,26% | 1,747 | 1,747 | 0,32 | 0,582 |
| Error | 12 | 65,411 | 9,59% | 65,411 | 5,451 |  |  |
| Lack-of-Fit | 10 | 55,982 | 8,21% | 55,982 | 5,598 | 1,19 | 0,541 |
| Pure Error | 2 | 9,429 | 1,38% | 9,429 | 4,715 |  |  |
| Total | 26 | 682,265 | 100,00% |  |  |  |  |

## Regression Equation in Uncoded Units

|  |  |  |
| --- | --- | --- |
| F\_mean\_16h(960min) | = | 179 + 247 Lac - 0,0184 HPMC\_Visc - 3,8 HPMC\_HP - 1,15 HPMC\_PS - 5,6 Lac\*Lac + 0,000000 HPMC\_Visc\*HPMC\_Visc + 1,58 HPMC\_HP\*HPMC\_HP + 0,0149 HPMC\_PS\*HPMC\_PS - 0,00436 Lac\*HPMC\_Visc - 5,35 Lac\*HPMC\_HP - 1,41 Lac\*HPMC\_PS + 0,000066 HPMC\_Visc\*HPMC\_HP + 0,000232 HPMC\_Visc\*HPMC\_PS - 0,321 HPMC\_HP\*HPMC\_PS |

## Fits and Diagnostics for All Observations

| Obs | F\_mean\_16h(960min) | Fit | SE Fit | 95% CI | Resid | Std Resid | Del Resid | HI |
| --- | --- | --- | --- | --- | --- | --- | --- | --- |
| 1 | 82,78 | 81,30 | 1,89 | (77,18; 85,42) | 1,47 | 1,08 | 1,08 | 0,655994 |
| 2 | 93,04 | 94,33 | 1,89 | (90,21; 98,45) | -1,29 | -0,94 | -0,94 | 0,655994 |
| 3 | 76,24 | 77,73 | 1,99 | (73,39; 82,08) | -1,49 | -1,23 | -1,26 | 0,729945 |
| 4 | 86,55 | 85,72 | 1,99 | (81,38; 90,07) | 0,83 | 0,69 | 0,67 | 0,729945 |
| 5 | 80,14 | 83,22 | 1,61 | (79,71; 86,73) | -3,08 | -1,82 | -2,05 | 0,476813 |
| 6 | 92,41 | 94,53 | 1,61 | (91,02; 98,04) | -2,12 | -1,25 | -1,29 | 0,476813 |
| 7 | 83,12 | 83,78 | 1,94 | (79,56; 88,00) | -0,66 | -0,51 | -0,49 | 0,688750 |
| 8 | 89,65 | 89,53 | 1,94 | (85,31; 93,75) | 0,12 | 0,09 | 0,09 | 0,688750 |
| 9 | 79,34 | 81,82 | 1,78 | (77,94; 85,70) | -2,48 | -1,64 | -1,79 | 0,580949 |
| 10 | 94,54 | 92,99 | 1,78 | (89,11; 96,87) | 1,55 | 1,03 | 1,03 | 0,580949 |
| 11 | 86,17 | 83,23 | 1,52 | (79,91; 86,55) | 2,93 | 1,66 | 1,81 | 0,425781 |
| 12 | 90,27 | 90,91 | 1,52 | (87,59; 94,23) | -0,64 | -0,36 | -0,35 | 0,425781 |
| 13 | 82,72 | 82,98 | 1,76 | (79,15; 86,82) | -0,26 | -0,17 | -0,16 | 0,569258 |
| 14 | 92,61 | 92,44 | 1,76 | (88,60; 96,28) | 0,17 | 0,11 | 0,11 | 0,569258 |
| 15 | 83,95 | 85,11 | 1,54 | (81,77; 88,46) | -1,17 | -0,66 | -0,65 | 0,433227 |
| 16 | 89,92 | 91,38 | 1,54 | (88,03; 94,73) | -1,46 | -0,83 | -0,82 | 0,433227 |
| 17 | 78,58 | 77,16 | 1,81 | (73,22; 81,09) | 1,42 | 0,96 | 0,96 | 0,597775 |
| 18 | 96,32 | 95,85 | 1,81 | (91,92; 99,78) | 0,47 | 0,32 | 0,30 | 0,597775 |
| 19 | 91,50 | 88,91 | 1,49 | (85,66; 92,15) | 2,59 | 1,44 | 1,52 | 0,407038 |
| 20 | 87,19 | 86,63 | 1,88 | (82,54; 90,73) | 0,56 | 0,40 | 0,39 | 0,647412 |
| 21 | 85,26 | 86,17 | 1,49 | (82,92; 89,42) | -0,91 | -0,51 | -0,49 | 0,407933 |
| 22 | 92,11 | 90,62 | 2,11 | (86,02; 95,22) | 1,49 | 1,49 | 1,59 | 0,817044 |
| 23 | 87,83 | 86,97 | 2,12 | (82,36; 91,59) | 0,85 | 0,87 | 0,86 | 0,823434 |
| 24 | 88,43 | 88,59 | 1,97 | (84,30; 92,89) | -0,17 | -0,13 | -0,13 | 0,713775 |
| 25 | 85,15 | 86,85 | 1,25 | (84,12; 89,59) | -1,70 | -0,86 | -0,85 | 0,288793 |
| 26 | 87,17 | 86,85 | 1,25 | (84,12; 89,59) | 0,32 | 0,16 | 0,16 | 0,288793 |
| 27 | 89,49 | 86,85 | 1,25 | (84,12; 89,59) | 2,64 | 1,34 | 1,39 | 0,288793 |

| Obs | Cook’s D | DFITS |
| --- | --- | --- |
| 1 | 0,15 | 1,49733 |
| 2 | 0,11 | -1,29154 |
| 3 | 0,27 | -2,07001 |
| 4 | 0,08 | 1,10173 |
| 5 | 0,20 | -1,96035 |
| 6 | 0,10 | -1,23050 |
| 7 | 0,04 | -0,73289 |
| 8 | 0,00 | 0,13457 |
| 9 | 0,25 | -2,10237 |
| 10 | 0,10 | 1,21359 |
| 11 | 0,14 | 1,55767 |
| 12 | 0,01 | -0,30075 |
| 13 | 0,00 | -0,18858 |
| 14 | 0,00 | 0,12192 |
| 15 | 0,02 | -0,56610 |
| 16 | 0,04 | -0,71568 |
| 17 | 0,09 | 1,16711 |
| 18 | 0,01 | 0,37101 |
| 19 | 0,10 | 1,25842 |
| 20 | 0,02 | 0,52731 |
| 21 | 0,01 | -0,40799 |
| 22 | 0,66 | 3,35153 |
| 23 | 0,23 | 1,85286 |
| 24 | 0,00 | -0,20369 |
| 25 | 0,02 | -0,54395 |
| 26 | 0,00 | 0,09890 |
| 27 | 0,05 | 0,88684 |

## Coded Coefficients

| Term | Coef | SE Coef | 95% CI | T-Value | P-Value | VIF |
| --- | --- | --- | --- | --- | --- | --- |
| Constant | 88,99 | 1,26 | (86,25; 91,73) | 70,71 | 0,000 |  |
| Lac | 7,75 | 1,04 | (5,48; 10,02) | 7,45 | 0,000 | 1,18 |
| HPMC\_Visc | -0,43 | 1,09 | (-2,80; 1,94) | -0,40 | 0,698 | 1,70 |
| HPMC\_HP | 1,87 | 1,06 | (-0,45; 4,19) | 1,76 | 0,105 | 1,26 |
| HPMC\_PS | 2,44 | 1,63 | (-1,12; 5,99) | 1,49 | 0,161 | 2,09 |
| Lac\*Lac | -0,97 | 2,07 | (-5,47; 3,54) | -0,47 | 0,649 | 1,30 |
| HPMC\_Visc\*HPMC\_Visc | 2,07 | 2,20 | (-2,72; 6,86) | 0,94 | 0,365 | 1,96 |
| HPMC\_HP\*HPMC\_HP | 1,55 | 2,09 | (-2,99; 6,10) | 0,75 | 0,470 | 1,81 |
| HPMC\_PS\*HPMC\_PS | 0,67 | 2,07 | (-3,85; 5,19) | 0,32 | 0,752 | 1,42 |
| Lac\*HPMC\_Visc | -3,38 | 2,28 | (-8,35; 1,59) | -1,48 | 0,165 | 1,49 |
| Lac\*HPMC\_HP | -1,63 | 2,39 | (-6,85; 3,58) | -0,68 | 0,508 | 1,17 |
| Lac\*HPMC\_PS | -1,84 | 3,70 | (-9,90; 6,21) | -0,50 | 0,627 | 1,42 |
| HPMC\_Visc\*HPMC\_HP | 0,91 | 2,72 | (-5,02; 6,84) | 0,33 | 0,745 | 2,73 |
| HPMC\_Visc\*HPMC\_PS | 6,82 | 3,85 | (-1,56; 15,20) | 1,77 | 0,101 | 2,74 |
| HPMC\_HP\*HPMC\_PS | -1,61 | 4,25 | (-10,86; 7,64) | -0,38 | 0,711 | 2,70 |

## Model Summary

| S | R-sq | R-sq(adj) | PRESS | R-sq(pred) | AICc | BIC |
| --- | --- | --- | --- | --- | --- | --- |
| 2,34306 | 88,98% | 76,12% | 355,634 | 40,50% | 187,11 | 153,44 |

## Analysis of Variance

| Source | DF | Seq SS | Contribution | Adj SS | Adj MS | F-Value | P-Value |
| --- | --- | --- | --- | --- | --- | --- | --- |
| Model | 14 | 531,839 | 88,98% | 531,839 | 37,989 | 6,92 | 0,001 |
| Linear | 4 | 476,308 | 79,69% | 345,201 | 86,300 | 15,72 | 0,000 |
| Lac | 1 | 427,608 | 71,54% | 304,427 | 304,427 | 55,45 | 0,000 |
| HPMC\_Visc | 1 | 26,277 | 4,40% | 0,867 | 0,867 | 0,16 | 0,698 |
| HPMC\_HP | 1 | 18,440 | 3,09% | 16,920 | 16,920 | 3,08 | 0,105 |
| HPMC\_PS | 1 | 3,984 | 0,67% | 12,261 | 12,261 | 2,23 | 0,161 |
| Square | 4 | 8,555 | 1,43% | 11,335 | 2,834 | 0,52 | 0,726 |
| Lac\*Lac | 1 | 5,324 | 0,89% | 1,196 | 1,196 | 0,22 | 0,649 |
| HPMC\_Visc\*HPMC\_Visc | 1 | 0,272 | 0,05% | 4,875 | 4,875 | 0,89 | 0,365 |
| HPMC\_HP\*HPMC\_HP | 1 | 0,051 | 0,01% | 3,049 | 3,049 | 0,56 | 0,470 |
| HPMC\_PS\*HPMC\_PS | 1 | 2,908 | 0,49% | 0,575 | 0,575 | 0,10 | 0,752 |
| 2-Way Interaction | 6 | 46,976 | 7,86% | 46,976 | 7,829 | 1,43 | 0,282 |
| Lac\*HPMC\_Visc | 1 | 12,178 | 2,04% | 12,022 | 12,022 | 2,19 | 0,165 |
| Lac\*HPMC\_HP | 1 | 3,733 | 0,62% | 2,553 | 2,553 | 0,46 | 0,508 |
| Lac\*HPMC\_PS | 1 | 1,367 | 0,23% | 1,367 | 1,367 | 0,25 | 0,627 |
| HPMC\_Visc\*HPMC\_HP | 1 | 12,328 | 2,06% | 0,611 | 0,611 | 0,11 | 0,745 |
| HPMC\_Visc\*HPMC\_PS | 1 | 16,579 | 2,77% | 17,285 | 17,285 | 3,15 | 0,101 |
| HPMC\_HP\*HPMC\_PS | 1 | 0,791 | 0,13% | 0,791 | 0,791 | 0,14 | 0,711 |
| Error | 12 | 65,879 | 11,02% | 65,879 | 5,490 |  |  |
| Lack-of-Fit | 10 | 57,374 | 9,60% | 57,374 | 5,737 | 1,35 | 0,499 |
| Pure Error | 2 | 8,505 | 1,42% | 8,505 | 4,253 |  |  |
| Total | 26 | 597,718 | 100,00% |  |  |  |  |

## Regression Equation in Uncoded Units

|  |  |  |
| --- | --- | --- |
| F\_mean\_17h(1020min) | = | 274 + 224 Lac - 0,0209 HPMC\_Visc - 11,6 HPMC\_HP - 2,07 HPMC\_PS - 15,4 Lac\*Lac + 0,000000 HPMC\_Visc\*HPMC\_Visc + 1,51 HPMC\_HP\*HPMC\_HP + 0,0124 HPMC\_PS\*HPMC\_PS - 0,00347 Lac\*HPMC\_Visc - 6,43 Lac\*HPMC\_HP - 1,00 Lac\*HPMC\_PS + 0,000230 HPMC\_Visc\*HPMC\_HP + 0,000239 HPMC\_Visc\*HPMC\_PS - 0,216 HPMC\_HP\*HPMC\_PS |

## Fits and Diagnostics for All Observations

| Obs | F\_mean\_17h(1020min) | Fit | SE Fit | 95% CI | Resid | Std Resid | Del Resid |
| --- | --- | --- | --- | --- | --- | --- | --- |
| 1 | 86,29 | 84,49 | 1,90 | (80,36; 88,63) | 1,80 | 1,31 | 1,35 |
| 2 | 94,99 | 96,28 | 1,90 | (92,14; 100,41) | -1,28 | -0,93 | -0,93 |
| 3 | 78,51 | 79,97 | 2,00 | (75,61; 84,33) | -1,46 | -1,20 | -1,22 |
| 4 | 88,72 | 87,69 | 2,00 | (83,32; 92,05) | 1,03 | 0,85 | 0,84 |
| 5 | 82,55 | 85,69 | 1,62 | (82,16; 89,21) | -3,14 | -1,85 | -2,10 |
| 6 | 94,03 | 95,61 | 1,62 | (92,08; 99,13) | -1,58 | -0,93 | -0,93 |
| 7 | 85,47 | 85,94 | 1,94 | (81,70; 90,18) | -0,47 | -0,36 | -0,35 |
| 8 | 91,50 | 91,24 | 1,94 | (87,00; 95,47) | 0,27 | 0,21 | 0,20 |
| 9 | 81,53 | 84,32 | 1,79 | (80,43; 88,21) | -2,79 | -1,84 | -2,08 |
| 10 | 96,32 | 94,75 | 1,79 | (90,86; 98,64) | 1,57 | 1,03 | 1,04 |
| 11 | 88,49 | 85,35 | 1,53 | (82,02; 88,68) | 3,14 | 1,77 | 1,97 |
| 12 | 92,37 | 92,85 | 1,53 | (89,52; 96,18) | -0,48 | -0,27 | -0,26 |
| 13 | 84,96 | 85,20 | 1,77 | (81,35; 89,06) | -0,25 | -0,16 | -0,15 |
| 14 | 93,70 | 93,69 | 1,77 | (89,84; 97,55) | 0,01 | 0,00 | 0,00 |
| 15 | 86,05 | 87,33 | 1,54 | (83,97; 90,69) | -1,28 | -0,73 | -0,71 |
| 16 | 91,96 | 93,35 | 1,54 | (89,99; 96,71) | -1,39 | -0,79 | -0,77 |
| 17 | 80,95 | 79,51 | 1,81 | (75,56; 83,46) | 1,44 | 0,97 | 0,96 |
| 18 | 96,72 | 96,58 | 1,81 | (92,64; 100,53) | 0,14 | 0,09 | 0,09 |
| 19 | 93,61 | 91,04 | 1,49 | (87,78; 94,29) | 2,57 | 1,43 | 1,50 |
| 20 | 89,04 | 88,87 | 1,89 | (84,76; 92,98) | 0,16 | 0,12 | 0,11 |
| 21 | 87,57 | 88,70 | 1,50 | (85,44; 91,96) | -1,13 | -0,63 | -0,61 |
| 22 | 93,78 | 92,39 | 2,12 | (87,77; 97,00) | 1,40 | 1,39 | 1,46 |
| 23 | 89,59 | 89,01 | 2,13 | (84,38; 93,65) | 0,58 | 0,58 | 0,57 |
| 24 | 90,60 | 90,57 | 1,98 | (86,25; 94,88) | 0,03 | 0,03 | 0,03 |
| 25 | 87,29 | 89,01 | 1,26 | (86,27; 91,76) | -1,72 | -0,87 | -0,86 |
| 26 | 89,45 | 89,01 | 1,26 | (86,27; 91,76) | 0,44 | 0,22 | 0,21 |
| 27 | 91,41 | 89,01 | 1,26 | (86,27; 91,76) | 2,40 | 1,21 | 1,24 |

| Obs | HI | Cook’s D | DFITS |
| --- | --- | --- | --- |
| 1 | 0,655994 | 0,22 | 1,86544 |
| 2 | 0,655994 | 0,11 | -1,28263 |
| 3 | 0,729945 | 0,26 | -2,01062 |
| 4 | 0,729945 | 0,13 | 1,37976 |
| 5 | 0,476813 | 0,21 | -2,00121 |
| 6 | 0,476813 | 0,05 | -0,88403 |
| 7 | 0,688750 | 0,02 | -0,51827 |
| 8 | 0,688750 | 0,01 | 0,29282 |
| 9 | 0,580949 | 0,31 | -2,44735 |
| 10 | 0,580949 | 0,10 | 1,22068 |
| 11 | 0,425781 | 0,15 | 1,69718 |
| 12 | 0,425781 | 0,00 | -0,22192 |
| 13 | 0,569258 | 0,00 | -0,17700 |
| 14 | 0,569258 | 0,00 | 0,00413 |
| 15 | 0,433227 | 0,03 | -0,62259 |
| 16 | 0,433227 | 0,03 | -0,67756 |
| 17 | 0,597775 | 0,09 | 1,17590 |
| 18 | 0,597775 | 0,00 | 0,10885 |
| 19 | 0,407038 | 0,09 | 1,24056 |
| 20 | 0,647412 | 0,00 | 0,15350 |
| 21 | 0,407933 | 0,02 | -0,50506 |
| 22 | 0,817044 | 0,58 | 3,07880 |
| 23 | 0,823434 | 0,11 | 1,22573 |
| 24 | 0,713775 | 0,00 | 0,03966 |
| 25 | 0,288793 | 0,02 | -0,55022 |
| 26 | 0,288793 | 0,00 | 0,13644 |
| 27 | 0,288793 | 0,04 | 0,79052 |

## Coded Coefficients

| Term | Coef | SE Coef | 95% CI | T-Value | P-Value | VIF |
| --- | --- | --- | --- | --- | --- | --- |
| Constant | 91,02 | 1,22 | (88,35; 93,68) | 74,35 | 0,000 |  |
| Lac | 7,04 | 1,01 | (4,83; 9,24) | 6,95 | 0,000 | 1,18 |
| HPMC\_Visc | -0,31 | 1,06 | (-2,61; 2,00) | -0,29 | 0,777 | 1,70 |
| HPMC\_HP | 1,55 | 1,03 | (-0,71; 3,80) | 1,50 | 0,160 | 1,26 |
| HPMC\_PS | 2,47 | 1,59 | (-0,99; 5,93) | 1,56 | 0,146 | 2,09 |
| Lac\*Lac | -1,58 | 2,01 | (-5,96; 2,81) | -0,78 | 0,448 | 1,30 |
| HPMC\_Visc\*HPMC\_Visc | 1,75 | 2,14 | (-2,91; 6,40) | 0,82 | 0,430 | 1,96 |
| HPMC\_HP\*HPMC\_HP | 1,24 | 2,03 | (-3,18; 5,66) | 0,61 | 0,552 | 1,81 |
| HPMC\_PS\*HPMC\_PS | 0,36 | 2,02 | (-4,03; 4,76) | 0,18 | 0,860 | 1,42 |
| Lac\*HPMC\_Visc | -3,07 | 2,22 | (-7,90; 1,77) | -1,38 | 0,192 | 1,49 |
| Lac\*HPMC\_HP | -2,26 | 2,33 | (-7,33; 2,82) | -0,97 | 0,351 | 1,17 |
| Lac\*HPMC\_PS | -1,36 | 3,59 | (-9,20; 6,47) | -0,38 | 0,711 | 1,42 |
| HPMC\_Visc\*HPMC\_HP | 1,31 | 2,65 | (-4,46; 7,08) | 0,49 | 0,630 | 2,73 |
| HPMC\_Visc\*HPMC\_PS | 6,41 | 3,74 | (-1,74; 14,57) | 1,71 | 0,112 | 2,74 |
| HPMC\_HP\*HPMC\_PS | -1,27 | 4,13 | (-10,27; 7,73) | -0,31 | 0,764 | 2,70 |

## Model Summary

| S | R-sq | R-sq(adj) | PRESS | R-sq(pred) | AICc | BIC |
| --- | --- | --- | --- | --- | --- | --- |
| 2,27904 | 88,14% | 74,30% | 318,712 | 39,35% | 185,61 | 151,94 |

## Analysis of Variance

| Source | DF | Seq SS | Contribution | Adj SS | Adj MS | F-Value | P-Value |
| --- | --- | --- | --- | --- | --- | --- | --- |
| Model | 14 | 463,143 | 88,14% | 463,143 | 33,082 | 6,37 | 0,001 |
| Linear | 4 | 403,058 | 76,70% | 284,819 | 71,205 | 13,71 | 0,000 |
| Lac | 1 | 361,840 | 68,86% | 251,037 | 251,037 | 48,33 | 0,000 |
| HPMC\_Visc | 1 | 23,561 | 4,48% | 0,434 | 0,434 | 0,08 | 0,777 |
| HPMC\_HP | 1 | 12,778 | 2,43% | 11,627 | 11,627 | 2,24 | 0,160 |
| HPMC\_PS | 1 | 4,880 | 0,93% | 12,559 | 12,559 | 2,42 | 0,146 |
| Square | 4 | 12,590 | 2,40% | 12,499 | 3,125 | 0,60 | 0,669 |
| Lac\*Lac | 1 | 8,102 | 1,54% | 3,196 | 3,196 | 0,62 | 0,448 |
| HPMC\_Visc\*HPMC\_Visc | 1 | 0,482 | 0,09% | 3,461 | 3,461 | 0,67 | 0,430 |
| HPMC\_HP\*HPMC\_HP | 1 | 0,005 | 0,00% | 1,943 | 1,943 | 0,37 | 0,552 |
| HPMC\_PS\*HPMC\_PS | 1 | 4,001 | 0,76% | 0,168 | 0,168 | 0,03 | 0,860 |
| 2-Way Interaction | 6 | 47,495 | 9,04% | 47,495 | 7,916 | 1,52 | 0,251 |
| Lac\*HPMC\_Visc | 1 | 11,166 | 2,12% | 9,923 | 9,923 | 1,91 | 0,192 |
| Lac\*HPMC\_HP | 1 | 6,187 | 1,18% | 4,881 | 4,881 | 0,94 | 0,351 |
| Lac\*HPMC\_PS | 1 | 0,748 | 0,14% | 0,748 | 0,748 | 0,14 | 0,711 |
| HPMC\_Visc\*HPMC\_HP | 1 | 14,107 | 2,68% | 1,271 | 1,271 | 0,24 | 0,630 |
| HPMC\_Visc\*HPMC\_PS | 1 | 14,797 | 2,82% | 15,269 | 15,269 | 2,94 | 0,112 |
| HPMC\_HP\*HPMC\_PS | 1 | 0,491 | 0,09% | 0,491 | 0,491 | 0,09 | 0,764 |
| Error | 12 | 62,328 | 11,86% | 62,328 | 5,194 |  |  |
| Lack-of-Fit | 10 | 54,476 | 10,37% | 54,476 | 5,448 | 1,39 | 0,490 |
| Pure Error | 2 | 7,852 | 1,49% | 7,852 | 3,926 |  |  |
| Total | 26 | 525,472 | 100,00% |  |  |  |  |

## Regression Equation in Uncoded Units

|  |  |  |
| --- | --- | --- |
| F\_mean\_18h(1080min) | = | 248 + 232 Lac - 0,0204 HPMC\_Visc - 9,5 HPMC\_HP - 1,65 HPMC\_PS - 25,3 Lac\*Lac + 0,000000 HPMC\_Visc\*HPMC\_Visc + 1,20 HPMC\_HP\*HPMC\_HP + 0,0067 HPMC\_PS\*HPMC\_PS - 0,00315 Lac\*HPMC\_Visc - 8,90 Lac\*HPMC\_HP - 0,74 Lac\*HPMC\_PS + 0,000332 HPMC\_Visc\*HPMC\_HP + 0,000224 HPMC\_Visc\*HPMC\_PS - 0,170 HPMC\_HP\*HPMC\_PS |

## Fits and Diagnostics for All Observations

| Obs | F\_mean\_18h(1080min) | Fit | SE Fit | 95% CI | Resid | Std Resid | Del Resid |
| --- | --- | --- | --- | --- | --- | --- | --- |
| 1 | 88,26 | 86,65 | 1,85 | (82,62; 90,67) | 1,62 | 1,21 | 1,24 |
| 2 | 96,84 | 97,83 | 1,85 | (93,81; 101,85) | -0,99 | -0,74 | -0,73 |
| 3 | 80,68 | 81,98 | 1,95 | (77,73; 86,22) | -1,30 | -1,10 | -1,11 |
| 4 | 90,40 | 89,39 | 1,95 | (85,14; 93,63) | 1,01 | 0,86 | 0,85 |
| 5 | 84,58 | 87,70 | 1,57 | (84,27; 91,13) | -3,12 | -1,89 | -2,16 |
| 6 | 95,18 | 96,50 | 1,57 | (93,07; 99,93) | -1,32 | -0,80 | -0,78 |
| 7 | 87,83 | 88,13 | 1,89 | (84,01; 92,25) | -0,30 | -0,24 | -0,23 |
| 8 | 92,85 | 92,45 | 1,89 | (88,33; 96,57) | 0,39 | 0,31 | 0,30 |
| 9 | 83,55 | 86,34 | 1,74 | (82,56; 90,13) | -2,80 | -1,90 | -2,17 |
| 10 | 97,78 | 96,47 | 1,74 | (92,69; 100,26) | 1,30 | 0,88 | 0,87 |
| 11 | 90,55 | 87,35 | 1,49 | (84,11; 90,59) | 3,19 | 1,85 | 2,09 |
| 12 | 94,34 | 94,59 | 1,49 | (91,35; 97,83) | -0,25 | -0,14 | -0,14 |
| 13 | 86,94 | 87,15 | 1,72 | (83,41; 90,90) | -0,21 | -0,14 | -0,14 |
| 14 | 94,59 | 94,71 | 1,72 | (90,96; 98,46) | -0,11 | -0,08 | -0,07 |
| 15 | 87,89 | 89,44 | 1,50 | (86,17; 92,71) | -1,55 | -0,90 | -0,89 |
| 16 | 93,80 | 94,86 | 1,50 | (91,59; 98,13) | -1,06 | -0,62 | -0,60 |
| 17 | 83,21 | 81,66 | 1,76 | (77,82; 85,50) | 1,55 | 1,07 | 1,08 |
| 18 | 97,05 | 97,23 | 1,76 | (93,39; 101,06) | -0,18 | -0,12 | -0,12 |
| 19 | 95,40 | 92,70 | 1,45 | (89,53; 95,87) | 2,70 | 1,54 | 1,64 |
| 20 | 90,56 | 90,73 | 1,83 | (86,74; 94,73) | -0,18 | -0,13 | -0,12 |
| 21 | 89,65 | 90,79 | 1,46 | (87,62; 93,96) | -1,14 | -0,65 | -0,63 |
| 22 | 95,01 | 93,74 | 2,06 | (89,25; 98,23) | 1,28 | 1,31 | 1,35 |
| 23 | 91,01 | 90,60 | 2,07 | (86,09; 95,10) | 0,41 | 0,43 | 0,41 |
| 24 | 92,52 | 92,37 | 1,93 | (88,18; 96,57) | 0,15 | 0,12 | 0,12 |
| 25 | 89,22 | 91,02 | 1,22 | (88,35; 93,69) | -1,80 | -0,94 | -0,93 |
| 26 | 91,58 | 91,02 | 1,22 | (88,35; 93,69) | 0,56 | 0,29 | 0,28 |
| 27 | 93,16 | 91,02 | 1,22 | (88,35; 93,69) | 2,14 | 1,11 | 1,12 |

| Obs | HI | Cook’s D | DFITS |
| --- | --- | --- | --- |
| 1 | 0,655994 | 0,19 | 1,70674 |
| 2 | 0,655994 | 0,07 | -1,00306 |
| 3 | 0,729945 | 0,22 | -1,82249 |
| 4 | 0,729945 | 0,13 | 1,39043 |
| 5 | 0,476813 | 0,22 | -2,06202 |
| 6 | 0,476813 | 0,04 | -0,74939 |
| 7 | 0,688750 | 0,01 | -0,34157 |
| 8 | 0,688750 | 0,01 | 0,44263 |
| 9 | 0,580949 | 0,33 | -2,55523 |
| 10 | 0,580949 | 0,07 | 1,02952 |
| 11 | 0,425781 | 0,17 | 1,80251 |
| 12 | 0,425781 | 0,00 | -0,11947 |
| 13 | 0,569258 | 0,00 | -0,15543 |
| 14 | 0,569258 | 0,00 | -0,08459 |
| 15 | 0,433227 | 0,04 | -0,78153 |
| 16 | 0,433227 | 0,02 | -0,52467 |
| 17 | 0,597775 | 0,11 | 1,31427 |
| 18 | 0,597775 | 0,00 | -0,14244 |
| 19 | 0,407038 | 0,11 | 1,36005 |
| 20 | 0,647412 | 0,00 | -0,16858 |
| 21 | 0,407933 | 0,02 | -0,52574 |
| 22 | 0,817044 | 0,51 | 2,86286 |
| 23 | 0,823434 | 0,06 | 0,89273 |
| 24 | 0,713775 | 0,00 | 0,18735 |
| 25 | 0,288793 | 0,02 | -0,59389 |
| 26 | 0,288793 | 0,00 | 0,17855 |
| 27 | 0,288793 | 0,03 | 0,71572 |

## Coded Coefficients

| Term | Coef | SE Coef | 95% CI | T-Value | P-Value | VIF |
| --- | --- | --- | --- | --- | --- | --- |
| Constant | 92,75 | 1,22 | (90,09; 95,42) | 75,75 | 0,000 |  |
| Lac | 6,36 | 1,01 | (4,15; 8,56) | 6,28 | 0,000 | 1,18 |
| HPMC\_Visc | -0,13 | 1,06 | (-2,43; 2,18) | -0,12 | 0,904 | 1,70 |
| HPMC\_HP | 1,13 | 1,04 | (-1,12; 3,39) | 1,10 | 0,295 | 1,26 |
| HPMC\_PS | 2,44 | 1,59 | (-1,02; 5,89) | 1,53 | 0,151 | 2,09 |
| Lac\*Lac | -2,14 | 2,01 | (-6,52; 2,25) | -1,06 | 0,309 | 1,30 |
| HPMC\_Visc\*HPMC\_Visc | 1,35 | 2,14 | (-3,31; 6,01) | 0,63 | 0,541 | 1,96 |
| HPMC\_HP\*HPMC\_HP | 0,76 | 2,03 | (-3,67; 5,18) | 0,37 | 0,716 | 1,81 |
| HPMC\_PS\*HPMC\_PS | 0,13 | 2,02 | (-4,27; 4,52) | 0,06 | 0,951 | 1,42 |
| Lac\*HPMC\_Visc | -2,71 | 2,22 | (-7,55; 2,12) | -1,22 | 0,245 | 1,49 |
| Lac\*HPMC\_HP | -2,43 | 2,33 | (-7,50; 2,65) | -1,04 | 0,318 | 1,17 |
| Lac\*HPMC\_PS | -1,19 | 3,60 | (-9,03; 6,64) | -0,33 | 0,746 | 1,42 |
| HPMC\_Visc\*HPMC\_HP | 1,35 | 2,65 | (-4,42; 7,13) | 0,51 | 0,618 | 2,73 |
| HPMC\_Visc\*HPMC\_PS | 5,98 | 3,74 | (-2,17; 14,13) | 1,60 | 0,136 | 2,74 |
| HPMC\_HP\*HPMC\_PS | -1,30 | 4,13 | (-10,30; 7,70) | -0,31 | 0,759 | 2,70 |

## Model Summary

| S | R-sq | R-sq(adj) | PRESS | R-sq(pred) | AICc | BIC |
| --- | --- | --- | --- | --- | --- | --- |
| 2,27974 | 86,26% | 70,24% | 305,526 | 32,71% | 185,63 | 151,96 |

## Analysis of Variance

| Source | DF | Seq SS | Contribution | Adj SS | Adj MS | F-Value | P-Value |
| --- | --- | --- | --- | --- | --- | --- | --- |
| Model | 14 | 391,654 | 86,26% | 391,654 | 27,975 | 5,38 | 0,003 |
| Linear | 4 | 331,264 | 72,96% | 229,998 | 57,500 | 11,06 | 0,001 |
| Lac | 1 | 298,769 | 65,81% | 204,742 | 204,742 | 39,39 | 0,000 |
| HPMC\_Visc | 1 | 18,375 | 4,05% | 0,078 | 0,078 | 0,02 | 0,904 |
| HPMC\_HP | 1 | 8,048 | 1,77% | 6,233 | 6,233 | 1,20 | 0,295 |
| HPMC\_PS | 1 | 6,073 | 1,34% | 12,233 | 12,233 | 2,35 | 0,151 |
| Square | 4 | 16,634 | 3,66% | 13,369 | 3,342 | 0,64 | 0,642 |
| Lac\*Lac | 1 | 10,769 | 2,37% | 5,867 | 5,867 | 1,13 | 0,309 |
| HPMC\_Visc\*HPMC\_Visc | 1 | 0,975 | 0,21% | 2,061 | 2,061 | 0,40 | 0,541 |
| HPMC\_HP\*HPMC\_HP | 1 | 0,199 | 0,04% | 0,723 | 0,723 | 0,14 | 0,716 |
| HPMC\_PS\*HPMC\_PS | 1 | 4,692 | 1,03% | 0,021 | 0,021 | 0,00 | 0,951 |
| 2-Way Interaction | 6 | 43,756 | 9,64% | 43,756 | 7,293 | 1,40 | 0,290 |
| Lac\*HPMC\_Visc | 1 | 8,954 | 1,97% | 7,770 | 7,770 | 1,50 | 0,245 |
| Lac\*HPMC\_HP | 1 | 6,923 | 1,52% | 5,642 | 5,642 | 1,09 | 0,318 |
| Lac\*HPMC\_PS | 1 | 0,571 | 0,13% | 0,571 | 0,571 | 0,11 | 0,746 |
| HPMC\_Visc\*HPMC\_HP | 1 | 14,005 | 3,08% | 1,358 | 1,358 | 0,26 | 0,618 |
| HPMC\_Visc\*HPMC\_PS | 1 | 12,789 | 2,82% | 13,266 | 13,266 | 2,55 | 0,136 |
| HPMC\_HP\*HPMC\_PS | 1 | 0,513 | 0,11% | 0,513 | 0,513 | 0,10 | 0,759 |
| Error | 12 | 62,366 | 13,74% | 62,366 | 5,197 |  |  |
| Lack-of-Fit | 10 | 54,778 | 12,07% | 54,778 | 5,478 | 1,44 | 0,477 |
| Pure Error | 2 | 7,588 | 1,67% | 7,588 | 3,794 |  |  |
| Total | 26 | 454,020 | 100,00% |  |  |  |  |

## Regression Equation in Uncoded Units

|  |  |  |
| --- | --- | --- |
| F\_mean\_19h(1140min) | = | 171 + 233 Lac - 0,0188 HPMC\_Visc - 0,6 HPMC\_HP - 0,85 HPMC\_PS - 34,2 Lac\*Lac + 0,000000 HPMC\_Visc\*HPMC\_Visc + 0,73 HPMC\_HP\*HPMC\_HP + 0,0023 HPMC\_PS\*HPMC\_PS - 0,00279 Lac\*HPMC\_Visc - 9,57 Lac\*HPMC\_HP - 0,65 Lac\*HPMC\_PS + 0,000343 HPMC\_Visc\*HPMC\_HP + 0,000209 HPMC\_Visc\*HPMC\_PS - 0,174 HPMC\_HP\*HPMC\_PS |

## Fits and Diagnostics for All Observations

| Obs | F\_mean\_19h(1140min) | Fit | SE Fit | 95% CI | Resid | Std Resid | Del Resid |
| --- | --- | --- | --- | --- | --- | --- | --- |
| 1 | 89,69 | 88,33 | 1,85 | (84,30; 92,35) | 1,37 | 1,02 | 1,02 |
| 2 | 97,82 | 98,66 | 1,85 | (94,64; 102,68) | -0,84 | -0,63 | -0,61 |
| 3 | 82,74 | 83,83 | 1,95 | (79,59; 88,07) | -1,09 | -0,92 | -0,92 |
| 4 | 91,74 | 90,83 | 1,95 | (86,58; 95,07) | 0,91 | 0,77 | 0,75 |
| 5 | 86,12 | 89,34 | 1,57 | (85,91; 92,77) | -3,22 | -1,95 | -2,27 |
| 6 | 96,01 | 97,17 | 1,57 | (93,74; 100,60) | -1,15 | -0,70 | -0,68 |
| 7 | 89,48 | 89,76 | 1,89 | (85,64; 93,88) | -0,27 | -0,21 | -0,21 |
| 8 | 93,94 | 93,50 | 1,89 | (89,38; 97,62) | 0,44 | 0,35 | 0,33 |
| 9 | 85,34 | 88,16 | 1,74 | (84,37; 91,94) | -2,82 | -1,91 | -2,20 |
| 10 | 98,82 | 97,55 | 1,74 | (93,76; 101,33) | 1,27 | 0,86 | 0,85 |
| 11 | 92,47 | 89,27 | 1,49 | (86,03; 92,51) | 3,20 | 1,85 | 2,10 |
| 12 | 95,75 | 96,01 | 1,49 | (92,77; 99,25) | -0,26 | -0,15 | -0,15 |
| 13 | 88,65 | 88,70 | 1,72 | (84,95; 92,44) | -0,04 | -0,03 | -0,03 |
| 14 | 94,93 | 95,34 | 1,72 | (91,60; 99,09) | -0,42 | -0,28 | -0,27 |
| 15 | 89,40 | 91,20 | 1,50 | (87,93; 94,47) | -1,80 | -1,05 | -1,05 |
| 16 | 95,49 | 96,05 | 1,50 | (92,78; 99,32) | -0,56 | -0,33 | -0,32 |
| 17 | 85,24 | 83,56 | 1,76 | (79,72; 87,40) | 1,68 | 1,16 | 1,18 |
| 18 | 97,28 | 97,63 | 1,76 | (93,79; 101,47) | -0,35 | -0,24 | -0,23 |
| 19 | 96,84 | 93,93 | 1,45 | (90,76; 97,10) | 2,91 | 1,66 | 1,81 |
| 20 | 91,99 | 92,36 | 1,83 | (88,36; 96,35) | -0,36 | -0,27 | -0,26 |
| 21 | 91,34 | 92,41 | 1,46 | (89,24; 95,58) | -1,08 | -0,61 | -0,60 |
| 22 | 95,78 | 94,55 | 2,06 | (90,06; 99,04) | 1,24 | 1,27 | 1,31 |
| 23 | 92,35 | 91,97 | 2,07 | (87,47; 96,48) | 0,37 | 0,39 | 0,38 |
| 24 | 94,13 | 93,95 | 1,93 | (89,75; 98,15) | 0,18 | 0,15 | 0,14 |
| 25 | 90,83 | 92,73 | 1,23 | (90,06; 95,40) | -1,90 | -0,99 | -0,99 |
| 26 | 93,46 | 92,73 | 1,23 | (90,06; 95,40) | 0,73 | 0,38 | 0,36 |
| 27 | 94,64 | 92,73 | 1,23 | (90,06; 95,40) | 1,90 | 0,99 | 0,99 |

| Obs | HI | Cook’s D | DFITS |
| --- | --- | --- | --- |
| 1 | 0,655994 | 0,13 | 1,41431 |
| 2 | 0,655994 | 0,05 | -0,84809 |
| 3 | 0,729945 | 0,15 | -1,50574 |
| 4 | 0,729945 | 0,11 | 1,23624 |
| 5 | 0,476813 | 0,23 | -2,16441 |
| 6 | 0,476813 | 0,03 | -0,65227 |
| 7 | 0,688750 | 0,01 | -0,30654 |
| 8 | 0,688750 | 0,02 | 0,49781 |
| 9 | 0,580949 | 0,34 | -2,58599 |
| 10 | 0,580949 | 0,07 | 0,99992 |
| 11 | 0,425781 | 0,17 | 1,80527 |
| 12 | 0,425781 | 0,00 | -0,12540 |
| 13 | 0,569258 | 0,00 | -0,03220 |
| 14 | 0,569258 | 0,01 | -0,30830 |
| 15 | 0,433227 | 0,06 | -0,92225 |
| 16 | 0,433227 | 0,01 | -0,27575 |
| 17 | 0,597775 | 0,13 | 1,44192 |
| 18 | 0,597775 | 0,01 | -0,28627 |
| 19 | 0,407038 | 0,13 | 1,49646 |
| 20 | 0,647412 | 0,01 | -0,35072 |
| 21 | 0,407933 | 0,02 | -0,49501 |
| 22 | 0,817044 | 0,48 | 2,75986 |
| 23 | 0,823434 | 0,05 | 0,81320 |
| 24 | 0,713775 | 0,00 | 0,21962 |
| 25 | 0,288793 | 0,03 | -0,62961 |
| 26 | 0,288793 | 0,00 | 0,23230 |
| 27 | 0,288793 | 0,03 | 0,63002 |

## Coded Coefficients

| Term | Coef | SE Coef | 95% CI | T-Value | P-Value | VIF |
| --- | --- | --- | --- | --- | --- | --- |
| Constant | 94,29 | 1,21 | (91,66; 96,92) | 78,17 | 0,000 |  |
| Lac | 5,618 | 0,997 | (3,445; 7,791) | 5,63 | 0,000 | 1,18 |
| HPMC\_Visc | 0,05 | 1,04 | (-2,22; 2,32) | 0,05 | 0,964 | 1,70 |
| HPMC\_HP | 0,77 | 1,02 | (-1,45; 2,99) | 0,75 | 0,466 | 1,26 |
| HPMC\_PS | 2,40 | 1,56 | (-1,00; 5,81) | 1,54 | 0,150 | 2,09 |
| Lac\*Lac | -2,53 | 1,98 | (-6,84; 1,79) | -1,27 | 0,227 | 1,30 |
| HPMC\_Visc\*HPMC\_Visc | 0,87 | 2,11 | (-3,72; 5,46) | 0,41 | 0,687 | 1,96 |
| HPMC\_HP\*HPMC\_HP | 0,20 | 2,00 | (-4,15; 4,56) | 0,10 | 0,921 | 1,81 |
| HPMC\_PS\*HPMC\_PS | -0,15 | 1,99 | (-4,48; 4,18) | -0,08 | 0,940 | 1,42 |
| Lac\*HPMC\_Visc | -2,34 | 2,19 | (-7,11; 2,42) | -1,07 | 0,305 | 1,49 |
| Lac\*HPMC\_HP | -2,58 | 2,29 | (-7,58; 2,42) | -1,12 | 0,283 | 1,17 |
| Lac\*HPMC\_PS | -0,81 | 3,54 | (-8,53; 6,90) | -0,23 | 0,822 | 1,42 |
| HPMC\_Visc\*HPMC\_HP | 1,61 | 2,61 | (-4,08; 7,30) | 0,62 | 0,549 | 2,73 |
| HPMC\_Visc\*HPMC\_PS | 5,40 | 3,69 | (-2,63; 13,43) | 1,47 | 0,169 | 2,74 |
| HPMC\_HP\*HPMC\_PS | -1,13 | 4,07 | (-10,00; 7,73) | -0,28 | 0,786 | 2,70 |

## Model Summary

| S | R-sq | R-sq(adj) | PRESS | R-sq(pred) | AICc | BIC |
| --- | --- | --- | --- | --- | --- | --- |
| 2,24557 | 84,26% | 65,90% | 284,194 | 26,09% | 184,81 | 151,14 |

## Analysis of Variance

| Source | DF | Seq SS | Contribution | Adj SS | Adj MS | F-Value | P-Value |
| --- | --- | --- | --- | --- | --- | --- | --- |
| Model | 14 | 324,016 | 84,26% | 324,016 | 23,144 | 4,59 | 0,006 |
| Linear | 4 | 263,249 | 68,46% | 179,198 | 44,799 | 8,88 | 0,001 |
| Lac | 1 | 237,011 | 61,64% | 159,978 | 159,978 | 31,73 | 0,000 |
| HPMC\_Visc | 1 | 14,015 | 3,64% | 0,011 | 0,011 | 0,00 | 0,964 |
| HPMC\_HP | 1 | 4,753 | 1,24% | 2,854 | 2,854 | 0,57 | 0,466 |
| HPMC\_PS | 1 | 7,470 | 1,94% | 11,901 | 11,901 | 2,36 | 0,150 |
| Square | 4 | 20,003 | 5,20% | 13,207 | 3,302 | 0,65 | 0,635 |
| Lac\*Lac | 1 | 11,850 | 3,08% | 8,182 | 8,182 | 1,62 | 0,227 |
| HPMC\_Visc\*HPMC\_Visc | 1 | 1,680 | 0,44% | 0,861 | 0,861 | 0,17 | 0,687 |
| HPMC\_HP\*HPMC\_HP | 1 | 0,911 | 0,24% | 0,052 | 0,052 | 0,01 | 0,921 |
| HPMC\_PS\*HPMC\_PS | 1 | 5,561 | 1,45% | 0,029 | 0,029 | 0,01 | 0,940 |
| 2-Way Interaction | 6 | 40,764 | 10,60% | 40,764 | 6,794 | 1,35 | 0,310 |
| Lac\*HPMC\_Visc | 1 | 7,277 | 1,89% | 5,791 | 5,791 | 1,15 | 0,305 |
| Lac\*HPMC\_HP | 1 | 7,432 | 1,93% | 6,372 | 6,372 | 1,26 | 0,283 |
| Lac\*HPMC\_PS | 1 | 0,267 | 0,07% | 0,267 | 0,267 | 0,05 | 0,822 |
| HPMC\_Visc\*HPMC\_HP | 1 | 14,939 | 3,89% | 1,921 | 1,921 | 0,38 | 0,549 |
| HPMC\_Visc\*HPMC\_PS | 1 | 10,459 | 2,72% | 10,827 | 10,827 | 2,15 | 0,169 |
| HPMC\_HP\*HPMC\_PS | 1 | 0,390 | 0,10% | 0,390 | 0,390 | 0,08 | 0,786 |
| Error | 12 | 60,511 | 15,74% | 60,511 | 5,043 |  |  |
| Lack-of-Fit | 10 | 53,021 | 13,79% | 53,021 | 5,302 | 1,42 | 0,483 |
| Pure Error | 2 | 7,490 | 1,95% | 7,490 | 3,745 |  |  |
| Total | 26 | 384,527 | 100,00% |  |  |  |  |

## Regression Equation in Uncoded Units

|  |  |  |
| --- | --- | --- |
| F\_mean\_20h(1200min) | = | 107 + 223 Lac - 0,0174 HPMC\_Visc + 7,2 HPMC\_HP - 0,17 HPMC\_PS - 40,4 Lac\*Lac + 0,000000 HPMC\_Visc\*HPMC\_Visc + 0,20 HPMC\_HP\*HPMC\_HP - 0,0028 HPMC\_PS\*HPMC\_PS - 0,00241 Lac\*HPMC\_Visc - 10,17 Lac\*HPMC\_HP - 0,44 Lac\*HPMC\_PS + 0,000408 HPMC\_Visc\*HPMC\_HP + 0,000189 HPMC\_Visc\*HPMC\_PS - 0,152 HPMC\_HP\*HPMC\_PS |

## Fits and Diagnostics for All Observations

| Obs | F\_mean\_20h(1200min) | Fit | SE Fit | 95% CI | Resid | Std Resid | Del Resid |
| --- | --- | --- | --- | --- | --- | --- | --- |
| 1 | 91,09 | 89,92 | 1,82 | (85,96; 93,88) | 1,17 | 0,89 | 0,88 |
| 2 | 98,57 | 99,27 | 1,82 | (95,30; 103,23) | -0,70 | -0,53 | -0,51 |
| 3 | 84,65 | 85,52 | 1,92 | (81,34; 89,70) | -0,87 | -0,75 | -0,73 |
| 4 | 92,73 | 91,93 | 1,92 | (87,75; 96,11) | 0,80 | 0,68 | 0,67 |
| 5 | 87,77 | 90,80 | 1,55 | (87,42; 94,18) | -3,02 | -1,86 | -2,11 |
| 6 | 96,43 | 97,55 | 1,55 | (94,17; 100,93) | -1,12 | -0,69 | -0,67 |
| 7 | 91,14 | 91,32 | 1,86 | (87,26; 95,38) | -0,18 | -0,14 | -0,13 |
| 8 | 94,77 | 94,37 | 1,86 | (90,31; 98,43) | 0,40 | 0,32 | 0,31 |
| 9 | 86,97 | 89,79 | 1,71 | (86,06; 93,51) | -2,81 | -1,93 | -2,23 |
| 10 | 99,59 | 98,44 | 1,71 | (94,71; 102,17) | 1,15 | 0,79 | 0,77 |
| 11 | 94,14 | 90,95 | 1,47 | (87,76; 94,15) | 3,19 | 1,87 | 2,13 |
| 12 | 96,67 | 97,18 | 1,47 | (93,99; 100,37) | -0,51 | -0,30 | -0,29 |
| 13 | 90,12 | 90,05 | 1,69 | (86,36; 93,74) | 0,07 | 0,05 | 0,04 |
| 14 | 95,06 | 95,83 | 1,69 | (92,14; 99,52) | -0,77 | -0,52 | -0,51 |
| 15 | 90,72 | 92,80 | 1,48 | (89,58; 96,02) | -2,08 | -1,23 | -1,26 |
| 16 | 96,90 | 97,09 | 1,48 | (93,87; 100,31) | -0,19 | -0,11 | -0,11 |
| 17 | 87,07 | 85,49 | 1,74 | (81,71; 89,27) | 1,58 | 1,11 | 1,12 |
| 18 | 97,73 | 97,94 | 1,74 | (94,16; 101,72) | -0,21 | -0,15 | -0,14 |
| 19 | 97,92 | 94,90 | 1,43 | (91,78; 98,03) | 3,01 | 1,74 | 1,93 |
| 20 | 93,23 | 93,68 | 1,81 | (89,75; 97,62) | -0,46 | -0,34 | -0,33 |
| 21 | 92,80 | 93,77 | 1,43 | (90,64; 96,89) | -0,97 | -0,56 | -0,54 |
| 22 | 96,33 | 95,12 | 2,03 | (90,69; 99,54) | 1,22 | 1,27 | 1,30 |
| 23 | 93,32 | 93,08 | 2,04 | (88,64; 97,52) | 0,24 | 0,26 | 0,25 |
| 24 | 95,63 | 95,28 | 1,90 | (91,15; 99,41) | 0,35 | 0,29 | 0,28 |
| 25 | 92,29 | 94,24 | 1,21 | (91,61; 96,87) | -1,95 | -1,03 | -1,03 |
| 26 | 95,16 | 94,24 | 1,21 | (91,61; 96,87) | 0,92 | 0,49 | 0,47 |
| 27 | 95,97 | 94,24 | 1,21 | (91,61; 96,87) | 1,74 | 0,92 | 0,91 |

| Obs | HI | Cook’s D | DFITS |
| --- | --- | --- | --- |
| 1 | 0,655994 | 0,10 | 1,21614 |
| 2 | 0,655994 | 0,04 | -0,70648 |
| 3 | 0,729945 | 0,10 | -1,20424 |
| 4 | 0,729945 | 0,08 | 1,09791 |
| 5 | 0,476813 | 0,21 | -2,01713 |
| 6 | 0,476813 | 0,03 | -0,64427 |
| 7 | 0,688750 | 0,00 | -0,19935 |
| 8 | 0,688750 | 0,01 | 0,45590 |
| 9 | 0,580949 | 0,35 | -2,62884 |
| 10 | 0,580949 | 0,06 | 0,91207 |
| 11 | 0,425781 | 0,17 | 1,83810 |
| 12 | 0,425781 | 0,00 | -0,24769 |
| 13 | 0,569258 | 0,00 | 0,04998 |
| 14 | 0,569258 | 0,02 | -0,58100 |
| 15 | 0,433227 | 0,08 | -1,09908 |
| 16 | 0,433227 | 0,00 | -0,09384 |
| 17 | 0,597775 | 0,12 | 1,36772 |
| 18 | 0,597775 | 0,00 | -0,17448 |
| 19 | 0,407038 | 0,14 | 1,59788 |
| 20 | 0,647412 | 0,01 | -0,44525 |
| 21 | 0,407933 | 0,01 | -0,45177 |
| 22 | 0,817044 | 0,48 | 2,75150 |
| 23 | 0,823434 | 0,02 | 0,53229 |
| 24 | 0,713775 | 0,01 | 0,43945 |
| 25 | 0,288793 | 0,03 | -0,65711 |
| 26 | 0,288793 | 0,01 | 0,30088 |
| 27 | 0,288793 | 0,02 | 0,57960 |

## Coded Coefficients

| Term | Coef | SE Coef | 95% CI | T-Value | P-Value | VIF |
| --- | --- | --- | --- | --- | --- | --- |
| Constant | 95,59 | 1,22 | (92,92; 98,25) | 78,07 | 0,000 |  |
| Lac | 4,85 | 1,01 | (2,65; 7,06) | 4,79 | 0,000 | 1,18 |
| HPMC\_Visc | 0,26 | 1,06 | (-2,05; 2,56) | 0,24 | 0,812 | 1,70 |
| HPMC\_HP | 0,45 | 1,03 | (-1,81; 2,70) | 0,43 | 0,674 | 1,26 |
| HPMC\_PS | 2,31 | 1,59 | (-1,15; 5,76) | 1,45 | 0,172 | 2,09 |
| Lac\*Lac | -2,98 | 2,01 | (-7,36; 1,41) | -1,48 | 0,165 | 1,30 |
| HPMC\_Visc\*HPMC\_Visc | 0,48 | 2,14 | (-4,18; 5,14) | 0,22 | 0,827 | 1,96 |
| HPMC\_HP\*HPMC\_HP | -0,24 | 2,03 | (-4,66; 4,18) | -0,12 | 0,908 | 1,81 |
| HPMC\_PS\*HPMC\_PS | -0,41 | 2,02 | (-4,80; 3,99) | -0,20 | 0,843 | 1,42 |
| Lac\*HPMC\_Visc | -1,86 | 2,22 | (-6,70; 2,97) | -0,84 | 0,418 | 1,49 |
| Lac\*HPMC\_HP | -2,47 | 2,33 | (-7,54; 2,61) | -1,06 | 0,311 | 1,17 |
| Lac\*HPMC\_PS | -0,33 | 3,59 | (-8,16; 7,50) | -0,09 | 0,928 | 1,42 |
| HPMC\_Visc\*HPMC\_HP | 2,16 | 2,65 | (-3,61; 7,93) | 0,82 | 0,431 | 2,73 |
| HPMC\_Visc\*HPMC\_PS | 4,82 | 3,74 | (-3,33; 12,97) | 1,29 | 0,222 | 2,74 |
| HPMC\_HP\*HPMC\_PS | -0,87 | 4,13 | (-9,87; 8,12) | -0,21 | 0,836 | 2,70 |

## Model Summary

| S | R-sq | R-sq(adj) | PRESS | R-sq(pred) | AICc | BIC |
| --- | --- | --- | --- | --- | --- | --- |
| 2,27929 | 80,70% | 58,19% | 286,080 | 11,44% | 185,62 | 151,95 |

## Analysis of Variance

| Source | DF | Seq SS | Contribution | Adj SS | Adj MS | F-Value | P-Value |
| --- | --- | --- | --- | --- | --- | --- | --- |
| Model | 14 | 260,696 | 80,70% | 260,696 | 18,621 | 3,58 | 0,016 |
| Linear | 4 | 197,425 | 61,12% | 133,472 | 33,368 | 6,42 | 0,005 |
| Lac | 1 | 177,182 | 54,85% | 119,335 | 119,335 | 22,97 | 0,000 |
| HPMC\_Visc | 1 | 10,124 | 3,13% | 0,306 | 0,306 | 0,06 | 0,812 |
| HPMC\_HP | 1 | 2,154 | 0,67% | 0,968 | 0,968 | 0,19 | 0,674 |
| HPMC\_PS | 1 | 7,965 | 2,47% | 10,961 | 10,961 | 2,11 | 0,172 |
| Square | 4 | 24,307 | 7,52% | 15,064 | 3,766 | 0,72 | 0,592 |
| Lac\*Lac | 1 | 13,969 | 4,32% | 11,364 | 11,364 | 2,19 | 0,165 |
| HPMC\_Visc\*HPMC\_Visc | 1 | 2,196 | 0,68% | 0,258 | 0,258 | 0,05 | 0,827 |
| HPMC\_HP\*HPMC\_HP | 1 | 1,588 | 0,49% | 0,072 | 0,072 | 0,01 | 0,908 |
| HPMC\_PS\*HPMC\_PS | 1 | 6,555 | 2,03% | 0,212 | 0,212 | 0,04 | 0,843 |
| 2-Way Interaction | 6 | 38,964 | 12,06% | 38,964 | 6,494 | 1,25 | 0,348 |
| Lac\*HPMC\_Visc | 1 | 5,259 | 1,63% | 3,659 | 3,659 | 0,70 | 0,418 |
| Lac\*HPMC\_HP | 1 | 6,429 | 1,99% | 5,820 | 5,820 | 1,12 | 0,311 |
| Lac\*HPMC\_PS | 1 | 0,044 | 0,01% | 0,044 | 0,044 | 0,01 | 0,928 |
| HPMC\_Visc\*HPMC\_HP | 1 | 18,618 | 5,76% | 3,452 | 3,452 | 0,66 | 0,431 |
| HPMC\_Visc\*HPMC\_PS | 1 | 8,383 | 2,59% | 8,612 | 8,612 | 1,66 | 0,222 |
| HPMC\_HP\*HPMC\_PS | 1 | 0,232 | 0,07% | 0,232 | 0,232 | 0,04 | 0,836 |
| Error | 12 | 62,342 | 19,30% | 62,342 | 5,195 |  |  |
| Lack-of-Fit | 10 | 55,496 | 17,18% | 55,496 | 5,550 | 1,62 | 0,441 |
| Pure Error | 2 | 6,846 | 2,12% | 6,846 | 3,423 |  |  |
| Total | 26 | 323,038 | 100,00% |  |  |  |  |

## Regression Equation in Uncoded Units

|  |  |  |
| --- | --- | --- |
| F\_mean\_21h(1260min) | = | 81 + 197 Lac - 0,0167 HPMC\_Visc + 10,5 HPMC\_HP + 0,29 HPMC\_PS - 47,6 Lac\*Lac + 0,000000 HPMC\_Visc\*HPMC\_Visc - 0,23 HPMC\_HP\*HPMC\_HP - 0,0075 HPMC\_PS\*HPMC\_PS - 0,00192 Lac\*HPMC\_Visc - 9,72 Lac\*HPMC\_HP - 0,18 Lac\*HPMC\_PS + 0,000547 HPMC\_Visc\*HPMC\_HP + 0,000168 HPMC\_Visc\*HPMC\_PS - 0,117 HPMC\_HP\*HPMC\_PS |

## Fits and Diagnostics for All Observations

| Obs | F\_mean\_21h(1260min) | Fit | SE Fit | 95% CI | Resid | Std Resid | Del Resid |
| --- | --- | --- | --- | --- | --- | --- | --- |
| 1 | 92,78 | 91,64 | 1,85 | (87,62; 95,66) | 1,13 | 0,85 | 0,84 |
| 2 | 99,08 | 99,68 | 1,85 | (95,65; 103,70) | -0,60 | -0,45 | -0,43 |
| 3 | 86,40 | 87,08 | 1,95 | (82,83; 91,32) | -0,67 | -0,57 | -0,55 |
| 4 | 93,38 | 92,69 | 1,95 | (88,45; 96,93) | 0,69 | 0,58 | 0,57 |
| 5 | 89,11 | 92,02 | 1,57 | (88,59; 95,45) | -2,91 | -1,77 | -1,97 |
| 6 | 96,64 | 97,64 | 1,57 | (94,21; 101,06) | -0,99 | -0,60 | -0,59 |
| 7 | 92,64 | 92,77 | 1,89 | (88,65; 96,90) | -0,13 | -0,10 | -0,10 |
| 8 | 95,76 | 95,26 | 1,89 | (91,14; 99,38) | 0,50 | 0,39 | 0,38 |
| 9 | 88,39 | 91,40 | 1,74 | (87,62; 95,19) | -3,01 | -2,04 | -2,42 |
| 10 | 100,23 | 99,08 | 1,74 | (95,30; 102,87) | 1,15 | 0,78 | 0,77 |
| 11 | 95,69 | 92,39 | 1,49 | (89,15; 95,63) | 3,30 | 1,91 | 2,19 |
| 12 | 97,35 | 97,99 | 1,49 | (94,75; 101,23) | -0,65 | -0,38 | -0,36 |
| 13 | 91,33 | 91,08 | 1,72 | (87,33; 94,82) | 0,25 | 0,17 | 0,16 |
| 14 | 94,95 | 96,06 | 1,72 | (92,31; 99,80) | -1,10 | -0,74 | -0,72 |
| 15 | 91,80 | 94,13 | 1,50 | (90,86; 97,40) | -2,33 | -1,36 | -1,41 |
| 16 | 97,98 | 97,94 | 1,50 | (94,67; 101,21) | 0,04 | 0,02 | 0,02 |
| 17 | 88,70 | 87,18 | 1,76 | (83,34; 91,02) | 1,52 | 1,05 | 1,06 |
| 18 | 97,69 | 97,87 | 1,76 | (94,03; 101,71) | -0,19 | -0,13 | -0,12 |
| 19 | 98,77 | 95,70 | 1,45 | (92,53; 98,87) | 3,07 | 1,75 | 1,94 |
| 20 | 94,24 | 94,84 | 1,83 | (90,84; 98,83) | -0,59 | -0,44 | -0,42 |
| 21 | 94,09 | 95,01 | 1,46 | (91,84; 98,18) | -0,92 | -0,53 | -0,51 |
| 22 | 96,73 | 95,57 | 2,06 | (91,08; 100,05) | 1,17 | 1,20 | 1,22 |
| 23 | 94,12 | 94,03 | 2,07 | (89,52; 98,54) | 0,09 | 0,09 | 0,09 |
| 24 | 96,83 | 96,31 | 1,93 | (92,12; 100,51) | 0,52 | 0,42 | 0,41 |
| 25 | 93,60 | 95,50 | 1,22 | (92,83; 98,17) | -1,90 | -0,99 | -0,99 |
| 26 | 96,59 | 95,50 | 1,22 | (92,83; 98,17) | 1,09 | 0,57 | 0,55 |
| 27 | 96,98 | 95,50 | 1,22 | (92,83; 98,17) | 1,48 | 0,77 | 0,76 |

| Obs | HI | Cook’s D | DFITS |  |
| --- | --- | --- | --- | --- |
| 1 | 0,655994 | 0,09 | 1,15778 |  |
| 2 | 0,655994 | 0,03 | -0,59410 |  |
| 3 | 0,729945 | 0,06 | -0,90812 |  |
| 4 | 0,729945 | 0,06 | 0,93264 |  |
| 5 | 0,476813 | 0,19 | -1,87818 |  |
| 6 | 0,476813 | 0,02 | -0,55889 |  |
| 7 | 0,688750 | 0,00 | -0,14805 |  |
| 8 | 0,688750 | 0,02 | 0,56413 |  |
| 9 | 0,580949 | 0,39 | -2,85067 | R |
| 10 | 0,580949 | 0,06 | 0,90075 |  |
| 11 | 0,425781 | 0,18 | 1,88625 |  |
| 12 | 0,425781 | 0,01 | -0,31131 |  |
| 13 | 0,569258 | 0,00 | 0,18533 |  |
| 14 | 0,569258 | 0,05 | -0,83213 |  |
| 15 | 0,433227 | 0,09 | -1,23316 |  |
| 16 | 0,433227 | 0,00 | 0,01801 |  |
| 17 | 0,597775 | 0,11 | 1,28850 |  |
| 18 | 0,597775 | 0,00 | -0,14990 |  |
| 19 | 0,407038 | 0,14 | 1,60904 |  |
| 20 | 0,647412 | 0,02 | -0,57391 |  |
| 21 | 0,407933 | 0,01 | -0,42401 |  |
| 22 | 0,817044 | 0,43 | 2,58478 |  |
| 23 | 0,823434 | 0,00 | 0,19588 |  |
| 24 | 0,713775 | 0,03 | 0,64465 |  |
| 25 | 0,288793 | 0,03 | -0,62894 |  |
| 26 | 0,288793 | 0,01 | 0,35178 |  |
| 27 | 0,288793 | 0,02 | 0,48246 |  |

R  Large residual

## Coded Coefficients

| Term | Coef | SE Coef | 95% CI | T-Value | P-Value | VIF |
| --- | --- | --- | --- | --- | --- | --- |
| Constant | 96,70 | 1,22 | (94,05; 99,35) | 79,43 | 0,000 |  |
| Lac | 4,01 | 1,01 | (1,82; 6,20) | 3,98 | 0,002 | 1,18 |
| HPMC\_Visc | 0,42 | 1,05 | (-1,87; 2,71) | 0,40 | 0,699 | 1,70 |
| HPMC\_HP | 0,15 | 1,03 | (-2,09; 2,40) | 0,15 | 0,883 | 1,26 |
| HPMC\_PS | 2,16 | 1,58 | (-1,28; 5,60) | 1,37 | 0,196 | 2,09 |
| Lac\*Lac | -3,42 | 2,00 | (-7,78; 0,94) | -1,71 | 0,113 | 1,30 |
| HPMC\_Visc\*HPMC\_Visc | 0,05 | 2,13 | (-4,58; 4,68) | 0,02 | 0,982 | 1,96 |
| HPMC\_HP\*HPMC\_HP | -0,73 | 2,02 | (-5,12; 3,67) | -0,36 | 0,724 | 1,81 |
| HPMC\_PS\*HPMC\_PS | -0,66 | 2,01 | (-5,03; 3,71) | -0,33 | 0,748 | 1,42 |
| Lac\*HPMC\_Visc | -1,60 | 2,21 | (-6,41; 3,21) | -0,73 | 0,482 | 1,49 |
| Lac\*HPMC\_HP | -2,26 | 2,32 | (-7,30; 2,79) | -0,97 | 0,349 | 1,17 |
| Lac\*HPMC\_PS | -0,18 | 3,57 | (-7,97; 7,61) | -0,05 | 0,961 | 1,42 |
| HPMC\_Visc\*HPMC\_HP | 2,40 | 2,63 | (-3,34; 8,14) | 0,91 | 0,380 | 2,73 |
| HPMC\_Visc\*HPMC\_PS | 4,25 | 3,72 | (-3,85; 12,36) | 1,14 | 0,275 | 2,74 |
| HPMC\_HP\*HPMC\_PS | -0,77 | 4,11 | (-9,72; 8,17) | -0,19 | 0,854 | 2,70 |

## Model Summary

| S | R-sq | R-sq(adj) | PRESS | R-sq(pred) | AICc | BIC |
| --- | --- | --- | --- | --- | --- | --- |
| 2,26640 | 76,83% | 49,79% | 281,518 | 0,00% | 185,31 | 151,64 |

## Analysis of Variance

| Source | DF | Seq SS | Contribution | Adj SS | Adj MS | F-Value | P-Value |
| --- | --- | --- | --- | --- | --- | --- | --- |
| Model | 14 | 204,350 | 76,83% | 204,350 | 14,5964 | 2,84 | 0,039 |
| Linear | 4 | 138,961 | 52,24% | 92,212 | 23,0531 | 4,49 | 0,019 |
| Lac | 1 | 123,030 | 46,25% | 81,528 | 81,5280 | 15,87 | 0,002 |
| HPMC\_Visc | 1 | 6,783 | 2,55% | 0,807 | 0,8068 | 0,16 | 0,699 |
| HPMC\_HP | 1 | 0,880 | 0,33% | 0,115 | 0,1154 | 0,02 | 0,883 |
| HPMC\_PS | 1 | 8,268 | 3,11% | 9,636 | 9,6364 | 1,88 | 0,196 |
| Square | 4 | 29,402 | 11,05% | 17,520 | 4,3800 | 0,85 | 0,519 |
| Lac\*Lac | 1 | 16,061 | 6,04% | 14,999 | 14,9988 | 2,92 | 0,113 |
| HPMC\_Visc\*HPMC\_Visc | 1 | 3,025 | 1,14% | 0,003 | 0,0029 | 0,00 | 0,982 |
| HPMC\_HP\*HPMC\_HP | 1 | 2,894 | 1,09% | 0,669 | 0,6693 | 0,13 | 0,724 |
| HPMC\_PS\*HPMC\_PS | 1 | 7,422 | 2,79% | 0,553 | 0,5534 | 0,11 | 0,748 |
| 2-Way Interaction | 6 | 35,987 | 13,53% | 35,987 | 5,9979 | 1,17 | 0,384 |
| Lac\*HPMC\_Visc | 1 | 4,086 | 1,54% | 2,707 | 2,7073 | 0,53 | 0,482 |
| Lac\*HPMC\_HP | 1 | 5,301 | 1,99% | 4,879 | 4,8789 | 0,95 | 0,349 |
| Lac\*HPMC\_PS | 1 | 0,013 | 0,00% | 0,013 | 0,0129 | 0,00 | 0,961 |
| HPMC\_Visc\*HPMC\_HP | 1 | 19,867 | 7,47% | 4,262 | 4,2618 | 0,83 | 0,380 |
| HPMC\_Visc\*HPMC\_PS | 1 | 6,539 | 2,46% | 6,718 | 6,7178 | 1,31 | 0,275 |
| HPMC\_HP\*HPMC\_PS | 1 | 0,182 | 0,07% | 0,182 | 0,1820 | 0,04 | 0,854 |
| Error | 12 | 61,639 | 23,17% | 61,639 | 5,1366 |  |  |
| Lack-of-Fit | 10 | 55,527 | 20,88% | 55,527 | 5,5527 | 1,82 | 0,407 |
| Pure Error | 2 | 6,112 | 2,30% | 6,112 | 3,0559 |  |  |
| Total | 26 | 265,988 | 100,00% |  |  |  |  |

## Regression Equation in Uncoded Units

|  |  |  |
| --- | --- | --- |
| F\_mean\_22h(1320min) | = | 22 + 184 Lac - 0,0153 HPMC\_Visc + 17,0 HPMC\_HP + 1,01 HPMC\_PS - 54,7 Lac\*Lac + 0,000000 HPMC\_Visc\*HPMC\_Visc - 0,71 HPMC\_HP\*HPMC\_HP - 0,0122 HPMC\_PS\*HPMC\_PS - 0,00165 Lac\*HPMC\_Visc - 8,90 Lac\*HPMC\_HP - 0,10 Lac\*HPMC\_PS + 0,000608 HPMC\_Visc\*HPMC\_HP + 0,000149 HPMC\_Visc\*HPMC\_PS - 0,104 HPMC\_HP\*HPMC\_PS |

## Fits and Diagnostics for All Observations

| Obs | F\_mean\_22h(1320min) | Fit | SE Fit | 95% CI | Resid | Std Resid | Del Resid |
| --- | --- | --- | --- | --- | --- | --- | --- |
| 1 | 94,06 | 92,94 | 1,84 | (88,94; 96,94) | 1,12 | 0,84 | 0,83 |
| 2 | 99,13 | 99,76 | 1,84 | (95,76; 103,76) | -0,63 | -0,47 | -0,46 |
| 3 | 87,98 | 88,53 | 1,94 | (84,31; 92,75) | -0,54 | -0,46 | -0,44 |
| 4 | 93,86 | 93,23 | 1,94 | (89,01; 97,45) | 0,63 | 0,53 | 0,52 |
| 5 | 90,34 | 93,08 | 1,56 | (89,67; 96,49) | -2,73 | -1,67 | -1,82 |
| 6 | 96,86 | 97,70 | 1,56 | (94,29; 101,11) | -0,84 | -0,51 | -0,49 |
| 7 | 93,92 | 93,97 | 1,88 | (89,87; 98,07) | -0,05 | -0,04 | -0,04 |
| 8 | 96,34 | 95,84 | 1,88 | (91,74; 99,94) | 0,50 | 0,39 | 0,38 |
| 9 | 89,63 | 92,76 | 1,73 | (88,99; 96,52) | -3,13 | -2,13 | -2,59 |
| 10 | 100,47 | 99,34 | 1,73 | (95,57; 103,10) | 1,13 | 0,77 | 0,76 |
| 11 | 97,02 | 93,77 | 1,48 | (90,55; 96,99) | 3,24 | 1,89 | 2,16 |
| 12 | 97,61 | 98,51 | 1,48 | (95,29; 101,74) | -0,91 | -0,53 | -0,51 |
| 13 | 92,37 | 92,00 | 1,71 | (88,28; 95,73) | 0,37 | 0,25 | 0,24 |
| 14 | 94,72 | 96,12 | 1,71 | (92,40; 99,85) | -1,40 | -0,94 | -0,94 |
| 15 | 92,90 | 95,34 | 1,49 | (92,09; 98,59) | -2,44 | -1,43 | -1,50 |
| 16 | 98,69 | 98,46 | 1,49 | (95,21; 101,71) | 0,23 | 0,13 | 0,13 |
| 17 | 90,13 | 88,73 | 1,75 | (84,91; 92,55) | 1,40 | 0,97 | 0,97 |
| 18 | 97,57 | 97,61 | 1,75 | (93,79; 101,42) | -0,04 | -0,03 | -0,02 |
| 19 | 99,38 | 96,30 | 1,45 | (93,15; 99,45) | 3,08 | 1,77 | 1,97 |
| 20 | 95,13 | 95,77 | 1,82 | (91,80; 99,75) | -0,65 | -0,48 | -0,46 |
| 21 | 95,20 | 95,94 | 1,45 | (92,79; 99,09) | -0,74 | -0,42 | -0,41 |
| 22 | 96,94 | 95,85 | 2,05 | (91,38; 100,31) | 1,10 | 1,13 | 1,15 |
| 23 | 94,80 | 94,85 | 2,06 | (90,37; 99,33) | -0,05 | -0,05 | -0,05 |
| 24 | 97,84 | 97,14 | 1,91 | (92,97; 101,31) | 0,70 | 0,57 | 0,56 |
| 25 | 94,78 | 96,59 | 1,22 | (93,93; 99,24) | -1,80 | -0,94 | -0,94 |
| 26 | 97,80 | 96,59 | 1,22 | (93,93; 99,24) | 1,21 | 0,64 | 0,62 |
| 27 | 97,82 | 96,59 | 1,22 | (93,93; 99,24) | 1,24 | 0,65 | 0,63 |

| Obs | HI | Cook’s D | DFITS |  |
| --- | --- | --- | --- | --- |
| 1 | 0,655994 | 0,09 | 1,14509 |  |
| 2 | 0,655994 | 0,03 | -0,63255 |  |
| 3 | 0,729945 | 0,04 | -0,73020 |  |
| 4 | 0,729945 | 0,05 | 0,85007 |  |
| 5 | 0,476813 | 0,17 | -1,73880 |  |
| 6 | 0,476813 | 0,02 | -0,47196 |  |
| 7 | 0,688750 | 0,00 | -0,05693 |  |
| 8 | 0,688750 | 0,02 | 0,56378 |  |
| 9 | 0,580949 | 0,42 | -3,04744 | R |
| 10 | 0,580949 | 0,06 | 0,89316 |  |
| 11 | 0,425781 | 0,18 | 1,85666 |  |
| 12 | 0,425781 | 0,01 | -0,44118 |  |
| 13 | 0,569258 | 0,01 | 0,27228 |  |
| 14 | 0,569258 | 0,08 | -1,07802 |  |
| 15 | 0,433227 | 0,10 | -1,31307 |  |
| 16 | 0,433227 | 0,00 | 0,11273 |  |
| 17 | 0,597775 | 0,09 | 1,18494 |  |
| 18 | 0,597775 | 0,00 | -0,03017 |  |
| 19 | 0,407038 | 0,14 | 1,63013 |  |
| 20 | 0,647412 | 0,03 | -0,62924 |  |
| 21 | 0,407933 | 0,01 | -0,34011 |  |
| 22 | 0,817044 | 0,38 | 2,42077 |  |
| 23 | 0,823434 | 0,00 | -0,11362 |  |
| 24 | 0,713775 | 0,05 | 0,87977 |  |
| 25 | 0,288793 | 0,02 | -0,59743 |  |
| 26 | 0,288793 | 0,01 | 0,39448 |  |
| 27 | 0,288793 | 0,01 | 0,40247 |  |

R  Large residual

## Coded Coefficients

| Term | Coef | SE Coef | 95% CI | T-Value | P-Value | VIF |
| --- | --- | --- | --- | --- | --- | --- |
| Constant | 97,62 | 1,20 | (95,01; 100,23) | 81,41 | 0,000 |  |
| Lac | 3,181 | 0,992 | (1,021; 5,342) | 3,21 | 0,008 | 1,18 |
| HPMC\_Visc | 0,55 | 1,04 | (-1,71; 2,80) | 0,53 | 0,607 | 1,70 |
| HPMC\_HP | -0,07 | 1,01 | (-2,28; 2,13) | -0,07 | 0,943 | 1,26 |
| HPMC\_PS | 2,08 | 1,55 | (-1,31; 5,47) | 1,34 | 0,206 | 2,09 |
| Lac\*Lac | -3,78 | 1,97 | (-8,08; 0,51) | -1,92 | 0,079 | 1,30 |
| HPMC\_Visc\*HPMC\_Visc | -0,42 | 2,09 | (-4,99; 4,14) | -0,20 | 0,843 | 1,96 |
| HPMC\_HP\*HPMC\_HP | -1,12 | 1,99 | (-5,45; 3,21) | -0,56 | 0,583 | 1,81 |
| HPMC\_PS\*HPMC\_PS | -0,93 | 1,98 | (-5,23; 3,38) | -0,47 | 0,648 | 1,42 |
| Lac\*HPMC\_Visc | -1,55 | 2,17 | (-6,28; 3,19) | -0,71 | 0,490 | 1,49 |
| Lac\*HPMC\_HP | -2,04 | 2,28 | (-7,01; 2,93) | -0,89 | 0,389 | 1,17 |
| Lac\*HPMC\_PS | -0,09 | 3,52 | (-7,77; 7,58) | -0,03 | 0,979 | 1,42 |
| HPMC\_Visc\*HPMC\_HP | 2,43 | 2,59 | (-3,22; 8,09) | 0,94 | 0,367 | 2,73 |
| HPMC\_Visc\*HPMC\_PS | 3,75 | 3,66 | (-4,24; 11,73) | 1,02 | 0,327 | 2,74 |
| HPMC\_HP\*HPMC\_PS | -0,65 | 4,04 | (-9,47; 8,16) | -0,16 | 0,874 | 2,70 |

## Model Summary

| S | R-sq | R-sq(adj) | PRESS | R-sq(pred) | AICc | BIC |
| --- | --- | --- | --- | --- | --- | --- |
| 2,23232 | 72,89% | 41,27% | 276,051 | 0,00% | 184,49 | 150,83 |

## Analysis of Variance

| Source | DF | Seq SS | Contribution | Adj SS | Adj MS | F-Value | P-Value |
| --- | --- | --- | --- | --- | --- | --- | --- |
| Model | 14 | 160,805 | 72,89% | 160,805 | 11,4861 | 2,30 | 0,077 |
| Linear | 4 | 94,188 | 42,70% | 60,648 | 15,1621 | 3,04 | 0,060 |
| Lac | 1 | 80,459 | 36,47% | 51,297 | 51,2974 | 10,29 | 0,008 |
| HPMC\_Visc | 1 | 4,262 | 1,93% | 1,390 | 1,3899 | 0,28 | 0,607 |
| HPMC\_HP | 1 | 0,348 | 0,16% | 0,027 | 0,0267 | 0,01 | 0,943 |
| HPMC\_PS | 1 | 9,120 | 4,13% | 8,926 | 8,9256 | 1,79 | 0,206 |
| Square | 4 | 34,480 | 15,63% | 19,801 | 4,9503 | 0,99 | 0,448 |
| Lac\*Lac | 1 | 17,518 | 7,94% | 18,352 | 18,3519 | 3,68 | 0,079 |
| HPMC\_Visc\*HPMC\_Visc | 1 | 4,429 | 2,01% | 0,205 | 0,2045 | 0,04 | 0,843 |
| HPMC\_HP\*HPMC\_HP | 1 | 4,166 | 1,89% | 1,590 | 1,5905 | 0,32 | 0,583 |
| HPMC\_PS\*HPMC\_PS | 1 | 8,368 | 3,79% | 1,095 | 1,0952 | 0,22 | 0,648 |
| 2-Way Interaction | 6 | 32,136 | 14,57% | 32,136 | 5,3561 | 1,07 | 0,429 |
| Lac\*HPMC\_Visc | 1 | 3,895 | 1,77% | 2,529 | 2,5286 | 0,51 | 0,490 |
| Lac\*HPMC\_HP | 1 | 4,279 | 1,94% | 3,979 | 3,9786 | 0,80 | 0,389 |
| Lac\*HPMC\_PS | 1 | 0,004 | 0,00% | 0,004 | 0,0036 | 0,00 | 0,979 |
| HPMC\_Visc\*HPMC\_HP | 1 | 18,744 | 8,50% | 4,383 | 4,3834 | 0,88 | 0,367 |
| HPMC\_Visc\*HPMC\_PS | 1 | 5,084 | 2,30% | 5,213 | 5,2134 | 1,05 | 0,327 |
| HPMC\_HP\*HPMC\_PS | 1 | 0,130 | 0,06% | 0,130 | 0,1300 | 0,03 | 0,874 |
| Error | 12 | 59,799 | 27,11% | 59,799 | 4,9833 |  |  |
| Lack-of-Fit | 10 | 54,606 | 24,75% | 54,606 | 5,4606 | 2,10 | 0,365 |
| Pure Error | 2 | 5,193 | 2,35% | 5,193 | 2,5965 |  |  |
| Total | 26 | 220,604 | 100,00% |  |  |  |  |

## Regression Equation in Uncoded Units

|  |  |  |
| --- | --- | --- |
| F\_mean\_23h(1380min) | = | -37 + 174 Lac - 0,0133 HPMC\_Visc + 22,4 HPMC\_HP + 1,75 HPMC\_PS - 60,5 Lac\*Lac - 0,000000 HPMC\_Visc\*HPMC\_Visc - 1,09 HPMC\_HP\*HPMC\_HP - 0,0171 HPMC\_PS\*HPMC\_PS - 0,00159 Lac\*HPMC\_Visc - 8,03 Lac\*HPMC\_HP - 0,05 Lac\*HPMC\_PS + 0,000616 HPMC\_Visc\*HPMC\_HP + 0,000131 HPMC\_Visc\*HPMC\_PS - 0,088 HPMC\_HP\*HPMC\_PS |

## Fits and Diagnostics for All Observations

| Obs | F\_mean\_23h(1380min) | Fit | SE Fit | 95% CI | Resid | Std Resid | Del Resid |
| --- | --- | --- | --- | --- | --- | --- | --- |
| 1 | 94,91 | 93,87 | 1,81 | (89,94; 97,81) | 1,04 | 0,79 | 0,78 |
| 2 | 99,06 | 99,65 | 1,81 | (95,71; 103,59) | -0,59 | -0,45 | -0,44 |
| 3 | 89,49 | 89,87 | 1,91 | (85,71; 94,02) | -0,38 | -0,33 | -0,31 |
| 4 | 94,10 | 93,57 | 1,91 | (89,41; 97,73) | 0,53 | 0,46 | 0,44 |
| 5 | 91,57 | 93,92 | 1,54 | (90,56; 97,28) | -2,35 | -1,45 | -1,53 |
| 6 | 96,95 | 97,70 | 1,54 | (94,35; 101,06) | -0,75 | -0,47 | -0,45 |
| 7 | 94,89 | 94,94 | 1,85 | (90,90; 98,98) | -0,05 | -0,04 | -0,04 |
| 8 | 96,68 | 96,09 | 1,85 | (92,05; 100,13) | 0,59 | 0,48 | 0,46 |
| 9 | 90,50 | 93,78 | 1,70 | (90,08; 97,49) | -3,28 | -2,27 | -2,87 |
| 10 | 100,49 | 99,38 | 1,70 | (95,68; 103,09) | 1,11 | 0,77 | 0,75 |
| 11 | 98,17 | 95,02 | 1,46 | (91,85; 98,19) | 3,15 | 1,86 | 2,11 |
| 12 | 97,60 | 98,86 | 1,46 | (95,68; 102,03) | -1,26 | -0,74 | -0,73 |
| 13 | 93,21 | 92,80 | 1,68 | (89,13; 96,47) | 0,41 | 0,28 | 0,27 |
| 14 | 94,53 | 96,19 | 1,68 | (92,52; 99,86) | -1,66 | -1,13 | -1,15 |
| 15 | 93,93 | 96,43 | 1,47 | (93,23; 99,63) | -2,50 | -1,49 | -1,58 |
| 16 | 99,12 | 98,79 | 1,47 | (95,59; 102,00) | 0,32 | 0,19 | 0,18 |
| 17 | 91,38 | 90,11 | 1,73 | (86,35; 93,87) | 1,27 | 0,90 | 0,89 |
| 18 | 97,43 | 97,28 | 1,73 | (93,52; 101,04) | 0,15 | 0,10 | 0,10 |
| 19 | 99,71 | 96,67 | 1,42 | (93,57; 99,77) | 3,03 | 1,77 | 1,96 |
| 20 | 95,79 | 96,45 | 1,80 | (92,54; 100,37) | -0,66 | -0,50 | -0,48 |
| 21 | 96,19 | 96,67 | 1,43 | (93,57; 99,78) | -0,49 | -0,28 | -0,27 |
| 22 | 97,12 | 96,13 | 2,02 | (91,73; 100,52) | 1,00 | 1,05 | 1,05 |
| 23 | 95,16 | 95,42 | 2,03 | (91,00; 99,83) | -0,25 | -0,27 | -0,26 |
| 24 | 98,74 | 97,79 | 1,89 | (93,68; 101,90) | 0,95 | 0,80 | 0,78 |
| 25 | 95,85 | 97,48 | 1,20 | (94,87; 100,09) | -1,63 | -0,87 | -0,86 |
| 26 | 98,74 | 97,48 | 1,20 | (94,87; 100,09) | 1,26 | 0,67 | 0,65 |
| 27 | 98,52 | 97,48 | 1,20 | (94,87; 100,09) | 1,04 | 0,55 | 0,54 |

| Obs | HI | Cook’s D | DFITS |  |
| --- | --- | --- | --- | --- |
| 1 | 0,655994 | 0,08 | 1,07594 |  |
| 2 | 0,655994 | 0,03 | -0,60226 |  |
| 3 | 0,729945 | 0,02 | -0,51764 |  |
| 4 | 0,729945 | 0,04 | 0,72526 |  |
| 5 | 0,476813 | 0,13 | -1,46217 |  |
| 6 | 0,476813 | 0,01 | -0,43019 |  |
| 7 | 0,688750 | 0,00 | -0,05675 |  |
| 8 | 0,688750 | 0,03 | 0,68516 |  |
| 9 | 0,580949 | 0,48 | -3,38486 | R |
| 10 | 0,580949 | 0,05 | 0,88426 |  |
| 11 | 0,425781 | 0,17 | 1,81665 |  |
| 12 | 0,425781 | 0,03 | -0,62771 |  |
| 13 | 0,569258 | 0,01 | 0,30748 |  |
| 14 | 0,569258 | 0,11 | -1,31658 |  |
| 15 | 0,433227 | 0,11 | -1,37715 |  |
| 16 | 0,433227 | 0,00 | 0,16011 |  |
| 17 | 0,597775 | 0,08 | 1,08541 |  |
| 18 | 0,597775 | 0,00 | 0,12028 |  |
| 19 | 0,407038 | 0,14 | 1,62751 |  |
| 20 | 0,647412 | 0,03 | -0,65656 |  |
| 21 | 0,407933 | 0,00 | -0,22569 |  |
| 22 | 0,817044 | 0,33 | 2,22017 |  |
| 23 | 0,823434 | 0,02 | -0,55377 |  |
| 24 | 0,713775 | 0,11 | 1,23715 |  |
| 25 | 0,288793 | 0,02 | -0,54693 |  |
| 26 | 0,288793 | 0,01 | 0,41537 |  |
| 27 | 0,288793 | 0,01 | 0,34288 |  |

R  Large residual

## Coded Coefficients

| Term | Coef | SE Coef | 95% CI | T-Value | P-Value | VIF |
| --- | --- | --- | --- | --- | --- | --- |
| Constant | 98,42 | 1,18 | (95,85; 100,98) | 83,61 | 0,000 |  |
| Lac | 2,384 | 0,973 | (0,263; 4,505) | 2,45 | 0,031 | 1,18 |
| HPMC\_Visc | 0,62 | 1,02 | (-1,60; 2,83) | 0,61 | 0,556 | 1,70 |
| HPMC\_HP | -0,288 | 0,995 | (-2,456; 1,880) | -0,29 | 0,777 | 1,26 |
| HPMC\_PS | 1,96 | 1,53 | (-1,36; 5,29) | 1,28 | 0,223 | 2,09 |
| Lac\*Lac | -4,13 | 1,93 | (-8,35; 0,08) | -2,14 | 0,054 | 1,30 |
| HPMC\_Visc\*HPMC\_Visc | -0,91 | 2,06 | (-5,39; 3,57) | -0,44 | 0,667 | 1,96 |
| HPMC\_HP\*HPMC\_HP | -1,46 | 1,95 | (-5,71; 2,79) | -0,75 | 0,468 | 1,81 |
| HPMC\_PS\*HPMC\_PS | -1,26 | 1,94 | (-5,48; 2,97) | -0,65 | 0,528 | 1,42 |
| Lac\*HPMC\_Visc | -1,76 | 2,13 | (-6,41; 2,89) | -0,83 | 0,425 | 1,49 |
| Lac\*HPMC\_HP | -1,92 | 2,24 | (-6,80; 2,96) | -0,86 | 0,409 | 1,17 |
| Lac\*HPMC\_PS | -0,12 | 3,46 | (-7,66; 7,41) | -0,04 | 0,972 | 1,42 |
| HPMC\_Visc\*HPMC\_HP | 2,42 | 2,55 | (-3,13; 7,97) | 0,95 | 0,361 | 2,73 |
| HPMC\_Visc\*HPMC\_PS | 3,20 | 3,60 | (-4,64; 11,04) | 0,89 | 0,391 | 2,74 |
| HPMC\_HP\*HPMC\_PS | -0,69 | 3,97 | (-9,34; 7,97) | -0,17 | 0,866 | 2,70 |

## Model Summary

| S | R-sq | R-sq(adj) | PRESS | R-sq(pred) | AICc | BIC |
| --- | --- | --- | --- | --- | --- | --- |
| 2,19152 | 69,74% | 34,43% | 273,911 | 0,00% | 183,50 | 149,83 |

## Analysis of Variance

| Source | DF | Seq SS | Contribution | Adj SS | Adj MS | F-Value | P-Value |
| --- | --- | --- | --- | --- | --- | --- | --- |
| Model | 14 | 132,799 | 69,74% | 132,799 | 9,4857 | 1,98 | 0,122 |
| Linear | 4 | 62,413 | 32,77% | 37,403 | 9,3508 | 1,95 | 0,167 |
| Lac | 1 | 49,714 | 26,11% | 28,813 | 28,8130 | 6,00 | 0,031 |
| HPMC\_Visc | 1 | 2,718 | 1,43% | 1,764 | 1,7642 | 0,37 | 0,556 |
| HPMC\_HP | 1 | 0,079 | 0,04% | 0,402 | 0,4022 | 0,08 | 0,777 |
| HPMC\_PS | 1 | 9,901 | 5,20% | 7,924 | 7,9236 | 1,65 | 0,223 |
| Square | 4 | 40,093 | 21,05% | 22,579 | 5,6449 | 1,18 | 0,370 |
| Lac\*Lac | 1 | 18,843 | 9,89% | 21,917 | 21,9174 | 4,56 | 0,054 |
| HPMC\_Visc\*HPMC\_Visc | 1 | 6,072 | 3,19% | 0,933 | 0,9329 | 0,19 | 0,667 |
| HPMC\_HP\*HPMC\_HP | 1 | 5,347 | 2,81% | 2,701 | 2,7007 | 0,56 | 0,468 |
| HPMC\_PS\*HPMC\_PS | 1 | 9,832 | 5,16% | 2,025 | 2,0252 | 0,42 | 0,528 |
| 2-Way Interaction | 6 | 30,293 | 15,91% | 30,293 | 5,0489 | 1,05 | 0,441 |
| Lac\*HPMC\_Visc | 1 | 4,901 | 2,57% | 3,281 | 3,2812 | 0,68 | 0,425 |
| Lac\*HPMC\_HP | 1 | 3,807 | 2,00% | 3,519 | 3,5193 | 0,73 | 0,409 |
| Lac\*HPMC\_PS | 1 | 0,006 | 0,00% | 0,006 | 0,0063 | 0,00 | 0,972 |
| HPMC\_Visc\*HPMC\_HP | 1 | 17,768 | 9,33% | 4,323 | 4,3230 | 0,90 | 0,361 |
| HPMC\_Visc\*HPMC\_PS | 1 | 3,669 | 1,93% | 3,802 | 3,8025 | 0,79 | 0,391 |
| HPMC\_HP\*HPMC\_PS | 1 | 0,143 | 0,08% | 0,143 | 0,1432 | 0,03 | 0,866 |
| Error | 12 | 57,633 | 30,26% | 57,633 | 4,8028 |  |  |
| Lack-of-Fit | 10 | 53,769 | 28,24% | 53,769 | 5,3769 | 2,78 | 0,293 |
| Pure Error | 2 | 3,864 | 2,03% | 3,864 | 1,9319 |  |  |
| Total | 26 | 190,433 | 100,00% |  |  |  |  |

## Regression Equation in Uncoded Units

|  |  |  |
| --- | --- | --- |
| F\_mean\_24h(1440min) | = | -120 + 176 Lac - 0,0109 HPMC\_Visc + 28,5 HPMC\_HP + 2,90 HPMC\_PS - 66,1 Lac\*Lac - 0,000000 HPMC\_Visc\*HPMC\_Visc - 1,42 HPMC\_HP\*HPMC\_HP - 0,0233 HPMC\_PS\*HPMC\_PS - 0,00181 Lac\*HPMC\_Visc - 7,55 Lac\*HPMC\_HP - 0,07 Lac\*HPMC\_PS + 0,000612 HPMC\_Visc\*HPMC\_HP + 0,000112 HPMC\_Visc\*HPMC\_PS - 0,092 HPMC\_HP\*HPMC\_PS |

## Fits and Diagnostics for All Observations

| Obs | F\_mean\_24h(1440min) | Fit | SE Fit | 95% CI | Resid | Std Resid | Del Resid |
| --- | --- | --- | --- | --- | --- | --- | --- |
| 1 | 95,53 | 94,53 | 1,77 | (90,66; 98,39) | 1,00 | 0,78 | 0,77 |
| 2 | 98,99 | 99,58 | 1,77 | (95,71; 103,45) | -0,59 | -0,46 | -0,44 |
| 3 | 90,80 | 91,04 | 1,87 | (86,96; 95,12) | -0,25 | -0,22 | -0,21 |
| 4 | 94,17 | 93,73 | 1,87 | (89,65; 97,81) | 0,44 | 0,39 | 0,37 |
| 5 | 92,51 | 94,59 | 1,51 | (91,29; 97,88) | -2,08 | -1,31 | -1,35 |
| 6 | 97,09 | 97,74 | 1,51 | (94,45; 101,04) | -0,65 | -0,41 | -0,40 |
| 7 | 95,86 | 95,86 | 1,82 | (91,90; 99,83) | -0,00 | -0,00 | -0,00 |
| 8 | 96,74 | 96,13 | 1,82 | (92,17; 100,10) | 0,61 | 0,50 | 0,48 |
| 9 | 91,28 | 94,62 | 1,67 | (90,98; 98,26) | -3,34 | -2,36 | -3,08 |
| 10 | 100,51 | 99,48 | 1,67 | (95,84; 103,12) | 1,03 | 0,72 | 0,71 |
| 11 | 99,12 | 96,14 | 1,43 | (93,03; 99,26) | 2,98 | 1,79 | 2,01 |
| 12 | 97,59 | 99,08 | 1,43 | (95,96; 102,19) | -1,48 | -0,89 | -0,89 |
| 13 | 93,88 | 93,43 | 1,65 | (89,83; 97,04) | 0,45 | 0,31 | 0,30 |
| 14 | 94,32 | 96,21 | 1,65 | (92,60; 99,81) | -1,88 | -1,31 | -1,35 |
| 15 | 94,86 | 97,39 | 1,44 | (94,25; 100,53) | -2,54 | -1,54 | -1,64 |
| 16 | 99,27 | 98,91 | 1,44 | (95,77; 102,06) | 0,36 | 0,22 | 0,21 |
| 17 | 92,44 | 91,30 | 1,69 | (87,61; 94,99) | 1,14 | 0,82 | 0,81 |
| 18 | 97,29 | 96,95 | 1,69 | (93,25; 100,64) | 0,34 | 0,25 | 0,24 |
| 19 | 99,95 | 96,96 | 1,40 | (93,92; 100,01) | 2,99 | 1,77 | 1,97 |
| 20 | 96,30 | 96,95 | 1,76 | (93,11; 100,79) | -0,65 | -0,50 | -0,48 |
| 21 | 97,03 | 97,31 | 1,40 | (94,26; 100,36) | -0,28 | -0,16 | -0,16 |
| 22 | 97,28 | 96,35 | 1,98 | (92,03; 100,66) | 0,93 | 1,00 | 1,00 |
| 23 | 95,41 | 95,82 | 1,99 | (91,48; 100,15) | -0,41 | -0,45 | -0,43 |
| 24 | 99,43 | 98,26 | 1,85 | (94,22; 102,29) | 1,17 | 1,00 | 1,00 |
| 25 | 96,90 | 98,26 | 1,18 | (95,69; 100,82) | -1,35 | -0,73 | -0,72 |
| 26 | 99,48 | 98,26 | 1,18 | (95,69; 100,82) | 1,22 | 0,66 | 0,64 |
| 27 | 99,10 | 98,26 | 1,18 | (95,69; 100,82) | 0,84 | 0,46 | 0,44 |

| Obs | HI | Cook’s D | DFITS |  |
| --- | --- | --- | --- | --- |
| 1 | 0,655994 | 0,08 | 1,06064 |  |
| 2 | 0,655994 | 0,03 | -0,61426 |  |
| 3 | 0,729945 | 0,01 | -0,34361 |  |
| 4 | 0,729945 | 0,03 | 0,61594 |  |
| 5 | 0,476813 | 0,10 | -1,29344 |  |
| 6 | 0,476813 | 0,01 | -0,37914 |  |
| 7 | 0,688750 | 0,00 | -0,00534 |  |
| 8 | 0,688750 | 0,04 | 0,71669 |  |
| 9 | 0,580949 | 0,51 | -3,62405 | R |
| 10 | 0,580949 | 0,05 | 0,83457 |  |
| 11 | 0,425781 | 0,16 | 1,72782 |  |
| 12 | 0,425781 | 0,04 | -0,76299 |  |
| 13 | 0,569258 | 0,01 | 0,34539 |  |
| 14 | 0,569258 | 0,15 | -1,55499 |  |
| 15 | 0,433227 | 0,12 | -1,43568 |  |
| 16 | 0,433227 | 0,00 | 0,18272 |  |
| 17 | 0,597775 | 0,07 | 0,98948 |  |
| 18 | 0,597775 | 0,01 | 0,28862 |  |
| 19 | 0,407038 | 0,14 | 1,63521 |  |
| 20 | 0,647412 | 0,03 | -0,65594 |  |
| 21 | 0,407933 | 0,00 | -0,13110 |  |
| 22 | 0,817044 | 0,30 | 2,10312 |  |
| 23 | 0,823434 | 0,06 | -0,93038 |  |
| 24 | 0,713775 | 0,17 | 1,57270 |  |
| 25 | 0,288793 | 0,01 | -0,45700 |  |
| 26 | 0,288793 | 0,01 | 0,41052 |  |
| 27 | 0,288793 | 0,01 | 0,28083 |  |

R  Large residual
