# Supplementary material for: Influence of Selected Hypromellose Functionality-Related Characteristics and Soluble/Insoluble Filler Ratio on Carvedilol Release from Matrix Tablets
Source: Pharmaceutics. 2025 Oct 21;17(10):1358. doi: 10.3390/pharmaceutics17101358 (PMC12566823; doi:10.3390/pharmaceutics17101358)
Supplement: Supplementary file 1 [file pharmaceutics-17-01358-s001.zip › Report_Mean Release Analysis_RSM(CCD)_Stepwise, Backward Elimination.htm]

# Mean Release Analysis, Response Surface Design (Central Composite Design), Stepwise Regression - Backward Elimination

## Backward Elimination of Terms

α to remove = 0,1

## Coded Coefficients

| Term | Coef | SE Coef | 95% CI | T-Value | P-Value | VIF |
| --- | --- | --- | --- | --- | --- | --- |
| Constant | 10,473 | 0,324 | (9,801; 11,144) | 32,33 | 0,000 |  |
| Lac | 2,621 | 0,586 | (1,405; 3,838) | 4,47 | 0,000 | 1,00 |
| HPMC\_Visc | 0,565 | 0,590 | (-0,658; 1,788) | 0,96 | 0,348 | 1,33 |
| HPMC\_PS | 1,846 | 0,801 | (0,185; 3,507) | 2,30 | 0,031 | 1,34 |
| HPMC\_Visc\*HPMC\_PS | 3,62 | 1,59 | (0,33; 6,92) | 2,28 | 0,033 | 1,25 |

## Model Summary

| S | R-sq | R-sq(adj) | PRESS | R-sq(pred) | AICc | BIC |
| --- | --- | --- | --- | --- | --- | --- |
| 1,43653 | 55,81% | 47,78% | 64,0538 | 37,66% | 106,85 | 110,43 |

## Analysis of Variance

| Source | DF | Seq SS | Contribution | Adj SS | Adj MS | F-Value | P-Value |
| --- | --- | --- | --- | --- | --- | --- | --- |
| Model | 4 | 57,349 | 55,81% | 57,349 | 14,337 | 6,95 | 0,001 |
| Linear | 3 | 46,626 | 45,38% | 52,207 | 17,402 | 8,43 | 0,001 |
| Lac | 1 | 41,230 | 40,13% | 41,230 | 41,230 | 19,98 | 0,000 |
| HPMC\_Visc | 1 | 0,444 | 0,43% | 1,896 | 1,896 | 0,92 | 0,348 |
| HPMC\_PS | 1 | 4,953 | 4,82% | 10,963 | 10,963 | 5,31 | 0,031 |
| 2-Way Interaction | 1 | 10,723 | 10,44% | 10,723 | 10,723 | 5,20 | 0,033 |
| HPMC\_Visc\*HPMC\_PS | 1 | 10,723 | 10,44% | 10,723 | 10,723 | 5,20 | 0,033 |
| Error | 22 | 45,400 | 44,19% | 45,400 | 2,064 |  |  |
| Lack-of-Fit | 20 | 31,370 | 30,53% | 31,370 | 1,569 | 0,22 | 0,975 |
| Pure Error | 2 | 14,029 | 13,65% | 14,029 | 7,015 |  |  |
| Total | 26 | 102,749 | 100,00% |  |  |  |  |

## Regression Equation in Uncoded Units

|  |  |  |
| --- | --- | --- |
| F\_mean\_0.17h(10min) | = | 105,0 + 10,49 Lac - 0,00868 HPMC\_Visc - 1,460 HPMC\_PS + 0,000127 HPMC\_Visc\*HPMC\_PS |

## Fits and Diagnostics for All Observations

| Obs | F\_mean\_0.17h(10min) | Fit | SE Fit | 95% CI | Resid | Std Resid | Del Resid |
| --- | --- | --- | --- | --- | --- | --- | --- |
| 1 | 9,095 | 8,941 | 0,612 | (7,672; 10,210) | 0,154 | 0,12 | 0,12 |
| 2 | 11,821 | 11,563 | 0,612 | (10,293; 12,832) | 0,259 | 0,20 | 0,19 |
| 3 | 7,323 | 7,038 | 0,816 | (5,346; 8,730) | 0,285 | 0,24 | 0,24 |
| 4 | 8,147 | 9,659 | 0,816 | (7,967; 11,352) | -1,512 | -1,28 | -1,30 |
| 5 | 7,099 | 8,882 | 0,548 | (7,746; 10,018) | -1,783 | -1,34 | -1,37 |
| 6 | 12,823 | 11,503 | 0,548 | (10,368; 12,639) | 1,319 | 0,99 | 0,99 |
| 7 | 7,484 | 7,841 | 0,652 | (6,490; 9,193) | -0,357 | -0,28 | -0,27 |
| 8 | 9,846 | 10,463 | 0,652 | (9,111; 11,814) | -0,617 | -0,48 | -0,47 |
| 9 | 8,623 | 8,496 | 0,628 | (7,192; 9,799) | 0,127 | 0,10 | 0,10 |
| 10 | 11,322 | 11,117 | 0,628 | (9,814; 12,421) | 0,205 | 0,16 | 0,15 |
| 11 | 9,398 | 9,805 | 0,586 | (8,589; 11,020) | -0,407 | -0,31 | -0,30 |
| 12 | 12,522 | 12,426 | 0,586 | (11,210; 13,642) | 0,096 | 0,07 | 0,07 |
| 13 | 7,874 | 8,405 | 0,710 | (6,933; 9,877) | -0,531 | -0,43 | -0,42 |
| 14 | 12,420 | 11,026 | 0,710 | (9,554; 12,498) | 1,394 | 1,12 | 1,12 |
| 15 | 10,497 | 10,087 | 0,678 | (8,680; 11,494) | 0,409 | 0,32 | 0,32 |
| 16 | 10,196 | 12,709 | 0,678 | (11,302; 14,115) | -2,512 | -1,98 | -2,14 |
| 17 | 8,246 | 7,705 | 0,659 | (6,338; 9,071) | 0,542 | 0,42 | 0,42 |
| 18 | 13,122 | 12,948 | 0,659 | (11,581; 14,314) | 0,174 | 0,14 | 0,13 |
| 19 | 9,647 | 9,742 | 0,564 | (8,572; 10,912) | -0,095 | -0,07 | -0,07 |
| 20 | 13,095 | 10,272 | 0,661 | (8,901; 11,643) | 2,823 | 2,21 | 2,45 |
| 21 | 10,472 | 10,246 | 0,309 | (9,604; 10,887) | 0,226 | 0,16 | 0,16 |
| 22 | 12,671 | 10,485 | 0,311 | (9,840; 11,129) | 2,186 | 1,56 | 1,61 |
| 23 | 9,297 | 9,421 | 0,835 | (7,690; 11,152) | -0,124 | -0,11 | -0,10 |
| 24 | 10,323 | 11,188 | 0,774 | (9,583; 12,793) | -0,865 | -0,71 | -0,71 |
| 25 | 8,148 | 10,326 | 0,301 | (9,703; 10,950) | -2,178 | -1,55 | -1,61 |
| 26 | 8,524 | 10,326 | 0,301 | (9,703; 10,950) | -1,803 | -1,28 | -1,30 |
| 27 | 12,912 | 10,326 | 0,301 | (9,703; 10,950) | 2,585 | 1,84 | 1,95 |

| Obs | HI | Cook’s D | DFITS |  |
| --- | --- | --- | --- | --- |
| 1 | 0,181510 | 0,00 | 0,05454 |  |
| 2 | 0,181510 | 0,00 | 0,09168 |  |
| 3 | 0,322651 | 0,01 | 0,16286 |  |
| 4 | 0,322651 | 0,16 | -0,89636 |  |
| 5 | 0,145319 | 0,06 | -0,56465 |  |
| 6 | 0,145319 | 0,03 | 0,40950 |  |
| 7 | 0,205858 | 0,00 | -0,13909 |  |
| 8 | 0,205858 | 0,01 | -0,24092 |  |
| 9 | 0,191390 | 0,00 | 0,04674 |  |
| 10 | 0,191390 | 0,00 | 0,07533 |  |
| 11 | 0,166538 | 0,00 | -0,13579 |  |
| 12 | 0,166538 | 0,00 | 0,03197 |  |
| 13 | 0,244159 | 0,01 | -0,23706 |  |
| 14 | 0,244159 | 0,08 | 0,63822 |  |
| 15 | 0,222999 | 0,01 | 0,16961 |  |
| 16 | 0,222999 | 0,23 | -1,14604 |  |
| 17 | 0,210453 | 0,01 | 0,21495 |  |
| 18 | 0,210453 | 0,00 | 0,06891 |  |
| 19 | 0,154147 | 0,00 | -0,03009 |  |
| 20 | 0,211709 | 0,26 | 1,27110 | R |
| 21 | 0,046409 | 0,00 | 0,03479 |  |
| 22 | 0,046809 | 0,02 | 0,35778 |  |
| 23 | 0,337586 | 0,00 | -0,07404 |  |
| 24 | 0,290228 | 0,04 | -0,45179 |  |
| 25 | 0,043786 | 0,02 | -0,34353 |  |
| 26 | 0,043786 | 0,02 | -0,27891 |  |
| 27 | 0,043786 | 0,03 | 0,41834 |  |

R  Large residual

## Backward Elimination of Terms

α to remove = 0,1

## Coded Coefficients

| Term | Coef | SE Coef | 95% CI | T-Value | P-Value | VIF |
| --- | --- | --- | --- | --- | --- | --- |
| Constant | 14,260 | 0,374 | (13,482; 15,038) | 38,13 | 0,000 |  |
| Lac | 2,999 | 0,703 | (1,538; 4,460) | 4,27 | 0,000 | 1,08 |
| HPMC\_Visc | 0,619 | 0,681 | (-0,797; 2,035) | 0,91 | 0,373 | 1,33 |
| HPMC\_PS | 2,530 | 0,925 | (0,606; 4,454) | 2,74 | 0,012 | 1,34 |
| Lac\*HPMC\_Visc | -2,39 | 1,37 | (-5,24; 0,47) | -1,74 | 0,097 | 1,08 |
| HPMC\_Visc\*HPMC\_PS | 4,31 | 1,84 | (0,49; 8,13) | 2,35 | 0,029 | 1,25 |

## Model Summary

| S | R-sq | R-sq(adj) | PRESS | R-sq(pred) | AICc | BIC |
| --- | --- | --- | --- | --- | --- | --- |
| 1,65892 | 63,84% | 55,23% | 84,2977 | 47,25% | 117,06 | 120,24 |

## Analysis of Variance

| Source | DF | Seq SS | Contribution | Adj SS | Adj MS | F-Value | P-Value |
| --- | --- | --- | --- | --- | --- | --- | --- |
| Model | 5 | 102,020 | 63,84% | 102,020 | 20,404 | 7,41 | 0,000 |
| Linear | 3 | 78,540 | 49,15% | 71,063 | 23,688 | 8,61 | 0,001 |
| Lac | 1 | 66,271 | 41,47% | 50,135 | 50,135 | 18,22 | 0,000 |
| HPMC\_Visc | 1 | 1,411 | 0,88% | 2,277 | 2,277 | 0,83 | 0,373 |
| HPMC\_PS | 1 | 10,858 | 6,79% | 20,587 | 20,587 | 7,48 | 0,012 |
| 2-Way Interaction | 2 | 23,480 | 14,69% | 23,480 | 11,740 | 4,27 | 0,028 |
| Lac\*HPMC\_Visc | 1 | 8,300 | 5,19% | 8,300 | 8,300 | 3,02 | 0,097 |
| HPMC\_Visc\*HPMC\_PS | 1 | 15,179 | 9,50% | 15,179 | 15,179 | 5,52 | 0,029 |
| Error | 21 | 57,792 | 36,16% | 57,792 | 2,752 |  |  |
| Lack-of-Fit | 19 | 40,167 | 25,13% | 40,167 | 2,114 | 0,24 | 0,968 |
| Pure Error | 2 | 17,625 | 11,03% | 17,625 | 8,812 |  |  |
| Total | 26 | 159,812 | 100,00% |  |  |  |  |

## Regression Equation in Uncoded Units

|  |  |  |
| --- | --- | --- |
| F\_mean\_0.33h(20min) | = | 107,4 + 45,2 Lac - 0,00911 HPMC\_Visc - 1,692 HPMC\_PS - 0,00245 Lac\*HPMC\_Visc + 0,000151 HPMC\_Visc\*HPMC\_PS |

## Fits and Diagnostics for All Observations

| Obs | F\_mean\_0.33h(20min) | Fit | SE Fit | 95% CI | Resid | Std Resid | Del Resid |
| --- | --- | --- | --- | --- | --- | --- | --- |
| 1 | 12,421 | 11,604 | 0,810 | (9,919; 13,288) | 0,817 | 0,56 | 0,56 |
| 2 | 16,290 | 16,302 | 0,810 | (14,617; 17,986) | -0,012 | -0,01 | -0,01 |
| 3 | 10,020 | 10,827 | 1,100 | (8,540; 13,114) | -0,807 | -0,65 | -0,64 |
| 4 | 11,393 | 12,180 | 1,100 | (9,893; 14,468) | -0,788 | -0,63 | -0,63 |
| 5 | 10,071 | 11,630 | 0,724 | (10,124; 13,136) | -1,559 | -1,04 | -1,05 |
| 6 | 17,069 | 16,179 | 0,724 | (14,673; 17,684) | 0,891 | 0,60 | 0,59 |
| 7 | 10,510 | 11,870 | 0,951 | (9,892; 13,848) | -1,359 | -1,00 | -1,00 |
| 8 | 13,414 | 13,173 | 0,951 | (11,195; 15,151) | 0,241 | 0,18 | 0,17 |
| 9 | 12,069 | 11,317 | 0,820 | (9,611; 13,023) | 0,752 | 0,52 | 0,51 |
| 10 | 15,416 | 15,968 | 0,820 | (14,262; 17,674) | -0,552 | -0,38 | -0,37 |
| 11 | 12,968 | 13,813 | 0,713 | (12,330; 15,296) | -0,845 | -0,56 | -0,55 |
| 12 | 16,492 | 16,357 | 0,713 | (14,873; 17,840) | 0,135 | 0,09 | 0,09 |
| 13 | 11,347 | 11,234 | 0,907 | (9,348; 13,120) | 0,113 | 0,08 | 0,08 |
| 14 | 16,913 | 15,906 | 0,907 | (14,020; 17,792) | 1,007 | 0,72 | 0,72 |
| 15 | 14,391 | 14,273 | 0,834 | (12,537; 16,008) | 0,118 | 0,08 | 0,08 |
| 16 | 13,890 | 16,598 | 0,834 | (14,862; 18,333) | -2,708 | -1,89 | -2,02 |
| 17 | 11,041 | 10,638 | 0,764 | (9,048; 12,227) | 0,403 | 0,27 | 0,27 |
| 18 | 17,442 | 17,531 | 0,764 | (15,941; 19,120) | -0,089 | -0,06 | -0,06 |
| 19 | 13,390 | 13,475 | 0,651 | (12,120; 14,829) | -0,084 | -0,06 | -0,05 |
| 20 | 17,586 | 13,921 | 0,763 | (12,334; 15,509) | 3,665 | 2,49 | 2,89 |
| 21 | 14,091 | 13,964 | 0,357 | (13,221; 14,708) | 0,127 | 0,08 | 0,08 |
| 22 | 17,139 | 14,299 | 0,359 | (13,553; 15,045) | 2,840 | 1,75 | 1,85 |
| 23 | 12,766 | 12,689 | 0,964 | (10,685; 14,693) | 0,077 | 0,06 | 0,06 |
| 24 | 14,769 | 15,459 | 0,894 | (13,600; 17,317) | -0,690 | -0,49 | -0,48 |
| 25 | 11,694 | 14,084 | 0,347 | (13,362; 14,806) | -2,391 | -1,47 | -1,52 |
| 26 | 11,921 | 14,084 | 0,347 | (13,362; 14,806) | -2,163 | -1,33 | -1,36 |
| 27 | 16,945 | 14,084 | 0,347 | (13,362; 14,806) | 2,861 | 1,76 | 1,86 |

| Obs | HI | Cook’s D | DFITS |  |
| --- | --- | --- | --- | --- |
| 1 | 0,238422 | 0,02 | 0,31055 |  |
| 2 | 0,238422 | 0,00 | -0,00455 |  |
| 3 | 0,439577 | 0,06 | -0,56744 |  |
| 4 | 0,439577 | 0,05 | -0,55353 |  |
| 5 | 0,190525 | 0,04 | -0,50789 |  |
| 6 | 0,190525 | 0,01 | 0,28492 |  |
| 7 | 0,328781 | 0,08 | -0,70006 |  |
| 8 | 0,328781 | 0,00 | 0,12124 |  |
| 9 | 0,244482 | 0,01 | 0,29137 |  |
| 10 | 0,244482 | 0,01 | -0,21319 |  |
| 11 | 0,184860 | 0,01 | -0,26406 |  |
| 12 | 0,184860 | 0,00 | 0,04198 |  |
| 13 | 0,298927 | 0,00 | 0,05175 |  |
| 14 | 0,298927 | 0,04 | 0,46780 |  |
| 15 | 0,253021 | 0,00 | 0,04684 |  |
| 16 | 0,253021 | 0,20 | -1,17721 |  |
| 17 | 0,212283 | 0,00 | 0,13893 |  |
| 18 | 0,212283 | 0,00 | -0,03071 |  |
| 19 | 0,154147 | 0,00 | -0,02303 |  |
| 20 | 0,211709 | 0,28 | 1,49850 | R |
| 21 | 0,046409 | 0,00 | 0,01686 |  |
| 22 | 0,046809 | 0,03 | 0,41041 |  |
| 23 | 0,337586 | 0,00 | 0,03963 |  |
| 24 | 0,290228 | 0,02 | -0,30970 |  |
| 25 | 0,043786 | 0,02 | -0,32502 |  |
| 26 | 0,043786 | 0,01 | -0,29105 |  |
| 27 | 0,043786 | 0,02 | 0,39906 |  |

R  Large residual

## Backward Elimination of Terms

α to remove = 0,1

## Coded Coefficients

| Term | Coef | SE Coef | 95% CI | T-Value | P-Value | VIF |
| --- | --- | --- | --- | --- | --- | --- |
| Constant | 16,670 | 0,398 | (15,842; 17,499) | 41,86 | 0,000 |  |
| Lac | 3,388 | 0,748 | (1,832; 4,943) | 4,53 | 0,000 | 1,08 |
| HPMC\_Visc | 0,609 | 0,725 | (-0,898; 2,117) | 0,84 | 0,410 | 1,33 |
| HPMC\_PS | 2,769 | 0,985 | (0,721; 4,817) | 2,81 | 0,010 | 1,34 |
| Lac\*HPMC\_Visc | -2,59 | 1,46 | (-5,63; 0,45) | -1,77 | 0,091 | 1,08 |
| HPMC\_Visc\*HPMC\_PS | 4,48 | 1,95 | (0,41; 8,54) | 2,29 | 0,033 | 1,25 |

## Model Summary

| S | R-sq | R-sq(adj) | PRESS | R-sq(pred) | AICc | BIC |
| --- | --- | --- | --- | --- | --- | --- |
| 1,76615 | 65,75% | 57,60% | 95,6997 | 49,97% | 120,45 | 123,62 |

## Analysis of Variance

| Source | DF | Seq SS | Contribution | Adj SS | Adj MS | F-Value | P-Value |
| --- | --- | --- | --- | --- | --- | --- | --- |
| Model | 5 | 125,774 | 65,75% | 125,774 | 25,155 | 8,06 | 0,000 |
| Linear | 3 | 99,647 | 52,10% | 89,317 | 29,772 | 9,54 | 0,000 |
| Lac | 1 | 83,920 | 43,87% | 63,976 | 63,976 | 20,51 | 0,000 |
| HPMC\_Visc | 1 | 2,053 | 1,07% | 2,204 | 2,204 | 0,71 | 0,410 |
| HPMC\_PS | 1 | 13,675 | 7,15% | 24,660 | 24,660 | 7,91 | 0,010 |
| 2-Way Interaction | 2 | 26,127 | 13,66% | 26,127 | 13,063 | 4,19 | 0,029 |
| Lac\*HPMC\_Visc | 1 | 9,777 | 5,11% | 9,777 | 9,777 | 3,13 | 0,091 |
| HPMC\_Visc\*HPMC\_PS | 1 | 16,350 | 8,55% | 16,350 | 16,350 | 5,24 | 0,033 |
| Error | 21 | 65,505 | 34,25% | 65,505 | 3,119 |  |  |
| Lack-of-Fit | 19 | 46,304 | 24,21% | 46,304 | 2,437 | 0,25 | 0,963 |
| Pure Error | 2 | 19,201 | 10,04% | 19,201 | 9,600 |  |  |
| Total | 26 | 191,280 | 100,00% |  |  |  |  |

## Regression Equation in Uncoded Units

|  |  |  |
| --- | --- | --- |
| F\_mean\_0.5h(30min) | = | 110,8 + 49,5 Lac - 0,00941 HPMC\_Visc - 1,737 HPMC\_PS - 0,00266 Lac\*HPMC\_Visc + 0,000156 HPMC\_Visc\*HPMC\_PS |

## Fits and Diagnostics for All Observations

| Obs | F\_mean\_0.5h(30min) | Fit | SE Fit | 95% CI | Resid | Std Resid | Del Resid |
| --- | --- | --- | --- | --- | --- | --- | --- |
| 1 | 14,629 | 13,724 | 0,862 | (11,931; 15,517) | 0,905 | 0,59 | 0,58 |
| 2 | 18,932 | 18,956 | 0,862 | (17,162; 20,749) | -0,024 | -0,02 | -0,01 |
| 3 | 11,941 | 12,903 | 1,171 | (10,468; 15,338) | -0,962 | -0,73 | -0,72 |
| 4 | 13,736 | 14,504 | 1,171 | (12,069; 16,940) | -0,768 | -0,58 | -0,57 |
| 5 | 12,093 | 13,764 | 0,771 | (12,160; 15,367) | -1,671 | -1,05 | -1,05 |
| 6 | 19,640 | 18,833 | 0,771 | (17,230; 20,436) | 0,807 | 0,51 | 0,50 |
| 7 | 12,627 | 14,013 | 1,013 | (11,907; 16,119) | -1,385 | -0,96 | -0,96 |
| 8 | 15,806 | 15,560 | 1,013 | (13,454; 17,666) | 0,246 | 0,17 | 0,17 |
| 9 | 14,264 | 13,522 | 0,873 | (11,706; 15,338) | 0,742 | 0,48 | 0,47 |
| 10 | 17,960 | 18,702 | 0,873 | (16,886; 20,519) | -0,743 | -0,48 | -0,47 |
| 11 | 15,264 | 16,103 | 0,759 | (14,524; 17,683) | -0,840 | -0,53 | -0,52 |
| 12 | 18,986 | 18,997 | 0,759 | (17,418; 20,576) | -0,011 | -0,01 | -0,01 |
| 13 | 13,544 | 13,450 | 0,966 | (11,442; 15,458) | 0,094 | 0,06 | 0,06 |
| 14 | 19,878 | 18,654 | 0,966 | (16,645; 20,662) | 1,225 | 0,83 | 0,82 |
| 15 | 16,784 | 16,590 | 0,888 | (14,743; 18,438) | 0,193 | 0,13 | 0,12 |
| 16 | 16,332 | 19,247 | 0,888 | (17,399; 21,094) | -2,914 | -1,91 | -2,05 |
| 17 | 12,909 | 12,616 | 0,814 | (10,923; 14,308) | 0,293 | 0,19 | 0,18 |
| 18 | 20,286 | 20,363 | 0,814 | (18,671; 22,055) | -0,077 | -0,05 | -0,05 |
| 19 | 15,883 | 15,902 | 0,693 | (14,460; 17,344) | -0,019 | -0,01 | -0,01 |
| 20 | 20,126 | 16,265 | 0,813 | (14,575; 17,955) | 3,861 | 2,46 | 2,85 |
| 21 | 16,410 | 16,355 | 0,380 | (15,563; 17,146) | 0,055 | 0,03 | 0,03 |
| 22 | 19,930 | 16,722 | 0,382 | (15,928; 17,517) | 3,208 | 1,86 | 1,99 |
| 23 | 15,034 | 14,905 | 1,026 | (12,771; 17,039) | 0,129 | 0,09 | 0,09 |
| 24 | 17,365 | 18,067 | 0,951 | (16,088; 20,046) | -0,702 | -0,47 | -0,46 |
| 25 | 14,088 | 16,489 | 0,370 | (15,721; 17,258) | -2,401 | -1,39 | -1,42 |
| 26 | 14,218 | 16,489 | 0,370 | (15,721; 17,258) | -2,271 | -1,31 | -1,34 |
| 27 | 19,519 | 16,489 | 0,370 | (15,721; 17,258) | 3,030 | 1,75 | 1,85 |

| Obs | HI | Cook’s D | DFITS |  |
| --- | --- | --- | --- | --- |
| 1 | 0,238422 | 0,02 | 0,32323 |  |
| 2 | 0,238422 | 0,00 | -0,00836 |  |
| 3 | 0,439577 | 0,07 | -0,63686 |  |
| 4 | 0,439577 | 0,04 | -0,50606 |  |
| 5 | 0,190525 | 0,04 | -0,51138 |  |
| 6 | 0,190525 | 0,01 | 0,24190 |  |
| 7 | 0,328781 | 0,07 | -0,66857 |  |
| 8 | 0,328781 | 0,00 | 0,11609 |  |
| 9 | 0,244482 | 0,01 | 0,27001 |  |
| 10 | 0,244482 | 0,01 | -0,27008 |  |
| 11 | 0,184860 | 0,01 | -0,24641 |  |
| 12 | 0,184860 | 0,00 | -0,00320 |  |
| 13 | 0,298927 | 0,00 | 0,04038 |  |
| 14 | 0,298927 | 0,05 | 0,53652 |  |
| 15 | 0,253021 | 0,00 | 0,07198 |  |
| 16 | 0,253021 | 0,21 | -1,19289 |  |
| 17 | 0,212283 | 0,00 | 0,09477 |  |
| 18 | 0,212283 | 0,00 | -0,02498 |  |
| 19 | 0,154147 | 0,00 | -0,00493 |  |
| 20 | 0,211709 | 0,27 | 1,47650 | R |
| 21 | 0,046409 | 0,00 | 0,00690 |  |
| 22 | 0,046809 | 0,03 | 0,44025 |  |
| 23 | 0,337586 | 0,00 | 0,06230 |  |
| 24 | 0,290228 | 0,02 | -0,29592 |  |
| 25 | 0,043786 | 0,01 | -0,30465 |  |
| 26 | 0,043786 | 0,01 | -0,28666 |  |
| 27 | 0,043786 | 0,02 | 0,39654 |  |

R  Large residual

## Backward Elimination of Terms

α to remove = 0,1

## Coded Coefficients

| Term | Coef | SE Coef | 95% CI | T-Value | P-Value | VIF |
| --- | --- | --- | --- | --- | --- | --- |
| Constant | 19,559 | 0,425 | (18,675; 20,444) | 46,00 | 0,000 |  |
| Lac | 3,801 | 0,799 | (2,140; 5,462) | 4,76 | 0,000 | 1,08 |
| HPMC\_Visc | 0,526 | 0,774 | (-1,084; 2,135) | 0,68 | 0,505 | 1,33 |
| HPMC\_PS | 2,99 | 1,05 | (0,80; 5,17) | 2,84 | 0,010 | 1,34 |
| Lac\*HPMC\_Visc | -2,79 | 1,56 | (-6,03; 0,46) | -1,78 | 0,089 | 1,08 |
| HPMC\_Visc\*HPMC\_PS | 4,66 | 2,09 | (0,32; 9,00) | 2,23 | 0,037 | 1,25 |

## Model Summary

| S | R-sq | R-sq(adj) | PRESS | R-sq(pred) | AICc | BIC |
| --- | --- | --- | --- | --- | --- | --- |
| 1,88590 | 67,31% | 59,52% | 109,417 | 52,11% | 123,99 | 127,17 |

## Analysis of Variance

| Source | DF | Seq SS | Contribution | Adj SS | Adj MS | F-Value | P-Value |
| --- | --- | --- | --- | --- | --- | --- | --- |
| Model | 5 | 153,770 | 67,31% | 153,770 | 30,754 | 8,65 | 0,000 |
| Linear | 3 | 124,736 | 54,60% | 110,782 | 36,927 | 10,38 | 0,000 |
| Lac | 1 | 104,807 | 45,88% | 80,529 | 80,529 | 22,64 | 0,000 |
| HPMC\_Visc | 1 | 3,514 | 1,54% | 1,639 | 1,639 | 0,46 | 0,505 |
| HPMC\_PS | 1 | 16,415 | 7,19% | 28,692 | 28,692 | 8,07 | 0,010 |
| 2-Way Interaction | 2 | 29,034 | 12,71% | 29,034 | 14,517 | 4,08 | 0,032 |
| Lac\*HPMC\_Visc | 1 | 11,306 | 4,95% | 11,306 | 11,306 | 3,18 | 0,089 |
| HPMC\_Visc\*HPMC\_PS | 1 | 17,728 | 7,76% | 17,728 | 17,728 | 4,98 | 0,037 |
| Error | 21 | 74,689 | 32,69% | 74,689 | 3,557 |  |  |
| Lack-of-Fit | 19 | 53,818 | 23,56% | 53,818 | 2,833 | 0,27 | 0,956 |
| Pure Error | 2 | 20,871 | 9,14% | 20,871 | 10,436 |  |  |
| Total | 26 | 228,459 | 100,00% |  |  |  |  |

## Regression Equation in Uncoded Units

|  |  |  |
| --- | --- | --- |
| F\_mean\_0.75h(45min) | = | 115,8 + 53,9 Lac - 0,00978 HPMC\_Visc - 1,794 HPMC\_PS - 0,00286 Lac\*HPMC\_Visc + 0,000163 HPMC\_Visc\*HPMC\_PS |

## Fits and Diagnostics for All Observations

| Obs | F\_mean\_0.75h(45min) | Fit | SE Fit | 95% CI | Resid | Std Resid | Del Resid |
| --- | --- | --- | --- | --- | --- | --- | --- |
| 1 | 17,369 | 16,375 | 0,921 | (14,460; 18,290) | 0,993 | 0,60 | 0,59 |
| 2 | 21,994 | 22,159 | 0,921 | (20,244; 24,074) | -0,165 | -0,10 | -0,10 |
| 3 | 14,259 | 15,395 | 1,250 | (12,795; 17,996) | -1,136 | -0,80 | -0,80 |
| 4 | 16,501 | 17,275 | 1,250 | (14,675; 19,875) | -0,775 | -0,55 | -0,54 |
| 5 | 14,687 | 16,420 | 0,823 | (14,708; 18,132) | -1,733 | -1,02 | -1,02 |
| 6 | 22,707 | 22,030 | 0,823 | (20,318; 23,742) | 0,678 | 0,40 | 0,39 |
| 7 | 15,190 | 16,569 | 1,081 | (14,320; 18,818) | -1,379 | -0,89 | -0,89 |
| 8 | 18,616 | 18,391 | 1,081 | (16,142; 20,639) | 0,225 | 0,15 | 0,14 |
| 9 | 16,906 | 16,232 | 0,932 | (14,293; 18,171) | 0,674 | 0,41 | 0,40 |
| 10 | 21,098 | 21,961 | 0,932 | (20,022; 23,900) | -0,863 | -0,53 | -0,52 |
| 11 | 18,055 | 18,842 | 0,811 | (17,156; 20,528) | -0,787 | -0,46 | -0,45 |
| 12 | 22,100 | 22,111 | 0,811 | (20,425; 23,797) | -0,011 | -0,01 | -0,01 |
| 13 | 16,264 | 16,169 | 1,031 | (14,025; 18,313) | 0,095 | 0,06 | 0,06 |
| 14 | 23,411 | 21,922 | 1,031 | (19,778; 24,066) | 1,489 | 0,94 | 0,94 |
| 15 | 19,597 | 19,349 | 0,949 | (17,376; 21,322) | 0,248 | 0,15 | 0,15 |
| 16 | 19,194 | 22,363 | 0,949 | (20,390; 24,336) | -3,169 | -1,94 | -2,10 |
| 17 | 15,271 | 15,064 | 0,869 | (13,257; 16,871) | 0,206 | 0,12 | 0,12 |
| 18 | 23,700 | 23,711 | 0,869 | (21,904; 25,518) | -0,011 | -0,01 | -0,01 |
| 19 | 18,969 | 18,878 | 0,740 | (17,338; 20,418) | 0,091 | 0,05 | 0,05 |
| 20 | 23,059 | 19,014 | 0,868 | (17,210; 20,819) | 4,045 | 2,42 | 2,77 |
| 21 | 19,298 | 19,239 | 0,406 | (18,395; 20,084) | 0,059 | 0,03 | 0,03 |
| 22 | 23,265 | 19,632 | 0,408 | (18,783; 20,480) | 3,634 | 1,97 | 2,13 |
| 23 | 17,796 | 17,646 | 1,096 | (15,368; 19,925) | 0,150 | 0,10 | 0,10 |
| 24 | 20,282 | 21,146 | 1,016 | (19,034; 23,259) | -0,864 | -0,54 | -0,53 |
| 25 | 16,879 | 19,388 | 0,395 | (18,567; 20,208) | -2,509 | -1,36 | -1,39 |
| 26 | 17,038 | 19,388 | 0,395 | (18,567; 20,208) | -2,350 | -1,27 | -1,29 |
| 27 | 22,552 | 19,388 | 0,395 | (18,567; 20,208) | 3,164 | 1,72 | 1,81 |

| Obs | HI | Cook’s D | DFITS |  |
| --- | --- | --- | --- | --- |
| 1 | 0,238422 | 0,02 | 0,33246 |  |
| 2 | 0,238422 | 0,00 | -0,05483 |  |
| 3 | 0,439577 | 0,08 | -0,70661 |  |
| 4 | 0,439577 | 0,04 | -0,47760 |  |
| 5 | 0,190525 | 0,04 | -0,49605 |  |
| 6 | 0,190525 | 0,01 | 0,18988 |  |
| 7 | 0,328781 | 0,07 | -0,62152 |  |
| 8 | 0,328781 | 0,00 | 0,09959 |  |
| 9 | 0,244482 | 0,01 | 0,22911 |  |
| 10 | 0,244482 | 0,01 | -0,29410 |  |
| 11 | 0,184860 | 0,01 | -0,21598 |  |
| 12 | 0,184860 | 0,00 | -0,00298 |  |
| 13 | 0,298927 | 0,00 | 0,03832 |  |
| 14 | 0,298927 | 0,06 | 0,61413 |  |
| 15 | 0,253021 | 0,00 | 0,08634 |  |
| 16 | 0,253021 | 0,21 | -1,21965 |  |
| 17 | 0,212283 | 0,00 | 0,06252 |  |
| 18 | 0,212283 | 0,00 | -0,00339 |  |
| 19 | 0,154147 | 0,00 | 0,02195 |  |
| 20 | 0,211709 | 0,26 | 1,43780 | R |
| 21 | 0,046409 | 0,00 | 0,00687 |  |
| 22 | 0,046809 | 0,03 | 0,47289 |  |
| 23 | 0,337586 | 0,00 | 0,06816 |  |
| 24 | 0,290228 | 0,02 | -0,34176 |  |
| 25 | 0,043786 | 0,01 | -0,29747 |  |
| 26 | 0,043786 | 0,01 | -0,27702 |  |
| 27 | 0,043786 | 0,02 | 0,38645 |  |

R  Large residual

## Backward Elimination of Terms

α to remove = 0,1

## Coded Coefficients

| Term | Coef | SE Coef | 95% CI | T-Value | P-Value | VIF |
| --- | --- | --- | --- | --- | --- | --- |
| Constant | 22,036 | 0,443 | (21,115; 22,957) | 49,76 | 0,000 |  |
| Lac | 4,135 | 0,832 | (2,405; 5,864) | 4,97 | 0,000 | 1,08 |
| HPMC\_Visc | 0,412 | 0,806 | (-1,265; 2,088) | 0,51 | 0,615 | 1,33 |
| HPMC\_PS | 3,06 | 1,10 | (0,79; 5,34) | 2,80 | 0,011 | 1,34 |
| Lac\*HPMC\_Visc | -2,99 | 1,63 | (-6,37; 0,39) | -1,84 | 0,080 | 1,08 |
| HPMC\_Visc\*HPMC\_PS | 4,74 | 2,17 | (0,22; 9,26) | 2,18 | 0,041 | 1,25 |

## Model Summary

| S | R-sq | R-sq(adj) | PRESS | R-sq(pred) | AICc | BIC |
| --- | --- | --- | --- | --- | --- | --- |
| 1,96416 | 68,68% | 61,22% | 119,102 | 53,95% | 126,19 | 129,36 |

## Analysis of Variance

| Source | DF | Seq SS | Contribution | Adj SS | Adj MS | F-Value | P-Value |
| --- | --- | --- | --- | --- | --- | --- | --- |
| Model | 5 | 177,634 | 68,68% | 177,634 | 35,527 | 9,21 | 0,000 |
| Linear | 3 | 146,282 | 56,56% | 128,141 | 42,714 | 11,07 | 0,000 |
| Lac | 1 | 123,731 | 47,84% | 95,303 | 95,303 | 24,70 | 0,000 |
| HPMC\_Visc | 1 | 5,152 | 1,99% | 1,007 | 1,007 | 0,26 | 0,615 |
| HPMC\_PS | 1 | 17,400 | 6,73% | 30,188 | 30,188 | 7,83 | 0,011 |
| 2-Way Interaction | 2 | 31,352 | 12,12% | 31,352 | 15,676 | 4,06 | 0,032 |
| Lac\*HPMC\_Visc | 1 | 13,019 | 5,03% | 13,019 | 13,019 | 3,37 | 0,080 |
| HPMC\_Visc\*HPMC\_PS | 1 | 18,333 | 7,09% | 18,333 | 18,333 | 4,75 | 0,041 |
| Error | 21 | 81,016 | 31,32% | 81,016 | 3,858 |  |  |
| Lack-of-Fit | 19 | 59,720 | 23,09% | 59,720 | 3,143 | 0,30 | 0,945 |
| Pure Error | 2 | 21,296 | 8,23% | 21,296 | 10,648 |  |  |
| Total | 26 | 258,650 | 100,00% |  |  |  |  |

## Regression Equation in Uncoded Units

|  |  |  |
| --- | --- | --- |
| F\_mean\_1h(60min) | = | 118,4 + 58,1 Lac - 0,00989 HPMC\_Visc - 1,821 HPMC\_PS - 0,00307 Lac\*HPMC\_Visc + 0,000166 HPMC\_Visc\*HPMC\_PS |

## Fits and Diagnostics for All Observations

| Obs | F\_mean\_1h(60min) | Fit | SE Fit | 95% CI | Resid | Std Resid | Del Resid |
| --- | --- | --- | --- | --- | --- | --- | --- |
| 1 | 19,761 | 18,688 | 0,959 | (16,694; 20,683) | 1,073 | 0,63 | 0,62 |
| 2 | 24,653 | 24,951 | 0,959 | (22,956; 26,945) | -0,297 | -0,17 | -0,17 |
| 3 | 16,350 | 17,621 | 1,302 | (14,912; 20,329) | -1,270 | -0,86 | -0,86 |
| 4 | 18,938 | 19,694 | 1,302 | (16,986; 22,402) | -0,756 | -0,51 | -0,51 |
| 5 | 16,856 | 18,733 | 0,857 | (16,950; 20,516) | -1,877 | -1,06 | -1,07 |
| 6 | 25,374 | 24,808 | 0,857 | (23,026; 26,591) | 0,565 | 0,32 | 0,31 |
| 7 | 17,428 | 18,818 | 1,126 | (16,476; 21,160) | -1,390 | -0,86 | -0,86 |
| 8 | 21,048 | 20,829 | 1,126 | (18,487; 23,171) | 0,219 | 0,14 | 0,13 |
| 9 | 19,146 | 18,559 | 0,971 | (16,539; 20,578) | 0,587 | 0,34 | 0,34 |
| 10 | 23,860 | 24,763 | 0,971 | (22,743; 26,782) | -0,903 | -0,53 | -0,52 |
| 11 | 20,444 | 21,168 | 0,844 | (19,412; 22,925) | -0,724 | -0,41 | -0,40 |
| 12 | 24,688 | 24,733 | 0,844 | (22,976; 26,489) | -0,045 | -0,03 | -0,02 |
| 13 | 18,608 | 18,498 | 1,074 | (16,265; 20,731) | 0,110 | 0,07 | 0,07 |
| 14 | 26,317 | 24,728 | 1,074 | (22,495; 26,961) | 1,589 | 0,97 | 0,96 |
| 15 | 21,982 | 21,681 | 0,988 | (19,627; 23,736) | 0,301 | 0,18 | 0,17 |
| 16 | 21,578 | 24,972 | 0,988 | (22,918; 27,027) | -3,394 | -2,00 | -2,17 |
| 17 | 17,356 | 17,188 | 0,905 | (15,306; 19,070) | 0,168 | 0,10 | 0,09 |
| 18 | 26,662 | 26,579 | 0,905 | (24,697; 28,461) | 0,083 | 0,05 | 0,05 |
| 19 | 21,678 | 21,468 | 0,771 | (19,864; 23,072) | 0,211 | 0,12 | 0,11 |
| 20 | 25,490 | 21,355 | 0,904 | (19,476; 23,235) | 4,135 | 2,37 | 2,70 |
| 21 | 21,834 | 21,730 | 0,423 | (20,850; 22,610) | 0,105 | 0,05 | 0,05 |
| 22 | 26,073 | 22,125 | 0,425 | (21,241; 23,009) | 3,948 | 2,06 | 2,25 |
| 23 | 20,282 | 20,096 | 1,141 | (17,722; 22,469) | 0,186 | 0,12 | 0,11 |
| 24 | 22,774 | 23,709 | 1,058 | (21,509; 25,910) | -0,935 | -0,57 | -0,56 |
| 25 | 19,368 | 21,883 | 0,411 | (21,028; 22,738) | -2,515 | -1,31 | -1,33 |
| 26 | 19,506 | 21,883 | 0,411 | (21,028; 22,738) | -2,377 | -1,24 | -1,25 |
| 27 | 25,088 | 21,883 | 0,411 | (21,028; 22,738) | 3,204 | 1,67 | 1,75 |

| Obs | HI | Cook’s D | DFITS |  |
| --- | --- | --- | --- | --- |
| 1 | 0,238422 | 0,02 | 0,34512 |  |
| 2 | 0,238422 | 0,00 | -0,09474 |  |
| 3 | 0,439577 | 0,10 | -0,76023 |  |
| 4 | 0,439577 | 0,03 | -0,44748 |  |
| 5 | 0,190525 | 0,04 | -0,51709 |  |
| 6 | 0,190525 | 0,00 | 0,15186 |  |
| 7 | 0,328781 | 0,06 | -0,60088 |  |
| 8 | 0,328781 | 0,00 | 0,09306 |  |
| 9 | 0,244482 | 0,01 | 0,19146 |  |
| 10 | 0,244482 | 0,02 | -0,29561 |  |
| 11 | 0,184860 | 0,01 | -0,19055 |  |
| 12 | 0,184860 | 0,00 | -0,01175 |  |
| 13 | 0,298927 | 0,00 | 0,04267 |  |
| 14 | 0,298927 | 0,07 | 0,63003 |  |
| 15 | 0,253021 | 0,00 | 0,10074 |  |
| 16 | 0,253021 | 0,23 | -1,26205 |  |
| 17 | 0,212283 | 0,00 | 0,04883 |  |
| 18 | 0,212283 | 0,00 | 0,02425 |  |
| 19 | 0,154147 | 0,00 | 0,04857 |  |
| 20 | 0,211709 | 0,25 | 1,40121 | R |
| 21 | 0,046409 | 0,00 | 0,01176 |  |
| 22 | 0,046809 | 0,03 | 0,49840 | R |
| 23 | 0,337586 | 0,00 | 0,08100 |  |
| 24 | 0,290228 | 0,02 | -0,35547 |  |
| 25 | 0,043786 | 0,01 | -0,28539 |  |
| 26 | 0,043786 | 0,01 | -0,26840 |  |
| 27 | 0,043786 | 0,02 | 0,37409 |  |

R  Large residual

## Backward Elimination of Terms

α to remove = 0,1

## Coded Coefficients

| Term | Coef | SE Coef | 95% CI | T-Value | P-Value | VIF |
| --- | --- | --- | --- | --- | --- | --- |
| Constant | 26,347 | 0,467 | (25,376; 27,319) | 56,40 | 0,000 |  |
| Lac | 4,668 | 0,877 | (2,843; 6,492) | 5,32 | 0,000 | 1,08 |
| HPMC\_Visc | 0,212 | 0,850 | (-1,557; 1,980) | 0,25 | 0,806 | 1,33 |
| HPMC\_PS | 3,18 | 1,16 | (0,78; 5,59) | 2,76 | 0,012 | 1,34 |
| Lac\*HPMC\_Visc | -3,20 | 1,72 | (-6,77; 0,36) | -1,87 | 0,076 | 1,08 |
| HPMC\_Visc\*HPMC\_PS | 4,87 | 2,29 | (0,10; 9,64) | 2,12 | 0,046 | 1,25 |

## Model Summary

| S | R-sq | R-sq(adj) | PRESS | R-sq(pred) | AICc | BIC |
| --- | --- | --- | --- | --- | --- | --- |
| 2,07186 | 70,77% | 63,81% | 132,514 | 57,03% | 129,07 | 132,24 |

## Analysis of Variance

| Source | DF | Seq SS | Contribution | Adj SS | Adj MS | F-Value | P-Value |
| --- | --- | --- | --- | --- | --- | --- | --- |
| Model | 5 | 218,275 | 70,77% | 218,275 | 43,655 | 10,17 | 0,000 |
| Linear | 3 | 183,973 | 59,65% | 159,270 | 53,090 | 12,37 | 0,000 |
| Lac | 1 | 156,277 | 50,67% | 121,463 | 121,463 | 28,30 | 0,000 |
| HPMC\_Visc | 1 | 8,715 | 2,83% | 0,266 | 0,266 | 0,06 | 0,806 |
| HPMC\_PS | 1 | 18,981 | 6,15% | 32,603 | 32,603 | 7,60 | 0,012 |
| 2-Way Interaction | 2 | 34,302 | 11,12% | 34,302 | 17,151 | 4,00 | 0,034 |
| Lac\*HPMC\_Visc | 1 | 14,966 | 4,85% | 14,966 | 14,966 | 3,49 | 0,076 |
| HPMC\_Visc\*HPMC\_PS | 1 | 19,336 | 6,27% | 19,336 | 19,336 | 4,50 | 0,046 |
| Error | 21 | 90,144 | 29,23% | 90,144 | 4,293 |  |  |
| Lack-of-Fit | 19 | 67,733 | 21,96% | 67,733 | 3,565 | 0,32 | 0,934 |
| Pure Error | 2 | 22,411 | 7,27% | 22,411 | 11,206 |  |  |
| Total | 26 | 308,419 | 100,00% |  |  |  |  |

## Regression Equation in Uncoded Units

|  |  |  |
| --- | --- | --- |
| F\_mean\_1.5h(90min) | = | 123,9 + 63,2 Lac - 0,01015 HPMC\_Visc - 1,87 HPMC\_PS - 0,00330 Lac\*HPMC\_Visc + 0,000170 HPMC\_Visc\*HPMC\_PS |

## Fits and Diagnostics for All Observations

| Obs | F\_mean\_1.5h(90min) | Fit | SE Fit | 95% CI | Resid | Std Resid | Del Resid |
| --- | --- | --- | --- | --- | --- | --- | --- |
| 1 | 23,960 | 22,791 | 1,012 | (20,687; 24,895) | 1,169 | 0,65 | 0,64 |
| 2 | 29,242 | 29,741 | 1,012 | (27,637; 31,844) | -0,498 | -0,28 | -0,27 |
| 3 | 20,030 | 21,481 | 1,374 | (18,624; 24,338) | -1,451 | -0,94 | -0,93 |
| 4 | 23,256 | 23,939 | 1,374 | (21,082; 26,796) | -0,683 | -0,44 | -0,43 |
| 5 | 20,814 | 22,831 | 0,904 | (20,951; 24,712) | -2,017 | -1,08 | -1,09 |
| 6 | 29,979 | 29,580 | 0,904 | (27,699; 31,461) | 0,399 | 0,21 | 0,21 |
| 7 | 21,354 | 22,715 | 1,188 | (20,245; 25,186) | -1,361 | -0,80 | -0,79 |
| 8 | 25,207 | 25,106 | 1,188 | (22,635; 27,577) | 0,101 | 0,06 | 0,06 |
| 9 | 23,222 | 22,680 | 1,024 | (20,550; 24,811) | 0,542 | 0,30 | 0,29 |
| 10 | 28,652 | 29,567 | 1,024 | (27,436; 31,697) | -0,915 | -0,51 | -0,50 |
| 11 | 24,520 | 25,226 | 0,891 | (23,373; 27,078) | -0,706 | -0,38 | -0,37 |
| 12 | 29,209 | 29,282 | 0,891 | (27,430; 31,135) | -0,073 | -0,04 | -0,04 |
| 13 | 22,644 | 22,623 | 1,133 | (20,267; 24,979) | 0,021 | 0,01 | 0,01 |
| 14 | 31,222 | 29,537 | 1,133 | (27,182; 31,893) | 1,685 | 0,97 | 0,97 |
| 15 | 26,099 | 25,742 | 1,042 | (23,574; 27,909) | 0,357 | 0,20 | 0,19 |
| 16 | 25,890 | 29,505 | 1,042 | (27,337; 31,672) | -3,614 | -2,02 | -2,19 |
| 17 | 21,094 | 20,960 | 0,955 | (18,974; 22,945) | 0,134 | 0,07 | 0,07 |
| 18 | 31,707 | 31,498 | 0,955 | (29,513; 33,483) | 0,210 | 0,11 | 0,11 |
| 19 | 26,440 | 25,979 | 0,813 | (24,287; 27,670) | 0,461 | 0,24 | 0,24 |
| 20 | 29,744 | 25,432 | 0,953 | (23,449; 27,414) | 4,313 | 2,34 | 2,66 |
| 21 | 26,102 | 26,067 | 0,446 | (25,139; 26,995) | 0,035 | 0,02 | 0,02 |
| 22 | 30,808 | 26,465 | 0,448 | (25,533; 27,397) | 4,343 | 2,15 | 2,37 |
| 23 | 24,646 | 24,372 | 1,204 | (21,869; 26,875) | 0,274 | 0,16 | 0,16 |
| 24 | 27,178 | 28,160 | 1,116 | (25,839; 30,481) | -0,982 | -0,56 | -0,55 |
| 25 | 23,641 | 26,229 | 0,434 | (25,327; 27,130) | -2,588 | -1,28 | -1,30 |
| 26 | 23,791 | 26,229 | 0,434 | (25,327; 27,130) | -2,438 | -1,20 | -1,22 |
| 27 | 29,512 | 26,229 | 0,434 | (25,327; 27,130) | 3,284 | 1,62 | 1,69 |

| Obs | HI | Cook’s D | DFITS |  |
| --- | --- | --- | --- | --- |
| 1 | 0,238422 | 0,02 | 0,35666 |  |
| 2 | 0,238422 | 0,00 | -0,15074 |  |
| 3 | 0,439577 | 0,11 | -0,82617 |  |
| 4 | 0,439577 | 0,03 | -0,38240 |  |
| 5 | 0,190525 | 0,05 | -0,52729 |  |
| 6 | 0,190525 | 0,00 | 0,10140 |  |
| 7 | 0,328781 | 0,05 | -0,55633 |  |
| 8 | 0,328781 | 0,00 | 0,04072 |  |
| 9 | 0,244482 | 0,00 | 0,16732 |  |
| 10 | 0,244482 | 0,01 | -0,28369 |  |
| 11 | 0,184860 | 0,01 | -0,17608 |  |
| 12 | 0,184860 | 0,00 | -0,01820 |  |
| 13 | 0,298927 | 0,00 | 0,00759 |  |
| 14 | 0,298927 | 0,07 | 0,63330 |  |
| 15 | 0,253021 | 0,00 | 0,11332 |  |
| 16 | 0,253021 | 0,23 | -1,27693 | R |
| 17 | 0,212283 | 0,00 | 0,03705 |  |
| 18 | 0,212283 | 0,00 | 0,05781 |  |
| 19 | 0,154147 | 0,00 | 0,10099 |  |
| 20 | 0,211709 | 0,25 | 1,37997 | R |
| 21 | 0,046409 | 0,00 | 0,00375 |  |
| 22 | 0,046809 | 0,04 | 0,52560 | R |
| 23 | 0,337586 | 0,00 | 0,11326 |  |
| 24 | 0,290228 | 0,02 | -0,35382 |  |
| 25 | 0,043786 | 0,01 | -0,27776 |  |
| 26 | 0,043786 | 0,01 | -0,26042 |  |
| 27 | 0,043786 | 0,02 | 0,36186 |  |

R  Large residual

## Backward Elimination of Terms

α to remove = 0,1

## Coded Coefficients

| Term | Coef | SE Coef | 95% CI | T-Value | P-Value | VIF |
| --- | --- | --- | --- | --- | --- | --- |
| Constant | 30,140 | 0,483 | (29,136; 31,145) | 62,43 | 0,000 |  |
| Lac | 5,113 | 0,907 | (3,227; 6,999) | 5,64 | 0,000 | 1,08 |
| HPMC\_Visc | 0,084 | 0,879 | (-1,744; 1,911) | 0,10 | 0,925 | 1,33 |
| HPMC\_PS | 3,27 | 1,19 | (0,78; 5,75) | 2,74 | 0,012 | 1,34 |
| Lac\*HPMC\_Visc | -3,38 | 1,77 | (-7,07; 0,31) | -1,91 | 0,070 | 1,08 |
| HPMC\_Visc\*HPMC\_PS | 5,02 | 2,37 | (0,09; 9,94) | 2,12 | 0,046 | 1,25 |

## Model Summary

| S | R-sq | R-sq(adj) | PRESS | R-sq(pred) | AICc | BIC |
| --- | --- | --- | --- | --- | --- | --- |
| 2,14149 | 72,59% | 66,06% | 142,042 | 59,57% | 130,85 | 134,03 |

## Analysis of Variance

| Source | DF | Seq SS | Contribution | Adj SS | Adj MS | F-Value | P-Value |
| --- | --- | --- | --- | --- | --- | --- | --- |
| Model | 5 | 255,03 | 72,59% | 255,033 | 51,007 | 11,12 | 0,000 |
| Linear | 3 | 217,85 | 62,00% | 187,358 | 62,453 | 13,62 | 0,000 |
| Lac | 1 | 186,33 | 53,03% | 145,739 | 145,739 | 31,78 | 0,000 |
| HPMC\_Visc | 1 | 11,63 | 3,31% | 0,041 | 0,041 | 0,01 | 0,925 |
| HPMC\_PS | 1 | 19,89 | 5,66% | 34,306 | 34,306 | 7,48 | 0,012 |
| 2-Way Interaction | 2 | 37,19 | 10,58% | 37,187 | 18,594 | 4,05 | 0,032 |
| Lac\*HPMC\_Visc | 1 | 16,65 | 4,74% | 16,651 | 16,651 | 3,63 | 0,070 |
| HPMC\_Visc\*HPMC\_PS | 1 | 20,54 | 5,85% | 20,536 | 20,536 | 4,48 | 0,046 |
| Error | 21 | 96,31 | 27,41% | 96,306 | 4,586 |  |  |
| Lack-of-Fit | 19 | 73,95 | 21,05% | 73,946 | 3,892 | 0,35 | 0,919 |
| Pure Error | 2 | 22,36 | 6,36% | 22,359 | 11,180 |  |  |
| Total | 26 | 351,34 | 100,00% |  |  |  |  |

## Regression Equation in Uncoded Units

|  |  |  |
| --- | --- | --- |
| F\_mean\_2h(120min) | = | 130,2 + 67,4 Lac - 0,01045 HPMC\_Visc - 1,92 HPMC\_PS - 0,00348 Lac\*HPMC\_Visc + 0,000175 HPMC\_Visc\*HPMC\_PS |

## Fits and Diagnostics for All Observations

| Obs | F\_mean\_2h(120min) | Fit | SE Fit | 95% CI | Resid | Std Resid | Del Resid |
| --- | --- | --- | --- | --- | --- | --- | --- |
| 1 | 27,647 | 26,397 | 1,046 | (24,222; 28,571) | 1,251 | 0,67 | 0,66 |
| 2 | 33,275 | 33,916 | 1,046 | (31,742; 36,091) | -0,642 | -0,34 | -0,34 |
| 3 | 23,330 | 24,917 | 1,420 | (21,965; 27,870) | -1,588 | -0,99 | -0,99 |
| 4 | 27,018 | 27,699 | 1,420 | (24,747; 30,652) | -0,681 | -0,42 | -0,42 |
| 5 | 24,319 | 26,431 | 0,935 | (24,487; 28,375) | -2,112 | -1,10 | -1,10 |
| 6 | 33,930 | 33,739 | 0,935 | (31,795; 35,683) | 0,191 | 0,10 | 0,10 |
| 7 | 24,866 | 26,184 | 1,228 | (23,630; 28,738) | -1,318 | -0,75 | -0,74 |
| 8 | 28,958 | 28,895 | 1,228 | (26,342; 31,449) | 0,062 | 0,04 | 0,03 |
| 9 | 26,769 | 26,271 | 1,059 | (24,069; 28,473) | 0,498 | 0,27 | 0,26 |
| 10 | 32,839 | 33,724 | 1,059 | (31,522; 35,926) | -0,885 | -0,48 | -0,47 |
| 11 | 28,140 | 28,812 | 0,921 | (26,898; 30,727) | -0,672 | -0,35 | -0,34 |
| 12 | 33,175 | 33,280 | 0,921 | (31,365; 35,195) | -0,105 | -0,05 | -0,05 |
| 13 | 26,177 | 26,211 | 1,171 | (23,776; 28,646) | -0,034 | -0,02 | -0,02 |
| 14 | 35,420 | 33,693 | 1,171 | (31,258; 36,128) | 1,726 | 0,96 | 0,96 |
| 15 | 29,734 | 29,334 | 1,077 | (27,094; 31,574) | 0,400 | 0,22 | 0,21 |
| 16 | 29,696 | 33,493 | 1,077 | (31,253; 35,733) | -3,797 | -2,05 | -2,24 |
| 17 | 24,325 | 24,297 | 0,987 | (22,245; 26,349) | 0,028 | 0,01 | 0,01 |
| 18 | 36,098 | 35,791 | 0,987 | (33,740; 37,843) | 0,306 | 0,16 | 0,16 |
| 19 | 30,544 | 29,894 | 0,841 | (28,145; 31,642) | 0,650 | 0,33 | 0,32 |
| 20 | 33,491 | 29,064 | 0,985 | (27,015; 31,113) | 4,427 | 2,33 | 2,64 |
| 21 | 29,839 | 29,878 | 0,461 | (28,918; 30,837) | -0,039 | -0,02 | -0,02 |
| 22 | 34,911 | 30,277 | 0,463 | (29,313; 31,240) | 4,635 | 2,22 | 2,47 |
| 23 | 28,553 | 28,155 | 1,244 | (25,568; 30,743) | 0,398 | 0,23 | 0,22 |
| 24 | 31,053 | 32,030 | 1,154 | (29,631; 34,429) | -0,977 | -0,54 | -0,53 |
| 25 | 27,483 | 30,044 | 0,448 | (29,112; 30,976) | -2,561 | -1,22 | -1,24 |
| 26 | 27,597 | 30,044 | 0,448 | (29,112; 30,976) | -2,447 | -1,17 | -1,18 |
| 27 | 33,330 | 30,044 | 0,448 | (29,112; 30,976) | 3,286 | 1,57 | 1,63 |

| Obs | HI | Cook’s D | DFITS |  |
| --- | --- | --- | --- | --- |
| 1 | 0,238422 | 0,02 | 0,36941 |  |
| 2 | 0,238422 | 0,01 | -0,18800 |  |
| 3 | 0,439577 | 0,13 | -0,87661 |  |
| 4 | 0,439577 | 0,02 | -0,36886 |  |
| 5 | 0,190525 | 0,05 | -0,53449 |  |
| 6 | 0,190525 | 0,00 | 0,04701 |  |
| 7 | 0,328781 | 0,05 | -0,52010 |  |
| 8 | 0,328781 | 0,00 | 0,02430 |  |
| 9 | 0,244482 | 0,00 | 0,14882 |  |
| 10 | 0,244482 | 0,01 | -0,26539 |  |
| 11 | 0,184860 | 0,00 | -0,16198 |  |
| 12 | 0,184860 | 0,00 | -0,02533 |  |
| 13 | 0,298927 | 0,00 | -0,01206 |  |
| 14 | 0,298927 | 0,07 | 0,62759 |  |
| 15 | 0,253021 | 0,00 | 0,12273 |  |
| 16 | 0,253021 | 0,24 | -1,30317 | R |
| 17 | 0,212283 | 0,00 | 0,00757 |  |
| 18 | 0,212283 | 0,00 | 0,08165 |  |
| 19 | 0,154147 | 0,00 | 0,13779 |  |
| 20 | 0,211709 | 0,24 | 1,36702 | R |
| 21 | 0,046409 | 0,00 | -0,00400 |  |
| 22 | 0,046809 | 0,04 | 0,54772 | R |
| 23 | 0,337586 | 0,00 | 0,15920 |  |
| 24 | 0,290228 | 0,02 | -0,34028 |  |
| 25 | 0,043786 | 0,01 | -0,26505 |  |
| 26 | 0,043786 | 0,01 | -0,25237 |  |
| 27 | 0,043786 | 0,02 | 0,34880 |  |

R  Large residual

## Backward Elimination of Terms

α to remove = 0,1

## Coded Coefficients

| Term | Coef | SE Coef | 95% CI | T-Value | P-Value | VIF |
| --- | --- | --- | --- | --- | --- | --- |
| Constant | 33,575 | 0,495 | (32,545; 34,605) | 67,81 | 0,000 |  |
| Lac | 5,472 | 0,930 | (3,538; 7,406) | 5,88 | 0,000 | 1,08 |
| HPMC\_Visc | -0,036 | 0,901 | (-1,911; 1,838) | -0,04 | 0,968 | 1,33 |
| HPMC\_PS | 3,31 | 1,22 | (0,77; 5,86) | 2,70 | 0,013 | 1,34 |
| Lac\*HPMC\_Visc | -3,57 | 1,82 | (-7,35; 0,21) | -1,96 | 0,063 | 1,08 |
| HPMC\_Visc\*HPMC\_PS | 5,18 | 2,43 | (0,13; 10,24) | 2,13 | 0,045 | 1,25 |

## Model Summary

| S | R-sq | R-sq(adj) | PRESS | R-sq(pred) | AICc | BIC |
| --- | --- | --- | --- | --- | --- | --- |
| 2,19604 | 74,00% | 67,81% | 149,451 | 61,63% | 132,21 | 135,39 |

## Analysis of Variance

| Source | DF | Seq SS | Contribution | Adj SS | Adj MS | F-Value | P-Value |
| --- | --- | --- | --- | --- | --- | --- | --- |
| Model | 5 | 288,19 | 74,00% | 288,195 | 57,639 | 11,95 | 0,000 |
| Linear | 3 | 247,70 | 63,60% | 211,669 | 70,556 | 14,63 | 0,000 |
| Lac | 1 | 212,96 | 54,68% | 166,944 | 166,944 | 34,62 | 0,000 |
| HPMC\_Visc | 1 | 14,61 | 3,75% | 0,008 | 0,008 | 0,00 | 0,968 |
| HPMC\_PS | 1 | 20,13 | 5,17% | 35,284 | 35,284 | 7,32 | 0,013 |
| 2-Way Interaction | 2 | 40,49 | 10,40% | 40,492 | 20,246 | 4,20 | 0,029 |
| Lac\*HPMC\_Visc | 1 | 18,56 | 4,77% | 18,561 | 18,561 | 3,85 | 0,063 |
| HPMC\_Visc\*HPMC\_PS | 1 | 21,93 | 5,63% | 21,931 | 21,931 | 4,55 | 0,045 |
| Error | 21 | 101,27 | 26,00% | 101,275 | 4,823 |  |  |
| Lack-of-Fit | 19 | 79,23 | 20,34% | 79,230 | 4,170 | 0,38 | 0,903 |
| Pure Error | 2 | 22,04 | 5,66% | 22,045 | 11,022 |  |  |
| Total | 26 | 389,47 | 100,00% |  |  |  |  |

## Regression Equation in Uncoded Units

|  |  |  |
| --- | --- | --- |
| F\_mean\_2.5h(150min) | = | 137,1 + 71,5 Lac - 0,01079 HPMC\_Visc - 2,00 HPMC\_PS - 0,00367 Lac\*HPMC\_Visc + 0,000181 HPMC\_Visc\*HPMC\_PS |

## Fits and Diagnostics for All Observations

| Obs | F\_mean\_2.5h(150min) | Fit | SE Fit | 95% CI | Resid | Std Resid | Del Resid |
| --- | --- | --- | --- | --- | --- | --- | --- |
| 1 | 30,966 | 29,688 | 1,072 | (27,458; 31,918) | 1,278 | 0,67 | 0,66 |
| 2 | 36,876 | 37,701 | 1,072 | (35,471; 39,931) | -0,825 | -0,43 | -0,42 |
| 3 | 26,375 | 28,061 | 1,456 | (25,033; 31,089) | -1,685 | -1,03 | -1,03 |
| 4 | 30,399 | 31,072 | 1,456 | (28,044; 34,100) | -0,673 | -0,41 | -0,40 |
| 5 | 27,471 | 29,713 | 0,959 | (27,720; 31,707) | -2,243 | -1,14 | -1,14 |
| 6 | 37,628 | 37,503 | 0,959 | (35,509; 39,496) | 0,125 | 0,06 | 0,06 |
| 7 | 28,090 | 29,356 | 1,259 | (26,738; 31,975) | -1,266 | -0,70 | -0,70 |
| 8 | 32,301 | 32,293 | 1,259 | (29,674; 34,912) | 0,008 | 0,00 | 0,00 |
| 9 | 29,986 | 29,514 | 1,086 | (27,256; 31,773) | 0,472 | 0,25 | 0,24 |
| 10 | 36,545 | 37,457 | 1,086 | (35,199; 39,716) | -0,912 | -0,48 | -0,47 |
| 11 | 31,457 | 32,079 | 0,944 | (30,115; 34,042) | -0,622 | -0,31 | -0,31 |
| 12 | 36,736 | 36,870 | 0,944 | (34,907; 38,834) | -0,134 | -0,07 | -0,07 |
| 13 | 29,407 | 29,447 | 1,201 | (26,950; 31,944) | -0,039 | -0,02 | -0,02 |
| 14 | 39,136 | 37,421 | 1,201 | (34,924; 39,918) | 1,715 | 0,93 | 0,93 |
| 15 | 33,013 | 32,607 | 1,105 | (30,310; 34,904) | 0,406 | 0,21 | 0,21 |
| 16 | 33,169 | 37,072 | 1,105 | (34,774; 39,369) | -3,902 | -2,06 | -2,25 |
| 17 | 27,300 | 27,359 | 1,012 | (25,255; 29,463) | -0,059 | -0,03 | -0,03 |
| 18 | 40,033 | 39,643 | 1,012 | (37,539; 41,747) | 0,390 | 0,20 | 0,20 |
| 19 | 34,291 | 33,437 | 0,862 | (31,644; 35,230) | 0,853 | 0,42 | 0,41 |
| 20 | 36,830 | 32,349 | 1,010 | (30,248; 34,450) | 4,481 | 2,30 | 2,59 |
| 21 | 33,170 | 33,332 | 0,473 | (32,348; 34,316) | -0,162 | -0,08 | -0,07 |
| 22 | 38,633 | 33,727 | 0,475 | (32,739; 34,715) | 4,906 | 2,29 | 2,58 |
| 23 | 32,129 | 31,618 | 1,276 | (28,965; 34,272) | 0,510 | 0,29 | 0,28 |
| 24 | 34,525 | 35,498 | 1,183 | (33,038; 37,958) | -0,973 | -0,53 | -0,52 |
| 25 | 30,945 | 33,501 | 0,460 | (32,545; 34,457) | -2,556 | -1,19 | -1,20 |
| 26 | 31,126 | 33,501 | 0,460 | (32,545; 34,457) | -2,375 | -1,11 | -1,11 |
| 27 | 36,783 | 33,501 | 0,460 | (32,545; 34,457) | 3,283 | 1,53 | 1,58 |

| Obs | HI | Cook’s D | DFITS |  |
| --- | --- | --- | --- | --- |
| 1 | 0,238422 | 0,02 | 0,36799 |  |
| 2 | 0,238422 | 0,01 | -0,23605 |  |
| 3 | 0,439577 | 0,14 | -0,90914 |  |
| 4 | 0,439577 | 0,02 | -0,35522 |  |
| 5 | 0,190525 | 0,05 | -0,55466 |  |
| 6 | 0,190525 | 0,00 | 0,02990 |  |
| 7 | 0,328781 | 0,04 | -0,48643 |  |
| 8 | 0,328781 | 0,00 | 0,00319 |  |
| 9 | 0,244482 | 0,00 | 0,13739 |  |
| 10 | 0,244482 | 0,01 | -0,26682 |  |
| 11 | 0,184860 | 0,00 | -0,14617 |  |
| 12 | 0,184860 | 0,00 | -0,03140 |  |
| 13 | 0,298927 | 0,00 | -0,01369 |  |
| 14 | 0,298927 | 0,06 | 0,60701 |  |
| 15 | 0,253021 | 0,00 | 0,12171 |  |
| 16 | 0,253021 | 0,24 | -1,30673 | R |
| 17 | 0,212283 | 0,00 | -0,01535 |  |
| 18 | 0,212283 | 0,00 | 0,10154 |  |
| 19 | 0,154147 | 0,01 | 0,17676 |  |
| 20 | 0,211709 | 0,24 | 1,34365 | R |
| 21 | 0,046409 | 0,00 | -0,01627 |  |
| 22 | 0,046809 | 0,04 | 0,57120 | R |
| 23 | 0,337586 | 0,01 | 0,19929 |  |
| 24 | 0,290228 | 0,02 | -0,33042 |  |
| 25 | 0,043786 | 0,01 | -0,25740 |  |
| 26 | 0,043786 | 0,01 | -0,23803 |  |
| 27 | 0,043786 | 0,02 | 0,33861 |  |

R  Large residual

## Backward Elimination of Terms

α to remove = 0,1

## Coded Coefficients

| Term | Coef | SE Coef | 95% CI | T-Value | P-Value | VIF |
| --- | --- | --- | --- | --- | --- | --- |
| Constant | 36,738 | 0,502 | (35,695; 37,782) | 73,22 | 0,000 |  |
| Lac | 5,795 | 0,943 | (3,835; 7,755) | 6,15 | 0,000 | 1,08 |
| HPMC\_Visc | -0,149 | 0,913 | (-2,049; 1,751) | -0,16 | 0,872 | 1,33 |
| HPMC\_PS | 3,33 | 1,24 | (0,75; 5,91) | 2,68 | 0,014 | 1,34 |
| Lac\*HPMC\_Visc | -3,65 | 1,84 | (-7,49; 0,18) | -1,98 | 0,061 | 1,08 |
| HPMC\_Visc\*HPMC\_PS | 5,26 | 2,46 | (0,14; 10,38) | 2,14 | 0,045 | 1,25 |

## Model Summary

| S | R-sq | R-sq(adj) | PRESS | R-sq(pred) | AICc | BIC |
| --- | --- | --- | --- | --- | --- | --- |
| 2,22552 | 75,30% | 69,42% | 153,378 | 63,58% | 132,93 | 136,11 |

## Analysis of Variance

| Source | DF | Seq SS | Contribution | Adj SS | Adj MS | F-Value | P-Value |
| --- | --- | --- | --- | --- | --- | --- | --- |
| Model | 5 | 317,08 | 75,30% | 317,076 | 63,415 | 12,80 | 0,000 |
| Linear | 3 | 275,04 | 65,32% | 234,360 | 78,120 | 15,77 | 0,000 |
| Lac | 1 | 237,54 | 56,41% | 187,250 | 187,250 | 37,81 | 0,000 |
| HPMC\_Visc | 1 | 17,38 | 4,13% | 0,132 | 0,132 | 0,03 | 0,872 |
| HPMC\_PS | 1 | 20,13 | 4,78% | 35,599 | 35,599 | 7,19 | 0,014 |
| 2-Way Interaction | 2 | 42,03 | 9,98% | 42,032 | 21,016 | 4,24 | 0,028 |
| Lac\*HPMC\_Visc | 1 | 19,43 | 4,61% | 19,426 | 19,426 | 3,92 | 0,061 |
| HPMC\_Visc\*HPMC\_PS | 1 | 22,61 | 5,37% | 22,606 | 22,606 | 4,56 | 0,045 |
| Error | 21 | 104,01 | 24,70% | 104,012 | 4,953 |  |  |
| Lack-of-Fit | 19 | 82,39 | 19,57% | 82,392 | 4,336 | 0,40 | 0,891 |
| Pure Error | 2 | 21,62 | 5,13% | 21,620 | 10,810 |  |  |
| Total | 26 | 421,09 | 100,00% |  |  |  |  |

## Regression Equation in Uncoded Units

|  |  |  |
| --- | --- | --- |
| F\_mean\_3h(180min) | = | 141,9 + 73,9 Lac - 0,01097 HPMC\_Visc - 2,03 HPMC\_PS - 0,00375 Lac\*HPMC\_Visc + 0,000184 HPMC\_Visc\*HPMC\_PS |

## Fits and Diagnostics for All Observations

| Obs | F\_mean\_3h(180min) | Fit | SE Fit | 95% CI | Resid | Std Resid | Del Resid |
| --- | --- | --- | --- | --- | --- | --- | --- |
| 1 | 34,028 | 32,751 | 1,087 | (30,491; 35,011) | 1,278 | 0,66 | 0,65 |
| 2 | 40,223 | 41,146 | 1,087 | (38,886; 43,406) | -0,923 | -0,48 | -0,47 |
| 3 | 29,168 | 30,973 | 1,476 | (27,905; 34,042) | -1,805 | -1,08 | -1,09 |
| 4 | 33,625 | 34,251 | 1,476 | (31,182; 37,320) | -0,626 | -0,38 | -0,37 |
| 5 | 30,469 | 32,768 | 0,971 | (30,747; 34,788) | -2,298 | -1,15 | -1,16 |
| 6 | 40,923 | 40,934 | 0,971 | (38,914; 42,954) | -0,011 | -0,01 | -0,01 |
| 7 | 31,112 | 32,280 | 1,276 | (29,626; 34,933) | -1,168 | -0,64 | -0,63 |
| 8 | 35,438 | 35,481 | 1,276 | (32,827; 38,135) | -0,043 | -0,02 | -0,02 |
| 9 | 32,975 | 32,549 | 1,100 | (30,261; 34,837) | 0,426 | 0,22 | 0,22 |
| 10 | 39,972 | 40,872 | 1,100 | (38,584; 43,161) | -0,901 | -0,47 | -0,46 |
| 11 | 34,494 | 35,073 | 0,957 | (33,083; 37,063) | -0,579 | -0,29 | -0,28 |
| 12 | 39,969 | 40,172 | 0,957 | (38,182; 42,162) | -0,203 | -0,10 | -0,10 |
| 13 | 32,410 | 32,477 | 1,217 | (29,947; 35,008) | -0,067 | -0,04 | -0,04 |
| 14 | 42,546 | 40,832 | 1,217 | (38,302; 43,363) | 1,713 | 0,92 | 0,92 |
| 15 | 35,988 | 35,598 | 1,119 | (33,270; 37,926) | 0,390 | 0,20 | 0,20 |
| 16 | 36,412 | 40,363 | 1,119 | (38,035; 42,691) | -3,951 | -2,05 | -2,24 |
| 17 | 30,093 | 30,205 | 1,025 | (28,072; 32,337) | -0,112 | -0,06 | -0,06 |
| 18 | 43,614 | 43,166 | 1,025 | (41,034; 45,298) | 0,449 | 0,23 | 0,22 |
| 19 | 37,657 | 36,707 | 0,874 | (34,890; 38,524) | 0,950 | 0,46 | 0,46 |
| 20 | 39,839 | 35,387 | 1,024 | (33,257; 37,516) | 4,453 | 2,25 | 2,53 |
| 21 | 36,222 | 36,516 | 0,479 | (35,519; 37,513) | -0,294 | -0,14 | -0,13 |
| 22 | 42,049 | 36,904 | 0,481 | (35,902; 37,905) | 5,146 | 2,37 | 2,70 |
| 23 | 35,448 | 34,817 | 1,293 | (32,128; 37,506) | 0,632 | 0,35 | 0,34 |
| 24 | 37,768 | 38,685 | 1,199 | (36,192; 41,178) | -0,917 | -0,49 | -0,48 |
| 25 | 34,152 | 36,685 | 0,466 | (35,717; 37,654) | -2,533 | -1,16 | -1,17 |
| 26 | 34,401 | 36,685 | 0,466 | (35,717; 37,654) | -2,284 | -1,05 | -1,05 |
| 27 | 39,967 | 36,685 | 0,466 | (35,717; 37,654) | 3,282 | 1,51 | 1,56 |

| Obs | HI | Cook’s D | DFITS |  |
| --- | --- | --- | --- | --- |
| 1 | 0,238422 | 0,02 | 0,36293 |  |
| 2 | 0,238422 | 0,01 | -0,26093 |  |
| 3 | 0,439577 | 0,15 | -0,96394 |  |
| 4 | 0,439577 | 0,02 | -0,32603 |  |
| 5 | 0,190525 | 0,05 | -0,56134 |  |
| 6 | 0,190525 | 0,00 | -0,00263 |  |
| 7 | 0,328781 | 0,03 | -0,44186 |  |
| 8 | 0,328781 | 0,00 | -0,01603 |  |
| 9 | 0,244482 | 0,00 | 0,12231 |  |
| 10 | 0,244482 | 0,01 | -0,25979 |  |
| 11 | 0,184860 | 0,00 | -0,13416 |  |
| 12 | 0,184860 | 0,00 | -0,04707 |  |
| 13 | 0,298927 | 0,00 | -0,02293 |  |
| 14 | 0,298927 | 0,06 | 0,59806 |  |
| 15 | 0,253021 | 0,00 | 0,11513 |  |
| 16 | 0,253021 | 0,24 | -1,30533 | R |
| 17 | 0,212283 | 0,00 | -0,02867 |  |
| 18 | 0,212283 | 0,00 | 0,11519 |  |
| 19 | 0,154147 | 0,01 | 0,19426 |  |
| 20 | 0,211709 | 0,23 | 1,30887 | R |
| 21 | 0,046409 | 0,00 | -0,02914 |  |
| 22 | 0,046809 | 0,05 | 0,59825 | R |
| 23 | 0,337586 | 0,01 | 0,24360 |  |
| 24 | 0,290228 | 0,02 | -0,30685 |  |
| 25 | 0,043786 | 0,01 | -0,25133 |  |
| 26 | 0,043786 | 0,01 | -0,22515 |  |
| 27 | 0,043786 | 0,02 | 0,33352 |  |

R  Large residual

## Backward Elimination of Terms

α to remove = 0,1

## Coded Coefficients

| Term | Coef | SE Coef | 95% CI | T-Value | P-Value | VIF |
| --- | --- | --- | --- | --- | --- | --- |
| Constant | 39,724 | 0,507 | (38,669; 40,779) | 78,33 | 0,000 |  |
| Lac | 6,105 | 0,953 | (4,124; 8,086) | 6,41 | 0,000 | 1,08 |
| HPMC\_Visc | -0,252 | 0,923 | (-2,172; 1,668) | -0,27 | 0,787 | 1,33 |
| HPMC\_PS | 3,32 | 1,25 | (0,72; 5,93) | 2,65 | 0,015 | 1,34 |
| Lac\*HPMC\_Visc | -3,76 | 1,86 | (-7,64; 0,11) | -2,02 | 0,056 | 1,08 |
| HPMC\_Visc\*HPMC\_PS | 5,34 | 2,49 | (0,16; 10,51) | 2,14 | 0,044 | 1,25 |

## Model Summary

| S | R-sq | R-sq(adj) | PRESS | R-sq(pred) | AICc | BIC |
| --- | --- | --- | --- | --- | --- | --- |
| 2,24928 | 76,53% | 70,94% | 156,590 | 65,41% | 133,50 | 136,68 |

## Analysis of Variance

| Source | DF | Seq SS | Contribution | Adj SS | Adj MS | F-Value | P-Value |
| --- | --- | --- | --- | --- | --- | --- | --- |
| Model | 5 | 346,41 | 76,53% | 346,407 | 69,281 | 13,69 | 0,000 |
| Linear | 3 | 302,55 | 66,84% | 256,820 | 85,607 | 16,92 | 0,000 |
| Lac | 1 | 262,71 | 58,04% | 207,818 | 207,818 | 41,08 | 0,000 |
| HPMC\_Visc | 1 | 20,02 | 4,42% | 0,378 | 0,378 | 0,07 | 0,787 |
| HPMC\_PS | 1 | 19,82 | 4,38% | 35,527 | 35,527 | 7,02 | 0,015 |
| 2-Way Interaction | 2 | 43,86 | 9,69% | 43,860 | 21,930 | 4,33 | 0,027 |
| Lac\*HPMC\_Visc | 1 | 20,61 | 4,55% | 20,614 | 20,614 | 4,07 | 0,056 |
| HPMC\_Visc\*HPMC\_PS | 1 | 23,25 | 5,14% | 23,246 | 23,246 | 4,59 | 0,044 |
| Error | 21 | 106,24 | 23,47% | 106,244 | 5,059 |  |  |
| Lack-of-Fit | 19 | 84,57 | 18,68% | 84,574 | 4,451 | 0,41 | 0,885 |
| Pure Error | 2 | 21,67 | 4,79% | 21,670 | 10,835 |  |  |
| Total | 26 | 452,65 | 100,00% |  |  |  |  |

## Regression Equation in Uncoded Units

|  |  |  |
| --- | --- | --- |
| F\_mean\_3.5h(210min) | = | 146,3 + 76,7 Lac - 0,01112 HPMC\_Visc - 2,07 HPMC\_PS - 0,00387 Lac\*HPMC\_Visc + 0,000187 HPMC\_Visc\*HPMC\_PS |

## Fits and Diagnostics for All Observations

| Obs | F\_mean\_3.5h(210min) | Fit | SE Fit | 95% CI | Resid | Std Resid | Del Resid |
| --- | --- | --- | --- | --- | --- | --- | --- |
| 1 | 36,866 | 35,630 | 1,098 | (33,346; 37,914) | 1,236 | 0,63 | 0,62 |
| 2 | 43,414 | 44,413 | 1,098 | (42,129; 46,697) | -0,999 | -0,51 | -0,50 |
| 3 | 31,856 | 33,743 | 1,491 | (30,642; 36,844) | -1,887 | -1,12 | -1,13 |
| 4 | 36,620 | 37,255 | 1,491 | (34,154; 40,356) | -0,635 | -0,38 | -0,37 |
| 5 | 33,315 | 35,638 | 0,982 | (33,597; 37,680) | -2,323 | -1,15 | -1,16 |
| 6 | 44,065 | 44,186 | 0,982 | (42,144; 46,228) | -0,121 | -0,06 | -0,06 |
| 7 | 33,956 | 35,057 | 1,290 | (32,375; 37,739) | -1,101 | -0,60 | -0,59 |
| 8 | 38,419 | 38,490 | 1,290 | (35,808; 41,172) | -0,072 | -0,04 | -0,04 |
| 9 | 35,760 | 35,390 | 1,112 | (33,077; 37,703) | 0,369 | 0,19 | 0,18 |
| 10 | 43,218 | 44,100 | 1,112 | (41,787; 46,412) | -0,881 | -0,45 | -0,44 |
| 11 | 37,353 | 37,897 | 0,967 | (35,886; 39,908) | -0,544 | -0,27 | -0,26 |
| 12 | 42,997 | 43,285 | 0,967 | (41,274; 45,296) | -0,288 | -0,14 | -0,14 |
| 13 | 35,261 | 35,313 | 1,230 | (32,755; 37,870) | -0,052 | -0,03 | -0,03 |
| 14 | 45,725 | 44,055 | 1,230 | (41,498; 46,613) | 1,670 | 0,89 | 0,88 |
| 15 | 38,758 | 38,420 | 1,131 | (36,067; 40,773) | 0,338 | 0,17 | 0,17 |
| 16 | 39,498 | 43,464 | 1,131 | (41,111; 45,817) | -3,966 | -2,04 | -2,22 |
| 17 | 32,680 | 32,880 | 1,036 | (30,724; 35,035) | -0,199 | -0,10 | -0,10 |
| 18 | 46,967 | 46,502 | 1,036 | (44,347; 48,657) | 0,465 | 0,23 | 0,23 |
| 19 | 40,842 | 39,789 | 0,883 | (37,952; 41,625) | 1,053 | 0,51 | 0,50 |
| 20 | 42,741 | 38,259 | 1,035 | (36,107; 40,411) | 4,482 | 2,24 | 2,51 |
| 21 | 39,143 | 39,522 | 0,485 | (38,514; 40,530) | -0,378 | -0,17 | -0,17 |
| 22 | 45,186 | 39,900 | 0,487 | (38,888; 40,912) | 5,285 | 2,41 | 2,76 |
| 23 | 38,661 | 37,852 | 1,307 | (35,134; 40,569) | 0,810 | 0,44 | 0,43 |
| 24 | 40,884 | 41,675 | 1,212 | (39,155; 44,195) | -0,792 | -0,42 | -0,41 |
| 25 | 37,155 | 39,691 | 0,471 | (38,712; 40,670) | -2,536 | -1,15 | -1,16 |
| 26 | 37,450 | 39,691 | 0,471 | (38,712; 40,670) | -2,241 | -1,02 | -1,02 |
| 27 | 42,998 | 39,691 | 0,471 | (38,712; 40,670) | 3,307 | 1,50 | 1,55 |

| Obs | HI | Cook’s D | DFITS |  |
| --- | --- | --- | --- | --- |
| 1 | 0,238422 | 0,02 | 0,34716 |  |
| 2 | 0,238422 | 0,01 | -0,27966 |  |
| 3 | 0,439577 | 0,16 | -0,99876 |  |
| 4 | 0,439577 | 0,02 | -0,32696 |  |
| 5 | 0,190525 | 0,05 | -0,56146 |  |
| 6 | 0,190525 | 0,00 | -0,02834 |  |
| 7 | 0,328781 | 0,03 | -0,41167 |  |
| 8 | 0,328781 | 0,00 | -0,02652 |  |
| 9 | 0,244482 | 0,00 | 0,10498 |  |
| 10 | 0,244482 | 0,01 | -0,25148 |  |
| 11 | 0,184860 | 0,00 | -0,12466 |  |
| 12 | 0,184860 | 0,00 | -0,06587 |  |
| 13 | 0,298927 | 0,00 | -0,01763 |  |
| 14 | 0,298927 | 0,06 | 0,57580 |  |
| 15 | 0,253021 | 0,00 | 0,09873 |  |
| 16 | 0,253021 | 0,23 | -1,29394 | R |
| 17 | 0,212283 | 0,00 | -0,05056 |  |
| 18 | 0,212283 | 0,00 | 0,11809 |  |
| 19 | 0,154147 | 0,01 | 0,21340 |  |
| 20 | 0,211709 | 0,23 | 1,30188 | R |
| 21 | 0,046409 | 0,00 | -0,03711 |  |
| 22 | 0,046809 | 0,05 | 0,61162 | R |
| 23 | 0,337586 | 0,02 | 0,30961 |  |
| 24 | 0,290228 | 0,01 | -0,26180 |  |
| 25 | 0,043786 | 0,01 | -0,24877 |  |
| 26 | 0,043786 | 0,01 | -0,21824 |  |
| 27 | 0,043786 | 0,02 | 0,33240 |  |

R  Large residual

## Backward Elimination of Terms

α to remove = 0,1

## Coded Coefficients

| Term | Coef | SE Coef | 95% CI | T-Value | P-Value | VIF |
| --- | --- | --- | --- | --- | --- | --- |
| Constant | 42,539 | 0,508 | (41,482; 43,596) | 83,70 | 0,000 |  |
| Lac | 6,438 | 0,955 | (4,452; 8,423) | 6,74 | 0,000 | 1,08 |
| HPMC\_Visc | -0,305 | 0,925 | (-2,229; 1,619) | -0,33 | 0,745 | 1,33 |
| HPMC\_PS | 3,34 | 1,26 | (0,73; 5,96) | 2,66 | 0,015 | 1,34 |
| Lac\*HPMC\_Visc | -3,90 | 1,87 | (-7,79; -0,02) | -2,09 | 0,049 | 1,08 |
| HPMC\_Visc\*HPMC\_PS | 5,31 | 2,50 | (0,13; 10,50) | 2,13 | 0,045 | 1,25 |

## Model Summary

| S | R-sq | R-sq(adj) | PRESS | R-sq(pred) | AICc | BIC |
| --- | --- | --- | --- | --- | --- | --- |
| 2,25431 | 78,00% | 72,76% | 157,662 | 67,50% | 133,63 | 136,80 |

## Analysis of Variance

| Source | DF | Seq SS | Contribution | Adj SS | Adj MS | F-Value | P-Value |
| --- | --- | --- | --- | --- | --- | --- | --- |
| Model | 5 | 378,33 | 78,00% | 378,331 | 75,666 | 14,89 | 0,000 |
| Linear | 3 | 333,09 | 68,67% | 281,751 | 93,917 | 18,48 | 0,000 |
| Lac | 1 | 291,37 | 60,07% | 231,070 | 231,070 | 45,47 | 0,000 |
| HPMC\_Visc | 1 | 21,46 | 4,42% | 0,551 | 0,551 | 0,11 | 0,745 |
| HPMC\_PS | 1 | 20,26 | 4,18% | 35,989 | 35,989 | 7,08 | 0,015 |
| 2-Way Interaction | 2 | 45,24 | 9,33% | 45,241 | 22,620 | 4,45 | 0,024 |
| Lac\*HPMC\_Visc | 1 | 22,18 | 4,57% | 22,180 | 22,180 | 4,36 | 0,049 |
| HPMC\_Visc\*HPMC\_PS | 1 | 23,06 | 4,75% | 23,061 | 23,061 | 4,54 | 0,045 |
| Error | 21 | 106,72 | 22,00% | 106,720 | 5,082 |  |  |
| Lack-of-Fit | 19 | 85,52 | 17,63% | 85,519 | 4,501 | 0,42 | 0,878 |
| Pure Error | 2 | 21,20 | 4,37% | 21,201 | 10,600 |  |  |
| Total | 26 | 485,05 | 100,00% |  |  |  |  |

## Regression Equation in Uncoded Units

|  |  |  |
| --- | --- | --- |
| F\_mean\_4h(240min) | = | 146,8 + 80,0 Lac - 0,01101 HPMC\_Visc - 2,05 HPMC\_PS - 0,00401 Lac\*HPMC\_Visc + 0,000186 HPMC\_Visc\*HPMC\_PS |

## Fits and Diagnostics for All Observations

| Obs | F\_mean\_4h(240min) | Fit | SE Fit | 95% CI | Resid | Std Resid | Del Resid |
| --- | --- | --- | --- | --- | --- | --- | --- |
| 1 | 39,017 | 38,257 | 1,101 | (35,967; 40,546) | 0,760 | 0,39 | 0,38 |
| 2 | 46,450 | 47,472 | 1,101 | (45,183; 49,761) | -1,022 | -0,52 | -0,51 |
| 3 | 34,367 | 36,400 | 1,495 | (33,292; 39,508) | -2,033 | -1,20 | -1,22 |
| 4 | 39,535 | 40,148 | 1,495 | (37,039; 43,256) | -0,612 | -0,36 | -0,36 |
| 5 | 36,008 | 38,269 | 0,984 | (36,223; 40,316) | -2,261 | -1,11 | -1,12 |
| 6 | 46,980 | 47,241 | 0,984 | (45,194; 49,287) | -0,261 | -0,13 | -0,13 |
| 7 | 36,677 | 37,716 | 1,293 | (35,027; 40,404) | -1,038 | -0,56 | -0,55 |
| 8 | 41,293 | 41,382 | 1,293 | (38,694; 44,070) | -0,089 | -0,05 | -0,05 |
| 9 | 38,416 | 38,042 | 1,115 | (35,724; 40,360) | 0,375 | 0,19 | 0,19 |
| 10 | 46,211 | 47,181 | 1,115 | (44,863; 49,499) | -0,969 | -0,49 | -0,49 |
| 11 | 40,034 | 40,553 | 0,969 | (38,537; 42,568) | -0,519 | -0,25 | -0,25 |
| 12 | 45,897 | 46,246 | 0,969 | (44,231; 48,262) | -0,350 | -0,17 | -0,17 |
| 13 | 38,009 | 37,968 | 1,233 | (35,405; 40,531) | 0,041 | 0,02 | 0,02 |
| 14 | 48,773 | 47,141 | 1,233 | (44,578; 49,704) | 1,632 | 0,86 | 0,86 |
| 15 | 41,448 | 41,078 | 1,134 | (38,720; 43,436) | 0,370 | 0,19 | 0,19 |
| 16 | 42,454 | 46,414 | 1,134 | (44,056; 48,773) | -3,961 | -2,03 | -2,21 |
| 17 | 35,187 | 35,345 | 1,039 | (33,185; 37,505) | -0,158 | -0,08 | -0,08 |
| 18 | 50,190 | 49,685 | 1,039 | (47,525; 51,845) | 0,505 | 0,25 | 0,25 |
| 19 | 43,871 | 42,660 | 0,885 | (40,820; 44,501) | 1,211 | 0,58 | 0,57 |
| 20 | 45,413 | 41,022 | 1,037 | (38,864; 43,179) | 4,391 | 2,19 | 2,44 |
| 21 | 41,861 | 42,343 | 0,486 | (41,334; 43,353) | -0,483 | -0,22 | -0,21 |
| 22 | 48,167 | 42,723 | 0,488 | (41,708; 43,737) | 5,444 | 2,47 | 2,87 |
| 23 | 41,719 | 40,653 | 1,310 | (37,929; 43,377) | 1,066 | 0,58 | 0,57 |
| 24 | 43,771 | 44,532 | 1,214 | (42,006; 47,057) | -0,760 | -0,40 | -0,39 |
| 25 | 40,004 | 42,515 | 0,472 | (41,534; 43,496) | -2,511 | -1,14 | -1,15 |
| 26 | 40,421 | 42,515 | 0,472 | (41,534; 43,496) | -2,094 | -0,95 | -0,95 |
| 27 | 45,840 | 42,515 | 0,472 | (41,534; 43,496) | 3,325 | 1,51 | 1,56 |

| Obs | HI | Cook’s D | DFITS |  |
| --- | --- | --- | --- | --- |
| 1 | 0,238422 | 0,01 | 0,21176 |  |
| 2 | 0,238422 | 0,01 | -0,28557 |  |
| 3 | 0,439577 | 0,19 | -1,07915 |  |
| 4 | 0,439577 | 0,02 | -0,31466 |  |
| 5 | 0,190525 | 0,05 | -0,54420 |  |
| 6 | 0,190525 | 0,00 | -0,06093 |  |
| 7 | 0,328781 | 0,03 | -0,38684 |  |
| 8 | 0,328781 | 0,00 | -0,03294 |  |
| 9 | 0,244482 | 0,00 | 0,10621 |  |
| 10 | 0,244482 | 0,01 | -0,27626 |  |
| 11 | 0,184860 | 0,00 | -0,11866 |  |
| 12 | 0,184860 | 0,00 | -0,07987 |  |
| 13 | 0,298927 | 0,00 | 0,01388 |  |
| 14 | 0,298927 | 0,05 | 0,56106 |  |
| 15 | 0,253021 | 0,00 | 0,10792 |  |
| 16 | 0,253021 | 0,23 | -1,28826 | R |
| 17 | 0,212283 | 0,00 | -0,03999 |  |
| 18 | 0,212283 | 0,00 | 0,12819 |  |
| 19 | 0,154147 | 0,01 | 0,24539 |  |
| 20 | 0,211709 | 0,22 | 1,26388 | R |
| 21 | 0,046409 | 0,00 | -0,04724 |  |
| 22 | 0,046809 | 0,05 | 0,63544 | R |
| 23 | 0,337586 | 0,03 | 0,40809 |  |
| 24 | 0,290228 | 0,01 | -0,25081 |  |
| 25 | 0,043786 | 0,01 | -0,24558 |  |
| 26 | 0,043786 | 0,01 | -0,20277 |  |
| 27 | 0,043786 | 0,02 | 0,33360 |  |

R  Large residual

## Backward Elimination of Terms

α to remove = 0,1

## Coded Coefficients

| Term | Coef | SE Coef | 95% CI | T-Value | P-Value | VIF |
| --- | --- | --- | --- | --- | --- | --- |
| Constant | 45,243 | 0,513 | (44,176; 46,311) | 88,15 | 0,000 |  |
| Lac | 6,728 | 0,964 | (4,723; 8,733) | 6,98 | 0,000 | 1,08 |
| HPMC\_Visc | -0,367 | 0,934 | (-2,310; 1,576) | -0,39 | 0,698 | 1,33 |
| HPMC\_PS | 3,34 | 1,27 | (0,70; 5,98) | 2,63 | 0,016 | 1,34 |
| Lac\*HPMC\_Visc | -4,02 | 1,89 | (-7,94; -0,10) | -2,13 | 0,045 | 1,08 |
| HPMC\_Visc\*HPMC\_PS | 5,45 | 2,52 | (0,21; 10,69) | 2,16 | 0,042 | 1,25 |

## Model Summary

| S | R-sq | R-sq(adj) | PRESS | R-sq(pred) | AICc | BIC |
| --- | --- | --- | --- | --- | --- | --- |
| 2,27633 | 78,97% | 73,96% | 161,326 | 68,82% | 134,15 | 137,33 |

## Analysis of Variance

| Source | DF | Seq SS | Contribution | Adj SS | Adj MS | F-Value | P-Value |
| --- | --- | --- | --- | --- | --- | --- | --- |
| Model | 5 | 408,52 | 78,97% | 408,519 | 81,704 | 15,77 | 0,000 |
| Linear | 3 | 360,70 | 69,72% | 304,277 | 101,426 | 19,57 | 0,000 |
| Lac | 1 | 317,55 | 61,38% | 252,374 | 252,374 | 48,70 | 0,000 |
| HPMC\_Visc | 1 | 23,41 | 4,52% | 0,799 | 0,799 | 0,15 | 0,698 |
| HPMC\_PS | 1 | 19,74 | 3,82% | 35,908 | 35,908 | 6,93 | 0,016 |
| 2-Way Interaction | 2 | 47,82 | 9,24% | 47,822 | 23,911 | 4,61 | 0,022 |
| Lac\*HPMC\_Visc | 1 | 23,55 | 4,55% | 23,549 | 23,549 | 4,54 | 0,045 |
| HPMC\_Visc\*HPMC\_PS | 1 | 24,27 | 4,69% | 24,273 | 24,273 | 4,68 | 0,042 |
| Error | 21 | 108,82 | 21,03% | 108,816 | 5,182 |  |  |
| Lack-of-Fit | 19 | 88,13 | 17,04% | 88,135 | 4,639 | 0,45 | 0,865 |
| Pure Error | 2 | 20,68 | 4,00% | 20,681 | 10,340 |  |  |
| Total | 26 | 517,33 | 100,00% |  |  |  |  |

## Regression Equation in Uncoded Units

|  |  |  |
| --- | --- | --- |
| F\_mean\_4.5h(270min) | = | 152,8 + 82,8 Lac - 0,01130 HPMC\_Visc - 2,12 HPMC\_PS - 0,00413 Lac\*HPMC\_Visc + 0,000191 HPMC\_Visc\*HPMC\_PS |

## Fits and Diagnostics for All Observations

| Obs | F\_mean\_4.5h(270min) | Fit | SE Fit | 95% CI | Resid | Std Resid | Del Resid |
| --- | --- | --- | --- | --- | --- | --- | --- |
| 1 | 41,486 | 40,843 | 1,111 | (38,532; 43,155) | 0,642 | 0,32 | 0,32 |
| 2 | 49,332 | 50,434 | 1,111 | (48,122; 52,745) | -1,102 | -0,55 | -0,55 |
| 3 | 36,774 | 38,904 | 1,509 | (35,766; 42,043) | -2,130 | -1,25 | -1,27 |
| 4 | 42,246 | 42,861 | 1,509 | (39,722; 45,999) | -0,614 | -0,36 | -0,35 |
| 5 | 38,574 | 40,847 | 0,994 | (38,780; 42,913) | -2,273 | -1,11 | -1,12 |
| 6 | 49,792 | 50,185 | 0,994 | (48,119; 52,252) | -0,393 | -0,19 | -0,19 |
| 7 | 39,260 | 40,236 | 1,305 | (37,522; 42,951) | -0,976 | -0,52 | -0,51 |
| 8 | 44,011 | 44,108 | 1,305 | (41,394; 46,823) | -0,097 | -0,05 | -0,05 |
| 9 | 40,920 | 40,562 | 1,126 | (38,221; 42,903) | 0,358 | 0,18 | 0,18 |
| 10 | 49,100 | 50,073 | 1,126 | (47,733; 52,414) | -0,974 | -0,49 | -0,48 |
| 11 | 42,660 | 43,116 | 0,979 | (41,081; 45,152) | -0,456 | -0,22 | -0,22 |
| 12 | 48,643 | 49,077 | 0,979 | (47,042; 51,113) | -0,435 | -0,21 | -0,21 |
| 13 | 40,531 | 40,477 | 1,245 | (37,889; 43,066) | 0,054 | 0,03 | 0,03 |
| 14 | 51,641 | 50,024 | 1,245 | (47,436; 52,612) | 1,618 | 0,85 | 0,84 |
| 15 | 44,033 | 43,646 | 1,145 | (41,265; 46,027) | 0,387 | 0,20 | 0,19 |
| 16 | 45,279 | 49,239 | 1,145 | (46,858; 51,620) | -3,960 | -2,01 | -2,19 |
| 17 | 37,538 | 37,749 | 1,049 | (35,568; 39,931) | -0,211 | -0,10 | -0,10 |
| 18 | 53,285 | 52,715 | 1,049 | (50,533; 54,896) | 0,570 | 0,28 | 0,28 |
| 19 | 46,770 | 45,414 | 0,894 | (43,555; 47,272) | 1,357 | 0,65 | 0,64 |
| 20 | 48,028 | 43,645 | 1,047 | (41,467; 45,823) | 4,383 | 2,17 | 2,40 |
| 21 | 44,425 | 45,062 | 0,490 | (44,042; 46,082) | -0,638 | -0,29 | -0,28 |
| 22 | 50,993 | 45,433 | 0,492 | (44,409; 46,458) | 5,560 | 2,50 | 2,91 |
| 23 | 44,696 | 43,413 | 1,323 | (40,663; 46,164) | 1,283 | 0,69 | 0,68 |
| 24 | 46,531 | 47,212 | 1,226 | (44,662; 49,762) | -0,681 | -0,36 | -0,35 |
| 25 | 42,724 | 45,232 | 0,476 | (44,241; 46,223) | -2,508 | -1,13 | -1,13 |
| 26 | 43,190 | 45,232 | 0,476 | (44,241; 46,223) | -2,042 | -0,92 | -0,91 |
| 27 | 48,512 | 45,232 | 0,476 | (44,241; 46,223) | 3,280 | 1,47 | 1,52 |

| Obs | HI | Cook’s D | DFITS |  |
| --- | --- | --- | --- | --- |
| 1 | 0,238422 | 0,01 | 0,17704 |  |
| 2 | 0,238422 | 0,02 | -0,30515 |  |
| 3 | 0,439577 | 0,20 | -1,12303 |  |
| 4 | 0,439577 | 0,02 | -0,31263 |  |
| 5 | 0,190525 | 0,05 | -0,54157 |  |
| 6 | 0,190525 | 0,00 | -0,09102 |  |
| 7 | 0,328781 | 0,02 | -0,35996 |  |
| 8 | 0,328781 | 0,00 | -0,03558 |  |
| 9 | 0,244482 | 0,00 | 0,10046 |  |
| 10 | 0,244482 | 0,01 | -0,27474 |  |
| 11 | 0,184860 | 0,00 | -0,10319 |  |
| 12 | 0,184860 | 0,00 | -0,09839 |  |
| 13 | 0,298927 | 0,00 | 0,01789 |  |
| 14 | 0,298927 | 0,05 | 0,55038 |  |
| 15 | 0,253021 | 0,00 | 0,11196 |  |
| 16 | 0,253021 | 0,23 | -1,27252 | R |
| 17 | 0,212283 | 0,00 | -0,05298 |  |
| 18 | 0,212283 | 0,00 | 0,14329 |  |
| 19 | 0,154147 | 0,01 | 0,27272 |  |
| 20 | 0,211709 | 0,21 | 1,24500 | R |
| 21 | 0,046409 | 0,00 | -0,06188 |  |
| 22 | 0,046809 | 0,05 | 0,64571 | R |
| 23 | 0,337586 | 0,04 | 0,48798 |  |
| 24 | 0,290228 | 0,01 | -0,22226 |  |
| 25 | 0,043786 | 0,01 | -0,24279 |  |
| 26 | 0,043786 | 0,01 | -0,19556 |  |
| 27 | 0,043786 | 0,02 | 0,32494 |  |

R  Large residual

## Backward Elimination of Terms

α to remove = 0,1

## Coded Coefficients

| Term | Coef | SE Coef | 95% CI | T-Value | P-Value | VIF |
| --- | --- | --- | --- | --- | --- | --- |
| Constant | 47,838 | 0,520 | (46,757; 48,919) | 92,03 | 0,000 |  |
| Lac | 7,016 | 0,976 | (4,985; 9,047) | 7,19 | 0,000 | 1,08 |
| HPMC\_Visc | -0,465 | 0,946 | (-2,433; 1,503) | -0,49 | 0,628 | 1,33 |
| HPMC\_PS | 3,26 | 1,29 | (0,59; 5,93) | 2,54 | 0,019 | 1,34 |
| Lac\*HPMC\_Visc | -4,10 | 1,91 | (-8,08; -0,13) | -2,15 | 0,044 | 1,08 |
| HPMC\_Visc\*HPMC\_PS | 5,42 | 2,55 | (0,11; 10,73) | 2,12 | 0,046 | 1,25 |

## Model Summary

| S | R-sq | R-sq(adj) | PRESS | R-sq(pred) | AICc | BIC |
| --- | --- | --- | --- | --- | --- | --- |
| 2,30558 | 79,64% | 74,79% | 166,158 | 69,70% | 134,84 | 138,02 |

## Analysis of Variance

| Source | DF | Seq SS | Contribution | Adj SS | Adj MS | F-Value | P-Value |
| --- | --- | --- | --- | --- | --- | --- | --- |
| Model | 5 | 436,68 | 79,64% | 436,679 | 87,336 | 16,43 | 0,000 |
| Linear | 3 | 388,17 | 70,79% | 326,252 | 108,751 | 20,46 | 0,000 |
| Lac | 1 | 344,22 | 62,78% | 274,445 | 274,445 | 51,63 | 0,000 |
| HPMC\_Visc | 1 | 25,47 | 4,65% | 1,284 | 1,284 | 0,24 | 0,628 |
| HPMC\_PS | 1 | 18,49 | 3,37% | 34,210 | 34,210 | 6,44 | 0,019 |
| 2-Way Interaction | 2 | 48,51 | 8,85% | 48,506 | 24,253 | 4,56 | 0,023 |
| Lac\*HPMC\_Visc | 1 | 24,53 | 4,47% | 24,528 | 24,528 | 4,61 | 0,044 |
| HPMC\_Visc\*HPMC\_PS | 1 | 23,98 | 4,37% | 23,978 | 23,978 | 4,51 | 0,046 |
| Error | 21 | 111,63 | 20,36% | 111,630 | 5,316 |  |  |
| Lack-of-Fit | 19 | 91,37 | 16,66% | 91,365 | 4,809 | 0,47 | 0,851 |
| Pure Error | 2 | 20,26 | 3,70% | 20,265 | 10,132 |  |  |
| Total | 26 | 548,31 | 100,00% |  |  |  |  |

## Regression Equation in Uncoded Units

|  |  |  |
| --- | --- | --- |
| F\_mean\_5h(300min) | = | 154,3 + 85,1 Lac - 0,01120 HPMC\_Visc - 2,12 HPMC\_PS - 0,00422 Lac\*HPMC\_Visc + 0,000189 HPMC\_Visc\*HPMC\_PS |

## Fits and Diagnostics for All Observations

| Obs | F\_mean\_5h(300min) | Fit | SE Fit | 95% CI | Resid | Std Resid | Del Resid | HI |
| --- | --- | --- | --- | --- | --- | --- | --- | --- |
| 1 | 43,97 | 43,35 | 1,13 | (41,01; 45,69) | 0,63 | 0,31 | 0,30 | 0,238422 |
| 2 | 52,11 | 53,29 | 1,13 | (50,94; 55,63) | -1,18 | -0,58 | -0,58 | 0,238422 |
| 3 | 39,10 | 41,38 | 1,53 | (38,20; 44,55) | -2,27 | -1,32 | -1,34 | 0,439577 |
| 4 | 44,90 | 45,56 | 1,53 | (42,38; 48,74) | -0,66 | -0,38 | -0,37 | 0,439577 |
| 5 | 40,99 | 43,34 | 1,01 | (41,25; 45,44) | -2,36 | -1,14 | -1,14 | 0,190525 |
| 6 | 52,48 | 53,02 | 1,01 | (50,93; 55,12) | -0,55 | -0,26 | -0,26 | 0,190525 |
| 7 | 41,89 | 42,69 | 1,32 | (39,94; 45,44) | -0,80 | -0,42 | -0,41 | 0,328781 |
| 8 | 46,67 | 46,79 | 1,32 | (44,04; 49,54) | -0,11 | -0,06 | -0,06 | 0,328781 |
| 9 | 43,32 | 43,03 | 1,14 | (40,66; 45,40) | 0,29 | 0,15 | 0,14 | 0,244482 |
| 10 | 51,86 | 52,88 | 1,14 | (50,51; 55,26) | -1,03 | -0,51 | -0,50 | 0,244482 |
| 11 | 45,16 | 45,54 | 0,99 | (43,48; 47,60) | -0,38 | -0,18 | -0,18 | 0,184860 |
| 12 | 51,24 | 51,77 | 0,99 | (49,71; 53,83) | -0,54 | -0,26 | -0,25 | 0,184860 |
| 13 | 43,03 | 42,94 | 1,26 | (40,32; 45,56) | 0,09 | 0,05 | 0,04 | 0,298927 |
| 14 | 54,48 | 52,83 | 1,26 | (50,21; 55,45) | 1,65 | 0,85 | 0,85 | 0,298927 |
| 15 | 46,29 | 46,06 | 1,16 | (43,64; 48,47) | 0,23 | 0,12 | 0,11 | 0,253021 |
| 16 | 48,02 | 51,91 | 1,16 | (49,50; 54,33) | -3,89 | -1,95 | -2,11 | 0,253021 |
| 17 | 39,83 | 40,06 | 1,06 | (37,85; 42,27) | -0,23 | -0,11 | -0,11 | 0,212283 |
| 18 | 56,28 | 55,63 | 1,06 | (53,42; 57,84) | 0,64 | 0,31 | 0,31 | 0,212283 |
| 19 | 49,59 | 48,10 | 0,91 | (46,22; 49,98) | 1,49 | 0,70 | 0,69 | 0,154147 |
| 20 | 50,54 | 46,16 | 1,06 | (43,95; 48,36) | 4,38 | 2,14 | 2,36 | 0,211709 |
| 21 | 46,88 | 47,68 | 0,50 | (46,65; 48,72) | -0,80 | -0,35 | -0,35 | 0,046409 |
| 22 | 53,74 | 48,04 | 0,50 | (47,00; 49,07) | 5,70 | 2,53 | 2,97 | 0,046809 |
| 23 | 47,57 | 46,10 | 1,34 | (43,32; 48,89) | 1,46 | 0,78 | 0,77 | 0,337586 |
| 24 | 49,16 | 49,76 | 1,24 | (47,18; 52,35) | -0,60 | -0,31 | -0,30 | 0,290228 |
| 25 | 45,31 | 47,85 | 0,48 | (46,84; 48,85) | -2,53 | -1,12 | -1,13 | 0,043786 |
| 26 | 45,93 | 47,85 | 0,48 | (46,84; 48,85) | -1,92 | -0,85 | -0,84 | 0,043786 |
| 27 | 51,11 | 47,85 | 0,48 | (46,84; 48,85) | 3,26 | 1,45 | 1,49 | 0,043786 |

| Obs | Cook’s D | DFITS |  |
| --- | --- | --- | --- |
| 1 | 0,01 | 0,17018 |  |
| 2 | 0,02 | -0,32198 |  |
| 3 | 0,23 | -1,18772 |  |
| 4 | 0,02 | -0,33193 |  |
| 5 | 0,05 | -0,55528 |  |
| 6 | 0,00 | -0,12522 |  |
| 7 | 0,01 | -0,28880 |  |
| 8 | 0,00 | -0,04158 |  |
| 9 | 0,00 | 0,08091 |  |
| 10 | 0,01 | -0,28583 |  |
| 11 | 0,00 | -0,08460 |  |
| 12 | 0,00 | -0,11971 |  |
| 13 | 0,00 | 0,02873 |  |
| 14 | 0,05 | 0,55368 |  |
| 15 | 0,00 | 0,06684 |  |
| 16 | 0,22 | -1,22562 |  |
| 17 | 0,00 | -0,05634 |  |
| 18 | 0,00 | 0,15935 |  |
| 19 | 0,01 | 0,29560 |  |
| 20 | 0,21 | 1,22405 | R |
| 21 | 0,00 | -0,07654 |  |
| 22 | 0,05 | 0,65777 | R |
| 23 | 0,05 | 0,55123 |  |
| 24 | 0,01 | -0,19278 |  |
| 25 | 0,01 | -0,24201 |  |
| 26 | 0,01 | -0,18064 |  |
| 27 | 0,02 | 0,31852 |  |

R  Large residual

## Backward Elimination of Terms

α to remove = 0,1

## Coded Coefficients

| Term | Coef | SE Coef | 95% CI | T-Value | P-Value | VIF |
| --- | --- | --- | --- | --- | --- | --- |
| Constant | 50,348 | 0,523 | (49,261; 51,436) | 96,29 | 0,000 |  |
| Lac | 7,273 | 0,982 | (5,231; 9,316) | 7,41 | 0,000 | 1,08 |
| HPMC\_Visc | -0,564 | 0,952 | (-2,544; 1,415) | -0,59 | 0,560 | 1,33 |
| HPMC\_PS | 3,21 | 1,29 | (0,52; 5,90) | 2,48 | 0,022 | 1,34 |
| Lac\*HPMC\_Visc | -4,17 | 1,92 | (-8,17; -0,17) | -2,17 | 0,042 | 1,08 |
| HPMC\_Visc\*HPMC\_PS | 5,50 | 2,57 | (0,16; 10,84) | 2,14 | 0,044 | 1,25 |

## Model Summary

| S | R-sq | R-sq(adj) | PRESS | R-sq(pred) | AICc | BIC |
| --- | --- | --- | --- | --- | --- | --- |
| 2,31914 | 80,44% | 75,78% | 168,224 | 70,86% | 135,16 | 138,33 |

## Analysis of Variance

| Source | DF | Seq SS | Contribution | Adj SS | Adj MS | F-Value | P-Value |
| --- | --- | --- | --- | --- | --- | --- | --- |
| Model | 5 | 464,40 | 80,44% | 464,400 | 92,880 | 17,27 | 0,000 |
| Linear | 3 | 414,35 | 71,77% | 347,536 | 115,845 | 21,54 | 0,000 |
| Lac | 1 | 368,86 | 63,89% | 294,947 | 294,947 | 54,84 | 0,000 |
| HPMC\_Visc | 1 | 28,15 | 4,88% | 1,891 | 1,891 | 0,35 | 0,560 |
| HPMC\_PS | 1 | 17,33 | 3,00% | 33,079 | 33,079 | 6,15 | 0,022 |
| 2-Way Interaction | 2 | 50,05 | 8,67% | 50,049 | 25,024 | 4,65 | 0,021 |
| Lac\*HPMC\_Visc | 1 | 25,34 | 4,39% | 25,339 | 25,339 | 4,71 | 0,042 |
| HPMC\_Visc\*HPMC\_PS | 1 | 24,71 | 4,28% | 24,710 | 24,710 | 4,59 | 0,044 |
| Error | 21 | 112,95 | 19,56% | 112,947 | 5,378 |  |  |
| Lack-of-Fit | 19 | 93,15 | 16,13% | 93,149 | 4,903 | 0,50 | 0,840 |
| Pure Error | 2 | 19,80 | 3,43% | 19,797 | 9,899 |  |  |
| Total | 26 | 577,35 | 100,00% |  |  |  |  |

## Regression Equation in Uncoded Units

|  |  |  |
| --- | --- | --- |
| F\_mean\_5.5h(330min) | = | 159,4 + 87,0 Lac - 0,01140 HPMC\_Visc - 2,16 HPMC\_PS - 0,00429 Lac\*HPMC\_Visc + 0,000192 HPMC\_Visc\*HPMC\_PS |

## Fits and Diagnostics for All Observations

| Obs | F\_mean\_5.5h(330min) | Fit | SE Fit | 95% CI | Resid | Std Resid | Del Resid |
| --- | --- | --- | --- | --- | --- | --- | --- |
| 1 | 46,43 | 45,80 | 1,13 | (43,45; 48,16) | 0,62 | 0,31 | 0,30 |
| 2 | 54,76 | 56,05 | 1,13 | (53,69; 58,40) | -1,29 | -0,64 | -0,63 |
| 3 | 41,35 | 43,71 | 1,54 | (40,51; 46,91) | -2,36 | -1,36 | -1,39 |
| 4 | 47,45 | 48,11 | 1,54 | (44,91; 51,31) | -0,65 | -0,38 | -0,37 |
| 5 | 43,40 | 45,79 | 1,01 | (43,68; 47,89) | -2,39 | -1,14 | -1,15 |
| 6 | 55,11 | 55,77 | 1,01 | (53,66; 57,87) | -0,66 | -0,32 | -0,31 |
| 7 | 44,34 | 45,02 | 1,33 | (42,25; 47,79) | -0,68 | -0,36 | -0,35 |
| 8 | 49,21 | 49,33 | 1,33 | (46,57; 52,10) | -0,13 | -0,07 | -0,06 |
| 9 | 45,59 | 45,41 | 1,15 | (43,02; 47,79) | 0,18 | 0,09 | 0,09 |
| 10 | 54,54 | 55,57 | 1,15 | (53,19; 57,95) | -1,03 | -0,51 | -0,50 |
| 11 | 47,60 | 47,90 | 1,00 | (45,83; 49,97) | -0,29 | -0,14 | -0,14 |
| 12 | 53,70 | 54,38 | 1,00 | (52,30; 56,45) | -0,68 | -0,32 | -0,32 |
| 13 | 45,42 | 45,31 | 1,27 | (42,67; 47,94) | 0,11 | 0,06 | 0,06 |
| 14 | 57,19 | 55,50 | 1,27 | (52,87; 58,14) | 1,68 | 0,87 | 0,86 |
| 15 | 48,59 | 48,41 | 1,17 | (45,99; 50,84) | 0,18 | 0,09 | 0,09 |
| 16 | 50,74 | 54,51 | 1,17 | (52,08; 56,93) | -3,77 | -1,88 | -2,01 |
| 17 | 42,07 | 42,32 | 1,07 | (40,10; 44,54) | -0,25 | -0,12 | -0,12 |
| 18 | 59,14 | 58,44 | 1,07 | (56,21; 60,66) | 0,70 | 0,34 | 0,33 |
| 19 | 52,30 | 50,70 | 0,91 | (48,81; 52,59) | 1,60 | 0,75 | 0,74 |
| 20 | 52,87 | 48,56 | 1,07 | (46,35; 50,78) | 4,30 | 2,09 | 2,29 |
| 21 | 49,29 | 50,22 | 0,50 | (49,18; 51,26) | -0,93 | -0,41 | -0,40 |
| 22 | 56,38 | 50,55 | 0,50 | (49,51; 51,60) | 5,83 | 2,57 | 3,04 |
| 23 | 50,31 | 48,72 | 1,35 | (45,91; 51,52) | 1,59 | 0,84 | 0,84 |
| 24 | 51,67 | 52,22 | 1,25 | (49,62; 54,82) | -0,55 | -0,28 | -0,28 |
| 25 | 47,85 | 50,38 | 0,49 | (49,37; 51,39) | -2,53 | -1,11 | -1,12 |
| 26 | 48,52 | 50,38 | 0,49 | (49,37; 51,39) | -1,86 | -0,82 | -0,81 |
| 27 | 53,60 | 50,38 | 0,49 | (49,37; 51,39) | 3,23 | 1,42 | 1,46 |

| Obs | HI | Cook’s D | DFITS |  |
| --- | --- | --- | --- | --- |
| 1 | 0,238422 | 0,00 | 0,16895 |  |
| 2 | 0,238422 | 0,02 | -0,35152 |  |
| 3 | 0,439577 | 0,24 | -1,22777 |  |
| 4 | 0,439577 | 0,02 | -0,32710 |  |
| 5 | 0,190525 | 0,05 | -0,55983 |  |
| 6 | 0,190525 | 0,00 | -0,14995 |  |
| 7 | 0,328781 | 0,01 | -0,24536 |  |
| 8 | 0,328781 | 0,00 | -0,04523 |  |
| 9 | 0,244482 | 0,00 | 0,05059 |  |
| 10 | 0,244482 | 0,01 | -0,28541 |  |
| 11 | 0,184860 | 0,00 | -0,06539 |  |
| 12 | 0,184860 | 0,00 | -0,15036 |  |
| 13 | 0,298927 | 0,00 | 0,03610 |  |
| 14 | 0,298927 | 0,05 | 0,56190 |  |
| 15 | 0,253021 | 0,00 | 0,05173 |  |
| 16 | 0,253021 | 0,20 | -1,17114 |  |
| 17 | 0,212283 | 0,00 | -0,06129 |  |
| 18 | 0,212283 | 0,01 | 0,17363 |  |
| 19 | 0,154147 | 0,02 | 0,31747 |  |
| 20 | 0,211709 | 0,20 | 1,18811 | R |
| 21 | 0,046409 | 0,00 | -0,08859 |  |
| 22 | 0,046809 | 0,05 | 0,67281 | R |
| 23 | 0,337586 | 0,06 | 0,59851 |  |
| 24 | 0,290228 | 0,01 | -0,17741 |  |
| 25 | 0,043786 | 0,01 | -0,23994 |  |
| 26 | 0,043786 | 0,01 | -0,17391 |  |
| 27 | 0,043786 | 0,02 | 0,31248 |  |

R  Large residual

## Backward Elimination of Terms

α to remove = 0,1

## Coded Coefficients

| Term | Coef | SE Coef | 95% CI | T-Value | P-Value | VIF |
| --- | --- | --- | --- | --- | --- | --- |
| Constant | 52,793 | 0,527 | (51,697; 53,889) | 100,18 | 0,000 |  |
| Lac | 7,507 | 0,990 | (5,448; 9,566) | 7,58 | 0,000 | 1,08 |
| HPMC\_Visc | -0,631 | 0,959 | (-2,626; 1,364) | -0,66 | 0,518 | 1,33 |
| HPMC\_PS | 3,19 | 1,30 | (0,48; 5,90) | 2,45 | 0,023 | 1,34 |
| Lac\*HPMC\_Visc | -4,19 | 1,94 | (-8,22; -0,16) | -2,16 | 0,042 | 1,08 |
| HPMC\_Visc\*HPMC\_PS | 5,65 | 2,59 | (0,27; 11,03) | 2,18 | 0,040 | 1,25 |

## Model Summary

| S | R-sq | R-sq(adj) | PRESS | R-sq(pred) | AICc | BIC |
| --- | --- | --- | --- | --- | --- | --- |
| 2,33731 | 81,04% | 76,52% | 171,278 | 71,69% | 135,58 | 138,75 |

## Analysis of Variance

| Source | DF | Seq SS | Contribution | Adj SS | Adj MS | F-Value | P-Value |
| --- | --- | --- | --- | --- | --- | --- | --- |
| Model | 5 | 490,21 | 81,04% | 490,208 | 98,042 | 17,95 | 0,000 |
| Linear | 3 | 438,55 | 72,50% | 368,018 | 122,673 | 22,46 | 0,000 |
| Lac | 1 | 391,44 | 64,71% | 314,185 | 314,185 | 57,51 | 0,000 |
| HPMC\_Visc | 1 | 30,45 | 5,03% | 2,360 | 2,360 | 0,43 | 0,518 |
| HPMC\_PS | 1 | 16,67 | 2,76% | 32,809 | 32,809 | 6,01 | 0,023 |
| 2-Way Interaction | 2 | 51,65 | 8,54% | 51,655 | 25,827 | 4,73 | 0,020 |
| Lac\*HPMC\_Visc | 1 | 25,60 | 4,23% | 25,597 | 25,597 | 4,69 | 0,042 |
| HPMC\_Visc\*HPMC\_PS | 1 | 26,06 | 4,31% | 26,058 | 26,058 | 4,77 | 0,040 |
| Error | 21 | 114,72 | 18,96% | 114,724 | 5,463 |  |  |
| Lack-of-Fit | 19 | 95,55 | 15,80% | 95,553 | 5,029 | 0,52 | 0,824 |
| Pure Error | 2 | 19,17 | 3,17% | 19,170 | 9,585 |  |  |
| Total | 26 | 604,93 | 100,00% |  |  |  |  |

## Regression Equation in Uncoded Units

|  |  |  |
| --- | --- | --- |
| F\_mean\_6h(360min) | = | 166,4 + 88,3 Lac - 0,01176 HPMC\_Visc - 2,23 HPMC\_PS - 0,00431 Lac\*HPMC\_Visc + 0,000197 HPMC\_Visc\*HPMC\_PS |

## Fits and Diagnostics for All Observations

| Obs | F\_mean\_6h(360min) | Fit | SE Fit | 95% CI | Resid | Std Resid | Del Resid | HI |
| --- | --- | --- | --- | --- | --- | --- | --- | --- |
| 1 | 48,90 | 48,20 | 1,14 | (45,83; 50,57) | 0,69 | 0,34 | 0,33 | 0,238422 |
| 2 | 57,28 | 58,69 | 1,14 | (56,32; 61,07) | -1,42 | -0,69 | -0,69 | 0,238422 |
| 3 | 43,50 | 45,95 | 1,55 | (42,73; 49,17) | -2,45 | -1,40 | -1,43 | 0,439577 |
| 4 | 49,90 | 50,57 | 1,55 | (47,34; 53,79) | -0,66 | -0,38 | -0,37 | 0,439577 |
| 5 | 45,73 | 48,17 | 1,02 | (46,05; 50,29) | -2,44 | -1,16 | -1,17 | 0,190525 |
| 6 | 57,66 | 58,40 | 1,02 | (56,28; 60,52) | -0,73 | -0,35 | -0,34 | 0,190525 |
| 7 | 46,73 | 47,27 | 1,34 | (44,49; 50,06) | -0,55 | -0,29 | -0,28 | 0,328781 |
| 8 | 51,68 | 51,80 | 1,34 | (49,02; 54,59) | -0,12 | -0,06 | -0,06 | 0,328781 |
| 9 | 47,79 | 47,73 | 1,16 | (45,32; 50,13) | 0,06 | 0,03 | 0,03 | 0,244482 |
| 10 | 57,12 | 58,14 | 1,16 | (55,73; 60,54) | -1,02 | -0,50 | -0,49 | 0,244482 |
| 11 | 50,00 | 50,22 | 1,00 | (48,13; 52,31) | -0,22 | -0,11 | -0,10 | 0,184860 |
| 12 | 56,14 | 56,93 | 1,00 | (54,84; 59,02) | -0,79 | -0,37 | -0,37 | 0,184860 |
| 13 | 47,76 | 47,61 | 1,28 | (44,96; 50,27) | 0,14 | 0,07 | 0,07 | 0,298927 |
| 14 | 59,76 | 58,06 | 1,28 | (55,40; 60,72) | 1,71 | 0,87 | 0,87 | 0,298927 |
| 15 | 50,89 | 50,73 | 1,18 | (48,29; 53,18) | 0,16 | 0,08 | 0,08 | 0,253021 |
| 16 | 53,40 | 57,05 | 1,18 | (54,61; 59,50) | -3,66 | -1,81 | -1,92 | 0,253021 |
| 17 | 44,23 | 44,54 | 1,08 | (42,30; 46,78) | -0,31 | -0,15 | -0,15 | 0,212283 |
| 18 | 61,87 | 61,13 | 1,08 | (58,89; 63,37) | 0,74 | 0,36 | 0,35 | 0,212283 |
| 19 | 54,96 | 53,19 | 0,92 | (51,29; 55,10) | 1,77 | 0,82 | 0,81 | 0,154147 |
| 20 | 55,19 | 50,92 | 1,08 | (48,69; 53,16) | 4,27 | 2,06 | 2,25 | 0,211709 |
| 21 | 51,57 | 52,68 | 0,50 | (51,63; 53,73) | -1,11 | -0,49 | -0,48 | 0,046409 |
| 22 | 58,92 | 53,00 | 0,51 | (51,95; 54,06) | 5,92 | 2,59 | 3,07 | 0,046809 |
| 23 | 52,92 | 51,23 | 1,36 | (48,41; 54,05) | 1,69 | 0,89 | 0,89 | 0,337586 |
| 24 | 54,10 | 54,63 | 1,26 | (52,01; 57,25) | -0,54 | -0,27 | -0,27 | 0,290228 |
| 25 | 50,29 | 52,84 | 0,49 | (51,82; 53,85) | -2,55 | -1,12 | -1,12 | 0,043786 |
| 26 | 51,08 | 52,84 | 0,49 | (51,82; 53,85) | -1,75 | -0,77 | -0,76 | 0,043786 |
| 27 | 56,00 | 52,84 | 0,49 | (51,82; 53,85) | 3,16 | 1,38 | 1,42 | 0,043786 |

| Obs | Cook’s D | DFITS |  |
| --- | --- | --- | --- |
| 1 | 0,01 | 0,18650 |  |
| 2 | 0,03 | -0,38348 |  |
| 3 | 0,26 | -1,26816 |  |
| 4 | 0,02 | -0,32920 |  |
| 5 | 0,05 | -0,56738 |  |
| 6 | 0,00 | -0,16576 |  |
| 7 | 0,01 | -0,19533 |  |
| 8 | 0,00 | -0,04298 |  |
| 9 | 0,00 | 0,01652 |  |
| 10 | 0,01 | -0,28019 |  |
| 11 | 0,00 | -0,04915 |  |
| 12 | 0,01 | -0,17435 |  |
| 13 | 0,00 | 0,04710 |  |
| 14 | 0,05 | 0,56556 |  |
| 15 | 0,00 | 0,04525 |  |
| 16 | 0,19 | -1,11937 |  |
| 17 | 0,00 | -0,07555 |  |
| 18 | 0,01 | 0,18183 |  |
| 19 | 0,02 | 0,34789 |  |
| 20 | 0,19 | 1,16427 | R |
| 21 | 0,00 | -0,10544 |  |
| 22 | 0,05 | 0,67981 | R |
| 23 | 0,07 | 0,63214 |  |
| 24 | 0,01 | -0,17020 |  |
| 25 | 0,01 | -0,24044 |  |
| 26 | 0,00 | -0,16257 |  |
| 27 | 0,01 | 0,30332 |  |

R  Large residual

## Backward Elimination of Terms

α to remove = 0,1

## Coded Coefficients

| Term | Coef | SE Coef | 95% CI | T-Value | P-Value | VIF |
| --- | --- | --- | --- | --- | --- | --- |
| Constant | 57,558 | 0,509 | (56,496; 58,619) | 113,06 | 0,000 |  |
| Lac | 7,985 | 0,940 | (6,024; 9,947) | 8,49 | 0,000 | 1,08 |
| HPMC\_Visc | -0,970 | 0,917 | (-2,882; 0,943) | -1,06 | 0,303 | 1,35 |
| HPMC\_HP | 1,858 | 0,910 | (-0,041; 3,757) | 2,04 | 0,055 | 1,03 |
| HPMC\_PS | 2,65 | 1,25 | (0,04; 5,26) | 2,11 | 0,047 | 1,37 |
| Lac\*HPMC\_Visc | -4,44 | 1,84 | (-8,28; -0,60) | -2,41 | 0,026 | 1,08 |
| HPMC\_Visc\*HPMC\_PS | 5,12 | 2,46 | (-0,02; 10,26) | 2,08 | 0,051 | 1,25 |

## Model Summary

| S | R-sq | R-sq(adj) | PRESS | R-sq(pred) | AICc | BIC |
| --- | --- | --- | --- | --- | --- | --- |
| 2,22067 | 85,11% | 80,64% | 168,760 | 74,52% | 135,60 | 137,97 |

## Analysis of Variance

| Source | DF | Seq SS | Contribution | Adj SS | Adj MS | F-Value | P-Value |
| --- | --- | --- | --- | --- | --- | --- | --- |
| Model | 6 | 563,780 | 85,11% | 563,780 | 93,963 | 19,05 | 0,000 |
| Linear | 4 | 513,762 | 77,56% | 429,072 | 107,268 | 21,75 | 0,000 |
| Lac | 1 | 442,639 | 66,82% | 355,518 | 355,518 | 72,09 | 0,000 |
| HPMC\_Visc | 1 | 32,892 | 4,97% | 5,518 | 5,518 | 1,12 | 0,303 |
| HPMC\_HP | 1 | 28,297 | 4,27% | 20,537 | 20,537 | 4,16 | 0,055 |
| HPMC\_PS | 1 | 9,934 | 1,50% | 22,045 | 22,045 | 4,47 | 0,047 |
| 2-Way Interaction | 2 | 50,018 | 7,55% | 50,018 | 25,009 | 5,07 | 0,017 |
| Lac\*HPMC\_Visc | 1 | 28,693 | 4,33% | 28,693 | 28,693 | 5,82 | 0,026 |
| HPMC\_Visc\*HPMC\_PS | 1 | 21,325 | 3,22% | 21,325 | 21,325 | 4,32 | 0,051 |
| Error | 20 | 98,628 | 14,89% | 98,628 | 4,931 |  |  |
| Lack-of-Fit | 18 | 80,091 | 12,09% | 80,091 | 4,449 | 0,48 | 0,846 |
| Pure Error | 2 | 18,537 | 2,80% | 18,537 | 9,268 |  |  |
| Total | 26 | 662,407 | 100,00% |  |  |  |  |

## Regression Equation in Uncoded Units

|  |  |  |
| --- | --- | --- |
| F\_mean\_7h(420min) | = | 140,1 + 93,6 Lac - 0,01043 HPMC\_Visc + 1,830 HPMC\_HP - 2,06 HPMC\_PS - 0,00456 Lac\*HPMC\_Visc + 0,000179 HPMC\_Visc\*HPMC\_PS |

## Fits and Diagnostics for All Observations

| Obs | F\_mean\_7h(420min) | Fit | SE Fit | 95% CI | Resid | Std Resid | Del Resid | HI |
| --- | --- | --- | --- | --- | --- | --- | --- | --- |
| 1 | 52,67 | 51,58 | 1,18 | (49,12; 54,05) | 1,09 | 0,58 | 0,57 | 0,283627 |
| 2 | 62,10 | 62,73 | 1,18 | (60,26; 65,19) | -0,62 | -0,33 | -0,32 | 0,283627 |
| 3 | 47,72 | 49,45 | 1,55 | (46,22; 52,69) | -1,74 | -1,09 | -1,10 | 0,488210 |
| 4 | 54,58 | 54,38 | 1,55 | (51,14; 57,62) | 0,20 | 0,13 | 0,13 | 0,488210 |
| 5 | 50,14 | 53,25 | 1,03 | (51,09; 55,41) | -3,11 | -1,58 | -1,65 | 0,216779 |
| 6 | 62,57 | 64,12 | 1,03 | (61,96; 66,27) | -1,55 | -0,79 | -0,78 | 0,216779 |
| 7 | 51,53 | 52,88 | 1,40 | (49,97; 55,79) | -1,36 | -0,79 | -0,78 | 0,394639 |
| 8 | 56,49 | 57,72 | 1,40 | (54,81; 60,63) | -1,22 | -0,71 | -0,70 | 0,394639 |
| 9 | 51,99 | 51,07 | 1,20 | (48,55; 53,58) | 0,93 | 0,50 | 0,49 | 0,294244 |
| 10 | 62,01 | 62,12 | 1,20 | (59,61; 64,64) | -0,11 | -0,06 | -0,06 | 0,294244 |
| 11 | 54,52 | 53,68 | 1,04 | (51,52; 55,85) | 0,84 | 0,43 | 0,42 | 0,218366 |
| 12 | 60,68 | 60,82 | 1,04 | (58,66; 62,99) | -0,14 | -0,07 | -0,07 | 0,218366 |
| 13 | 52,28 | 52,96 | 1,31 | (50,23; 55,69) | -0,68 | -0,38 | -0,37 | 0,347162 |
| 14 | 64,73 | 64,06 | 1,31 | (61,33; 66,78) | 0,68 | 0,38 | 0,37 | 0,347162 |
| 15 | 54,73 | 55,33 | 1,13 | (52,98; 57,68) | -0,60 | -0,31 | -0,31 | 0,258117 |
| 16 | 58,35 | 62,06 | 1,13 | (59,71; 64,42) | -3,71 | -1,94 | -2,10 | 0,258117 |
| 17 | 48,46 | 48,76 | 1,03 | (46,62; 50,90) | -0,30 | -0,15 | -0,15 | 0,213198 |
| 18 | 67,03 | 66,40 | 1,03 | (64,26; 68,54) | 0,63 | 0,32 | 0,32 | 0,213198 |
| 19 | 59,91 | 58,16 | 0,88 | (56,32; 60,00) | 1,75 | 0,86 | 0,85 | 0,157071 |
| 20 | 59,53 | 55,18 | 1,03 | (53,03; 57,32) | 4,35 | 2,21 | 2,48 | 0,214445 |
| 21 | 55,96 | 55,69 | 0,92 | (53,78; 57,61) | 0,27 | 0,13 | 0,13 | 0,170686 |
| 22 | 63,83 | 59,64 | 1,11 | (57,32; 61,96) | 4,19 | 2,18 | 2,44 | 0,251394 |
| 23 | 57,98 | 56,35 | 1,31 | (53,63; 59,08) | 1,63 | 0,91 | 0,90 | 0,346058 |
| 24 | 58,69 | 58,77 | 1,21 | (56,24; 61,29) | -0,07 | -0,04 | -0,04 | 0,297564 |
| 25 | 54,89 | 57,58 | 0,47 | (56,60; 58,56) | -2,69 | -1,24 | -1,26 | 0,044701 |
| 26 | 55,90 | 57,58 | 0,47 | (56,60; 58,56) | -1,68 | -0,77 | -0,77 | 0,044701 |
| 27 | 60,60 | 57,58 | 0,47 | (56,60; 58,56) | 3,02 | 1,39 | 1,43 | 0,044701 |

| Obs | Cook’s D | DFITS |  |
| --- | --- | --- | --- |
| 1 | 0,02 | 0,35851 |  |
| 2 | 0,01 | -0,20432 |  |
| 3 | 0,16 | -1,07394 |  |
| 4 | 0,00 | 0,12239 |  |
| 5 | 0,10 | -0,86718 |  |
| 6 | 0,02 | -0,41102 |  |
| 7 | 0,06 | -0,62834 |  |
| 8 | 0,05 | -0,56509 |  |
| 9 | 0,01 | 0,31555 |  |
| 10 | 0,00 | -0,03789 |  |
| 11 | 0,01 | 0,22093 |  |
| 12 | 0,00 | -0,03793 |  |
| 13 | 0,01 | -0,26854 |  |
| 14 | 0,01 | 0,26843 |  |
| 15 | 0,00 | -0,18017 |  |
| 16 | 0,19 | -1,23776 |  |
| 17 | 0,00 | -0,07694 |  |
| 18 | 0,00 | 0,16398 |  |
| 19 | 0,02 | 0,36891 |  |
| 20 | 0,19 | 1,29421 | R |
| 21 | 0,00 | 0,05826 |  |
| 22 | 0,23 | 1,41176 | R |
| 23 | 0,06 | 0,65801 |  |
| 24 | 0,00 | -0,02455 |  |
| 25 | 0,01 | -0,27175 |  |
| 26 | 0,00 | -0,16549 |  |
| 27 | 0,01 | 0,30834 |  |

R  Large residual

## Backward Elimination of Terms

α to remove = 0,1

## Coded Coefficients

| Term | Coef | SE Coef | 95% CI | T-Value | P-Value | VIF |
| --- | --- | --- | --- | --- | --- | --- |
| Constant | 61,934 | 0,510 | (60,870; 62,997) | 121,48 | 0,000 |  |
| Lac | 8,328 | 0,942 | (6,364; 10,293) | 8,84 | 0,000 | 1,08 |
| HPMC\_Visc | -1,110 | 0,918 | (-3,026; 0,805) | -1,21 | 0,241 | 1,35 |
| HPMC\_HP | 2,047 | 0,912 | (0,146; 3,949) | 2,25 | 0,036 | 1,03 |
| HPMC\_PS | 2,58 | 1,25 | (-0,04; 5,19) | 2,05 | 0,053 | 1,37 |
| Lac\*HPMC\_Visc | -4,45 | 1,84 | (-8,30; -0,61) | -2,42 | 0,025 | 1,08 |
| HPMC\_Visc\*HPMC\_PS | 5,34 | 2,47 | (0,19; 10,48) | 2,16 | 0,043 | 1,25 |

## Model Summary

| S | R-sq | R-sq(adj) | PRESS | R-sq(pred) | AICc | BIC |
| --- | --- | --- | --- | --- | --- | --- |
| 2,22382 | 86,05% | 81,87% | 170,263 | 75,99% | 135,68 | 138,04 |

## Analysis of Variance

| Source | DF | Seq SS | Contribution | Adj SS | Adj MS | F-Value | P-Value |
| --- | --- | --- | --- | --- | --- | --- | --- |
| Model | 6 | 610,126 | 86,05% | 610,126 | 101,688 | 20,56 | 0,000 |
| Linear | 4 | 558,040 | 78,70% | 466,922 | 116,731 | 23,60 | 0,000 |
| Lac | 1 | 478,903 | 67,54% | 386,681 | 386,681 | 78,19 | 0,000 |
| HPMC\_Visc | 1 | 37,307 | 5,26% | 7,234 | 7,234 | 1,46 | 0,241 |
| HPMC\_HP | 1 | 33,265 | 4,69% | 24,945 | 24,945 | 5,04 | 0,036 |
| HPMC\_PS | 1 | 8,565 | 1,21% | 20,827 | 20,827 | 4,21 | 0,053 |
| 2-Way Interaction | 2 | 52,086 | 7,35% | 52,086 | 26,043 | 5,27 | 0,015 |
| Lac\*HPMC\_Visc | 1 | 28,910 | 4,08% | 28,910 | 28,910 | 5,85 | 0,025 |
| HPMC\_Visc\*HPMC\_PS | 1 | 23,176 | 3,27% | 23,176 | 23,176 | 4,69 | 0,043 |
| Error | 20 | 98,907 | 13,95% | 98,907 | 4,945 |  |  |
| Lack-of-Fit | 18 | 81,689 | 11,52% | 81,689 | 4,538 | 0,53 | 0,821 |
| Pure Error | 2 | 17,218 | 2,43% | 17,218 | 8,609 |  |  |
| Total | 26 | 709,033 | 100,00% |  |  |  |  |

## Regression Equation in Uncoded Units

|  |  |  |
| --- | --- | --- |
| F\_mean\_8h(480min) | = | 150,3 + 95,2 Lac - 0,01099 HPMC\_Visc + 2,017 HPMC\_HP - 2,17 HPMC\_PS - 0,00458 Lac\*HPMC\_Visc + 0,000187 HPMC\_Visc\*HPMC\_PS |

## Fits and Diagnostics for All Observations

| Obs | F\_mean\_8h(480min) | Fit | SE Fit | 95% CI | Resid | Std Resid | Del Resid | HI |
| --- | --- | --- | --- | --- | --- | --- | --- | --- |
| 1 | 56,98 | 55,80 | 1,18 | (53,33; 58,27) | 1,17 | 0,62 | 0,61 | 0,283627 |
| 2 | 66,57 | 67,30 | 1,18 | (64,83; 69,77) | -0,73 | -0,39 | -0,38 | 0,283627 |
| 3 | 51,65 | 53,38 | 1,55 | (50,14; 56,62) | -1,74 | -1,09 | -1,10 | 0,488210 |
| 4 | 58,93 | 58,64 | 1,55 | (55,40; 61,88) | 0,29 | 0,18 | 0,18 | 0,488210 |
| 5 | 54,34 | 57,62 | 1,04 | (55,46; 59,78) | -3,27 | -1,66 | -1,75 | 0,216779 |
| 6 | 67,11 | 68,84 | 1,04 | (66,68; 71,00) | -1,72 | -0,88 | -0,87 | 0,216779 |
| 7 | 55,61 | 57,05 | 1,40 | (54,14; 59,97) | -1,45 | -0,84 | -0,83 | 0,394639 |
| 8 | 61,03 | 62,22 | 1,40 | (59,30; 65,13) | -1,18 | -0,68 | -0,68 | 0,394639 |
| 9 | 55,84 | 55,14 | 1,21 | (52,62; 57,66) | 0,70 | 0,38 | 0,37 | 0,294244 |
| 10 | 66,54 | 66,55 | 1,21 | (64,03; 69,07) | -0,01 | -0,00 | -0,00 | 0,294244 |
| 11 | 58,96 | 57,77 | 1,04 | (55,60; 59,93) | 1,19 | 0,61 | 0,60 | 0,218366 |
| 12 | 64,88 | 65,24 | 1,04 | (63,08; 67,41) | -0,36 | -0,18 | -0,18 | 0,218366 |
| 13 | 56,55 | 57,22 | 1,31 | (54,48; 59,95) | -0,66 | -0,37 | -0,36 | 0,347162 |
| 14 | 69,33 | 68,67 | 1,31 | (65,93; 71,40) | 0,66 | 0,37 | 0,36 | 0,347162 |
| 15 | 58,88 | 59,53 | 1,13 | (57,17; 61,88) | -0,65 | -0,34 | -0,33 | 0,258117 |
| 16 | 62,99 | 66,60 | 1,13 | (64,24; 68,95) | -3,61 | -1,88 | -2,02 | 0,258117 |
| 17 | 52,52 | 52,81 | 1,03 | (50,67; 54,95) | -0,29 | -0,15 | -0,14 | 0,213198 |
| 18 | 71,83 | 71,14 | 1,03 | (69,00; 73,28) | 0,69 | 0,35 | 0,34 | 0,213198 |
| 19 | 64,58 | 62,64 | 0,88 | (60,80; 64,47) | 1,94 | 0,95 | 0,95 | 0,157071 |
| 20 | 63,57 | 59,36 | 1,03 | (57,21; 61,51) | 4,21 | 2,14 | 2,37 | 0,214445 |
| 21 | 60,02 | 59,92 | 0,92 | (58,00; 61,83) | 0,10 | 0,05 | 0,05 | 0,170686 |
| 22 | 68,38 | 64,22 | 1,12 | (61,89; 66,54) | 4,16 | 2,16 | 2,41 | 0,251394 |
| 23 | 62,68 | 60,88 | 1,31 | (58,15; 63,61) | 1,80 | 1,00 | 1,00 | 0,346058 |
| 24 | 63,03 | 63,01 | 1,21 | (60,48; 65,55) | 0,02 | 0,01 | 0,01 | 0,297564 |
| 25 | 59,29 | 61,98 | 0,47 | (61,00; 62,96) | -2,69 | -1,24 | -1,26 | 0,044701 |
| 26 | 60,51 | 61,98 | 0,47 | (61,00; 62,96) | -1,46 | -0,67 | -0,66 | 0,044701 |
| 27 | 64,87 | 61,98 | 0,47 | (61,00; 62,96) | 2,89 | 1,33 | 1,36 | 0,044701 |

| Obs | Cook’s D | DFITS |  |
| --- | --- | --- | --- |
| 1 | 0,02 | 0,38616 |  |
| 2 | 0,01 | -0,24023 |  |
| 3 | 0,16 | -1,07143 |  |
| 4 | 0,00 | 0,17361 |  |
| 5 | 0,11 | -0,91870 |  |
| 6 | 0,03 | -0,45833 |  |
| 7 | 0,07 | -0,67072 |  |
| 8 | 0,04 | -0,54505 |  |
| 9 | 0,01 | 0,23777 |  |
| 10 | 0,00 | -0,00314 |  |
| 11 | 0,01 | 0,31534 |  |
| 12 | 0,00 | -0,09475 |  |
| 13 | 0,01 | -0,26364 |  |
| 14 | 0,01 | 0,26384 |  |
| 15 | 0,01 | -0,19525 |  |
| 16 | 0,18 | -1,19336 |  |
| 17 | 0,00 | -0,07448 |  |
| 18 | 0,00 | 0,17873 |  |
| 19 | 0,02 | 0,40906 |  |
| 20 | 0,18 | 1,23736 | R |
| 21 | 0,00 | 0,02154 |  |
| 22 | 0,22 | 1,39601 | R |
| 23 | 0,08 | 0,72682 |  |
| 24 | 0,00 | 0,00658 |  |
| 25 | 0,01 | -0,27161 |  |
| 26 | 0,00 | -0,14351 |  |
| 27 | 0,01 | 0,29394 |  |

R  Large residual

## Backward Elimination of Terms

α to remove = 0,1

## Coded Coefficients

| Term | Coef | SE Coef | 95% CI | T-Value | P-Value | VIF |
| --- | --- | --- | --- | --- | --- | --- |
| Constant | 66,089 | 0,522 | (64,999; 67,179) | 126,49 | 0,000 |  |
| Lac | 8,670 | 0,965 | (6,657; 10,684) | 8,98 | 0,000 | 1,08 |
| HPMC\_Visc | -1,268 | 0,941 | (-3,230; 0,695) | -1,35 | 0,193 | 1,35 |
| HPMC\_HP | 2,171 | 0,934 | (0,223; 4,120) | 2,32 | 0,031 | 1,03 |
| HPMC\_PS | 2,38 | 1,29 | (-0,30; 5,06) | 1,85 | 0,079 | 1,37 |
| Lac\*HPMC\_Visc | -4,33 | 1,89 | (-8,27; -0,39) | -2,29 | 0,033 | 1,08 |
| HPMC\_Visc\*HPMC\_PS | 5,60 | 2,53 | (0,33; 10,87) | 2,22 | 0,038 | 1,25 |

## Model Summary

| S | R-sq | R-sq(adj) | PRESS | R-sq(pred) | AICc | BIC |
| --- | --- | --- | --- | --- | --- | --- |
| 2,27915 | 86,24% | 82,12% | 178,487 | 76,37% | 137,01 | 139,37 |

## Analysis of Variance

| Source | DF | Seq SS | Contribution | Adj SS | Adj MS | F-Value | P-Value |
| --- | --- | --- | --- | --- | --- | --- | --- |
| Model | 6 | 651,292 | 86,24% | 651,292 | 108,549 | 20,90 | 0,000 |
| Linear | 4 | 598,428 | 79,24% | 501,846 | 125,461 | 24,15 | 0,000 |
| Lac | 1 | 514,461 | 68,12% | 419,127 | 419,127 | 80,69 | 0,000 |
| HPMC\_Visc | 1 | 41,697 | 5,52% | 9,428 | 9,428 | 1,81 | 0,193 |
| HPMC\_HP | 1 | 36,251 | 4,80% | 28,060 | 28,060 | 5,40 | 0,031 |
| HPMC\_PS | 1 | 6,019 | 0,80% | 17,803 | 17,803 | 3,43 | 0,079 |
| 2-Way Interaction | 2 | 52,864 | 7,00% | 52,864 | 26,432 | 5,09 | 0,016 |
| Lac\*HPMC\_Visc | 1 | 27,345 | 3,62% | 27,345 | 27,345 | 5,26 | 0,033 |
| HPMC\_Visc\*HPMC\_PS | 1 | 25,519 | 3,38% | 25,519 | 25,519 | 4,91 | 0,038 |
| Error | 20 | 103,891 | 13,76% | 103,891 | 5,195 |  |  |
| Lack-of-Fit | 18 | 86,933 | 11,51% | 86,933 | 4,830 | 0,57 | 0,799 |
| Pure Error | 2 | 16,958 | 2,25% | 16,958 | 8,479 |  |  |
| Total | 26 | 755,182 | 100,00% |  |  |  |  |

## Regression Equation in Uncoded Units

|  |  |  |
| --- | --- | --- |
| F\_mean\_9h(540min) | = | 164,5 + 94,9 Lac - 0,01174 HPMC\_Visc + 2,139 HPMC\_HP - 2,32 HPMC\_PS - 0,00445 Lac\*HPMC\_Visc + 0,000196 HPMC\_Visc\*HPMC\_PS |

## Fits and Diagnostics for All Observations

| Obs | F\_mean\_9h(540min) | Fit | SE Fit | 95% CI | Resid | Std Resid | Del Resid | HI |
| --- | --- | --- | --- | --- | --- | --- | --- | --- |
| 1 | 61,82 | 59,95 | 1,21 | (57,41; 62,48) | 1,87 | 0,97 | 0,97 | 0,283627 |
| 2 | 70,69 | 71,70 | 1,21 | (69,17; 74,23) | -1,01 | -0,52 | -0,51 | 0,283627 |
| 3 | 55,31 | 57,13 | 1,59 | (53,81; 60,45) | -1,81 | -1,11 | -1,12 | 0,488210 |
| 4 | 63,03 | 62,81 | 1,59 | (59,49; 66,13) | 0,22 | 0,14 | 0,13 | 0,488210 |
| 5 | 58,31 | 61,83 | 1,06 | (59,62; 64,05) | -3,52 | -1,74 | -1,85 | 0,216779 |
| 6 | 71,25 | 73,32 | 1,06 | (71,10; 75,53) | -2,06 | -1,02 | -1,02 | 0,216779 |
| 7 | 59,80 | 60,95 | 1,43 | (57,96; 63,93) | -1,14 | -0,64 | -0,63 | 0,394639 |
| 8 | 65,38 | 66,54 | 1,43 | (63,55; 69,52) | -1,16 | -0,65 | -0,64 | 0,394639 |
| 9 | 59,43 | 59,03 | 1,24 | (56,46; 61,61) | 0,40 | 0,21 | 0,20 | 0,294244 |
| 10 | 70,74 | 70,70 | 1,24 | (68,12; 73,28) | 0,03 | 0,02 | 0,02 | 0,294244 |
| 11 | 63,16 | 61,62 | 1,07 | (59,40; 63,84) | 1,54 | 0,76 | 0,76 | 0,218366 |
| 12 | 68,85 | 69,46 | 1,07 | (67,24; 71,68) | -0,61 | -0,30 | -0,30 | 0,218366 |
| 13 | 60,61 | 61,21 | 1,34 | (58,41; 64,01) | -0,59 | -0,32 | -0,32 | 0,347162 |
| 14 | 73,59 | 72,91 | 1,34 | (70,11; 75,72) | 0,68 | 0,37 | 0,36 | 0,347162 |
| 15 | 62,64 | 63,44 | 1,16 | (61,02; 65,85) | -0,80 | -0,41 | -0,40 | 0,258117 |
| 16 | 67,27 | 70,89 | 1,16 | (68,47; 73,30) | -3,62 | -1,84 | -1,97 | 0,258117 |
| 17 | 56,36 | 56,68 | 1,05 | (54,48; 58,87) | -0,32 | -0,16 | -0,15 | 0,213198 |
| 18 | 77,06 | 75,65 | 1,05 | (73,45; 77,84) | 1,42 | 0,70 | 0,69 | 0,213198 |
| 19 | 68,90 | 66,90 | 0,90 | (65,01; 68,78) | 2,00 | 0,96 | 0,95 | 0,157071 |
| 20 | 67,37 | 63,33 | 1,06 | (61,13; 65,53) | 4,04 | 2,00 | 2,18 | 0,214445 |
| 21 | 63,91 | 64,00 | 0,94 | (62,04; 65,97) | -0,09 | -0,05 | -0,04 | 0,170686 |
| 22 | 72,51 | 68,50 | 1,14 | (66,12; 70,89) | 4,01 | 2,03 | 2,23 | 0,251394 |
| 23 | 67,11 | 65,33 | 1,34 | (62,53; 68,13) | 1,78 | 0,96 | 0,96 | 0,346058 |
| 24 | 67,04 | 66,93 | 1,24 | (64,33; 69,52) | 0,12 | 0,06 | 0,06 | 0,297564 |
| 25 | 63,34 | 66,16 | 0,48 | (65,16; 67,17) | -2,82 | -1,27 | -1,29 | 0,044701 |
| 26 | 64,82 | 66,16 | 0,48 | (65,16; 67,17) | -1,34 | -0,60 | -0,59 | 0,044701 |
| 27 | 68,96 | 66,16 | 0,48 | (65,16; 67,17) | 2,80 | 1,26 | 1,28 | 0,044701 |

| Obs | Cook’s D | DFITS |  |
| --- | --- | --- | --- |
| 1 | 0,05 | 0,60923 |  |
| 2 | 0,02 | -0,32272 |  |
| 3 | 0,17 | -1,09382 |  |
| 4 | 0,00 | 0,12877 |  |
| 5 | 0,12 | -0,97125 |  |
| 6 | 0,04 | -0,53861 |  |
| 7 | 0,04 | -0,51225 |  |
| 8 | 0,04 | -0,51953 |  |
| 9 | 0,00 | 0,13080 |  |
| 10 | 0,00 | 0,01107 |  |
| 11 | 0,02 | 0,39955 |  |
| 12 | 0,00 | -0,15631 |  |
| 13 | 0,01 | -0,22976 |  |
| 14 | 0,01 | 0,26279 |  |
| 15 | 0,01 | -0,23546 |  |
| 16 | 0,17 | -1,16388 |  |
| 17 | 0,00 | -0,07920 |  |
| 18 | 0,02 | 0,36015 |  |
| 19 | 0,02 | 0,41159 |  |
| 20 | 0,16 | 1,13943 | R |
| 21 | 0,00 | -0,02000 |  |
| 22 | 0,20 | 1,29013 | R |
| 23 | 0,07 | 0,69978 |  |
| 24 | 0,00 | 0,03852 |  |
| 25 | 0,01 | -0,27819 |  |
| 26 | 0,00 | -0,12838 |  |
| 27 | 0,01 | 0,27586 |  |

R  Large residual

## Backward Elimination of Terms

α to remove = 0,1

## Coded Coefficients

| Term | Coef | SE Coef | 95% CI | T-Value | P-Value | VIF |
| --- | --- | --- | --- | --- | --- | --- |
| Constant | 70,074 | 0,559 | (68,908; 71,239) | 125,44 | 0,000 |  |
| Lac | 9,37 | 1,03 | (7,22; 11,53) | 9,08 | 0,000 | 1,08 |
| HPMC\_Visc | -1,32 | 1,01 | (-3,42; 0,77) | -1,32 | 0,203 | 1,35 |
| HPMC\_HP | 2,296 | 0,999 | (0,213; 4,380) | 2,30 | 0,032 | 1,03 |
| HPMC\_PS | 2,30 | 1,38 | (-0,57; 5,17) | 1,67 | 0,110 | 1,37 |
| Lac\*HPMC\_Visc | -4,42 | 2,02 | (-8,63; -0,21) | -2,19 | 0,041 | 1,08 |
| HPMC\_Visc\*HPMC\_PS | 5,98 | 2,70 | (0,35; 11,62) | 2,21 | 0,039 | 1,25 |

## Model Summary

| S | R-sq | R-sq(adj) | PRESS | R-sq(pred) | AICc | BIC |
| --- | --- | --- | --- | --- | --- | --- |
| 2,43681 | 86,22% | 82,09% | 201,526 | 76,62% | 140,62 | 142,98 |

## Analysis of Variance

| Source | DF | Seq SS | Contribution | Adj SS | Adj MS | F-Value | P-Value |
| --- | --- | --- | --- | --- | --- | --- | --- |
| Model | 6 | 743,069 | 86,22% | 743,07 | 123,845 | 20,86 | 0,000 |
| Linear | 4 | 685,518 | 79,54% | 575,76 | 143,939 | 24,24 | 0,000 |
| Lac | 1 | 597,032 | 69,27% | 489,94 | 489,938 | 82,51 | 0,000 |
| HPMC\_Visc | 1 | 44,050 | 5,11% | 10,28 | 10,285 | 1,73 | 0,203 |
| HPMC\_HP | 1 | 39,790 | 4,62% | 31,38 | 31,384 | 5,29 | 0,032 |
| HPMC\_PS | 1 | 4,646 | 0,54% | 16,63 | 16,632 | 2,80 | 0,110 |
| 2-Way Interaction | 2 | 57,551 | 6,68% | 57,55 | 28,776 | 4,85 | 0,019 |
| Lac\*HPMC\_Visc | 1 | 28,431 | 3,30% | 28,43 | 28,431 | 4,79 | 0,041 |
| HPMC\_Visc\*HPMC\_PS | 1 | 29,120 | 3,38% | 29,12 | 29,120 | 4,90 | 0,039 |
| Error | 20 | 118,761 | 13,78% | 118,76 | 5,938 |  |  |
| Lack-of-Fit | 18 | 102,459 | 11,89% | 102,46 | 5,692 | 0,70 | 0,735 |
| Pure Error | 2 | 16,301 | 1,89% | 16,30 | 8,151 |  |  |
| Total | 26 | 861,830 | 100,00% |  |  |  |  |

## Regression Equation in Uncoded Units

|  |  |  |
| --- | --- | --- |
| F\_mean\_10h(600min) | = | 178,8 + 98,9 Lac - 0,01264 HPMC\_Visc + 2,263 HPMC\_HP - 2,51 HPMC\_PS - 0,00454 Lac\*HPMC\_Visc + 0,000209 HPMC\_Visc\*HPMC\_PS |

## Fits and Diagnostics for All Observations

| Obs | F\_mean\_10h(600min) | Fit | SE Fit | 95% CI | Resid | Std Resid | Del Resid | HI |
| --- | --- | --- | --- | --- | --- | --- | --- | --- |
| 1 | 65,67 | 63,59 | 1,30 | (60,88; 66,29) | 2,08 | 1,01 | 1,01 | 0,283627 |
| 2 | 74,63 | 76,10 | 1,30 | (73,40; 78,81) | -1,47 | -0,71 | -0,71 | 0,283627 |
| 3 | 58,83 | 60,55 | 1,70 | (57,00; 64,10) | -1,73 | -0,99 | -0,99 | 0,488210 |
| 4 | 66,93 | 66,88 | 1,70 | (63,33; 70,43) | 0,04 | 0,03 | 0,02 | 0,488210 |
| 5 | 62,05 | 65,56 | 1,13 | (63,19; 67,92) | -3,50 | -1,62 | -1,70 | 0,216779 |
| 6 | 75,06 | 77,80 | 1,13 | (75,43; 80,16) | -2,74 | -1,27 | -1,29 | 0,216779 |
| 7 | 63,63 | 64,55 | 1,53 | (61,36; 67,75) | -0,93 | -0,49 | -0,48 | 0,394639 |
| 8 | 69,51 | 70,79 | 1,53 | (67,60; 73,98) | -1,28 | -0,68 | -0,67 | 0,394639 |
| 9 | 62,76 | 62,45 | 1,32 | (59,69; 65,21) | 0,31 | 0,15 | 0,15 | 0,294244 |
| 10 | 74,79 | 74,88 | 1,32 | (72,12; 77,64) | -0,09 | -0,04 | -0,04 | 0,294244 |
| 11 | 67,12 | 65,19 | 1,14 | (62,81; 67,56) | 1,94 | 0,90 | 0,89 | 0,218366 |
| 12 | 72,64 | 73,72 | 1,14 | (71,34; 76,09) | -1,08 | -0,50 | -0,49 | 0,218366 |
| 13 | 64,44 | 64,72 | 1,44 | (61,72; 67,71) | -0,28 | -0,14 | -0,14 | 0,347162 |
| 14 | 77,49 | 77,19 | 1,44 | (74,20; 80,19) | 0,30 | 0,15 | 0,15 | 0,347162 |
| 15 | 66,38 | 67,10 | 1,24 | (64,51; 69,68) | -0,71 | -0,34 | -0,33 | 0,258117 |
| 16 | 71,30 | 75,22 | 1,24 | (72,64; 77,81) | -3,92 | -1,87 | -2,01 | 0,258117 |
| 17 | 60,11 | 59,95 | 1,13 | (57,61; 62,30) | 0,16 | 0,07 | 0,07 | 0,213198 |
| 18 | 84,23 | 80,36 | 1,13 | (78,01; 82,71) | 3,87 | 1,79 | 1,90 | 0,213198 |
| 19 | 72,93 | 70,89 | 0,97 | (68,87; 72,90) | 2,04 | 0,91 | 0,91 | 0,157071 |
| 20 | 70,91 | 67,19 | 1,13 | (64,84; 69,55) | 3,71 | 1,72 | 1,81 | 0,214445 |
| 21 | 67,57 | 67,89 | 1,01 | (65,79; 69,99) | -0,32 | -0,15 | -0,14 | 0,170686 |
| 22 | 76,19 | 72,61 | 1,22 | (70,06; 75,16) | 3,58 | 1,70 | 1,79 | 0,251394 |
| 23 | 71,17 | 69,50 | 1,43 | (66,51; 72,49) | 1,67 | 0,85 | 0,84 | 0,346058 |
| 24 | 70,77 | 70,72 | 1,33 | (67,95; 73,49) | 0,05 | 0,02 | 0,02 | 0,297564 |
| 25 | 67,14 | 70,16 | 0,52 | (69,08; 71,23) | -3,01 | -1,27 | -1,29 | 0,044701 |
| 26 | 68,89 | 70,16 | 0,52 | (69,08; 71,23) | -1,26 | -0,53 | -0,52 | 0,044701 |
| 27 | 72,73 | 70,16 | 0,52 | (69,08; 71,23) | 2,57 | 1,08 | 1,08 | 0,044701 |

| Obs | Cook’s D | DFITS |
| --- | --- | --- |
| 1 | 0,06 | 0,63480 |
| 2 | 0,03 | -0,44371 |
| 3 | 0,13 | -0,96702 |
| 4 | 0,00 | 0,02441 |
| 5 | 0,10 | -0,89421 |
| 6 | 0,06 | -0,67843 |
| 7 | 0,02 | -0,38710 |
| 8 | 0,04 | -0,53792 |
| 9 | 0,00 | 0,09688 |
| 10 | 0,00 | -0,02643 |
| 11 | 0,03 | 0,47275 |
| 12 | 0,01 | -0,26025 |
| 13 | 0,00 | -0,09943 |
| 14 | 0,00 | 0,10906 |
| 15 | 0,01 | -0,19601 |
| 16 | 0,17 | -1,18274 |
| 17 | 0,00 | 0,03711 |
| 18 | 0,12 | 0,99052 |
| 19 | 0,02 | 0,39264 |
| 20 | 0,12 | 0,94829 |
| 21 | 0,00 | -0,06425 |
| 22 | 0,14 | 1,03614 |
| 23 | 0,05 | 0,61299 |
| 24 | 0,00 | 0,01457 |
| 25 | 0,01 | -0,27816 |
| 26 | 0,00 | -0,11263 |
| 27 | 0,01 | 0,23426 |

## Backward Elimination of Terms

α to remove = 0,1

## Coded Coefficients

| Term | Coef | SE Coef | 95% CI | T-Value | P-Value | VIF |
| --- | --- | --- | --- | --- | --- | --- |
| Constant | 73,695 | 0,560 | (72,526; 74,864) | 131,51 | 0,000 |  |
| Lac | 9,65 | 1,04 | (7,49; 11,81) | 9,32 | 0,000 | 1,08 |
| HPMC\_Visc | -1,41 | 1,01 | (-3,51; 0,70) | -1,39 | 0,179 | 1,35 |
| HPMC\_HP | 2,46 | 1,00 | (0,37; 4,55) | 2,45 | 0,024 | 1,03 |
| HPMC\_PS | 2,30 | 1,38 | (-0,57; 5,18) | 1,67 | 0,111 | 1,37 |
| Lac\*HPMC\_Visc | -4,72 | 2,03 | (-8,95; -0,50) | -2,33 | 0,030 | 1,08 |
| HPMC\_Visc\*HPMC\_PS | 5,66 | 2,71 | (0,01; 11,32) | 2,09 | 0,050 | 1,25 |

## Model Summary

| S | R-sq | R-sq(adj) | PRESS | R-sq(pred) | AICc | BIC |
| --- | --- | --- | --- | --- | --- | --- |
| 2,44431 | 86,86% | 82,92% | 203,583 | 77,61% | 140,78 | 143,15 |

## Analysis of Variance

| Source | DF | Seq SS | Contribution | Adj SS | Adj MS | F-Value | P-Value |
| --- | --- | --- | --- | --- | --- | --- | --- |
| Model | 6 | 789,733 | 86,86% | 789,73 | 131,622 | 22,03 | 0,000 |
| Linear | 4 | 731,154 | 80,42% | 612,07 | 153,016 | 25,61 | 0,000 |
| Lac | 1 | 635,132 | 69,85% | 518,76 | 518,756 | 86,83 | 0,000 |
| HPMC\_Visc | 1 | 45,887 | 5,05% | 11,61 | 11,609 | 1,94 | 0,179 |
| HPMC\_HP | 1 | 44,933 | 4,94% | 35,91 | 35,910 | 6,01 | 0,024 |
| HPMC\_PS | 1 | 5,202 | 0,57% | 16,66 | 16,658 | 2,79 | 0,111 |
| 2-Way Interaction | 2 | 58,579 | 6,44% | 58,58 | 29,290 | 4,90 | 0,019 |
| Lac\*HPMC\_Visc | 1 | 32,505 | 3,58% | 32,51 | 32,505 | 5,44 | 0,030 |
| HPMC\_Visc\*HPMC\_PS | 1 | 26,074 | 2,87% | 26,07 | 26,074 | 4,36 | 0,050 |
| Error | 20 | 119,493 | 13,14% | 119,49 | 5,975 |  |  |
| Lack-of-Fit | 18 | 104,259 | 11,47% | 104,26 | 5,792 | 0,76 | 0,707 |
| Pure Error | 2 | 15,233 | 1,68% | 15,23 | 7,617 |  |  |
| Total | 26 | 909,226 | 100,00% |  |  |  |  |

## Regression Equation in Uncoded Units

|  |  |  |
| --- | --- | --- |
| F\_mean\_11h(660min) | = | 168,0 + 104,2 Lac - 0,01172 HPMC\_Visc + 2,420 HPMC\_HP - 2,36 HPMC\_PS - 0,00486 Lac\*HPMC\_Visc + 0,000198 HPMC\_Visc\*HPMC\_PS |

## Fits and Diagnostics for All Observations

| Obs | F\_mean\_11h(660min) | Fit | SE Fit | 95% CI | Resid | Std Resid | Del Resid | HI |
| --- | --- | --- | --- | --- | --- | --- | --- | --- |
| 1 | 68,86 | 66,85 | 1,30 | (64,13; 69,56) | 2,01 | 0,97 | 0,97 | 0,283627 |
| 2 | 78,48 | 79,86 | 1,30 | (77,14; 82,57) | -1,38 | -0,67 | -0,66 | 0,283627 |
| 3 | 62,20 | 64,09 | 1,71 | (60,53; 67,66) | -1,89 | -1,08 | -1,09 | 0,488210 |
| 4 | 70,58 | 70,48 | 1,71 | (66,92; 74,05) | 0,10 | 0,06 | 0,06 | 0,488210 |
| 5 | 65,58 | 68,99 | 1,14 | (66,62; 71,37) | -3,41 | -1,58 | -1,64 | 0,216779 |
| 6 | 78,49 | 81,71 | 1,14 | (79,33; 84,08) | -3,21 | -1,49 | -1,53 | 0,216779 |
| 7 | 67,32 | 68,25 | 1,54 | (65,05; 71,46) | -0,93 | -0,49 | -0,48 | 0,394639 |
| 8 | 73,44 | 74,54 | 1,54 | (71,34; 77,75) | -1,10 | -0,58 | -0,57 | 0,394639 |
| 9 | 65,94 | 65,87 | 1,33 | (63,10; 68,64) | 0,07 | 0,03 | 0,03 | 0,294244 |
| 10 | 78,64 | 78,79 | 1,33 | (76,02; 81,55) | -0,14 | -0,07 | -0,07 | 0,294244 |
| 11 | 70,91 | 68,60 | 1,14 | (66,22; 70,98) | 2,31 | 1,07 | 1,07 | 0,218366 |
| 12 | 76,19 | 77,34 | 1,14 | (74,96; 79,73) | -1,15 | -0,53 | -0,52 | 0,218366 |
| 13 | 68,14 | 68,34 | 1,44 | (65,34; 71,34) | -0,20 | -0,10 | -0,10 | 0,347162 |
| 14 | 82,05 | 81,30 | 1,44 | (78,29; 84,30) | 0,75 | 0,38 | 0,37 | 0,347162 |
| 15 | 70,11 | 70,61 | 1,24 | (68,02; 73,20) | -0,49 | -0,23 | -0,23 | 0,258117 |
| 16 | 75,05 | 78,92 | 1,24 | (76,33; 81,51) | -3,87 | -1,84 | -1,96 | 0,258117 |
| 17 | 63,67 | 63,25 | 1,13 | (60,90; 65,60) | 0,42 | 0,19 | 0,19 | 0,213198 |
| 18 | 88,47 | 84,32 | 1,13 | (81,96; 86,67) | 4,15 | 1,91 | 2,06 | 0,213198 |
| 19 | 76,75 | 74,61 | 0,97 | (72,59; 76,63) | 2,14 | 0,95 | 0,95 | 0,157071 |
| 20 | 74,25 | 70,75 | 1,13 | (68,39; 73,11) | 3,50 | 1,62 | 1,69 | 0,214445 |
| 21 | 70,99 | 71,36 | 1,01 | (69,25; 73,46) | -0,37 | -0,17 | -0,16 | 0,170686 |
| 22 | 79,48 | 76,40 | 1,23 | (73,85; 78,96) | 3,08 | 1,46 | 1,50 | 0,251394 |
| 23 | 74,80 | 73,05 | 1,44 | (70,05; 76,05) | 1,76 | 0,89 | 0,88 | 0,346058 |
| 24 | 74,21 | 74,43 | 1,33 | (71,64; 77,21) | -0,21 | -0,10 | -0,10 | 0,297564 |
| 25 | 70,68 | 73,78 | 0,52 | (72,71; 74,86) | -3,10 | -1,30 | -1,32 | 0,044701 |
| 26 | 72,61 | 73,78 | 0,52 | (72,71; 74,86) | -1,17 | -0,49 | -0,48 | 0,044701 |
| 27 | 76,13 | 73,78 | 0,52 | (72,71; 74,86) | 2,34 | 0,98 | 0,98 | 0,044701 |

| Obs | Cook’s D | DFITS |
| --- | --- | --- |
| 1 | 0,05 | 0,60992 |
| 2 | 0,03 | -0,41249 |
| 3 | 0,16 | -1,06121 |
| 4 | 0,00 | 0,05373 |
| 5 | 0,10 | -0,86356 |
| 6 | 0,09 | -0,80746 |
| 7 | 0,02 | -0,38712 |
| 8 | 0,03 | -0,45880 |
| 9 | 0,00 | 0,02062 |
| 10 | 0,00 | -0,04401 |
| 11 | 0,05 | 0,56637 |
| 12 | 0,01 | -0,27695 |
| 13 | 0,00 | -0,07035 |
| 14 | 0,01 | 0,27242 |
| 15 | 0,00 | -0,13476 |
| 16 | 0,17 | -1,15888 |
| 17 | 0,00 | 0,09759 |
| 18 | 0,14 | 1,07433 |
| 19 | 0,02 | 0,41068 |
| 20 | 0,10 | 0,88295 |
| 21 | 0,00 | -0,07365 |
| 22 | 0,10 | 0,86968 |
| 23 | 0,06 | 0,64370 |
| 24 | 0,00 | -0,06574 |
| 25 | 0,01 | -0,28584 |
| 26 | 0,00 | -0,10385 |
| 27 | 0,01 | 0,21201 |

## Backward Elimination of Terms

α to remove = 0,1

## Coded Coefficients

| Term | Coef | SE Coef | 95% CI | T-Value | P-Value | VIF |
| --- | --- | --- | --- | --- | --- | --- |
| Constant | 76,989 | 0,540 | (75,863; 78,115) | 142,61 | 0,000 |  |
| Lac | 9,678 | 0,997 | (7,598; 11,759) | 9,70 | 0,000 | 1,08 |
| HPMC\_Visc | -1,508 | 0,972 | (-3,536; 0,520) | -1,55 | 0,137 | 1,35 |
| HPMC\_HP | 2,497 | 0,965 | (0,484; 4,511) | 2,59 | 0,018 | 1,03 |
| HPMC\_PS | 2,32 | 1,33 | (-0,45; 5,10) | 1,75 | 0,096 | 1,37 |
| Lac\*HPMC\_Visc | -4,89 | 1,95 | (-8,96; -0,83) | -2,51 | 0,021 | 1,08 |
| HPMC\_Visc\*HPMC\_PS | 5,20 | 2,61 | (-0,25; 10,65) | 1,99 | 0,060 | 1,25 |

## Model Summary

| S | R-sq | R-sq(adj) | PRESS | R-sq(pred) | AICc | BIC |
| --- | --- | --- | --- | --- | --- | --- |
| 2,35481 | 87,82% | 84,17% | 192,097 | 78,91% | 138,77 | 141,14 |

## Analysis of Variance

| Source | DF | Seq SS | Contribution | Adj SS | Adj MS | F-Value | P-Value |
| --- | --- | --- | --- | --- | --- | --- | --- |
| Model | 6 | 799,869 | 87,82% | 799,87 | 133,312 | 24,04 | 0,000 |
| Linear | 4 | 742,961 | 81,57% | 620,61 | 155,152 | 27,98 | 0,000 |
| Lac | 1 | 642,010 | 70,49% | 522,23 | 522,231 | 94,18 | 0,000 |
| HPMC\_Visc | 1 | 48,372 | 5,31% | 13,34 | 13,336 | 2,40 | 0,137 |
| HPMC\_HP | 1 | 46,356 | 5,09% | 37,11 | 37,108 | 6,69 | 0,018 |
| HPMC\_PS | 1 | 6,223 | 0,68% | 16,95 | 16,953 | 3,06 | 0,096 |
| 2-Way Interaction | 2 | 56,909 | 6,25% | 56,91 | 28,454 | 5,13 | 0,016 |
| Lac\*HPMC\_Visc | 1 | 34,905 | 3,83% | 34,91 | 34,905 | 6,29 | 0,021 |
| HPMC\_Visc\*HPMC\_PS | 1 | 22,004 | 2,42% | 22,00 | 22,004 | 3,97 | 0,060 |
| Error | 20 | 110,902 | 12,18% | 110,90 | 5,545 |  |  |
| Lack-of-Fit | 18 | 96,486 | 10,59% | 96,49 | 5,360 | 0,74 | 0,714 |
| Pure Error | 2 | 14,416 | 1,58% | 14,42 | 7,208 |  |  |
| Total | 26 | 910,772 | 100,00% |  |  |  |  |

## Regression Equation in Uncoded Units

|  |  |  |
| --- | --- | --- |
| F\_mean\_12h(720min) | = | 154,6 + 106,7 Lac - 0,01053 HPMC\_Visc + 2,460 HPMC\_HP - 2,14 HPMC\_PS - 0,00503 Lac\*HPMC\_Visc + 0,000182 HPMC\_Visc\*HPMC\_PS |

## Fits and Diagnostics for All Observations

| Obs | F\_mean\_12h(720min) | Fit | SE Fit | 95% CI | Resid | Std Resid | Del Resid | HI |
| --- | --- | --- | --- | --- | --- | --- | --- | --- |
| 1 | 72,06 | 70,02 | 1,25 | (67,41; 72,64) | 2,04 | 1,02 | 1,02 | 0,283627 |
| 2 | 81,93 | 83,18 | 1,25 | (80,57; 85,80) | -1,26 | -0,63 | -0,62 | 0,283627 |
| 3 | 65,37 | 67,50 | 1,65 | (64,07; 70,93) | -2,13 | -1,26 | -1,29 | 0,488210 |
| 4 | 74,08 | 73,81 | 1,65 | (70,37; 77,24) | 0,27 | 0,16 | 0,16 | 0,488210 |
| 5 | 68,88 | 72,23 | 1,10 | (69,95; 74,52) | -3,35 | -1,61 | -1,68 | 0,216779 |
| 6 | 81,72 | 85,09 | 1,10 | (82,80; 87,38) | -3,37 | -1,62 | -1,69 | 0,216779 |
| 7 | 70,73 | 71,66 | 1,48 | (68,57; 74,74) | -0,93 | -0,51 | -0,50 | 0,394639 |
| 8 | 77,00 | 77,86 | 1,48 | (74,77; 80,94) | -0,86 | -0,47 | -0,46 | 0,394639 |
| 9 | 68,90 | 69,27 | 1,28 | (66,61; 71,94) | -0,37 | -0,19 | -0,18 | 0,294244 |
| 10 | 82,58 | 82,34 | 1,28 | (79,67; 85,00) | 0,24 | 0,12 | 0,12 | 0,294244 |
| 11 | 74,43 | 71,84 | 1,10 | (69,54; 74,13) | 2,59 | 1,24 | 1,26 | 0,218366 |
| 12 | 79,56 | 80,58 | 1,10 | (78,29; 82,88) | -1,02 | -0,49 | -0,48 | 0,218366 |
| 13 | 71,59 | 71,82 | 1,39 | (68,93; 74,72) | -0,23 | -0,12 | -0,12 | 0,347162 |
| 14 | 86,15 | 84,93 | 1,39 | (82,04; 87,83) | 1,21 | 0,64 | 0,63 | 0,347162 |
| 15 | 73,46 | 73,86 | 1,20 | (71,36; 76,35) | -0,40 | -0,20 | -0,19 | 0,258117 |
| 16 | 78,59 | 82,15 | 1,20 | (79,66; 84,65) | -3,57 | -1,76 | -1,86 | 0,258117 |
| 17 | 67,03 | 66,49 | 1,09 | (64,22; 68,76) | 0,54 | 0,26 | 0,25 | 0,213198 |
| 18 | 91,02 | 87,69 | 1,09 | (85,42; 89,95) | 3,33 | 1,59 | 1,66 | 0,213198 |
| 19 | 80,23 | 78,04 | 0,93 | (76,10; 79,99) | 2,19 | 1,01 | 1,01 | 0,157071 |
| 20 | 77,31 | 74,00 | 1,09 | (71,72; 76,27) | 3,32 | 1,59 | 1,66 | 0,214445 |
| 21 | 74,10 | 74,62 | 0,97 | (72,59; 76,65) | -0,52 | -0,24 | -0,23 | 0,170686 |
| 22 | 82,47 | 79,75 | 1,18 | (77,29; 82,22) | 2,72 | 1,33 | 1,36 | 0,251394 |
| 23 | 78,08 | 76,22 | 1,39 | (73,33; 79,11) | 1,86 | 0,98 | 0,97 | 0,346058 |
| 24 | 77,41 | 77,88 | 1,28 | (75,21; 80,56) | -0,48 | -0,24 | -0,24 | 0,297564 |
| 25 | 74,01 | 77,09 | 0,50 | (76,05; 78,13) | -3,08 | -1,34 | -1,36 | 0,044701 |
| 26 | 76,10 | 77,09 | 0,50 | (76,05; 78,13) | -0,99 | -0,43 | -0,42 | 0,044701 |
| 27 | 79,34 | 77,09 | 0,50 | (76,05; 78,13) | 2,25 | 0,98 | 0,98 | 0,044701 |

| Obs | Cook’s D | DFITS |
| --- | --- | --- |
| 1 | 0,06 | 0,64363 |
| 2 | 0,02 | -0,39143 |
| 3 | 0,22 | -1,25506 |
| 4 | 0,00 | 0,15258 |
| 5 | 0,10 | -0,88313 |
| 6 | 0,10 | -0,89001 |
| 7 | 0,02 | -0,40308 |
| 8 | 0,02 | -0,37290 |
| 9 | 0,00 | -0,11677 |
| 10 | 0,00 | 0,07555 |
| 11 | 0,06 | 0,66721 |
| 12 | 0,01 | -0,25519 |
| 13 | 0,00 | -0,08712 |
| 14 | 0,03 | 0,45830 |
| 15 | 0,00 | -0,11237 |
| 16 | 0,15 | -1,09947 |
| 17 | 0,00 | 0,13131 |
| 18 | 0,10 | 0,86610 |
| 19 | 0,03 | 0,43728 |
| 20 | 0,10 | 0,86538 |
| 21 | 0,00 | -0,10645 |
| 22 | 0,09 | 0,78890 |
| 23 | 0,07 | 0,70874 |
| 24 | 0,00 | -0,15441 |
| 25 | 0,01 | -0,29517 |
| 26 | 0,00 | -0,09071 |
| 27 | 0,01 | 0,21156 |

## Backward Elimination of Terms

α to remove = 0,1

## Coded Coefficients

| Term | Coef | SE Coef | 95% CI | T-Value | P-Value | VIF |
| --- | --- | --- | --- | --- | --- | --- |
| Constant | 80,024 | 0,520 | (78,940; 81,109) | 153,92 | 0,000 |  |
| Lac | 9,677 | 0,960 | (7,674; 11,681) | 10,08 | 0,000 | 1,08 |
| HPMC\_Visc | -1,473 | 0,936 | (-3,426; 0,480) | -1,57 | 0,131 | 1,35 |
| HPMC\_HP | 2,356 | 0,930 | (0,417; 4,295) | 2,53 | 0,020 | 1,03 |
| HPMC\_PS | 2,33 | 1,28 | (-0,34; 4,99) | 1,82 | 0,084 | 1,37 |
| Lac\*HPMC\_Visc | -4,66 | 1,88 | (-8,58; -0,74) | -2,48 | 0,022 | 1,08 |
| HPMC\_Visc\*HPMC\_PS | 5,08 | 2,51 | (-0,16; 10,33) | 2,02 | 0,057 | 1,25 |

## Model Summary

| S | R-sq | R-sq(adj) | PRESS | R-sq(pred) | AICc | BIC |
| --- | --- | --- | --- | --- | --- | --- |
| 2,26782 | 88,42% | 84,95% | 179,625 | 79,78% | 136,74 | 139,10 |

## Analysis of Variance

| Source | DF | Seq SS | Contribution | Adj SS | Adj MS | F-Value | P-Value |
| --- | --- | --- | --- | --- | --- | --- | --- |
| Model | 6 | 785,586 | 88,42% | 785,59 | 130,931 | 25,46 | 0,000 |
| Linear | 4 | 732,966 | 82,50% | 615,02 | 153,755 | 29,90 | 0,000 |
| Lac | 1 | 637,882 | 71,80% | 522,13 | 522,132 | 101,52 | 0,000 |
| HPMC\_Visc | 1 | 46,874 | 5,28% | 12,73 | 12,734 | 2,48 | 0,131 |
| HPMC\_HP | 1 | 41,747 | 4,70% | 33,04 | 33,040 | 6,42 | 0,020 |
| HPMC\_PS | 1 | 6,463 | 0,73% | 16,98 | 16,979 | 3,30 | 0,084 |
| 2-Way Interaction | 2 | 52,620 | 5,92% | 52,62 | 26,310 | 5,12 | 0,016 |
| Lac\*HPMC\_Visc | 1 | 31,593 | 3,56% | 31,59 | 31,593 | 6,14 | 0,022 |
| HPMC\_Visc\*HPMC\_PS | 1 | 21,027 | 2,37% | 21,03 | 21,027 | 4,09 | 0,057 |
| Error | 20 | 102,860 | 11,58% | 102,86 | 5,143 |  |  |
| Lack-of-Fit | 18 | 89,503 | 10,07% | 89,50 | 4,972 | 0,74 | 0,714 |
| Pure Error | 2 | 13,357 | 1,50% | 13,36 | 6,678 |  |  |
| Total | 26 | 888,446 | 100,00% |  |  |  |  |

## Regression Equation in Uncoded Units

|  |  |  |
| --- | --- | --- |
| F\_mean\_13h(780min) | = | 156,7 + 103,4 Lac - 0,01036 HPMC\_Visc + 2,321 HPMC\_HP - 2,08 HPMC\_PS - 0,00479 Lac\*HPMC\_Visc + 0,000178 HPMC\_Visc\*HPMC\_PS |

## Fits and Diagnostics for All Observations

| Obs | F\_mean\_13h(780min) | Fit | SE Fit | 95% CI | Resid | Std Resid | Del Resid | HI |
| --- | --- | --- | --- | --- | --- | --- | --- | --- |
| 1 | 75,21 | 73,20 | 1,21 | (70,68; 75,72) | 2,01 | 1,05 | 1,05 | 0,283627 |
| 2 | 85,11 | 86,19 | 1,21 | (83,67; 88,71) | -1,08 | -0,56 | -0,55 | 0,283627 |
| 3 | 68,33 | 70,63 | 1,58 | (67,33; 73,94) | -2,30 | -1,42 | -1,45 | 0,488210 |
| 4 | 77,45 | 77,10 | 1,58 | (73,79; 80,40) | 0,36 | 0,22 | 0,21 | 0,488210 |
| 5 | 72,00 | 75,28 | 1,06 | (73,08; 77,48) | -3,28 | -1,63 | -1,71 | 0,216779 |
| 6 | 84,62 | 87,98 | 1,06 | (85,78; 90,19) | -3,37 | -1,68 | -1,76 | 0,216779 |
| 7 | 73,78 | 74,60 | 1,42 | (71,63; 77,57) | -0,81 | -0,46 | -0,45 | 0,394639 |
| 8 | 80,48 | 80,97 | 1,42 | (78,00; 83,94) | -0,49 | -0,28 | -0,27 | 0,394639 |
| 9 | 71,67 | 72,50 | 1,23 | (69,93; 75,06) | -0,83 | -0,44 | -0,43 | 0,294244 |
| 10 | 86,27 | 85,40 | 1,23 | (82,83; 87,96) | 0,88 | 0,46 | 0,45 | 0,294244 |
| 11 | 77,67 | 74,92 | 1,06 | (72,71; 77,13) | 2,75 | 1,37 | 1,40 | 0,218366 |
| 12 | 82,65 | 83,71 | 1,06 | (81,50; 85,92) | -1,06 | -0,53 | -0,52 | 0,218366 |
| 13 | 74,71 | 74,90 | 1,34 | (72,12; 77,69) | -0,20 | -0,11 | -0,10 | 0,347162 |
| 14 | 88,78 | 87,85 | 1,34 | (85,06; 90,63) | 0,93 | 0,51 | 0,50 | 0,347162 |
| 15 | 76,50 | 76,84 | 1,15 | (74,43; 79,24) | -0,33 | -0,17 | -0,17 | 0,258117 |
| 16 | 81,89 | 85,20 | 1,15 | (82,80; 87,60) | -3,31 | -1,69 | -1,78 | 0,258117 |
| 17 | 70,16 | 69,57 | 1,05 | (67,39; 71,76) | 0,59 | 0,29 | 0,29 | 0,213198 |
| 18 | 93,34 | 90,68 | 1,05 | (88,49; 92,86) | 2,66 | 1,32 | 1,35 | 0,213198 |
| 19 | 83,43 | 81,07 | 0,90 | (79,19; 82,94) | 2,37 | 1,14 | 1,15 | 0,157071 |
| 20 | 80,14 | 77,11 | 1,05 | (74,92; 79,30) | 3,03 | 1,51 | 1,56 | 0,214445 |
| 21 | 77,18 | 77,79 | 0,94 | (75,83; 79,74) | -0,60 | -0,29 | -0,29 | 0,170686 |
| 22 | 85,23 | 82,65 | 1,14 | (80,27; 85,02) | 2,58 | 1,32 | 1,34 | 0,251394 |
| 23 | 81,03 | 79,23 | 1,33 | (76,44; 82,01) | 1,80 | 0,98 | 0,98 | 0,346058 |
| 24 | 80,49 | 80,97 | 1,24 | (78,39; 83,55) | -0,48 | -0,25 | -0,25 | 0,297564 |
| 25 | 77,11 | 80,13 | 0,48 | (79,13; 81,13) | -3,01 | -1,36 | -1,39 | 0,044701 |
| 26 | 79,19 | 80,13 | 0,48 | (79,13; 81,13) | -0,93 | -0,42 | -0,41 | 0,044701 |
| 27 | 82,25 | 80,13 | 0,48 | (79,13; 81,13) | 2,13 | 0,96 | 0,96 | 0,044701 |

| Obs | Cook’s D | DFITS |
| --- | --- | --- |
| 1 | 0,06 | 0,66021 |
| 2 | 0,02 | -0,34747 |
| 3 | 0,27 | -1,42004 |
| 4 | 0,01 | 0,20979 |
| 5 | 0,11 | -0,89942 |
| 6 | 0,11 | -0,92808 |
| 7 | 0,02 | -0,36521 |
| 8 | 0,01 | -0,21743 |
| 9 | 0,01 | -0,27523 |
| 10 | 0,01 | 0,29178 |
| 11 | 0,08 | 0,74260 |
| 12 | 0,01 | -0,27506 |
| 13 | 0,00 | -0,07609 |
| 14 | 0,02 | 0,36347 |
| 15 | 0,00 | -0,09837 |
| 16 | 0,14 | -1,05253 |
| 17 | 0,00 | 0,14846 |
| 18 | 0,07 | 0,70318 |
| 19 | 0,03 | 0,49473 |
| 20 | 0,09 | 0,81493 |
| 21 | 0,00 | -0,12952 |
| 22 | 0,08 | 0,77712 |
| 23 | 0,07 | 0,71258 |
| 24 | 0,00 | -0,15988 |
| 25 | 0,01 | -0,30070 |
| 26 | 0,00 | -0,08902 |
| 27 | 0,01 | 0,20702 |

## Backward Elimination of Terms

α to remove = 0,1

## Coded Coefficients

| Term | Coef | SE Coef | 95% CI | T-Value | P-Value | VIF |
| --- | --- | --- | --- | --- | --- | --- |
| Constant | 82,813 | 0,490 | (81,790; 83,835) | 168,95 | 0,000 |  |
| Lac | 9,515 | 0,906 | (7,626; 11,404) | 10,51 | 0,000 | 1,08 |
| HPMC\_Visc | -1,273 | 0,883 | (-3,115; 0,568) | -1,44 | 0,165 | 1,35 |
| HPMC\_HP | 2,239 | 0,876 | (0,411; 4,067) | 2,55 | 0,019 | 1,03 |
| HPMC\_PS | 2,21 | 1,21 | (-0,30; 4,73) | 1,84 | 0,081 | 1,37 |
| Lac\*HPMC\_Visc | -4,25 | 1,77 | (-7,94; -0,55) | -2,40 | 0,026 | 1,08 |
| HPMC\_Visc\*HPMC\_PS | 4,77 | 2,37 | (-0,17; 9,72) | 2,01 | 0,058 | 1,25 |

## Model Summary

| S | R-sq | R-sq(adj) | PRESS | R-sq(pred) | AICc | BIC |
| --- | --- | --- | --- | --- | --- | --- |
| 2,13807 | 88,97% | 85,66% | 163,157 | 80,32% | 133,55 | 135,92 |

## Analysis of Variance

| Source | DF | Seq SS | Contribution | Adj SS | Adj MS | F-Value | P-Value |
| --- | --- | --- | --- | --- | --- | --- | --- |
| Model | 6 | 737,502 | 88,97% | 737,502 | 122,917 | 26,89 | 0,000 |
| Linear | 4 | 692,649 | 83,56% | 585,077 | 146,269 | 32,00 | 0,000 |
| Lac | 1 | 611,236 | 73,74% | 504,767 | 504,767 | 110,42 | 0,000 |
| HPMC\_Visc | 1 | 37,705 | 4,55% | 9,514 | 9,514 | 2,08 | 0,165 |
| HPMC\_HP | 1 | 37,718 | 4,55% | 29,838 | 29,838 | 6,53 | 0,019 |
| HPMC\_PS | 1 | 5,990 | 0,72% | 15,406 | 15,406 | 3,37 | 0,081 |
| 2-Way Interaction | 2 | 44,853 | 5,41% | 44,853 | 22,427 | 4,91 | 0,018 |
| Lac\*HPMC\_Visc | 1 | 26,311 | 3,17% | 26,311 | 26,311 | 5,76 | 0,026 |
| HPMC\_Visc\*HPMC\_PS | 1 | 18,543 | 2,24% | 18,543 | 18,543 | 4,06 | 0,058 |
| Error | 20 | 91,427 | 11,03% | 91,427 | 4,571 |  |  |
| Lack-of-Fit | 18 | 79,226 | 9,56% | 79,226 | 4,401 | 0,72 | 0,724 |
| Pure Error | 2 | 12,200 | 1,47% | 12,200 | 6,100 |  |  |
| Total | 26 | 828,929 | 100,00% |  |  |  |  |

## Regression Equation in Uncoded Units

|  |  |  |
| --- | --- | --- |
| F\_mean\_14h(840min) | = | 153,9 + 97,1 Lac - 0,00977 HPMC\_Visc + 2,206 HPMC\_HP - 1,95 HPMC\_PS - 0,00437 Lac\*HPMC\_Visc + 0,000167 HPMC\_Visc\*HPMC\_PS |

## Fits and Diagnostics for All Observations

| Obs | F\_mean\_14h(840min) | Fit | SE Fit | 95% CI | Resid | Std Resid | Del Resid |
| --- | --- | --- | --- | --- | --- | --- | --- |
| 1 | 77,720 | 76,128 | 1,139 | (73,752; 78,503) | 1,593 | 0,88 | 0,87 |
| 2 | 87,872 | 88,668 | 1,139 | (86,293; 91,043) | -0,796 | -0,44 | -0,43 |
| 3 | 71,142 | 73,773 | 1,494 | (70,657; 76,889) | -2,631 | -1,72 | -1,82 |
| 4 | 80,866 | 80,358 | 1,494 | (77,242; 83,475) | 0,508 | 0,33 | 0,32 |
| 5 | 74,970 | 78,114 | 0,995 | (76,037; 80,190) | -3,144 | -1,66 | -1,74 |
| 6 | 87,433 | 90,388 | 0,995 | (88,312; 92,465) | -2,955 | -1,56 | -1,62 |
| 7 | 77,141 | 77,538 | 1,343 | (74,737; 80,340) | -0,397 | -0,24 | -0,23 |
| 8 | 84,045 | 84,035 | 1,343 | (81,233; 86,836) | 0,011 | 0,01 | 0,01 |
| 9 | 74,297 | 75,490 | 1,160 | (73,071; 77,910) | -1,193 | -0,66 | -0,65 |
| 10 | 89,522 | 87,947 | 1,160 | (85,528; 90,367) | 1,574 | 0,88 | 0,87 |
| 11 | 80,758 | 77,811 | 0,999 | (75,727; 79,895) | 2,948 | 1,56 | 1,62 |
| 12 | 85,507 | 86,515 | 0,999 | (84,431; 88,599) | -1,008 | -0,53 | -0,52 |
| 13 | 77,648 | 77,784 | 1,260 | (75,156; 80,412) | -0,137 | -0,08 | -0,08 |
| 14 | 90,387 | 90,278 | 1,260 | (87,650; 92,906) | 0,109 | 0,06 | 0,06 |
| 15 | 79,239 | 79,632 | 1,086 | (77,366; 81,898) | -0,394 | -0,21 | -0,21 |
| 16 | 84,863 | 87,948 | 1,086 | (85,682; 90,214) | -3,085 | -1,68 | -1,76 |
| 17 | 73,177 | 72,572 | 0,987 | (70,513; 74,631) | 0,605 | 0,32 | 0,31 |
| 18 | 94,946 | 93,197 | 0,987 | (91,138; 95,257) | 1,749 | 0,92 | 0,92 |
| 19 | 86,403 | 83,682 | 0,847 | (81,915; 85,450) | 2,720 | 1,39 | 1,42 |
| 20 | 82,719 | 80,174 | 0,990 | (78,109; 82,240) | 2,545 | 1,34 | 1,37 |
| 21 | 80,080 | 80,661 | 0,883 | (78,819; 82,504) | -0,581 | -0,30 | -0,29 |
| 22 | 87,653 | 85,290 | 1,072 | (83,053; 87,526) | 2,363 | 1,28 | 1,30 |
| 23 | 83,594 | 82,003 | 1,258 | (79,379; 84,626) | 1,592 | 0,92 | 0,92 |
| 24 | 83,371 | 83,696 | 1,166 | (81,263; 86,129) | -0,325 | -0,18 | -0,18 |
| 25 | 80,007 | 82,885 | 0,452 | (81,942; 83,828) | -2,878 | -1,38 | -1,41 |
| 26 | 82,056 | 82,885 | 0,452 | (81,942; 83,828) | -0,829 | -0,40 | -0,39 |
| 27 | 84,924 | 82,885 | 0,452 | (81,942; 83,828) | 2,039 | 0,98 | 0,97 |

| Obs | HI | Cook’s D | DFITS |
| --- | --- | --- | --- |
| 1 | 0,283627 | 0,04 | 0,55048 |
| 2 | 0,283627 | 0,01 | -0,27124 |
| 3 | 0,488210 | 0,40 | -1,77385 |
| 4 | 0,488210 | 0,02 | 0,31674 |
| 5 | 0,216779 | 0,11 | -0,91776 |
| 6 | 0,216779 | 0,10 | -0,85463 |
| 7 | 0,394639 | 0,01 | -0,18829 |
| 8 | 0,394639 | 0,00 | 0,00508 |
| 9 | 0,294244 | 0,03 | -0,42286 |
| 10 | 0,294244 | 0,05 | 0,56249 |
| 11 | 0,218366 | 0,10 | 0,85715 |
| 12 | 0,218366 | 0,01 | -0,27675 |
| 13 | 0,347162 | 0,00 | -0,05619 |
| 14 | 0,347162 | 0,00 | 0,04490 |
| 15 | 0,258117 | 0,00 | -0,12303 |
| 16 | 0,258117 | 0,14 | -1,03887 |
| 17 | 0,213198 | 0,00 | 0,16219 |
| 18 | 0,213198 | 0,03 | 0,47804 |
| 19 | 0,157071 | 0,05 | 0,61325 |
| 20 | 0,214445 | 0,07 | 0,71691 |
| 21 | 0,170686 | 0,00 | -0,13220 |
| 22 | 0,251394 | 0,08 | 0,75295 |
| 23 | 0,346058 | 0,06 | 0,66702 |
| 24 | 0,297564 | 0,00 | -0,11525 |
| 25 | 0,044701 | 0,01 | -0,30519 |
| 26 | 0,044701 | 0,00 | -0,08395 |
| 27 | 0,044701 | 0,01 | 0,21081 |

## Backward Elimination of Terms

α to remove = 0,1

## Coded Coefficients

| Term | Coef | SE Coef | 95% CI | T-Value | P-Value | VIF |
| --- | --- | --- | --- | --- | --- | --- |
| Constant | 85,319 | 0,480 | (84,318; 86,320) | 177,78 | 0,000 |  |
| Lac | 9,185 | 0,887 | (7,335; 11,034) | 10,36 | 0,000 | 1,08 |
| HPMC\_Visc | -1,136 | 0,864 | (-2,939; 0,666) | -1,31 | 0,203 | 1,35 |
| HPMC\_HP | 2,042 | 0,858 | (0,252; 3,832) | 2,38 | 0,027 | 1,03 |
| HPMC\_PS | 2,08 | 1,18 | (-0,38; 4,55) | 1,76 | 0,093 | 1,37 |
| Lac\*HPMC\_Visc | -3,64 | 1,73 | (-7,26; -0,03) | -2,10 | 0,049 | 1,08 |
| HPMC\_Visc\*HPMC\_PS | 4,53 | 2,32 | (-0,31; 9,37) | 1,95 | 0,065 | 1,25 |

## Model Summary

| S | R-sq | R-sq(adj) | PRESS | R-sq(pred) | AICc | BIC |
| --- | --- | --- | --- | --- | --- | --- |
| 2,09344 | 88,38% | 84,89% | 162,169 | 78,50% | 132,42 | 134,78 |

## Analysis of Variance

| Source | DF | Seq SS | Contribution | Adj SS | Adj MS | F-Value | P-Value |
| --- | --- | --- | --- | --- | --- | --- | --- |
| Model | 6 | 666,658 | 88,38% | 666,658 | 111,110 | 25,35 | 0,000 |
| Linear | 4 | 630,641 | 83,61% | 538,023 | 134,506 | 30,69 | 0,000 |
| Lac | 1 | 562,249 | 74,54% | 470,307 | 470,307 | 107,31 | 0,000 |
| HPMC\_Visc | 1 | 31,578 | 4,19% | 7,577 | 7,577 | 1,73 | 0,203 |
| HPMC\_HP | 1 | 31,576 | 4,19% | 24,823 | 24,823 | 5,66 | 0,027 |
| HPMC\_PS | 1 | 5,239 | 0,69% | 13,633 | 13,633 | 3,11 | 0,093 |
| 2-Way Interaction | 2 | 36,017 | 4,77% | 36,017 | 18,009 | 4,11 | 0,032 |
| Lac\*HPMC\_Visc | 1 | 19,347 | 2,56% | 19,347 | 19,347 | 4,41 | 0,049 |
| HPMC\_Visc\*HPMC\_PS | 1 | 16,671 | 2,21% | 16,671 | 16,671 | 3,80 | 0,065 |
| Error | 20 | 87,650 | 11,62% | 87,650 | 4,382 |  |  |
| Lack-of-Fit | 18 | 77,000 | 10,21% | 77,000 | 4,278 | 0,80 | 0,688 |
| Pure Error | 2 | 10,650 | 1,41% | 10,650 | 5,325 |  |  |
| Total | 26 | 754,308 | 100,00% |  |  |  |  |

## Regression Equation in Uncoded Units

|  |  |  |
| --- | --- | --- |
| F\_mean\_15h(900min) | = | 155,7 + 87,4 Lac - 0,00944 HPMC\_Visc + 2,012 HPMC\_HP - 1,85 HPMC\_PS - 0,00375 Lac\*HPMC\_Visc + 0,000158 HPMC\_Visc\*HPMC\_PS |

## Fits and Diagnostics for All Observations

| Obs | F\_mean\_15h(900min) | Fit | SE Fit | 95% CI | Resid | Std Resid | Del Resid |
| --- | --- | --- | --- | --- | --- | --- | --- |
| 1 | 80,606 | 79,048 | 1,115 | (76,722; 81,373) | 1,558 | 0,88 | 0,87 |
| 2 | 90,402 | 90,827 | 1,115 | (88,501; 93,152) | -0,425 | -0,24 | -0,23 |
| 3 | 73,720 | 76,653 | 1,463 | (73,601; 79,704) | -2,932 | -1,96 | -2,12 |
| 4 | 83,969 | 83,325 | 1,463 | (80,274; 86,376) | 0,644 | 0,43 | 0,42 |
| 5 | 77,633 | 80,847 | 0,975 | (78,814; 82,881) | -3,214 | -1,73 | -1,83 |
| 6 | 89,900 | 92,398 | 0,975 | (90,365; 94,431) | -2,498 | -1,35 | -1,38 |
| 7 | 80,197 | 80,118 | 1,315 | (77,375; 82,861) | 0,079 | 0,05 | 0,05 |
| 8 | 87,181 | 86,714 | 1,315 | (83,971; 89,457) | 0,467 | 0,29 | 0,28 |
| 9 | 76,944 | 78,427 | 1,136 | (76,058; 80,795) | -1,483 | -0,84 | -0,84 |
| 10 | 92,342 | 90,134 | 1,136 | (87,765; 92,503) | 2,208 | 1,26 | 1,27 |
| 11 | 83,565 | 80,506 | 0,978 | (78,466; 82,547) | 3,059 | 1,65 | 1,73 |
| 12 | 87,990 | 88,996 | 0,978 | (86,955; 91,036) | -1,006 | -0,54 | -0,53 |
| 13 | 80,335 | 80,513 | 1,233 | (77,940; 83,086) | -0,178 | -0,11 | -0,10 |
| 14 | 91,608 | 92,252 | 1,233 | (89,679; 94,825) | -0,644 | -0,38 | -0,37 |
| 15 | 81,597 | 82,169 | 1,064 | (79,950; 84,388) | -0,572 | -0,32 | -0,31 |
| 16 | 87,555 | 90,325 | 1,064 | (88,107; 92,544) | -2,771 | -1,54 | -1,59 |
| 17 | 75,884 | 75,510 | 0,967 | (73,494; 77,526) | 0,374 | 0,20 | 0,20 |
| 18 | 95,792 | 95,247 | 0,967 | (93,231; 97,263) | 0,545 | 0,29 | 0,29 |
| 19 | 89,091 | 86,077 | 0,830 | (84,346; 87,807) | 3,014 | 1,57 | 1,63 |
| 20 | 85,073 | 82,904 | 0,969 | (80,882; 84,926) | 2,169 | 1,17 | 1,18 |
| 21 | 82,700 | 83,348 | 0,865 | (81,544; 85,153) | -0,649 | -0,34 | -0,33 |
| 22 | 89,972 | 87,577 | 1,050 | (85,388; 89,767) | 2,395 | 1,32 | 1,35 |
| 23 | 85,860 | 84,555 | 1,232 | (81,986; 87,124) | 1,305 | 0,77 | 0,76 |
| 24 | 86,024 | 86,135 | 1,142 | (83,753; 88,517) | -0,111 | -0,06 | -0,06 |
| 25 | 82,743 | 85,378 | 0,443 | (84,455; 86,302) | -2,636 | -1,29 | -1,31 |
| 26 | 84,715 | 85,378 | 0,443 | (84,455; 86,302) | -0,663 | -0,32 | -0,32 |
| 27 | 87,342 | 85,378 | 0,443 | (84,455; 86,302) | 1,964 | 0,96 | 0,96 |

| Obs | HI | Cook’s D | DFITS |
| --- | --- | --- | --- |
| 1 | 0,283627 | 0,04 | 0,54994 |
| 2 | 0,283627 | 0,00 | -0,14733 |
| 3 | 0,488210 | 0,52 | -2,07309 |
| 4 | 0,488210 | 0,03 | 0,41158 |
| 5 | 0,216779 | 0,12 | -0,96519 |
| 6 | 0,216779 | 0,07 | -0,72524 |
| 7 | 0,394639 | 0,00 | 0,03832 |
| 8 | 0,394639 | 0,01 | 0,22613 |
| 9 | 0,294244 | 0,04 | -0,54037 |
| 10 | 0,294244 | 0,09 | 0,82314 |
| 11 | 0,218366 | 0,11 | 0,91635 |
| 12 | 0,218366 | 0,01 | -0,28197 |
| 13 | 0,347162 | 0,00 | -0,07494 |
| 14 | 0,347162 | 0,01 | -0,27169 |
| 15 | 0,258117 | 0,00 | -0,18273 |
| 16 | 0,258117 | 0,12 | -0,94061 |
| 17 | 0,213198 | 0,00 | 0,10240 |
| 18 | 0,213198 | 0,00 | 0,14922 |
| 19 | 0,157071 | 0,07 | 0,70458 |
| 20 | 0,214445 | 0,05 | 0,61660 |
| 21 | 0,170686 | 0,00 | -0,15091 |
| 22 | 0,251394 | 0,08 | 0,78173 |
| 23 | 0,346058 | 0,04 | 0,55488 |
| 24 | 0,297564 | 0,00 | -0,04006 |
| 25 | 0,044701 | 0,01 | -0,28360 |
| 26 | 0,044701 | 0,00 | -0,06851 |
| 27 | 0,044701 | 0,01 | 0,20720 |

## Backward Elimination of Terms

α to remove = 0,1

## Coded Coefficients

| Term | Coef | SE Coef | 95% CI | T-Value | P-Value | VIF |
| --- | --- | --- | --- | --- | --- | --- |
| Constant | 87,609 | 0,473 | (86,622; 88,597) | 185,07 | 0,000 |  |
| Lac | 8,692 | 0,875 | (6,867; 10,516) | 9,94 | 0,000 | 1,08 |
| HPMC\_Visc | -0,970 | 0,853 | (-2,748; 0,809) | -1,14 | 0,269 | 1,35 |
| HPMC\_HP | 1,870 | 0,846 | (0,104; 3,635) | 2,21 | 0,039 | 1,03 |
| HPMC\_PS | 2,01 | 1,17 | (-0,42; 4,44) | 1,73 | 0,100 | 1,37 |
| Lac\*HPMC\_Visc | -3,50 | 1,71 | (-7,07; 0,07) | -2,05 | 0,054 | 1,08 |
| HPMC\_Visc\*HPMC\_PS | 4,60 | 2,29 | (-0,18; 9,37) | 2,01 | 0,058 | 1,25 |

## Model Summary

| S | R-sq | R-sq(adj) | PRESS | R-sq(pred) | AICc | BIC |
| --- | --- | --- | --- | --- | --- | --- |
| 2,06492 | 87,50% | 83,75% | 163,582 | 76,02% | 131,67 | 134,04 |

## Analysis of Variance

| Source | DF | Seq SS | Contribution | Adj SS | Adj MS | F-Value | P-Value |
| --- | --- | --- | --- | --- | --- | --- | --- |
| Model | 6 | 596,987 | 87,50% | 596,987 | 99,498 | 23,33 | 0,000 |
| Linear | 4 | 561,941 | 82,36% | 478,670 | 119,667 | 28,07 | 0,000 |
| Lac | 1 | 504,288 | 73,91% | 421,165 | 421,165 | 98,77 | 0,000 |
| HPMC\_Visc | 1 | 26,365 | 3,86% | 5,516 | 5,516 | 1,29 | 0,269 |
| HPMC\_HP | 1 | 26,767 | 3,92% | 20,804 | 20,804 | 4,88 | 0,039 |
| HPMC\_PS | 1 | 4,521 | 0,66% | 12,723 | 12,723 | 2,98 | 0,100 |
| 2-Way Interaction | 2 | 35,046 | 5,14% | 35,046 | 17,523 | 4,11 | 0,032 |
| Lac\*HPMC\_Visc | 1 | 17,858 | 2,62% | 17,858 | 17,858 | 4,19 | 0,054 |
| HPMC\_Visc\*HPMC\_PS | 1 | 17,188 | 2,52% | 17,188 | 17,188 | 4,03 | 0,058 |
| Error | 20 | 85,278 | 12,50% | 85,278 | 4,264 |  |  |
| Lack-of-Fit | 18 | 75,849 | 11,12% | 75,849 | 4,214 | 0,89 | 0,652 |
| Pure Error | 2 | 9,429 | 1,38% | 9,429 | 4,715 |  |  |
| Total | 26 | 682,265 | 100,00% |  |  |  |  |

## Regression Equation in Uncoded Units

|  |  |  |
| --- | --- | --- |
| F\_mean\_16h(960min) | = | 164,0 + 83,4 Lac - 0,00964 HPMC\_Visc + 1,842 HPMC\_HP - 1,90 HPMC\_PS - 0,00360 Lac\*HPMC\_Visc + 0,000161 HPMC\_Visc\*HPMC\_PS |

## Fits and Diagnostics for All Observations

| Obs | F\_mean\_16h(960min) | Fit | SE Fit | 95% CI | Resid | Std Resid | Del Resid |
| --- | --- | --- | --- | --- | --- | --- | --- |
| 1 | 82,777 | 81,671 | 1,100 | (79,378; 83,965) | 1,105 | 0,63 | 0,62 |
| 2 | 93,041 | 92,856 | 1,100 | (90,562; 95,149) | 0,185 | 0,11 | 0,10 |
| 3 | 76,241 | 79,399 | 1,443 | (76,389; 82,408) | -3,157 | -2,14 | -2,37 |
| 4 | 86,554 | 85,676 | 1,443 | (82,667; 88,686) | 0,878 | 0,59 | 0,58 |
| 5 | 80,143 | 83,307 | 0,961 | (81,302; 85,313) | -3,165 | -1,73 | -1,83 |
| 6 | 92,409 | 94,272 | 0,961 | (92,267; 96,278) | -1,863 | -1,02 | -1,02 |
| 7 | 83,119 | 82,649 | 1,297 | (79,944; 85,355) | 0,470 | 0,29 | 0,29 |
| 8 | 89,650 | 88,854 | 1,297 | (86,148; 91,560) | 0,796 | 0,50 | 0,49 |
| 9 | 79,338 | 80,966 | 1,120 | (78,630; 83,303) | -1,628 | -0,94 | -0,94 |
| 10 | 94,542 | 92,081 | 1,120 | (89,745; 94,418) | 2,460 | 1,42 | 1,46 |
| 11 | 86,168 | 83,132 | 0,965 | (81,119; 85,145) | 3,036 | 1,66 | 1,75 |
| 12 | 90,270 | 91,156 | 0,965 | (89,143; 93,168) | -0,886 | -0,49 | -0,48 |
| 13 | 82,722 | 82,851 | 1,217 | (80,313; 85,389) | -0,129 | -0,08 | -0,08 |
| 14 | 92,607 | 93,997 | 1,217 | (91,459; 96,535) | -1,390 | -0,83 | -0,83 |
| 15 | 83,948 | 84,690 | 1,049 | (82,502; 86,878) | -0,742 | -0,42 | -0,41 |
| 16 | 89,920 | 92,393 | 1,049 | (90,205; 94,582) | -2,473 | -1,39 | -1,43 |
| 17 | 78,580 | 78,302 | 0,953 | (76,313; 80,291) | 0,278 | 0,15 | 0,15 |
| 18 | 96,318 | 96,999 | 0,953 | (95,010; 98,988) | -0,681 | -0,37 | -0,36 |
| 19 | 91,500 | 88,200 | 0,818 | (86,493; 89,907) | 3,300 | 1,74 | 1,84 |
| 20 | 87,193 | 85,392 | 0,956 | (83,397; 87,386) | 1,801 | 0,98 | 0,98 |
| 21 | 85,260 | 85,791 | 0,853 | (84,011; 87,570) | -0,530 | -0,28 | -0,28 |
| 22 | 92,111 | 89,672 | 1,035 | (87,512; 91,832) | 2,439 | 1,37 | 1,40 |
| 23 | 87,826 | 86,904 | 1,215 | (84,370; 89,438) | 0,922 | 0,55 | 0,54 |
| 24 | 88,425 | 88,321 | 1,126 | (85,971; 90,670) | 0,104 | 0,06 | 0,06 |
| 25 | 85,154 | 87,650 | 0,437 | (86,740; 88,561) | -2,497 | -1,24 | -1,25 |
| 26 | 87,173 | 87,650 | 0,437 | (86,740; 88,561) | -0,478 | -0,24 | -0,23 |
| 27 | 89,493 | 87,650 | 0,437 | (86,740; 88,561) | 1,843 | 0,91 | 0,91 |

| Obs | HI | Cook’s D | DFITS |  |
| --- | --- | --- | --- | --- |
| 1 | 0,283627 | 0,02 | 0,39182 |  |
| 2 | 0,283627 | 0,00 | 0,06500 |  |
| 3 | 0,488210 | 0,62 | -2,31611 | R |
| 4 | 0,488210 | 0,05 | 0,57094 |  |
| 5 | 0,216779 | 0,12 | -0,96313 |  |
| 6 | 0,216779 | 0,04 | -0,53681 |  |
| 7 | 0,394639 | 0,01 | 0,23067 |  |
| 8 | 0,394639 | 0,02 | 0,39223 |  |
| 9 | 0,294244 | 0,05 | -0,60404 |  |
| 10 | 0,294244 | 0,12 | 0,94109 |  |
| 11 | 0,218366 | 0,11 | 0,92295 |  |
| 12 | 0,218366 | 0,01 | -0,25145 |  |
| 13 | 0,347162 | 0,00 | -0,05509 |  |
| 14 | 0,347162 | 0,05 | -0,60261 |  |
| 15 | 0,258117 | 0,01 | -0,24090 |  |
| 16 | 0,258117 | 0,10 | -0,84121 |  |
| 17 | 0,213198 | 0,00 | 0,07702 |  |
| 18 | 0,213198 | 0,01 | -0,18920 |  |
| 19 | 0,157071 | 0,08 | 0,79504 |  |
| 20 | 0,214445 | 0,04 | 0,51382 |  |
| 21 | 0,170686 | 0,00 | -0,12493 |  |
| 22 | 0,251394 | 0,09 | 0,80986 |  |
| 23 | 0,346058 | 0,02 | 0,39435 |  |
| 24 | 0,297564 | 0,00 | 0,03828 |  |
| 25 | 0,044701 | 0,01 | -0,27140 |  |
| 26 | 0,044701 | 0,00 | -0,04997 |  |
| 27 | 0,044701 | 0,01 | 0,19663 |  |

R  Large residual

## Backward Elimination of Terms

α to remove = 0,1

## Coded Coefficients

| Term | Coef | SE Coef | 95% CI | T-Value | P-Value | VIF |
| --- | --- | --- | --- | --- | --- | --- |
| Constant | 89,474 | 0,517 | (88,402; 90,546) | 173,15 | 0,000 |  |
| Lac | 8,442 | 0,936 | (6,502; 10,382) | 9,02 | 0,000 | 1,00 |
| HPMC\_Visc | -0,764 | 0,941 | (-2,715; 1,187) | -0,81 | 0,425 | 1,33 |
| HPMC\_PS | 2,32 | 1,28 | (-0,33; 4,97) | 1,82 | 0,083 | 1,34 |
| HPMC\_Visc\*HPMC\_PS | 5,14 | 2,54 | (-0,12; 10,40) | 2,03 | 0,055 | 1,25 |

## Model Summary

| S | R-sq | R-sq(adj) | PRESS | R-sq(pred) | AICc | BIC |
| --- | --- | --- | --- | --- | --- | --- |
| 2,29189 | 80,67% | 77,15% | 167,738 | 71,94% | 132,08 | 135,65 |

## Analysis of Variance

| Source | DF | Seq SS | Contribution | Adj SS | Adj MS | F-Value | P-Value |
| --- | --- | --- | --- | --- | --- | --- | --- |
| Model | 4 | 482,158 | 80,67% | 482,158 | 120,539 | 22,95 | 0,000 |
| Linear | 3 | 460,557 | 77,05% | 462,349 | 154,116 | 29,34 | 0,000 |
| Lac | 1 | 427,608 | 71,54% | 427,608 | 427,608 | 81,41 | 0,000 |
| HPMC\_Visc | 1 | 26,277 | 4,40% | 3,464 | 3,464 | 0,66 | 0,425 |
| HPMC\_PS | 1 | 6,672 | 1,12% | 17,372 | 17,372 | 3,31 | 0,083 |
| 2-Way Interaction | 1 | 21,601 | 3,61% | 21,601 | 21,601 | 4,11 | 0,055 |
| HPMC\_Visc\*HPMC\_PS | 1 | 21,601 | 3,61% | 21,601 | 21,601 | 4,11 | 0,055 |
| Error | 22 | 115,560 | 19,33% | 115,560 | 5,253 |  |  |
| Lack-of-Fit | 20 | 107,055 | 17,91% | 107,055 | 5,353 | 1,26 | 0,534 |
| Pure Error | 2 | 8,505 | 1,42% | 8,505 | 4,253 |  |  |
| Total | 26 | 597,718 | 100,00% |  |  |  |  |

## Regression Equation in Uncoded Units

|  |  |  |
| --- | --- | --- |
| F\_mean\_17h(1020min) | = | 222,4 + 33,77 Lac - 0,01272 HPMC\_Visc - 2,11 HPMC\_PS + 0,000180 HPMC\_Visc\*HPMC\_PS |

## Fits and Diagnostics for All Observations

| Obs | F\_mean\_17h(1020min) | Fit | SE Fit | 95% CI | Resid | Std Resid | Del Resid |
| --- | --- | --- | --- | --- | --- | --- | --- |
| 1 | 86,287 | 86,128 | 0,976 | (84,103; 88,153) | 0,159 | 0,08 | 0,07 |
| 2 | 94,993 | 94,570 | 0,976 | (92,545; 96,595) | 0,424 | 0,20 | 0,20 |
| 3 | 78,511 | 81,330 | 1,302 | (78,630; 84,030) | -2,819 | -1,49 | -1,54 |
| 4 | 88,721 | 89,772 | 1,302 | (87,072; 92,472) | -1,051 | -0,56 | -0,55 |
| 5 | 82,549 | 85,923 | 0,874 | (84,112; 87,735) | -3,375 | -1,59 | -1,65 |
| 6 | 94,030 | 94,366 | 0,874 | (92,554; 96,177) | -0,335 | -0,16 | -0,15 |
| 7 | 85,468 | 82,381 | 1,040 | (80,225; 84,538) | 3,086 | 1,51 | 1,56 |
| 8 | 91,504 | 90,823 | 1,040 | (88,667; 92,980) | 0,681 | 0,33 | 0,33 |
| 9 | 81,530 | 85,269 | 1,003 | (83,189; 87,348) | -3,738 | -1,81 | -1,92 |
| 10 | 96,321 | 93,711 | 1,003 | (91,631; 95,790) | 2,610 | 1,27 | 1,29 |
| 11 | 88,495 | 85,804 | 0,935 | (83,864; 87,743) | 2,691 | 1,29 | 1,31 |
| 12 | 92,370 | 94,246 | 0,935 | (92,306; 96,185) | -1,875 | -0,90 | -0,89 |
| 13 | 84,957 | 85,122 | 1,132 | (82,773; 87,471) | -0,165 | -0,08 | -0,08 |
| 14 | 93,700 | 93,564 | 1,132 | (91,215; 95,913) | 0,136 | 0,07 | 0,07 |
| 15 | 86,046 | 86,045 | 1,082 | (83,800; 88,290) | 0,001 | 0,00 | 0,00 |
| 16 | 91,962 | 94,487 | 1,082 | (92,242; 96,732) | -2,525 | -1,25 | -1,27 |
| 17 | 80,949 | 81,128 | 1,051 | (78,948; 83,309) | -0,180 | -0,09 | -0,09 |
| 18 | 96,721 | 98,012 | 1,051 | (95,832; 100,193) | -1,291 | -0,63 | -0,63 |
| 19 | 93,609 | 89,975 | 0,900 | (88,109; 91,841) | 3,634 | 1,72 | 1,81 |
| 20 | 89,036 | 87,664 | 1,055 | (85,477; 89,851) | 1,372 | 0,67 | 0,67 |
| 21 | 87,569 | 89,465 | 0,494 | (88,441; 90,489) | -1,897 | -0,85 | -0,84 |
| 22 | 93,783 | 89,656 | 0,496 | (88,628; 90,685) | 4,127 | 1,84 | 1,96 |
| 23 | 89,589 | 88,684 | 1,332 | (85,922; 91,445) | 0,905 | 0,49 | 0,48 |
| 24 | 90,600 | 90,616 | 1,235 | (88,055; 93,176) | -0,016 | -0,01 | -0,01 |
| 25 | 87,288 | 89,570 | 0,480 | (88,576; 90,565) | -2,283 | -1,02 | -1,02 |
| 26 | 89,453 | 89,570 | 0,480 | (88,576; 90,565) | -0,117 | -0,05 | -0,05 |
| 27 | 91,410 | 89,570 | 0,480 | (88,576; 90,565) | 1,840 | 0,82 | 0,81 |

| Obs | HI | Cook’s D | DFITS |
| --- | --- | --- | --- |
| 1 | 0,181510 | 0,00 | 0,03529 |
| 2 | 0,181510 | 0,00 | 0,09409 |
| 3 | 0,322651 | 0,21 | -1,06315 |
| 4 | 0,322651 | 0,03 | -0,37835 |
| 5 | 0,145319 | 0,09 | -0,68218 |
| 6 | 0,145319 | 0,00 | -0,06381 |
| 7 | 0,205858 | 0,12 | 0,79405 |
| 8 | 0,205858 | 0,01 | 0,16626 |
| 9 | 0,191390 | 0,16 | -0,93492 |
| 10 | 0,191390 | 0,08 | 0,62519 |
| 11 | 0,166538 | 0,07 | 0,58409 |
| 12 | 0,166538 | 0,03 | -0,39877 |
| 13 | 0,244159 | 0,00 | -0,04604 |
| 14 | 0,244159 | 0,00 | 0,03804 |
| 15 | 0,222999 | 0,00 | 0,00028 |
| 16 | 0,222999 | 0,09 | -0,67880 |
| 17 | 0,210453 | 0,00 | -0,04447 |
| 18 | 0,210453 | 0,02 | -0,32282 |
| 19 | 0,154147 | 0,11 | 0,77320 |
| 20 | 0,211709 | 0,02 | 0,34505 |
| 21 | 0,046409 | 0,01 | -0,18570 |
| 22 | 0,046809 | 0,03 | 0,43428 |
| 23 | 0,337586 | 0,02 | 0,34011 |
| 24 | 0,290228 | 0,00 | -0,00502 |
| 25 | 0,043786 | 0,01 | -0,21814 |
| 26 | 0,043786 | 0,00 | -0,01092 |
| 27 | 0,043786 | 0,01 | 0,17434 |

## Backward Elimination of Terms

α to remove = 0,1

## Coded Coefficients

| Term | Coef | SE Coef | 95% CI | T-Value | P-Value | VIF |
| --- | --- | --- | --- | --- | --- | --- |
| Constant | 91,239 | 0,505 | (90,192; 92,286) | 180,72 | 0,000 |  |
| Lac | 7,766 | 0,914 | (5,870; 9,662) | 8,50 | 0,000 | 1,00 |
| HPMC\_Visc | -0,637 | 0,919 | (-2,543; 1,269) | -0,69 | 0,495 | 1,33 |
| HPMC\_PS | 2,40 | 1,25 | (-0,19; 4,99) | 1,92 | 0,068 | 1,34 |
| HPMC\_Visc\*HPMC\_PS | 5,25 | 2,48 | (0,11; 10,39) | 2,12 | 0,046 | 1,25 |

## Model Summary

| S | R-sq | R-sq(adj) | PRESS | R-sq(pred) | AICc | BIC |
| --- | --- | --- | --- | --- | --- | --- |
| 2,23920 | 79,01% | 75,19% | 161,477 | 69,27% | 130,82 | 134,40 |

## Analysis of Variance

| Source | DF | Seq SS | Contribution | Adj SS | Adj MS | F-Value | P-Value |
| --- | --- | --- | --- | --- | --- | --- | --- |
| Model | 4 | 415,164 | 79,01% | 415,164 | 103,791 | 20,70 | 0,000 |
| Linear | 3 | 392,649 | 74,72% | 395,530 | 131,843 | 26,30 | 0,000 |
| Lac | 1 | 361,840 | 68,86% | 361,840 | 361,840 | 72,17 | 0,000 |
| HPMC\_Visc | 1 | 23,561 | 4,48% | 2,411 | 2,411 | 0,48 | 0,495 |
| HPMC\_PS | 1 | 7,248 | 1,38% | 18,542 | 18,542 | 3,70 | 0,068 |
| 2-Way Interaction | 1 | 22,515 | 4,28% | 22,515 | 22,515 | 4,49 | 0,046 |
| HPMC\_Visc\*HPMC\_PS | 1 | 22,515 | 4,28% | 22,515 | 22,515 | 4,49 | 0,046 |
| Error | 22 | 110,308 | 20,99% | 110,308 | 5,014 |  |  |
| Lack-of-Fit | 20 | 102,456 | 19,50% | 102,456 | 5,123 | 1,30 | 0,522 |
| Pure Error | 2 | 7,852 | 1,49% | 7,852 | 3,926 |  |  |
| Total | 26 | 525,472 | 100,00% |  |  |  |  |

## Regression Equation in Uncoded Units

|  |  |  |
| --- | --- | --- |
| F\_mean\_18h(1080min) | = | 227,9 + 31,06 Lac - 0,01295 HPMC\_Visc - 2,15 HPMC\_PS + 0,000184 HPMC\_Visc\*HPMC\_PS |

## Fits and Diagnostics for All Observations

| Obs | F\_mean\_18h(1080min) | Fit | SE Fit | 95% CI | Resid | Std Resid | Del Resid |
| --- | --- | --- | --- | --- | --- | --- | --- |
| 1 | 88,263 | 88,141 | 0,954 | (86,162; 90,119) | 0,122 | 0,06 | 0,06 |
| 2 | 96,841 | 95,906 | 0,954 | (93,928; 97,885) | 0,935 | 0,46 | 0,45 |
| 3 | 80,676 | 83,433 | 1,272 | (80,795; 86,071) | -2,757 | -1,50 | -1,54 |
| 4 | 90,400 | 91,199 | 1,272 | (88,561; 93,836) | -0,799 | -0,43 | -0,43 |
| 5 | 84,581 | 87,943 | 0,854 | (86,173; 89,714) | -3,362 | -1,62 | -1,69 |
| 6 | 95,182 | 95,709 | 0,854 | (93,939; 97,479) | -0,527 | -0,25 | -0,25 |
| 7 | 87,829 | 84,515 | 1,016 | (82,408; 86,622) | 3,315 | 1,66 | 1,74 |
| 8 | 92,846 | 92,280 | 1,016 | (90,173; 94,387) | 0,566 | 0,28 | 0,28 |
| 9 | 83,545 | 87,285 | 0,980 | (85,254; 89,317) | -3,740 | -1,86 | -1,98 |
| 10 | 97,777 | 95,051 | 0,980 | (93,019; 97,083) | 2,726 | 1,35 | 1,38 |
| 11 | 90,546 | 87,952 | 0,914 | (86,056; 89,847) | 2,594 | 1,27 | 1,29 |
| 12 | 94,342 | 95,717 | 0,914 | (93,822; 97,612) | -1,375 | -0,67 | -0,66 |
| 13 | 86,942 | 87,137 | 1,106 | (84,843; 89,432) | -0,196 | -0,10 | -0,10 |
| 14 | 94,594 | 94,903 | 1,106 | (92,608; 97,198) | -0,309 | -0,16 | -0,16 |
| 15 | 87,894 | 88,213 | 1,057 | (86,020; 90,406) | -0,318 | -0,16 | -0,16 |
| 16 | 93,801 | 95,978 | 1,057 | (93,785; 98,171) | -2,177 | -1,10 | -1,11 |
| 17 | 83,208 | 83,544 | 1,027 | (81,413; 85,674) | -0,336 | -0,17 | -0,16 |
| 18 | 97,049 | 99,075 | 1,027 | (96,945; 101,206) | -2,026 | -1,02 | -1,02 |
| 19 | 95,396 | 91,611 | 0,879 | (89,788; 93,434) | 3,786 | 1,84 | 1,95 |
| 20 | 90,556 | 89,530 | 1,030 | (87,393; 91,667) | 1,026 | 0,52 | 0,51 |
| 21 | 89,650 | 91,201 | 0,482 | (90,201; 92,202) | -1,551 | -0,71 | -0,70 |
| 22 | 95,014 | 91,410 | 0,484 | (90,405; 92,415) | 3,604 | 1,65 | 1,72 |
| 23 | 91,006 | 90,367 | 1,301 | (87,669; 93,065) | 0,640 | 0,35 | 0,34 |
| 24 | 92,525 | 92,394 | 1,206 | (89,893; 94,896) | 0,130 | 0,07 | 0,07 |
| 25 | 89,220 | 91,309 | 0,469 | (90,338; 92,281) | -2,089 | -0,95 | -0,95 |
| 26 | 91,582 | 91,309 | 0,469 | (90,338; 92,281) | 0,272 | 0,12 | 0,12 |
| 27 | 93,157 | 91,309 | 0,469 | (90,338; 92,281) | 1,847 | 0,84 | 0,84 |

| Obs | HI | Cook’s D | DFITS |
| --- | --- | --- | --- |
| 1 | 0,181510 | 0,00 | 0,02778 |
| 2 | 0,181510 | 0,01 | 0,21339 |
| 3 | 0,322651 | 0,21 | -1,06431 |
| 4 | 0,322651 | 0,02 | -0,29362 |
| 5 | 0,145319 | 0,09 | -0,69747 |
| 6 | 0,145319 | 0,00 | -0,10270 |
| 7 | 0,205858 | 0,14 | 0,88356 |
| 8 | 0,205858 | 0,00 | 0,14125 |
| 9 | 0,191390 | 0,16 | -0,96141 |
| 10 | 0,191390 | 0,09 | 0,67215 |
| 11 | 0,166538 | 0,06 | 0,57565 |
| 12 | 0,166538 | 0,02 | -0,29691 |
| 13 | 0,244159 | 0,00 | -0,05578 |
| 14 | 0,244159 | 0,00 | -0,08818 |
| 15 | 0,222999 | 0,00 | -0,08449 |
| 16 | 0,222999 | 0,07 | -0,59403 |
| 17 | 0,210453 | 0,00 | -0,08513 |
| 18 | 0,210453 | 0,06 | -0,52617 |
| 19 | 0,154147 | 0,12 | 0,83337 |
| 20 | 0,211709 | 0,01 | 0,26283 |
| 21 | 0,046409 | 0,00 | -0,15470 |
| 22 | 0,046809 | 0,03 | 0,38127 |
| 23 | 0,337586 | 0,01 | 0,24544 |
| 24 | 0,290228 | 0,00 | 0,04319 |
| 25 | 0,043786 | 0,01 | -0,20376 |
| 26 | 0,043786 | 0,00 | 0,02600 |
| 27 | 0,043786 | 0,01 | 0,17931 |

## Backward Elimination of Terms

α to remove = 0,1

## Coded Coefficients

| Term | Coef | SE Coef | 95% CI | T-Value | P-Value | VIF |
| --- | --- | --- | --- | --- | --- | --- |
| Constant | 92,681 | 0,493 | (91,659; 93,704) | 188,00 | 0,000 |  |
| Lac | 7,057 | 0,893 | (5,205; 8,908) | 7,91 | 0,000 | 1,00 |
| HPMC\_Visc | -0,398 | 0,897 | (-2,259; 1,464) | -0,44 | 0,662 | 1,33 |
| HPMC\_PS | 2,50 | 1,22 | (-0,03; 5,03) | 2,05 | 0,052 | 1,34 |
| HPMC\_Visc\*HPMC\_PS | 5,38 | 2,42 | (0,36; 10,40) | 2,22 | 0,037 | 1,25 |

## Model Summary

| S | R-sq | R-sq(adj) | PRESS | R-sq(pred) | AICc | BIC |
| --- | --- | --- | --- | --- | --- | --- |
| 2,18657 | 76,83% | 72,62% | 154,658 | 65,94% | 129,54 | 133,11 |

## Analysis of Variance

| Source | DF | Seq SS | Contribution | Adj SS | Adj MS | F-Value | P-Value |
| --- | --- | --- | --- | --- | --- | --- | --- |
| Model | 4 | 348,836 | 76,83% | 348,836 | 87,209 | 18,24 | 0,000 |
| Linear | 3 | 325,213 | 71,63% | 329,992 | 109,997 | 23,01 | 0,000 |
| Lac | 1 | 298,769 | 65,81% | 298,769 | 298,769 | 62,49 | 0,000 |
| HPMC\_Visc | 1 | 18,375 | 4,05% | 0,939 | 0,939 | 0,20 | 0,662 |
| HPMC\_PS | 1 | 8,069 | 1,78% | 20,137 | 20,137 | 4,21 | 0,052 |
| 2-Way Interaction | 1 | 23,624 | 5,20% | 23,624 | 23,624 | 4,94 | 0,037 |
| HPMC\_Visc\*HPMC\_PS | 1 | 23,624 | 5,20% | 23,624 | 23,624 | 4,94 | 0,037 |
| Error | 22 | 105,184 | 23,17% | 105,184 | 4,781 |  |  |
| Lack-of-Fit | 20 | 97,596 | 21,50% | 97,596 | 4,880 | 1,29 | 0,527 |
| Pure Error | 2 | 7,588 | 1,67% | 7,588 | 3,794 |  |  |
| Total | 26 | 454,020 | 100,00% |  |  |  |  |

## Regression Equation in Uncoded Units

|  |  |  |
| --- | --- | --- |
| F\_mean\_19h(1140min) | = | 233,2 + 28,23 Lac - 0,01320 HPMC\_Visc - 2,20 HPMC\_PS + 0,000188 HPMC\_Visc\*HPMC\_PS |

## Fits and Diagnostics for All Observations

| Obs | F\_mean\_19h(1140min) | Fit | SE Fit | 95% CI | Resid | Std Resid | Del Resid |
| --- | --- | --- | --- | --- | --- | --- | --- |
| 1 | 89,693 | 89,765 | 0,932 | (87,833; 91,697) | -0,071 | -0,04 | -0,04 |
| 2 | 97,816 | 96,821 | 0,932 | (94,889; 98,753) | 0,994 | 0,50 | 0,49 |
| 3 | 82,737 | 85,286 | 1,242 | (82,710; 87,862) | -2,549 | -1,42 | -1,45 |
| 4 | 91,735 | 92,342 | 1,242 | (89,766; 94,918) | -0,607 | -0,34 | -0,33 |
| 5 | 86,118 | 89,582 | 0,834 | (87,853; 91,310) | -3,464 | -1,71 | -1,80 |
| 6 | 96,013 | 96,638 | 0,834 | (94,909; 98,367) | -0,625 | -0,31 | -0,30 |
| 7 | 89,485 | 86,407 | 0,992 | (84,350; 88,465) | 3,077 | 1,58 | 1,64 |
| 8 | 93,940 | 93,464 | 0,992 | (91,406; 95,521) | 0,476 | 0,24 | 0,24 |
| 9 | 85,335 | 88,922 | 0,957 | (86,938; 90,905) | -3,586 | -1,82 | -1,93 |
| 10 | 98,817 | 95,978 | 0,957 | (93,994; 97,962) | 2,839 | 1,44 | 1,48 |
| 11 | 92,469 | 89,821 | 0,892 | (87,970; 91,671) | 2,648 | 1,33 | 1,35 |
| 12 | 95,751 | 96,877 | 0,892 | (95,027; 98,728) | -1,127 | -0,56 | -0,56 |
| 13 | 88,653 | 88,772 | 1,080 | (86,531; 91,013) | -0,120 | -0,06 | -0,06 |
| 14 | 94,926 | 95,829 | 1,080 | (93,588; 98,069) | -0,903 | -0,47 | -0,47 |
| 15 | 89,395 | 90,114 | 1,033 | (87,972; 92,255) | -0,719 | -0,37 | -0,37 |
| 16 | 95,487 | 97,170 | 1,033 | (95,029; 99,312) | -1,683 | -0,87 | -0,87 |
| 17 | 85,239 | 85,648 | 1,003 | (83,567; 87,728) | -0,409 | -0,21 | -0,21 |
| 18 | 97,278 | 99,761 | 1,003 | (97,680; 101,841) | -2,483 | -1,28 | -1,30 |
| 19 | 96,840 | 92,811 | 0,858 | (91,031; 94,591) | 4,029 | 2,00 | 2,16 |
| 20 | 91,993 | 91,180 | 1,006 | (89,093; 93,266) | 0,813 | 0,42 | 0,41 |
| 21 | 91,337 | 92,592 | 0,471 | (91,615; 93,569) | -1,256 | -0,59 | -0,58 |
| 22 | 95,783 | 92,830 | 0,473 | (91,848; 93,811) | 2,954 | 1,38 | 1,41 |
| 23 | 92,347 | 91,679 | 1,270 | (89,044; 94,314) | 0,668 | 0,38 | 0,37 |
| 24 | 94,127 | 93,839 | 1,178 | (91,396; 96,282) | 0,288 | 0,16 | 0,15 |
| 25 | 90,831 | 92,704 | 0,458 | (91,755; 93,653) | -1,873 | -0,88 | -0,87 |
| 26 | 93,460 | 92,704 | 0,458 | (91,755; 93,653) | 0,756 | 0,35 | 0,35 |
| 27 | 94,635 | 92,704 | 0,458 | (91,755; 93,653) | 1,931 | 0,90 | 0,90 |

| Obs | HI | Cook’s D | DFITS |  |
| --- | --- | --- | --- | --- |
| 1 | 0,181510 | 0,00 | -0,01659 |  |
| 2 | 0,181510 | 0,01 | 0,23262 |  |
| 3 | 0,322651 | 0,19 | -1,00184 |  |
| 4 | 0,322651 | 0,01 | -0,22814 |  |
| 5 | 0,145319 | 0,10 | -0,74153 |  |
| 6 | 0,145319 | 0,00 | -0,12479 |  |
| 7 | 0,205858 | 0,13 | 0,83430 |  |
| 8 | 0,205858 | 0,00 | 0,12176 |  |
| 9 | 0,191390 | 0,16 | -0,94106 |  |
| 10 | 0,191390 | 0,10 | 0,72131 |  |
| 11 | 0,166538 | 0,07 | 0,60399 |  |
| 12 | 0,166538 | 0,01 | -0,24832 |  |
| 13 | 0,244159 | 0,00 | -0,03494 |  |
| 14 | 0,244159 | 0,01 | -0,26508 |  |
| 15 | 0,222999 | 0,01 | -0,19574 |  |
| 16 | 0,222999 | 0,04 | -0,46516 |  |
| 17 | 0,210453 | 0,00 | -0,10618 |  |
| 18 | 0,210453 | 0,09 | -0,66985 |  |
| 19 | 0,154147 | 0,15 | 0,92405 | R |
| 20 | 0,211709 | 0,01 | 0,21282 |  |
| 21 | 0,046409 | 0,00 | -0,12777 |  |
| 22 | 0,046809 | 0,02 | 0,31351 |  |
| 23 | 0,337586 | 0,01 | 0,26259 |  |
| 24 | 0,290228 | 0,00 | 0,09775 |  |
| 25 | 0,043786 | 0,01 | -0,18643 |  |
| 26 | 0,043786 | 0,00 | 0,07413 |  |
| 27 | 0,043786 | 0,01 | 0,19241 |  |

R  Large residual

## Backward Elimination of Terms

α to remove = 0,1

## Coded Coefficients

| Term | Coef | SE Coef | 95% CI | T-Value | P-Value | VIF |
| --- | --- | --- | --- | --- | --- | --- |
| Constant | 93,928 | 0,480 | (92,933; 94,923) | 195,77 | 0,000 |  |
| Lac | 6,285 | 0,869 | (4,483; 8,087) | 7,23 | 0,000 | 1,00 |
| HPMC\_Visc | -0,163 | 0,873 | (-1,974; 1,649) | -0,19 | 0,854 | 1,33 |
| HPMC\_PS | 2,62 | 1,19 | (0,16; 5,08) | 2,21 | 0,038 | 1,34 |
| HPMC\_Visc\*HPMC\_PS | 5,51 | 2,36 | (0,63; 10,40) | 2,34 | 0,029 | 1,25 |

## Model Summary

| S | R-sq | R-sq(adj) | PRESS | R-sq(pred) | AICc | BIC |
| --- | --- | --- | --- | --- | --- | --- |
| 2,12799 | 74,09% | 69,38% | 147,049 | 61,76% | 128,07 | 131,65 |

## Analysis of Variance

| Source | DF | Seq SS | Contribution | Adj SS | Adj MS | F-Value | P-Value |
| --- | --- | --- | --- | --- | --- | --- | --- |
| Model | 4 | 284,904 | 74,09% | 284,904 | 71,226 | 15,73 | 0,000 |
| Linear | 3 | 260,110 | 67,64% | 266,834 | 88,945 | 19,64 | 0,000 |
| Lac | 1 | 237,011 | 61,64% | 237,011 | 237,011 | 52,34 | 0,000 |
| HPMC\_Visc | 1 | 14,015 | 3,64% | 0,157 | 0,157 | 0,03 | 0,854 |
| HPMC\_PS | 1 | 9,084 | 2,36% | 22,025 | 22,025 | 4,86 | 0,038 |
| 2-Way Interaction | 1 | 24,794 | 6,45% | 24,794 | 24,794 | 5,48 | 0,029 |
| HPMC\_Visc\*HPMC\_PS | 1 | 24,794 | 6,45% | 24,794 | 24,794 | 5,48 | 0,029 |
| Error | 22 | 99,623 | 25,91% | 99,623 | 4,528 |  |  |
| Lack-of-Fit | 20 | 92,133 | 23,96% | 92,133 | 4,607 | 1,23 | 0,542 |
| Pure Error | 2 | 7,490 | 1,95% | 7,490 | 3,745 |  |  |
| Total | 26 | 384,527 | 100,00% |  |  |  |  |

## Regression Equation in Uncoded Units

|  |  |  |
| --- | --- | --- |
| F\_mean\_20h(1200min) | = | 238,4 + 25,14 Lac - 0,01346 HPMC\_Visc - 2,25 HPMC\_PS + 0,000193 HPMC\_Visc\*HPMC\_PS |

## Fits and Diagnostics for All Observations

| Obs | F\_mean\_20h(1200min) | Fit | SE Fit | 95% CI | Resid | Std Resid | Del Resid |
| --- | --- | --- | --- | --- | --- | --- | --- |
| 1 | 91,090 | 91,224 | 0,907 | (89,344; 93,104) | -0,135 | -0,07 | -0,07 |
| 2 | 98,570 | 97,509 | 0,907 | (95,629; 99,389) | 1,061 | 0,55 | 0,54 |
| 3 | 84,646 | 86,961 | 1,209 | (84,454; 89,468) | -2,315 | -1,32 | -1,35 |
| 4 | 92,728 | 93,246 | 1,209 | (90,739; 95,753) | -0,518 | -0,30 | -0,29 |
| 5 | 87,775 | 91,056 | 0,811 | (89,374; 92,738) | -3,281 | -1,67 | -1,74 |
| 6 | 96,426 | 97,341 | 0,811 | (95,659; 99,023) | -0,915 | -0,47 | -0,46 |
| 7 | 91,144 | 88,125 | 0,966 | (86,123; 90,128) | 3,019 | 1,59 | 1,65 |
| 8 | 94,766 | 94,410 | 0,966 | (92,408; 96,413) | 0,356 | 0,19 | 0,18 |
| 9 | 86,973 | 90,401 | 0,931 | (88,470; 92,331) | -3,427 | -1,79 | -1,89 |
| 10 | 99,587 | 96,686 | 0,931 | (94,755; 98,617) | 2,901 | 1,52 | 1,57 |
| 11 | 94,143 | 91,527 | 0,868 | (89,726; 93,328) | 2,616 | 1,35 | 1,37 |
| 12 | 96,671 | 97,812 | 0,868 | (96,011; 99,613) | -1,142 | -0,59 | -0,58 |
| 13 | 90,115 | 90,251 | 1,051 | (88,071; 92,432) | -0,136 | -0,07 | -0,07 |
| 14 | 95,062 | 96,536 | 1,051 | (94,356; 98,717) | -1,474 | -0,80 | -0,79 |
| 15 | 90,723 | 91,853 | 1,005 | (89,769; 93,937) | -1,130 | -0,60 | -0,59 |
| 16 | 96,903 | 98,138 | 1,005 | (96,054; 100,222) | -1,234 | -0,66 | -0,65 |
| 17 | 87,069 | 87,618 | 0,976 | (85,594; 89,643) | -0,550 | -0,29 | -0,28 |
| 18 | 97,726 | 100,188 | 0,976 | (98,164; 102,213) | -2,462 | -1,30 | -1,32 |
| 19 | 97,916 | 93,821 | 0,835 | (92,088; 95,554) | 4,095 | 2,09 | 2,28 |
| 20 | 93,229 | 92,627 | 0,979 | (90,596; 94,657) | 0,602 | 0,32 | 0,31 |
| 21 | 92,798 | 93,787 | 0,458 | (92,836; 94,738) | -0,989 | -0,48 | -0,47 |
| 22 | 96,332 | 94,054 | 0,460 | (93,099; 95,009) | 2,278 | 1,10 | 1,10 |
| 23 | 93,320 | 92,784 | 1,236 | (90,220; 95,348) | 0,536 | 0,31 | 0,30 |
| 24 | 95,627 | 95,101 | 1,146 | (92,723; 97,478) | 0,526 | 0,29 | 0,29 |
| 25 | 92,291 | 93,903 | 0,445 | (92,980; 94,827) | -1,613 | -0,77 | -0,77 |
| 26 | 95,163 | 93,903 | 0,445 | (92,980; 94,827) | 1,260 | 0,61 | 0,60 |
| 27 | 95,974 | 93,903 | 0,445 | (92,980; 94,827) | 2,070 | 0,99 | 0,99 |

| Obs | HI | Cook’s D | DFITS |  |
| --- | --- | --- | --- | --- |
| 1 | 0,181510 | 0,00 | -0,032188 |  |
| 2 | 0,181510 | 0,01 | 0,255344 |  |
| 3 | 0,322651 | 0,17 | -0,929155 |  |
| 4 | 0,322651 | 0,01 | -0,199680 |  |
| 5 | 0,145319 | 0,09 | -0,718942 |  |
| 6 | 0,145319 | 0,01 | -0,188347 |  |
| 7 | 0,205858 | 0,13 | 0,841964 |  |
| 8 | 0,205858 | 0,00 | 0,093379 |  |
| 9 | 0,191390 | 0,15 | -0,921183 |  |
| 10 | 0,191390 | 0,11 | 0,761602 |  |
| 11 | 0,166538 | 0,07 | 0,613959 |  |
| 12 | 0,166538 | 0,01 | -0,258674 |  |
| 13 | 0,244159 | 0,00 | -0,040897 |  |
| 14 | 0,244159 | 0,04 | -0,448964 |  |
| 15 | 0,222999 | 0,02 | -0,317922 |  |
| 16 | 0,222999 | 0,02 | -0,347888 |  |
| 17 | 0,210453 | 0,00 | -0,146893 |  |
| 18 | 0,210453 | 0,09 | -0,683659 |  |
| 19 | 0,154147 | 0,16 | 0,975076 | R |
| 20 | 0,211709 | 0,01 | 0,161737 |  |
| 21 | 0,046409 | 0,00 | -0,103077 |  |
| 22 | 0,046809 | 0,01 | 0,244130 |  |
| 23 | 0,337586 | 0,01 | 0,216441 |  |
| 24 | 0,290228 | 0,01 | 0,183661 |  |
| 25 | 0,043786 | 0,01 | -0,164271 |  |
| 26 | 0,043786 | 0,00 | 0,127642 |  |
| 27 | 0,043786 | 0,01 | 0,212840 |  |

R  Large residual

## Backward Elimination of Terms

α to remove = 0,1

## Coded Coefficients

| Term | Coef | SE Coef | 95% CI | T-Value | P-Value | VIF |
| --- | --- | --- | --- | --- | --- | --- |
| Constant | 95,607 | 0,577 | (94,407; 96,807) | 165,65 | 0,000 |  |
| Lac | 5,434 | 0,832 | (3,705; 7,164) | 6,53 | 0,000 | 1,00 |
| HPMC\_Visc | 0,032 | 0,836 | (-1,707; 1,771) | 0,04 | 0,970 | 1,33 |
| HPMC\_PS | 2,61 | 1,14 | (0,25; 4,98) | 2,30 | 0,032 | 1,34 |
| Lac\*Lac | -2,90 | 1,58 | (-6,18; 0,38) | -1,84 | 0,081 | 1,00 |
| HPMC\_Visc\*HPMC\_PS | 5,60 | 2,25 | (0,91; 10,29) | 2,48 | 0,022 | 1,25 |

## Model Summary

| S | R-sq | R-sq(adj) | PRESS | R-sq(pred) | AICc | BIC |
| --- | --- | --- | --- | --- | --- | --- |
| 2,03722 | 73,02% | 66,60% | 140,937 | 56,37% | 128,16 | 131,33 |

## Analysis of Variance

| Source | DF | Seq SS | Contribution | Adj SS | Adj MS | F-Value | P-Value |
| --- | --- | --- | --- | --- | --- | --- | --- |
| Model | 5 | 235,883 | 73,02% | 235,883 | 47,177 | 11,37 | 0,000 |
| Linear | 3 | 196,314 | 60,77% | 204,270 | 68,090 | 16,41 | 0,000 |
| Lac | 1 | 177,182 | 54,85% | 177,182 | 177,182 | 42,69 | 0,000 |
| HPMC\_Visc | 1 | 10,124 | 3,13% | 0,006 | 0,006 | 0,00 | 0,970 |
| HPMC\_PS | 1 | 9,008 | 2,79% | 21,984 | 21,984 | 5,30 | 0,032 |
| Square | 1 | 13,995 | 4,33% | 13,988 | 13,988 | 3,37 | 0,081 |
| Lac\*Lac | 1 | 13,995 | 4,33% | 13,988 | 13,988 | 3,37 | 0,081 |
| 2-Way Interaction | 1 | 25,573 | 7,92% | 25,573 | 25,573 | 6,16 | 0,022 |
| HPMC\_Visc\*HPMC\_PS | 1 | 25,573 | 7,92% | 25,573 | 25,573 | 6,16 | 0,022 |
| Error | 21 | 87,155 | 26,98% | 87,155 | 4,150 |  |  |
| Lack-of-Fit | 19 | 80,309 | 24,86% | 80,309 | 4,227 | 1,23 | 0,540 |
| Pure Error | 2 | 6,846 | 2,12% | 6,846 | 3,423 |  |  |
| Total | 26 | 323,038 | 100,00% |  |  |  |  |

## Regression Equation in Uncoded Units

|  |  |  |
| --- | --- | --- |
| F\_mean\_21h(1260min) | = | 232,4 + 68,1 Lac - 0,01362 HPMC\_Visc - 2,29 HPMC\_PS - 46,4 Lac\*Lac + 0,000196 HPMC\_Visc\*HPMC\_PS |

## Fits and Diagnostics for All Observations

| Obs | F\_mean\_21h(1260min) | Fit | SE Fit | 95% CI | Resid | Std Resid | Del Resid |
| --- | --- | --- | --- | --- | --- | --- | --- |
| 1 | 92,78 | 92,48 | 0,87 | (90,67; 94,29) | 0,29 | 0,16 | 0,16 |
| 2 | 99,08 | 97,92 | 0,87 | (96,11; 99,72) | 1,17 | 0,63 | 0,62 |
| 3 | 86,40 | 88,44 | 1,16 | (86,03; 90,85) | -2,04 | -1,22 | -1,23 |
| 4 | 93,38 | 93,88 | 1,16 | (91,47; 96,29) | -0,50 | -0,30 | -0,29 |
| 5 | 89,11 | 92,32 | 0,78 | (90,70; 93,94) | -3,21 | -1,70 | -1,79 |
| 6 | 96,64 | 97,75 | 0,78 | (96,14; 99,37) | -1,11 | -0,59 | -0,58 |
| 7 | 92,64 | 89,62 | 0,93 | (87,70; 91,55) | 3,02 | 1,66 | 1,74 |
| 8 | 95,76 | 95,06 | 0,93 | (93,13; 96,98) | 0,71 | 0,39 | 0,38 |
| 9 | 88,39 | 91,62 | 0,89 | (89,76; 93,48) | -3,23 | -1,76 | -1,87 |
| 10 | 100,23 | 97,05 | 0,89 | (95,20; 98,91) | 3,18 | 1,74 | 1,83 |
| 11 | 95,69 | 92,95 | 0,83 | (91,22; 94,68) | 2,74 | 1,48 | 1,52 |
| 12 | 97,35 | 98,38 | 0,83 | (96,65; 100,11) | -1,04 | -0,56 | -0,55 |
| 13 | 91,33 | 91,46 | 1,01 | (89,37; 93,56) | -0,13 | -0,08 | -0,07 |
| 14 | 94,95 | 96,90 | 1,01 | (94,80; 98,99) | -1,94 | -1,10 | -1,10 |
| 15 | 91,80 | 93,29 | 0,96 | (91,29; 95,30) | -1,49 | -0,83 | -0,82 |
| 16 | 97,98 | 98,73 | 0,96 | (96,72; 100,73) | -0,75 | -0,42 | -0,41 |
| 17 | 88,70 | 87,22 | 1,54 | (84,01; 90,42) | 1,48 | 1,11 | 1,12 |
| 18 | 97,69 | 98,08 | 1,54 | (94,88; 101,29) | -0,40 | -0,30 | -0,29 |
| 19 | 98,77 | 95,30 | 0,88 | (93,48; 97,12) | 3,47 | 1,89 | 2,02 |
| 20 | 94,24 | 94,49 | 1,00 | (92,41; 96,56) | -0,25 | -0,14 | -0,14 |
| 21 | 94,09 | 95,43 | 0,56 | (94,26; 96,60) | -1,35 | -0,69 | -0,68 |
| 22 | 96,73 | 95,71 | 0,56 | (94,54; 96,88) | 1,02 | 0,52 | 0,51 |
| 23 | 94,12 | 94,44 | 1,24 | (91,87; 97,01) | -0,32 | -0,20 | -0,19 |
| 24 | 96,83 | 96,70 | 1,15 | (94,31; 99,09) | 0,13 | 0,08 | 0,07 |
| 25 | 93,60 | 95,55 | 0,55 | (94,40; 96,70) | -1,95 | -0,99 | -0,99 |
| 26 | 96,59 | 95,55 | 0,55 | (94,40; 96,70) | 1,05 | 0,53 | 0,52 |
| 27 | 96,98 | 95,55 | 0,55 | (94,40; 96,70) | 1,44 | 0,73 | 0,72 |

| Obs | HI | Cook’s D | DFITS |
| --- | --- | --- | --- |
| 1 | 0,181848 | 0,00 | 0,07338 |
| 2 | 0,181848 | 0,01 | 0,29388 |
| 3 | 0,323174 | 0,12 | -0,85135 |
| 4 | 0,323174 | 0,01 | -0,20006 |
| 5 | 0,145676 | 0,08 | -0,74020 |
| 6 | 0,145676 | 0,01 | -0,23987 |
| 7 | 0,206416 | 0,12 | 0,88925 |
| 8 | 0,206416 | 0,01 | 0,19463 |
| 9 | 0,191815 | 0,12 | -0,90910 |
| 10 | 0,191815 | 0,12 | 0,89132 |
| 11 | 0,167095 | 0,07 | 0,68114 |
| 12 | 0,167095 | 0,01 | -0,24548 |
| 13 | 0,244597 | 0,00 | -0,04180 |
| 14 | 0,244597 | 0,07 | -0,62816 |
| 15 | 0,223583 | 0,03 | -0,44247 |
| 16 | 0,223583 | 0,01 | -0,21946 |
| 17 | 0,573092 | 0,28 | 1,29837 |
| 18 | 0,573092 | 0,02 | -0,33926 |
| 19 | 0,184894 | 0,13 | 0,96351 |
| 20 | 0,239930 | 0,00 | -0,07653 |
| 21 | 0,076218 | 0,01 | -0,19503 |
| 22 | 0,076343 | 0,00 | 0,14769 |
| 23 | 0,368545 | 0,00 | -0,14569 |
| 24 | 0,318905 | 0,00 | 0,05129 |
| 25 | 0,073524 | 0,01 | -0,27944 |
| 26 | 0,073524 | 0,00 | 0,14775 |
| 27 | 0,073524 | 0,01 | 0,20385 |

## Backward Elimination of Terms

α to remove = 0,1

## Coded Coefficients

| Term | Coef | SE Coef | 95% CI | T-Value | P-Value | VIF |
| --- | --- | --- | --- | --- | --- | --- |
| Constant | 96,422 | 0,562 | (95,247; 97,598) | 171,64 | 0,000 |  |
| Lac | 4,528 | 0,799 | (2,855; 6,201) | 5,66 | 0,000 | 1,00 |
| HPMC\_Visc | 0,523 | 0,824 | (-1,202; 2,249) | 0,63 | 0,533 | 1,40 |
| HPMC\_HP | 0,365 | 0,819 | (-1,348; 2,079) | 0,45 | 0,660 | 1,07 |
| HPMC\_PS | 2,43 | 1,11 | (0,11; 4,75) | 2,19 | 0,041 | 1,39 |
| Lac\*Lac | -3,13 | 1,52 | (-6,30; 0,05) | -2,06 | 0,053 | 1,00 |
| HPMC\_Visc\*HPMC\_HP | 2,68 | 1,52 | (-0,50; 5,85) | 1,77 | 0,093 | 1,21 |
| HPMC\_Visc\*HPMC\_PS | 4,76 | 2,23 | (0,09; 9,43) | 2,13 | 0,046 | 1,32 |

## Model Summary

| S | R-sq | R-sq(adj) | PRESS | R-sq(pred) | AICc | BIC |
| --- | --- | --- | --- | --- | --- | --- |
| 1,95800 | 72,61% | 62,53% | 143,005 | 46,24% | 132,01 | 133,08 |

## Analysis of Variance

| Source | DF | Seq SS | Contribution | Adj SS | Adj MS | F-Value | P-Value |
| --- | --- | --- | --- | --- | --- | --- | --- |
| Model | 7 | 193,147 | 72,61% | 193,147 | 27,592 | 7,20 | 0,000 |
| Linear | 4 | 138,961 | 52,24% | 144,507 | 36,127 | 9,42 | 0,000 |
| Lac | 1 | 123,030 | 46,25% | 123,030 | 123,030 | 32,09 | 0,000 |
| HPMC\_Visc | 1 | 6,783 | 2,55% | 1,544 | 1,544 | 0,40 | 0,533 |
| HPMC\_HP | 1 | 0,880 | 0,33% | 0,764 | 0,764 | 0,20 | 0,660 |
| HPMC\_PS | 1 | 8,268 | 3,11% | 18,421 | 18,421 | 4,80 | 0,041 |
| Square | 1 | 16,061 | 6,04% | 16,301 | 16,301 | 4,25 | 0,053 |
| Lac\*Lac | 1 | 16,061 | 6,04% | 16,301 | 16,301 | 4,25 | 0,053 |
| 2-Way Interaction | 2 | 38,124 | 14,33% | 38,124 | 19,062 | 4,97 | 0,018 |
| HPMC\_Visc\*HPMC\_HP | 1 | 20,689 | 7,78% | 11,954 | 11,954 | 3,12 | 0,093 |
| HPMC\_Visc\*HPMC\_PS | 1 | 17,435 | 6,55% | 17,435 | 17,435 | 4,55 | 0,046 |
| Error | 19 | 72,842 | 27,39% | 72,842 | 3,834 |  |  |
| Lack-of-Fit | 17 | 66,730 | 25,09% | 66,730 | 3,925 | 1,28 | 0,525 |
| Pure Error | 2 | 6,112 | 2,30% | 6,112 | 3,056 |  |  |
| Total | 26 | 265,988 | 100,00% |  |  |  |  |

## Regression Equation in Uncoded Units

|  |  |  |
| --- | --- | --- |
| F\_mean\_22h(1320min) | = | 289,9 + 68,2 Lac - 0,01788 HPMC\_Visc - 8,80 HPMC\_HP - 1,92 HPMC\_PS - 50,0 Lac\*Lac + 0,000678 HPMC\_Visc\*HPMC\_HP + 0,000166 HPMC\_Visc\*HPMC\_PS |

## Fits and Diagnostics for All Observations

| Obs | F\_mean\_22h(1320min) | Fit | SE Fit | 95% CI | Resid | Std Resid | Del Resid |
| --- | --- | --- | --- | --- | --- | --- | --- |
| 1 | 94,06 | 94,35 | 1,03 | (92,19; 96,51) | -0,30 | -0,18 | -0,17 |
| 2 | 99,13 | 98,88 | 1,03 | (96,72; 101,04) | 0,25 | 0,15 | 0,15 |
| 3 | 87,98 | 88,77 | 1,32 | (86,00; 91,54) | -0,79 | -0,54 | -0,53 |
| 4 | 93,86 | 93,30 | 1,32 | (90,53; 96,07) | 0,56 | 0,39 | 0,38 |
| 5 | 90,34 | 92,87 | 0,89 | (91,01; 94,72) | -2,52 | -1,45 | -1,49 |
| 6 | 96,86 | 97,40 | 0,89 | (95,54; 99,25) | -0,54 | -0,31 | -0,30 |
| 7 | 93,92 | 92,56 | 1,32 | (89,79; 95,33) | 1,36 | 0,94 | 0,94 |
| 8 | 96,34 | 97,09 | 1,32 | (94,32; 99,86) | -0,75 | -0,52 | -0,51 |
| 9 | 89,63 | 93,67 | 1,11 | (91,34; 96,00) | -4,04 | -2,51 | -2,98 |
| 10 | 100,47 | 98,20 | 1,11 | (95,87; 100,53) | 2,27 | 1,41 | 1,45 |
| 11 | 97,02 | 93,76 | 0,92 | (91,85; 95,68) | 3,25 | 1,88 | 2,03 |
| 12 | 97,61 | 98,29 | 0,92 | (96,37; 100,21) | -0,69 | -0,40 | -0,39 |
| 13 | 92,37 | 91,92 | 1,13 | (89,56; 94,28) | 0,45 | 0,28 | 0,27 |
| 14 | 94,72 | 96,45 | 1,13 | (94,09; 98,81) | -1,73 | -1,08 | -1,09 |
| 15 | 92,90 | 94,72 | 0,94 | (92,76; 96,69) | -1,82 | -1,06 | -1,06 |
| 16 | 98,69 | 99,25 | 0,94 | (97,29; 101,21) | -0,56 | -0,33 | -0,32 |
| 17 | 90,13 | 88,62 | 1,49 | (85,51; 91,73) | 1,51 | 1,18 | 1,19 |
| 18 | 97,57 | 97,68 | 1,49 | (94,57; 100,79) | -0,11 | -0,09 | -0,08 |
| 19 | 99,38 | 95,85 | 0,85 | (94,07; 97,63) | 3,53 | 2,00 | 2,19 |
| 20 | 95,13 | 95,41 | 0,97 | (93,39; 97,44) | -0,29 | -0,17 | -0,16 |
| 21 | 95,20 | 96,28 | 0,88 | (94,44; 98,12) | -1,08 | -0,62 | -0,61 |
| 22 | 96,94 | 96,52 | 1,04 | (94,34; 98,70) | 0,42 | 0,25 | 0,25 |
| 23 | 94,80 | 95,12 | 1,21 | (92,59; 97,64) | -0,32 | -0,21 | -0,20 |
| 24 | 97,84 | 97,49 | 1,12 | (95,15; 99,83) | 0,35 | 0,22 | 0,21 |
| 25 | 94,78 | 96,28 | 0,54 | (95,15; 97,40) | -1,49 | -0,79 | -0,79 |
| 26 | 97,80 | 96,28 | 0,54 | (95,15; 97,40) | 1,52 | 0,81 | 0,80 |
| 27 | 97,82 | 96,28 | 0,54 | (95,15; 97,40) | 1,55 | 0,82 | 0,81 |

| Obs | HI | Cook’s D | DFITS |  |
| --- | --- | --- | --- | --- |
| 1 | 0,277172 | 0,00 | -0,10726 |  |
| 2 | 0,277172 | 0,00 | 0,09113 |  |
| 3 | 0,457798 | 0,03 | -0,49101 |  |
| 4 | 0,457798 | 0,02 | 0,35064 |  |
| 5 | 0,205107 | 0,07 | -0,75800 |  |
| 6 | 0,205107 | 0,00 | -0,15286 |  |
| 7 | 0,457290 | 0,09 | 0,86280 |  |
| 8 | 0,457290 | 0,03 | -0,46735 |  |
| 9 | 0,323159 | 0,38 | -2,06140 | R |
| 10 | 0,323159 | 0,12 | 1,00316 |  |
| 11 | 0,219006 | 0,12 | 1,07375 |  |
| 12 | 0,219006 | 0,01 | -0,20505 |  |
| 13 | 0,331534 | 0,00 | 0,19215 |  |
| 14 | 0,331534 | 0,07 | -0,76504 |  |
| 15 | 0,229133 | 0,04 | -0,57983 |  |
| 16 | 0,229133 | 0,00 | -0,17372 |  |
| 17 | 0,575594 | 0,24 | 1,38961 |  |
| 18 | 0,575594 | 0,00 | -0,09720 |  |
| 19 | 0,188718 | 0,12 | 1,05743 | R |
| 20 | 0,244362 | 0,00 | -0,09362 |  |
| 21 | 0,201449 | 0,01 | -0,30524 |  |
| 22 | 0,281809 | 0,00 | 0,15533 |  |
| 23 | 0,379535 | 0,00 | -0,15639 |  |
| 24 | 0,326342 | 0,00 | 0,14661 |  |
| 25 | 0,075399 | 0,01 | -0,22423 |  |
| 26 | 0,075399 | 0,01 | 0,22872 |  |
| 27 | 0,075399 | 0,01 | 0,23242 |  |

R  Large residual

## Backward Elimination of Terms

α to remove = 0,1

## Coded Coefficients

| Term | Coef | SE Coef | 95% CI | T-Value | P-Value | VIF |
| --- | --- | --- | --- | --- | --- | --- |
| Constant | 97,161 | 0,559 | (95,999; 98,323) | 173,94 | 0,000 |  |
| Lac | 3,662 | 0,805 | (1,988; 5,336) | 4,55 | 0,000 | 1,00 |
| HPMC\_Visc | 0,463 | 0,809 | (-1,219; 2,146) | 0,57 | 0,573 | 1,33 |
| HPMC\_PS | 2,69 | 1,10 | (0,40; 4,97) | 2,44 | 0,024 | 1,34 |
| Lac\*Lac | -3,24 | 1,53 | (-6,42; -0,07) | -2,12 | 0,046 | 1,00 |
| HPMC\_Visc\*HPMC\_PS | 5,78 | 2,18 | (1,24; 10,32) | 2,65 | 0,015 | 1,25 |

## Model Summary

| S | R-sq | R-sq(adj) | PRESS | R-sq(pred) | AICc | BIC |
| --- | --- | --- | --- | --- | --- | --- |
| 1,97162 | 63,00% | 54,19% | 130,917 | 40,66% | 126,39 | 129,57 |

## Analysis of Variance

| Source | DF | Seq SS | Contribution | Adj SS | Adj MS | F-Value | P-Value |
| --- | --- | --- | --- | --- | --- | --- | --- |
| Model | 5 | 138,971 | 63,00% | 138,971 | 27,794 | 7,15 | 0,000 |
| Linear | 3 | 94,164 | 42,68% | 104,972 | 34,991 | 9,00 | 0,000 |
| Lac | 1 | 80,459 | 36,47% | 80,459 | 80,459 | 20,70 | 0,000 |
| HPMC\_Visc | 1 | 4,262 | 1,93% | 1,275 | 1,275 | 0,33 | 0,573 |
| HPMC\_PS | 1 | 9,443 | 4,28% | 23,195 | 23,195 | 5,97 | 0,024 |
| Square | 1 | 17,522 | 7,94% | 17,514 | 17,514 | 4,51 | 0,046 |
| Lac\*Lac | 1 | 17,522 | 7,94% | 17,514 | 17,514 | 4,51 | 0,046 |
| 2-Way Interaction | 1 | 27,285 | 12,37% | 27,285 | 27,285 | 7,02 | 0,015 |
| HPMC\_Visc\*HPMC\_PS | 1 | 27,285 | 12,37% | 27,285 | 27,285 | 7,02 | 0,015 |
| Error | 21 | 81,633 | 37,00% | 81,633 | 3,887 |  |  |
| Lack-of-Fit | 19 | 76,440 | 34,65% | 76,440 | 4,023 | 1,55 | 0,464 |
| Pure Error | 2 | 5,193 | 2,35% | 5,193 | 2,596 |  |  |
| Total | 26 | 220,604 | 100,00% |  |  |  |  |

## Regression Equation in Uncoded Units

|  |  |  |
| --- | --- | --- |
| F\_mean\_23h(1380min) | = | 240,0 + 66,5 Lac - 0,01396 HPMC\_Visc - 2,365 HPMC\_PS - 51,9 Lac\*Lac + 0,000202 HPMC\_Visc\*HPMC\_PS |

## Fits and Diagnostics for All Observations

| Obs | F\_mean\_23h(1380min) | Fit | SE Fit | 95% CI | Resid | Std Resid | Del Resid |
| --- | --- | --- | --- | --- | --- | --- | --- |
| 1 | 94,91 | 94,54 | 0,84 | (92,80; 96,29) | 0,37 | 0,21 | 0,20 |
| 2 | 99,06 | 98,21 | 0,84 | (96,46; 99,95) | 0,85 | 0,48 | 0,47 |
| 3 | 89,49 | 90,98 | 1,12 | (88,65; 93,31) | -1,49 | -0,92 | -0,92 |
| 4 | 94,10 | 94,64 | 1,12 | (92,31; 96,97) | -0,54 | -0,33 | -0,33 |
| 5 | 91,57 | 94,40 | 0,75 | (92,84; 95,97) | -2,83 | -1,55 | -1,61 |
| 6 | 96,95 | 98,06 | 0,75 | (96,50; 99,63) | -1,11 | -0,61 | -0,60 |
| 7 | 94,89 | 92,20 | 0,90 | (90,34; 94,07) | 2,69 | 1,53 | 1,58 |
| 8 | 96,68 | 95,86 | 0,90 | (94,00; 97,73) | 0,82 | 0,47 | 0,46 |
| 9 | 90,50 | 93,65 | 0,86 | (91,86; 95,45) | -3,15 | -1,78 | -1,88 |
| 10 | 100,49 | 97,31 | 0,86 | (95,52; 99,11) | 3,17 | 1,79 | 1,90 |
| 11 | 98,17 | 95,41 | 0,81 | (93,73; 97,08) | 2,76 | 1,53 | 1,59 |
| 12 | 97,60 | 99,07 | 0,81 | (97,39; 100,74) | -1,47 | -0,82 | -0,81 |
| 13 | 93,21 | 93,48 | 0,98 | (91,46; 95,51) | -0,27 | -0,16 | -0,15 |
| 14 | 94,53 | 97,15 | 0,98 | (95,12; 99,17) | -2,61 | -1,52 | -1,58 |
| 15 | 93,93 | 95,80 | 0,93 | (93,86; 97,74) | -1,87 | -1,08 | -1,08 |
| 16 | 99,12 | 99,46 | 0,93 | (97,53; 101,40) | -0,35 | -0,20 | -0,20 |
| 17 | 91,38 | 90,11 | 1,49 | (87,01; 93,22) | 1,27 | 0,98 | 0,98 |
| 18 | 97,43 | 97,44 | 1,49 | (94,33; 100,54) | -0,01 | -0,01 | -0,01 |
| 19 | 99,71 | 96,41 | 0,85 | (94,65; 98,17) | 3,30 | 1,85 | 1,98 |
| 20 | 95,79 | 96,44 | 0,97 | (94,43; 98,45) | -0,65 | -0,38 | -0,37 |
| 21 | 96,19 | 96,91 | 0,54 | (95,77; 98,04) | -0,72 | -0,38 | -0,37 |
| 22 | 97,12 | 97,22 | 0,54 | (96,08; 98,35) | -0,09 | -0,05 | -0,05 |
| 23 | 95,16 | 95,86 | 1,20 | (93,37; 98,35) | -0,69 | -0,44 | -0,43 |
| 24 | 98,74 | 98,16 | 1,11 | (95,85; 100,48) | 0,58 | 0,36 | 0,35 |
| 25 | 95,85 | 97,02 | 0,53 | (95,91; 98,13) | -1,17 | -0,62 | -0,61 |
| 26 | 98,74 | 97,02 | 0,53 | (95,91; 98,13) | 1,72 | 0,91 | 0,90 |
| 27 | 98,52 | 97,02 | 0,53 | (95,91; 98,13) | 1,51 | 0,79 | 0,79 |

| Obs | HI | Cook’s D | DFITS |
| --- | --- | --- | --- |
| 1 | 0,181848 | 0,00 | 0,09509 |
| 2 | 0,181848 | 0,01 | 0,22154 |
| 3 | 0,323174 | 0,07 | -0,63246 |
| 4 | 0,323174 | 0,01 | -0,22595 |
| 5 | 0,145676 | 0,07 | -0,66458 |
| 6 | 0,145676 | 0,01 | -0,24840 |
| 7 | 0,206416 | 0,10 | 0,80795 |
| 8 | 0,206416 | 0,01 | 0,23348 |
| 9 | 0,191815 | 0,12 | -0,91634 |
| 10 | 0,191815 | 0,13 | 0,92481 |
| 11 | 0,167095 | 0,08 | 0,71136 |
| 12 | 0,167095 | 0,02 | -0,36277 |
| 13 | 0,244597 | 0,00 | -0,08804 |
| 14 | 0,244597 | 0,13 | -0,89789 |
| 15 | 0,223583 | 0,06 | -0,58104 |
| 16 | 0,223583 | 0,00 | -0,10537 |
| 17 | 0,573092 | 0,22 | 1,14040 |
| 18 | 0,573092 | 0,00 | -0,00859 |
| 19 | 0,184894 | 0,13 | 0,94078 |
| 20 | 0,239930 | 0,01 | -0,20754 |
| 21 | 0,076218 | 0,00 | -0,10655 |
| 22 | 0,076343 | 0,00 | -0,01373 |
| 23 | 0,368545 | 0,02 | -0,33057 |
| 24 | 0,318905 | 0,01 | 0,23929 |
| 25 | 0,073524 | 0,01 | -0,17150 |
| 26 | 0,073524 | 0,01 | 0,25395 |
| 27 | 0,073524 | 0,01 | 0,22139 |

## Backward Elimination of Terms

α to remove = 0,1

## Coded Coefficients

| Term | Coef | SE Coef | 95% CI | T-Value | P-Value | VIF |
| --- | --- | --- | --- | --- | --- | --- |
| Constant | 97,709 | 0,557 | (96,550; 98,867) | 175,43 | 0,000 |  |
| Lac | 2,878 | 0,803 | (1,209; 4,547) | 3,59 | 0,002 | 1,00 |
| HPMC\_Visc | 0,633 | 0,807 | (-1,045; 2,311) | 0,78 | 0,441 | 1,33 |
| HPMC\_PS | 2,74 | 1,10 | (0,46; 5,02) | 2,50 | 0,021 | 1,34 |
| Lac\*Lac | -3,36 | 1,52 | (-6,53; -0,19) | -2,21 | 0,039 | 1,00 |
| HPMC\_Visc\*HPMC\_PS | 5,86 | 2,18 | (1,34; 10,39) | 2,69 | 0,014 | 1,25 |

## Model Summary

| S | R-sq | R-sq(adj) | PRESS | R-sq(pred) | AICc | BIC |
| --- | --- | --- | --- | --- | --- | --- |
| 1,96584 | 57,38% | 47,24% | 129,780 | 31,85% | 126,23 | 129,41 |

## Analysis of Variance

| Source | DF | Seq SS | Contribution | Adj SS | Adj MS | F-Value | P-Value |
| --- | --- | --- | --- | --- | --- | --- | --- |
| Model | 5 | 109,278 | 57,38% | 109,278 | 21,856 | 5,66 | 0,002 |
| Linear | 3 | 62,385 | 32,76% | 74,452 | 24,817 | 6,42 | 0,003 |
| Lac | 1 | 49,714 | 26,11% | 49,714 | 49,714 | 12,86 | 0,002 |
| HPMC\_Visc | 1 | 2,718 | 1,43% | 2,379 | 2,379 | 0,62 | 0,441 |
| HPMC\_PS | 1 | 9,953 | 5,23% | 24,194 | 24,194 | 6,26 | 0,021 |
| Square | 1 | 18,838 | 9,89% | 18,829 | 18,829 | 4,87 | 0,039 |
| Lac\*Lac | 1 | 18,838 | 9,89% | 18,829 | 18,829 | 4,87 | 0,039 |
| 2-Way Interaction | 1 | 28,055 | 14,73% | 28,055 | 28,055 | 7,26 | 0,014 |
| HPMC\_Visc\*HPMC\_PS | 1 | 28,055 | 14,73% | 28,055 | 28,055 | 7,26 | 0,014 |
| Error | 21 | 81,155 | 42,62% | 81,155 | 3,865 |  |  |
| Lack-of-Fit | 19 | 77,291 | 40,59% | 77,291 | 4,068 | 2,11 | 0,371 |
| Pure Error | 2 | 3,864 | 2,03% | 3,864 | 1,932 |  |  |
| Total | 26 | 190,433 | 100,00% |  |  |  |  |

## Regression Equation in Uncoded Units

|  |  |  |
| --- | --- | --- |
| F\_mean\_24h(1440min) | = | 243,2 + 65,3 Lac - 0,01411 HPMC\_Visc - 2,395 HPMC\_PS - 53,8 Lac\*Lac + 0,000205 HPMC\_Visc\*HPMC\_PS |

## Fits and Diagnostics for All Observations

| Obs | F\_mean\_24h(1440min) | Fit | SE Fit | 95% CI | Resid | Std Resid | Del Resid |
| --- | --- | --- | --- | --- | --- | --- | --- |
| 1 | 95,53 | 95,33 | 0,84 | (93,59; 97,08) | 0,20 | 0,11 | 0,11 |
| 2 | 98,99 | 98,21 | 0,84 | (96,47; 99,95) | 0,78 | 0,44 | 0,43 |
| 3 | 90,80 | 91,94 | 1,12 | (89,62; 94,26) | -1,14 | -0,71 | -0,70 |
| 4 | 94,17 | 94,82 | 1,12 | (92,49; 97,14) | -0,65 | -0,40 | -0,39 |
| 5 | 92,51 | 95,20 | 0,75 | (93,64; 96,76) | -2,69 | -1,48 | -1,53 |
| 6 | 97,09 | 98,08 | 0,75 | (96,52; 99,64) | -0,99 | -0,54 | -0,53 |
| 7 | 95,86 | 93,19 | 0,89 | (91,33; 95,05) | 2,67 | 1,53 | 1,58 |
| 8 | 96,74 | 96,07 | 0,89 | (94,21; 97,92) | 0,68 | 0,39 | 0,38 |
| 9 | 91,28 | 94,44 | 0,86 | (92,65; 96,24) | -3,17 | -1,79 | -1,90 |
| 10 | 100,51 | 97,32 | 0,86 | (95,53; 99,11) | 3,18 | 1,80 | 1,91 |
| 11 | 99,12 | 96,36 | 0,80 | (94,69; 98,03) | 2,76 | 1,54 | 1,59 |
| 12 | 97,59 | 99,24 | 0,80 | (97,57; 100,91) | -1,65 | -0,92 | -0,92 |
| 13 | 93,88 | 94,27 | 0,97 | (92,25; 96,30) | -0,39 | -0,23 | -0,22 |
| 14 | 94,32 | 97,15 | 0,97 | (95,13; 99,17) | -2,83 | -1,66 | -1,73 |
| 15 | 94,86 | 96,78 | 0,93 | (94,85; 98,71) | -1,93 | -1,11 | -1,12 |
| 16 | 99,27 | 99,66 | 0,93 | (97,73; 101,59) | -0,39 | -0,22 | -0,22 |
| 17 | 92,44 | 91,29 | 1,49 | (88,20; 94,39) | 1,15 | 0,90 | 0,89 |
| 18 | 97,29 | 97,05 | 1,49 | (93,95; 100,14) | 0,24 | 0,19 | 0,18 |
| 19 | 99,95 | 96,78 | 0,85 | (95,03; 98,54) | 3,17 | 1,79 | 1,89 |
| 20 | 96,30 | 97,14 | 0,96 | (95,13; 99,14) | -0,83 | -0,49 | -0,48 |
| 21 | 97,03 | 97,42 | 0,54 | (96,29; 98,55) | -0,39 | -0,21 | -0,20 |
| 22 | 97,28 | 97,75 | 0,54 | (96,62; 98,88) | -0,47 | -0,25 | -0,24 |
| 23 | 95,41 | 96,32 | 1,19 | (93,84; 98,81) | -0,92 | -0,59 | -0,58 |
| 24 | 99,43 | 98,70 | 1,11 | (96,39; 101,01) | 0,73 | 0,45 | 0,44 |
| 25 | 96,90 | 97,53 | 0,53 | (96,42; 98,64) | -0,63 | -0,33 | -0,33 |
| 26 | 99,48 | 97,53 | 0,53 | (96,42; 98,64) | 1,94 | 1,03 | 1,03 |
| 27 | 99,10 | 97,53 | 0,53 | (96,42; 98,64) | 1,57 | 0,83 | 0,82 |

| Obs | HI | Cook’s D | DFITS |
| --- | --- | --- | --- |
| 1 | 0,181848 | 0,00 | 0,05138 |
| 2 | 0,181848 | 0,01 | 0,20178 |
| 3 | 0,323174 | 0,04 | -0,48294 |
| 4 | 0,323174 | 0,01 | -0,27221 |
| 5 | 0,145676 | 0,06 | -0,63036 |
| 6 | 0,145676 | 0,01 | -0,22050 |
| 7 | 0,206416 | 0,10 | 0,80511 |
| 8 | 0,206416 | 0,01 | 0,19298 |
| 9 | 0,191815 | 0,13 | -0,92550 |
| 10 | 0,191815 | 0,13 | 0,93107 |
| 11 | 0,167095 | 0,08 | 0,71298 |
| 12 | 0,167095 | 0,03 | -0,41046 |
| 13 | 0,244597 | 0,00 | -0,12743 |
| 14 | 0,244597 | 0,15 | -0,98584 |
| 15 | 0,223583 | 0,06 | -0,60003 |
| 16 | 0,223583 | 0,00 | -0,11683 |
| 17 | 0,573092 | 0,18 | 1,03290 |
| 18 | 0,573092 | 0,01 | 0,21034 |
| 19 | 0,184894 | 0,12 | 0,90112 |
| 20 | 0,239930 | 0,01 | -0,26862 |
| 21 | 0,076218 | 0,00 | -0,05778 |
| 22 | 0,076343 | 0,00 | -0,06928 |
| 23 | 0,368545 | 0,03 | -0,44178 |
| 24 | 0,318905 | 0,02 | 0,30130 |
| 25 | 0,073524 | 0,00 | -0,09185 |
| 26 | 0,073524 | 0,01 | 0,28972 |
| 27 | 0,073524 | 0,01 | 0,23131 |
