# Supplementary material for: Influence of Selected Hypromellose Functionality-Related Characteristics and Soluble/Insoluble Filler Ratio on Carvedilol Release from Matrix Tablets
Source: Pharmaceutics. 2025 Oct 21;17(10):1358. doi: 10.3390/pharmaceutics17101358 (PMC12566823; doi:10.3390/pharmaceutics17101358)
Supplement: Supplementary file 1 [file pharmaceutics-17-01358-s001.zip › Report_Mean Release Analysis_RSM(CCD)_Stepwise, Forward Information Criteria (BIC).htm]

# Mean Release Analysis, Response Surface Design (Central Composite Design), Stepwise Regression - Forward, Information Criteria (BIC)

## Forward Selection of Terms

Achieved minimum BIC =  108,74

## Coded Coefficients

| Term | Coef | SE Coef | 95% CI | T-Value | P-Value | VIF |
| --- | --- | --- | --- | --- | --- | --- |
| Constant | 10,109 | 0,302 | (9,487; 10,731) | 33,49 | 0,000 |  |
| Lac | 2,621 | 0,640 | (1,302; 3,940) | 4,09 | 0,000 | 1,00 |

## Model Summary

| S | R-sq | R-sq(adj) | PRESS | R-sq(pred) | AICc | BIC |
| --- | --- | --- | --- | --- | --- | --- |
| 1,56868 | 40,13% | 37,73% | 69,7030 | 32,16% | 105,90 | 108,74 |

## Analysis of Variance

| Source | DF | Seq SS | Contribution | Adj SS | Adj MS | F-Value | P-Value |
| --- | --- | --- | --- | --- | --- | --- | --- |
| Model | 1 | 41,23 | 40,13% | 41,23 | 41,230 | 16,75 | 0,000 |
| Linear | 1 | 41,23 | 40,13% | 41,23 | 41,230 | 16,75 | 0,000 |
| Lac | 1 | 41,23 | 40,13% | 41,23 | 41,230 | 16,75 | 0,000 |
| Error | 25 | 61,52 | 59,87% | 61,52 | 2,461 |  |  |
| Lack-of-Fit | 23 | 47,49 | 46,22% | 47,49 | 2,065 | 0,29 | 0,949 |
| Pure Error | 2 | 14,03 | 13,65% | 14,03 | 7,015 |  |  |
| Total | 26 | 102,75 | 100,00% |  |  |  |  |

## Regression Equation in Uncoded Units

|  |  |  |
| --- | --- | --- |
| F\_mean\_0.17h(10min) | = | 4,87 + 10,49 Lac |

## Fits and Diagnostics for All Observations

| Obs | F\_mean\_0.17h(10min) | Fit | SE Fit | 95% CI | Resid | Std Resid | Del Resid |
| --- | --- | --- | --- | --- | --- | --- | --- |
| 1 | 9,095 | 8,798 | 0,440 | (7,892; 9,705) | 0,297 | 0,20 | 0,19 |
| 2 | 11,821 | 11,420 | 0,440 | (10,513; 12,326) | 0,402 | 0,27 | 0,26 |
| 3 | 7,323 | 8,798 | 0,440 | (7,892; 9,705) | -1,475 | -0,98 | -0,98 |
| 4 | 8,147 | 11,420 | 0,440 | (10,513; 12,326) | -3,272 | -2,17 | -2,36 |
| 5 | 7,099 | 8,798 | 0,440 | (7,892; 9,705) | -1,700 | -1,13 | -1,14 |
| 6 | 12,823 | 11,420 | 0,440 | (10,513; 12,326) | 1,403 | 0,93 | 0,93 |
| 7 | 7,484 | 8,798 | 0,440 | (7,892; 9,705) | -1,314 | -0,87 | -0,87 |
| 8 | 9,846 | 11,420 | 0,440 | (10,513; 12,326) | -1,574 | -1,05 | -1,05 |
| 9 | 8,623 | 8,798 | 0,440 | (7,892; 9,705) | -0,176 | -0,12 | -0,11 |
| 10 | 11,322 | 11,420 | 0,440 | (10,513; 12,326) | -0,098 | -0,07 | -0,06 |
| 11 | 9,398 | 8,798 | 0,440 | (7,892; 9,705) | 0,599 | 0,40 | 0,39 |
| 12 | 12,522 | 11,420 | 0,440 | (10,513; 12,326) | 1,102 | 0,73 | 0,73 |
| 13 | 7,874 | 8,798 | 0,440 | (7,892; 9,705) | -0,925 | -0,61 | -0,61 |
| 14 | 12,420 | 11,420 | 0,440 | (10,513; 12,326) | 1,001 | 0,66 | 0,66 |
| 15 | 10,497 | 8,798 | 0,440 | (7,892; 9,705) | 1,698 | 1,13 | 1,13 |
| 16 | 10,196 | 11,420 | 0,440 | (10,513; 12,326) | -1,223 | -0,81 | -0,81 |
| 17 | 8,246 | 7,488 | 0,708 | (6,030; 8,946) | 0,759 | 0,54 | 0,53 |
| 18 | 13,122 | 12,730 | 0,708 | (11,272; 14,189) | 0,391 | 0,28 | 0,27 |
| 19 | 9,647 | 10,109 | 0,302 | (9,487; 10,731) | -0,463 | -0,30 | -0,29 |
| 20 | 13,095 | 10,109 | 0,302 | (9,487; 10,731) | 2,986 | 1,94 | 2,06 |
| 21 | 10,472 | 10,109 | 0,302 | (9,487; 10,731) | 0,363 | 0,24 | 0,23 |
| 22 | 12,671 | 10,109 | 0,302 | (9,487; 10,731) | 2,562 | 1,66 | 1,73 |
| 23 | 9,297 | 10,109 | 0,302 | (9,487; 10,731) | -0,812 | -0,53 | -0,52 |
| 24 | 10,323 | 10,109 | 0,302 | (9,487; 10,731) | 0,214 | 0,14 | 0,14 |
| 25 | 8,148 | 10,109 | 0,302 | (9,487; 10,731) | -1,961 | -1,27 | -1,29 |
| 26 | 8,524 | 10,109 | 0,302 | (9,487; 10,731) | -1,585 | -1,03 | -1,03 |
| 27 | 12,912 | 10,109 | 0,302 | (9,487; 10,731) | 2,802 | 1,82 | 1,92 |

| Obs | HI | Cook’s D | DFITS |  |
| --- | --- | --- | --- | --- |
| 1 | 0,078704 | 0,00 | 0,056490 |  |
| 2 | 0,078704 | 0,00 | 0,076474 |  |
| 3 | 0,078704 | 0,04 | -0,286121 |  |
| 4 | 0,078704 | 0,20 | -0,691104 | R |
| 5 | 0,078704 | 0,05 | -0,331848 |  |
| 6 | 0,078704 | 0,04 | 0,271618 |  |
| 7 | 0,078704 | 0,03 | -0,253881 |  |
| 8 | 0,078704 | 0,05 | -0,306085 |  |
| 9 | 0,078704 | 0,00 | -0,033399 |  |
| 10 | 0,078704 | 0,00 | -0,018631 |  |
| 11 | 0,078704 | 0,01 | 0,114344 |  |
| 12 | 0,078704 | 0,02 | 0,211903 |  |
| 13 | 0,078704 | 0,02 | -0,177185 |  |
| 14 | 0,078704 | 0,02 | 0,192017 |  |
| 15 | 0,078704 | 0,05 | 0,331541 |  |
| 16 | 0,078704 | 0,03 | -0,235830 |  |
| 17 | 0,203704 | 0,04 | 0,270226 |  |
| 18 | 0,203704 | 0,01 | 0,138784 |  |
| 19 | 0,037037 | 0,00 | -0,057839 |  |
| 20 | 0,037037 | 0,07 | 0,404362 |  |
| 21 | 0,037037 | 0,00 | 0,045331 |  |
| 22 | 0,037037 | 0,05 | 0,339117 |  |
| 23 | 0,037037 | 0,01 | -0,101984 |  |
| 24 | 0,037037 | 0,00 | 0,026722 |  |
| 25 | 0,037037 | 0,03 | -0,253178 |  |
| 26 | 0,037037 | 0,02 | -0,202240 |  |
| 27 | 0,037037 | 0,06 | 0,375610 |  |

R  Large residual

## Forward Selection of Terms

Achieved minimum BIC =  119,56

## Coded Coefficients

| Term | Coef | SE Coef | 95% CI | T-Value | P-Value | VIF |
| --- | --- | --- | --- | --- | --- | --- |
| Constant | 13,846 | 0,354 | (13,114; 14,577) | 39,09 | 0,000 |  |
| Lac | 3,323 | 0,751 | (1,773; 4,874) | 4,42 | 0,000 | 1,00 |
| HPMC\_PS | 1,686 | 0,886 | (-0,142; 3,514) | 1,90 | 0,069 | 1,00 |

## Model Summary

| S | R-sq | R-sq(adj) | PRESS | R-sq(pred) | AICc | BIC |
| --- | --- | --- | --- | --- | --- | --- |
| 1,84024 | 49,14% | 44,90% | 96,3524 | 39,71% | 116,20 | 119,56 |

## Analysis of Variance

| Source | DF | Seq SS | Contribution | Adj SS | Adj MS | F-Value | P-Value |
| --- | --- | --- | --- | --- | --- | --- | --- |
| Model | 2 | 78,54 | 49,14% | 78,54 | 39,268 | 11,60 | 0,000 |
| Linear | 2 | 78,54 | 49,14% | 78,54 | 39,268 | 11,60 | 0,000 |
| Lac | 1 | 66,27 | 41,47% | 66,27 | 66,271 | 19,57 | 0,000 |
| HPMC\_PS | 1 | 12,26 | 7,67% | 12,26 | 12,265 | 3,62 | 0,069 |
| Error | 24 | 81,28 | 50,86% | 81,28 | 3,386 |  |  |
| Lack-of-Fit | 22 | 63,65 | 39,83% | 63,65 | 2,893 | 0,33 | 0,932 |
| Pure Error | 2 | 17,62 | 11,03% | 17,62 | 8,812 |  |  |
| Total | 26 | 159,81 | 100,00% |  |  |  |  |

## Regression Equation in Uncoded Units

|  |  |  |
| --- | --- | --- |
| F\_mean\_0.33h(20min) | = | -8,76 + 13,29 Lac + 0,229 HPMC\_PS |

## Fits and Diagnostics for All Observations

| Obs | F\_mean\_0.33h(20min) | Fit | SE Fit | 95% CI | Resid | Std Resid | Del Resid |
| --- | --- | --- | --- | --- | --- | --- | --- |
| 1 | 12,421 | 11,767 | 0,558 | (10,615; 12,919) | 0,654 | 0,37 | 0,37 |
| 2 | 16,290 | 15,090 | 0,558 | (13,939; 16,242) | 1,199 | 0,68 | 0,68 |
| 3 | 10,020 | 11,209 | 0,722 | (9,718; 12,699) | -1,189 | -0,70 | -0,69 |
| 4 | 11,393 | 14,532 | 0,722 | (13,041; 16,023) | -3,140 | -1,85 | -1,96 |
| 5 | 10,071 | 11,894 | 0,536 | (10,787; 13,001) | -1,823 | -1,04 | -1,04 |
| 6 | 17,069 | 15,218 | 0,536 | (14,111; 16,324) | 1,852 | 1,05 | 1,05 |
| 7 | 10,510 | 11,527 | 0,617 | (10,254; 12,801) | -1,017 | -0,59 | -0,58 |
| 8 | 13,414 | 14,851 | 0,617 | (13,577; 16,124) | -1,437 | -0,83 | -0,82 |
| 9 | 12,069 | 12,883 | 0,638 | (11,566; 14,199) | -0,814 | -0,47 | -0,46 |
| 10 | 15,416 | 16,206 | 0,638 | (14,890; 17,523) | -0,790 | -0,46 | -0,45 |
| 11 | 12,968 | 12,539 | 0,551 | (11,401; 13,677) | 0,429 | 0,24 | 0,24 |
| 12 | 16,492 | 15,862 | 0,551 | (14,724; 17,001) | 0,629 | 0,36 | 0,35 |
| 13 | 11,347 | 13,060 | 0,697 | (11,622; 14,498) | -1,714 | -1,01 | -1,01 |
| 14 | 16,913 | 16,384 | 0,697 | (14,946; 17,821) | 0,529 | 0,31 | 0,30 |
| 15 | 14,391 | 12,634 | 0,571 | (11,455; 13,812) | 1,757 | 1,00 | 1,00 |
| 16 | 13,890 | 15,957 | 0,571 | (14,779; 17,135) | -2,067 | -1,18 | -1,19 |
| 17 | 11,041 | 10,464 | 0,831 | (8,749; 12,179) | 0,577 | 0,35 | 0,34 |
| 18 | 17,442 | 17,111 | 0,831 | (15,396; 18,825) | 0,331 | 0,20 | 0,20 |
| 19 | 13,390 | 14,003 | 0,365 | (13,248; 14,757) | -0,612 | -0,34 | -0,33 |
| 20 | 17,586 | 13,609 | 0,373 | (12,840; 14,379) | 3,977 | 2,21 | 2,42 |
| 21 | 14,091 | 13,669 | 0,364 | (12,917; 14,421) | 0,423 | 0,23 | 0,23 |
| 22 | 17,139 | 13,937 | 0,358 | (13,197; 14,677) | 3,202 | 1,77 | 1,86 |
| 23 | 12,766 | 12,160 | 0,947 | (10,205; 14,115) | 0,606 | 0,38 | 0,38 |
| 24 | 14,769 | 15,531 | 0,961 | (13,548; 17,514) | -0,762 | -0,49 | -0,48 |
| 25 | 11,694 | 13,787 | 0,355 | (13,055; 14,520) | -2,093 | -1,16 | -1,17 |
| 26 | 11,921 | 13,787 | 0,355 | (13,055; 14,520) | -1,866 | -1,03 | -1,03 |
| 27 | 16,945 | 13,787 | 0,355 | (13,055; 14,520) | 3,158 | 1,75 | 1,83 |

| Obs | HI | Cook’s D | DFITS |  |
| --- | --- | --- | --- | --- |
| 1 | 0,091956 | 0,00 | 0,116515 |  |
| 2 | 0,091956 | 0,02 | 0,215184 |  |
| 3 | 0,154060 | 0,03 | -0,296456 |  |
| 4 | 0,154060 | 0,21 | -0,837261 |  |
| 5 | 0,084910 | 0,03 | -0,315946 |  |
| 6 | 0,084910 | 0,03 | 0,321144 |  |
| 7 | 0,112397 | 0,01 | -0,205806 |  |
| 8 | 0,112397 | 0,03 | -0,292870 |  |
| 9 | 0,120141 | 0,01 | -0,171394 |  |
| 10 | 0,120141 | 0,01 | -0,166298 |  |
| 11 | 0,089807 | 0,00 | 0,075310 |  |
| 12 | 0,089807 | 0,00 | 0,110514 |  |
| 13 | 0,143301 | 0,06 | -0,411558 |  |
| 14 | 0,143301 | 0,01 | 0,124595 |  |
| 15 | 0,096233 | 0,04 | 0,327787 |  |
| 16 | 0,096233 | 0,05 | -0,388944 |  |
| 17 | 0,203866 | 0,01 | 0,174515 |  |
| 18 | 0,203866 | 0,00 | 0,099992 |  |
| 19 | 0,039415 | 0,00 | -0,067462 |  |
| 20 | 0,041065 | 0,07 | 0,500720 | R |
| 21 | 0,039207 | 0,00 | 0,046398 |  |
| 22 | 0,037944 | 0,04 | 0,369933 |  |
| 23 | 0,264924 | 0,02 | 0,226383 |  |
| 24 | 0,272505 | 0,03 | -0,292231 |  |
| 25 | 0,037200 | 0,02 | -0,229617 |  |
| 26 | 0,037200 | 0,01 | -0,203417 |  |
| 27 | 0,037200 | 0,04 | 0,360306 |  |

R  Large residual

## Forward Selection of Terms

Achieved minimum BIC =  122,80

## Coded Coefficients

| Term | Coef | SE Coef | 95% CI | T-Value | P-Value | VIF |
| --- | --- | --- | --- | --- | --- | --- |
| Constant | 16,245 | 0,376 | (15,468; 17,021) | 43,19 | 0,000 |  |
| Lac | 3,740 | 0,798 | (2,093; 5,386) | 4,69 | 0,000 | 1,00 |
| HPMC\_PS | 1,909 | 0,940 | (-0,032; 3,850) | 2,03 | 0,054 | 1,00 |

## Model Summary

| S | R-sq | R-sq(adj) | PRESS | R-sq(pred) | AICc | BIC |
| --- | --- | --- | --- | --- | --- | --- |
| 1,95398 | 52,09% | 48,10% | 108,673 | 43,19% | 119,43 | 122,80 |

## Analysis of Variance

| Source | DF | Seq SS | Contribution | Adj SS | Adj MS | F-Value | P-Value |
| --- | --- | --- | --- | --- | --- | --- | --- |
| Model | 2 | 99,65 | 52,09% | 99,65 | 49,823 | 13,05 | 0,000 |
| Linear | 2 | 99,65 | 52,09% | 99,65 | 49,823 | 13,05 | 0,000 |
| Lac | 1 | 83,92 | 43,87% | 83,92 | 83,920 | 21,98 | 0,000 |
| HPMC\_PS | 1 | 15,73 | 8,22% | 15,73 | 15,727 | 4,12 | 0,054 |
| Error | 24 | 91,63 | 47,91% | 91,63 | 3,818 |  |  |
| Lack-of-Fit | 22 | 72,43 | 37,87% | 72,43 | 3,292 | 0,34 | 0,925 |
| Pure Error | 2 | 19,20 | 10,04% | 19,20 | 9,600 |  |  |
| Total | 26 | 191,28 | 100,00% |  |  |  |  |

## Regression Equation in Uncoded Units

|  |  |  |
| --- | --- | --- |
| F\_mean\_0.5h(30min) | = | -9,31 + 14,96 Lac + 0,260 HPMC\_PS |

## Fits and Diagnostics for All Observations

| Obs | F\_mean\_0.5h(30min) | Fit | SE Fit | 95% CI | Resid | Std Resid | Del Resid |
| --- | --- | --- | --- | --- | --- | --- | --- |
| 1 | 14,629 | 13,903 | 0,593 | (12,680; 15,125) | 0,726 | 0,39 | 0,38 |
| 2 | 18,932 | 17,642 | 0,593 | (16,420; 18,865) | 1,290 | 0,69 | 0,68 |
| 3 | 11,941 | 13,270 | 0,767 | (11,688; 14,853) | -1,329 | -0,74 | -0,73 |
| 4 | 13,736 | 17,010 | 0,767 | (15,427; 18,593) | -3,274 | -1,82 | -1,92 |
| 5 | 12,093 | 14,047 | 0,569 | (12,872; 15,222) | -1,954 | -1,05 | -1,05 |
| 6 | 19,640 | 17,787 | 0,569 | (16,611; 18,962) | 1,853 | 0,99 | 0,99 |
| 7 | 12,627 | 13,631 | 0,655 | (12,279; 14,983) | -1,004 | -0,55 | -0,54 |
| 8 | 15,806 | 17,371 | 0,655 | (16,019; 18,723) | -1,565 | -0,85 | -0,85 |
| 9 | 14,264 | 15,166 | 0,677 | (13,769; 16,564) | -0,902 | -0,49 | -0,48 |
| 10 | 17,960 | 18,906 | 0,677 | (17,508; 20,304) | -0,946 | -0,52 | -0,51 |
| 11 | 15,264 | 14,777 | 0,586 | (13,568; 15,985) | 0,487 | 0,26 | 0,26 |
| 12 | 18,986 | 18,517 | 0,586 | (17,308; 19,725) | 0,469 | 0,25 | 0,25 |
| 13 | 13,544 | 15,367 | 0,740 | (13,840; 16,894) | -1,823 | -1,01 | -1,01 |
| 14 | 19,878 | 19,107 | 0,740 | (17,580; 20,634) | 0,771 | 0,43 | 0,42 |
| 15 | 16,784 | 14,884 | 0,606 | (13,633; 16,135) | 1,900 | 1,02 | 1,02 |
| 16 | 16,332 | 18,624 | 0,606 | (17,373; 19,875) | -2,292 | -1,23 | -1,25 |
| 17 | 12,909 | 12,439 | 0,882 | (10,618; 14,259) | 0,470 | 0,27 | 0,26 |
| 18 | 20,286 | 19,918 | 0,882 | (18,097; 21,739) | 0,367 | 0,21 | 0,21 |
| 19 | 15,883 | 16,422 | 0,388 | (15,622; 17,223) | -0,539 | -0,28 | -0,28 |
| 20 | 20,126 | 15,977 | 0,396 | (15,160; 16,795) | 4,149 | 2,17 | 2,37 |
| 21 | 16,410 | 16,044 | 0,387 | (15,246; 16,843) | 0,366 | 0,19 | 0,19 |
| 22 | 19,930 | 16,348 | 0,381 | (15,563; 17,134) | 3,582 | 1,87 | 1,98 |
| 23 | 15,034 | 14,336 | 1,006 | (12,260; 16,412) | 0,698 | 0,42 | 0,41 |
| 24 | 17,365 | 18,153 | 1,020 | (16,048; 20,259) | -0,788 | -0,47 | -0,47 |
| 25 | 14,088 | 16,178 | 0,377 | (15,401; 16,956) | -2,090 | -1,09 | -1,09 |
| 26 | 14,218 | 16,178 | 0,377 | (15,401; 16,956) | -1,960 | -1,02 | -1,02 |
| 27 | 19,519 | 16,178 | 0,377 | (15,401; 16,956) | 3,340 | 1,74 | 1,82 |

| Obs | HI | Cook’s D | DFITS |  |
| --- | --- | --- | --- | --- |
| 1 | 0,091956 | 0,01 | 0,121897 |  |
| 2 | 0,091956 | 0,02 | 0,217967 |  |
| 3 | 0,154060 | 0,03 | -0,312616 |  |
| 4 | 0,154060 | 0,20 | -0,819815 |  |
| 5 | 0,084910 | 0,03 | -0,319015 |  |
| 6 | 0,084910 | 0,03 | 0,301931 |  |
| 7 | 0,112397 | 0,01 | -0,191121 |  |
| 8 | 0,112397 | 0,03 | -0,300785 |  |
| 9 | 0,120141 | 0,01 | -0,178972 |  |
| 10 | 0,120141 | 0,01 | -0,187840 |  |
| 11 | 0,089807 | 0,00 | 0,080372 |  |
| 12 | 0,089807 | 0,00 | 0,077455 |  |
| 13 | 0,143301 | 0,06 | -0,412427 |  |
| 14 | 0,143301 | 0,01 | 0,171387 |  |
| 15 | 0,096233 | 0,04 | 0,334031 |  |
| 16 | 0,096233 | 0,05 | -0,407227 |  |
| 17 | 0,203866 | 0,01 | 0,133736 |  |
| 18 | 0,203866 | 0,00 | 0,104432 |  |
| 19 | 0,039415 | 0,00 | -0,055950 |  |
| 20 | 0,041065 | 0,07 | 0,489857 | R |
| 21 | 0,039207 | 0,00 | 0,037774 |  |
| 22 | 0,037944 | 0,05 | 0,393071 |  |
| 23 | 0,264924 | 0,02 | 0,245731 |  |
| 24 | 0,272505 | 0,03 | -0,284749 |  |
| 25 | 0,037200 | 0,02 | -0,215155 |  |
| 26 | 0,037200 | 0,01 | -0,201166 |  |
| 27 | 0,037200 | 0,04 | 0,358696 |  |

R  Large residual

## Forward Selection of Terms

Achieved minimum BIC =  126,17

## Coded Coefficients

| Term | Coef | SE Coef | 95% CI | T-Value | P-Value | VIF |
| --- | --- | --- | --- | --- | --- | --- |
| Constant | 19,131 | 0,400 | (18,304; 19,957) | 47,78 | 0,000 |  |
| Lac | 4,179 | 0,849 | (2,427; 5,932) | 4,92 | 0,000 | 1,00 |
| HPMC\_PS | 2,14 | 1,00 | (0,08; 4,21) | 2,14 | 0,043 | 1,00 |

## Model Summary

| S | R-sq | R-sq(adj) | PRESS | R-sq(pred) | AICc | BIC |
| --- | --- | --- | --- | --- | --- | --- |
| 2,07986 | 54,56% | 50,77% | 123,662 | 45,87% | 122,81 | 126,17 |

## Analysis of Variance

| Source | DF | Seq SS | Contribution | Adj SS | Adj MS | F-Value | P-Value |
| --- | --- | --- | --- | --- | --- | --- | --- |
| Model | 2 | 124,64 | 54,56% | 124,64 | 62,320 | 14,41 | 0,000 |
| Linear | 2 | 124,64 | 54,56% | 124,64 | 62,320 | 14,41 | 0,000 |
| Lac | 1 | 104,81 | 45,88% | 104,81 | 104,807 | 24,23 | 0,000 |
| HPMC\_PS | 1 | 19,83 | 8,68% | 19,83 | 19,832 | 4,58 | 0,043 |
| Error | 24 | 103,82 | 45,44% | 103,82 | 4,326 |  |  |
| Lack-of-Fit | 22 | 82,95 | 36,31% | 82,95 | 3,770 | 0,36 | 0,915 |
| Pure Error | 2 | 20,87 | 9,14% | 20,87 | 10,436 |  |  |
| Total | 26 | 228,46 | 100,00% |  |  |  |  |

## Regression Equation in Uncoded Units

|  |  |  |
| --- | --- | --- |
| F\_mean\_0.75h(45min) | = | -9,53 + 16,72 Lac + 0,291 HPMC\_PS |

## Fits and Diagnostics for All Observations

| Obs | F\_mean\_0.75h(45min) | Fit | SE Fit | 95% CI | Resid | Std Resid | Del Resid |
| --- | --- | --- | --- | --- | --- | --- | --- |
| 1 | 17,369 | 16,511 | 0,631 | (15,209; 17,813) | 0,858 | 0,43 | 0,43 |
| 2 | 21,994 | 20,690 | 0,631 | (19,389; 21,992) | 1,304 | 0,66 | 0,65 |
| 3 | 14,259 | 15,801 | 0,816 | (14,116; 17,486) | -1,542 | -0,81 | -0,80 |
| 4 | 16,501 | 19,980 | 0,816 | (18,296; 21,665) | -3,480 | -1,82 | -1,92 |
| 5 | 14,687 | 16,673 | 0,606 | (15,422; 17,924) | -1,985 | -1,00 | -1,00 |
| 6 | 22,707 | 20,852 | 0,606 | (19,601; 22,103) | 1,855 | 0,93 | 0,93 |
| 7 | 15,190 | 16,206 | 0,697 | (14,767; 17,645) | -1,016 | -0,52 | -0,51 |
| 8 | 18,616 | 20,386 | 0,697 | (18,946; 21,825) | -1,770 | -0,90 | -0,90 |
| 9 | 16,906 | 17,930 | 0,721 | (16,442; 19,418) | -1,024 | -0,53 | -0,52 |
| 10 | 21,098 | 22,109 | 0,721 | (20,622; 23,597) | -1,011 | -0,52 | -0,51 |
| 11 | 18,055 | 17,493 | 0,623 | (16,206; 18,779) | 0,562 | 0,28 | 0,28 |
| 12 | 22,100 | 21,672 | 0,623 | (20,386; 22,959) | 0,428 | 0,22 | 0,21 |
| 13 | 16,264 | 18,155 | 0,787 | (16,530; 19,780) | -1,891 | -0,98 | -0,98 |
| 14 | 23,411 | 22,335 | 0,787 | (20,710; 23,960) | 1,077 | 0,56 | 0,55 |
| 15 | 19,597 | 17,613 | 0,645 | (16,281; 18,945) | 1,983 | 1,00 | 1,00 |
| 16 | 19,194 | 21,793 | 0,645 | (20,461; 23,124) | -2,599 | -1,31 | -1,34 |
| 17 | 15,271 | 14,877 | 0,939 | (12,939; 16,815) | 0,394 | 0,21 | 0,21 |
| 18 | 23,700 | 23,236 | 0,939 | (21,298; 25,174) | 0,464 | 0,25 | 0,24 |
| 19 | 18,969 | 19,330 | 0,413 | (18,478; 20,183) | -0,361 | -0,18 | -0,17 |
| 20 | 23,059 | 18,831 | 0,421 | (17,961; 19,700) | 4,229 | 2,08 | 2,24 |
| 21 | 19,298 | 18,906 | 0,412 | (18,056; 19,756) | 0,392 | 0,19 | 0,19 |
| 22 | 23,265 | 19,247 | 0,405 | (18,411; 20,084) | 4,018 | 1,97 | 2,11 |
| 23 | 17,796 | 16,987 | 1,071 | (14,778; 19,197) | 0,809 | 0,45 | 0,45 |
| 24 | 20,282 | 21,274 | 1,086 | (19,033; 23,515) | -0,992 | -0,56 | -0,55 |
| 25 | 16,879 | 19,056 | 0,401 | (18,229; 19,884) | -2,177 | -1,07 | -1,07 |
| 26 | 17,038 | 19,056 | 0,401 | (18,229; 19,884) | -2,019 | -0,99 | -0,99 |
| 27 | 22,552 | 19,056 | 0,401 | (18,229; 19,884) | 3,496 | 1,71 | 1,79 |

| Obs | HI | Cook’s D | DFITS |  |
| --- | --- | --- | --- | --- |
| 1 | 0,091956 | 0,01 | 0,135365 |  |
| 2 | 0,091956 | 0,01 | 0,206768 |  |
| 3 | 0,154060 | 0,04 | -0,341426 |  |
| 4 | 0,154060 | 0,20 | -0,818474 |  |
| 5 | 0,084910 | 0,03 | -0,303934 |  |
| 6 | 0,084910 | 0,03 | 0,283260 |  |
| 7 | 0,112397 | 0,01 | -0,181683 |  |
| 8 | 0,112397 | 0,03 | -0,320111 |  |
| 9 | 0,120141 | 0,01 | -0,191029 |  |
| 10 | 0,120141 | 0,01 | -0,188569 |  |
| 11 | 0,089807 | 0,00 | 0,087216 |  |
| 12 | 0,089807 | 0,00 | 0,066377 |  |
| 13 | 0,143301 | 0,05 | -0,401543 |  |
| 14 | 0,143301 | 0,02 | 0,225381 |  |
| 15 | 0,096233 | 0,04 | 0,327370 |  |
| 16 | 0,096233 | 0,06 | -0,435842 |  |
| 17 | 0,203866 | 0,00 | 0,105221 |  |
| 18 | 0,203866 | 0,01 | 0,123964 |  |
| 19 | 0,039415 | 0,00 | -0,035133 |  |
| 20 | 0,041065 | 0,06 | 0,464358 | R |
| 21 | 0,039207 | 0,00 | 0,038093 |  |
| 22 | 0,037944 | 0,05 | 0,418217 |  |
| 23 | 0,264924 | 0,02 | 0,267800 |  |
| 24 | 0,272505 | 0,04 | -0,337147 |  |
| 25 | 0,037200 | 0,01 | -0,210349 |  |
| 26 | 0,037200 | 0,01 | -0,194339 |  |
| 27 | 0,037200 | 0,04 | 0,351792 |  |

R  Large residual

## Forward Selection of Terms

Achieved minimum BIC =  128,40

## Coded Coefficients

| Term | Coef | SE Coef | 95% CI | T-Value | P-Value | VIF |
| --- | --- | --- | --- | --- | --- | --- |
| Constant | 21,616 | 0,417 | (20,755; 22,478) | 51,81 | 0,000 |  |
| Lac | 4,541 | 0,885 | (2,715; 6,368) | 5,13 | 0,000 | 1,00 |
| HPMC\_PS | 2,26 | 1,04 | (0,11; 4,42) | 2,17 | 0,040 | 1,00 |

## Model Summary

| S | R-sq | R-sq(adj) | PRESS | R-sq(pred) | AICc | BIC |
| --- | --- | --- | --- | --- | --- | --- |
| 2,16772 | 56,40% | 52,76% | 134,939 | 47,83% | 125,04 | 128,40 |

## Analysis of Variance

| Source | DF | Seq SS | Contribution | Adj SS | Adj MS | F-Value | P-Value |
| --- | --- | --- | --- | --- | --- | --- | --- |
| Model | 2 | 145,87 | 56,40% | 145,87 | 72,937 | 15,52 | 0,000 |
| Linear | 2 | 145,87 | 56,40% | 145,87 | 72,937 | 15,52 | 0,000 |
| Lac | 1 | 123,73 | 47,84% | 123,73 | 123,731 | 26,33 | 0,000 |
| HPMC\_PS | 1 | 22,14 | 8,56% | 22,14 | 22,144 | 4,71 | 0,040 |
| Error | 24 | 112,78 | 43,60% | 112,78 | 4,699 |  |  |
| Lack-of-Fit | 22 | 91,48 | 35,37% | 91,48 | 4,158 | 0,39 | 0,900 |
| Pure Error | 2 | 21,30 | 8,23% | 21,30 | 10,648 |  |  |
| Total | 26 | 258,65 | 100,00% |  |  |  |  |

## Regression Equation in Uncoded Units

|  |  |  |
| --- | --- | --- |
| F\_mean\_1h(60min) | = | -8,9 + 18,16 Lac + 0,308 HPMC\_PS |

## Fits and Diagnostics for All Observations

| Obs | F\_mean\_1h(60min) | Fit | SE Fit | 95% CI | Resid | Std Resid | Del Resid |
| --- | --- | --- | --- | --- | --- | --- | --- |
| 1 | 19,761 | 18,786 | 0,657 | (17,429; 20,142) | 0,976 | 0,47 | 0,46 |
| 2 | 24,653 | 23,327 | 0,657 | (21,970; 24,683) | 1,327 | 0,64 | 0,63 |
| 3 | 16,350 | 18,036 | 0,851 | (16,280; 19,792) | -1,685 | -0,85 | -0,84 |
| 4 | 18,938 | 22,577 | 0,851 | (20,821; 24,333) | -3,639 | -1,83 | -1,93 |
| 5 | 16,856 | 18,957 | 0,632 | (17,653; 20,260) | -2,101 | -1,01 | -1,01 |
| 6 | 25,374 | 23,498 | 0,632 | (22,194; 24,801) | 1,876 | 0,90 | 0,90 |
| 7 | 17,428 | 18,464 | 0,727 | (16,964; 19,963) | -1,036 | -0,51 | -0,50 |
| 8 | 21,048 | 23,005 | 0,727 | (21,505; 24,505) | -1,956 | -0,96 | -0,96 |
| 9 | 19,146 | 20,285 | 0,751 | (18,734; 21,836) | -1,139 | -0,56 | -0,55 |
| 10 | 23,860 | 24,826 | 0,751 | (23,276; 26,377) | -0,967 | -0,48 | -0,47 |
| 11 | 20,444 | 19,823 | 0,650 | (18,482; 21,164) | 0,621 | 0,30 | 0,29 |
| 12 | 24,688 | 24,364 | 0,650 | (23,024; 25,705) | 0,324 | 0,16 | 0,15 |
| 13 | 18,608 | 20,523 | 0,821 | (18,830; 22,217) | -1,915 | -0,95 | -0,95 |
| 14 | 26,317 | 25,064 | 0,821 | (23,371; 26,758) | 1,253 | 0,62 | 0,62 |
| 15 | 21,982 | 19,950 | 0,672 | (18,562; 21,338) | 2,032 | 0,99 | 0,99 |
| 16 | 21,578 | 24,491 | 0,672 | (23,104; 25,879) | -2,913 | -1,41 | -1,45 |
| 17 | 17,356 | 16,997 | 0,979 | (14,977; 19,017) | 0,359 | 0,19 | 0,18 |
| 18 | 26,662 | 26,079 | 0,979 | (24,059; 28,099) | 0,583 | 0,30 | 0,30 |
| 19 | 21,678 | 21,827 | 0,430 | (20,939; 22,716) | -0,149 | -0,07 | -0,07 |
| 20 | 25,490 | 21,299 | 0,439 | (20,393; 22,206) | 4,191 | 1,97 | 2,11 |
| 21 | 21,834 | 21,379 | 0,429 | (20,493; 22,265) | 0,456 | 0,21 | 0,21 |
| 22 | 26,073 | 21,740 | 0,422 | (20,868; 22,611) | 4,334 | 2,04 | 2,19 |
| 23 | 20,282 | 19,351 | 1,116 | (17,049; 21,654) | 0,930 | 0,50 | 0,49 |
| 24 | 22,774 | 23,881 | 1,132 | (21,546; 26,217) | -1,108 | -0,60 | -0,59 |
| 25 | 19,368 | 21,538 | 0,418 | (20,675; 22,401) | -2,170 | -1,02 | -1,02 |
| 26 | 19,506 | 21,538 | 0,418 | (20,675; 22,401) | -2,032 | -0,96 | -0,95 |
| 27 | 25,088 | 21,538 | 0,418 | (20,675; 22,401) | 3,550 | 1,67 | 1,74 |

| Obs | HI | Cook’s D | DFITS |  |
| --- | --- | --- | --- | --- |
| 1 | 0,091956 | 0,01 | 0,147829 |  |
| 2 | 0,091956 | 0,01 | 0,201836 |  |
| 3 | 0,154060 | 0,04 | -0,358470 |  |
| 4 | 0,154060 | 0,20 | -0,821667 |  |
| 5 | 0,084910 | 0,03 | -0,308815 |  |
| 6 | 0,084910 | 0,03 | 0,274524 |  |
| 7 | 0,112397 | 0,01 | -0,177615 |  |
| 8 | 0,112397 | 0,04 | -0,340263 |  |
| 9 | 0,120141 | 0,01 | -0,204035 |  |
| 10 | 0,120141 | 0,01 | -0,172804 |  |
| 11 | 0,089807 | 0,00 | 0,092517 |  |
| 12 | 0,089807 | 0,00 | 0,048141 |  |
| 13 | 0,143301 | 0,05 | -0,389678 |  |
| 14 | 0,143301 | 0,02 | 0,252041 |  |
| 15 | 0,096233 | 0,03 | 0,321554 |  |
| 16 | 0,096233 | 0,07 | -0,471669 |  |
| 17 | 0,203866 | 0,00 | 0,092001 |  |
| 18 | 0,203866 | 0,01 | 0,149625 |  |
| 19 | 0,039415 | 0,00 | -0,013897 |  |
| 20 | 0,041065 | 0,06 | 0,436971 |  |
| 21 | 0,039207 | 0,00 | 0,042463 |  |
| 22 | 0,037944 | 0,05 | 0,435773 | R |
| 23 | 0,264924 | 0,03 | 0,295658 |  |
| 24 | 0,272505 | 0,04 | -0,361636 |  |
| 25 | 0,037200 | 0,01 | -0,200714 |  |
| 26 | 0,037200 | 0,01 | -0,187382 |  |
| 27 | 0,037200 | 0,04 | 0,341571 |  |

R  Large residual

## Forward Selection of Terms

Achieved minimum BIC =  131,38

## Coded Coefficients

| Term | Coef | SE Coef | 95% CI | T-Value | P-Value | VIF |
| --- | --- | --- | --- | --- | --- | --- |
| Constant | 25,945 | 0,441 | (25,035; 26,855) | 58,85 | 0,000 |  |
| Lac | 5,104 | 0,935 | (3,174; 7,033) | 5,46 | 0,000 | 1,00 |
| HPMC\_PS | 2,47 | 1,10 | (0,19; 4,74) | 2,24 | 0,035 | 1,00 |

## Model Summary

| S | R-sq | R-sq(adj) | PRESS | R-sq(pred) | AICc | BIC |
| --- | --- | --- | --- | --- | --- | --- |
| 2,29048 | 59,18% | 55,77% | 151,544 | 50,86% | 128,01 | 131,38 |

## Analysis of Variance

| Source | DF | Seq SS | Contribution | Adj SS | Adj MS | F-Value | P-Value |
| --- | --- | --- | --- | --- | --- | --- | --- |
| Model | 2 | 182,51 | 59,18% | 182,51 | 91,254 | 17,39 | 0,000 |
| Linear | 2 | 182,51 | 59,18% | 182,51 | 91,254 | 17,39 | 0,000 |
| Lac | 1 | 156,28 | 50,67% | 156,28 | 156,277 | 29,79 | 0,000 |
| HPMC\_PS | 1 | 26,23 | 8,50% | 26,23 | 26,231 | 5,00 | 0,035 |
| Error | 24 | 125,91 | 40,82% | 125,91 | 5,246 |  |  |
| Lack-of-Fit | 22 | 103,50 | 33,56% | 103,50 | 4,705 | 0,42 | 0,884 |
| Pure Error | 2 | 22,41 | 7,27% | 22,41 | 11,206 |  |  |
| Total | 26 | 308,42 | 100,00% |  |  |  |  |

## Regression Equation in Uncoded Units

|  |  |  |
| --- | --- | --- |
| F\_mean\_1.5h(90min) | = | -7,6 + 20,41 Lac + 0,335 HPMC\_PS |

## Fits and Diagnostics for All Observations

| Obs | F\_mean\_1.5h(90min) | Fit | SE Fit | 95% CI | Resid | Std Resid | Del Resid |
| --- | --- | --- | --- | --- | --- | --- | --- |
| 1 | 23,960 | 22,783 | 0,695 | (21,350; 24,217) | 1,177 | 0,54 | 0,53 |
| 2 | 29,242 | 27,887 | 0,695 | (26,453; 29,320) | 1,356 | 0,62 | 0,61 |
| 3 | 20,030 | 21,967 | 0,899 | (20,111; 23,822) | -1,937 | -0,92 | -0,92 |
| 4 | 23,256 | 27,070 | 0,899 | (25,215; 28,926) | -3,814 | -1,81 | -1,91 |
| 5 | 20,814 | 22,969 | 0,667 | (21,592; 24,347) | -2,155 | -0,98 | -0,98 |
| 6 | 29,979 | 28,073 | 0,667 | (26,695; 29,450) | 1,906 | 0,87 | 0,87 |
| 7 | 21,354 | 22,433 | 0,768 | (20,848; 24,018) | -1,079 | -0,50 | -0,49 |
| 8 | 25,207 | 27,536 | 0,768 | (25,951; 29,121) | -2,329 | -1,08 | -1,08 |
| 9 | 23,222 | 24,415 | 0,794 | (22,777; 26,054) | -1,193 | -0,56 | -0,55 |
| 10 | 28,652 | 29,519 | 0,794 | (27,880; 31,157) | -0,867 | -0,40 | -0,40 |
| 11 | 24,520 | 23,912 | 0,686 | (22,496; 25,329) | 0,607 | 0,28 | 0,27 |
| 12 | 29,209 | 29,016 | 0,686 | (27,599; 30,433) | 0,193 | 0,09 | 0,09 |
| 13 | 22,644 | 24,675 | 0,867 | (22,885; 26,464) | -2,031 | -0,96 | -0,96 |
| 14 | 31,222 | 29,778 | 0,867 | (27,989; 31,568) | 1,444 | 0,68 | 0,67 |
| 15 | 26,099 | 24,051 | 0,711 | (22,584; 25,517) | 2,048 | 0,94 | 0,94 |
| 16 | 25,890 | 29,154 | 0,711 | (27,688; 30,621) | -3,264 | -1,50 | -1,54 |
| 17 | 21,094 | 20,756 | 1,034 | (18,621; 22,890) | 0,338 | 0,17 | 0,16 |
| 18 | 31,707 | 30,963 | 1,034 | (28,828; 33,097) | 0,745 | 0,36 | 0,36 |
| 19 | 26,440 | 26,174 | 0,455 | (25,236; 27,113) | 0,265 | 0,12 | 0,12 |
| 20 | 29,744 | 25,600 | 0,464 | (24,642; 26,558) | 4,145 | 1,85 | 1,95 |
| 21 | 26,102 | 25,686 | 0,454 | (24,750; 26,622) | 0,416 | 0,19 | 0,18 |
| 22 | 30,808 | 26,079 | 0,446 | (25,158; 27,000) | 4,730 | 2,11 | 2,28 |
| 23 | 24,646 | 23,480 | 1,179 | (21,046; 25,913) | 1,166 | 0,59 | 0,59 |
| 24 | 27,178 | 28,410 | 1,196 | (25,942; 30,878) | -1,232 | -0,63 | -0,62 |
| 25 | 23,641 | 25,859 | 0,442 | (24,948; 26,771) | -2,219 | -0,99 | -0,99 |
| 26 | 23,791 | 25,859 | 0,442 | (24,948; 26,771) | -2,068 | -0,92 | -0,92 |
| 27 | 29,512 | 25,859 | 0,442 | (24,948; 26,771) | 3,653 | 1,63 | 1,69 |

| Obs | HI | Cook’s D | DFITS |  |
| --- | --- | --- | --- | --- |
| 1 | 0,091956 | 0,01 | 0,169043 |  |
| 2 | 0,091956 | 0,01 | 0,195048 |  |
| 3 | 0,154060 | 0,05 | -0,391094 |  |
| 4 | 0,154060 | 0,20 | -0,814073 |  |
| 5 | 0,084910 | 0,03 | -0,299438 |  |
| 6 | 0,084910 | 0,02 | 0,263552 |  |
| 7 | 0,112397 | 0,01 | -0,175079 |  |
| 8 | 0,112397 | 0,05 | -0,385453 |  |
| 9 | 0,120141 | 0,01 | -0,202223 |  |
| 10 | 0,120141 | 0,01 | -0,146436 |  |
| 11 | 0,089807 | 0,00 | 0,085581 |  |
| 12 | 0,089807 | 0,00 | 0,027158 |  |
| 13 | 0,143301 | 0,05 | -0,391066 |  |
| 14 | 0,143301 | 0,03 | 0,275404 |  |
| 15 | 0,096233 | 0,03 | 0,306110 |  |
| 16 | 0,096233 | 0,08 | -0,502954 |  |
| 17 | 0,203866 | 0,00 | 0,082056 |  |
| 18 | 0,203866 | 0,01 | 0,180995 |  |
| 19 | 0,039415 | 0,00 | 0,023459 |  |
| 20 | 0,041065 | 0,05 | 0,404194 |  |
| 21 | 0,039207 | 0,00 | 0,036683 |  |
| 22 | 0,037944 | 0,06 | 0,453261 | R |
| 23 | 0,264924 | 0,04 | 0,351632 |  |
| 24 | 0,272505 | 0,05 | -0,380996 |  |
| 25 | 0,037200 | 0,01 | -0,193923 |  |
| 26 | 0,037200 | 0,01 | -0,180306 |  |
| 27 | 0,037200 | 0,03 | 0,331547 |  |

R  Large residual

## Forward Selection of Terms

Achieved minimum BIC =  133,47

## Coded Coefficients

| Term | Coef | SE Coef | 95% CI | T-Value | P-Value | VIF |
| --- | --- | --- | --- | --- | --- | --- |
| Constant | 29,744 | 0,458 | (28,798; 30,690) | 64,90 | 0,000 |  |
| Lac | 5,573 | 0,972 | (3,566; 7,579) | 5,73 | 0,000 | 1,00 |
| HPMC\_PS | 2,59 | 1,15 | (0,22; 4,96) | 2,26 | 0,033 | 1,00 |

## Model Summary

| S | R-sq | R-sq(adj) | PRESS | R-sq(pred) | AICc | BIC |
| --- | --- | --- | --- | --- | --- | --- |
| 2,38097 | 61,27% | 58,05% | 164,631 | 53,14% | 130,11 | 133,47 |

## Analysis of Variance

| Source | DF | Seq SS | Contribution | Adj SS | Adj MS | F-Value | P-Value |
| --- | --- | --- | --- | --- | --- | --- | --- |
| Model | 2 | 215,28 | 61,27% | 215,28 | 107,641 | 18,99 | 0,000 |
| Linear | 2 | 215,28 | 61,27% | 215,28 | 107,641 | 18,99 | 0,000 |
| Lac | 1 | 186,33 | 53,03% | 186,33 | 186,326 | 32,87 | 0,000 |
| HPMC\_PS | 1 | 28,96 | 8,24% | 28,96 | 28,956 | 5,11 | 0,033 |
| Error | 24 | 136,06 | 38,73% | 136,06 | 5,669 |  |  |
| Lack-of-Fit | 22 | 113,70 | 32,36% | 113,70 | 5,168 | 0,46 | 0,861 |
| Pure Error | 2 | 22,36 | 6,36% | 22,36 | 11,180 |  |  |
| Total | 26 | 351,34 | 100,00% |  |  |  |  |

## Regression Equation in Uncoded Units

|  |  |  |
| --- | --- | --- |
| F\_mean\_2h(120min) | = | -5,9 + 22,29 Lac + 0,352 HPMC\_PS |

## Fits and Diagnostics for All Observations

| Obs | F\_mean\_2h(120min) | Fit | SE Fit | 95% CI | Resid | Std Resid | Del Resid |
| --- | --- | --- | --- | --- | --- | --- | --- |
| 1 | 27,647 | 26,317 | 0,722 | (24,827; 27,807) | 1,330 | 0,59 | 0,58 |
| 2 | 33,275 | 31,890 | 0,722 | (30,399; 33,380) | 1,385 | 0,61 | 0,60 |
| 3 | 23,330 | 25,459 | 0,935 | (23,531; 27,388) | -2,130 | -0,97 | -0,97 |
| 4 | 27,018 | 31,032 | 0,935 | (29,103; 32,961) | -4,014 | -1,83 | -1,93 |
| 5 | 24,319 | 26,513 | 0,694 | (25,081; 27,944) | -2,194 | -0,96 | -0,96 |
| 6 | 33,930 | 32,085 | 0,694 | (30,653; 33,517) | 1,845 | 0,81 | 0,80 |
| 7 | 24,866 | 25,949 | 0,798 | (24,301; 27,596) | -1,083 | -0,48 | -0,47 |
| 8 | 28,958 | 31,521 | 0,798 | (29,874; 33,169) | -2,564 | -1,14 | -1,15 |
| 9 | 26,769 | 28,032 | 0,825 | (26,329; 29,735) | -1,263 | -0,57 | -0,56 |
| 10 | 32,839 | 33,604 | 0,825 | (31,901; 35,308) | -0,766 | -0,34 | -0,34 |
| 11 | 28,140 | 27,503 | 0,714 | (26,031; 28,976) | 0,637 | 0,28 | 0,27 |
| 12 | 33,175 | 33,076 | 0,714 | (31,603; 34,549) | 0,099 | 0,04 | 0,04 |
| 13 | 26,177 | 28,304 | 0,901 | (26,444; 30,164) | -2,127 | -0,97 | -0,96 |
| 14 | 35,420 | 33,877 | 0,901 | (32,017; 35,737) | 1,543 | 0,70 | 0,69 |
| 15 | 29,734 | 27,649 | 0,739 | (26,124; 29,173) | 2,085 | 0,92 | 0,92 |
| 16 | 29,696 | 33,222 | 0,739 | (31,697; 34,746) | -3,526 | -1,56 | -1,61 |
| 17 | 24,325 | 24,082 | 1,075 | (21,863; 26,300) | 0,244 | 0,11 | 0,11 |
| 18 | 36,098 | 35,227 | 1,075 | (33,008; 37,446) | 0,871 | 0,41 | 0,40 |
| 19 | 30,544 | 29,985 | 0,473 | (29,010; 30,961) | 0,559 | 0,24 | 0,23 |
| 20 | 33,491 | 29,381 | 0,482 | (28,385; 30,377) | 4,110 | 1,76 | 1,85 |
| 21 | 29,839 | 29,472 | 0,471 | (28,499; 30,445) | 0,367 | 0,16 | 0,15 |
| 22 | 34,911 | 29,885 | 0,464 | (28,928; 30,842) | 5,027 | 2,15 | 2,35 |
| 23 | 28,553 | 27,154 | 1,226 | (24,625; 29,683) | 1,399 | 0,69 | 0,68 |
| 24 | 31,053 | 32,334 | 1,243 | (29,769; 34,899) | -1,281 | -0,63 | -0,62 |
| 25 | 27,483 | 29,654 | 0,459 | (28,706; 30,602) | -2,171 | -0,93 | -0,93 |
| 26 | 27,597 | 29,654 | 0,459 | (28,706; 30,602) | -2,057 | -0,88 | -0,88 |
| 27 | 33,330 | 29,654 | 0,459 | (28,706; 30,602) | 3,676 | 1,57 | 1,63 |

| Obs | HI | Cook’s D | DFITS |  |
| --- | --- | --- | --- | --- |
| 1 | 0,091956 | 0,01 | 0,184000 |  |
| 2 | 0,091956 | 0,01 | 0,191659 |  |
| 3 | 0,154060 | 0,06 | -0,414521 |  |
| 4 | 0,154060 | 0,20 | -0,825719 |  |
| 5 | 0,084910 | 0,03 | -0,292926 |  |
| 6 | 0,084910 | 0,02 | 0,244885 |  |
| 7 | 0,112397 | 0,01 | -0,168949 |  |
| 8 | 0,112397 | 0,06 | -0,409414 |  |
| 9 | 0,120141 | 0,01 | -0,205973 |  |
| 10 | 0,120141 | 0,01 | -0,124352 |  |
| 11 | 0,089807 | 0,00 | 0,086361 |  |
| 12 | 0,089807 | 0,00 | 0,013373 |  |
| 13 | 0,143301 | 0,05 | -0,394218 |  |
| 14 | 0,143301 | 0,03 | 0,283231 |  |
| 15 | 0,096233 | 0,03 | 0,299575 |  |
| 16 | 0,096233 | 0,09 | -0,524843 |  |
| 17 | 0,203866 | 0,00 | 0,056886 |  |
| 18 | 0,203866 | 0,01 | 0,203756 |  |
| 19 | 0,039415 | 0,00 | 0,047520 |  |
| 20 | 0,041065 | 0,04 | 0,382700 |  |
| 21 | 0,039207 | 0,00 | 0,031086 |  |
| 22 | 0,037944 | 0,06 | 0,465821 | R |
| 23 | 0,264924 | 0,06 | 0,406771 |  |
| 24 | 0,272505 | 0,05 | -0,381056 |  |
| 25 | 0,037200 | 0,01 | -0,182152 |  |
| 26 | 0,037200 | 0,01 | -0,172226 |  |
| 27 | 0,037200 | 0,03 | 0,319733 |  |

R  Large residual

## Forward Selection of Terms

Achieved minimum BIC =  135,32

## Coded Coefficients

| Term | Coef | SE Coef | 95% CI | T-Value | P-Value | VIF |
| --- | --- | --- | --- | --- | --- | --- |
| Constant | 33,182 | 0,474 | (32,203; 34,161) | 69,97 | 0,000 |  |
| Lac | 5,96 | 1,01 | (3,88; 8,03) | 5,92 | 0,000 | 1,00 |
| HPMC\_PS | 2,67 | 1,19 | (0,23; 5,12) | 2,25 | 0,034 | 1,00 |

## Model Summary

| S | R-sq | R-sq(adj) | PRESS | R-sq(pred) | AICc | BIC |
| --- | --- | --- | --- | --- | --- | --- |
| 2,46370 | 62,60% | 59,48% | 177,155 | 54,51% | 131,95 | 135,32 |

## Analysis of Variance

| Source | DF | Seq SS | Contribution | Adj SS | Adj MS | F-Value | P-Value |
| --- | --- | --- | --- | --- | --- | --- | --- |
| Model | 2 | 243,79 | 62,60% | 243,79 | 121,897 | 20,08 | 0,000 |
| Linear | 2 | 243,79 | 62,60% | 243,79 | 121,897 | 20,08 | 0,000 |
| Lac | 1 | 212,96 | 54,68% | 212,96 | 212,960 | 35,09 | 0,000 |
| HPMC\_PS | 1 | 30,83 | 7,92% | 30,83 | 30,834 | 5,08 | 0,034 |
| Error | 24 | 145,68 | 37,40% | 145,68 | 6,070 |  |  |
| Lack-of-Fit | 22 | 123,63 | 31,74% | 123,63 | 5,620 | 0,51 | 0,836 |
| Pure Error | 2 | 22,04 | 5,66% | 22,04 | 11,022 |  |  |
| Total | 26 | 389,47 | 100,00% |  |  |  |  |

## Regression Equation in Uncoded Units

|  |  |  |
| --- | --- | --- |
| F\_mean\_2.5h(150min) | = | -4,0 + 23,83 Lac + 0,363 HPMC\_PS |

## Fits and Diagnostics for All Observations

| Obs | F\_mean\_2.5h(150min) | Fit | SE Fit | 95% CI | Resid | Std Resid | Del Resid |
| --- | --- | --- | --- | --- | --- | --- | --- |
| 1 | 30,966 | 29,542 | 0,747 | (28,000; 31,084) | 1,424 | 0,61 | 0,60 |
| 2 | 36,876 | 35,500 | 0,747 | (33,958; 37,042) | 1,377 | 0,59 | 0,58 |
| 3 | 26,375 | 28,657 | 0,967 | (26,661; 30,653) | -2,281 | -1,01 | -1,01 |
| 4 | 30,399 | 34,615 | 0,967 | (32,619; 36,610) | -4,215 | -1,86 | -1,97 |
| 5 | 27,471 | 29,744 | 0,718 | (28,262; 31,225) | -2,273 | -0,96 | -0,96 |
| 6 | 37,628 | 35,701 | 0,718 | (34,220; 37,183) | 1,926 | 0,82 | 0,81 |
| 7 | 28,090 | 29,162 | 0,826 | (27,457; 30,867) | -1,072 | -0,46 | -0,45 |
| 8 | 32,301 | 35,120 | 0,826 | (33,415; 36,824) | -2,818 | -1,21 | -1,23 |
| 9 | 29,986 | 31,312 | 0,854 | (29,549; 33,074) | -1,325 | -0,57 | -0,57 |
| 10 | 36,545 | 37,269 | 0,854 | (35,507; 39,032) | -0,724 | -0,31 | -0,31 |
| 11 | 31,457 | 30,766 | 0,738 | (29,243; 32,290) | 0,690 | 0,29 | 0,29 |
| 12 | 36,736 | 36,724 | 0,738 | (35,200; 38,248) | 0,012 | 0,01 | 0,01 |
| 13 | 29,407 | 31,593 | 0,933 | (29,668; 33,517) | -2,185 | -0,96 | -0,96 |
| 14 | 39,136 | 37,550 | 0,933 | (35,625; 39,475) | 1,585 | 0,70 | 0,69 |
| 15 | 33,013 | 30,916 | 0,764 | (29,339; 32,494) | 2,097 | 0,90 | 0,89 |
| 16 | 33,169 | 36,874 | 0,764 | (35,297; 38,451) | -3,705 | -1,58 | -1,64 |
| 17 | 27,300 | 27,132 | 1,112 | (24,836; 29,427) | 0,168 | 0,08 | 0,07 |
| 18 | 40,033 | 39,047 | 1,112 | (36,751; 41,343) | 0,986 | 0,45 | 0,44 |
| 19 | 34,291 | 33,431 | 0,489 | (32,421; 34,440) | 0,860 | 0,36 | 0,35 |
| 20 | 36,830 | 32,808 | 0,499 | (31,777; 33,838) | 4,023 | 1,67 | 1,74 |
| 21 | 33,170 | 32,901 | 0,488 | (31,895; 33,908) | 0,269 | 0,11 | 0,11 |
| 22 | 38,633 | 33,327 | 0,480 | (32,337; 34,318) | 5,306 | 2,20 | 2,40 |
| 23 | 32,129 | 30,509 | 1,268 | (27,892; 33,126) | 1,619 | 0,77 | 0,76 |
| 24 | 34,525 | 35,855 | 1,286 | (33,200; 38,509) | -1,330 | -0,63 | -0,62 |
| 25 | 30,945 | 33,089 | 0,475 | (32,109; 34,070) | -2,144 | -0,89 | -0,88 |
| 26 | 31,126 | 33,089 | 0,475 | (32,109; 34,070) | -1,964 | -0,81 | -0,81 |
| 27 | 36,783 | 33,089 | 0,475 | (32,109; 34,070) | 3,694 | 1,53 | 1,57 |

| Obs | HI | Cook’s D | DFITS |  |
| --- | --- | --- | --- | --- |
| 1 | 0,091956 | 0,01 | 0,190396 |  |
| 2 | 0,091956 | 0,01 | 0,184013 |  |
| 3 | 0,154060 | 0,06 | -0,429785 |  |
| 4 | 0,154060 | 0,21 | -0,840072 |  |
| 5 | 0,084910 | 0,03 | -0,293359 |  |
| 6 | 0,084910 | 0,02 | 0,247185 |  |
| 7 | 0,112397 | 0,01 | -0,161561 |  |
| 8 | 0,112397 | 0,06 | -0,436572 |  |
| 9 | 0,120141 | 0,01 | -0,208916 |  |
| 10 | 0,120141 | 0,00 | -0,113594 |  |
| 11 | 0,089807 | 0,00 | 0,090487 |  |
| 12 | 0,089807 | 0,00 | 0,001606 |  |
| 13 | 0,143301 | 0,05 | -0,391256 |  |
| 14 | 0,143301 | 0,03 | 0,281217 |  |
| 15 | 0,096233 | 0,03 | 0,290883 |  |
| 16 | 0,096233 | 0,09 | -0,533897 |  |
| 17 | 0,203866 | 0,00 | 0,037932 |  |
| 18 | 0,203866 | 0,02 | 0,223179 |  |
| 19 | 0,039415 | 0,00 | 0,070800 |  |
| 20 | 0,041065 | 0,04 | 0,359239 |  |
| 21 | 0,039207 | 0,00 | 0,022022 |  |
| 22 | 0,037944 | 0,06 | 0,477529 | R |
| 23 | 0,264924 | 0,07 | 0,456160 |  |
| 24 | 0,272505 | 0,05 | -0,382315 |  |
| 25 | 0,037200 | 0,01 | -0,173557 |  |
| 26 | 0,037200 | 0,01 | -0,158500 |  |
| 27 | 0,037200 | 0,03 | 0,309495 |  |

R  Large residual

## Forward Selection of Terms

Achieved minimum BIC =  136,35

## Coded Coefficients

| Term | Coef | SE Coef | 95% CI | T-Value | P-Value | VIF |
| --- | --- | --- | --- | --- | --- | --- |
| Constant | 36,354 | 0,483 | (35,357; 37,352) | 75,21 | 0,000 |  |
| Lac | 6,29 | 1,03 | (4,18; 8,41) | 6,14 | 0,000 | 1,00 |
| HPMC\_PS | 2,73 | 1,21 | (0,24; 5,23) | 2,26 | 0,033 | 1,00 |

## Model Summary

| S | R-sq | R-sq(adj) | PRESS | R-sq(pred) | AICc | BIC |
| --- | --- | --- | --- | --- | --- | --- |
| 2,51115 | 64,06% | 61,06% | 184,728 | 56,13% | 132,98 | 136,35 |

## Analysis of Variance

| Source | DF | Seq SS | Contribution | Adj SS | Adj MS | F-Value | P-Value |
| --- | --- | --- | --- | --- | --- | --- | --- |
| Model | 2 | 269,75 | 64,06% | 269,75 | 134,873 | 21,39 | 0,000 |
| Linear | 2 | 269,75 | 64,06% | 269,75 | 134,873 | 21,39 | 0,000 |
| Lac | 1 | 237,54 | 56,41% | 237,54 | 237,540 | 37,67 | 0,000 |
| HPMC\_PS | 1 | 32,21 | 7,65% | 32,21 | 32,207 | 5,11 | 0,033 |
| Error | 24 | 151,34 | 35,94% | 151,34 | 6,306 |  |  |
| Lack-of-Fit | 22 | 129,72 | 30,81% | 129,72 | 5,896 | 0,55 | 0,817 |
| Pure Error | 2 | 21,62 | 5,13% | 21,62 | 10,810 |  |  |
| Total | 26 | 421,09 | 100,00% |  |  |  |  |

## Regression Equation in Uncoded Units

|  |  |  |
| --- | --- | --- |
| F\_mean\_3h(180min) | = | -2,1 + 25,17 Lac + 0,371 HPMC\_PS |

## Fits and Diagnostics for All Observations

| Obs | F\_mean\_3h(180min) | Fit | SE Fit | 95% CI | Resid | Std Resid | Del Resid |
| --- | --- | --- | --- | --- | --- | --- | --- |
| 1 | 34,028 | 32,533 | 0,761 | (30,961; 34,104) | 1,496 | 0,63 | 0,62 |
| 2 | 40,223 | 38,825 | 0,761 | (37,253; 40,396) | 1,398 | 0,58 | 0,58 |
| 3 | 29,168 | 31,628 | 0,986 | (29,594; 33,662) | -2,460 | -1,07 | -1,07 |
| 4 | 33,625 | 37,920 | 0,986 | (35,886; 39,954) | -4,295 | -1,86 | -1,97 |
| 5 | 30,469 | 32,739 | 0,732 | (31,229; 34,249) | -2,270 | -0,94 | -0,94 |
| 6 | 40,923 | 39,031 | 0,732 | (37,521; 40,541) | 1,892 | 0,79 | 0,78 |
| 7 | 31,112 | 32,144 | 0,842 | (30,407; 33,882) | -1,033 | -0,44 | -0,43 |
| 8 | 35,438 | 38,436 | 0,842 | (36,699; 40,174) | -2,998 | -1,27 | -1,28 |
| 9 | 32,975 | 34,341 | 0,870 | (32,545; 36,138) | -1,366 | -0,58 | -0,57 |
| 10 | 39,972 | 40,633 | 0,870 | (38,837; 42,430) | -0,662 | -0,28 | -0,28 |
| 11 | 34,494 | 33,784 | 0,753 | (32,231; 35,337) | 0,710 | 0,30 | 0,29 |
| 12 | 39,969 | 40,076 | 0,753 | (38,523; 41,629) | -0,107 | -0,04 | -0,04 |
| 13 | 32,410 | 34,628 | 0,951 | (32,666; 36,590) | -2,218 | -0,95 | -0,95 |
| 14 | 42,546 | 40,920 | 0,951 | (38,958; 42,882) | 1,625 | 0,70 | 0,69 |
| 15 | 35,988 | 33,937 | 0,779 | (32,329; 35,545) | 2,051 | 0,86 | 0,85 |
| 16 | 36,412 | 40,229 | 0,779 | (38,622; 41,837) | -3,818 | -1,60 | -1,66 |
| 17 | 30,093 | 29,968 | 1,134 | (27,627; 32,308) | 0,125 | 0,06 | 0,05 |
| 18 | 43,614 | 42,552 | 1,134 | (40,212; 44,892) | 1,063 | 0,47 | 0,47 |
| 19 | 37,657 | 36,609 | 0,499 | (35,580; 37,638) | 1,048 | 0,43 | 0,42 |
| 20 | 39,839 | 35,972 | 0,509 | (34,921; 37,022) | 3,868 | 1,57 | 1,63 |
| 21 | 36,222 | 36,068 | 0,497 | (35,041; 37,094) | 0,154 | 0,06 | 0,06 |
| 22 | 42,049 | 36,503 | 0,489 | (35,493; 37,512) | 5,547 | 2,25 | 2,48 |
| 23 | 35,448 | 33,623 | 1,293 | (30,955; 36,290) | 1,825 | 0,85 | 0,84 |
| 24 | 37,768 | 39,086 | 1,311 | (36,380; 41,791) | -1,317 | -0,62 | -0,61 |
| 25 | 34,152 | 36,260 | 0,484 | (35,260; 37,259) | -2,107 | -0,86 | -0,85 |
| 26 | 34,401 | 36,260 | 0,484 | (35,260; 37,259) | -1,858 | -0,75 | -0,75 |
| 27 | 39,967 | 36,260 | 0,484 | (35,260; 37,259) | 3,708 | 1,50 | 1,55 |

| Obs | HI | Cook’s D | DFITS |  |
| --- | --- | --- | --- | --- |
| 1 | 0,091956 | 0,01 | 0,196338 |  |
| 2 | 0,091956 | 0,01 | 0,183327 |  |
| 3 | 0,154060 | 0,07 | -0,455917 |  |
| 4 | 0,154060 | 0,21 | -0,839827 |  |
| 5 | 0,084910 | 0,03 | -0,287127 |  |
| 6 | 0,084910 | 0,02 | 0,237948 |  |
| 7 | 0,112397 | 0,01 | -0,152641 |  |
| 8 | 0,112397 | 0,07 | -0,456984 |  |
| 9 | 0,120141 | 0,02 | -0,211340 |  |
| 10 | 0,120141 | 0,00 | -0,101775 |  |
| 11 | 0,089807 | 0,00 | 0,091353 |  |
| 12 | 0,089807 | 0,00 | -0,013776 |  |
| 13 | 0,143301 | 0,05 | -0,389544 |  |
| 14 | 0,143301 | 0,03 | 0,282855 |  |
| 15 | 0,096233 | 0,03 | 0,278713 |  |
| 16 | 0,096233 | 0,09 | -0,540463 |  |
| 17 | 0,203866 | 0,00 | 0,027736 |  |
| 18 | 0,203866 | 0,02 | 0,236096 |  |
| 19 | 0,039415 | 0,00 | 0,084768 |  |
| 20 | 0,041065 | 0,04 | 0,336425 |  |
| 21 | 0,039207 | 0,00 | 0,012393 |  |
| 22 | 0,037944 | 0,07 | 0,492967 | R |
| 23 | 0,264924 | 0,09 | 0,505906 |  |
| 24 | 0,272505 | 0,05 | -0,371447 |  |
| 25 | 0,037200 | 0,01 | -0,167149 |  |
| 26 | 0,037200 | 0,01 | -0,146866 |  |
| 27 | 0,037200 | 0,03 | 0,304265 |  |

R  Large residual

## Forward Selection of Terms

Achieved minimum BIC =  137,31

## Coded Coefficients

| Term | Coef | SE Coef | 95% CI | T-Value | P-Value | VIF |
| --- | --- | --- | --- | --- | --- | --- |
| Constant | 39,348 | 0,492 | (38,332; 40,364) | 79,96 | 0,000 |  |
| Lac | 6,62 | 1,04 | (4,46; 8,77) | 6,34 | 0,000 | 1,00 |
| HPMC\_PS | 2,77 | 1,23 | (0,23; 5,31) | 2,25 | 0,034 | 1,00 |

## Model Summary

| S | R-sq | R-sq(adj) | PRESS | R-sq(pred) | AICc | BIC |
| --- | --- | --- | --- | --- | --- | --- |
| 2,55653 | 65,35% | 62,46% | 192,158 | 57,55% | 133,95 | 137,31 |

## Analysis of Variance

| Source | DF | Seq SS | Contribution | Adj SS | Adj MS | F-Value | P-Value |
| --- | --- | --- | --- | --- | --- | --- | --- |
| Model | 2 | 295,79 | 65,35% | 295,79 | 147,896 | 22,63 | 0,000 |
| Linear | 2 | 295,79 | 65,35% | 295,79 | 147,896 | 22,63 | 0,000 |
| Lac | 1 | 262,71 | 58,04% | 262,71 | 262,710 | 40,20 | 0,000 |
| HPMC\_PS | 1 | 33,08 | 7,31% | 33,08 | 33,081 | 5,06 | 0,034 |
| Error | 24 | 156,86 | 34,65% | 156,86 | 6,536 |  |  |
| Lack-of-Fit | 22 | 135,19 | 29,87% | 135,19 | 6,145 | 0,57 | 0,805 |
| Pure Error | 2 | 21,67 | 4,79% | 21,67 | 10,835 |  |  |
| Total | 26 | 452,65 | 100,00% |  |  |  |  |

## Regression Equation in Uncoded Units

|  |  |  |
| --- | --- | --- |
| F\_mean\_3.5h(210min) | = | -0,1 + 26,47 Lac + 0,376 HPMC\_PS |

## Fits and Diagnostics for All Observations

| Obs | F\_mean\_3.5h(210min) | Fit | SE Fit | 95% CI | Resid | Std Resid | Del Resid |
| --- | --- | --- | --- | --- | --- | --- | --- |
| 1 | 36,866 | 35,355 | 0,775 | (33,755; 36,955) | 1,511 | 0,62 | 0,61 |
| 2 | 43,414 | 41,972 | 0,775 | (40,372; 43,572) | 1,442 | 0,59 | 0,58 |
| 3 | 31,856 | 34,438 | 1,003 | (32,367; 36,509) | -2,582 | -1,10 | -1,10 |
| 4 | 36,620 | 41,055 | 1,003 | (38,984; 43,126) | -4,435 | -1,89 | -2,00 |
| 5 | 33,315 | 35,564 | 0,745 | (34,026; 37,101) | -2,249 | -0,92 | -0,92 |
| 6 | 44,065 | 42,181 | 0,745 | (40,643; 43,718) | 1,884 | 0,77 | 0,76 |
| 7 | 33,956 | 34,961 | 0,857 | (33,192; 36,730) | -1,005 | -0,42 | -0,41 |
| 8 | 38,419 | 41,578 | 0,857 | (39,809; 43,347) | -3,159 | -1,31 | -1,33 |
| 9 | 35,760 | 37,188 | 0,886 | (35,359; 39,017) | -1,428 | -0,60 | -0,59 |
| 10 | 43,218 | 43,805 | 0,886 | (41,976; 45,634) | -0,587 | -0,24 | -0,24 |
| 11 | 37,353 | 36,623 | 0,766 | (35,042; 38,204) | 0,730 | 0,30 | 0,29 |
| 12 | 42,997 | 43,240 | 0,766 | (41,659; 44,821) | -0,243 | -0,10 | -0,10 |
| 13 | 35,261 | 37,479 | 0,968 | (35,481; 39,476) | -2,218 | -0,94 | -0,93 |
| 14 | 45,725 | 44,096 | 0,968 | (42,098; 46,093) | 1,629 | 0,69 | 0,68 |
| 15 | 38,758 | 36,778 | 0,793 | (35,142; 38,415) | 1,979 | 0,81 | 0,81 |
| 16 | 39,498 | 43,395 | 0,793 | (41,759; 45,032) | -3,897 | -1,60 | -1,66 |
| 17 | 32,680 | 32,635 | 1,154 | (30,253; 35,017) | 0,045 | 0,02 | 0,02 |
| 18 | 46,967 | 45,869 | 1,154 | (43,487; 48,252) | 1,098 | 0,48 | 0,47 |
| 19 | 40,842 | 39,606 | 0,508 | (38,558; 40,653) | 1,236 | 0,49 | 0,49 |
| 20 | 42,741 | 38,960 | 0,518 | (37,891; 40,030) | 3,781 | 1,51 | 1,55 |
| 21 | 39,143 | 39,058 | 0,506 | (38,013; 40,102) | 0,086 | 0,03 | 0,03 |
| 22 | 45,186 | 39,499 | 0,498 | (38,471; 40,526) | 5,687 | 2,27 | 2,50 |
| 23 | 38,661 | 36,580 | 1,316 | (33,864; 39,296) | 2,082 | 0,95 | 0,95 |
| 24 | 40,884 | 42,116 | 1,335 | (39,362; 44,871) | -1,233 | -0,57 | -0,56 |
| 25 | 37,155 | 39,252 | 0,493 | (38,234; 40,270) | -2,097 | -0,84 | -0,83 |
| 26 | 37,450 | 39,252 | 0,493 | (38,234; 40,270) | -1,802 | -0,72 | -0,71 |
| 27 | 42,998 | 39,252 | 0,493 | (38,234; 40,270) | 3,746 | 1,49 | 1,53 |

| Obs | HI | Cook’s D | DFITS |  |
| --- | --- | --- | --- | --- |
| 1 | 0,091956 | 0,01 | 0,194784 |  |
| 2 | 0,091956 | 0,01 | 0,185757 |  |
| 3 | 0,154060 | 0,07 | -0,470663 |  |
| 4 | 0,154060 | 0,22 | -0,853745 |  |
| 5 | 0,084910 | 0,03 | -0,279171 |  |
| 6 | 0,084910 | 0,02 | 0,232625 |  |
| 7 | 0,112397 | 0,01 | -0,145956 |  |
| 8 | 0,112397 | 0,07 | -0,474286 |  |
| 9 | 0,120141 | 0,02 | -0,217029 |  |
| 10 | 0,120141 | 0,00 | -0,088583 |  |
| 11 | 0,089807 | 0,00 | 0,092224 |  |
| 12 | 0,089807 | 0,00 | -0,030633 |  |
| 13 | 0,143301 | 0,05 | -0,382340 |  |
| 14 | 0,143301 | 0,03 | 0,278363 |  |
| 15 | 0,096233 | 0,02 | 0,263824 |  |
| 16 | 0,096233 | 0,09 | -0,542135 |  |
| 17 | 0,203866 | 0,00 | 0,009850 |  |
| 18 | 0,203866 | 0,02 | 0,239555 |  |
| 19 | 0,039415 | 0,00 | 0,098320 |  |
| 20 | 0,041065 | 0,03 | 0,321598 |  |
| 21 | 0,039207 | 0,00 | 0,006780 |  |
| 22 | 0,037944 | 0,07 | 0,497425 | R |
| 23 | 0,264924 | 0,11 | 0,568926 |  |
| 24 | 0,272505 | 0,04 | -0,341033 |  |
| 25 | 0,037200 | 0,01 | -0,163260 |  |
| 26 | 0,037200 | 0,01 | -0,139762 |  |
| 27 | 0,037200 | 0,03 | 0,301701 |  |

R  Large residual

## Forward Selection of Terms

Achieved minimum BIC =  137,75

## Coded Coefficients

| Term | Coef | SE Coef | 95% CI | T-Value | P-Value | VIF |
| --- | --- | --- | --- | --- | --- | --- |
| Constant | 42,172 | 0,496 | (41,148; 43,196) | 85,01 | 0,000 |  |
| Lac | 6,97 | 1,05 | (4,80; 9,14) | 6,62 | 0,000 | 1,00 |
| HPMC\_PS | 2,82 | 1,24 | (0,26; 5,38) | 2,27 | 0,032 | 1,00 |

## Model Summary

| S | R-sq | R-sq(adj) | PRESS | R-sq(pred) | AICc | BIC |
| --- | --- | --- | --- | --- | --- | --- |
| 2,57722 | 67,14% | 64,40% | 196,175 | 59,56% | 134,38 | 137,75 |

## Analysis of Variance

| Source | DF | Seq SS | Contribution | Adj SS | Adj MS | F-Value | P-Value |
| --- | --- | --- | --- | --- | --- | --- | --- |
| Model | 2 | 325,64 | 67,14% | 325,64 | 162,821 | 24,51 | 0,000 |
| Linear | 2 | 325,64 | 67,14% | 325,64 | 162,821 | 24,51 | 0,000 |
| Lac | 1 | 291,37 | 60,07% | 291,37 | 291,368 | 43,87 | 0,000 |
| HPMC\_PS | 1 | 34,27 | 7,07% | 34,27 | 34,273 | 5,16 | 0,032 |
| Error | 24 | 159,41 | 32,86% | 159,41 | 6,642 |  |  |
| Lack-of-Fit | 22 | 138,21 | 28,49% | 138,21 | 6,282 | 0,59 | 0,792 |
| Pure Error | 2 | 21,20 | 4,37% | 21,20 | 10,600 |  |  |
| Total | 26 | 485,05 | 100,00% |  |  |  |  |

## Regression Equation in Uncoded Units

|  |  |  |
| --- | --- | --- |
| F\_mean\_4h(240min) | = | 1,5 + 27,87 Lac + 0,383 HPMC\_PS |

## Fits and Diagnostics for All Observations

| Obs | F\_mean\_4h(240min) | Fit | SE Fit | 95% CI | Resid | Std Resid | Del Resid |
| --- | --- | --- | --- | --- | --- | --- | --- |
| 1 | 39,017 | 37,990 | 0,782 | (36,377; 39,603) | 1,026 | 0,42 | 0,41 |
| 2 | 46,450 | 44,959 | 0,782 | (43,346; 46,572) | 1,491 | 0,61 | 0,60 |
| 3 | 34,367 | 37,057 | 1,012 | (34,969; 39,145) | -2,690 | -1,13 | -1,14 |
| 4 | 39,535 | 44,026 | 1,012 | (41,938; 46,114) | -4,491 | -1,89 | -2,01 |
| 5 | 36,008 | 38,203 | 0,751 | (36,653; 39,753) | -2,195 | -0,89 | -0,89 |
| 6 | 46,980 | 45,172 | 0,751 | (43,622; 46,722) | 1,808 | 0,73 | 0,73 |
| 7 | 36,677 | 37,590 | 0,864 | (35,806; 39,373) | -0,912 | -0,38 | -0,37 |
| 8 | 41,293 | 44,558 | 0,864 | (42,775; 46,342) | -3,266 | -1,34 | -1,37 |
| 9 | 38,416 | 39,856 | 0,893 | (38,012; 41,700) | -1,440 | -0,60 | -0,59 |
| 10 | 46,211 | 46,825 | 0,893 | (44,981; 48,668) | -0,613 | -0,25 | -0,25 |
| 11 | 40,034 | 39,281 | 0,772 | (37,687; 40,875) | 0,753 | 0,31 | 0,30 |
| 12 | 45,897 | 46,250 | 0,772 | (44,656; 47,844) | -0,353 | -0,14 | -0,14 |
| 13 | 38,009 | 40,152 | 0,976 | (38,139; 42,166) | -2,143 | -0,90 | -0,89 |
| 14 | 48,773 | 47,121 | 0,976 | (45,107; 49,134) | 1,652 | 0,69 | 0,68 |
| 15 | 41,448 | 39,439 | 0,799 | (37,789; 41,089) | 2,008 | 0,82 | 0,81 |
| 16 | 42,454 | 46,408 | 0,799 | (44,758; 48,058) | -3,954 | -1,61 | -1,67 |
| 17 | 35,187 | 35,105 | 1,164 | (32,704; 37,507) | 0,081 | 0,04 | 0,03 |
| 18 | 50,190 | 49,043 | 1,164 | (46,641; 51,444) | 1,148 | 0,50 | 0,49 |
| 19 | 43,871 | 42,434 | 0,512 | (41,378; 43,490) | 1,437 | 0,57 | 0,56 |
| 20 | 45,413 | 41,777 | 0,522 | (40,699; 42,855) | 3,636 | 1,44 | 1,48 |
| 21 | 41,861 | 41,876 | 0,510 | (40,823; 42,929) | -0,015 | -0,01 | -0,01 |
| 22 | 48,167 | 42,325 | 0,502 | (41,289; 43,361) | 5,842 | 2,31 | 2,57 |
| 23 | 41,719 | 39,354 | 1,327 | (36,616; 42,092) | 2,365 | 1,07 | 1,07 |
| 24 | 43,771 | 44,989 | 1,345 | (42,213; 47,766) | -1,218 | -0,55 | -0,55 |
| 25 | 40,004 | 42,074 | 0,497 | (41,048; 43,100) | -2,070 | -0,82 | -0,81 |
| 26 | 40,421 | 42,074 | 0,497 | (41,048; 43,100) | -1,653 | -0,65 | -0,65 |
| 27 | 45,840 | 42,074 | 0,497 | (41,048; 43,100) | 3,766 | 1,49 | 1,53 |

| Obs | HI | Cook’s D | DFITS |  |
| --- | --- | --- | --- | --- |
| 1 | 0,091956 | 0,01 | 0,130687 |  |
| 2 | 0,091956 | 0,01 | 0,190608 |  |
| 3 | 0,154060 | 0,08 | -0,487412 |  |
| 4 | 0,154060 | 0,22 | -0,858199 |  |
| 5 | 0,084910 | 0,02 | -0,269984 |  |
| 6 | 0,084910 | 0,02 | 0,221184 |  |
| 7 | 0,112397 | 0,01 | -0,131272 |  |
| 8 | 0,112397 | 0,08 | -0,487236 |  |
| 9 | 0,120141 | 0,02 | -0,217052 |  |
| 10 | 0,120141 | 0,00 | -0,091913 |  |
| 11 | 0,089807 | 0,00 | 0,094298 |  |
| 12 | 0,089807 | 0,00 | -0,044179 |  |
| 13 | 0,143301 | 0,04 | -0,365890 |  |
| 14 | 0,143301 | 0,03 | 0,280127 |  |
| 15 | 0,096233 | 0,02 | 0,265585 |  |
| 16 | 0,096233 | 0,09 | -0,546035 |  |
| 17 | 0,203866 | 0,00 | 0,017536 |  |
| 18 | 0,203866 | 0,02 | 0,248539 |  |
| 19 | 0,039415 | 0,00 | 0,113608 |  |
| 20 | 0,041065 | 0,03 | 0,305345 |  |
| 21 | 0,039207 | 0,00 | -0,001168 |  |
| 22 | 0,037944 | 0,07 | 0,509535 | R |
| 23 | 0,264924 | 0,14 | 0,644663 |  |
| 24 | 0,272505 | 0,04 | -0,334211 |  |
| 25 | 0,037200 | 0,01 | -0,159770 |  |
| 26 | 0,037200 | 0,01 | -0,126932 |  |
| 27 | 0,037200 | 0,03 | 0,300798 |  |

R  Large residual

## Forward Selection of Terms

Achieved minimum BIC =  138,06

## Coded Coefficients

| Term | Coef | SE Coef | 95% CI | T-Value | P-Value | VIF |
| --- | --- | --- | --- | --- | --- | --- |
| Constant | 45,374 | 0,510 | (44,310; 46,438) | 88,98 | 0,000 |  |
| Lac | 6,728 | 0,942 | (4,763; 8,693) | 7,14 | 0,000 | 1,08 |
| HPMC\_Visc | -0,507 | 0,918 | (-2,423; 1,408) | -0,55 | 0,587 | 1,35 |
| HPMC\_HP | 1,287 | 0,912 | (-0,615; 3,189) | 1,41 | 0,173 | 1,03 |
| HPMC\_PS | 3,07 | 1,26 | (0,45; 5,69) | 2,45 | 0,024 | 1,37 |
| Lac\*HPMC\_Visc | -4,02 | 1,84 | (-7,86; -0,18) | -2,18 | 0,041 | 1,08 |
| HPMC\_Visc\*HPMC\_PS | 5,24 | 2,47 | (0,10; 10,39) | 2,13 | 0,046 | 1,25 |

## Model Summary

| S | R-sq | R-sq(adj) | PRESS | R-sq(pred) | AICc | BIC |
| --- | --- | --- | --- | --- | --- | --- |
| 2,22435 | 80,87% | 75,13% | 166,840 | 67,75% | 135,69 | 138,06 |

## Analysis of Variance

| Source | DF | Seq SS | Contribution | Adj SS | Adj MS | F-Value | P-Value |
| --- | --- | --- | --- | --- | --- | --- | --- |
| Model | 6 | 418,38 | 80,87% | 418,380 | 69,730 | 14,09 | 0,000 |
| Linear | 4 | 372,48 | 72,00% | 314,138 | 78,535 | 15,87 | 0,000 |
| Lac | 1 | 317,55 | 61,38% | 252,374 | 252,374 | 51,01 | 0,000 |
| HPMC\_Visc | 1 | 23,41 | 4,52% | 1,509 | 1,509 | 0,31 | 0,587 |
| HPMC\_HP | 1 | 16,21 | 3,13% | 9,861 | 9,861 | 1,99 | 0,173 |
| HPMC\_PS | 1 | 15,31 | 2,96% | 29,582 | 29,582 | 5,98 | 0,024 |
| 2-Way Interaction | 2 | 45,90 | 8,87% | 45,899 | 22,949 | 4,64 | 0,022 |
| Lac\*HPMC\_Visc | 1 | 23,55 | 4,55% | 23,549 | 23,549 | 4,76 | 0,041 |
| HPMC\_Visc\*HPMC\_PS | 1 | 22,35 | 4,32% | 22,350 | 22,350 | 4,52 | 0,046 |
| Error | 20 | 98,95 | 19,13% | 98,954 | 4,948 |  |  |
| Lack-of-Fit | 18 | 78,27 | 15,13% | 78,273 | 4,349 | 0,42 | 0,879 |
| Pure Error | 2 | 20,68 | 4,00% | 20,681 | 10,340 |  |  |
| Total | 26 | 517,33 | 100,00% |  |  |  |  |

## Regression Equation in Uncoded Units

|  |  |  |
| --- | --- | --- |
| F\_mean\_4.5h(270min) | = | 137,1 + 82,8 Lac - 0,01083 HPMC\_Visc + 1,268 HPMC\_HP - 2,06 HPMC\_PS - 0,00413 Lac\*HPMC\_Visc + 0,000183 HPMC\_Visc\*HPMC\_PS |

## Fits and Diagnostics for All Observations

| Obs | F\_mean\_4.5h(270min) | Fit | SE Fit | 95% CI | Resid | Std Resid | Del Resid |
| --- | --- | --- | --- | --- | --- | --- | --- |
| 1 | 41,49 | 40,18 | 1,18 | (37,70; 42,65) | 1,31 | 0,70 | 0,69 |
| 2 | 49,33 | 49,77 | 1,18 | (47,29; 52,24) | -0,43 | -0,23 | -0,23 |
| 3 | 36,77 | 38,21 | 1,55 | (34,97; 41,45) | -1,44 | -0,90 | -0,90 |
| 4 | 42,25 | 42,17 | 1,55 | (38,93; 45,41) | 0,08 | 0,05 | 0,05 |
| 5 | 38,57 | 41,36 | 1,04 | (39,20; 43,52) | -2,78 | -1,41 | -1,45 |
| 6 | 49,79 | 50,69 | 1,04 | (48,53; 52,85) | -0,90 | -0,46 | -0,45 |
| 7 | 39,26 | 41,04 | 1,40 | (38,13; 43,96) | -1,78 | -1,03 | -1,03 |
| 8 | 44,01 | 44,91 | 1,40 | (42,00; 47,83) | -0,90 | -0,52 | -0,51 |
| 9 | 40,92 | 39,86 | 1,21 | (37,34; 42,38) | 1,06 | 0,57 | 0,56 |
| 10 | 49,10 | 49,37 | 1,21 | (46,86; 51,89) | -0,27 | -0,15 | -0,14 |
| 11 | 42,66 | 42,54 | 1,04 | (40,37; 44,71) | 0,12 | 0,06 | 0,06 |
| 12 | 48,64 | 48,50 | 1,04 | (46,33; 50,67) | 0,14 | 0,07 | 0,07 |
| 13 | 40,53 | 41,17 | 1,31 | (38,43; 43,90) | -0,64 | -0,35 | -0,35 |
| 14 | 51,64 | 50,71 | 1,31 | (47,98; 53,45) | 0,93 | 0,52 | 0,51 |
| 15 | 44,03 | 43,87 | 1,13 | (41,51; 46,23) | 0,16 | 0,09 | 0,08 |
| 16 | 45,28 | 49,46 | 1,13 | (47,11; 51,82) | -4,18 | -2,18 | -2,44 |
| 17 | 37,54 | 37,84 | 1,03 | (35,70; 39,99) | -0,31 | -0,16 | -0,15 |
| 18 | 53,28 | 52,81 | 1,03 | (50,67; 54,95) | 0,48 | 0,24 | 0,24 |
| 19 | 46,77 | 45,58 | 0,88 | (43,74; 47,42) | 1,19 | 0,58 | 0,57 |
| 20 | 48,03 | 43,48 | 1,03 | (41,33; 45,63) | 4,55 | 2,31 | 2,62 |
| 21 | 44,42 | 43,96 | 0,92 | (42,04; 45,87) | 0,47 | 0,23 | 0,23 |
| 22 | 50,99 | 46,85 | 1,12 | (44,53; 49,18) | 4,14 | 2,15 | 2,39 |
| 23 | 44,70 | 43,70 | 1,31 | (40,97; 46,43) | 0,99 | 0,55 | 0,54 |
| 24 | 46,53 | 46,94 | 1,21 | (44,41; 49,47) | -0,41 | -0,22 | -0,22 |
| 25 | 42,72 | 45,33 | 0,47 | (44,35; 46,31) | -2,60 | -1,20 | -1,21 |
| 26 | 43,19 | 45,33 | 0,47 | (44,35; 46,31) | -2,14 | -0,98 | -0,98 |
| 27 | 48,51 | 45,33 | 0,47 | (44,35; 46,31) | 3,18 | 1,46 | 1,51 |

| Obs | HI | Cook’s D | DFITS |  |
| --- | --- | --- | --- | --- |
| 1 | 0,283627 | 0,03 | 0,43204 |  |
| 2 | 0,283627 | 0,00 | -0,14168 |  |
| 3 | 0,488210 | 0,11 | -0,87820 |  |
| 4 | 0,488210 | 0,00 | 0,04668 |  |
| 5 | 0,216779 | 0,08 | -0,76373 |  |
| 6 | 0,216779 | 0,01 | -0,23625 |  |
| 7 | 0,394639 | 0,10 | -0,83284 |  |
| 8 | 0,394639 | 0,03 | -0,41344 |  |
| 9 | 0,294244 | 0,02 | 0,35931 |  |
| 10 | 0,294244 | 0,00 | -0,09200 |  |
| 11 | 0,218366 | 0,00 | 0,03118 |  |
| 12 | 0,218366 | 0,00 | 0,03673 |  |
| 13 | 0,347162 | 0,01 | -0,25238 |  |
| 14 | 0,347162 | 0,02 | 0,36947 |  |
| 15 | 0,258117 | 0,00 | 0,04900 |  |
| 16 | 0,258117 | 0,24 | -1,43875 | R |
| 17 | 0,213198 | 0,00 | -0,07879 |  |
| 18 | 0,213198 | 0,00 | 0,12242 |  |
| 19 | 0,157071 | 0,01 | 0,24662 |  |
| 20 | 0,214445 | 0,21 | 1,37098 | R |
| 21 | 0,170686 | 0,00 | 0,10260 |  |
| 22 | 0,251394 | 0,22 | 1,38558 | R |
| 23 | 0,346058 | 0,02 | 0,39474 |  |
| 24 | 0,297564 | 0,00 | -0,14037 |  |
| 25 | 0,044701 | 0,01 | -0,26205 |  |
| 26 | 0,044701 | 0,01 | -0,21247 |  |
| 27 | 0,044701 | 0,01 | 0,32688 |  |

R  Large residual

## Forward Selection of Terms

Achieved minimum BIC =  138,39

## Coded Coefficients

| Term | Coef | SE Coef | 95% CI | T-Value | P-Value | VIF |
| --- | --- | --- | --- | --- | --- | --- |
| Constant | 47,979 | 0,513 | (46,908; 49,049) | 93,52 | 0,000 |  |
| Lac | 7,016 | 0,948 | (5,039; 8,993) | 7,40 | 0,000 | 1,08 |
| HPMC\_Visc | -0,616 | 0,924 | (-2,544; 1,311) | -0,67 | 0,512 | 1,35 |
| HPMC\_HP | 1,388 | 0,917 | (-0,526; 3,302) | 1,51 | 0,146 | 1,03 |
| HPMC\_PS | 2,97 | 1,26 | (0,33; 5,60) | 2,35 | 0,029 | 1,37 |
| Lac\*HPMC\_Visc | -4,10 | 1,85 | (-7,97; -0,24) | -2,21 | 0,039 | 1,08 |
| HPMC\_Visc\*HPMC\_PS | 5,19 | 2,48 | (0,02; 10,37) | 2,09 | 0,049 | 1,25 |

## Model Summary

| S | R-sq | R-sq(adj) | PRESS | R-sq(pred) | AICc | BIC |
| --- | --- | --- | --- | --- | --- | --- |
| 2,23791 | 81,73% | 76,25% | 169,487 | 69,09% | 136,02 | 138,39 |

## Analysis of Variance

| Source | DF | Seq SS | Contribution | Adj SS | Adj MS | F-Value | P-Value |
| --- | --- | --- | --- | --- | --- | --- | --- |
| Model | 6 | 448,14 | 81,73% | 448,144 | 74,691 | 14,91 | 0,000 |
| Linear | 4 | 401,69 | 73,26% | 337,717 | 84,429 | 16,86 | 0,000 |
| Lac | 1 | 344,22 | 62,78% | 274,445 | 274,445 | 54,80 | 0,000 |
| HPMC\_Visc | 1 | 25,47 | 4,65% | 2,229 | 2,229 | 0,45 | 0,512 |
| HPMC\_HP | 1 | 18,04 | 3,29% | 11,465 | 11,465 | 2,29 | 0,146 |
| HPMC\_PS | 1 | 13,96 | 2,55% | 27,662 | 27,662 | 5,52 | 0,029 |
| 2-Way Interaction | 2 | 46,46 | 8,47% | 46,455 | 23,228 | 4,64 | 0,022 |
| Lac\*HPMC\_Visc | 1 | 24,53 | 4,47% | 24,528 | 24,528 | 4,90 | 0,039 |
| HPMC\_Visc\*HPMC\_PS | 1 | 21,93 | 4,00% | 21,928 | 21,928 | 4,38 | 0,049 |
| Error | 20 | 100,17 | 18,27% | 100,165 | 5,008 |  |  |
| Lack-of-Fit | 18 | 79,90 | 14,57% | 79,901 | 4,439 | 0,44 | 0,869 |
| Pure Error | 2 | 20,26 | 3,70% | 20,265 | 10,132 |  |  |
| Total | 26 | 548,31 | 100,00% |  |  |  |  |

## Regression Equation in Uncoded Units

|  |  |  |
| --- | --- | --- |
| F\_mean\_5h(300min) | = | 137,3 + 85,1 Lac - 0,01069 HPMC\_Visc + 1,367 HPMC\_HP - 2,05 HPMC\_PS - 0,00422 Lac\*HPMC\_Visc + 0,000181 HPMC\_Visc\*HPMC\_PS |

## Fits and Diagnostics for All Observations

| Obs | F\_mean\_5h(300min) | Fit | SE Fit | 95% CI | Resid | Std Resid | Del Resid | HI |
| --- | --- | --- | --- | --- | --- | --- | --- | --- |
| 1 | 43,97 | 42,63 | 1,19 | (40,14; 45,11) | 1,35 | 0,71 | 0,70 | 0,283627 |
| 2 | 52,11 | 52,57 | 1,19 | (50,08; 55,05) | -0,46 | -0,24 | -0,24 | 0,283627 |
| 3 | 39,10 | 40,63 | 1,56 | (37,37; 43,89) | -1,53 | -0,95 | -0,95 | 0,488210 |
| 4 | 44,90 | 44,82 | 1,56 | (41,55; 48,08) | 0,09 | 0,05 | 0,05 | 0,488210 |
| 5 | 40,99 | 43,89 | 1,04 | (41,72; 46,07) | -2,91 | -1,47 | -1,51 | 0,216779 |
| 6 | 52,48 | 53,57 | 1,04 | (51,40; 55,75) | -1,10 | -0,55 | -0,54 | 0,216779 |
| 7 | 41,89 | 43,56 | 1,41 | (40,62; 46,49) | -1,66 | -0,96 | -0,95 | 0,394639 |
| 8 | 46,67 | 47,66 | 1,41 | (44,72; 50,59) | -0,98 | -0,57 | -0,56 | 0,394639 |
| 9 | 43,32 | 42,27 | 1,21 | (39,74; 44,81) | 1,05 | 0,56 | 0,55 | 0,294244 |
| 10 | 51,86 | 52,13 | 1,21 | (49,60; 54,66) | -0,27 | -0,14 | -0,14 | 0,294244 |
| 11 | 45,16 | 44,92 | 1,05 | (42,74; 47,10) | 0,24 | 0,12 | 0,12 | 0,218366 |
| 12 | 51,24 | 51,15 | 1,05 | (48,97; 53,33) | 0,08 | 0,04 | 0,04 | 0,218366 |
| 13 | 43,03 | 43,68 | 1,32 | (40,93; 46,43) | -0,66 | -0,36 | -0,36 | 0,347162 |
| 14 | 54,48 | 53,57 | 1,32 | (50,82; 56,32) | 0,90 | 0,50 | 0,49 | 0,347162 |
| 15 | 46,29 | 46,30 | 1,14 | (43,93; 48,67) | -0,01 | -0,00 | -0,00 | 0,258117 |
| 16 | 48,02 | 52,16 | 1,14 | (49,78; 54,53) | -4,13 | -2,14 | -2,38 | 0,258117 |
| 17 | 39,83 | 40,16 | 1,03 | (38,01; 42,32) | -0,33 | -0,17 | -0,16 | 0,213198 |
| 18 | 56,28 | 55,74 | 1,03 | (53,58; 57,89) | 0,54 | 0,27 | 0,27 | 0,213198 |
| 19 | 49,59 | 48,29 | 0,89 | (46,44; 50,14) | 1,30 | 0,63 | 0,62 | 0,157071 |
| 20 | 50,54 | 45,98 | 1,04 | (43,82; 48,14) | 4,56 | 2,30 | 2,61 | 0,214445 |
| 21 | 46,88 | 46,49 | 0,92 | (44,56; 48,42) | 0,40 | 0,19 | 0,19 | 0,170686 |
| 22 | 53,74 | 49,57 | 1,12 | (47,23; 51,91) | 4,17 | 2,16 | 2,40 | 0,251394 |
| 23 | 47,57 | 46,42 | 1,32 | (43,67; 49,16) | 1,15 | 0,64 | 0,63 | 0,346058 |
| 24 | 49,16 | 49,47 | 1,22 | (46,93; 52,02) | -0,31 | -0,16 | -0,16 | 0,297564 |
| 25 | 45,31 | 47,95 | 0,47 | (46,96; 48,94) | -2,64 | -1,20 | -1,22 | 0,044701 |
| 26 | 45,93 | 47,95 | 0,47 | (46,96; 48,94) | -2,02 | -0,92 | -0,92 | 0,044701 |
| 27 | 51,11 | 47,95 | 0,47 | (46,96; 48,94) | 3,16 | 1,44 | 1,49 | 0,044701 |

| Obs | Cook’s D | DFITS |  |
| --- | --- | --- | --- |
| 1 | 0,03 | 0,44126 |  |
| 2 | 0,00 | -0,14814 |  |
| 3 | 0,12 | -0,92820 |  |
| 4 | 0,00 | 0,05123 |  |
| 5 | 0,09 | -0,79631 |  |
| 6 | 0,01 | -0,28604 |  |
| 7 | 0,09 | -0,76997 |  |
| 8 | 0,03 | -0,44828 |  |
| 9 | 0,02 | 0,35332 |  |
| 10 | 0,00 | -0,09045 |  |
| 11 | 0,00 | 0,06282 |  |
| 12 | 0,00 | 0,02199 |  |
| 13 | 0,01 | -0,25896 |  |
| 14 | 0,02 | 0,35772 |  |
| 15 | 0,00 | -0,00217 |  |
| 16 | 0,23 | -1,40418 | R |
| 17 | 0,00 | -0,08438 |  |
| 18 | 0,00 | 0,13820 |  |
| 19 | 0,01 | 0,26970 |  |
| 20 | 0,21 | 1,36410 | R |
| 21 | 0,00 | 0,08592 |  |
| 22 | 0,22 | 1,38918 | R |
| 23 | 0,03 | 0,45575 |  |
| 24 | 0,00 | -0,10448 |  |
| 25 | 0,01 | -0,26378 |  |
| 26 | 0,01 | -0,19887 |  |
| 27 | 0,01 | 0,32189 |  |

R  Large residual

## Forward Selection of Terms

Achieved minimum BIC =  138,33

## Coded Coefficients

| Term | Coef | SE Coef | 95% CI | T-Value | P-Value | VIF |
| --- | --- | --- | --- | --- | --- | --- |
| Constant | 50,498 | 0,512 | (49,429; 51,567) | 98,54 | 0,000 |  |
| Lac | 7,273 | 0,947 | (5,299; 9,248) | 7,68 | 0,000 | 1,08 |
| HPMC\_Visc | -0,726 | 0,923 | (-2,651; 1,200) | -0,79 | 0,441 | 1,35 |
| HPMC\_HP | 1,478 | 0,916 | (-0,433; 3,390) | 1,61 | 0,122 | 1,03 |
| HPMC\_PS | 2,89 | 1,26 | (0,26; 5,53) | 2,29 | 0,033 | 1,37 |
| Lac\*HPMC\_Visc | -4,17 | 1,85 | (-8,03; -0,31) | -2,25 | 0,036 | 1,08 |
| HPMC\_Visc\*HPMC\_PS | 5,26 | 2,48 | (0,09; 10,43) | 2,12 | 0,047 | 1,25 |

## Model Summary

| S | R-sq | R-sq(adj) | PRESS | R-sq(pred) | AICc | BIC |
| --- | --- | --- | --- | --- | --- | --- |
| 2,23543 | 82,69% | 77,50% | 169,592 | 70,63% | 135,96 | 138,33 |

## Analysis of Variance

| Source | DF | Seq SS | Contribution | Adj SS | Adj MS | F-Value | P-Value |
| --- | --- | --- | --- | --- | --- | --- | --- |
| Model | 6 | 477,40 | 82,69% | 477,404 | 79,567 | 15,92 | 0,000 |
| Linear | 4 | 429,57 | 74,40% | 360,540 | 90,135 | 18,04 | 0,000 |
| Lac | 1 | 368,86 | 63,89% | 294,947 | 294,947 | 59,02 | 0,000 |
| HPMC\_Visc | 1 | 28,15 | 4,88% | 3,089 | 3,089 | 0,62 | 0,441 |
| HPMC\_HP | 1 | 19,80 | 3,43% | 13,004 | 13,004 | 2,60 | 0,122 |
| HPMC\_PS | 1 | 12,75 | 2,21% | 26,311 | 26,311 | 5,27 | 0,033 |
| 2-Way Interaction | 2 | 47,84 | 8,29% | 47,839 | 23,919 | 4,79 | 0,020 |
| Lac\*HPMC\_Visc | 1 | 25,34 | 4,39% | 25,339 | 25,339 | 5,07 | 0,036 |
| HPMC\_Visc\*HPMC\_PS | 1 | 22,50 | 3,90% | 22,500 | 22,500 | 4,50 | 0,047 |
| Error | 20 | 99,94 | 17,31% | 99,943 | 4,997 |  |  |
| Lack-of-Fit | 18 | 80,15 | 13,88% | 80,146 | 4,453 | 0,45 | 0,863 |
| Pure Error | 2 | 19,80 | 3,43% | 19,797 | 9,899 |  |  |
| Total | 26 | 577,35 | 100,00% |  |  |  |  |

## Regression Equation in Uncoded Units

|  |  |  |
| --- | --- | --- |
| F\_mean\_5.5h(330min) | = | 141,3 + 87,0 Lac - 0,01085 HPMC\_Visc + 1,456 HPMC\_HP - 2,09 HPMC\_PS - 0,00429 Lac\*HPMC\_Visc + 0,000184 HPMC\_Visc\*HPMC\_PS |

## Fits and Diagnostics for All Observations

| Obs | F\_mean\_5.5h(330min) | Fit | SE Fit | 95% CI | Resid | Std Resid | Del Resid |
| --- | --- | --- | --- | --- | --- | --- | --- |
| 1 | 46,43 | 45,04 | 1,19 | (42,55; 47,52) | 1,39 | 0,74 | 0,73 |
| 2 | 54,76 | 55,28 | 1,19 | (52,80; 57,76) | -0,52 | -0,28 | -0,27 |
| 3 | 41,35 | 42,92 | 1,56 | (39,66; 46,17) | -1,56 | -0,98 | -0,97 |
| 4 | 47,45 | 47,31 | 1,56 | (44,06; 50,57) | 0,14 | 0,09 | 0,09 |
| 5 | 43,40 | 46,37 | 1,04 | (44,20; 48,54) | -2,97 | -1,50 | -1,56 |
| 6 | 55,11 | 56,35 | 1,04 | (54,18; 58,52) | -1,24 | -0,63 | -0,62 |
| 7 | 44,34 | 45,95 | 1,40 | (43,02; 48,87) | -1,61 | -0,92 | -0,92 |
| 8 | 49,21 | 50,26 | 1,40 | (47,33; 53,19) | -1,05 | -0,60 | -0,59 |
| 9 | 45,59 | 44,60 | 1,21 | (42,08; 47,13) | 0,99 | 0,53 | 0,52 |
| 10 | 54,54 | 54,77 | 1,21 | (52,24; 57,29) | -0,23 | -0,12 | -0,12 |
| 11 | 47,60 | 47,24 | 1,04 | (45,06; 49,42) | 0,37 | 0,18 | 0,18 |
| 12 | 53,70 | 53,72 | 1,04 | (51,54; 55,90) | -0,02 | -0,01 | -0,01 |
| 13 | 45,42 | 46,10 | 1,32 | (43,35; 48,85) | -0,68 | -0,38 | -0,37 |
| 14 | 57,19 | 56,30 | 1,32 | (53,55; 59,04) | 0,89 | 0,49 | 0,48 |
| 15 | 48,59 | 48,67 | 1,14 | (46,30; 51,04) | -0,07 | -0,04 | -0,04 |
| 16 | 50,74 | 54,77 | 1,14 | (52,40; 57,13) | -4,03 | -2,09 | -2,31 |
| 17 | 42,07 | 42,43 | 1,03 | (40,28; 44,58) | -0,36 | -0,18 | -0,18 |
| 18 | 59,14 | 58,54 | 1,03 | (56,39; 60,70) | 0,59 | 0,30 | 0,29 |
| 19 | 52,30 | 50,89 | 0,89 | (49,05; 52,74) | 1,41 | 0,69 | 0,68 |
| 20 | 52,87 | 48,38 | 1,04 | (46,22; 50,54) | 4,49 | 2,27 | 2,56 |
| 21 | 49,29 | 48,95 | 0,92 | (47,02; 50,87) | 0,34 | 0,17 | 0,16 |
| 22 | 56,38 | 52,18 | 1,12 | (49,85; 54,52) | 4,20 | 2,17 | 2,42 |
| 23 | 50,31 | 49,05 | 1,32 | (46,31; 51,79) | 1,26 | 0,70 | 0,69 |
| 24 | 51,67 | 51,91 | 1,22 | (49,37; 54,46) | -0,25 | -0,13 | -0,13 |
| 25 | 47,85 | 50,49 | 0,47 | (49,50; 51,47) | -2,64 | -1,21 | -1,22 |
| 26 | 48,52 | 50,49 | 0,47 | (49,50; 51,47) | -1,97 | -0,90 | -0,90 |
| 27 | 53,60 | 50,49 | 0,47 | (49,50; 51,47) | 3,12 | 1,43 | 1,47 |

| Obs | HI | Cook’s D | DFITS |  |
| --- | --- | --- | --- | --- |
| 1 | 0,283627 | 0,03 | 0,45726 |  |
| 2 | 0,283627 | 0,00 | -0,17003 |  |
| 3 | 0,488210 | 0,13 | -0,95180 |  |
| 4 | 0,488210 | 0,00 | 0,08360 |  |
| 5 | 0,216779 | 0,09 | -0,81823 |  |
| 6 | 0,216779 | 0,02 | -0,32555 |  |
| 7 | 0,394639 | 0,08 | -0,74261 |  |
| 8 | 0,394639 | 0,03 | -0,48005 |  |
| 9 | 0,294244 | 0,02 | 0,33344 |  |
| 10 | 0,294244 | 0,00 | -0,07560 |  |
| 11 | 0,218366 | 0,00 | 0,09539 |  |
| 12 | 0,218366 | 0,00 | -0,00409 |  |
| 13 | 0,347162 | 0,01 | -0,26933 |  |
| 14 | 0,347162 | 0,02 | 0,35214 |  |
| 15 | 0,258117 | 0,00 | -0,02236 |  |
| 16 | 0,258117 | 0,22 | -1,36009 | R |
| 17 | 0,213198 | 0,00 | -0,09168 |  |
| 18 | 0,213198 | 0,00 | 0,15244 |  |
| 19 | 0,157071 | 0,01 | 0,29217 |  |
| 20 | 0,214445 | 0,20 | 1,33993 | R |
| 21 | 0,170686 | 0,00 | 0,07457 |  |
| 22 | 0,251394 | 0,23 | 1,40169 | R |
| 23 | 0,346058 | 0,04 | 0,50108 |  |
| 24 | 0,297564 | 0,00 | -0,08318 |  |
| 25 | 0,044701 | 0,01 | -0,26421 |  |
| 26 | 0,044701 | 0,01 | -0,19380 |  |
| 27 | 0,044701 | 0,01 | 0,31733 |  |

R  Large residual

## Forward Selection of Terms

Achieved minimum BIC =  138,36

## Coded Coefficients

| Term | Coef | SE Coef | 95% CI | T-Value | P-Value | VIF |
| --- | --- | --- | --- | --- | --- | --- |
| Constant | 52,952 | 0,513 | (51,882; 54,021) | 103,27 | 0,000 |  |
| Lac | 7,507 | 0,947 | (5,531; 9,483) | 7,92 | 0,000 | 1,08 |
| HPMC\_Visc | -0,802 | 0,923 | (-2,728; 1,125) | -0,87 | 0,396 | 1,35 |
| HPMC\_HP | 1,570 | 0,917 | (-0,343; 3,482) | 1,71 | 0,102 | 1,03 |
| HPMC\_PS | 2,86 | 1,26 | (0,23; 5,49) | 2,27 | 0,035 | 1,37 |
| Lac\*HPMC\_Visc | -4,19 | 1,85 | (-8,06; -0,33) | -2,26 | 0,035 | 1,08 |
| HPMC\_Visc\*HPMC\_PS | 5,39 | 2,48 | (0,22; 10,57) | 2,17 | 0,042 | 1,25 |

## Model Summary

| S | R-sq | R-sq(adj) | PRESS | R-sq(pred) | AICc | BIC |
| --- | --- | --- | --- | --- | --- | --- |
| 2,23671 | 83,46% | 78,50% | 170,227 | 71,86% | 135,99 | 138,36 |

## Analysis of Variance

| Source | DF | Seq SS | Contribution | Adj SS | Adj MS | F-Value | P-Value |
| --- | --- | --- | --- | --- | --- | --- | --- |
| Model | 6 | 504,87 | 83,46% | 504,874 | 84,146 | 16,82 | 0,000 |
| Linear | 4 | 455,62 | 75,32% | 382,684 | 95,671 | 19,12 | 0,000 |
| Lac | 1 | 391,44 | 64,71% | 314,185 | 314,185 | 62,80 | 0,000 |
| HPMC\_Visc | 1 | 30,45 | 5,03% | 3,770 | 3,770 | 0,75 | 0,396 |
| HPMC\_HP | 1 | 21,79 | 3,60% | 14,666 | 14,666 | 2,93 | 0,102 |
| HPMC\_PS | 1 | 11,95 | 1,98% | 25,723 | 25,723 | 5,14 | 0,035 |
| 2-Way Interaction | 2 | 49,25 | 8,14% | 49,250 | 24,625 | 4,92 | 0,018 |
| Lac\*HPMC\_Visc | 1 | 25,60 | 4,23% | 25,597 | 25,597 | 5,12 | 0,035 |
| HPMC\_Visc\*HPMC\_PS | 1 | 23,65 | 3,91% | 23,653 | 23,653 | 4,73 | 0,042 |
| Error | 20 | 100,06 | 16,54% | 100,058 | 5,003 |  |  |
| Lack-of-Fit | 18 | 80,89 | 13,37% | 80,887 | 4,494 | 0,47 | 0,853 |
| Pure Error | 2 | 19,17 | 3,17% | 19,170 | 9,585 |  |  |
| Total | 26 | 604,93 | 100,00% |  |  |  |  |

## Regression Equation in Uncoded Units

|  |  |  |
| --- | --- | --- |
| F\_mean\_6h(360min) | = | 147,2 + 88,3 Lac - 0,01118 HPMC\_Visc + 1,547 HPMC\_HP - 2,16 HPMC\_PS - 0,00431 Lac\*HPMC\_Visc + 0,000188 HPMC\_Visc\*HPMC\_PS |

## Fits and Diagnostics for All Observations

| Obs | F\_mean\_6h(360min) | Fit | SE Fit | 95% CI | Resid | Std Resid | Del Resid | HI |
| --- | --- | --- | --- | --- | --- | --- | --- | --- |
| 1 | 48,90 | 47,39 | 1,19 | (44,90; 49,87) | 1,51 | 0,80 | 0,79 | 0,283627 |
| 2 | 57,28 | 57,88 | 1,19 | (55,39; 60,36) | -0,60 | -0,32 | -0,31 | 0,283627 |
| 3 | 43,50 | 45,10 | 1,56 | (41,84; 48,36) | -1,60 | -1,00 | -1,00 | 0,488210 |
| 4 | 49,90 | 49,72 | 1,56 | (46,46; 52,98) | 0,18 | 0,11 | 0,11 | 0,488210 |
| 5 | 45,73 | 48,79 | 1,04 | (46,62; 50,96) | -3,06 | -1,55 | -1,60 | 0,216779 |
| 6 | 57,66 | 59,02 | 1,04 | (56,85; 61,19) | -1,35 | -0,68 | -0,67 | 0,216779 |
| 7 | 46,73 | 48,26 | 1,41 | (45,32; 51,19) | -1,53 | -0,88 | -0,87 | 0,394639 |
| 8 | 51,68 | 52,78 | 1,41 | (49,85; 55,72) | -1,10 | -0,63 | -0,62 | 0,394639 |
| 9 | 47,79 | 46,87 | 1,21 | (44,34; 49,40) | 0,91 | 0,49 | 0,48 | 0,294244 |
| 10 | 57,12 | 57,28 | 1,21 | (54,75; 59,81) | -0,16 | -0,09 | -0,09 | 0,294244 |
| 11 | 50,00 | 49,52 | 1,05 | (47,34; 51,70) | 0,48 | 0,24 | 0,24 | 0,218366 |
| 12 | 56,14 | 56,23 | 1,05 | (54,05; 58,41) | -0,09 | -0,04 | -0,04 | 0,218366 |
| 13 | 47,76 | 48,45 | 1,32 | (45,71; 51,20) | -0,70 | -0,39 | -0,38 | 0,347162 |
| 14 | 59,76 | 58,90 | 1,32 | (56,15; 61,65) | 0,86 | 0,48 | 0,47 | 0,347162 |
| 15 | 50,89 | 51,00 | 1,14 | (48,63; 53,37) | -0,11 | -0,06 | -0,06 | 0,258117 |
| 16 | 53,40 | 57,33 | 1,14 | (54,96; 59,70) | -3,93 | -2,04 | -2,23 | 0,258117 |
| 17 | 44,23 | 44,66 | 1,03 | (42,51; 46,81) | -0,43 | -0,21 | -0,21 | 0,213198 |
| 18 | 61,87 | 61,25 | 1,03 | (59,09; 63,40) | 0,63 | 0,32 | 0,31 | 0,213198 |
| 19 | 54,96 | 53,40 | 0,89 | (51,55; 55,25) | 1,56 | 0,76 | 0,75 | 0,157071 |
| 20 | 55,19 | 50,72 | 1,04 | (48,56; 52,88) | 4,47 | 2,25 | 2,54 | 0,214445 |
| 21 | 51,57 | 51,33 | 0,92 | (49,40; 53,26) | 0,24 | 0,12 | 0,11 | 0,170686 |
| 22 | 58,92 | 54,74 | 1,12 | (52,40; 57,08) | 4,18 | 2,16 | 2,41 | 0,251394 |
| 23 | 52,92 | 51,58 | 1,32 | (48,84; 54,33) | 1,34 | 0,74 | 0,73 | 0,346058 |
| 24 | 54,10 | 54,30 | 1,22 | (51,76; 56,85) | -0,21 | -0,11 | -0,11 | 0,297564 |
| 25 | 50,29 | 52,95 | 0,47 | (51,97; 53,94) | -2,67 | -1,22 | -1,24 | 0,044701 |
| 26 | 51,08 | 52,95 | 0,47 | (51,97; 53,94) | -1,87 | -0,86 | -0,85 | 0,044701 |
| 27 | 56,00 | 52,95 | 0,47 | (51,97; 53,94) | 3,05 | 1,39 | 1,43 | 0,044701 |

| Obs | Cook’s D | DFITS |  |
| --- | --- | --- | --- |
| 1 | 0,04 | 0,49680 |  |
| 2 | 0,01 | -0,19543 |  |
| 3 | 0,14 | -0,97695 |  |
| 4 | 0,00 | 0,10734 |  |
| 5 | 0,09 | -0,84431 |  |
| 6 | 0,02 | -0,35508 |  |
| 7 | 0,07 | -0,70534 |  |
| 8 | 0,04 | -0,50399 |  |
| 9 | 0,01 | 0,30820 |  |
| 10 | 0,00 | -0,05525 |  |
| 11 | 0,00 | 0,12468 |  |
| 12 | 0,00 | -0,02293 |  |
| 13 | 0,01 | -0,27493 |  |
| 14 | 0,02 | 0,34181 |  |
| 15 | 0,00 | -0,03356 |  |
| 16 | 0,21 | -1,31817 | R |
| 17 | 0,00 | -0,10882 |  |
| 18 | 0,00 | 0,16060 |  |
| 19 | 0,02 | 0,32409 |  |
| 20 | 0,20 | 1,32933 | R |
| 21 | 0,00 | 0,05180 |  |
| 22 | 0,22 | 1,39462 | R |
| 23 | 0,04 | 0,53293 |  |
| 24 | 0,00 | -0,07045 |  |
| 25 | 0,01 | -0,26746 |  |
| 26 | 0,00 | -0,18374 |  |
| 27 | 0,01 | 0,30946 |  |

R  Large residual

## Forward Selection of Terms

Achieved minimum BIC =  137,97

## Coded Coefficients

| Term | Coef | SE Coef | 95% CI | T-Value | P-Value | VIF |
| --- | --- | --- | --- | --- | --- | --- |
| Constant | 57,558 | 0,509 | (56,496; 58,619) | 113,06 | 0,000 |  |
| Lac | 7,985 | 0,940 | (6,024; 9,947) | 8,49 | 0,000 | 1,08 |
| HPMC\_Visc | -0,970 | 0,917 | (-2,882; 0,943) | -1,06 | 0,303 | 1,35 |
| HPMC\_HP | 1,858 | 0,910 | (-0,041; 3,757) | 2,04 | 0,055 | 1,03 |
| HPMC\_PS | 2,65 | 1,25 | (0,04; 5,26) | 2,11 | 0,047 | 1,37 |
| Lac\*HPMC\_Visc | -4,44 | 1,84 | (-8,28; -0,60) | -2,41 | 0,026 | 1,08 |
| HPMC\_Visc\*HPMC\_PS | 5,12 | 2,46 | (-0,02; 10,26) | 2,08 | 0,051 | 1,25 |

## Model Summary

| S | R-sq | R-sq(adj) | PRESS | R-sq(pred) | AICc | BIC |
| --- | --- | --- | --- | --- | --- | --- |
| 2,22067 | 85,11% | 80,64% | 168,760 | 74,52% | 135,60 | 137,97 |

## Analysis of Variance

| Source | DF | Seq SS | Contribution | Adj SS | Adj MS | F-Value | P-Value |
| --- | --- | --- | --- | --- | --- | --- | --- |
| Model | 6 | 563,780 | 85,11% | 563,780 | 93,963 | 19,05 | 0,000 |
| Linear | 4 | 513,762 | 77,56% | 429,072 | 107,268 | 21,75 | 0,000 |
| Lac | 1 | 442,639 | 66,82% | 355,518 | 355,518 | 72,09 | 0,000 |
| HPMC\_Visc | 1 | 32,892 | 4,97% | 5,518 | 5,518 | 1,12 | 0,303 |
| HPMC\_HP | 1 | 28,297 | 4,27% | 20,537 | 20,537 | 4,16 | 0,055 |
| HPMC\_PS | 1 | 9,934 | 1,50% | 22,045 | 22,045 | 4,47 | 0,047 |
| 2-Way Interaction | 2 | 50,018 | 7,55% | 50,018 | 25,009 | 5,07 | 0,017 |
| Lac\*HPMC\_Visc | 1 | 28,693 | 4,33% | 28,693 | 28,693 | 5,82 | 0,026 |
| HPMC\_Visc\*HPMC\_PS | 1 | 21,325 | 3,22% | 21,325 | 21,325 | 4,32 | 0,051 |
| Error | 20 | 98,628 | 14,89% | 98,628 | 4,931 |  |  |
| Lack-of-Fit | 18 | 80,091 | 12,09% | 80,091 | 4,449 | 0,48 | 0,846 |
| Pure Error | 2 | 18,537 | 2,80% | 18,537 | 9,268 |  |  |
| Total | 26 | 662,407 | 100,00% |  |  |  |  |

## Regression Equation in Uncoded Units

|  |  |  |
| --- | --- | --- |
| F\_mean\_7h(420min) | = | 140,1 + 93,6 Lac - 0,01043 HPMC\_Visc + 1,830 HPMC\_HP - 2,06 HPMC\_PS - 0,00456 Lac\*HPMC\_Visc + 0,000179 HPMC\_Visc\*HPMC\_PS |

## Fits and Diagnostics for All Observations

| Obs | F\_mean\_7h(420min) | Fit | SE Fit | 95% CI | Resid | Std Resid | Del Resid | HI |
| --- | --- | --- | --- | --- | --- | --- | --- | --- |
| 1 | 52,67 | 51,58 | 1,18 | (49,12; 54,05) | 1,09 | 0,58 | 0,57 | 0,283627 |
| 2 | 62,10 | 62,73 | 1,18 | (60,26; 65,19) | -0,62 | -0,33 | -0,32 | 0,283627 |
| 3 | 47,72 | 49,45 | 1,55 | (46,22; 52,69) | -1,74 | -1,09 | -1,10 | 0,488210 |
| 4 | 54,58 | 54,38 | 1,55 | (51,14; 57,62) | 0,20 | 0,13 | 0,13 | 0,488210 |
| 5 | 50,14 | 53,25 | 1,03 | (51,09; 55,41) | -3,11 | -1,58 | -1,65 | 0,216779 |
| 6 | 62,57 | 64,12 | 1,03 | (61,96; 66,27) | -1,55 | -0,79 | -0,78 | 0,216779 |
| 7 | 51,53 | 52,88 | 1,40 | (49,97; 55,79) | -1,36 | -0,79 | -0,78 | 0,394639 |
| 8 | 56,49 | 57,72 | 1,40 | (54,81; 60,63) | -1,22 | -0,71 | -0,70 | 0,394639 |
| 9 | 51,99 | 51,07 | 1,20 | (48,55; 53,58) | 0,93 | 0,50 | 0,49 | 0,294244 |
| 10 | 62,01 | 62,12 | 1,20 | (59,61; 64,64) | -0,11 | -0,06 | -0,06 | 0,294244 |
| 11 | 54,52 | 53,68 | 1,04 | (51,52; 55,85) | 0,84 | 0,43 | 0,42 | 0,218366 |
| 12 | 60,68 | 60,82 | 1,04 | (58,66; 62,99) | -0,14 | -0,07 | -0,07 | 0,218366 |
| 13 | 52,28 | 52,96 | 1,31 | (50,23; 55,69) | -0,68 | -0,38 | -0,37 | 0,347162 |
| 14 | 64,73 | 64,06 | 1,31 | (61,33; 66,78) | 0,68 | 0,38 | 0,37 | 0,347162 |
| 15 | 54,73 | 55,33 | 1,13 | (52,98; 57,68) | -0,60 | -0,31 | -0,31 | 0,258117 |
| 16 | 58,35 | 62,06 | 1,13 | (59,71; 64,42) | -3,71 | -1,94 | -2,10 | 0,258117 |
| 17 | 48,46 | 48,76 | 1,03 | (46,62; 50,90) | -0,30 | -0,15 | -0,15 | 0,213198 |
| 18 | 67,03 | 66,40 | 1,03 | (64,26; 68,54) | 0,63 | 0,32 | 0,32 | 0,213198 |
| 19 | 59,91 | 58,16 | 0,88 | (56,32; 60,00) | 1,75 | 0,86 | 0,85 | 0,157071 |
| 20 | 59,53 | 55,18 | 1,03 | (53,03; 57,32) | 4,35 | 2,21 | 2,48 | 0,214445 |
| 21 | 55,96 | 55,69 | 0,92 | (53,78; 57,61) | 0,27 | 0,13 | 0,13 | 0,170686 |
| 22 | 63,83 | 59,64 | 1,11 | (57,32; 61,96) | 4,19 | 2,18 | 2,44 | 0,251394 |
| 23 | 57,98 | 56,35 | 1,31 | (53,63; 59,08) | 1,63 | 0,91 | 0,90 | 0,346058 |
| 24 | 58,69 | 58,77 | 1,21 | (56,24; 61,29) | -0,07 | -0,04 | -0,04 | 0,297564 |
| 25 | 54,89 | 57,58 | 0,47 | (56,60; 58,56) | -2,69 | -1,24 | -1,26 | 0,044701 |
| 26 | 55,90 | 57,58 | 0,47 | (56,60; 58,56) | -1,68 | -0,77 | -0,77 | 0,044701 |
| 27 | 60,60 | 57,58 | 0,47 | (56,60; 58,56) | 3,02 | 1,39 | 1,43 | 0,044701 |

| Obs | Cook’s D | DFITS |  |
| --- | --- | --- | --- |
| 1 | 0,02 | 0,35851 |  |
| 2 | 0,01 | -0,20432 |  |
| 3 | 0,16 | -1,07394 |  |
| 4 | 0,00 | 0,12239 |  |
| 5 | 0,10 | -0,86718 |  |
| 6 | 0,02 | -0,41102 |  |
| 7 | 0,06 | -0,62834 |  |
| 8 | 0,05 | -0,56509 |  |
| 9 | 0,01 | 0,31555 |  |
| 10 | 0,00 | -0,03789 |  |
| 11 | 0,01 | 0,22093 |  |
| 12 | 0,00 | -0,03793 |  |
| 13 | 0,01 | -0,26854 |  |
| 14 | 0,01 | 0,26843 |  |
| 15 | 0,00 | -0,18017 |  |
| 16 | 0,19 | -1,23776 |  |
| 17 | 0,00 | -0,07694 |  |
| 18 | 0,00 | 0,16398 |  |
| 19 | 0,02 | 0,36891 |  |
| 20 | 0,19 | 1,29421 | R |
| 21 | 0,00 | 0,05826 |  |
| 22 | 0,23 | 1,41176 | R |
| 23 | 0,06 | 0,65801 |  |
| 24 | 0,00 | -0,02455 |  |
| 25 | 0,01 | -0,27175 |  |
| 26 | 0,00 | -0,16549 |  |
| 27 | 0,01 | 0,30834 |  |

R  Large residual

## Forward Selection of Terms

Achieved minimum BIC =  138,04

## Coded Coefficients

| Term | Coef | SE Coef | 95% CI | T-Value | P-Value | VIF |
| --- | --- | --- | --- | --- | --- | --- |
| Constant | 61,934 | 0,510 | (60,870; 62,997) | 121,48 | 0,000 |  |
| Lac | 8,328 | 0,942 | (6,364; 10,293) | 8,84 | 0,000 | 1,08 |
| HPMC\_Visc | -1,110 | 0,918 | (-3,026; 0,805) | -1,21 | 0,241 | 1,35 |
| HPMC\_HP | 2,047 | 0,912 | (0,146; 3,949) | 2,25 | 0,036 | 1,03 |
| HPMC\_PS | 2,58 | 1,25 | (-0,04; 5,19) | 2,05 | 0,053 | 1,37 |
| Lac\*HPMC\_Visc | -4,45 | 1,84 | (-8,30; -0,61) | -2,42 | 0,025 | 1,08 |
| HPMC\_Visc\*HPMC\_PS | 5,34 | 2,47 | (0,19; 10,48) | 2,16 | 0,043 | 1,25 |

## Model Summary

| S | R-sq | R-sq(adj) | PRESS | R-sq(pred) | AICc | BIC |
| --- | --- | --- | --- | --- | --- | --- |
| 2,22382 | 86,05% | 81,87% | 170,263 | 75,99% | 135,68 | 138,04 |

## Analysis of Variance

| Source | DF | Seq SS | Contribution | Adj SS | Adj MS | F-Value | P-Value |
| --- | --- | --- | --- | --- | --- | --- | --- |
| Model | 6 | 610,126 | 86,05% | 610,126 | 101,688 | 20,56 | 0,000 |
| Linear | 4 | 558,040 | 78,70% | 466,922 | 116,731 | 23,60 | 0,000 |
| Lac | 1 | 478,903 | 67,54% | 386,681 | 386,681 | 78,19 | 0,000 |
| HPMC\_Visc | 1 | 37,307 | 5,26% | 7,234 | 7,234 | 1,46 | 0,241 |
| HPMC\_HP | 1 | 33,265 | 4,69% | 24,945 | 24,945 | 5,04 | 0,036 |
| HPMC\_PS | 1 | 8,565 | 1,21% | 20,827 | 20,827 | 4,21 | 0,053 |
| 2-Way Interaction | 2 | 52,086 | 7,35% | 52,086 | 26,043 | 5,27 | 0,015 |
| Lac\*HPMC\_Visc | 1 | 28,910 | 4,08% | 28,910 | 28,910 | 5,85 | 0,025 |
| HPMC\_Visc\*HPMC\_PS | 1 | 23,176 | 3,27% | 23,176 | 23,176 | 4,69 | 0,043 |
| Error | 20 | 98,907 | 13,95% | 98,907 | 4,945 |  |  |
| Lack-of-Fit | 18 | 81,689 | 11,52% | 81,689 | 4,538 | 0,53 | 0,821 |
| Pure Error | 2 | 17,218 | 2,43% | 17,218 | 8,609 |  |  |
| Total | 26 | 709,033 | 100,00% |  |  |  |  |

## Regression Equation in Uncoded Units

|  |  |  |
| --- | --- | --- |
| F\_mean\_8h(480min) | = | 150,3 + 95,2 Lac - 0,01099 HPMC\_Visc + 2,017 HPMC\_HP - 2,17 HPMC\_PS - 0,00458 Lac\*HPMC\_Visc + 0,000187 HPMC\_Visc\*HPMC\_PS |

## Fits and Diagnostics for All Observations

| Obs | F\_mean\_8h(480min) | Fit | SE Fit | 95% CI | Resid | Std Resid | Del Resid | HI |
| --- | --- | --- | --- | --- | --- | --- | --- | --- |
| 1 | 56,98 | 55,80 | 1,18 | (53,33; 58,27) | 1,17 | 0,62 | 0,61 | 0,283627 |
| 2 | 66,57 | 67,30 | 1,18 | (64,83; 69,77) | -0,73 | -0,39 | -0,38 | 0,283627 |
| 3 | 51,65 | 53,38 | 1,55 | (50,14; 56,62) | -1,74 | -1,09 | -1,10 | 0,488210 |
| 4 | 58,93 | 58,64 | 1,55 | (55,40; 61,88) | 0,29 | 0,18 | 0,18 | 0,488210 |
| 5 | 54,34 | 57,62 | 1,04 | (55,46; 59,78) | -3,27 | -1,66 | -1,75 | 0,216779 |
| 6 | 67,11 | 68,84 | 1,04 | (66,68; 71,00) | -1,72 | -0,88 | -0,87 | 0,216779 |
| 7 | 55,61 | 57,05 | 1,40 | (54,14; 59,97) | -1,45 | -0,84 | -0,83 | 0,394639 |
| 8 | 61,03 | 62,22 | 1,40 | (59,30; 65,13) | -1,18 | -0,68 | -0,68 | 0,394639 |
| 9 | 55,84 | 55,14 | 1,21 | (52,62; 57,66) | 0,70 | 0,38 | 0,37 | 0,294244 |
| 10 | 66,54 | 66,55 | 1,21 | (64,03; 69,07) | -0,01 | -0,00 | -0,00 | 0,294244 |
| 11 | 58,96 | 57,77 | 1,04 | (55,60; 59,93) | 1,19 | 0,61 | 0,60 | 0,218366 |
| 12 | 64,88 | 65,24 | 1,04 | (63,08; 67,41) | -0,36 | -0,18 | -0,18 | 0,218366 |
| 13 | 56,55 | 57,22 | 1,31 | (54,48; 59,95) | -0,66 | -0,37 | -0,36 | 0,347162 |
| 14 | 69,33 | 68,67 | 1,31 | (65,93; 71,40) | 0,66 | 0,37 | 0,36 | 0,347162 |
| 15 | 58,88 | 59,53 | 1,13 | (57,17; 61,88) | -0,65 | -0,34 | -0,33 | 0,258117 |
| 16 | 62,99 | 66,60 | 1,13 | (64,24; 68,95) | -3,61 | -1,88 | -2,02 | 0,258117 |
| 17 | 52,52 | 52,81 | 1,03 | (50,67; 54,95) | -0,29 | -0,15 | -0,14 | 0,213198 |
| 18 | 71,83 | 71,14 | 1,03 | (69,00; 73,28) | 0,69 | 0,35 | 0,34 | 0,213198 |
| 19 | 64,58 | 62,64 | 0,88 | (60,80; 64,47) | 1,94 | 0,95 | 0,95 | 0,157071 |
| 20 | 63,57 | 59,36 | 1,03 | (57,21; 61,51) | 4,21 | 2,14 | 2,37 | 0,214445 |
| 21 | 60,02 | 59,92 | 0,92 | (58,00; 61,83) | 0,10 | 0,05 | 0,05 | 0,170686 |
| 22 | 68,38 | 64,22 | 1,12 | (61,89; 66,54) | 4,16 | 2,16 | 2,41 | 0,251394 |
| 23 | 62,68 | 60,88 | 1,31 | (58,15; 63,61) | 1,80 | 1,00 | 1,00 | 0,346058 |
| 24 | 63,03 | 63,01 | 1,21 | (60,48; 65,55) | 0,02 | 0,01 | 0,01 | 0,297564 |
| 25 | 59,29 | 61,98 | 0,47 | (61,00; 62,96) | -2,69 | -1,24 | -1,26 | 0,044701 |
| 26 | 60,51 | 61,98 | 0,47 | (61,00; 62,96) | -1,46 | -0,67 | -0,66 | 0,044701 |
| 27 | 64,87 | 61,98 | 0,47 | (61,00; 62,96) | 2,89 | 1,33 | 1,36 | 0,044701 |

| Obs | Cook’s D | DFITS |  |
| --- | --- | --- | --- |
| 1 | 0,02 | 0,38616 |  |
| 2 | 0,01 | -0,24023 |  |
| 3 | 0,16 | -1,07143 |  |
| 4 | 0,00 | 0,17361 |  |
| 5 | 0,11 | -0,91870 |  |
| 6 | 0,03 | -0,45833 |  |
| 7 | 0,07 | -0,67072 |  |
| 8 | 0,04 | -0,54505 |  |
| 9 | 0,01 | 0,23777 |  |
| 10 | 0,00 | -0,00314 |  |
| 11 | 0,01 | 0,31534 |  |
| 12 | 0,00 | -0,09475 |  |
| 13 | 0,01 | -0,26364 |  |
| 14 | 0,01 | 0,26384 |  |
| 15 | 0,01 | -0,19525 |  |
| 16 | 0,18 | -1,19336 |  |
| 17 | 0,00 | -0,07448 |  |
| 18 | 0,00 | 0,17873 |  |
| 19 | 0,02 | 0,40906 |  |
| 20 | 0,18 | 1,23736 | R |
| 21 | 0,00 | 0,02154 |  |
| 22 | 0,22 | 1,39601 | R |
| 23 | 0,08 | 0,72682 |  |
| 24 | 0,00 | 0,00658 |  |
| 25 | 0,01 | -0,27161 |  |
| 26 | 0,00 | -0,14351 |  |
| 27 | 0,01 | 0,29394 |  |

R  Large residual

## Forward Selection of Terms

Achieved minimum BIC =  139,37

## Coded Coefficients

| Term | Coef | SE Coef | 95% CI | T-Value | P-Value | VIF |
| --- | --- | --- | --- | --- | --- | --- |
| Constant | 66,089 | 0,522 | (64,999; 67,179) | 126,49 | 0,000 |  |
| Lac | 8,670 | 0,965 | (6,657; 10,684) | 8,98 | 0,000 | 1,08 |
| HPMC\_Visc | -1,268 | 0,941 | (-3,230; 0,695) | -1,35 | 0,193 | 1,35 |
| HPMC\_HP | 2,171 | 0,934 | (0,223; 4,120) | 2,32 | 0,031 | 1,03 |
| HPMC\_PS | 2,38 | 1,29 | (-0,30; 5,06) | 1,85 | 0,079 | 1,37 |
| Lac\*HPMC\_Visc | -4,33 | 1,89 | (-8,27; -0,39) | -2,29 | 0,033 | 1,08 |
| HPMC\_Visc\*HPMC\_PS | 5,60 | 2,53 | (0,33; 10,87) | 2,22 | 0,038 | 1,25 |

## Model Summary

| S | R-sq | R-sq(adj) | PRESS | R-sq(pred) | AICc | BIC |
| --- | --- | --- | --- | --- | --- | --- |
| 2,27915 | 86,24% | 82,12% | 178,487 | 76,37% | 137,01 | 139,37 |

## Analysis of Variance

| Source | DF | Seq SS | Contribution | Adj SS | Adj MS | F-Value | P-Value |
| --- | --- | --- | --- | --- | --- | --- | --- |
| Model | 6 | 651,292 | 86,24% | 651,292 | 108,549 | 20,90 | 0,000 |
| Linear | 4 | 598,428 | 79,24% | 501,846 | 125,461 | 24,15 | 0,000 |
| Lac | 1 | 514,461 | 68,12% | 419,127 | 419,127 | 80,69 | 0,000 |
| HPMC\_Visc | 1 | 41,697 | 5,52% | 9,428 | 9,428 | 1,81 | 0,193 |
| HPMC\_HP | 1 | 36,251 | 4,80% | 28,060 | 28,060 | 5,40 | 0,031 |
| HPMC\_PS | 1 | 6,019 | 0,80% | 17,803 | 17,803 | 3,43 | 0,079 |
| 2-Way Interaction | 2 | 52,864 | 7,00% | 52,864 | 26,432 | 5,09 | 0,016 |
| Lac\*HPMC\_Visc | 1 | 27,345 | 3,62% | 27,345 | 27,345 | 5,26 | 0,033 |
| HPMC\_Visc\*HPMC\_PS | 1 | 25,519 | 3,38% | 25,519 | 25,519 | 4,91 | 0,038 |
| Error | 20 | 103,891 | 13,76% | 103,891 | 5,195 |  |  |
| Lack-of-Fit | 18 | 86,933 | 11,51% | 86,933 | 4,830 | 0,57 | 0,799 |
| Pure Error | 2 | 16,958 | 2,25% | 16,958 | 8,479 |  |  |
| Total | 26 | 755,182 | 100,00% |  |  |  |  |

## Regression Equation in Uncoded Units

|  |  |  |
| --- | --- | --- |
| F\_mean\_9h(540min) | = | 164,5 + 94,9 Lac - 0,01174 HPMC\_Visc + 2,139 HPMC\_HP - 2,32 HPMC\_PS - 0,00445 Lac\*HPMC\_Visc + 0,000196 HPMC\_Visc\*HPMC\_PS |

## Fits and Diagnostics for All Observations

| Obs | F\_mean\_9h(540min) | Fit | SE Fit | 95% CI | Resid | Std Resid | Del Resid | HI |
| --- | --- | --- | --- | --- | --- | --- | --- | --- |
| 1 | 61,82 | 59,95 | 1,21 | (57,41; 62,48) | 1,87 | 0,97 | 0,97 | 0,283627 |
| 2 | 70,69 | 71,70 | 1,21 | (69,17; 74,23) | -1,01 | -0,52 | -0,51 | 0,283627 |
| 3 | 55,31 | 57,13 | 1,59 | (53,81; 60,45) | -1,81 | -1,11 | -1,12 | 0,488210 |
| 4 | 63,03 | 62,81 | 1,59 | (59,49; 66,13) | 0,22 | 0,14 | 0,13 | 0,488210 |
| 5 | 58,31 | 61,83 | 1,06 | (59,62; 64,05) | -3,52 | -1,74 | -1,85 | 0,216779 |
| 6 | 71,25 | 73,32 | 1,06 | (71,10; 75,53) | -2,06 | -1,02 | -1,02 | 0,216779 |
| 7 | 59,80 | 60,95 | 1,43 | (57,96; 63,93) | -1,14 | -0,64 | -0,63 | 0,394639 |
| 8 | 65,38 | 66,54 | 1,43 | (63,55; 69,52) | -1,16 | -0,65 | -0,64 | 0,394639 |
| 9 | 59,43 | 59,03 | 1,24 | (56,46; 61,61) | 0,40 | 0,21 | 0,20 | 0,294244 |
| 10 | 70,74 | 70,70 | 1,24 | (68,12; 73,28) | 0,03 | 0,02 | 0,02 | 0,294244 |
| 11 | 63,16 | 61,62 | 1,07 | (59,40; 63,84) | 1,54 | 0,76 | 0,76 | 0,218366 |
| 12 | 68,85 | 69,46 | 1,07 | (67,24; 71,68) | -0,61 | -0,30 | -0,30 | 0,218366 |
| 13 | 60,61 | 61,21 | 1,34 | (58,41; 64,01) | -0,59 | -0,32 | -0,32 | 0,347162 |
| 14 | 73,59 | 72,91 | 1,34 | (70,11; 75,72) | 0,68 | 0,37 | 0,36 | 0,347162 |
| 15 | 62,64 | 63,44 | 1,16 | (61,02; 65,85) | -0,80 | -0,41 | -0,40 | 0,258117 |
| 16 | 67,27 | 70,89 | 1,16 | (68,47; 73,30) | -3,62 | -1,84 | -1,97 | 0,258117 |
| 17 | 56,36 | 56,68 | 1,05 | (54,48; 58,87) | -0,32 | -0,16 | -0,15 | 0,213198 |
| 18 | 77,06 | 75,65 | 1,05 | (73,45; 77,84) | 1,42 | 0,70 | 0,69 | 0,213198 |
| 19 | 68,90 | 66,90 | 0,90 | (65,01; 68,78) | 2,00 | 0,96 | 0,95 | 0,157071 |
| 20 | 67,37 | 63,33 | 1,06 | (61,13; 65,53) | 4,04 | 2,00 | 2,18 | 0,214445 |
| 21 | 63,91 | 64,00 | 0,94 | (62,04; 65,97) | -0,09 | -0,05 | -0,04 | 0,170686 |
| 22 | 72,51 | 68,50 | 1,14 | (66,12; 70,89) | 4,01 | 2,03 | 2,23 | 0,251394 |
| 23 | 67,11 | 65,33 | 1,34 | (62,53; 68,13) | 1,78 | 0,96 | 0,96 | 0,346058 |
| 24 | 67,04 | 66,93 | 1,24 | (64,33; 69,52) | 0,12 | 0,06 | 0,06 | 0,297564 |
| 25 | 63,34 | 66,16 | 0,48 | (65,16; 67,17) | -2,82 | -1,27 | -1,29 | 0,044701 |
| 26 | 64,82 | 66,16 | 0,48 | (65,16; 67,17) | -1,34 | -0,60 | -0,59 | 0,044701 |
| 27 | 68,96 | 66,16 | 0,48 | (65,16; 67,17) | 2,80 | 1,26 | 1,28 | 0,044701 |

| Obs | Cook’s D | DFITS |  |
| --- | --- | --- | --- |
| 1 | 0,05 | 0,60923 |  |
| 2 | 0,02 | -0,32272 |  |
| 3 | 0,17 | -1,09382 |  |
| 4 | 0,00 | 0,12877 |  |
| 5 | 0,12 | -0,97125 |  |
| 6 | 0,04 | -0,53861 |  |
| 7 | 0,04 | -0,51225 |  |
| 8 | 0,04 | -0,51953 |  |
| 9 | 0,00 | 0,13080 |  |
| 10 | 0,00 | 0,01107 |  |
| 11 | 0,02 | 0,39955 |  |
| 12 | 0,00 | -0,15631 |  |
| 13 | 0,01 | -0,22976 |  |
| 14 | 0,01 | 0,26279 |  |
| 15 | 0,01 | -0,23546 |  |
| 16 | 0,17 | -1,16388 |  |
| 17 | 0,00 | -0,07920 |  |
| 18 | 0,02 | 0,36015 |  |
| 19 | 0,02 | 0,41159 |  |
| 20 | 0,16 | 1,13943 | R |
| 21 | 0,00 | -0,02000 |  |
| 22 | 0,20 | 1,29013 | R |
| 23 | 0,07 | 0,69978 |  |
| 24 | 0,00 | 0,03852 |  |
| 25 | 0,01 | -0,27819 |  |
| 26 | 0,00 | -0,12838 |  |
| 27 | 0,01 | 0,27586 |  |

R  Large residual

## Forward Selection of Terms

Achieved minimum BIC =  142,98

## Coded Coefficients

| Term | Coef | SE Coef | 95% CI | T-Value | P-Value | VIF |
| --- | --- | --- | --- | --- | --- | --- |
| Constant | 70,074 | 0,559 | (68,908; 71,239) | 125,44 | 0,000 |  |
| Lac | 9,37 | 1,03 | (7,22; 11,53) | 9,08 | 0,000 | 1,08 |
| HPMC\_Visc | -1,32 | 1,01 | (-3,42; 0,77) | -1,32 | 0,203 | 1,35 |
| HPMC\_HP | 2,296 | 0,999 | (0,213; 4,380) | 2,30 | 0,032 | 1,03 |
| HPMC\_PS | 2,30 | 1,38 | (-0,57; 5,17) | 1,67 | 0,110 | 1,37 |
| Lac\*HPMC\_Visc | -4,42 | 2,02 | (-8,63; -0,21) | -2,19 | 0,041 | 1,08 |
| HPMC\_Visc\*HPMC\_PS | 5,98 | 2,70 | (0,35; 11,62) | 2,21 | 0,039 | 1,25 |

## Model Summary

| S | R-sq | R-sq(adj) | PRESS | R-sq(pred) | AICc | BIC |
| --- | --- | --- | --- | --- | --- | --- |
| 2,43681 | 86,22% | 82,09% | 201,526 | 76,62% | 140,62 | 142,98 |

## Analysis of Variance

| Source | DF | Seq SS | Contribution | Adj SS | Adj MS | F-Value | P-Value |
| --- | --- | --- | --- | --- | --- | --- | --- |
| Model | 6 | 743,069 | 86,22% | 743,07 | 123,845 | 20,86 | 0,000 |
| Linear | 4 | 685,518 | 79,54% | 575,76 | 143,939 | 24,24 | 0,000 |
| Lac | 1 | 597,032 | 69,27% | 489,94 | 489,938 | 82,51 | 0,000 |
| HPMC\_Visc | 1 | 44,050 | 5,11% | 10,28 | 10,285 | 1,73 | 0,203 |
| HPMC\_HP | 1 | 39,790 | 4,62% | 31,38 | 31,384 | 5,29 | 0,032 |
| HPMC\_PS | 1 | 4,646 | 0,54% | 16,63 | 16,632 | 2,80 | 0,110 |
| 2-Way Interaction | 2 | 57,551 | 6,68% | 57,55 | 28,776 | 4,85 | 0,019 |
| Lac\*HPMC\_Visc | 1 | 28,431 | 3,30% | 28,43 | 28,431 | 4,79 | 0,041 |
| HPMC\_Visc\*HPMC\_PS | 1 | 29,120 | 3,38% | 29,12 | 29,120 | 4,90 | 0,039 |
| Error | 20 | 118,761 | 13,78% | 118,76 | 5,938 |  |  |
| Lack-of-Fit | 18 | 102,459 | 11,89% | 102,46 | 5,692 | 0,70 | 0,735 |
| Pure Error | 2 | 16,301 | 1,89% | 16,30 | 8,151 |  |  |
| Total | 26 | 861,830 | 100,00% |  |  |  |  |

## Regression Equation in Uncoded Units

|  |  |  |
| --- | --- | --- |
| F\_mean\_10h(600min) | = | 178,8 + 98,9 Lac - 0,01264 HPMC\_Visc + 2,263 HPMC\_HP - 2,51 HPMC\_PS - 0,00454 Lac\*HPMC\_Visc + 0,000209 HPMC\_Visc\*HPMC\_PS |

## Fits and Diagnostics for All Observations

| Obs | F\_mean\_10h(600min) | Fit | SE Fit | 95% CI | Resid | Std Resid | Del Resid | HI |
| --- | --- | --- | --- | --- | --- | --- | --- | --- |
| 1 | 65,67 | 63,59 | 1,30 | (60,88; 66,29) | 2,08 | 1,01 | 1,01 | 0,283627 |
| 2 | 74,63 | 76,10 | 1,30 | (73,40; 78,81) | -1,47 | -0,71 | -0,71 | 0,283627 |
| 3 | 58,83 | 60,55 | 1,70 | (57,00; 64,10) | -1,73 | -0,99 | -0,99 | 0,488210 |
| 4 | 66,93 | 66,88 | 1,70 | (63,33; 70,43) | 0,04 | 0,03 | 0,02 | 0,488210 |
| 5 | 62,05 | 65,56 | 1,13 | (63,19; 67,92) | -3,50 | -1,62 | -1,70 | 0,216779 |
| 6 | 75,06 | 77,80 | 1,13 | (75,43; 80,16) | -2,74 | -1,27 | -1,29 | 0,216779 |
| 7 | 63,63 | 64,55 | 1,53 | (61,36; 67,75) | -0,93 | -0,49 | -0,48 | 0,394639 |
| 8 | 69,51 | 70,79 | 1,53 | (67,60; 73,98) | -1,28 | -0,68 | -0,67 | 0,394639 |
| 9 | 62,76 | 62,45 | 1,32 | (59,69; 65,21) | 0,31 | 0,15 | 0,15 | 0,294244 |
| 10 | 74,79 | 74,88 | 1,32 | (72,12; 77,64) | -0,09 | -0,04 | -0,04 | 0,294244 |
| 11 | 67,12 | 65,19 | 1,14 | (62,81; 67,56) | 1,94 | 0,90 | 0,89 | 0,218366 |
| 12 | 72,64 | 73,72 | 1,14 | (71,34; 76,09) | -1,08 | -0,50 | -0,49 | 0,218366 |
| 13 | 64,44 | 64,72 | 1,44 | (61,72; 67,71) | -0,28 | -0,14 | -0,14 | 0,347162 |
| 14 | 77,49 | 77,19 | 1,44 | (74,20; 80,19) | 0,30 | 0,15 | 0,15 | 0,347162 |
| 15 | 66,38 | 67,10 | 1,24 | (64,51; 69,68) | -0,71 | -0,34 | -0,33 | 0,258117 |
| 16 | 71,30 | 75,22 | 1,24 | (72,64; 77,81) | -3,92 | -1,87 | -2,01 | 0,258117 |
| 17 | 60,11 | 59,95 | 1,13 | (57,61; 62,30) | 0,16 | 0,07 | 0,07 | 0,213198 |
| 18 | 84,23 | 80,36 | 1,13 | (78,01; 82,71) | 3,87 | 1,79 | 1,90 | 0,213198 |
| 19 | 72,93 | 70,89 | 0,97 | (68,87; 72,90) | 2,04 | 0,91 | 0,91 | 0,157071 |
| 20 | 70,91 | 67,19 | 1,13 | (64,84; 69,55) | 3,71 | 1,72 | 1,81 | 0,214445 |
| 21 | 67,57 | 67,89 | 1,01 | (65,79; 69,99) | -0,32 | -0,15 | -0,14 | 0,170686 |
| 22 | 76,19 | 72,61 | 1,22 | (70,06; 75,16) | 3,58 | 1,70 | 1,79 | 0,251394 |
| 23 | 71,17 | 69,50 | 1,43 | (66,51; 72,49) | 1,67 | 0,85 | 0,84 | 0,346058 |
| 24 | 70,77 | 70,72 | 1,33 | (67,95; 73,49) | 0,05 | 0,02 | 0,02 | 0,297564 |
| 25 | 67,14 | 70,16 | 0,52 | (69,08; 71,23) | -3,01 | -1,27 | -1,29 | 0,044701 |
| 26 | 68,89 | 70,16 | 0,52 | (69,08; 71,23) | -1,26 | -0,53 | -0,52 | 0,044701 |
| 27 | 72,73 | 70,16 | 0,52 | (69,08; 71,23) | 2,57 | 1,08 | 1,08 | 0,044701 |

| Obs | Cook’s D | DFITS |
| --- | --- | --- |
| 1 | 0,06 | 0,63480 |
| 2 | 0,03 | -0,44371 |
| 3 | 0,13 | -0,96702 |
| 4 | 0,00 | 0,02441 |
| 5 | 0,10 | -0,89421 |
| 6 | 0,06 | -0,67843 |
| 7 | 0,02 | -0,38710 |
| 8 | 0,04 | -0,53792 |
| 9 | 0,00 | 0,09688 |
| 10 | 0,00 | -0,02643 |
| 11 | 0,03 | 0,47275 |
| 12 | 0,01 | -0,26025 |
| 13 | 0,00 | -0,09943 |
| 14 | 0,00 | 0,10906 |
| 15 | 0,01 | -0,19601 |
| 16 | 0,17 | -1,18274 |
| 17 | 0,00 | 0,03711 |
| 18 | 0,12 | 0,99052 |
| 19 | 0,02 | 0,39264 |
| 20 | 0,12 | 0,94829 |
| 21 | 0,00 | -0,06425 |
| 22 | 0,14 | 1,03614 |
| 23 | 0,05 | 0,61299 |
| 24 | 0,00 | 0,01457 |
| 25 | 0,01 | -0,27816 |
| 26 | 0,00 | -0,11263 |
| 27 | 0,01 | 0,23426 |

## Forward Selection of Terms

Achieved minimum BIC =  142,84

## Coded Coefficients

| Term | Coef | SE Coef | 95% CI | T-Value | P-Value | VIF |
| --- | --- | --- | --- | --- | --- | --- |
| Constant | 73,136 | 0,535 | (72,027; 74,245) | 136,74 | 0,000 |  |
| Lac | 9,65 | 1,11 | (7,35; 11,95) | 8,70 | 0,000 | 1,08 |
| HPMC\_Visc | -2,517 | 0,933 | (-4,452; -0,583) | -2,70 | 0,013 | 1,00 |
| HPMC\_HP | 2,72 | 1,06 | (0,52; 4,91) | 2,56 | 0,018 | 1,00 |
| Lac\*HPMC\_Visc | -4,72 | 2,17 | (-9,22; -0,23) | -2,18 | 0,040 | 1,08 |

## Model Summary

| S | R-sq | R-sq(adj) | PRESS | R-sq(pred) | AICc | BIC |
| --- | --- | --- | --- | --- | --- | --- |
| 2,61784 | 83,42% | 80,40% | 247,731 | 72,75% | 139,26 | 142,84 |

## Analysis of Variance

| Source | DF | Seq SS | Contribution | Adj SS | Adj MS | F-Value | P-Value |
| --- | --- | --- | --- | --- | --- | --- | --- |
| Model | 4 | 758,46 | 83,42% | 758,46 | 189,614 | 27,67 | 0,000 |
| Linear | 3 | 725,95 | 79,84% | 609,58 | 203,192 | 29,65 | 0,000 |
| Lac | 1 | 635,13 | 69,85% | 518,76 | 518,756 | 75,70 | 0,000 |
| HPMC\_Visc | 1 | 45,89 | 5,05% | 49,92 | 49,915 | 7,28 | 0,013 |
| HPMC\_HP | 1 | 44,93 | 4,94% | 44,93 | 44,933 | 6,56 | 0,018 |
| 2-Way Interaction | 1 | 32,51 | 3,58% | 32,51 | 32,505 | 4,74 | 0,040 |
| Lac\*HPMC\_Visc | 1 | 32,51 | 3,58% | 32,51 | 32,505 | 4,74 | 0,040 |
| Error | 22 | 150,77 | 16,58% | 150,77 | 6,853 |  |  |
| Lack-of-Fit | 20 | 135,54 | 14,91% | 135,54 | 6,777 | 0,89 | 0,655 |
| Pure Error | 2 | 15,23 | 1,68% | 15,23 | 7,617 |  |  |
| Total | 26 | 909,23 | 100,00% |  |  |  |  |

## Regression Equation in Uncoded Units

|  |  |  |
| --- | --- | --- |
| F\_mean\_11h(660min) | = | 4,4 + 104,2 Lac + 0,00178 HPMC\_Visc + 2,68 HPMC\_HP - 0,00486 Lac\*HPMC\_Visc |

## Fits and Diagnostics for All Observations

| Obs | F\_mean\_11h(660min) | Fit | SE Fit | 95% CI | Resid | Std Resid | Del Resid | HI |
| --- | --- | --- | --- | --- | --- | --- | --- | --- |
| 1 | 68,86 | 66,47 | 1,26 | (63,85; 69,08) | 2,39 | 1,04 | 1,04 | 0,231482 |
| 2 | 78,48 | 79,47 | 1,26 | (76,86; 82,09) | -0,99 | -0,43 | -0,42 | 0,231482 |
| 3 | 62,20 | 66,16 | 1,55 | (62,95; 69,38) | -3,96 | -1,88 | -2,00 | 0,351061 |
| 4 | 70,58 | 72,55 | 1,55 | (69,34; 75,77) | -1,97 | -0,94 | -0,93 | 0,351061 |
| 5 | 65,58 | 68,97 | 1,11 | (66,68; 71,27) | -3,39 | -1,43 | -1,46 | 0,178686 |
| 6 | 78,49 | 81,68 | 1,11 | (79,39; 83,98) | -3,19 | -1,35 | -1,37 | 0,178686 |
| 7 | 67,32 | 69,49 | 1,54 | (66,30; 72,68) | -2,17 | -1,02 | -1,02 | 0,344801 |
| 8 | 73,44 | 75,78 | 1,54 | (72,59; 78,97) | -2,34 | -1,10 | -1,11 | 0,344801 |
| 9 | 65,94 | 66,57 | 1,22 | (64,04; 69,11) | -0,64 | -0,27 | -0,27 | 0,217619 |
| 10 | 78,64 | 79,49 | 1,22 | (76,96; 82,02) | -0,85 | -0,37 | -0,36 | 0,217619 |
| 11 | 70,91 | 66,99 | 0,95 | (65,02; 68,96) | 3,91 | 1,60 | 1,67 | 0,131235 |
| 12 | 76,19 | 75,74 | 0,95 | (73,77; 77,70) | 0,45 | 0,19 | 0,18 | 0,131235 |
| 13 | 68,14 | 69,53 | 1,25 | (66,95; 72,11) | -1,39 | -0,60 | -0,59 | 0,226191 |
| 14 | 82,05 | 82,49 | 1,25 | (79,91; 85,07) | -0,44 | -0,19 | -0,19 | 0,226191 |
| 15 | 70,11 | 68,74 | 0,99 | (66,68; 70,80) | 1,38 | 0,57 | 0,56 | 0,143742 |
| 16 | 75,05 | 77,05 | 0,99 | (74,99; 79,11) | -2,00 | -0,83 | -0,82 | 0,143742 |
| 17 | 63,67 | 62,93 | 1,19 | (60,46; 65,40) | 0,74 | 0,32 | 0,31 | 0,206936 |
| 18 | 88,47 | 83,99 | 1,19 | (81,52; 86,46) | 4,47 | 1,92 | 2,05 | 0,206936 |
| 19 | 76,75 | 75,45 | 0,96 | (73,46; 77,44) | 1,30 | 0,53 | 0,52 | 0,134128 |
| 20 | 74,25 | 70,15 | 1,17 | (67,72; 72,58) | 4,10 | 1,75 | 1,85 | 0,200265 |
| 21 | 70,99 | 70,87 | 1,05 | (68,70; 73,05) | 0,11 | 0,05 | 0,05 | 0,161088 |
| 22 | 79,48 | 76,14 | 1,30 | (73,45; 78,84) | 3,34 | 1,47 | 1,51 | 0,246222 |
| 23 | 74,80 | 73,59 | 0,52 | (72,51; 74,67) | 1,22 | 0,47 | 0,47 | 0,039752 |
| 24 | 74,21 | 73,35 | 0,52 | (72,27; 74,43) | 0,87 | 0,34 | 0,33 | 0,039724 |
| 25 | 70,68 | 73,46 | 0,51 | (72,40; 74,53) | -2,78 | -1,08 | -1,09 | 0,038439 |
| 26 | 72,61 | 73,46 | 0,51 | (72,40; 74,53) | -0,85 | -0,33 | -0,32 | 0,038439 |
| 27 | 76,13 | 73,46 | 0,51 | (72,40; 74,53) | 2,67 | 1,04 | 1,04 | 0,038439 |

| Obs | Cook’s D | DFITS |
| --- | --- | --- |
| 1 | 0,07 | 0,57297 |
| 2 | 0,01 | -0,23309 |
| 3 | 0,38 | -1,47360 |
| 4 | 0,09 | -0,68582 |
| 5 | 0,09 | -0,68324 |
| 6 | 0,08 | -0,63999 |
| 7 | 0,11 | -0,74308 |
| 8 | 0,13 | -0,80446 |
| 9 | 0,00 | -0,14191 |
| 10 | 0,01 | -0,18917 |
| 11 | 0,08 | 0,64829 |
| 12 | 0,00 | 0,07071 |
| 13 | 0,02 | -0,32063 |
| 14 | 0,00 | -0,10024 |
| 15 | 0,01 | 0,22920 |
| 16 | 0,02 | -0,33564 |
| 17 | 0,01 | 0,15850 |
| 18 | 0,19 | 1,04892 |
| 19 | 0,01 | 0,20577 |
| 20 | 0,15 | 0,92342 |
| 21 | 0,00 | 0,02013 |
| 22 | 0,14 | 0,86433 |
| 23 | 0,00 | 0,09470 |
| 24 | 0,00 | 0,06732 |
| 25 | 0,01 | -0,21722 |
| 26 | 0,00 | -0,06466 |
| 27 | 0,01 | 0,20802 |

## Forward Selection of Terms

Achieved minimum BIC =  140,67

## Coded Coefficients

| Term | Coef | SE Coef | 95% CI | T-Value | P-Value | VIF |
| --- | --- | --- | --- | --- | --- | --- |
| Constant | 76,469 | 0,514 | (75,403; 77,535) | 148,83 | 0,000 |  |
| Lac | 9,68 | 1,07 | (7,47; 11,89) | 9,09 | 0,000 | 1,08 |
| HPMC\_Visc | -2,584 | 0,896 | (-4,442; -0,725) | -2,88 | 0,009 | 1,00 |
| HPMC\_HP | 2,76 | 1,02 | (0,65; 4,87) | 2,71 | 0,013 | 1,00 |
| Lac\*HPMC\_Visc | -4,89 | 2,08 | (-9,22; -0,57) | -2,35 | 0,028 | 1,08 |

## Model Summary

| S | R-sq | R-sq(adj) | PRESS | R-sq(pred) | AICc | BIC |
| --- | --- | --- | --- | --- | --- | --- |
| 2,51476 | 84,72% | 81,95% | 227,738 | 75,00% | 137,09 | 140,67 |

## Analysis of Variance

| Source | DF | Seq SS | Contribution | Adj SS | Adj MS | F-Value | P-Value |
| --- | --- | --- | --- | --- | --- | --- | --- |
| Model | 4 | 771,64 | 84,72% | 771,64 | 192,911 | 30,50 | 0,000 |
| Linear | 3 | 736,74 | 80,89% | 616,96 | 205,653 | 32,52 | 0,000 |
| Lac | 1 | 642,01 | 70,49% | 522,23 | 522,231 | 82,58 | 0,000 |
| HPMC\_Visc | 1 | 48,37 | 5,31% | 52,57 | 52,572 | 8,31 | 0,009 |
| HPMC\_HP | 1 | 46,36 | 5,09% | 46,36 | 46,356 | 7,33 | 0,013 |
| 2-Way Interaction | 1 | 34,91 | 3,83% | 34,91 | 34,905 | 5,52 | 0,028 |
| Lac\*HPMC\_Visc | 1 | 34,91 | 3,83% | 34,91 | 34,905 | 5,52 | 0,028 |
| Error | 22 | 139,13 | 15,28% | 139,13 | 6,324 |  |  |
| Lack-of-Fit | 20 | 124,71 | 13,69% | 124,71 | 6,236 | 0,87 | 0,665 |
| Pure Error | 2 | 14,42 | 1,58% | 14,42 | 7,208 |  |  |
| Total | 26 | 910,77 | 100,00% |  |  |  |  |

## Regression Equation in Uncoded Units

|  |  |  |
| --- | --- | --- |
| F\_mean\_12h(720min) | = | 6,3 + 106,7 Lac + 0,00185 HPMC\_Visc + 2,72 HPMC\_HP - 0,00503 Lac\*HPMC\_Visc |

## Fits and Diagnostics for All Observations

| Obs | F\_mean\_12h(720min) | Fit | SE Fit | 95% CI | Resid | Std Resid | Del Resid |
| --- | --- | --- | --- | --- | --- | --- | --- |
| 1 | 72,058 | 69,739 | 1,210 | (67,229; 72,248) | 2,320 | 1,05 | 1,05 |
| 2 | 81,925 | 82,902 | 1,210 | (80,392; 85,411) | -0,976 | -0,44 | -0,43 |
| 3 | 65,371 | 69,463 | 1,490 | (66,373; 72,553) | -4,091 | -2,02 | -2,19 |
| 4 | 74,075 | 75,766 | 1,490 | (72,676; 78,857) | -1,691 | -0,83 | -0,83 |
| 5 | 68,884 | 72,284 | 1,063 | (70,080; 74,489) | -3,400 | -1,49 | -1,54 |
| 6 | 81,718 | 85,141 | 1,063 | (82,936; 87,345) | -3,423 | -1,50 | -1,55 |
| 7 | 70,725 | 72,842 | 1,477 | (69,779; 75,904) | -2,116 | -1,04 | -1,04 |
| 8 | 76,996 | 79,043 | 1,477 | (75,981; 82,106) | -2,047 | -1,01 | -1,01 |
| 9 | 68,904 | 69,848 | 1,173 | (67,415; 72,281) | -0,944 | -0,42 | -0,42 |
| 10 | 82,575 | 82,915 | 1,173 | (80,482; 85,348) | -0,340 | -0,15 | -0,15 |
| 11 | 74,429 | 70,291 | 0,911 | (68,402; 72,181) | 4,138 | 1,77 | 1,86 |
| 12 | 79,559 | 79,036 | 0,911 | (77,147; 80,926) | 0,523 | 0,22 | 0,22 |
| 13 | 71,590 | 72,851 | 1,196 | (70,371; 75,332) | -1,261 | -0,57 | -0,56 |
| 14 | 86,147 | 85,961 | 1,196 | (83,481; 88,441) | 0,186 | 0,08 | 0,08 |
| 15 | 73,461 | 72,067 | 0,953 | (70,090; 74,044) | 1,395 | 0,60 | 0,59 |
| 16 | 78,588 | 80,364 | 0,953 | (78,386; 82,341) | -1,776 | -0,76 | -0,76 |
| 17 | 67,033 | 66,208 | 1,144 | (63,835; 68,580) | 0,825 | 0,37 | 0,36 |
| 18 | 91,018 | 87,401 | 1,144 | (85,029; 89,774) | 3,617 | 1,61 | 1,68 |
| 19 | 80,233 | 78,849 | 0,921 | (76,939; 80,759) | 1,384 | 0,59 | 0,58 |
| 20 | 77,312 | 73,410 | 1,125 | (71,076; 75,744) | 3,902 | 1,74 | 1,82 |
| 21 | 74,103 | 74,177 | 1,009 | (72,084; 76,271) | -0,074 | -0,03 | -0,03 |
| 22 | 82,470 | 79,526 | 1,248 | (76,938; 82,114) | 2,945 | 1,35 | 1,38 |
| 23 | 78,081 | 76,936 | 0,501 | (75,896; 77,976) | 1,145 | 0,46 | 0,46 |
| 24 | 77,405 | 76,691 | 0,501 | (75,651; 77,730) | 0,714 | 0,29 | 0,28 |
| 25 | 74,015 | 76,804 | 0,493 | (75,782; 77,827) | -2,790 | -1,13 | -1,14 |
| 26 | 76,104 | 76,804 | 0,493 | (75,782; 77,827) | -0,700 | -0,28 | -0,28 |
| 27 | 79,343 | 76,804 | 0,493 | (75,782; 77,827) | 2,539 | 1,03 | 1,03 |

| Obs | HI | Cook’s D | DFITS |  |
| --- | --- | --- | --- | --- |
| 1 | 0,231482 | 0,07 | 0,57893 |  |
| 2 | 0,231482 | 0,01 | -0,23848 |  |
| 3 | 0,351061 | 0,44 | -1,60799 | R |
| 4 | 0,351061 | 0,08 | -0,60963 |  |
| 5 | 0,178686 | 0,10 | -0,71720 |  |
| 6 | 0,178686 | 0,10 | -0,72253 |  |
| 7 | 0,344801 | 0,11 | -0,75566 |  |
| 8 | 0,344801 | 0,11 | -0,72981 |  |
| 9 | 0,217619 | 0,01 | -0,21958 |  |
| 10 | 0,217619 | 0,00 | -0,07877 |  |
| 11 | 0,131235 | 0,09 | 0,72355 |  |
| 12 | 0,131235 | 0,00 | 0,08482 |  |
| 13 | 0,226191 | 0,02 | -0,30347 |  |
| 14 | 0,226191 | 0,00 | 0,04440 |  |
| 15 | 0,143742 | 0,01 | 0,24187 |  |
| 16 | 0,143742 | 0,02 | -0,30962 |  |
| 17 | 0,206936 | 0,01 | 0,18445 |  |
| 18 | 0,206936 | 0,14 | 0,85845 |  |
| 19 | 0,134128 | 0,01 | 0,22924 |  |
| 20 | 0,200265 | 0,15 | 0,91317 |  |
| 21 | 0,161088 | 0,00 | -0,01381 |  |
| 22 | 0,246222 | 0,12 | 0,78631 |  |
| 23 | 0,039752 | 0,00 | 0,09278 |  |
| 24 | 0,039724 | 0,00 | 0,05771 |  |
| 25 | 0,038439 | 0,01 | -0,22770 |  |
| 26 | 0,038439 | 0,00 | -0,05557 |  |
| 27 | 0,038439 | 0,01 | 0,20614 |  |

R  Large residual

## Forward Selection of Terms

Achieved minimum BIC =  138,91

## Coded Coefficients

| Term | Coef | SE Coef | 95% CI | T-Value | P-Value | VIF |
| --- | --- | --- | --- | --- | --- | --- |
| Constant | 79,514 | 0,497 | (78,483; 80,546) | 159,88 | 0,000 |  |
| Lac | 9,68 | 1,03 | (7,54; 11,82) | 9,39 | 0,000 | 1,08 |
| HPMC\_Visc | -2,539 | 0,867 | (-4,338; -0,741) | -2,93 | 0,008 | 1,00 |
| HPMC\_HP | 2,617 | 0,986 | (0,572; 4,662) | 2,65 | 0,014 | 1,00 |
| Lac\*HPMC\_Visc | -4,66 | 2,02 | (-8,84; -0,47) | -2,31 | 0,031 | 1,08 |

## Model Summary

| S | R-sq | R-sq(adj) | PRESS | R-sq(pred) | AICc | BIC |
| --- | --- | --- | --- | --- | --- | --- |
| 2,43413 | 85,33% | 82,66% | 213,132 | 76,01% | 135,33 | 138,91 |

## Analysis of Variance

| Source | DF | Seq SS | Contribution | Adj SS | Adj MS | F-Value | P-Value |
| --- | --- | --- | --- | --- | --- | --- | --- |
| Model | 4 | 758,10 | 85,33% | 758,10 | 189,524 | 31,99 | 0,000 |
| Linear | 3 | 726,50 | 81,77% | 610,75 | 203,584 | 34,36 | 0,000 |
| Lac | 1 | 637,88 | 71,80% | 522,13 | 522,132 | 88,12 | 0,000 |
| HPMC\_Visc | 1 | 46,87 | 5,28% | 50,79 | 50,790 | 8,57 | 0,008 |
| HPMC\_HP | 1 | 41,75 | 4,70% | 41,75 | 41,747 | 7,05 | 0,014 |
| 2-Way Interaction | 1 | 31,59 | 3,56% | 31,59 | 31,593 | 5,33 | 0,031 |
| Lac\*HPMC\_Visc | 1 | 31,59 | 3,56% | 31,59 | 31,593 | 5,33 | 0,031 |
| Error | 22 | 130,35 | 14,67% | 130,35 | 5,925 |  |  |
| Lack-of-Fit | 20 | 116,99 | 13,17% | 116,99 | 5,850 | 0,88 | 0,661 |
| Pure Error | 2 | 13,36 | 1,50% | 13,36 | 6,678 |  |  |
| Total | 26 | 888,45 | 100,00% |  |  |  |  |

## Regression Equation in Uncoded Units

|  |  |  |
| --- | --- | --- |
| F\_mean\_13h(780min) | = | 12,2 + 103,4 Lac + 0,00174 HPMC\_Visc + 2,578 HPMC\_HP - 0,00479 Lac\*HPMC\_Visc |

## Fits and Diagnostics for All Observations

| Obs | F\_mean\_13h(780min) | Fit | SE Fit | 95% CI | Resid | Std Resid | Del Resid |
| --- | --- | --- | --- | --- | --- | --- | --- |
| 1 | 75,207 | 72,939 | 1,171 | (70,510; 75,368) | 2,268 | 1,06 | 1,07 |
| 2 | 85,112 | 85,932 | 1,171 | (83,503; 88,361) | -0,820 | -0,38 | -0,38 |
| 3 | 68,335 | 72,563 | 1,442 | (69,572; 75,554) | -4,228 | -2,16 | -2,37 |
| 4 | 77,455 | 79,030 | 1,442 | (76,039; 82,021) | -1,575 | -0,80 | -0,80 |
| 5 | 72,004 | 75,350 | 1,029 | (73,216; 77,484) | -3,346 | -1,52 | -1,57 |
| 6 | 84,615 | 88,051 | 1,029 | (85,917; 90,185) | -3,435 | -1,56 | -1,61 |
| 7 | 73,784 | 75,768 | 1,429 | (72,803; 78,732) | -1,984 | -1,01 | -1,01 |
| 8 | 80,481 | 82,137 | 1,429 | (79,173; 85,101) | -1,656 | -0,84 | -0,83 |
| 9 | 71,666 | 73,042 | 1,136 | (70,687; 75,397) | -1,376 | -0,64 | -0,63 |
| 10 | 86,275 | 85,943 | 1,136 | (83,588; 88,298) | 0,332 | 0,15 | 0,15 |
| 11 | 77,673 | 73,390 | 0,882 | (71,561; 75,219) | 4,282 | 1,89 | 2,01 |
| 12 | 82,648 | 82,179 | 0,882 | (80,351; 84,008) | 0,469 | 0,21 | 0,20 |
| 13 | 74,708 | 75,892 | 1,158 | (73,492; 78,293) | -1,184 | -0,55 | -0,54 |
| 14 | 88,777 | 88,834 | 1,158 | (86,433; 91,235) | -0,057 | -0,03 | -0,03 |
| 15 | 76,503 | 75,067 | 0,923 | (73,154; 76,981) | 1,435 | 0,64 | 0,63 |
| 16 | 81,890 | 83,430 | 0,923 | (81,517; 85,344) | -1,540 | -0,68 | -0,68 |
| 17 | 70,162 | 69,298 | 1,107 | (67,002; 71,594) | 0,864 | 0,40 | 0,39 |
| 18 | 93,340 | 90,401 | 1,107 | (88,104; 92,697) | 2,939 | 1,36 | 1,38 |
| 19 | 83,434 | 81,861 | 0,891 | (80,012; 83,709) | 1,574 | 0,69 | 0,69 |
| 20 | 80,136 | 76,524 | 1,089 | (74,265; 78,783) | 3,612 | 1,66 | 1,73 |
| 21 | 77,183 | 77,356 | 0,977 | (75,329; 79,382) | -0,173 | -0,08 | -0,08 |
| 22 | 85,226 | 82,425 | 1,208 | (79,921; 84,930) | 2,800 | 1,33 | 1,35 |
| 23 | 81,026 | 79,980 | 0,485 | (78,974; 80,987) | 1,045 | 0,44 | 0,43 |
| 24 | 80,490 | 79,749 | 0,485 | (78,743; 80,755) | 0,741 | 0,31 | 0,30 |
| 25 | 77,114 | 79,849 | 0,477 | (78,859; 80,839) | -2,735 | -1,15 | -1,15 |
| 26 | 79,194 | 79,849 | 0,477 | (78,859; 80,839) | -0,655 | -0,27 | -0,27 |
| 27 | 82,251 | 79,849 | 0,477 | (78,859; 80,839) | 2,402 | 1,01 | 1,01 |

| Obs | HI | Cook’s D | DFITS |  |
| --- | --- | --- | --- | --- |
| 1 | 0,231482 | 0,07 | 0,58514 |  |
| 2 | 0,231482 | 0,01 | -0,20668 |  |
| 3 | 0,351061 | 0,50 | -1,74471 | R |
| 4 | 0,351061 | 0,07 | -0,58588 |  |
| 5 | 0,178686 | 0,10 | -0,73035 |  |
| 6 | 0,178686 | 0,11 | -0,75238 |  |
| 7 | 0,344801 | 0,11 | -0,73069 |  |
| 8 | 0,344801 | 0,07 | -0,60550 |  |
| 9 | 0,217619 | 0,02 | -0,33229 |  |
| 10 | 0,217619 | 0,00 | 0,07957 |  |
| 11 | 0,131235 | 0,11 | 0,78295 |  |
| 12 | 0,131235 | 0,00 | 0,07854 |  |
| 13 | 0,226191 | 0,02 | -0,29422 |  |
| 14 | 0,226191 | 0,00 | -0,01413 |  |
| 15 | 0,143742 | 0,01 | 0,25749 |  |
| 16 | 0,143742 | 0,02 | -0,27668 |  |
| 17 | 0,206936 | 0,01 | 0,19957 |  |
| 18 | 0,206936 | 0,10 | 0,70692 |  |
| 19 | 0,134128 | 0,01 | 0,27015 |  |
| 20 | 0,200265 | 0,14 | 0,86728 |  |
| 21 | 0,161088 | 0,00 | -0,03324 |  |
| 22 | 0,246222 | 0,11 | 0,77133 |  |
| 23 | 0,039752 | 0,00 | 0,08751 |  |
| 24 | 0,039724 | 0,00 | 0,06191 |  |
| 25 | 0,038439 | 0,01 | -0,23087 |  |
| 26 | 0,038439 | 0,00 | -0,05373 |  |
| 27 | 0,038439 | 0,01 | 0,20127 |  |

R  Large residual

## Forward Selection of Terms

Achieved minimum BIC =  135,75

## Coded Coefficients

| Term | Coef | SE Coef | 95% CI | T-Value | P-Value | VIF |
| --- | --- | --- | --- | --- | --- | --- |
| Constant | 82,333 | 0,469 | (81,360; 83,306) | 175,52 | 0,000 |  |
| Lac | 9,515 | 0,972 | (7,499; 11,532) | 9,79 | 0,000 | 1,08 |
| HPMC\_Visc | -2,283 | 0,818 | (-3,980; -0,586) | -2,79 | 0,011 | 1,00 |
| HPMC\_HP | 2,488 | 0,930 | (0,559; 4,416) | 2,68 | 0,014 | 1,00 |
| Lac\*HPMC\_Visc | -4,25 | 1,90 | (-8,19; -0,30) | -2,23 | 0,036 | 1,08 |

## Model Summary

| S | R-sq | R-sq(adj) | PRESS | R-sq(pred) | AICc | BIC |
| --- | --- | --- | --- | --- | --- | --- |
| 2,29584 | 86,01% | 83,47% | 189,246 | 77,17% | 132,17 | 135,75 |

## Analysis of Variance

| Source | DF | Seq SS | Contribution | Adj SS | Adj MS | F-Value | P-Value |
| --- | --- | --- | --- | --- | --- | --- | --- |
| Model | 4 | 712,97 | 86,01% | 712,97 | 178,242 | 33,82 | 0,000 |
| Linear | 3 | 686,66 | 82,84% | 580,19 | 193,397 | 36,69 | 0,000 |
| Lac | 1 | 611,24 | 73,74% | 504,77 | 504,767 | 95,77 | 0,000 |
| HPMC\_Visc | 1 | 37,70 | 4,55% | 41,05 | 41,052 | 7,79 | 0,011 |
| HPMC\_HP | 1 | 37,72 | 4,55% | 37,72 | 37,718 | 7,16 | 0,014 |
| 2-Way Interaction | 1 | 26,31 | 3,17% | 26,31 | 26,311 | 4,99 | 0,036 |
| Lac\*HPMC\_Visc | 1 | 26,31 | 3,17% | 26,31 | 26,311 | 4,99 | 0,036 |
| Error | 22 | 115,96 | 13,99% | 115,96 | 5,271 |  |  |
| Lack-of-Fit | 20 | 103,76 | 12,52% | 103,76 | 5,188 | 0,85 | 0,671 |
| Pure Error | 2 | 12,20 | 1,47% | 12,20 | 6,100 |  |  |
| Total | 26 | 828,93 | 100,00% |  |  |  |  |

## Regression Equation in Uncoded Units

|  |  |  |
| --- | --- | --- |
| F\_mean\_14h(840min) | = | 18,5 + 97,1 Lac + 0,00160 HPMC\_Visc + 2,451 HPMC\_HP - 0,00437 Lac\*HPMC\_Visc |

## Fits and Diagnostics for All Observations

| Obs | F\_mean\_14h(840min) | Fit | SE Fit | 95% CI | Resid | Std Resid | Del Resid |
| --- | --- | --- | --- | --- | --- | --- | --- |
| 1 | 77,720 | 75,895 | 1,105 | (73,604; 78,185) | 1,826 | 0,91 | 0,90 |
| 2 | 87,872 | 88,435 | 1,105 | (86,144; 90,726) | -0,563 | -0,28 | -0,27 |
| 3 | 71,142 | 75,596 | 1,360 | (72,775; 78,417) | -4,454 | -2,41 | -2,74 |
| 4 | 80,866 | 82,182 | 1,360 | (79,360; 85,003) | -1,316 | -0,71 | -0,70 |
| 5 | 74,970 | 78,189 | 0,970 | (76,176; 80,201) | -3,219 | -1,55 | -1,60 |
| 6 | 87,433 | 90,463 | 0,970 | (88,450; 92,476) | -3,030 | -1,46 | -1,50 |
| 7 | 77,141 | 78,643 | 1,348 | (75,848; 81,439) | -1,503 | -0,81 | -0,80 |
| 8 | 84,045 | 85,140 | 1,348 | (82,344; 87,936) | -1,094 | -0,59 | -0,58 |
| 9 | 74,297 | 75,993 | 1,071 | (73,772; 78,214) | -1,696 | -0,84 | -0,83 |
| 10 | 89,522 | 88,450 | 1,071 | (86,229; 90,671) | 1,072 | 0,53 | 0,52 |
| 11 | 80,758 | 76,361 | 0,832 | (74,637; 78,086) | 4,397 | 2,05 | 2,23 |
| 12 | 85,507 | 85,066 | 0,832 | (83,341; 86,791) | 0,441 | 0,21 | 0,20 |
| 13 | 77,648 | 78,702 | 1,092 | (76,438; 80,967) | -1,055 | -0,52 | -0,51 |
| 14 | 90,387 | 91,196 | 1,092 | (88,932; 93,461) | -0,809 | -0,40 | -0,39 |
| 15 | 79,239 | 77,960 | 0,870 | (76,155; 79,765) | 1,279 | 0,60 | 0,59 |
| 16 | 84,863 | 86,275 | 0,870 | (84,470; 88,081) | -1,413 | -0,67 | -0,66 |
| 17 | 73,177 | 72,314 | 1,044 | (70,148; 74,480) | 0,863 | 0,42 | 0,41 |
| 18 | 94,946 | 92,939 | 1,044 | (90,773; 95,105) | 2,006 | 0,98 | 0,98 |
| 19 | 86,403 | 84,432 | 0,841 | (82,689; 86,176) | 1,970 | 0,92 | 0,92 |
| 20 | 82,719 | 79,621 | 1,027 | (77,490; 81,752) | 3,098 | 1,51 | 1,56 |
| 21 | 80,080 | 80,257 | 0,921 | (78,346; 82,168) | -0,177 | -0,08 | -0,08 |
| 22 | 87,653 | 85,085 | 1,139 | (82,722; 87,447) | 2,568 | 1,29 | 1,31 |
| 23 | 83,594 | 82,742 | 0,458 | (81,793; 83,691) | 0,852 | 0,38 | 0,37 |
| 24 | 83,371 | 82,520 | 0,458 | (81,571; 83,469) | 0,851 | 0,38 | 0,37 |
| 25 | 80,007 | 82,627 | 0,450 | (81,693; 83,560) | -2,620 | -1,16 | -1,17 |
| 26 | 82,056 | 82,627 | 0,450 | (81,693; 83,560) | -0,571 | -0,25 | -0,25 |
| 27 | 84,924 | 82,627 | 0,450 | (81,693; 83,560) | 2,297 | 1,02 | 1,02 |

| Obs | HI | Cook’s D | DFITS |  |
| --- | --- | --- | --- | --- |
| 1 | 0,231482 | 0,05 | 0,49572 |  |
| 2 | 0,231482 | 0,00 | -0,15038 |  |
| 3 | 0,351061 | 0,63 | -2,01682 | R |
| 4 | 0,351061 | 0,05 | -0,51722 |  |
| 5 | 0,178686 | 0,10 | -0,74680 |  |
| 6 | 0,178686 | 0,09 | -0,69810 |  |
| 7 | 0,344801 | 0,07 | -0,58180 |  |
| 8 | 0,344801 | 0,04 | -0,42074 |  |
| 9 | 0,217619 | 0,04 | -0,43733 |  |
| 10 | 0,217619 | 0,02 | 0,27367 |  |
| 11 | 0,131235 | 0,13 | 0,86792 | R |
| 12 | 0,131235 | 0,00 | 0,07832 |  |
| 13 | 0,226191 | 0,02 | -0,27758 |  |
| 14 | 0,226191 | 0,01 | -0,21237 |  |
| 15 | 0,143742 | 0,01 | 0,24299 |  |
| 16 | 0,143742 | 0,01 | -0,26894 |  |
| 17 | 0,206936 | 0,01 | 0,21143 |  |
| 18 | 0,206936 | 0,05 | 0,50085 |  |
| 19 | 0,134128 | 0,03 | 0,36172 |  |
| 20 | 0,200265 | 0,11 | 0,77911 |  |
| 21 | 0,161088 | 0,00 | -0,03603 |  |
| 22 | 0,246222 | 0,11 | 0,74819 |  |
| 23 | 0,039752 | 0,00 | 0,07557 |  |
| 24 | 0,039724 | 0,00 | 0,07538 |  |
| 25 | 0,038439 | 0,01 | -0,23467 |  |
| 26 | 0,038439 | 0,00 | -0,04961 |  |
| 27 | 0,038439 | 0,01 | 0,20420 |  |

R  Large residual

## Forward Selection of Terms

Achieved minimum BIC =  134,21

## Coded Coefficients

| Term | Coef | SE Coef | 95% CI | T-Value | P-Value | VIF |
| --- | --- | --- | --- | --- | --- | --- |
| Constant | 84,864 | 0,456 | (83,919; 85,810) | 186,13 | 0,000 |  |
| Lac | 9,185 | 0,945 | (7,225; 11,145) | 9,72 | 0,000 | 1,08 |
| HPMC\_Visc | -2,089 | 0,795 | (-3,738; -0,440) | -2,63 | 0,015 | 1,00 |
| HPMC\_HP | 2,276 | 0,904 | (0,401; 4,151) | 2,52 | 0,020 | 1,00 |
| Lac\*HPMC\_Visc | -3,64 | 1,85 | (-7,48; 0,19) | -1,97 | 0,061 | 1,08 |

## Model Summary

| S | R-sq | R-sq(adj) | PRESS | R-sq(pred) | AICc | BIC |
| --- | --- | --- | --- | --- | --- | --- |
| 2,23159 | 85,48% | 82,83% | 179,732 | 76,17% | 130,64 | 134,21 |

## Analysis of Variance

| Source | DF | Seq SS | Contribution | Adj SS | Adj MS | F-Value | P-Value |
| --- | --- | --- | --- | --- | --- | --- | --- |
| Model | 4 | 644,75 | 85,48% | 644,75 | 161,187 | 32,37 | 0,000 |
| Linear | 3 | 625,40 | 82,91% | 533,46 | 177,820 | 35,71 | 0,000 |
| Lac | 1 | 562,25 | 74,54% | 470,31 | 470,307 | 94,44 | 0,000 |
| HPMC\_Visc | 1 | 31,58 | 4,19% | 34,38 | 34,380 | 6,90 | 0,015 |
| HPMC\_HP | 1 | 31,58 | 4,19% | 31,58 | 31,576 | 6,34 | 0,020 |
| 2-Way Interaction | 1 | 19,35 | 2,56% | 19,35 | 19,347 | 3,88 | 0,061 |
| Lac\*HPMC\_Visc | 1 | 19,35 | 2,56% | 19,35 | 19,347 | 3,88 | 0,061 |
| Error | 22 | 109,56 | 14,52% | 109,56 | 4,980 |  |  |
| Lack-of-Fit | 20 | 98,91 | 13,11% | 98,91 | 4,945 | 0,93 | 0,640 |
| Pure Error | 2 | 10,65 | 1,41% | 10,65 | 5,325 |  |  |
| Total | 26 | 754,31 | 100,00% |  |  |  |  |

## Regression Equation in Uncoded Units

|  |  |  |
| --- | --- | --- |
| F\_mean\_15h(900min) | = | 27,2 + 87,4 Lac + 0,001336 HPMC\_Visc + 2,242 HPMC\_HP - 0,00375 Lac\*HPMC\_Visc |

## Fits and Diagnostics for All Observations

| Obs | F\_mean\_15h(900min) | Fit | SE Fit | 95% CI | Resid | Std Resid | Del Resid |
| --- | --- | --- | --- | --- | --- | --- | --- |
| 1 | 80,606 | 78,821 | 1,074 | (76,595; 81,048) | 1,784 | 0,91 | 0,91 |
| 2 | 90,402 | 90,600 | 1,074 | (88,374; 92,827) | -0,199 | -0,10 | -0,10 |
| 3 | 73,720 | 78,377 | 1,322 | (75,635; 81,119) | -4,656 | -2,59 | -3,04 |
| 4 | 83,969 | 85,049 | 1,322 | (82,307; 87,791) | -1,080 | -0,60 | -0,59 |
| 5 | 77,633 | 80,913 | 0,943 | (78,956; 82,869) | -3,279 | -1,62 | -1,69 |
| 6 | 89,900 | 92,463 | 0,943 | (90,507; 94,420) | -2,564 | -1,27 | -1,29 |
| 7 | 80,197 | 81,162 | 1,310 | (78,445; 83,880) | -0,965 | -0,53 | -0,53 |
| 8 | 87,181 | 87,758 | 1,310 | (85,041; 90,476) | -0,577 | -0,32 | -0,31 |
| 9 | 76,944 | 78,909 | 1,041 | (76,750; 81,068) | -1,965 | -1,00 | -1,00 |
| 10 | 92,342 | 90,616 | 1,041 | (88,457; 92,775) | 1,726 | 0,87 | 0,87 |
| 11 | 83,565 | 79,138 | 0,808 | (77,461; 80,814) | 4,427 | 2,13 | 2,33 |
| 12 | 87,990 | 87,627 | 0,808 | (85,951; 89,304) | 0,363 | 0,17 | 0,17 |
| 13 | 80,335 | 81,389 | 1,061 | (79,188; 83,590) | -1,054 | -0,54 | -0,53 |
| 14 | 91,608 | 93,128 | 1,061 | (90,927; 95,329) | -1,520 | -0,77 | -0,77 |
| 15 | 81,597 | 80,589 | 0,846 | (78,834; 82,344) | 1,008 | 0,49 | 0,48 |
| 16 | 87,555 | 88,745 | 0,846 | (86,990; 90,500) | -1,190 | -0,58 | -0,57 |
| 17 | 75,884 | 75,265 | 1,015 | (73,159; 77,370) | 0,620 | 0,31 | 0,31 |
| 18 | 95,792 | 95,002 | 1,015 | (92,896; 97,107) | 0,790 | 0,40 | 0,39 |
| 19 | 89,091 | 86,785 | 0,817 | (85,090; 88,480) | 2,305 | 1,11 | 1,12 |
| 20 | 85,073 | 82,383 | 0,999 | (80,311; 84,454) | 2,690 | 1,35 | 1,38 |
| 21 | 82,700 | 82,965 | 0,896 | (81,108; 84,823) | -0,266 | -0,13 | -0,13 |
| 22 | 89,972 | 87,382 | 1,107 | (85,086; 89,679) | 2,590 | 1,34 | 1,36 |
| 23 | 85,860 | 85,239 | 0,445 | (84,316; 86,161) | 0,621 | 0,28 | 0,28 |
| 24 | 86,024 | 85,036 | 0,445 | (84,113; 85,958) | 0,988 | 0,45 | 0,44 |
| 25 | 82,743 | 85,133 | 0,438 | (84,226; 86,040) | -2,390 | -1,09 | -1,10 |
| 26 | 84,715 | 85,133 | 0,438 | (84,226; 86,040) | -0,418 | -0,19 | -0,19 |
| 27 | 87,342 | 85,133 | 0,438 | (84,226; 86,040) | 2,209 | 1,01 | 1,01 |

| Obs | HI | Cook’s D | DFITS |  |
| --- | --- | --- | --- | --- |
| 1 | 0,231482 | 0,05 | 0,49855 |  |
| 2 | 0,231482 | 0,00 | -0,05446 |  |
| 3 | 0,351061 | 0,73 | -2,23262 | R |
| 4 | 0,351061 | 0,04 | -0,43515 |  |
| 5 | 0,178686 | 0,11 | -0,78751 |  |
| 6 | 0,178686 | 0,07 | -0,59999 |  |
| 7 | 0,344801 | 0,03 | -0,38108 |  |
| 8 | 0,344801 | 0,01 | -0,22699 |  |
| 9 | 0,217619 | 0,06 | -0,52491 |  |
| 10 | 0,217619 | 0,04 | 0,45854 |  |
| 11 | 0,131235 | 0,14 | 0,90704 | R |
| 12 | 0,131235 | 0,00 | 0,06629 |  |
| 13 | 0,226191 | 0,02 | -0,28551 |  |
| 14 | 0,226191 | 0,04 | -0,41469 |  |
| 15 | 0,143742 | 0,01 | 0,19655 |  |
| 16 | 0,143742 | 0,01 | -0,23253 |  |
| 17 | 0,206936 | 0,01 | 0,15599 |  |
| 18 | 0,206936 | 0,01 | 0,19919 |  |
| 19 | 0,134128 | 0,04 | 0,43939 |  |
| 20 | 0,200265 | 0,09 | 0,68808 |  |
| 21 | 0,161088 | 0,00 | -0,05565 |  |
| 22 | 0,246222 | 0,12 | 0,77873 |  |
| 23 | 0,039752 | 0,00 | 0,05657 |  |
| 24 | 0,039724 | 0,00 | 0,09021 |  |
| 25 | 0,038439 | 0,01 | -0,21940 |  |
| 26 | 0,038439 | 0,00 | -0,03732 |  |
| 27 | 0,038439 | 0,01 | 0,20196 |  |

R  Large residual

## Forward Selection of Terms

Achieved minimum BIC =  133,57

## Coded Coefficients

| Term | Coef | SE Coef | 95% CI | T-Value | P-Value | VIF |
| --- | --- | --- | --- | --- | --- | --- |
| Constant | 87,151 | 0,451 | (86,217; 88,086) | 193,43 | 0,000 |  |
| Lac | 8,692 | 0,934 | (6,755; 10,628) | 9,31 | 0,000 | 1,08 |
| HPMC\_Visc | -1,910 | 0,786 | (-3,539; -0,280) | -2,43 | 0,024 | 1,00 |
| HPMC\_HP | 2,096 | 0,893 | (0,243; 3,948) | 2,35 | 0,028 | 1,00 |
| Lac\*HPMC\_Visc | -3,50 | 1,83 | (-7,29; 0,29) | -1,92 | 0,068 | 1,08 |

## Model Summary

| S | R-sq | R-sq(adj) | PRESS | R-sq(pred) | AICc | BIC |
| --- | --- | --- | --- | --- | --- | --- |
| 2,20523 | 84,32% | 81,47% | 177,542 | 73,98% | 130,00 | 133,57 |

## Analysis of Variance

| Source | DF | Seq SS | Contribution | Adj SS | Adj MS | F-Value | P-Value |
| --- | --- | --- | --- | --- | --- | --- | --- |
| Model | 4 | 575,278 | 84,32% | 575,278 | 143,819 | 29,57 | 0,000 |
| Linear | 3 | 557,419 | 81,70% | 474,297 | 158,099 | 32,51 | 0,000 |
| Lac | 1 | 504,288 | 73,91% | 421,165 | 421,165 | 86,60 | 0,000 |
| HPMC\_Visc | 1 | 26,365 | 3,86% | 28,723 | 28,723 | 5,91 | 0,024 |
| HPMC\_HP | 1 | 26,767 | 3,92% | 26,767 | 26,767 | 5,50 | 0,028 |
| 2-Way Interaction | 1 | 17,858 | 2,62% | 17,858 | 17,858 | 3,67 | 0,068 |
| Lac\*HPMC\_Visc | 1 | 17,858 | 2,62% | 17,858 | 17,858 | 3,67 | 0,068 |
| Error | 22 | 106,987 | 15,68% | 106,987 | 4,863 |  |  |
| Lack-of-Fit | 20 | 97,558 | 14,30% | 97,558 | 4,878 | 1,03 | 0,603 |
| Pure Error | 2 | 9,429 | 1,38% | 9,429 | 4,715 |  |  |
| Total | 26 | 682,265 | 100,00% |  |  |  |  |

## Regression Equation in Uncoded Units

|  |  |  |
| --- | --- | --- |
| F\_mean\_16h(960min) | = | 32,5 + 83,4 Lac + 0,001309 HPMC\_Visc + 2,065 HPMC\_HP - 0,00360 Lac\*HPMC\_Visc |

## Fits and Diagnostics for All Observations

| Obs | F\_mean\_16h(960min) | Fit | SE Fit | 95% CI | Resid | Std Resid | Del Resid |
| --- | --- | --- | --- | --- | --- | --- | --- |
| 1 | 82,777 | 81,408 | 1,061 | (79,207; 83,608) | 1,369 | 0,71 | 0,70 |
| 2 | 93,041 | 92,592 | 1,061 | (90,391; 94,792) | 0,449 | 0,23 | 0,23 |
| 3 | 76,241 | 81,120 | 1,307 | (78,411; 83,830) | -4,879 | -2,75 | -3,31 |
| 4 | 86,554 | 87,398 | 1,307 | (84,688; 90,108) | -0,844 | -0,47 | -0,47 |
| 5 | 80,143 | 83,339 | 0,932 | (81,406; 85,272) | -3,196 | -1,60 | -1,66 |
| 6 | 92,409 | 94,303 | 0,932 | (92,370; 96,237) | -1,894 | -0,95 | -0,95 |
| 7 | 83,119 | 83,687 | 1,295 | (81,001; 86,372) | -0,567 | -0,32 | -0,31 |
| 8 | 89,650 | 89,891 | 1,295 | (87,206; 92,577) | -0,241 | -0,14 | -0,13 |
| 9 | 79,338 | 81,490 | 1,029 | (79,357; 83,623) | -2,152 | -1,10 | -1,11 |
| 10 | 94,542 | 92,605 | 1,029 | (90,472; 94,739) | 1,936 | 0,99 | 0,99 |
| 11 | 86,168 | 81,778 | 0,799 | (80,121; 83,435) | 4,390 | 2,14 | 2,34 |
| 12 | 90,270 | 89,802 | 0,799 | (88,145; 91,458) | 0,468 | 0,23 | 0,22 |
| 13 | 82,722 | 83,773 | 1,049 | (81,598; 85,948) | -1,051 | -0,54 | -0,53 |
| 14 | 92,607 | 94,918 | 1,049 | (92,743; 97,093) | -2,312 | -1,19 | -1,20 |
| 15 | 83,948 | 83,122 | 0,836 | (81,388; 84,856) | 0,826 | 0,40 | 0,40 |
| 16 | 89,920 | 90,825 | 0,836 | (89,091; 92,559) | -0,905 | -0,44 | -0,44 |
| 17 | 78,580 | 78,047 | 1,003 | (75,967; 80,128) | 0,532 | 0,27 | 0,27 |
| 18 | 96,318 | 96,745 | 1,003 | (94,664; 98,825) | -0,426 | -0,22 | -0,21 |
| 19 | 91,500 | 88,906 | 0,808 | (87,231; 90,581) | 2,594 | 1,26 | 1,28 |
| 20 | 87,193 | 84,880 | 0,987 | (82,834; 86,927) | 2,313 | 1,17 | 1,18 |
| 21 | 85,260 | 85,400 | 0,885 | (83,565; 87,236) | -0,140 | -0,07 | -0,07 |
| 22 | 92,111 | 89,468 | 1,094 | (87,198; 91,737) | 2,644 | 1,38 | 1,41 |
| 23 | 87,826 | 87,492 | 0,440 | (86,580; 88,404) | 0,333 | 0,15 | 0,15 |
| 24 | 88,425 | 87,305 | 0,440 | (86,394; 88,217) | 1,120 | 0,52 | 0,51 |
| 25 | 85,154 | 87,396 | 0,432 | (86,499; 88,293) | -2,242 | -1,04 | -1,04 |
| 26 | 87,173 | 87,396 | 0,432 | (86,499; 88,293) | -0,223 | -0,10 | -0,10 |
| 27 | 89,493 | 87,396 | 0,432 | (86,499; 88,293) | 2,097 | 0,97 | 0,97 |

| Obs | HI | Cook’s D | DFITS |  |
| --- | --- | --- | --- | --- |
| 1 | 0,231482 | 0,03 | 0,38413 |  |
| 2 | 0,231482 | 0,00 | 0,12466 |  |
| 3 | 0,351061 | 0,82 | -2,43449 | R |
| 4 | 0,351061 | 0,02 | -0,34299 |  |
| 5 | 0,178686 | 0,11 | -0,77520 |  |
| 6 | 0,178686 | 0,04 | -0,44098 |  |
| 7 | 0,344801 | 0,01 | -0,22579 |  |
| 8 | 0,344801 | 0,00 | -0,09591 |  |
| 9 | 0,217619 | 0,07 | -0,58480 |  |
| 10 | 0,217619 | 0,05 | 0,52337 |  |
| 11 | 0,131235 | 0,14 | 0,91099 | R |
| 12 | 0,131235 | 0,00 | 0,08662 |  |
| 13 | 0,226191 | 0,02 | -0,28812 |  |
| 14 | 0,226191 | 0,08 | -0,65078 |  |
| 15 | 0,143742 | 0,01 | 0,16268 |  |
| 16 | 0,143742 | 0,01 | -0,17837 |  |
| 17 | 0,206936 | 0,00 | 0,13548 |  |
| 18 | 0,206936 | 0,00 | -0,10847 |  |
| 19 | 0,134128 | 0,05 | 0,50483 |  |
| 20 | 0,200265 | 0,07 | 0,59224 |  |
| 21 | 0,161088 | 0,00 | -0,02962 |  |
| 22 | 0,246222 | 0,12 | 0,80675 |  |
| 23 | 0,039752 | 0,00 | 0,03069 |  |
| 24 | 0,039724 | 0,00 | 0,10361 |  |
| 25 | 0,038439 | 0,01 | -0,20769 |  |
| 26 | 0,038439 | 0,00 | -0,02018 |  |
| 27 | 0,038439 | 0,01 | 0,19361 |  |

R  Large residual

## Forward Selection of Terms

Achieved minimum BIC =  134,56

## Coded Coefficients

| Term | Coef | SE Coef | 95% CI | T-Value | P-Value | VIF |
| --- | --- | --- | --- | --- | --- | --- |
| Constant | 89,143 | 0,477 | (88,156; 90,130) | 186,86 | 0,000 |  |
| Lac | 8,442 | 0,953 | (6,470; 10,414) | 8,86 | 0,000 | 1,00 |
| HPMC\_Visc | -1,893 | 0,832 | (-3,614; -0,172) | -2,27 | 0,033 | 1,00 |
| HPMC\_HP | 1,739 | 0,946 | (-0,217; 3,696) | 1,84 | 0,079 | 1,00 |

## Model Summary

| S | R-sq | R-sq(adj) | PRESS | R-sq(pred) | AICc | BIC |
| --- | --- | --- | --- | --- | --- | --- |
| 2,33493 | 79,02% | 76,28% | 178,649 | 70,11% | 130,94 | 134,56 |

## Analysis of Variance

| Source | DF | Seq SS | Contribution | Adj SS | Adj MS | F-Value | P-Value |
| --- | --- | --- | --- | --- | --- | --- | --- |
| Model | 3 | 472,325 | 79,02% | 472,325 | 157,442 | 28,88 | 0,000 |
| Linear | 3 | 472,325 | 79,02% | 472,325 | 157,442 | 28,88 | 0,000 |
| Lac | 1 | 427,608 | 71,54% | 427,608 | 427,608 | 78,43 | 0,000 |
| HPMC\_Visc | 1 | 26,277 | 4,40% | 28,215 | 28,215 | 5,18 | 0,033 |
| HPMC\_HP | 1 | 18,440 | 3,09% | 18,440 | 18,440 | 3,38 | 0,079 |
| Error | 23 | 125,393 | 20,98% | 125,393 | 5,452 |  |  |
| Lack-of-Fit | 21 | 116,888 | 19,56% | 116,888 | 5,566 | 1,31 | 0,522 |
| Pure Error | 2 | 8,505 | 1,42% | 8,505 | 4,253 |  |  |
| Total | 26 | 597,718 | 100,00% |  |  |  |  |

## Regression Equation in Uncoded Units

|  |  |  |
| --- | --- | --- |
| F\_mean\_17h(1020min) | = | 62,59 + 33,77 Lac - 0,000487 HPMC\_Visc + 1,714 HPMC\_HP |

## Fits and Diagnostics for All Observations

| Obs | F\_mean\_17h(1020min) | Fit | SE Fit | 95% CI | Resid | Std Resid | Del Resid |
| --- | --- | --- | --- | --- | --- | --- | --- |
| 1 | 86,287 | 85,015 | 0,976 | (82,997; 87,034) | 1,271 | 0,60 | 0,59 |
| 2 | 94,993 | 93,458 | 0,976 | (91,439; 95,476) | 1,536 | 0,72 | 0,72 |
| 3 | 78,511 | 82,310 | 1,130 | (79,972; 84,647) | -3,799 | -1,86 | -1,97 |
| 4 | 88,721 | 90,752 | 1,130 | (88,414; 93,089) | -2,030 | -0,99 | -0,99 |
| 5 | 82,549 | 86,508 | 0,853 | (84,743; 88,273) | -3,959 | -1,82 | -1,93 |
| 6 | 94,030 | 94,950 | 0,853 | (93,185; 96,715) | -0,920 | -0,42 | -0,42 |
| 7 | 85,468 | 84,403 | 1,100 | (82,128; 86,678) | 1,065 | 0,52 | 0,51 |
| 8 | 91,504 | 92,845 | 1,100 | (90,570; 95,120) | -1,341 | -0,65 | -0,64 |
| 9 | 81,530 | 85,049 | 0,947 | (83,090; 87,008) | -3,519 | -1,65 | -1,72 |
| 10 | 96,321 | 93,491 | 0,947 | (91,532; 95,450) | 2,830 | 1,33 | 1,35 |
| 11 | 88,495 | 83,733 | 0,785 | (82,110; 85,356) | 4,761 | 2,17 | 2,37 |
| 12 | 92,370 | 92,175 | 0,785 | (90,552; 93,798) | 0,195 | 0,09 | 0,09 |
| 13 | 84,957 | 86,959 | 0,967 | (84,959; 88,959) | -2,002 | -0,94 | -0,94 |
| 14 | 93,700 | 95,401 | 0,967 | (93,401; 97,401) | -1,701 | -0,80 | -0,79 |
| 15 | 86,046 | 84,688 | 0,787 | (83,059; 86,317) | 1,358 | 0,62 | 0,61 |
| 16 | 91,962 | 93,130 | 0,787 | (91,501; 94,759) | -1,168 | -0,53 | -0,52 |
| 17 | 80,949 | 80,962 | 1,057 | (78,774; 83,149) | -0,013 | -0,01 | -0,01 |
| 18 | 96,721 | 97,846 | 1,057 | (95,659; 100,034) | -1,125 | -0,54 | -0,53 |
| 19 | 93,609 | 90,907 | 0,855 | (89,138; 92,676) | 2,702 | 1,24 | 1,26 |
| 20 | 89,036 | 86,950 | 1,045 | (84,789; 89,112) | 2,086 | 1,00 | 1,00 |
| 21 | 87,569 | 87,745 | 0,937 | (85,807; 89,684) | -0,177 | -0,08 | -0,08 |
| 22 | 93,783 | 91,101 | 1,159 | (88,705; 93,498) | 2,682 | 1,32 | 1,35 |
| 23 | 89,589 | 89,506 | 0,466 | (88,543; 90,469) | 0,083 | 0,04 | 0,04 |
| 24 | 90,600 | 89,354 | 0,465 | (88,391; 90,317) | 1,246 | 0,54 | 0,54 |
| 25 | 87,288 | 89,404 | 0,458 | (88,457; 90,351) | -2,116 | -0,92 | -0,92 |
| 26 | 89,453 | 89,404 | 0,458 | (88,457; 90,351) | 0,049 | 0,02 | 0,02 |
| 27 | 91,410 | 89,404 | 0,458 | (88,457; 90,351) | 2,006 | 0,88 | 0,87 |

| Obs | HI | Cook’s D | DFITS |  |
| --- | --- | --- | --- | --- |
| 1 | 0,174570 | 0,02 | 0,27166 |  |
| 2 | 0,174570 | 0,03 | 0,32941 |  |
| 3 | 0,234135 | 0,26 | -1,09050 |  |
| 4 | 0,234135 | 0,08 | -0,54925 |  |
| 5 | 0,133480 | 0,13 | -0,75584 |  |
| 6 | 0,133480 | 0,01 | -0,16308 |  |
| 7 | 0,221878 | 0,02 | 0,27162 |  |
| 8 | 0,221878 | 0,03 | -0,34308 |  |
| 9 | 0,164527 | 0,13 | -0,76202 |  |
| 10 | 0,164527 | 0,09 | 0,59875 |  |
| 11 | 0,112913 | 0,15 | 0,84667 | R |
| 12 | 0,112913 | 0,00 | 0,03096 |  |
| 13 | 0,171423 | 0,05 | -0,42742 |  |
| 14 | 0,171423 | 0,03 | -0,36101 |  |
| 15 | 0,113720 | 0,01 | 0,21831 |  |
| 16 | 0,113720 | 0,01 | -0,18730 |  |
| 17 | 0,205106 | 0,00 | -0,00319 |  |
| 18 | 0,205106 | 0,02 | -0,27026 |  |
| 19 | 0,134128 | 0,06 | 0,49565 |  |
| 20 | 0,200265 | 0,06 | 0,49990 |  |
| 21 | 0,161088 | 0,00 | -0,03539 |  |
| 22 | 0,246222 | 0,14 | 0,76925 |  |
| 23 | 0,039752 | 0,00 | 0,00718 |  |
| 24 | 0,039724 | 0,00 | 0,10903 |  |
| 25 | 0,038439 | 0,01 | -0,18421 |  |
| 26 | 0,038439 | 0,00 | 0,00419 |  |
| 27 | 0,038439 | 0,01 | 0,17426 |  |

R  Large residual

## Forward Selection of Terms

Achieved minimum BIC =  134,26

## Coded Coefficients

| Term | Coef | SE Coef | 95% CI | T-Value | P-Value | VIF |
| --- | --- | --- | --- | --- | --- | --- |
| Constant | 90,679 | 0,478 | (89,692; 91,666) | 189,58 | 0,000 |  |
| Lac | 7,766 | 0,986 | (5,730; 9,801) | 7,87 | 0,000 | 1,00 |
| HPMC\_Visc | -1,728 | 0,860 | (-3,503; 0,047) | -2,01 | 0,056 | 1,00 |

## Model Summary

| S | R-sq | R-sq(adj) | PRESS | R-sq(pred) | AICc | BIC |
| --- | --- | --- | --- | --- | --- | --- |
| 2,41584 | 73,34% | 71,12% | 180,264 | 65,69% | 130,89 | 134,26 |

## Analysis of Variance

| Source | DF | Seq SS | Contribution | Adj SS | Adj MS | F-Value | P-Value |
| --- | --- | --- | --- | --- | --- | --- | --- |
| Model | 2 | 385,400 | 73,34% | 385,400 | 192,700 | 33,02 | 0,000 |
| Linear | 2 | 385,400 | 73,34% | 385,400 | 192,700 | 33,02 | 0,000 |
| Lac | 1 | 361,840 | 68,86% | 361,840 | 361,840 | 62,00 | 0,000 |
| HPMC\_Visc | 1 | 23,561 | 4,48% | 23,561 | 23,561 | 4,04 | 0,056 |
| Error | 24 | 140,071 | 26,66% | 140,071 | 5,836 |  |  |
| Lack-of-Fit | 22 | 132,219 | 25,16% | 132,219 | 6,010 | 1,53 | 0,470 |
| Pure Error | 2 | 7,852 | 1,49% | 7,852 | 3,926 |  |  |
| Total | 26 | 525,472 | 100,00% |  |  |  |  |

## Regression Equation in Uncoded Units

|  |  |  |
| --- | --- | --- |
| F\_mean\_18h(1080min) | = | 81,15 + 31,06 Lac - 0,000444 HPMC\_Visc |

## Fits and Diagnostics for All Observations

| Obs | F\_mean\_18h(1080min) | Fit | SE Fit | 95% CI | Resid | Std Resid | Del Resid |
| --- | --- | --- | --- | --- | --- | --- | --- |
| 1 | 88,263 | 88,026 | 0,842 | (86,288; 89,764) | 0,237 | 0,10 | 0,10 |
| 2 | 96,841 | 95,792 | 0,842 | (94,054; 97,530) | 1,050 | 0,46 | 0,46 |
| 3 | 80,676 | 85,605 | 0,978 | (83,586; 87,623) | -4,929 | -2,23 | -2,45 |
| 4 | 90,400 | 93,370 | 0,978 | (91,352; 95,389) | -2,971 | -1,34 | -1,37 |
| 5 | 84,581 | 87,918 | 0,811 | (86,243; 89,592) | -3,337 | -1,47 | -1,50 |
| 6 | 95,182 | 95,684 | 0,811 | (94,009; 97,358) | -0,502 | -0,22 | -0,22 |
| 7 | 87,829 | 85,569 | 0,991 | (83,523; 87,614) | 2,261 | 1,03 | 1,03 |
| 8 | 92,846 | 93,334 | 0,991 | (91,289; 95,380) | -0,488 | -0,22 | -0,22 |
| 9 | 83,545 | 87,992 | 0,832 | (86,274; 89,710) | -4,447 | -1,96 | -2,09 |
| 10 | 97,777 | 95,758 | 0,832 | (94,040; 97,475) | 2,019 | 0,89 | 0,89 |
| 11 | 90,546 | 86,466 | 0,732 | (84,956; 87,977) | 4,079 | 1,77 | 1,86 |
| 12 | 94,342 | 94,232 | 0,732 | (92,721; 95,743) | 0,110 | 0,05 | 0,05 |
| 13 | 86,942 | 88,007 | 0,837 | (86,280; 89,734) | -1,065 | -0,47 | -0,46 |
| 14 | 94,594 | 95,773 | 0,837 | (94,046; 97,500) | -1,179 | -0,52 | -0,51 |
| 15 | 87,894 | 86,308 | 0,765 | (84,729; 87,887) | 1,586 | 0,69 | 0,68 |
| 16 | 93,801 | 94,074 | 0,765 | (92,495; 95,653) | -0,273 | -0,12 | -0,12 |
| 17 | 83,208 | 83,237 | 1,091 | (80,985; 85,490) | -0,029 | -0,01 | -0,01 |
| 18 | 97,049 | 98,769 | 1,091 | (96,516; 101,021) | -1,720 | -0,80 | -0,79 |
| 19 | 95,396 | 92,407 | 0,880 | (90,590; 94,224) | 2,990 | 1,33 | 1,35 |
| 20 | 90,556 | 88,951 | 1,078 | (86,727; 91,175) | 1,605 | 0,74 | 0,74 |
| 21 | 89,650 | 90,991 | 0,467 | (90,027; 91,954) | -1,341 | -0,57 | -0,56 |
| 22 | 95,014 | 90,879 | 0,465 | (89,919; 91,839) | 4,136 | 1,74 | 1,83 |
| 23 | 91,006 | 91,127 | 0,478 | (90,141; 92,114) | -0,121 | -0,05 | -0,05 |
| 24 | 92,525 | 91,145 | 0,480 | (90,154; 92,136) | 1,380 | 0,58 | 0,57 |
| 25 | 89,220 | 91,003 | 0,468 | (90,038; 91,968) | -1,783 | -0,75 | -0,75 |
| 26 | 91,582 | 91,003 | 0,468 | (90,038; 91,968) | 0,579 | 0,24 | 0,24 |
| 27 | 93,157 | 91,003 | 0,468 | (90,038; 91,968) | 2,154 | 0,91 | 0,91 |

| Obs | HI | Cook’s D | DFITS |  |
| --- | --- | --- | --- | --- |
| 1 | 0,121521 | 0,00 | 0,03811 |  |
| 2 | 0,121521 | 0,01 | 0,16954 |  |
| 3 | 0,163929 | 0,33 | -1,08637 | R |
| 4 | 0,163929 | 0,12 | -0,60627 |  |
| 5 | 0,112791 | 0,09 | -0,53643 |  |
| 6 | 0,112791 | 0,00 | -0,07700 |  |
| 7 | 0,168329 | 0,07 | 0,46221 |  |
| 8 | 0,168329 | 0,00 | -0,09770 |  |
| 9 | 0,118673 | 0,17 | -0,76855 |  |
| 10 | 0,118673 | 0,04 | 0,32525 |  |
| 11 | 0,091792 | 0,11 | 0,59146 |  |
| 12 | 0,091792 | 0,00 | 0,01484 |  |
| 13 | 0,119923 | 0,01 | -0,17068 |  |
| 14 | 0,119923 | 0,01 | -0,18905 |  |
| 15 | 0,100307 | 0,02 | 0,22852 |  |
| 16 | 0,100307 | 0,00 | -0,03895 |  |
| 17 | 0,204116 | 0,00 | -0,00672 |  |
| 18 | 0,204116 | 0,05 | -0,40092 |  |
| 19 | 0,132807 | 0,09 | 0,52896 |  |
| 20 | 0,199025 | 0,05 | 0,36639 |  |
| 21 | 0,037352 | 0,00 | -0,10980 |  |
| 22 | 0,037065 | 0,04 | 0,35857 |  |
| 23 | 0,039146 | 0,00 | -0,01010 |  |
| 24 | 0,039495 | 0,00 | 0,11649 |  |
| 25 | 0,037449 | 0,01 | -0,14701 |  |
| 26 | 0,037449 | 0,00 | 0,04720 |  |
| 27 | 0,037449 | 0,01 | 0,17855 |  |

R  Large residual

## Forward Selection of Terms

Achieved minimum BIC =  133,63

## Coded Coefficients

| Term | Coef | SE Coef | 95% CI | T-Value | P-Value | VIF |
| --- | --- | --- | --- | --- | --- | --- |
| Constant | 92,106 | 0,473 | (91,130; 93,082) | 194,80 | 0,000 |  |
| Lac | 7,057 | 0,975 | (5,044; 9,069) | 7,24 | 0,000 | 1,00 |
| HPMC\_Visc | -1,526 | 0,850 | (-3,280; 0,229) | -1,79 | 0,085 | 1,00 |

## Model Summary

| S | R-sq | R-sq(adj) | PRESS | R-sq(pred) | AICc | BIC |
| --- | --- | --- | --- | --- | --- | --- |
| 2,38814 | 69,85% | 67,34% | 176,994 | 61,02% | 130,27 | 133,63 |

## Analysis of Variance

| Source | DF | Seq SS | Contribution | Adj SS | Adj MS | F-Value | P-Value |
| --- | --- | --- | --- | --- | --- | --- | --- |
| Model | 2 | 317,143 | 69,85% | 317,143 | 158,572 | 27,80 | 0,000 |
| Linear | 2 | 317,143 | 69,85% | 317,143 | 158,572 | 27,80 | 0,000 |
| Lac | 1 | 298,769 | 65,81% | 298,769 | 298,769 | 52,39 | 0,000 |
| HPMC\_Visc | 1 | 18,375 | 4,05% | 18,375 | 18,375 | 3,22 | 0,085 |
| Error | 24 | 136,877 | 30,15% | 136,877 | 5,703 |  |  |
| Lack-of-Fit | 22 | 129,289 | 28,48% | 129,289 | 5,877 | 1,55 | 0,466 |
| Pure Error | 2 | 7,588 | 1,67% | 7,588 | 3,794 |  |  |
| Total | 26 | 454,020 | 100,00% |  |  |  |  |

## Regression Equation in Uncoded Units

|  |  |  |
| --- | --- | --- |
| F\_mean\_19h(1140min) | = | 83,29 + 28,23 Lac - 0,000392 HPMC\_Visc |

## Fits and Diagnostics for All Observations

| Obs | F\_mean\_19h(1140min) | Fit | SE Fit | 95% CI | Resid | Std Resid | Del Resid |
| --- | --- | --- | --- | --- | --- | --- | --- |
| 1 | 89,693 | 89,664 | 0,833 | (87,946; 91,382) | 0,029 | 0,01 | 0,01 |
| 2 | 97,816 | 96,720 | 0,833 | (95,002; 98,439) | 1,095 | 0,49 | 0,48 |
| 3 | 82,737 | 87,525 | 0,967 | (85,530; 89,521) | -4,789 | -2,19 | -2,40 |
| 4 | 91,735 | 94,582 | 0,967 | (92,586; 96,578) | -2,847 | -1,30 | -1,32 |
| 5 | 86,118 | 89,568 | 0,802 | (87,913; 91,224) | -3,450 | -1,53 | -1,58 |
| 6 | 96,013 | 96,625 | 0,802 | (94,970; 98,280) | -0,612 | -0,27 | -0,27 |
| 7 | 89,485 | 87,494 | 0,980 | (85,471; 89,516) | 1,991 | 0,91 | 0,91 |
| 8 | 93,940 | 94,550 | 0,980 | (92,528; 96,572) | -0,610 | -0,28 | -0,27 |
| 9 | 85,335 | 89,634 | 0,823 | (87,936; 91,332) | -4,299 | -1,92 | -2,04 |
| 10 | 98,817 | 96,690 | 0,823 | (94,992; 98,388) | 2,127 | 0,95 | 0,95 |
| 11 | 92,469 | 88,286 | 0,724 | (86,793; 89,780) | 4,182 | 1,84 | 1,94 |
| 12 | 95,751 | 95,343 | 0,724 | (93,850; 96,836) | 0,407 | 0,18 | 0,18 |
| 13 | 88,653 | 89,647 | 0,827 | (87,940; 91,354) | -0,995 | -0,44 | -0,44 |
| 14 | 94,926 | 96,704 | 0,827 | (94,997; 98,411) | -1,778 | -0,79 | -0,79 |
| 15 | 89,395 | 88,147 | 0,756 | (86,586; 89,708) | 1,249 | 0,55 | 0,54 |
| 16 | 95,487 | 95,203 | 0,756 | (93,642; 96,764) | 0,284 | 0,13 | 0,12 |
| 17 | 85,239 | 85,336 | 1,079 | (83,109; 87,562) | -0,097 | -0,05 | -0,04 |
| 18 | 97,278 | 99,449 | 1,079 | (97,222; 101,676) | -2,171 | -1,02 | -1,02 |
| 19 | 96,840 | 93,632 | 0,870 | (91,835; 95,428) | 3,208 | 1,44 | 1,48 |
| 20 | 91,993 | 90,580 | 1,065 | (88,381; 92,779) | 1,413 | 0,66 | 0,65 |
| 21 | 91,337 | 92,381 | 0,462 | (91,429; 93,334) | -1,045 | -0,45 | -0,44 |
| 22 | 95,783 | 92,282 | 0,460 | (91,333; 93,231) | 3,501 | 1,49 | 1,54 |
| 23 | 92,347 | 92,502 | 0,472 | (91,527; 93,477) | -0,155 | -0,07 | -0,06 |
| 24 | 94,127 | 92,518 | 0,475 | (91,538; 93,497) | 1,609 | 0,69 | 0,68 |
| 25 | 90,831 | 92,392 | 0,462 | (91,438; 93,346) | -1,561 | -0,67 | -0,66 |
| 26 | 93,460 | 92,392 | 0,462 | (91,438; 93,346) | 1,068 | 0,46 | 0,45 |
| 27 | 94,635 | 92,392 | 0,462 | (91,438; 93,346) | 2,243 | 0,96 | 0,96 |

| Obs | HI | Cook’s D | DFITS |  |
| --- | --- | --- | --- | --- |
| 1 | 0,121521 | 0,00 | 0,00477 |  |
| 2 | 0,121521 | 0,01 | 0,17903 |  |
| 3 | 0,163929 | 0,31 | -1,06304 | R |
| 4 | 0,163929 | 0,11 | -0,58631 |  |
| 5 | 0,112791 | 0,10 | -0,56375 |  |
| 6 | 0,112791 | 0,00 | -0,09504 |  |
| 7 | 0,168329 | 0,06 | 0,40983 |  |
| 8 | 0,168329 | 0,01 | -0,12355 |  |
| 9 | 0,118673 | 0,17 | -0,74849 |  |
| 10 | 0,118673 | 0,04 | 0,34731 |  |
| 11 | 0,091792 | 0,11 | 0,61696 |  |
| 12 | 0,091792 | 0,00 | 0,05576 |  |
| 13 | 0,119923 | 0,01 | -0,16108 |  |
| 14 | 0,119923 | 0,03 | -0,29059 |  |
| 15 | 0,100307 | 0,01 | 0,18131 |  |
| 16 | 0,100307 | 0,00 | 0,04100 |  |
| 17 | 0,204116 | 0,00 | -0,02246 |  |
| 18 | 0,204116 | 0,09 | -0,51635 |  |
| 19 | 0,132807 | 0,11 | 0,57824 |  |
| 20 | 0,199025 | 0,04 | 0,32554 |  |
| 21 | 0,037352 | 0,00 | -0,08632 |  |
| 22 | 0,037065 | 0,03 | 0,30128 |  |
| 23 | 0,039146 | 0,00 | -0,01308 |  |
| 24 | 0,039495 | 0,01 | 0,13784 |  |
| 25 | 0,037449 | 0,01 | -0,12986 |  |
| 26 | 0,037449 | 0,00 | 0,08841 |  |
| 27 | 0,037449 | 0,01 | 0,18849 |  |

R  Large residual

## Forward Selection of Terms

Achieved minimum BIC =  131,51

## Coded Coefficients

| Term | Coef | SE Coef | 95% CI | T-Value | P-Value | VIF |
| --- | --- | --- | --- | --- | --- | --- |
| Constant | 94,519 | 0,579 | (93,315; 95,723) | 163,22 | 0,000 |  |
| Lac | 6,285 | 0,834 | (4,550; 8,020) | 7,53 | 0,000 | 1,00 |
| HPMC\_Visc | -0,177 | 0,839 | (-1,922; 1,568) | -0,21 | 0,835 | 1,33 |
| HPMC\_PS | 2,61 | 1,14 | (0,23; 4,98) | 2,29 | 0,033 | 1,34 |
| Lac\*Lac | -2,67 | 1,58 | (-5,96; 0,62) | -1,69 | 0,106 | 1,00 |
| HPMC\_Visc\*HPMC\_PS | 5,51 | 2,26 | (0,81; 10,22) | 2,44 | 0,024 | 1,25 |

## Model Summary

| S | R-sq | R-sq(adj) | PRESS | R-sq(pred) | AICc | BIC |
| --- | --- | --- | --- | --- | --- | --- |
| 2,04403 | 77,18% | 71,75% | 141,275 | 63,26% | 128,34 | 131,51 |

## Analysis of Variance

| Source | DF | Seq SS | Contribution | Adj SS | Adj MS | F-Value | P-Value |
| --- | --- | --- | --- | --- | --- | --- | --- |
| Model | 5 | 296,788 | 77,18% | 296,788 | 59,358 | 14,21 | 0,000 |
| Linear | 3 | 260,110 | 67,64% | 266,794 | 88,931 | 21,29 | 0,000 |
| Lac | 1 | 237,011 | 61,64% | 237,011 | 237,011 | 56,73 | 0,000 |
| HPMC\_Visc | 1 | 14,015 | 3,64% | 0,185 | 0,185 | 0,04 | 0,835 |
| HPMC\_PS | 1 | 9,084 | 2,36% | 21,828 | 21,828 | 5,22 | 0,033 |
| Square | 1 | 11,891 | 3,09% | 11,884 | 11,884 | 2,84 | 0,106 |
| Lac\*Lac | 1 | 11,891 | 3,09% | 11,884 | 11,884 | 2,84 | 0,106 |
| 2-Way Interaction | 1 | 24,787 | 6,45% | 24,787 | 24,787 | 5,93 | 0,024 |
| HPMC\_Visc\*HPMC\_PS | 1 | 24,787 | 6,45% | 24,787 | 24,787 | 5,93 | 0,024 |
| Error | 21 | 87,739 | 22,82% | 87,739 | 4,178 |  |  |
| Lack-of-Fit | 19 | 80,249 | 20,87% | 80,249 | 4,224 | 1,13 | 0,572 |
| Pure Error | 2 | 7,490 | 1,95% | 7,490 | 3,745 |  |  |
| Total | 26 | 384,527 | 100,00% |  |  |  |  |

## Regression Equation in Uncoded Units

|  |  |  |
| --- | --- | --- |
| F\_mean\_20h(1200min) | = | 228,4 + 67,9 Lac - 0,01346 HPMC\_Visc - 2,25 HPMC\_PS - 42,7 Lac\*Lac + 0,000193 HPMC\_Visc\*HPMC\_PS |

## Fits and Diagnostics for All Observations

| Obs | F\_mean\_20h(1200min) | Fit | SE Fit | 95% CI | Resid | Std Resid | Del Resid |
| --- | --- | --- | --- | --- | --- | --- | --- |
| 1 | 91,09 | 91,16 | 0,87 | (89,35; 92,97) | -0,07 | -0,04 | -0,04 |
| 2 | 98,57 | 97,45 | 0,87 | (95,63; 99,26) | 1,12 | 0,61 | 0,60 |
| 3 | 84,65 | 86,88 | 1,16 | (84,47; 89,30) | -2,24 | -1,33 | -1,36 |
| 4 | 92,73 | 93,17 | 1,16 | (90,75; 95,58) | -0,44 | -0,26 | -0,26 |
| 5 | 87,77 | 90,99 | 0,78 | (89,37; 92,61) | -3,22 | -1,70 | -1,79 |
| 6 | 96,43 | 97,28 | 0,78 | (95,65; 98,90) | -0,85 | -0,45 | -0,44 |
| 7 | 91,14 | 88,04 | 0,93 | (86,11; 89,98) | 3,10 | 1,70 | 1,79 |
| 8 | 94,77 | 94,33 | 0,93 | (92,40; 96,26) | 0,44 | 0,24 | 0,23 |
| 9 | 86,97 | 90,33 | 0,90 | (88,47; 92,19) | -3,36 | -1,83 | -1,94 |
| 10 | 99,59 | 96,61 | 0,90 | (94,75; 98,48) | 2,97 | 1,62 | 1,69 |
| 11 | 94,14 | 91,45 | 0,84 | (89,71; 93,18) | 2,70 | 1,45 | 1,49 |
| 12 | 96,67 | 97,73 | 0,84 | (95,99; 99,47) | -1,06 | -0,57 | -0,56 |
| 13 | 90,12 | 90,18 | 1,01 | (88,08; 92,28) | -0,06 | -0,04 | -0,04 |
| 14 | 95,06 | 96,46 | 1,01 | (94,36; 98,57) | -1,40 | -0,79 | -0,78 |
| 15 | 90,72 | 91,77 | 0,97 | (89,76; 93,78) | -1,05 | -0,58 | -0,57 |
| 16 | 96,90 | 98,05 | 0,97 | (96,04; 100,06) | -1,15 | -0,64 | -0,63 |
| 17 | 87,07 | 85,54 | 1,55 | (82,32; 88,76) | 1,53 | 1,14 | 1,15 |
| 18 | 97,73 | 98,11 | 1,55 | (94,89; 101,33) | -0,39 | -0,29 | -0,28 |
| 19 | 97,92 | 94,43 | 0,88 | (92,60; 96,25) | 3,49 | 1,89 | 2,03 |
| 20 | 93,23 | 93,21 | 1,00 | (91,12; 95,29) | 0,02 | 0,01 | 0,01 |
| 21 | 92,80 | 94,38 | 0,56 | (93,21; 95,56) | -1,58 | -0,81 | -0,80 |
| 22 | 96,33 | 94,65 | 0,56 | (93,47; 95,82) | 1,69 | 0,86 | 0,85 |
| 23 | 93,32 | 93,39 | 1,24 | (90,81; 95,97) | -0,07 | -0,04 | -0,04 |
| 24 | 95,63 | 95,68 | 1,15 | (93,28; 98,09) | -0,06 | -0,03 | -0,03 |
| 25 | 92,29 | 94,50 | 0,55 | (93,35; 95,65) | -2,21 | -1,12 | -1,13 |
| 26 | 95,16 | 94,50 | 0,55 | (93,35; 95,65) | 0,67 | 0,34 | 0,33 |
| 27 | 95,97 | 94,50 | 0,55 | (93,35; 95,65) | 1,48 | 0,75 | 0,74 |

| Obs | HI | Cook’s D | DFITS |
| --- | --- | --- | --- |
| 1 | 0,181848 | 0,00 | -0,01774 |
| 2 | 0,181848 | 0,01 | 0,28232 |
| 3 | 0,323174 | 0,14 | -0,93725 |
| 4 | 0,323174 | 0,01 | -0,17624 |
| 5 | 0,145676 | 0,08 | -0,73889 |
| 6 | 0,145676 | 0,01 | -0,18221 |
| 7 | 0,206416 | 0,13 | 0,91288 |
| 8 | 0,206416 | 0,00 | 0,11964 |
| 9 | 0,191815 | 0,13 | -0,94686 |
| 10 | 0,191815 | 0,10 | 0,82199 |
| 11 | 0,167095 | 0,07 | 0,66609 |
| 12 | 0,167095 | 0,01 | -0,25037 |
| 13 | 0,244597 | 0,00 | -0,02005 |
| 14 | 0,244597 | 0,03 | -0,44487 |
| 15 | 0,223583 | 0,02 | -0,30680 |
| 16 | 0,223583 | 0,02 | -0,33801 |
| 17 | 0,573092 | 0,29 | 1,33447 |
| 18 | 0,573092 | 0,02 | -0,32752 |
| 19 | 0,184894 | 0,14 | 0,96518 |
| 20 | 0,239930 | 0,00 | 0,00708 |
| 21 | 0,076218 | 0,01 | -0,22957 |
| 22 | 0,076343 | 0,01 | 0,24503 |
| 23 | 0,368545 | 0,00 | -0,03226 |
| 24 | 0,318905 | 0,00 | -0,02288 |
| 25 | 0,073524 | 0,02 | -0,31807 |
| 26 | 0,073524 | 0,00 | 0,09322 |
| 27 | 0,073524 | 0,01 | 0,20903 |

## Forward Selection of Terms

Achieved minimum BIC =  132,05

## Coded Coefficients

| Term | Coef | SE Coef | 95% CI | T-Value | P-Value | VIF |
| --- | --- | --- | --- | --- | --- | --- |
| Constant | 94,513 | 0,465 | (93,556; 95,471) | 203,32 | 0,000 |  |
| Lac | 5,434 | 0,986 | (3,403; 7,465) | 5,51 | 0,000 | 1,00 |

## Model Summary

| S | R-sq | R-sq(adj) | PRESS | R-sq(pred) | AICc | BIC |
| --- | --- | --- | --- | --- | --- | --- |
| 2,41541 | 54,85% | 53,04% | 169,941 | 47,39% | 129,21 | 132,05 |

## Analysis of Variance

| Source | DF | Seq SS | Contribution | Adj SS | Adj MS | F-Value | P-Value |
| --- | --- | --- | --- | --- | --- | --- | --- |
| Model | 1 | 177,182 | 54,85% | 177,182 | 177,182 | 30,37 | 0,000 |
| Linear | 1 | 177,182 | 54,85% | 177,182 | 177,182 | 30,37 | 0,000 |
| Lac | 1 | 177,182 | 54,85% | 177,182 | 177,182 | 30,37 | 0,000 |
| Error | 25 | 145,856 | 45,15% | 145,856 | 5,834 |  |  |
| Lack-of-Fit | 23 | 139,009 | 43,03% | 139,009 | 6,044 | 1,77 | 0,425 |
| Pure Error | 2 | 6,846 | 2,12% | 6,846 | 3,423 |  |  |
| Total | 26 | 323,038 | 100,00% |  |  |  |  |

## Regression Equation in Uncoded Units

|  |  |  |
| --- | --- | --- |
| F\_mean\_21h(1260min) | = | 83,64 + 21,74 Lac |

## Fits and Diagnostics for All Observations

| Obs | F\_mean\_21h(1260min) | Fit | SE Fit | 95% CI | Resid | Std Resid | Del Resid |
| --- | --- | --- | --- | --- | --- | --- | --- |
| 1 | 92,775 | 91,796 | 0,678 | (90,401; 93,192) | 0,979 | 0,42 | 0,42 |
| 2 | 99,081 | 97,230 | 0,678 | (95,835; 98,626) | 1,851 | 0,80 | 0,79 |
| 3 | 86,403 | 91,796 | 0,678 | (90,401; 93,192) | -5,393 | -2,33 | -2,57 |
| 4 | 93,381 | 97,230 | 0,678 | (95,835; 98,626) | -3,850 | -1,66 | -1,72 |
| 5 | 89,109 | 91,796 | 0,678 | (90,401; 93,192) | -2,687 | -1,16 | -1,17 |
| 6 | 96,642 | 97,230 | 0,678 | (95,835; 98,626) | -0,588 | -0,25 | -0,25 |
| 7 | 92,642 | 91,796 | 0,678 | (90,401; 93,192) | 0,846 | 0,36 | 0,36 |
| 8 | 95,762 | 97,230 | 0,678 | (95,835; 98,626) | -1,468 | -0,63 | -0,63 |
| 9 | 88,388 | 91,796 | 0,678 | (90,401; 93,192) | -3,408 | -1,47 | -1,51 |
| 10 | 100,232 | 97,230 | 0,678 | (95,835; 98,626) | 3,002 | 1,29 | 1,31 |
| 11 | 95,691 | 91,796 | 0,678 | (90,401; 93,192) | 3,894 | 1,68 | 1,75 |
| 12 | 97,345 | 97,230 | 0,678 | (95,835; 98,626) | 0,115 | 0,05 | 0,05 |
| 13 | 91,329 | 91,796 | 0,678 | (90,401; 93,192) | -0,467 | -0,20 | -0,20 |
| 14 | 94,952 | 97,230 | 0,678 | (95,835; 98,626) | -2,278 | -0,98 | -0,98 |
| 15 | 91,802 | 91,796 | 0,678 | (90,401; 93,192) | 0,006 | 0,00 | 0,00 |
| 16 | 97,979 | 97,230 | 0,678 | (95,835; 98,626) | 0,749 | 0,32 | 0,32 |
| 17 | 88,698 | 89,079 | 1,090 | (86,834; 91,324) | -0,381 | -0,18 | -0,17 |
| 18 | 97,685 | 99,947 | 1,090 | (97,702; 102,193) | -2,262 | -1,05 | -1,05 |
| 19 | 98,771 | 94,513 | 0,465 | (93,556; 95,471) | 4,257 | 1,80 | 1,89 |
| 20 | 94,241 | 94,513 | 0,465 | (93,556; 95,471) | -0,272 | -0,11 | -0,11 |
| 21 | 94,086 | 94,513 | 0,465 | (93,556; 95,471) | -0,428 | -0,18 | -0,18 |
| 22 | 96,734 | 94,513 | 0,465 | (93,556; 95,471) | 2,221 | 0,94 | 0,93 |
| 23 | 94,121 | 94,513 | 0,465 | (93,556; 95,471) | -0,392 | -0,17 | -0,16 |
| 24 | 96,831 | 94,513 | 0,465 | (93,556; 95,471) | 2,318 | 0,98 | 0,98 |
| 25 | 93,601 | 94,513 | 0,465 | (93,556; 95,471) | -0,913 | -0,39 | -0,38 |
| 26 | 96,593 | 94,513 | 0,465 | (93,556; 95,471) | 2,080 | 0,88 | 0,87 |
| 27 | 96,982 | 94,513 | 0,465 | (93,556; 95,471) | 2,469 | 1,04 | 1,04 |

| Obs | HI | Cook’s D | DFITS |  |
| --- | --- | --- | --- | --- |
| 1 | 0,078704 | 0,01 | 0,121350 |  |
| 2 | 0,078704 | 0,03 | 0,231606 |  |
| 3 | 0,078704 | 0,23 | -0,752602 | R |
| 4 | 0,078704 | 0,12 | -0,504110 |  |
| 5 | 0,078704 | 0,06 | -0,341176 |  |
| 6 | 0,078704 | 0,00 | -0,072718 |  |
| 7 | 0,078704 | 0,01 | 0,104774 |  |
| 8 | 0,078704 | 0,02 | -0,182791 |  |
| 9 | 0,078704 | 0,09 | -0,440381 |  |
| 10 | 0,078704 | 0,07 | 0,383917 |  |
| 11 | 0,078704 | 0,12 | 0,510725 |  |
| 12 | 0,078704 | 0,00 | 0,014206 |  |
| 13 | 0,078704 | 0,00 | -0,057748 |  |
| 14 | 0,078704 | 0,04 | -0,287030 |  |
| 15 | 0,078704 | 0,00 | 0,000778 |  |
| 16 | 0,078704 | 0,00 | 0,092672 |  |
| 17 | 0,203704 | 0,00 | -0,087720 |  |
| 18 | 0,203704 | 0,14 | -0,532026 |  |
| 19 | 0,037037 | 0,06 | 0,369824 |  |
| 20 | 0,037037 | 0,00 | -0,022040 |  |
| 21 | 0,037037 | 0,00 | -0,034682 |  |
| 22 | 0,037037 | 0,02 | 0,183278 |  |
| 23 | 0,037037 | 0,00 | -0,031802 |  |
| 24 | 0,037037 | 0,02 | 0,191585 |  |
| 25 | 0,037037 | 0,00 | -0,074202 |  |
| 26 | 0,037037 | 0,01 | 0,171276 |  |
| 27 | 0,037037 | 0,02 | 0,204612 |  |

R  Large residual

## Forward Selection of Terms

Achieved minimum BIC =  131,51

## Coded Coefficients

| Term | Coef | SE Coef | 95% CI | T-Value | P-Value | VIF |
| --- | --- | --- | --- | --- | --- | --- |
| Constant | 95,307 | 0,460 | (94,359; 96,255) | 207,10 | 0,000 |  |
| Lac | 4,528 | 0,976 | (2,518; 6,539) | 4,64 | 0,000 | 1,00 |

## Model Summary

| S | R-sq | R-sq(adj) | PRESS | R-sq(pred) | AICc | BIC |
| --- | --- | --- | --- | --- | --- | --- |
| 2,39130 | 46,25% | 44,10% | 166,772 | 37,30% | 128,67 | 131,51 |

## Analysis of Variance

| Source | DF | Seq SS | Contribution | Adj SS | Adj MS | F-Value | P-Value |
| --- | --- | --- | --- | --- | --- | --- | --- |
| Model | 1 | 123,030 | 46,25% | 123,030 | 123,030 | 21,52 | 0,000 |
| Linear | 1 | 123,030 | 46,25% | 123,030 | 123,030 | 21,52 | 0,000 |
| Lac | 1 | 123,030 | 46,25% | 123,030 | 123,030 | 21,52 | 0,000 |
| Error | 25 | 142,958 | 53,75% | 142,958 | 5,718 |  |  |
| Lack-of-Fit | 23 | 136,846 | 51,45% | 136,846 | 5,950 | 1,95 | 0,395 |
| Pure Error | 2 | 6,112 | 2,30% | 6,112 | 3,056 |  |  |
| Total | 26 | 265,988 | 100,00% |  |  |  |  |

## Regression Equation in Uncoded Units

|  |  |  |
| --- | --- | --- |
| F\_mean\_22h(1320min) | = | 86,25 + 18,11 Lac |

## Fits and Diagnostics for All Observations

| Obs | F\_mean\_22h(1320min) | Fit | SE Fit | 95% CI | Resid | Std Resid | Del Resid |
| --- | --- | --- | --- | --- | --- | --- | --- |
| 1 | 94,056 | 93,043 | 0,671 | (91,661; 94,425) | 1,013 | 0,44 | 0,43 |
| 2 | 99,131 | 97,571 | 0,671 | (96,189; 98,953) | 1,560 | 0,68 | 0,67 |
| 3 | 87,985 | 93,043 | 0,671 | (91,661; 94,425) | -5,058 | -2,20 | -2,41 |
| 4 | 93,861 | 97,571 | 0,671 | (96,189; 98,953) | -3,710 | -1,62 | -1,67 |
| 5 | 90,343 | 93,043 | 0,671 | (91,661; 94,425) | -2,700 | -1,18 | -1,19 |
| 6 | 96,858 | 97,571 | 0,671 | (96,189; 98,953) | -0,713 | -0,31 | -0,31 |
| 7 | 93,920 | 93,043 | 0,671 | (91,661; 94,425) | 0,877 | 0,38 | 0,38 |
| 8 | 96,339 | 97,571 | 0,671 | (96,189; 98,953) | -1,232 | -0,54 | -0,53 |
| 9 | 89,629 | 93,043 | 0,671 | (91,661; 94,425) | -3,413 | -1,49 | -1,53 |
| 10 | 100,470 | 97,571 | 0,671 | (96,189; 98,953) | 2,899 | 1,26 | 1,28 |
| 11 | 97,015 | 93,043 | 0,671 | (91,661; 94,425) | 3,972 | 1,73 | 1,81 |
| 12 | 97,606 | 97,571 | 0,671 | (96,189; 98,953) | 0,035 | 0,02 | 0,01 |
| 13 | 92,370 | 93,043 | 0,671 | (91,661; 94,425) | -0,673 | -0,29 | -0,29 |
| 14 | 94,719 | 97,571 | 0,671 | (96,189; 98,953) | -2,852 | -1,24 | -1,26 |
| 15 | 92,903 | 93,043 | 0,671 | (91,661; 94,425) | -0,140 | -0,06 | -0,06 |
| 16 | 98,692 | 97,571 | 0,671 | (96,189; 98,953) | 1,121 | 0,49 | 0,48 |
| 17 | 90,128 | 90,779 | 1,079 | (88,556; 93,002) | -0,651 | -0,31 | -0,30 |
| 18 | 97,569 | 99,835 | 1,079 | (97,612; 102,058) | -2,266 | -1,06 | -1,06 |
| 19 | 99,382 | 95,307 | 0,460 | (94,359; 96,255) | 4,075 | 1,74 | 1,81 |
| 20 | 95,127 | 95,307 | 0,460 | (94,359; 96,255) | -0,181 | -0,08 | -0,08 |
| 21 | 95,199 | 95,307 | 0,460 | (94,359; 96,255) | -0,108 | -0,05 | -0,04 |
| 22 | 96,942 | 95,307 | 0,460 | (94,359; 96,255) | 1,635 | 0,70 | 0,69 |
| 23 | 94,799 | 95,307 | 0,460 | (94,359; 96,255) | -0,508 | -0,22 | -0,21 |
| 24 | 97,837 | 95,307 | 0,460 | (94,359; 96,255) | 2,530 | 1,08 | 1,08 |
| 25 | 94,785 | 95,307 | 0,460 | (94,359; 96,255) | -0,522 | -0,22 | -0,22 |
| 26 | 97,801 | 95,307 | 0,460 | (94,359; 96,255) | 2,494 | 1,06 | 1,07 |
| 27 | 97,824 | 95,307 | 0,460 | (94,359; 96,255) | 2,517 | 1,07 | 1,08 |

| Obs | HI | Cook’s D | DFITS |  |
| --- | --- | --- | --- | --- |
| 1 | 0,078704 | 0,01 | 0,126834 |  |
| 2 | 0,078704 | 0,02 | 0,196478 |  |
| 3 | 0,078704 | 0,21 | -0,703074 | R |
| 4 | 0,078704 | 0,11 | -0,489139 |  |
| 5 | 0,078704 | 0,06 | -0,346532 |  |
| 6 | 0,078704 | 0,00 | -0,089147 |  |
| 7 | 0,078704 | 0,01 | 0,109742 |  |
| 8 | 0,078704 | 0,01 | -0,154620 |  |
| 9 | 0,078704 | 0,09 | -0,446079 |  |
| 10 | 0,078704 | 0,07 | 0,373771 |  |
| 11 | 0,078704 | 0,13 | 0,528289 |  |
| 12 | 0,078704 | 0,00 | 0,004309 |  |
| 13 | 0,078704 | 0,00 | -0,084145 |  |
| 14 | 0,078704 | 0,07 | -0,367352 |  |
| 15 | 0,078704 | 0,00 | -0,017485 |  |
| 16 | 0,078704 | 0,01 | 0,140496 |  |
| 17 | 0,203704 | 0,01 | -0,151485 |  |
| 18 | 0,203704 | 0,14 | -0,538476 |  |
| 19 | 0,037037 | 0,06 | 0,355866 |  |
| 20 | 0,037037 | 0,00 | -0,014785 |  |
| 21 | 0,037037 | 0,00 | -0,008818 |  |
| 22 | 0,037037 | 0,01 | 0,135187 |  |
| 23 | 0,037037 | 0,00 | -0,041636 |  |
| 24 | 0,037037 | 0,02 | 0,212128 |  |
| 25 | 0,037037 | 0,00 | -0,042801 |  |
| 26 | 0,037037 | 0,02 | 0,208974 |  |
| 27 | 0,037037 | 0,02 | 0,211061 |  |

R  Large residual

## Forward Selection of Terms

Achieved minimum BIC =  130,66

## Coded Coefficients

| Term | Coef | SE Coef | 95% CI | T-Value | P-Value | VIF |
| --- | --- | --- | --- | --- | --- | --- |
| Constant | 96,641 | 0,584 | (95,436; 97,845) | 165,59 | 0,000 |  |
| Lac | 3,662 | 0,923 | (1,757; 5,566) | 3,97 | 0,001 | 1,00 |
| Lac\*Lac | -3,24 | 1,75 | (-6,86; 0,37) | -1,85 | 0,076 | 1,00 |

## Model Summary

| S | R-sq | R-sq(adj) | PRESS | R-sq(pred) | AICc | BIC |
| --- | --- | --- | --- | --- | --- | --- |
| 2,26028 | 44,42% | 39,79% | 155,625 | 29,46% | 127,30 | 130,66 |

## Analysis of Variance

| Source | DF | Seq SS | Contribution | Adj SS | Adj MS | F-Value | P-Value |
| --- | --- | --- | --- | --- | --- | --- | --- |
| Model | 2 | 97,991 | 44,42% | 97,991 | 48,996 | 9,59 | 0,001 |
| Linear | 1 | 80,459 | 36,47% | 80,459 | 80,459 | 15,75 | 0,001 |
| Lac | 1 | 80,459 | 36,47% | 80,459 | 80,459 | 15,75 | 0,001 |
| Square | 1 | 17,533 | 7,95% | 17,533 | 17,533 | 3,43 | 0,076 |
| Lac\*Lac | 1 | 17,533 | 7,95% | 17,533 | 17,533 | 3,43 | 0,076 |
| Error | 24 | 122,612 | 55,58% | 122,612 | 5,109 |  |  |
| Lack-of-Fit | 22 | 117,419 | 53,23% | 117,419 | 5,337 | 2,06 | 0,379 |
| Pure Error | 2 | 5,193 | 2,35% | 5,193 | 2,596 |  |  |
| Total | 26 | 220,604 | 100,00% |  |  |  |  |

## Regression Equation in Uncoded Units

|  |  |  |
| --- | --- | --- |
| F\_mean\_23h(1380min) | = | 76,34 + 66,5 Lac - 51,9 Lac\*Lac |

## Fits and Diagnostics for All Observations

| Obs | F\_mean\_23h(1380min) | Fit | SE Fit | 95% CI | Resid | Std Resid | Del Resid |
| --- | --- | --- | --- | --- | --- | --- | --- |
| 1 | 94,91 | 94,00 | 0,64 | (92,69; 95,31) | 0,91 | 0,42 | 0,41 |
| 2 | 99,06 | 97,66 | 0,64 | (96,35; 98,97) | 1,40 | 0,64 | 0,64 |
| 3 | 89,49 | 94,00 | 0,64 | (92,69; 95,31) | -4,51 | -2,08 | -2,25 |
| 4 | 94,10 | 97,66 | 0,64 | (96,35; 98,97) | -3,56 | -1,64 | -1,71 |
| 5 | 91,57 | 94,00 | 0,64 | (92,69; 95,31) | -2,42 | -1,12 | -1,12 |
| 6 | 96,95 | 97,66 | 0,64 | (96,35; 98,97) | -0,71 | -0,33 | -0,32 |
| 7 | 94,89 | 94,00 | 0,64 | (92,69; 95,31) | 0,89 | 0,41 | 0,40 |
| 8 | 96,68 | 97,66 | 0,64 | (96,35; 98,97) | -0,98 | -0,45 | -0,44 |
| 9 | 90,50 | 94,00 | 0,64 | (92,69; 95,31) | -3,50 | -1,61 | -1,67 |
| 10 | 100,49 | 97,66 | 0,64 | (96,35; 98,97) | 2,83 | 1,30 | 1,32 |
| 11 | 98,17 | 94,00 | 0,64 | (92,69; 95,31) | 4,17 | 1,92 | 2,04 |
| 12 | 97,60 | 97,66 | 0,64 | (96,35; 98,97) | -0,06 | -0,03 | -0,03 |
| 13 | 93,21 | 94,00 | 0,64 | (92,69; 95,31) | -0,79 | -0,36 | -0,36 |
| 14 | 94,53 | 97,66 | 0,64 | (96,35; 98,97) | -3,13 | -1,44 | -1,48 |
| 15 | 93,93 | 94,00 | 0,64 | (92,69; 95,31) | -0,07 | -0,03 | -0,03 |
| 16 | 99,12 | 97,66 | 0,64 | (96,35; 98,97) | 1,45 | 0,67 | 0,66 |
| 17 | 91,38 | 89,74 | 1,70 | (86,22; 93,25) | 1,65 | 1,11 | 1,11 |
| 18 | 97,43 | 97,06 | 1,70 | (93,55; 100,57) | 0,37 | 0,25 | 0,24 |
| 19 | 99,71 | 96,64 | 0,58 | (95,44; 97,85) | 3,06 | 1,40 | 1,43 |
| 20 | 95,79 | 96,64 | 0,58 | (95,44; 97,85) | -0,85 | -0,39 | -0,38 |
| 21 | 96,19 | 96,64 | 0,58 | (95,44; 97,85) | -0,45 | -0,21 | -0,20 |
| 22 | 97,12 | 96,64 | 0,58 | (95,44; 97,85) | 0,48 | 0,22 | 0,22 |
| 23 | 95,16 | 96,64 | 0,58 | (95,44; 97,85) | -1,48 | -0,68 | -0,67 |
| 24 | 98,74 | 96,64 | 0,58 | (95,44; 97,85) | 2,10 | 0,96 | 0,96 |
| 25 | 95,85 | 96,64 | 0,58 | (95,44; 97,85) | -0,80 | -0,36 | -0,36 |
| 26 | 98,74 | 96,64 | 0,58 | (95,44; 97,85) | 2,10 | 0,96 | 0,96 |
| 27 | 98,52 | 96,64 | 0,58 | (95,44; 97,85) | 1,88 | 0,86 | 0,86 |

| Obs | HI | Cook’s D | DFITS |  |  |
| --- | --- | --- | --- | --- | --- |
| 1 | 0,079167 | 0,01 | 0,12124 |  |  |
| 2 | 0,079167 | 0,01 | 0,18671 |  |  |
| 3 | 0,079167 | 0,12 | -0,65911 | R |  |
| 4 | 0,079167 | 0,08 | -0,50024 |  |  |
| 5 | 0,079167 | 0,04 | -0,32954 |  |  |
| 6 | 0,079167 | 0,00 | -0,09416 |  |  |
| 7 | 0,079167 | 0,00 | 0,11840 |  |  |
| 8 | 0,079167 | 0,01 | -0,12976 |  |  |
| 9 | 0,079167 | 0,07 | -0,48983 |  |  |
| 10 | 0,079167 | 0,05 | 0,38816 |  |  |
| 11 | 0,079167 | 0,11 | 0,59946 |  |  |
| 12 | 0,079167 | 0,00 | -0,00821 |  |  |
| 13 | 0,079167 | 0,00 | -0,10437 |  |  |
| 14 | 0,079167 | 0,06 | -0,43313 |  |  |
| 15 | 0,079167 | 0,00 | -0,00922 |  |  |
| 16 | 0,079167 | 0,01 | 0,19432 |  |  |
| 17 | 0,566667 | 0,53 | 1,27311 |  | X |
| 18 | 0,566667 | 0,03 | 0,27844 |  | X |
| 19 | 0,066667 | 0,05 | 0,38320 |  |  |
| 20 | 0,066667 | 0,00 | -0,10220 |  |  |
| 21 | 0,066667 | 0,00 | -0,05439 |  |  |
| 22 | 0,066667 | 0,00 | 0,05801 |  |  |
| 23 | 0,066667 | 0,01 | -0,17854 |  |  |
| 24 | 0,066667 | 0,02 | 0,25678 |  |  |
| 25 | 0,066667 | 0,00 | -0,09554 |  |  |
| 26 | 0,066667 | 0,02 | 0,25613 |  |  |
| 27 | 0,066667 | 0,02 | 0,22919 |  |  |

R  Large residual  
X  Unusual X

## Forward Selection of Terms

Achieved minimum BIC =  130,49

## Coded Coefficients

| Term | Coef | SE Coef | 95% CI | T-Value | P-Value | VIF |
| --- | --- | --- | --- | --- | --- | --- |
| Constant | 97,160 | 0,582 | (95,960; 98,361) | 167,02 | 0,000 |  |
| Lac | 2,878 | 0,920 | (0,980; 4,777) | 3,13 | 0,005 | 1,00 |
| Lac\*Lac | -3,37 | 1,75 | (-6,97; 0,24) | -1,93 | 0,066 | 1,00 |

## Model Summary

| S | R-sq | R-sq(adj) | PRESS | R-sq(pred) | AICc | BIC |
| --- | --- | --- | --- | --- | --- | --- |
| 2,25308 | 36,02% | 30,69% | 154,123 | 19,07% | 127,12 | 130,49 |

## Analysis of Variance

| Source | DF | Seq SS | Contribution | Adj SS | Adj MS | F-Value | P-Value |
| --- | --- | --- | --- | --- | --- | --- | --- |
| Model | 2 | 68,599 | 36,02% | 68,599 | 34,300 | 6,76 | 0,005 |
| Linear | 1 | 49,714 | 26,11% | 49,714 | 49,714 | 9,79 | 0,005 |
| Lac | 1 | 49,714 | 26,11% | 49,714 | 49,714 | 9,79 | 0,005 |
| Square | 1 | 18,886 | 9,92% | 18,886 | 18,886 | 3,72 | 0,066 |
| Lac\*Lac | 1 | 18,886 | 9,92% | 18,886 | 18,886 | 3,72 | 0,066 |
| Error | 24 | 121,833 | 63,98% | 121,833 | 5,076 |  |  |
| Lack-of-Fit | 22 | 117,969 | 61,95% | 117,969 | 5,362 | 2,78 | 0,298 |
| Pure Error | 2 | 3,864 | 2,03% | 3,864 | 1,932 |  |  |
| Total | 26 | 190,433 | 100,00% |  |  |  |  |

## Regression Equation in Uncoded Units

|  |  |  |
| --- | --- | --- |
| F\_mean\_24h(1440min) | = | 77,94 + 65,4 Lac - 53,9 Lac\*Lac |

## Fits and Diagnostics for All Observations

| Obs | F\_mean\_24h(1440min) | Fit | SE Fit | 95% CI | Resid | Std Resid | Del Resid |
| --- | --- | --- | --- | --- | --- | --- | --- |
| 1 | 95,53 | 94,88 | 0,63 | (93,57; 96,19) | 0,65 | 0,30 | 0,30 |
| 2 | 98,99 | 97,76 | 0,63 | (96,45; 99,07) | 1,23 | 0,57 | 0,56 |
| 3 | 90,80 | 94,88 | 0,63 | (93,57; 96,19) | -4,08 | -1,89 | -2,00 |
| 4 | 94,17 | 97,76 | 0,63 | (96,45; 99,07) | -3,59 | -1,66 | -1,73 |
| 5 | 92,51 | 94,88 | 0,63 | (93,57; 96,19) | -2,37 | -1,10 | -1,10 |
| 6 | 97,09 | 97,76 | 0,63 | (96,45; 99,07) | -0,67 | -0,31 | -0,30 |
| 7 | 95,86 | 94,88 | 0,63 | (93,57; 96,19) | 0,98 | 0,45 | 0,45 |
| 8 | 96,74 | 97,76 | 0,63 | (96,45; 99,07) | -1,01 | -0,47 | -0,46 |
| 9 | 91,28 | 94,88 | 0,63 | (93,57; 96,19) | -3,60 | -1,67 | -1,73 |
| 10 | 100,51 | 97,76 | 0,63 | (96,45; 99,07) | 2,75 | 1,27 | 1,29 |
| 11 | 99,12 | 94,88 | 0,63 | (93,57; 96,19) | 4,24 | 1,96 | 2,10 |
| 12 | 97,59 | 97,76 | 0,63 | (96,45; 99,07) | -0,17 | -0,08 | -0,08 |
| 13 | 93,88 | 94,88 | 0,63 | (93,57; 96,19) | -1,00 | -0,46 | -0,45 |
| 14 | 94,32 | 97,76 | 0,63 | (96,45; 99,07) | -3,43 | -1,59 | -1,64 |
| 15 | 94,86 | 94,88 | 0,63 | (93,57; 96,19) | -0,02 | -0,01 | -0,01 |
| 16 | 99,27 | 97,76 | 0,63 | (96,45; 99,07) | 1,52 | 0,70 | 0,69 |
| 17 | 92,44 | 90,92 | 1,70 | (87,42; 94,42) | 1,53 | 1,03 | 1,03 |
| 18 | 97,29 | 96,67 | 1,70 | (93,17; 100,17) | 0,62 | 0,42 | 0,41 |
| 19 | 99,95 | 97,16 | 0,58 | (95,96; 98,36) | 2,79 | 1,28 | 1,30 |
| 20 | 96,30 | 97,16 | 0,58 | (95,96; 98,36) | -0,86 | -0,39 | -0,39 |
| 21 | 97,03 | 97,16 | 0,58 | (95,96; 98,36) | -0,13 | -0,06 | -0,06 |
| 22 | 97,28 | 97,16 | 0,58 | (95,96; 98,36) | 0,12 | 0,06 | 0,05 |
| 23 | 95,41 | 97,16 | 0,58 | (95,96; 98,36) | -1,75 | -0,81 | -0,80 |
| 24 | 99,43 | 97,16 | 0,58 | (95,96; 98,36) | 2,27 | 1,04 | 1,04 |
| 25 | 96,90 | 97,16 | 0,58 | (95,96; 98,36) | -0,26 | -0,12 | -0,12 |
| 26 | 99,48 | 97,16 | 0,58 | (95,96; 98,36) | 2,32 | 1,06 | 1,07 |
| 27 | 99,10 | 97,16 | 0,58 | (95,96; 98,36) | 1,94 | 0,89 | 0,89 |

| Obs | HI | Cook’s D | DFITS |  |
| --- | --- | --- | --- | --- |
| 1 | 0,079167 | 0,00 | 0,08663 |  |
| 2 | 0,079167 | 0,01 | 0,16428 |  |
| 3 | 0,079167 | 0,10 | -0,58751 |  |
| 4 | 0,079167 | 0,08 | -0,50648 |  |
| 5 | 0,079167 | 0,03 | -0,32262 |  |
| 6 | 0,079167 | 0,00 | -0,08858 |  |
| 7 | 0,079167 | 0,01 | 0,13065 |  |
| 8 | 0,079167 | 0,01 | -0,13535 |  |
| 9 | 0,079167 | 0,08 | -0,50832 |  |
| 10 | 0,079167 | 0,05 | 0,37778 |  |
| 11 | 0,079167 | 0,11 | 0,61442 |  |
| 12 | 0,079167 | 0,00 | -0,02211 |  |
| 13 | 0,079167 | 0,01 | -0,13286 |  |
| 14 | 0,079167 | 0,07 | -0,48180 |  |
| 15 | 0,079167 | 0,00 | -0,00313 |  |
| 16 | 0,079167 | 0,01 | 0,20334 |  |
| 17 | 0,566667 | 0,46 | 1,17978 | X |
| 18 | 0,566667 | 0,08 | 0,46675 | X |
| 19 | 0,066667 | 0,04 | 0,34795 |  |
| 20 | 0,066667 | 0,00 | -0,10353 |  |
| 21 | 0,066667 | 0,00 | -0,01585 |  |
| 22 | 0,066667 | 0,00 | 0,01460 |  |
| 23 | 0,066667 | 0,02 | -0,21382 |  |
| 24 | 0,066667 | 0,03 | 0,27876 |  |
| 25 | 0,066667 | 0,00 | -0,03102 |  |
| 26 | 0,066667 | 0,03 | 0,28518 |  |
| 27 | 0,066667 | 0,02 | 0,23695 |  |

X  Unusual X
