# Supplementary material for: Influence of Selected Hypromellose Functionality-Related Characteristics and Soluble/Insoluble Filler Ratio on Carvedilol Release from Matrix Tablets
Source: Pharmaceutics. 2025 Oct 21;17(10):1358. doi: 10.3390/pharmaceutics17101358 (PMC12566823; doi:10.3390/pharmaceutics17101358)
Supplement: Supplementary file 1 [file pharmaceutics-17-01358-s001.zip › Report_Mean Release Analysis_RSM(CCD)_Stepwise, Forward Selection.htm]

# Mean Release Analysis, Response Surface Design (Central Composite Design), Stepwise Regression - Forward Selection

## Forward Selection of Terms

α to enter = 0,25

## Coded Coefficients

| Term | Coef | SE Coef | 95% CI | T-Value | P-Value | VIF |
| --- | --- | --- | --- | --- | --- | --- |
| Constant | 10,118 | 0,294 | (9,511; 10,726) | 34,37 | 0,000 |  |
| Lac | 2,621 | 0,624 | (1,333; 3,910) | 4,20 | 0,000 | 1,00 |
| HPMC\_PS | 1,115 | 0,736 | (-0,404; 2,635) | 1,51 | 0,143 | 1,00 |

## Model Summary

| S | R-sq | R-sq(adj) | PRESS | R-sq(pred) | AICc | BIC |
| --- | --- | --- | --- | --- | --- | --- |
| 1,52958 | 45,35% | 40,80% | 66,8980 | 34,89% | 106,21 | 109,58 |

## Analysis of Variance

| Source | DF | Seq SS | Contribution | Adj SS | Adj MS | F-Value | P-Value |
| --- | --- | --- | --- | --- | --- | --- | --- |
| Model | 2 | 46,598 | 45,35% | 46,598 | 23,299 | 9,96 | 0,001 |
| Linear | 2 | 46,598 | 45,35% | 46,598 | 23,299 | 9,96 | 0,001 |
| Lac | 1 | 41,230 | 40,13% | 41,230 | 41,230 | 17,62 | 0,000 |
| HPMC\_PS | 1 | 5,368 | 5,22% | 5,368 | 5,368 | 2,29 | 0,143 |
| Error | 24 | 56,151 | 54,65% | 56,151 | 2,340 |  |  |
| Lack-of-Fit | 22 | 42,122 | 40,99% | 42,122 | 1,915 | 0,27 | 0,958 |
| Pure Error | 2 | 14,029 | 13,65% | 14,029 | 7,015 |  |  |
| Total | 26 | 102,749 | 100,00% |  |  |  |  |

## Regression Equation in Uncoded Units

|  |  |  |
| --- | --- | --- |
| F\_mean\_0.17h(10min) | = | -5,69 + 10,49 Lac + 0,152 HPMC\_PS |

## Fits and Diagnostics for All Observations

| Obs | F\_mean\_0.17h(10min) | Fit | SE Fit | 95% CI | Resid | Std Resid | Del Resid |
| --- | --- | --- | --- | --- | --- | --- | --- |
| 1 | 9,095 | 8,532 | 0,464 | (7,574; 9,489) | 0,563 | 0,39 | 0,38 |
| 2 | 11,821 | 11,153 | 0,464 | (10,196; 12,110) | 0,668 | 0,46 | 0,45 |
| 3 | 7,323 | 8,162 | 0,600 | (6,923; 9,401) | -0,839 | -0,60 | -0,59 |
| 4 | 8,147 | 10,784 | 0,600 | (9,545; 12,023) | -2,636 | -1,87 | -1,99 |
| 5 | 7,099 | 8,616 | 0,446 | (7,696; 9,536) | -1,517 | -1,04 | -1,04 |
| 6 | 12,823 | 11,237 | 0,446 | (10,317; 12,157) | 1,586 | 1,08 | 1,09 |
| 7 | 7,484 | 8,373 | 0,513 | (7,315; 9,431) | -0,889 | -0,62 | -0,61 |
| 8 | 9,846 | 10,994 | 0,513 | (9,936; 12,053) | -1,148 | -0,80 | -0,79 |
| 9 | 8,623 | 9,270 | 0,530 | (8,176; 10,364) | -0,647 | -0,45 | -0,44 |
| 10 | 11,322 | 11,891 | 0,530 | (10,797; 12,986) | -0,570 | -0,40 | -0,39 |
| 11 | 9,398 | 9,042 | 0,458 | (8,096; 9,989) | 0,355 | 0,24 | 0,24 |
| 12 | 12,522 | 11,664 | 0,458 | (10,718; 12,610) | 0,858 | 0,59 | 0,58 |
| 13 | 7,874 | 9,387 | 0,579 | (8,192; 10,582) | -1,513 | -1,07 | -1,07 |
| 14 | 12,420 | 12,009 | 0,579 | (10,814; 13,204) | 0,412 | 0,29 | 0,29 |
| 15 | 10,497 | 9,105 | 0,474 | (8,126; 10,084) | 1,391 | 0,96 | 0,96 |
| 16 | 10,196 | 11,726 | 0,474 | (10,747; 12,706) | -1,530 | -1,05 | -1,05 |
| 17 | 8,246 | 7,458 | 0,691 | (6,033; 8,884) | 0,788 | 0,58 | 0,57 |
| 18 | 13,122 | 12,701 | 0,691 | (11,276; 14,126) | 0,421 | 0,31 | 0,30 |
| 19 | 9,647 | 10,222 | 0,304 | (9,595; 10,849) | -0,575 | -0,38 | -0,38 |
| 20 | 13,095 | 9,962 | 0,310 | (9,322; 10,602) | 3,133 | 2,09 | 2,26 |
| 21 | 10,472 | 10,001 | 0,303 | (9,376; 10,626) | 0,471 | 0,31 | 0,31 |
| 22 | 12,671 | 10,179 | 0,298 | (9,564; 10,794) | 2,492 | 1,66 | 1,73 |
| 23 | 9,297 | 9,003 | 0,787 | (7,378; 10,628) | 0,294 | 0,22 | 0,22 |
| 24 | 10,323 | 11,233 | 0,798 | (9,585; 12,881) | -0,910 | -0,70 | -0,69 |
| 25 | 8,148 | 10,080 | 0,295 | (9,471; 10,688) | -1,932 | -1,29 | -1,31 |
| 26 | 8,524 | 10,080 | 0,295 | (9,471; 10,688) | -1,556 | -1,04 | -1,04 |
| 27 | 12,912 | 10,080 | 0,295 | (9,471; 10,688) | 2,832 | 1,89 | 2,00 |

| Obs | HI | Cook’s D | DFITS |  |
| --- | --- | --- | --- | --- |
| 1 | 0,091956 | 0,01 | 0,120814 |  |
| 2 | 0,091956 | 0,01 | 0,143450 |  |
| 3 | 0,154060 | 0,02 | -0,251067 |  |
| 4 | 0,154060 | 0,21 | -0,847342 |  |
| 5 | 0,084910 | 0,03 | -0,316370 |  |
| 6 | 0,084910 | 0,04 | 0,331354 |  |
| 7 | 0,112397 | 0,02 | -0,216644 |  |
| 8 | 0,112397 | 0,03 | -0,281380 |  |
| 9 | 0,120141 | 0,01 | -0,163868 |  |
| 10 | 0,120141 | 0,01 | -0,144079 |  |
| 11 | 0,089807 | 0,00 | 0,074931 |  |
| 12 | 0,089807 | 0,01 | 0,182114 |  |
| 13 | 0,143301 | 0,06 | -0,438562 |  |
| 14 | 0,143301 | 0,00 | 0,116652 |  |
| 15 | 0,096233 | 0,03 | 0,311685 |  |
| 16 | 0,096233 | 0,04 | -0,344189 |  |
| 17 | 0,203866 | 0,03 | 0,288158 |  |
| 18 | 0,203866 | 0,01 | 0,153096 |  |
| 19 | 0,039415 | 0,00 | -0,076359 |  |
| 20 | 0,041065 | 0,06 | 0,468545 | R |
| 21 | 0,039207 | 0,00 | 0,062208 |  |
| 22 | 0,037944 | 0,04 | 0,343263 |  |
| 23 | 0,264924 | 0,01 | 0,131707 |  |
| 24 | 0,272505 | 0,06 | -0,422349 |  |
| 25 | 0,037200 | 0,02 | -0,256683 |  |
| 26 | 0,037200 | 0,01 | -0,204098 |  |
| 27 | 0,037200 | 0,05 | 0,393446 |  |

R  Large residual

## Forward Selection of Terms

α to enter = 0,25

## Coded Coefficients

| Term | Coef | SE Coef | 95% CI | T-Value | P-Value | VIF |
| --- | --- | --- | --- | --- | --- | --- |
| Constant | 13,846 | 0,354 | (13,114; 14,577) | 39,09 | 0,000 |  |
| Lac | 3,323 | 0,751 | (1,773; 4,874) | 4,42 | 0,000 | 1,00 |
| HPMC\_PS | 1,686 | 0,886 | (-0,142; 3,514) | 1,90 | 0,069 | 1,00 |

## Model Summary

| S | R-sq | R-sq(adj) | PRESS | R-sq(pred) | AICc | BIC |
| --- | --- | --- | --- | --- | --- | --- |
| 1,84024 | 49,14% | 44,90% | 96,3524 | 39,71% | 116,20 | 119,56 |

## Analysis of Variance

| Source | DF | Seq SS | Contribution | Adj SS | Adj MS | F-Value | P-Value |
| --- | --- | --- | --- | --- | --- | --- | --- |
| Model | 2 | 78,54 | 49,14% | 78,54 | 39,268 | 11,60 | 0,000 |
| Linear | 2 | 78,54 | 49,14% | 78,54 | 39,268 | 11,60 | 0,000 |
| Lac | 1 | 66,27 | 41,47% | 66,27 | 66,271 | 19,57 | 0,000 |
| HPMC\_PS | 1 | 12,26 | 7,67% | 12,26 | 12,265 | 3,62 | 0,069 |
| Error | 24 | 81,28 | 50,86% | 81,28 | 3,386 |  |  |
| Lack-of-Fit | 22 | 63,65 | 39,83% | 63,65 | 2,893 | 0,33 | 0,932 |
| Pure Error | 2 | 17,62 | 11,03% | 17,62 | 8,812 |  |  |
| Total | 26 | 159,81 | 100,00% |  |  |  |  |

## Regression Equation in Uncoded Units

|  |  |  |
| --- | --- | --- |
| F\_mean\_0.33h(20min) | = | -8,76 + 13,29 Lac + 0,229 HPMC\_PS |

## Fits and Diagnostics for All Observations

| Obs | F\_mean\_0.33h(20min) | Fit | SE Fit | 95% CI | Resid | Std Resid | Del Resid |
| --- | --- | --- | --- | --- | --- | --- | --- |
| 1 | 12,421 | 11,767 | 0,558 | (10,615; 12,919) | 0,654 | 0,37 | 0,37 |
| 2 | 16,290 | 15,090 | 0,558 | (13,939; 16,242) | 1,199 | 0,68 | 0,68 |
| 3 | 10,020 | 11,209 | 0,722 | (9,718; 12,699) | -1,189 | -0,70 | -0,69 |
| 4 | 11,393 | 14,532 | 0,722 | (13,041; 16,023) | -3,140 | -1,85 | -1,96 |
| 5 | 10,071 | 11,894 | 0,536 | (10,787; 13,001) | -1,823 | -1,04 | -1,04 |
| 6 | 17,069 | 15,218 | 0,536 | (14,111; 16,324) | 1,852 | 1,05 | 1,05 |
| 7 | 10,510 | 11,527 | 0,617 | (10,254; 12,801) | -1,017 | -0,59 | -0,58 |
| 8 | 13,414 | 14,851 | 0,617 | (13,577; 16,124) | -1,437 | -0,83 | -0,82 |
| 9 | 12,069 | 12,883 | 0,638 | (11,566; 14,199) | -0,814 | -0,47 | -0,46 |
| 10 | 15,416 | 16,206 | 0,638 | (14,890; 17,523) | -0,790 | -0,46 | -0,45 |
| 11 | 12,968 | 12,539 | 0,551 | (11,401; 13,677) | 0,429 | 0,24 | 0,24 |
| 12 | 16,492 | 15,862 | 0,551 | (14,724; 17,001) | 0,629 | 0,36 | 0,35 |
| 13 | 11,347 | 13,060 | 0,697 | (11,622; 14,498) | -1,714 | -1,01 | -1,01 |
| 14 | 16,913 | 16,384 | 0,697 | (14,946; 17,821) | 0,529 | 0,31 | 0,30 |
| 15 | 14,391 | 12,634 | 0,571 | (11,455; 13,812) | 1,757 | 1,00 | 1,00 |
| 16 | 13,890 | 15,957 | 0,571 | (14,779; 17,135) | -2,067 | -1,18 | -1,19 |
| 17 | 11,041 | 10,464 | 0,831 | (8,749; 12,179) | 0,577 | 0,35 | 0,34 |
| 18 | 17,442 | 17,111 | 0,831 | (15,396; 18,825) | 0,331 | 0,20 | 0,20 |
| 19 | 13,390 | 14,003 | 0,365 | (13,248; 14,757) | -0,612 | -0,34 | -0,33 |
| 20 | 17,586 | 13,609 | 0,373 | (12,840; 14,379) | 3,977 | 2,21 | 2,42 |
| 21 | 14,091 | 13,669 | 0,364 | (12,917; 14,421) | 0,423 | 0,23 | 0,23 |
| 22 | 17,139 | 13,937 | 0,358 | (13,197; 14,677) | 3,202 | 1,77 | 1,86 |
| 23 | 12,766 | 12,160 | 0,947 | (10,205; 14,115) | 0,606 | 0,38 | 0,38 |
| 24 | 14,769 | 15,531 | 0,961 | (13,548; 17,514) | -0,762 | -0,49 | -0,48 |
| 25 | 11,694 | 13,787 | 0,355 | (13,055; 14,520) | -2,093 | -1,16 | -1,17 |
| 26 | 11,921 | 13,787 | 0,355 | (13,055; 14,520) | -1,866 | -1,03 | -1,03 |
| 27 | 16,945 | 13,787 | 0,355 | (13,055; 14,520) | 3,158 | 1,75 | 1,83 |

| Obs | HI | Cook’s D | DFITS |  |
| --- | --- | --- | --- | --- |
| 1 | 0,091956 | 0,00 | 0,116515 |  |
| 2 | 0,091956 | 0,02 | 0,215184 |  |
| 3 | 0,154060 | 0,03 | -0,296456 |  |
| 4 | 0,154060 | 0,21 | -0,837261 |  |
| 5 | 0,084910 | 0,03 | -0,315946 |  |
| 6 | 0,084910 | 0,03 | 0,321144 |  |
| 7 | 0,112397 | 0,01 | -0,205806 |  |
| 8 | 0,112397 | 0,03 | -0,292870 |  |
| 9 | 0,120141 | 0,01 | -0,171394 |  |
| 10 | 0,120141 | 0,01 | -0,166298 |  |
| 11 | 0,089807 | 0,00 | 0,075310 |  |
| 12 | 0,089807 | 0,00 | 0,110514 |  |
| 13 | 0,143301 | 0,06 | -0,411558 |  |
| 14 | 0,143301 | 0,01 | 0,124595 |  |
| 15 | 0,096233 | 0,04 | 0,327787 |  |
| 16 | 0,096233 | 0,05 | -0,388944 |  |
| 17 | 0,203866 | 0,01 | 0,174515 |  |
| 18 | 0,203866 | 0,00 | 0,099992 |  |
| 19 | 0,039415 | 0,00 | -0,067462 |  |
| 20 | 0,041065 | 0,07 | 0,500720 | R |
| 21 | 0,039207 | 0,00 | 0,046398 |  |
| 22 | 0,037944 | 0,04 | 0,369933 |  |
| 23 | 0,264924 | 0,02 | 0,226383 |  |
| 24 | 0,272505 | 0,03 | -0,292231 |  |
| 25 | 0,037200 | 0,02 | -0,229617 |  |
| 26 | 0,037200 | 0,01 | -0,203417 |  |
| 27 | 0,037200 | 0,04 | 0,360306 |  |

R  Large residual

## Forward Selection of Terms

α to enter = 0,25

## Coded Coefficients

| Term | Coef | SE Coef | 95% CI | T-Value | P-Value | VIF |
| --- | --- | --- | --- | --- | --- | --- |
| Constant | 16,245 | 0,376 | (15,468; 17,021) | 43,19 | 0,000 |  |
| Lac | 3,740 | 0,798 | (2,093; 5,386) | 4,69 | 0,000 | 1,00 |
| HPMC\_PS | 1,909 | 0,940 | (-0,032; 3,850) | 2,03 | 0,054 | 1,00 |

## Model Summary

| S | R-sq | R-sq(adj) | PRESS | R-sq(pred) | AICc | BIC |
| --- | --- | --- | --- | --- | --- | --- |
| 1,95398 | 52,09% | 48,10% | 108,673 | 43,19% | 119,43 | 122,80 |

## Analysis of Variance

| Source | DF | Seq SS | Contribution | Adj SS | Adj MS | F-Value | P-Value |
| --- | --- | --- | --- | --- | --- | --- | --- |
| Model | 2 | 99,65 | 52,09% | 99,65 | 49,823 | 13,05 | 0,000 |
| Linear | 2 | 99,65 | 52,09% | 99,65 | 49,823 | 13,05 | 0,000 |
| Lac | 1 | 83,92 | 43,87% | 83,92 | 83,920 | 21,98 | 0,000 |
| HPMC\_PS | 1 | 15,73 | 8,22% | 15,73 | 15,727 | 4,12 | 0,054 |
| Error | 24 | 91,63 | 47,91% | 91,63 | 3,818 |  |  |
| Lack-of-Fit | 22 | 72,43 | 37,87% | 72,43 | 3,292 | 0,34 | 0,925 |
| Pure Error | 2 | 19,20 | 10,04% | 19,20 | 9,600 |  |  |
| Total | 26 | 191,28 | 100,00% |  |  |  |  |

## Regression Equation in Uncoded Units

|  |  |  |
| --- | --- | --- |
| F\_mean\_0.5h(30min) | = | -9,31 + 14,96 Lac + 0,260 HPMC\_PS |

## Fits and Diagnostics for All Observations

| Obs | F\_mean\_0.5h(30min) | Fit | SE Fit | 95% CI | Resid | Std Resid | Del Resid |
| --- | --- | --- | --- | --- | --- | --- | --- |
| 1 | 14,629 | 13,903 | 0,593 | (12,680; 15,125) | 0,726 | 0,39 | 0,38 |
| 2 | 18,932 | 17,642 | 0,593 | (16,420; 18,865) | 1,290 | 0,69 | 0,68 |
| 3 | 11,941 | 13,270 | 0,767 | (11,688; 14,853) | -1,329 | -0,74 | -0,73 |
| 4 | 13,736 | 17,010 | 0,767 | (15,427; 18,593) | -3,274 | -1,82 | -1,92 |
| 5 | 12,093 | 14,047 | 0,569 | (12,872; 15,222) | -1,954 | -1,05 | -1,05 |
| 6 | 19,640 | 17,787 | 0,569 | (16,611; 18,962) | 1,853 | 0,99 | 0,99 |
| 7 | 12,627 | 13,631 | 0,655 | (12,279; 14,983) | -1,004 | -0,55 | -0,54 |
| 8 | 15,806 | 17,371 | 0,655 | (16,019; 18,723) | -1,565 | -0,85 | -0,85 |
| 9 | 14,264 | 15,166 | 0,677 | (13,769; 16,564) | -0,902 | -0,49 | -0,48 |
| 10 | 17,960 | 18,906 | 0,677 | (17,508; 20,304) | -0,946 | -0,52 | -0,51 |
| 11 | 15,264 | 14,777 | 0,586 | (13,568; 15,985) | 0,487 | 0,26 | 0,26 |
| 12 | 18,986 | 18,517 | 0,586 | (17,308; 19,725) | 0,469 | 0,25 | 0,25 |
| 13 | 13,544 | 15,367 | 0,740 | (13,840; 16,894) | -1,823 | -1,01 | -1,01 |
| 14 | 19,878 | 19,107 | 0,740 | (17,580; 20,634) | 0,771 | 0,43 | 0,42 |
| 15 | 16,784 | 14,884 | 0,606 | (13,633; 16,135) | 1,900 | 1,02 | 1,02 |
| 16 | 16,332 | 18,624 | 0,606 | (17,373; 19,875) | -2,292 | -1,23 | -1,25 |
| 17 | 12,909 | 12,439 | 0,882 | (10,618; 14,259) | 0,470 | 0,27 | 0,26 |
| 18 | 20,286 | 19,918 | 0,882 | (18,097; 21,739) | 0,367 | 0,21 | 0,21 |
| 19 | 15,883 | 16,422 | 0,388 | (15,622; 17,223) | -0,539 | -0,28 | -0,28 |
| 20 | 20,126 | 15,977 | 0,396 | (15,160; 16,795) | 4,149 | 2,17 | 2,37 |
| 21 | 16,410 | 16,044 | 0,387 | (15,246; 16,843) | 0,366 | 0,19 | 0,19 |
| 22 | 19,930 | 16,348 | 0,381 | (15,563; 17,134) | 3,582 | 1,87 | 1,98 |
| 23 | 15,034 | 14,336 | 1,006 | (12,260; 16,412) | 0,698 | 0,42 | 0,41 |
| 24 | 17,365 | 18,153 | 1,020 | (16,048; 20,259) | -0,788 | -0,47 | -0,47 |
| 25 | 14,088 | 16,178 | 0,377 | (15,401; 16,956) | -2,090 | -1,09 | -1,09 |
| 26 | 14,218 | 16,178 | 0,377 | (15,401; 16,956) | -1,960 | -1,02 | -1,02 |
| 27 | 19,519 | 16,178 | 0,377 | (15,401; 16,956) | 3,340 | 1,74 | 1,82 |

| Obs | HI | Cook’s D | DFITS |  |
| --- | --- | --- | --- | --- |
| 1 | 0,091956 | 0,01 | 0,121897 |  |
| 2 | 0,091956 | 0,02 | 0,217967 |  |
| 3 | 0,154060 | 0,03 | -0,312616 |  |
| 4 | 0,154060 | 0,20 | -0,819815 |  |
| 5 | 0,084910 | 0,03 | -0,319015 |  |
| 6 | 0,084910 | 0,03 | 0,301931 |  |
| 7 | 0,112397 | 0,01 | -0,191121 |  |
| 8 | 0,112397 | 0,03 | -0,300785 |  |
| 9 | 0,120141 | 0,01 | -0,178972 |  |
| 10 | 0,120141 | 0,01 | -0,187840 |  |
| 11 | 0,089807 | 0,00 | 0,080372 |  |
| 12 | 0,089807 | 0,00 | 0,077455 |  |
| 13 | 0,143301 | 0,06 | -0,412427 |  |
| 14 | 0,143301 | 0,01 | 0,171387 |  |
| 15 | 0,096233 | 0,04 | 0,334031 |  |
| 16 | 0,096233 | 0,05 | -0,407227 |  |
| 17 | 0,203866 | 0,01 | 0,133736 |  |
| 18 | 0,203866 | 0,00 | 0,104432 |  |
| 19 | 0,039415 | 0,00 | -0,055950 |  |
| 20 | 0,041065 | 0,07 | 0,489857 | R |
| 21 | 0,039207 | 0,00 | 0,037774 |  |
| 22 | 0,037944 | 0,05 | 0,393071 |  |
| 23 | 0,264924 | 0,02 | 0,245731 |  |
| 24 | 0,272505 | 0,03 | -0,284749 |  |
| 25 | 0,037200 | 0,02 | -0,215155 |  |
| 26 | 0,037200 | 0,01 | -0,201166 |  |
| 27 | 0,037200 | 0,04 | 0,358696 |  |

R  Large residual

## Forward Selection of Terms

α to enter = 0,25

## Coded Coefficients

| Term | Coef | SE Coef | 95% CI | T-Value | P-Value | VIF |
| --- | --- | --- | --- | --- | --- | --- |
| Constant | 19,131 | 0,400 | (18,304; 19,957) | 47,78 | 0,000 |  |
| Lac | 4,179 | 0,849 | (2,427; 5,932) | 4,92 | 0,000 | 1,00 |
| HPMC\_PS | 2,14 | 1,00 | (0,08; 4,21) | 2,14 | 0,043 | 1,00 |

## Model Summary

| S | R-sq | R-sq(adj) | PRESS | R-sq(pred) | AICc | BIC |
| --- | --- | --- | --- | --- | --- | --- |
| 2,07986 | 54,56% | 50,77% | 123,662 | 45,87% | 122,81 | 126,17 |

## Analysis of Variance

| Source | DF | Seq SS | Contribution | Adj SS | Adj MS | F-Value | P-Value |
| --- | --- | --- | --- | --- | --- | --- | --- |
| Model | 2 | 124,64 | 54,56% | 124,64 | 62,320 | 14,41 | 0,000 |
| Linear | 2 | 124,64 | 54,56% | 124,64 | 62,320 | 14,41 | 0,000 |
| Lac | 1 | 104,81 | 45,88% | 104,81 | 104,807 | 24,23 | 0,000 |
| HPMC\_PS | 1 | 19,83 | 8,68% | 19,83 | 19,832 | 4,58 | 0,043 |
| Error | 24 | 103,82 | 45,44% | 103,82 | 4,326 |  |  |
| Lack-of-Fit | 22 | 82,95 | 36,31% | 82,95 | 3,770 | 0,36 | 0,915 |
| Pure Error | 2 | 20,87 | 9,14% | 20,87 | 10,436 |  |  |
| Total | 26 | 228,46 | 100,00% |  |  |  |  |

## Regression Equation in Uncoded Units

|  |  |  |
| --- | --- | --- |
| F\_mean\_0.75h(45min) | = | -9,53 + 16,72 Lac + 0,291 HPMC\_PS |

## Fits and Diagnostics for All Observations

| Obs | F\_mean\_0.75h(45min) | Fit | SE Fit | 95% CI | Resid | Std Resid | Del Resid |
| --- | --- | --- | --- | --- | --- | --- | --- |
| 1 | 17,369 | 16,511 | 0,631 | (15,209; 17,813) | 0,858 | 0,43 | 0,43 |
| 2 | 21,994 | 20,690 | 0,631 | (19,389; 21,992) | 1,304 | 0,66 | 0,65 |
| 3 | 14,259 | 15,801 | 0,816 | (14,116; 17,486) | -1,542 | -0,81 | -0,80 |
| 4 | 16,501 | 19,980 | 0,816 | (18,296; 21,665) | -3,480 | -1,82 | -1,92 |
| 5 | 14,687 | 16,673 | 0,606 | (15,422; 17,924) | -1,985 | -1,00 | -1,00 |
| 6 | 22,707 | 20,852 | 0,606 | (19,601; 22,103) | 1,855 | 0,93 | 0,93 |
| 7 | 15,190 | 16,206 | 0,697 | (14,767; 17,645) | -1,016 | -0,52 | -0,51 |
| 8 | 18,616 | 20,386 | 0,697 | (18,946; 21,825) | -1,770 | -0,90 | -0,90 |
| 9 | 16,906 | 17,930 | 0,721 | (16,442; 19,418) | -1,024 | -0,53 | -0,52 |
| 10 | 21,098 | 22,109 | 0,721 | (20,622; 23,597) | -1,011 | -0,52 | -0,51 |
| 11 | 18,055 | 17,493 | 0,623 | (16,206; 18,779) | 0,562 | 0,28 | 0,28 |
| 12 | 22,100 | 21,672 | 0,623 | (20,386; 22,959) | 0,428 | 0,22 | 0,21 |
| 13 | 16,264 | 18,155 | 0,787 | (16,530; 19,780) | -1,891 | -0,98 | -0,98 |
| 14 | 23,411 | 22,335 | 0,787 | (20,710; 23,960) | 1,077 | 0,56 | 0,55 |
| 15 | 19,597 | 17,613 | 0,645 | (16,281; 18,945) | 1,983 | 1,00 | 1,00 |
| 16 | 19,194 | 21,793 | 0,645 | (20,461; 23,124) | -2,599 | -1,31 | -1,34 |
| 17 | 15,271 | 14,877 | 0,939 | (12,939; 16,815) | 0,394 | 0,21 | 0,21 |
| 18 | 23,700 | 23,236 | 0,939 | (21,298; 25,174) | 0,464 | 0,25 | 0,24 |
| 19 | 18,969 | 19,330 | 0,413 | (18,478; 20,183) | -0,361 | -0,18 | -0,17 |
| 20 | 23,059 | 18,831 | 0,421 | (17,961; 19,700) | 4,229 | 2,08 | 2,24 |
| 21 | 19,298 | 18,906 | 0,412 | (18,056; 19,756) | 0,392 | 0,19 | 0,19 |
| 22 | 23,265 | 19,247 | 0,405 | (18,411; 20,084) | 4,018 | 1,97 | 2,11 |
| 23 | 17,796 | 16,987 | 1,071 | (14,778; 19,197) | 0,809 | 0,45 | 0,45 |
| 24 | 20,282 | 21,274 | 1,086 | (19,033; 23,515) | -0,992 | -0,56 | -0,55 |
| 25 | 16,879 | 19,056 | 0,401 | (18,229; 19,884) | -2,177 | -1,07 | -1,07 |
| 26 | 17,038 | 19,056 | 0,401 | (18,229; 19,884) | -2,019 | -0,99 | -0,99 |
| 27 | 22,552 | 19,056 | 0,401 | (18,229; 19,884) | 3,496 | 1,71 | 1,79 |

| Obs | HI | Cook’s D | DFITS |  |
| --- | --- | --- | --- | --- |
| 1 | 0,091956 | 0,01 | 0,135365 |  |
| 2 | 0,091956 | 0,01 | 0,206768 |  |
| 3 | 0,154060 | 0,04 | -0,341426 |  |
| 4 | 0,154060 | 0,20 | -0,818474 |  |
| 5 | 0,084910 | 0,03 | -0,303934 |  |
| 6 | 0,084910 | 0,03 | 0,283260 |  |
| 7 | 0,112397 | 0,01 | -0,181683 |  |
| 8 | 0,112397 | 0,03 | -0,320111 |  |
| 9 | 0,120141 | 0,01 | -0,191029 |  |
| 10 | 0,120141 | 0,01 | -0,188569 |  |
| 11 | 0,089807 | 0,00 | 0,087216 |  |
| 12 | 0,089807 | 0,00 | 0,066377 |  |
| 13 | 0,143301 | 0,05 | -0,401543 |  |
| 14 | 0,143301 | 0,02 | 0,225381 |  |
| 15 | 0,096233 | 0,04 | 0,327370 |  |
| 16 | 0,096233 | 0,06 | -0,435842 |  |
| 17 | 0,203866 | 0,00 | 0,105221 |  |
| 18 | 0,203866 | 0,01 | 0,123964 |  |
| 19 | 0,039415 | 0,00 | -0,035133 |  |
| 20 | 0,041065 | 0,06 | 0,464358 | R |
| 21 | 0,039207 | 0,00 | 0,038093 |  |
| 22 | 0,037944 | 0,05 | 0,418217 |  |
| 23 | 0,264924 | 0,02 | 0,267800 |  |
| 24 | 0,272505 | 0,04 | -0,337147 |  |
| 25 | 0,037200 | 0,01 | -0,210349 |  |
| 26 | 0,037200 | 0,01 | -0,194339 |  |
| 27 | 0,037200 | 0,04 | 0,351792 |  |

R  Large residual

## Forward Selection of Terms

α to enter = 0,25

## Coded Coefficients

| Term | Coef | SE Coef | 95% CI | T-Value | P-Value | VIF |
| --- | --- | --- | --- | --- | --- | --- |
| Constant | 21,616 | 0,417 | (20,755; 22,478) | 51,81 | 0,000 |  |
| Lac | 4,541 | 0,885 | (2,715; 6,368) | 5,13 | 0,000 | 1,00 |
| HPMC\_PS | 2,26 | 1,04 | (0,11; 4,42) | 2,17 | 0,040 | 1,00 |

## Model Summary

| S | R-sq | R-sq(adj) | PRESS | R-sq(pred) | AICc | BIC |
| --- | --- | --- | --- | --- | --- | --- |
| 2,16772 | 56,40% | 52,76% | 134,939 | 47,83% | 125,04 | 128,40 |

## Analysis of Variance

| Source | DF | Seq SS | Contribution | Adj SS | Adj MS | F-Value | P-Value |
| --- | --- | --- | --- | --- | --- | --- | --- |
| Model | 2 | 145,87 | 56,40% | 145,87 | 72,937 | 15,52 | 0,000 |
| Linear | 2 | 145,87 | 56,40% | 145,87 | 72,937 | 15,52 | 0,000 |
| Lac | 1 | 123,73 | 47,84% | 123,73 | 123,731 | 26,33 | 0,000 |
| HPMC\_PS | 1 | 22,14 | 8,56% | 22,14 | 22,144 | 4,71 | 0,040 |
| Error | 24 | 112,78 | 43,60% | 112,78 | 4,699 |  |  |
| Lack-of-Fit | 22 | 91,48 | 35,37% | 91,48 | 4,158 | 0,39 | 0,900 |
| Pure Error | 2 | 21,30 | 8,23% | 21,30 | 10,648 |  |  |
| Total | 26 | 258,65 | 100,00% |  |  |  |  |

## Regression Equation in Uncoded Units

|  |  |  |
| --- | --- | --- |
| F\_mean\_1h(60min) | = | -8,9 + 18,16 Lac + 0,308 HPMC\_PS |

## Fits and Diagnostics for All Observations

| Obs | F\_mean\_1h(60min) | Fit | SE Fit | 95% CI | Resid | Std Resid | Del Resid |
| --- | --- | --- | --- | --- | --- | --- | --- |
| 1 | 19,761 | 18,786 | 0,657 | (17,429; 20,142) | 0,976 | 0,47 | 0,46 |
| 2 | 24,653 | 23,327 | 0,657 | (21,970; 24,683) | 1,327 | 0,64 | 0,63 |
| 3 | 16,350 | 18,036 | 0,851 | (16,280; 19,792) | -1,685 | -0,85 | -0,84 |
| 4 | 18,938 | 22,577 | 0,851 | (20,821; 24,333) | -3,639 | -1,83 | -1,93 |
| 5 | 16,856 | 18,957 | 0,632 | (17,653; 20,260) | -2,101 | -1,01 | -1,01 |
| 6 | 25,374 | 23,498 | 0,632 | (22,194; 24,801) | 1,876 | 0,90 | 0,90 |
| 7 | 17,428 | 18,464 | 0,727 | (16,964; 19,963) | -1,036 | -0,51 | -0,50 |
| 8 | 21,048 | 23,005 | 0,727 | (21,505; 24,505) | -1,956 | -0,96 | -0,96 |
| 9 | 19,146 | 20,285 | 0,751 | (18,734; 21,836) | -1,139 | -0,56 | -0,55 |
| 10 | 23,860 | 24,826 | 0,751 | (23,276; 26,377) | -0,967 | -0,48 | -0,47 |
| 11 | 20,444 | 19,823 | 0,650 | (18,482; 21,164) | 0,621 | 0,30 | 0,29 |
| 12 | 24,688 | 24,364 | 0,650 | (23,024; 25,705) | 0,324 | 0,16 | 0,15 |
| 13 | 18,608 | 20,523 | 0,821 | (18,830; 22,217) | -1,915 | -0,95 | -0,95 |
| 14 | 26,317 | 25,064 | 0,821 | (23,371; 26,758) | 1,253 | 0,62 | 0,62 |
| 15 | 21,982 | 19,950 | 0,672 | (18,562; 21,338) | 2,032 | 0,99 | 0,99 |
| 16 | 21,578 | 24,491 | 0,672 | (23,104; 25,879) | -2,913 | -1,41 | -1,45 |
| 17 | 17,356 | 16,997 | 0,979 | (14,977; 19,017) | 0,359 | 0,19 | 0,18 |
| 18 | 26,662 | 26,079 | 0,979 | (24,059; 28,099) | 0,583 | 0,30 | 0,30 |
| 19 | 21,678 | 21,827 | 0,430 | (20,939; 22,716) | -0,149 | -0,07 | -0,07 |
| 20 | 25,490 | 21,299 | 0,439 | (20,393; 22,206) | 4,191 | 1,97 | 2,11 |
| 21 | 21,834 | 21,379 | 0,429 | (20,493; 22,265) | 0,456 | 0,21 | 0,21 |
| 22 | 26,073 | 21,740 | 0,422 | (20,868; 22,611) | 4,334 | 2,04 | 2,19 |
| 23 | 20,282 | 19,351 | 1,116 | (17,049; 21,654) | 0,930 | 0,50 | 0,49 |
| 24 | 22,774 | 23,881 | 1,132 | (21,546; 26,217) | -1,108 | -0,60 | -0,59 |
| 25 | 19,368 | 21,538 | 0,418 | (20,675; 22,401) | -2,170 | -1,02 | -1,02 |
| 26 | 19,506 | 21,538 | 0,418 | (20,675; 22,401) | -2,032 | -0,96 | -0,95 |
| 27 | 25,088 | 21,538 | 0,418 | (20,675; 22,401) | 3,550 | 1,67 | 1,74 |

| Obs | HI | Cook’s D | DFITS |  |
| --- | --- | --- | --- | --- |
| 1 | 0,091956 | 0,01 | 0,147829 |  |
| 2 | 0,091956 | 0,01 | 0,201836 |  |
| 3 | 0,154060 | 0,04 | -0,358470 |  |
| 4 | 0,154060 | 0,20 | -0,821667 |  |
| 5 | 0,084910 | 0,03 | -0,308815 |  |
| 6 | 0,084910 | 0,03 | 0,274524 |  |
| 7 | 0,112397 | 0,01 | -0,177615 |  |
| 8 | 0,112397 | 0,04 | -0,340263 |  |
| 9 | 0,120141 | 0,01 | -0,204035 |  |
| 10 | 0,120141 | 0,01 | -0,172804 |  |
| 11 | 0,089807 | 0,00 | 0,092517 |  |
| 12 | 0,089807 | 0,00 | 0,048141 |  |
| 13 | 0,143301 | 0,05 | -0,389678 |  |
| 14 | 0,143301 | 0,02 | 0,252041 |  |
| 15 | 0,096233 | 0,03 | 0,321554 |  |
| 16 | 0,096233 | 0,07 | -0,471669 |  |
| 17 | 0,203866 | 0,00 | 0,092001 |  |
| 18 | 0,203866 | 0,01 | 0,149625 |  |
| 19 | 0,039415 | 0,00 | -0,013897 |  |
| 20 | 0,041065 | 0,06 | 0,436971 |  |
| 21 | 0,039207 | 0,00 | 0,042463 |  |
| 22 | 0,037944 | 0,05 | 0,435773 | R |
| 23 | 0,264924 | 0,03 | 0,295658 |  |
| 24 | 0,272505 | 0,04 | -0,361636 |  |
| 25 | 0,037200 | 0,01 | -0,200714 |  |
| 26 | 0,037200 | 0,01 | -0,187382 |  |
| 27 | 0,037200 | 0,04 | 0,341571 |  |

R  Large residual

## Forward Selection of Terms

α to enter = 0,25

## Coded Coefficients

| Term | Coef | SE Coef | 95% CI | T-Value | P-Value | VIF |
| --- | --- | --- | --- | --- | --- | --- |
| Constant | 25,945 | 0,441 | (25,035; 26,855) | 58,85 | 0,000 |  |
| Lac | 5,104 | 0,935 | (3,174; 7,033) | 5,46 | 0,000 | 1,00 |
| HPMC\_PS | 2,47 | 1,10 | (0,19; 4,74) | 2,24 | 0,035 | 1,00 |

## Model Summary

| S | R-sq | R-sq(adj) | PRESS | R-sq(pred) | AICc | BIC |
| --- | --- | --- | --- | --- | --- | --- |
| 2,29048 | 59,18% | 55,77% | 151,544 | 50,86% | 128,01 | 131,38 |

## Analysis of Variance

| Source | DF | Seq SS | Contribution | Adj SS | Adj MS | F-Value | P-Value |
| --- | --- | --- | --- | --- | --- | --- | --- |
| Model | 2 | 182,51 | 59,18% | 182,51 | 91,254 | 17,39 | 0,000 |
| Linear | 2 | 182,51 | 59,18% | 182,51 | 91,254 | 17,39 | 0,000 |
| Lac | 1 | 156,28 | 50,67% | 156,28 | 156,277 | 29,79 | 0,000 |
| HPMC\_PS | 1 | 26,23 | 8,50% | 26,23 | 26,231 | 5,00 | 0,035 |
| Error | 24 | 125,91 | 40,82% | 125,91 | 5,246 |  |  |
| Lack-of-Fit | 22 | 103,50 | 33,56% | 103,50 | 4,705 | 0,42 | 0,884 |
| Pure Error | 2 | 22,41 | 7,27% | 22,41 | 11,206 |  |  |
| Total | 26 | 308,42 | 100,00% |  |  |  |  |

## Regression Equation in Uncoded Units

|  |  |  |
| --- | --- | --- |
| F\_mean\_1.5h(90min) | = | -7,6 + 20,41 Lac + 0,335 HPMC\_PS |

## Fits and Diagnostics for All Observations

| Obs | F\_mean\_1.5h(90min) | Fit | SE Fit | 95% CI | Resid | Std Resid | Del Resid |
| --- | --- | --- | --- | --- | --- | --- | --- |
| 1 | 23,960 | 22,783 | 0,695 | (21,350; 24,217) | 1,177 | 0,54 | 0,53 |
| 2 | 29,242 | 27,887 | 0,695 | (26,453; 29,320) | 1,356 | 0,62 | 0,61 |
| 3 | 20,030 | 21,967 | 0,899 | (20,111; 23,822) | -1,937 | -0,92 | -0,92 |
| 4 | 23,256 | 27,070 | 0,899 | (25,215; 28,926) | -3,814 | -1,81 | -1,91 |
| 5 | 20,814 | 22,969 | 0,667 | (21,592; 24,347) | -2,155 | -0,98 | -0,98 |
| 6 | 29,979 | 28,073 | 0,667 | (26,695; 29,450) | 1,906 | 0,87 | 0,87 |
| 7 | 21,354 | 22,433 | 0,768 | (20,848; 24,018) | -1,079 | -0,50 | -0,49 |
| 8 | 25,207 | 27,536 | 0,768 | (25,951; 29,121) | -2,329 | -1,08 | -1,08 |
| 9 | 23,222 | 24,415 | 0,794 | (22,777; 26,054) | -1,193 | -0,56 | -0,55 |
| 10 | 28,652 | 29,519 | 0,794 | (27,880; 31,157) | -0,867 | -0,40 | -0,40 |
| 11 | 24,520 | 23,912 | 0,686 | (22,496; 25,329) | 0,607 | 0,28 | 0,27 |
| 12 | 29,209 | 29,016 | 0,686 | (27,599; 30,433) | 0,193 | 0,09 | 0,09 |
| 13 | 22,644 | 24,675 | 0,867 | (22,885; 26,464) | -2,031 | -0,96 | -0,96 |
| 14 | 31,222 | 29,778 | 0,867 | (27,989; 31,568) | 1,444 | 0,68 | 0,67 |
| 15 | 26,099 | 24,051 | 0,711 | (22,584; 25,517) | 2,048 | 0,94 | 0,94 |
| 16 | 25,890 | 29,154 | 0,711 | (27,688; 30,621) | -3,264 | -1,50 | -1,54 |
| 17 | 21,094 | 20,756 | 1,034 | (18,621; 22,890) | 0,338 | 0,17 | 0,16 |
| 18 | 31,707 | 30,963 | 1,034 | (28,828; 33,097) | 0,745 | 0,36 | 0,36 |
| 19 | 26,440 | 26,174 | 0,455 | (25,236; 27,113) | 0,265 | 0,12 | 0,12 |
| 20 | 29,744 | 25,600 | 0,464 | (24,642; 26,558) | 4,145 | 1,85 | 1,95 |
| 21 | 26,102 | 25,686 | 0,454 | (24,750; 26,622) | 0,416 | 0,19 | 0,18 |
| 22 | 30,808 | 26,079 | 0,446 | (25,158; 27,000) | 4,730 | 2,11 | 2,28 |
| 23 | 24,646 | 23,480 | 1,179 | (21,046; 25,913) | 1,166 | 0,59 | 0,59 |
| 24 | 27,178 | 28,410 | 1,196 | (25,942; 30,878) | -1,232 | -0,63 | -0,62 |
| 25 | 23,641 | 25,859 | 0,442 | (24,948; 26,771) | -2,219 | -0,99 | -0,99 |
| 26 | 23,791 | 25,859 | 0,442 | (24,948; 26,771) | -2,068 | -0,92 | -0,92 |
| 27 | 29,512 | 25,859 | 0,442 | (24,948; 26,771) | 3,653 | 1,63 | 1,69 |

| Obs | HI | Cook’s D | DFITS |  |
| --- | --- | --- | --- | --- |
| 1 | 0,091956 | 0,01 | 0,169043 |  |
| 2 | 0,091956 | 0,01 | 0,195048 |  |
| 3 | 0,154060 | 0,05 | -0,391094 |  |
| 4 | 0,154060 | 0,20 | -0,814073 |  |
| 5 | 0,084910 | 0,03 | -0,299438 |  |
| 6 | 0,084910 | 0,02 | 0,263552 |  |
| 7 | 0,112397 | 0,01 | -0,175079 |  |
| 8 | 0,112397 | 0,05 | -0,385453 |  |
| 9 | 0,120141 | 0,01 | -0,202223 |  |
| 10 | 0,120141 | 0,01 | -0,146436 |  |
| 11 | 0,089807 | 0,00 | 0,085581 |  |
| 12 | 0,089807 | 0,00 | 0,027158 |  |
| 13 | 0,143301 | 0,05 | -0,391066 |  |
| 14 | 0,143301 | 0,03 | 0,275404 |  |
| 15 | 0,096233 | 0,03 | 0,306110 |  |
| 16 | 0,096233 | 0,08 | -0,502954 |  |
| 17 | 0,203866 | 0,00 | 0,082056 |  |
| 18 | 0,203866 | 0,01 | 0,180995 |  |
| 19 | 0,039415 | 0,00 | 0,023459 |  |
| 20 | 0,041065 | 0,05 | 0,404194 |  |
| 21 | 0,039207 | 0,00 | 0,036683 |  |
| 22 | 0,037944 | 0,06 | 0,453261 | R |
| 23 | 0,264924 | 0,04 | 0,351632 |  |
| 24 | 0,272505 | 0,05 | -0,380996 |  |
| 25 | 0,037200 | 0,01 | -0,193923 |  |
| 26 | 0,037200 | 0,01 | -0,180306 |  |
| 27 | 0,037200 | 0,03 | 0,331547 |  |

R  Large residual

## Forward Selection of Terms

α to enter = 0,25

## Coded Coefficients

| Term | Coef | SE Coef | 95% CI | T-Value | P-Value | VIF |
| --- | --- | --- | --- | --- | --- | --- |
| Constant | 29,744 | 0,458 | (28,798; 30,690) | 64,90 | 0,000 |  |
| Lac | 5,573 | 0,972 | (3,566; 7,579) | 5,73 | 0,000 | 1,00 |
| HPMC\_PS | 2,59 | 1,15 | (0,22; 4,96) | 2,26 | 0,033 | 1,00 |

## Model Summary

| S | R-sq | R-sq(adj) | PRESS | R-sq(pred) | AICc | BIC |
| --- | --- | --- | --- | --- | --- | --- |
| 2,38097 | 61,27% | 58,05% | 164,631 | 53,14% | 130,11 | 133,47 |

## Analysis of Variance

| Source | DF | Seq SS | Contribution | Adj SS | Adj MS | F-Value | P-Value |
| --- | --- | --- | --- | --- | --- | --- | --- |
| Model | 2 | 215,28 | 61,27% | 215,28 | 107,641 | 18,99 | 0,000 |
| Linear | 2 | 215,28 | 61,27% | 215,28 | 107,641 | 18,99 | 0,000 |
| Lac | 1 | 186,33 | 53,03% | 186,33 | 186,326 | 32,87 | 0,000 |
| HPMC\_PS | 1 | 28,96 | 8,24% | 28,96 | 28,956 | 5,11 | 0,033 |
| Error | 24 | 136,06 | 38,73% | 136,06 | 5,669 |  |  |
| Lack-of-Fit | 22 | 113,70 | 32,36% | 113,70 | 5,168 | 0,46 | 0,861 |
| Pure Error | 2 | 22,36 | 6,36% | 22,36 | 11,180 |  |  |
| Total | 26 | 351,34 | 100,00% |  |  |  |  |

## Regression Equation in Uncoded Units

|  |  |  |
| --- | --- | --- |
| F\_mean\_2h(120min) | = | -5,9 + 22,29 Lac + 0,352 HPMC\_PS |

## Fits and Diagnostics for All Observations

| Obs | F\_mean\_2h(120min) | Fit | SE Fit | 95% CI | Resid | Std Resid | Del Resid |
| --- | --- | --- | --- | --- | --- | --- | --- |
| 1 | 27,647 | 26,317 | 0,722 | (24,827; 27,807) | 1,330 | 0,59 | 0,58 |
| 2 | 33,275 | 31,890 | 0,722 | (30,399; 33,380) | 1,385 | 0,61 | 0,60 |
| 3 | 23,330 | 25,459 | 0,935 | (23,531; 27,388) | -2,130 | -0,97 | -0,97 |
| 4 | 27,018 | 31,032 | 0,935 | (29,103; 32,961) | -4,014 | -1,83 | -1,93 |
| 5 | 24,319 | 26,513 | 0,694 | (25,081; 27,944) | -2,194 | -0,96 | -0,96 |
| 6 | 33,930 | 32,085 | 0,694 | (30,653; 33,517) | 1,845 | 0,81 | 0,80 |
| 7 | 24,866 | 25,949 | 0,798 | (24,301; 27,596) | -1,083 | -0,48 | -0,47 |
| 8 | 28,958 | 31,521 | 0,798 | (29,874; 33,169) | -2,564 | -1,14 | -1,15 |
| 9 | 26,769 | 28,032 | 0,825 | (26,329; 29,735) | -1,263 | -0,57 | -0,56 |
| 10 | 32,839 | 33,604 | 0,825 | (31,901; 35,308) | -0,766 | -0,34 | -0,34 |
| 11 | 28,140 | 27,503 | 0,714 | (26,031; 28,976) | 0,637 | 0,28 | 0,27 |
| 12 | 33,175 | 33,076 | 0,714 | (31,603; 34,549) | 0,099 | 0,04 | 0,04 |
| 13 | 26,177 | 28,304 | 0,901 | (26,444; 30,164) | -2,127 | -0,97 | -0,96 |
| 14 | 35,420 | 33,877 | 0,901 | (32,017; 35,737) | 1,543 | 0,70 | 0,69 |
| 15 | 29,734 | 27,649 | 0,739 | (26,124; 29,173) | 2,085 | 0,92 | 0,92 |
| 16 | 29,696 | 33,222 | 0,739 | (31,697; 34,746) | -3,526 | -1,56 | -1,61 |
| 17 | 24,325 | 24,082 | 1,075 | (21,863; 26,300) | 0,244 | 0,11 | 0,11 |
| 18 | 36,098 | 35,227 | 1,075 | (33,008; 37,446) | 0,871 | 0,41 | 0,40 |
| 19 | 30,544 | 29,985 | 0,473 | (29,010; 30,961) | 0,559 | 0,24 | 0,23 |
| 20 | 33,491 | 29,381 | 0,482 | (28,385; 30,377) | 4,110 | 1,76 | 1,85 |
| 21 | 29,839 | 29,472 | 0,471 | (28,499; 30,445) | 0,367 | 0,16 | 0,15 |
| 22 | 34,911 | 29,885 | 0,464 | (28,928; 30,842) | 5,027 | 2,15 | 2,35 |
| 23 | 28,553 | 27,154 | 1,226 | (24,625; 29,683) | 1,399 | 0,69 | 0,68 |
| 24 | 31,053 | 32,334 | 1,243 | (29,769; 34,899) | -1,281 | -0,63 | -0,62 |
| 25 | 27,483 | 29,654 | 0,459 | (28,706; 30,602) | -2,171 | -0,93 | -0,93 |
| 26 | 27,597 | 29,654 | 0,459 | (28,706; 30,602) | -2,057 | -0,88 | -0,88 |
| 27 | 33,330 | 29,654 | 0,459 | (28,706; 30,602) | 3,676 | 1,57 | 1,63 |

| Obs | HI | Cook’s D | DFITS |  |
| --- | --- | --- | --- | --- |
| 1 | 0,091956 | 0,01 | 0,184000 |  |
| 2 | 0,091956 | 0,01 | 0,191659 |  |
| 3 | 0,154060 | 0,06 | -0,414521 |  |
| 4 | 0,154060 | 0,20 | -0,825719 |  |
| 5 | 0,084910 | 0,03 | -0,292926 |  |
| 6 | 0,084910 | 0,02 | 0,244885 |  |
| 7 | 0,112397 | 0,01 | -0,168949 |  |
| 8 | 0,112397 | 0,06 | -0,409414 |  |
| 9 | 0,120141 | 0,01 | -0,205973 |  |
| 10 | 0,120141 | 0,01 | -0,124352 |  |
| 11 | 0,089807 | 0,00 | 0,086361 |  |
| 12 | 0,089807 | 0,00 | 0,013373 |  |
| 13 | 0,143301 | 0,05 | -0,394218 |  |
| 14 | 0,143301 | 0,03 | 0,283231 |  |
| 15 | 0,096233 | 0,03 | 0,299575 |  |
| 16 | 0,096233 | 0,09 | -0,524843 |  |
| 17 | 0,203866 | 0,00 | 0,056886 |  |
| 18 | 0,203866 | 0,01 | 0,203756 |  |
| 19 | 0,039415 | 0,00 | 0,047520 |  |
| 20 | 0,041065 | 0,04 | 0,382700 |  |
| 21 | 0,039207 | 0,00 | 0,031086 |  |
| 22 | 0,037944 | 0,06 | 0,465821 | R |
| 23 | 0,264924 | 0,06 | 0,406771 |  |
| 24 | 0,272505 | 0,05 | -0,381056 |  |
| 25 | 0,037200 | 0,01 | -0,182152 |  |
| 26 | 0,037200 | 0,01 | -0,172226 |  |
| 27 | 0,037200 | 0,03 | 0,319733 |  |

R  Large residual

## Forward Selection of Terms

α to enter = 0,25

## Coded Coefficients

| Term | Coef | SE Coef | 95% CI | T-Value | P-Value | VIF |
| --- | --- | --- | --- | --- | --- | --- |
| Constant | 33,182 | 0,474 | (32,203; 34,161) | 69,97 | 0,000 |  |
| Lac | 5,96 | 1,01 | (3,88; 8,03) | 5,92 | 0,000 | 1,00 |
| HPMC\_PS | 2,67 | 1,19 | (0,23; 5,12) | 2,25 | 0,034 | 1,00 |

## Model Summary

| S | R-sq | R-sq(adj) | PRESS | R-sq(pred) | AICc | BIC |
| --- | --- | --- | --- | --- | --- | --- |
| 2,46370 | 62,60% | 59,48% | 177,155 | 54,51% | 131,95 | 135,32 |

## Analysis of Variance

| Source | DF | Seq SS | Contribution | Adj SS | Adj MS | F-Value | P-Value |
| --- | --- | --- | --- | --- | --- | --- | --- |
| Model | 2 | 243,79 | 62,60% | 243,79 | 121,897 | 20,08 | 0,000 |
| Linear | 2 | 243,79 | 62,60% | 243,79 | 121,897 | 20,08 | 0,000 |
| Lac | 1 | 212,96 | 54,68% | 212,96 | 212,960 | 35,09 | 0,000 |
| HPMC\_PS | 1 | 30,83 | 7,92% | 30,83 | 30,834 | 5,08 | 0,034 |
| Error | 24 | 145,68 | 37,40% | 145,68 | 6,070 |  |  |
| Lack-of-Fit | 22 | 123,63 | 31,74% | 123,63 | 5,620 | 0,51 | 0,836 |
| Pure Error | 2 | 22,04 | 5,66% | 22,04 | 11,022 |  |  |
| Total | 26 | 389,47 | 100,00% |  |  |  |  |

## Regression Equation in Uncoded Units

|  |  |  |
| --- | --- | --- |
| F\_mean\_2.5h(150min) | = | -4,0 + 23,83 Lac + 0,363 HPMC\_PS |

## Fits and Diagnostics for All Observations

| Obs | F\_mean\_2.5h(150min) | Fit | SE Fit | 95% CI | Resid | Std Resid | Del Resid |
| --- | --- | --- | --- | --- | --- | --- | --- |
| 1 | 30,966 | 29,542 | 0,747 | (28,000; 31,084) | 1,424 | 0,61 | 0,60 |
| 2 | 36,876 | 35,500 | 0,747 | (33,958; 37,042) | 1,377 | 0,59 | 0,58 |
| 3 | 26,375 | 28,657 | 0,967 | (26,661; 30,653) | -2,281 | -1,01 | -1,01 |
| 4 | 30,399 | 34,615 | 0,967 | (32,619; 36,610) | -4,215 | -1,86 | -1,97 |
| 5 | 27,471 | 29,744 | 0,718 | (28,262; 31,225) | -2,273 | -0,96 | -0,96 |
| 6 | 37,628 | 35,701 | 0,718 | (34,220; 37,183) | 1,926 | 0,82 | 0,81 |
| 7 | 28,090 | 29,162 | 0,826 | (27,457; 30,867) | -1,072 | -0,46 | -0,45 |
| 8 | 32,301 | 35,120 | 0,826 | (33,415; 36,824) | -2,818 | -1,21 | -1,23 |
| 9 | 29,986 | 31,312 | 0,854 | (29,549; 33,074) | -1,325 | -0,57 | -0,57 |
| 10 | 36,545 | 37,269 | 0,854 | (35,507; 39,032) | -0,724 | -0,31 | -0,31 |
| 11 | 31,457 | 30,766 | 0,738 | (29,243; 32,290) | 0,690 | 0,29 | 0,29 |
| 12 | 36,736 | 36,724 | 0,738 | (35,200; 38,248) | 0,012 | 0,01 | 0,01 |
| 13 | 29,407 | 31,593 | 0,933 | (29,668; 33,517) | -2,185 | -0,96 | -0,96 |
| 14 | 39,136 | 37,550 | 0,933 | (35,625; 39,475) | 1,585 | 0,70 | 0,69 |
| 15 | 33,013 | 30,916 | 0,764 | (29,339; 32,494) | 2,097 | 0,90 | 0,89 |
| 16 | 33,169 | 36,874 | 0,764 | (35,297; 38,451) | -3,705 | -1,58 | -1,64 |
| 17 | 27,300 | 27,132 | 1,112 | (24,836; 29,427) | 0,168 | 0,08 | 0,07 |
| 18 | 40,033 | 39,047 | 1,112 | (36,751; 41,343) | 0,986 | 0,45 | 0,44 |
| 19 | 34,291 | 33,431 | 0,489 | (32,421; 34,440) | 0,860 | 0,36 | 0,35 |
| 20 | 36,830 | 32,808 | 0,499 | (31,777; 33,838) | 4,023 | 1,67 | 1,74 |
| 21 | 33,170 | 32,901 | 0,488 | (31,895; 33,908) | 0,269 | 0,11 | 0,11 |
| 22 | 38,633 | 33,327 | 0,480 | (32,337; 34,318) | 5,306 | 2,20 | 2,40 |
| 23 | 32,129 | 30,509 | 1,268 | (27,892; 33,126) | 1,619 | 0,77 | 0,76 |
| 24 | 34,525 | 35,855 | 1,286 | (33,200; 38,509) | -1,330 | -0,63 | -0,62 |
| 25 | 30,945 | 33,089 | 0,475 | (32,109; 34,070) | -2,144 | -0,89 | -0,88 |
| 26 | 31,126 | 33,089 | 0,475 | (32,109; 34,070) | -1,964 | -0,81 | -0,81 |
| 27 | 36,783 | 33,089 | 0,475 | (32,109; 34,070) | 3,694 | 1,53 | 1,57 |

| Obs | HI | Cook’s D | DFITS |  |
| --- | --- | --- | --- | --- |
| 1 | 0,091956 | 0,01 | 0,190396 |  |
| 2 | 0,091956 | 0,01 | 0,184013 |  |
| 3 | 0,154060 | 0,06 | -0,429785 |  |
| 4 | 0,154060 | 0,21 | -0,840072 |  |
| 5 | 0,084910 | 0,03 | -0,293359 |  |
| 6 | 0,084910 | 0,02 | 0,247185 |  |
| 7 | 0,112397 | 0,01 | -0,161561 |  |
| 8 | 0,112397 | 0,06 | -0,436572 |  |
| 9 | 0,120141 | 0,01 | -0,208916 |  |
| 10 | 0,120141 | 0,00 | -0,113594 |  |
| 11 | 0,089807 | 0,00 | 0,090487 |  |
| 12 | 0,089807 | 0,00 | 0,001606 |  |
| 13 | 0,143301 | 0,05 | -0,391256 |  |
| 14 | 0,143301 | 0,03 | 0,281217 |  |
| 15 | 0,096233 | 0,03 | 0,290883 |  |
| 16 | 0,096233 | 0,09 | -0,533897 |  |
| 17 | 0,203866 | 0,00 | 0,037932 |  |
| 18 | 0,203866 | 0,02 | 0,223179 |  |
| 19 | 0,039415 | 0,00 | 0,070800 |  |
| 20 | 0,041065 | 0,04 | 0,359239 |  |
| 21 | 0,039207 | 0,00 | 0,022022 |  |
| 22 | 0,037944 | 0,06 | 0,477529 | R |
| 23 | 0,264924 | 0,07 | 0,456160 |  |
| 24 | 0,272505 | 0,05 | -0,382315 |  |
| 25 | 0,037200 | 0,01 | -0,173557 |  |
| 26 | 0,037200 | 0,01 | -0,158500 |  |
| 27 | 0,037200 | 0,03 | 0,309495 |  |

R  Large residual

## Forward Selection of Terms

α to enter = 0,25

## Coded Coefficients

| Term | Coef | SE Coef | 95% CI | T-Value | P-Value | VIF |
| --- | --- | --- | --- | --- | --- | --- |
| Constant | 36,354 | 0,483 | (35,357; 37,352) | 75,21 | 0,000 |  |
| Lac | 6,29 | 1,03 | (4,18; 8,41) | 6,14 | 0,000 | 1,00 |
| HPMC\_PS | 2,73 | 1,21 | (0,24; 5,23) | 2,26 | 0,033 | 1,00 |

## Model Summary

| S | R-sq | R-sq(adj) | PRESS | R-sq(pred) | AICc | BIC |
| --- | --- | --- | --- | --- | --- | --- |
| 2,51115 | 64,06% | 61,06% | 184,728 | 56,13% | 132,98 | 136,35 |

## Analysis of Variance

| Source | DF | Seq SS | Contribution | Adj SS | Adj MS | F-Value | P-Value |
| --- | --- | --- | --- | --- | --- | --- | --- |
| Model | 2 | 269,75 | 64,06% | 269,75 | 134,873 | 21,39 | 0,000 |
| Linear | 2 | 269,75 | 64,06% | 269,75 | 134,873 | 21,39 | 0,000 |
| Lac | 1 | 237,54 | 56,41% | 237,54 | 237,540 | 37,67 | 0,000 |
| HPMC\_PS | 1 | 32,21 | 7,65% | 32,21 | 32,207 | 5,11 | 0,033 |
| Error | 24 | 151,34 | 35,94% | 151,34 | 6,306 |  |  |
| Lack-of-Fit | 22 | 129,72 | 30,81% | 129,72 | 5,896 | 0,55 | 0,817 |
| Pure Error | 2 | 21,62 | 5,13% | 21,62 | 10,810 |  |  |
| Total | 26 | 421,09 | 100,00% |  |  |  |  |

## Regression Equation in Uncoded Units

|  |  |  |
| --- | --- | --- |
| F\_mean\_3h(180min) | = | -2,1 + 25,17 Lac + 0,371 HPMC\_PS |

## Fits and Diagnostics for All Observations

| Obs | F\_mean\_3h(180min) | Fit | SE Fit | 95% CI | Resid | Std Resid | Del Resid |
| --- | --- | --- | --- | --- | --- | --- | --- |
| 1 | 34,028 | 32,533 | 0,761 | (30,961; 34,104) | 1,496 | 0,63 | 0,62 |
| 2 | 40,223 | 38,825 | 0,761 | (37,253; 40,396) | 1,398 | 0,58 | 0,58 |
| 3 | 29,168 | 31,628 | 0,986 | (29,594; 33,662) | -2,460 | -1,07 | -1,07 |
| 4 | 33,625 | 37,920 | 0,986 | (35,886; 39,954) | -4,295 | -1,86 | -1,97 |
| 5 | 30,469 | 32,739 | 0,732 | (31,229; 34,249) | -2,270 | -0,94 | -0,94 |
| 6 | 40,923 | 39,031 | 0,732 | (37,521; 40,541) | 1,892 | 0,79 | 0,78 |
| 7 | 31,112 | 32,144 | 0,842 | (30,407; 33,882) | -1,033 | -0,44 | -0,43 |
| 8 | 35,438 | 38,436 | 0,842 | (36,699; 40,174) | -2,998 | -1,27 | -1,28 |
| 9 | 32,975 | 34,341 | 0,870 | (32,545; 36,138) | -1,366 | -0,58 | -0,57 |
| 10 | 39,972 | 40,633 | 0,870 | (38,837; 42,430) | -0,662 | -0,28 | -0,28 |
| 11 | 34,494 | 33,784 | 0,753 | (32,231; 35,337) | 0,710 | 0,30 | 0,29 |
| 12 | 39,969 | 40,076 | 0,753 | (38,523; 41,629) | -0,107 | -0,04 | -0,04 |
| 13 | 32,410 | 34,628 | 0,951 | (32,666; 36,590) | -2,218 | -0,95 | -0,95 |
| 14 | 42,546 | 40,920 | 0,951 | (38,958; 42,882) | 1,625 | 0,70 | 0,69 |
| 15 | 35,988 | 33,937 | 0,779 | (32,329; 35,545) | 2,051 | 0,86 | 0,85 |
| 16 | 36,412 | 40,229 | 0,779 | (38,622; 41,837) | -3,818 | -1,60 | -1,66 |
| 17 | 30,093 | 29,968 | 1,134 | (27,627; 32,308) | 0,125 | 0,06 | 0,05 |
| 18 | 43,614 | 42,552 | 1,134 | (40,212; 44,892) | 1,063 | 0,47 | 0,47 |
| 19 | 37,657 | 36,609 | 0,499 | (35,580; 37,638) | 1,048 | 0,43 | 0,42 |
| 20 | 39,839 | 35,972 | 0,509 | (34,921; 37,022) | 3,868 | 1,57 | 1,63 |
| 21 | 36,222 | 36,068 | 0,497 | (35,041; 37,094) | 0,154 | 0,06 | 0,06 |
| 22 | 42,049 | 36,503 | 0,489 | (35,493; 37,512) | 5,547 | 2,25 | 2,48 |
| 23 | 35,448 | 33,623 | 1,293 | (30,955; 36,290) | 1,825 | 0,85 | 0,84 |
| 24 | 37,768 | 39,086 | 1,311 | (36,380; 41,791) | -1,317 | -0,62 | -0,61 |
| 25 | 34,152 | 36,260 | 0,484 | (35,260; 37,259) | -2,107 | -0,86 | -0,85 |
| 26 | 34,401 | 36,260 | 0,484 | (35,260; 37,259) | -1,858 | -0,75 | -0,75 |
| 27 | 39,967 | 36,260 | 0,484 | (35,260; 37,259) | 3,708 | 1,50 | 1,55 |

| Obs | HI | Cook’s D | DFITS |  |
| --- | --- | --- | --- | --- |
| 1 | 0,091956 | 0,01 | 0,196338 |  |
| 2 | 0,091956 | 0,01 | 0,183327 |  |
| 3 | 0,154060 | 0,07 | -0,455917 |  |
| 4 | 0,154060 | 0,21 | -0,839827 |  |
| 5 | 0,084910 | 0,03 | -0,287127 |  |
| 6 | 0,084910 | 0,02 | 0,237948 |  |
| 7 | 0,112397 | 0,01 | -0,152641 |  |
| 8 | 0,112397 | 0,07 | -0,456984 |  |
| 9 | 0,120141 | 0,02 | -0,211340 |  |
| 10 | 0,120141 | 0,00 | -0,101775 |  |
| 11 | 0,089807 | 0,00 | 0,091353 |  |
| 12 | 0,089807 | 0,00 | -0,013776 |  |
| 13 | 0,143301 | 0,05 | -0,389544 |  |
| 14 | 0,143301 | 0,03 | 0,282855 |  |
| 15 | 0,096233 | 0,03 | 0,278713 |  |
| 16 | 0,096233 | 0,09 | -0,540463 |  |
| 17 | 0,203866 | 0,00 | 0,027736 |  |
| 18 | 0,203866 | 0,02 | 0,236096 |  |
| 19 | 0,039415 | 0,00 | 0,084768 |  |
| 20 | 0,041065 | 0,04 | 0,336425 |  |
| 21 | 0,039207 | 0,00 | 0,012393 |  |
| 22 | 0,037944 | 0,07 | 0,492967 | R |
| 23 | 0,264924 | 0,09 | 0,505906 |  |
| 24 | 0,272505 | 0,05 | -0,371447 |  |
| 25 | 0,037200 | 0,01 | -0,167149 |  |
| 26 | 0,037200 | 0,01 | -0,146866 |  |
| 27 | 0,037200 | 0,03 | 0,304265 |  |

R  Large residual

## Forward Selection of Terms

α to enter = 0,25

## Coded Coefficients

| Term | Coef | SE Coef | 95% CI | T-Value | P-Value | VIF |
| --- | --- | --- | --- | --- | --- | --- |
| Constant | 39,348 | 0,492 | (38,332; 40,364) | 79,96 | 0,000 |  |
| Lac | 6,62 | 1,04 | (4,46; 8,77) | 6,34 | 0,000 | 1,00 |
| HPMC\_PS | 2,77 | 1,23 | (0,23; 5,31) | 2,25 | 0,034 | 1,00 |

## Model Summary

| S | R-sq | R-sq(adj) | PRESS | R-sq(pred) | AICc | BIC |
| --- | --- | --- | --- | --- | --- | --- |
| 2,55653 | 65,35% | 62,46% | 192,158 | 57,55% | 133,95 | 137,31 |

## Analysis of Variance

| Source | DF | Seq SS | Contribution | Adj SS | Adj MS | F-Value | P-Value |
| --- | --- | --- | --- | --- | --- | --- | --- |
| Model | 2 | 295,79 | 65,35% | 295,79 | 147,896 | 22,63 | 0,000 |
| Linear | 2 | 295,79 | 65,35% | 295,79 | 147,896 | 22,63 | 0,000 |
| Lac | 1 | 262,71 | 58,04% | 262,71 | 262,710 | 40,20 | 0,000 |
| HPMC\_PS | 1 | 33,08 | 7,31% | 33,08 | 33,081 | 5,06 | 0,034 |
| Error | 24 | 156,86 | 34,65% | 156,86 | 6,536 |  |  |
| Lack-of-Fit | 22 | 135,19 | 29,87% | 135,19 | 6,145 | 0,57 | 0,805 |
| Pure Error | 2 | 21,67 | 4,79% | 21,67 | 10,835 |  |  |
| Total | 26 | 452,65 | 100,00% |  |  |  |  |

## Regression Equation in Uncoded Units

|  |  |  |
| --- | --- | --- |
| F\_mean\_3.5h(210min) | = | -0,1 + 26,47 Lac + 0,376 HPMC\_PS |

## Fits and Diagnostics for All Observations

| Obs | F\_mean\_3.5h(210min) | Fit | SE Fit | 95% CI | Resid | Std Resid | Del Resid |
| --- | --- | --- | --- | --- | --- | --- | --- |
| 1 | 36,866 | 35,355 | 0,775 | (33,755; 36,955) | 1,511 | 0,62 | 0,61 |
| 2 | 43,414 | 41,972 | 0,775 | (40,372; 43,572) | 1,442 | 0,59 | 0,58 |
| 3 | 31,856 | 34,438 | 1,003 | (32,367; 36,509) | -2,582 | -1,10 | -1,10 |
| 4 | 36,620 | 41,055 | 1,003 | (38,984; 43,126) | -4,435 | -1,89 | -2,00 |
| 5 | 33,315 | 35,564 | 0,745 | (34,026; 37,101) | -2,249 | -0,92 | -0,92 |
| 6 | 44,065 | 42,181 | 0,745 | (40,643; 43,718) | 1,884 | 0,77 | 0,76 |
| 7 | 33,956 | 34,961 | 0,857 | (33,192; 36,730) | -1,005 | -0,42 | -0,41 |
| 8 | 38,419 | 41,578 | 0,857 | (39,809; 43,347) | -3,159 | -1,31 | -1,33 |
| 9 | 35,760 | 37,188 | 0,886 | (35,359; 39,017) | -1,428 | -0,60 | -0,59 |
| 10 | 43,218 | 43,805 | 0,886 | (41,976; 45,634) | -0,587 | -0,24 | -0,24 |
| 11 | 37,353 | 36,623 | 0,766 | (35,042; 38,204) | 0,730 | 0,30 | 0,29 |
| 12 | 42,997 | 43,240 | 0,766 | (41,659; 44,821) | -0,243 | -0,10 | -0,10 |
| 13 | 35,261 | 37,479 | 0,968 | (35,481; 39,476) | -2,218 | -0,94 | -0,93 |
| 14 | 45,725 | 44,096 | 0,968 | (42,098; 46,093) | 1,629 | 0,69 | 0,68 |
| 15 | 38,758 | 36,778 | 0,793 | (35,142; 38,415) | 1,979 | 0,81 | 0,81 |
| 16 | 39,498 | 43,395 | 0,793 | (41,759; 45,032) | -3,897 | -1,60 | -1,66 |
| 17 | 32,680 | 32,635 | 1,154 | (30,253; 35,017) | 0,045 | 0,02 | 0,02 |
| 18 | 46,967 | 45,869 | 1,154 | (43,487; 48,252) | 1,098 | 0,48 | 0,47 |
| 19 | 40,842 | 39,606 | 0,508 | (38,558; 40,653) | 1,236 | 0,49 | 0,49 |
| 20 | 42,741 | 38,960 | 0,518 | (37,891; 40,030) | 3,781 | 1,51 | 1,55 |
| 21 | 39,143 | 39,058 | 0,506 | (38,013; 40,102) | 0,086 | 0,03 | 0,03 |
| 22 | 45,186 | 39,499 | 0,498 | (38,471; 40,526) | 5,687 | 2,27 | 2,50 |
| 23 | 38,661 | 36,580 | 1,316 | (33,864; 39,296) | 2,082 | 0,95 | 0,95 |
| 24 | 40,884 | 42,116 | 1,335 | (39,362; 44,871) | -1,233 | -0,57 | -0,56 |
| 25 | 37,155 | 39,252 | 0,493 | (38,234; 40,270) | -2,097 | -0,84 | -0,83 |
| 26 | 37,450 | 39,252 | 0,493 | (38,234; 40,270) | -1,802 | -0,72 | -0,71 |
| 27 | 42,998 | 39,252 | 0,493 | (38,234; 40,270) | 3,746 | 1,49 | 1,53 |

| Obs | HI | Cook’s D | DFITS |  |
| --- | --- | --- | --- | --- |
| 1 | 0,091956 | 0,01 | 0,194784 |  |
| 2 | 0,091956 | 0,01 | 0,185757 |  |
| 3 | 0,154060 | 0,07 | -0,470663 |  |
| 4 | 0,154060 | 0,22 | -0,853745 |  |
| 5 | 0,084910 | 0,03 | -0,279171 |  |
| 6 | 0,084910 | 0,02 | 0,232625 |  |
| 7 | 0,112397 | 0,01 | -0,145956 |  |
| 8 | 0,112397 | 0,07 | -0,474286 |  |
| 9 | 0,120141 | 0,02 | -0,217029 |  |
| 10 | 0,120141 | 0,00 | -0,088583 |  |
| 11 | 0,089807 | 0,00 | 0,092224 |  |
| 12 | 0,089807 | 0,00 | -0,030633 |  |
| 13 | 0,143301 | 0,05 | -0,382340 |  |
| 14 | 0,143301 | 0,03 | 0,278363 |  |
| 15 | 0,096233 | 0,02 | 0,263824 |  |
| 16 | 0,096233 | 0,09 | -0,542135 |  |
| 17 | 0,203866 | 0,00 | 0,009850 |  |
| 18 | 0,203866 | 0,02 | 0,239555 |  |
| 19 | 0,039415 | 0,00 | 0,098320 |  |
| 20 | 0,041065 | 0,03 | 0,321598 |  |
| 21 | 0,039207 | 0,00 | 0,006780 |  |
| 22 | 0,037944 | 0,07 | 0,497425 | R |
| 23 | 0,264924 | 0,11 | 0,568926 |  |
| 24 | 0,272505 | 0,04 | -0,341033 |  |
| 25 | 0,037200 | 0,01 | -0,163260 |  |
| 26 | 0,037200 | 0,01 | -0,139762 |  |
| 27 | 0,037200 | 0,03 | 0,301701 |  |

R  Large residual

## Forward Selection of Terms

α to enter = 0,25

## Coded Coefficients

| Term | Coef | SE Coef | 95% CI | T-Value | P-Value | VIF |
| --- | --- | --- | --- | --- | --- | --- |
| Constant | 42,172 | 0,496 | (41,148; 43,196) | 85,01 | 0,000 |  |
| Lac | 6,97 | 1,05 | (4,80; 9,14) | 6,62 | 0,000 | 1,00 |
| HPMC\_PS | 2,82 | 1,24 | (0,26; 5,38) | 2,27 | 0,032 | 1,00 |

## Model Summary

| S | R-sq | R-sq(adj) | PRESS | R-sq(pred) | AICc | BIC |
| --- | --- | --- | --- | --- | --- | --- |
| 2,57722 | 67,14% | 64,40% | 196,175 | 59,56% | 134,38 | 137,75 |

## Analysis of Variance

| Source | DF | Seq SS | Contribution | Adj SS | Adj MS | F-Value | P-Value |
| --- | --- | --- | --- | --- | --- | --- | --- |
| Model | 2 | 325,64 | 67,14% | 325,64 | 162,821 | 24,51 | 0,000 |
| Linear | 2 | 325,64 | 67,14% | 325,64 | 162,821 | 24,51 | 0,000 |
| Lac | 1 | 291,37 | 60,07% | 291,37 | 291,368 | 43,87 | 0,000 |
| HPMC\_PS | 1 | 34,27 | 7,07% | 34,27 | 34,273 | 5,16 | 0,032 |
| Error | 24 | 159,41 | 32,86% | 159,41 | 6,642 |  |  |
| Lack-of-Fit | 22 | 138,21 | 28,49% | 138,21 | 6,282 | 0,59 | 0,792 |
| Pure Error | 2 | 21,20 | 4,37% | 21,20 | 10,600 |  |  |
| Total | 26 | 485,05 | 100,00% |  |  |  |  |

## Regression Equation in Uncoded Units

|  |  |  |
| --- | --- | --- |
| F\_mean\_4h(240min) | = | 1,5 + 27,87 Lac + 0,383 HPMC\_PS |

## Fits and Diagnostics for All Observations

| Obs | F\_mean\_4h(240min) | Fit | SE Fit | 95% CI | Resid | Std Resid | Del Resid |
| --- | --- | --- | --- | --- | --- | --- | --- |
| 1 | 39,017 | 37,990 | 0,782 | (36,377; 39,603) | 1,026 | 0,42 | 0,41 |
| 2 | 46,450 | 44,959 | 0,782 | (43,346; 46,572) | 1,491 | 0,61 | 0,60 |
| 3 | 34,367 | 37,057 | 1,012 | (34,969; 39,145) | -2,690 | -1,13 | -1,14 |
| 4 | 39,535 | 44,026 | 1,012 | (41,938; 46,114) | -4,491 | -1,89 | -2,01 |
| 5 | 36,008 | 38,203 | 0,751 | (36,653; 39,753) | -2,195 | -0,89 | -0,89 |
| 6 | 46,980 | 45,172 | 0,751 | (43,622; 46,722) | 1,808 | 0,73 | 0,73 |
| 7 | 36,677 | 37,590 | 0,864 | (35,806; 39,373) | -0,912 | -0,38 | -0,37 |
| 8 | 41,293 | 44,558 | 0,864 | (42,775; 46,342) | -3,266 | -1,34 | -1,37 |
| 9 | 38,416 | 39,856 | 0,893 | (38,012; 41,700) | -1,440 | -0,60 | -0,59 |
| 10 | 46,211 | 46,825 | 0,893 | (44,981; 48,668) | -0,613 | -0,25 | -0,25 |
| 11 | 40,034 | 39,281 | 0,772 | (37,687; 40,875) | 0,753 | 0,31 | 0,30 |
| 12 | 45,897 | 46,250 | 0,772 | (44,656; 47,844) | -0,353 | -0,14 | -0,14 |
| 13 | 38,009 | 40,152 | 0,976 | (38,139; 42,166) | -2,143 | -0,90 | -0,89 |
| 14 | 48,773 | 47,121 | 0,976 | (45,107; 49,134) | 1,652 | 0,69 | 0,68 |
| 15 | 41,448 | 39,439 | 0,799 | (37,789; 41,089) | 2,008 | 0,82 | 0,81 |
| 16 | 42,454 | 46,408 | 0,799 | (44,758; 48,058) | -3,954 | -1,61 | -1,67 |
| 17 | 35,187 | 35,105 | 1,164 | (32,704; 37,507) | 0,081 | 0,04 | 0,03 |
| 18 | 50,190 | 49,043 | 1,164 | (46,641; 51,444) | 1,148 | 0,50 | 0,49 |
| 19 | 43,871 | 42,434 | 0,512 | (41,378; 43,490) | 1,437 | 0,57 | 0,56 |
| 20 | 45,413 | 41,777 | 0,522 | (40,699; 42,855) | 3,636 | 1,44 | 1,48 |
| 21 | 41,861 | 41,876 | 0,510 | (40,823; 42,929) | -0,015 | -0,01 | -0,01 |
| 22 | 48,167 | 42,325 | 0,502 | (41,289; 43,361) | 5,842 | 2,31 | 2,57 |
| 23 | 41,719 | 39,354 | 1,327 | (36,616; 42,092) | 2,365 | 1,07 | 1,07 |
| 24 | 43,771 | 44,989 | 1,345 | (42,213; 47,766) | -1,218 | -0,55 | -0,55 |
| 25 | 40,004 | 42,074 | 0,497 | (41,048; 43,100) | -2,070 | -0,82 | -0,81 |
| 26 | 40,421 | 42,074 | 0,497 | (41,048; 43,100) | -1,653 | -0,65 | -0,65 |
| 27 | 45,840 | 42,074 | 0,497 | (41,048; 43,100) | 3,766 | 1,49 | 1,53 |

| Obs | HI | Cook’s D | DFITS |  |
| --- | --- | --- | --- | --- |
| 1 | 0,091956 | 0,01 | 0,130687 |  |
| 2 | 0,091956 | 0,01 | 0,190608 |  |
| 3 | 0,154060 | 0,08 | -0,487412 |  |
| 4 | 0,154060 | 0,22 | -0,858199 |  |
| 5 | 0,084910 | 0,02 | -0,269984 |  |
| 6 | 0,084910 | 0,02 | 0,221184 |  |
| 7 | 0,112397 | 0,01 | -0,131272 |  |
| 8 | 0,112397 | 0,08 | -0,487236 |  |
| 9 | 0,120141 | 0,02 | -0,217052 |  |
| 10 | 0,120141 | 0,00 | -0,091913 |  |
| 11 | 0,089807 | 0,00 | 0,094298 |  |
| 12 | 0,089807 | 0,00 | -0,044179 |  |
| 13 | 0,143301 | 0,04 | -0,365890 |  |
| 14 | 0,143301 | 0,03 | 0,280127 |  |
| 15 | 0,096233 | 0,02 | 0,265585 |  |
| 16 | 0,096233 | 0,09 | -0,546035 |  |
| 17 | 0,203866 | 0,00 | 0,017536 |  |
| 18 | 0,203866 | 0,02 | 0,248539 |  |
| 19 | 0,039415 | 0,00 | 0,113608 |  |
| 20 | 0,041065 | 0,03 | 0,305345 |  |
| 21 | 0,039207 | 0,00 | -0,001168 |  |
| 22 | 0,037944 | 0,07 | 0,509535 | R |
| 23 | 0,264924 | 0,14 | 0,644663 |  |
| 24 | 0,272505 | 0,04 | -0,334211 |  |
| 25 | 0,037200 | 0,01 | -0,159770 |  |
| 26 | 0,037200 | 0,01 | -0,126932 |  |
| 27 | 0,037200 | 0,03 | 0,300798 |  |

R  Large residual

## Forward Selection of Terms

α to enter = 0,25

## Coded Coefficients

| Term | Coef | SE Coef | 95% CI | T-Value | P-Value | VIF |
| --- | --- | --- | --- | --- | --- | --- |
| Constant | 44,408 | 0,713 | (42,911; 45,905) | 62,32 | 0,000 |  |
| Lac | 6,728 | 0,906 | (4,825; 8,631) | 7,43 | 0,000 | 1,08 |
| HPMC\_Visc | -0,006 | 0,936 | (-1,972; 1,960) | -0,01 | 0,995 | 1,51 |
| HPMC\_HP | 1,776 | 0,926 | (-0,169; 3,722) | 1,92 | 0,071 | 1,14 |
| HPMC\_PS | 4,03 | 1,32 | (1,25; 6,81) | 3,04 | 0,007 | 1,66 |
| HPMC\_Visc\*HPMC\_Visc | 2,32 | 1,73 | (-1,32; 5,95) | 1,34 | 0,197 | 1,45 |
| HPMC\_HP\*HPMC\_HP | 2,48 | 1,57 | (-0,83; 5,78) | 1,58 | 0,133 | 1,23 |
| Lac\*HPMC\_Visc | -4,02 | 1,77 | (-7,74; -0,30) | -2,27 | 0,036 | 1,08 |
| HPMC\_Visc\*HPMC\_PS | 8,09 | 2,90 | (2,00; 14,18) | 2,79 | 0,012 | 1,87 |

## Model Summary

| S | R-sq | R-sq(adj) | PRESS | R-sq(pred) | AICc | BIC |
| --- | --- | --- | --- | --- | --- | --- |
| 2,13841 | 84,09% | 77,02% | 287,976 | 44,33% | 140,47 | 139,68 |

## Analysis of Variance

| Source | DF | Seq SS | Contribution | Adj SS | Adj MS | F-Value | P-Value |
| --- | --- | --- | --- | --- | --- | --- | --- |
| Model | 8 | 435,024 | 84,09% | 435,024 | 54,378 | 11,89 | 0,000 |
| Linear | 4 | 372,481 | 72,00% | 330,155 | 82,539 | 18,05 | 0,000 |
| Lac | 1 | 317,551 | 61,38% | 252,374 | 252,374 | 55,19 | 0,000 |
| HPMC\_Visc | 1 | 23,408 | 4,52% | 0,000 | 0,000 | 0,00 | 0,995 |
| HPMC\_HP | 1 | 16,213 | 3,13% | 16,835 | 16,835 | 3,68 | 0,071 |
| HPMC\_PS | 1 | 15,308 | 2,96% | 42,321 | 42,321 | 9,25 | 0,007 |
| Square | 2 | 3,340 | 0,65% | 16,644 | 8,322 | 1,82 | 0,191 |
| HPMC\_Visc\*HPMC\_Visc | 1 | 0,256 | 0,05% | 8,200 | 8,200 | 1,79 | 0,197 |
| HPMC\_HP\*HPMC\_HP | 1 | 3,084 | 0,60% | 11,346 | 11,346 | 2,48 | 0,133 |
| 2-Way Interaction | 2 | 59,202 | 11,44% | 59,202 | 29,601 | 6,47 | 0,008 |
| Lac\*HPMC\_Visc | 1 | 23,549 | 4,55% | 23,549 | 23,549 | 5,15 | 0,036 |
| HPMC\_Visc\*HPMC\_PS | 1 | 35,653 | 6,89% | 35,653 | 35,653 | 7,80 | 0,012 |
| Error | 18 | 82,310 | 15,91% | 82,310 | 4,573 |  |  |
| Lack-of-Fit | 16 | 61,629 | 11,91% | 61,629 | 3,852 | 0,37 | 0,901 |
| Pure Error | 2 | 20,681 | 4,00% | 20,681 | 10,340 |  |  |
| Total | 26 | 517,334 | 100,00% |  |  |  |  |

## Regression Equation in Uncoded Units

|  |  |  |
| --- | --- | --- |
| F\_mean\_4.5h(270min) | = | 458 + 82,8 Lac - 0,02177 HPMC\_Visc - 43,8 HPMC\_HP - 3,27 HPMC\_PS + 0,000000 HPMC\_Visc\*HPMC\_Visc + 2,40 HPMC\_HP\*HPMC\_HP - 0,00413 Lac\*HPMC\_Visc + 0,000283 HPMC\_Visc\*HPMC\_PS |

## Fits and Diagnostics for All Observations

| Obs | F\_mean\_4.5h(270min) | Fit | SE Fit | 95% CI | Resid | Std Resid | Del Resid |
| --- | --- | --- | --- | --- | --- | --- | --- |
| 1 | 41,49 | 41,23 | 1,27 | (38,56; 43,90) | 0,26 | 0,15 | 0,15 |
| 2 | 49,33 | 50,82 | 1,27 | (48,15; 53,49) | -1,49 | -0,86 | -0,86 |
| 3 | 36,77 | 38,03 | 1,51 | (34,86; 41,21) | -1,26 | -0,83 | -0,82 |
| 4 | 42,25 | 41,99 | 1,51 | (38,81; 45,16) | 0,26 | 0,17 | 0,17 |
| 5 | 38,57 | 41,40 | 1,04 | (39,22; 43,57) | -2,83 | -1,51 | -1,57 |
| 6 | 49,79 | 50,74 | 1,04 | (48,56; 52,91) | -0,95 | -0,51 | -0,49 |
| 7 | 39,26 | 41,23 | 1,35 | (38,39; 44,06) | -1,97 | -1,19 | -1,20 |
| 8 | 44,01 | 45,10 | 1,35 | (42,27; 47,93) | -1,09 | -0,66 | -0,65 |
| 9 | 40,92 | 40,05 | 1,17 | (37,60; 42,50) | 0,87 | 0,48 | 0,47 |
| 10 | 49,10 | 49,56 | 1,17 | (47,11; 52,01) | -0,46 | -0,26 | -0,25 |
| 11 | 42,66 | 42,40 | 1,01 | (40,29; 44,51) | 0,26 | 0,14 | 0,13 |
| 12 | 48,64 | 48,36 | 1,01 | (46,25; 50,47) | 0,28 | 0,15 | 0,14 |
| 13 | 40,53 | 41,06 | 1,26 | (38,41; 43,71) | -0,53 | -0,31 | -0,30 |
| 14 | 51,64 | 50,61 | 1,26 | (47,96; 53,26) | 1,03 | 0,60 | 0,59 |
| 15 | 44,03 | 43,86 | 1,09 | (41,57; 46,15) | 0,17 | 0,09 | 0,09 |
| 16 | 45,28 | 49,45 | 1,09 | (47,16; 51,74) | -4,17 | -2,27 | -2,61 |
| 17 | 37,54 | 36,83 | 1,12 | (34,47; 39,19) | 0,71 | 0,39 | 0,38 |
| 18 | 53,28 | 51,80 | 1,12 | (49,44; 54,16) | 1,49 | 0,82 | 0,81 |
| 19 | 46,77 | 46,24 | 1,30 | (43,51; 48,96) | 0,54 | 0,31 | 0,31 |
| 20 | 48,03 | 44,79 | 1,67 | (41,28; 48,30) | 3,24 | 2,43 | 2,88 |
| 21 | 44,42 | 44,91 | 1,32 | (42,15; 47,68) | -0,49 | -0,29 | -0,28 |
| 22 | 50,99 | 48,86 | 1,89 | (44,90; 52,82) | 2,13 | 2,12 | 2,37 |
| 23 | 44,70 | 42,52 | 1,40 | (39,57; 45,47) | 2,18 | 1,35 | 1,38 |
| 24 | 46,53 | 46,19 | 1,23 | (43,60; 48,78) | 0,34 | 0,19 | 0,19 |
| 25 | 42,72 | 44,32 | 0,70 | (42,84; 45,79) | -1,59 | -0,79 | -0,78 |
| 26 | 43,19 | 44,32 | 0,70 | (42,84; 45,79) | -1,13 | -0,56 | -0,55 |
| 27 | 48,51 | 44,32 | 0,70 | (42,84; 45,79) | 4,20 | 2,08 | 2,32 |

| Obs | HI | Cook’s D | DFITS |  |
| --- | --- | --- | --- | --- |
| 1 | 0,353486 | 0,00 | 0,10844 |  |
| 2 | 0,353486 | 0,05 | -0,63398 |  |
| 3 | 0,499779 | 0,08 | -0,82411 |  |
| 4 | 0,499779 | 0,00 | 0,16556 |  |
| 5 | 0,234473 | 0,08 | -0,86915 |  |
| 6 | 0,234473 | 0,01 | -0,27388 |  |
| 7 | 0,397964 | 0,10 | -0,97655 |  |
| 8 | 0,397964 | 0,03 | -0,52518 |  |
| 9 | 0,297247 | 0,01 | 0,30808 |  |
| 10 | 0,297247 | 0,00 | -0,16362 |  |
| 11 | 0,221111 | 0,00 | 0,07141 |  |
| 12 | 0,221111 | 0,00 | 0,07722 |  |
| 13 | 0,347879 | 0,01 | -0,21859 |  |
| 14 | 0,347879 | 0,02 | 0,42921 |  |
| 15 | 0,260234 | 0,00 | 0,05457 |  |
| 16 | 0,260234 | 0,20 | -1,54782 | R |
| 17 | 0,276514 | 0,01 | 0,23407 |  |
| 18 | 0,276514 | 0,03 | 0,50064 |  |
| 19 | 0,366835 | 0,01 | 0,23336 |  |
| 20 | 0,610759 | 1,03 | 3,60406 | R |
| 21 | 0,379931 | 0,01 | -0,22178 |  |
| 22 | 0,777643 | 1,74 | 4,43452 | R |
| 23 | 0,431273 | 0,15 | 1,20385 |  |
| 24 | 0,332135 | 0,00 | 0,13375 |  |
| 25 | 0,108017 | 0,01 | -0,27122 |  |
| 26 | 0,108017 | 0,00 | -0,19009 |  |
| 27 | 0,108017 | 0,06 | 0,80600 | R |

R  Large residual

## Forward Selection of Terms

α to enter = 0,25

## Coded Coefficients

| Term | Coef | SE Coef | 95% CI | T-Value | P-Value | VIF |
| --- | --- | --- | --- | --- | --- | --- |
| Constant | 47,028 | 0,720 | (45,515; 48,540) | 65,32 | 0,000 |  |
| Lac | 7,016 | 0,915 | (5,093; 8,939) | 7,67 | 0,000 | 1,08 |
| HPMC\_Visc | -0,122 | 0,946 | (-2,109; 1,865) | -0,13 | 0,899 | 1,51 |
| HPMC\_HP | 1,868 | 0,936 | (-0,097; 3,834) | 2,00 | 0,061 | 1,14 |
| HPMC\_PS | 3,91 | 1,34 | (1,10; 6,72) | 2,92 | 0,009 | 1,66 |
| HPMC\_Visc\*HPMC\_Visc | 2,29 | 1,75 | (-1,38; 5,96) | 1,31 | 0,207 | 1,45 |
| HPMC\_HP\*HPMC\_HP | 2,43 | 1,59 | (-0,91; 5,77) | 1,53 | 0,143 | 1,23 |
| Lac\*HPMC\_Visc | -4,10 | 1,79 | (-7,86; -0,34) | -2,29 | 0,034 | 1,08 |
| HPMC\_Visc\*HPMC\_PS | 8,00 | 2,93 | (1,85; 14,15) | 2,73 | 0,014 | 1,87 |

## Model Summary

| S | R-sq | R-sq(adj) | PRESS | R-sq(pred) | AICc | BIC |
| --- | --- | --- | --- | --- | --- | --- |
| 2,16086 | 84,67% | 77,86% | 299,035 | 45,46% | 141,03 | 140,24 |

## Analysis of Variance

| Source | DF | Seq SS | Contribution | Adj SS | Adj MS | F-Value | P-Value |
| --- | --- | --- | --- | --- | --- | --- | --- |
| Model | 8 | 464,261 | 84,67% | 464,261 | 58,033 | 12,43 | 0,000 |
| Linear | 4 | 401,688 | 73,26% | 353,241 | 88,310 | 18,91 | 0,000 |
| Lac | 1 | 344,216 | 62,78% | 274,445 | 274,445 | 58,78 | 0,000 |
| HPMC\_Visc | 1 | 25,469 | 4,65% | 0,078 | 0,078 | 0,02 | 0,899 |
| HPMC\_HP | 1 | 18,040 | 3,29% | 18,623 | 18,623 | 3,99 | 0,061 |
| HPMC\_PS | 1 | 13,963 | 2,55% | 39,935 | 39,935 | 8,55 | 0,009 |
| Square | 2 | 3,183 | 0,58% | 16,118 | 8,059 | 1,73 | 0,206 |
| HPMC\_Visc\*HPMC\_Visc | 1 | 0,254 | 0,05% | 7,997 | 7,997 | 1,71 | 0,207 |
| HPMC\_HP\*HPMC\_HP | 1 | 2,929 | 0,53% | 10,934 | 10,934 | 2,34 | 0,143 |
| 2-Way Interaction | 2 | 59,390 | 10,83% | 59,390 | 29,695 | 6,36 | 0,008 |
| Lac\*HPMC\_Visc | 1 | 24,528 | 4,47% | 24,528 | 24,528 | 5,25 | 0,034 |
| HPMC\_Visc\*HPMC\_PS | 1 | 34,862 | 6,36% | 34,862 | 34,862 | 7,47 | 0,014 |
| Error | 18 | 84,048 | 15,33% | 84,048 | 4,669 |  |  |
| Lack-of-Fit | 16 | 63,783 | 11,63% | 63,783 | 3,986 | 0,39 | 0,890 |
| Pure Error | 2 | 20,265 | 3,70% | 20,265 | 10,132 |  |  |
| Total | 26 | 548,309 | 100,00% |  |  |  |  |

## Regression Equation in Uncoded Units

|  |  |  |
| --- | --- | --- |
| F\_mean\_5h(300min) | = | 453 + 85,1 Lac - 0,02149 HPMC\_Visc - 42,9 HPMC\_HP - 3,25 HPMC\_PS + 0,000000 HPMC\_Visc\*HPMC\_Visc + 2,36 HPMC\_HP\*HPMC\_HP - 0,00422 Lac\*HPMC\_Visc + 0,000280 HPMC\_Visc\*HPMC\_PS |

## Fits and Diagnostics for All Observations

| Obs | F\_mean\_5h(300min) | Fit | SE Fit | 95% CI | Resid | Std Resid | Del Resid | HI |
| --- | --- | --- | --- | --- | --- | --- | --- | --- |
| 1 | 43,97 | 43,66 | 1,28 | (40,96; 46,36) | 0,31 | 0,18 | 0,17 | 0,353486 |
| 2 | 52,11 | 53,60 | 1,28 | (50,90; 56,30) | -1,49 | -0,86 | -0,85 | 0,353486 |
| 3 | 39,10 | 40,45 | 1,53 | (37,24; 43,66) | -1,35 | -0,88 | -0,88 | 0,499779 |
| 4 | 44,90 | 44,64 | 1,53 | (41,43; 47,85) | 0,26 | 0,17 | 0,17 | 0,499779 |
| 5 | 40,99 | 43,94 | 1,05 | (41,74; 46,14) | -2,95 | -1,56 | -1,63 | 0,234473 |
| 6 | 52,48 | 53,62 | 1,05 | (51,42; 55,82) | -1,14 | -0,60 | -0,59 | 0,234473 |
| 7 | 41,89 | 43,74 | 1,36 | (40,88; 46,60) | -1,85 | -1,10 | -1,11 | 0,397964 |
| 8 | 46,67 | 47,84 | 1,36 | (44,98; 50,71) | -1,17 | -0,70 | -0,69 | 0,397964 |
| 9 | 43,32 | 42,46 | 1,18 | (39,98; 44,93) | 0,86 | 0,48 | 0,46 | 0,297247 |
| 10 | 51,86 | 52,32 | 1,18 | (49,84; 54,79) | -0,46 | -0,25 | -0,25 | 0,297247 |
| 11 | 45,16 | 44,78 | 1,02 | (42,64; 46,91) | 0,38 | 0,20 | 0,19 | 0,221111 |
| 12 | 51,24 | 51,01 | 1,02 | (48,88; 53,15) | 0,22 | 0,12 | 0,11 | 0,221111 |
| 13 | 43,03 | 43,58 | 1,27 | (40,90; 46,26) | -0,55 | -0,32 | -0,31 | 0,347879 |
| 14 | 54,48 | 53,47 | 1,27 | (50,79; 56,15) | 1,01 | 0,58 | 0,57 | 0,347879 |
| 15 | 46,29 | 46,29 | 1,10 | (43,97; 48,60) | 0,00 | 0,00 | 0,00 | 0,260234 |
| 16 | 48,02 | 52,15 | 1,10 | (49,83; 54,46) | -4,12 | -2,22 | -2,53 | 0,260234 |
| 17 | 39,83 | 39,17 | 1,14 | (36,78; 41,55) | 0,67 | 0,36 | 0,35 | 0,276514 |
| 18 | 56,28 | 54,74 | 1,14 | (52,35; 57,13) | 1,54 | 0,84 | 0,83 | 0,276514 |
| 19 | 49,59 | 48,93 | 1,31 | (46,18; 51,68) | 0,66 | 0,38 | 0,37 | 0,366835 |
| 20 | 50,54 | 47,27 | 1,69 | (43,73; 50,82) | 3,26 | 2,42 | 2,86 | 0,610759 |
| 21 | 46,88 | 47,43 | 1,33 | (44,63; 50,23) | -0,54 | -0,32 | -0,31 | 0,379931 |
| 22 | 53,74 | 51,53 | 1,91 | (47,53; 55,54) | 2,21 | 2,16 | 2,45 | 0,777643 |
| 23 | 47,57 | 45,25 | 1,42 | (42,27; 48,23) | 2,31 | 1,42 | 1,46 | 0,431273 |
| 24 | 49,16 | 48,73 | 1,25 | (46,11; 51,35) | 0,43 | 0,24 | 0,24 | 0,332135 |
| 25 | 45,31 | 46,95 | 0,71 | (45,46; 48,45) | -1,64 | -0,80 | -0,79 | 0,108017 |
| 26 | 45,93 | 46,95 | 0,71 | (45,46; 48,45) | -1,02 | -0,50 | -0,49 | 0,108017 |
| 27 | 51,11 | 46,95 | 0,71 | (45,46; 48,45) | 4,16 | 2,04 | 2,26 | 0,108017 |

| Obs | Cook’s D | DFITS |  |
| --- | --- | --- | --- |
| 1 | 0,00 | 0,12858 |  |
| 2 | 0,04 | -0,63001 |  |
| 3 | 0,09 | -0,87536 |  |
| 4 | 0,00 | 0,16808 |  |
| 5 | 0,08 | -0,90260 |  |
| 6 | 0,01 | -0,32800 |  |
| 7 | 0,09 | -0,90201 |  |
| 8 | 0,04 | -0,55798 |  |
| 9 | 0,01 | 0,30214 |  |
| 10 | 0,00 | -0,15967 |  |
| 11 | 0,00 | 0,10344 |  |
| 12 | 0,00 | 0,06080 |  |
| 13 | 0,01 | -0,22542 |  |
| 14 | 0,02 | 0,41401 |  |
| 15 | 0,00 | 0,00083 |  |
| 16 | 0,19 | -1,49955 | R |
| 17 | 0,01 | 0,21865 |  |
| 18 | 0,03 | 0,51214 |  |
| 19 | 0,01 | 0,28359 |  |
| 20 | 1,02 | 3,58785 | R |
| 21 | 0,01 | -0,24331 |  |
| 22 | 1,82 | 4,57489 | R |
| 23 | 0,17 | 1,27550 |  |
| 24 | 0,00 | 0,16807 |  |
| 25 | 0,01 | -0,27661 |  |
| 26 | 0,00 | -0,17062 |  |
| 27 | 0,06 | 0,78521 | R |

R  Large residual

## Forward Selection of Terms

α to enter = 0,25

## Coded Coefficients

| Term | Coef | SE Coef | 95% CI | T-Value | P-Value | VIF |
| --- | --- | --- | --- | --- | --- | --- |
| Constant | 49,564 | 0,721 | (48,048; 51,079) | 68,71 | 0,000 |  |
| Lac | 7,273 | 0,917 | (5,347; 9,200) | 7,93 | 0,000 | 1,08 |
| HPMC\_Visc | -0,241 | 0,947 | (-2,232; 1,749) | -0,25 | 0,802 | 1,51 |
| HPMC\_HP | 1,952 | 0,937 | (-0,017; 3,921) | 2,08 | 0,052 | 1,14 |
| HPMC\_PS | 3,82 | 1,34 | (1,01; 6,64) | 2,85 | 0,011 | 1,66 |
| HPMC\_Visc\*HPMC\_Visc | 2,24 | 1,75 | (-1,44; 5,92) | 1,28 | 0,217 | 1,45 |
| HPMC\_HP\*HPMC\_HP | 2,40 | 1,59 | (-0,94; 5,74) | 1,51 | 0,149 | 1,23 |
| Lac\*HPMC\_Visc | -4,17 | 1,79 | (-7,94; -0,40) | -2,33 | 0,032 | 1,08 |
| HPMC\_Visc\*HPMC\_PS | 8,02 | 2,93 | (1,85; 14,18) | 2,73 | 0,014 | 1,87 |

## Model Summary

| S | R-sq | R-sq(adj) | PRESS | R-sq(pred) | AICc | BIC |
| --- | --- | --- | --- | --- | --- | --- |
| 2,16476 | 85,39% | 78,90% | 303,549 | 47,42% | 141,13 | 140,34 |

## Analysis of Variance

| Source | DF | Seq SS | Contribution | Adj SS | Adj MS | F-Value | P-Value |
| --- | --- | --- | --- | --- | --- | --- | --- |
| Model | 8 | 492,996 | 85,39% | 492,996 | 61,625 | 13,15 | 0,000 |
| Linear | 4 | 429,565 | 74,40% | 375,584 | 93,896 | 20,04 | 0,000 |
| Lac | 1 | 368,864 | 63,89% | 294,947 | 294,947 | 62,94 | 0,000 |
| HPMC\_Visc | 1 | 28,152 | 4,88% | 0,304 | 0,304 | 0,06 | 0,802 |
| HPMC\_HP | 1 | 19,803 | 3,43% | 20,330 | 20,330 | 4,34 | 0,052 |
| HPMC\_PS | 1 | 12,746 | 2,21% | 38,101 | 38,101 | 8,13 | 0,011 |
| Square | 2 | 3,106 | 0,54% | 15,592 | 7,796 | 1,66 | 0,217 |
| HPMC\_Visc\*HPMC\_Visc | 1 | 0,339 | 0,06% | 7,657 | 7,657 | 1,63 | 0,217 |
| HPMC\_HP\*HPMC\_HP | 1 | 2,767 | 0,48% | 10,652 | 10,652 | 2,27 | 0,149 |
| 2-Way Interaction | 2 | 60,325 | 10,45% | 60,325 | 30,162 | 6,44 | 0,008 |
| Lac\*HPMC\_Visc | 1 | 25,339 | 4,39% | 25,339 | 25,339 | 5,41 | 0,032 |
| HPMC\_Visc\*HPMC\_PS | 1 | 34,986 | 6,06% | 34,986 | 34,986 | 7,47 | 0,014 |
| Error | 18 | 84,351 | 14,61% | 84,351 | 4,686 |  |  |
| Lack-of-Fit | 16 | 64,554 | 11,18% | 64,554 | 4,035 | 0,41 | 0,882 |
| Pure Error | 2 | 19,797 | 3,43% | 19,797 | 9,899 |  |  |
| Total | 26 | 577,347 | 100,00% |  |  |  |  |

## Regression Equation in Uncoded Units

|  |  |  |
| --- | --- | --- |
| F\_mean\_5.5h(330min) | = | 452 + 87,0 Lac - 0,02143 HPMC\_Visc - 42,2 HPMC\_HP - 3,27 HPMC\_PS + 0,000000 HPMC\_Visc\*HPMC\_Visc + 2,33 HPMC\_HP\*HPMC\_HP - 0,00429 Lac\*HPMC\_Visc + 0,000280 HPMC\_Visc\*HPMC\_PS |

## Fits and Diagnostics for All Observations

| Obs | F\_mean\_5.5h(330min) | Fit | SE Fit | 95% CI | Resid | Std Resid | Del Resid |
| --- | --- | --- | --- | --- | --- | --- | --- |
| 1 | 46,43 | 46,05 | 1,29 | (43,35; 48,76) | 0,37 | 0,22 | 0,21 |
| 2 | 54,76 | 56,30 | 1,29 | (53,59; 59,00) | -1,54 | -0,88 | -0,88 |
| 3 | 41,35 | 42,74 | 1,53 | (39,53; 45,96) | -1,39 | -0,91 | -0,90 |
| 4 | 47,45 | 47,14 | 1,53 | (43,93; 50,36) | 0,31 | 0,20 | 0,20 |
| 5 | 43,40 | 46,41 | 1,05 | (44,21; 48,62) | -3,01 | -1,59 | -1,67 |
| 6 | 55,11 | 56,39 | 1,05 | (54,19; 58,60) | -1,29 | -0,68 | -0,67 |
| 7 | 44,34 | 46,13 | 1,37 | (43,26; 48,99) | -1,79 | -1,06 | -1,07 |
| 8 | 49,21 | 50,44 | 1,37 | (47,57; 53,31) | -1,23 | -0,73 | -0,72 |
| 9 | 45,59 | 44,79 | 1,18 | (42,31; 47,27) | 0,80 | 0,44 | 0,43 |
| 10 | 54,54 | 54,95 | 1,18 | (52,47; 57,43) | -0,41 | -0,23 | -0,22 |
| 11 | 47,60 | 47,10 | 1,02 | (44,96; 49,24) | 0,50 | 0,26 | 0,26 |
| 12 | 53,70 | 53,58 | 1,02 | (51,44; 55,72) | 0,12 | 0,06 | 0,06 |
| 13 | 45,42 | 46,00 | 1,28 | (43,31; 48,68) | -0,58 | -0,33 | -0,32 |
| 14 | 57,19 | 56,19 | 1,28 | (53,51; 58,88) | 0,99 | 0,57 | 0,56 |
| 15 | 48,59 | 48,66 | 1,10 | (46,34; 50,98) | -0,06 | -0,03 | -0,03 |
| 16 | 50,74 | 54,75 | 1,10 | (52,43; 57,07) | -4,02 | -2,16 | -2,43 |
| 17 | 42,07 | 41,45 | 1,14 | (39,06; 43,84) | 0,62 | 0,34 | 0,33 |
| 18 | 59,14 | 57,56 | 1,14 | (55,17; 59,96) | 1,57 | 0,85 | 0,85 |
| 19 | 52,30 | 51,52 | 1,31 | (48,77; 54,28) | 0,78 | 0,45 | 0,44 |
| 20 | 52,87 | 49,64 | 1,69 | (46,08; 53,19) | 3,23 | 2,39 | 2,81 |
| 21 | 49,29 | 49,88 | 1,33 | (47,07; 52,68) | -0,59 | -0,34 | -0,34 |
| 22 | 56,38 | 54,13 | 1,91 | (50,12; 58,14) | 2,25 | 2,21 | 2,51 |
| 23 | 50,31 | 47,91 | 1,42 | (44,92; 50,89) | 2,40 | 1,47 | 1,53 |
| 24 | 51,67 | 51,19 | 1,25 | (48,56; 53,81) | 0,48 | 0,27 | 0,27 |
| 25 | 47,85 | 49,51 | 0,71 | (48,01; 51,00) | -1,66 | -0,81 | -0,80 |
| 26 | 48,52 | 49,51 | 0,71 | (48,01; 51,00) | -0,99 | -0,48 | -0,47 |
| 27 | 53,60 | 49,51 | 0,71 | (48,01; 51,00) | 4,10 | 2,00 | 2,21 |

| Obs | HI | Cook’s D | DFITS |  |
| --- | --- | --- | --- | --- |
| 1 | 0,353486 | 0,00 | 0,15492 |  |
| 2 | 0,353486 | 0,05 | -0,65018 |  |
| 3 | 0,499779 | 0,09 | -0,90096 |  |
| 4 | 0,499779 | 0,00 | 0,19917 |  |
| 5 | 0,234473 | 0,09 | -0,92354 |  |
| 6 | 0,234473 | 0,02 | -0,36967 |  |
| 7 | 0,397964 | 0,08 | -0,86796 |  |
| 8 | 0,397964 | 0,04 | -0,58818 |  |
| 9 | 0,297247 | 0,01 | 0,28150 |  |
| 10 | 0,297247 | 0,00 | -0,14292 |  |
| 11 | 0,221111 | 0,00 | 0,13630 |  |
| 12 | 0,221111 | 0,00 | 0,03270 |  |
| 13 | 0,347879 | 0,01 | -0,23604 |  |
| 14 | 0,347879 | 0,02 | 0,40637 |  |
| 15 | 0,260234 | 0,00 | -0,01986 |  |
| 16 | 0,260234 | 0,18 | -1,44361 | R |
| 17 | 0,276514 | 0,00 | 0,20330 |  |
| 18 | 0,276514 | 0,03 | 0,52417 |  |
| 19 | 0,366835 | 0,01 | 0,33706 |  |
| 20 | 0,610759 | 1,00 | 3,52568 | R |
| 21 | 0,379931 | 0,01 | -0,26318 |  |
| 22 | 0,777643 | 1,89 | 4,69280 | R |
| 23 | 0,431273 | 0,18 | 1,32942 |  |
| 24 | 0,332135 | 0,00 | 0,18744 |  |
| 25 | 0,108017 | 0,01 | -0,27929 |  |
| 26 | 0,108017 | 0,00 | -0,16451 |  |
| 27 | 0,108017 | 0,05 | 0,76857 | R |

R  Large residual

## Forward Selection of Terms

α to enter = 0,25

## Coded Coefficients

| Term | Coef | SE Coef | 95% CI | T-Value | P-Value | VIF |
| --- | --- | --- | --- | --- | --- | --- |
| Constant | 52,020 | 0,723 | (50,502; 53,539) | 71,98 | 0,000 |  |
| Lac | 7,507 | 0,919 | (5,577; 9,437) | 8,17 | 0,000 | 1,08 |
| HPMC\_Visc | -0,315 | 0,949 | (-2,309; 1,680) | -0,33 | 0,744 | 1,51 |
| HPMC\_HP | 2,035 | 0,939 | (0,062; 4,008) | 2,17 | 0,044 | 1,14 |
| HPMC\_PS | 3,79 | 1,34 | (0,97; 6,61) | 2,82 | 0,011 | 1,66 |
| HPMC\_Visc\*HPMC\_Visc | 2,27 | 1,75 | (-1,42; 5,95) | 1,29 | 0,213 | 1,45 |
| HPMC\_HP\*HPMC\_HP | 2,35 | 1,59 | (-1,00; 5,70) | 1,47 | 0,158 | 1,23 |
| Lac\*HPMC\_Visc | -4,19 | 1,80 | (-7,97; -0,42) | -2,33 | 0,031 | 1,08 |
| HPMC\_Visc\*HPMC\_PS | 8,16 | 2,94 | (1,98; 14,33) | 2,77 | 0,012 | 1,87 |

## Model Summary

| S | R-sq | R-sq(adj) | PRESS | R-sq(pred) | AICc | BIC |
| --- | --- | --- | --- | --- | --- | --- |
| 2,16893 | 86,00% | 79,78% | 306,996 | 49,25% | 141,23 | 140,44 |

## Analysis of Variance

| Source | DF | Seq SS | Contribution | Adj SS | Adj MS | F-Value | P-Value |
| --- | --- | --- | --- | --- | --- | --- | --- |
| Model | 8 | 520,255 | 86,00% | 520,255 | 65,032 | 13,82 | 0,000 |
| Linear | 4 | 455,624 | 75,32% | 397,506 | 99,376 | 21,12 | 0,000 |
| Lac | 1 | 391,437 | 64,71% | 314,185 | 314,185 | 66,79 | 0,000 |
| HPMC\_Visc | 1 | 30,450 | 5,03% | 0,517 | 0,517 | 0,11 | 0,744 |
| HPMC\_HP | 1 | 21,785 | 3,60% | 22,094 | 22,094 | 4,70 | 0,044 |
| HPMC\_PS | 1 | 11,952 | 1,98% | 37,477 | 37,477 | 7,97 | 0,011 |
| Square | 2 | 2,814 | 0,47% | 15,381 | 7,690 | 1,63 | 0,223 |
| HPMC\_Visc\*HPMC\_Visc | 1 | 0,365 | 0,06% | 7,852 | 7,852 | 1,67 | 0,213 |
| HPMC\_HP\*HPMC\_HP | 1 | 2,448 | 0,40% | 10,228 | 10,228 | 2,17 | 0,158 |
| 2-Way Interaction | 2 | 61,817 | 10,22% | 61,817 | 30,908 | 6,57 | 0,007 |
| Lac\*HPMC\_Visc | 1 | 25,597 | 4,23% | 25,597 | 25,597 | 5,44 | 0,031 |
| HPMC\_Visc\*HPMC\_PS | 1 | 36,220 | 5,99% | 36,220 | 36,220 | 7,70 | 0,012 |
| Error | 18 | 84,677 | 14,00% | 84,677 | 4,704 |  |  |
| Lack-of-Fit | 16 | 65,507 | 10,83% | 65,507 | 4,094 | 0,43 | 0,872 |
| Pure Error | 2 | 19,170 | 3,17% | 19,170 | 9,585 |  |  |
| Total | 26 | 604,932 | 100,00% |  |  |  |  |

## Regression Equation in Uncoded Units

|  |  |  |
| --- | --- | --- |
| F\_mean\_6h(360min) | = | 455 + 88,3 Lac - 0,02183 HPMC\_Visc - 41,2 HPMC\_HP - 3,34 HPMC\_PS + 0,000000 HPMC\_Visc\*HPMC\_Visc + 2,28 HPMC\_HP\*HPMC\_HP - 0,00431 Lac\*HPMC\_Visc + 0,000285 HPMC\_Visc\*HPMC\_PS |

## Fits and Diagnostics for All Observations

| Obs | F\_mean\_6h(360min) | Fit | SE Fit | 95% CI | Resid | Std Resid | Del Resid | HI |
| --- | --- | --- | --- | --- | --- | --- | --- | --- |
| 1 | 48,90 | 48,40 | 1,29 | (45,69; 51,11) | 0,49 | 0,28 | 0,28 | 0,353486 |
| 2 | 57,28 | 58,89 | 1,29 | (56,18; 61,60) | -1,62 | -0,93 | -0,92 | 0,353486 |
| 3 | 43,50 | 44,92 | 1,53 | (41,70; 48,15) | -1,42 | -0,93 | -0,92 | 0,499779 |
| 4 | 49,90 | 49,54 | 1,53 | (46,32; 52,76) | 0,36 | 0,23 | 0,23 | 0,499779 |
| 5 | 45,73 | 48,84 | 1,05 | (46,63; 51,05) | -3,11 | -1,64 | -1,73 | 0,234473 |
| 6 | 57,66 | 59,07 | 1,05 | (56,86; 61,28) | -1,41 | -0,74 | -0,73 | 0,234473 |
| 7 | 46,73 | 48,44 | 1,37 | (45,56; 51,31) | -1,71 | -1,02 | -1,02 | 0,397964 |
| 8 | 51,68 | 52,97 | 1,37 | (50,09; 55,84) | -1,29 | -0,76 | -0,75 | 0,397964 |
| 9 | 47,79 | 47,05 | 1,18 | (44,57; 49,54) | 0,73 | 0,40 | 0,39 | 0,297247 |
| 10 | 57,12 | 57,46 | 1,18 | (54,98; 59,95) | -0,35 | -0,19 | -0,18 | 0,297247 |
| 11 | 50,00 | 49,38 | 1,02 | (47,24; 51,52) | 0,62 | 0,32 | 0,31 | 0,221111 |
| 12 | 56,14 | 56,09 | 1,02 | (53,94; 58,23) | 0,05 | 0,03 | 0,03 | 0,221111 |
| 13 | 47,76 | 48,35 | 1,28 | (45,67; 51,04) | -0,60 | -0,34 | -0,33 | 0,347879 |
| 14 | 59,76 | 58,80 | 1,28 | (56,11; 61,49) | 0,97 | 0,55 | 0,54 | 0,347879 |
| 15 | 50,89 | 51,00 | 1,11 | (48,67; 53,32) | -0,11 | -0,06 | -0,05 | 0,260234 |
| 16 | 53,40 | 57,32 | 1,11 | (55,00; 59,65) | -3,92 | -2,10 | -2,35 | 0,260234 |
| 17 | 44,23 | 43,68 | 1,14 | (41,29; 46,08) | 0,55 | 0,30 | 0,29 | 0,276514 |
| 18 | 61,87 | 60,27 | 1,14 | (57,87; 62,67) | 1,60 | 0,87 | 0,86 | 0,276514 |
| 19 | 54,96 | 54,06 | 1,31 | (51,30; 56,82) | 0,90 | 0,52 | 0,51 | 0,366835 |
| 20 | 55,19 | 52,02 | 1,70 | (48,46; 55,58) | 3,17 | 2,35 | 2,74 | 0,610759 |
| 21 | 51,57 | 52,22 | 1,34 | (49,41; 55,03) | -0,66 | -0,38 | -0,37 | 0,379931 |
| 22 | 58,92 | 56,63 | 1,91 | (52,61; 60,65) | 2,29 | 2,24 | 2,56 | 0,777643 |
| 23 | 52,92 | 50,44 | 1,42 | (47,45; 53,44) | 2,48 | 1,52 | 1,58 | 0,431273 |
| 24 | 54,10 | 53,58 | 1,25 | (50,95; 56,20) | 0,52 | 0,29 | 0,28 | 0,332135 |
| 25 | 50,29 | 51,98 | 0,71 | (50,48; 53,48) | -1,69 | -0,83 | -0,82 | 0,108017 |
| 26 | 51,08 | 51,98 | 0,71 | (50,48; 53,48) | -0,89 | -0,44 | -0,43 | 0,108017 |
| 27 | 56,00 | 51,98 | 0,71 | (50,48; 53,48) | 4,02 | 1,96 | 2,15 | 0,108017 |

| Obs | Cook’s D | DFITS |  |
| --- | --- | --- | --- |
| 1 | 0,00 | 0,20436 |  |
| 2 | 0,05 | -0,68230 |  |
| 3 | 0,10 | -0,92216 |  |
| 4 | 0,01 | 0,22819 |  |
| 5 | 0,09 | -0,95557 |  |
| 6 | 0,02 | -0,40468 |  |
| 7 | 0,08 | -0,82749 |  |
| 8 | 0,04 | -0,61333 |  |
| 9 | 0,01 | 0,25629 |  |
| 10 | 0,00 | -0,12030 |  |
| 11 | 0,00 | 0,16718 |  |
| 12 | 0,00 | 0,01362 |  |
| 13 | 0,01 | -0,24201 |  |
| 14 | 0,02 | 0,39454 |  |
| 15 | 0,00 | -0,03254 |  |
| 16 | 0,17 | -1,39589 | R |
| 17 | 0,00 | 0,17972 |  |
| 18 | 0,03 | 0,53299 |  |
| 19 | 0,02 | 0,38985 |  |
| 20 | 0,96 | 3,42696 | R |
| 21 | 0,01 | -0,29336 |  |
| 22 | 1,95 | 4,79626 | R |
| 23 | 0,19 | 1,37301 |  |
| 24 | 0,00 | 0,20036 |  |
| 25 | 0,01 | -0,28487 |  |
| 26 | 0,00 | -0,14845 |  |
| 27 | 0,05 | 0,74958 |  |

R  Large residual

## Forward Selection of Terms

α to enter = 0,25

## Coded Coefficients

| Term | Coef | SE Coef | 95% CI | T-Value | P-Value | VIF |
| --- | --- | --- | --- | --- | --- | --- |
| Constant | 57,558 | 0,509 | (56,496; 58,619) | 113,06 | 0,000 |  |
| Lac | 7,985 | 0,940 | (6,024; 9,947) | 8,49 | 0,000 | 1,08 |
| HPMC\_Visc | -0,970 | 0,917 | (-2,882; 0,943) | -1,06 | 0,303 | 1,35 |
| HPMC\_HP | 1,858 | 0,910 | (-0,041; 3,757) | 2,04 | 0,055 | 1,03 |
| HPMC\_PS | 2,65 | 1,25 | (0,04; 5,26) | 2,11 | 0,047 | 1,37 |
| Lac\*HPMC\_Visc | -4,44 | 1,84 | (-8,28; -0,60) | -2,41 | 0,026 | 1,08 |
| HPMC\_Visc\*HPMC\_PS | 5,12 | 2,46 | (-0,02; 10,26) | 2,08 | 0,051 | 1,25 |

## Model Summary

| S | R-sq | R-sq(adj) | PRESS | R-sq(pred) | AICc | BIC |
| --- | --- | --- | --- | --- | --- | --- |
| 2,22067 | 85,11% | 80,64% | 168,760 | 74,52% | 135,60 | 137,97 |

## Analysis of Variance

| Source | DF | Seq SS | Contribution | Adj SS | Adj MS | F-Value | P-Value |
| --- | --- | --- | --- | --- | --- | --- | --- |
| Model | 6 | 563,780 | 85,11% | 563,780 | 93,963 | 19,05 | 0,000 |
| Linear | 4 | 513,762 | 77,56% | 429,072 | 107,268 | 21,75 | 0,000 |
| Lac | 1 | 442,639 | 66,82% | 355,518 | 355,518 | 72,09 | 0,000 |
| HPMC\_Visc | 1 | 32,892 | 4,97% | 5,518 | 5,518 | 1,12 | 0,303 |
| HPMC\_HP | 1 | 28,297 | 4,27% | 20,537 | 20,537 | 4,16 | 0,055 |
| HPMC\_PS | 1 | 9,934 | 1,50% | 22,045 | 22,045 | 4,47 | 0,047 |
| 2-Way Interaction | 2 | 50,018 | 7,55% | 50,018 | 25,009 | 5,07 | 0,017 |
| Lac\*HPMC\_Visc | 1 | 28,693 | 4,33% | 28,693 | 28,693 | 5,82 | 0,026 |
| HPMC\_Visc\*HPMC\_PS | 1 | 21,325 | 3,22% | 21,325 | 21,325 | 4,32 | 0,051 |
| Error | 20 | 98,628 | 14,89% | 98,628 | 4,931 |  |  |
| Lack-of-Fit | 18 | 80,091 | 12,09% | 80,091 | 4,449 | 0,48 | 0,846 |
| Pure Error | 2 | 18,537 | 2,80% | 18,537 | 9,268 |  |  |
| Total | 26 | 662,407 | 100,00% |  |  |  |  |

## Regression Equation in Uncoded Units

|  |  |  |
| --- | --- | --- |
| F\_mean\_7h(420min) | = | 140,1 + 93,6 Lac - 0,01043 HPMC\_Visc + 1,830 HPMC\_HP - 2,06 HPMC\_PS - 0,00456 Lac\*HPMC\_Visc + 0,000179 HPMC\_Visc\*HPMC\_PS |

## Fits and Diagnostics for All Observations

| Obs | F\_mean\_7h(420min) | Fit | SE Fit | 95% CI | Resid | Std Resid | Del Resid | HI |
| --- | --- | --- | --- | --- | --- | --- | --- | --- |
| 1 | 52,67 | 51,58 | 1,18 | (49,12; 54,05) | 1,09 | 0,58 | 0,57 | 0,283627 |
| 2 | 62,10 | 62,73 | 1,18 | (60,26; 65,19) | -0,62 | -0,33 | -0,32 | 0,283627 |
| 3 | 47,72 | 49,45 | 1,55 | (46,22; 52,69) | -1,74 | -1,09 | -1,10 | 0,488210 |
| 4 | 54,58 | 54,38 | 1,55 | (51,14; 57,62) | 0,20 | 0,13 | 0,13 | 0,488210 |
| 5 | 50,14 | 53,25 | 1,03 | (51,09; 55,41) | -3,11 | -1,58 | -1,65 | 0,216779 |
| 6 | 62,57 | 64,12 | 1,03 | (61,96; 66,27) | -1,55 | -0,79 | -0,78 | 0,216779 |
| 7 | 51,53 | 52,88 | 1,40 | (49,97; 55,79) | -1,36 | -0,79 | -0,78 | 0,394639 |
| 8 | 56,49 | 57,72 | 1,40 | (54,81; 60,63) | -1,22 | -0,71 | -0,70 | 0,394639 |
| 9 | 51,99 | 51,07 | 1,20 | (48,55; 53,58) | 0,93 | 0,50 | 0,49 | 0,294244 |
| 10 | 62,01 | 62,12 | 1,20 | (59,61; 64,64) | -0,11 | -0,06 | -0,06 | 0,294244 |
| 11 | 54,52 | 53,68 | 1,04 | (51,52; 55,85) | 0,84 | 0,43 | 0,42 | 0,218366 |
| 12 | 60,68 | 60,82 | 1,04 | (58,66; 62,99) | -0,14 | -0,07 | -0,07 | 0,218366 |
| 13 | 52,28 | 52,96 | 1,31 | (50,23; 55,69) | -0,68 | -0,38 | -0,37 | 0,347162 |
| 14 | 64,73 | 64,06 | 1,31 | (61,33; 66,78) | 0,68 | 0,38 | 0,37 | 0,347162 |
| 15 | 54,73 | 55,33 | 1,13 | (52,98; 57,68) | -0,60 | -0,31 | -0,31 | 0,258117 |
| 16 | 58,35 | 62,06 | 1,13 | (59,71; 64,42) | -3,71 | -1,94 | -2,10 | 0,258117 |
| 17 | 48,46 | 48,76 | 1,03 | (46,62; 50,90) | -0,30 | -0,15 | -0,15 | 0,213198 |
| 18 | 67,03 | 66,40 | 1,03 | (64,26; 68,54) | 0,63 | 0,32 | 0,32 | 0,213198 |
| 19 | 59,91 | 58,16 | 0,88 | (56,32; 60,00) | 1,75 | 0,86 | 0,85 | 0,157071 |
| 20 | 59,53 | 55,18 | 1,03 | (53,03; 57,32) | 4,35 | 2,21 | 2,48 | 0,214445 |
| 21 | 55,96 | 55,69 | 0,92 | (53,78; 57,61) | 0,27 | 0,13 | 0,13 | 0,170686 |
| 22 | 63,83 | 59,64 | 1,11 | (57,32; 61,96) | 4,19 | 2,18 | 2,44 | 0,251394 |
| 23 | 57,98 | 56,35 | 1,31 | (53,63; 59,08) | 1,63 | 0,91 | 0,90 | 0,346058 |
| 24 | 58,69 | 58,77 | 1,21 | (56,24; 61,29) | -0,07 | -0,04 | -0,04 | 0,297564 |
| 25 | 54,89 | 57,58 | 0,47 | (56,60; 58,56) | -2,69 | -1,24 | -1,26 | 0,044701 |
| 26 | 55,90 | 57,58 | 0,47 | (56,60; 58,56) | -1,68 | -0,77 | -0,77 | 0,044701 |
| 27 | 60,60 | 57,58 | 0,47 | (56,60; 58,56) | 3,02 | 1,39 | 1,43 | 0,044701 |

| Obs | Cook’s D | DFITS |  |
| --- | --- | --- | --- |
| 1 | 0,02 | 0,35851 |  |
| 2 | 0,01 | -0,20432 |  |
| 3 | 0,16 | -1,07394 |  |
| 4 | 0,00 | 0,12239 |  |
| 5 | 0,10 | -0,86718 |  |
| 6 | 0,02 | -0,41102 |  |
| 7 | 0,06 | -0,62834 |  |
| 8 | 0,05 | -0,56509 |  |
| 9 | 0,01 | 0,31555 |  |
| 10 | 0,00 | -0,03789 |  |
| 11 | 0,01 | 0,22093 |  |
| 12 | 0,00 | -0,03793 |  |
| 13 | 0,01 | -0,26854 |  |
| 14 | 0,01 | 0,26843 |  |
| 15 | 0,00 | -0,18017 |  |
| 16 | 0,19 | -1,23776 |  |
| 17 | 0,00 | -0,07694 |  |
| 18 | 0,00 | 0,16398 |  |
| 19 | 0,02 | 0,36891 |  |
| 20 | 0,19 | 1,29421 | R |
| 21 | 0,00 | 0,05826 |  |
| 22 | 0,23 | 1,41176 | R |
| 23 | 0,06 | 0,65801 |  |
| 24 | 0,00 | -0,02455 |  |
| 25 | 0,01 | -0,27175 |  |
| 26 | 0,00 | -0,16549 |  |
| 27 | 0,01 | 0,30834 |  |

R  Large residual

## Forward Selection of Terms

α to enter = 0,25

## Coded Coefficients

| Term | Coef | SE Coef | 95% CI | T-Value | P-Value | VIF |
| --- | --- | --- | --- | --- | --- | --- |
| Constant | 61,934 | 0,510 | (60,870; 62,997) | 121,48 | 0,000 |  |
| Lac | 8,328 | 0,942 | (6,364; 10,293) | 8,84 | 0,000 | 1,08 |
| HPMC\_Visc | -1,110 | 0,918 | (-3,026; 0,805) | -1,21 | 0,241 | 1,35 |
| HPMC\_HP | 2,047 | 0,912 | (0,146; 3,949) | 2,25 | 0,036 | 1,03 |
| HPMC\_PS | 2,58 | 1,25 | (-0,04; 5,19) | 2,05 | 0,053 | 1,37 |
| Lac\*HPMC\_Visc | -4,45 | 1,84 | (-8,30; -0,61) | -2,42 | 0,025 | 1,08 |
| HPMC\_Visc\*HPMC\_PS | 5,34 | 2,47 | (0,19; 10,48) | 2,16 | 0,043 | 1,25 |

## Model Summary

| S | R-sq | R-sq(adj) | PRESS | R-sq(pred) | AICc | BIC |
| --- | --- | --- | --- | --- | --- | --- |
| 2,22382 | 86,05% | 81,87% | 170,263 | 75,99% | 135,68 | 138,04 |

## Analysis of Variance

| Source | DF | Seq SS | Contribution | Adj SS | Adj MS | F-Value | P-Value |
| --- | --- | --- | --- | --- | --- | --- | --- |
| Model | 6 | 610,126 | 86,05% | 610,126 | 101,688 | 20,56 | 0,000 |
| Linear | 4 | 558,040 | 78,70% | 466,922 | 116,731 | 23,60 | 0,000 |
| Lac | 1 | 478,903 | 67,54% | 386,681 | 386,681 | 78,19 | 0,000 |
| HPMC\_Visc | 1 | 37,307 | 5,26% | 7,234 | 7,234 | 1,46 | 0,241 |
| HPMC\_HP | 1 | 33,265 | 4,69% | 24,945 | 24,945 | 5,04 | 0,036 |
| HPMC\_PS | 1 | 8,565 | 1,21% | 20,827 | 20,827 | 4,21 | 0,053 |
| 2-Way Interaction | 2 | 52,086 | 7,35% | 52,086 | 26,043 | 5,27 | 0,015 |
| Lac\*HPMC\_Visc | 1 | 28,910 | 4,08% | 28,910 | 28,910 | 5,85 | 0,025 |
| HPMC\_Visc\*HPMC\_PS | 1 | 23,176 | 3,27% | 23,176 | 23,176 | 4,69 | 0,043 |
| Error | 20 | 98,907 | 13,95% | 98,907 | 4,945 |  |  |
| Lack-of-Fit | 18 | 81,689 | 11,52% | 81,689 | 4,538 | 0,53 | 0,821 |
| Pure Error | 2 | 17,218 | 2,43% | 17,218 | 8,609 |  |  |
| Total | 26 | 709,033 | 100,00% |  |  |  |  |

## Regression Equation in Uncoded Units

|  |  |  |
| --- | --- | --- |
| F\_mean\_8h(480min) | = | 150,3 + 95,2 Lac - 0,01099 HPMC\_Visc + 2,017 HPMC\_HP - 2,17 HPMC\_PS - 0,00458 Lac\*HPMC\_Visc + 0,000187 HPMC\_Visc\*HPMC\_PS |

## Fits and Diagnostics for All Observations

| Obs | F\_mean\_8h(480min) | Fit | SE Fit | 95% CI | Resid | Std Resid | Del Resid | HI |
| --- | --- | --- | --- | --- | --- | --- | --- | --- |
| 1 | 56,98 | 55,80 | 1,18 | (53,33; 58,27) | 1,17 | 0,62 | 0,61 | 0,283627 |
| 2 | 66,57 | 67,30 | 1,18 | (64,83; 69,77) | -0,73 | -0,39 | -0,38 | 0,283627 |
| 3 | 51,65 | 53,38 | 1,55 | (50,14; 56,62) | -1,74 | -1,09 | -1,10 | 0,488210 |
| 4 | 58,93 | 58,64 | 1,55 | (55,40; 61,88) | 0,29 | 0,18 | 0,18 | 0,488210 |
| 5 | 54,34 | 57,62 | 1,04 | (55,46; 59,78) | -3,27 | -1,66 | -1,75 | 0,216779 |
| 6 | 67,11 | 68,84 | 1,04 | (66,68; 71,00) | -1,72 | -0,88 | -0,87 | 0,216779 |
| 7 | 55,61 | 57,05 | 1,40 | (54,14; 59,97) | -1,45 | -0,84 | -0,83 | 0,394639 |
| 8 | 61,03 | 62,22 | 1,40 | (59,30; 65,13) | -1,18 | -0,68 | -0,68 | 0,394639 |
| 9 | 55,84 | 55,14 | 1,21 | (52,62; 57,66) | 0,70 | 0,38 | 0,37 | 0,294244 |
| 10 | 66,54 | 66,55 | 1,21 | (64,03; 69,07) | -0,01 | -0,00 | -0,00 | 0,294244 |
| 11 | 58,96 | 57,77 | 1,04 | (55,60; 59,93) | 1,19 | 0,61 | 0,60 | 0,218366 |
| 12 | 64,88 | 65,24 | 1,04 | (63,08; 67,41) | -0,36 | -0,18 | -0,18 | 0,218366 |
| 13 | 56,55 | 57,22 | 1,31 | (54,48; 59,95) | -0,66 | -0,37 | -0,36 | 0,347162 |
| 14 | 69,33 | 68,67 | 1,31 | (65,93; 71,40) | 0,66 | 0,37 | 0,36 | 0,347162 |
| 15 | 58,88 | 59,53 | 1,13 | (57,17; 61,88) | -0,65 | -0,34 | -0,33 | 0,258117 |
| 16 | 62,99 | 66,60 | 1,13 | (64,24; 68,95) | -3,61 | -1,88 | -2,02 | 0,258117 |
| 17 | 52,52 | 52,81 | 1,03 | (50,67; 54,95) | -0,29 | -0,15 | -0,14 | 0,213198 |
| 18 | 71,83 | 71,14 | 1,03 | (69,00; 73,28) | 0,69 | 0,35 | 0,34 | 0,213198 |
| 19 | 64,58 | 62,64 | 0,88 | (60,80; 64,47) | 1,94 | 0,95 | 0,95 | 0,157071 |
| 20 | 63,57 | 59,36 | 1,03 | (57,21; 61,51) | 4,21 | 2,14 | 2,37 | 0,214445 |
| 21 | 60,02 | 59,92 | 0,92 | (58,00; 61,83) | 0,10 | 0,05 | 0,05 | 0,170686 |
| 22 | 68,38 | 64,22 | 1,12 | (61,89; 66,54) | 4,16 | 2,16 | 2,41 | 0,251394 |
| 23 | 62,68 | 60,88 | 1,31 | (58,15; 63,61) | 1,80 | 1,00 | 1,00 | 0,346058 |
| 24 | 63,03 | 63,01 | 1,21 | (60,48; 65,55) | 0,02 | 0,01 | 0,01 | 0,297564 |
| 25 | 59,29 | 61,98 | 0,47 | (61,00; 62,96) | -2,69 | -1,24 | -1,26 | 0,044701 |
| 26 | 60,51 | 61,98 | 0,47 | (61,00; 62,96) | -1,46 | -0,67 | -0,66 | 0,044701 |
| 27 | 64,87 | 61,98 | 0,47 | (61,00; 62,96) | 2,89 | 1,33 | 1,36 | 0,044701 |

| Obs | Cook’s D | DFITS |  |
| --- | --- | --- | --- |
| 1 | 0,02 | 0,38616 |  |
| 2 | 0,01 | -0,24023 |  |
| 3 | 0,16 | -1,07143 |  |
| 4 | 0,00 | 0,17361 |  |
| 5 | 0,11 | -0,91870 |  |
| 6 | 0,03 | -0,45833 |  |
| 7 | 0,07 | -0,67072 |  |
| 8 | 0,04 | -0,54505 |  |
| 9 | 0,01 | 0,23777 |  |
| 10 | 0,00 | -0,00314 |  |
| 11 | 0,01 | 0,31534 |  |
| 12 | 0,00 | -0,09475 |  |
| 13 | 0,01 | -0,26364 |  |
| 14 | 0,01 | 0,26384 |  |
| 15 | 0,01 | -0,19525 |  |
| 16 | 0,18 | -1,19336 |  |
| 17 | 0,00 | -0,07448 |  |
| 18 | 0,00 | 0,17873 |  |
| 19 | 0,02 | 0,40906 |  |
| 20 | 0,18 | 1,23736 | R |
| 21 | 0,00 | 0,02154 |  |
| 22 | 0,22 | 1,39601 | R |
| 23 | 0,08 | 0,72682 |  |
| 24 | 0,00 | 0,00658 |  |
| 25 | 0,01 | -0,27161 |  |
| 26 | 0,00 | -0,14351 |  |
| 27 | 0,01 | 0,29394 |  |

R  Large residual

## Forward Selection of Terms

α to enter = 0,25

## Coded Coefficients

| Term | Coef | SE Coef | 95% CI | T-Value | P-Value | VIF |
| --- | --- | --- | --- | --- | --- | --- |
| Constant | 65,533 | 0,507 | (64,481; 66,584) | 129,28 | 0,000 |  |
| Lac | 8,67 | 1,05 | (6,49; 10,85) | 8,25 | 0,000 | 1,08 |
| HPMC\_Visc | -2,394 | 0,884 | (-4,227; -0,560) | -2,71 | 0,013 | 1,00 |
| HPMC\_HP | 2,44 | 1,00 | (0,35; 4,52) | 2,43 | 0,024 | 1,00 |
| Lac\*HPMC\_Visc | -4,33 | 2,06 | (-8,60; -0,07) | -2,11 | 0,047 | 1,08 |

## Model Summary

| S | R-sq | R-sq(adj) | PRESS | R-sq(pred) | AICc | BIC |
| --- | --- | --- | --- | --- | --- | --- |
| 2,48110 | 82,07% | 78,81% | 225,600 | 70,13% | 136,36 | 139,94 |

## Analysis of Variance

| Source | DF | Seq SS | Contribution | Adj SS | Adj MS | F-Value | P-Value |
| --- | --- | --- | --- | --- | --- | --- | --- |
| Model | 4 | 619,75 | 82,07% | 619,75 | 154,938 | 25,17 | 0,000 |
| Linear | 3 | 592,41 | 78,45% | 497,07 | 165,692 | 26,92 | 0,000 |
| Lac | 1 | 514,46 | 68,12% | 419,13 | 419,127 | 68,09 | 0,000 |
| HPMC\_Visc | 1 | 41,70 | 5,52% | 45,14 | 45,137 | 7,33 | 0,013 |
| HPMC\_HP | 1 | 36,25 | 4,80% | 36,25 | 36,251 | 5,89 | 0,024 |
| 2-Way Interaction | 1 | 27,34 | 3,62% | 27,34 | 27,345 | 4,44 | 0,047 |
| Lac\*HPMC\_Visc | 1 | 27,34 | 3,62% | 27,34 | 27,345 | 4,44 | 0,047 |
| Error | 22 | 135,43 | 17,93% | 135,43 | 6,156 |  |  |
| Lack-of-Fit | 20 | 118,47 | 15,69% | 118,47 | 5,924 | 0,70 | 0,738 |
| Pure Error | 2 | 16,96 | 2,25% | 16,96 | 8,479 |  |  |
| Total | 26 | 755,18 | 100,00% |  |  |  |  |

## Regression Equation in Uncoded Units

|  |  |  |
| --- | --- | --- |
| F\_mean\_9h(540min) | = | 3,6 + 94,9 Lac + 0,00161 HPMC\_Visc + 2,403 HPMC\_HP - 0,00445 Lac\*HPMC\_Visc |

## Fits and Diagnostics for All Observations

| Obs | F\_mean\_9h(540min) | Fit | SE Fit | 95% CI | Resid | Std Resid | Del Resid |
| --- | --- | --- | --- | --- | --- | --- | --- |
| 1 | 61,817 | 59,601 | 1,194 | (57,126; 62,077) | 2,216 | 1,02 | 1,02 |
| 2 | 70,693 | 71,356 | 1,194 | (68,880; 73,832) | -0,663 | -0,30 | -0,30 |
| 3 | 55,315 | 59,207 | 1,470 | (56,158; 62,256) | -3,892 | -1,95 | -2,09 |
| 4 | 63,033 | 64,891 | 1,470 | (61,842; 67,939) | -1,857 | -0,93 | -0,93 |
| 5 | 58,314 | 61,846 | 1,049 | (59,671; 64,021) | -3,532 | -1,57 | -1,63 |
| 6 | 71,253 | 73,329 | 1,049 | (71,154; 75,504) | -2,076 | -0,92 | -0,92 |
| 7 | 59,803 | 62,193 | 1,457 | (59,171; 65,214) | -2,390 | -1,19 | -1,20 |
| 8 | 65,380 | 67,786 | 1,457 | (64,764; 70,807) | -2,406 | -1,20 | -1,21 |
| 9 | 59,432 | 59,696 | 1,157 | (57,296; 62,097) | -0,265 | -0,12 | -0,12 |
| 10 | 70,737 | 71,366 | 1,157 | (68,965; 73,766) | -0,628 | -0,29 | -0,28 |
| 11 | 63,159 | 59,993 | 0,899 | (58,129; 61,857) | 3,165 | 1,37 | 1,40 |
| 12 | 68,853 | 67,838 | 0,899 | (65,974; 69,702) | 1,015 | 0,44 | 0,43 |
| 13 | 60,614 | 62,353 | 1,180 | (59,906; 64,800) | -1,739 | -0,80 | -0,79 |
| 14 | 73,593 | 74,060 | 1,180 | (71,613; 76,507) | -0,467 | -0,21 | -0,21 |
| 15 | 62,639 | 61,554 | 0,941 | (59,603; 63,504) | 1,085 | 0,47 | 0,46 |
| 16 | 67,266 | 69,001 | 0,941 | (67,050; 70,952) | -1,735 | -0,76 | -0,75 |
| 17 | 56,364 | 56,366 | 1,129 | (54,026; 58,707) | -0,002 | -0,00 | -0,00 |
| 18 | 77,064 | 75,333 | 1,129 | (72,993; 77,674) | 1,731 | 0,78 | 0,78 |
| 19 | 68,896 | 67,746 | 0,909 | (65,862; 69,631) | 1,150 | 0,50 | 0,49 |
| 20 | 67,371 | 62,718 | 1,110 | (60,416; 65,021) | 4,653 | 2,10 | 2,29 |
| 21 | 63,909 | 63,526 | 0,996 | (61,461; 65,591) | 0,383 | 0,17 | 0,16 |
| 22 | 72,513 | 68,248 | 1,231 | (65,695; 70,802) | 4,264 | 1,98 | 2,13 |
| 23 | 67,108 | 65,974 | 0,495 | (64,948; 67,000) | 1,133 | 0,47 | 0,46 |
| 24 | 67,042 | 65,758 | 0,495 | (64,733; 66,784) | 1,283 | 0,53 | 0,52 |
| 25 | 63,344 | 65,850 | 0,486 | (64,841; 66,859) | -2,506 | -1,03 | -1,03 |
| 26 | 64,819 | 65,850 | 0,486 | (64,841; 66,859) | -1,031 | -0,42 | -0,42 |
| 27 | 68,961 | 65,850 | 0,486 | (64,841; 66,859) | 3,111 | 1,28 | 1,30 |

| Obs | HI | Cook’s D | DFITS |  |
| --- | --- | --- | --- | --- |
| 1 | 0,231482 | 0,06 | 0,55953 |  |
| 2 | 0,231482 | 0,01 | -0,16387 |  |
| 3 | 0,351061 | 0,41 | -1,53822 |  |
| 4 | 0,351061 | 0,09 | -0,68124 |  |
| 5 | 0,178686 | 0,11 | -0,75966 |  |
| 6 | 0,178686 | 0,04 | -0,42922 |  |
| 7 | 0,344801 | 0,15 | -0,87204 |  |
| 8 | 0,344801 | 0,15 | -0,87824 |  |
| 9 | 0,217619 | 0,00 | -0,06215 |  |
| 10 | 0,217619 | 0,00 | -0,14784 |  |
| 11 | 0,131235 | 0,06 | 0,54338 |  |
| 12 | 0,131235 | 0,01 | 0,16747 |  |
| 13 | 0,226191 | 0,04 | -0,42720 |  |
| 14 | 0,226191 | 0,00 | -0,11318 |  |
| 15 | 0,143742 | 0,01 | 0,19015 |  |
| 16 | 0,143742 | 0,02 | -0,30649 |  |
| 17 | 0,206936 | 0,00 | -0,00044 |  |
| 18 | 0,206936 | 0,03 | 0,39651 |  |
| 19 | 0,134128 | 0,01 | 0,19258 |  |
| 20 | 0,200265 | 0,22 | 1,14625 | R |
| 21 | 0,161088 | 0,00 | 0,07217 |  |
| 22 | 0,246222 | 0,26 | 1,21935 |  |
| 23 | 0,039752 | 0,00 | 0,09313 |  |
| 24 | 0,039724 | 0,00 | 0,10557 |  |
| 25 | 0,038439 | 0,01 | -0,20621 |  |
| 26 | 0,038439 | 0,00 | -0,08308 |  |
| 27 | 0,038439 | 0,01 | 0,25960 |  |

R  Large residual

## Forward Selection of Terms

α to enter = 0,25

## Coded Coefficients

| Term | Coef | SE Coef | 95% CI | T-Value | P-Value | VIF |
| --- | --- | --- | --- | --- | --- | --- |
| Constant | 69,486 | 0,538 | (68,371; 70,602) | 129,16 | 0,000 |  |
| Lac | 9,37 | 1,12 | (7,06; 11,69) | 8,41 | 0,000 | 1,08 |
| HPMC\_Visc | -2,462 | 0,938 | (-4,408; -0,517) | -2,62 | 0,015 | 1,00 |
| HPMC\_HP | 2,56 | 1,07 | (0,34; 4,77) | 2,40 | 0,026 | 1,00 |
| Lac\*HPMC\_Visc | -4,42 | 2,18 | (-8,94; 0,11) | -2,03 | 0,055 | 1,08 |

## Model Summary

| S | R-sq | R-sq(adj) | PRESS | R-sq(pred) | AICc | BIC |
| --- | --- | --- | --- | --- | --- | --- |
| 2,63306 | 82,30% | 79,08% | 252,543 | 70,70% | 139,57 | 143,15 |

## Analysis of Variance

| Source | DF | Seq SS | Contribution | Adj SS | Adj MS | F-Value | P-Value |
| --- | --- | --- | --- | --- | --- | --- | --- |
| Model | 4 | 709,30 | 82,30% | 709,30 | 177,326 | 25,58 | 0,000 |
| Linear | 3 | 680,87 | 79,00% | 573,78 | 191,259 | 27,59 | 0,000 |
| Lac | 1 | 597,03 | 69,27% | 489,94 | 489,938 | 70,67 | 0,000 |
| HPMC\_Visc | 1 | 44,05 | 5,11% | 47,76 | 47,758 | 6,89 | 0,015 |
| HPMC\_HP | 1 | 39,79 | 4,62% | 39,79 | 39,790 | 5,74 | 0,026 |
| 2-Way Interaction | 1 | 28,43 | 3,30% | 28,43 | 28,431 | 4,10 | 0,055 |
| Lac\*HPMC\_Visc | 1 | 28,43 | 3,30% | 28,43 | 28,431 | 4,10 | 0,055 |
| Error | 22 | 152,53 | 17,70% | 152,53 | 6,933 |  |  |
| Lack-of-Fit | 20 | 136,22 | 15,81% | 136,22 | 6,811 | 0,84 | 0,677 |
| Pure Error | 2 | 16,30 | 1,89% | 16,30 | 8,151 |  |  |
| Total | 26 | 861,83 | 100,00% |  |  |  |  |

## Regression Equation in Uncoded Units

|  |  |  |
| --- | --- | --- |
| F\_mean\_10h(600min) | = | 4,8 + 98,9 Lac + 0,00164 HPMC\_Visc + 2,52 HPMC\_HP - 0,00454 Lac\*HPMC\_Visc |

## Fits and Diagnostics for All Observations

| Obs | F\_mean\_10h(600min) | Fit | SE Fit | 95% CI | Resid | Std Resid | Del Resid | HI |
| --- | --- | --- | --- | --- | --- | --- | --- | --- |
| 1 | 65,67 | 63,14 | 1,27 | (60,51; 65,76) | 2,53 | 1,10 | 1,10 | 0,231482 |
| 2 | 74,63 | 75,66 | 1,27 | (73,03; 78,28) | -1,03 | -0,44 | -0,44 | 0,231482 |
| 3 | 58,83 | 62,70 | 1,56 | (59,47; 65,94) | -3,88 | -1,83 | -1,94 | 0,351061 |
| 4 | 66,93 | 69,03 | 1,56 | (65,80; 72,27) | -2,11 | -0,99 | -0,99 | 0,351061 |
| 5 | 62,05 | 65,49 | 1,11 | (63,18; 67,80) | -3,44 | -1,44 | -1,48 | 0,178686 |
| 6 | 75,06 | 77,73 | 1,11 | (75,42; 80,04) | -2,67 | -1,12 | -1,13 | 0,178686 |
| 7 | 63,63 | 65,83 | 1,55 | (62,62; 69,04) | -2,21 | -1,03 | -1,04 | 0,344801 |
| 8 | 69,51 | 72,07 | 1,55 | (68,86; 75,27) | -2,56 | -1,20 | -1,21 | 0,344801 |
| 9 | 62,76 | 63,24 | 1,23 | (60,69; 65,78) | -0,47 | -0,20 | -0,20 | 0,217619 |
| 10 | 74,79 | 75,67 | 1,23 | (73,12; 78,22) | -0,87 | -0,38 | -0,37 | 0,217619 |
| 11 | 67,12 | 63,53 | 0,95 | (61,56; 65,51) | 3,59 | 1,46 | 1,50 | 0,131235 |
| 12 | 72,64 | 72,07 | 0,95 | (70,09; 74,04) | 0,57 | 0,23 | 0,23 | 0,131235 |
| 13 | 64,44 | 66,02 | 1,25 | (63,42; 68,62) | -1,58 | -0,68 | -0,67 | 0,226191 |
| 14 | 77,49 | 78,49 | 1,25 | (75,89; 81,09) | -1,00 | -0,43 | -0,42 | 0,226191 |
| 15 | 66,38 | 65,17 | 1,00 | (63,10; 67,24) | 1,22 | 0,50 | 0,49 | 0,143742 |
| 16 | 71,30 | 73,30 | 1,00 | (71,23; 75,37) | -1,99 | -0,82 | -0,81 | 0,143742 |
| 17 | 60,11 | 59,61 | 1,20 | (57,12; 62,09) | 0,51 | 0,22 | 0,21 | 0,206936 |
| 18 | 84,23 | 80,01 | 1,20 | (77,53; 82,50) | 4,21 | 1,80 | 1,90 | 0,206936 |
| 19 | 72,93 | 71,76 | 0,96 | (69,76; 73,76) | 1,17 | 0,48 | 0,47 | 0,134128 |
| 20 | 70,91 | 66,58 | 1,18 | (64,14; 69,03) | 4,32 | 1,84 | 1,95 | 0,200265 |
| 21 | 67,57 | 67,38 | 1,06 | (65,18; 69,57) | 0,19 | 0,08 | 0,08 | 0,161088 |
| 22 | 76,19 | 72,33 | 1,31 | (69,62; 75,04) | 3,86 | 1,69 | 1,77 | 0,246222 |
| 23 | 71,17 | 69,94 | 0,52 | (68,85; 71,03) | 1,24 | 0,48 | 0,47 | 0,039752 |
| 24 | 70,77 | 69,71 | 0,52 | (68,62; 70,80) | 1,06 | 0,41 | 0,40 | 0,039724 |
| 25 | 67,14 | 69,81 | 0,52 | (68,74; 70,88) | -2,67 | -1,03 | -1,03 | 0,038439 |
| 26 | 68,89 | 69,81 | 0,52 | (68,74; 70,88) | -0,92 | -0,35 | -0,35 | 0,038439 |
| 27 | 72,73 | 69,81 | 0,52 | (68,74; 70,88) | 2,92 | 1,13 | 1,14 | 0,038439 |

| Obs | Cook’s D | DFITS |
| --- | --- | --- |
| 1 | 0,07 | 0,60389 |
| 2 | 0,01 | -0,23920 |
| 3 | 0,36 | -1,42666 |
| 4 | 0,11 | -0,73019 |
| 5 | 0,09 | -0,68957 |
| 6 | 0,05 | -0,52477 |
| 7 | 0,11 | -0,75208 |
| 8 | 0,15 | -0,88065 |
| 9 | 0,00 | -0,10480 |
| 10 | 0,01 | -0,19402 |
| 11 | 0,06 | 0,58458 |
| 12 | 0,00 | 0,08857 |
| 13 | 0,03 | -0,36317 |
| 14 | 0,01 | -0,22866 |
| 15 | 0,01 | 0,20086 |
| 16 | 0,02 | -0,33265 |
| 17 | 0,00 | 0,10761 |
| 18 | 0,17 | 0,97110 |
| 19 | 0,01 | 0,18443 |
| 20 | 0,17 | 0,97543 |
| 21 | 0,00 | 0,03376 |
| 22 | 0,19 | 1,01150 |
| 23 | 0,00 | 0,09572 |
| 24 | 0,00 | 0,08178 |
| 25 | 0,01 | -0,20684 |
| 26 | 0,00 | -0,06952 |
| 27 | 0,01 | 0,22724 |

## Forward Selection of Terms

α to enter = 0,25

## Coded Coefficients

| Term | Coef | SE Coef | 95% CI | T-Value | P-Value | VIF |
| --- | --- | --- | --- | --- | --- | --- |
| Constant | 73,136 | 0,535 | (72,027; 74,245) | 136,74 | 0,000 |  |
| Lac | 9,65 | 1,11 | (7,35; 11,95) | 8,70 | 0,000 | 1,08 |
| HPMC\_Visc | -2,517 | 0,933 | (-4,452; -0,583) | -2,70 | 0,013 | 1,00 |
| HPMC\_HP | 2,72 | 1,06 | (0,52; 4,91) | 2,56 | 0,018 | 1,00 |
| Lac\*HPMC\_Visc | -4,72 | 2,17 | (-9,22; -0,23) | -2,18 | 0,040 | 1,08 |

## Model Summary

| S | R-sq | R-sq(adj) | PRESS | R-sq(pred) | AICc | BIC |
| --- | --- | --- | --- | --- | --- | --- |
| 2,61784 | 83,42% | 80,40% | 247,731 | 72,75% | 139,26 | 142,84 |

## Analysis of Variance

| Source | DF | Seq SS | Contribution | Adj SS | Adj MS | F-Value | P-Value |
| --- | --- | --- | --- | --- | --- | --- | --- |
| Model | 4 | 758,46 | 83,42% | 758,46 | 189,614 | 27,67 | 0,000 |
| Linear | 3 | 725,95 | 79,84% | 609,58 | 203,192 | 29,65 | 0,000 |
| Lac | 1 | 635,13 | 69,85% | 518,76 | 518,756 | 75,70 | 0,000 |
| HPMC\_Visc | 1 | 45,89 | 5,05% | 49,92 | 49,915 | 7,28 | 0,013 |
| HPMC\_HP | 1 | 44,93 | 4,94% | 44,93 | 44,933 | 6,56 | 0,018 |
| 2-Way Interaction | 1 | 32,51 | 3,58% | 32,51 | 32,505 | 4,74 | 0,040 |
| Lac\*HPMC\_Visc | 1 | 32,51 | 3,58% | 32,51 | 32,505 | 4,74 | 0,040 |
| Error | 22 | 150,77 | 16,58% | 150,77 | 6,853 |  |  |
| Lack-of-Fit | 20 | 135,54 | 14,91% | 135,54 | 6,777 | 0,89 | 0,655 |
| Pure Error | 2 | 15,23 | 1,68% | 15,23 | 7,617 |  |  |
| Total | 26 | 909,23 | 100,00% |  |  |  |  |

## Regression Equation in Uncoded Units

|  |  |  |
| --- | --- | --- |
| F\_mean\_11h(660min) | = | 4,4 + 104,2 Lac + 0,00178 HPMC\_Visc + 2,68 HPMC\_HP - 0,00486 Lac\*HPMC\_Visc |

## Fits and Diagnostics for All Observations

| Obs | F\_mean\_11h(660min) | Fit | SE Fit | 95% CI | Resid | Std Resid | Del Resid | HI |
| --- | --- | --- | --- | --- | --- | --- | --- | --- |
| 1 | 68,86 | 66,47 | 1,26 | (63,85; 69,08) | 2,39 | 1,04 | 1,04 | 0,231482 |
| 2 | 78,48 | 79,47 | 1,26 | (76,86; 82,09) | -0,99 | -0,43 | -0,42 | 0,231482 |
| 3 | 62,20 | 66,16 | 1,55 | (62,95; 69,38) | -3,96 | -1,88 | -2,00 | 0,351061 |
| 4 | 70,58 | 72,55 | 1,55 | (69,34; 75,77) | -1,97 | -0,94 | -0,93 | 0,351061 |
| 5 | 65,58 | 68,97 | 1,11 | (66,68; 71,27) | -3,39 | -1,43 | -1,46 | 0,178686 |
| 6 | 78,49 | 81,68 | 1,11 | (79,39; 83,98) | -3,19 | -1,35 | -1,37 | 0,178686 |
| 7 | 67,32 | 69,49 | 1,54 | (66,30; 72,68) | -2,17 | -1,02 | -1,02 | 0,344801 |
| 8 | 73,44 | 75,78 | 1,54 | (72,59; 78,97) | -2,34 | -1,10 | -1,11 | 0,344801 |
| 9 | 65,94 | 66,57 | 1,22 | (64,04; 69,11) | -0,64 | -0,27 | -0,27 | 0,217619 |
| 10 | 78,64 | 79,49 | 1,22 | (76,96; 82,02) | -0,85 | -0,37 | -0,36 | 0,217619 |
| 11 | 70,91 | 66,99 | 0,95 | (65,02; 68,96) | 3,91 | 1,60 | 1,67 | 0,131235 |
| 12 | 76,19 | 75,74 | 0,95 | (73,77; 77,70) | 0,45 | 0,19 | 0,18 | 0,131235 |
| 13 | 68,14 | 69,53 | 1,25 | (66,95; 72,11) | -1,39 | -0,60 | -0,59 | 0,226191 |
| 14 | 82,05 | 82,49 | 1,25 | (79,91; 85,07) | -0,44 | -0,19 | -0,19 | 0,226191 |
| 15 | 70,11 | 68,74 | 0,99 | (66,68; 70,80) | 1,38 | 0,57 | 0,56 | 0,143742 |
| 16 | 75,05 | 77,05 | 0,99 | (74,99; 79,11) | -2,00 | -0,83 | -0,82 | 0,143742 |
| 17 | 63,67 | 62,93 | 1,19 | (60,46; 65,40) | 0,74 | 0,32 | 0,31 | 0,206936 |
| 18 | 88,47 | 83,99 | 1,19 | (81,52; 86,46) | 4,47 | 1,92 | 2,05 | 0,206936 |
| 19 | 76,75 | 75,45 | 0,96 | (73,46; 77,44) | 1,30 | 0,53 | 0,52 | 0,134128 |
| 20 | 74,25 | 70,15 | 1,17 | (67,72; 72,58) | 4,10 | 1,75 | 1,85 | 0,200265 |
| 21 | 70,99 | 70,87 | 1,05 | (68,70; 73,05) | 0,11 | 0,05 | 0,05 | 0,161088 |
| 22 | 79,48 | 76,14 | 1,30 | (73,45; 78,84) | 3,34 | 1,47 | 1,51 | 0,246222 |
| 23 | 74,80 | 73,59 | 0,52 | (72,51; 74,67) | 1,22 | 0,47 | 0,47 | 0,039752 |
| 24 | 74,21 | 73,35 | 0,52 | (72,27; 74,43) | 0,87 | 0,34 | 0,33 | 0,039724 |
| 25 | 70,68 | 73,46 | 0,51 | (72,40; 74,53) | -2,78 | -1,08 | -1,09 | 0,038439 |
| 26 | 72,61 | 73,46 | 0,51 | (72,40; 74,53) | -0,85 | -0,33 | -0,32 | 0,038439 |
| 27 | 76,13 | 73,46 | 0,51 | (72,40; 74,53) | 2,67 | 1,04 | 1,04 | 0,038439 |

| Obs | Cook’s D | DFITS |
| --- | --- | --- |
| 1 | 0,07 | 0,57297 |
| 2 | 0,01 | -0,23309 |
| 3 | 0,38 | -1,47360 |
| 4 | 0,09 | -0,68582 |
| 5 | 0,09 | -0,68324 |
| 6 | 0,08 | -0,63999 |
| 7 | 0,11 | -0,74308 |
| 8 | 0,13 | -0,80446 |
| 9 | 0,00 | -0,14191 |
| 10 | 0,01 | -0,18917 |
| 11 | 0,08 | 0,64829 |
| 12 | 0,00 | 0,07071 |
| 13 | 0,02 | -0,32063 |
| 14 | 0,00 | -0,10024 |
| 15 | 0,01 | 0,22920 |
| 16 | 0,02 | -0,33564 |
| 17 | 0,01 | 0,15850 |
| 18 | 0,19 | 1,04892 |
| 19 | 0,01 | 0,20577 |
| 20 | 0,15 | 0,92342 |
| 21 | 0,00 | 0,02013 |
| 22 | 0,14 | 0,86433 |
| 23 | 0,00 | 0,09470 |
| 24 | 0,00 | 0,06732 |
| 25 | 0,01 | -0,21722 |
| 26 | 0,00 | -0,06466 |
| 27 | 0,01 | 0,20802 |

## Forward Selection of Terms

α to enter = 0,25

## Coded Coefficients

| Term | Coef | SE Coef | 95% CI | T-Value | P-Value | VIF |
| --- | --- | --- | --- | --- | --- | --- |
| Constant | 76,469 | 0,514 | (75,403; 77,535) | 148,83 | 0,000 |  |
| Lac | 9,68 | 1,07 | (7,47; 11,89) | 9,09 | 0,000 | 1,08 |
| HPMC\_Visc | -2,584 | 0,896 | (-4,442; -0,725) | -2,88 | 0,009 | 1,00 |
| HPMC\_HP | 2,76 | 1,02 | (0,65; 4,87) | 2,71 | 0,013 | 1,00 |
| Lac\*HPMC\_Visc | -4,89 | 2,08 | (-9,22; -0,57) | -2,35 | 0,028 | 1,08 |

## Model Summary

| S | R-sq | R-sq(adj) | PRESS | R-sq(pred) | AICc | BIC |
| --- | --- | --- | --- | --- | --- | --- |
| 2,51476 | 84,72% | 81,95% | 227,738 | 75,00% | 137,09 | 140,67 |

## Analysis of Variance

| Source | DF | Seq SS | Contribution | Adj SS | Adj MS | F-Value | P-Value |
| --- | --- | --- | --- | --- | --- | --- | --- |
| Model | 4 | 771,64 | 84,72% | 771,64 | 192,911 | 30,50 | 0,000 |
| Linear | 3 | 736,74 | 80,89% | 616,96 | 205,653 | 32,52 | 0,000 |
| Lac | 1 | 642,01 | 70,49% | 522,23 | 522,231 | 82,58 | 0,000 |
| HPMC\_Visc | 1 | 48,37 | 5,31% | 52,57 | 52,572 | 8,31 | 0,009 |
| HPMC\_HP | 1 | 46,36 | 5,09% | 46,36 | 46,356 | 7,33 | 0,013 |
| 2-Way Interaction | 1 | 34,91 | 3,83% | 34,91 | 34,905 | 5,52 | 0,028 |
| Lac\*HPMC\_Visc | 1 | 34,91 | 3,83% | 34,91 | 34,905 | 5,52 | 0,028 |
| Error | 22 | 139,13 | 15,28% | 139,13 | 6,324 |  |  |
| Lack-of-Fit | 20 | 124,71 | 13,69% | 124,71 | 6,236 | 0,87 | 0,665 |
| Pure Error | 2 | 14,42 | 1,58% | 14,42 | 7,208 |  |  |
| Total | 26 | 910,77 | 100,00% |  |  |  |  |

## Regression Equation in Uncoded Units

|  |  |  |
| --- | --- | --- |
| F\_mean\_12h(720min) | = | 6,3 + 106,7 Lac + 0,00185 HPMC\_Visc + 2,72 HPMC\_HP - 0,00503 Lac\*HPMC\_Visc |

## Fits and Diagnostics for All Observations

| Obs | F\_mean\_12h(720min) | Fit | SE Fit | 95% CI | Resid | Std Resid | Del Resid |
| --- | --- | --- | --- | --- | --- | --- | --- |
| 1 | 72,058 | 69,739 | 1,210 | (67,229; 72,248) | 2,320 | 1,05 | 1,05 |
| 2 | 81,925 | 82,902 | 1,210 | (80,392; 85,411) | -0,976 | -0,44 | -0,43 |
| 3 | 65,371 | 69,463 | 1,490 | (66,373; 72,553) | -4,091 | -2,02 | -2,19 |
| 4 | 74,075 | 75,766 | 1,490 | (72,676; 78,857) | -1,691 | -0,83 | -0,83 |
| 5 | 68,884 | 72,284 | 1,063 | (70,080; 74,489) | -3,400 | -1,49 | -1,54 |
| 6 | 81,718 | 85,141 | 1,063 | (82,936; 87,345) | -3,423 | -1,50 | -1,55 |
| 7 | 70,725 | 72,842 | 1,477 | (69,779; 75,904) | -2,116 | -1,04 | -1,04 |
| 8 | 76,996 | 79,043 | 1,477 | (75,981; 82,106) | -2,047 | -1,01 | -1,01 |
| 9 | 68,904 | 69,848 | 1,173 | (67,415; 72,281) | -0,944 | -0,42 | -0,42 |
| 10 | 82,575 | 82,915 | 1,173 | (80,482; 85,348) | -0,340 | -0,15 | -0,15 |
| 11 | 74,429 | 70,291 | 0,911 | (68,402; 72,181) | 4,138 | 1,77 | 1,86 |
| 12 | 79,559 | 79,036 | 0,911 | (77,147; 80,926) | 0,523 | 0,22 | 0,22 |
| 13 | 71,590 | 72,851 | 1,196 | (70,371; 75,332) | -1,261 | -0,57 | -0,56 |
| 14 | 86,147 | 85,961 | 1,196 | (83,481; 88,441) | 0,186 | 0,08 | 0,08 |
| 15 | 73,461 | 72,067 | 0,953 | (70,090; 74,044) | 1,395 | 0,60 | 0,59 |
| 16 | 78,588 | 80,364 | 0,953 | (78,386; 82,341) | -1,776 | -0,76 | -0,76 |
| 17 | 67,033 | 66,208 | 1,144 | (63,835; 68,580) | 0,825 | 0,37 | 0,36 |
| 18 | 91,018 | 87,401 | 1,144 | (85,029; 89,774) | 3,617 | 1,61 | 1,68 |
| 19 | 80,233 | 78,849 | 0,921 | (76,939; 80,759) | 1,384 | 0,59 | 0,58 |
| 20 | 77,312 | 73,410 | 1,125 | (71,076; 75,744) | 3,902 | 1,74 | 1,82 |
| 21 | 74,103 | 74,177 | 1,009 | (72,084; 76,271) | -0,074 | -0,03 | -0,03 |
| 22 | 82,470 | 79,526 | 1,248 | (76,938; 82,114) | 2,945 | 1,35 | 1,38 |
| 23 | 78,081 | 76,936 | 0,501 | (75,896; 77,976) | 1,145 | 0,46 | 0,46 |
| 24 | 77,405 | 76,691 | 0,501 | (75,651; 77,730) | 0,714 | 0,29 | 0,28 |
| 25 | 74,015 | 76,804 | 0,493 | (75,782; 77,827) | -2,790 | -1,13 | -1,14 |
| 26 | 76,104 | 76,804 | 0,493 | (75,782; 77,827) | -0,700 | -0,28 | -0,28 |
| 27 | 79,343 | 76,804 | 0,493 | (75,782; 77,827) | 2,539 | 1,03 | 1,03 |

| Obs | HI | Cook’s D | DFITS |  |
| --- | --- | --- | --- | --- |
| 1 | 0,231482 | 0,07 | 0,57893 |  |
| 2 | 0,231482 | 0,01 | -0,23848 |  |
| 3 | 0,351061 | 0,44 | -1,60799 | R |
| 4 | 0,351061 | 0,08 | -0,60963 |  |
| 5 | 0,178686 | 0,10 | -0,71720 |  |
| 6 | 0,178686 | 0,10 | -0,72253 |  |
| 7 | 0,344801 | 0,11 | -0,75566 |  |
| 8 | 0,344801 | 0,11 | -0,72981 |  |
| 9 | 0,217619 | 0,01 | -0,21958 |  |
| 10 | 0,217619 | 0,00 | -0,07877 |  |
| 11 | 0,131235 | 0,09 | 0,72355 |  |
| 12 | 0,131235 | 0,00 | 0,08482 |  |
| 13 | 0,226191 | 0,02 | -0,30347 |  |
| 14 | 0,226191 | 0,00 | 0,04440 |  |
| 15 | 0,143742 | 0,01 | 0,24187 |  |
| 16 | 0,143742 | 0,02 | -0,30962 |  |
| 17 | 0,206936 | 0,01 | 0,18445 |  |
| 18 | 0,206936 | 0,14 | 0,85845 |  |
| 19 | 0,134128 | 0,01 | 0,22924 |  |
| 20 | 0,200265 | 0,15 | 0,91317 |  |
| 21 | 0,161088 | 0,00 | -0,01381 |  |
| 22 | 0,246222 | 0,12 | 0,78631 |  |
| 23 | 0,039752 | 0,00 | 0,09278 |  |
| 24 | 0,039724 | 0,00 | 0,05771 |  |
| 25 | 0,038439 | 0,01 | -0,22770 |  |
| 26 | 0,038439 | 0,00 | -0,05557 |  |
| 27 | 0,038439 | 0,01 | 0,20614 |  |

R  Large residual

## Forward Selection of Terms

α to enter = 0,25

## Coded Coefficients

| Term | Coef | SE Coef | 95% CI | T-Value | P-Value | VIF |
| --- | --- | --- | --- | --- | --- | --- |
| Constant | 79,514 | 0,497 | (78,483; 80,546) | 159,88 | 0,000 |  |
| Lac | 9,68 | 1,03 | (7,54; 11,82) | 9,39 | 0,000 | 1,08 |
| HPMC\_Visc | -2,539 | 0,867 | (-4,338; -0,741) | -2,93 | 0,008 | 1,00 |
| HPMC\_HP | 2,617 | 0,986 | (0,572; 4,662) | 2,65 | 0,014 | 1,00 |
| Lac\*HPMC\_Visc | -4,66 | 2,02 | (-8,84; -0,47) | -2,31 | 0,031 | 1,08 |

## Model Summary

| S | R-sq | R-sq(adj) | PRESS | R-sq(pred) | AICc | BIC |
| --- | --- | --- | --- | --- | --- | --- |
| 2,43413 | 85,33% | 82,66% | 213,132 | 76,01% | 135,33 | 138,91 |

## Analysis of Variance

| Source | DF | Seq SS | Contribution | Adj SS | Adj MS | F-Value | P-Value |
| --- | --- | --- | --- | --- | --- | --- | --- |
| Model | 4 | 758,10 | 85,33% | 758,10 | 189,524 | 31,99 | 0,000 |
| Linear | 3 | 726,50 | 81,77% | 610,75 | 203,584 | 34,36 | 0,000 |
| Lac | 1 | 637,88 | 71,80% | 522,13 | 522,132 | 88,12 | 0,000 |
| HPMC\_Visc | 1 | 46,87 | 5,28% | 50,79 | 50,790 | 8,57 | 0,008 |
| HPMC\_HP | 1 | 41,75 | 4,70% | 41,75 | 41,747 | 7,05 | 0,014 |
| 2-Way Interaction | 1 | 31,59 | 3,56% | 31,59 | 31,593 | 5,33 | 0,031 |
| Lac\*HPMC\_Visc | 1 | 31,59 | 3,56% | 31,59 | 31,593 | 5,33 | 0,031 |
| Error | 22 | 130,35 | 14,67% | 130,35 | 5,925 |  |  |
| Lack-of-Fit | 20 | 116,99 | 13,17% | 116,99 | 5,850 | 0,88 | 0,661 |
| Pure Error | 2 | 13,36 | 1,50% | 13,36 | 6,678 |  |  |
| Total | 26 | 888,45 | 100,00% |  |  |  |  |

## Regression Equation in Uncoded Units

|  |  |  |
| --- | --- | --- |
| F\_mean\_13h(780min) | = | 12,2 + 103,4 Lac + 0,00174 HPMC\_Visc + 2,578 HPMC\_HP - 0,00479 Lac\*HPMC\_Visc |

## Fits and Diagnostics for All Observations

| Obs | F\_mean\_13h(780min) | Fit | SE Fit | 95% CI | Resid | Std Resid | Del Resid |
| --- | --- | --- | --- | --- | --- | --- | --- |
| 1 | 75,207 | 72,939 | 1,171 | (70,510; 75,368) | 2,268 | 1,06 | 1,07 |
| 2 | 85,112 | 85,932 | 1,171 | (83,503; 88,361) | -0,820 | -0,38 | -0,38 |
| 3 | 68,335 | 72,563 | 1,442 | (69,572; 75,554) | -4,228 | -2,16 | -2,37 |
| 4 | 77,455 | 79,030 | 1,442 | (76,039; 82,021) | -1,575 | -0,80 | -0,80 |
| 5 | 72,004 | 75,350 | 1,029 | (73,216; 77,484) | -3,346 | -1,52 | -1,57 |
| 6 | 84,615 | 88,051 | 1,029 | (85,917; 90,185) | -3,435 | -1,56 | -1,61 |
| 7 | 73,784 | 75,768 | 1,429 | (72,803; 78,732) | -1,984 | -1,01 | -1,01 |
| 8 | 80,481 | 82,137 | 1,429 | (79,173; 85,101) | -1,656 | -0,84 | -0,83 |
| 9 | 71,666 | 73,042 | 1,136 | (70,687; 75,397) | -1,376 | -0,64 | -0,63 |
| 10 | 86,275 | 85,943 | 1,136 | (83,588; 88,298) | 0,332 | 0,15 | 0,15 |
| 11 | 77,673 | 73,390 | 0,882 | (71,561; 75,219) | 4,282 | 1,89 | 2,01 |
| 12 | 82,648 | 82,179 | 0,882 | (80,351; 84,008) | 0,469 | 0,21 | 0,20 |
| 13 | 74,708 | 75,892 | 1,158 | (73,492; 78,293) | -1,184 | -0,55 | -0,54 |
| 14 | 88,777 | 88,834 | 1,158 | (86,433; 91,235) | -0,057 | -0,03 | -0,03 |
| 15 | 76,503 | 75,067 | 0,923 | (73,154; 76,981) | 1,435 | 0,64 | 0,63 |
| 16 | 81,890 | 83,430 | 0,923 | (81,517; 85,344) | -1,540 | -0,68 | -0,68 |
| 17 | 70,162 | 69,298 | 1,107 | (67,002; 71,594) | 0,864 | 0,40 | 0,39 |
| 18 | 93,340 | 90,401 | 1,107 | (88,104; 92,697) | 2,939 | 1,36 | 1,38 |
| 19 | 83,434 | 81,861 | 0,891 | (80,012; 83,709) | 1,574 | 0,69 | 0,69 |
| 20 | 80,136 | 76,524 | 1,089 | (74,265; 78,783) | 3,612 | 1,66 | 1,73 |
| 21 | 77,183 | 77,356 | 0,977 | (75,329; 79,382) | -0,173 | -0,08 | -0,08 |
| 22 | 85,226 | 82,425 | 1,208 | (79,921; 84,930) | 2,800 | 1,33 | 1,35 |
| 23 | 81,026 | 79,980 | 0,485 | (78,974; 80,987) | 1,045 | 0,44 | 0,43 |
| 24 | 80,490 | 79,749 | 0,485 | (78,743; 80,755) | 0,741 | 0,31 | 0,30 |
| 25 | 77,114 | 79,849 | 0,477 | (78,859; 80,839) | -2,735 | -1,15 | -1,15 |
| 26 | 79,194 | 79,849 | 0,477 | (78,859; 80,839) | -0,655 | -0,27 | -0,27 |
| 27 | 82,251 | 79,849 | 0,477 | (78,859; 80,839) | 2,402 | 1,01 | 1,01 |

| Obs | HI | Cook’s D | DFITS |  |
| --- | --- | --- | --- | --- |
| 1 | 0,231482 | 0,07 | 0,58514 |  |
| 2 | 0,231482 | 0,01 | -0,20668 |  |
| 3 | 0,351061 | 0,50 | -1,74471 | R |
| 4 | 0,351061 | 0,07 | -0,58588 |  |
| 5 | 0,178686 | 0,10 | -0,73035 |  |
| 6 | 0,178686 | 0,11 | -0,75238 |  |
| 7 | 0,344801 | 0,11 | -0,73069 |  |
| 8 | 0,344801 | 0,07 | -0,60550 |  |
| 9 | 0,217619 | 0,02 | -0,33229 |  |
| 10 | 0,217619 | 0,00 | 0,07957 |  |
| 11 | 0,131235 | 0,11 | 0,78295 |  |
| 12 | 0,131235 | 0,00 | 0,07854 |  |
| 13 | 0,226191 | 0,02 | -0,29422 |  |
| 14 | 0,226191 | 0,00 | -0,01413 |  |
| 15 | 0,143742 | 0,01 | 0,25749 |  |
| 16 | 0,143742 | 0,02 | -0,27668 |  |
| 17 | 0,206936 | 0,01 | 0,19957 |  |
| 18 | 0,206936 | 0,10 | 0,70692 |  |
| 19 | 0,134128 | 0,01 | 0,27015 |  |
| 20 | 0,200265 | 0,14 | 0,86728 |  |
| 21 | 0,161088 | 0,00 | -0,03324 |  |
| 22 | 0,246222 | 0,11 | 0,77133 |  |
| 23 | 0,039752 | 0,00 | 0,08751 |  |
| 24 | 0,039724 | 0,00 | 0,06191 |  |
| 25 | 0,038439 | 0,01 | -0,23087 |  |
| 26 | 0,038439 | 0,00 | -0,05373 |  |
| 27 | 0,038439 | 0,01 | 0,20127 |  |

R  Large residual

## Forward Selection of Terms

α to enter = 0,25

## Coded Coefficients

| Term | Coef | SE Coef | 95% CI | T-Value | P-Value | VIF |
| --- | --- | --- | --- | --- | --- | --- |
| Constant | 82,333 | 0,469 | (81,360; 83,306) | 175,52 | 0,000 |  |
| Lac | 9,515 | 0,972 | (7,499; 11,532) | 9,79 | 0,000 | 1,08 |
| HPMC\_Visc | -2,283 | 0,818 | (-3,980; -0,586) | -2,79 | 0,011 | 1,00 |
| HPMC\_HP | 2,488 | 0,930 | (0,559; 4,416) | 2,68 | 0,014 | 1,00 |
| Lac\*HPMC\_Visc | -4,25 | 1,90 | (-8,19; -0,30) | -2,23 | 0,036 | 1,08 |

## Model Summary

| S | R-sq | R-sq(adj) | PRESS | R-sq(pred) | AICc | BIC |
| --- | --- | --- | --- | --- | --- | --- |
| 2,29584 | 86,01% | 83,47% | 189,246 | 77,17% | 132,17 | 135,75 |

## Analysis of Variance

| Source | DF | Seq SS | Contribution | Adj SS | Adj MS | F-Value | P-Value |
| --- | --- | --- | --- | --- | --- | --- | --- |
| Model | 4 | 712,97 | 86,01% | 712,97 | 178,242 | 33,82 | 0,000 |
| Linear | 3 | 686,66 | 82,84% | 580,19 | 193,397 | 36,69 | 0,000 |
| Lac | 1 | 611,24 | 73,74% | 504,77 | 504,767 | 95,77 | 0,000 |
| HPMC\_Visc | 1 | 37,70 | 4,55% | 41,05 | 41,052 | 7,79 | 0,011 |
| HPMC\_HP | 1 | 37,72 | 4,55% | 37,72 | 37,718 | 7,16 | 0,014 |
| 2-Way Interaction | 1 | 26,31 | 3,17% | 26,31 | 26,311 | 4,99 | 0,036 |
| Lac\*HPMC\_Visc | 1 | 26,31 | 3,17% | 26,31 | 26,311 | 4,99 | 0,036 |
| Error | 22 | 115,96 | 13,99% | 115,96 | 5,271 |  |  |
| Lack-of-Fit | 20 | 103,76 | 12,52% | 103,76 | 5,188 | 0,85 | 0,671 |
| Pure Error | 2 | 12,20 | 1,47% | 12,20 | 6,100 |  |  |
| Total | 26 | 828,93 | 100,00% |  |  |  |  |

## Regression Equation in Uncoded Units

|  |  |  |
| --- | --- | --- |
| F\_mean\_14h(840min) | = | 18,5 + 97,1 Lac + 0,00160 HPMC\_Visc + 2,451 HPMC\_HP - 0,00437 Lac\*HPMC\_Visc |

## Fits and Diagnostics for All Observations

| Obs | F\_mean\_14h(840min) | Fit | SE Fit | 95% CI | Resid | Std Resid | Del Resid |
| --- | --- | --- | --- | --- | --- | --- | --- |
| 1 | 77,720 | 75,895 | 1,105 | (73,604; 78,185) | 1,826 | 0,91 | 0,90 |
| 2 | 87,872 | 88,435 | 1,105 | (86,144; 90,726) | -0,563 | -0,28 | -0,27 |
| 3 | 71,142 | 75,596 | 1,360 | (72,775; 78,417) | -4,454 | -2,41 | -2,74 |
| 4 | 80,866 | 82,182 | 1,360 | (79,360; 85,003) | -1,316 | -0,71 | -0,70 |
| 5 | 74,970 | 78,189 | 0,970 | (76,176; 80,201) | -3,219 | -1,55 | -1,60 |
| 6 | 87,433 | 90,463 | 0,970 | (88,450; 92,476) | -3,030 | -1,46 | -1,50 |
| 7 | 77,141 | 78,643 | 1,348 | (75,848; 81,439) | -1,503 | -0,81 | -0,80 |
| 8 | 84,045 | 85,140 | 1,348 | (82,344; 87,936) | -1,094 | -0,59 | -0,58 |
| 9 | 74,297 | 75,993 | 1,071 | (73,772; 78,214) | -1,696 | -0,84 | -0,83 |
| 10 | 89,522 | 88,450 | 1,071 | (86,229; 90,671) | 1,072 | 0,53 | 0,52 |
| 11 | 80,758 | 76,361 | 0,832 | (74,637; 78,086) | 4,397 | 2,05 | 2,23 |
| 12 | 85,507 | 85,066 | 0,832 | (83,341; 86,791) | 0,441 | 0,21 | 0,20 |
| 13 | 77,648 | 78,702 | 1,092 | (76,438; 80,967) | -1,055 | -0,52 | -0,51 |
| 14 | 90,387 | 91,196 | 1,092 | (88,932; 93,461) | -0,809 | -0,40 | -0,39 |
| 15 | 79,239 | 77,960 | 0,870 | (76,155; 79,765) | 1,279 | 0,60 | 0,59 |
| 16 | 84,863 | 86,275 | 0,870 | (84,470; 88,081) | -1,413 | -0,67 | -0,66 |
| 17 | 73,177 | 72,314 | 1,044 | (70,148; 74,480) | 0,863 | 0,42 | 0,41 |
| 18 | 94,946 | 92,939 | 1,044 | (90,773; 95,105) | 2,006 | 0,98 | 0,98 |
| 19 | 86,403 | 84,432 | 0,841 | (82,689; 86,176) | 1,970 | 0,92 | 0,92 |
| 20 | 82,719 | 79,621 | 1,027 | (77,490; 81,752) | 3,098 | 1,51 | 1,56 |
| 21 | 80,080 | 80,257 | 0,921 | (78,346; 82,168) | -0,177 | -0,08 | -0,08 |
| 22 | 87,653 | 85,085 | 1,139 | (82,722; 87,447) | 2,568 | 1,29 | 1,31 |
| 23 | 83,594 | 82,742 | 0,458 | (81,793; 83,691) | 0,852 | 0,38 | 0,37 |
| 24 | 83,371 | 82,520 | 0,458 | (81,571; 83,469) | 0,851 | 0,38 | 0,37 |
| 25 | 80,007 | 82,627 | 0,450 | (81,693; 83,560) | -2,620 | -1,16 | -1,17 |
| 26 | 82,056 | 82,627 | 0,450 | (81,693; 83,560) | -0,571 | -0,25 | -0,25 |
| 27 | 84,924 | 82,627 | 0,450 | (81,693; 83,560) | 2,297 | 1,02 | 1,02 |

| Obs | HI | Cook’s D | DFITS |  |
| --- | --- | --- | --- | --- |
| 1 | 0,231482 | 0,05 | 0,49572 |  |
| 2 | 0,231482 | 0,00 | -0,15038 |  |
| 3 | 0,351061 | 0,63 | -2,01682 | R |
| 4 | 0,351061 | 0,05 | -0,51722 |  |
| 5 | 0,178686 | 0,10 | -0,74680 |  |
| 6 | 0,178686 | 0,09 | -0,69810 |  |
| 7 | 0,344801 | 0,07 | -0,58180 |  |
| 8 | 0,344801 | 0,04 | -0,42074 |  |
| 9 | 0,217619 | 0,04 | -0,43733 |  |
| 10 | 0,217619 | 0,02 | 0,27367 |  |
| 11 | 0,131235 | 0,13 | 0,86792 | R |
| 12 | 0,131235 | 0,00 | 0,07832 |  |
| 13 | 0,226191 | 0,02 | -0,27758 |  |
| 14 | 0,226191 | 0,01 | -0,21237 |  |
| 15 | 0,143742 | 0,01 | 0,24299 |  |
| 16 | 0,143742 | 0,01 | -0,26894 |  |
| 17 | 0,206936 | 0,01 | 0,21143 |  |
| 18 | 0,206936 | 0,05 | 0,50085 |  |
| 19 | 0,134128 | 0,03 | 0,36172 |  |
| 20 | 0,200265 | 0,11 | 0,77911 |  |
| 21 | 0,161088 | 0,00 | -0,03603 |  |
| 22 | 0,246222 | 0,11 | 0,74819 |  |
| 23 | 0,039752 | 0,00 | 0,07557 |  |
| 24 | 0,039724 | 0,00 | 0,07538 |  |
| 25 | 0,038439 | 0,01 | -0,23467 |  |
| 26 | 0,038439 | 0,00 | -0,04961 |  |
| 27 | 0,038439 | 0,01 | 0,20420 |  |

R  Large residual

## Forward Selection of Terms

α to enter = 0,25

## Coded Coefficients

| Term | Coef | SE Coef | 95% CI | T-Value | P-Value | VIF |
| --- | --- | --- | --- | --- | --- | --- |
| Constant | 84,874 | 0,450 | (83,937; 85,810) | 188,47 | 0,000 |  |
| Lac | 9,185 | 0,933 | (7,244; 11,126) | 9,84 | 0,000 | 1,08 |
| HPMC\_Visc | -1,771 | 0,826 | (-3,488; -0,053) | -2,14 | 0,044 | 1,11 |
| HPMC\_HP | 2,481 | 0,908 | (0,594; 4,369) | 2,73 | 0,012 | 1,04 |
| Lac\*HPMC\_Visc | -3,64 | 1,83 | (-7,44; 0,15) | -2,00 | 0,059 | 1,08 |
| HPMC\_Visc\*HPMC\_HP | 2,07 | 1,66 | (-1,38; 5,52) | 1,25 | 0,226 | 1,15 |

## Model Summary

| S | R-sq | R-sq(adj) | PRESS | R-sq(pred) | AICc | BIC |
| --- | --- | --- | --- | --- | --- | --- |
| 2,20384 | 86,48% | 83,26% | 195,951 | 74,02% | 132,40 | 135,58 |

## Analysis of Variance

| Source | DF | Seq SS | Contribution | Adj SS | Adj MS | F-Value | P-Value |
| --- | --- | --- | --- | --- | --- | --- | --- |
| Model | 5 | 652,313 | 86,48% | 652,313 | 130,463 | 26,86 | 0,000 |
| Linear | 3 | 625,402 | 82,91% | 529,765 | 176,588 | 36,36 | 0,000 |
| Lac | 1 | 562,249 | 74,54% | 470,307 | 470,307 | 96,83 | 0,000 |
| HPMC\_Visc | 1 | 31,578 | 4,19% | 22,334 | 22,334 | 4,60 | 0,044 |
| HPMC\_HP | 1 | 31,576 | 4,19% | 36,291 | 36,291 | 7,47 | 0,012 |
| 2-Way Interaction | 2 | 26,911 | 3,57% | 26,911 | 13,456 | 2,77 | 0,086 |
| Lac\*HPMC\_Visc | 1 | 19,347 | 2,56% | 19,347 | 19,347 | 3,98 | 0,059 |
| HPMC\_Visc\*HPMC\_HP | 1 | 7,564 | 1,00% | 7,564 | 7,564 | 1,56 | 0,226 |
| Error | 21 | 101,995 | 13,52% | 101,995 | 4,857 |  |  |
| Lack-of-Fit | 19 | 91,345 | 12,11% | 91,345 | 4,808 | 0,90 | 0,649 |
| Pure Error | 2 | 10,650 | 1,41% | 10,650 | 5,325 |  |  |
| Total | 26 | 754,308 | 100,00% |  |  |  |  |

## Regression Equation in Uncoded Units

|  |  |  |
| --- | --- | --- |
| F\_mean\_15h(900min) | = | 91,4 + 87,4 Lac - 0,00355 HPMC\_Visc - 4,64 HPMC\_HP - 0,00375 Lac\*HPMC\_Visc + 0,000525 HPMC\_Visc\*HPMC\_HP |

## Fits and Diagnostics for All Observations

| Obs | F\_mean\_15h(900min) | Fit | SE Fit | 95% CI | Resid | Std Resid | Del Resid |
| --- | --- | --- | --- | --- | --- | --- | --- |
| 1 | 80,606 | 79,520 | 1,199 | (77,026; 82,013) | 1,086 | 0,59 | 0,58 |
| 2 | 90,402 | 91,298 | 1,199 | (88,805; 93,792) | -0,897 | -0,49 | -0,48 |
| 3 | 73,720 | 77,378 | 1,532 | (74,193; 80,563) | -3,658 | -2,31 | -2,61 |
| 4 | 83,969 | 84,050 | 1,532 | (80,865; 87,235) | -0,081 | -0,05 | -0,05 |
| 5 | 77,633 | 80,481 | 0,994 | (78,415; 82,548) | -2,848 | -1,45 | -1,49 |
| 6 | 89,900 | 92,032 | 0,994 | (89,965; 94,098) | -2,132 | -1,08 | -1,09 |
| 7 | 80,197 | 82,194 | 1,535 | (79,000; 85,387) | -1,996 | -1,26 | -1,28 |
| 8 | 87,181 | 88,790 | 1,535 | (85,596; 91,983) | -1,609 | -1,02 | -1,02 |
| 9 | 76,944 | 79,534 | 1,144 | (77,156; 81,912) | -2,590 | -1,37 | -1,41 |
| 10 | 92,342 | 91,241 | 1,144 | (88,863; 93,619) | 1,101 | 0,58 | 0,57 |
| 11 | 83,565 | 78,922 | 0,817 | (77,224; 80,621) | 4,643 | 2,27 | 2,55 |
| 12 | 87,990 | 87,412 | 0,817 | (85,713; 89,111) | 0,578 | 0,28 | 0,28 |
| 13 | 80,335 | 80,666 | 1,198 | (78,175; 83,156) | -0,331 | -0,18 | -0,17 |
| 14 | 91,608 | 92,405 | 1,198 | (89,914; 94,895) | -0,797 | -0,43 | -0,42 |
| 15 | 81,597 | 80,825 | 0,857 | (79,043; 82,606) | 0,773 | 0,38 | 0,37 |
| 16 | 87,555 | 88,981 | 0,857 | (87,199; 90,762) | -1,426 | -0,70 | -0,69 |
| 17 | 75,884 | 75,224 | 1,003 | (73,138; 77,310) | 0,660 | 0,34 | 0,33 |
| 18 | 95,792 | 94,961 | 1,003 | (92,875; 97,047) | 0,831 | 0,42 | 0,41 |
| 19 | 89,091 | 86,614 | 0,819 | (84,912; 88,317) | 2,477 | 1,21 | 1,22 |
| 20 | 85,073 | 82,318 | 0,988 | (80,264; 84,372) | 2,755 | 1,40 | 1,43 |
| 21 | 82,700 | 83,086 | 0,890 | (81,236; 84,937) | -0,386 | -0,19 | -0,19 |
| 22 | 89,972 | 87,320 | 1,095 | (85,044; 89,597) | 2,652 | 1,39 | 1,42 |
| 23 | 85,860 | 85,190 | 0,441 | (84,273; 86,107) | 0,670 | 0,31 | 0,30 |
| 24 | 86,024 | 85,020 | 0,439 | (84,107; 85,934) | 1,004 | 0,46 | 0,46 |
| 25 | 82,743 | 85,093 | 0,433 | (84,192; 85,994) | -2,350 | -1,09 | -1,09 |
| 26 | 84,715 | 85,093 | 0,433 | (84,192; 85,994) | -0,377 | -0,17 | -0,17 |
| 27 | 87,342 | 85,093 | 0,433 | (84,192; 85,994) | 2,250 | 1,04 | 1,04 |

| Obs | HI | Cook’s D | DFITS |  |
| --- | --- | --- | --- | --- |
| 1 | 0,295939 | 0,02 | 0,37465 |  |
| 2 | 0,295939 | 0,02 | -0,30861 |  |
| 3 | 0,482953 | 0,83 | -2,51983 | R |
| 4 | 0,482953 | 0,00 | -0,04809 |  |
| 5 | 0,203286 | 0,09 | -0,75224 |  |
| 6 | 0,203286 | 0,05 | -0,54994 |  |
| 7 | 0,485441 | 0,25 | -1,24522 |  |
| 8 | 0,485441 | 0,16 | -0,98920 |  |
| 9 | 0,269274 | 0,12 | -0,85381 |  |
| 10 | 0,269274 | 0,02 | 0,34896 |  |
| 11 | 0,137369 | 0,14 | 1,01660 | R |
| 12 | 0,137369 | 0,00 | 0,11023 |  |
| 13 | 0,295333 | 0,00 | -0,11307 |  |
| 14 | 0,295333 | 0,01 | -0,27331 |  |
| 15 | 0,151077 | 0,00 | 0,15724 |  |
| 16 | 0,151077 | 0,01 | -0,29257 |  |
| 17 | 0,207151 | 0,00 | 0,16827 |  |
| 18 | 0,207151 | 0,01 | 0,21208 |  |
| 19 | 0,138002 | 0,04 | 0,49003 |  |
| 20 | 0,200816 | 0,08 | 0,71824 |  |
| 21 | 0,163018 | 0,00 | -0,08261 |  |
| 22 | 0,246731 | 0,10 | 0,81246 |  |
| 23 | 0,040065 | 0,00 | 0,06199 |  |
| 24 | 0,039756 | 0,00 | 0,09276 |  |
| 25 | 0,038655 | 0,01 | -0,21907 |  |
| 26 | 0,038655 | 0,00 | -0,03420 |  |
| 27 | 0,038655 | 0,01 | 0,20921 |  |

R  Large residual

## Forward Selection of Terms

α to enter = 0,25

## Coded Coefficients

| Term | Coef | SE Coef | 95% CI | T-Value | P-Value | VIF |
| --- | --- | --- | --- | --- | --- | --- |
| Constant | 87,162 | 0,439 | (86,250; 88,074) | 198,68 | 0,000 |  |
| Lac | 8,692 | 0,909 | (6,801; 10,582) | 9,56 | 0,000 | 1,08 |
| HPMC\_Visc | -1,540 | 0,804 | (-3,213; 0,133) | -1,91 | 0,069 | 1,11 |
| HPMC\_HP | 2,334 | 0,884 | (0,495; 4,173) | 2,64 | 0,015 | 1,04 |
| Lac\*HPMC\_Visc | -3,50 | 1,78 | (-7,20; 0,20) | -1,97 | 0,062 | 1,08 |
| HPMC\_Visc\*HPMC\_HP | 2,41 | 1,62 | (-0,96; 5,77) | 1,49 | 0,152 | 1,15 |

## Model Summary

| S | R-sq | R-sq(adj) | PRESS | R-sq(pred) | AICc | BIC |
| --- | --- | --- | --- | --- | --- | --- |
| 2,14691 | 85,81% | 82,44% | 186,866 | 72,61% | 130,99 | 134,17 |

## Analysis of Variance

| Source | DF | Seq SS | Contribution | Adj SS | Adj MS | F-Value | P-Value |
| --- | --- | --- | --- | --- | --- | --- | --- |
| Model | 5 | 585,472 | 85,81% | 585,472 | 117,094 | 25,40 | 0,000 |
| Linear | 3 | 557,419 | 81,70% | 470,841 | 156,947 | 34,05 | 0,000 |
| Lac | 1 | 504,288 | 73,91% | 421,165 | 421,165 | 91,37 | 0,000 |
| HPMC\_Visc | 1 | 26,365 | 3,86% | 16,890 | 16,890 | 3,66 | 0,069 |
| HPMC\_HP | 1 | 26,767 | 3,92% | 32,105 | 32,105 | 6,97 | 0,015 |
| 2-Way Interaction | 2 | 28,052 | 4,11% | 28,052 | 14,026 | 3,04 | 0,069 |
| Lac\*HPMC\_Visc | 1 | 17,858 | 2,62% | 17,858 | 17,858 | 3,87 | 0,062 |
| HPMC\_Visc\*HPMC\_HP | 1 | 10,194 | 1,49% | 10,194 | 10,194 | 2,21 | 0,152 |
| Error | 21 | 96,793 | 14,19% | 96,793 | 4,609 |  |  |
| Lack-of-Fit | 19 | 87,364 | 12,80% | 87,364 | 4,598 | 0,98 | 0,622 |
| Pure Error | 2 | 9,429 | 1,38% | 9,429 | 4,715 |  |  |
| Total | 26 | 682,265 | 100,00% |  |  |  |  |

## Regression Equation in Uncoded Units

|  |  |  |
| --- | --- | --- |
| F\_mean\_16h(960min) | = | 107,0 + 83,4 Lac - 0,00437 HPMC\_Visc - 5,93 HPMC\_HP - 0,00360 Lac\*HPMC\_Visc + 0,000609 HPMC\_Visc\*HPMC\_HP |

## Fits and Diagnostics for All Observations

| Obs | F\_mean\_16h(960min) | Fit | SE Fit | 95% CI | Resid | Std Resid | Del Resid |
| --- | --- | --- | --- | --- | --- | --- | --- |
| 1 | 82,777 | 82,218 | 1,168 | (79,790; 84,647) | 0,558 | 0,31 | 0,30 |
| 2 | 93,041 | 93,402 | 1,168 | (90,974; 95,831) | -0,362 | -0,20 | -0,20 |
| 3 | 76,241 | 79,961 | 1,492 | (76,858; 83,064) | -3,719 | -2,41 | -2,76 |
| 4 | 86,554 | 86,238 | 1,492 | (83,136; 89,341) | 0,316 | 0,20 | 0,20 |
| 5 | 80,143 | 82,838 | 0,968 | (80,825; 84,851) | -2,695 | -1,41 | -1,44 |
| 6 | 92,409 | 93,803 | 0,968 | (91,790; 95,816) | -1,393 | -0,73 | -0,72 |
| 7 | 83,119 | 84,884 | 1,496 | (81,773; 87,995) | -1,765 | -1,15 | -1,15 |
| 8 | 89,650 | 91,089 | 1,496 | (87,978; 94,199) | -1,439 | -0,93 | -0,93 |
| 9 | 79,338 | 82,216 | 1,114 | (79,899; 84,533) | -2,877 | -1,57 | -1,63 |
| 10 | 94,542 | 93,331 | 1,114 | (91,014; 95,648) | 1,211 | 0,66 | 0,65 |
| 11 | 86,168 | 81,528 | 0,796 | (79,873; 83,182) | 4,640 | 2,33 | 2,64 |
| 12 | 90,270 | 89,551 | 0,796 | (87,897; 91,206) | 0,718 | 0,36 | 0,35 |
| 13 | 82,722 | 82,933 | 1,167 | (80,507; 85,359) | -0,211 | -0,12 | -0,11 |
| 14 | 92,607 | 94,079 | 1,167 | (91,652; 96,505) | -1,472 | -0,82 | -0,81 |
| 15 | 83,948 | 83,395 | 0,834 | (81,660; 85,131) | 0,553 | 0,28 | 0,27 |
| 16 | 89,920 | 91,099 | 0,834 | (89,363; 92,834) | -1,179 | -0,60 | -0,59 |
| 17 | 78,580 | 78,001 | 0,977 | (75,968; 80,033) | 0,579 | 0,30 | 0,30 |
| 18 | 96,318 | 96,698 | 0,977 | (94,666; 98,730) | -0,379 | -0,20 | -0,19 |
| 19 | 91,500 | 88,707 | 0,798 | (87,049; 90,366) | 2,793 | 1,40 | 1,44 |
| 20 | 87,193 | 84,805 | 0,962 | (82,804; 86,806) | 2,388 | 1,24 | 1,26 |
| 21 | 85,260 | 85,540 | 0,867 | (83,738; 87,343) | -0,280 | -0,14 | -0,14 |
| 22 | 92,111 | 89,396 | 1,066 | (87,178; 91,613) | 2,716 | 1,46 | 1,50 |
| 23 | 87,826 | 87,436 | 0,430 | (86,542; 88,329) | 0,390 | 0,19 | 0,18 |
| 24 | 88,425 | 87,287 | 0,428 | (86,397; 88,178) | 1,138 | 0,54 | 0,53 |
| 25 | 85,154 | 87,349 | 0,422 | (86,471; 88,227) | -2,195 | -1,04 | -1,05 |
| 26 | 87,173 | 87,349 | 0,422 | (86,471; 88,227) | -0,176 | -0,08 | -0,08 |
| 27 | 89,493 | 87,349 | 0,422 | (86,471; 88,227) | 2,144 | 1,02 | 1,02 |

| Obs | HI | Cook’s D | DFITS |  |
| --- | --- | --- | --- | --- |
| 1 | 0,295939 | 0,01 | 0,19660 |  |
| 2 | 0,295939 | 0,00 | -0,12717 |  |
| 3 | 0,482953 | 0,90 | -2,67131 | R |
| 4 | 0,482953 | 0,01 | 0,19324 |  |
| 5 | 0,203286 | 0,08 | -0,72847 |  |
| 6 | 0,203286 | 0,02 | -0,36301 |  |
| 7 | 0,485441 | 0,21 | -1,12182 |  |
| 8 | 0,485441 | 0,14 | -0,90458 |  |
| 9 | 0,269274 | 0,15 | -0,98846 |  |
| 10 | 0,269274 | 0,03 | 0,39492 |  |
| 11 | 0,137369 | 0,14 | 1,05200 | R |
| 12 | 0,137369 | 0,00 | 0,14074 |  |
| 13 | 0,295333 | 0,00 | -0,07415 |  |
| 14 | 0,295333 | 0,05 | -0,52441 |  |
| 15 | 0,151077 | 0,00 | 0,11525 |  |
| 16 | 0,151077 | 0,01 | -0,24740 |  |
| 17 | 0,207151 | 0,00 | 0,15144 |  |
| 18 | 0,207151 | 0,00 | -0,09912 |  |
| 19 | 0,138002 | 0,05 | 0,57467 |  |
| 20 | 0,200816 | 0,06 | 0,63240 |  |
| 21 | 0,163018 | 0,00 | -0,06143 |  |
| 22 | 0,246731 | 0,12 | 0,85858 |  |
| 23 | 0,040065 | 0,00 | 0,03699 |  |
| 24 | 0,039756 | 0,00 | 0,10815 |  |
| 25 | 0,038655 | 0,01 | -0,20959 |  |
| 26 | 0,038655 | 0,00 | -0,01641 |  |
| 27 | 0,038655 | 0,01 | 0,20441 |  |

R  Large residual

## Forward Selection of Terms

α to enter = 0,25

## Coded Coefficients

| Term | Coef | SE Coef | 95% CI | T-Value | P-Value | VIF |
| --- | --- | --- | --- | --- | --- | --- |
| Constant | 89,156 | 0,446 | (88,229; 90,082) | 200,12 | 0,000 |  |
| Lac | 8,049 | 0,923 | (6,129; 9,969) | 8,72 | 0,000 | 1,08 |
| HPMC\_Visc | -1,469 | 0,817 | (-3,167; 0,230) | -1,80 | 0,087 | 1,11 |
| HPMC\_HP | 2,012 | 0,898 | (0,145; 3,880) | 2,24 | 0,036 | 1,04 |
| Lac\*HPMC\_Visc | -2,89 | 1,81 | (-6,65; 0,87) | -1,60 | 0,124 | 1,08 |
| HPMC\_Visc\*HPMC\_HP | 2,76 | 1,64 | (-0,66; 6,17) | 1,68 | 0,108 | 1,15 |

## Model Summary

| S | R-sq | R-sq(adj) | PRESS | R-sq(pred) | AICc | BIC |
| --- | --- | --- | --- | --- | --- | --- |
| 2,18018 | 83,30% | 79,32% | 191,929 | 67,89% | 131,82 | 135,00 |

## Analysis of Variance

| Source | DF | Seq SS | Contribution | Adj SS | Adj MS | F-Value | P-Value |
| --- | --- | --- | --- | --- | --- | --- | --- |
| Model | 5 | 497,901 | 83,30% | 497,901 | 99,580 | 20,95 | 0,000 |
| Linear | 3 | 472,325 | 79,02% | 400,972 | 133,657 | 28,12 | 0,000 |
| Lac | 1 | 427,608 | 71,54% | 361,175 | 361,175 | 75,99 | 0,000 |
| HPMC\_Visc | 1 | 26,277 | 4,40% | 15,366 | 15,366 | 3,23 | 0,087 |
| HPMC\_HP | 1 | 18,440 | 3,09% | 23,871 | 23,871 | 5,02 | 0,036 |
| 2-Way Interaction | 2 | 25,577 | 4,28% | 25,577 | 12,788 | 2,69 | 0,091 |
| Lac\*HPMC\_Visc | 1 | 12,178 | 2,04% | 12,178 | 12,178 | 2,56 | 0,124 |
| HPMC\_Visc\*HPMC\_HP | 1 | 13,398 | 2,24% | 13,398 | 13,398 | 2,82 | 0,108 |
| Error | 21 | 99,817 | 16,70% | 99,817 | 4,753 |  |  |
| Lack-of-Fit | 19 | 91,312 | 15,28% | 91,312 | 4,806 | 1,13 | 0,571 |
| Pure Error | 2 | 8,505 | 1,42% | 8,505 | 4,253 |  |  |
| Total | 26 | 597,718 | 100,00% |  |  |  |  |

## Regression Equation in Uncoded Units

|  |  |  |
| --- | --- | --- |
| F\_mean\_17h(1020min) | = | 128,7 + 72,4 Lac - 0,00551 HPMC\_Visc - 7,45 HPMC\_HP - 0,00297 Lac\*HPMC\_Visc + 0,000698 HPMC\_Visc\*HPMC\_HP |

## Fits and Diagnostics for All Observations

| Obs | F\_mean\_17h(1020min) | Fit | SE Fit | 95% CI | Resid | Std Resid | Del Resid |
| --- | --- | --- | --- | --- | --- | --- | --- |
| 1 | 86,287 | 85,112 | 1,186 | (82,646; 87,579) | 1,174 | 0,64 | 0,63 |
| 2 | 94,993 | 95,219 | 1,186 | (92,753; 97,686) | -0,226 | -0,12 | -0,12 |
| 3 | 78,511 | 82,173 | 1,515 | (79,023; 85,324) | -3,663 | -2,34 | -2,65 |
| 4 | 88,721 | 88,229 | 1,515 | (85,078; 91,380) | 0,492 | 0,31 | 0,31 |
| 5 | 82,549 | 85,192 | 0,983 | (83,148; 87,236) | -2,643 | -1,36 | -1,39 |
| 6 | 94,030 | 95,118 | 0,983 | (93,074; 97,162) | -1,088 | -0,56 | -0,55 |
| 7 | 85,468 | 86,999 | 1,519 | (83,840; 90,158) | -1,531 | -0,98 | -0,98 |
| 8 | 91,504 | 92,994 | 1,519 | (89,835; 96,153) | -1,490 | -0,95 | -0,95 |
| 9 | 81,530 | 85,077 | 1,131 | (82,724; 87,430) | -3,547 | -1,90 | -2,04 |
| 10 | 96,321 | 95,127 | 1,131 | (92,774; 97,480) | 1,194 | 0,64 | 0,63 |
| 11 | 88,495 | 83,919 | 0,808 | (82,239; 85,599) | 4,576 | 2,26 | 2,53 |
| 12 | 92,370 | 91,416 | 0,808 | (89,736; 93,097) | 0,954 | 0,47 | 0,46 |
| 13 | 84,957 | 85,180 | 1,185 | (82,716; 87,644) | -0,223 | -0,12 | -0,12 |
| 14 | 93,700 | 95,255 | 1,185 | (92,791; 97,719) | -1,555 | -0,85 | -0,84 |
| 15 | 86,046 | 85,606 | 0,847 | (83,844; 87,368) | 0,440 | 0,22 | 0,21 |
| 16 | 91,962 | 92,839 | 0,847 | (91,076; 94,601) | -0,877 | -0,44 | -0,43 |
| 17 | 80,949 | 80,759 | 0,992 | (78,695; 82,823) | 0,190 | 0,10 | 0,10 |
| 18 | 96,721 | 97,942 | 0,992 | (95,878; 100,005) | -1,221 | -0,63 | -0,62 |
| 19 | 93,609 | 90,679 | 0,810 | (88,995; 92,364) | 2,930 | 1,45 | 1,49 |
| 20 | 89,036 | 86,865 | 0,977 | (84,833; 88,896) | 2,172 | 1,11 | 1,12 |
| 21 | 87,569 | 87,906 | 0,880 | (86,075; 89,737) | -0,337 | -0,17 | -0,17 |
| 22 | 93,783 | 91,019 | 1,083 | (88,767; 93,271) | 2,764 | 1,46 | 1,50 |
| 23 | 89,589 | 89,441 | 0,436 | (88,534; 90,349) | 0,147 | 0,07 | 0,07 |
| 24 | 90,600 | 89,333 | 0,435 | (88,429; 90,237) | 1,267 | 0,59 | 0,58 |
| 25 | 87,288 | 89,350 | 0,429 | (88,459; 90,242) | -2,063 | -0,96 | -0,96 |
| 26 | 89,453 | 89,350 | 0,429 | (88,459; 90,242) | 0,103 | 0,05 | 0,05 |
| 27 | 91,410 | 89,350 | 0,429 | (88,459; 90,242) | 2,060 | 0,96 | 0,96 |

| Obs | HI | Cook’s D | DFITS |  |
| --- | --- | --- | --- | --- |
| 1 | 0,295939 | 0,03 | 0,41025 |  |
| 2 | 0,295939 | 0,00 | -0,07819 |  |
| 3 | 0,482953 | 0,85 | -2,56136 | R |
| 4 | 0,482953 | 0,02 | 0,29682 |  |
| 5 | 0,203286 | 0,08 | -0,70102 |  |
| 6 | 0,203286 | 0,01 | -0,27760 |  |
| 7 | 0,485441 | 0,15 | -0,95005 |  |
| 8 | 0,485441 | 0,14 | -0,92310 |  |
| 9 | 0,269274 | 0,22 | -1,23929 |  |
| 10 | 0,269274 | 0,03 | 0,38316 |  |
| 11 | 0,137369 | 0,14 | 1,01158 | R |
| 12 | 0,137369 | 0,01 | 0,18448 |  |
| 13 | 0,295333 | 0,00 | -0,07706 |  |
| 14 | 0,295333 | 0,05 | -0,54622 |  |
| 15 | 0,151077 | 0,00 | 0,09033 |  |
| 16 | 0,151077 | 0,01 | -0,18052 |  |
| 17 | 0,207151 | 0,00 | 0,04874 |  |
| 18 | 0,207151 | 0,02 | -0,31669 |  |
| 19 | 0,138002 | 0,06 | 0,59568 |  |
| 20 | 0,200816 | 0,05 | 0,56201 |  |
| 21 | 0,163018 | 0,00 | -0,07290 |  |
| 22 | 0,246731 | 0,12 | 0,86082 |  |
| 23 | 0,040065 | 0,00 | 0,01376 |  |
| 24 | 0,039756 | 0,00 | 0,11872 |  |
| 25 | 0,038655 | 0,01 | -0,19316 |  |
| 26 | 0,038655 | 0,00 | 0,00941 |  |
| 27 | 0,038655 | 0,01 | 0,19288 |  |

R  Large residual

## Forward Selection of Terms

α to enter = 0,25

## Coded Coefficients

| Term | Coef | SE Coef | 95% CI | T-Value | P-Value | VIF |
| --- | --- | --- | --- | --- | --- | --- |
| Constant | 91,369 | 0,574 | (90,172; 92,565) | 159,30 | 0,000 |  |
| Lac | 7,389 | 0,910 | (5,490; 9,288) | 8,12 | 0,000 | 1,08 |
| HPMC\_Visc | -1,337 | 0,805 | (-3,017; 0,343) | -1,66 | 0,113 | 1,11 |
| HPMC\_HP | 1,737 | 0,885 | (-0,110; 3,584) | 1,96 | 0,064 | 1,04 |
| Lac\*Lac | -2,24 | 1,67 | (-5,71; 1,23) | -1,35 | 0,193 | 1,00 |
| Lac\*HPMC\_Visc | -2,77 | 1,78 | (-6,48; 0,95) | -1,55 | 0,136 | 1,08 |
| HPMC\_Visc\*HPMC\_HP | 2,97 | 1,62 | (-0,41; 6,35) | 1,83 | 0,082 | 1,15 |

## Model Summary

| S | R-sq | R-sq(adj) | PRESS | R-sq(pred) | AICc | BIC |
| --- | --- | --- | --- | --- | --- | --- |
| 2,14977 | 82,41% | 77,13% | 193,631 | 63,15% | 133,85 | 136,22 |

## Analysis of Variance

| Source | DF | Seq SS | Contribution | Adj SS | Adj MS | F-Value | P-Value |
| --- | --- | --- | --- | --- | --- | --- | --- |
| Model | 6 | 433,041 | 82,41% | 433,041 | 72,174 | 15,62 | 0,000 |
| Linear | 3 | 398,179 | 75,78% | 335,359 | 111,786 | 24,19 | 0,000 |
| Lac | 1 | 361,840 | 68,86% | 304,404 | 304,404 | 65,87 | 0,000 |
| HPMC\_Visc | 1 | 23,561 | 4,48% | 12,734 | 12,734 | 2,76 | 0,113 |
| HPMC\_HP | 1 | 12,778 | 2,43% | 17,781 | 17,781 | 3,85 | 0,064 |
| Square | 1 | 8,177 | 1,56% | 8,368 | 8,368 | 1,81 | 0,193 |
| Lac\*Lac | 1 | 8,177 | 1,56% | 8,368 | 8,368 | 1,81 | 0,193 |
| 2-Way Interaction | 2 | 26,686 | 5,08% | 26,686 | 13,343 | 2,89 | 0,079 |
| Lac\*HPMC\_Visc | 1 | 11,166 | 2,12% | 11,166 | 11,166 | 2,42 | 0,136 |
| HPMC\_Visc\*HPMC\_HP | 1 | 15,520 | 2,95% | 15,520 | 15,520 | 3,36 | 0,082 |
| Error | 20 | 92,430 | 17,59% | 92,430 | 4,622 |  |  |
| Lack-of-Fit | 18 | 84,578 | 16,10% | 84,578 | 4,699 | 1,20 | 0,550 |
| Pure Error | 2 | 7,852 | 1,49% | 7,852 | 3,926 |  |  |
| Total | 26 | 525,472 | 100,00% |  |  |  |  |

## Regression Equation in Uncoded Units

|  |  |  |
| --- | --- | --- |
| F\_mean\_18h(1080min) | = | 133,0 + 103,9 Lac - 0,00604 HPMC\_Visc - 8,44 HPMC\_HP - 35,9 Lac\*Lac - 0,00285 Lac\*HPMC\_Visc + 0,000752 HPMC\_Visc\*HPMC\_HP |

## Fits and Diagnostics for All Observations

| Obs | F\_mean\_18h(1080min) | Fit | SE Fit | 95% CI | Resid | Std Resid | Del Resid |
| --- | --- | --- | --- | --- | --- | --- | --- |
| 1 | 88,26 | 87,35 | 1,17 | (84,91; 89,79) | 0,91 | 0,51 | 0,50 |
| 2 | 96,84 | 96,71 | 1,17 | (94,27; 99,15) | 0,13 | 0,07 | 0,07 |
| 3 | 80,68 | 84,30 | 1,50 | (81,18; 87,42) | -3,63 | -2,35 | -2,69 |
| 4 | 90,40 | 89,78 | 1,50 | (86,66; 92,90) | 0,62 | 0,40 | 0,39 |
| 5 | 84,58 | 87,04 | 0,97 | (85,02; 89,07) | -2,46 | -1,28 | -1,31 |
| 6 | 95,18 | 96,23 | 0,97 | (94,21; 98,26) | -1,05 | -0,55 | -0,54 |
| 7 | 87,83 | 88,97 | 1,50 | (85,85; 92,10) | -1,14 | -0,74 | -0,73 |
| 8 | 92,85 | 94,39 | 1,50 | (91,27; 97,52) | -1,55 | -1,00 | -1,00 |
| 9 | 83,55 | 87,30 | 1,12 | (84,97; 89,63) | -3,75 | -2,04 | -2,24 |
| 10 | 97,78 | 96,60 | 1,12 | (94,27; 98,93) | 1,17 | 0,64 | 0,63 |
| 11 | 90,55 | 86,03 | 0,80 | (84,36; 87,69) | 4,52 | 2,26 | 2,56 |
| 12 | 94,34 | 92,89 | 0,80 | (91,22; 94,55) | 1,45 | 0,73 | 0,72 |
| 13 | 86,94 | 86,94 | 1,17 | (84,50; 89,38) | 0,00 | 0,00 | 0,00 |
| 14 | 94,59 | 96,27 | 1,17 | (93,83; 98,71) | -1,68 | -0,93 | -0,93 |
| 15 | 87,89 | 87,57 | 0,84 | (85,83; 89,32) | 0,32 | 0,16 | 0,16 |
| 16 | 93,80 | 94,18 | 0,84 | (92,43; 95,92) | -0,38 | -0,19 | -0,19 |
| 17 | 83,21 | 81,41 | 1,62 | (78,02; 84,79) | 1,80 | 1,28 | 1,30 |
| 18 | 97,05 | 97,22 | 1,62 | (93,84; 100,61) | -0,17 | -0,12 | -0,12 |
| 19 | 95,40 | 92,80 | 0,88 | (90,96; 94,64) | 2,60 | 1,33 | 1,35 |
| 20 | 90,56 | 89,22 | 1,03 | (87,07; 91,37) | 1,33 | 0,71 | 0,70 |
| 21 | 89,65 | 90,41 | 0,95 | (88,44; 92,38) | -0,76 | -0,39 | -0,38 |
| 22 | 95,01 | 92,92 | 1,13 | (90,56; 95,27) | 2,10 | 1,15 | 1,16 |
| 23 | 91,01 | 91,64 | 0,57 | (90,46; 92,83) | -0,64 | -0,31 | -0,30 |
| 24 | 92,52 | 91,57 | 0,57 | (90,39; 92,75) | 0,96 | 0,46 | 0,45 |
| 25 | 89,22 | 91,56 | 0,56 | (90,38; 92,73) | -2,34 | -1,13 | -1,13 |
| 26 | 91,58 | 91,56 | 0,56 | (90,38; 92,73) | 0,03 | 0,01 | 0,01 |
| 27 | 93,16 | 91,56 | 0,56 | (90,38; 92,73) | 1,60 | 0,77 | 0,76 |

| Obs | HI | Cook’s D | DFITS |  |
| --- | --- | --- | --- | --- |
| 1 | 0,296213 | 0,02 | 0,32158 |  |
| 2 | 0,296213 | 0,00 | 0,04548 |  |
| 3 | 0,483632 | 0,74 | -2,60192 | R |
| 4 | 0,483632 | 0,02 | 0,37780 |  |
| 5 | 0,203762 | 0,06 | -0,66100 |  |
| 6 | 0,203762 | 0,01 | -0,27165 |  |
| 7 | 0,485925 | 0,07 | -0,71229 |  |
| 8 | 0,485925 | 0,14 | -0,97704 |  |
| 9 | 0,269560 | 0,22 | -1,35920 | R |
| 10 | 0,269560 | 0,02 | 0,38222 |  |
| 11 | 0,137879 | 0,12 | 1,02309 | R |
| 12 | 0,137879 | 0,01 | 0,28769 |  |
| 13 | 0,295857 | 0,00 | 0,00035 |  |
| 14 | 0,295857 | 0,05 | -0,60023 |  |
| 15 | 0,151588 | 0,00 | 0,06725 |  |
| 16 | 0,151588 | 0,00 | -0,07870 |  |
| 17 | 0,570267 | 0,31 | 1,49813 |  |
| 18 | 0,570267 | 0,00 | -0,13904 |  |
| 19 | 0,168356 | 0,05 | 0,60889 |  |
| 20 | 0,229212 | 0,02 | 0,38074 |  |
| 21 | 0,193350 | 0,01 | -0,18831 |  |
| 22 | 0,275624 | 0,07 | 0,71305 |  |
| 23 | 0,069753 | 0,00 | -0,08234 |  |
| 24 | 0,069548 | 0,00 | 0,12356 |  |
| 25 | 0,068263 | 0,01 | -0,30685 |  |
| 26 | 0,068263 | 0,00 | 0,00329 |  |
| 27 | 0,068263 | 0,01 | 0,20663 |  |

R  Large residual

## Forward Selection of Terms

α to enter = 0,25

## Coded Coefficients

| Term | Coef | SE Coef | 95% CI | T-Value | P-Value | VIF |
| --- | --- | --- | --- | --- | --- | --- |
| Constant | 92,833 | 0,571 | (91,639; 94,028) | 162,66 | 0,000 |  |
| Lac | 6,395 | 0,944 | (4,420; 8,370) | 6,78 | 0,000 | 1,17 |
| HPMC\_Visc | -1,130 | 0,801 | (-2,808; 0,547) | -1,41 | 0,175 | 1,11 |
| HPMC\_HP | 1,434 | 0,881 | (-0,410; 3,278) | 1,63 | 0,120 | 1,04 |
| Lac\*Lac | -2,58 | 1,66 | (-6,05; 0,89) | -1,56 | 0,136 | 1,00 |
| Lac\*HPMC\_Visc | -2,33 | 1,78 | (-6,05; 1,39) | -1,31 | 0,205 | 1,08 |
| Lac\*HPMC\_HP | -2,61 | 2,12 | (-7,05; 1,83) | -1,23 | 0,234 | 1,11 |
| HPMC\_Visc\*HPMC\_HP | 2,93 | 1,61 | (-0,44; 6,30) | 1,82 | 0,085 | 1,15 |

## Model Summary

| S | R-sq | R-sq(adj) | PRESS | R-sq(pred) | AICc | BIC |
| --- | --- | --- | --- | --- | --- | --- |
| 2,13908 | 80,85% | 73,80% | 203,072 | 55,27% | 136,78 | 137,86 |

## Analysis of Variance

| Source | DF | Seq SS | Contribution | Adj SS | Adj MS | F-Value | P-Value |
| --- | --- | --- | --- | --- | --- | --- | --- |
| Model | 7 | 367,083 | 80,85% | 367,083 | 52,440 | 11,46 | 0,000 |
| Linear | 3 | 325,192 | 71,62% | 231,668 | 77,223 | 16,88 | 0,000 |
| Lac | 1 | 298,769 | 65,81% | 210,146 | 210,146 | 45,93 | 0,000 |
| HPMC\_Visc | 1 | 18,375 | 4,05% | 9,100 | 9,100 | 1,99 | 0,175 |
| HPMC\_HP | 1 | 8,048 | 1,77% | 12,115 | 12,115 | 2,65 | 0,120 |
| Square | 1 | 10,865 | 2,39% | 11,082 | 11,082 | 2,42 | 0,136 |
| Lac\*Lac | 1 | 10,865 | 2,39% | 11,082 | 11,082 | 2,42 | 0,136 |
| 2-Way Interaction | 3 | 31,027 | 6,83% | 31,027 | 10,342 | 2,26 | 0,114 |
| Lac\*HPMC\_Visc | 1 | 8,954 | 1,97% | 7,872 | 7,872 | 1,72 | 0,205 |
| Lac\*HPMC\_HP | 1 | 6,923 | 1,52% | 6,923 | 6,923 | 1,51 | 0,234 |
| HPMC\_Visc\*HPMC\_HP | 1 | 15,149 | 3,34% | 15,149 | 15,149 | 3,31 | 0,085 |
| Error | 19 | 86,937 | 19,15% | 86,937 | 4,576 |  |  |
| Lack-of-Fit | 17 | 79,349 | 17,48% | 79,349 | 4,668 | 1,23 | 0,540 |
| Pure Error | 2 | 7,588 | 1,67% | 7,588 | 3,794 |  |  |
| Total | 26 | 454,020 | 100,00% |  |  |  |  |

## Regression Equation in Uncoded Units

|  |  |  |
| --- | --- | --- |
| F\_mean\_19h(1140min) | = | 90,4 + 196,7 Lac - 0,00613 HPMC\_Visc - 3,47 HPMC\_HP - 41,3 Lac\*Lac - 0,00240 Lac\*HPMC\_Visc - 10,29 Lac\*HPMC\_HP + 0,000743 HPMC\_Visc\*HPMC\_HP |

## Fits and Diagnostics for All Observations

| Obs | F\_mean\_19h(1140min) | Fit | SE Fit | 95% CI | Resid | Std Resid | Del Resid |
| --- | --- | --- | --- | --- | --- | --- | --- |
| 1 | 89,69 | 88,50 | 1,31 | (85,76; 91,23) | 1,20 | 0,71 | 0,70 |
| 2 | 97,82 | 98,43 | 1,31 | (95,70; 101,16) | -0,62 | -0,36 | -0,36 |
| 3 | 82,74 | 85,44 | 1,65 | (81,99; 88,88) | -2,70 | -1,98 | -2,16 |
| 4 | 91,74 | 92,19 | 1,65 | (88,74; 95,64) | -0,45 | -0,33 | -0,32 |
| 5 | 86,12 | 89,14 | 1,04 | (86,96; 91,32) | -3,02 | -1,62 | -1,70 |
| 6 | 96,01 | 96,51 | 1,04 | (94,33; 98,69) | -0,50 | -0,27 | -0,26 |
| 7 | 89,48 | 91,30 | 1,60 | (87,95; 94,66) | -1,82 | -1,29 | -1,31 |
| 8 | 93,94 | 94,80 | 1,60 | (91,45; 98,16) | -0,86 | -0,61 | -0,60 |
| 9 | 85,34 | 88,48 | 1,24 | (85,89; 91,08) | -3,15 | -1,81 | -1,93 |
| 10 | 98,82 | 98,27 | 1,24 | (95,68; 100,86) | 0,55 | 0,31 | 0,31 |
| 11 | 92,47 | 87,43 | 0,88 | (85,58; 89,28) | 5,04 | 2,59 | 3,13 |
| 12 | 95,75 | 94,62 | 0,88 | (92,77; 96,47) | 1,13 | 0,58 | 0,57 |
| 13 | 88,65 | 89,25 | 1,31 | (86,50; 91,99) | -0,59 | -0,35 | -0,34 |
| 14 | 94,93 | 96,21 | 1,31 | (93,46; 98,96) | -1,28 | -0,76 | -0,75 |
| 15 | 89,40 | 89,62 | 0,88 | (87,76; 91,47) | -0,22 | -0,11 | -0,11 |
| 16 | 95,49 | 94,90 | 0,88 | (93,05; 96,75) | 0,58 | 0,30 | 0,29 |
| 17 | 85,24 | 83,45 | 1,62 | (80,05; 86,85) | 1,79 | 1,29 | 1,31 |
| 18 | 97,28 | 97,39 | 1,62 | (93,99; 100,79) | -0,11 | -0,08 | -0,08 |
| 19 | 96,84 | 94,07 | 0,88 | (92,24; 95,91) | 2,77 | 1,42 | 1,46 |
| 20 | 91,99 | 90,95 | 1,02 | (88,81; 93,09) | 1,04 | 0,56 | 0,54 |
| 21 | 91,34 | 92,13 | 0,94 | (90,16; 94,10) | -0,80 | -0,41 | -0,41 |
| 22 | 95,78 | 94,06 | 1,12 | (91,71; 96,41) | 1,72 | 0,95 | 0,94 |
| 23 | 92,35 | 93,08 | 0,56 | (91,89; 94,26) | -0,73 | -0,35 | -0,35 |
| 24 | 94,13 | 93,03 | 0,56 | (91,85; 94,21) | 1,10 | 0,53 | 0,52 |
| 25 | 90,83 | 93,00 | 0,56 | (91,83; 94,17) | -2,17 | -1,05 | -1,05 |
| 26 | 93,46 | 93,00 | 0,56 | (91,83; 94,17) | 0,46 | 0,22 | 0,22 |
| 27 | 94,64 | 93,00 | 0,56 | (91,83; 94,17) | 1,64 | 0,79 | 0,79 |

| Obs | HI | Cook’s D | DFITS |  |
| --- | --- | --- | --- | --- |
| 1 | 0,372323 | 0,04 | 0,53639 |  |
| 2 | 0,372323 | 0,01 | -0,27397 |  |
| 3 | 0,593111 | 0,71 | -2,60904 |  |
| 4 | 0,593111 | 0,02 | -0,39093 |  |
| 5 | 0,236855 | 0,10 | -0,94440 |  |
| 6 | 0,236855 | 0,00 | -0,14493 |  |
| 7 | 0,562661 | 0,27 | -1,48610 |  |
| 8 | 0,562661 | 0,06 | -0,68132 |  |
| 9 | 0,335212 | 0,21 | -1,37132 |  |
| 10 | 0,335212 | 0,01 | 0,21790 |  |
| 11 | 0,170225 | 0,17 | 1,41692 | R |
| 12 | 0,170225 | 0,01 | 0,25782 |  |
| 13 | 0,376616 | 0,01 | -0,26675 |  |
| 14 | 0,376616 | 0,04 | -0,58312 |  |
| 15 | 0,170976 | 0,00 | -0,05009 |  |
| 16 | 0,170976 | 0,00 | 0,13300 |  |
| 17 | 0,576706 | 0,28 | 1,53291 |  |
| 18 | 0,576706 | 0,00 | -0,09301 |  |
| 19 | 0,168356 | 0,05 | 0,65650 |  |
| 20 | 0,229212 | 0,01 | 0,29704 |  |
| 21 | 0,193350 | 0,01 | -0,19839 |  |
| 22 | 0,275624 | 0,04 | 0,58277 |  |
| 23 | 0,069753 | 0,00 | -0,09461 |  |
| 24 | 0,069548 | 0,00 | 0,14283 |  |
| 25 | 0,068263 | 0,01 | -0,28479 |  |
| 26 | 0,068263 | 0,00 | 0,05912 |  |
| 27 | 0,068263 | 0,01 | 0,21251 |  |

R  Large residual

## Forward Selection of Terms

α to enter = 0,25

## Coded Coefficients

| Term | Coef | SE Coef | 95% CI | T-Value | P-Value | VIF |
| --- | --- | --- | --- | --- | --- | --- |
| Constant | 94,115 | 0,586 | (92,902; 95,328) | 160,51 | 0,000 |  |
| Lac | 6,285 | 0,927 | (4,367; 8,203) | 6,78 | 0,000 | 1,00 |
| HPMC\_PS | 1,99 | 1,09 | (-0,27; 4,25) | 1,82 | 0,081 | 1,00 |
| Lac\*Lac | -2,65 | 1,76 | (-6,29; 0,99) | -1,51 | 0,145 | 1,00 |

## Model Summary

| S | R-sq | R-sq(adj) | PRESS | R-sq(pred) | AICc | BIC |
| --- | --- | --- | --- | --- | --- | --- |
| 2,27078 | 69,16% | 65,13% | 167,547 | 56,43% | 129,44 | 133,06 |

## Analysis of Variance

| Source | DF | Seq SS | Contribution | Adj SS | Adj MS | F-Value | P-Value |
| --- | --- | --- | --- | --- | --- | --- | --- |
| Model | 3 | 265,929 | 69,16% | 265,929 | 88,643 | 17,19 | 0,000 |
| Linear | 2 | 254,217 | 66,11% | 254,134 | 127,067 | 24,64 | 0,000 |
| Lac | 1 | 237,011 | 61,64% | 237,011 | 237,011 | 45,96 | 0,000 |
| HPMC\_PS | 1 | 17,206 | 4,47% | 17,123 | 17,123 | 3,32 | 0,081 |
| Square | 1 | 11,712 | 3,05% | 11,712 | 11,712 | 2,27 | 0,145 |
| Lac\*Lac | 1 | 11,712 | 3,05% | 11,712 | 11,712 | 2,27 | 0,145 |
| Error | 23 | 118,598 | 30,84% | 118,598 | 5,156 |  |  |
| Lack-of-Fit | 21 | 111,108 | 28,89% | 111,108 | 5,291 | 1,41 | 0,496 |
| Pure Error | 2 | 7,490 | 1,95% | 7,490 | 3,745 |  |  |
| Total | 26 | 384,527 | 100,00% |  |  |  |  |

## Regression Equation in Uncoded Units

|  |  |  |
| --- | --- | --- |
| F\_mean\_20h(1200min) | = | 52,1 + 67,6 Lac + 0,271 HPMC\_PS - 42,4 Lac\*Lac |

## Fits and Diagnostics for All Observations

| Obs | F\_mean\_20h(1200min) | Fit | SE Fit | 95% CI | Resid | Std Resid | Del Resid |
| --- | --- | --- | --- | --- | --- | --- | --- |
| 1 | 91,09 | 89,82 | 0,69 | (88,39; 91,25) | 1,27 | 0,59 | 0,58 |
| 2 | 98,57 | 96,10 | 0,69 | (94,67; 97,53) | 2,47 | 1,14 | 1,15 |
| 3 | 84,65 | 89,16 | 0,89 | (87,31; 91,00) | -4,51 | -2,16 | -2,37 |
| 4 | 92,73 | 95,44 | 0,89 | (93,60; 97,29) | -2,71 | -1,30 | -1,32 |
| 5 | 87,77 | 89,97 | 0,66 | (88,60; 91,34) | -2,19 | -1,01 | -1,01 |
| 6 | 96,43 | 96,25 | 0,66 | (94,88; 97,63) | 0,17 | 0,08 | 0,08 |
| 7 | 91,14 | 89,53 | 0,76 | (87,96; 91,11) | 1,61 | 0,75 | 0,75 |
| 8 | 94,77 | 95,82 | 0,76 | (94,24; 97,40) | -1,05 | -0,49 | -0,48 |
| 9 | 86,97 | 91,14 | 0,79 | (89,50; 92,77) | -4,16 | -1,95 | -2,09 |
| 10 | 99,59 | 97,42 | 0,79 | (95,79; 99,05) | 2,17 | 1,02 | 1,02 |
| 11 | 94,14 | 90,73 | 0,68 | (89,32; 92,14) | 3,41 | 1,58 | 1,63 |
| 12 | 96,67 | 97,01 | 0,68 | (95,60; 98,43) | -0,34 | -0,16 | -0,16 |
| 13 | 90,12 | 91,35 | 0,86 | (89,56; 93,13) | -1,23 | -0,59 | -0,58 |
| 14 | 95,06 | 97,63 | 0,86 | (95,85; 99,41) | -2,57 | -1,22 | -1,24 |
| 15 | 90,72 | 90,84 | 0,71 | (89,38; 92,30) | -0,12 | -0,06 | -0,05 |
| 16 | 96,90 | 97,13 | 0,71 | (95,67; 98,59) | -0,22 | -0,10 | -0,10 |
| 17 | 87,07 | 85,11 | 1,71 | (81,57; 88,65) | 1,96 | 1,31 | 1,33 |
| 18 | 97,73 | 97,68 | 1,71 | (94,14; 101,22) | 0,05 | 0,03 | 0,03 |
| 19 | 97,92 | 94,30 | 0,60 | (93,07; 95,53) | 3,62 | 1,65 | 1,72 |
| 20 | 93,23 | 93,84 | 0,60 | (92,59; 95,09) | -0,61 | -0,28 | -0,27 |
| 21 | 92,80 | 93,91 | 0,60 | (92,67; 95,14) | -1,11 | -0,51 | -0,50 |
| 22 | 96,33 | 94,22 | 0,59 | (93,00; 95,44) | 2,11 | 0,96 | 0,96 |
| 23 | 93,32 | 92,12 | 1,23 | (89,57; 94,68) | 1,20 | 0,63 | 0,62 |
| 24 | 95,63 | 96,11 | 1,25 | (93,53; 98,69) | -0,48 | -0,25 | -0,25 |
| 25 | 92,29 | 94,05 | 0,59 | (92,83; 95,26) | -1,76 | -0,80 | -0,79 |
| 26 | 95,16 | 94,05 | 0,59 | (92,83; 95,26) | 1,12 | 0,51 | 0,50 |
| 27 | 95,97 | 94,05 | 0,59 | (92,83; 95,26) | 1,93 | 0,88 | 0,87 |

| Obs | HI | Cook’s D | DFITS |  |  |
| --- | --- | --- | --- | --- | --- |
| 1 | 0,092405 | 0,01 | 0,18491 |  |  |
| 2 | 0,092405 | 0,03 | 0,36653 |  |  |
| 3 | 0,154489 | 0,21 | -1,01199 | R |  |
| 4 | 0,154489 | 0,08 | -0,56461 |  |  |
| 5 | 0,085364 | 0,02 | -0,30864 |  |  |
| 6 | 0,085364 | 0,00 | 0,02382 |  |  |
| 7 | 0,112838 | 0,02 | 0,26589 |  |  |
| 8 | 0,112838 | 0,01 | -0,17268 |  |  |
| 9 | 0,120630 | 0,13 | -0,77547 |  |  |
| 10 | 0,120630 | 0,04 | 0,37705 |  |  |
| 11 | 0,090283 | 0,06 | 0,51414 |  |  |
| 12 | 0,090283 | 0,00 | -0,04900 |  |  |
| 13 | 0,143796 | 0,01 | -0,23648 |  |  |
| 14 | 0,143796 | 0,06 | -0,50664 |  |  |
| 15 | 0,096712 | 0,00 | -0,01761 |  |  |
| 16 | 0,096712 | 0,00 | -0,03311 |  |  |
| 17 | 0,566788 | 0,56 | 1,52383 |  | X |
| 18 | 0,566788 | 0,00 | 0,03441 |  | X |
| 19 | 0,068996 | 0,05 | 0,46784 |  |  |
| 20 | 0,070758 | 0,00 | -0,07502 |  |  |
| 21 | 0,068883 | 0,00 | -0,13528 |  |  |
| 22 | 0,067544 | 0,02 | 0,25833 |  |  |
| 23 | 0,295031 | 0,04 | 0,40058 |  |  |
| 24 | 0,301653 | 0,01 | -0,16287 |  |  |
| 25 | 0,066842 | 0,01 | -0,21245 |  |  |
| 26 | 0,066842 | 0,00 | 0,13404 |  |  |
| 27 | 0,066842 | 0,01 | 0,23394 |  |  |

R  Large residual  
X  Unusual X

## Forward Selection of Terms

α to enter = 0,25

## Coded Coefficients

| Term | Coef | SE Coef | 95% CI | T-Value | P-Value | VIF |
| --- | --- | --- | --- | --- | --- | --- |
| Constant | 95,169 | 0,581 | (93,967; 96,371) | 163,75 | 0,000 |  |
| Lac | 5,434 | 0,919 | (3,533; 7,335) | 5,91 | 0,000 | 1,00 |
| HPMC\_PS | 1,89 | 1,08 | (-0,35; 4,13) | 1,74 | 0,094 | 1,00 |
| Lac\*Lac | -2,88 | 1,74 | (-6,49; 0,72) | -1,65 | 0,112 | 1,00 |

## Model Summary

| S | R-sq | R-sq(adj) | PRESS | R-sq(pred) | AICc | BIC |
| --- | --- | --- | --- | --- | --- | --- |
| 2,25077 | 63,93% | 59,23% | 163,884 | 49,27% | 128,96 | 132,58 |

## Analysis of Variance

| Source | DF | Seq SS | Contribution | Adj SS | Adj MS | F-Value | P-Value |
| --- | --- | --- | --- | --- | --- | --- | --- |
| Model | 3 | 206,520 | 63,93% | 206,520 | 68,840 | 13,59 | 0,000 |
| Linear | 2 | 192,678 | 59,65% | 192,593 | 96,297 | 19,01 | 0,000 |
| Lac | 1 | 177,182 | 54,85% | 177,182 | 177,182 | 34,97 | 0,000 |
| HPMC\_PS | 1 | 15,496 | 4,80% | 15,411 | 15,411 | 3,04 | 0,094 |
| Square | 1 | 13,842 | 4,29% | 13,842 | 13,842 | 2,73 | 0,112 |
| Lac\*Lac | 1 | 13,842 | 4,29% | 13,842 | 13,842 | 2,73 | 0,112 |
| Error | 23 | 116,518 | 36,07% | 116,518 | 5,066 |  |  |
| Lack-of-Fit | 21 | 109,671 | 33,95% | 109,671 | 5,222 | 1,53 | 0,470 |
| Pure Error | 2 | 6,846 | 2,12% | 6,846 | 3,423 |  |  |
| Total | 26 | 323,038 | 100,00% |  |  |  |  |

## Regression Equation in Uncoded Units

|  |  |  |
| --- | --- | --- |
| F\_mean\_21h(1260min) | = | 54,9 + 67,8 Lac + 0,257 HPMC\_PS - 46,1 Lac\*Lac |

## Fits and Diagnostics for All Observations

| Obs | F\_mean\_21h(1260min) | Fit | SE Fit | 95% CI | Resid | Std Resid | Del Resid |
| --- | --- | --- | --- | --- | --- | --- | --- |
| 1 | 92,78 | 91,26 | 0,68 | (89,85; 92,68) | 1,51 | 0,70 | 0,70 |
| 2 | 99,08 | 96,70 | 0,68 | (95,28; 98,11) | 2,38 | 1,11 | 1,12 |
| 3 | 86,40 | 90,64 | 0,88 | (88,81; 92,47) | -4,24 | -2,05 | -2,21 |
| 4 | 93,38 | 96,07 | 0,88 | (94,24; 97,90) | -2,69 | -1,30 | -1,32 |
| 5 | 89,11 | 91,41 | 0,66 | (90,05; 92,77) | -2,30 | -1,07 | -1,07 |
| 6 | 96,64 | 96,84 | 0,66 | (95,48; 98,20) | -0,20 | -0,09 | -0,09 |
| 7 | 92,64 | 91,00 | 0,76 | (89,43; 92,56) | 1,65 | 0,78 | 0,77 |
| 8 | 95,76 | 96,43 | 0,76 | (94,87; 97,99) | -0,67 | -0,31 | -0,31 |
| 9 | 88,39 | 92,52 | 0,78 | (90,90; 94,13) | -4,13 | -1,96 | -2,09 |
| 10 | 100,23 | 97,95 | 0,78 | (96,33; 99,57) | 2,28 | 1,08 | 1,09 |
| 11 | 95,69 | 92,13 | 0,68 | (90,73; 93,53) | 3,56 | 1,66 | 1,73 |
| 12 | 97,35 | 97,56 | 0,68 | (96,16; 98,96) | -0,22 | -0,10 | -0,10 |
| 13 | 91,33 | 92,71 | 0,85 | (90,95; 94,48) | -1,38 | -0,66 | -0,66 |
| 14 | 94,95 | 98,15 | 0,85 | (96,38; 99,91) | -3,20 | -1,53 | -1,58 |
| 15 | 91,80 | 92,24 | 0,70 | (90,79; 93,68) | -0,43 | -0,20 | -0,20 |
| 16 | 97,98 | 97,67 | 0,70 | (96,22; 99,12) | 0,31 | 0,14 | 0,14 |
| 17 | 88,70 | 86,79 | 1,69 | (83,28; 90,29) | 1,91 | 1,29 | 1,31 |
| 18 | 97,69 | 97,66 | 1,69 | (94,15; 101,16) | 0,03 | 0,02 | 0,02 |
| 19 | 98,77 | 95,35 | 0,59 | (94,12; 96,57) | 3,43 | 1,58 | 1,63 |
| 20 | 94,24 | 94,90 | 0,60 | (93,67; 96,14) | -0,66 | -0,31 | -0,30 |
| 21 | 94,09 | 94,97 | 0,59 | (93,75; 96,19) | -0,89 | -0,41 | -0,40 |
| 22 | 96,73 | 95,27 | 0,58 | (94,06; 96,48) | 1,46 | 0,67 | 0,66 |
| 23 | 94,12 | 93,28 | 1,22 | (90,75; 95,81) | 0,84 | 0,45 | 0,44 |
| 24 | 96,83 | 97,06 | 1,24 | (94,50; 99,62) | -0,23 | -0,12 | -0,12 |
| 25 | 93,60 | 95,10 | 0,58 | (93,90; 96,31) | -1,50 | -0,69 | -0,68 |
| 26 | 96,59 | 95,10 | 0,58 | (93,90; 96,31) | 1,49 | 0,69 | 0,68 |
| 27 | 96,98 | 95,10 | 0,58 | (93,90; 96,31) | 1,88 | 0,86 | 0,86 |

| Obs | HI | Cook’s D | DFITS |  |  |
| --- | --- | --- | --- | --- | --- |
| 1 | 0,092405 | 0,01 | 0,22230 |  |  |
| 2 | 0,092405 | 0,03 | 0,35651 |  |  |
| 3 | 0,154489 | 0,19 | -0,94604 | R |  |
| 4 | 0,154489 | 0,08 | -0,56492 |  |  |
| 5 | 0,085364 | 0,03 | -0,32711 |  |  |
| 6 | 0,085364 | 0,00 | -0,02757 |  |  |
| 7 | 0,112838 | 0,02 | 0,27454 |  |  |
| 8 | 0,112838 | 0,00 | -0,11002 |  |  |
| 9 | 0,120630 | 0,13 | -0,77562 |  |  |
| 10 | 0,120630 | 0,04 | 0,40217 |  |  |
| 11 | 0,090283 | 0,07 | 0,54466 |  |  |
| 12 | 0,090283 | 0,00 | -0,03138 |  |  |
| 13 | 0,143796 | 0,02 | -0,26911 |  |  |
| 14 | 0,143796 | 0,10 | -0,64922 |  |  |
| 15 | 0,096712 | 0,00 | -0,06489 |  |  |
| 16 | 0,096712 | 0,00 | 0,04624 |  |  |
| 17 | 0,566788 | 0,54 | 1,49764 |  | X |
| 18 | 0,566788 | 0,00 | 0,02197 |  | X |
| 19 | 0,068996 | 0,05 | 0,44469 |  |  |
| 20 | 0,070758 | 0,00 | -0,08265 |  |  |
| 21 | 0,068883 | 0,00 | -0,10880 |  |  |
| 22 | 0,067544 | 0,01 | 0,17884 |  |  |
| 23 | 0,295031 | 0,02 | 0,28296 |  |  |
| 24 | 0,301653 | 0,00 | -0,07786 |  |  |
| 25 | 0,066842 | 0,01 | -0,18285 |  |  |
| 26 | 0,066842 | 0,01 | 0,18119 |  |  |
| 27 | 0,066842 | 0,01 | 0,22987 |  |  |

R  Large residual  
X  Unusual X

## Forward Selection of Terms

α to enter = 0,25

## Coded Coefficients

| Term | Coef | SE Coef | 95% CI | T-Value | P-Value | VIF |
| --- | --- | --- | --- | --- | --- | --- |
| Constant | 96,009 | 0,573 | (94,824; 97,194) | 167,57 | 0,000 |  |
| Lac | 4,528 | 0,906 | (2,654; 6,402) | 5,00 | 0,000 | 1,00 |
| HPMC\_PS | 1,78 | 1,07 | (-0,43; 3,99) | 1,67 | 0,109 | 1,00 |
| Lac\*Lac | -3,09 | 1,72 | (-6,65; 0,46) | -1,80 | 0,085 | 1,00 |

## Model Summary

| S | R-sq | R-sq(adj) | PRESS | R-sq(pred) | AICc | BIC |
| --- | --- | --- | --- | --- | --- | --- |
| 2,21884 | 57,43% | 51,88% | 158,258 | 40,50% | 128,19 | 131,81 |

## Analysis of Variance

| Source | DF | Seq SS | Contribution | Adj SS | Adj MS | F-Value | P-Value |
| --- | --- | --- | --- | --- | --- | --- | --- |
| Model | 3 | 152,754 | 57,43% | 152,754 | 50,918 | 10,34 | 0,000 |
| Linear | 2 | 136,798 | 51,43% | 136,712 | 68,356 | 13,88 | 0,000 |
| Lac | 1 | 123,030 | 46,25% | 123,030 | 123,030 | 24,99 | 0,000 |
| HPMC\_PS | 1 | 13,768 | 5,18% | 13,682 | 13,682 | 2,78 | 0,109 |
| Square | 1 | 15,956 | 6,00% | 15,956 | 15,956 | 3,24 | 0,085 |
| Lac\*Lac | 1 | 15,956 | 6,00% | 15,956 | 15,956 | 3,24 | 0,085 |
| Error | 23 | 113,234 | 42,57% | 113,234 | 4,923 |  |  |
| Lack-of-Fit | 21 | 107,122 | 40,27% | 107,122 | 5,101 | 1,67 | 0,442 |
| Pure Error | 2 | 6,112 | 2,30% | 6,112 | 3,056 |  |  |
| Total | 26 | 265,988 | 100,00% |  |  |  |  |

## Regression Equation in Uncoded Units

|  |  |  |
| --- | --- | --- |
| F\_mean\_22h(1320min) | = | 57,7 + 67,6 Lac + 0,242 HPMC\_PS - 49,5 Lac\*Lac |

## Fits and Diagnostics for All Observations

| Obs | F\_mean\_22h(1320min) | Fit | SE Fit | 95% CI | Resid | Std Resid | Del Resid |
| --- | --- | --- | --- | --- | --- | --- | --- |
| 1 | 94,06 | 92,53 | 0,67 | (91,14; 93,93) | 1,52 | 0,72 | 0,71 |
| 2 | 99,13 | 97,06 | 0,67 | (95,66; 98,45) | 2,07 | 0,98 | 0,98 |
| 3 | 87,98 | 91,94 | 0,87 | (90,14; 93,75) | -3,96 | -1,94 | -2,07 |
| 4 | 93,86 | 96,47 | 0,87 | (94,67; 98,27) | -2,61 | -1,28 | -1,30 |
| 5 | 90,34 | 92,67 | 0,65 | (91,32; 94,01) | -2,32 | -1,09 | -1,10 |
| 6 | 96,86 | 97,19 | 0,65 | (95,85; 98,53) | -0,34 | -0,16 | -0,15 |
| 7 | 93,92 | 92,28 | 0,75 | (90,74; 93,82) | 1,64 | 0,79 | 0,78 |
| 8 | 96,34 | 96,81 | 0,75 | (95,26; 98,35) | -0,47 | -0,22 | -0,22 |
| 9 | 89,63 | 93,71 | 0,77 | (92,12; 95,30) | -4,08 | -1,96 | -2,10 |
| 10 | 100,47 | 98,24 | 0,77 | (96,64; 99,83) | 2,23 | 1,07 | 1,08 |
| 11 | 97,02 | 93,35 | 0,67 | (91,97; 94,73) | 3,67 | 1,73 | 1,82 |
| 12 | 97,61 | 97,87 | 0,67 | (96,50; 99,25) | -0,27 | -0,13 | -0,12 |
| 13 | 92,37 | 93,90 | 0,84 | (92,16; 95,64) | -1,53 | -0,74 | -0,74 |
| 14 | 94,72 | 98,43 | 0,84 | (96,68; 100,17) | -3,71 | -1,81 | -1,91 |
| 15 | 92,90 | 93,45 | 0,69 | (92,02; 94,87) | -0,54 | -0,26 | -0,25 |
| 16 | 98,69 | 97,97 | 0,69 | (96,55; 99,40) | 0,72 | 0,34 | 0,33 |
| 17 | 90,13 | 88,33 | 1,67 | (84,87; 91,78) | 1,80 | 1,23 | 1,25 |
| 18 | 97,57 | 97,38 | 1,67 | (93,93; 100,84) | 0,19 | 0,13 | 0,13 |
| 19 | 99,38 | 96,18 | 0,58 | (94,97; 97,38) | 3,21 | 1,50 | 1,54 |
| 20 | 95,13 | 95,76 | 0,59 | (94,54; 96,98) | -0,63 | -0,30 | -0,29 |
| 21 | 95,20 | 95,82 | 0,58 | (94,62; 97,03) | -0,62 | -0,29 | -0,29 |
| 22 | 96,94 | 96,11 | 0,58 | (94,91; 97,30) | 0,84 | 0,39 | 0,38 |
| 23 | 94,80 | 94,23 | 1,21 | (91,74; 96,72) | 0,57 | 0,31 | 0,30 |
| 24 | 97,84 | 97,79 | 1,22 | (95,27; 100,31) | 0,05 | 0,03 | 0,02 |
| 25 | 94,78 | 95,95 | 0,57 | (94,76; 97,13) | -1,16 | -0,54 | -0,53 |
| 26 | 97,80 | 95,95 | 0,57 | (94,76; 97,13) | 1,85 | 0,86 | 0,86 |
| 27 | 97,82 | 95,95 | 0,57 | (94,76; 97,13) | 1,88 | 0,88 | 0,87 |

| Obs | HI | Cook’s D | DFITS |  |
| --- | --- | --- | --- | --- |
| 1 | 0,092405 | 0,01 | 0,22763 |  |
| 2 | 0,092405 | 0,02 | 0,31247 |  |
| 3 | 0,154489 | 0,17 | -0,88651 |  |
| 4 | 0,154489 | 0,07 | -0,55458 |  |
| 5 | 0,085364 | 0,03 | -0,33582 |  |
| 6 | 0,085364 | 0,00 | -0,04730 |  |
| 7 | 0,112838 | 0,02 | 0,27778 |  |
| 8 | 0,112838 | 0,00 | -0,07806 |  |
| 9 | 0,120630 | 0,13 | -0,77844 |  |
| 10 | 0,120630 | 0,04 | 0,39860 |  |
| 11 | 0,090283 | 0,07 | 0,57284 |  |
| 12 | 0,090283 | 0,00 | -0,03922 |  |
| 13 | 0,143796 | 0,02 | -0,30183 |  |
| 14 | 0,143796 | 0,14 | -0,78093 |  |
| 15 | 0,096712 | 0,00 | -0,08266 |  |
| 16 | 0,096712 | 0,00 | 0,10907 |  |
| 17 | 0,566788 | 0,50 | 1,42899 | X |
| 18 | 0,566788 | 0,01 | 0,14402 | X |
| 19 | 0,068996 | 0,04 | 0,41988 |  |
| 20 | 0,070758 | 0,00 | -0,08007 |  |
| 21 | 0,068883 | 0,00 | -0,07754 |  |
| 22 | 0,067544 | 0,00 | 0,10303 |  |
| 23 | 0,295031 | 0,01 | 0,19404 |  |
| 24 | 0,301653 | 0,00 | 0,01634 |  |
| 25 | 0,066842 | 0,01 | -0,14290 |  |
| 26 | 0,066842 | 0,01 | 0,23009 |  |
| 27 | 0,066842 | 0,01 | 0,23315 |  |

X  Unusual X

## Forward Selection of Terms

α to enter = 0,25

## Coded Coefficients

| Term | Coef | SE Coef | 95% CI | T-Value | P-Value | VIF |
| --- | --- | --- | --- | --- | --- | --- |
| Constant | 96,653 | 0,564 | (95,487; 97,820) | 171,39 | 0,000 |  |
| Lac | 3,662 | 0,892 | (1,818; 5,506) | 4,11 | 0,000 | 1,00 |
| HPMC\_PS | 1,73 | 1,05 | (-0,44; 3,90) | 1,65 | 0,113 | 1,00 |
| Lac\*Lac | -3,24 | 1,69 | (-6,73; 0,26) | -1,91 | 0,068 | 1,00 |

## Model Summary

| S | R-sq | R-sq(adj) | PRESS | R-sq(pred) | AICc | BIC |
| --- | --- | --- | --- | --- | --- | --- |
| 2,18388 | 50,28% | 43,79% | 152,765 | 30,75% | 127,33 | 130,95 |

## Analysis of Variance

| Source | DF | Seq SS | Contribution | Adj SS | Adj MS | F-Value | P-Value |
| --- | --- | --- | --- | --- | --- | --- | --- |
| Model | 3 | 110,909 | 50,28% | 110,909 | 36,970 | 7,75 | 0,001 |
| Linear | 2 | 93,463 | 42,37% | 93,376 | 46,688 | 9,79 | 0,001 |
| Lac | 1 | 80,459 | 36,47% | 80,459 | 80,459 | 16,87 | 0,000 |
| HPMC\_PS | 1 | 13,004 | 5,89% | 12,917 | 12,917 | 2,71 | 0,113 |
| Square | 1 | 17,446 | 7,91% | 17,446 | 17,446 | 3,66 | 0,068 |
| Lac\*Lac | 1 | 17,446 | 7,91% | 17,446 | 17,446 | 3,66 | 0,068 |
| Error | 23 | 109,695 | 49,72% | 109,695 | 4,769 |  |  |
| Lack-of-Fit | 21 | 104,502 | 47,37% | 104,502 | 4,976 | 1,92 | 0,399 |
| Pure Error | 2 | 5,193 | 2,35% | 5,193 | 2,596 |  |  |
| Total | 26 | 220,604 | 100,00% |  |  |  |  |

## Regression Equation in Uncoded Units

|  |  |  |
| --- | --- | --- |
| F\_mean\_23h(1380min) | = | 60,0 + 66,4 Lac + 0,235 HPMC\_PS - 51,8 Lac\*Lac |

## Fits and Diagnostics for All Observations

| Obs | F\_mean\_23h(1380min) | Fit | SE Fit | 95% CI | Resid | Std Resid | Del Resid |
| --- | --- | --- | --- | --- | --- | --- | --- |
| 1 | 94,91 | 93,59 | 0,66 | (92,21; 94,96) | 1,33 | 0,64 | 0,63 |
| 2 | 99,06 | 97,25 | 0,66 | (95,87; 98,62) | 1,81 | 0,87 | 0,87 |
| 3 | 89,49 | 93,01 | 0,86 | (91,24; 94,79) | -3,52 | -1,75 | -1,84 |
| 4 | 94,10 | 96,67 | 0,86 | (94,90; 98,45) | -2,57 | -1,28 | -1,30 |
| 5 | 91,57 | 93,72 | 0,64 | (92,40; 95,04) | -2,14 | -1,03 | -1,03 |
| 6 | 96,95 | 97,38 | 0,64 | (96,06; 98,70) | -0,43 | -0,20 | -0,20 |
| 7 | 94,89 | 93,34 | 0,73 | (91,82; 94,86) | 1,55 | 0,75 | 0,75 |
| 8 | 96,68 | 97,00 | 0,73 | (95,48; 98,52) | -0,32 | -0,15 | -0,15 |
| 9 | 90,50 | 94,73 | 0,76 | (93,16; 96,30) | -4,23 | -2,06 | -2,24 |
| 10 | 100,49 | 98,39 | 0,76 | (96,82; 99,96) | 2,10 | 1,02 | 1,02 |
| 11 | 98,17 | 94,38 | 0,66 | (93,02; 95,74) | 3,79 | 1,82 | 1,92 |
| 12 | 97,60 | 98,04 | 0,66 | (96,68; 99,40) | -0,44 | -0,21 | -0,21 |
| 13 | 93,21 | 94,91 | 0,83 | (93,20; 96,63) | -1,70 | -0,84 | -0,84 |
| 14 | 94,53 | 98,57 | 0,83 | (96,86; 100,29) | -4,04 | -2,00 | -2,15 |
| 15 | 93,93 | 94,48 | 0,68 | (93,07; 95,88) | -0,55 | -0,26 | -0,26 |
| 16 | 99,12 | 98,14 | 0,68 | (96,73; 99,54) | 0,98 | 0,47 | 0,46 |
| 17 | 91,38 | 89,70 | 1,64 | (86,29; 93,10) | 1,69 | 1,17 | 1,18 |
| 18 | 97,43 | 97,02 | 1,64 | (93,62; 100,42) | 0,41 | 0,28 | 0,28 |
| 19 | 99,71 | 96,81 | 0,57 | (95,63; 98,00) | 2,89 | 1,37 | 1,40 |
| 20 | 95,79 | 96,41 | 0,58 | (95,21; 97,61) | -0,62 | -0,29 | -0,29 |
| 21 | 96,19 | 96,47 | 0,57 | (95,29; 97,66) | -0,28 | -0,13 | -0,13 |
| 22 | 97,12 | 96,75 | 0,57 | (95,57; 97,92) | 0,38 | 0,18 | 0,18 |
| 23 | 95,16 | 94,92 | 1,19 | (92,47; 97,38) | 0,24 | 0,13 | 0,13 |
| 24 | 98,74 | 98,38 | 1,20 | (95,90; 100,86) | 0,36 | 0,20 | 0,19 |
| 25 | 95,85 | 96,59 | 0,56 | (95,43; 97,76) | -0,75 | -0,35 | -0,35 |
| 26 | 98,74 | 96,59 | 0,56 | (95,43; 97,76) | 2,14 | 1,02 | 1,02 |
| 27 | 98,52 | 96,59 | 0,56 | (95,43; 97,76) | 1,93 | 0,92 | 0,91 |

| Obs | HI | Cook’s D | DFITS |  |  |
| --- | --- | --- | --- | --- | --- |
| 1 | 0,092405 | 0,01 | 0,20072 |  |  |
| 2 | 0,092405 | 0,02 | 0,27640 |  |  |
| 3 | 0,154489 | 0,14 | -0,78813 |  |  |
| 4 | 0,154489 | 0,08 | -0,55632 |  |  |
| 5 | 0,085364 | 0,02 | -0,31361 |  |  |
| 6 | 0,085364 | 0,00 | -0,06114 |  |  |
| 7 | 0,112838 | 0,02 | 0,26631 |  |  |
| 8 | 0,112838 | 0,00 | -0,05376 |  |  |
| 9 | 0,120630 | 0,15 | -0,82834 | R |  |
| 10 | 0,120630 | 0,04 | 0,37939 |  |  |
| 11 | 0,090283 | 0,08 | 0,60552 |  |  |
| 12 | 0,090283 | 0,00 | -0,06529 |  |  |
| 13 | 0,143796 | 0,03 | -0,34253 |  |  |
| 14 | 0,143796 | 0,17 | -0,88196 |  |  |
| 15 | 0,096712 | 0,00 | -0,08426 |  |  |
| 16 | 0,096712 | 0,01 | 0,15160 |  |  |
| 17 | 0,566788 | 0,45 | 1,35481 |  | X |
| 18 | 0,566788 | 0,03 | 0,31899 |  | X |
| 19 | 0,068996 | 0,03 | 0,38118 |  |  |
| 20 | 0,070758 | 0,00 | -0,07969 |  |  |
| 21 | 0,068883 | 0,00 | -0,03591 |  |  |
| 22 | 0,067544 | 0,00 | 0,04711 |  |  |
| 23 | 0,295031 | 0,00 | 0,08342 |  |  |
| 24 | 0,301653 | 0,00 | 0,12660 |  |  |
| 25 | 0,066842 | 0,00 | -0,09301 |  |  |
| 26 | 0,066842 | 0,02 | 0,27219 |  |  |
| 27 | 0,066842 | 0,01 | 0,24403 |  |  |

R  Large residual  
X  Unusual X

## Forward Selection of Terms

α to enter = 0,25

## Coded Coefficients

| Term | Coef | SE Coef | 95% CI | T-Value | P-Value | VIF |
| --- | --- | --- | --- | --- | --- | --- |
| Constant | 97,172 | 0,563 | (96,007; 98,337) | 172,52 | 0,000 |  |
| Lac | 2,878 | 0,890 | (1,036; 4,721) | 3,23 | 0,004 | 1,00 |
| HPMC\_PS | 1,70 | 1,05 | (-0,48; 3,87) | 1,61 | 0,120 | 1,00 |
| Lac\*Lac | -3,36 | 1,69 | (-6,85; 0,14) | -1,99 | 0,059 | 1,00 |

## Model Summary

| S | R-sq | R-sq(adj) | PRESS | R-sq(pred) | AICc | BIC |
| --- | --- | --- | --- | --- | --- | --- |
| 2,18121 | 42,54% | 35,04% | 152,131 | 20,11% | 127,26 | 130,89 |

## Analysis of Variance

| Source | DF | Seq SS | Contribution | Adj SS | Adj MS | F-Value | P-Value |
| --- | --- | --- | --- | --- | --- | --- | --- |
| Model | 3 | 81,006 | 42,54% | 81,006 | 27,002 | 5,68 | 0,005 |
| Linear | 2 | 62,209 | 32,67% | 62,120 | 31,060 | 6,53 | 0,006 |
| Lac | 1 | 49,714 | 26,11% | 49,714 | 49,714 | 10,45 | 0,004 |
| HPMC\_PS | 1 | 12,495 | 6,56% | 12,406 | 12,406 | 2,61 | 0,120 |
| Square | 1 | 18,797 | 9,87% | 18,797 | 18,797 | 3,95 | 0,059 |
| Lac\*Lac | 1 | 18,797 | 9,87% | 18,797 | 18,797 | 3,95 | 0,059 |
| Error | 23 | 109,427 | 57,46% | 109,427 | 4,758 |  |  |
| Lack-of-Fit | 21 | 105,563 | 55,43% | 105,563 | 5,027 | 2,60 | 0,314 |
| Pure Error | 2 | 3,864 | 2,03% | 3,864 | 1,932 |  |  |
| Total | 26 | 190,433 | 100,00% |  |  |  |  |

## Regression Equation in Uncoded Units

|  |  |  |
| --- | --- | --- |
| F\_mean\_24h(1440min) | = | 61,9 + 65,2 Lac + 0,230 HPMC\_PS - 53,7 Lac\*Lac |

## Fits and Diagnostics for All Observations

| Obs | F\_mean\_24h(1440min) | Fit | SE Fit | 95% CI | Resid | Std Resid | Del Resid |
| --- | --- | --- | --- | --- | --- | --- | --- |
| 1 | 95,53 | 94,47 | 0,66 | (93,10; 95,85) | 1,06 | 0,51 | 0,50 |
| 2 | 98,99 | 97,35 | 0,66 | (95,98; 98,72) | 1,63 | 0,79 | 0,78 |
| 3 | 90,80 | 93,91 | 0,86 | (92,14; 95,69) | -3,12 | -1,55 | -1,61 |
| 4 | 94,17 | 96,79 | 0,86 | (95,02; 98,56) | -2,62 | -1,31 | -1,33 |
| 5 | 92,51 | 94,60 | 0,64 | (93,28; 95,92) | -2,09 | -1,00 | -1,00 |
| 6 | 97,09 | 97,48 | 0,64 | (96,16; 98,80) | -0,39 | -0,19 | -0,18 |
| 7 | 95,86 | 94,23 | 0,73 | (92,72; 95,75) | 1,63 | 0,79 | 0,78 |
| 8 | 96,74 | 97,11 | 0,73 | (95,60; 98,63) | -0,37 | -0,18 | -0,18 |
| 9 | 91,28 | 95,60 | 0,76 | (94,03; 97,16) | -4,32 | -2,11 | -2,30 |
| 10 | 100,51 | 98,48 | 0,76 | (96,91; 100,04) | 2,03 | 0,99 | 0,99 |
| 11 | 99,12 | 95,25 | 0,66 | (93,89; 96,61) | 3,87 | 1,86 | 1,97 |
| 12 | 97,59 | 98,13 | 0,66 | (96,77; 99,49) | -0,54 | -0,26 | -0,25 |
| 13 | 93,88 | 95,77 | 0,83 | (94,06; 97,49) | -1,89 | -0,94 | -0,93 |
| 14 | 94,32 | 98,65 | 0,83 | (96,94; 100,36) | -4,33 | -2,14 | -2,35 |
| 15 | 94,86 | 95,35 | 0,68 | (93,94; 96,75) | -0,49 | -0,24 | -0,23 |
| 16 | 99,27 | 98,22 | 0,68 | (96,82; 99,63) | 1,05 | 0,51 | 0,50 |
| 17 | 92,44 | 90,88 | 1,64 | (87,48; 94,27) | 1,57 | 1,09 | 1,10 |
| 18 | 97,29 | 96,63 | 1,64 | (93,24; 100,03) | 0,65 | 0,46 | 0,45 |
| 19 | 99,95 | 97,33 | 0,57 | (96,14; 98,52) | 2,62 | 1,25 | 1,26 |
| 20 | 96,30 | 96,93 | 0,58 | (95,73; 98,14) | -0,63 | -0,30 | -0,30 |
| 21 | 97,03 | 96,99 | 0,57 | (95,81; 98,18) | 0,03 | 0,02 | 0,02 |
| 22 | 97,28 | 97,26 | 0,57 | (96,09; 98,44) | 0,02 | 0,01 | 0,01 |
| 23 | 95,41 | 95,48 | 1,18 | (93,03; 97,93) | -0,07 | -0,04 | -0,04 |
| 24 | 99,43 | 98,87 | 1,20 | (96,39; 101,35) | 0,56 | 0,31 | 0,30 |
| 25 | 96,90 | 97,11 | 0,56 | (95,95; 98,28) | -0,21 | -0,10 | -0,10 |
| 26 | 99,48 | 97,11 | 0,56 | (95,95; 98,28) | 2,36 | 1,12 | 1,13 |
| 27 | 99,10 | 97,11 | 0,56 | (95,95; 98,28) | 1,99 | 0,94 | 0,94 |

| Obs | HI | Cook’s D | DFITS |  |  |
| --- | --- | --- | --- | --- | --- |
| 1 | 0,092405 | 0,01 | 0,15957 |  |  |
| 2 | 0,092405 | 0,02 | 0,24881 |  |  |
| 3 | 0,154489 | 0,11 | -0,68666 |  |  |
| 4 | 0,154489 | 0,08 | -0,56817 |  |  |
| 5 | 0,085364 | 0,02 | -0,30630 |  |  |
| 6 | 0,085364 | 0,00 | -0,05571 |  |  |
| 7 | 0,112838 | 0,02 | 0,27993 |  |  |
| 8 | 0,112838 | 0,00 | -0,06260 |  |  |
| 9 | 0,120630 | 0,15 | -0,85164 | R |  |
| 10 | 0,120630 | 0,03 | 0,36762 |  |  |
| 11 | 0,090283 | 0,09 | 0,62172 |  |  |
| 12 | 0,090283 | 0,00 | -0,07977 |  |  |
| 13 | 0,143796 | 0,04 | -0,38307 |  |  |
| 14 | 0,143796 | 0,19 | -0,96104 | R |  |
| 15 | 0,096712 | 0,00 | -0,07575 |  |  |
| 16 | 0,096712 | 0,01 | 0,16289 |  |  |
| 17 | 0,566788 | 0,39 | 1,25383 |  | X |
| 18 | 0,566788 | 0,07 | 0,51265 |  | X |
| 19 | 0,068996 | 0,03 | 0,34373 |  |  |
| 20 | 0,070758 | 0,00 | -0,08144 |  |  |
| 21 | 0,068883 | 0,00 | 0,00429 |  |  |
| 22 | 0,067544 | 0,00 | 0,00215 |  |  |
| 23 | 0,295031 | 0,00 | -0,02468 |  |  |
| 24 | 0,301653 | 0,01 | 0,19745 |  |  |
| 25 | 0,066842 | 0,00 | -0,02626 |  |  |
| 26 | 0,066842 | 0,02 | 0,30187 |  |  |
| 27 | 0,066842 | 0,02 | 0,25151 |  |  |

R  Large residual  
X  Unusual X
