# Supplementary material for: Influence of Selected Hypromellose Functionality-Related Characteristics and Soluble/Insoluble Filler Ratio on Carvedilol Release from Matrix Tablets
Source: Pharmaceutics. 2025 Oct 21;17(10):1358. doi: 10.3390/pharmaceutics17101358 (PMC12566823; doi:10.3390/pharmaceutics17101358)
Supplement: Supplementary file 1 [file pharmaceutics-17-01358-s001.zip › Report_Mean Release Analysis_RSM(CCD)_Stepwise, Stepwise.htm]

# Mean Release Analysis, Response Surface Design (Central Composite Design), Stepwise Regression - Stepwise

## Stepwise Selection of Terms

α to enter = 0,15; α to remove = 0,15

## Coded Coefficients

| Term | Coef | SE Coef | 95% CI | T-Value | P-Value | VIF |
| --- | --- | --- | --- | --- | --- | --- |
| Constant | 10,118 | 0,294 | (9,511; 10,726) | 34,37 | 0,000 |  |
| Lac | 2,621 | 0,624 | (1,333; 3,910) | 4,20 | 0,000 | 1,00 |
| HPMC\_PS | 1,115 | 0,736 | (-0,404; 2,635) | 1,51 | 0,143 | 1,00 |

## Model Summary

| S | R-sq | R-sq(adj) | PRESS | R-sq(pred) | AICc | BIC |
| --- | --- | --- | --- | --- | --- | --- |
| 1,52958 | 45,35% | 40,80% | 66,8980 | 34,89% | 106,21 | 109,58 |

## Analysis of Variance

| Source | DF | Seq SS | Contribution | Adj SS | Adj MS | F-Value | P-Value |
| --- | --- | --- | --- | --- | --- | --- | --- |
| Model | 2 | 46,598 | 45,35% | 46,598 | 23,299 | 9,96 | 0,001 |
| Linear | 2 | 46,598 | 45,35% | 46,598 | 23,299 | 9,96 | 0,001 |
| Lac | 1 | 41,230 | 40,13% | 41,230 | 41,230 | 17,62 | 0,000 |
| HPMC\_PS | 1 | 5,368 | 5,22% | 5,368 | 5,368 | 2,29 | 0,143 |
| Error | 24 | 56,151 | 54,65% | 56,151 | 2,340 |  |  |
| Lack-of-Fit | 22 | 42,122 | 40,99% | 42,122 | 1,915 | 0,27 | 0,958 |
| Pure Error | 2 | 14,029 | 13,65% | 14,029 | 7,015 |  |  |
| Total | 26 | 102,749 | 100,00% |  |  |  |  |

## Regression Equation in Uncoded Units

|  |  |  |
| --- | --- | --- |
| F\_mean\_0.17h(10min) | = | -5,69 + 10,49 Lac + 0,152 HPMC\_PS |

## Fits and Diagnostics for All Observations

| Obs | F\_mean\_0.17h(10min) | Fit | SE Fit | 95% CI | Resid | Std Resid | Del Resid |
| --- | --- | --- | --- | --- | --- | --- | --- |
| 1 | 9,095 | 8,532 | 0,464 | (7,574; 9,489) | 0,563 | 0,39 | 0,38 |
| 2 | 11,821 | 11,153 | 0,464 | (10,196; 12,110) | 0,668 | 0,46 | 0,45 |
| 3 | 7,323 | 8,162 | 0,600 | (6,923; 9,401) | -0,839 | -0,60 | -0,59 |
| 4 | 8,147 | 10,784 | 0,600 | (9,545; 12,023) | -2,636 | -1,87 | -1,99 |
| 5 | 7,099 | 8,616 | 0,446 | (7,696; 9,536) | -1,517 | -1,04 | -1,04 |
| 6 | 12,823 | 11,237 | 0,446 | (10,317; 12,157) | 1,586 | 1,08 | 1,09 |
| 7 | 7,484 | 8,373 | 0,513 | (7,315; 9,431) | -0,889 | -0,62 | -0,61 |
| 8 | 9,846 | 10,994 | 0,513 | (9,936; 12,053) | -1,148 | -0,80 | -0,79 |
| 9 | 8,623 | 9,270 | 0,530 | (8,176; 10,364) | -0,647 | -0,45 | -0,44 |
| 10 | 11,322 | 11,891 | 0,530 | (10,797; 12,986) | -0,570 | -0,40 | -0,39 |
| 11 | 9,398 | 9,042 | 0,458 | (8,096; 9,989) | 0,355 | 0,24 | 0,24 |
| 12 | 12,522 | 11,664 | 0,458 | (10,718; 12,610) | 0,858 | 0,59 | 0,58 |
| 13 | 7,874 | 9,387 | 0,579 | (8,192; 10,582) | -1,513 | -1,07 | -1,07 |
| 14 | 12,420 | 12,009 | 0,579 | (10,814; 13,204) | 0,412 | 0,29 | 0,29 |
| 15 | 10,497 | 9,105 | 0,474 | (8,126; 10,084) | 1,391 | 0,96 | 0,96 |
| 16 | 10,196 | 11,726 | 0,474 | (10,747; 12,706) | -1,530 | -1,05 | -1,05 |
| 17 | 8,246 | 7,458 | 0,691 | (6,033; 8,884) | 0,788 | 0,58 | 0,57 |
| 18 | 13,122 | 12,701 | 0,691 | (11,276; 14,126) | 0,421 | 0,31 | 0,30 |
| 19 | 9,647 | 10,222 | 0,304 | (9,595; 10,849) | -0,575 | -0,38 | -0,38 |
| 20 | 13,095 | 9,962 | 0,310 | (9,322; 10,602) | 3,133 | 2,09 | 2,26 |
| 21 | 10,472 | 10,001 | 0,303 | (9,376; 10,626) | 0,471 | 0,31 | 0,31 |
| 22 | 12,671 | 10,179 | 0,298 | (9,564; 10,794) | 2,492 | 1,66 | 1,73 |
| 23 | 9,297 | 9,003 | 0,787 | (7,378; 10,628) | 0,294 | 0,22 | 0,22 |
| 24 | 10,323 | 11,233 | 0,798 | (9,585; 12,881) | -0,910 | -0,70 | -0,69 |
| 25 | 8,148 | 10,080 | 0,295 | (9,471; 10,688) | -1,932 | -1,29 | -1,31 |
| 26 | 8,524 | 10,080 | 0,295 | (9,471; 10,688) | -1,556 | -1,04 | -1,04 |
| 27 | 12,912 | 10,080 | 0,295 | (9,471; 10,688) | 2,832 | 1,89 | 2,00 |

| Obs | HI | Cook’s D | DFITS |  |
| --- | --- | --- | --- | --- |
| 1 | 0,091956 | 0,01 | 0,120814 |  |
| 2 | 0,091956 | 0,01 | 0,143450 |  |
| 3 | 0,154060 | 0,02 | -0,251067 |  |
| 4 | 0,154060 | 0,21 | -0,847342 |  |
| 5 | 0,084910 | 0,03 | -0,316370 |  |
| 6 | 0,084910 | 0,04 | 0,331354 |  |
| 7 | 0,112397 | 0,02 | -0,216644 |  |
| 8 | 0,112397 | 0,03 | -0,281380 |  |
| 9 | 0,120141 | 0,01 | -0,163868 |  |
| 10 | 0,120141 | 0,01 | -0,144079 |  |
| 11 | 0,089807 | 0,00 | 0,074931 |  |
| 12 | 0,089807 | 0,01 | 0,182114 |  |
| 13 | 0,143301 | 0,06 | -0,438562 |  |
| 14 | 0,143301 | 0,00 | 0,116652 |  |
| 15 | 0,096233 | 0,03 | 0,311685 |  |
| 16 | 0,096233 | 0,04 | -0,344189 |  |
| 17 | 0,203866 | 0,03 | 0,288158 |  |
| 18 | 0,203866 | 0,01 | 0,153096 |  |
| 19 | 0,039415 | 0,00 | -0,076359 |  |
| 20 | 0,041065 | 0,06 | 0,468545 | R |
| 21 | 0,039207 | 0,00 | 0,062208 |  |
| 22 | 0,037944 | 0,04 | 0,343263 |  |
| 23 | 0,264924 | 0,01 | 0,131707 |  |
| 24 | 0,272505 | 0,06 | -0,422349 |  |
| 25 | 0,037200 | 0,02 | -0,256683 |  |
| 26 | 0,037200 | 0,01 | -0,204098 |  |
| 27 | 0,037200 | 0,05 | 0,393446 |  |

R  Large residual

## Stepwise Selection of Terms

α to enter = 0,15; α to remove = 0,15

## Coded Coefficients

| Term | Coef | SE Coef | 95% CI | T-Value | P-Value | VIF |
| --- | --- | --- | --- | --- | --- | --- |
| Constant | 13,846 | 0,354 | (13,114; 14,577) | 39,09 | 0,000 |  |
| Lac | 3,323 | 0,751 | (1,773; 4,874) | 4,42 | 0,000 | 1,00 |
| HPMC\_PS | 1,686 | 0,886 | (-0,142; 3,514) | 1,90 | 0,069 | 1,00 |

## Model Summary

| S | R-sq | R-sq(adj) | PRESS | R-sq(pred) | AICc | BIC |
| --- | --- | --- | --- | --- | --- | --- |
| 1,84024 | 49,14% | 44,90% | 96,3524 | 39,71% | 116,20 | 119,56 |

## Analysis of Variance

| Source | DF | Seq SS | Contribution | Adj SS | Adj MS | F-Value | P-Value |
| --- | --- | --- | --- | --- | --- | --- | --- |
| Model | 2 | 78,54 | 49,14% | 78,54 | 39,268 | 11,60 | 0,000 |
| Linear | 2 | 78,54 | 49,14% | 78,54 | 39,268 | 11,60 | 0,000 |
| Lac | 1 | 66,27 | 41,47% | 66,27 | 66,271 | 19,57 | 0,000 |
| HPMC\_PS | 1 | 12,26 | 7,67% | 12,26 | 12,265 | 3,62 | 0,069 |
| Error | 24 | 81,28 | 50,86% | 81,28 | 3,386 |  |  |
| Lack-of-Fit | 22 | 63,65 | 39,83% | 63,65 | 2,893 | 0,33 | 0,932 |
| Pure Error | 2 | 17,62 | 11,03% | 17,62 | 8,812 |  |  |
| Total | 26 | 159,81 | 100,00% |  |  |  |  |

## Regression Equation in Uncoded Units

|  |  |  |
| --- | --- | --- |
| F\_mean\_0.33h(20min) | = | -8,76 + 13,29 Lac + 0,229 HPMC\_PS |

## Fits and Diagnostics for All Observations

| Obs | F\_mean\_0.33h(20min) | Fit | SE Fit | 95% CI | Resid | Std Resid | Del Resid |
| --- | --- | --- | --- | --- | --- | --- | --- |
| 1 | 12,421 | 11,767 | 0,558 | (10,615; 12,919) | 0,654 | 0,37 | 0,37 |
| 2 | 16,290 | 15,090 | 0,558 | (13,939; 16,242) | 1,199 | 0,68 | 0,68 |
| 3 | 10,020 | 11,209 | 0,722 | (9,718; 12,699) | -1,189 | -0,70 | -0,69 |
| 4 | 11,393 | 14,532 | 0,722 | (13,041; 16,023) | -3,140 | -1,85 | -1,96 |
| 5 | 10,071 | 11,894 | 0,536 | (10,787; 13,001) | -1,823 | -1,04 | -1,04 |
| 6 | 17,069 | 15,218 | 0,536 | (14,111; 16,324) | 1,852 | 1,05 | 1,05 |
| 7 | 10,510 | 11,527 | 0,617 | (10,254; 12,801) | -1,017 | -0,59 | -0,58 |
| 8 | 13,414 | 14,851 | 0,617 | (13,577; 16,124) | -1,437 | -0,83 | -0,82 |
| 9 | 12,069 | 12,883 | 0,638 | (11,566; 14,199) | -0,814 | -0,47 | -0,46 |
| 10 | 15,416 | 16,206 | 0,638 | (14,890; 17,523) | -0,790 | -0,46 | -0,45 |
| 11 | 12,968 | 12,539 | 0,551 | (11,401; 13,677) | 0,429 | 0,24 | 0,24 |
| 12 | 16,492 | 15,862 | 0,551 | (14,724; 17,001) | 0,629 | 0,36 | 0,35 |
| 13 | 11,347 | 13,060 | 0,697 | (11,622; 14,498) | -1,714 | -1,01 | -1,01 |
| 14 | 16,913 | 16,384 | 0,697 | (14,946; 17,821) | 0,529 | 0,31 | 0,30 |
| 15 | 14,391 | 12,634 | 0,571 | (11,455; 13,812) | 1,757 | 1,00 | 1,00 |
| 16 | 13,890 | 15,957 | 0,571 | (14,779; 17,135) | -2,067 | -1,18 | -1,19 |
| 17 | 11,041 | 10,464 | 0,831 | (8,749; 12,179) | 0,577 | 0,35 | 0,34 |
| 18 | 17,442 | 17,111 | 0,831 | (15,396; 18,825) | 0,331 | 0,20 | 0,20 |
| 19 | 13,390 | 14,003 | 0,365 | (13,248; 14,757) | -0,612 | -0,34 | -0,33 |
| 20 | 17,586 | 13,609 | 0,373 | (12,840; 14,379) | 3,977 | 2,21 | 2,42 |
| 21 | 14,091 | 13,669 | 0,364 | (12,917; 14,421) | 0,423 | 0,23 | 0,23 |
| 22 | 17,139 | 13,937 | 0,358 | (13,197; 14,677) | 3,202 | 1,77 | 1,86 |
| 23 | 12,766 | 12,160 | 0,947 | (10,205; 14,115) | 0,606 | 0,38 | 0,38 |
| 24 | 14,769 | 15,531 | 0,961 | (13,548; 17,514) | -0,762 | -0,49 | -0,48 |
| 25 | 11,694 | 13,787 | 0,355 | (13,055; 14,520) | -2,093 | -1,16 | -1,17 |
| 26 | 11,921 | 13,787 | 0,355 | (13,055; 14,520) | -1,866 | -1,03 | -1,03 |
| 27 | 16,945 | 13,787 | 0,355 | (13,055; 14,520) | 3,158 | 1,75 | 1,83 |

| Obs | HI | Cook’s D | DFITS |  |
| --- | --- | --- | --- | --- |
| 1 | 0,091956 | 0,00 | 0,116515 |  |
| 2 | 0,091956 | 0,02 | 0,215184 |  |
| 3 | 0,154060 | 0,03 | -0,296456 |  |
| 4 | 0,154060 | 0,21 | -0,837261 |  |
| 5 | 0,084910 | 0,03 | -0,315946 |  |
| 6 | 0,084910 | 0,03 | 0,321144 |  |
| 7 | 0,112397 | 0,01 | -0,205806 |  |
| 8 | 0,112397 | 0,03 | -0,292870 |  |
| 9 | 0,120141 | 0,01 | -0,171394 |  |
| 10 | 0,120141 | 0,01 | -0,166298 |  |
| 11 | 0,089807 | 0,00 | 0,075310 |  |
| 12 | 0,089807 | 0,00 | 0,110514 |  |
| 13 | 0,143301 | 0,06 | -0,411558 |  |
| 14 | 0,143301 | 0,01 | 0,124595 |  |
| 15 | 0,096233 | 0,04 | 0,327787 |  |
| 16 | 0,096233 | 0,05 | -0,388944 |  |
| 17 | 0,203866 | 0,01 | 0,174515 |  |
| 18 | 0,203866 | 0,00 | 0,099992 |  |
| 19 | 0,039415 | 0,00 | -0,067462 |  |
| 20 | 0,041065 | 0,07 | 0,500720 | R |
| 21 | 0,039207 | 0,00 | 0,046398 |  |
| 22 | 0,037944 | 0,04 | 0,369933 |  |
| 23 | 0,264924 | 0,02 | 0,226383 |  |
| 24 | 0,272505 | 0,03 | -0,292231 |  |
| 25 | 0,037200 | 0,02 | -0,229617 |  |
| 26 | 0,037200 | 0,01 | -0,203417 |  |
| 27 | 0,037200 | 0,04 | 0,360306 |  |

R  Large residual

## Stepwise Selection of Terms

α to enter = 0,15; α to remove = 0,15

## Coded Coefficients

| Term | Coef | SE Coef | 95% CI | T-Value | P-Value | VIF |
| --- | --- | --- | --- | --- | --- | --- |
| Constant | 16,245 | 0,376 | (15,468; 17,021) | 43,19 | 0,000 |  |
| Lac | 3,740 | 0,798 | (2,093; 5,386) | 4,69 | 0,000 | 1,00 |
| HPMC\_PS | 1,909 | 0,940 | (-0,032; 3,850) | 2,03 | 0,054 | 1,00 |

## Model Summary

| S | R-sq | R-sq(adj) | PRESS | R-sq(pred) | AICc | BIC |
| --- | --- | --- | --- | --- | --- | --- |
| 1,95398 | 52,09% | 48,10% | 108,673 | 43,19% | 119,43 | 122,80 |

## Analysis of Variance

| Source | DF | Seq SS | Contribution | Adj SS | Adj MS | F-Value | P-Value |
| --- | --- | --- | --- | --- | --- | --- | --- |
| Model | 2 | 99,65 | 52,09% | 99,65 | 49,823 | 13,05 | 0,000 |
| Linear | 2 | 99,65 | 52,09% | 99,65 | 49,823 | 13,05 | 0,000 |
| Lac | 1 | 83,92 | 43,87% | 83,92 | 83,920 | 21,98 | 0,000 |
| HPMC\_PS | 1 | 15,73 | 8,22% | 15,73 | 15,727 | 4,12 | 0,054 |
| Error | 24 | 91,63 | 47,91% | 91,63 | 3,818 |  |  |
| Lack-of-Fit | 22 | 72,43 | 37,87% | 72,43 | 3,292 | 0,34 | 0,925 |
| Pure Error | 2 | 19,20 | 10,04% | 19,20 | 9,600 |  |  |
| Total | 26 | 191,28 | 100,00% |  |  |  |  |

## Regression Equation in Uncoded Units

|  |  |  |
| --- | --- | --- |
| F\_mean\_0.5h(30min) | = | -9,31 + 14,96 Lac + 0,260 HPMC\_PS |

## Fits and Diagnostics for All Observations

| Obs | F\_mean\_0.5h(30min) | Fit | SE Fit | 95% CI | Resid | Std Resid | Del Resid |
| --- | --- | --- | --- | --- | --- | --- | --- |
| 1 | 14,629 | 13,903 | 0,593 | (12,680; 15,125) | 0,726 | 0,39 | 0,38 |
| 2 | 18,932 | 17,642 | 0,593 | (16,420; 18,865) | 1,290 | 0,69 | 0,68 |
| 3 | 11,941 | 13,270 | 0,767 | (11,688; 14,853) | -1,329 | -0,74 | -0,73 |
| 4 | 13,736 | 17,010 | 0,767 | (15,427; 18,593) | -3,274 | -1,82 | -1,92 |
| 5 | 12,093 | 14,047 | 0,569 | (12,872; 15,222) | -1,954 | -1,05 | -1,05 |
| 6 | 19,640 | 17,787 | 0,569 | (16,611; 18,962) | 1,853 | 0,99 | 0,99 |
| 7 | 12,627 | 13,631 | 0,655 | (12,279; 14,983) | -1,004 | -0,55 | -0,54 |
| 8 | 15,806 | 17,371 | 0,655 | (16,019; 18,723) | -1,565 | -0,85 | -0,85 |
| 9 | 14,264 | 15,166 | 0,677 | (13,769; 16,564) | -0,902 | -0,49 | -0,48 |
| 10 | 17,960 | 18,906 | 0,677 | (17,508; 20,304) | -0,946 | -0,52 | -0,51 |
| 11 | 15,264 | 14,777 | 0,586 | (13,568; 15,985) | 0,487 | 0,26 | 0,26 |
| 12 | 18,986 | 18,517 | 0,586 | (17,308; 19,725) | 0,469 | 0,25 | 0,25 |
| 13 | 13,544 | 15,367 | 0,740 | (13,840; 16,894) | -1,823 | -1,01 | -1,01 |
| 14 | 19,878 | 19,107 | 0,740 | (17,580; 20,634) | 0,771 | 0,43 | 0,42 |
| 15 | 16,784 | 14,884 | 0,606 | (13,633; 16,135) | 1,900 | 1,02 | 1,02 |
| 16 | 16,332 | 18,624 | 0,606 | (17,373; 19,875) | -2,292 | -1,23 | -1,25 |
| 17 | 12,909 | 12,439 | 0,882 | (10,618; 14,259) | 0,470 | 0,27 | 0,26 |
| 18 | 20,286 | 19,918 | 0,882 | (18,097; 21,739) | 0,367 | 0,21 | 0,21 |
| 19 | 15,883 | 16,422 | 0,388 | (15,622; 17,223) | -0,539 | -0,28 | -0,28 |
| 20 | 20,126 | 15,977 | 0,396 | (15,160; 16,795) | 4,149 | 2,17 | 2,37 |
| 21 | 16,410 | 16,044 | 0,387 | (15,246; 16,843) | 0,366 | 0,19 | 0,19 |
| 22 | 19,930 | 16,348 | 0,381 | (15,563; 17,134) | 3,582 | 1,87 | 1,98 |
| 23 | 15,034 | 14,336 | 1,006 | (12,260; 16,412) | 0,698 | 0,42 | 0,41 |
| 24 | 17,365 | 18,153 | 1,020 | (16,048; 20,259) | -0,788 | -0,47 | -0,47 |
| 25 | 14,088 | 16,178 | 0,377 | (15,401; 16,956) | -2,090 | -1,09 | -1,09 |
| 26 | 14,218 | 16,178 | 0,377 | (15,401; 16,956) | -1,960 | -1,02 | -1,02 |
| 27 | 19,519 | 16,178 | 0,377 | (15,401; 16,956) | 3,340 | 1,74 | 1,82 |

| Obs | HI | Cook’s D | DFITS |  |
| --- | --- | --- | --- | --- |
| 1 | 0,091956 | 0,01 | 0,121897 |  |
| 2 | 0,091956 | 0,02 | 0,217967 |  |
| 3 | 0,154060 | 0,03 | -0,312616 |  |
| 4 | 0,154060 | 0,20 | -0,819815 |  |
| 5 | 0,084910 | 0,03 | -0,319015 |  |
| 6 | 0,084910 | 0,03 | 0,301931 |  |
| 7 | 0,112397 | 0,01 | -0,191121 |  |
| 8 | 0,112397 | 0,03 | -0,300785 |  |
| 9 | 0,120141 | 0,01 | -0,178972 |  |
| 10 | 0,120141 | 0,01 | -0,187840 |  |
| 11 | 0,089807 | 0,00 | 0,080372 |  |
| 12 | 0,089807 | 0,00 | 0,077455 |  |
| 13 | 0,143301 | 0,06 | -0,412427 |  |
| 14 | 0,143301 | 0,01 | 0,171387 |  |
| 15 | 0,096233 | 0,04 | 0,334031 |  |
| 16 | 0,096233 | 0,05 | -0,407227 |  |
| 17 | 0,203866 | 0,01 | 0,133736 |  |
| 18 | 0,203866 | 0,00 | 0,104432 |  |
| 19 | 0,039415 | 0,00 | -0,055950 |  |
| 20 | 0,041065 | 0,07 | 0,489857 | R |
| 21 | 0,039207 | 0,00 | 0,037774 |  |
| 22 | 0,037944 | 0,05 | 0,393071 |  |
| 23 | 0,264924 | 0,02 | 0,245731 |  |
| 24 | 0,272505 | 0,03 | -0,284749 |  |
| 25 | 0,037200 | 0,02 | -0,215155 |  |
| 26 | 0,037200 | 0,01 | -0,201166 |  |
| 27 | 0,037200 | 0,04 | 0,358696 |  |

R  Large residual

## Stepwise Selection of Terms

α to enter = 0,15; α to remove = 0,15

## Coded Coefficients

| Term | Coef | SE Coef | 95% CI | T-Value | P-Value | VIF |
| --- | --- | --- | --- | --- | --- | --- |
| Constant | 19,131 | 0,400 | (18,304; 19,957) | 47,78 | 0,000 |  |
| Lac | 4,179 | 0,849 | (2,427; 5,932) | 4,92 | 0,000 | 1,00 |
| HPMC\_PS | 2,14 | 1,00 | (0,08; 4,21) | 2,14 | 0,043 | 1,00 |

## Model Summary

| S | R-sq | R-sq(adj) | PRESS | R-sq(pred) | AICc | BIC |
| --- | --- | --- | --- | --- | --- | --- |
| 2,07986 | 54,56% | 50,77% | 123,662 | 45,87% | 122,81 | 126,17 |

## Analysis of Variance

| Source | DF | Seq SS | Contribution | Adj SS | Adj MS | F-Value | P-Value |
| --- | --- | --- | --- | --- | --- | --- | --- |
| Model | 2 | 124,64 | 54,56% | 124,64 | 62,320 | 14,41 | 0,000 |
| Linear | 2 | 124,64 | 54,56% | 124,64 | 62,320 | 14,41 | 0,000 |
| Lac | 1 | 104,81 | 45,88% | 104,81 | 104,807 | 24,23 | 0,000 |
| HPMC\_PS | 1 | 19,83 | 8,68% | 19,83 | 19,832 | 4,58 | 0,043 |
| Error | 24 | 103,82 | 45,44% | 103,82 | 4,326 |  |  |
| Lack-of-Fit | 22 | 82,95 | 36,31% | 82,95 | 3,770 | 0,36 | 0,915 |
| Pure Error | 2 | 20,87 | 9,14% | 20,87 | 10,436 |  |  |
| Total | 26 | 228,46 | 100,00% |  |  |  |  |

## Regression Equation in Uncoded Units

|  |  |  |
| --- | --- | --- |
| F\_mean\_0.75h(45min) | = | -9,53 + 16,72 Lac + 0,291 HPMC\_PS |

## Fits and Diagnostics for All Observations

| Obs | F\_mean\_0.75h(45min) | Fit | SE Fit | 95% CI | Resid | Std Resid | Del Resid |
| --- | --- | --- | --- | --- | --- | --- | --- |
| 1 | 17,369 | 16,511 | 0,631 | (15,209; 17,813) | 0,858 | 0,43 | 0,43 |
| 2 | 21,994 | 20,690 | 0,631 | (19,389; 21,992) | 1,304 | 0,66 | 0,65 |
| 3 | 14,259 | 15,801 | 0,816 | (14,116; 17,486) | -1,542 | -0,81 | -0,80 |
| 4 | 16,501 | 19,980 | 0,816 | (18,296; 21,665) | -3,480 | -1,82 | -1,92 |
| 5 | 14,687 | 16,673 | 0,606 | (15,422; 17,924) | -1,985 | -1,00 | -1,00 |
| 6 | 22,707 | 20,852 | 0,606 | (19,601; 22,103) | 1,855 | 0,93 | 0,93 |
| 7 | 15,190 | 16,206 | 0,697 | (14,767; 17,645) | -1,016 | -0,52 | -0,51 |
| 8 | 18,616 | 20,386 | 0,697 | (18,946; 21,825) | -1,770 | -0,90 | -0,90 |
| 9 | 16,906 | 17,930 | 0,721 | (16,442; 19,418) | -1,024 | -0,53 | -0,52 |
| 10 | 21,098 | 22,109 | 0,721 | (20,622; 23,597) | -1,011 | -0,52 | -0,51 |
| 11 | 18,055 | 17,493 | 0,623 | (16,206; 18,779) | 0,562 | 0,28 | 0,28 |
| 12 | 22,100 | 21,672 | 0,623 | (20,386; 22,959) | 0,428 | 0,22 | 0,21 |
| 13 | 16,264 | 18,155 | 0,787 | (16,530; 19,780) | -1,891 | -0,98 | -0,98 |
| 14 | 23,411 | 22,335 | 0,787 | (20,710; 23,960) | 1,077 | 0,56 | 0,55 |
| 15 | 19,597 | 17,613 | 0,645 | (16,281; 18,945) | 1,983 | 1,00 | 1,00 |
| 16 | 19,194 | 21,793 | 0,645 | (20,461; 23,124) | -2,599 | -1,31 | -1,34 |
| 17 | 15,271 | 14,877 | 0,939 | (12,939; 16,815) | 0,394 | 0,21 | 0,21 |
| 18 | 23,700 | 23,236 | 0,939 | (21,298; 25,174) | 0,464 | 0,25 | 0,24 |
| 19 | 18,969 | 19,330 | 0,413 | (18,478; 20,183) | -0,361 | -0,18 | -0,17 |
| 20 | 23,059 | 18,831 | 0,421 | (17,961; 19,700) | 4,229 | 2,08 | 2,24 |
| 21 | 19,298 | 18,906 | 0,412 | (18,056; 19,756) | 0,392 | 0,19 | 0,19 |
| 22 | 23,265 | 19,247 | 0,405 | (18,411; 20,084) | 4,018 | 1,97 | 2,11 |
| 23 | 17,796 | 16,987 | 1,071 | (14,778; 19,197) | 0,809 | 0,45 | 0,45 |
| 24 | 20,282 | 21,274 | 1,086 | (19,033; 23,515) | -0,992 | -0,56 | -0,55 |
| 25 | 16,879 | 19,056 | 0,401 | (18,229; 19,884) | -2,177 | -1,07 | -1,07 |
| 26 | 17,038 | 19,056 | 0,401 | (18,229; 19,884) | -2,019 | -0,99 | -0,99 |
| 27 | 22,552 | 19,056 | 0,401 | (18,229; 19,884) | 3,496 | 1,71 | 1,79 |

| Obs | HI | Cook’s D | DFITS |  |
| --- | --- | --- | --- | --- |
| 1 | 0,091956 | 0,01 | 0,135365 |  |
| 2 | 0,091956 | 0,01 | 0,206768 |  |
| 3 | 0,154060 | 0,04 | -0,341426 |  |
| 4 | 0,154060 | 0,20 | -0,818474 |  |
| 5 | 0,084910 | 0,03 | -0,303934 |  |
| 6 | 0,084910 | 0,03 | 0,283260 |  |
| 7 | 0,112397 | 0,01 | -0,181683 |  |
| 8 | 0,112397 | 0,03 | -0,320111 |  |
| 9 | 0,120141 | 0,01 | -0,191029 |  |
| 10 | 0,120141 | 0,01 | -0,188569 |  |
| 11 | 0,089807 | 0,00 | 0,087216 |  |
| 12 | 0,089807 | 0,00 | 0,066377 |  |
| 13 | 0,143301 | 0,05 | -0,401543 |  |
| 14 | 0,143301 | 0,02 | 0,225381 |  |
| 15 | 0,096233 | 0,04 | 0,327370 |  |
| 16 | 0,096233 | 0,06 | -0,435842 |  |
| 17 | 0,203866 | 0,00 | 0,105221 |  |
| 18 | 0,203866 | 0,01 | 0,123964 |  |
| 19 | 0,039415 | 0,00 | -0,035133 |  |
| 20 | 0,041065 | 0,06 | 0,464358 | R |
| 21 | 0,039207 | 0,00 | 0,038093 |  |
| 22 | 0,037944 | 0,05 | 0,418217 |  |
| 23 | 0,264924 | 0,02 | 0,267800 |  |
| 24 | 0,272505 | 0,04 | -0,337147 |  |
| 25 | 0,037200 | 0,01 | -0,210349 |  |
| 26 | 0,037200 | 0,01 | -0,194339 |  |
| 27 | 0,037200 | 0,04 | 0,351792 |  |

R  Large residual

## Stepwise Selection of Terms

α to enter = 0,15; α to remove = 0,15

## Coded Coefficients

| Term | Coef | SE Coef | 95% CI | T-Value | P-Value | VIF |
| --- | --- | --- | --- | --- | --- | --- |
| Constant | 21,616 | 0,417 | (20,755; 22,478) | 51,81 | 0,000 |  |
| Lac | 4,541 | 0,885 | (2,715; 6,368) | 5,13 | 0,000 | 1,00 |
| HPMC\_PS | 2,26 | 1,04 | (0,11; 4,42) | 2,17 | 0,040 | 1,00 |

## Model Summary

| S | R-sq | R-sq(adj) | PRESS | R-sq(pred) | AICc | BIC |
| --- | --- | --- | --- | --- | --- | --- |
| 2,16772 | 56,40% | 52,76% | 134,939 | 47,83% | 125,04 | 128,40 |

## Analysis of Variance

| Source | DF | Seq SS | Contribution | Adj SS | Adj MS | F-Value | P-Value |
| --- | --- | --- | --- | --- | --- | --- | --- |
| Model | 2 | 145,87 | 56,40% | 145,87 | 72,937 | 15,52 | 0,000 |
| Linear | 2 | 145,87 | 56,40% | 145,87 | 72,937 | 15,52 | 0,000 |
| Lac | 1 | 123,73 | 47,84% | 123,73 | 123,731 | 26,33 | 0,000 |
| HPMC\_PS | 1 | 22,14 | 8,56% | 22,14 | 22,144 | 4,71 | 0,040 |
| Error | 24 | 112,78 | 43,60% | 112,78 | 4,699 |  |  |
| Lack-of-Fit | 22 | 91,48 | 35,37% | 91,48 | 4,158 | 0,39 | 0,900 |
| Pure Error | 2 | 21,30 | 8,23% | 21,30 | 10,648 |  |  |
| Total | 26 | 258,65 | 100,00% |  |  |  |  |

## Regression Equation in Uncoded Units

|  |  |  |
| --- | --- | --- |
| F\_mean\_1h(60min) | = | -8,9 + 18,16 Lac + 0,308 HPMC\_PS |

## Fits and Diagnostics for All Observations

| Obs | F\_mean\_1h(60min) | Fit | SE Fit | 95% CI | Resid | Std Resid | Del Resid |
| --- | --- | --- | --- | --- | --- | --- | --- |
| 1 | 19,761 | 18,786 | 0,657 | (17,429; 20,142) | 0,976 | 0,47 | 0,46 |
| 2 | 24,653 | 23,327 | 0,657 | (21,970; 24,683) | 1,327 | 0,64 | 0,63 |
| 3 | 16,350 | 18,036 | 0,851 | (16,280; 19,792) | -1,685 | -0,85 | -0,84 |
| 4 | 18,938 | 22,577 | 0,851 | (20,821; 24,333) | -3,639 | -1,83 | -1,93 |
| 5 | 16,856 | 18,957 | 0,632 | (17,653; 20,260) | -2,101 | -1,01 | -1,01 |
| 6 | 25,374 | 23,498 | 0,632 | (22,194; 24,801) | 1,876 | 0,90 | 0,90 |
| 7 | 17,428 | 18,464 | 0,727 | (16,964; 19,963) | -1,036 | -0,51 | -0,50 |
| 8 | 21,048 | 23,005 | 0,727 | (21,505; 24,505) | -1,956 | -0,96 | -0,96 |
| 9 | 19,146 | 20,285 | 0,751 | (18,734; 21,836) | -1,139 | -0,56 | -0,55 |
| 10 | 23,860 | 24,826 | 0,751 | (23,276; 26,377) | -0,967 | -0,48 | -0,47 |
| 11 | 20,444 | 19,823 | 0,650 | (18,482; 21,164) | 0,621 | 0,30 | 0,29 |
| 12 | 24,688 | 24,364 | 0,650 | (23,024; 25,705) | 0,324 | 0,16 | 0,15 |
| 13 | 18,608 | 20,523 | 0,821 | (18,830; 22,217) | -1,915 | -0,95 | -0,95 |
| 14 | 26,317 | 25,064 | 0,821 | (23,371; 26,758) | 1,253 | 0,62 | 0,62 |
| 15 | 21,982 | 19,950 | 0,672 | (18,562; 21,338) | 2,032 | 0,99 | 0,99 |
| 16 | 21,578 | 24,491 | 0,672 | (23,104; 25,879) | -2,913 | -1,41 | -1,45 |
| 17 | 17,356 | 16,997 | 0,979 | (14,977; 19,017) | 0,359 | 0,19 | 0,18 |
| 18 | 26,662 | 26,079 | 0,979 | (24,059; 28,099) | 0,583 | 0,30 | 0,30 |
| 19 | 21,678 | 21,827 | 0,430 | (20,939; 22,716) | -0,149 | -0,07 | -0,07 |
| 20 | 25,490 | 21,299 | 0,439 | (20,393; 22,206) | 4,191 | 1,97 | 2,11 |
| 21 | 21,834 | 21,379 | 0,429 | (20,493; 22,265) | 0,456 | 0,21 | 0,21 |
| 22 | 26,073 | 21,740 | 0,422 | (20,868; 22,611) | 4,334 | 2,04 | 2,19 |
| 23 | 20,282 | 19,351 | 1,116 | (17,049; 21,654) | 0,930 | 0,50 | 0,49 |
| 24 | 22,774 | 23,881 | 1,132 | (21,546; 26,217) | -1,108 | -0,60 | -0,59 |
| 25 | 19,368 | 21,538 | 0,418 | (20,675; 22,401) | -2,170 | -1,02 | -1,02 |
| 26 | 19,506 | 21,538 | 0,418 | (20,675; 22,401) | -2,032 | -0,96 | -0,95 |
| 27 | 25,088 | 21,538 | 0,418 | (20,675; 22,401) | 3,550 | 1,67 | 1,74 |

| Obs | HI | Cook’s D | DFITS |  |
| --- | --- | --- | --- | --- |
| 1 | 0,091956 | 0,01 | 0,147829 |  |
| 2 | 0,091956 | 0,01 | 0,201836 |  |
| 3 | 0,154060 | 0,04 | -0,358470 |  |
| 4 | 0,154060 | 0,20 | -0,821667 |  |
| 5 | 0,084910 | 0,03 | -0,308815 |  |
| 6 | 0,084910 | 0,03 | 0,274524 |  |
| 7 | 0,112397 | 0,01 | -0,177615 |  |
| 8 | 0,112397 | 0,04 | -0,340263 |  |
| 9 | 0,120141 | 0,01 | -0,204035 |  |
| 10 | 0,120141 | 0,01 | -0,172804 |  |
| 11 | 0,089807 | 0,00 | 0,092517 |  |
| 12 | 0,089807 | 0,00 | 0,048141 |  |
| 13 | 0,143301 | 0,05 | -0,389678 |  |
| 14 | 0,143301 | 0,02 | 0,252041 |  |
| 15 | 0,096233 | 0,03 | 0,321554 |  |
| 16 | 0,096233 | 0,07 | -0,471669 |  |
| 17 | 0,203866 | 0,00 | 0,092001 |  |
| 18 | 0,203866 | 0,01 | 0,149625 |  |
| 19 | 0,039415 | 0,00 | -0,013897 |  |
| 20 | 0,041065 | 0,06 | 0,436971 |  |
| 21 | 0,039207 | 0,00 | 0,042463 |  |
| 22 | 0,037944 | 0,05 | 0,435773 | R |
| 23 | 0,264924 | 0,03 | 0,295658 |  |
| 24 | 0,272505 | 0,04 | -0,361636 |  |
| 25 | 0,037200 | 0,01 | -0,200714 |  |
| 26 | 0,037200 | 0,01 | -0,187382 |  |
| 27 | 0,037200 | 0,04 | 0,341571 |  |

R  Large residual

## Stepwise Selection of Terms

α to enter = 0,15; α to remove = 0,15

## Coded Coefficients

| Term | Coef | SE Coef | 95% CI | T-Value | P-Value | VIF |
| --- | --- | --- | --- | --- | --- | --- |
| Constant | 25,945 | 0,441 | (25,035; 26,855) | 58,85 | 0,000 |  |
| Lac | 5,104 | 0,935 | (3,174; 7,033) | 5,46 | 0,000 | 1,00 |
| HPMC\_PS | 2,47 | 1,10 | (0,19; 4,74) | 2,24 | 0,035 | 1,00 |

## Model Summary

| S | R-sq | R-sq(adj) | PRESS | R-sq(pred) | AICc | BIC |
| --- | --- | --- | --- | --- | --- | --- |
| 2,29048 | 59,18% | 55,77% | 151,544 | 50,86% | 128,01 | 131,38 |

## Analysis of Variance

| Source | DF | Seq SS | Contribution | Adj SS | Adj MS | F-Value | P-Value |
| --- | --- | --- | --- | --- | --- | --- | --- |
| Model | 2 | 182,51 | 59,18% | 182,51 | 91,254 | 17,39 | 0,000 |
| Linear | 2 | 182,51 | 59,18% | 182,51 | 91,254 | 17,39 | 0,000 |
| Lac | 1 | 156,28 | 50,67% | 156,28 | 156,277 | 29,79 | 0,000 |
| HPMC\_PS | 1 | 26,23 | 8,50% | 26,23 | 26,231 | 5,00 | 0,035 |
| Error | 24 | 125,91 | 40,82% | 125,91 | 5,246 |  |  |
| Lack-of-Fit | 22 | 103,50 | 33,56% | 103,50 | 4,705 | 0,42 | 0,884 |
| Pure Error | 2 | 22,41 | 7,27% | 22,41 | 11,206 |  |  |
| Total | 26 | 308,42 | 100,00% |  |  |  |  |

## Regression Equation in Uncoded Units

|  |  |  |
| --- | --- | --- |
| F\_mean\_1.5h(90min) | = | -7,6 + 20,41 Lac + 0,335 HPMC\_PS |

## Fits and Diagnostics for All Observations

| Obs | F\_mean\_1.5h(90min) | Fit | SE Fit | 95% CI | Resid | Std Resid | Del Resid |
| --- | --- | --- | --- | --- | --- | --- | --- |
| 1 | 23,960 | 22,783 | 0,695 | (21,350; 24,217) | 1,177 | 0,54 | 0,53 |
| 2 | 29,242 | 27,887 | 0,695 | (26,453; 29,320) | 1,356 | 0,62 | 0,61 |
| 3 | 20,030 | 21,967 | 0,899 | (20,111; 23,822) | -1,937 | -0,92 | -0,92 |
| 4 | 23,256 | 27,070 | 0,899 | (25,215; 28,926) | -3,814 | -1,81 | -1,91 |
| 5 | 20,814 | 22,969 | 0,667 | (21,592; 24,347) | -2,155 | -0,98 | -0,98 |
| 6 | 29,979 | 28,073 | 0,667 | (26,695; 29,450) | 1,906 | 0,87 | 0,87 |
| 7 | 21,354 | 22,433 | 0,768 | (20,848; 24,018) | -1,079 | -0,50 | -0,49 |
| 8 | 25,207 | 27,536 | 0,768 | (25,951; 29,121) | -2,329 | -1,08 | -1,08 |
| 9 | 23,222 | 24,415 | 0,794 | (22,777; 26,054) | -1,193 | -0,56 | -0,55 |
| 10 | 28,652 | 29,519 | 0,794 | (27,880; 31,157) | -0,867 | -0,40 | -0,40 |
| 11 | 24,520 | 23,912 | 0,686 | (22,496; 25,329) | 0,607 | 0,28 | 0,27 |
| 12 | 29,209 | 29,016 | 0,686 | (27,599; 30,433) | 0,193 | 0,09 | 0,09 |
| 13 | 22,644 | 24,675 | 0,867 | (22,885; 26,464) | -2,031 | -0,96 | -0,96 |
| 14 | 31,222 | 29,778 | 0,867 | (27,989; 31,568) | 1,444 | 0,68 | 0,67 |
| 15 | 26,099 | 24,051 | 0,711 | (22,584; 25,517) | 2,048 | 0,94 | 0,94 |
| 16 | 25,890 | 29,154 | 0,711 | (27,688; 30,621) | -3,264 | -1,50 | -1,54 |
| 17 | 21,094 | 20,756 | 1,034 | (18,621; 22,890) | 0,338 | 0,17 | 0,16 |
| 18 | 31,707 | 30,963 | 1,034 | (28,828; 33,097) | 0,745 | 0,36 | 0,36 |
| 19 | 26,440 | 26,174 | 0,455 | (25,236; 27,113) | 0,265 | 0,12 | 0,12 |
| 20 | 29,744 | 25,600 | 0,464 | (24,642; 26,558) | 4,145 | 1,85 | 1,95 |
| 21 | 26,102 | 25,686 | 0,454 | (24,750; 26,622) | 0,416 | 0,19 | 0,18 |
| 22 | 30,808 | 26,079 | 0,446 | (25,158; 27,000) | 4,730 | 2,11 | 2,28 |
| 23 | 24,646 | 23,480 | 1,179 | (21,046; 25,913) | 1,166 | 0,59 | 0,59 |
| 24 | 27,178 | 28,410 | 1,196 | (25,942; 30,878) | -1,232 | -0,63 | -0,62 |
| 25 | 23,641 | 25,859 | 0,442 | (24,948; 26,771) | -2,219 | -0,99 | -0,99 |
| 26 | 23,791 | 25,859 | 0,442 | (24,948; 26,771) | -2,068 | -0,92 | -0,92 |
| 27 | 29,512 | 25,859 | 0,442 | (24,948; 26,771) | 3,653 | 1,63 | 1,69 |

| Obs | HI | Cook’s D | DFITS |  |
| --- | --- | --- | --- | --- |
| 1 | 0,091956 | 0,01 | 0,169043 |  |
| 2 | 0,091956 | 0,01 | 0,195048 |  |
| 3 | 0,154060 | 0,05 | -0,391094 |  |
| 4 | 0,154060 | 0,20 | -0,814073 |  |
| 5 | 0,084910 | 0,03 | -0,299438 |  |
| 6 | 0,084910 | 0,02 | 0,263552 |  |
| 7 | 0,112397 | 0,01 | -0,175079 |  |
| 8 | 0,112397 | 0,05 | -0,385453 |  |
| 9 | 0,120141 | 0,01 | -0,202223 |  |
| 10 | 0,120141 | 0,01 | -0,146436 |  |
| 11 | 0,089807 | 0,00 | 0,085581 |  |
| 12 | 0,089807 | 0,00 | 0,027158 |  |
| 13 | 0,143301 | 0,05 | -0,391066 |  |
| 14 | 0,143301 | 0,03 | 0,275404 |  |
| 15 | 0,096233 | 0,03 | 0,306110 |  |
| 16 | 0,096233 | 0,08 | -0,502954 |  |
| 17 | 0,203866 | 0,00 | 0,082056 |  |
| 18 | 0,203866 | 0,01 | 0,180995 |  |
| 19 | 0,039415 | 0,00 | 0,023459 |  |
| 20 | 0,041065 | 0,05 | 0,404194 |  |
| 21 | 0,039207 | 0,00 | 0,036683 |  |
| 22 | 0,037944 | 0,06 | 0,453261 | R |
| 23 | 0,264924 | 0,04 | 0,351632 |  |
| 24 | 0,272505 | 0,05 | -0,380996 |  |
| 25 | 0,037200 | 0,01 | -0,193923 |  |
| 26 | 0,037200 | 0,01 | -0,180306 |  |
| 27 | 0,037200 | 0,03 | 0,331547 |  |

R  Large residual

## Stepwise Selection of Terms

α to enter = 0,15; α to remove = 0,15

## Coded Coefficients

| Term | Coef | SE Coef | 95% CI | T-Value | P-Value | VIF |
| --- | --- | --- | --- | --- | --- | --- |
| Constant | 29,744 | 0,458 | (28,798; 30,690) | 64,90 | 0,000 |  |
| Lac | 5,573 | 0,972 | (3,566; 7,579) | 5,73 | 0,000 | 1,00 |
| HPMC\_PS | 2,59 | 1,15 | (0,22; 4,96) | 2,26 | 0,033 | 1,00 |

## Model Summary

| S | R-sq | R-sq(adj) | PRESS | R-sq(pred) | AICc | BIC |
| --- | --- | --- | --- | --- | --- | --- |
| 2,38097 | 61,27% | 58,05% | 164,631 | 53,14% | 130,11 | 133,47 |

## Analysis of Variance

| Source | DF | Seq SS | Contribution | Adj SS | Adj MS | F-Value | P-Value |
| --- | --- | --- | --- | --- | --- | --- | --- |
| Model | 2 | 215,28 | 61,27% | 215,28 | 107,641 | 18,99 | 0,000 |
| Linear | 2 | 215,28 | 61,27% | 215,28 | 107,641 | 18,99 | 0,000 |
| Lac | 1 | 186,33 | 53,03% | 186,33 | 186,326 | 32,87 | 0,000 |
| HPMC\_PS | 1 | 28,96 | 8,24% | 28,96 | 28,956 | 5,11 | 0,033 |
| Error | 24 | 136,06 | 38,73% | 136,06 | 5,669 |  |  |
| Lack-of-Fit | 22 | 113,70 | 32,36% | 113,70 | 5,168 | 0,46 | 0,861 |
| Pure Error | 2 | 22,36 | 6,36% | 22,36 | 11,180 |  |  |
| Total | 26 | 351,34 | 100,00% |  |  |  |  |

## Regression Equation in Uncoded Units

|  |  |  |
| --- | --- | --- |
| F\_mean\_2h(120min) | = | -5,9 + 22,29 Lac + 0,352 HPMC\_PS |

## Fits and Diagnostics for All Observations

| Obs | F\_mean\_2h(120min) | Fit | SE Fit | 95% CI | Resid | Std Resid | Del Resid |
| --- | --- | --- | --- | --- | --- | --- | --- |
| 1 | 27,647 | 26,317 | 0,722 | (24,827; 27,807) | 1,330 | 0,59 | 0,58 |
| 2 | 33,275 | 31,890 | 0,722 | (30,399; 33,380) | 1,385 | 0,61 | 0,60 |
| 3 | 23,330 | 25,459 | 0,935 | (23,531; 27,388) | -2,130 | -0,97 | -0,97 |
| 4 | 27,018 | 31,032 | 0,935 | (29,103; 32,961) | -4,014 | -1,83 | -1,93 |
| 5 | 24,319 | 26,513 | 0,694 | (25,081; 27,944) | -2,194 | -0,96 | -0,96 |
| 6 | 33,930 | 32,085 | 0,694 | (30,653; 33,517) | 1,845 | 0,81 | 0,80 |
| 7 | 24,866 | 25,949 | 0,798 | (24,301; 27,596) | -1,083 | -0,48 | -0,47 |
| 8 | 28,958 | 31,521 | 0,798 | (29,874; 33,169) | -2,564 | -1,14 | -1,15 |
| 9 | 26,769 | 28,032 | 0,825 | (26,329; 29,735) | -1,263 | -0,57 | -0,56 |
| 10 | 32,839 | 33,604 | 0,825 | (31,901; 35,308) | -0,766 | -0,34 | -0,34 |
| 11 | 28,140 | 27,503 | 0,714 | (26,031; 28,976) | 0,637 | 0,28 | 0,27 |
| 12 | 33,175 | 33,076 | 0,714 | (31,603; 34,549) | 0,099 | 0,04 | 0,04 |
| 13 | 26,177 | 28,304 | 0,901 | (26,444; 30,164) | -2,127 | -0,97 | -0,96 |
| 14 | 35,420 | 33,877 | 0,901 | (32,017; 35,737) | 1,543 | 0,70 | 0,69 |
| 15 | 29,734 | 27,649 | 0,739 | (26,124; 29,173) | 2,085 | 0,92 | 0,92 |
| 16 | 29,696 | 33,222 | 0,739 | (31,697; 34,746) | -3,526 | -1,56 | -1,61 |
| 17 | 24,325 | 24,082 | 1,075 | (21,863; 26,300) | 0,244 | 0,11 | 0,11 |
| 18 | 36,098 | 35,227 | 1,075 | (33,008; 37,446) | 0,871 | 0,41 | 0,40 |
| 19 | 30,544 | 29,985 | 0,473 | (29,010; 30,961) | 0,559 | 0,24 | 0,23 |
| 20 | 33,491 | 29,381 | 0,482 | (28,385; 30,377) | 4,110 | 1,76 | 1,85 |
| 21 | 29,839 | 29,472 | 0,471 | (28,499; 30,445) | 0,367 | 0,16 | 0,15 |
| 22 | 34,911 | 29,885 | 0,464 | (28,928; 30,842) | 5,027 | 2,15 | 2,35 |
| 23 | 28,553 | 27,154 | 1,226 | (24,625; 29,683) | 1,399 | 0,69 | 0,68 |
| 24 | 31,053 | 32,334 | 1,243 | (29,769; 34,899) | -1,281 | -0,63 | -0,62 |
| 25 | 27,483 | 29,654 | 0,459 | (28,706; 30,602) | -2,171 | -0,93 | -0,93 |
| 26 | 27,597 | 29,654 | 0,459 | (28,706; 30,602) | -2,057 | -0,88 | -0,88 |
| 27 | 33,330 | 29,654 | 0,459 | (28,706; 30,602) | 3,676 | 1,57 | 1,63 |

| Obs | HI | Cook’s D | DFITS |  |
| --- | --- | --- | --- | --- |
| 1 | 0,091956 | 0,01 | 0,184000 |  |
| 2 | 0,091956 | 0,01 | 0,191659 |  |
| 3 | 0,154060 | 0,06 | -0,414521 |  |
| 4 | 0,154060 | 0,20 | -0,825719 |  |
| 5 | 0,084910 | 0,03 | -0,292926 |  |
| 6 | 0,084910 | 0,02 | 0,244885 |  |
| 7 | 0,112397 | 0,01 | -0,168949 |  |
| 8 | 0,112397 | 0,06 | -0,409414 |  |
| 9 | 0,120141 | 0,01 | -0,205973 |  |
| 10 | 0,120141 | 0,01 | -0,124352 |  |
| 11 | 0,089807 | 0,00 | 0,086361 |  |
| 12 | 0,089807 | 0,00 | 0,013373 |  |
| 13 | 0,143301 | 0,05 | -0,394218 |  |
| 14 | 0,143301 | 0,03 | 0,283231 |  |
| 15 | 0,096233 | 0,03 | 0,299575 |  |
| 16 | 0,096233 | 0,09 | -0,524843 |  |
| 17 | 0,203866 | 0,00 | 0,056886 |  |
| 18 | 0,203866 | 0,01 | 0,203756 |  |
| 19 | 0,039415 | 0,00 | 0,047520 |  |
| 20 | 0,041065 | 0,04 | 0,382700 |  |
| 21 | 0,039207 | 0,00 | 0,031086 |  |
| 22 | 0,037944 | 0,06 | 0,465821 | R |
| 23 | 0,264924 | 0,06 | 0,406771 |  |
| 24 | 0,272505 | 0,05 | -0,381056 |  |
| 25 | 0,037200 | 0,01 | -0,182152 |  |
| 26 | 0,037200 | 0,01 | -0,172226 |  |
| 27 | 0,037200 | 0,03 | 0,319733 |  |

R  Large residual

## Stepwise Selection of Terms

α to enter = 0,15; α to remove = 0,15

## Coded Coefficients

| Term | Coef | SE Coef | 95% CI | T-Value | P-Value | VIF |
| --- | --- | --- | --- | --- | --- | --- |
| Constant | 33,182 | 0,474 | (32,203; 34,161) | 69,97 | 0,000 |  |
| Lac | 5,96 | 1,01 | (3,88; 8,03) | 5,92 | 0,000 | 1,00 |
| HPMC\_PS | 2,67 | 1,19 | (0,23; 5,12) | 2,25 | 0,034 | 1,00 |

## Model Summary

| S | R-sq | R-sq(adj) | PRESS | R-sq(pred) | AICc | BIC |
| --- | --- | --- | --- | --- | --- | --- |
| 2,46370 | 62,60% | 59,48% | 177,155 | 54,51% | 131,95 | 135,32 |

## Analysis of Variance

| Source | DF | Seq SS | Contribution | Adj SS | Adj MS | F-Value | P-Value |
| --- | --- | --- | --- | --- | --- | --- | --- |
| Model | 2 | 243,79 | 62,60% | 243,79 | 121,897 | 20,08 | 0,000 |
| Linear | 2 | 243,79 | 62,60% | 243,79 | 121,897 | 20,08 | 0,000 |
| Lac | 1 | 212,96 | 54,68% | 212,96 | 212,960 | 35,09 | 0,000 |
| HPMC\_PS | 1 | 30,83 | 7,92% | 30,83 | 30,834 | 5,08 | 0,034 |
| Error | 24 | 145,68 | 37,40% | 145,68 | 6,070 |  |  |
| Lack-of-Fit | 22 | 123,63 | 31,74% | 123,63 | 5,620 | 0,51 | 0,836 |
| Pure Error | 2 | 22,04 | 5,66% | 22,04 | 11,022 |  |  |
| Total | 26 | 389,47 | 100,00% |  |  |  |  |

## Regression Equation in Uncoded Units

|  |  |  |
| --- | --- | --- |
| F\_mean\_2.5h(150min) | = | -4,0 + 23,83 Lac + 0,363 HPMC\_PS |

## Fits and Diagnostics for All Observations

| Obs | F\_mean\_2.5h(150min) | Fit | SE Fit | 95% CI | Resid | Std Resid | Del Resid |
| --- | --- | --- | --- | --- | --- | --- | --- |
| 1 | 30,966 | 29,542 | 0,747 | (28,000; 31,084) | 1,424 | 0,61 | 0,60 |
| 2 | 36,876 | 35,500 | 0,747 | (33,958; 37,042) | 1,377 | 0,59 | 0,58 |
| 3 | 26,375 | 28,657 | 0,967 | (26,661; 30,653) | -2,281 | -1,01 | -1,01 |
| 4 | 30,399 | 34,615 | 0,967 | (32,619; 36,610) | -4,215 | -1,86 | -1,97 |
| 5 | 27,471 | 29,744 | 0,718 | (28,262; 31,225) | -2,273 | -0,96 | -0,96 |
| 6 | 37,628 | 35,701 | 0,718 | (34,220; 37,183) | 1,926 | 0,82 | 0,81 |
| 7 | 28,090 | 29,162 | 0,826 | (27,457; 30,867) | -1,072 | -0,46 | -0,45 |
| 8 | 32,301 | 35,120 | 0,826 | (33,415; 36,824) | -2,818 | -1,21 | -1,23 |
| 9 | 29,986 | 31,312 | 0,854 | (29,549; 33,074) | -1,325 | -0,57 | -0,57 |
| 10 | 36,545 | 37,269 | 0,854 | (35,507; 39,032) | -0,724 | -0,31 | -0,31 |
| 11 | 31,457 | 30,766 | 0,738 | (29,243; 32,290) | 0,690 | 0,29 | 0,29 |
| 12 | 36,736 | 36,724 | 0,738 | (35,200; 38,248) | 0,012 | 0,01 | 0,01 |
| 13 | 29,407 | 31,593 | 0,933 | (29,668; 33,517) | -2,185 | -0,96 | -0,96 |
| 14 | 39,136 | 37,550 | 0,933 | (35,625; 39,475) | 1,585 | 0,70 | 0,69 |
| 15 | 33,013 | 30,916 | 0,764 | (29,339; 32,494) | 2,097 | 0,90 | 0,89 |
| 16 | 33,169 | 36,874 | 0,764 | (35,297; 38,451) | -3,705 | -1,58 | -1,64 |
| 17 | 27,300 | 27,132 | 1,112 | (24,836; 29,427) | 0,168 | 0,08 | 0,07 |
| 18 | 40,033 | 39,047 | 1,112 | (36,751; 41,343) | 0,986 | 0,45 | 0,44 |
| 19 | 34,291 | 33,431 | 0,489 | (32,421; 34,440) | 0,860 | 0,36 | 0,35 |
| 20 | 36,830 | 32,808 | 0,499 | (31,777; 33,838) | 4,023 | 1,67 | 1,74 |
| 21 | 33,170 | 32,901 | 0,488 | (31,895; 33,908) | 0,269 | 0,11 | 0,11 |
| 22 | 38,633 | 33,327 | 0,480 | (32,337; 34,318) | 5,306 | 2,20 | 2,40 |
| 23 | 32,129 | 30,509 | 1,268 | (27,892; 33,126) | 1,619 | 0,77 | 0,76 |
| 24 | 34,525 | 35,855 | 1,286 | (33,200; 38,509) | -1,330 | -0,63 | -0,62 |
| 25 | 30,945 | 33,089 | 0,475 | (32,109; 34,070) | -2,144 | -0,89 | -0,88 |
| 26 | 31,126 | 33,089 | 0,475 | (32,109; 34,070) | -1,964 | -0,81 | -0,81 |
| 27 | 36,783 | 33,089 | 0,475 | (32,109; 34,070) | 3,694 | 1,53 | 1,57 |

| Obs | HI | Cook’s D | DFITS |  |
| --- | --- | --- | --- | --- |
| 1 | 0,091956 | 0,01 | 0,190396 |  |
| 2 | 0,091956 | 0,01 | 0,184013 |  |
| 3 | 0,154060 | 0,06 | -0,429785 |  |
| 4 | 0,154060 | 0,21 | -0,840072 |  |
| 5 | 0,084910 | 0,03 | -0,293359 |  |
| 6 | 0,084910 | 0,02 | 0,247185 |  |
| 7 | 0,112397 | 0,01 | -0,161561 |  |
| 8 | 0,112397 | 0,06 | -0,436572 |  |
| 9 | 0,120141 | 0,01 | -0,208916 |  |
| 10 | 0,120141 | 0,00 | -0,113594 |  |
| 11 | 0,089807 | 0,00 | 0,090487 |  |
| 12 | 0,089807 | 0,00 | 0,001606 |  |
| 13 | 0,143301 | 0,05 | -0,391256 |  |
| 14 | 0,143301 | 0,03 | 0,281217 |  |
| 15 | 0,096233 | 0,03 | 0,290883 |  |
| 16 | 0,096233 | 0,09 | -0,533897 |  |
| 17 | 0,203866 | 0,00 | 0,037932 |  |
| 18 | 0,203866 | 0,02 | 0,223179 |  |
| 19 | 0,039415 | 0,00 | 0,070800 |  |
| 20 | 0,041065 | 0,04 | 0,359239 |  |
| 21 | 0,039207 | 0,00 | 0,022022 |  |
| 22 | 0,037944 | 0,06 | 0,477529 | R |
| 23 | 0,264924 | 0,07 | 0,456160 |  |
| 24 | 0,272505 | 0,05 | -0,382315 |  |
| 25 | 0,037200 | 0,01 | -0,173557 |  |
| 26 | 0,037200 | 0,01 | -0,158500 |  |
| 27 | 0,037200 | 0,03 | 0,309495 |  |

R  Large residual

## Stepwise Selection of Terms

α to enter = 0,15; α to remove = 0,15

## Coded Coefficients

| Term | Coef | SE Coef | 95% CI | T-Value | P-Value | VIF |
| --- | --- | --- | --- | --- | --- | --- |
| Constant | 36,354 | 0,483 | (35,357; 37,352) | 75,21 | 0,000 |  |
| Lac | 6,29 | 1,03 | (4,18; 8,41) | 6,14 | 0,000 | 1,00 |
| HPMC\_PS | 2,73 | 1,21 | (0,24; 5,23) | 2,26 | 0,033 | 1,00 |

## Model Summary

| S | R-sq | R-sq(adj) | PRESS | R-sq(pred) | AICc | BIC |
| --- | --- | --- | --- | --- | --- | --- |
| 2,51115 | 64,06% | 61,06% | 184,728 | 56,13% | 132,98 | 136,35 |

## Analysis of Variance

| Source | DF | Seq SS | Contribution | Adj SS | Adj MS | F-Value | P-Value |
| --- | --- | --- | --- | --- | --- | --- | --- |
| Model | 2 | 269,75 | 64,06% | 269,75 | 134,873 | 21,39 | 0,000 |
| Linear | 2 | 269,75 | 64,06% | 269,75 | 134,873 | 21,39 | 0,000 |
| Lac | 1 | 237,54 | 56,41% | 237,54 | 237,540 | 37,67 | 0,000 |
| HPMC\_PS | 1 | 32,21 | 7,65% | 32,21 | 32,207 | 5,11 | 0,033 |
| Error | 24 | 151,34 | 35,94% | 151,34 | 6,306 |  |  |
| Lack-of-Fit | 22 | 129,72 | 30,81% | 129,72 | 5,896 | 0,55 | 0,817 |
| Pure Error | 2 | 21,62 | 5,13% | 21,62 | 10,810 |  |  |
| Total | 26 | 421,09 | 100,00% |  |  |  |  |

## Regression Equation in Uncoded Units

|  |  |  |
| --- | --- | --- |
| F\_mean\_3h(180min) | = | -2,1 + 25,17 Lac + 0,371 HPMC\_PS |

## Fits and Diagnostics for All Observations

| Obs | F\_mean\_3h(180min) | Fit | SE Fit | 95% CI | Resid | Std Resid | Del Resid |
| --- | --- | --- | --- | --- | --- | --- | --- |
| 1 | 34,028 | 32,533 | 0,761 | (30,961; 34,104) | 1,496 | 0,63 | 0,62 |
| 2 | 40,223 | 38,825 | 0,761 | (37,253; 40,396) | 1,398 | 0,58 | 0,58 |
| 3 | 29,168 | 31,628 | 0,986 | (29,594; 33,662) | -2,460 | -1,07 | -1,07 |
| 4 | 33,625 | 37,920 | 0,986 | (35,886; 39,954) | -4,295 | -1,86 | -1,97 |
| 5 | 30,469 | 32,739 | 0,732 | (31,229; 34,249) | -2,270 | -0,94 | -0,94 |
| 6 | 40,923 | 39,031 | 0,732 | (37,521; 40,541) | 1,892 | 0,79 | 0,78 |
| 7 | 31,112 | 32,144 | 0,842 | (30,407; 33,882) | -1,033 | -0,44 | -0,43 |
| 8 | 35,438 | 38,436 | 0,842 | (36,699; 40,174) | -2,998 | -1,27 | -1,28 |
| 9 | 32,975 | 34,341 | 0,870 | (32,545; 36,138) | -1,366 | -0,58 | -0,57 |
| 10 | 39,972 | 40,633 | 0,870 | (38,837; 42,430) | -0,662 | -0,28 | -0,28 |
| 11 | 34,494 | 33,784 | 0,753 | (32,231; 35,337) | 0,710 | 0,30 | 0,29 |
| 12 | 39,969 | 40,076 | 0,753 | (38,523; 41,629) | -0,107 | -0,04 | -0,04 |
| 13 | 32,410 | 34,628 | 0,951 | (32,666; 36,590) | -2,218 | -0,95 | -0,95 |
| 14 | 42,546 | 40,920 | 0,951 | (38,958; 42,882) | 1,625 | 0,70 | 0,69 |
| 15 | 35,988 | 33,937 | 0,779 | (32,329; 35,545) | 2,051 | 0,86 | 0,85 |
| 16 | 36,412 | 40,229 | 0,779 | (38,622; 41,837) | -3,818 | -1,60 | -1,66 |
| 17 | 30,093 | 29,968 | 1,134 | (27,627; 32,308) | 0,125 | 0,06 | 0,05 |
| 18 | 43,614 | 42,552 | 1,134 | (40,212; 44,892) | 1,063 | 0,47 | 0,47 |
| 19 | 37,657 | 36,609 | 0,499 | (35,580; 37,638) | 1,048 | 0,43 | 0,42 |
| 20 | 39,839 | 35,972 | 0,509 | (34,921; 37,022) | 3,868 | 1,57 | 1,63 |
| 21 | 36,222 | 36,068 | 0,497 | (35,041; 37,094) | 0,154 | 0,06 | 0,06 |
| 22 | 42,049 | 36,503 | 0,489 | (35,493; 37,512) | 5,547 | 2,25 | 2,48 |
| 23 | 35,448 | 33,623 | 1,293 | (30,955; 36,290) | 1,825 | 0,85 | 0,84 |
| 24 | 37,768 | 39,086 | 1,311 | (36,380; 41,791) | -1,317 | -0,62 | -0,61 |
| 25 | 34,152 | 36,260 | 0,484 | (35,260; 37,259) | -2,107 | -0,86 | -0,85 |
| 26 | 34,401 | 36,260 | 0,484 | (35,260; 37,259) | -1,858 | -0,75 | -0,75 |
| 27 | 39,967 | 36,260 | 0,484 | (35,260; 37,259) | 3,708 | 1,50 | 1,55 |

| Obs | HI | Cook’s D | DFITS |  |
| --- | --- | --- | --- | --- |
| 1 | 0,091956 | 0,01 | 0,196338 |  |
| 2 | 0,091956 | 0,01 | 0,183327 |  |
| 3 | 0,154060 | 0,07 | -0,455917 |  |
| 4 | 0,154060 | 0,21 | -0,839827 |  |
| 5 | 0,084910 | 0,03 | -0,287127 |  |
| 6 | 0,084910 | 0,02 | 0,237948 |  |
| 7 | 0,112397 | 0,01 | -0,152641 |  |
| 8 | 0,112397 | 0,07 | -0,456984 |  |
| 9 | 0,120141 | 0,02 | -0,211340 |  |
| 10 | 0,120141 | 0,00 | -0,101775 |  |
| 11 | 0,089807 | 0,00 | 0,091353 |  |
| 12 | 0,089807 | 0,00 | -0,013776 |  |
| 13 | 0,143301 | 0,05 | -0,389544 |  |
| 14 | 0,143301 | 0,03 | 0,282855 |  |
| 15 | 0,096233 | 0,03 | 0,278713 |  |
| 16 | 0,096233 | 0,09 | -0,540463 |  |
| 17 | 0,203866 | 0,00 | 0,027736 |  |
| 18 | 0,203866 | 0,02 | 0,236096 |  |
| 19 | 0,039415 | 0,00 | 0,084768 |  |
| 20 | 0,041065 | 0,04 | 0,336425 |  |
| 21 | 0,039207 | 0,00 | 0,012393 |  |
| 22 | 0,037944 | 0,07 | 0,492967 | R |
| 23 | 0,264924 | 0,09 | 0,505906 |  |
| 24 | 0,272505 | 0,05 | -0,371447 |  |
| 25 | 0,037200 | 0,01 | -0,167149 |  |
| 26 | 0,037200 | 0,01 | -0,146866 |  |
| 27 | 0,037200 | 0,03 | 0,304265 |  |

R  Large residual

## Stepwise Selection of Terms

α to enter = 0,15; α to remove = 0,15

## Coded Coefficients

| Term | Coef | SE Coef | 95% CI | T-Value | P-Value | VIF |
| --- | --- | --- | --- | --- | --- | --- |
| Constant | 39,348 | 0,492 | (38,332; 40,364) | 79,96 | 0,000 |  |
| Lac | 6,62 | 1,04 | (4,46; 8,77) | 6,34 | 0,000 | 1,00 |
| HPMC\_PS | 2,77 | 1,23 | (0,23; 5,31) | 2,25 | 0,034 | 1,00 |

## Model Summary

| S | R-sq | R-sq(adj) | PRESS | R-sq(pred) | AICc | BIC |
| --- | --- | --- | --- | --- | --- | --- |
| 2,55653 | 65,35% | 62,46% | 192,158 | 57,55% | 133,95 | 137,31 |

## Analysis of Variance

| Source | DF | Seq SS | Contribution | Adj SS | Adj MS | F-Value | P-Value |
| --- | --- | --- | --- | --- | --- | --- | --- |
| Model | 2 | 295,79 | 65,35% | 295,79 | 147,896 | 22,63 | 0,000 |
| Linear | 2 | 295,79 | 65,35% | 295,79 | 147,896 | 22,63 | 0,000 |
| Lac | 1 | 262,71 | 58,04% | 262,71 | 262,710 | 40,20 | 0,000 |
| HPMC\_PS | 1 | 33,08 | 7,31% | 33,08 | 33,081 | 5,06 | 0,034 |
| Error | 24 | 156,86 | 34,65% | 156,86 | 6,536 |  |  |
| Lack-of-Fit | 22 | 135,19 | 29,87% | 135,19 | 6,145 | 0,57 | 0,805 |
| Pure Error | 2 | 21,67 | 4,79% | 21,67 | 10,835 |  |  |
| Total | 26 | 452,65 | 100,00% |  |  |  |  |

## Regression Equation in Uncoded Units

|  |  |  |
| --- | --- | --- |
| F\_mean\_3.5h(210min) | = | -0,1 + 26,47 Lac + 0,376 HPMC\_PS |

## Fits and Diagnostics for All Observations

| Obs | F\_mean\_3.5h(210min) | Fit | SE Fit | 95% CI | Resid | Std Resid | Del Resid |
| --- | --- | --- | --- | --- | --- | --- | --- |
| 1 | 36,866 | 35,355 | 0,775 | (33,755; 36,955) | 1,511 | 0,62 | 0,61 |
| 2 | 43,414 | 41,972 | 0,775 | (40,372; 43,572) | 1,442 | 0,59 | 0,58 |
| 3 | 31,856 | 34,438 | 1,003 | (32,367; 36,509) | -2,582 | -1,10 | -1,10 |
| 4 | 36,620 | 41,055 | 1,003 | (38,984; 43,126) | -4,435 | -1,89 | -2,00 |
| 5 | 33,315 | 35,564 | 0,745 | (34,026; 37,101) | -2,249 | -0,92 | -0,92 |
| 6 | 44,065 | 42,181 | 0,745 | (40,643; 43,718) | 1,884 | 0,77 | 0,76 |
| 7 | 33,956 | 34,961 | 0,857 | (33,192; 36,730) | -1,005 | -0,42 | -0,41 |
| 8 | 38,419 | 41,578 | 0,857 | (39,809; 43,347) | -3,159 | -1,31 | -1,33 |
| 9 | 35,760 | 37,188 | 0,886 | (35,359; 39,017) | -1,428 | -0,60 | -0,59 |
| 10 | 43,218 | 43,805 | 0,886 | (41,976; 45,634) | -0,587 | -0,24 | -0,24 |
| 11 | 37,353 | 36,623 | 0,766 | (35,042; 38,204) | 0,730 | 0,30 | 0,29 |
| 12 | 42,997 | 43,240 | 0,766 | (41,659; 44,821) | -0,243 | -0,10 | -0,10 |
| 13 | 35,261 | 37,479 | 0,968 | (35,481; 39,476) | -2,218 | -0,94 | -0,93 |
| 14 | 45,725 | 44,096 | 0,968 | (42,098; 46,093) | 1,629 | 0,69 | 0,68 |
| 15 | 38,758 | 36,778 | 0,793 | (35,142; 38,415) | 1,979 | 0,81 | 0,81 |
| 16 | 39,498 | 43,395 | 0,793 | (41,759; 45,032) | -3,897 | -1,60 | -1,66 |
| 17 | 32,680 | 32,635 | 1,154 | (30,253; 35,017) | 0,045 | 0,02 | 0,02 |
| 18 | 46,967 | 45,869 | 1,154 | (43,487; 48,252) | 1,098 | 0,48 | 0,47 |
| 19 | 40,842 | 39,606 | 0,508 | (38,558; 40,653) | 1,236 | 0,49 | 0,49 |
| 20 | 42,741 | 38,960 | 0,518 | (37,891; 40,030) | 3,781 | 1,51 | 1,55 |
| 21 | 39,143 | 39,058 | 0,506 | (38,013; 40,102) | 0,086 | 0,03 | 0,03 |
| 22 | 45,186 | 39,499 | 0,498 | (38,471; 40,526) | 5,687 | 2,27 | 2,50 |
| 23 | 38,661 | 36,580 | 1,316 | (33,864; 39,296) | 2,082 | 0,95 | 0,95 |
| 24 | 40,884 | 42,116 | 1,335 | (39,362; 44,871) | -1,233 | -0,57 | -0,56 |
| 25 | 37,155 | 39,252 | 0,493 | (38,234; 40,270) | -2,097 | -0,84 | -0,83 |
| 26 | 37,450 | 39,252 | 0,493 | (38,234; 40,270) | -1,802 | -0,72 | -0,71 |
| 27 | 42,998 | 39,252 | 0,493 | (38,234; 40,270) | 3,746 | 1,49 | 1,53 |

| Obs | HI | Cook’s D | DFITS |  |
| --- | --- | --- | --- | --- |
| 1 | 0,091956 | 0,01 | 0,194784 |  |
| 2 | 0,091956 | 0,01 | 0,185757 |  |
| 3 | 0,154060 | 0,07 | -0,470663 |  |
| 4 | 0,154060 | 0,22 | -0,853745 |  |
| 5 | 0,084910 | 0,03 | -0,279171 |  |
| 6 | 0,084910 | 0,02 | 0,232625 |  |
| 7 | 0,112397 | 0,01 | -0,145956 |  |
| 8 | 0,112397 | 0,07 | -0,474286 |  |
| 9 | 0,120141 | 0,02 | -0,217029 |  |
| 10 | 0,120141 | 0,00 | -0,088583 |  |
| 11 | 0,089807 | 0,00 | 0,092224 |  |
| 12 | 0,089807 | 0,00 | -0,030633 |  |
| 13 | 0,143301 | 0,05 | -0,382340 |  |
| 14 | 0,143301 | 0,03 | 0,278363 |  |
| 15 | 0,096233 | 0,02 | 0,263824 |  |
| 16 | 0,096233 | 0,09 | -0,542135 |  |
| 17 | 0,203866 | 0,00 | 0,009850 |  |
| 18 | 0,203866 | 0,02 | 0,239555 |  |
| 19 | 0,039415 | 0,00 | 0,098320 |  |
| 20 | 0,041065 | 0,03 | 0,321598 |  |
| 21 | 0,039207 | 0,00 | 0,006780 |  |
| 22 | 0,037944 | 0,07 | 0,497425 | R |
| 23 | 0,264924 | 0,11 | 0,568926 |  |
| 24 | 0,272505 | 0,04 | -0,341033 |  |
| 25 | 0,037200 | 0,01 | -0,163260 |  |
| 26 | 0,037200 | 0,01 | -0,139762 |  |
| 27 | 0,037200 | 0,03 | 0,301701 |  |

R  Large residual

## Stepwise Selection of Terms

α to enter = 0,15; α to remove = 0,15

## Coded Coefficients

| Term | Coef | SE Coef | 95% CI | T-Value | P-Value | VIF |
| --- | --- | --- | --- | --- | --- | --- |
| Constant | 42,172 | 0,496 | (41,148; 43,196) | 85,01 | 0,000 |  |
| Lac | 6,97 | 1,05 | (4,80; 9,14) | 6,62 | 0,000 | 1,00 |
| HPMC\_PS | 2,82 | 1,24 | (0,26; 5,38) | 2,27 | 0,032 | 1,00 |

## Model Summary

| S | R-sq | R-sq(adj) | PRESS | R-sq(pred) | AICc | BIC |
| --- | --- | --- | --- | --- | --- | --- |
| 2,57722 | 67,14% | 64,40% | 196,175 | 59,56% | 134,38 | 137,75 |

## Analysis of Variance

| Source | DF | Seq SS | Contribution | Adj SS | Adj MS | F-Value | P-Value |
| --- | --- | --- | --- | --- | --- | --- | --- |
| Model | 2 | 325,64 | 67,14% | 325,64 | 162,821 | 24,51 | 0,000 |
| Linear | 2 | 325,64 | 67,14% | 325,64 | 162,821 | 24,51 | 0,000 |
| Lac | 1 | 291,37 | 60,07% | 291,37 | 291,368 | 43,87 | 0,000 |
| HPMC\_PS | 1 | 34,27 | 7,07% | 34,27 | 34,273 | 5,16 | 0,032 |
| Error | 24 | 159,41 | 32,86% | 159,41 | 6,642 |  |  |
| Lack-of-Fit | 22 | 138,21 | 28,49% | 138,21 | 6,282 | 0,59 | 0,792 |
| Pure Error | 2 | 21,20 | 4,37% | 21,20 | 10,600 |  |  |
| Total | 26 | 485,05 | 100,00% |  |  |  |  |

## Regression Equation in Uncoded Units

|  |  |  |
| --- | --- | --- |
| F\_mean\_4h(240min) | = | 1,5 + 27,87 Lac + 0,383 HPMC\_PS |

## Fits and Diagnostics for All Observations

| Obs | F\_mean\_4h(240min) | Fit | SE Fit | 95% CI | Resid | Std Resid | Del Resid |
| --- | --- | --- | --- | --- | --- | --- | --- |
| 1 | 39,017 | 37,990 | 0,782 | (36,377; 39,603) | 1,026 | 0,42 | 0,41 |
| 2 | 46,450 | 44,959 | 0,782 | (43,346; 46,572) | 1,491 | 0,61 | 0,60 |
| 3 | 34,367 | 37,057 | 1,012 | (34,969; 39,145) | -2,690 | -1,13 | -1,14 |
| 4 | 39,535 | 44,026 | 1,012 | (41,938; 46,114) | -4,491 | -1,89 | -2,01 |
| 5 | 36,008 | 38,203 | 0,751 | (36,653; 39,753) | -2,195 | -0,89 | -0,89 |
| 6 | 46,980 | 45,172 | 0,751 | (43,622; 46,722) | 1,808 | 0,73 | 0,73 |
| 7 | 36,677 | 37,590 | 0,864 | (35,806; 39,373) | -0,912 | -0,38 | -0,37 |
| 8 | 41,293 | 44,558 | 0,864 | (42,775; 46,342) | -3,266 | -1,34 | -1,37 |
| 9 | 38,416 | 39,856 | 0,893 | (38,012; 41,700) | -1,440 | -0,60 | -0,59 |
| 10 | 46,211 | 46,825 | 0,893 | (44,981; 48,668) | -0,613 | -0,25 | -0,25 |
| 11 | 40,034 | 39,281 | 0,772 | (37,687; 40,875) | 0,753 | 0,31 | 0,30 |
| 12 | 45,897 | 46,250 | 0,772 | (44,656; 47,844) | -0,353 | -0,14 | -0,14 |
| 13 | 38,009 | 40,152 | 0,976 | (38,139; 42,166) | -2,143 | -0,90 | -0,89 |
| 14 | 48,773 | 47,121 | 0,976 | (45,107; 49,134) | 1,652 | 0,69 | 0,68 |
| 15 | 41,448 | 39,439 | 0,799 | (37,789; 41,089) | 2,008 | 0,82 | 0,81 |
| 16 | 42,454 | 46,408 | 0,799 | (44,758; 48,058) | -3,954 | -1,61 | -1,67 |
| 17 | 35,187 | 35,105 | 1,164 | (32,704; 37,507) | 0,081 | 0,04 | 0,03 |
| 18 | 50,190 | 49,043 | 1,164 | (46,641; 51,444) | 1,148 | 0,50 | 0,49 |
| 19 | 43,871 | 42,434 | 0,512 | (41,378; 43,490) | 1,437 | 0,57 | 0,56 |
| 20 | 45,413 | 41,777 | 0,522 | (40,699; 42,855) | 3,636 | 1,44 | 1,48 |
| 21 | 41,861 | 41,876 | 0,510 | (40,823; 42,929) | -0,015 | -0,01 | -0,01 |
| 22 | 48,167 | 42,325 | 0,502 | (41,289; 43,361) | 5,842 | 2,31 | 2,57 |
| 23 | 41,719 | 39,354 | 1,327 | (36,616; 42,092) | 2,365 | 1,07 | 1,07 |
| 24 | 43,771 | 44,989 | 1,345 | (42,213; 47,766) | -1,218 | -0,55 | -0,55 |
| 25 | 40,004 | 42,074 | 0,497 | (41,048; 43,100) | -2,070 | -0,82 | -0,81 |
| 26 | 40,421 | 42,074 | 0,497 | (41,048; 43,100) | -1,653 | -0,65 | -0,65 |
| 27 | 45,840 | 42,074 | 0,497 | (41,048; 43,100) | 3,766 | 1,49 | 1,53 |

| Obs | HI | Cook’s D | DFITS |  |
| --- | --- | --- | --- | --- |
| 1 | 0,091956 | 0,01 | 0,130687 |  |
| 2 | 0,091956 | 0,01 | 0,190608 |  |
| 3 | 0,154060 | 0,08 | -0,487412 |  |
| 4 | 0,154060 | 0,22 | -0,858199 |  |
| 5 | 0,084910 | 0,02 | -0,269984 |  |
| 6 | 0,084910 | 0,02 | 0,221184 |  |
| 7 | 0,112397 | 0,01 | -0,131272 |  |
| 8 | 0,112397 | 0,08 | -0,487236 |  |
| 9 | 0,120141 | 0,02 | -0,217052 |  |
| 10 | 0,120141 | 0,00 | -0,091913 |  |
| 11 | 0,089807 | 0,00 | 0,094298 |  |
| 12 | 0,089807 | 0,00 | -0,044179 |  |
| 13 | 0,143301 | 0,04 | -0,365890 |  |
| 14 | 0,143301 | 0,03 | 0,280127 |  |
| 15 | 0,096233 | 0,02 | 0,265585 |  |
| 16 | 0,096233 | 0,09 | -0,546035 |  |
| 17 | 0,203866 | 0,00 | 0,017536 |  |
| 18 | 0,203866 | 0,02 | 0,248539 |  |
| 19 | 0,039415 | 0,00 | 0,113608 |  |
| 20 | 0,041065 | 0,03 | 0,305345 |  |
| 21 | 0,039207 | 0,00 | -0,001168 |  |
| 22 | 0,037944 | 0,07 | 0,509535 | R |
| 23 | 0,264924 | 0,14 | 0,644663 |  |
| 24 | 0,272505 | 0,04 | -0,334211 |  |
| 25 | 0,037200 | 0,01 | -0,159770 |  |
| 26 | 0,037200 | 0,01 | -0,126932 |  |
| 27 | 0,037200 | 0,03 | 0,300798 |  |

R  Large residual

## Stepwise Selection of Terms

α to enter = 0,15; α to remove = 0,15

## Coded Coefficients

| Term | Coef | SE Coef | 95% CI | T-Value | P-Value | VIF |
| --- | --- | --- | --- | --- | --- | --- |
| Constant | 44,874 | 0,505 | (43,831; 45,917) | 88,83 | 0,000 |  |
| Lac | 7,27 | 1,07 | (5,06; 9,49) | 6,79 | 0,000 | 1,00 |
| HPMC\_PS | 2,83 | 1,26 | (0,22; 5,43) | 2,24 | 0,035 | 1,00 |

## Model Summary

| S | R-sq | R-sq(adj) | PRESS | R-sq(pred) | AICc | BIC |
| --- | --- | --- | --- | --- | --- | --- |
| 2,62438 | 68,05% | 65,39% | 204,552 | 60,46% | 135,36 | 138,73 |

## Analysis of Variance

| Source | DF | Seq SS | Contribution | Adj SS | Adj MS | F-Value | P-Value |
| --- | --- | --- | --- | --- | --- | --- | --- |
| Model | 2 | 352,04 | 68,05% | 352,04 | 176,018 | 25,56 | 0,000 |
| Linear | 2 | 352,04 | 68,05% | 352,04 | 176,018 | 25,56 | 0,000 |
| Lac | 1 | 317,55 | 61,38% | 317,55 | 317,551 | 46,11 | 0,000 |
| HPMC\_PS | 1 | 34,49 | 6,67% | 34,49 | 34,486 | 5,01 | 0,035 |
| Error | 24 | 165,30 | 31,95% | 165,30 | 6,887 |  |  |
| Lack-of-Fit | 22 | 144,62 | 27,95% | 144,62 | 6,573 | 0,64 | 0,770 |
| Pure Error | 2 | 20,68 | 4,00% | 20,68 | 10,340 |  |  |
| Total | 26 | 517,33 | 100,00% |  |  |  |  |

## Regression Equation in Uncoded Units

|  |  |  |
| --- | --- | --- |
| F\_mean\_4.5h(270min) | = | 3,6 + 29,10 Lac + 0,384 HPMC\_PS |

## Fits and Diagnostics for All Observations

| Obs | F\_mean\_4.5h(270min) | Fit | SE Fit | 95% CI | Resid | Std Resid | Del Resid |
| --- | --- | --- | --- | --- | --- | --- | --- |
| 1 | 41,486 | 40,537 | 0,796 | (38,895; 42,180) | 0,948 | 0,38 | 0,37 |
| 2 | 49,332 | 47,812 | 0,796 | (46,170; 49,455) | 1,519 | 0,61 | 0,60 |
| 3 | 36,774 | 39,601 | 1,030 | (37,475; 41,727) | -2,827 | -1,17 | -1,18 |
| 4 | 42,246 | 46,876 | 1,030 | (44,750; 49,002) | -4,630 | -1,92 | -2,04 |
| 5 | 38,574 | 40,751 | 0,765 | (39,172; 42,329) | -2,177 | -0,87 | -0,86 |
| 6 | 49,792 | 48,026 | 0,765 | (46,447; 49,604) | 1,766 | 0,70 | 0,70 |
| 7 | 39,260 | 40,135 | 0,880 | (38,320; 41,951) | -0,876 | -0,35 | -0,35 |
| 8 | 44,011 | 47,410 | 0,880 | (45,594; 49,226) | -3,399 | -1,37 | -1,40 |
| 9 | 40,920 | 42,409 | 0,910 | (40,531; 44,286) | -1,489 | -0,60 | -0,60 |
| 10 | 49,100 | 49,684 | 0,910 | (47,806; 51,561) | -0,584 | -0,24 | -0,23 |
| 11 | 42,660 | 41,832 | 0,786 | (40,209; 43,455) | 0,828 | 0,33 | 0,32 |
| 12 | 48,643 | 49,107 | 0,786 | (47,484; 50,730) | -0,464 | -0,19 | -0,18 |
| 13 | 40,531 | 42,706 | 0,993 | (40,656; 44,756) | -2,175 | -0,90 | -0,89 |
| 14 | 51,641 | 49,981 | 0,993 | (47,930; 52,031) | 1,661 | 0,68 | 0,68 |
| 15 | 44,033 | 41,991 | 0,814 | (40,311; 43,671) | 2,042 | 0,82 | 0,81 |
| 16 | 45,279 | 49,266 | 0,814 | (47,586; 50,946) | -3,987 | -1,60 | -1,65 |
| 17 | 37,538 | 37,501 | 1,185 | (35,055; 39,947) | 0,037 | 0,02 | 0,02 |
| 18 | 53,285 | 52,051 | 1,185 | (49,605; 54,497) | 1,234 | 0,53 | 0,52 |
| 19 | 46,770 | 45,137 | 0,521 | (44,062; 46,213) | 1,633 | 0,63 | 0,63 |
| 20 | 48,028 | 44,478 | 0,532 | (43,381; 45,576) | 3,550 | 1,38 | 1,41 |
| 21 | 44,425 | 44,577 | 0,520 | (43,505; 45,650) | -0,153 | -0,06 | -0,06 |
| 22 | 50,993 | 45,028 | 0,511 | (43,973; 46,083) | 5,965 | 2,32 | 2,57 |
| 23 | 44,696 | 42,047 | 1,351 | (39,260; 44,835) | 2,649 | 1,18 | 1,19 |
| 24 | 46,531 | 47,700 | 1,370 | (44,873; 50,528) | -1,170 | -0,52 | -0,51 |
| 25 | 42,724 | 44,776 | 0,506 | (43,731; 45,821) | -2,052 | -0,80 | -0,79 |
| 26 | 43,190 | 44,776 | 0,506 | (43,731; 45,821) | -1,586 | -0,62 | -0,61 |
| 27 | 48,512 | 44,776 | 0,506 | (43,731; 45,821) | 3,736 | 1,45 | 1,49 |

| Obs | HI | Cook’s D | DFITS |  |
| --- | --- | --- | --- | --- |
| 1 | 0,091956 | 0,00 | 0,118504 |  |
| 2 | 0,091956 | 0,01 | 0,190738 |  |
| 3 | 0,154060 | 0,08 | -0,503915 |  |
| 4 | 0,154060 | 0,22 | -0,870890 |  |
| 5 | 0,084910 | 0,02 | -0,262726 |  |
| 6 | 0,084910 | 0,02 | 0,211986 |  |
| 7 | 0,112397 | 0,01 | -0,123699 |  |
| 8 | 0,112397 | 0,08 | -0,498968 |  |
| 9 | 0,120141 | 0,02 | -0,220490 |  |
| 10 | 0,120141 | 0,00 | -0,085916 |  |
| 11 | 0,089807 | 0,00 | 0,101956 |  |
| 12 | 0,089807 | 0,00 | -0,057065 |  |
| 13 | 0,143301 | 0,04 | -0,364637 |  |
| 14 | 0,143301 | 0,03 | 0,276402 |  |
| 15 | 0,096233 | 0,02 | 0,265227 |  |
| 16 | 0,096233 | 0,09 | -0,539969 |  |
| 17 | 0,203866 | 0,00 | 0,007861 |  |
| 18 | 0,203866 | 0,02 | 0,262566 |  |
| 19 | 0,039415 | 0,01 | 0,126986 |  |
| 20 | 0,041065 | 0,03 | 0,291651 |  |
| 21 | 0,039207 | 0,00 | -0,011737 |  |
| 22 | 0,037944 | 0,07 | 0,511375 | R |
| 23 | 0,264924 | 0,17 | 0,712670 |  |
| 24 | 0,272505 | 0,03 | -0,314837 |  |
| 25 | 0,037200 | 0,01 | -0,155439 |  |
| 26 | 0,037200 | 0,00 | -0,119484 |  |
| 27 | 0,037200 | 0,03 | 0,292254 |  |

R  Large residual

## Stepwise Selection of Terms

α to enter = 0,15; α to remove = 0,15

## Coded Coefficients

| Term | Coef | SE Coef | 95% CI | T-Value | P-Value | VIF |
| --- | --- | --- | --- | --- | --- | --- |
| Constant | 47,484 | 0,513 | (46,426; 48,543) | 92,61 | 0,000 |  |
| Lac | 7,57 | 1,09 | (5,33; 9,82) | 6,96 | 0,000 | 1,00 |
| HPMC\_PS | 2,80 | 1,28 | (0,15; 5,44) | 2,18 | 0,039 | 1,00 |

## Model Summary

| S | R-sq | R-sq(adj) | PRESS | R-sq(pred) | AICc | BIC |
| --- | --- | --- | --- | --- | --- | --- |
| 2,66381 | 68,94% | 66,35% | 211,672 | 61,40% | 136,17 | 139,53 |

## Analysis of Variance

| Source | DF | Seq SS | Contribution | Adj SS | Adj MS | F-Value | P-Value |
| --- | --- | --- | --- | --- | --- | --- | --- |
| Model | 2 | 378,01 | 68,94% | 378,01 | 189,004 | 26,64 | 0,000 |
| Linear | 2 | 378,01 | 68,94% | 378,01 | 189,004 | 26,64 | 0,000 |
| Lac | 1 | 344,22 | 62,78% | 344,22 | 344,216 | 48,51 | 0,000 |
| HPMC\_PS | 1 | 33,79 | 6,16% | 33,79 | 33,792 | 4,76 | 0,039 |
| Error | 24 | 170,30 | 31,06% | 170,30 | 7,096 |  |  |
| Lack-of-Fit | 22 | 150,04 | 27,36% | 150,04 | 6,820 | 0,67 | 0,752 |
| Pure Error | 2 | 20,26 | 3,70% | 20,26 | 10,132 |  |  |
| Total | 26 | 548,31 | 100,00% |  |  |  |  |

## Regression Equation in Uncoded Units

|  |  |  |
| --- | --- | --- |
| F\_mean\_5h(300min) | = | 5,8 + 30,30 Lac + 0,380 HPMC\_PS |

## Fits and Diagnostics for All Observations

| Obs | F\_mean\_5h(300min) | Fit | SE Fit | 95% CI | Resid | Std Resid | Del Resid |
| --- | --- | --- | --- | --- | --- | --- | --- |
| 1 | 43,974 | 43,005 | 0,808 | (41,338; 44,672) | 0,969 | 0,38 | 0,37 |
| 2 | 52,109 | 50,579 | 0,808 | (48,912; 52,247) | 1,529 | 0,60 | 0,59 |
| 3 | 39,103 | 42,079 | 1,046 | (39,921; 44,236) | -2,975 | -1,21 | -1,23 |
| 4 | 44,902 | 49,653 | 1,046 | (47,495; 51,811) | -4,751 | -1,94 | -2,07 |
| 5 | 40,987 | 43,216 | 0,776 | (41,614; 44,818) | -2,229 | -0,87 | -0,87 |
| 6 | 52,477 | 50,791 | 0,776 | (49,189; 52,393) | 1,686 | 0,66 | 0,65 |
| 7 | 41,891 | 42,607 | 0,893 | (40,764; 44,450) | -0,716 | -0,29 | -0,28 |
| 8 | 46,673 | 50,182 | 0,893 | (48,338; 52,025) | -3,508 | -1,40 | -1,43 |
| 9 | 43,320 | 44,858 | 0,923 | (42,952; 46,763) | -1,537 | -0,62 | -0,61 |
| 10 | 51,859 | 52,432 | 0,923 | (50,526; 54,337) | -0,572 | -0,23 | -0,22 |
| 11 | 45,159 | 44,287 | 0,798 | (42,639; 45,934) | 0,872 | 0,34 | 0,34 |
| 12 | 51,236 | 51,861 | 0,798 | (50,213; 53,509) | -0,625 | -0,25 | -0,24 |
| 13 | 43,025 | 45,152 | 1,008 | (43,071; 47,233) | -2,126 | -0,86 | -0,86 |
| 14 | 54,479 | 52,726 | 1,008 | (50,645; 54,807) | 1,753 | 0,71 | 0,70 |
| 15 | 46,291 | 44,444 | 0,826 | (42,738; 46,149) | 1,847 | 0,73 | 0,72 |
| 16 | 48,024 | 52,018 | 0,826 | (50,313; 53,724) | -3,994 | -1,58 | -1,63 |
| 17 | 39,834 | 39,813 | 1,203 | (37,331; 42,295) | 0,021 | 0,01 | 0,01 |
| 18 | 56,276 | 54,962 | 1,203 | (52,479; 57,444) | 1,314 | 0,55 | 0,54 |
| 19 | 49,589 | 47,745 | 0,529 | (46,653; 48,836) | 1,844 | 0,71 | 0,70 |
| 20 | 50,538 | 47,092 | 0,540 | (45,978; 48,207) | 3,446 | 1,32 | 1,34 |
| 21 | 46,884 | 47,191 | 0,527 | (46,102; 48,279) | -0,306 | -0,12 | -0,11 |
| 22 | 53,739 | 47,636 | 0,519 | (46,566; 48,707) | 6,103 | 2,34 | 2,60 |
| 23 | 47,568 | 44,686 | 1,371 | (41,857; 47,516) | 2,881 | 1,26 | 1,28 |
| 24 | 49,163 | 50,282 | 1,391 | (47,412; 53,152) | -1,119 | -0,49 | -0,48 |
| 25 | 45,315 | 47,387 | 0,514 | (46,327; 48,448) | -2,073 | -0,79 | -0,79 |
| 26 | 45,931 | 47,387 | 0,514 | (46,327; 48,448) | -1,456 | -0,56 | -0,55 |
| 27 | 51,110 | 47,387 | 0,514 | (46,327; 48,448) | 3,723 | 1,42 | 1,46 |

| Obs | HI | Cook’s D | DFITS |  |
| --- | --- | --- | --- | --- |
| 1 | 0,091956 | 0,00 | 0,119256 |  |
| 2 | 0,091956 | 0,01 | 0,189131 |  |
| 3 | 0,154060 | 0,09 | -0,523631 |  |
| 4 | 0,154060 | 0,23 | -0,882099 |  |
| 5 | 0,084910 | 0,02 | -0,265104 |  |
| 6 | 0,084910 | 0,01 | 0,199145 |  |
| 7 | 0,112397 | 0,00 | -0,099530 |  |
| 8 | 0,112397 | 0,08 | -0,508097 |  |
| 9 | 0,120141 | 0,02 | -0,224350 |  |
| 10 | 0,120141 | 0,00 | -0,082961 |  |
| 11 | 0,089807 | 0,00 | 0,105803 |  |
| 12 | 0,089807 | 0,00 | -0,075741 |  |
| 13 | 0,143301 | 0,04 | -0,350776 |  |
| 14 | 0,143301 | 0,03 | 0,287644 |  |
| 15 | 0,096233 | 0,02 | 0,235602 |  |
| 16 | 0,096233 | 0,09 | -0,532130 |  |
| 17 | 0,203866 | 0,00 | 0,004349 |  |
| 18 | 0,203866 | 0,03 | 0,275668 |  |
| 19 | 0,039415 | 0,01 | 0,141543 |  |
| 20 | 0,041065 | 0,02 | 0,277888 |  |
| 21 | 0,039207 | 0,00 | -0,023206 |  |
| 22 | 0,037944 | 0,07 | 0,516623 | R |
| 23 | 0,264924 | 0,19 | 0,767327 |  |
| 24 | 0,272505 | 0,03 | -0,296570 |  |
| 25 | 0,037200 | 0,01 | -0,154634 |  |
| 26 | 0,037200 | 0,00 | -0,107897 |  |
| 27 | 0,037200 | 0,03 | 0,286455 |  |

R  Large residual

## Stepwise Selection of Terms

α to enter = 0,15; α to remove = 0,15

## Coded Coefficients

| Term | Coef | SE Coef | 95% CI | T-Value | P-Value | VIF |
| --- | --- | --- | --- | --- | --- | --- |
| Constant | 50,002 | 0,520 | (48,929; 51,075) | 96,17 | 0,000 |  |
| Lac | 7,84 | 1,10 | (5,56; 10,12) | 7,11 | 0,000 | 1,00 |
| HPMC\_PS | 2,78 | 1,30 | (0,10; 5,46) | 2,14 | 0,043 | 1,00 |

## Model Summary

| S | R-sq | R-sq(adj) | PRESS | R-sq(pred) | AICc | BIC |
| --- | --- | --- | --- | --- | --- | --- |
| 2,70108 | 69,67% | 67,14% | 218,699 | 62,12% | 136,92 | 140,28 |

## Analysis of Variance

| Source | DF | Seq SS | Contribution | Adj SS | Adj MS | F-Value | P-Value |
| --- | --- | --- | --- | --- | --- | --- | --- |
| Model | 2 | 402,25 | 69,67% | 402,25 | 201,124 | 27,57 | 0,000 |
| Linear | 2 | 402,25 | 69,67% | 402,25 | 201,124 | 27,57 | 0,000 |
| Lac | 1 | 368,86 | 63,89% | 368,86 | 368,864 | 50,56 | 0,000 |
| HPMC\_PS | 1 | 33,38 | 5,78% | 33,38 | 33,383 | 4,58 | 0,043 |
| Error | 24 | 175,10 | 30,33% | 175,10 | 7,296 |  |  |
| Lack-of-Fit | 22 | 155,30 | 26,90% | 155,30 | 7,059 | 0,71 | 0,733 |
| Pure Error | 2 | 19,80 | 3,43% | 19,80 | 9,899 |  |  |
| Total | 26 | 577,35 | 100,00% |  |  |  |  |

## Regression Equation in Uncoded Units

|  |  |  |
| --- | --- | --- |
| F\_mean\_5.5h(330min) | = | 8,0 + 31,36 Lac + 0,378 HPMC\_PS |

## Fits and Diagnostics for All Observations

| Obs | F\_mean\_5.5h(330min) | Fit | SE Fit | 95% CI | Resid | Std Resid | Del Resid |
| --- | --- | --- | --- | --- | --- | --- | --- |
| 1 | 46,429 | 45,393 | 0,819 | (43,703; 47,084) | 1,036 | 0,40 | 0,40 |
| 2 | 54,757 | 53,234 | 0,819 | (51,544; 54,925) | 1,523 | 0,59 | 0,58 |
| 3 | 41,355 | 44,473 | 1,060 | (42,284; 46,661) | -3,118 | -1,25 | -1,27 |
| 4 | 47,454 | 52,313 | 1,060 | (50,125; 54,501) | -4,859 | -1,96 | -2,09 |
| 5 | 43,398 | 45,603 | 0,787 | (43,979; 47,228) | -2,205 | -0,85 | -0,85 |
| 6 | 55,109 | 53,444 | 0,787 | (51,820; 55,069) | 1,665 | 0,64 | 0,64 |
| 7 | 44,340 | 44,998 | 0,906 | (43,129; 46,867) | -0,658 | -0,26 | -0,25 |
| 8 | 49,205 | 52,839 | 0,906 | (50,970; 54,708) | -3,633 | -1,43 | -1,46 |
| 9 | 45,593 | 47,235 | 0,936 | (45,302; 49,167) | -1,642 | -0,65 | -0,64 |
| 10 | 54,540 | 55,075 | 0,936 | (53,143; 57,008) | -0,536 | -0,21 | -0,21 |
| 11 | 47,604 | 46,667 | 0,809 | (44,997; 48,338) | 0,937 | 0,36 | 0,36 |
| 12 | 53,701 | 54,508 | 0,809 | (52,838; 56,179) | -0,807 | -0,31 | -0,31 |
| 13 | 45,418 | 47,527 | 1,022 | (45,417; 49,637) | -2,109 | -0,84 | -0,84 |
| 14 | 57,186 | 55,368 | 1,022 | (53,257; 57,478) | 1,818 | 0,73 | 0,72 |
| 15 | 48,594 | 46,824 | 0,838 | (45,094; 48,553) | 1,770 | 0,69 | 0,68 |
| 16 | 50,739 | 54,664 | 0,838 | (52,935; 56,394) | -3,926 | -1,53 | -1,58 |
| 17 | 42,074 | 42,065 | 1,220 | (39,547; 44,582) | 0,009 | 0,00 | 0,00 |
| 18 | 59,138 | 57,746 | 1,220 | (55,229; 60,263) | 1,392 | 0,58 | 0,57 |
| 19 | 52,302 | 50,261 | 0,536 | (49,154; 51,367) | 2,042 | 0,77 | 0,76 |
| 20 | 52,869 | 49,612 | 0,547 | (48,483; 50,742) | 3,257 | 1,23 | 1,25 |
| 21 | 49,290 | 49,710 | 0,535 | (48,606; 50,814) | -0,420 | -0,16 | -0,16 |
| 22 | 56,382 | 50,153 | 0,526 | (49,067; 51,239) | 6,229 | 2,35 | 2,62 |
| 23 | 50,310 | 47,221 | 1,390 | (44,351; 50,090) | 3,089 | 1,33 | 1,36 |
| 24 | 51,668 | 52,783 | 1,410 | (49,873; 55,693) | -1,114 | -0,48 | -0,48 |
| 25 | 47,852 | 49,905 | 0,521 | (48,830; 50,981) | -2,054 | -0,77 | -0,77 |
| 26 | 48,521 | 49,905 | 0,521 | (48,830; 50,981) | -1,384 | -0,52 | -0,51 |
| 27 | 53,605 | 49,905 | 0,521 | (48,830; 50,981) | 3,699 | 1,40 | 1,43 |

| Obs | HI | Cook’s D | DFITS |  |
| --- | --- | --- | --- | --- |
| 1 | 0,091956 | 0,01 | 0,125808 |  |
| 2 | 0,091956 | 0,01 | 0,185650 |  |
| 3 | 0,154060 | 0,10 | -0,542391 |  |
| 4 | 0,154060 | 0,23 | -0,891309 |  |
| 5 | 0,084910 | 0,02 | -0,258463 |  |
| 6 | 0,084910 | 0,01 | 0,193830 |  |
| 7 | 0,112397 | 0,00 | -0,090248 |  |
| 8 | 0,112397 | 0,09 | -0,519957 |  |
| 9 | 0,120141 | 0,02 | -0,236475 |  |
| 10 | 0,120141 | 0,00 | -0,076540 |  |
| 11 | 0,089807 | 0,00 | 0,112102 |  |
| 12 | 0,089807 | 0,00 | -0,096517 |  |
| 13 | 0,143301 | 0,04 | -0,342898 |  |
| 14 | 0,143301 | 0,03 | 0,294454 |  |
| 15 | 0,096233 | 0,02 | 0,222433 |  |
| 16 | 0,096233 | 0,08 | -0,514033 |  |
| 17 | 0,203866 | 0,00 | 0,001920 |  |
| 18 | 0,203866 | 0,03 | 0,288220 |  |
| 19 | 0,039415 | 0,01 | 0,154871 |  |
| 20 | 0,041065 | 0,02 | 0,257695 |  |
| 21 | 0,039207 | 0,00 | -0,031399 |  |
| 22 | 0,037944 | 0,07 | 0,520975 | R |
| 23 | 0,264924 | 0,21 | 0,814831 |  |
| 24 | 0,272505 | 0,03 | -0,291242 |  |
| 25 | 0,037200 | 0,01 | -0,151010 |  |
| 26 | 0,037200 | 0,00 | -0,101093 |  |
| 27 | 0,037200 | 0,03 | 0,280199 |  |

R  Large residual

## Stepwise Selection of Terms

α to enter = 0,15; α to remove = 0,15

## Coded Coefficients

| Term | Coef | SE Coef | 95% CI | T-Value | P-Value | VIF |
| --- | --- | --- | --- | --- | --- | --- |
| Constant | 52,444 | 0,527 | (51,355; 53,532) | 99,45 | 0,000 |  |
| Lac | 8,08 | 1,12 | (5,77; 10,39) | 7,22 | 0,000 | 1,00 |
| HPMC\_PS | 2,78 | 1,32 | (0,06; 5,50) | 2,11 | 0,046 | 1,00 |

## Model Summary

| S | R-sq | R-sq(adj) | PRESS | R-sq(pred) | AICc | BIC |
| --- | --- | --- | --- | --- | --- | --- |
| 2,73949 | 70,23% | 67,74% | 225,893 | 62,66% | 137,68 | 141,05 |

## Analysis of Variance

| Source | DF | Seq SS | Contribution | Adj SS | Adj MS | F-Value | P-Value |
| --- | --- | --- | --- | --- | --- | --- | --- |
| Model | 2 | 424,82 | 70,23% | 424,82 | 212,408 | 28,30 | 0,000 |
| Linear | 2 | 424,82 | 70,23% | 424,82 | 212,408 | 28,30 | 0,000 |
| Lac | 1 | 391,44 | 64,71% | 391,44 | 391,437 | 52,16 | 0,000 |
| HPMC\_PS | 1 | 33,38 | 5,52% | 33,38 | 33,380 | 4,45 | 0,046 |
| Error | 24 | 180,11 | 29,77% | 180,11 | 7,505 |  |  |
| Lack-of-Fit | 22 | 160,94 | 26,61% | 160,94 | 7,316 | 0,76 | 0,710 |
| Pure Error | 2 | 19,17 | 3,17% | 19,17 | 9,585 |  |  |
| Total | 26 | 604,93 | 100,00% |  |  |  |  |

## Regression Equation in Uncoded Units

|  |  |  |
| --- | --- | --- |
| F\_mean\_6h(360min) | = | 10,0 + 32,31 Lac + 0,378 HPMC\_PS |

## Fits and Diagnostics for All Observations

| Obs | F\_mean\_6h(360min) | Fit | SE Fit | 95% CI | Resid | Std Resid | Del Resid |
| --- | --- | --- | --- | --- | --- | --- | --- |
| 1 | 48,896 | 47,717 | 0,831 | (46,003; 49,432) | 1,179 | 0,45 | 0,44 |
| 2 | 57,276 | 55,794 | 0,831 | (54,080; 57,509) | 1,482 | 0,57 | 0,56 |
| 3 | 43,503 | 46,796 | 1,075 | (44,577; 49,016) | -3,293 | -1,31 | -1,33 |
| 4 | 49,901 | 54,874 | 1,075 | (52,654; 57,093) | -4,972 | -1,97 | -2,11 |
| 5 | 45,732 | 47,927 | 0,798 | (46,280; 49,575) | -2,196 | -0,84 | -0,83 |
| 6 | 57,664 | 56,004 | 0,798 | (54,357; 57,652) | 1,660 | 0,63 | 0,63 |
| 7 | 46,726 | 47,322 | 0,918 | (45,426; 49,217) | -0,596 | -0,23 | -0,23 |
| 8 | 51,682 | 55,399 | 0,918 | (53,503; 57,295) | -3,717 | -1,44 | -1,48 |
| 9 | 47,788 | 49,559 | 0,950 | (47,599; 51,518) | -1,771 | -0,69 | -0,68 |
| 10 | 57,117 | 57,636 | 0,950 | (55,676; 59,595) | -0,519 | -0,20 | -0,20 |
| 11 | 49,996 | 48,991 | 0,821 | (47,297; 50,686) | 1,005 | 0,38 | 0,38 |
| 12 | 56,138 | 57,068 | 0,821 | (55,374; 58,763) | -0,931 | -0,36 | -0,35 |
| 13 | 47,758 | 49,851 | 1,037 | (47,711; 51,991) | -2,092 | -0,83 | -0,82 |
| 14 | 59,764 | 57,928 | 1,037 | (55,788; 60,068) | 1,836 | 0,72 | 0,72 |
| 15 | 50,892 | 49,147 | 0,850 | (47,393; 50,901) | 1,745 | 0,67 | 0,66 |
| 16 | 53,398 | 57,224 | 0,850 | (55,471; 58,978) | -3,827 | -1,47 | -1,51 |
| 17 | 44,235 | 44,270 | 1,237 | (41,717; 46,823) | -0,036 | -0,01 | -0,01 |
| 18 | 61,873 | 60,424 | 1,237 | (57,872; 62,977) | 1,449 | 0,59 | 0,58 |
| 19 | 54,961 | 52,703 | 0,544 | (51,580; 53,825) | 2,258 | 0,84 | 0,84 |
| 20 | 55,193 | 52,054 | 0,555 | (50,909; 53,200) | 3,139 | 1,17 | 1,18 |
| 21 | 51,567 | 52,152 | 0,542 | (51,032; 53,271) | -0,585 | -0,22 | -0,21 |
| 22 | 58,919 | 52,595 | 0,534 | (51,494; 53,696) | 6,324 | 2,35 | 2,63 |
| 23 | 52,923 | 49,663 | 1,410 | (46,753; 52,573) | 3,260 | 1,39 | 1,42 |
| 24 | 54,096 | 55,225 | 1,430 | (52,273; 58,176) | -1,129 | -0,48 | -0,48 |
| 25 | 50,285 | 52,347 | 0,528 | (51,257; 53,438) | -2,062 | -0,77 | -0,76 |
| 26 | 51,083 | 52,347 | 0,528 | (51,257; 53,438) | -1,264 | -0,47 | -0,46 |
| 27 | 56,002 | 52,347 | 0,528 | (51,257; 53,438) | 3,654 | 1,36 | 1,39 |

| Obs | HI | Cook’s D | DFITS |  |
| --- | --- | --- | --- | --- |
| 1 | 0,091956 | 0,01 | 0,141252 |  |
| 2 | 0,091956 | 0,01 | 0,178032 |  |
| 3 | 0,154060 | 0,10 | -0,566532 |  |
| 4 | 0,154060 | 0,24 | -0,900707 |  |
| 5 | 0,084910 | 0,02 | -0,253592 |  |
| 6 | 0,084910 | 0,01 | 0,190451 |  |
| 7 | 0,112397 | 0,00 | -0,080509 |  |
| 8 | 0,112397 | 0,09 | -0,524946 |  |
| 9 | 0,120141 | 0,02 | -0,251746 |  |
| 10 | 0,120141 | 0,00 | -0,073077 |  |
| 11 | 0,089807 | 0,00 | 0,118580 |  |
| 12 | 0,089807 | 0,00 | -0,109806 |  |
| 13 | 0,143301 | 0,04 | -0,335191 |  |
| 14 | 0,143301 | 0,03 | 0,293150 |  |
| 15 | 0,096233 | 0,02 | 0,216014 |  |
| 16 | 0,096233 | 0,08 | -0,492058 |  |
| 17 | 0,203866 | 0,00 | -0,007239 |  |
| 18 | 0,203866 | 0,03 | 0,295756 |  |
| 19 | 0,039415 | 0,01 | 0,169274 |  |
| 20 | 0,041065 | 0,02 | 0,244069 |  |
| 21 | 0,039207 | 0,00 | -0,043119 |  |
| 22 | 0,037944 | 0,07 | 0,521748 | R |
| 23 | 0,264924 | 0,23 | 0,850548 |  |
| 24 | 0,272505 | 0,03 | -0,290852 |  |
| 25 | 0,037200 | 0,01 | -0,149469 |  |
| 26 | 0,037200 | 0,00 | -0,090918 |  |
| 27 | 0,037200 | 0,02 | 0,272301 |  |

R  Large residual

## Stepwise Selection of Terms

α to enter = 0,15; α to remove = 0,15

## Coded Coefficients

| Term | Coef | SE Coef | 95% CI | T-Value | P-Value | VIF |
| --- | --- | --- | --- | --- | --- | --- |
| Constant | 57,035 | 0,496 | (56,006; 58,065) | 114,89 | 0,000 |  |
| Lac | 7,99 | 1,03 | (5,85; 10,12) | 7,76 | 0,000 | 1,08 |
| HPMC\_Visc | -2,126 | 0,866 | (-3,921; -0,330) | -2,46 | 0,022 | 1,00 |
| HPMC\_HP | 2,155 | 0,984 | (0,114; 4,196) | 2,19 | 0,039 | 1,00 |
| Lac\*HPMC\_Visc | -4,44 | 2,01 | (-8,61; -0,26) | -2,20 | 0,038 | 1,08 |

## Model Summary

| S | R-sq | R-sq(adj) | PRESS | R-sq(pred) | AICc | BIC |
| --- | --- | --- | --- | --- | --- | --- |
| 2,42980 | 80,39% | 76,83% | 217,020 | 67,24% | 135,23 | 138,81 |

## Analysis of Variance

| Source | DF | Seq SS | Contribution | Adj SS | Adj MS | F-Value | P-Value |
| --- | --- | --- | --- | --- | --- | --- | --- |
| Model | 4 | 532,52 | 80,39% | 532,52 | 133,130 | 22,55 | 0,000 |
| Linear | 3 | 503,83 | 76,06% | 416,71 | 138,902 | 23,53 | 0,000 |
| Lac | 1 | 442,64 | 66,82% | 355,52 | 355,518 | 60,22 | 0,000 |
| HPMC\_Visc | 1 | 32,89 | 4,97% | 35,59 | 35,591 | 6,03 | 0,022 |
| HPMC\_HP | 1 | 28,30 | 4,27% | 28,30 | 28,297 | 4,79 | 0,039 |
| 2-Way Interaction | 1 | 28,69 | 4,33% | 28,69 | 28,693 | 4,86 | 0,038 |
| Lac\*HPMC\_Visc | 1 | 28,69 | 4,33% | 28,69 | 28,693 | 4,86 | 0,038 |
| Error | 22 | 129,89 | 19,61% | 129,89 | 5,904 |  |  |
| Lack-of-Fit | 20 | 111,35 | 16,81% | 111,35 | 5,567 | 0,60 | 0,786 |
| Pure Error | 2 | 18,54 | 2,80% | 18,54 | 9,268 |  |  |
| Total | 26 | 662,41 | 100,00% |  |  |  |  |

## Regression Equation in Uncoded Units

|  |  |  |
| --- | --- | --- |
| F\_mean\_7h(420min) | = | -2,5 + 93,6 Lac + 0,00174 HPMC\_Visc + 2,123 HPMC\_HP - 0,00456 Lac\*HPMC\_Visc |

## Fits and Diagnostics for All Observations

| Obs | F\_mean\_7h(420min) | Fit | SE Fit | 95% CI | Resid | Std Resid | Del Resid |
| --- | --- | --- | --- | --- | --- | --- | --- |
| 1 | 52,672 | 51,423 | 1,169 | (48,998; 53,847) | 1,249 | 0,59 | 0,58 |
| 2 | 62,103 | 62,568 | 1,169 | (60,143; 64,992) | -0,465 | -0,22 | -0,21 |
| 3 | 47,717 | 51,487 | 1,440 | (48,501; 54,473) | -3,770 | -1,93 | -2,06 |
| 4 | 54,584 | 56,413 | 1,440 | (53,427; 59,399) | -1,828 | -0,93 | -0,93 |
| 5 | 50,142 | 53,424 | 1,027 | (51,294; 55,554) | -3,282 | -1,49 | -1,54 |
| 6 | 62,567 | 64,291 | 1,027 | (62,161; 66,421) | -1,724 | -0,78 | -0,78 |
| 7 | 51,527 | 54,131 | 1,427 | (51,172; 57,090) | -2,605 | -1,32 | -1,35 |
| 8 | 56,493 | 58,964 | 1,427 | (56,005; 61,923) | -2,472 | -1,26 | -1,27 |
| 9 | 51,995 | 51,512 | 1,133 | (49,162; 53,863) | 0,482 | 0,22 | 0,22 |
| 10 | 62,010 | 62,570 | 1,133 | (60,219; 64,921) | -0,560 | -0,26 | -0,25 |
| 11 | 54,521 | 52,035 | 0,880 | (50,209; 53,860) | 2,486 | 1,10 | 1,10 |
| 12 | 60,678 | 59,174 | 0,880 | (57,348; 60,999) | 1,504 | 0,66 | 0,66 |
| 13 | 52,284 | 53,857 | 1,156 | (51,461; 56,254) | -1,574 | -0,74 | -0,73 |
| 14 | 64,731 | 64,953 | 1,156 | (62,557; 67,350) | -0,223 | -0,10 | -0,10 |
| 15 | 54,733 | 53,440 | 0,921 | (51,530; 55,351) | 1,292 | 0,57 | 0,57 |
| 16 | 58,353 | 60,173 | 0,921 | (58,263; 62,084) | -1,820 | -0,81 | -0,80 |
| 17 | 48,463 | 48,499 | 1,105 | (46,207; 50,791) | -0,036 | -0,02 | -0,02 |
| 18 | 67,034 | 66,136 | 1,105 | (63,843; 68,428) | 0,898 | 0,41 | 0,41 |
| 19 | 59,914 | 59,002 | 0,890 | (57,156; 60,847) | 0,912 | 0,40 | 0,40 |
| 20 | 59,528 | 54,538 | 1,087 | (52,283; 56,793) | 4,990 | 2,30 | 2,57 |
| 21 | 55,960 | 55,264 | 0,975 | (53,242; 57,287) | 0,696 | 0,31 | 0,31 |
| 22 | 63,831 | 59,436 | 1,206 | (56,935; 61,936) | 4,395 | 2,08 | 2,27 |
| 23 | 57,984 | 57,428 | 0,484 | (56,423; 58,433) | 0,556 | 0,23 | 0,23 |
| 24 | 58,694 | 57,238 | 0,484 | (56,233; 58,242) | 1,457 | 0,61 | 0,60 |
| 25 | 54,892 | 57,317 | 0,476 | (56,329; 58,305) | -2,425 | -1,02 | -1,02 |
| 26 | 55,902 | 57,317 | 0,476 | (56,329; 58,305) | -1,415 | -0,59 | -0,58 |
| 27 | 60,597 | 57,317 | 0,476 | (56,329; 58,305) | 3,280 | 1,38 | 1,41 |

| Obs | HI | Cook’s D | DFITS |  |
| --- | --- | --- | --- | --- |
| 1 | 0,231482 | 0,02 | 0,31695 |  |
| 2 | 0,231482 | 0,00 | -0,11710 |  |
| 3 | 0,351061 | 0,40 | -1,51814 |  |
| 4 | 0,351061 | 0,09 | -0,68500 |  |
| 5 | 0,178686 | 0,10 | -0,71632 |  |
| 6 | 0,178686 | 0,03 | -0,36181 |  |
| 7 | 0,344801 | 0,18 | -0,97841 |  |
| 8 | 0,344801 | 0,17 | -0,92445 |  |
| 9 | 0,217619 | 0,00 | 0,11575 |  |
| 10 | 0,217619 | 0,00 | -0,13438 |  |
| 11 | 0,131235 | 0,04 | 0,42879 |  |
| 12 | 0,131235 | 0,01 | 0,25469 |  |
| 13 | 0,226191 | 0,03 | -0,39375 |  |
| 14 | 0,226191 | 0,00 | -0,05507 |  |
| 15 | 0,143742 | 0,01 | 0,23181 |  |
| 16 | 0,143742 | 0,02 | -0,32902 |  |
| 17 | 0,206936 | 0,00 | -0,00823 |  |
| 18 | 0,206936 | 0,01 | 0,20789 |  |
| 19 | 0,134128 | 0,01 | 0,15574 |  |
| 20 | 0,200265 | 0,26 | 1,28770 | R |
| 21 | 0,161088 | 0,00 | 0,13416 |  |
| 22 | 0,246222 | 0,28 | 1,29863 | R |
| 23 | 0,039752 | 0,00 | 0,04647 |  |
| 24 | 0,039724 | 0,00 | 0,12261 |  |
| 25 | 0,038439 | 0,01 | -0,20369 |  |
| 26 | 0,038439 | 0,00 | -0,11696 |  |
| 27 | 0,038439 | 0,02 | 0,28128 |  |

R  Large residual

## Stepwise Selection of Terms

α to enter = 0,15; α to remove = 0,15

## Coded Coefficients

| Term | Coef | SE Coef | 95% CI | T-Value | P-Value | VIF |
| --- | --- | --- | --- | --- | --- | --- |
| Constant | 61,395 | 0,498 | (60,362; 62,427) | 123,31 | 0,000 |  |
| Lac | 8,33 | 1,03 | (6,19; 10,47) | 8,07 | 0,000 | 1,08 |
| HPMC\_Visc | -2,266 | 0,868 | (-4,066; -0,465) | -2,61 | 0,016 | 1,00 |
| HPMC\_HP | 2,336 | 0,987 | (0,289; 4,383) | 2,37 | 0,027 | 1,00 |
| Lac\*HPMC\_Visc | -4,45 | 2,02 | (-8,64; -0,27) | -2,21 | 0,038 | 1,08 |

## Model Summary

| S | R-sq | R-sq(adj) | PRESS | R-sq(pred) | AICc | BIC |
| --- | --- | --- | --- | --- | --- | --- |
| 2,43692 | 81,57% | 78,22% | 218,744 | 69,15% | 135,39 | 138,97 |

## Analysis of Variance

| Source | DF | Seq SS | Contribution | Adj SS | Adj MS | F-Value | P-Value |
| --- | --- | --- | --- | --- | --- | --- | --- |
| Model | 4 | 578,38 | 81,57% | 578,38 | 144,596 | 24,35 | 0,000 |
| Linear | 3 | 549,47 | 77,50% | 457,25 | 152,418 | 25,67 | 0,000 |
| Lac | 1 | 478,90 | 67,54% | 386,68 | 386,681 | 65,11 | 0,000 |
| HPMC\_Visc | 1 | 37,31 | 5,26% | 40,43 | 40,425 | 6,81 | 0,016 |
| HPMC\_HP | 1 | 33,26 | 4,69% | 33,26 | 33,265 | 5,60 | 0,027 |
| 2-Way Interaction | 1 | 28,91 | 4,08% | 28,91 | 28,910 | 4,87 | 0,038 |
| Lac\*HPMC\_Visc | 1 | 28,91 | 4,08% | 28,91 | 28,910 | 4,87 | 0,038 |
| Error | 22 | 130,65 | 18,43% | 130,65 | 5,939 |  |  |
| Lack-of-Fit | 20 | 113,43 | 16,00% | 113,43 | 5,672 | 0,66 | 0,757 |
| Pure Error | 2 | 17,22 | 2,43% | 17,22 | 8,609 |  |  |
| Total | 26 | 709,03 | 100,00% |  |  |  |  |

## Regression Equation in Uncoded Units

|  |  |  |
| --- | --- | --- |
| F\_mean\_8h(480min) | = | -0,1 + 95,2 Lac + 0,00171 HPMC\_Visc + 2,302 HPMC\_HP - 0,00458 Lac\*HPMC\_Visc |

## Fits and Diagnostics for All Observations

| Obs | F\_mean\_8h(480min) | Fit | SE Fit | 95% CI | Resid | Std Resid | Del Resid |
| --- | --- | --- | --- | --- | --- | --- | --- |
| 1 | 56,975 | 55,573 | 1,172 | (53,142; 58,005) | 1,402 | 0,66 | 0,65 |
| 2 | 66,566 | 67,073 | 1,172 | (64,641; 69,504) | -0,507 | -0,24 | -0,23 |
| 3 | 51,645 | 55,448 | 1,444 | (52,453; 58,442) | -3,803 | -1,94 | -2,08 |
| 4 | 58,928 | 60,705 | 1,444 | (57,710; 63,699) | -1,776 | -0,90 | -0,90 |
| 5 | 54,344 | 57,735 | 1,030 | (55,598; 59,871) | -3,390 | -1,54 | -1,59 |
| 6 | 67,113 | 68,955 | 1,030 | (66,819; 71,092) | -1,842 | -0,83 | -0,83 |
| 7 | 55,606 | 58,312 | 1,431 | (55,344; 61,280) | -2,706 | -1,37 | -1,40 |
| 8 | 61,034 | 63,476 | 1,431 | (60,508; 66,443) | -2,442 | -1,24 | -1,25 |
| 9 | 55,842 | 55,668 | 1,137 | (53,310; 58,025) | 0,175 | 0,08 | 0,08 |
| 10 | 66,542 | 67,080 | 1,137 | (64,722; 69,437) | -0,538 | -0,25 | -0,24 |
| 11 | 58,958 | 56,112 | 0,883 | (54,281; 57,942) | 2,846 | 1,25 | 1,27 |
| 12 | 64,883 | 63,590 | 0,883 | (61,759; 65,421) | 1,293 | 0,57 | 0,56 |
| 13 | 56,552 | 58,211 | 1,159 | (55,808; 60,615) | -1,659 | -0,77 | -0,77 |
| 14 | 69,331 | 69,662 | 1,159 | (67,258; 72,066) | -0,330 | -0,15 | -0,15 |
| 15 | 58,879 | 57,623 | 0,924 | (55,706; 59,539) | 1,256 | 0,56 | 0,55 |
| 16 | 62,992 | 64,693 | 0,924 | (62,777; 66,609) | -1,702 | -0,75 | -0,75 |
| 17 | 52,523 | 52,529 | 1,109 | (50,230; 54,828) | -0,006 | -0,00 | -0,00 |
| 18 | 71,833 | 70,857 | 1,109 | (68,558; 73,156) | 0,976 | 0,45 | 0,44 |
| 19 | 64,575 | 63,488 | 0,892 | (61,637; 65,338) | 1,088 | 0,48 | 0,47 |
| 20 | 63,570 | 58,726 | 1,091 | (56,465; 60,988) | 4,844 | 2,22 | 2,47 |
| 21 | 60,016 | 59,467 | 0,978 | (57,439; 61,496) | 0,549 | 0,25 | 0,24 |
| 22 | 68,378 | 63,993 | 1,209 | (61,485; 66,501) | 4,385 | 2,07 | 2,26 |
| 23 | 62,678 | 61,810 | 0,486 | (60,803; 62,818) | 0,868 | 0,36 | 0,36 |
| 24 | 63,034 | 61,603 | 0,486 | (60,596; 62,611) | 1,431 | 0,60 | 0,59 |
| 25 | 59,286 | 61,693 | 0,478 | (60,702; 62,684) | -2,407 | -1,01 | -1,01 |
| 26 | 60,514 | 61,693 | 0,478 | (60,702; 62,684) | -1,179 | -0,49 | -0,48 |
| 27 | 64,869 | 61,693 | 0,478 | (60,702; 62,684) | 3,176 | 1,33 | 1,35 |

| Obs | HI | Cook’s D | DFITS |  |
| --- | --- | --- | --- | --- |
| 1 | 0,231482 | 0,03 | 0,35527 |  |
| 2 | 0,231482 | 0,00 | -0,12729 |  |
| 3 | 0,351061 | 0,41 | -1,52844 |  |
| 4 | 0,351061 | 0,09 | -0,66272 |  |
| 5 | 0,178686 | 0,10 | -0,74034 |  |
| 6 | 0,178686 | 0,03 | -0,38627 |  |
| 7 | 0,344801 | 0,20 | -1,01689 |  |
| 8 | 0,344801 | 0,16 | -0,90969 |  |
| 9 | 0,217619 | 0,00 | 0,04173 |  |
| 10 | 0,217619 | 0,00 | -0,12881 |  |
| 11 | 0,131235 | 0,05 | 0,49382 |  |
| 12 | 0,131235 | 0,01 | 0,21774 |  |
| 13 | 0,226191 | 0,04 | -0,41457 |  |
| 14 | 0,226191 | 0,00 | -0,08148 |  |
| 15 | 0,143742 | 0,01 | 0,22457 |  |
| 16 | 0,143742 | 0,02 | -0,30606 |  |
| 17 | 0,206936 | 0,00 | -0,00139 |  |
| 18 | 0,206936 | 0,01 | 0,22552 |  |
| 19 | 0,134128 | 0,01 | 0,18541 |  |
| 20 | 0,200265 | 0,25 | 1,23407 | R |
| 21 | 0,161088 | 0,00 | 0,10543 |  |
| 22 | 0,246222 | 0,28 | 1,29017 | R |
| 23 | 0,039752 | 0,00 | 0,07249 |  |
| 24 | 0,039724 | 0,00 | 0,12003 |  |
| 25 | 0,038439 | 0,01 | -0,20148 |  |
| 26 | 0,038439 | 0,00 | -0,09694 |  |
| 27 | 0,038439 | 0,01 | 0,27075 |  |

R  Large residual

## Stepwise Selection of Terms

α to enter = 0,15; α to remove = 0,15

## Coded Coefficients

| Term | Coef | SE Coef | 95% CI | T-Value | P-Value | VIF |
| --- | --- | --- | --- | --- | --- | --- |
| Constant | 65,533 | 0,507 | (64,481; 66,584) | 129,28 | 0,000 |  |
| Lac | 8,67 | 1,05 | (6,49; 10,85) | 8,25 | 0,000 | 1,08 |
| HPMC\_Visc | -2,394 | 0,884 | (-4,227; -0,560) | -2,71 | 0,013 | 1,00 |
| HPMC\_HP | 2,44 | 1,00 | (0,35; 4,52) | 2,43 | 0,024 | 1,00 |
| Lac\*HPMC\_Visc | -4,33 | 2,06 | (-8,60; -0,07) | -2,11 | 0,047 | 1,08 |

## Model Summary

| S | R-sq | R-sq(adj) | PRESS | R-sq(pred) | AICc | BIC |
| --- | --- | --- | --- | --- | --- | --- |
| 2,48110 | 82,07% | 78,81% | 225,600 | 70,13% | 136,36 | 139,94 |

## Analysis of Variance

| Source | DF | Seq SS | Contribution | Adj SS | Adj MS | F-Value | P-Value |
| --- | --- | --- | --- | --- | --- | --- | --- |
| Model | 4 | 619,75 | 82,07% | 619,75 | 154,938 | 25,17 | 0,000 |
| Linear | 3 | 592,41 | 78,45% | 497,07 | 165,692 | 26,92 | 0,000 |
| Lac | 1 | 514,46 | 68,12% | 419,13 | 419,127 | 68,09 | 0,000 |
| HPMC\_Visc | 1 | 41,70 | 5,52% | 45,14 | 45,137 | 7,33 | 0,013 |
| HPMC\_HP | 1 | 36,25 | 4,80% | 36,25 | 36,251 | 5,89 | 0,024 |
| 2-Way Interaction | 1 | 27,34 | 3,62% | 27,34 | 27,345 | 4,44 | 0,047 |
| Lac\*HPMC\_Visc | 1 | 27,34 | 3,62% | 27,34 | 27,345 | 4,44 | 0,047 |
| Error | 22 | 135,43 | 17,93% | 135,43 | 6,156 |  |  |
| Lack-of-Fit | 20 | 118,47 | 15,69% | 118,47 | 5,924 | 0,70 | 0,738 |
| Pure Error | 2 | 16,96 | 2,25% | 16,96 | 8,479 |  |  |
| Total | 26 | 755,18 | 100,00% |  |  |  |  |

## Regression Equation in Uncoded Units

|  |  |  |
| --- | --- | --- |
| F\_mean\_9h(540min) | = | 3,6 + 94,9 Lac + 0,00161 HPMC\_Visc + 2,403 HPMC\_HP - 0,00445 Lac\*HPMC\_Visc |

## Fits and Diagnostics for All Observations

| Obs | F\_mean\_9h(540min) | Fit | SE Fit | 95% CI | Resid | Std Resid | Del Resid |
| --- | --- | --- | --- | --- | --- | --- | --- |
| 1 | 61,817 | 59,601 | 1,194 | (57,126; 62,077) | 2,216 | 1,02 | 1,02 |
| 2 | 70,693 | 71,356 | 1,194 | (68,880; 73,832) | -0,663 | -0,30 | -0,30 |
| 3 | 55,315 | 59,207 | 1,470 | (56,158; 62,256) | -3,892 | -1,95 | -2,09 |
| 4 | 63,033 | 64,891 | 1,470 | (61,842; 67,939) | -1,857 | -0,93 | -0,93 |
| 5 | 58,314 | 61,846 | 1,049 | (59,671; 64,021) | -3,532 | -1,57 | -1,63 |
| 6 | 71,253 | 73,329 | 1,049 | (71,154; 75,504) | -2,076 | -0,92 | -0,92 |
| 7 | 59,803 | 62,193 | 1,457 | (59,171; 65,214) | -2,390 | -1,19 | -1,20 |
| 8 | 65,380 | 67,786 | 1,457 | (64,764; 70,807) | -2,406 | -1,20 | -1,21 |
| 9 | 59,432 | 59,696 | 1,157 | (57,296; 62,097) | -0,265 | -0,12 | -0,12 |
| 10 | 70,737 | 71,366 | 1,157 | (68,965; 73,766) | -0,628 | -0,29 | -0,28 |
| 11 | 63,159 | 59,993 | 0,899 | (58,129; 61,857) | 3,165 | 1,37 | 1,40 |
| 12 | 68,853 | 67,838 | 0,899 | (65,974; 69,702) | 1,015 | 0,44 | 0,43 |
| 13 | 60,614 | 62,353 | 1,180 | (59,906; 64,800) | -1,739 | -0,80 | -0,79 |
| 14 | 73,593 | 74,060 | 1,180 | (71,613; 76,507) | -0,467 | -0,21 | -0,21 |
| 15 | 62,639 | 61,554 | 0,941 | (59,603; 63,504) | 1,085 | 0,47 | 0,46 |
| 16 | 67,266 | 69,001 | 0,941 | (67,050; 70,952) | -1,735 | -0,76 | -0,75 |
| 17 | 56,364 | 56,366 | 1,129 | (54,026; 58,707) | -0,002 | -0,00 | -0,00 |
| 18 | 77,064 | 75,333 | 1,129 | (72,993; 77,674) | 1,731 | 0,78 | 0,78 |
| 19 | 68,896 | 67,746 | 0,909 | (65,862; 69,631) | 1,150 | 0,50 | 0,49 |
| 20 | 67,371 | 62,718 | 1,110 | (60,416; 65,021) | 4,653 | 2,10 | 2,29 |
| 21 | 63,909 | 63,526 | 0,996 | (61,461; 65,591) | 0,383 | 0,17 | 0,16 |
| 22 | 72,513 | 68,248 | 1,231 | (65,695; 70,802) | 4,264 | 1,98 | 2,13 |
| 23 | 67,108 | 65,974 | 0,495 | (64,948; 67,000) | 1,133 | 0,47 | 0,46 |
| 24 | 67,042 | 65,758 | 0,495 | (64,733; 66,784) | 1,283 | 0,53 | 0,52 |
| 25 | 63,344 | 65,850 | 0,486 | (64,841; 66,859) | -2,506 | -1,03 | -1,03 |
| 26 | 64,819 | 65,850 | 0,486 | (64,841; 66,859) | -1,031 | -0,42 | -0,42 |
| 27 | 68,961 | 65,850 | 0,486 | (64,841; 66,859) | 3,111 | 1,28 | 1,30 |

| Obs | HI | Cook’s D | DFITS |  |
| --- | --- | --- | --- | --- |
| 1 | 0,231482 | 0,06 | 0,55953 |  |
| 2 | 0,231482 | 0,01 | -0,16387 |  |
| 3 | 0,351061 | 0,41 | -1,53822 |  |
| 4 | 0,351061 | 0,09 | -0,68124 |  |
| 5 | 0,178686 | 0,11 | -0,75966 |  |
| 6 | 0,178686 | 0,04 | -0,42922 |  |
| 7 | 0,344801 | 0,15 | -0,87204 |  |
| 8 | 0,344801 | 0,15 | -0,87824 |  |
| 9 | 0,217619 | 0,00 | -0,06215 |  |
| 10 | 0,217619 | 0,00 | -0,14784 |  |
| 11 | 0,131235 | 0,06 | 0,54338 |  |
| 12 | 0,131235 | 0,01 | 0,16747 |  |
| 13 | 0,226191 | 0,04 | -0,42720 |  |
| 14 | 0,226191 | 0,00 | -0,11318 |  |
| 15 | 0,143742 | 0,01 | 0,19015 |  |
| 16 | 0,143742 | 0,02 | -0,30649 |  |
| 17 | 0,206936 | 0,00 | -0,00044 |  |
| 18 | 0,206936 | 0,03 | 0,39651 |  |
| 19 | 0,134128 | 0,01 | 0,19258 |  |
| 20 | 0,200265 | 0,22 | 1,14625 | R |
| 21 | 0,161088 | 0,00 | 0,07217 |  |
| 22 | 0,246222 | 0,26 | 1,21935 |  |
| 23 | 0,039752 | 0,00 | 0,09313 |  |
| 24 | 0,039724 | 0,00 | 0,10557 |  |
| 25 | 0,038439 | 0,01 | -0,20621 |  |
| 26 | 0,038439 | 0,00 | -0,08308 |  |
| 27 | 0,038439 | 0,01 | 0,25960 |  |

R  Large residual

## Stepwise Selection of Terms

α to enter = 0,15; α to remove = 0,15

## Coded Coefficients

| Term | Coef | SE Coef | 95% CI | T-Value | P-Value | VIF |
| --- | --- | --- | --- | --- | --- | --- |
| Constant | 69,486 | 0,538 | (68,371; 70,602) | 129,16 | 0,000 |  |
| Lac | 9,37 | 1,12 | (7,06; 11,69) | 8,41 | 0,000 | 1,08 |
| HPMC\_Visc | -2,462 | 0,938 | (-4,408; -0,517) | -2,62 | 0,015 | 1,00 |
| HPMC\_HP | 2,56 | 1,07 | (0,34; 4,77) | 2,40 | 0,026 | 1,00 |
| Lac\*HPMC\_Visc | -4,42 | 2,18 | (-8,94; 0,11) | -2,03 | 0,055 | 1,08 |

## Model Summary

| S | R-sq | R-sq(adj) | PRESS | R-sq(pred) | AICc | BIC |
| --- | --- | --- | --- | --- | --- | --- |
| 2,63306 | 82,30% | 79,08% | 252,543 | 70,70% | 139,57 | 143,15 |

## Analysis of Variance

| Source | DF | Seq SS | Contribution | Adj SS | Adj MS | F-Value | P-Value |
| --- | --- | --- | --- | --- | --- | --- | --- |
| Model | 4 | 709,30 | 82,30% | 709,30 | 177,326 | 25,58 | 0,000 |
| Linear | 3 | 680,87 | 79,00% | 573,78 | 191,259 | 27,59 | 0,000 |
| Lac | 1 | 597,03 | 69,27% | 489,94 | 489,938 | 70,67 | 0,000 |
| HPMC\_Visc | 1 | 44,05 | 5,11% | 47,76 | 47,758 | 6,89 | 0,015 |
| HPMC\_HP | 1 | 39,79 | 4,62% | 39,79 | 39,790 | 5,74 | 0,026 |
| 2-Way Interaction | 1 | 28,43 | 3,30% | 28,43 | 28,431 | 4,10 | 0,055 |
| Lac\*HPMC\_Visc | 1 | 28,43 | 3,30% | 28,43 | 28,431 | 4,10 | 0,055 |
| Error | 22 | 152,53 | 17,70% | 152,53 | 6,933 |  |  |
| Lack-of-Fit | 20 | 136,22 | 15,81% | 136,22 | 6,811 | 0,84 | 0,677 |
| Pure Error | 2 | 16,30 | 1,89% | 16,30 | 8,151 |  |  |
| Total | 26 | 861,83 | 100,00% |  |  |  |  |

## Regression Equation in Uncoded Units

|  |  |  |
| --- | --- | --- |
| F\_mean\_10h(600min) | = | 4,8 + 98,9 Lac + 0,00164 HPMC\_Visc + 2,52 HPMC\_HP - 0,00454 Lac\*HPMC\_Visc |

## Fits and Diagnostics for All Observations

| Obs | F\_mean\_10h(600min) | Fit | SE Fit | 95% CI | Resid | Std Resid | Del Resid | HI |
| --- | --- | --- | --- | --- | --- | --- | --- | --- |
| 1 | 65,67 | 63,14 | 1,27 | (60,51; 65,76) | 2,53 | 1,10 | 1,10 | 0,231482 |
| 2 | 74,63 | 75,66 | 1,27 | (73,03; 78,28) | -1,03 | -0,44 | -0,44 | 0,231482 |
| 3 | 58,83 | 62,70 | 1,56 | (59,47; 65,94) | -3,88 | -1,83 | -1,94 | 0,351061 |
| 4 | 66,93 | 69,03 | 1,56 | (65,80; 72,27) | -2,11 | -0,99 | -0,99 | 0,351061 |
| 5 | 62,05 | 65,49 | 1,11 | (63,18; 67,80) | -3,44 | -1,44 | -1,48 | 0,178686 |
| 6 | 75,06 | 77,73 | 1,11 | (75,42; 80,04) | -2,67 | -1,12 | -1,13 | 0,178686 |
| 7 | 63,63 | 65,83 | 1,55 | (62,62; 69,04) | -2,21 | -1,03 | -1,04 | 0,344801 |
| 8 | 69,51 | 72,07 | 1,55 | (68,86; 75,27) | -2,56 | -1,20 | -1,21 | 0,344801 |
| 9 | 62,76 | 63,24 | 1,23 | (60,69; 65,78) | -0,47 | -0,20 | -0,20 | 0,217619 |
| 10 | 74,79 | 75,67 | 1,23 | (73,12; 78,22) | -0,87 | -0,38 | -0,37 | 0,217619 |
| 11 | 67,12 | 63,53 | 0,95 | (61,56; 65,51) | 3,59 | 1,46 | 1,50 | 0,131235 |
| 12 | 72,64 | 72,07 | 0,95 | (70,09; 74,04) | 0,57 | 0,23 | 0,23 | 0,131235 |
| 13 | 64,44 | 66,02 | 1,25 | (63,42; 68,62) | -1,58 | -0,68 | -0,67 | 0,226191 |
| 14 | 77,49 | 78,49 | 1,25 | (75,89; 81,09) | -1,00 | -0,43 | -0,42 | 0,226191 |
| 15 | 66,38 | 65,17 | 1,00 | (63,10; 67,24) | 1,22 | 0,50 | 0,49 | 0,143742 |
| 16 | 71,30 | 73,30 | 1,00 | (71,23; 75,37) | -1,99 | -0,82 | -0,81 | 0,143742 |
| 17 | 60,11 | 59,61 | 1,20 | (57,12; 62,09) | 0,51 | 0,22 | 0,21 | 0,206936 |
| 18 | 84,23 | 80,01 | 1,20 | (77,53; 82,50) | 4,21 | 1,80 | 1,90 | 0,206936 |
| 19 | 72,93 | 71,76 | 0,96 | (69,76; 73,76) | 1,17 | 0,48 | 0,47 | 0,134128 |
| 20 | 70,91 | 66,58 | 1,18 | (64,14; 69,03) | 4,32 | 1,84 | 1,95 | 0,200265 |
| 21 | 67,57 | 67,38 | 1,06 | (65,18; 69,57) | 0,19 | 0,08 | 0,08 | 0,161088 |
| 22 | 76,19 | 72,33 | 1,31 | (69,62; 75,04) | 3,86 | 1,69 | 1,77 | 0,246222 |
| 23 | 71,17 | 69,94 | 0,52 | (68,85; 71,03) | 1,24 | 0,48 | 0,47 | 0,039752 |
| 24 | 70,77 | 69,71 | 0,52 | (68,62; 70,80) | 1,06 | 0,41 | 0,40 | 0,039724 |
| 25 | 67,14 | 69,81 | 0,52 | (68,74; 70,88) | -2,67 | -1,03 | -1,03 | 0,038439 |
| 26 | 68,89 | 69,81 | 0,52 | (68,74; 70,88) | -0,92 | -0,35 | -0,35 | 0,038439 |
| 27 | 72,73 | 69,81 | 0,52 | (68,74; 70,88) | 2,92 | 1,13 | 1,14 | 0,038439 |

| Obs | Cook’s D | DFITS |
| --- | --- | --- |
| 1 | 0,07 | 0,60389 |
| 2 | 0,01 | -0,23920 |
| 3 | 0,36 | -1,42666 |
| 4 | 0,11 | -0,73019 |
| 5 | 0,09 | -0,68957 |
| 6 | 0,05 | -0,52477 |
| 7 | 0,11 | -0,75208 |
| 8 | 0,15 | -0,88065 |
| 9 | 0,00 | -0,10480 |
| 10 | 0,01 | -0,19402 |
| 11 | 0,06 | 0,58458 |
| 12 | 0,00 | 0,08857 |
| 13 | 0,03 | -0,36317 |
| 14 | 0,01 | -0,22866 |
| 15 | 0,01 | 0,20086 |
| 16 | 0,02 | -0,33265 |
| 17 | 0,00 | 0,10761 |
| 18 | 0,17 | 0,97110 |
| 19 | 0,01 | 0,18443 |
| 20 | 0,17 | 0,97543 |
| 21 | 0,00 | 0,03376 |
| 22 | 0,19 | 1,01150 |
| 23 | 0,00 | 0,09572 |
| 24 | 0,00 | 0,08178 |
| 25 | 0,01 | -0,20684 |
| 26 | 0,00 | -0,06952 |
| 27 | 0,01 | 0,22724 |

## Stepwise Selection of Terms

α to enter = 0,15; α to remove = 0,15

## Coded Coefficients

| Term | Coef | SE Coef | 95% CI | T-Value | P-Value | VIF |
| --- | --- | --- | --- | --- | --- | --- |
| Constant | 73,136 | 0,535 | (72,027; 74,245) | 136,74 | 0,000 |  |
| Lac | 9,65 | 1,11 | (7,35; 11,95) | 8,70 | 0,000 | 1,08 |
| HPMC\_Visc | -2,517 | 0,933 | (-4,452; -0,583) | -2,70 | 0,013 | 1,00 |
| HPMC\_HP | 2,72 | 1,06 | (0,52; 4,91) | 2,56 | 0,018 | 1,00 |
| Lac\*HPMC\_Visc | -4,72 | 2,17 | (-9,22; -0,23) | -2,18 | 0,040 | 1,08 |

## Model Summary

| S | R-sq | R-sq(adj) | PRESS | R-sq(pred) | AICc | BIC |
| --- | --- | --- | --- | --- | --- | --- |
| 2,61784 | 83,42% | 80,40% | 247,731 | 72,75% | 139,26 | 142,84 |

## Analysis of Variance

| Source | DF | Seq SS | Contribution | Adj SS | Adj MS | F-Value | P-Value |
| --- | --- | --- | --- | --- | --- | --- | --- |
| Model | 4 | 758,46 | 83,42% | 758,46 | 189,614 | 27,67 | 0,000 |
| Linear | 3 | 725,95 | 79,84% | 609,58 | 203,192 | 29,65 | 0,000 |
| Lac | 1 | 635,13 | 69,85% | 518,76 | 518,756 | 75,70 | 0,000 |
| HPMC\_Visc | 1 | 45,89 | 5,05% | 49,92 | 49,915 | 7,28 | 0,013 |
| HPMC\_HP | 1 | 44,93 | 4,94% | 44,93 | 44,933 | 6,56 | 0,018 |
| 2-Way Interaction | 1 | 32,51 | 3,58% | 32,51 | 32,505 | 4,74 | 0,040 |
| Lac\*HPMC\_Visc | 1 | 32,51 | 3,58% | 32,51 | 32,505 | 4,74 | 0,040 |
| Error | 22 | 150,77 | 16,58% | 150,77 | 6,853 |  |  |
| Lack-of-Fit | 20 | 135,54 | 14,91% | 135,54 | 6,777 | 0,89 | 0,655 |
| Pure Error | 2 | 15,23 | 1,68% | 15,23 | 7,617 |  |  |
| Total | 26 | 909,23 | 100,00% |  |  |  |  |

## Regression Equation in Uncoded Units

|  |  |  |
| --- | --- | --- |
| F\_mean\_11h(660min) | = | 4,4 + 104,2 Lac + 0,00178 HPMC\_Visc + 2,68 HPMC\_HP - 0,00486 Lac\*HPMC\_Visc |

## Fits and Diagnostics for All Observations

| Obs | F\_mean\_11h(660min) | Fit | SE Fit | 95% CI | Resid | Std Resid | Del Resid | HI |
| --- | --- | --- | --- | --- | --- | --- | --- | --- |
| 1 | 68,86 | 66,47 | 1,26 | (63,85; 69,08) | 2,39 | 1,04 | 1,04 | 0,231482 |
| 2 | 78,48 | 79,47 | 1,26 | (76,86; 82,09) | -0,99 | -0,43 | -0,42 | 0,231482 |
| 3 | 62,20 | 66,16 | 1,55 | (62,95; 69,38) | -3,96 | -1,88 | -2,00 | 0,351061 |
| 4 | 70,58 | 72,55 | 1,55 | (69,34; 75,77) | -1,97 | -0,94 | -0,93 | 0,351061 |
| 5 | 65,58 | 68,97 | 1,11 | (66,68; 71,27) | -3,39 | -1,43 | -1,46 | 0,178686 |
| 6 | 78,49 | 81,68 | 1,11 | (79,39; 83,98) | -3,19 | -1,35 | -1,37 | 0,178686 |
| 7 | 67,32 | 69,49 | 1,54 | (66,30; 72,68) | -2,17 | -1,02 | -1,02 | 0,344801 |
| 8 | 73,44 | 75,78 | 1,54 | (72,59; 78,97) | -2,34 | -1,10 | -1,11 | 0,344801 |
| 9 | 65,94 | 66,57 | 1,22 | (64,04; 69,11) | -0,64 | -0,27 | -0,27 | 0,217619 |
| 10 | 78,64 | 79,49 | 1,22 | (76,96; 82,02) | -0,85 | -0,37 | -0,36 | 0,217619 |
| 11 | 70,91 | 66,99 | 0,95 | (65,02; 68,96) | 3,91 | 1,60 | 1,67 | 0,131235 |
| 12 | 76,19 | 75,74 | 0,95 | (73,77; 77,70) | 0,45 | 0,19 | 0,18 | 0,131235 |
| 13 | 68,14 | 69,53 | 1,25 | (66,95; 72,11) | -1,39 | -0,60 | -0,59 | 0,226191 |
| 14 | 82,05 | 82,49 | 1,25 | (79,91; 85,07) | -0,44 | -0,19 | -0,19 | 0,226191 |
| 15 | 70,11 | 68,74 | 0,99 | (66,68; 70,80) | 1,38 | 0,57 | 0,56 | 0,143742 |
| 16 | 75,05 | 77,05 | 0,99 | (74,99; 79,11) | -2,00 | -0,83 | -0,82 | 0,143742 |
| 17 | 63,67 | 62,93 | 1,19 | (60,46; 65,40) | 0,74 | 0,32 | 0,31 | 0,206936 |
| 18 | 88,47 | 83,99 | 1,19 | (81,52; 86,46) | 4,47 | 1,92 | 2,05 | 0,206936 |
| 19 | 76,75 | 75,45 | 0,96 | (73,46; 77,44) | 1,30 | 0,53 | 0,52 | 0,134128 |
| 20 | 74,25 | 70,15 | 1,17 | (67,72; 72,58) | 4,10 | 1,75 | 1,85 | 0,200265 |
| 21 | 70,99 | 70,87 | 1,05 | (68,70; 73,05) | 0,11 | 0,05 | 0,05 | 0,161088 |
| 22 | 79,48 | 76,14 | 1,30 | (73,45; 78,84) | 3,34 | 1,47 | 1,51 | 0,246222 |
| 23 | 74,80 | 73,59 | 0,52 | (72,51; 74,67) | 1,22 | 0,47 | 0,47 | 0,039752 |
| 24 | 74,21 | 73,35 | 0,52 | (72,27; 74,43) | 0,87 | 0,34 | 0,33 | 0,039724 |
| 25 | 70,68 | 73,46 | 0,51 | (72,40; 74,53) | -2,78 | -1,08 | -1,09 | 0,038439 |
| 26 | 72,61 | 73,46 | 0,51 | (72,40; 74,53) | -0,85 | -0,33 | -0,32 | 0,038439 |
| 27 | 76,13 | 73,46 | 0,51 | (72,40; 74,53) | 2,67 | 1,04 | 1,04 | 0,038439 |

| Obs | Cook’s D | DFITS |
| --- | --- | --- |
| 1 | 0,07 | 0,57297 |
| 2 | 0,01 | -0,23309 |
| 3 | 0,38 | -1,47360 |
| 4 | 0,09 | -0,68582 |
| 5 | 0,09 | -0,68324 |
| 6 | 0,08 | -0,63999 |
| 7 | 0,11 | -0,74308 |
| 8 | 0,13 | -0,80446 |
| 9 | 0,00 | -0,14191 |
| 10 | 0,01 | -0,18917 |
| 11 | 0,08 | 0,64829 |
| 12 | 0,00 | 0,07071 |
| 13 | 0,02 | -0,32063 |
| 14 | 0,00 | -0,10024 |
| 15 | 0,01 | 0,22920 |
| 16 | 0,02 | -0,33564 |
| 17 | 0,01 | 0,15850 |
| 18 | 0,19 | 1,04892 |
| 19 | 0,01 | 0,20577 |
| 20 | 0,15 | 0,92342 |
| 21 | 0,00 | 0,02013 |
| 22 | 0,14 | 0,86433 |
| 23 | 0,00 | 0,09470 |
| 24 | 0,00 | 0,06732 |
| 25 | 0,01 | -0,21722 |
| 26 | 0,00 | -0,06466 |
| 27 | 0,01 | 0,20802 |

## Stepwise Selection of Terms

α to enter = 0,15; α to remove = 0,15

## Coded Coefficients

| Term | Coef | SE Coef | 95% CI | T-Value | P-Value | VIF |
| --- | --- | --- | --- | --- | --- | --- |
| Constant | 76,469 | 0,514 | (75,403; 77,535) | 148,83 | 0,000 |  |
| Lac | 9,68 | 1,07 | (7,47; 11,89) | 9,09 | 0,000 | 1,08 |
| HPMC\_Visc | -2,584 | 0,896 | (-4,442; -0,725) | -2,88 | 0,009 | 1,00 |
| HPMC\_HP | 2,76 | 1,02 | (0,65; 4,87) | 2,71 | 0,013 | 1,00 |
| Lac\*HPMC\_Visc | -4,89 | 2,08 | (-9,22; -0,57) | -2,35 | 0,028 | 1,08 |

## Model Summary

| S | R-sq | R-sq(adj) | PRESS | R-sq(pred) | AICc | BIC |
| --- | --- | --- | --- | --- | --- | --- |
| 2,51476 | 84,72% | 81,95% | 227,738 | 75,00% | 137,09 | 140,67 |

## Analysis of Variance

| Source | DF | Seq SS | Contribution | Adj SS | Adj MS | F-Value | P-Value |
| --- | --- | --- | --- | --- | --- | --- | --- |
| Model | 4 | 771,64 | 84,72% | 771,64 | 192,911 | 30,50 | 0,000 |
| Linear | 3 | 736,74 | 80,89% | 616,96 | 205,653 | 32,52 | 0,000 |
| Lac | 1 | 642,01 | 70,49% | 522,23 | 522,231 | 82,58 | 0,000 |
| HPMC\_Visc | 1 | 48,37 | 5,31% | 52,57 | 52,572 | 8,31 | 0,009 |
| HPMC\_HP | 1 | 46,36 | 5,09% | 46,36 | 46,356 | 7,33 | 0,013 |
| 2-Way Interaction | 1 | 34,91 | 3,83% | 34,91 | 34,905 | 5,52 | 0,028 |
| Lac\*HPMC\_Visc | 1 | 34,91 | 3,83% | 34,91 | 34,905 | 5,52 | 0,028 |
| Error | 22 | 139,13 | 15,28% | 139,13 | 6,324 |  |  |
| Lack-of-Fit | 20 | 124,71 | 13,69% | 124,71 | 6,236 | 0,87 | 0,665 |
| Pure Error | 2 | 14,42 | 1,58% | 14,42 | 7,208 |  |  |
| Total | 26 | 910,77 | 100,00% |  |  |  |  |

## Regression Equation in Uncoded Units

|  |  |  |
| --- | --- | --- |
| F\_mean\_12h(720min) | = | 6,3 + 106,7 Lac + 0,00185 HPMC\_Visc + 2,72 HPMC\_HP - 0,00503 Lac\*HPMC\_Visc |

## Fits and Diagnostics for All Observations

| Obs | F\_mean\_12h(720min) | Fit | SE Fit | 95% CI | Resid | Std Resid | Del Resid |
| --- | --- | --- | --- | --- | --- | --- | --- |
| 1 | 72,058 | 69,739 | 1,210 | (67,229; 72,248) | 2,320 | 1,05 | 1,05 |
| 2 | 81,925 | 82,902 | 1,210 | (80,392; 85,411) | -0,976 | -0,44 | -0,43 |
| 3 | 65,371 | 69,463 | 1,490 | (66,373; 72,553) | -4,091 | -2,02 | -2,19 |
| 4 | 74,075 | 75,766 | 1,490 | (72,676; 78,857) | -1,691 | -0,83 | -0,83 |
| 5 | 68,884 | 72,284 | 1,063 | (70,080; 74,489) | -3,400 | -1,49 | -1,54 |
| 6 | 81,718 | 85,141 | 1,063 | (82,936; 87,345) | -3,423 | -1,50 | -1,55 |
| 7 | 70,725 | 72,842 | 1,477 | (69,779; 75,904) | -2,116 | -1,04 | -1,04 |
| 8 | 76,996 | 79,043 | 1,477 | (75,981; 82,106) | -2,047 | -1,01 | -1,01 |
| 9 | 68,904 | 69,848 | 1,173 | (67,415; 72,281) | -0,944 | -0,42 | -0,42 |
| 10 | 82,575 | 82,915 | 1,173 | (80,482; 85,348) | -0,340 | -0,15 | -0,15 |
| 11 | 74,429 | 70,291 | 0,911 | (68,402; 72,181) | 4,138 | 1,77 | 1,86 |
| 12 | 79,559 | 79,036 | 0,911 | (77,147; 80,926) | 0,523 | 0,22 | 0,22 |
| 13 | 71,590 | 72,851 | 1,196 | (70,371; 75,332) | -1,261 | -0,57 | -0,56 |
| 14 | 86,147 | 85,961 | 1,196 | (83,481; 88,441) | 0,186 | 0,08 | 0,08 |
| 15 | 73,461 | 72,067 | 0,953 | (70,090; 74,044) | 1,395 | 0,60 | 0,59 |
| 16 | 78,588 | 80,364 | 0,953 | (78,386; 82,341) | -1,776 | -0,76 | -0,76 |
| 17 | 67,033 | 66,208 | 1,144 | (63,835; 68,580) | 0,825 | 0,37 | 0,36 |
| 18 | 91,018 | 87,401 | 1,144 | (85,029; 89,774) | 3,617 | 1,61 | 1,68 |
| 19 | 80,233 | 78,849 | 0,921 | (76,939; 80,759) | 1,384 | 0,59 | 0,58 |
| 20 | 77,312 | 73,410 | 1,125 | (71,076; 75,744) | 3,902 | 1,74 | 1,82 |
| 21 | 74,103 | 74,177 | 1,009 | (72,084; 76,271) | -0,074 | -0,03 | -0,03 |
| 22 | 82,470 | 79,526 | 1,248 | (76,938; 82,114) | 2,945 | 1,35 | 1,38 |
| 23 | 78,081 | 76,936 | 0,501 | (75,896; 77,976) | 1,145 | 0,46 | 0,46 |
| 24 | 77,405 | 76,691 | 0,501 | (75,651; 77,730) | 0,714 | 0,29 | 0,28 |
| 25 | 74,015 | 76,804 | 0,493 | (75,782; 77,827) | -2,790 | -1,13 | -1,14 |
| 26 | 76,104 | 76,804 | 0,493 | (75,782; 77,827) | -0,700 | -0,28 | -0,28 |
| 27 | 79,343 | 76,804 | 0,493 | (75,782; 77,827) | 2,539 | 1,03 | 1,03 |

| Obs | HI | Cook’s D | DFITS |  |
| --- | --- | --- | --- | --- |
| 1 | 0,231482 | 0,07 | 0,57893 |  |
| 2 | 0,231482 | 0,01 | -0,23848 |  |
| 3 | 0,351061 | 0,44 | -1,60799 | R |
| 4 | 0,351061 | 0,08 | -0,60963 |  |
| 5 | 0,178686 | 0,10 | -0,71720 |  |
| 6 | 0,178686 | 0,10 | -0,72253 |  |
| 7 | 0,344801 | 0,11 | -0,75566 |  |
| 8 | 0,344801 | 0,11 | -0,72981 |  |
| 9 | 0,217619 | 0,01 | -0,21958 |  |
| 10 | 0,217619 | 0,00 | -0,07877 |  |
| 11 | 0,131235 | 0,09 | 0,72355 |  |
| 12 | 0,131235 | 0,00 | 0,08482 |  |
| 13 | 0,226191 | 0,02 | -0,30347 |  |
| 14 | 0,226191 | 0,00 | 0,04440 |  |
| 15 | 0,143742 | 0,01 | 0,24187 |  |
| 16 | 0,143742 | 0,02 | -0,30962 |  |
| 17 | 0,206936 | 0,01 | 0,18445 |  |
| 18 | 0,206936 | 0,14 | 0,85845 |  |
| 19 | 0,134128 | 0,01 | 0,22924 |  |
| 20 | 0,200265 | 0,15 | 0,91317 |  |
| 21 | 0,161088 | 0,00 | -0,01381 |  |
| 22 | 0,246222 | 0,12 | 0,78631 |  |
| 23 | 0,039752 | 0,00 | 0,09278 |  |
| 24 | 0,039724 | 0,00 | 0,05771 |  |
| 25 | 0,038439 | 0,01 | -0,22770 |  |
| 26 | 0,038439 | 0,00 | -0,05557 |  |
| 27 | 0,038439 | 0,01 | 0,20614 |  |

R  Large residual

## Stepwise Selection of Terms

α to enter = 0,15; α to remove = 0,15

## Coded Coefficients

| Term | Coef | SE Coef | 95% CI | T-Value | P-Value | VIF |
| --- | --- | --- | --- | --- | --- | --- |
| Constant | 79,514 | 0,497 | (78,483; 80,546) | 159,88 | 0,000 |  |
| Lac | 9,68 | 1,03 | (7,54; 11,82) | 9,39 | 0,000 | 1,08 |
| HPMC\_Visc | -2,539 | 0,867 | (-4,338; -0,741) | -2,93 | 0,008 | 1,00 |
| HPMC\_HP | 2,617 | 0,986 | (0,572; 4,662) | 2,65 | 0,014 | 1,00 |
| Lac\*HPMC\_Visc | -4,66 | 2,02 | (-8,84; -0,47) | -2,31 | 0,031 | 1,08 |

## Model Summary

| S | R-sq | R-sq(adj) | PRESS | R-sq(pred) | AICc | BIC |
| --- | --- | --- | --- | --- | --- | --- |
| 2,43413 | 85,33% | 82,66% | 213,132 | 76,01% | 135,33 | 138,91 |

## Analysis of Variance

| Source | DF | Seq SS | Contribution | Adj SS | Adj MS | F-Value | P-Value |
| --- | --- | --- | --- | --- | --- | --- | --- |
| Model | 4 | 758,10 | 85,33% | 758,10 | 189,524 | 31,99 | 0,000 |
| Linear | 3 | 726,50 | 81,77% | 610,75 | 203,584 | 34,36 | 0,000 |
| Lac | 1 | 637,88 | 71,80% | 522,13 | 522,132 | 88,12 | 0,000 |
| HPMC\_Visc | 1 | 46,87 | 5,28% | 50,79 | 50,790 | 8,57 | 0,008 |
| HPMC\_HP | 1 | 41,75 | 4,70% | 41,75 | 41,747 | 7,05 | 0,014 |
| 2-Way Interaction | 1 | 31,59 | 3,56% | 31,59 | 31,593 | 5,33 | 0,031 |
| Lac\*HPMC\_Visc | 1 | 31,59 | 3,56% | 31,59 | 31,593 | 5,33 | 0,031 |
| Error | 22 | 130,35 | 14,67% | 130,35 | 5,925 |  |  |
| Lack-of-Fit | 20 | 116,99 | 13,17% | 116,99 | 5,850 | 0,88 | 0,661 |
| Pure Error | 2 | 13,36 | 1,50% | 13,36 | 6,678 |  |  |
| Total | 26 | 888,45 | 100,00% |  |  |  |  |

## Regression Equation in Uncoded Units

|  |  |  |
| --- | --- | --- |
| F\_mean\_13h(780min) | = | 12,2 + 103,4 Lac + 0,00174 HPMC\_Visc + 2,578 HPMC\_HP - 0,00479 Lac\*HPMC\_Visc |

## Fits and Diagnostics for All Observations

| Obs | F\_mean\_13h(780min) | Fit | SE Fit | 95% CI | Resid | Std Resid | Del Resid |
| --- | --- | --- | --- | --- | --- | --- | --- |
| 1 | 75,207 | 72,939 | 1,171 | (70,510; 75,368) | 2,268 | 1,06 | 1,07 |
| 2 | 85,112 | 85,932 | 1,171 | (83,503; 88,361) | -0,820 | -0,38 | -0,38 |
| 3 | 68,335 | 72,563 | 1,442 | (69,572; 75,554) | -4,228 | -2,16 | -2,37 |
| 4 | 77,455 | 79,030 | 1,442 | (76,039; 82,021) | -1,575 | -0,80 | -0,80 |
| 5 | 72,004 | 75,350 | 1,029 | (73,216; 77,484) | -3,346 | -1,52 | -1,57 |
| 6 | 84,615 | 88,051 | 1,029 | (85,917; 90,185) | -3,435 | -1,56 | -1,61 |
| 7 | 73,784 | 75,768 | 1,429 | (72,803; 78,732) | -1,984 | -1,01 | -1,01 |
| 8 | 80,481 | 82,137 | 1,429 | (79,173; 85,101) | -1,656 | -0,84 | -0,83 |
| 9 | 71,666 | 73,042 | 1,136 | (70,687; 75,397) | -1,376 | -0,64 | -0,63 |
| 10 | 86,275 | 85,943 | 1,136 | (83,588; 88,298) | 0,332 | 0,15 | 0,15 |
| 11 | 77,673 | 73,390 | 0,882 | (71,561; 75,219) | 4,282 | 1,89 | 2,01 |
| 12 | 82,648 | 82,179 | 0,882 | (80,351; 84,008) | 0,469 | 0,21 | 0,20 |
| 13 | 74,708 | 75,892 | 1,158 | (73,492; 78,293) | -1,184 | -0,55 | -0,54 |
| 14 | 88,777 | 88,834 | 1,158 | (86,433; 91,235) | -0,057 | -0,03 | -0,03 |
| 15 | 76,503 | 75,067 | 0,923 | (73,154; 76,981) | 1,435 | 0,64 | 0,63 |
| 16 | 81,890 | 83,430 | 0,923 | (81,517; 85,344) | -1,540 | -0,68 | -0,68 |
| 17 | 70,162 | 69,298 | 1,107 | (67,002; 71,594) | 0,864 | 0,40 | 0,39 |
| 18 | 93,340 | 90,401 | 1,107 | (88,104; 92,697) | 2,939 | 1,36 | 1,38 |
| 19 | 83,434 | 81,861 | 0,891 | (80,012; 83,709) | 1,574 | 0,69 | 0,69 |
| 20 | 80,136 | 76,524 | 1,089 | (74,265; 78,783) | 3,612 | 1,66 | 1,73 |
| 21 | 77,183 | 77,356 | 0,977 | (75,329; 79,382) | -0,173 | -0,08 | -0,08 |
| 22 | 85,226 | 82,425 | 1,208 | (79,921; 84,930) | 2,800 | 1,33 | 1,35 |
| 23 | 81,026 | 79,980 | 0,485 | (78,974; 80,987) | 1,045 | 0,44 | 0,43 |
| 24 | 80,490 | 79,749 | 0,485 | (78,743; 80,755) | 0,741 | 0,31 | 0,30 |
| 25 | 77,114 | 79,849 | 0,477 | (78,859; 80,839) | -2,735 | -1,15 | -1,15 |
| 26 | 79,194 | 79,849 | 0,477 | (78,859; 80,839) | -0,655 | -0,27 | -0,27 |
| 27 | 82,251 | 79,849 | 0,477 | (78,859; 80,839) | 2,402 | 1,01 | 1,01 |

| Obs | HI | Cook’s D | DFITS |  |
| --- | --- | --- | --- | --- |
| 1 | 0,231482 | 0,07 | 0,58514 |  |
| 2 | 0,231482 | 0,01 | -0,20668 |  |
| 3 | 0,351061 | 0,50 | -1,74471 | R |
| 4 | 0,351061 | 0,07 | -0,58588 |  |
| 5 | 0,178686 | 0,10 | -0,73035 |  |
| 6 | 0,178686 | 0,11 | -0,75238 |  |
| 7 | 0,344801 | 0,11 | -0,73069 |  |
| 8 | 0,344801 | 0,07 | -0,60550 |  |
| 9 | 0,217619 | 0,02 | -0,33229 |  |
| 10 | 0,217619 | 0,00 | 0,07957 |  |
| 11 | 0,131235 | 0,11 | 0,78295 |  |
| 12 | 0,131235 | 0,00 | 0,07854 |  |
| 13 | 0,226191 | 0,02 | -0,29422 |  |
| 14 | 0,226191 | 0,00 | -0,01413 |  |
| 15 | 0,143742 | 0,01 | 0,25749 |  |
| 16 | 0,143742 | 0,02 | -0,27668 |  |
| 17 | 0,206936 | 0,01 | 0,19957 |  |
| 18 | 0,206936 | 0,10 | 0,70692 |  |
| 19 | 0,134128 | 0,01 | 0,27015 |  |
| 20 | 0,200265 | 0,14 | 0,86728 |  |
| 21 | 0,161088 | 0,00 | -0,03324 |  |
| 22 | 0,246222 | 0,11 | 0,77133 |  |
| 23 | 0,039752 | 0,00 | 0,08751 |  |
| 24 | 0,039724 | 0,00 | 0,06191 |  |
| 25 | 0,038439 | 0,01 | -0,23087 |  |
| 26 | 0,038439 | 0,00 | -0,05373 |  |
| 27 | 0,038439 | 0,01 | 0,20127 |  |

R  Large residual

## Stepwise Selection of Terms

α to enter = 0,15; α to remove = 0,15

## Coded Coefficients

| Term | Coef | SE Coef | 95% CI | T-Value | P-Value | VIF |
| --- | --- | --- | --- | --- | --- | --- |
| Constant | 82,333 | 0,469 | (81,360; 83,306) | 175,52 | 0,000 |  |
| Lac | 9,515 | 0,972 | (7,499; 11,532) | 9,79 | 0,000 | 1,08 |
| HPMC\_Visc | -2,283 | 0,818 | (-3,980; -0,586) | -2,79 | 0,011 | 1,00 |
| HPMC\_HP | 2,488 | 0,930 | (0,559; 4,416) | 2,68 | 0,014 | 1,00 |
| Lac\*HPMC\_Visc | -4,25 | 1,90 | (-8,19; -0,30) | -2,23 | 0,036 | 1,08 |

## Model Summary

| S | R-sq | R-sq(adj) | PRESS | R-sq(pred) | AICc | BIC |
| --- | --- | --- | --- | --- | --- | --- |
| 2,29584 | 86,01% | 83,47% | 189,246 | 77,17% | 132,17 | 135,75 |

## Analysis of Variance

| Source | DF | Seq SS | Contribution | Adj SS | Adj MS | F-Value | P-Value |
| --- | --- | --- | --- | --- | --- | --- | --- |
| Model | 4 | 712,97 | 86,01% | 712,97 | 178,242 | 33,82 | 0,000 |
| Linear | 3 | 686,66 | 82,84% | 580,19 | 193,397 | 36,69 | 0,000 |
| Lac | 1 | 611,24 | 73,74% | 504,77 | 504,767 | 95,77 | 0,000 |
| HPMC\_Visc | 1 | 37,70 | 4,55% | 41,05 | 41,052 | 7,79 | 0,011 |
| HPMC\_HP | 1 | 37,72 | 4,55% | 37,72 | 37,718 | 7,16 | 0,014 |
| 2-Way Interaction | 1 | 26,31 | 3,17% | 26,31 | 26,311 | 4,99 | 0,036 |
| Lac\*HPMC\_Visc | 1 | 26,31 | 3,17% | 26,31 | 26,311 | 4,99 | 0,036 |
| Error | 22 | 115,96 | 13,99% | 115,96 | 5,271 |  |  |
| Lack-of-Fit | 20 | 103,76 | 12,52% | 103,76 | 5,188 | 0,85 | 0,671 |
| Pure Error | 2 | 12,20 | 1,47% | 12,20 | 6,100 |  |  |
| Total | 26 | 828,93 | 100,00% |  |  |  |  |

## Regression Equation in Uncoded Units

|  |  |  |
| --- | --- | --- |
| F\_mean\_14h(840min) | = | 18,5 + 97,1 Lac + 0,00160 HPMC\_Visc + 2,451 HPMC\_HP - 0,00437 Lac\*HPMC\_Visc |

## Fits and Diagnostics for All Observations

| Obs | F\_mean\_14h(840min) | Fit | SE Fit | 95% CI | Resid | Std Resid | Del Resid |
| --- | --- | --- | --- | --- | --- | --- | --- |
| 1 | 77,720 | 75,895 | 1,105 | (73,604; 78,185) | 1,826 | 0,91 | 0,90 |
| 2 | 87,872 | 88,435 | 1,105 | (86,144; 90,726) | -0,563 | -0,28 | -0,27 |
| 3 | 71,142 | 75,596 | 1,360 | (72,775; 78,417) | -4,454 | -2,41 | -2,74 |
| 4 | 80,866 | 82,182 | 1,360 | (79,360; 85,003) | -1,316 | -0,71 | -0,70 |
| 5 | 74,970 | 78,189 | 0,970 | (76,176; 80,201) | -3,219 | -1,55 | -1,60 |
| 6 | 87,433 | 90,463 | 0,970 | (88,450; 92,476) | -3,030 | -1,46 | -1,50 |
| 7 | 77,141 | 78,643 | 1,348 | (75,848; 81,439) | -1,503 | -0,81 | -0,80 |
| 8 | 84,045 | 85,140 | 1,348 | (82,344; 87,936) | -1,094 | -0,59 | -0,58 |
| 9 | 74,297 | 75,993 | 1,071 | (73,772; 78,214) | -1,696 | -0,84 | -0,83 |
| 10 | 89,522 | 88,450 | 1,071 | (86,229; 90,671) | 1,072 | 0,53 | 0,52 |
| 11 | 80,758 | 76,361 | 0,832 | (74,637; 78,086) | 4,397 | 2,05 | 2,23 |
| 12 | 85,507 | 85,066 | 0,832 | (83,341; 86,791) | 0,441 | 0,21 | 0,20 |
| 13 | 77,648 | 78,702 | 1,092 | (76,438; 80,967) | -1,055 | -0,52 | -0,51 |
| 14 | 90,387 | 91,196 | 1,092 | (88,932; 93,461) | -0,809 | -0,40 | -0,39 |
| 15 | 79,239 | 77,960 | 0,870 | (76,155; 79,765) | 1,279 | 0,60 | 0,59 |
| 16 | 84,863 | 86,275 | 0,870 | (84,470; 88,081) | -1,413 | -0,67 | -0,66 |
| 17 | 73,177 | 72,314 | 1,044 | (70,148; 74,480) | 0,863 | 0,42 | 0,41 |
| 18 | 94,946 | 92,939 | 1,044 | (90,773; 95,105) | 2,006 | 0,98 | 0,98 |
| 19 | 86,403 | 84,432 | 0,841 | (82,689; 86,176) | 1,970 | 0,92 | 0,92 |
| 20 | 82,719 | 79,621 | 1,027 | (77,490; 81,752) | 3,098 | 1,51 | 1,56 |
| 21 | 80,080 | 80,257 | 0,921 | (78,346; 82,168) | -0,177 | -0,08 | -0,08 |
| 22 | 87,653 | 85,085 | 1,139 | (82,722; 87,447) | 2,568 | 1,29 | 1,31 |
| 23 | 83,594 | 82,742 | 0,458 | (81,793; 83,691) | 0,852 | 0,38 | 0,37 |
| 24 | 83,371 | 82,520 | 0,458 | (81,571; 83,469) | 0,851 | 0,38 | 0,37 |
| 25 | 80,007 | 82,627 | 0,450 | (81,693; 83,560) | -2,620 | -1,16 | -1,17 |
| 26 | 82,056 | 82,627 | 0,450 | (81,693; 83,560) | -0,571 | -0,25 | -0,25 |
| 27 | 84,924 | 82,627 | 0,450 | (81,693; 83,560) | 2,297 | 1,02 | 1,02 |

| Obs | HI | Cook’s D | DFITS |  |
| --- | --- | --- | --- | --- |
| 1 | 0,231482 | 0,05 | 0,49572 |  |
| 2 | 0,231482 | 0,00 | -0,15038 |  |
| 3 | 0,351061 | 0,63 | -2,01682 | R |
| 4 | 0,351061 | 0,05 | -0,51722 |  |
| 5 | 0,178686 | 0,10 | -0,74680 |  |
| 6 | 0,178686 | 0,09 | -0,69810 |  |
| 7 | 0,344801 | 0,07 | -0,58180 |  |
| 8 | 0,344801 | 0,04 | -0,42074 |  |
| 9 | 0,217619 | 0,04 | -0,43733 |  |
| 10 | 0,217619 | 0,02 | 0,27367 |  |
| 11 | 0,131235 | 0,13 | 0,86792 | R |
| 12 | 0,131235 | 0,00 | 0,07832 |  |
| 13 | 0,226191 | 0,02 | -0,27758 |  |
| 14 | 0,226191 | 0,01 | -0,21237 |  |
| 15 | 0,143742 | 0,01 | 0,24299 |  |
| 16 | 0,143742 | 0,01 | -0,26894 |  |
| 17 | 0,206936 | 0,01 | 0,21143 |  |
| 18 | 0,206936 | 0,05 | 0,50085 |  |
| 19 | 0,134128 | 0,03 | 0,36172 |  |
| 20 | 0,200265 | 0,11 | 0,77911 |  |
| 21 | 0,161088 | 0,00 | -0,03603 |  |
| 22 | 0,246222 | 0,11 | 0,74819 |  |
| 23 | 0,039752 | 0,00 | 0,07557 |  |
| 24 | 0,039724 | 0,00 | 0,07538 |  |
| 25 | 0,038439 | 0,01 | -0,23467 |  |
| 26 | 0,038439 | 0,00 | -0,04961 |  |
| 27 | 0,038439 | 0,01 | 0,20420 |  |

R  Large residual

## Stepwise Selection of Terms

α to enter = 0,15; α to remove = 0,15

## Coded Coefficients

| Term | Coef | SE Coef | 95% CI | T-Value | P-Value | VIF |
| --- | --- | --- | --- | --- | --- | --- |
| Constant | 84,864 | 0,456 | (83,919; 85,810) | 186,13 | 0,000 |  |
| Lac | 9,185 | 0,945 | (7,225; 11,145) | 9,72 | 0,000 | 1,08 |
| HPMC\_Visc | -2,089 | 0,795 | (-3,738; -0,440) | -2,63 | 0,015 | 1,00 |
| HPMC\_HP | 2,276 | 0,904 | (0,401; 4,151) | 2,52 | 0,020 | 1,00 |
| Lac\*HPMC\_Visc | -3,64 | 1,85 | (-7,48; 0,19) | -1,97 | 0,061 | 1,08 |

## Model Summary

| S | R-sq | R-sq(adj) | PRESS | R-sq(pred) | AICc | BIC |
| --- | --- | --- | --- | --- | --- | --- |
| 2,23159 | 85,48% | 82,83% | 179,732 | 76,17% | 130,64 | 134,21 |

## Analysis of Variance

| Source | DF | Seq SS | Contribution | Adj SS | Adj MS | F-Value | P-Value |
| --- | --- | --- | --- | --- | --- | --- | --- |
| Model | 4 | 644,75 | 85,48% | 644,75 | 161,187 | 32,37 | 0,000 |
| Linear | 3 | 625,40 | 82,91% | 533,46 | 177,820 | 35,71 | 0,000 |
| Lac | 1 | 562,25 | 74,54% | 470,31 | 470,307 | 94,44 | 0,000 |
| HPMC\_Visc | 1 | 31,58 | 4,19% | 34,38 | 34,380 | 6,90 | 0,015 |
| HPMC\_HP | 1 | 31,58 | 4,19% | 31,58 | 31,576 | 6,34 | 0,020 |
| 2-Way Interaction | 1 | 19,35 | 2,56% | 19,35 | 19,347 | 3,88 | 0,061 |
| Lac\*HPMC\_Visc | 1 | 19,35 | 2,56% | 19,35 | 19,347 | 3,88 | 0,061 |
| Error | 22 | 109,56 | 14,52% | 109,56 | 4,980 |  |  |
| Lack-of-Fit | 20 | 98,91 | 13,11% | 98,91 | 4,945 | 0,93 | 0,640 |
| Pure Error | 2 | 10,65 | 1,41% | 10,65 | 5,325 |  |  |
| Total | 26 | 754,31 | 100,00% |  |  |  |  |

## Regression Equation in Uncoded Units

|  |  |  |
| --- | --- | --- |
| F\_mean\_15h(900min) | = | 27,2 + 87,4 Lac + 0,001336 HPMC\_Visc + 2,242 HPMC\_HP - 0,00375 Lac\*HPMC\_Visc |

## Fits and Diagnostics for All Observations

| Obs | F\_mean\_15h(900min) | Fit | SE Fit | 95% CI | Resid | Std Resid | Del Resid |
| --- | --- | --- | --- | --- | --- | --- | --- |
| 1 | 80,606 | 78,821 | 1,074 | (76,595; 81,048) | 1,784 | 0,91 | 0,91 |
| 2 | 90,402 | 90,600 | 1,074 | (88,374; 92,827) | -0,199 | -0,10 | -0,10 |
| 3 | 73,720 | 78,377 | 1,322 | (75,635; 81,119) | -4,656 | -2,59 | -3,04 |
| 4 | 83,969 | 85,049 | 1,322 | (82,307; 87,791) | -1,080 | -0,60 | -0,59 |
| 5 | 77,633 | 80,913 | 0,943 | (78,956; 82,869) | -3,279 | -1,62 | -1,69 |
| 6 | 89,900 | 92,463 | 0,943 | (90,507; 94,420) | -2,564 | -1,27 | -1,29 |
| 7 | 80,197 | 81,162 | 1,310 | (78,445; 83,880) | -0,965 | -0,53 | -0,53 |
| 8 | 87,181 | 87,758 | 1,310 | (85,041; 90,476) | -0,577 | -0,32 | -0,31 |
| 9 | 76,944 | 78,909 | 1,041 | (76,750; 81,068) | -1,965 | -1,00 | -1,00 |
| 10 | 92,342 | 90,616 | 1,041 | (88,457; 92,775) | 1,726 | 0,87 | 0,87 |
| 11 | 83,565 | 79,138 | 0,808 | (77,461; 80,814) | 4,427 | 2,13 | 2,33 |
| 12 | 87,990 | 87,627 | 0,808 | (85,951; 89,304) | 0,363 | 0,17 | 0,17 |
| 13 | 80,335 | 81,389 | 1,061 | (79,188; 83,590) | -1,054 | -0,54 | -0,53 |
| 14 | 91,608 | 93,128 | 1,061 | (90,927; 95,329) | -1,520 | -0,77 | -0,77 |
| 15 | 81,597 | 80,589 | 0,846 | (78,834; 82,344) | 1,008 | 0,49 | 0,48 |
| 16 | 87,555 | 88,745 | 0,846 | (86,990; 90,500) | -1,190 | -0,58 | -0,57 |
| 17 | 75,884 | 75,265 | 1,015 | (73,159; 77,370) | 0,620 | 0,31 | 0,31 |
| 18 | 95,792 | 95,002 | 1,015 | (92,896; 97,107) | 0,790 | 0,40 | 0,39 |
| 19 | 89,091 | 86,785 | 0,817 | (85,090; 88,480) | 2,305 | 1,11 | 1,12 |
| 20 | 85,073 | 82,383 | 0,999 | (80,311; 84,454) | 2,690 | 1,35 | 1,38 |
| 21 | 82,700 | 82,965 | 0,896 | (81,108; 84,823) | -0,266 | -0,13 | -0,13 |
| 22 | 89,972 | 87,382 | 1,107 | (85,086; 89,679) | 2,590 | 1,34 | 1,36 |
| 23 | 85,860 | 85,239 | 0,445 | (84,316; 86,161) | 0,621 | 0,28 | 0,28 |
| 24 | 86,024 | 85,036 | 0,445 | (84,113; 85,958) | 0,988 | 0,45 | 0,44 |
| 25 | 82,743 | 85,133 | 0,438 | (84,226; 86,040) | -2,390 | -1,09 | -1,10 |
| 26 | 84,715 | 85,133 | 0,438 | (84,226; 86,040) | -0,418 | -0,19 | -0,19 |
| 27 | 87,342 | 85,133 | 0,438 | (84,226; 86,040) | 2,209 | 1,01 | 1,01 |

| Obs | HI | Cook’s D | DFITS |  |
| --- | --- | --- | --- | --- |
| 1 | 0,231482 | 0,05 | 0,49855 |  |
| 2 | 0,231482 | 0,00 | -0,05446 |  |
| 3 | 0,351061 | 0,73 | -2,23262 | R |
| 4 | 0,351061 | 0,04 | -0,43515 |  |
| 5 | 0,178686 | 0,11 | -0,78751 |  |
| 6 | 0,178686 | 0,07 | -0,59999 |  |
| 7 | 0,344801 | 0,03 | -0,38108 |  |
| 8 | 0,344801 | 0,01 | -0,22699 |  |
| 9 | 0,217619 | 0,06 | -0,52491 |  |
| 10 | 0,217619 | 0,04 | 0,45854 |  |
| 11 | 0,131235 | 0,14 | 0,90704 | R |
| 12 | 0,131235 | 0,00 | 0,06629 |  |
| 13 | 0,226191 | 0,02 | -0,28551 |  |
| 14 | 0,226191 | 0,04 | -0,41469 |  |
| 15 | 0,143742 | 0,01 | 0,19655 |  |
| 16 | 0,143742 | 0,01 | -0,23253 |  |
| 17 | 0,206936 | 0,01 | 0,15599 |  |
| 18 | 0,206936 | 0,01 | 0,19919 |  |
| 19 | 0,134128 | 0,04 | 0,43939 |  |
| 20 | 0,200265 | 0,09 | 0,68808 |  |
| 21 | 0,161088 | 0,00 | -0,05565 |  |
| 22 | 0,246222 | 0,12 | 0,77873 |  |
| 23 | 0,039752 | 0,00 | 0,05657 |  |
| 24 | 0,039724 | 0,00 | 0,09021 |  |
| 25 | 0,038439 | 0,01 | -0,21940 |  |
| 26 | 0,038439 | 0,00 | -0,03732 |  |
| 27 | 0,038439 | 0,01 | 0,20196 |  |

R  Large residual

## Stepwise Selection of Terms

α to enter = 0,15; α to remove = 0,15

## Coded Coefficients

| Term | Coef | SE Coef | 95% CI | T-Value | P-Value | VIF |
| --- | --- | --- | --- | --- | --- | --- |
| Constant | 87,151 | 0,451 | (86,217; 88,086) | 193,43 | 0,000 |  |
| Lac | 8,692 | 0,934 | (6,755; 10,628) | 9,31 | 0,000 | 1,08 |
| HPMC\_Visc | -1,910 | 0,786 | (-3,539; -0,280) | -2,43 | 0,024 | 1,00 |
| HPMC\_HP | 2,096 | 0,893 | (0,243; 3,948) | 2,35 | 0,028 | 1,00 |
| Lac\*HPMC\_Visc | -3,50 | 1,83 | (-7,29; 0,29) | -1,92 | 0,068 | 1,08 |

## Model Summary

| S | R-sq | R-sq(adj) | PRESS | R-sq(pred) | AICc | BIC |
| --- | --- | --- | --- | --- | --- | --- |
| 2,20523 | 84,32% | 81,47% | 177,542 | 73,98% | 130,00 | 133,57 |

## Analysis of Variance

| Source | DF | Seq SS | Contribution | Adj SS | Adj MS | F-Value | P-Value |
| --- | --- | --- | --- | --- | --- | --- | --- |
| Model | 4 | 575,278 | 84,32% | 575,278 | 143,819 | 29,57 | 0,000 |
| Linear | 3 | 557,419 | 81,70% | 474,297 | 158,099 | 32,51 | 0,000 |
| Lac | 1 | 504,288 | 73,91% | 421,165 | 421,165 | 86,60 | 0,000 |
| HPMC\_Visc | 1 | 26,365 | 3,86% | 28,723 | 28,723 | 5,91 | 0,024 |
| HPMC\_HP | 1 | 26,767 | 3,92% | 26,767 | 26,767 | 5,50 | 0,028 |
| 2-Way Interaction | 1 | 17,858 | 2,62% | 17,858 | 17,858 | 3,67 | 0,068 |
| Lac\*HPMC\_Visc | 1 | 17,858 | 2,62% | 17,858 | 17,858 | 3,67 | 0,068 |
| Error | 22 | 106,987 | 15,68% | 106,987 | 4,863 |  |  |
| Lack-of-Fit | 20 | 97,558 | 14,30% | 97,558 | 4,878 | 1,03 | 0,603 |
| Pure Error | 2 | 9,429 | 1,38% | 9,429 | 4,715 |  |  |
| Total | 26 | 682,265 | 100,00% |  |  |  |  |

## Regression Equation in Uncoded Units

|  |  |  |
| --- | --- | --- |
| F\_mean\_16h(960min) | = | 32,5 + 83,4 Lac + 0,001309 HPMC\_Visc + 2,065 HPMC\_HP - 0,00360 Lac\*HPMC\_Visc |

## Fits and Diagnostics for All Observations

| Obs | F\_mean\_16h(960min) | Fit | SE Fit | 95% CI | Resid | Std Resid | Del Resid |
| --- | --- | --- | --- | --- | --- | --- | --- |
| 1 | 82,777 | 81,408 | 1,061 | (79,207; 83,608) | 1,369 | 0,71 | 0,70 |
| 2 | 93,041 | 92,592 | 1,061 | (90,391; 94,792) | 0,449 | 0,23 | 0,23 |
| 3 | 76,241 | 81,120 | 1,307 | (78,411; 83,830) | -4,879 | -2,75 | -3,31 |
| 4 | 86,554 | 87,398 | 1,307 | (84,688; 90,108) | -0,844 | -0,47 | -0,47 |
| 5 | 80,143 | 83,339 | 0,932 | (81,406; 85,272) | -3,196 | -1,60 | -1,66 |
| 6 | 92,409 | 94,303 | 0,932 | (92,370; 96,237) | -1,894 | -0,95 | -0,95 |
| 7 | 83,119 | 83,687 | 1,295 | (81,001; 86,372) | -0,567 | -0,32 | -0,31 |
| 8 | 89,650 | 89,891 | 1,295 | (87,206; 92,577) | -0,241 | -0,14 | -0,13 |
| 9 | 79,338 | 81,490 | 1,029 | (79,357; 83,623) | -2,152 | -1,10 | -1,11 |
| 10 | 94,542 | 92,605 | 1,029 | (90,472; 94,739) | 1,936 | 0,99 | 0,99 |
| 11 | 86,168 | 81,778 | 0,799 | (80,121; 83,435) | 4,390 | 2,14 | 2,34 |
| 12 | 90,270 | 89,802 | 0,799 | (88,145; 91,458) | 0,468 | 0,23 | 0,22 |
| 13 | 82,722 | 83,773 | 1,049 | (81,598; 85,948) | -1,051 | -0,54 | -0,53 |
| 14 | 92,607 | 94,918 | 1,049 | (92,743; 97,093) | -2,312 | -1,19 | -1,20 |
| 15 | 83,948 | 83,122 | 0,836 | (81,388; 84,856) | 0,826 | 0,40 | 0,40 |
| 16 | 89,920 | 90,825 | 0,836 | (89,091; 92,559) | -0,905 | -0,44 | -0,44 |
| 17 | 78,580 | 78,047 | 1,003 | (75,967; 80,128) | 0,532 | 0,27 | 0,27 |
| 18 | 96,318 | 96,745 | 1,003 | (94,664; 98,825) | -0,426 | -0,22 | -0,21 |
| 19 | 91,500 | 88,906 | 0,808 | (87,231; 90,581) | 2,594 | 1,26 | 1,28 |
| 20 | 87,193 | 84,880 | 0,987 | (82,834; 86,927) | 2,313 | 1,17 | 1,18 |
| 21 | 85,260 | 85,400 | 0,885 | (83,565; 87,236) | -0,140 | -0,07 | -0,07 |
| 22 | 92,111 | 89,468 | 1,094 | (87,198; 91,737) | 2,644 | 1,38 | 1,41 |
| 23 | 87,826 | 87,492 | 0,440 | (86,580; 88,404) | 0,333 | 0,15 | 0,15 |
| 24 | 88,425 | 87,305 | 0,440 | (86,394; 88,217) | 1,120 | 0,52 | 0,51 |
| 25 | 85,154 | 87,396 | 0,432 | (86,499; 88,293) | -2,242 | -1,04 | -1,04 |
| 26 | 87,173 | 87,396 | 0,432 | (86,499; 88,293) | -0,223 | -0,10 | -0,10 |
| 27 | 89,493 | 87,396 | 0,432 | (86,499; 88,293) | 2,097 | 0,97 | 0,97 |

| Obs | HI | Cook’s D | DFITS |  |
| --- | --- | --- | --- | --- |
| 1 | 0,231482 | 0,03 | 0,38413 |  |
| 2 | 0,231482 | 0,00 | 0,12466 |  |
| 3 | 0,351061 | 0,82 | -2,43449 | R |
| 4 | 0,351061 | 0,02 | -0,34299 |  |
| 5 | 0,178686 | 0,11 | -0,77520 |  |
| 6 | 0,178686 | 0,04 | -0,44098 |  |
| 7 | 0,344801 | 0,01 | -0,22579 |  |
| 8 | 0,344801 | 0,00 | -0,09591 |  |
| 9 | 0,217619 | 0,07 | -0,58480 |  |
| 10 | 0,217619 | 0,05 | 0,52337 |  |
| 11 | 0,131235 | 0,14 | 0,91099 | R |
| 12 | 0,131235 | 0,00 | 0,08662 |  |
| 13 | 0,226191 | 0,02 | -0,28812 |  |
| 14 | 0,226191 | 0,08 | -0,65078 |  |
| 15 | 0,143742 | 0,01 | 0,16268 |  |
| 16 | 0,143742 | 0,01 | -0,17837 |  |
| 17 | 0,206936 | 0,00 | 0,13548 |  |
| 18 | 0,206936 | 0,00 | -0,10847 |  |
| 19 | 0,134128 | 0,05 | 0,50483 |  |
| 20 | 0,200265 | 0,07 | 0,59224 |  |
| 21 | 0,161088 | 0,00 | -0,02962 |  |
| 22 | 0,246222 | 0,12 | 0,80675 |  |
| 23 | 0,039752 | 0,00 | 0,03069 |  |
| 24 | 0,039724 | 0,00 | 0,10361 |  |
| 25 | 0,038439 | 0,01 | -0,20769 |  |
| 26 | 0,038439 | 0,00 | -0,02018 |  |
| 27 | 0,038439 | 0,01 | 0,19361 |  |

R  Large residual

## Stepwise Selection of Terms

α to enter = 0,15; α to remove = 0,15

## Coded Coefficients

| Term | Coef | SE Coef | 95% CI | T-Value | P-Value | VIF |
| --- | --- | --- | --- | --- | --- | --- |
| Constant | 89,156 | 0,446 | (88,229; 90,082) | 200,12 | 0,000 |  |
| Lac | 8,049 | 0,923 | (6,129; 9,969) | 8,72 | 0,000 | 1,08 |
| HPMC\_Visc | -1,469 | 0,817 | (-3,167; 0,230) | -1,80 | 0,087 | 1,11 |
| HPMC\_HP | 2,012 | 0,898 | (0,145; 3,880) | 2,24 | 0,036 | 1,04 |
| Lac\*HPMC\_Visc | -2,89 | 1,81 | (-6,65; 0,87) | -1,60 | 0,124 | 1,08 |
| HPMC\_Visc\*HPMC\_HP | 2,76 | 1,64 | (-0,66; 6,17) | 1,68 | 0,108 | 1,15 |

## Model Summary

| S | R-sq | R-sq(adj) | PRESS | R-sq(pred) | AICc | BIC |
| --- | --- | --- | --- | --- | --- | --- |
| 2,18018 | 83,30% | 79,32% | 191,929 | 67,89% | 131,82 | 135,00 |

## Analysis of Variance

| Source | DF | Seq SS | Contribution | Adj SS | Adj MS | F-Value | P-Value |
| --- | --- | --- | --- | --- | --- | --- | --- |
| Model | 5 | 497,901 | 83,30% | 497,901 | 99,580 | 20,95 | 0,000 |
| Linear | 3 | 472,325 | 79,02% | 400,972 | 133,657 | 28,12 | 0,000 |
| Lac | 1 | 427,608 | 71,54% | 361,175 | 361,175 | 75,99 | 0,000 |
| HPMC\_Visc | 1 | 26,277 | 4,40% | 15,366 | 15,366 | 3,23 | 0,087 |
| HPMC\_HP | 1 | 18,440 | 3,09% | 23,871 | 23,871 | 5,02 | 0,036 |
| 2-Way Interaction | 2 | 25,577 | 4,28% | 25,577 | 12,788 | 2,69 | 0,091 |
| Lac\*HPMC\_Visc | 1 | 12,178 | 2,04% | 12,178 | 12,178 | 2,56 | 0,124 |
| HPMC\_Visc\*HPMC\_HP | 1 | 13,398 | 2,24% | 13,398 | 13,398 | 2,82 | 0,108 |
| Error | 21 | 99,817 | 16,70% | 99,817 | 4,753 |  |  |
| Lack-of-Fit | 19 | 91,312 | 15,28% | 91,312 | 4,806 | 1,13 | 0,571 |
| Pure Error | 2 | 8,505 | 1,42% | 8,505 | 4,253 |  |  |
| Total | 26 | 597,718 | 100,00% |  |  |  |  |

## Regression Equation in Uncoded Units

|  |  |  |
| --- | --- | --- |
| F\_mean\_17h(1020min) | = | 128,7 + 72,4 Lac - 0,00551 HPMC\_Visc - 7,45 HPMC\_HP - 0,00297 Lac\*HPMC\_Visc + 0,000698 HPMC\_Visc\*HPMC\_HP |

## Fits and Diagnostics for All Observations

| Obs | F\_mean\_17h(1020min) | Fit | SE Fit | 95% CI | Resid | Std Resid | Del Resid |
| --- | --- | --- | --- | --- | --- | --- | --- |
| 1 | 86,287 | 85,112 | 1,186 | (82,646; 87,579) | 1,174 | 0,64 | 0,63 |
| 2 | 94,993 | 95,219 | 1,186 | (92,753; 97,686) | -0,226 | -0,12 | -0,12 |
| 3 | 78,511 | 82,173 | 1,515 | (79,023; 85,324) | -3,663 | -2,34 | -2,65 |
| 4 | 88,721 | 88,229 | 1,515 | (85,078; 91,380) | 0,492 | 0,31 | 0,31 |
| 5 | 82,549 | 85,192 | 0,983 | (83,148; 87,236) | -2,643 | -1,36 | -1,39 |
| 6 | 94,030 | 95,118 | 0,983 | (93,074; 97,162) | -1,088 | -0,56 | -0,55 |
| 7 | 85,468 | 86,999 | 1,519 | (83,840; 90,158) | -1,531 | -0,98 | -0,98 |
| 8 | 91,504 | 92,994 | 1,519 | (89,835; 96,153) | -1,490 | -0,95 | -0,95 |
| 9 | 81,530 | 85,077 | 1,131 | (82,724; 87,430) | -3,547 | -1,90 | -2,04 |
| 10 | 96,321 | 95,127 | 1,131 | (92,774; 97,480) | 1,194 | 0,64 | 0,63 |
| 11 | 88,495 | 83,919 | 0,808 | (82,239; 85,599) | 4,576 | 2,26 | 2,53 |
| 12 | 92,370 | 91,416 | 0,808 | (89,736; 93,097) | 0,954 | 0,47 | 0,46 |
| 13 | 84,957 | 85,180 | 1,185 | (82,716; 87,644) | -0,223 | -0,12 | -0,12 |
| 14 | 93,700 | 95,255 | 1,185 | (92,791; 97,719) | -1,555 | -0,85 | -0,84 |
| 15 | 86,046 | 85,606 | 0,847 | (83,844; 87,368) | 0,440 | 0,22 | 0,21 |
| 16 | 91,962 | 92,839 | 0,847 | (91,076; 94,601) | -0,877 | -0,44 | -0,43 |
| 17 | 80,949 | 80,759 | 0,992 | (78,695; 82,823) | 0,190 | 0,10 | 0,10 |
| 18 | 96,721 | 97,942 | 0,992 | (95,878; 100,005) | -1,221 | -0,63 | -0,62 |
| 19 | 93,609 | 90,679 | 0,810 | (88,995; 92,364) | 2,930 | 1,45 | 1,49 |
| 20 | 89,036 | 86,865 | 0,977 | (84,833; 88,896) | 2,172 | 1,11 | 1,12 |
| 21 | 87,569 | 87,906 | 0,880 | (86,075; 89,737) | -0,337 | -0,17 | -0,17 |
| 22 | 93,783 | 91,019 | 1,083 | (88,767; 93,271) | 2,764 | 1,46 | 1,50 |
| 23 | 89,589 | 89,441 | 0,436 | (88,534; 90,349) | 0,147 | 0,07 | 0,07 |
| 24 | 90,600 | 89,333 | 0,435 | (88,429; 90,237) | 1,267 | 0,59 | 0,58 |
| 25 | 87,288 | 89,350 | 0,429 | (88,459; 90,242) | -2,063 | -0,96 | -0,96 |
| 26 | 89,453 | 89,350 | 0,429 | (88,459; 90,242) | 0,103 | 0,05 | 0,05 |
| 27 | 91,410 | 89,350 | 0,429 | (88,459; 90,242) | 2,060 | 0,96 | 0,96 |

| Obs | HI | Cook’s D | DFITS |  |
| --- | --- | --- | --- | --- |
| 1 | 0,295939 | 0,03 | 0,41025 |  |
| 2 | 0,295939 | 0,00 | -0,07819 |  |
| 3 | 0,482953 | 0,85 | -2,56136 | R |
| 4 | 0,482953 | 0,02 | 0,29682 |  |
| 5 | 0,203286 | 0,08 | -0,70102 |  |
| 6 | 0,203286 | 0,01 | -0,27760 |  |
| 7 | 0,485441 | 0,15 | -0,95005 |  |
| 8 | 0,485441 | 0,14 | -0,92310 |  |
| 9 | 0,269274 | 0,22 | -1,23929 |  |
| 10 | 0,269274 | 0,03 | 0,38316 |  |
| 11 | 0,137369 | 0,14 | 1,01158 | R |
| 12 | 0,137369 | 0,01 | 0,18448 |  |
| 13 | 0,295333 | 0,00 | -0,07706 |  |
| 14 | 0,295333 | 0,05 | -0,54622 |  |
| 15 | 0,151077 | 0,00 | 0,09033 |  |
| 16 | 0,151077 | 0,01 | -0,18052 |  |
| 17 | 0,207151 | 0,00 | 0,04874 |  |
| 18 | 0,207151 | 0,02 | -0,31669 |  |
| 19 | 0,138002 | 0,06 | 0,59568 |  |
| 20 | 0,200816 | 0,05 | 0,56201 |  |
| 21 | 0,163018 | 0,00 | -0,07290 |  |
| 22 | 0,246731 | 0,12 | 0,86082 |  |
| 23 | 0,040065 | 0,00 | 0,01376 |  |
| 24 | 0,039756 | 0,00 | 0,11872 |  |
| 25 | 0,038655 | 0,01 | -0,19316 |  |
| 26 | 0,038655 | 0,00 | 0,00941 |  |
| 27 | 0,038655 | 0,01 | 0,19288 |  |

R  Large residual

## Stepwise Selection of Terms

α to enter = 0,15; α to remove = 0,15

## Coded Coefficients

| Term | Coef | SE Coef | 95% CI | T-Value | P-Value | VIF |
| --- | --- | --- | --- | --- | --- | --- |
| Constant | 90,873 | 0,448 | (89,942; 91,804) | 202,98 | 0,000 |  |
| Lac | 7,389 | 0,928 | (5,460; 9,319) | 7,96 | 0,000 | 1,08 |
| HPMC\_Visc | -1,331 | 0,821 | (-3,038; 0,376) | -1,62 | 0,120 | 1,11 |
| HPMC\_HP | 1,740 | 0,902 | (-0,137; 3,616) | 1,93 | 0,067 | 1,04 |
| Lac\*HPMC\_Visc | -2,77 | 1,82 | (-6,54; 1,01) | -1,53 | 0,142 | 1,08 |
| HPMC\_Visc\*HPMC\_HP | 2,95 | 1,65 | (-0,48; 6,38) | 1,79 | 0,088 | 1,15 |

## Model Summary

| S | R-sq | R-sq(adj) | PRESS | R-sq(pred) | AICc | BIC |
| --- | --- | --- | --- | --- | --- | --- |
| 2,19087 | 80,82% | 76,25% | 193,316 | 63,21% | 132,08 | 135,26 |

## Analysis of Variance

| Source | DF | Seq SS | Contribution | Adj SS | Adj MS | F-Value | P-Value |
| --- | --- | --- | --- | --- | --- | --- | --- |
| Model | 5 | 424,673 | 80,82% | 424,673 | 84,935 | 17,70 | 0,000 |
| Linear | 3 | 398,179 | 75,78% | 335,305 | 111,768 | 23,29 | 0,000 |
| Lac | 1 | 361,840 | 68,86% | 304,404 | 304,404 | 63,42 | 0,000 |
| HPMC\_Visc | 1 | 23,561 | 4,48% | 12,618 | 12,618 | 2,63 | 0,120 |
| HPMC\_HP | 1 | 12,778 | 2,43% | 17,845 | 17,845 | 3,72 | 0,067 |
| 2-Way Interaction | 2 | 26,495 | 5,04% | 26,495 | 13,247 | 2,76 | 0,086 |
| Lac\*HPMC\_Visc | 1 | 11,166 | 2,12% | 11,166 | 11,166 | 2,33 | 0,142 |
| HPMC\_Visc\*HPMC\_HP | 1 | 15,329 | 2,92% | 15,329 | 15,329 | 3,19 | 0,088 |
| Error | 21 | 100,798 | 19,18% | 100,798 | 4,800 |  |  |
| Lack-of-Fit | 19 | 92,946 | 17,69% | 92,946 | 4,892 | 1,25 | 0,537 |
| Pure Error | 2 | 7,852 | 1,49% | 7,852 | 3,926 |  |  |
| Total | 26 | 525,472 | 100,00% |  |  |  |  |

## Regression Equation in Uncoded Units

|  |  |  |
| --- | --- | --- |
| F\_mean\_18h(1080min) | = | 140,9 + 68,0 Lac - 0,00600 HPMC\_Visc - 8,38 HPMC\_HP - 0,00285 Lac\*HPMC\_Visc + 0,000747 HPMC\_Visc\*HPMC\_HP |

## Fits and Diagnostics for All Observations

| Obs | F\_mean\_18h(1080min) | Fit | SE Fit | 95% CI | Resid | Std Resid | Del Resid |
| --- | --- | --- | --- | --- | --- | --- | --- |
| 1 | 88,263 | 87,400 | 1,192 | (84,921; 89,878) | 0,863 | 0,47 | 0,46 |
| 2 | 96,841 | 96,760 | 1,192 | (94,281; 99,238) | 0,082 | 0,04 | 0,04 |
| 3 | 80,676 | 84,378 | 1,523 | (81,212; 87,544) | -3,702 | -2,35 | -2,67 |
| 4 | 90,400 | 89,859 | 1,523 | (86,692; 93,025) | 0,541 | 0,34 | 0,34 |
| 5 | 84,581 | 87,107 | 0,988 | (85,053; 89,162) | -2,526 | -1,29 | -1,31 |
| 6 | 95,182 | 96,294 | 0,988 | (94,240; 98,348) | -1,112 | -0,57 | -0,56 |
| 7 | 87,829 | 89,036 | 1,526 | (85,861; 92,210) | -1,206 | -0,77 | -0,76 |
| 8 | 92,846 | 94,458 | 1,526 | (91,284; 97,633) | -1,612 | -1,03 | -1,03 |
| 9 | 83,545 | 87,347 | 1,137 | (84,982; 89,711) | -3,801 | -2,03 | -2,21 |
| 10 | 97,777 | 96,652 | 1,137 | (94,288; 99,016) | 1,125 | 0,60 | 0,59 |
| 11 | 90,546 | 86,093 | 0,812 | (84,404; 87,781) | 4,453 | 2,19 | 2,43 |
| 12 | 94,342 | 92,954 | 0,812 | (91,265; 94,642) | 1,388 | 0,68 | 0,67 |
| 13 | 86,942 | 87,007 | 1,191 | (84,531; 89,483) | -0,065 | -0,04 | -0,03 |
| 14 | 94,594 | 96,337 | 1,191 | (93,861; 98,813) | -1,743 | -0,95 | -0,95 |
| 15 | 87,894 | 87,637 | 0,852 | (85,866; 89,407) | 0,258 | 0,13 | 0,12 |
| 16 | 93,801 | 94,244 | 0,852 | (92,473; 96,015) | -0,443 | -0,22 | -0,21 |
| 17 | 83,208 | 83,149 | 0,997 | (81,076; 85,223) | 0,059 | 0,03 | 0,03 |
| 18 | 97,049 | 98,967 | 0,997 | (96,893; 101,040) | -1,918 | -0,98 | -0,98 |
| 19 | 95,396 | 92,293 | 0,814 | (90,600; 93,985) | 3,104 | 1,53 | 1,58 |
| 20 | 90,556 | 88,733 | 0,982 | (86,691; 90,775) | 1,822 | 0,93 | 0,93 |
| 21 | 89,650 | 89,905 | 0,885 | (88,066; 91,745) | -0,255 | -0,13 | -0,12 |
| 22 | 95,014 | 92,425 | 1,088 | (90,162; 94,688) | 2,589 | 1,36 | 1,39 |
| 23 | 91,006 | 91,146 | 0,439 | (90,234; 92,058) | -0,140 | -0,07 | -0,06 |
| 24 | 92,525 | 91,069 | 0,437 | (90,161; 91,978) | 1,456 | 0,68 | 0,67 |
| 25 | 89,220 | 91,058 | 0,431 | (90,162; 91,954) | -1,838 | -0,86 | -0,85 |
| 26 | 91,582 | 91,058 | 0,431 | (90,162; 91,954) | 0,524 | 0,24 | 0,24 |
| 27 | 93,157 | 91,058 | 0,431 | (90,162; 91,954) | 2,099 | 0,98 | 0,98 |

| Obs | HI | Cook’s D | DFITS |  |
| --- | --- | --- | --- | --- |
| 1 | 0,295939 | 0,02 | 0,29874 |  |
| 2 | 0,295939 | 0,00 | 0,02816 |  |
| 3 | 0,482953 | 0,86 | -2,58183 | R |
| 4 | 0,482953 | 0,02 | 0,32472 |  |
| 5 | 0,203286 | 0,07 | -0,66379 |  |
| 6 | 0,203286 | 0,01 | -0,28251 |  |
| 7 | 0,485441 | 0,09 | -0,73792 |  |
| 8 | 0,485441 | 0,17 | -0,99772 |  |
| 9 | 0,269274 | 0,25 | -1,34110 | R |
| 10 | 0,269274 | 0,02 | 0,35894 |  |
| 11 | 0,137369 | 0,13 | 0,97002 | R |
| 12 | 0,137369 | 0,01 | 0,26868 |  |
| 13 | 0,295333 | 0,00 | -0,02241 |  |
| 14 | 0,295333 | 0,06 | -0,61187 |  |
| 15 | 0,151077 | 0,00 | 0,05256 |  |
| 16 | 0,151077 | 0,00 | -0,09051 |  |
| 17 | 0,207151 | 0,00 | 0,01502 |  |
| 18 | 0,207151 | 0,04 | -0,50202 |  |
| 19 | 0,138002 | 0,06 | 0,63183 |  |
| 20 | 0,200816 | 0,04 | 0,46488 |  |
| 21 | 0,163018 | 0,00 | -0,05486 |  |
| 22 | 0,246731 | 0,10 | 0,79650 |  |
| 23 | 0,040065 | 0,00 | -0,01298 |  |
| 24 | 0,039756 | 0,00 | 0,13614 |  |
| 25 | 0,038655 | 0,00 | -0,17044 |  |
| 26 | 0,038655 | 0,00 | 0,04777 |  |
| 27 | 0,038655 | 0,01 | 0,19568 |  |

R  Large residual

## Stepwise Selection of Terms

α to enter = 0,15; α to remove = 0,15

## Coded Coefficients

| Term | Coef | SE Coef | 95% CI | T-Value | P-Value | VIF |
| --- | --- | --- | --- | --- | --- | --- |
| Constant | 92,106 | 0,473 | (91,130; 93,082) | 194,80 | 0,000 |  |
| Lac | 7,057 | 0,975 | (5,044; 9,069) | 7,24 | 0,000 | 1,00 |
| HPMC\_Visc | -1,526 | 0,850 | (-3,280; 0,229) | -1,79 | 0,085 | 1,00 |

## Model Summary

| S | R-sq | R-sq(adj) | PRESS | R-sq(pred) | AICc | BIC |
| --- | --- | --- | --- | --- | --- | --- |
| 2,38814 | 69,85% | 67,34% | 176,994 | 61,02% | 130,27 | 133,63 |

## Analysis of Variance

| Source | DF | Seq SS | Contribution | Adj SS | Adj MS | F-Value | P-Value |
| --- | --- | --- | --- | --- | --- | --- | --- |
| Model | 2 | 317,143 | 69,85% | 317,143 | 158,572 | 27,80 | 0,000 |
| Linear | 2 | 317,143 | 69,85% | 317,143 | 158,572 | 27,80 | 0,000 |
| Lac | 1 | 298,769 | 65,81% | 298,769 | 298,769 | 52,39 | 0,000 |
| HPMC\_Visc | 1 | 18,375 | 4,05% | 18,375 | 18,375 | 3,22 | 0,085 |
| Error | 24 | 136,877 | 30,15% | 136,877 | 5,703 |  |  |
| Lack-of-Fit | 22 | 129,289 | 28,48% | 129,289 | 5,877 | 1,55 | 0,466 |
| Pure Error | 2 | 7,588 | 1,67% | 7,588 | 3,794 |  |  |
| Total | 26 | 454,020 | 100,00% |  |  |  |  |

## Regression Equation in Uncoded Units

|  |  |  |
| --- | --- | --- |
| F\_mean\_19h(1140min) | = | 83,29 + 28,23 Lac - 0,000392 HPMC\_Visc |

## Fits and Diagnostics for All Observations

| Obs | F\_mean\_19h(1140min) | Fit | SE Fit | 95% CI | Resid | Std Resid | Del Resid |
| --- | --- | --- | --- | --- | --- | --- | --- |
| 1 | 89,693 | 89,664 | 0,833 | (87,946; 91,382) | 0,029 | 0,01 | 0,01 |
| 2 | 97,816 | 96,720 | 0,833 | (95,002; 98,439) | 1,095 | 0,49 | 0,48 |
| 3 | 82,737 | 87,525 | 0,967 | (85,530; 89,521) | -4,789 | -2,19 | -2,40 |
| 4 | 91,735 | 94,582 | 0,967 | (92,586; 96,578) | -2,847 | -1,30 | -1,32 |
| 5 | 86,118 | 89,568 | 0,802 | (87,913; 91,224) | -3,450 | -1,53 | -1,58 |
| 6 | 96,013 | 96,625 | 0,802 | (94,970; 98,280) | -0,612 | -0,27 | -0,27 |
| 7 | 89,485 | 87,494 | 0,980 | (85,471; 89,516) | 1,991 | 0,91 | 0,91 |
| 8 | 93,940 | 94,550 | 0,980 | (92,528; 96,572) | -0,610 | -0,28 | -0,27 |
| 9 | 85,335 | 89,634 | 0,823 | (87,936; 91,332) | -4,299 | -1,92 | -2,04 |
| 10 | 98,817 | 96,690 | 0,823 | (94,992; 98,388) | 2,127 | 0,95 | 0,95 |
| 11 | 92,469 | 88,286 | 0,724 | (86,793; 89,780) | 4,182 | 1,84 | 1,94 |
| 12 | 95,751 | 95,343 | 0,724 | (93,850; 96,836) | 0,407 | 0,18 | 0,18 |
| 13 | 88,653 | 89,647 | 0,827 | (87,940; 91,354) | -0,995 | -0,44 | -0,44 |
| 14 | 94,926 | 96,704 | 0,827 | (94,997; 98,411) | -1,778 | -0,79 | -0,79 |
| 15 | 89,395 | 88,147 | 0,756 | (86,586; 89,708) | 1,249 | 0,55 | 0,54 |
| 16 | 95,487 | 95,203 | 0,756 | (93,642; 96,764) | 0,284 | 0,13 | 0,12 |
| 17 | 85,239 | 85,336 | 1,079 | (83,109; 87,562) | -0,097 | -0,05 | -0,04 |
| 18 | 97,278 | 99,449 | 1,079 | (97,222; 101,676) | -2,171 | -1,02 | -1,02 |
| 19 | 96,840 | 93,632 | 0,870 | (91,835; 95,428) | 3,208 | 1,44 | 1,48 |
| 20 | 91,993 | 90,580 | 1,065 | (88,381; 92,779) | 1,413 | 0,66 | 0,65 |
| 21 | 91,337 | 92,381 | 0,462 | (91,429; 93,334) | -1,045 | -0,45 | -0,44 |
| 22 | 95,783 | 92,282 | 0,460 | (91,333; 93,231) | 3,501 | 1,49 | 1,54 |
| 23 | 92,347 | 92,502 | 0,472 | (91,527; 93,477) | -0,155 | -0,07 | -0,06 |
| 24 | 94,127 | 92,518 | 0,475 | (91,538; 93,497) | 1,609 | 0,69 | 0,68 |
| 25 | 90,831 | 92,392 | 0,462 | (91,438; 93,346) | -1,561 | -0,67 | -0,66 |
| 26 | 93,460 | 92,392 | 0,462 | (91,438; 93,346) | 1,068 | 0,46 | 0,45 |
| 27 | 94,635 | 92,392 | 0,462 | (91,438; 93,346) | 2,243 | 0,96 | 0,96 |

| Obs | HI | Cook’s D | DFITS |  |
| --- | --- | --- | --- | --- |
| 1 | 0,121521 | 0,00 | 0,00477 |  |
| 2 | 0,121521 | 0,01 | 0,17903 |  |
| 3 | 0,163929 | 0,31 | -1,06304 | R |
| 4 | 0,163929 | 0,11 | -0,58631 |  |
| 5 | 0,112791 | 0,10 | -0,56375 |  |
| 6 | 0,112791 | 0,00 | -0,09504 |  |
| 7 | 0,168329 | 0,06 | 0,40983 |  |
| 8 | 0,168329 | 0,01 | -0,12355 |  |
| 9 | 0,118673 | 0,17 | -0,74849 |  |
| 10 | 0,118673 | 0,04 | 0,34731 |  |
| 11 | 0,091792 | 0,11 | 0,61696 |  |
| 12 | 0,091792 | 0,00 | 0,05576 |  |
| 13 | 0,119923 | 0,01 | -0,16108 |  |
| 14 | 0,119923 | 0,03 | -0,29059 |  |
| 15 | 0,100307 | 0,01 | 0,18131 |  |
| 16 | 0,100307 | 0,00 | 0,04100 |  |
| 17 | 0,204116 | 0,00 | -0,02246 |  |
| 18 | 0,204116 | 0,09 | -0,51635 |  |
| 19 | 0,132807 | 0,11 | 0,57824 |  |
| 20 | 0,199025 | 0,04 | 0,32554 |  |
| 21 | 0,037352 | 0,00 | -0,08632 |  |
| 22 | 0,037065 | 0,03 | 0,30128 |  |
| 23 | 0,039146 | 0,00 | -0,01308 |  |
| 24 | 0,039495 | 0,01 | 0,13784 |  |
| 25 | 0,037449 | 0,01 | -0,12986 |  |
| 26 | 0,037449 | 0,00 | 0,08841 |  |
| 27 | 0,037449 | 0,01 | 0,18849 |  |

R  Large residual

## Stepwise Selection of Terms

α to enter = 0,15; α to remove = 0,15

## Coded Coefficients

| Term | Coef | SE Coef | 95% CI | T-Value | P-Value | VIF |
| --- | --- | --- | --- | --- | --- | --- |
| Constant | 94,115 | 0,586 | (92,902; 95,328) | 160,51 | 0,000 |  |
| Lac | 6,285 | 0,927 | (4,367; 8,203) | 6,78 | 0,000 | 1,00 |
| HPMC\_PS | 1,99 | 1,09 | (-0,27; 4,25) | 1,82 | 0,081 | 1,00 |
| Lac\*Lac | -2,65 | 1,76 | (-6,29; 0,99) | -1,51 | 0,145 | 1,00 |

## Model Summary

| S | R-sq | R-sq(adj) | PRESS | R-sq(pred) | AICc | BIC |
| --- | --- | --- | --- | --- | --- | --- |
| 2,27078 | 69,16% | 65,13% | 167,547 | 56,43% | 129,44 | 133,06 |

## Analysis of Variance

| Source | DF | Seq SS | Contribution | Adj SS | Adj MS | F-Value | P-Value |
| --- | --- | --- | --- | --- | --- | --- | --- |
| Model | 3 | 265,929 | 69,16% | 265,929 | 88,643 | 17,19 | 0,000 |
| Linear | 2 | 254,217 | 66,11% | 254,134 | 127,067 | 24,64 | 0,000 |
| Lac | 1 | 237,011 | 61,64% | 237,011 | 237,011 | 45,96 | 0,000 |
| HPMC\_PS | 1 | 17,206 | 4,47% | 17,123 | 17,123 | 3,32 | 0,081 |
| Square | 1 | 11,712 | 3,05% | 11,712 | 11,712 | 2,27 | 0,145 |
| Lac\*Lac | 1 | 11,712 | 3,05% | 11,712 | 11,712 | 2,27 | 0,145 |
| Error | 23 | 118,598 | 30,84% | 118,598 | 5,156 |  |  |
| Lack-of-Fit | 21 | 111,108 | 28,89% | 111,108 | 5,291 | 1,41 | 0,496 |
| Pure Error | 2 | 7,490 | 1,95% | 7,490 | 3,745 |  |  |
| Total | 26 | 384,527 | 100,00% |  |  |  |  |

## Regression Equation in Uncoded Units

|  |  |  |
| --- | --- | --- |
| F\_mean\_20h(1200min) | = | 52,1 + 67,6 Lac + 0,271 HPMC\_PS - 42,4 Lac\*Lac |

## Fits and Diagnostics for All Observations

| Obs | F\_mean\_20h(1200min) | Fit | SE Fit | 95% CI | Resid | Std Resid | Del Resid |
| --- | --- | --- | --- | --- | --- | --- | --- |
| 1 | 91,09 | 89,82 | 0,69 | (88,39; 91,25) | 1,27 | 0,59 | 0,58 |
| 2 | 98,57 | 96,10 | 0,69 | (94,67; 97,53) | 2,47 | 1,14 | 1,15 |
| 3 | 84,65 | 89,16 | 0,89 | (87,31; 91,00) | -4,51 | -2,16 | -2,37 |
| 4 | 92,73 | 95,44 | 0,89 | (93,60; 97,29) | -2,71 | -1,30 | -1,32 |
| 5 | 87,77 | 89,97 | 0,66 | (88,60; 91,34) | -2,19 | -1,01 | -1,01 |
| 6 | 96,43 | 96,25 | 0,66 | (94,88; 97,63) | 0,17 | 0,08 | 0,08 |
| 7 | 91,14 | 89,53 | 0,76 | (87,96; 91,11) | 1,61 | 0,75 | 0,75 |
| 8 | 94,77 | 95,82 | 0,76 | (94,24; 97,40) | -1,05 | -0,49 | -0,48 |
| 9 | 86,97 | 91,14 | 0,79 | (89,50; 92,77) | -4,16 | -1,95 | -2,09 |
| 10 | 99,59 | 97,42 | 0,79 | (95,79; 99,05) | 2,17 | 1,02 | 1,02 |
| 11 | 94,14 | 90,73 | 0,68 | (89,32; 92,14) | 3,41 | 1,58 | 1,63 |
| 12 | 96,67 | 97,01 | 0,68 | (95,60; 98,43) | -0,34 | -0,16 | -0,16 |
| 13 | 90,12 | 91,35 | 0,86 | (89,56; 93,13) | -1,23 | -0,59 | -0,58 |
| 14 | 95,06 | 97,63 | 0,86 | (95,85; 99,41) | -2,57 | -1,22 | -1,24 |
| 15 | 90,72 | 90,84 | 0,71 | (89,38; 92,30) | -0,12 | -0,06 | -0,05 |
| 16 | 96,90 | 97,13 | 0,71 | (95,67; 98,59) | -0,22 | -0,10 | -0,10 |
| 17 | 87,07 | 85,11 | 1,71 | (81,57; 88,65) | 1,96 | 1,31 | 1,33 |
| 18 | 97,73 | 97,68 | 1,71 | (94,14; 101,22) | 0,05 | 0,03 | 0,03 |
| 19 | 97,92 | 94,30 | 0,60 | (93,07; 95,53) | 3,62 | 1,65 | 1,72 |
| 20 | 93,23 | 93,84 | 0,60 | (92,59; 95,09) | -0,61 | -0,28 | -0,27 |
| 21 | 92,80 | 93,91 | 0,60 | (92,67; 95,14) | -1,11 | -0,51 | -0,50 |
| 22 | 96,33 | 94,22 | 0,59 | (93,00; 95,44) | 2,11 | 0,96 | 0,96 |
| 23 | 93,32 | 92,12 | 1,23 | (89,57; 94,68) | 1,20 | 0,63 | 0,62 |
| 24 | 95,63 | 96,11 | 1,25 | (93,53; 98,69) | -0,48 | -0,25 | -0,25 |
| 25 | 92,29 | 94,05 | 0,59 | (92,83; 95,26) | -1,76 | -0,80 | -0,79 |
| 26 | 95,16 | 94,05 | 0,59 | (92,83; 95,26) | 1,12 | 0,51 | 0,50 |
| 27 | 95,97 | 94,05 | 0,59 | (92,83; 95,26) | 1,93 | 0,88 | 0,87 |

| Obs | HI | Cook’s D | DFITS |  |  |
| --- | --- | --- | --- | --- | --- |
| 1 | 0,092405 | 0,01 | 0,18491 |  |  |
| 2 | 0,092405 | 0,03 | 0,36653 |  |  |
| 3 | 0,154489 | 0,21 | -1,01199 | R |  |
| 4 | 0,154489 | 0,08 | -0,56461 |  |  |
| 5 | 0,085364 | 0,02 | -0,30864 |  |  |
| 6 | 0,085364 | 0,00 | 0,02382 |  |  |
| 7 | 0,112838 | 0,02 | 0,26589 |  |  |
| 8 | 0,112838 | 0,01 | -0,17268 |  |  |
| 9 | 0,120630 | 0,13 | -0,77547 |  |  |
| 10 | 0,120630 | 0,04 | 0,37705 |  |  |
| 11 | 0,090283 | 0,06 | 0,51414 |  |  |
| 12 | 0,090283 | 0,00 | -0,04900 |  |  |
| 13 | 0,143796 | 0,01 | -0,23648 |  |  |
| 14 | 0,143796 | 0,06 | -0,50664 |  |  |
| 15 | 0,096712 | 0,00 | -0,01761 |  |  |
| 16 | 0,096712 | 0,00 | -0,03311 |  |  |
| 17 | 0,566788 | 0,56 | 1,52383 |  | X |
| 18 | 0,566788 | 0,00 | 0,03441 |  | X |
| 19 | 0,068996 | 0,05 | 0,46784 |  |  |
| 20 | 0,070758 | 0,00 | -0,07502 |  |  |
| 21 | 0,068883 | 0,00 | -0,13528 |  |  |
| 22 | 0,067544 | 0,02 | 0,25833 |  |  |
| 23 | 0,295031 | 0,04 | 0,40058 |  |  |
| 24 | 0,301653 | 0,01 | -0,16287 |  |  |
| 25 | 0,066842 | 0,01 | -0,21245 |  |  |
| 26 | 0,066842 | 0,00 | 0,13404 |  |  |
| 27 | 0,066842 | 0,01 | 0,23394 |  |  |

R  Large residual  
X  Unusual X

## Stepwise Selection of Terms

α to enter = 0,15; α to remove = 0,15

## Coded Coefficients

| Term | Coef | SE Coef | 95% CI | T-Value | P-Value | VIF |
| --- | --- | --- | --- | --- | --- | --- |
| Constant | 95,169 | 0,581 | (93,967; 96,371) | 163,75 | 0,000 |  |
| Lac | 5,434 | 0,919 | (3,533; 7,335) | 5,91 | 0,000 | 1,00 |
| HPMC\_PS | 1,89 | 1,08 | (-0,35; 4,13) | 1,74 | 0,094 | 1,00 |
| Lac\*Lac | -2,88 | 1,74 | (-6,49; 0,72) | -1,65 | 0,112 | 1,00 |

## Model Summary

| S | R-sq | R-sq(adj) | PRESS | R-sq(pred) | AICc | BIC |
| --- | --- | --- | --- | --- | --- | --- |
| 2,25077 | 63,93% | 59,23% | 163,884 | 49,27% | 128,96 | 132,58 |

## Analysis of Variance

| Source | DF | Seq SS | Contribution | Adj SS | Adj MS | F-Value | P-Value |
| --- | --- | --- | --- | --- | --- | --- | --- |
| Model | 3 | 206,520 | 63,93% | 206,520 | 68,840 | 13,59 | 0,000 |
| Linear | 2 | 192,678 | 59,65% | 192,593 | 96,297 | 19,01 | 0,000 |
| Lac | 1 | 177,182 | 54,85% | 177,182 | 177,182 | 34,97 | 0,000 |
| HPMC\_PS | 1 | 15,496 | 4,80% | 15,411 | 15,411 | 3,04 | 0,094 |
| Square | 1 | 13,842 | 4,29% | 13,842 | 13,842 | 2,73 | 0,112 |
| Lac\*Lac | 1 | 13,842 | 4,29% | 13,842 | 13,842 | 2,73 | 0,112 |
| Error | 23 | 116,518 | 36,07% | 116,518 | 5,066 |  |  |
| Lack-of-Fit | 21 | 109,671 | 33,95% | 109,671 | 5,222 | 1,53 | 0,470 |
| Pure Error | 2 | 6,846 | 2,12% | 6,846 | 3,423 |  |  |
| Total | 26 | 323,038 | 100,00% |  |  |  |  |

## Regression Equation in Uncoded Units

|  |  |  |
| --- | --- | --- |
| F\_mean\_21h(1260min) | = | 54,9 + 67,8 Lac + 0,257 HPMC\_PS - 46,1 Lac\*Lac |

## Fits and Diagnostics for All Observations

| Obs | F\_mean\_21h(1260min) | Fit | SE Fit | 95% CI | Resid | Std Resid | Del Resid |
| --- | --- | --- | --- | --- | --- | --- | --- |
| 1 | 92,78 | 91,26 | 0,68 | (89,85; 92,68) | 1,51 | 0,70 | 0,70 |
| 2 | 99,08 | 96,70 | 0,68 | (95,28; 98,11) | 2,38 | 1,11 | 1,12 |
| 3 | 86,40 | 90,64 | 0,88 | (88,81; 92,47) | -4,24 | -2,05 | -2,21 |
| 4 | 93,38 | 96,07 | 0,88 | (94,24; 97,90) | -2,69 | -1,30 | -1,32 |
| 5 | 89,11 | 91,41 | 0,66 | (90,05; 92,77) | -2,30 | -1,07 | -1,07 |
| 6 | 96,64 | 96,84 | 0,66 | (95,48; 98,20) | -0,20 | -0,09 | -0,09 |
| 7 | 92,64 | 91,00 | 0,76 | (89,43; 92,56) | 1,65 | 0,78 | 0,77 |
| 8 | 95,76 | 96,43 | 0,76 | (94,87; 97,99) | -0,67 | -0,31 | -0,31 |
| 9 | 88,39 | 92,52 | 0,78 | (90,90; 94,13) | -4,13 | -1,96 | -2,09 |
| 10 | 100,23 | 97,95 | 0,78 | (96,33; 99,57) | 2,28 | 1,08 | 1,09 |
| 11 | 95,69 | 92,13 | 0,68 | (90,73; 93,53) | 3,56 | 1,66 | 1,73 |
| 12 | 97,35 | 97,56 | 0,68 | (96,16; 98,96) | -0,22 | -0,10 | -0,10 |
| 13 | 91,33 | 92,71 | 0,85 | (90,95; 94,48) | -1,38 | -0,66 | -0,66 |
| 14 | 94,95 | 98,15 | 0,85 | (96,38; 99,91) | -3,20 | -1,53 | -1,58 |
| 15 | 91,80 | 92,24 | 0,70 | (90,79; 93,68) | -0,43 | -0,20 | -0,20 |
| 16 | 97,98 | 97,67 | 0,70 | (96,22; 99,12) | 0,31 | 0,14 | 0,14 |
| 17 | 88,70 | 86,79 | 1,69 | (83,28; 90,29) | 1,91 | 1,29 | 1,31 |
| 18 | 97,69 | 97,66 | 1,69 | (94,15; 101,16) | 0,03 | 0,02 | 0,02 |
| 19 | 98,77 | 95,35 | 0,59 | (94,12; 96,57) | 3,43 | 1,58 | 1,63 |
| 20 | 94,24 | 94,90 | 0,60 | (93,67; 96,14) | -0,66 | -0,31 | -0,30 |
| 21 | 94,09 | 94,97 | 0,59 | (93,75; 96,19) | -0,89 | -0,41 | -0,40 |
| 22 | 96,73 | 95,27 | 0,58 | (94,06; 96,48) | 1,46 | 0,67 | 0,66 |
| 23 | 94,12 | 93,28 | 1,22 | (90,75; 95,81) | 0,84 | 0,45 | 0,44 |
| 24 | 96,83 | 97,06 | 1,24 | (94,50; 99,62) | -0,23 | -0,12 | -0,12 |
| 25 | 93,60 | 95,10 | 0,58 | (93,90; 96,31) | -1,50 | -0,69 | -0,68 |
| 26 | 96,59 | 95,10 | 0,58 | (93,90; 96,31) | 1,49 | 0,69 | 0,68 |
| 27 | 96,98 | 95,10 | 0,58 | (93,90; 96,31) | 1,88 | 0,86 | 0,86 |

| Obs | HI | Cook’s D | DFITS |  |  |
| --- | --- | --- | --- | --- | --- |
| 1 | 0,092405 | 0,01 | 0,22230 |  |  |
| 2 | 0,092405 | 0,03 | 0,35651 |  |  |
| 3 | 0,154489 | 0,19 | -0,94604 | R |  |
| 4 | 0,154489 | 0,08 | -0,56492 |  |  |
| 5 | 0,085364 | 0,03 | -0,32711 |  |  |
| 6 | 0,085364 | 0,00 | -0,02757 |  |  |
| 7 | 0,112838 | 0,02 | 0,27454 |  |  |
| 8 | 0,112838 | 0,00 | -0,11002 |  |  |
| 9 | 0,120630 | 0,13 | -0,77562 |  |  |
| 10 | 0,120630 | 0,04 | 0,40217 |  |  |
| 11 | 0,090283 | 0,07 | 0,54466 |  |  |
| 12 | 0,090283 | 0,00 | -0,03138 |  |  |
| 13 | 0,143796 | 0,02 | -0,26911 |  |  |
| 14 | 0,143796 | 0,10 | -0,64922 |  |  |
| 15 | 0,096712 | 0,00 | -0,06489 |  |  |
| 16 | 0,096712 | 0,00 | 0,04624 |  |  |
| 17 | 0,566788 | 0,54 | 1,49764 |  | X |
| 18 | 0,566788 | 0,00 | 0,02197 |  | X |
| 19 | 0,068996 | 0,05 | 0,44469 |  |  |
| 20 | 0,070758 | 0,00 | -0,08265 |  |  |
| 21 | 0,068883 | 0,00 | -0,10880 |  |  |
| 22 | 0,067544 | 0,01 | 0,17884 |  |  |
| 23 | 0,295031 | 0,02 | 0,28296 |  |  |
| 24 | 0,301653 | 0,00 | -0,07786 |  |  |
| 25 | 0,066842 | 0,01 | -0,18285 |  |  |
| 26 | 0,066842 | 0,01 | 0,18119 |  |  |
| 27 | 0,066842 | 0,01 | 0,22987 |  |  |

R  Large residual  
X  Unusual X

## Stepwise Selection of Terms

α to enter = 0,15; α to remove = 0,15

## Coded Coefficients

| Term | Coef | SE Coef | 95% CI | T-Value | P-Value | VIF |
| --- | --- | --- | --- | --- | --- | --- |
| Constant | 96,009 | 0,573 | (94,824; 97,194) | 167,57 | 0,000 |  |
| Lac | 4,528 | 0,906 | (2,654; 6,402) | 5,00 | 0,000 | 1,00 |
| HPMC\_PS | 1,78 | 1,07 | (-0,43; 3,99) | 1,67 | 0,109 | 1,00 |
| Lac\*Lac | -3,09 | 1,72 | (-6,65; 0,46) | -1,80 | 0,085 | 1,00 |

## Model Summary

| S | R-sq | R-sq(adj) | PRESS | R-sq(pred) | AICc | BIC |
| --- | --- | --- | --- | --- | --- | --- |
| 2,21884 | 57,43% | 51,88% | 158,258 | 40,50% | 128,19 | 131,81 |

## Analysis of Variance

| Source | DF | Seq SS | Contribution | Adj SS | Adj MS | F-Value | P-Value |
| --- | --- | --- | --- | --- | --- | --- | --- |
| Model | 3 | 152,754 | 57,43% | 152,754 | 50,918 | 10,34 | 0,000 |
| Linear | 2 | 136,798 | 51,43% | 136,712 | 68,356 | 13,88 | 0,000 |
| Lac | 1 | 123,030 | 46,25% | 123,030 | 123,030 | 24,99 | 0,000 |
| HPMC\_PS | 1 | 13,768 | 5,18% | 13,682 | 13,682 | 2,78 | 0,109 |
| Square | 1 | 15,956 | 6,00% | 15,956 | 15,956 | 3,24 | 0,085 |
| Lac\*Lac | 1 | 15,956 | 6,00% | 15,956 | 15,956 | 3,24 | 0,085 |
| Error | 23 | 113,234 | 42,57% | 113,234 | 4,923 |  |  |
| Lack-of-Fit | 21 | 107,122 | 40,27% | 107,122 | 5,101 | 1,67 | 0,442 |
| Pure Error | 2 | 6,112 | 2,30% | 6,112 | 3,056 |  |  |
| Total | 26 | 265,988 | 100,00% |  |  |  |  |

## Regression Equation in Uncoded Units

|  |  |  |
| --- | --- | --- |
| F\_mean\_22h(1320min) | = | 57,7 + 67,6 Lac + 0,242 HPMC\_PS - 49,5 Lac\*Lac |

## Fits and Diagnostics for All Observations

| Obs | F\_mean\_22h(1320min) | Fit | SE Fit | 95% CI | Resid | Std Resid | Del Resid |
| --- | --- | --- | --- | --- | --- | --- | --- |
| 1 | 94,06 | 92,53 | 0,67 | (91,14; 93,93) | 1,52 | 0,72 | 0,71 |
| 2 | 99,13 | 97,06 | 0,67 | (95,66; 98,45) | 2,07 | 0,98 | 0,98 |
| 3 | 87,98 | 91,94 | 0,87 | (90,14; 93,75) | -3,96 | -1,94 | -2,07 |
| 4 | 93,86 | 96,47 | 0,87 | (94,67; 98,27) | -2,61 | -1,28 | -1,30 |
| 5 | 90,34 | 92,67 | 0,65 | (91,32; 94,01) | -2,32 | -1,09 | -1,10 |
| 6 | 96,86 | 97,19 | 0,65 | (95,85; 98,53) | -0,34 | -0,16 | -0,15 |
| 7 | 93,92 | 92,28 | 0,75 | (90,74; 93,82) | 1,64 | 0,79 | 0,78 |
| 8 | 96,34 | 96,81 | 0,75 | (95,26; 98,35) | -0,47 | -0,22 | -0,22 |
| 9 | 89,63 | 93,71 | 0,77 | (92,12; 95,30) | -4,08 | -1,96 | -2,10 |
| 10 | 100,47 | 98,24 | 0,77 | (96,64; 99,83) | 2,23 | 1,07 | 1,08 |
| 11 | 97,02 | 93,35 | 0,67 | (91,97; 94,73) | 3,67 | 1,73 | 1,82 |
| 12 | 97,61 | 97,87 | 0,67 | (96,50; 99,25) | -0,27 | -0,13 | -0,12 |
| 13 | 92,37 | 93,90 | 0,84 | (92,16; 95,64) | -1,53 | -0,74 | -0,74 |
| 14 | 94,72 | 98,43 | 0,84 | (96,68; 100,17) | -3,71 | -1,81 | -1,91 |
| 15 | 92,90 | 93,45 | 0,69 | (92,02; 94,87) | -0,54 | -0,26 | -0,25 |
| 16 | 98,69 | 97,97 | 0,69 | (96,55; 99,40) | 0,72 | 0,34 | 0,33 |
| 17 | 90,13 | 88,33 | 1,67 | (84,87; 91,78) | 1,80 | 1,23 | 1,25 |
| 18 | 97,57 | 97,38 | 1,67 | (93,93; 100,84) | 0,19 | 0,13 | 0,13 |
| 19 | 99,38 | 96,18 | 0,58 | (94,97; 97,38) | 3,21 | 1,50 | 1,54 |
| 20 | 95,13 | 95,76 | 0,59 | (94,54; 96,98) | -0,63 | -0,30 | -0,29 |
| 21 | 95,20 | 95,82 | 0,58 | (94,62; 97,03) | -0,62 | -0,29 | -0,29 |
| 22 | 96,94 | 96,11 | 0,58 | (94,91; 97,30) | 0,84 | 0,39 | 0,38 |
| 23 | 94,80 | 94,23 | 1,21 | (91,74; 96,72) | 0,57 | 0,31 | 0,30 |
| 24 | 97,84 | 97,79 | 1,22 | (95,27; 100,31) | 0,05 | 0,03 | 0,02 |
| 25 | 94,78 | 95,95 | 0,57 | (94,76; 97,13) | -1,16 | -0,54 | -0,53 |
| 26 | 97,80 | 95,95 | 0,57 | (94,76; 97,13) | 1,85 | 0,86 | 0,86 |
| 27 | 97,82 | 95,95 | 0,57 | (94,76; 97,13) | 1,88 | 0,88 | 0,87 |

| Obs | HI | Cook’s D | DFITS |  |
| --- | --- | --- | --- | --- |
| 1 | 0,092405 | 0,01 | 0,22763 |  |
| 2 | 0,092405 | 0,02 | 0,31247 |  |
| 3 | 0,154489 | 0,17 | -0,88651 |  |
| 4 | 0,154489 | 0,07 | -0,55458 |  |
| 5 | 0,085364 | 0,03 | -0,33582 |  |
| 6 | 0,085364 | 0,00 | -0,04730 |  |
| 7 | 0,112838 | 0,02 | 0,27778 |  |
| 8 | 0,112838 | 0,00 | -0,07806 |  |
| 9 | 0,120630 | 0,13 | -0,77844 |  |
| 10 | 0,120630 | 0,04 | 0,39860 |  |
| 11 | 0,090283 | 0,07 | 0,57284 |  |
| 12 | 0,090283 | 0,00 | -0,03922 |  |
| 13 | 0,143796 | 0,02 | -0,30183 |  |
| 14 | 0,143796 | 0,14 | -0,78093 |  |
| 15 | 0,096712 | 0,00 | -0,08266 |  |
| 16 | 0,096712 | 0,00 | 0,10907 |  |
| 17 | 0,566788 | 0,50 | 1,42899 | X |
| 18 | 0,566788 | 0,01 | 0,14402 | X |
| 19 | 0,068996 | 0,04 | 0,41988 |  |
| 20 | 0,070758 | 0,00 | -0,08007 |  |
| 21 | 0,068883 | 0,00 | -0,07754 |  |
| 22 | 0,067544 | 0,00 | 0,10303 |  |
| 23 | 0,295031 | 0,01 | 0,19404 |  |
| 24 | 0,301653 | 0,00 | 0,01634 |  |
| 25 | 0,066842 | 0,01 | -0,14290 |  |
| 26 | 0,066842 | 0,01 | 0,23009 |  |
| 27 | 0,066842 | 0,01 | 0,23315 |  |

X  Unusual X

## Stepwise Selection of Terms

α to enter = 0,15; α to remove = 0,15

## Coded Coefficients

| Term | Coef | SE Coef | 95% CI | T-Value | P-Value | VIF |
| --- | --- | --- | --- | --- | --- | --- |
| Constant | 96,653 | 0,564 | (95,487; 97,820) | 171,39 | 0,000 |  |
| Lac | 3,662 | 0,892 | (1,818; 5,506) | 4,11 | 0,000 | 1,00 |
| HPMC\_PS | 1,73 | 1,05 | (-0,44; 3,90) | 1,65 | 0,113 | 1,00 |
| Lac\*Lac | -3,24 | 1,69 | (-6,73; 0,26) | -1,91 | 0,068 | 1,00 |

## Model Summary

| S | R-sq | R-sq(adj) | PRESS | R-sq(pred) | AICc | BIC |
| --- | --- | --- | --- | --- | --- | --- |
| 2,18388 | 50,28% | 43,79% | 152,765 | 30,75% | 127,33 | 130,95 |

## Analysis of Variance

| Source | DF | Seq SS | Contribution | Adj SS | Adj MS | F-Value | P-Value |
| --- | --- | --- | --- | --- | --- | --- | --- |
| Model | 3 | 110,909 | 50,28% | 110,909 | 36,970 | 7,75 | 0,001 |
| Linear | 2 | 93,463 | 42,37% | 93,376 | 46,688 | 9,79 | 0,001 |
| Lac | 1 | 80,459 | 36,47% | 80,459 | 80,459 | 16,87 | 0,000 |
| HPMC\_PS | 1 | 13,004 | 5,89% | 12,917 | 12,917 | 2,71 | 0,113 |
| Square | 1 | 17,446 | 7,91% | 17,446 | 17,446 | 3,66 | 0,068 |
| Lac\*Lac | 1 | 17,446 | 7,91% | 17,446 | 17,446 | 3,66 | 0,068 |
| Error | 23 | 109,695 | 49,72% | 109,695 | 4,769 |  |  |
| Lack-of-Fit | 21 | 104,502 | 47,37% | 104,502 | 4,976 | 1,92 | 0,399 |
| Pure Error | 2 | 5,193 | 2,35% | 5,193 | 2,596 |  |  |
| Total | 26 | 220,604 | 100,00% |  |  |  |  |

## Regression Equation in Uncoded Units

|  |  |  |
| --- | --- | --- |
| F\_mean\_23h(1380min) | = | 60,0 + 66,4 Lac + 0,235 HPMC\_PS - 51,8 Lac\*Lac |

## Fits and Diagnostics for All Observations

| Obs | F\_mean\_23h(1380min) | Fit | SE Fit | 95% CI | Resid | Std Resid | Del Resid |
| --- | --- | --- | --- | --- | --- | --- | --- |
| 1 | 94,91 | 93,59 | 0,66 | (92,21; 94,96) | 1,33 | 0,64 | 0,63 |
| 2 | 99,06 | 97,25 | 0,66 | (95,87; 98,62) | 1,81 | 0,87 | 0,87 |
| 3 | 89,49 | 93,01 | 0,86 | (91,24; 94,79) | -3,52 | -1,75 | -1,84 |
| 4 | 94,10 | 96,67 | 0,86 | (94,90; 98,45) | -2,57 | -1,28 | -1,30 |
| 5 | 91,57 | 93,72 | 0,64 | (92,40; 95,04) | -2,14 | -1,03 | -1,03 |
| 6 | 96,95 | 97,38 | 0,64 | (96,06; 98,70) | -0,43 | -0,20 | -0,20 |
| 7 | 94,89 | 93,34 | 0,73 | (91,82; 94,86) | 1,55 | 0,75 | 0,75 |
| 8 | 96,68 | 97,00 | 0,73 | (95,48; 98,52) | -0,32 | -0,15 | -0,15 |
| 9 | 90,50 | 94,73 | 0,76 | (93,16; 96,30) | -4,23 | -2,06 | -2,24 |
| 10 | 100,49 | 98,39 | 0,76 | (96,82; 99,96) | 2,10 | 1,02 | 1,02 |
| 11 | 98,17 | 94,38 | 0,66 | (93,02; 95,74) | 3,79 | 1,82 | 1,92 |
| 12 | 97,60 | 98,04 | 0,66 | (96,68; 99,40) | -0,44 | -0,21 | -0,21 |
| 13 | 93,21 | 94,91 | 0,83 | (93,20; 96,63) | -1,70 | -0,84 | -0,84 |
| 14 | 94,53 | 98,57 | 0,83 | (96,86; 100,29) | -4,04 | -2,00 | -2,15 |
| 15 | 93,93 | 94,48 | 0,68 | (93,07; 95,88) | -0,55 | -0,26 | -0,26 |
| 16 | 99,12 | 98,14 | 0,68 | (96,73; 99,54) | 0,98 | 0,47 | 0,46 |
| 17 | 91,38 | 89,70 | 1,64 | (86,29; 93,10) | 1,69 | 1,17 | 1,18 |
| 18 | 97,43 | 97,02 | 1,64 | (93,62; 100,42) | 0,41 | 0,28 | 0,28 |
| 19 | 99,71 | 96,81 | 0,57 | (95,63; 98,00) | 2,89 | 1,37 | 1,40 |
| 20 | 95,79 | 96,41 | 0,58 | (95,21; 97,61) | -0,62 | -0,29 | -0,29 |
| 21 | 96,19 | 96,47 | 0,57 | (95,29; 97,66) | -0,28 | -0,13 | -0,13 |
| 22 | 97,12 | 96,75 | 0,57 | (95,57; 97,92) | 0,38 | 0,18 | 0,18 |
| 23 | 95,16 | 94,92 | 1,19 | (92,47; 97,38) | 0,24 | 0,13 | 0,13 |
| 24 | 98,74 | 98,38 | 1,20 | (95,90; 100,86) | 0,36 | 0,20 | 0,19 |
| 25 | 95,85 | 96,59 | 0,56 | (95,43; 97,76) | -0,75 | -0,35 | -0,35 |
| 26 | 98,74 | 96,59 | 0,56 | (95,43; 97,76) | 2,14 | 1,02 | 1,02 |
| 27 | 98,52 | 96,59 | 0,56 | (95,43; 97,76) | 1,93 | 0,92 | 0,91 |

| Obs | HI | Cook’s D | DFITS |  |  |
| --- | --- | --- | --- | --- | --- |
| 1 | 0,092405 | 0,01 | 0,20072 |  |  |
| 2 | 0,092405 | 0,02 | 0,27640 |  |  |
| 3 | 0,154489 | 0,14 | -0,78813 |  |  |
| 4 | 0,154489 | 0,08 | -0,55632 |  |  |
| 5 | 0,085364 | 0,02 | -0,31361 |  |  |
| 6 | 0,085364 | 0,00 | -0,06114 |  |  |
| 7 | 0,112838 | 0,02 | 0,26631 |  |  |
| 8 | 0,112838 | 0,00 | -0,05376 |  |  |
| 9 | 0,120630 | 0,15 | -0,82834 | R |  |
| 10 | 0,120630 | 0,04 | 0,37939 |  |  |
| 11 | 0,090283 | 0,08 | 0,60552 |  |  |
| 12 | 0,090283 | 0,00 | -0,06529 |  |  |
| 13 | 0,143796 | 0,03 | -0,34253 |  |  |
| 14 | 0,143796 | 0,17 | -0,88196 |  |  |
| 15 | 0,096712 | 0,00 | -0,08426 |  |  |
| 16 | 0,096712 | 0,01 | 0,15160 |  |  |
| 17 | 0,566788 | 0,45 | 1,35481 |  | X |
| 18 | 0,566788 | 0,03 | 0,31899 |  | X |
| 19 | 0,068996 | 0,03 | 0,38118 |  |  |
| 20 | 0,070758 | 0,00 | -0,07969 |  |  |
| 21 | 0,068883 | 0,00 | -0,03591 |  |  |
| 22 | 0,067544 | 0,00 | 0,04711 |  |  |
| 23 | 0,295031 | 0,00 | 0,08342 |  |  |
| 24 | 0,301653 | 0,00 | 0,12660 |  |  |
| 25 | 0,066842 | 0,00 | -0,09301 |  |  |
| 26 | 0,066842 | 0,02 | 0,27219 |  |  |
| 27 | 0,066842 | 0,01 | 0,24403 |  |  |

R  Large residual  
X  Unusual X

## Stepwise Selection of Terms

α to enter = 0,15; α to remove = 0,15

## Coded Coefficients

| Term | Coef | SE Coef | 95% CI | T-Value | P-Value | VIF |
| --- | --- | --- | --- | --- | --- | --- |
| Constant | 97,172 | 0,563 | (96,007; 98,337) | 172,52 | 0,000 |  |
| Lac | 2,878 | 0,890 | (1,036; 4,721) | 3,23 | 0,004 | 1,00 |
| HPMC\_PS | 1,70 | 1,05 | (-0,48; 3,87) | 1,61 | 0,120 | 1,00 |
| Lac\*Lac | -3,36 | 1,69 | (-6,85; 0,14) | -1,99 | 0,059 | 1,00 |

## Model Summary

| S | R-sq | R-sq(adj) | PRESS | R-sq(pred) | AICc | BIC |
| --- | --- | --- | --- | --- | --- | --- |
| 2,18121 | 42,54% | 35,04% | 152,131 | 20,11% | 127,26 | 130,89 |

## Analysis of Variance

| Source | DF | Seq SS | Contribution | Adj SS | Adj MS | F-Value | P-Value |
| --- | --- | --- | --- | --- | --- | --- | --- |
| Model | 3 | 81,006 | 42,54% | 81,006 | 27,002 | 5,68 | 0,005 |
| Linear | 2 | 62,209 | 32,67% | 62,120 | 31,060 | 6,53 | 0,006 |
| Lac | 1 | 49,714 | 26,11% | 49,714 | 49,714 | 10,45 | 0,004 |
| HPMC\_PS | 1 | 12,495 | 6,56% | 12,406 | 12,406 | 2,61 | 0,120 |
| Square | 1 | 18,797 | 9,87% | 18,797 | 18,797 | 3,95 | 0,059 |
| Lac\*Lac | 1 | 18,797 | 9,87% | 18,797 | 18,797 | 3,95 | 0,059 |
| Error | 23 | 109,427 | 57,46% | 109,427 | 4,758 |  |  |
| Lack-of-Fit | 21 | 105,563 | 55,43% | 105,563 | 5,027 | 2,60 | 0,314 |
| Pure Error | 2 | 3,864 | 2,03% | 3,864 | 1,932 |  |  |
| Total | 26 | 190,433 | 100,00% |  |  |  |  |

## Regression Equation in Uncoded Units

|  |  |  |
| --- | --- | --- |
| F\_mean\_24h(1440min) | = | 61,9 + 65,2 Lac + 0,230 HPMC\_PS - 53,7 Lac\*Lac |

## Fits and Diagnostics for All Observations

| Obs | F\_mean\_24h(1440min) | Fit | SE Fit | 95% CI | Resid | Std Resid | Del Resid |
| --- | --- | --- | --- | --- | --- | --- | --- |
| 1 | 95,53 | 94,47 | 0,66 | (93,10; 95,85) | 1,06 | 0,51 | 0,50 |
| 2 | 98,99 | 97,35 | 0,66 | (95,98; 98,72) | 1,63 | 0,79 | 0,78 |
| 3 | 90,80 | 93,91 | 0,86 | (92,14; 95,69) | -3,12 | -1,55 | -1,61 |
| 4 | 94,17 | 96,79 | 0,86 | (95,02; 98,56) | -2,62 | -1,31 | -1,33 |
| 5 | 92,51 | 94,60 | 0,64 | (93,28; 95,92) | -2,09 | -1,00 | -1,00 |
| 6 | 97,09 | 97,48 | 0,64 | (96,16; 98,80) | -0,39 | -0,19 | -0,18 |
| 7 | 95,86 | 94,23 | 0,73 | (92,72; 95,75) | 1,63 | 0,79 | 0,78 |
| 8 | 96,74 | 97,11 | 0,73 | (95,60; 98,63) | -0,37 | -0,18 | -0,18 |
| 9 | 91,28 | 95,60 | 0,76 | (94,03; 97,16) | -4,32 | -2,11 | -2,30 |
| 10 | 100,51 | 98,48 | 0,76 | (96,91; 100,04) | 2,03 | 0,99 | 0,99 |
| 11 | 99,12 | 95,25 | 0,66 | (93,89; 96,61) | 3,87 | 1,86 | 1,97 |
| 12 | 97,59 | 98,13 | 0,66 | (96,77; 99,49) | -0,54 | -0,26 | -0,25 |
| 13 | 93,88 | 95,77 | 0,83 | (94,06; 97,49) | -1,89 | -0,94 | -0,93 |
| 14 | 94,32 | 98,65 | 0,83 | (96,94; 100,36) | -4,33 | -2,14 | -2,35 |
| 15 | 94,86 | 95,35 | 0,68 | (93,94; 96,75) | -0,49 | -0,24 | -0,23 |
| 16 | 99,27 | 98,22 | 0,68 | (96,82; 99,63) | 1,05 | 0,51 | 0,50 |
| 17 | 92,44 | 90,88 | 1,64 | (87,48; 94,27) | 1,57 | 1,09 | 1,10 |
| 18 | 97,29 | 96,63 | 1,64 | (93,24; 100,03) | 0,65 | 0,46 | 0,45 |
| 19 | 99,95 | 97,33 | 0,57 | (96,14; 98,52) | 2,62 | 1,25 | 1,26 |
| 20 | 96,30 | 96,93 | 0,58 | (95,73; 98,14) | -0,63 | -0,30 | -0,30 |
| 21 | 97,03 | 96,99 | 0,57 | (95,81; 98,18) | 0,03 | 0,02 | 0,02 |
| 22 | 97,28 | 97,26 | 0,57 | (96,09; 98,44) | 0,02 | 0,01 | 0,01 |
| 23 | 95,41 | 95,48 | 1,18 | (93,03; 97,93) | -0,07 | -0,04 | -0,04 |
| 24 | 99,43 | 98,87 | 1,20 | (96,39; 101,35) | 0,56 | 0,31 | 0,30 |
| 25 | 96,90 | 97,11 | 0,56 | (95,95; 98,28) | -0,21 | -0,10 | -0,10 |
| 26 | 99,48 | 97,11 | 0,56 | (95,95; 98,28) | 2,36 | 1,12 | 1,13 |
| 27 | 99,10 | 97,11 | 0,56 | (95,95; 98,28) | 1,99 | 0,94 | 0,94 |

| Obs | HI | Cook’s D | DFITS |  |  |
| --- | --- | --- | --- | --- | --- |
| 1 | 0,092405 | 0,01 | 0,15957 |  |  |
| 2 | 0,092405 | 0,02 | 0,24881 |  |  |
| 3 | 0,154489 | 0,11 | -0,68666 |  |  |
| 4 | 0,154489 | 0,08 | -0,56817 |  |  |
| 5 | 0,085364 | 0,02 | -0,30630 |  |  |
| 6 | 0,085364 | 0,00 | -0,05571 |  |  |
| 7 | 0,112838 | 0,02 | 0,27993 |  |  |
| 8 | 0,112838 | 0,00 | -0,06260 |  |  |
| 9 | 0,120630 | 0,15 | -0,85164 | R |  |
| 10 | 0,120630 | 0,03 | 0,36762 |  |  |
| 11 | 0,090283 | 0,09 | 0,62172 |  |  |
| 12 | 0,090283 | 0,00 | -0,07977 |  |  |
| 13 | 0,143796 | 0,04 | -0,38307 |  |  |
| 14 | 0,143796 | 0,19 | -0,96104 | R |  |
| 15 | 0,096712 | 0,00 | -0,07575 |  |  |
| 16 | 0,096712 | 0,01 | 0,16289 |  |  |
| 17 | 0,566788 | 0,39 | 1,25383 |  | X |
| 18 | 0,566788 | 0,07 | 0,51265 |  | X |
| 19 | 0,068996 | 0,03 | 0,34373 |  |  |
| 20 | 0,070758 | 0,00 | -0,08144 |  |  |
| 21 | 0,068883 | 0,00 | 0,00429 |  |  |
| 22 | 0,067544 | 0,00 | 0,00215 |  |  |
| 23 | 0,295031 | 0,00 | -0,02468 |  |  |
| 24 | 0,301653 | 0,01 | 0,19745 |  |  |
| 25 | 0,066842 | 0,00 | -0,02626 |  |  |
| 26 | 0,066842 | 0,02 | 0,30187 |  |  |
| 27 | 0,066842 | 0,02 | 0,25151 |  |  |

R  Large residual  
X  Unusual X
