# Supplementary material for: Influence of Selected Hypromellose Functionality-Related Characteristics and Soluble/Insoluble Filler Ratio on Carvedilol Release from Matrix Tablets
Source: Pharmaceutics. 2025 Oct 21;17(10):1358. doi: 10.3390/pharmaceutics17101358 (PMC12566823; doi:10.3390/pharmaceutics17101358)
Supplement: Supplementary file 1 [file pharmaceutics-17-01358-s001.zip › Report_SD of Release Analysis_RSM(CCD)_Full model.htm]

# SD of Release Analysis, Response Surface Design (Central Composite Design), Full model

## Coded Coefficients

| Term | Coef | SE Coef | 95% CI | T-Value | P-Value | VIF |
| --- | --- | --- | --- | --- | --- | --- |
| Constant | 1,563 | 0,481 | (0,514; 2,612) | 3,25 | 0,007 |  |
| Lac | 1,082 | 0,398 | (0,215; 1,949) | 2,72 | 0,019 | 1,18 |
| HPMC\_Visc | -0,480 | 0,416 | (-1,386; 0,426) | -1,15 | 0,271 | 1,70 |
| HPMC\_HP | 0,049 | 0,407 | (-0,837; 0,936) | 0,12 | 0,906 | 1,26 |
| HPMC\_PS | -0,181 | 0,624 | (-1,541; 1,179) | -0,29 | 0,777 | 2,09 |
| Lac\*Lac | 1,000 | 0,791 | (-0,724; 2,723) | 1,26 | 0,230 | 1,30 |
| HPMC\_Visc\*HPMC\_Visc | 0,258 | 0,841 | (-1,574; 2,090) | 0,31 | 0,764 | 1,96 |
| HPMC\_HP\*HPMC\_HP | -0,376 | 0,798 | (-2,114; 1,363) | -0,47 | 0,646 | 1,81 |
| HPMC\_PS\*HPMC\_PS | -0,596 | 0,793 | (-2,324; 1,131) | -0,75 | 0,467 | 1,42 |
| Lac\*HPMC\_Visc | -1,626 | 0,872 | (-3,527; 0,275) | -1,86 | 0,087 | 1,49 |
| Lac\*HPMC\_HP | 0,716 | 0,916 | (-1,279; 2,711) | 0,78 | 0,449 | 1,17 |
| Lac\*HPMC\_PS | -0,78 | 1,41 | (-3,86; 2,30) | -0,55 | 0,591 | 1,42 |
| HPMC\_Visc\*HPMC\_HP | -0,60 | 1,04 | (-2,87; 1,67) | -0,57 | 0,577 | 2,73 |
| HPMC\_Visc\*HPMC\_PS | 0,81 | 1,47 | (-2,40; 4,01) | 0,55 | 0,593 | 2,74 |
| HPMC\_HP\*HPMC\_PS | -0,91 | 1,62 | (-4,45; 2,63) | -0,56 | 0,585 | 2,70 |

## Model Summary

| S | R-sq | R-sq(adj) | PRESS | R-sq(pred) | AICc | BIC |
| --- | --- | --- | --- | --- | --- | --- |
| 0,896138 | 65,86% | 26,02% | 35,4869 | 0,00% | 135,21 | 101,54 |

## Analysis of Variance

| Source | DF | Seq SS | Contribution | Adj SS | Adj MS | F-Value | P-Value |
| --- | --- | --- | --- | --- | --- | --- | --- |
| Model | 14 | 18,5884 | 65,86% | 18,5884 | 1,32774 | 1,65 | 0,194 |
| Linear | 4 | 11,0095 | 39,01% | 7,1734 | 1,79336 | 2,23 | 0,126 |
| Lac | 1 | 8,8741 | 31,44% | 5,9368 | 5,93676 | 7,39 | 0,019 |
| HPMC\_Visc | 1 | 1,6796 | 5,95% | 1,0683 | 1,06833 | 1,33 | 0,271 |
| HPMC\_HP | 1 | 0,3130 | 1,11% | 0,0117 | 0,01168 | 0,01 | 0,906 |
| HPMC\_PS | 1 | 0,1428 | 0,51% | 0,0676 | 0,06765 | 0,08 | 0,777 |
| Square | 4 | 3,9178 | 13,88% | 3,2317 | 0,80792 | 1,01 | 0,442 |
| Lac\*Lac | 1 | 2,2673 | 8,03% | 1,2826 | 1,28264 | 1,60 | 0,230 |
| HPMC\_Visc\*HPMC\_Visc | 1 | 0,0033 | 0,01% | 0,0755 | 0,07555 | 0,09 | 0,764 |
| HPMC\_HP\*HPMC\_HP | 1 | 0,6337 | 2,25% | 0,1780 | 0,17800 | 0,22 | 0,646 |
| HPMC\_PS\*HPMC\_PS | 1 | 1,0136 | 3,59% | 0,4542 | 0,45416 | 0,57 | 0,467 |
| 2-Way Interaction | 6 | 3,6610 | 12,97% | 3,6610 | 0,61017 | 0,76 | 0,615 |
| Lac\*HPMC\_Visc | 1 | 2,6155 | 9,27% | 2,7877 | 2,78770 | 3,47 | 0,087 |
| Lac\*HPMC\_HP | 1 | 0,3601 | 1,28% | 0,4912 | 0,49116 | 0,61 | 0,449 |
| Lac\*HPMC\_PS | 1 | 0,2452 | 0,87% | 0,2452 | 0,24522 | 0,31 | 0,591 |
| HPMC\_Visc\*HPMC\_HP | 1 | 0,0079 | 0,03% | 0,2638 | 0,26385 | 0,33 | 0,577 |
| HPMC\_Visc\*HPMC\_PS | 1 | 0,1790 | 0,63% | 0,2415 | 0,24153 | 0,30 | 0,593 |
| HPMC\_HP\*HPMC\_PS | 1 | 0,2532 | 0,90% | 0,2532 | 0,25324 | 0,32 | 0,585 |
| Error | 12 | 9,6368 | 34,14% | 9,6368 | 0,80306 |  |  |
| Lack-of-Fit | 10 | 7,0382 | 24,94% | 7,0382 | 0,70382 | 0,54 | 0,792 |
| Pure Error | 2 | 2,5985 | 9,21% | 2,5985 | 1,29927 |  |  |
| Total | 26 | 28,2252 | 100,00% |  |  |  |  |

## Regression Equation in Uncoded Units

|  |  |  |
| --- | --- | --- |
| F\_SD\_0.17h(10min) | = | -163 + 13,8 Lac - 0,00028 HPMC\_Visc + 16,1 HPMC\_HP + 2,50 HPMC\_PS + 16,0 Lac\*Lac + 0,000000 HPMC\_Visc\*HPMC\_Visc - 0,365 HPMC\_HP\*HPMC\_HP - 0,0110 HPMC\_PS\*HPMC\_PS - 0,001672 Lac\*HPMC\_Visc + 2,82 Lac\*HPMC\_HP - 0,425 Lac\*HPMC\_PS - 0,000151 HPMC\_Visc\*HPMC\_HP + 0,000028 HPMC\_Visc\*HPMC\_PS - 0,122 HPMC\_HP\*HPMC\_PS |

## Fits and Diagnostics for All Observations

| Obs | F\_SD\_0.17h(10min) | Fit | SE Fit | 95% CI | Resid | Std Resid | Del Resid |
| --- | --- | --- | --- | --- | --- | --- | --- |
| 1 | 1,134 | 0,778 | 0,726 | (-0,804; 2,359) | 0,357 | 0,68 | 0,66 |
| 2 | 2,346 | 2,694 | 0,726 | (1,113; 4,275) | -0,348 | -0,66 | -0,65 |
| 3 | 0,741 | 0,914 | 0,766 | (-0,754; 2,583) | -0,173 | -0,37 | -0,36 |
| 4 | 0,785 | 0,789 | 0,766 | (-0,879; 2,457) | -0,004 | -0,01 | -0,01 |
| 5 | 0,860 | 1,233 | 0,619 | (-0,115; 2,581) | -0,373 | -0,58 | -0,56 |
| 6 | 5,070 | 3,652 | 0,619 | (2,304; 5,000) | 1,418 | 2,19 | 2,70 |
| 7 | 0,912 | 0,979 | 0,744 | (-0,641; 2,600) | -0,067 | -0,13 | -0,13 |
| 8 | 1,271 | 1,551 | 0,744 | (-0,070; 3,171) | -0,280 | -0,56 | -0,54 |
| 9 | 1,144 | 0,930 | 0,683 | (-0,558; 2,418) | 0,214 | 0,37 | 0,35 |
| 10 | 2,506 | 2,326 | 0,683 | (0,838; 3,815) | 0,179 | 0,31 | 0,30 |
| 11 | 0,245 | 1,602 | 0,585 | (0,328; 2,876) | -1,357 | -2,00 | -2,34 |
| 12 | 1,852 | 1,869 | 0,585 | (0,595; 3,143) | -0,018 | -0,03 | -0,02 |
| 13 | 0,750 | 0,607 | 0,676 | (-0,866; 2,080) | 0,143 | 0,24 | 0,23 |
| 14 | 2,649 | 2,715 | 0,676 | (1,242; 4,188) | -0,067 | -0,11 | -0,11 |
| 15 | 1,393 | 1,325 | 0,590 | (0,040; 2,610) | 0,068 | 0,10 | 0,10 |
| 16 | 1,650 | 1,863 | 0,590 | (0,578; 3,149) | -0,213 | -0,32 | -0,30 |
| 17 | 1,815 | 1,285 | 0,693 | (-0,224; 2,795) | 0,529 | 0,93 | 0,93 |
| 18 | 3,638 | 4,037 | 0,693 | (2,527; 5,546) | -0,399 | -0,70 | -0,69 |
| 19 | 1,034 | 2,160 | 0,572 | (0,914; 3,405) | -1,126 | -1,63 | -1,77 |
| 20 | 2,043 | 1,303 | 0,721 | (-0,268; 2,874) | 0,740 | 1,39 | 1,45 |
| 21 | 1,406 | 1,057 | 0,572 | (-0,190; 2,304) | 0,348 | 0,50 | 0,49 |
| 22 | 1,178 | 1,298 | 0,810 | (-0,467; 3,063) | -0,120 | -0,31 | -0,30 |
| 23 | 1,271 | 1,415 | 0,813 | (-0,357; 3,186) | -0,144 | -0,38 | -0,37 |
| 24 | 1,024 | 0,826 | 0,757 | (-0,824; 2,475) | 0,199 | 0,41 | 0,40 |
| 25 | 0,676 | 1,661 | 0,482 | (0,612; 2,711) | -0,985 | -1,30 | -1,35 |
| 26 | 1,846 | 1,661 | 0,482 | (0,612; 2,711) | 0,185 | 0,24 | 0,23 |
| 27 | 2,956 | 1,661 | 0,482 | (0,612; 2,711) | 1,295 | 1,71 | 1,89 |

| Obs | HI | Cook’s D | DFITS |  |
| --- | --- | --- | --- | --- |
| 1 | 0,655994 | 0,06 | 0,91523 |  |
| 2 | 0,655994 | 0,06 | -0,89200 |  |
| 3 | 0,729945 | 0,02 | -0,58966 |  |
| 4 | 0,729945 | 0,00 | -0,01517 |  |
| 5 | 0,476813 | 0,02 | -0,53323 |  |
| 6 | 0,476813 | 0,29 | 2,57838 | R |
| 7 | 0,688750 | 0,00 | -0,19230 |  |
| 8 | 0,688750 | 0,05 | -0,80859 |  |
| 9 | 0,580949 | 0,01 | 0,41768 |  |
| 10 | 0,580949 | 0,01 | 0,35001 |  |
| 11 | 0,425781 | 0,20 | -2,01741 |  |
| 12 | 0,425781 | 0,00 | -0,02133 |  |
| 13 | 0,569258 | 0,01 | 0,26862 |  |
| 14 | 0,569258 | 0,00 | -0,12493 |  |
| 15 | 0,433227 | 0,00 | 0,08402 |  |
| 16 | 0,433227 | 0,01 | -0,26584 |  |
| 17 | 0,597775 | 0,09 | 1,12896 |  |
| 18 | 0,597775 | 0,05 | -0,83635 |  |
| 19 | 0,407038 | 0,12 | -1,46726 |  |
| 20 | 0,647412 | 0,24 | 1,97006 |  |
| 21 | 0,407933 | 0,01 | 0,40565 |  |
| 22 | 0,817044 | 0,03 | -0,63464 |  |
| 23 | 0,823434 | 0,05 | -0,79388 |  |
| 24 | 0,713775 | 0,03 | 0,63135 |  |
| 25 | 0,288793 | 0,05 | -0,85806 |  |
| 26 | 0,288793 | 0,00 | 0,14937 |  |
| 27 | 0,288793 | 0,08 | 1,20247 |  |

R  Large residual

## Coded Coefficients

| Term | Coef | SE Coef | 95% CI | T-Value | P-Value | VIF |
| --- | --- | --- | --- | --- | --- | --- |
| Constant | 1,743 | 0,482 | (0,693; 2,793) | 3,62 | 0,004 |  |
| Lac | 0,929 | 0,399 | (0,061; 1,797) | 2,33 | 0,038 | 1,18 |
| HPMC\_Visc | -0,536 | 0,416 | (-1,443; 0,371) | -1,29 | 0,222 | 1,70 |
| HPMC\_HP | 0,089 | 0,407 | (-0,798; 0,977) | 0,22 | 0,830 | 1,26 |
| HPMC\_PS | -0,044 | 0,625 | (-1,405; 1,318) | -0,07 | 0,945 | 2,09 |
| Lac\*Lac | 1,070 | 0,792 | (-0,655; 2,796) | 1,35 | 0,201 | 1,30 |
| HPMC\_Visc\*HPMC\_Visc | 0,455 | 0,842 | (-1,379; 2,289) | 0,54 | 0,599 | 1,96 |
| HPMC\_HP\*HPMC\_HP | -0,365 | 0,799 | (-2,106; 1,375) | -0,46 | 0,656 | 1,81 |
| HPMC\_PS\*HPMC\_PS | -0,661 | 0,794 | (-2,391; 1,069) | -0,83 | 0,421 | 1,42 |
| Lac\*HPMC\_Visc | -1,773 | 0,874 | (-3,676; 0,130) | -2,03 | 0,065 | 1,49 |
| Lac\*HPMC\_HP | 0,636 | 0,917 | (-1,362; 2,633) | 0,69 | 0,501 | 1,17 |
| Lac\*HPMC\_PS | -1,00 | 1,42 | (-4,08; 2,08) | -0,71 | 0,494 | 1,42 |
| HPMC\_Visc\*HPMC\_HP | -0,77 | 1,04 | (-3,04; 1,51) | -0,74 | 0,476 | 2,73 |
| HPMC\_Visc\*HPMC\_PS | 1,06 | 1,47 | (-2,14; 4,27) | 0,72 | 0,484 | 2,74 |
| HPMC\_HP\*HPMC\_PS | -0,84 | 1,63 | (-4,38; 2,71) | -0,51 | 0,617 | 2,70 |

## Model Summary

| S | R-sq | R-sq(adj) | PRESS | R-sq(pred) | AICc | BIC |
| --- | --- | --- | --- | --- | --- | --- |
| 0,897234 | 66,18% | 26,72% | 34,7837 | 0,00% | 135,27 | 101,61 |

## Analysis of Variance

| Source | DF | Seq SS | Contribution | Adj SS | Adj MS | F-Value | P-Value |
| --- | --- | --- | --- | --- | --- | --- | --- |
| Model | 14 | 18,9030 | 66,18% | 18,9030 | 1,35021 | 1,68 | 0,188 |
| Linear | 4 | 10,1597 | 35,57% | 6,2558 | 1,56396 | 1,94 | 0,168 |
| Lac | 1 | 7,2042 | 25,22% | 4,3756 | 4,37560 | 5,44 | 0,038 |
| HPMC\_Visc | 1 | 2,3835 | 8,34% | 1,3329 | 1,33291 | 1,66 | 0,222 |
| HPMC\_HP | 1 | 0,4972 | 1,74% | 0,0388 | 0,03883 | 0,05 | 0,830 |
| HPMC\_PS | 1 | 0,0749 | 0,26% | 0,0039 | 0,00393 | 0,00 | 0,945 |
| Square | 4 | 4,5036 | 15,77% | 3,9327 | 0,98318 | 1,22 | 0,352 |
| Lac\*Lac | 1 | 2,4567 | 8,60% | 1,4704 | 1,47040 | 1,83 | 0,201 |
| HPMC\_Visc\*HPMC\_Visc | 1 | 0,0522 | 0,18% | 0,2352 | 0,23523 | 0,29 | 0,599 |
| HPMC\_HP\*HPMC\_HP | 1 | 0,6950 | 2,43% | 0,1684 | 0,16843 | 0,21 | 0,656 |
| HPMC\_PS\*HPMC\_PS | 1 | 1,2996 | 4,55% | 0,5580 | 0,55800 | 0,69 | 0,421 |
| 2-Way Interaction | 6 | 4,2397 | 14,84% | 4,2397 | 0,70661 | 0,88 | 0,539 |
| Lac\*HPMC\_Visc | 1 | 2,9524 | 10,34% | 3,3162 | 3,31621 | 4,12 | 0,065 |
| Lac\*HPMC\_HP | 1 | 0,2353 | 0,82% | 0,3870 | 0,38701 | 0,48 | 0,501 |
| Lac\*HPMC\_PS | 1 | 0,4013 | 1,40% | 0,4013 | 0,40127 | 0,50 | 0,494 |
| HPMC\_Visc\*HPMC\_HP | 1 | 0,0921 | 0,32% | 0,4352 | 0,43525 | 0,54 | 0,476 |
| HPMC\_Visc\*HPMC\_PS | 1 | 0,3460 | 1,21% | 0,4208 | 0,42083 | 0,52 | 0,484 |
| HPMC\_HP\*HPMC\_PS | 1 | 0,2127 | 0,74% | 0,2127 | 0,21266 | 0,26 | 0,617 |
| Error | 12 | 9,6603 | 33,82% | 9,6603 | 0,80503 |  |  |
| Lack-of-Fit | 10 | 7,2333 | 25,32% | 7,2333 | 0,72333 | 0,60 | 0,765 |
| Pure Error | 2 | 2,4270 | 8,50% | 2,4270 | 1,21351 |  |  |
| Total | 26 | 28,5633 | 100,00% |  |  |  |  |

## Regression Equation in Uncoded Units

|  |  |  |
| --- | --- | --- |
| F\_SD\_0.33h(20min) | = | -163 + 25,3 Lac - 0,00079 HPMC\_Visc + 16,0 HPMC\_HP + 2,53 HPMC\_PS + 17,1 Lac\*Lac + 0,000000 HPMC\_Visc\*HPMC\_Visc - 0,355 HPMC\_HP\*HPMC\_HP - 0,0122 HPMC\_PS\*HPMC\_PS - 0,001823 Lac\*HPMC\_Visc + 2,51 Lac\*HPMC\_HP - 0,543 Lac\*HPMC\_PS - 0,000194 HPMC\_Visc\*HPMC\_HP + 0,000037 HPMC\_Visc\*HPMC\_PS - 0,112 HPMC\_HP\*HPMC\_PS |

## Fits and Diagnostics for All Observations

| Obs | F\_SD\_0.33h(20min) | Fit | SE Fit | 95% CI | Resid | Std Resid | Del Resid |
| --- | --- | --- | --- | --- | --- | --- | --- |
| 1 | 1,310 | 0,994 | 0,727 | (-0,590; 2,577) | 0,316 | 0,60 | 0,58 |
| 2 | 2,624 | 2,974 | 0,727 | (1,391; 4,557) | -0,350 | -0,67 | -0,65 |
| 3 | 0,977 | 1,094 | 0,767 | (-0,576; 2,764) | -0,117 | -0,25 | -0,24 |
| 4 | 0,852 | 0,901 | 0,767 | (-0,769; 2,571) | -0,049 | -0,11 | -0,10 |
| 5 | 1,255 | 1,601 | 0,620 | (0,251; 2,951) | -0,346 | -0,53 | -0,52 |
| 6 | 5,302 | 3,983 | 0,620 | (2,633; 5,333) | 1,319 | 2,03 | 2,40 |
| 7 | 1,054 | 1,158 | 0,745 | (-0,465; 2,780) | -0,104 | -0,21 | -0,20 |
| 8 | 1,174 | 1,518 | 0,745 | (-0,104; 3,141) | -0,344 | -0,69 | -0,67 |
| 9 | 1,561 | 1,152 | 0,684 | (-0,338; 2,642) | 0,409 | 0,70 | 0,69 |
| 10 | 2,614 | 2,462 | 0,684 | (0,972; 3,952) | 0,152 | 0,26 | 0,25 |
| 11 | 0,419 | 1,919 | 0,585 | (0,643; 3,194) | -1,499 | -2,21 | -2,74 |
| 12 | 2,099 | 1,996 | 0,585 | (0,721; 3,272) | 0,102 | 0,15 | 0,14 |
| 13 | 1,208 | 1,082 | 0,677 | (-0,393; 2,557) | 0,126 | 0,21 | 0,20 |
| 14 | 2,965 | 2,994 | 0,677 | (1,519; 4,469) | -0,030 | -0,05 | -0,05 |
| 15 | 1,951 | 1,710 | 0,591 | (0,424; 2,997) | 0,241 | 0,36 | 0,34 |
| 16 | 1,714 | 1,982 | 0,591 | (0,695; 3,269) | -0,268 | -0,40 | -0,38 |
| 17 | 2,092 | 1,661 | 0,694 | (0,149; 3,172) | 0,431 | 0,76 | 0,74 |
| 18 | 3,864 | 4,185 | 0,694 | (2,673; 5,696) | -0,321 | -0,56 | -0,55 |
| 19 | 1,408 | 2,566 | 0,572 | (1,318; 3,813) | -1,158 | -1,68 | -1,83 |
| 20 | 2,367 | 1,592 | 0,722 | (0,019; 3,165) | 0,775 | 1,45 | 1,53 |
| 21 | 1,467 | 1,191 | 0,573 | (-0,057; 2,440) | 0,276 | 0,40 | 0,39 |
| 22 | 1,483 | 1,568 | 0,811 | (-0,199; 3,335) | -0,084 | -0,22 | -0,21 |
| 23 | 1,413 | 1,487 | 0,814 | (-0,287; 3,261) | -0,074 | -0,20 | -0,19 |
| 24 | 1,115 | 1,011 | 0,758 | (-0,641; 2,663) | 0,104 | 0,22 | 0,21 |
| 25 | 0,866 | 1,852 | 0,482 | (0,802; 2,903) | -0,986 | -1,30 | -1,35 |
| 26 | 2,122 | 1,852 | 0,482 | (0,802; 2,903) | 0,270 | 0,36 | 0,34 |
| 27 | 3,062 | 1,852 | 0,482 | (0,802; 2,903) | 1,210 | 1,60 | 1,73 |

| Obs | HI | Cook’s D | DFITS |  |
| --- | --- | --- | --- | --- |
| 1 | 0,655994 | 0,05 | 0,80688 |  |
| 2 | 0,655994 | 0,06 | -0,89698 |  |
| 3 | 0,729945 | 0,01 | -0,39456 |  |
| 4 | 0,729945 | 0,00 | -0,16537 |  |
| 5 | 0,476813 | 0,02 | -0,49273 |  |
| 6 | 0,476813 | 0,25 | 2,29450 | R |
| 7 | 0,688750 | 0,01 | -0,29509 |  |
| 8 | 0,688750 | 0,07 | -1,00003 |  |
| 9 | 0,580949 | 0,05 | 0,81069 |  |
| 10 | 0,580949 | 0,01 | 0,29615 |  |
| 11 | 0,425781 | 0,24 | -2,35754 | R |
| 12 | 0,425781 | 0,00 | 0,12401 |  |
| 13 | 0,569258 | 0,00 | 0,23513 |  |
| 14 | 0,569258 | 0,00 | -0,05583 |  |
| 15 | 0,433227 | 0,01 | 0,30011 |  |
| 16 | 0,433227 | 0,01 | -0,33463 |  |
| 17 | 0,597775 | 0,06 | 0,90706 |  |
| 18 | 0,597775 | 0,03 | -0,66740 |  |
| 19 | 0,407038 | 0,13 | -1,51838 |  |
| 20 | 0,647412 | 0,26 | 2,07791 |  |
| 21 | 0,407933 | 0,01 | 0,31992 |  |
| 22 | 0,817044 | 0,01 | -0,44628 |  |
| 23 | 0,823434 | 0,01 | -0,40917 |  |
| 24 | 0,713775 | 0,01 | 0,32870 |  |
| 25 | 0,288793 | 0,05 | -0,85813 |  |
| 26 | 0,288793 | 0,00 | 0,21861 |  |
| 27 | 0,288793 | 0,07 | 1,09929 |  |

R  Large residual

## Coded Coefficients

| Term | Coef | SE Coef | 95% CI | T-Value | P-Value | VIF |
| --- | --- | --- | --- | --- | --- | --- |
| Constant | 1,915 | 0,494 | (0,838; 2,992) | 3,87 | 0,002 |  |
| Lac | 0,924 | 0,409 | (0,033; 1,814) | 2,26 | 0,043 | 1,18 |
| HPMC\_Visc | -0,581 | 0,427 | (-1,512; 0,349) | -1,36 | 0,199 | 1,70 |
| HPMC\_HP | 0,124 | 0,418 | (-0,786; 1,035) | 0,30 | 0,771 | 1,26 |
| HPMC\_PS | -0,040 | 0,641 | (-1,437; 1,356) | -0,06 | 0,951 | 2,09 |
| Lac\*Lac | 1,069 | 0,812 | (-0,701; 2,839) | 1,32 | 0,213 | 1,30 |
| HPMC\_Visc\*HPMC\_Visc | 0,352 | 0,864 | (-1,530; 2,233) | 0,41 | 0,691 | 1,96 |
| HPMC\_HP\*HPMC\_HP | -0,552 | 0,819 | (-2,337; 1,234) | -0,67 | 0,514 | 1,81 |
| HPMC\_PS\*HPMC\_PS | -0,812 | 0,814 | (-2,586; 0,963) | -1,00 | 0,339 | 1,42 |
| Lac\*HPMC\_Visc | -1,766 | 0,896 | (-3,718; 0,187) | -1,97 | 0,072 | 1,49 |
| Lac\*HPMC\_HP | 0,642 | 0,941 | (-1,407; 2,692) | 0,68 | 0,508 | 1,17 |
| Lac\*HPMC\_PS | -0,95 | 1,45 | (-4,11; 2,21) | -0,65 | 0,525 | 1,42 |
| HPMC\_Visc\*HPMC\_HP | -0,65 | 1,07 | (-2,98; 1,68) | -0,61 | 0,556 | 2,73 |
| HPMC\_Visc\*HPMC\_PS | 0,86 | 1,51 | (-2,43; 4,15) | 0,57 | 0,578 | 2,74 |
| HPMC\_HP\*HPMC\_PS | -0,50 | 1,67 | (-4,13; 3,14) | -0,30 | 0,771 | 2,70 |

## Model Summary

| S | R-sq | R-sq(adj) | PRESS | R-sq(pred) | AICc | BIC |
| --- | --- | --- | --- | --- | --- | --- |
| 0,920351 | 66,22% | 26,81% | 36,8549 | 0,00% | 136,65 | 102,98 |

## Analysis of Variance

| Source | DF | Seq SS | Contribution | Adj SS | Adj MS | F-Value | P-Value |
| --- | --- | --- | --- | --- | --- | --- | --- |
| Model | 14 | 19,9266 | 66,22% | 19,9266 | 1,42333 | 1,68 | 0,187 |
| Linear | 4 | 10,5204 | 34,96% | 6,5913 | 1,64783 | 1,95 | 0,167 |
| Lac | 1 | 7,1034 | 23,61% | 4,3252 | 4,32518 | 5,11 | 0,043 |
| HPMC\_Visc | 1 | 2,6774 | 8,90% | 1,5687 | 1,56869 | 1,85 | 0,199 |
| HPMC\_HP | 1 | 0,6764 | 2,25% | 0,0748 | 0,07481 | 0,09 | 0,771 |
| HPMC\_PS | 1 | 0,0632 | 0,21% | 0,0034 | 0,00337 | 0,00 | 0,951 |
| Square | 4 | 5,3429 | 17,76% | 4,6748 | 1,16870 | 1,38 | 0,299 |
| Lac\*Lac | 1 | 2,8348 | 9,42% | 1,4669 | 1,46692 | 1,73 | 0,213 |
| HPMC\_Visc\*HPMC\_Visc | 1 | 0,0550 | 0,18% | 0,1406 | 0,14057 | 0,17 | 0,691 |
| HPMC\_HP\*HPMC\_HP | 1 | 0,8762 | 2,91% | 0,3839 | 0,38392 | 0,45 | 0,514 |
| HPMC\_PS\*HPMC\_PS | 1 | 1,5769 | 5,24% | 0,8413 | 0,84132 | 0,99 | 0,339 |
| 2-Way Interaction | 6 | 4,0633 | 13,50% | 4,0633 | 0,67722 | 0,80 | 0,588 |
| Lac\*HPMC\_Visc | 1 | 2,9858 | 9,92% | 3,2895 | 3,28952 | 3,88 | 0,072 |
| Lac\*HPMC\_HP | 1 | 0,2495 | 0,83% | 0,3952 | 0,39517 | 0,47 | 0,508 |
| Lac\*HPMC\_PS | 1 | 0,3624 | 1,20% | 0,3624 | 0,36238 | 0,43 | 0,525 |
| HPMC\_Visc\*HPMC\_HP | 1 | 0,1491 | 0,50% | 0,3115 | 0,31152 | 0,37 | 0,556 |
| HPMC\_Visc\*HPMC\_PS | 1 | 0,2411 | 0,80% | 0,2763 | 0,27628 | 0,33 | 0,578 |
| HPMC\_HP\*HPMC\_PS | 1 | 0,0754 | 0,25% | 0,0754 | 0,07542 | 0,09 | 0,771 |
| Error | 12 | 10,1645 | 33,78% | 10,1645 | 0,84705 |  |  |
| Lack-of-Fit | 10 | 7,8284 | 26,02% | 7,8284 | 0,78284 | 0,67 | 0,729 |
| Pure Error | 2 | 2,3362 | 7,76% | 2,3362 | 1,16808 |  |  |
| Total | 26 | 30,0911 | 100,00% |  |  |  |  |

## Regression Equation in Uncoded Units

|  |  |  |
| --- | --- | --- |
| F\_SD\_0.5h(30min) | = | -166 + 23,1 Lac - 0,00041 HPMC\_Visc + 15,9 HPMC\_HP + 2,57 HPMC\_PS + 17,1 Lac\*Lac + 0,000000 HPMC\_Visc\*HPMC\_Visc - 0,535 HPMC\_HP\*HPMC\_HP - 0,0150 HPMC\_PS\*HPMC\_PS - 0,001816 Lac\*HPMC\_Visc + 2,53 Lac\*HPMC\_HP - 0,516 Lac\*HPMC\_PS - 0,000164 HPMC\_Visc\*HPMC\_HP + 0,000030 HPMC\_Visc\*HPMC\_PS - 0,067 HPMC\_HP\*HPMC\_PS |

## Fits and Diagnostics for All Observations

| Obs | F\_SD\_0.5h(30min) | Fit | SE Fit | 95% CI | Resid | Std Resid | Del Resid | HI |
| --- | --- | --- | --- | --- | --- | --- | --- | --- |
| 1 | 1,373 | 1,112 | 0,745 | (-0,512; 2,736) | 0,261 | 0,48 | 0,47 | 0,655994 |
| 2 | 2,735 | 3,065 | 0,745 | (1,441; 4,689) | -0,330 | -0,61 | -0,59 | 0,655994 |
| 3 | 1,066 | 1,184 | 0,786 | (-0,529; 2,898) | -0,119 | -0,25 | -0,24 | 0,729945 |
| 4 | 0,888 | 0,957 | 0,786 | (-0,756; 2,670) | -0,069 | -0,14 | -0,14 | 0,729945 |
| 5 | 1,512 | 1,711 | 0,636 | (0,326; 3,095) | -0,199 | -0,30 | -0,29 | 0,476813 |
| 6 | 5,374 | 4,076 | 0,636 | (2,691; 5,460) | 1,298 | 1,95 | 2,26 | 0,476813 |
| 7 | 1,170 | 1,236 | 0,764 | (-0,428; 2,901) | -0,066 | -0,13 | -0,12 | 0,688750 |
| 8 | 1,178 | 1,580 | 0,764 | (-0,084; 3,245) | -0,402 | -0,78 | -0,77 | 0,688750 |
| 9 | 1,738 | 1,186 | 0,701 | (-0,342; 2,715) | 0,551 | 0,93 | 0,92 | 0,580949 |
| 10 | 2,574 | 2,502 | 0,701 | (0,973; 4,030) | 0,073 | 0,12 | 0,12 | 0,580949 |
| 11 | 0,359 | 1,958 | 0,601 | (0,650; 3,267) | -1,599 | -2,29 | -2,93 | 0,425781 |
| 12 | 2,121 | 2,039 | 0,601 | (0,731; 3,348) | 0,081 | 0,12 | 0,11 | 0,425781 |
| 13 | 1,384 | 1,281 | 0,694 | (-0,232; 2,794) | 0,103 | 0,17 | 0,16 | 0,569258 |
| 14 | 3,249 | 3,211 | 0,694 | (1,698; 4,724) | 0,038 | 0,06 | 0,06 | 0,569258 |
| 15 | 2,159 | 1,852 | 0,606 | (0,532; 3,171) | 0,308 | 0,44 | 0,43 | 0,433227 |
| 16 | 1,865 | 2,134 | 0,606 | (0,814; 3,454) | -0,269 | -0,39 | -0,37 | 0,433227 |
| 17 | 2,180 | 1,842 | 0,712 | (0,292; 3,393) | 0,338 | 0,58 | 0,56 | 0,597775 |
| 18 | 4,096 | 4,349 | 0,712 | (2,798; 5,899) | -0,252 | -0,43 | -0,42 | 0,597775 |
| 19 | 1,429 | 2,700 | 0,587 | (1,421; 3,979) | -1,271 | -1,79 | -2,01 | 0,407038 |
| 20 | 2,438 | 1,616 | 0,741 | (0,003; 3,230) | 0,822 | 1,50 | 1,60 | 0,647412 |
| 21 | 1,537 | 1,198 | 0,588 | (-0,083; 2,478) | 0,339 | 0,48 | 0,46 | 0,407933 |
| 22 | 1,468 | 1,597 | 0,832 | (-0,215; 3,410) | -0,129 | -0,33 | -0,31 | 0,817044 |
| 23 | 1,412 | 1,481 | 0,835 | (-0,339; 3,300) | -0,069 | -0,18 | -0,17 | 0,823434 |
| 24 | 1,115 | 1,030 | 0,778 | (-0,664; 2,724) | 0,085 | 0,17 | 0,17 | 0,713775 |
| 25 | 1,007 | 2,026 | 0,495 | (0,949; 3,104) | -1,019 | -1,31 | -1,36 | 0,288793 |
| 26 | 2,421 | 2,026 | 0,495 | (0,949; 3,104) | 0,395 | 0,51 | 0,49 | 0,288793 |
| 27 | 3,130 | 2,026 | 0,495 | (0,949; 3,104) | 1,104 | 1,42 | 1,49 | 0,288793 |

| Obs | Cook’s D | DFITS |  |
| --- | --- | --- | --- |
| 1 | 0,03 | 0,64504 |  |
| 2 | 0,05 | -0,82023 |  |
| 3 | 0,01 | -0,39160 |  |
| 4 | 0,00 | -0,22812 |  |
| 5 | 0,01 | -0,27391 |  |
| 6 | 0,23 | 2,15595 |  |
| 7 | 0,00 | -0,18377 |  |
| 8 | 0,09 | -1,14535 |  |
| 9 | 0,08 | 1,08243 |  |
| 10 | 0,00 | 0,13758 |  |
| 11 | 0,26 | -2,52283 | R |
| 12 | 0,00 | 0,09596 |  |
| 13 | 0,00 | 0,18702 |  |
| 14 | 0,00 | 0,06844 |  |
| 15 | 0,01 | 0,37471 |  |
| 16 | 0,01 | -0,32719 |  |
| 17 | 0,03 | 0,68494 |  |
| 18 | 0,02 | -0,50839 |  |
| 19 | 0,15 | -1,66317 |  |
| 20 | 0,28 | 2,16623 |  |
| 21 | 0,01 | 0,38446 |  |
| 22 | 0,03 | -0,66503 |  |
| 23 | 0,01 | -0,37038 |  |
| 24 | 0,00 | 0,26222 |  |
| 25 | 0,05 | -0,86585 |  |
| 26 | 0,01 | 0,31371 |  |
| 27 | 0,05 | 0,95126 |  |

R  Large residual

## Coded Coefficients

| Term | Coef | SE Coef | 95% CI | T-Value | P-Value | VIF |
| --- | --- | --- | --- | --- | --- | --- |
| Constant | 2,051 | 0,525 | (0,906; 3,196) | 3,90 | 0,002 |  |
| Lac | 1,008 | 0,434 | (0,061; 1,954) | 2,32 | 0,039 | 1,18 |
| HPMC\_Visc | -0,538 | 0,454 | (-1,527; 0,451) | -1,18 | 0,259 | 1,70 |
| HPMC\_HP | 0,161 | 0,444 | (-0,807; 1,129) | 0,36 | 0,723 | 1,26 |
| HPMC\_PS | 0,030 | 0,681 | (-1,454; 1,515) | 0,04 | 0,965 | 2,09 |
| Lac\*Lac | 1,155 | 0,864 | (-0,727; 3,036) | 1,34 | 0,206 | 1,30 |
| HPMC\_Visc\*HPMC\_Visc | 0,334 | 0,918 | (-1,665; 2,334) | 0,36 | 0,722 | 1,96 |
| HPMC\_HP\*HPMC\_HP | -0,709 | 0,871 | (-2,606; 1,189) | -0,81 | 0,432 | 1,81 |
| HPMC\_PS\*HPMC\_PS | -0,915 | 0,866 | (-2,801; 0,971) | -1,06 | 0,311 | 1,42 |
| Lac\*HPMC\_Visc | -1,754 | 0,952 | (-3,829; 0,321) | -1,84 | 0,090 | 1,49 |
| Lac\*HPMC\_HP | 0,77 | 1,00 | (-1,41; 2,94) | 0,77 | 0,458 | 1,17 |
| Lac\*HPMC\_PS | -0,75 | 1,54 | (-4,11; 2,61) | -0,49 | 0,636 | 1,42 |
| HPMC\_Visc\*HPMC\_HP | -0,50 | 1,14 | (-2,98; 1,98) | -0,44 | 0,668 | 2,73 |
| HPMC\_Visc\*HPMC\_PS | 0,90 | 1,61 | (-2,60; 4,40) | 0,56 | 0,584 | 2,74 |
| HPMC\_HP\*HPMC\_PS | -0,07 | 1,77 | (-3,93; 3,79) | -0,04 | 0,968 | 2,70 |

## Model Summary

| S | R-sq | R-sq(adj) | PRESS | R-sq(pred) | AICc | BIC |
| --- | --- | --- | --- | --- | --- | --- |
| 0,978173 | 66,02% | 26,38% | 40,8986 | 0,00% | 139,94 | 106,27 |

## Analysis of Variance

| Source | DF | Seq SS | Contribution | Adj SS | Adj MS | F-Value | P-Value |
| --- | --- | --- | --- | --- | --- | --- | --- |
| Model | 14 | 22,3096 | 66,02% | 22,3096 | 1,59354 | 1,67 | 0,191 |
| Linear | 4 | 11,4054 | 33,75% | 7,3344 | 1,83360 | 1,92 | 0,172 |
| Lac | 1 | 7,9624 | 23,56% | 5,1453 | 5,14530 | 5,38 | 0,039 |
| HPMC\_Visc | 1 | 2,5476 | 7,54% | 1,3421 | 1,34210 | 1,40 | 0,259 |
| HPMC\_HP | 1 | 0,8386 | 2,48% | 0,1259 | 0,12593 | 0,13 | 0,723 |
| HPMC\_PS | 1 | 0,0569 | 0,17% | 0,0019 | 0,00192 | 0,00 | 0,965 |
| Square | 4 | 6,5899 | 19,50% | 5,8337 | 1,45842 | 1,52 | 0,257 |
| Lac\*Lac | 1 | 3,5015 | 10,36% | 1,7114 | 1,71135 | 1,79 | 0,206 |
| HPMC\_Visc\*HPMC\_Visc | 1 | 0,0600 | 0,18% | 0,1269 | 0,12693 | 0,13 | 0,722 |
| HPMC\_HP\*HPMC\_HP | 1 | 1,0975 | 3,25% | 0,6338 | 0,63380 | 0,66 | 0,432 |
| HPMC\_PS\*HPMC\_PS | 1 | 1,9309 | 5,71% | 1,0696 | 1,06962 | 1,12 | 0,311 |
| 2-Way Interaction | 6 | 4,3143 | 12,77% | 4,3143 | 0,71904 | 0,75 | 0,620 |
| Lac\*HPMC\_Visc | 1 | 3,1704 | 9,38% | 3,2456 | 3,24557 | 3,39 | 0,090 |
| Lac\*HPMC\_HP | 1 | 0,4296 | 1,27% | 0,5623 | 0,56232 | 0,59 | 0,458 |
| Lac\*HPMC\_PS | 1 | 0,2261 | 0,67% | 0,2261 | 0,22612 | 0,24 | 0,636 |
| HPMC\_Visc\*HPMC\_HP | 1 | 0,1843 | 0,55% | 0,1849 | 0,18493 | 0,19 | 0,668 |
| HPMC\_Visc\*HPMC\_PS | 1 | 0,3022 | 0,89% | 0,3023 | 0,30231 | 0,32 | 0,584 |
| HPMC\_HP\*HPMC\_PS | 1 | 0,0016 | 0,00% | 0,0016 | 0,00164 | 0,00 | 0,968 |
| Error | 12 | 11,4819 | 33,98% | 11,4819 | 0,95682 |  |  |
| Lack-of-Fit | 10 | 8,6221 | 25,52% | 8,6221 | 0,86221 | 0,60 | 0,761 |
| Pure Error | 2 | 2,8598 | 8,46% | 2,8598 | 1,42991 |  |  |
| Total | 26 | 33,7915 | 100,00% |  |  |  |  |

## Regression Equation in Uncoded Units

|  |  |  |
| --- | --- | --- |
| F\_SD\_0.75h(45min) | = | -140 + 9,7 Lac - 0,00083 HPMC\_Visc + 14,1 HPMC\_HP + 2,23 HPMC\_PS + 18,5 Lac\*Lac + 0,000000 HPMC\_Visc\*HPMC\_Visc - 0,688 HPMC\_HP\*HPMC\_HP - 0,0169 HPMC\_PS\*HPMC\_PS - 0,001804 Lac\*HPMC\_Visc + 3,02 Lac\*HPMC\_HP - 0,408 Lac\*HPMC\_PS - 0,000127 HPMC\_Visc\*HPMC\_HP + 0,000032 HPMC\_Visc\*HPMC\_PS - 0,010 HPMC\_HP\*HPMC\_PS |

## Fits and Diagnostics for All Observations

| Obs | F\_SD\_0.75h(45min) | Fit | SE Fit | 95% CI | Resid | Std Resid | Del Resid |
| --- | --- | --- | --- | --- | --- | --- | --- |
| 1 | 1,455 | 1,288 | 0,792 | (-0,438; 3,015) | 0,167 | 0,29 | 0,28 |
| 2 | 2,942 | 3,178 | 0,792 | (1,451; 4,904) | -0,236 | -0,41 | -0,40 |
| 3 | 1,206 | 1,321 | 0,836 | (-0,500; 3,142) | -0,115 | -0,23 | -0,22 |
| 4 | 0,920 | 0,977 | 0,836 | (-0,843; 2,798) | -0,057 | -0,11 | -0,11 |
| 5 | 1,752 | 1,754 | 0,675 | (0,283; 3,226) | -0,002 | -0,00 | -0,00 |
| 6 | 5,464 | 4,187 | 0,675 | (2,715; 5,658) | 1,277 | 1,81 | 2,02 |
| 7 | 1,248 | 1,279 | 0,812 | (-0,490; 3,048) | -0,031 | -0,06 | -0,05 |
| 8 | 1,255 | 1,697 | 0,812 | (-0,072; 3,465) | -0,442 | -0,81 | -0,80 |
| 9 | 1,854 | 1,121 | 0,746 | (-0,503; 2,746) | 0,733 | 1,16 | 1,18 |
| 10 | 2,459 | 2,511 | 0,746 | (0,886; 4,135) | -0,052 | -0,08 | -0,08 |
| 11 | 0,287 | 1,992 | 0,638 | (0,601; 3,382) | -1,705 | -2,30 | -2,95 |
| 12 | 2,231 | 2,142 | 0,638 | (0,751; 3,533) | 0,089 | 0,12 | 0,11 |
| 13 | 1,318 | 1,315 | 0,738 | (-0,293; 2,923) | 0,002 | 0,00 | 0,00 |
| 14 | 3,625 | 3,475 | 0,738 | (1,867; 5,084) | 0,150 | 0,23 | 0,22 |
| 15 | 2,361 | 1,982 | 0,644 | (0,579; 3,385) | 0,379 | 0,51 | 0,50 |
| 16 | 2,163 | 2,427 | 0,644 | (1,024; 3,829) | -0,264 | -0,36 | -0,34 |
| 17 | 2,258 | 1,985 | 0,756 | (0,337; 3,633) | 0,273 | 0,44 | 0,42 |
| 18 | 4,381 | 4,627 | 0,756 | (2,979; 6,275) | -0,246 | -0,40 | -0,38 |
| 19 | 1,390 | 2,782 | 0,624 | (1,422; 4,141) | -1,391 | -1,85 | -2,09 |
| 20 | 2,566 | 1,735 | 0,787 | (0,020; 3,449) | 0,831 | 1,43 | 1,50 |
| 21 | 1,553 | 1,195 | 0,625 | (-0,166; 2,556) | 0,358 | 0,48 | 0,46 |
| 22 | 1,446 | 1,617 | 0,884 | (-0,309; 3,543) | -0,171 | -0,41 | -0,39 |
| 23 | 1,379 | 1,471 | 0,888 | (-0,463; 3,405) | -0,092 | -0,22 | -0,21 |
| 24 | 1,118 | 1,033 | 0,826 | (-0,768; 2,833) | 0,085 | 0,16 | 0,16 |
| 25 | 0,949 | 2,151 | 0,526 | (1,006; 3,297) | -1,203 | -1,46 | -1,54 |
| 26 | 2,757 | 2,151 | 0,526 | (1,006; 3,297) | 0,606 | 0,73 | 0,72 |
| 27 | 3,208 | 2,151 | 0,526 | (1,006; 3,297) | 1,057 | 1,28 | 1,32 |

| Obs | HI | Cook’s D | DFITS |  |
| --- | --- | --- | --- | --- |
| 1 | 0,655994 | 0,01 | 0,38595 |  |
| 2 | 0,655994 | 0,02 | -0,54693 |  |
| 3 | 0,729945 | 0,01 | -0,35775 |  |
| 4 | 0,729945 | 0,00 | -0,17682 |  |
| 5 | 0,476813 | 0,00 | -0,00232 |  |
| 6 | 0,476813 | 0,20 | 1,93298 |  |
| 7 | 0,688750 | 0,00 | -0,08029 |  |
| 8 | 0,688750 | 0,10 | -1,18532 |  |
| 9 | 0,580949 | 0,12 | 1,38445 |  |
| 10 | 0,580949 | 0,00 | -0,09232 |  |
| 11 | 0,425781 | 0,26 | -2,53600 | R |
| 12 | 0,425781 | 0,00 | 0,09886 |  |
| 13 | 0,569258 | 0,00 | 0,00358 |  |
| 14 | 0,569258 | 0,00 | 0,25721 |  |
| 15 | 0,433227 | 0,01 | 0,43518 |  |
| 16 | 0,433227 | 0,01 | -0,30118 |  |
| 17 | 0,597775 | 0,02 | 0,51747 |  |
| 18 | 0,597775 | 0,02 | -0,46635 |  |
| 19 | 0,407038 | 0,16 | -1,73212 |  |
| 20 | 0,647412 | 0,25 | 2,03813 |  |
| 21 | 0,407933 | 0,01 | 0,38208 |  |
| 22 | 0,817044 | 0,05 | -0,83326 |  |
| 23 | 0,823434 | 0,02 | -0,46281 |  |
| 24 | 0,713775 | 0,00 | 0,24543 |  |
| 25 | 0,288793 | 0,06 | -0,98061 |  |
| 26 | 0,288793 | 0,01 | 0,45834 |  |
| 27 | 0,288793 | 0,04 | 0,84126 |  |

R  Large residual

## Coded Coefficients

| Term | Coef | SE Coef | 95% CI | T-Value | P-Value | VIF |
| --- | --- | --- | --- | --- | --- | --- |
| Constant | 2,128 | 0,557 | (0,915; 3,342) | 3,82 | 0,002 |  |
| Lac | 1,085 | 0,461 | (0,082; 2,089) | 2,36 | 0,036 | 1,18 |
| HPMC\_Visc | -0,481 | 0,481 | (-1,529; 0,568) | -1,00 | 0,338 | 1,70 |
| HPMC\_HP | 0,212 | 0,471 | (-0,814; 1,238) | 0,45 | 0,661 | 1,26 |
| HPMC\_PS | 0,098 | 0,722 | (-1,475; 1,671) | 0,14 | 0,894 | 2,09 |
| Lac\*Lac | 1,338 | 0,915 | (-0,657; 3,332) | 1,46 | 0,170 | 1,30 |
| HPMC\_Visc\*HPMC\_Visc | 0,330 | 0,973 | (-1,790; 2,450) | 0,34 | 0,740 | 1,96 |
| HPMC\_HP\*HPMC\_HP | -0,766 | 0,923 | (-2,777; 1,245) | -0,83 | 0,423 | 1,81 |
| HPMC\_PS\*HPMC\_PS | -0,966 | 0,918 | (-2,966; 1,033) | -1,05 | 0,313 | 1,42 |
| Lac\*HPMC\_Visc | -1,68 | 1,01 | (-3,88; 0,52) | -1,66 | 0,122 | 1,49 |
| Lac\*HPMC\_HP | 0,90 | 1,06 | (-1,41; 3,21) | 0,85 | 0,412 | 1,17 |
| Lac\*HPMC\_PS | -0,58 | 1,64 | (-4,14; 2,99) | -0,35 | 0,731 | 1,42 |
| HPMC\_Visc\*HPMC\_HP | -0,40 | 1,21 | (-3,02; 2,23) | -0,33 | 0,746 | 2,73 |
| HPMC\_Visc\*HPMC\_PS | 0,96 | 1,70 | (-2,74; 4,67) | 0,57 | 0,581 | 2,74 |
| HPMC\_HP\*HPMC\_PS | 0,23 | 1,88 | (-3,86; 4,32) | 0,12 | 0,905 | 2,70 |

## Model Summary

| S | R-sq | R-sq(adj) | PRESS | R-sq(pred) | AICc | BIC |
| --- | --- | --- | --- | --- | --- | --- |
| 1,03694 | 65,40% | 25,03% | 46,1804 | 0,00% | 143,09 | 109,42 |

## Analysis of Variance

| Source | DF | Seq SS | Contribution | Adj SS | Adj MS | F-Value | P-Value |
| --- | --- | --- | --- | --- | --- | --- | --- |
| Model | 14 | 24,3853 | 65,40% | 24,3853 | 1,74181 | 1,62 | 0,204 |
| Linear | 4 | 12,0065 | 32,20% | 8,0411 | 2,01028 | 1,87 | 0,181 |
| Lac | 1 | 8,6439 | 23,18% | 5,9705 | 5,97050 | 5,55 | 0,036 |
| HPMC\_Visc | 1 | 2,2785 | 6,11% | 1,0721 | 1,07206 | 1,00 | 0,338 |
| HPMC\_HP | 1 | 1,0358 | 2,78% | 0,2176 | 0,21760 | 0,20 | 0,661 |
| HPMC\_PS | 1 | 0,0483 | 0,13% | 0,0197 | 0,01974 | 0,02 | 0,894 |
| Square | 4 | 7,9186 | 21,24% | 7,1529 | 1,78824 | 1,66 | 0,223 |
| Lac\*Lac | 1 | 4,5767 | 12,27% | 2,2967 | 2,29673 | 2,14 | 0,170 |
| HPMC\_Visc\*HPMC\_Visc | 1 | 0,0577 | 0,15% | 0,1238 | 0,12379 | 0,12 | 0,740 |
| HPMC\_HP\*HPMC\_HP | 1 | 1,1336 | 3,04% | 0,7402 | 0,74024 | 0,69 | 0,423 |
| HPMC\_PS\*HPMC\_PS | 1 | 2,1506 | 5,77% | 1,1928 | 1,19281 | 1,11 | 0,313 |
| 2-Way Interaction | 6 | 4,4603 | 11,96% | 4,4603 | 0,74338 | 0,69 | 0,661 |
| Lac\*HPMC\_Visc | 1 | 3,0537 | 8,19% | 2,9773 | 2,97727 | 2,77 | 0,122 |
| Lac\*HPMC\_HP | 1 | 0,6692 | 1,79% | 0,7773 | 0,77727 | 0,72 | 0,412 |
| Lac\*HPMC\_PS | 1 | 0,1336 | 0,36% | 0,1336 | 0,13364 | 0,12 | 0,731 |
| HPMC\_Visc\*HPMC\_HP | 1 | 0,2125 | 0,57% | 0,1178 | 0,11783 | 0,11 | 0,746 |
| HPMC\_Visc\*HPMC\_PS | 1 | 0,3751 | 1,01% | 0,3454 | 0,34535 | 0,32 | 0,581 |
| HPMC\_HP\*HPMC\_PS | 1 | 0,0161 | 0,04% | 0,0161 | 0,01612 | 0,01 | 0,905 |
| Error | 12 | 12,9029 | 34,60% | 12,9029 | 1,07524 |  |  |
| Lack-of-Fit | 10 | 9,4937 | 25,46% | 9,4937 | 0,94937 | 0,56 | 0,784 |
| Pure Error | 2 | 3,4092 | 9,14% | 3,4092 | 1,70461 |  |  |
| Total | 26 | 37,2882 | 100,00% |  |  |  |  |

## Regression Equation in Uncoded Units

|  |  |  |
| --- | --- | --- |
| F\_SD\_1h(60min) | = | -112 - 5,5 Lac - 0,00124 HPMC\_Visc + 11,7 HPMC\_HP + 1,91 HPMC\_PS + 21,4 Lac\*Lac + 0,000000 HPMC\_Visc\*HPMC\_Visc - 0,744 HPMC\_HP\*HPMC\_HP - 0,0179 HPMC\_PS\*HPMC\_PS - 0,00173 Lac\*HPMC\_Visc + 3,55 Lac\*HPMC\_HP - 0,314 Lac\*HPMC\_PS - 0,000101 HPMC\_Visc\*HPMC\_HP + 0,000034 HPMC\_Visc\*HPMC\_PS + 0,031 HPMC\_HP\*HPMC\_PS |

## Fits and Diagnostics for All Observations

| Obs | F\_SD\_1h(60min) | Fit | SE Fit | 95% CI | Resid | Std Resid | Del Resid | HI |
| --- | --- | --- | --- | --- | --- | --- | --- | --- |
| 1 | 1,575 | 1,457 | 0,840 | (-0,373; 3,287) | 0,118 | 0,19 | 0,19 | 0,655994 |
| 2 | 3,009 | 3,231 | 0,840 | (1,401; 5,061) | -0,222 | -0,37 | -0,35 | 0,655994 |
| 3 | 1,284 | 1,447 | 0,886 | (-0,484; 3,377) | -0,163 | -0,30 | -0,29 | 0,729945 |
| 4 | 0,992 | 1,030 | 0,886 | (-0,900; 2,960) | -0,038 | -0,07 | -0,07 | 0,729945 |
| 5 | 1,922 | 1,802 | 0,716 | (0,242; 3,362) | 0,119 | 0,16 | 0,15 | 0,476813 |
| 6 | 5,554 | 4,262 | 0,716 | (2,702; 5,823) | 1,292 | 1,72 | 1,90 | 0,476813 |
| 7 | 1,297 | 1,315 | 0,861 | (-0,560; 3,190) | -0,019 | -0,03 | -0,03 | 0,688750 |
| 8 | 1,379 | 1,859 | 0,861 | (-0,016; 3,734) | -0,481 | -0,83 | -0,82 | 0,688750 |
| 9 | 1,905 | 1,102 | 0,790 | (-0,621; 2,824) | 0,804 | 1,20 | 1,22 | 0,580949 |
| 10 | 2,436 | 2,498 | 0,790 | (0,776; 4,220) | -0,062 | -0,09 | -0,09 | 0,580949 |
| 11 | 0,289 | 2,031 | 0,677 | (0,557; 3,505) | -1,742 | -2,22 | -2,76 | 0,425781 |
| 12 | 2,220 | 2,246 | 0,677 | (0,772; 3,720) | -0,026 | -0,03 | -0,03 | 0,425781 |
| 13 | 1,323 | 1,356 | 0,782 | (-0,348; 3,061) | -0,033 | -0,05 | -0,05 | 0,569258 |
| 14 | 3,913 | 3,687 | 0,782 | (1,983; 5,392) | 0,226 | 0,33 | 0,32 | 0,569258 |
| 15 | 2,468 | 2,082 | 0,683 | (0,595; 3,569) | 0,386 | 0,49 | 0,48 | 0,433227 |
| 16 | 2,474 | 2,695 | 0,683 | (1,207; 4,182) | -0,221 | -0,28 | -0,27 | 0,433227 |
| 17 | 2,438 | 2,181 | 0,802 | (0,434; 3,927) | 0,257 | 0,39 | 0,38 | 0,597775 |
| 18 | 4,683 | 4,924 | 0,802 | (3,177; 6,671) | -0,241 | -0,37 | -0,35 | 0,597775 |
| 19 | 1,281 | 2,799 | 0,662 | (1,357; 4,240) | -1,518 | -1,90 | -2,18 | 0,407038 |
| 20 | 2,722 | 1,825 | 0,834 | (0,007; 3,643) | 0,897 | 1,46 | 1,54 | 0,647412 |
| 21 | 1,632 | 1,197 | 0,662 | (-0,246; 2,640) | 0,434 | 0,54 | 0,53 | 0,407933 |
| 22 | 1,475 | 1,689 | 0,937 | (-0,353; 3,731) | -0,214 | -0,48 | -0,47 | 0,817044 |
| 23 | 1,360 | 1,451 | 0,941 | (-0,599; 3,501) | -0,091 | -0,21 | -0,20 | 0,823434 |
| 24 | 1,112 | 1,036 | 0,876 | (-0,873; 2,944) | 0,076 | 0,14 | 0,13 | 0,713775 |
| 25 | 0,864 | 2,214 | 0,557 | (1,000; 3,429) | -1,351 | -1,54 | -1,65 | 0,288793 |
| 26 | 3,042 | 2,214 | 0,557 | (1,000; 3,429) | 0,827 | 0,95 | 0,94 | 0,288793 |
| 27 | 3,200 | 2,214 | 0,557 | (1,000; 3,429) | 0,986 | 1,13 | 1,14 | 0,288793 |

| Obs | Cook’s D | DFITS |  |
| --- | --- | --- | --- |
| 1 | 0,00 | 0,25669 |  |
| 2 | 0,02 | -0,48612 |  |
| 3 | 0,02 | -0,47821 |  |
| 4 | 0,00 | -0,11195 |  |
| 5 | 0,00 | 0,14535 |  |
| 6 | 0,18 | 1,81437 |  |
| 7 | 0,00 | -0,04611 |  |
| 8 | 0,10 | -1,21900 |  |
| 9 | 0,13 | 1,43823 |  |
| 10 | 0,00 | -0,10416 |  |
| 11 | 0,24 | -2,37903 | R |
| 12 | 0,00 | -0,02724 |  |
| 13 | 0,00 | -0,05303 |  |
| 14 | 0,01 | 0,36658 |  |
| 15 | 0,01 | 0,41826 |  |
| 16 | 0,00 | -0,23759 |  |
| 17 | 0,02 | 0,45935 |  |
| 18 | 0,01 | -0,43099 |  |
| 19 | 0,17 | -1,80367 |  |
| 20 | 0,26 | 2,08349 |  |
| 21 | 0,01 | 0,43784 |  |
| 22 | 0,07 | -0,98753 |  |
| 23 | 0,01 | -0,43075 |  |
| 24 | 0,00 | 0,20848 |  |
| 25 | 0,06 | -1,05284 |  |
| 26 | 0,02 | 0,60009 |  |
| 27 | 0,03 | 0,72713 |  |

R  Large residual

## Coded Coefficients

| Term | Coef | SE Coef | 95% CI | T-Value | P-Value | VIF |
| --- | --- | --- | --- | --- | --- | --- |
| Constant | 2,228 | 0,622 | (0,873; 3,582) | 3,58 | 0,004 |  |
| Lac | 1,205 | 0,514 | (0,085; 2,325) | 2,34 | 0,037 | 1,18 |
| HPMC\_Visc | -0,375 | 0,537 | (-1,545; 0,795) | -0,70 | 0,498 | 1,70 |
| HPMC\_HP | 0,314 | 0,526 | (-0,831; 1,459) | 0,60 | 0,561 | 1,26 |
| HPMC\_PS | 0,231 | 0,806 | (-1,525; 1,987) | 0,29 | 0,780 | 2,09 |
| Lac\*Lac | 1,60 | 1,02 | (-0,63; 3,82) | 1,56 | 0,144 | 1,30 |
| HPMC\_Visc\*HPMC\_Visc | 0,38 | 1,09 | (-1,99; 2,74) | 0,35 | 0,736 | 1,96 |
| HPMC\_HP\*HPMC\_HP | -0,86 | 1,03 | (-3,10; 1,39) | -0,83 | 0,423 | 1,81 |
| HPMC\_PS\*HPMC\_PS | -0,99 | 1,02 | (-3,22; 1,24) | -0,97 | 0,353 | 1,42 |
| Lac\*HPMC\_Visc | -1,59 | 1,13 | (-4,05; 0,86) | -1,41 | 0,183 | 1,49 |
| Lac\*HPMC\_HP | 1,10 | 1,18 | (-1,48; 3,68) | 0,93 | 0,371 | 1,17 |
| Lac\*HPMC\_PS | -0,25 | 1,83 | (-4,22; 3,73) | -0,14 | 0,895 | 1,42 |
| HPMC\_Visc\*HPMC\_HP | -0,14 | 1,35 | (-3,07; 2,79) | -0,10 | 0,919 | 2,73 |
| HPMC\_Visc\*HPMC\_PS | 1,13 | 1,90 | (-3,01; 5,27) | 0,60 | 0,562 | 2,74 |
| HPMC\_HP\*HPMC\_PS | 0,82 | 2,10 | (-3,75; 5,39) | 0,39 | 0,702 | 2,70 |

## Model Summary

| S | R-sq | R-sq(adj) | PRESS | R-sq(pred) | AICc | BIC |
| --- | --- | --- | --- | --- | --- | --- |
| 1,15735 | 63,79% | 21,54% | 56,5426 | 0,00% | 149,02 | 115,35 |

## Analysis of Variance

| Source | DF | Seq SS | Contribution | Adj SS | Adj MS | F-Value | P-Value |
| --- | --- | --- | --- | --- | --- | --- | --- |
| Model | 14 | 28,3120 | 63,79% | 28,3120 | 2,02228 | 1,51 | 0,240 |
| Linear | 4 | 13,2182 | 29,78% | 9,4049 | 2,35123 | 1,76 | 0,203 |
| Lac | 1 | 9,8057 | 22,09% | 7,3566 | 7,35660 | 5,49 | 0,037 |
| HPMC\_Visc | 1 | 1,9543 | 4,40% | 0,6533 | 0,65327 | 0,49 | 0,498 |
| HPMC\_HP | 1 | 1,4134 | 3,18% | 0,4780 | 0,47800 | 0,36 | 0,561 |
| HPMC\_PS | 1 | 0,0449 | 0,10% | 0,1096 | 0,10960 | 0,08 | 0,780 |
| Square | 4 | 9,8765 | 22,25% | 9,1790 | 2,29475 | 1,71 | 0,212 |
| Lac\*Lac | 1 | 6,2254 | 14,03% | 3,2708 | 3,27076 | 2,44 | 0,144 |
| HPMC\_Visc\*HPMC\_Visc | 1 | 0,0745 | 0,17% | 0,1601 | 0,16013 | 0,12 | 0,736 |
| HPMC\_HP\*HPMC\_HP | 1 | 1,1877 | 2,68% | 0,9229 | 0,92291 | 0,69 | 0,423 |
| HPMC\_PS\*HPMC\_PS | 1 | 2,3888 | 5,38% | 1,2515 | 1,25146 | 0,93 | 0,353 |
| 2-Way Interaction | 6 | 5,2173 | 11,75% | 5,2173 | 0,86955 | 0,65 | 0,691 |
| Lac\*HPMC\_Visc | 1 | 3,0729 | 6,92% | 2,6751 | 2,67506 | 2,00 | 0,183 |
| Lac\*HPMC\_HP | 1 | 1,1443 | 2,58% | 1,1581 | 1,15806 | 0,86 | 0,371 |
| Lac\*HPMC\_PS | 1 | 0,0244 | 0,06% | 0,0244 | 0,02444 | 0,02 | 0,895 |
| HPMC\_Visc\*HPMC\_HP | 1 | 0,1856 | 0,42% | 0,0145 | 0,01453 | 0,01 | 0,919 |
| HPMC\_Visc\*HPMC\_PS | 1 | 0,5846 | 1,32% | 0,4774 | 0,47742 | 0,36 | 0,562 |
| HPMC\_HP\*HPMC\_PS | 1 | 0,2055 | 0,46% | 0,2055 | 0,20550 | 0,15 | 0,702 |
| Error | 12 | 16,0736 | 36,21% | 16,0736 | 1,33946 |  |  |
| Lack-of-Fit | 10 | 10,8491 | 24,44% | 10,8491 | 1,08491 | 0,42 | 0,860 |
| Pure Error | 2 | 5,2244 | 11,77% | 5,2244 | 2,61222 |  |  |
| Total | 26 | 44,3855 | 100,00% |  |  |  |  |

## Regression Equation in Uncoded Units

|  |  |  |
| --- | --- | --- |
| F\_SD\_1.5h(90min) | = | -46 - 30,3 Lac - 0,00237 HPMC\_Visc + 6,7 HPMC\_HP + 1,07 HPMC\_PS + 25,5 Lac\*Lac + 0,000000 HPMC\_Visc\*HPMC\_Visc - 0,83 HPMC\_HP\*HPMC\_HP - 0,0183 HPMC\_PS\*HPMC\_PS - 0,00164 Lac\*HPMC\_Visc + 4,33 Lac\*HPMC\_HP - 0,134 Lac\*HPMC\_PS - 0,000035 HPMC\_Visc\*HPMC\_HP + 0,000040 HPMC\_Visc\*HPMC\_PS + 0,110 HPMC\_HP\*HPMC\_PS |

## Fits and Diagnostics for All Observations

| Obs | F\_SD\_1.5h(90min) | Fit | SE Fit | 95% CI | Resid | Std Resid | Del Resid | HI |
| --- | --- | --- | --- | --- | --- | --- | --- | --- |
| 1 | 1,783 | 1,767 | 0,937 | (-0,275; 3,809) | 0,016 | 0,02 | 0,02 | 0,655994 |
| 2 | 3,215 | 3,373 | 0,937 | (1,331; 5,416) | -0,159 | -0,23 | -0,22 | 0,655994 |
| 3 | 1,392 | 1,629 | 0,989 | (-0,526; 3,783) | -0,236 | -0,39 | -0,38 | 0,729945 |
| 4 | 1,054 | 1,052 | 0,989 | (-1,103; 3,206) | 0,003 | 0,00 | 0,00 | 0,729945 |
| 5 | 2,184 | 1,851 | 0,799 | (0,110; 3,593) | 0,333 | 0,40 | 0,38 | 0,476813 |
| 6 | 5,687 | 4,358 | 0,799 | (2,617; 6,099) | 1,329 | 1,59 | 1,71 | 0,476813 |
| 7 | 1,391 | 1,408 | 0,960 | (-0,685; 3,500) | -0,017 | -0,03 | -0,03 | 0,688750 |
| 8 | 1,603 | 2,099 | 0,960 | (0,006; 4,192) | -0,496 | -0,77 | -0,75 | 0,688750 |
| 9 | 1,901 | 1,031 | 0,882 | (-0,891; 2,953) | 0,870 | 1,16 | 1,18 | 0,580949 |
| 10 | 2,432 | 2,488 | 0,882 | (0,566; 4,410) | -0,056 | -0,07 | -0,07 | 0,580949 |
| 11 | 0,302 | 2,044 | 0,755 | (0,398; 3,689) | -1,742 | -1,99 | -2,32 | 0,425781 |
| 12 | 2,133 | 2,369 | 0,755 | (0,724; 4,015) | -0,237 | -0,27 | -0,26 | 0,425781 |
| 13 | 1,297 | 1,393 | 0,873 | (-0,510; 3,295) | -0,096 | -0,13 | -0,12 | 0,569258 |
| 14 | 4,384 | 4,034 | 0,873 | (2,132; 5,937) | 0,350 | 0,46 | 0,44 | 0,569258 |
| 15 | 2,605 | 2,248 | 0,762 | (0,588; 3,907) | 0,357 | 0,41 | 0,39 | 0,433227 |
| 16 | 3,017 | 3,127 | 0,762 | (1,467; 4,786) | -0,110 | -0,13 | -0,12 | 0,433227 |
| 17 | 2,706 | 2,434 | 0,895 | (0,485; 4,384) | 0,272 | 0,37 | 0,36 | 0,597775 |
| 18 | 5,041 | 5,339 | 0,895 | (3,390; 7,289) | -0,299 | -0,41 | -0,39 | 0,597775 |
| 19 | 1,124 | 2,842 | 0,738 | (1,233; 4,451) | -1,718 | -1,93 | -2,22 | 0,407038 |
| 20 | 2,952 | 1,982 | 0,931 | (-0,047; 4,011) | 0,970 | 1,41 | 1,48 | 0,647412 |
| 21 | 1,733 | 1,186 | 0,739 | (-0,425; 2,796) | 0,547 | 0,61 | 0,60 | 0,407933 |
| 22 | 1,503 | 1,798 | 1,046 | (-0,481; 4,078) | -0,296 | -0,60 | -0,58 | 0,817044 |
| 23 | 1,348 | 1,455 | 1,050 | (-0,833; 3,743) | -0,106 | -0,22 | -0,21 | 0,823434 |
| 24 | 1,137 | 1,063 | 0,978 | (-1,067; 3,194) | 0,074 | 0,12 | 0,11 | 0,713775 |
| 25 | 0,582 | 2,290 | 0,622 | (0,935; 3,645) | -1,708 | -1,75 | -1,94 | 0,288793 |
| 26 | 3,530 | 2,290 | 0,622 | (0,935; 3,645) | 1,240 | 1,27 | 1,31 | 0,288793 |
| 27 | 3,205 | 2,290 | 0,622 | (0,935; 3,645) | 0,915 | 0,94 | 0,93 | 0,288793 |

| Obs | Cook’s D | DFITS |
| --- | --- | --- |
| 1 | 0,00 | 0,03081 |
| 2 | 0,01 | -0,30946 |
| 3 | 0,03 | -0,62296 |
| 4 | 0,00 | 0,00684 |
| 5 | 0,01 | 0,36596 |
| 6 | 0,15 | 1,63287 |
| 7 | 0,00 | -0,03776 |
| 8 | 0,09 | -1,12176 |
| 9 | 0,12 | 1,38870 |
| 10 | 0,00 | -0,08439 |
| 11 | 0,19 | -1,99818 |
| 12 | 0,00 | -0,22320 |
| 13 | 0,00 | -0,13877 |
| 14 | 0,02 | 0,51138 |
| 15 | 0,01 | 0,34525 |
| 16 | 0,00 | -0,10528 |
| 17 | 0,01 | 0,43422 |
| 18 | 0,02 | -0,47843 |
| 19 | 0,17 | -1,84000 |
| 20 | 0,24 | 2,00529 |
| 21 | 0,02 | 0,49610 |
| 22 | 0,11 | -1,22688 |
| 23 | 0,01 | -0,45345 |
| 24 | 0,00 | 0,18095 |
| 25 | 0,08 | -1,23698 |
| 26 | 0,04 | 0,83308 |
| 27 | 0,02 | 0,59396 |

## Coded Coefficients

| Term | Coef | SE Coef | 95% CI | T-Value | P-Value | VIF |
| --- | --- | --- | --- | --- | --- | --- |
| Constant | 2,318 | 0,664 | (0,871; 3,766) | 3,49 | 0,004 |  |
| Lac | 1,285 | 0,549 | (0,088; 2,482) | 2,34 | 0,037 | 1,18 |
| HPMC\_Visc | -0,253 | 0,574 | (-1,503; 0,998) | -0,44 | 0,668 | 1,70 |
| HPMC\_HP | 0,386 | 0,561 | (-0,837; 1,609) | 0,69 | 0,505 | 1,26 |
| HPMC\_PS | 0,379 | 0,861 | (-1,497; 2,255) | 0,44 | 0,667 | 2,09 |
| Lac\*Lac | 1,76 | 1,09 | (-0,62; 4,14) | 1,61 | 0,132 | 1,30 |
| HPMC\_Visc\*HPMC\_Visc | 0,46 | 1,16 | (-2,07; 2,99) | 0,40 | 0,698 | 1,96 |
| HPMC\_HP\*HPMC\_HP | -0,89 | 1,10 | (-3,29; 1,51) | -0,81 | 0,436 | 1,81 |
| HPMC\_PS\*HPMC\_PS | -1,02 | 1,09 | (-3,40; 1,37) | -0,93 | 0,371 | 1,42 |
| Lac\*HPMC\_Visc | -1,49 | 1,20 | (-4,11; 1,14) | -1,23 | 0,241 | 1,49 |
| Lac\*HPMC\_HP | 1,24 | 1,26 | (-1,51; 4,00) | 0,98 | 0,345 | 1,17 |
| Lac\*HPMC\_PS | 0,10 | 1,95 | (-4,15; 4,35) | 0,05 | 0,959 | 1,42 |
| HPMC\_Visc\*HPMC\_HP | 0,01 | 1,44 | (-3,12; 3,14) | 0,01 | 0,993 | 2,73 |
| HPMC\_Visc\*HPMC\_PS | 1,43 | 2,03 | (-2,99; 5,86) | 0,71 | 0,494 | 2,74 |
| HPMC\_HP\*HPMC\_PS | 1,19 | 2,24 | (-3,70; 6,07) | 0,53 | 0,606 | 2,70 |

## Model Summary

| S | R-sq | R-sq(adj) | PRESS | R-sq(pred) | AICc | BIC |
| --- | --- | --- | --- | --- | --- | --- |
| 1,23650 | 63,19% | 20,25% | 63,0251 | 0,00% | 152,59 | 118,92 |

## Analysis of Variance

| Source | DF | Seq SS | Contribution | Adj SS | Adj MS | F-Value | P-Value |
| --- | --- | --- | --- | --- | --- | --- | --- |
| Model | 14 | 31,5012 | 63,19% | 31,5012 | 2,25009 | 1,47 | 0,254 |
| Linear | 4 | 13,8569 | 27,80% | 10,4445 | 2,61112 | 1,71 | 0,213 |
| Lac | 1 | 10,4859 | 21,04% | 8,3700 | 8,37000 | 5,47 | 0,037 |
| HPMC\_Visc | 1 | 1,5993 | 3,21% | 0,2965 | 0,29647 | 0,19 | 0,668 |
| HPMC\_HP | 1 | 1,7442 | 3,50% | 0,7234 | 0,72343 | 0,47 | 0,505 |
| HPMC\_PS | 1 | 0,0276 | 0,06% | 0,2966 | 0,29662 | 0,19 | 0,667 |
| Square | 4 | 11,4581 | 22,99% | 10,6857 | 2,67142 | 1,75 | 0,204 |
| Lac\*Lac | 1 | 7,3283 | 14,70% | 3,9855 | 3,98548 | 2,61 | 0,132 |
| HPMC\_Visc\*HPMC\_Visc | 1 | 0,0815 | 0,16% | 0,2423 | 0,24228 | 0,16 | 0,698 |
| HPMC\_HP\*HPMC\_HP | 1 | 1,2469 | 2,50% | 0,9932 | 0,99323 | 0,65 | 0,436 |
| HPMC\_PS\*HPMC\_PS | 1 | 2,8014 | 5,62% | 1,3201 | 1,32008 | 0,86 | 0,371 |
| 2-Way Interaction | 6 | 6,1862 | 12,41% | 6,1862 | 1,03103 | 0,67 | 0,673 |
| Lac\*HPMC\_Visc | 1 | 3,0470 | 6,11% | 2,3265 | 2,32649 | 1,52 | 0,241 |
| Lac\*HPMC\_HP | 1 | 1,6063 | 3,22% | 1,4771 | 1,47713 | 0,97 | 0,345 |
| Lac\*HPMC\_PS | 1 | 0,0042 | 0,01% | 0,0042 | 0,00421 | 0,00 | 0,959 |
| HPMC\_Visc\*HPMC\_HP | 1 | 0,1444 | 0,29% | 0,0001 | 0,00014 | 0,00 | 0,993 |
| HPMC\_Visc\*HPMC\_PS | 1 | 0,9564 | 1,92% | 0,7621 | 0,76214 | 0,50 | 0,494 |
| HPMC\_HP\*HPMC\_PS | 1 | 0,4280 | 0,86% | 0,4280 | 0,42796 | 0,28 | 0,606 |
| Error | 12 | 18,3471 | 36,81% | 18,3471 | 1,52893 |  |  |
| Lack-of-Fit | 10 | 11,1481 | 22,36% | 11,1481 | 1,11481 | 0,31 | 0,917 |
| Pure Error | 2 | 7,1991 | 14,44% | 7,1991 | 3,59954 |  |  |
| Total | 26 | 49,8483 | 100,00% |  |  |  |  |

## Regression Equation in Uncoded Units

|  |  |  |
| --- | --- | --- |
| F\_SD\_2h(120min) | = | 5 - 52,7 Lac - 0,00365 HPMC\_Visc + 3,1 HPMC\_HP + 0,46 HPMC\_PS + 28,2 Lac\*Lac + 0,000000 HPMC\_Visc\*HPMC\_Visc - 0,86 HPMC\_HP\*HPMC\_HP - 0,0188 HPMC\_PS\*HPMC\_PS - 0,00153 Lac\*HPMC\_Visc + 4,89 Lac\*HPMC\_HP + 0,06 Lac\*HPMC\_PS + 0,000003 HPMC\_Visc\*HPMC\_HP + 0,000050 HPMC\_Visc\*HPMC\_PS + 0,159 HPMC\_HP\*HPMC\_PS |

## Fits and Diagnostics for All Observations

| Obs | F\_SD\_2h(120min) | Fit | SE Fit | 95% CI | Resid | Std Resid | Del Resid | HI |
| --- | --- | --- | --- | --- | --- | --- | --- | --- |
| 1 | 2,004 | 2,037 | 1,001 | (-0,145; 4,219) | -0,033 | -0,05 | -0,04 | 0,655994 |
| 2 | 3,271 | 3,459 | 1,001 | (1,277; 5,641) | -0,188 | -0,26 | -0,25 | 0,655994 |
| 3 | 1,456 | 1,756 | 1,056 | (-0,546; 4,057) | -0,300 | -0,47 | -0,45 | 0,729945 |
| 4 | 1,045 | 1,024 | 1,056 | (-1,278; 3,326) | 0,021 | 0,03 | 0,03 | 0,729945 |
| 5 | 2,398 | 1,938 | 0,854 | (0,077; 3,798) | 0,460 | 0,51 | 0,50 | 0,476813 |
| 6 | 5,712 | 4,424 | 0,854 | (2,564; 6,284) | 1,288 | 1,44 | 1,52 | 0,476813 |
| 7 | 1,491 | 1,493 | 1,026 | (-0,743; 3,729) | -0,003 | -0,00 | -0,00 | 0,688750 |
| 8 | 1,746 | 2,273 | 1,026 | (0,037; 4,509) | -0,527 | -0,76 | -0,75 | 0,688750 |
| 9 | 1,819 | 0,970 | 0,942 | (-1,084; 3,023) | 0,849 | 1,06 | 1,07 | 0,580949 |
| 10 | 2,487 | 2,480 | 0,942 | (0,427; 4,534) | 0,006 | 0,01 | 0,01 | 0,580949 |
| 11 | 0,460 | 2,099 | 0,807 | (0,341; 3,857) | -1,639 | -1,75 | -1,94 | 0,425781 |
| 12 | 2,050 | 2,531 | 0,807 | (0,773; 4,289) | -0,482 | -0,51 | -0,50 | 0,425781 |
| 13 | 1,302 | 1,378 | 0,933 | (-0,655; 3,410) | -0,076 | -0,09 | -0,09 | 0,569258 |
| 14 | 4,644 | 4,264 | 0,933 | (2,231; 6,297) | 0,380 | 0,47 | 0,45 | 0,569258 |
| 15 | 2,712 | 2,403 | 0,814 | (0,630; 4,176) | 0,308 | 0,33 | 0,32 | 0,433227 |
| 16 | 3,524 | 3,510 | 0,814 | (1,737; 5,284) | 0,014 | 0,01 | 0,01 | 0,433227 |
| 17 | 2,852 | 2,625 | 0,956 | (0,542; 4,708) | 0,226 | 0,29 | 0,28 | 0,597775 |
| 18 | 5,365 | 5,611 | 0,956 | (3,528; 7,694) | -0,246 | -0,31 | -0,30 | 0,597775 |
| 19 | 1,098 | 2,885 | 0,789 | (1,167; 4,604) | -1,787 | -1,88 | -2,14 | 0,407038 |
| 20 | 3,212 | 2,187 | 0,995 | (0,019; 4,355) | 1,025 | 1,40 | 1,46 | 0,647412 |
| 21 | 1,875 | 1,209 | 0,790 | (-0,512; 2,929) | 0,666 | 0,70 | 0,68 | 0,407933 |
| 22 | 1,577 | 1,924 | 1,118 | (-0,511; 4,359) | -0,347 | -0,66 | -0,64 | 0,817044 |
| 23 | 1,359 | 1,446 | 1,122 | (-0,999; 3,891) | -0,086 | -0,17 | -0,16 | 0,823434 |
| 24 | 1,152 | 1,099 | 1,045 | (-1,177; 3,376) | 0,052 | 0,08 | 0,08 | 0,713775 |
| 25 | 0,358 | 2,356 | 0,664 | (0,908; 3,804) | -1,998 | -1,92 | -2,20 | 0,288793 |
| 26 | 3,982 | 2,356 | 0,664 | (0,908; 3,804) | 1,626 | 1,56 | 1,67 | 0,288793 |
| 27 | 3,145 | 2,356 | 0,664 | (0,908; 3,804) | 0,789 | 0,76 | 0,74 | 0,288793 |

| Obs | Cook’s D | DFITS |
| --- | --- | --- |
| 1 | 0,00 | -0,06077 |
| 2 | 0,01 | -0,34293 |
| 3 | 0,04 | -0,74112 |
| 4 | 0,00 | 0,05250 |
| 5 | 0,02 | 0,47545 |
| 6 | 0,13 | 1,44681 |
| 7 | 0,00 | -0,00550 |
| 8 | 0,09 | -1,11611 |
| 9 | 0,10 | 1,25662 |
| 10 | 0,00 | 0,00914 |
| 11 | 0,15 | -1,67072 |
| 12 | 0,01 | -0,42865 |
| 13 | 0,00 | -0,10291 |
| 14 | 0,02 | 0,52058 |
| 15 | 0,01 | 0,27868 |
| 16 | 0,00 | 0,01236 |
| 17 | 0,01 | 0,33809 |
| 18 | 0,01 | -0,36834 |
| 19 | 0,16 | -1,77131 |
| 20 | 0,24 | 1,97807 |
| 21 | 0,02 | 0,56826 |
| 22 | 0,13 | -1,35391 |
| 23 | 0,01 | -0,34393 |
| 24 | 0,00 | 0,11920 |
| 25 | 0,10 | -1,40276 |
| 26 | 0,07 | 1,06541 |
| 27 | 0,02 | 0,47311 |

## Coded Coefficients

| Term | Coef | SE Coef | 95% CI | T-Value | P-Value | VIF |
| --- | --- | --- | --- | --- | --- | --- |
| Constant | 2,373 | 0,692 | (0,866; 3,881) | 3,43 | 0,005 |  |
| Lac | 1,341 | 0,572 | (0,095; 2,587) | 2,34 | 0,037 | 1,18 |
| HPMC\_Visc | -0,153 | 0,598 | (-1,455; 1,149) | -0,26 | 0,802 | 1,70 |
| HPMC\_HP | 0,462 | 0,585 | (-0,813; 1,736) | 0,79 | 0,445 | 1,26 |
| HPMC\_PS | 0,491 | 0,897 | (-1,463; 2,445) | 0,55 | 0,594 | 2,09 |
| Lac\*Lac | 1,90 | 1,14 | (-0,58; 4,38) | 1,67 | 0,120 | 1,30 |
| HPMC\_Visc\*HPMC\_Visc | 0,53 | 1,21 | (-2,11; 3,16) | 0,43 | 0,671 | 1,96 |
| HPMC\_HP\*HPMC\_HP | -0,89 | 1,15 | (-3,39; 1,61) | -0,77 | 0,454 | 1,81 |
| HPMC\_PS\*HPMC\_PS | -0,98 | 1,14 | (-3,47; 1,50) | -0,86 | 0,405 | 1,42 |
| Lac\*HPMC\_Visc | -1,39 | 1,25 | (-4,12; 1,34) | -1,11 | 0,290 | 1,49 |
| Lac\*HPMC\_HP | 1,47 | 1,32 | (-1,40; 4,33) | 1,11 | 0,287 | 1,17 |
| Lac\*HPMC\_PS | 0,38 | 2,03 | (-4,05; 4,80) | 0,19 | 0,855 | 1,42 |
| HPMC\_Visc\*HPMC\_HP | 0,15 | 1,50 | (-3,11; 3,41) | 0,10 | 0,921 | 2,73 |
| HPMC\_Visc\*HPMC\_PS | 1,59 | 2,11 | (-3,02; 6,20) | 0,75 | 0,466 | 2,74 |
| HPMC\_HP\*HPMC\_PS | 1,43 | 2,33 | (-3,65; 6,52) | 0,61 | 0,550 | 2,70 |

## Model Summary

| S | R-sq | R-sq(adj) | PRESS | R-sq(pred) | AICc | BIC |
| --- | --- | --- | --- | --- | --- | --- |
| 1,28781 | 62,88% | 19,57% | 67,6666 | 0,00% | 154,79 | 121,12 |

## Analysis of Variance

| Source | DF | Seq SS | Contribution | Adj SS | Adj MS | F-Value | P-Value |
| --- | --- | --- | --- | --- | --- | --- | --- |
| Model | 14 | 33,7093 | 62,88% | 33,7093 | 2,40781 | 1,45 | 0,262 |
| Linear | 4 | 14,0642 | 26,23% | 11,3900 | 2,84750 | 1,72 | 0,211 |
| Lac | 1 | 10,6500 | 19,87% | 9,1125 | 9,11250 | 5,49 | 0,037 |
| HPMC\_Visc | 1 | 1,2882 | 2,40% | 0,1087 | 0,10869 | 0,07 | 0,802 |
| HPMC\_HP | 1 | 2,1157 | 3,95% | 1,0331 | 1,03314 | 0,62 | 0,445 |
| HPMC\_PS | 1 | 0,0102 | 0,02% | 0,4977 | 0,49775 | 0,30 | 0,594 |
| Square | 4 | 12,3572 | 23,05% | 11,6572 | 2,91430 | 1,76 | 0,202 |
| Lac\*Lac | 1 | 8,2022 | 15,30% | 4,6415 | 4,64151 | 2,80 | 0,120 |
| HPMC\_Visc\*HPMC\_Visc | 1 | 0,0989 | 0,18% | 0,3138 | 0,31377 | 0,19 | 0,671 |
| HPMC\_HP\*HPMC\_HP | 1 | 1,2134 | 2,26% | 0,9927 | 0,99266 | 0,60 | 0,454 |
| HPMC\_PS\*HPMC\_PS | 1 | 2,8427 | 5,30% | 1,2367 | 1,23674 | 0,75 | 0,405 |
| 2-Way Interaction | 6 | 7,2879 | 13,59% | 7,2879 | 1,21464 | 0,73 | 0,633 |
| Lac\*HPMC\_Visc | 1 | 2,9477 | 5,50% | 2,0305 | 2,03052 | 1,22 | 0,290 |
| Lac\*HPMC\_HP | 1 | 2,3594 | 4,40% | 2,0577 | 2,05769 | 1,24 | 0,287 |
| Lac\*HPMC\_PS | 1 | 0,0577 | 0,11% | 0,0577 | 0,05774 | 0,03 | 0,855 |
| HPMC\_Visc\*HPMC\_HP | 1 | 0,0979 | 0,18% | 0,0169 | 0,01692 | 0,01 | 0,921 |
| HPMC\_Visc\*HPMC\_PS | 1 | 1,1983 | 2,24% | 0,9382 | 0,93818 | 0,57 | 0,466 |
| HPMC\_HP\*HPMC\_PS | 1 | 0,6269 | 1,17% | 0,6269 | 0,62686 | 0,38 | 0,550 |
| Error | 12 | 19,9015 | 37,12% | 19,9015 | 1,65846 |  |  |
| Lack-of-Fit | 10 | 11,2938 | 21,07% | 11,2938 | 1,12938 | 0,26 | 0,941 |
| Pure Error | 2 | 8,6077 | 16,06% | 8,6077 | 4,30384 |  |  |
| Total | 26 | 53,6108 | 100,00% |  |  |  |  |

## Regression Equation in Uncoded Units

|  |  |  |
| --- | --- | --- |
| F\_SD\_2.5h(150min) | = | 49 - 74,9 Lac - 0,00450 HPMC\_Visc - 0,0 HPMC\_HP - 0,07 HPMC\_PS + 30,4 Lac\*Lac + 0,000000 HPMC\_Visc\*HPMC\_Visc - 0,86 HPMC\_HP\*HPMC\_HP - 0,0182 HPMC\_PS\*HPMC\_PS - 0,00143 Lac\*HPMC\_Visc + 5,78 Lac\*HPMC\_HP + 0,21 Lac\*HPMC\_PS + 0,000038 HPMC\_Visc\*HPMC\_HP + 0,000056 HPMC\_Visc\*HPMC\_PS + 0,192 HPMC\_HP\*HPMC\_PS |

## Fits and Diagnostics for All Observations

| Obs | F\_SD\_2.5h(150min) | Fit | SE Fit | 95% CI | Resid | Std Resid | Del Resid |
| --- | --- | --- | --- | --- | --- | --- | --- |
| 1 | 2,206 | 2,273 | 1,043 | (0,000; 4,546) | -0,067 | -0,09 | -0,08 |
| 2 | 3,259 | 3,451 | 1,043 | (1,178; 5,724) | -0,192 | -0,25 | -0,24 |
| 3 | 1,540 | 1,911 | 1,100 | (-0,486; 4,309) | -0,371 | -0,55 | -0,54 |
| 4 | 1,023 | 0,974 | 1,100 | (-1,423; 3,372) | 0,049 | 0,07 | 0,07 |
| 5 | 2,526 | 1,982 | 0,889 | (0,044; 3,919) | 0,544 | 0,58 | 0,57 |
| 6 | 5,694 | 4,459 | 0,889 | (2,521; 6,396) | 1,235 | 1,33 | 1,37 |
| 7 | 1,584 | 1,578 | 1,069 | (-0,750; 3,907) | 0,005 | 0,01 | 0,01 |
| 8 | 1,938 | 2,481 | 1,069 | (0,153; 4,810) | -0,543 | -0,76 | -0,74 |
| 9 | 1,796 | 0,996 | 0,982 | (-1,143; 3,135) | 0,799 | 0,96 | 0,96 |
| 10 | 2,518 | 2,457 | 0,982 | (0,319; 4,596) | 0,061 | 0,07 | 0,07 |
| 11 | 0,651 | 2,155 | 0,840 | (0,324; 3,986) | -1,504 | -1,54 | -1,65 |
| 12 | 1,918 | 2,614 | 0,840 | (0,783; 4,445) | -0,696 | -0,71 | -0,70 |
| 13 | 1,302 | 1,372 | 0,972 | (-0,745; 3,489) | -0,069 | -0,08 | -0,08 |
| 14 | 4,896 | 4,481 | 0,972 | (2,364; 6,598) | 0,415 | 0,49 | 0,48 |
| 15 | 2,719 | 2,503 | 0,848 | (0,657; 4,350) | 0,216 | 0,22 | 0,21 |
| 16 | 3,911 | 3,806 | 0,848 | (1,960; 5,653) | 0,105 | 0,11 | 0,10 |
| 17 | 3,004 | 2,783 | 0,996 | (0,613; 4,952) | 0,222 | 0,27 | 0,26 |
| 18 | 5,581 | 5,800 | 0,996 | (3,630; 7,969) | -0,218 | -0,27 | -0,26 |
| 19 | 1,072 | 2,903 | 0,822 | (1,113; 4,694) | -1,831 | -1,85 | -2,09 |
| 20 | 3,414 | 2,338 | 1,036 | (0,080; 4,596) | 1,076 | 1,41 | 1,47 |
| 21 | 1,961 | 1,215 | 0,823 | (-0,577; 3,007) | 0,746 | 0,75 | 0,74 |
| 22 | 1,672 | 2,047 | 1,164 | (-0,489; 4,583) | -0,375 | -0,68 | -0,66 |
| 23 | 1,382 | 1,456 | 1,169 | (-1,090; 4,002) | -0,074 | -0,14 | -0,13 |
| 24 | 1,233 | 1,185 | 1,088 | (-1,186; 3,555) | 0,048 | 0,07 | 0,07 |
| 25 | 0,242 | 2,389 | 0,692 | (0,881; 3,897) | -2,147 | -1,98 | -2,31 |
| 26 | 4,290 | 2,389 | 0,692 | (0,881; 3,897) | 1,900 | 1,75 | 1,94 |
| 27 | 3,056 | 2,389 | 0,692 | (0,881; 3,897) | 0,667 | 0,61 | 0,60 |

| Obs | HI | Cook’s D | DFITS |
| --- | --- | --- | --- |
| 1 | 0,655994 | 0,00 | -0,11667 |
| 2 | 0,655994 | 0,01 | -0,33628 |
| 3 | 0,729945 | 0,06 | -0,88445 |
| 4 | 0,729945 | 0,00 | 0,11457 |
| 5 | 0,476813 | 0,02 | 0,54191 |
| 6 | 0,476813 | 0,11 | 1,31131 |
| 7 | 0,688750 | 0,00 | 0,01064 |
| 8 | 0,688750 | 0,08 | -1,10340 |
| 9 | 0,580949 | 0,08 | 1,12504 |
| 10 | 0,580949 | 0,00 | 0,08254 |
| 11 | 0,425781 | 0,12 | -1,41908 |
| 12 | 0,425781 | 0,03 | -0,60092 |
| 13 | 0,569258 | 0,00 | -0,09048 |
| 14 | 0,569258 | 0,02 | 0,54626 |
| 15 | 0,433227 | 0,00 | 0,18650 |
| 16 | 0,433227 | 0,00 | 0,09037 |
| 17 | 0,597775 | 0,01 | 0,31783 |
| 18 | 0,597775 | 0,01 | -0,31302 |
| 19 | 0,407038 | 0,16 | -1,73123 |
| 20 | 0,647412 | 0,24 | 1,99840 |
| 21 | 0,407933 | 0,03 | 0,61290 |
| 22 | 0,817044 | 0,14 | -1,40336 |
| 23 | 0,823434 | 0,01 | -0,28416 |
| 24 | 0,713775 | 0,00 | 0,10471 |
| 25 | 0,288793 | 0,11 | -1,46887 |
| 26 | 0,288793 | 0,08 | 1,23704 |
| 27 | 0,288793 | 0,01 | 0,38062 |

## Coded Coefficients

| Term | Coef | SE Coef | 95% CI | T-Value | P-Value | VIF |
| --- | --- | --- | --- | --- | --- | --- |
| Constant | 2,421 | 0,713 | (0,868; 3,974) | 3,40 | 0,005 |  |
| Lac | 1,396 | 0,589 | (0,112; 2,680) | 2,37 | 0,035 | 1,18 |
| HPMC\_Visc | -0,078 | 0,616 | (-1,419; 1,264) | -0,13 | 0,902 | 1,70 |
| HPMC\_HP | 0,538 | 0,602 | (-0,774; 1,851) | 0,89 | 0,389 | 1,26 |
| HPMC\_PS | 0,600 | 0,924 | (-1,413; 2,614) | 0,65 | 0,528 | 2,09 |
| Lac\*Lac | 2,01 | 1,17 | (-0,54; 4,56) | 1,71 | 0,112 | 1,30 |
| HPMC\_Visc\*HPMC\_Visc | 0,58 | 1,24 | (-2,13; 3,29) | 0,46 | 0,651 | 1,96 |
| HPMC\_HP\*HPMC\_HP | -0,87 | 1,18 | (-3,44; 1,70) | -0,74 | 0,476 | 1,81 |
| HPMC\_PS\*HPMC\_PS | -0,94 | 1,17 | (-3,50; 1,62) | -0,80 | 0,438 | 1,42 |
| Lac\*HPMC\_Visc | -1,31 | 1,29 | (-4,13; 1,50) | -1,02 | 0,329 | 1,49 |
| Lac\*HPMC\_HP | 1,63 | 1,36 | (-1,33; 4,58) | 1,20 | 0,254 | 1,17 |
| Lac\*HPMC\_PS | 0,55 | 2,09 | (-4,01; 5,11) | 0,26 | 0,796 | 1,42 |
| HPMC\_Visc\*HPMC\_HP | 0,25 | 1,54 | (-3,11; 3,61) | 0,16 | 0,874 | 2,73 |
| HPMC\_Visc\*HPMC\_PS | 1,78 | 2,18 | (-2,97; 6,52) | 0,82 | 0,430 | 2,74 |
| HPMC\_HP\*HPMC\_PS | 1,69 | 2,40 | (-3,55; 6,92) | 0,70 | 0,496 | 2,70 |

## Model Summary

| S | R-sq | R-sq(adj) | PRESS | R-sq(pred) | AICc | BIC |
| --- | --- | --- | --- | --- | --- | --- |
| 1,32682 | 63,03% | 19,90% | 70,7340 | 0,00% | 156,40 | 122,73 |

## Analysis of Variance

| Source | DF | Seq SS | Contribution | Adj SS | Adj MS | F-Value | P-Value |
| --- | --- | --- | --- | --- | --- | --- | --- |
| Model | 14 | 36,0169 | 63,03% | 36,0169 | 2,57263 | 1,46 | 0,258 |
| Linear | 4 | 14,6766 | 25,68% | 12,6010 | 3,15026 | 1,79 | 0,196 |
| Lac | 1 | 11,0139 | 19,27% | 9,8797 | 9,87973 | 5,61 | 0,035 |
| HPMC\_Visc | 1 | 1,1239 | 1,97% | 0,0281 | 0,02809 | 0,02 | 0,902 |
| HPMC\_HP | 1 | 2,5358 | 4,44% | 1,4057 | 1,40574 | 0,80 | 0,389 |
| HPMC\_PS | 1 | 0,0030 | 0,01% | 0,7434 | 0,74341 | 0,42 | 0,528 |
| Square | 4 | 12,9405 | 22,65% | 12,2855 | 3,07137 | 1,74 | 0,205 |
| Lac\*Lac | 1 | 8,8298 | 15,45% | 5,1705 | 5,17054 | 2,94 | 0,112 |
| HPMC\_Visc\*HPMC\_Visc | 1 | 0,0945 | 0,17% | 0,3798 | 0,37977 | 0,22 | 0,651 |
| HPMC\_HP\*HPMC\_HP | 1 | 1,1571 | 2,02% | 0,9524 | 0,95239 | 0,54 | 0,476 |
| HPMC\_PS\*HPMC\_PS | 1 | 2,8591 | 5,00% | 1,1344 | 1,13442 | 0,64 | 0,438 |
| 2-Way Interaction | 6 | 8,3998 | 14,70% | 8,3998 | 1,39996 | 0,80 | 0,591 |
| Lac\*HPMC\_Visc | 1 | 2,8340 | 4,96% | 1,8227 | 1,82271 | 1,04 | 0,329 |
| Lac\*HPMC\_HP | 1 | 2,9741 | 5,20% | 2,5323 | 2,53230 | 1,44 | 0,254 |
| Lac\*HPMC\_PS | 1 | 0,1227 | 0,21% | 0,1227 | 0,12271 | 0,07 | 0,796 |
| HPMC\_Visc\*HPMC\_HP | 1 | 0,0856 | 0,15% | 0,0459 | 0,04587 | 0,03 | 0,874 |
| HPMC\_Visc\*HPMC\_PS | 1 | 1,5171 | 2,65% | 1,1752 | 1,17524 | 0,67 | 0,430 |
| HPMC\_HP\*HPMC\_PS | 1 | 0,8662 | 1,52% | 0,8662 | 0,86622 | 0,49 | 0,496 |
| Error | 12 | 21,1253 | 36,97% | 21,1253 | 1,76044 |  |  |
| Lack-of-Fit | 10 | 11,1998 | 19,60% | 11,1998 | 1,11998 | 0,23 | 0,958 |
| Pure Error | 2 | 9,9255 | 17,37% | 9,9255 | 4,96274 |  |  |
| Total | 26 | 57,1422 | 100,00% |  |  |  |  |

## Regression Equation in Uncoded Units

|  |  |  |
| --- | --- | --- |
| F\_SD\_3h(180min) | = | 91 - 89,9 Lac - 0,00531 HPMC\_Visc - 3,3 HPMC\_HP - 0,62 HPMC\_PS + 32,1 Lac\*Lac + 0,000000 HPMC\_Visc\*HPMC\_Visc - 0,84 HPMC\_HP\*HPMC\_HP - 0,0174 HPMC\_PS\*HPMC\_PS - 0,00135 Lac\*HPMC\_Visc + 6,41 Lac\*HPMC\_HP + 0,30 Lac\*HPMC\_PS + 0,000063 HPMC\_Visc\*HPMC\_HP + 0,000062 HPMC\_Visc\*HPMC\_PS + 0,226 HPMC\_HP\*HPMC\_PS |

## Fits and Diagnostics for All Observations

| Obs | F\_SD\_3h(180min) | Fit | SE Fit | 95% CI | Resid | Std Resid | Del Resid | HI |
| --- | --- | --- | --- | --- | --- | --- | --- | --- |
| 1 | 2,375 | 2,456 | 1,075 | (0,114; 4,797) | -0,080 | -0,10 | -0,10 | 0,655994 |
| 2 | 3,270 | 3,479 | 1,075 | (1,137; 5,820) | -0,208 | -0,27 | -0,26 | 0,655994 |
| 3 | 1,617 | 2,006 | 1,134 | (-0,464; 4,476) | -0,389 | -0,56 | -0,55 | 0,729945 |
| 4 | 0,976 | 0,954 | 1,134 | (-1,516; 3,424) | 0,022 | 0,03 | 0,03 | 0,729945 |
| 5 | 2,604 | 2,022 | 0,916 | (0,026; 4,018) | 0,582 | 0,61 | 0,59 | 0,476813 |
| 6 | 5,741 | 4,510 | 0,916 | (2,514; 6,506) | 1,231 | 1,28 | 1,32 | 0,476813 |
| 7 | 1,658 | 1,613 | 1,101 | (-0,787; 4,012) | 0,045 | 0,06 | 0,06 | 0,688750 |
| 8 | 2,097 | 2,631 | 1,101 | (0,232; 5,031) | -0,535 | -0,72 | -0,71 | 0,688750 |
| 9 | 1,707 | 0,988 | 1,011 | (-1,215; 3,192) | 0,718 | 0,84 | 0,83 | 0,580949 |
| 10 | 2,506 | 2,418 | 1,011 | (0,214; 4,621) | 0,088 | 0,10 | 0,10 | 0,580949 |
| 11 | 0,812 | 2,197 | 0,866 | (0,310; 4,083) | -1,385 | -1,38 | -1,44 | 0,425781 |
| 12 | 1,867 | 2,685 | 0,866 | (0,798; 4,571) | -0,818 | -0,81 | -0,80 | 0,425781 |
| 13 | 1,422 | 1,398 | 1,001 | (-0,783; 3,579) | 0,024 | 0,03 | 0,03 | 0,569258 |
| 14 | 5,046 | 4,666 | 1,001 | (2,485; 6,848) | 0,379 | 0,44 | 0,42 | 0,569258 |
| 15 | 2,717 | 2,604 | 0,873 | (0,702; 4,507) | 0,113 | 0,11 | 0,11 | 0,433227 |
| 16 | 4,260 | 4,057 | 0,873 | (2,155; 5,960) | 0,203 | 0,20 | 0,19 | 0,433227 |
| 17 | 3,076 | 2,891 | 1,026 | (0,656; 5,127) | 0,185 | 0,22 | 0,21 | 0,597775 |
| 18 | 5,780 | 5,962 | 1,026 | (3,727; 8,198) | -0,182 | -0,22 | -0,21 | 0,597775 |
| 19 | 1,085 | 2,921 | 0,847 | (1,077; 4,766) | -1,836 | -1,80 | -2,01 | 0,407038 |
| 20 | 3,525 | 2,449 | 1,068 | (0,123; 4,775) | 1,076 | 1,37 | 1,42 | 0,647412 |
| 21 | 2,072 | 1,229 | 0,847 | (-0,618; 3,075) | 0,843 | 0,83 | 0,81 | 0,407933 |
| 22 | 1,762 | 2,189 | 1,199 | (-0,424; 4,802) | -0,427 | -0,75 | -0,74 | 0,817044 |
| 23 | 1,412 | 1,484 | 1,204 | (-1,139; 4,107) | -0,072 | -0,13 | -0,12 | 0,823434 |
| 24 | 1,311 | 1,264 | 1,121 | (-1,178; 3,706) | 0,048 | 0,07 | 0,06 | 0,713775 |
| 25 | 0,138 | 2,420 | 0,713 | (0,866; 3,973) | -2,282 | -2,04 | -2,41 | 0,288793 |
| 26 | 4,535 | 2,420 | 0,713 | (0,866; 3,973) | 2,115 | 1,89 | 2,16 | 0,288793 |
| 27 | 2,962 | 2,420 | 0,713 | (0,866; 3,973) | 0,542 | 0,48 | 0,47 | 0,288793 |

| Obs | Cook’s D | DFITS |  |
| --- | --- | --- | --- |
| 1 | 0,00 | -0,13656 |  |
| 2 | 0,01 | -0,35494 |  |
| 3 | 0,06 | -0,90083 |  |
| 4 | 0,00 | 0,05063 |  |
| 5 | 0,02 | 0,56311 |  |
| 6 | 0,10 | 1,26258 |  |
| 7 | 0,00 | 0,08720 |  |
| 8 | 0,08 | -1,05223 |  |
| 9 | 0,06 | 0,97169 |  |
| 10 | 0,00 | 0,11555 |  |
| 11 | 0,09 | -1,23783 |  |
| 12 | 0,03 | -0,68998 |  |
| 13 | 0,00 | 0,03050 |  |
| 14 | 0,02 | 0,48297 |  |
| 15 | 0,00 | 0,09459 |  |
| 16 | 0,00 | 0,17006 |  |
| 17 | 0,00 | 0,25676 |  |
| 18 | 0,00 | -0,25337 |  |
| 19 | 0,15 | -1,66782 |  |
| 20 | 0,23 | 1,92893 |  |
| 21 | 0,03 | 0,67561 |  |
| 22 | 0,17 | -1,55774 |  |
| 23 | 0,01 | -0,26804 |  |
| 24 | 0,00 | 0,10119 |  |
| 25 | 0,11 | -1,53883 | R |
| 26 | 0,10 | 1,37607 |  |
| 27 | 0,01 | 0,29856 |  |

R  Large residual

## Coded Coefficients

| Term | Coef | SE Coef | 95% CI | T-Value | P-Value | VIF |
| --- | --- | --- | --- | --- | --- | --- |
| Constant | 2,490 | 0,722 | (0,917; 4,063) | 3,45 | 0,005 |  |
| Lac | 1,479 | 0,597 | (0,178; 2,780) | 2,48 | 0,029 | 1,18 |
| HPMC\_Visc | -0,049 | 0,624 | (-1,409; 1,310) | -0,08 | 0,938 | 1,70 |
| HPMC\_HP | 0,613 | 0,610 | (-0,716; 1,943) | 1,01 | 0,335 | 1,26 |
| HPMC\_PS | 0,637 | 0,936 | (-1,403; 2,677) | 0,68 | 0,509 | 2,09 |
| Lac\*Lac | 2,06 | 1,19 | (-0,53; 4,64) | 1,73 | 0,109 | 1,30 |
| HPMC\_Visc\*HPMC\_Visc | 0,58 | 1,26 | (-2,17; 3,33) | 0,46 | 0,652 | 1,96 |
| HPMC\_HP\*HPMC\_HP | -0,87 | 1,20 | (-3,48; 1,73) | -0,73 | 0,480 | 1,81 |
| HPMC\_PS\*HPMC\_PS | -0,91 | 1,19 | (-3,50; 1,68) | -0,77 | 0,458 | 1,42 |
| Lac\*HPMC\_Visc | -1,17 | 1,31 | (-4,02; 1,69) | -0,89 | 0,391 | 1,49 |
| Lac\*HPMC\_HP | 1,82 | 1,37 | (-1,17; 4,81) | 1,32 | 0,210 | 1,17 |
| Lac\*HPMC\_PS | 0,82 | 2,12 | (-3,79; 5,44) | 0,39 | 0,704 | 1,42 |
| HPMC\_Visc\*HPMC\_HP | 0,31 | 1,56 | (-3,10; 3,71) | 0,20 | 0,847 | 2,73 |
| HPMC\_Visc\*HPMC\_PS | 1,80 | 2,21 | (-3,01; 6,60) | 0,81 | 0,432 | 2,74 |
| HPMC\_HP\*HPMC\_PS | 1,78 | 2,44 | (-3,52; 7,09) | 0,73 | 0,478 | 2,70 |

## Model Summary

| S | R-sq | R-sq(adj) | PRESS | R-sq(pred) | AICc | BIC |
| --- | --- | --- | --- | --- | --- | --- |
| 1,34432 | 63,75% | 21,45% | 72,2925 | 0,00% | 157,11 | 123,44 |

## Analysis of Variance

| Source | DF | Seq SS | Contribution | Adj SS | Adj MS | F-Value | P-Value |
| --- | --- | --- | --- | --- | --- | --- | --- |
| Model | 14 | 38,1306 | 63,75% | 38,1306 | 2,7236 | 1,51 | 0,241 |
| Linear | 4 | 15,7133 | 26,27% | 14,2525 | 3,5631 | 1,97 | 0,163 |
| Lac | 1 | 11,5864 | 19,37% | 11,0918 | 11,0918 | 6,14 | 0,029 |
| HPMC\_Visc | 1 | 1,0380 | 1,74% | 0,0112 | 0,0112 | 0,01 | 0,938 |
| HPMC\_HP | 1 | 3,0885 | 5,16% | 1,8255 | 1,8255 | 1,01 | 0,335 |
| HPMC\_PS | 1 | 0,0004 | 0,00% | 0,8368 | 0,8368 | 0,46 | 0,509 |
| Square | 4 | 13,1571 | 22,00% | 12,5591 | 3,1398 | 1,74 | 0,206 |
| Lac\*Lac | 1 | 9,1653 | 15,32% | 5,4313 | 5,4313 | 3,01 | 0,109 |
| HPMC\_Visc\*HPMC\_Visc | 1 | 0,0952 | 0,16% | 0,3860 | 0,3860 | 0,21 | 0,652 |
| HPMC\_HP\*HPMC\_HP | 1 | 1,1436 | 1,91% | 0,9622 | 0,9622 | 0,53 | 0,480 |
| HPMC\_PS\*HPMC\_PS | 1 | 2,7530 | 4,60% | 1,0622 | 1,0622 | 0,59 | 0,458 |
| 2-Way Interaction | 6 | 9,2602 | 15,48% | 9,2602 | 1,5434 | 0,85 | 0,554 |
| Lac\*HPMC\_Visc | 1 | 2,5388 | 4,24% | 1,4331 | 1,4331 | 0,79 | 0,391 |
| Lac\*HPMC\_HP | 1 | 3,8490 | 6,43% | 3,1708 | 3,1708 | 1,75 | 0,210 |
| Lac\*HPMC\_PS | 1 | 0,2734 | 0,46% | 0,2734 | 0,2734 | 0,15 | 0,704 |
| HPMC\_Visc\*HPMC\_HP | 1 | 0,0709 | 0,12% | 0,0707 | 0,0707 | 0,04 | 0,847 |
| HPMC\_Visc\*HPMC\_PS | 1 | 1,5607 | 2,61% | 1,1965 | 1,1965 | 0,66 | 0,432 |
| HPMC\_HP\*HPMC\_PS | 1 | 0,9673 | 1,62% | 0,9673 | 0,9673 | 0,54 | 0,478 |
| Error | 12 | 21,6864 | 36,25% | 21,6864 | 1,8072 |  |  |
| Lack-of-Fit | 10 | 11,2359 | 18,78% | 11,2359 | 1,1236 | 0,22 | 0,963 |
| Pure Error | 2 | 10,4506 | 17,47% | 10,4506 | 5,2253 |  |  |
| Total | 26 | 59,8171 | 100,00% |  |  |  |  |

## Regression Equation in Uncoded Units

|  |  |  |
| --- | --- | --- |
| F\_SD\_3.5h(210min) | = | 113 - 110,0 Lac - 0,00557 HPMC\_Visc - 4,6 HPMC\_HP - 0,90 HPMC\_PS + 32,9 Lac\*Lac + 0,000000 HPMC\_Visc\*HPMC\_Visc - 0,85 HPMC\_HP\*HPMC\_HP - 0,0169 HPMC\_PS\*HPMC\_PS - 0,00120 Lac\*HPMC\_Visc + 7,17 Lac\*HPMC\_HP + 0,45 Lac\*HPMC\_PS + 0,000078 HPMC\_Visc\*HPMC\_HP + 0,000063 HPMC\_Visc\*HPMC\_PS + 0,239 HPMC\_HP\*HPMC\_PS |

## Fits and Diagnostics for All Observations

| Obs | F\_SD\_3.5h(210min) | Fit | SE Fit | 95% CI | Resid | Std Resid | Del Resid |
| --- | --- | --- | --- | --- | --- | --- | --- |
| 1 | 2,522 | 2,621 | 1,089 | (0,249; 4,994) | -0,099 | -0,13 | -0,12 |
| 2 | 3,262 | 3,414 | 1,089 | (1,042; 5,787) | -0,153 | -0,19 | -0,19 |
| 3 | 1,694 | 2,102 | 1,149 | (-0,400; 4,605) | -0,409 | -0,58 | -0,57 |
| 4 | 0,947 | 0,933 | 1,149 | (-1,569; 3,436) | 0,014 | 0,02 | 0,02 |
| 5 | 2,736 | 2,097 | 0,928 | (0,075; 4,120) | 0,639 | 0,66 | 0,64 |
| 6 | 5,756 | 4,565 | 0,928 | (2,542; 6,587) | 1,191 | 1,22 | 1,25 |
| 7 | 1,729 | 1,653 | 1,116 | (-0,778; 4,083) | 0,076 | 0,10 | 0,10 |
| 8 | 2,304 | 2,846 | 1,116 | (0,415; 5,277) | -0,542 | -0,72 | -0,71 |
| 9 | 1,695 | 1,036 | 1,025 | (-1,197; 3,268) | 0,660 | 0,76 | 0,74 |
| 10 | 2,498 | 2,426 | 1,025 | (0,194; 4,659) | 0,072 | 0,08 | 0,08 |
| 11 | 0,934 | 2,203 | 0,877 | (0,292; 4,115) | -1,270 | -1,25 | -1,28 |
| 12 | 1,818 | 2,768 | 0,877 | (0,857; 4,679) | -0,950 | -0,93 | -0,93 |
| 13 | 1,470 | 1,416 | 1,014 | (-0,794; 3,626) | 0,053 | 0,06 | 0,06 |
| 14 | 5,236 | 4,884 | 1,014 | (2,674; 7,094) | 0,352 | 0,40 | 0,38 |
| 15 | 2,553 | 2,612 | 0,885 | (0,684; 4,540) | -0,059 | -0,06 | -0,06 |
| 16 | 4,551 | 4,296 | 0,885 | (2,368; 6,224) | 0,254 | 0,25 | 0,24 |
| 17 | 3,148 | 2,966 | 1,039 | (0,701; 5,230) | 0,183 | 0,21 | 0,21 |
| 18 | 5,967 | 6,107 | 1,039 | (3,843; 8,372) | -0,140 | -0,16 | -0,16 |
| 19 | 1,110 | 2,967 | 0,858 | (1,098; 4,836) | -1,857 | -1,79 | -2,01 |
| 20 | 3,635 | 2,523 | 1,082 | (0,166; 4,880) | 1,111 | 1,39 | 1,46 |
| 21 | 2,104 | 1,231 | 0,859 | (-0,640; 3,102) | 0,873 | 0,84 | 0,83 |
| 22 | 1,898 | 2,325 | 1,215 | (-0,322; 4,973) | -0,427 | -0,74 | -0,73 |
| 23 | 1,445 | 1,547 | 1,220 | (-1,111; 4,205) | -0,102 | -0,18 | -0,17 |
| 24 | 1,463 | 1,362 | 1,136 | (-1,113; 3,836) | 0,101 | 0,14 | 0,13 |
| 25 | 0,199 | 2,479 | 0,722 | (0,905; 4,053) | -2,280 | -2,01 | -2,36 |
| 26 | 4,741 | 2,479 | 0,722 | (0,905; 4,053) | 2,262 | 1,99 | 2,34 |
| 27 | 2,925 | 2,479 | 0,722 | (0,905; 4,053) | 0,446 | 0,39 | 0,38 |

| Obs | HI | Cook’s D | DFITS |  |
| --- | --- | --- | --- | --- |
| 1 | 0,655994 | 0,00 | -0,16570 |  |
| 2 | 0,655994 | 0,00 | -0,25671 |  |
| 3 | 0,729945 | 0,06 | -0,93387 |  |
| 4 | 0,729945 | 0,00 | 0,03055 |  |
| 5 | 0,476813 | 0,03 | 0,61160 |  |
| 6 | 0,476813 | 0,09 | 1,19653 |  |
| 7 | 0,688750 | 0,00 | 0,14417 |  |
| 8 | 0,688750 | 0,08 | -1,05264 |  |
| 9 | 0,580949 | 0,05 | 0,87559 |  |
| 10 | 0,580949 | 0,00 | 0,09357 |  |
| 11 | 0,425781 | 0,08 | -1,10117 |  |
| 12 | 0,425781 | 0,04 | -0,79823 |  |
| 13 | 0,569258 | 0,00 | 0,06632 |  |
| 14 | 0,569258 | 0,01 | 0,44167 |  |
| 15 | 0,433227 | 0,00 | -0,04853 |  |
| 16 | 0,433227 | 0,00 | 0,21094 |  |
| 17 | 0,597775 | 0,00 | 0,25063 |  |
| 18 | 0,597775 | 0,00 | -0,19207 |  |
| 19 | 0,407038 | 0,15 | -1,66281 |  |
| 20 | 0,647412 | 0,24 | 1,97285 |  |
| 21 | 0,407933 | 0,03 | 0,69151 |  |
| 22 | 0,817044 | 0,16 | -1,53863 |  |
| 23 | 0,823434 | 0,01 | -0,37445 |  |
| 24 | 0,713775 | 0,00 | 0,21287 |  |
| 25 | 0,288793 | 0,11 | -1,50681 | R |
| 26 | 0,288793 | 0,11 | 1,48865 |  |
| 27 | 0,288793 | 0,00 | 0,24171 |  |

R  Large residual

## Coded Coefficients

| Term | Coef | SE Coef | 95% CI | T-Value | P-Value | VIF |
| --- | --- | --- | --- | --- | --- | --- |
| Constant | 2,527 | 0,714 | (0,972; 4,083) | 3,54 | 0,004 |  |
| Lac | 1,501 | 0,590 | (0,214; 2,787) | 2,54 | 0,026 | 1,18 |
| HPMC\_Visc | -0,001 | 0,617 | (-1,345; 1,343) | -0,00 | 0,998 | 1,70 |
| HPMC\_HP | 0,654 | 0,604 | (-0,661; 1,969) | 1,08 | 0,300 | 1,26 |
| HPMC\_PS | 0,768 | 0,926 | (-1,248; 2,785) | 0,83 | 0,423 | 2,09 |
| Lac\*Lac | 2,14 | 1,17 | (-0,42; 4,69) | 1,82 | 0,093 | 1,30 |
| HPMC\_Visc\*HPMC\_Visc | 0,68 | 1,25 | (-2,03; 3,40) | 0,55 | 0,594 | 1,96 |
| HPMC\_HP\*HPMC\_HP | -0,86 | 1,18 | (-3,44; 1,72) | -0,73 | 0,482 | 1,81 |
| HPMC\_PS\*HPMC\_PS | -0,83 | 1,18 | (-3,39; 1,74) | -0,70 | 0,496 | 1,42 |
| Lac\*HPMC\_Visc | -0,82 | 1,29 | (-3,64; 2,00) | -0,63 | 0,539 | 1,49 |
| Lac\*HPMC\_HP | 2,09 | 1,36 | (-0,87; 5,05) | 1,54 | 0,150 | 1,17 |
| Lac\*HPMC\_PS | 1,30 | 2,10 | (-3,26; 5,87) | 0,62 | 0,545 | 1,42 |
| HPMC\_Visc\*HPMC\_HP | 0,79 | 1,54 | (-2,57; 4,16) | 0,51 | 0,618 | 2,73 |
| HPMC\_Visc\*HPMC\_PS | 2,08 | 2,18 | (-2,68; 6,83) | 0,95 | 0,360 | 2,74 |
| HPMC\_HP\*HPMC\_PS | 2,52 | 2,41 | (-2,72; 7,77) | 1,05 | 0,315 | 2,70 |

## Model Summary

| S | R-sq | R-sq(adj) | PRESS | R-sq(pred) | AICc | BIC |
| --- | --- | --- | --- | --- | --- | --- |
| 1,32920 | 65,26% | 24,72% | 73,0498 | 0,00% | 156,49 | 122,83 |

## Analysis of Variance

| Source | DF | Seq SS | Contribution | Adj SS | Adj MS | F-Value | P-Value |
| --- | --- | --- | --- | --- | --- | --- | --- |
| Model | 14 | 39,8186 | 65,26% | 39,8186 | 2,8442 | 1,61 | 0,207 |
| Linear | 4 | 14,5930 | 23,92% | 15,1841 | 3,7960 | 2,15 | 0,137 |
| Lac | 1 | 10,5133 | 17,23% | 11,4158 | 11,4158 | 6,46 | 0,026 |
| HPMC\_Visc | 1 | 1,3921 | 2,28% | 0,0000 | 0,0000 | 0,00 | 0,998 |
| HPMC\_HP | 1 | 2,6710 | 4,38% | 2,0775 | 2,0775 | 1,18 | 0,300 |
| HPMC\_PS | 1 | 0,0165 | 0,03% | 1,2176 | 1,2176 | 0,69 | 0,423 |
| Square | 4 | 13,2925 | 21,78% | 12,9490 | 3,2372 | 1,83 | 0,188 |
| Lac\*Lac | 1 | 9,5064 | 15,58% | 5,8666 | 5,8666 | 3,32 | 0,093 |
| HPMC\_Visc\*HPMC\_Visc | 1 | 0,1279 | 0,21% | 0,5309 | 0,5309 | 0,30 | 0,594 |
| HPMC\_HP\*HPMC\_HP | 1 | 0,9001 | 1,48% | 0,9293 | 0,9293 | 0,53 | 0,482 |
| HPMC\_PS\*HPMC\_PS | 1 | 2,7581 | 4,52% | 0,8726 | 0,8726 | 0,49 | 0,496 |
| 2-Way Interaction | 6 | 11,9331 | 19,56% | 11,9331 | 1,9888 | 1,13 | 0,404 |
| Lac\*HPMC\_Visc | 1 | 1,7870 | 2,93% | 0,7056 | 0,7056 | 0,40 | 0,539 |
| Lac\*HPMC\_HP | 1 | 5,3249 | 8,73% | 4,1771 | 4,1771 | 2,36 | 0,150 |
| Lac\*HPMC\_PS | 1 | 0,6845 | 1,12% | 0,6845 | 0,6845 | 0,39 | 0,545 |
| HPMC\_Visc\*HPMC\_HP | 1 | 0,0001 | 0,00% | 0,4640 | 0,4640 | 0,26 | 0,618 |
| HPMC\_Visc\*HPMC\_PS | 1 | 2,1949 | 3,60% | 1,5985 | 1,5985 | 0,90 | 0,360 |
| HPMC\_HP\*HPMC\_PS | 1 | 1,9418 | 3,18% | 1,9418 | 1,9418 | 1,10 | 0,315 |
| Error | 12 | 21,2013 | 34,74% | 21,2013 | 1,7668 |  |  |
| Lack-of-Fit | 10 | 10,9853 | 18,00% | 10,9853 | 1,0985 | 0,22 | 0,963 |
| Pure Error | 2 | 10,2160 | 16,74% | 10,2160 | 5,1080 |  |  |
| Total | 26 | 61,0199 | 100,00% |  |  |  |  |

## Regression Equation in Uncoded Units

|  |  |  |
| --- | --- | --- |
| F\_SD\_4h(240min) | = | 229 - 144,3 Lac - 0,00775 HPMC\_Visc - 13,9 HPMC\_HP - 2,31 HPMC\_PS + 34,2 Lac\*Lac + 0,000000 HPMC\_Visc\*HPMC\_Visc - 0,83 HPMC\_HP\*HPMC\_HP - 0,0153 HPMC\_PS\*HPMC\_PS - 0,00084 Lac\*HPMC\_Visc + 8,23 Lac\*HPMC\_HP + 0,71 Lac\*HPMC\_PS + 0,000200 HPMC\_Visc\*HPMC\_HP + 0,000073 HPMC\_Visc\*HPMC\_PS + 0,338 HPMC\_HP\*HPMC\_PS |

## Fits and Diagnostics for All Observations

| Obs | F\_SD\_4h(240min) | Fit | SE Fit | 95% CI | Resid | Std Resid | Del Resid | HI |
| --- | --- | --- | --- | --- | --- | --- | --- | --- |
| 1 | 3,590 | 3,346 | 1,077 | (1,000; 5,691) | 0,244 | 0,31 | 0,30 | 0,655994 |
| 2 | 3,304 | 3,600 | 1,077 | (1,255; 5,946) | -0,297 | -0,38 | -0,37 | 0,655994 |
| 3 | 1,736 | 2,242 | 1,136 | (-0,232; 4,716) | -0,505 | -0,73 | -0,72 | 0,729945 |
| 4 | 0,962 | 0,854 | 1,136 | (-1,620; 3,328) | 0,108 | 0,16 | 0,15 | 0,729945 |
| 5 | 2,780 | 2,212 | 0,918 | (0,212; 4,212) | 0,568 | 0,59 | 0,57 | 0,476813 |
| 6 | 5,733 | 4,448 | 0,918 | (2,448; 6,448) | 1,285 | 1,34 | 1,39 | 0,476813 |
| 7 | 1,835 | 1,623 | 1,103 | (-0,780; 4,027) | 0,212 | 0,29 | 0,27 | 0,688750 |
| 8 | 2,436 | 3,026 | 1,103 | (0,622; 5,429) | -0,590 | -0,79 | -0,78 | 0,688750 |
| 9 | 1,672 | 1,196 | 1,013 | (-1,012; 3,403) | 0,476 | 0,55 | 0,54 | 0,580949 |
| 10 | 2,478 | 2,383 | 1,013 | (0,176; 4,590) | 0,095 | 0,11 | 0,11 | 0,580949 |
| 11 | 1,058 | 2,152 | 0,867 | (0,262; 4,041) | -1,093 | -1,09 | -1,09 | 0,425781 |
| 12 | 1,842 | 2,777 | 0,867 | (0,888; 4,667) | -0,936 | -0,93 | -0,92 | 0,425781 |
| 13 | 1,556 | 1,450 | 1,003 | (-0,735; 3,635) | 0,107 | 0,12 | 0,12 | 0,569258 |
| 14 | 5,364 | 5,055 | 1,003 | (2,870; 7,240) | 0,310 | 0,35 | 0,34 | 0,569258 |
| 15 | 2,538 | 2,672 | 0,875 | (0,765; 4,578) | -0,134 | -0,13 | -0,13 | 0,433227 |
| 16 | 4,690 | 4,650 | 0,875 | (2,744; 6,556) | 0,041 | 0,04 | 0,04 | 0,433227 |
| 17 | 3,204 | 3,155 | 1,028 | (0,916; 5,394) | 0,049 | 0,06 | 0,06 | 0,597775 |
| 18 | 6,124 | 6,147 | 1,028 | (3,907; 8,386) | -0,022 | -0,03 | -0,03 | 0,597775 |
| 19 | 1,161 | 3,072 | 0,848 | (1,224; 4,919) | -1,911 | -1,87 | -2,12 | 0,407038 |
| 20 | 3,679 | 2,581 | 1,070 | (0,251; 4,912) | 1,097 | 1,39 | 1,45 | 0,647412 |
| 21 | 2,159 | 1,395 | 0,849 | (-0,455; 3,244) | 0,764 | 0,75 | 0,73 | 0,407933 |
| 22 | 1,999 | 2,405 | 1,201 | (-0,213; 5,023) | -0,406 | -0,71 | -0,70 | 0,817044 |
| 23 | 1,482 | 1,667 | 1,206 | (-0,961; 4,295) | -0,185 | -0,33 | -0,32 | 0,823434 |
| 24 | 1,619 | 1,423 | 1,123 | (-1,024; 3,869) | 0,196 | 0,28 | 0,27 | 0,713775 |
| 25 | 0,362 | 2,513 | 0,714 | (0,956; 4,069) | -2,151 | -1,92 | -2,21 | 0,288793 |
| 26 | 4,876 | 2,513 | 0,714 | (0,956; 4,069) | 2,363 | 2,11 | 2,54 | 0,288793 |
| 27 | 2,824 | 2,513 | 0,714 | (0,956; 4,069) | 0,312 | 0,28 | 0,27 | 0,288793 |

| Obs | Cook’s D | DFITS |  |
| --- | --- | --- | --- |
| 1 | 0,01 | 0,41553 |  |
| 2 | 0,02 | -0,50615 |  |
| 3 | 0,10 | -1,17818 |  |
| 4 | 0,00 | 0,24700 |  |
| 5 | 0,02 | 0,54768 |  |
| 6 | 0,11 | 1,32470 |  |
| 7 | 0,01 | 0,40891 |  |
| 8 | 0,09 | -1,16332 |  |
| 9 | 0,03 | 0,63186 |  |
| 10 | 0,00 | 0,12493 |  |
| 11 | 0,06 | -0,94239 |  |
| 12 | 0,04 | -0,79490 |  |
| 13 | 0,00 | 0,13464 |  |
| 14 | 0,01 | 0,39262 |  |
| 15 | 0,00 | -0,11201 |  |
| 16 | 0,00 | 0,03393 |  |
| 17 | 0,00 | 0,06839 |  |
| 18 | 0,00 | -0,03082 |  |
| 19 | 0,16 | -1,75766 |  |
| 20 | 0,24 | 1,96918 |  |
| 21 | 0,03 | 0,60823 |  |
| 22 | 0,15 | -1,47597 |  |
| 23 | 0,03 | -0,68628 |  |
| 24 | 0,01 | 0,41876 |  |
| 25 | 0,10 | -1,40586 |  |
| 26 | 0,12 | 1,62106 | R |
| 27 | 0,00 | 0,17023 |  |

R  Large residual

## Coded Coefficients

| Term | Coef | SE Coef | 95% CI | T-Value | P-Value | VIF |
| --- | --- | --- | --- | --- | --- | --- |
| Constant | 2,608 | 0,705 | (1,071; 4,145) | 3,70 | 0,003 |  |
| Lac | 1,508 | 0,583 | (0,237; 2,779) | 2,59 | 0,024 | 1,18 |
| HPMC\_Visc | 0,031 | 0,610 | (-1,297; 1,359) | 0,05 | 0,960 | 1,70 |
| HPMC\_HP | 0,715 | 0,596 | (-0,584; 2,014) | 1,20 | 0,254 | 1,26 |
| HPMC\_PS | 0,778 | 0,915 | (-1,215; 2,771) | 0,85 | 0,412 | 2,09 |
| Lac\*Lac | 2,19 | 1,16 | (-0,33; 4,72) | 1,89 | 0,083 | 1,30 |
| HPMC\_Visc\*HPMC\_Visc | 0,64 | 1,23 | (-2,05; 3,32) | 0,52 | 0,614 | 1,96 |
| HPMC\_HP\*HPMC\_HP | -0,89 | 1,17 | (-3,44; 1,66) | -0,76 | 0,460 | 1,81 |
| HPMC\_PS\*HPMC\_PS | -0,82 | 1,16 | (-3,35; 1,71) | -0,71 | 0,494 | 1,42 |
| Lac\*HPMC\_Visc | -0,76 | 1,28 | (-3,54; 2,03) | -0,59 | 0,564 | 1,49 |
| Lac\*HPMC\_HP | 2,11 | 1,34 | (-0,81; 5,04) | 1,57 | 0,142 | 1,17 |
| Lac\*HPMC\_PS | 1,57 | 2,07 | (-2,94; 6,09) | 0,76 | 0,463 | 1,42 |
| HPMC\_Visc\*HPMC\_HP | 0,88 | 1,53 | (-2,44; 4,21) | 0,58 | 0,573 | 2,73 |
| HPMC\_Visc\*HPMC\_PS | 2,02 | 2,16 | (-2,68; 6,72) | 0,94 | 0,367 | 2,74 |
| HPMC\_HP\*HPMC\_PS | 2,59 | 2,38 | (-2,59; 7,78) | 1,09 | 0,297 | 2,70 |

## Model Summary

| S | R-sq | R-sq(adj) | PRESS | R-sq(pred) | AICc | BIC |
| --- | --- | --- | --- | --- | --- | --- |
| 1,31335 | 66,58% | 27,59% | 71,7914 | 0,00% | 155,85 | 122,18 |

## Analysis of Variance

| Source | DF | Seq SS | Contribution | Adj SS | Adj MS | F-Value | P-Value |
| --- | --- | --- | --- | --- | --- | --- | --- |
| Model | 14 | 41,2398 | 66,58% | 41,2398 | 2,9457 | 1,71 | 0,179 |
| Linear | 4 | 14,7932 | 23,88% | 15,6064 | 3,9016 | 2,26 | 0,123 |
| Lac | 1 | 10,4120 | 16,81% | 11,5288 | 11,5288 | 6,68 | 0,024 |
| HPMC\_Visc | 1 | 1,2266 | 1,98% | 0,0045 | 0,0045 | 0,00 | 0,960 |
| HPMC\_HP | 1 | 3,1449 | 5,08% | 2,4785 | 2,4785 | 1,44 | 0,254 |
| HPMC\_PS | 1 | 0,0097 | 0,02% | 1,2484 | 1,2484 | 0,72 | 0,412 |
| Square | 4 | 13,7947 | 22,27% | 13,4188 | 3,3547 | 1,94 | 0,168 |
| Lac\*Lac | 1 | 10,0596 | 16,24% | 6,1700 | 6,1700 | 3,58 | 0,083 |
| HPMC\_Visc\*HPMC\_Visc | 1 | 0,1064 | 0,17% | 0,4633 | 0,4633 | 0,27 | 0,614 |
| HPMC\_HP\*HPMC\_HP | 1 | 0,9260 | 1,49% | 1,0048 | 1,0048 | 0,58 | 0,460 |
| HPMC\_PS\*HPMC\_PS | 1 | 2,7027 | 4,36% | 0,8590 | 0,8590 | 0,50 | 0,494 |
| 2-Way Interaction | 6 | 12,6518 | 20,43% | 12,6518 | 2,1086 | 1,22 | 0,360 |
| Lac\*HPMC\_Visc | 1 | 1,8606 | 3,00% | 0,6062 | 0,6062 | 0,35 | 0,564 |
| Lac\*HPMC\_HP | 1 | 5,6313 | 9,09% | 4,2724 | 4,2724 | 2,48 | 0,142 |
| Lac\*HPMC\_PS | 1 | 0,9936 | 1,60% | 0,9936 | 0,9936 | 0,58 | 0,463 |
| HPMC\_Visc\*HPMC\_HP | 1 | 0,0038 | 0,01% | 0,5783 | 0,5783 | 0,34 | 0,573 |
| HPMC\_Visc\*HPMC\_PS | 1 | 2,1132 | 3,41% | 1,5160 | 1,5160 | 0,88 | 0,367 |
| HPMC\_HP\*HPMC\_PS | 1 | 2,0494 | 3,31% | 2,0494 | 2,0494 | 1,19 | 0,297 |
| Error | 12 | 20,6988 | 33,42% | 20,6988 | 1,7249 |  |  |
| Lack-of-Fit | 10 | 10,5278 | 17,00% | 10,5278 | 1,0528 | 0,21 | 0,966 |
| Pure Error | 2 | 10,1710 | 16,42% | 10,1710 | 5,0855 |  |  |
| Total | 26 | 61,9386 | 100,00% |  |  |  |  |

## Regression Equation in Uncoded Units

|  |  |  |
| --- | --- | --- |
| F\_SD\_4.5h(270min) | = | 239 - 156,9 Lac - 0,00778 HPMC\_Visc - 14,3 HPMC\_HP - 2,46 HPMC\_PS + 35,1 Lac\*Lac + 0,000000 HPMC\_Visc\*HPMC\_Visc - 0,87 HPMC\_HP\*HPMC\_HP - 0,0152 HPMC\_PS\*HPMC\_PS - 0,00078 Lac\*HPMC\_Visc + 8,32 Lac\*HPMC\_HP + 0,85 Lac\*HPMC\_PS + 0,000224 HPMC\_Visc\*HPMC\_HP + 0,000071 HPMC\_Visc\*HPMC\_PS + 0,347 HPMC\_HP\*HPMC\_PS |

## Fits and Diagnostics for All Observations

| Obs | F\_SD\_4.5h(270min) | Fit | SE Fit | 95% CI | Resid | Std Resid | Del Resid |
| --- | --- | --- | --- | --- | --- | --- | --- |
| 1 | 3,719 | 3,440 | 1,064 | (1,122; 5,758) | 0,279 | 0,36 | 0,35 |
| 2 | 3,300 | 3,576 | 1,064 | (1,258; 5,894) | -0,276 | -0,36 | -0,34 |
| 3 | 1,817 | 2,335 | 1,122 | (-0,110; 4,780) | -0,518 | -0,76 | -0,75 |
| 4 | 0,927 | 0,823 | 1,122 | (-1,622; 3,268) | 0,104 | 0,15 | 0,15 |
| 5 | 2,851 | 2,291 | 0,907 | (0,315; 4,267) | 0,560 | 0,59 | 0,57 |
| 6 | 5,701 | 4,455 | 0,907 | (2,479; 6,431) | 1,247 | 1,31 | 1,36 |
| 7 | 2,055 | 1,791 | 1,090 | (-0,584; 4,166) | 0,264 | 0,36 | 0,35 |
| 8 | 2,561 | 3,150 | 1,090 | (0,776; 5,525) | -0,589 | -0,80 | -0,79 |
| 9 | 1,609 | 1,203 | 1,001 | (-0,978; 3,384) | 0,406 | 0,48 | 0,46 |
| 10 | 2,514 | 2,451 | 1,001 | (0,270; 4,632) | 0,063 | 0,07 | 0,07 |
| 11 | 1,195 | 2,167 | 0,857 | (0,300; 4,034) | -0,972 | -0,98 | -0,97 |
| 12 | 1,899 | 2,857 | 0,857 | (0,989; 4,724) | -0,957 | -0,96 | -0,96 |
| 13 | 1,622 | 1,476 | 0,991 | (-0,683; 3,635) | 0,146 | 0,17 | 0,16 |
| 14 | 5,475 | 5,195 | 0,991 | (3,036; 7,354) | 0,280 | 0,32 | 0,31 |
| 15 | 2,481 | 2,738 | 0,864 | (0,854; 4,621) | -0,256 | -0,26 | -0,25 |
| 16 | 4,851 | 4,816 | 0,864 | (2,932; 6,699) | 0,035 | 0,04 | 0,03 |
| 17 | 3,317 | 3,294 | 1,015 | (1,081; 5,506) | 0,023 | 0,03 | 0,03 |
| 18 | 6,281 | 6,257 | 1,015 | (4,044; 8,469) | 0,024 | 0,03 | 0,03 |
| 19 | 1,209 | 3,082 | 0,838 | (1,257; 4,908) | -1,874 | -1,85 | -2,10 |
| 20 | 3,713 | 2,630 | 1,057 | (0,328; 4,933) | 1,083 | 1,39 | 1,45 |
| 21 | 2,151 | 1,395 | 0,839 | (-0,432; 3,223) | 0,756 | 0,75 | 0,73 |
| 22 | 2,102 | 2,501 | 1,187 | (-0,085; 5,088) | -0,400 | -0,71 | -0,70 |
| 23 | 1,513 | 1,721 | 1,192 | (-0,876; 4,317) | -0,207 | -0,38 | -0,36 |
| 24 | 1,738 | 1,503 | 1,110 | (-0,915; 3,920) | 0,235 | 0,33 | 0,32 |
| 25 | 0,501 | 2,582 | 0,706 | (1,045; 4,120) | -2,081 | -1,88 | -2,14 |
| 26 | 5,011 | 2,582 | 0,706 | (1,045; 4,120) | 2,429 | 2,19 | 2,71 |
| 27 | 2,782 | 2,582 | 0,706 | (1,045; 4,120) | 0,200 | 0,18 | 0,17 |

| Obs | HI | Cook’s D | DFITS |  |
| --- | --- | --- | --- | --- |
| 1 | 0,655994 | 0,02 | 0,48190 |  |
| 2 | 0,655994 | 0,02 | -0,47626 |  |
| 3 | 0,729945 | 0,10 | -1,22496 |  |
| 4 | 0,729945 | 0,00 | 0,23907 |  |
| 5 | 0,476813 | 0,02 | 0,54631 |  |
| 6 | 0,476813 | 0,10 | 1,29627 |  |
| 7 | 0,688750 | 0,02 | 0,51624 |  |
| 8 | 0,688750 | 0,10 | -1,17774 |  |
| 9 | 0,580949 | 0,02 | 0,54348 |  |
| 10 | 0,580949 | 0,00 | 0,08305 |  |
| 11 | 0,425781 | 0,05 | -0,83952 |  |
| 12 | 0,425781 | 0,05 | -0,82538 |  |
| 13 | 0,569258 | 0,00 | 0,18652 |  |
| 14 | 0,569258 | 0,01 | 0,35867 |  |
| 15 | 0,433227 | 0,00 | -0,21751 |  |
| 16 | 0,433227 | 0,00 | 0,02984 |  |
| 17 | 0,597775 | 0,00 | 0,03174 |  |
| 18 | 0,597775 | 0,00 | 0,03362 |  |
| 19 | 0,407038 | 0,16 | -1,73941 |  |
| 20 | 0,647412 | 0,24 | 1,96577 |  |
| 21 | 0,407933 | 0,03 | 0,60854 |  |
| 22 | 0,817044 | 0,15 | -1,47059 |  |
| 23 | 0,823434 | 0,04 | -0,78184 |  |
| 24 | 0,713775 | 0,02 | 0,50806 |  |
| 25 | 0,288793 | 0,10 | -1,36452 |  |
| 26 | 0,288793 | 0,13 | 1,72845 | R |
| 27 | 0,288793 | 0,00 | 0,11014 |  |

R  Large residual

## Coded Coefficients

| Term | Coef | SE Coef | 95% CI | T-Value | P-Value | VIF |
| --- | --- | --- | --- | --- | --- | --- |
| Constant | 2,660 | 0,701 | (1,132; 4,188) | 3,79 | 0,003 |  |
| Lac | 1,585 | 0,580 | (0,321; 2,848) | 2,73 | 0,018 | 1,18 |
| HPMC\_Visc | 0,003 | 0,606 | (-1,317; 1,323) | 0,01 | 0,996 | 1,70 |
| HPMC\_HP | 0,740 | 0,593 | (-0,552; 2,031) | 1,25 | 0,236 | 1,26 |
| HPMC\_PS | 0,745 | 0,909 | (-1,235; 2,726) | 0,82 | 0,428 | 2,09 |
| Lac\*Lac | 2,20 | 1,15 | (-0,31; 4,71) | 1,91 | 0,081 | 1,30 |
| HPMC\_Visc\*HPMC\_Visc | 0,60 | 1,22 | (-2,07; 3,27) | 0,49 | 0,634 | 1,96 |
| HPMC\_HP\*HPMC\_HP | -0,90 | 1,16 | (-3,44; 1,63) | -0,78 | 0,452 | 1,81 |
| HPMC\_PS\*HPMC\_PS | -0,86 | 1,16 | (-3,38; 1,66) | -0,74 | 0,471 | 1,42 |
| Lac\*HPMC\_Visc | -0,75 | 1,27 | (-3,52; 2,02) | -0,59 | 0,567 | 1,49 |
| Lac\*HPMC\_HP | 2,13 | 1,33 | (-0,78; 5,04) | 1,60 | 0,136 | 1,17 |
| Lac\*HPMC\_PS | 1,52 | 2,06 | (-2,97; 6,01) | 0,74 | 0,475 | 1,42 |
| HPMC\_Visc\*HPMC\_HP | 0,72 | 1,52 | (-2,59; 4,03) | 0,47 | 0,644 | 2,73 |
| HPMC\_Visc\*HPMC\_PS | 1,85 | 2,14 | (-2,82; 6,52) | 0,86 | 0,406 | 2,74 |
| HPMC\_HP\*HPMC\_PS | 2,39 | 2,37 | (-2,77; 7,54) | 1,01 | 0,333 | 2,70 |

## Model Summary

| S | R-sq | R-sq(adj) | PRESS | R-sq(pred) | AICc | BIC |
| --- | --- | --- | --- | --- | --- | --- |
| 1,30558 | 67,39% | 29,34% | 71,9383 | 0,00% | 155,53 | 121,86 |

## Analysis of Variance

| Source | DF | Seq SS | Contribution | Adj SS | Adj MS | F-Value | P-Value |
| --- | --- | --- | --- | --- | --- | --- | --- |
| Model | 14 | 42,2666 | 67,39% | 42,2666 | 3,0190 | 1,77 | 0,164 |
| Linear | 4 | 16,3938 | 26,14% | 16,9453 | 4,2363 | 2,49 | 0,100 |
| Lac | 1 | 11,6033 | 18,50% | 12,7287 | 12,7287 | 7,47 | 0,018 |
| HPMC\_Visc | 1 | 1,1357 | 1,81% | 0,0000 | 0,0000 | 0,00 | 0,996 |
| HPMC\_HP | 1 | 3,6544 | 5,83% | 2,6549 | 2,6549 | 1,56 | 0,236 |
| HPMC\_PS | 1 | 0,0005 | 0,00% | 1,1458 | 1,1458 | 0,67 | 0,428 |
| Square | 4 | 13,9771 | 22,28% | 13,6145 | 3,4036 | 2,00 | 0,159 |
| Lac\*Lac | 1 | 10,2491 | 16,34% | 6,2011 | 6,2011 | 3,64 | 0,081 |
| HPMC\_Visc\*HPMC\_Visc | 1 | 0,1174 | 0,19% | 0,4065 | 0,4065 | 0,24 | 0,634 |
| HPMC\_HP\*HPMC\_HP | 1 | 0,9676 | 1,54% | 1,0321 | 1,0321 | 0,61 | 0,452 |
| HPMC\_PS\*HPMC\_PS | 1 | 2,6429 | 4,21% | 0,9425 | 0,9425 | 0,55 | 0,471 |
| 2-Way Interaction | 6 | 11,8957 | 18,97% | 11,8957 | 1,9826 | 1,16 | 0,386 |
| Lac\*HPMC\_Visc | 1 | 1,7741 | 2,83% | 0,5914 | 0,5914 | 0,35 | 0,567 |
| Lac\*HPMC\_HP | 1 | 5,6819 | 9,06% | 4,3479 | 4,3479 | 2,55 | 0,136 |
| Lac\*HPMC\_PS | 1 | 0,9290 | 1,48% | 0,9290 | 0,9290 | 0,55 | 0,475 |
| HPMC\_Visc\*HPMC\_HP | 1 | 0,0049 | 0,01% | 0,3834 | 0,3834 | 0,22 | 0,644 |
| HPMC\_Visc\*HPMC\_PS | 1 | 1,7683 | 2,82% | 1,2658 | 1,2658 | 0,74 | 0,406 |
| HPMC\_HP\*HPMC\_PS | 1 | 1,7375 | 2,77% | 1,7375 | 1,7375 | 1,02 | 0,333 |
| Error | 12 | 20,4544 | 32,61% | 20,4544 | 1,7045 |  |  |
| Lack-of-Fit | 10 | 10,6221 | 16,94% | 10,6221 | 1,0622 | 0,22 | 0,962 |
| Pure Error | 2 | 9,8323 | 15,68% | 9,8323 | 4,9161 |  |  |
| Total | 26 | 62,7210 | 100,00% |  |  |  |  |

## Regression Equation in Uncoded Units

|  |  |  |
| --- | --- | --- |
| F\_SD\_5h(300min) | = | 204 - 155,6 Lac - 0,00691 HPMC\_Visc - 11,6 HPMC\_HP - 2,00 HPMC\_PS + 35,2 Lac\*Lac + 0,000000 HPMC\_Visc\*HPMC\_Visc - 0,88 HPMC\_HP\*HPMC\_HP - 0,0159 HPMC\_PS\*HPMC\_PS - 0,00077 Lac\*HPMC\_Visc + 8,40 Lac\*HPMC\_HP + 0,83 Lac\*HPMC\_PS + 0,000182 HPMC\_Visc\*HPMC\_HP + 0,000065 HPMC\_Visc\*HPMC\_PS + 0,320 HPMC\_HP\*HPMC\_PS |

## Fits and Diagnostics for All Observations

| Obs | F\_SD\_5h(300min) | Fit | SE Fit | 95% CI | Resid | Std Resid | Del Resid | HI |
| --- | --- | --- | --- | --- | --- | --- | --- | --- |
| 1 | 3,441 | 3,287 | 1,057 | (0,984; 5,591) | 0,154 | 0,20 | 0,19 | 0,655994 |
| 2 | 3,335 | 3,493 | 1,057 | (1,189; 5,797) | -0,158 | -0,21 | -0,20 | 0,655994 |
| 3 | 1,801 | 2,345 | 1,115 | (-0,085; 4,776) | -0,544 | -0,80 | -0,79 | 0,729945 |
| 4 | 0,963 | 0,933 | 1,115 | (-1,498; 3,363) | 0,031 | 0,05 | 0,04 | 0,729945 |
| 5 | 2,870 | 2,322 | 0,902 | (0,358; 4,286) | 0,548 | 0,58 | 0,56 | 0,476813 |
| 6 | 5,707 | 4,569 | 0,902 | (2,605; 6,533) | 1,138 | 1,21 | 1,23 | 0,476813 |
| 7 | 2,089 | 1,794 | 1,084 | (-0,566; 4,155) | 0,295 | 0,40 | 0,39 | 0,688750 |
| 8 | 2,667 | 3,266 | 1,084 | (0,905; 5,627) | -0,599 | -0,82 | -0,81 | 0,688750 |
| 9 | 1,635 | 1,226 | 0,995 | (-0,942; 3,394) | 0,409 | 0,48 | 0,47 | 0,580949 |
| 10 | 2,462 | 2,510 | 0,995 | (0,342; 4,678) | -0,048 | -0,06 | -0,05 | 0,580949 |
| 11 | 1,282 | 2,189 | 0,852 | (0,333; 4,045) | -0,907 | -0,92 | -0,91 | 0,425781 |
| 12 | 1,933 | 2,937 | 0,852 | (1,081; 4,793) | -1,004 | -1,02 | -1,02 | 0,425781 |
| 13 | 1,739 | 1,551 | 0,985 | (-0,596; 3,697) | 0,188 | 0,22 | 0,21 | 0,569258 |
| 14 | 5,625 | 5,321 | 0,985 | (3,174; 7,467) | 0,304 | 0,35 | 0,34 | 0,569258 |
| 15 | 2,218 | 2,707 | 0,859 | (0,835; 4,579) | -0,489 | -0,50 | -0,48 | 0,433227 |
| 16 | 4,946 | 4,853 | 0,859 | (2,981; 6,726) | 0,093 | 0,09 | 0,09 | 0,433227 |
| 17 | 3,375 | 3,276 | 1,009 | (1,076; 5,475) | 0,100 | 0,12 | 0,12 | 0,597775 |
| 18 | 6,437 | 6,390 | 1,009 | (4,190; 8,589) | 0,048 | 0,06 | 0,06 | 0,597775 |
| 19 | 1,280 | 3,122 | 0,833 | (1,307; 4,937) | -1,841 | -1,83 | -2,07 | 0,407038 |
| 20 | 3,821 | 2,660 | 1,050 | (0,372; 4,949) | 1,161 | 1,50 | 1,59 | 0,647412 |
| 21 | 2,214 | 1,362 | 0,834 | (-0,455; 3,179) | 0,852 | 0,85 | 0,84 | 0,407933 |
| 22 | 2,176 | 2,576 | 1,180 | (0,004; 5,147) | -0,400 | -0,72 | -0,70 | 0,817044 |
| 23 | 1,516 | 1,705 | 1,185 | (-0,876; 4,286) | -0,189 | -0,34 | -0,33 | 0,823434 |
| 24 | 1,817 | 1,558 | 1,103 | (-0,846; 3,961) | 0,259 | 0,37 | 0,36 | 0,713775 |
| 25 | 0,679 | 2,634 | 0,702 | (1,106; 4,163) | -1,955 | -1,78 | -1,98 | 0,288793 |
| 26 | 5,109 | 2,634 | 0,702 | (1,106; 4,163) | 2,475 | 2,25 | 2,83 | 0,288793 |
| 27 | 2,715 | 2,634 | 0,702 | (1,106; 4,163) | 0,081 | 0,07 | 0,07 | 0,288793 |

| Obs | Cook’s D | DFITS |  |
| --- | --- | --- | --- |
| 1 | 0,01 | 0,26584 |  |
| 2 | 0,01 | -0,27260 |  |
| 3 | 0,12 | -1,29756 |  |
| 4 | 0,00 | 0,07117 |  |
| 5 | 0,02 | 0,53788 |  |
| 6 | 0,09 | 1,17535 |  |
| 7 | 0,02 | 0,58069 |  |
| 8 | 0,10 | -1,20480 |  |
| 9 | 0,02 | 0,55071 |  |
| 10 | 0,00 | -0,06405 |  |
| 11 | 0,04 | -0,78348 |  |
| 12 | 0,05 | -0,87546 |  |
| 13 | 0,00 | 0,24261 |  |
| 14 | 0,01 | 0,39269 |  |
| 15 | 0,01 | -0,42121 |  |
| 16 | 0,00 | 0,07881 |  |
| 17 | 0,00 | 0,14038 |  |
| 18 | 0,00 | 0,06737 |  |
| 19 | 0,15 | -1,71158 |  |
| 20 | 0,27 | 2,15444 |  |
| 21 | 0,03 | 0,69505 |  |
| 22 | 0,15 | -1,48197 |  |
| 23 | 0,04 | -0,71447 |  |
| 24 | 0,02 | 0,56393 |  |
| 25 | 0,09 | -1,26174 |  |
| 26 | 0,14 | 1,80189 | R |
| 27 | 0,00 | 0,04480 |  |

R  Large residual

## Coded Coefficients

| Term | Coef | SE Coef | 95% CI | T-Value | P-Value | VIF |
| --- | --- | --- | --- | --- | --- | --- |
| Constant | 2,718 | 0,694 | (1,205; 4,230) | 3,91 | 0,002 |  |
| Lac | 1,580 | 0,574 | (0,329; 2,831) | 2,75 | 0,018 | 1,18 |
| HPMC\_Visc | -0,017 | 0,600 | (-1,324; 1,290) | -0,03 | 0,978 | 1,70 |
| HPMC\_HP | 0,780 | 0,587 | (-0,498; 2,059) | 1,33 | 0,208 | 1,26 |
| HPMC\_PS | 0,756 | 0,900 | (-1,205; 2,717) | 0,84 | 0,418 | 2,09 |
| Lac\*Lac | 2,22 | 1,14 | (-0,27; 4,70) | 1,94 | 0,076 | 1,30 |
| HPMC\_Visc\*HPMC\_Visc | 0,58 | 1,21 | (-2,06; 3,22) | 0,48 | 0,641 | 1,96 |
| HPMC\_HP\*HPMC\_HP | -0,93 | 1,15 | (-3,43; 1,58) | -0,80 | 0,437 | 1,81 |
| HPMC\_PS\*HPMC\_PS | -0,90 | 1,14 | (-3,39; 1,59) | -0,79 | 0,448 | 1,42 |
| Lac\*HPMC\_Visc | -0,67 | 1,26 | (-3,41; 2,07) | -0,53 | 0,603 | 1,49 |
| Lac\*HPMC\_HP | 2,15 | 1,32 | (-0,73; 5,03) | 1,63 | 0,130 | 1,17 |
| Lac\*HPMC\_PS | 1,71 | 2,04 | (-2,73; 6,15) | 0,84 | 0,418 | 1,42 |
| HPMC\_Visc\*HPMC\_HP | 0,74 | 1,50 | (-2,53; 4,01) | 0,49 | 0,631 | 2,73 |
| HPMC\_Visc\*HPMC\_PS | 1,72 | 2,12 | (-2,90; 6,35) | 0,81 | 0,433 | 2,74 |
| HPMC\_HP\*HPMC\_PS | 2,40 | 2,34 | (-2,70; 7,51) | 1,03 | 0,325 | 2,70 |

## Model Summary

| S | R-sq | R-sq(adj) | PRESS | R-sq(pred) | AICc | BIC |
| --- | --- | --- | --- | --- | --- | --- |
| 1,29258 | 68,22% | 31,15% | 71,7981 | 0,00% | 154,99 | 121,32 |

## Analysis of Variance

| Source | DF | Seq SS | Contribution | Adj SS | Adj MS | F-Value | P-Value |
| --- | --- | --- | --- | --- | --- | --- | --- |
| Model | 14 | 43,0448 | 68,22% | 43,0448 | 3,0746 | 1,84 | 0,148 |
| Linear | 4 | 16,5935 | 26,30% | 17,2991 | 4,3248 | 2,59 | 0,090 |
| Lac | 1 | 11,2876 | 17,89% | 12,6585 | 12,6585 | 7,58 | 0,018 |
| HPMC\_Visc | 1 | 1,2153 | 1,93% | 0,0013 | 0,0013 | 0,00 | 0,978 |
| HPMC\_HP | 1 | 4,0885 | 6,48% | 2,9546 | 2,9546 | 1,77 | 0,208 |
| HPMC\_PS | 1 | 0,0021 | 0,00% | 1,1779 | 1,1779 | 0,71 | 0,418 |
| Square | 4 | 14,3060 | 22,67% | 14,0087 | 3,5022 | 2,10 | 0,144 |
| Lac\*Lac | 1 | 10,5421 | 16,71% | 6,3071 | 6,3071 | 3,77 | 0,076 |
| HPMC\_Visc\*HPMC\_Visc | 1 | 0,1487 | 0,24% | 0,3829 | 0,3829 | 0,23 | 0,641 |
| HPMC\_HP\*HPMC\_HP | 1 | 0,9388 | 1,49% | 1,0801 | 1,0801 | 0,65 | 0,437 |
| HPMC\_PS\*HPMC\_PS | 1 | 2,6764 | 4,24% | 1,0296 | 1,0296 | 0,62 | 0,448 |
| 2-Way Interaction | 6 | 12,1453 | 19,25% | 12,1453 | 2,0242 | 1,21 | 0,365 |
| Lac\*HPMC\_Visc | 1 | 1,7154 | 2,72% | 0,4766 | 0,4766 | 0,29 | 0,603 |
| Lac\*HPMC\_HP | 1 | 5,9140 | 9,37% | 4,4242 | 4,4242 | 2,65 | 0,130 |
| Lac\*HPMC\_PS | 1 | 1,1745 | 1,86% | 1,1745 | 1,1745 | 0,70 | 0,418 |
| HPMC\_Visc\*HPMC\_HP | 1 | 0,0061 | 0,01% | 0,4049 | 0,4049 | 0,24 | 0,631 |
| HPMC\_Visc\*HPMC\_PS | 1 | 1,5745 | 2,50% | 1,1017 | 1,1017 | 0,66 | 0,433 |
| HPMC\_HP\*HPMC\_PS | 1 | 1,7608 | 2,79% | 1,7608 | 1,7608 | 1,05 | 0,325 |
| Error | 12 | 20,0491 | 31,78% | 20,0491 | 1,6708 |  |  |
| Lack-of-Fit | 10 | 10,4113 | 16,50% | 10,4113 | 1,0411 | 0,22 | 0,962 |
| Pure Error | 2 | 9,6379 | 15,28% | 9,6379 | 4,8189 |  |  |
| Total | 26 | 63,0939 | 100,00% |  |  |  |  |

## Regression Equation in Uncoded Units

|  |  |  |
| --- | --- | --- |
| F\_SD\_5.5h(330min) | = | 201 - 164,8 Lac - 0,00666 HPMC\_Visc - 11,4 HPMC\_HP - 1,91 HPMC\_PS + 35,5 Lac\*Lac + 0,000000 HPMC\_Visc\*HPMC\_Visc - 0,90 HPMC\_HP\*HPMC\_HP - 0,0166 HPMC\_PS\*HPMC\_PS - 0,00069 Lac\*HPMC\_Visc + 8,47 Lac\*HPMC\_HP + 0,93 Lac\*HPMC\_PS + 0,000187 HPMC\_Visc\*HPMC\_HP + 0,000060 HPMC\_Visc\*HPMC\_PS + 0,322 HPMC\_HP\*HPMC\_PS |

## Fits and Diagnostics for All Observations

| Obs | F\_SD\_5.5h(330min) | Fit | SE Fit | 95% CI | Resid | Std Resid | Del Resid |
| --- | --- | --- | --- | --- | --- | --- | --- |
| 1 | 3,526 | 3,361 | 1,047 | (1,080; 5,642) | 0,165 | 0,22 | 0,21 |
| 2 | 3,308 | 3,448 | 1,047 | (1,167; 5,729) | -0,139 | -0,18 | -0,18 |
| 3 | 1,841 | 2,408 | 1,104 | (0,002; 4,814) | -0,567 | -0,84 | -0,83 |
| 4 | 0,944 | 0,920 | 1,104 | (-1,486; 3,326) | 0,024 | 0,04 | 0,03 |
| 5 | 2,963 | 2,419 | 0,893 | (0,475; 4,364) | 0,544 | 0,58 | 0,56 |
| 6 | 5,682 | 4,583 | 0,893 | (2,639; 6,528) | 1,099 | 1,18 | 1,20 |
| 7 | 2,209 | 1,884 | 1,073 | (-0,453; 4,221) | 0,325 | 0,45 | 0,43 |
| 8 | 2,783 | 3,341 | 1,073 | (1,003; 5,678) | -0,558 | -0,77 | -0,76 |
| 9 | 1,594 | 1,291 | 0,985 | (-0,856; 3,437) | 0,303 | 0,36 | 0,35 |
| 10 | 2,466 | 2,583 | 0,985 | (0,437; 4,730) | -0,117 | -0,14 | -0,13 |
| 11 | 1,341 | 2,195 | 0,843 | (0,357; 4,032) | -0,854 | -0,87 | -0,86 |
| 12 | 1,952 | 2,984 | 0,843 | (1,146; 4,822) | -1,032 | -1,05 | -1,06 |
| 13 | 1,815 | 1,646 | 0,975 | (-0,479; 3,771) | 0,170 | 0,20 | 0,19 |
| 14 | 5,753 | 5,463 | 0,975 | (3,339; 7,588) | 0,290 | 0,34 | 0,33 |
| 15 | 2,137 | 2,726 | 0,851 | (0,872; 4,580) | -0,589 | -0,61 | -0,59 |
| 16 | 4,968 | 4,943 | 0,851 | (3,090; 6,797) | 0,025 | 0,03 | 0,02 |
| 17 | 3,517 | 3,379 | 0,999 | (1,201; 5,556) | 0,138 | 0,17 | 0,16 |
| 18 | 6,530 | 6,440 | 0,999 | (4,263; 8,617) | 0,090 | 0,11 | 0,11 |
| 19 | 1,439 | 3,193 | 0,825 | (1,396; 4,989) | -1,754 | -1,76 | -1,96 |
| 20 | 3,843 | 2,685 | 1,040 | (0,419; 4,951) | 1,158 | 1,51 | 1,60 |
| 21 | 2,282 | 1,363 | 0,826 | (-0,436; 3,162) | 0,919 | 0,92 | 0,92 |
| 22 | 2,258 | 2,655 | 1,168 | (0,110; 5,201) | -0,398 | -0,72 | -0,70 |
| 23 | 1,448 | 1,684 | 1,173 | (-0,872; 4,240) | -0,236 | -0,43 | -0,42 |
| 24 | 1,969 | 1,615 | 1,092 | (-0,764; 3,995) | 0,353 | 0,51 | 0,49 |
| 25 | 0,837 | 2,692 | 0,695 | (1,179; 4,206) | -1,855 | -1,70 | -1,87 |
| 26 | 5,209 | 2,692 | 0,695 | (1,179; 4,206) | 2,517 | 2,31 | 2,96 |
| 27 | 2,672 | 2,692 | 0,695 | (1,179; 4,206) | -0,020 | -0,02 | -0,02 |

| Obs | HI | Cook’s D | DFITS |  |
| --- | --- | --- | --- | --- |
| 1 | 0,655994 | 0,01 | 0,28777 |  |
| 2 | 0,655994 | 0,00 | -0,24322 |  |
| 3 | 0,729945 | 0,13 | -1,36985 |  |
| 4 | 0,729945 | 0,00 | 0,05663 |  |
| 5 | 0,476813 | 0,02 | 0,53936 |  |
| 6 | 0,476813 | 0,08 | 1,14223 |  |
| 7 | 0,688750 | 0,03 | 0,64702 |  |
| 8 | 0,688750 | 0,09 | -1,13099 |  |
| 9 | 0,580949 | 0,01 | 0,41009 |  |
| 10 | 0,580949 | 0,00 | -0,15764 |  |
| 11 | 0,425781 | 0,04 | -0,74256 |  |
| 12 | 0,425781 | 0,05 | -0,91154 |  |
| 13 | 0,569258 | 0,00 | 0,22031 |  |
| 14 | 0,569258 | 0,01 | 0,37770 |  |
| 15 | 0,433227 | 0,02 | -0,51465 |  |
| 16 | 0,433227 | 0,00 | 0,02166 |  |
| 17 | 0,597775 | 0,00 | 0,19684 |  |
| 18 | 0,597775 | 0,00 | 0,12814 |  |
| 19 | 0,407038 | 0,14 | -1,62322 |  |
| 20 | 0,647412 | 0,28 | 2,17368 |  |
| 21 | 0,407933 | 0,04 | 0,76195 |  |
| 22 | 0,817044 | 0,15 | -1,48731 |  |
| 23 | 0,823434 | 0,06 | -0,90580 |  |
| 24 | 0,713775 | 0,04 | 0,78121 |  |
| 25 | 0,288793 | 0,08 | -1,19193 |  |
| 26 | 0,288793 | 0,14 | 1,88933 | R |
| 27 | 0,288793 | 0,00 | -0,01141 |  |

R  Large residual

## Coded Coefficients

| Term | Coef | SE Coef | 95% CI | T-Value | P-Value | VIF |
| --- | --- | --- | --- | --- | --- | --- |
| Constant | 2,750 | 0,702 | (1,220; 4,280) | 3,92 | 0,002 |  |
| Lac | 1,588 | 0,581 | (0,323; 2,853) | 2,73 | 0,018 | 1,18 |
| HPMC\_Visc | -0,008 | 0,607 | (-1,330; 1,314) | -0,01 | 0,990 | 1,70 |
| HPMC\_HP | 0,853 | 0,594 | (-0,440; 2,147) | 1,44 | 0,176 | 1,26 |
| HPMC\_PS | 0,817 | 0,911 | (-1,167; 2,801) | 0,90 | 0,387 | 2,09 |
| Lac\*Lac | 2,26 | 1,15 | (-0,26; 4,77) | 1,96 | 0,074 | 1,30 |
| HPMC\_Visc\*HPMC\_Visc | 0,63 | 1,23 | (-2,04; 3,31) | 0,52 | 0,616 | 1,96 |
| HPMC\_HP\*HPMC\_HP | -0,90 | 1,16 | (-3,44; 1,63) | -0,78 | 0,453 | 1,81 |
| HPMC\_PS\*HPMC\_PS | -0,90 | 1,16 | (-3,43; 1,62) | -0,78 | 0,449 | 1,42 |
| Lac\*HPMC\_Visc | -0,50 | 1,27 | (-3,28; 2,27) | -0,39 | 0,701 | 1,49 |
| Lac\*HPMC\_HP | 2,27 | 1,34 | (-0,65; 5,18) | 1,70 | 0,116 | 1,17 |
| Lac\*HPMC\_PS | 1,99 | 2,06 | (-2,50; 6,49) | 0,97 | 0,353 | 1,42 |
| HPMC\_Visc\*HPMC\_HP | 0,91 | 1,52 | (-2,40; 4,22) | 0,60 | 0,560 | 2,73 |
| HPMC\_Visc\*HPMC\_PS | 1,81 | 2,15 | (-2,87; 6,48) | 0,84 | 0,417 | 2,74 |
| HPMC\_HP\*HPMC\_PS | 2,68 | 2,37 | (-2,49; 7,84) | 1,13 | 0,281 | 2,70 |

## Model Summary

| S | R-sq | R-sq(adj) | PRESS | R-sq(pred) | AICc | BIC |
| --- | --- | --- | --- | --- | --- | --- |
| 1,30758 | 68,65% | 32,08% | 75,5818 | 0,00% | 155,61 | 121,94 |

## Analysis of Variance

| Source | DF | Seq SS | Contribution | Adj SS | Adj MS | F-Value | P-Value |
| --- | --- | --- | --- | --- | --- | --- | --- |
| Model | 14 | 44,9337 | 68,65% | 44,9337 | 3,2095 | 1,88 | 0,140 |
| Linear | 4 | 16,6225 | 25,40% | 18,2353 | 4,5588 | 2,67 | 0,084 |
| Lac | 1 | 10,7435 | 16,41% | 12,7837 | 12,7837 | 7,48 | 0,018 |
| HPMC\_Visc | 1 | 1,3985 | 2,14% | 0,0003 | 0,0003 | 0,00 | 0,990 |
| HPMC\_HP | 1 | 4,4793 | 6,84% | 3,5309 | 3,5309 | 2,07 | 0,176 |
| HPMC\_PS | 1 | 0,0012 | 0,00% | 1,3752 | 1,3752 | 0,80 | 0,387 |
| Square | 4 | 14,6015 | 22,31% | 14,3950 | 3,5987 | 2,10 | 0,143 |
| Lac\*Lac | 1 | 10,8061 | 16,51% | 6,5497 | 6,5497 | 3,83 | 0,074 |
| HPMC\_Visc\*HPMC\_Visc | 1 | 0,1931 | 0,29% | 0,4541 | 0,4541 | 0,27 | 0,616 |
| HPMC\_HP\*HPMC\_HP | 1 | 0,7871 | 1,20% | 1,0274 | 1,0274 | 0,60 | 0,453 |
| HPMC\_PS\*HPMC\_PS | 1 | 2,8152 | 4,30% | 1,0457 | 1,0457 | 0,61 | 0,449 |
| 2-Way Interaction | 6 | 13,7097 | 20,95% | 13,7097 | 2,2850 | 1,34 | 0,314 |
| Lac\*HPMC\_Visc | 1 | 1,4465 | 2,21% | 0,2653 | 0,2653 | 0,16 | 0,701 |
| Lac\*HPMC\_HP | 1 | 6,7243 | 10,27% | 4,9162 | 4,9162 | 2,88 | 0,116 |
| Lac\*HPMC\_PS | 1 | 1,5953 | 2,44% | 1,5953 | 1,5953 | 0,93 | 0,353 |
| HPMC\_Visc\*HPMC\_HP | 1 | 0,0000 | 0,00% | 0,6151 | 0,6151 | 0,36 | 0,560 |
| HPMC\_Visc\*HPMC\_PS | 1 | 1,7620 | 2,69% | 1,2095 | 1,2095 | 0,71 | 0,417 |
| HPMC\_HP\*HPMC\_PS | 1 | 2,1815 | 3,33% | 2,1815 | 2,1815 | 1,28 | 0,281 |
| Error | 12 | 20,5172 | 31,35% | 20,5172 | 1,7098 |  |  |
| Lack-of-Fit | 10 | 10,6902 | 16,33% | 10,6902 | 1,0690 | 0,22 | 0,962 |
| Pure Error | 2 | 9,8270 | 15,01% | 9,8270 | 4,9135 |  |  |
| Total | 26 | 65,4509 | 100,00% |  |  |  |  |

## Regression Equation in Uncoded Units

|  |  |  |
| --- | --- | --- |
| F\_SD\_6h(360min) | = | 243 - 182,9 Lac - 0,00746 HPMC\_Visc - 15,1 HPMC\_HP - 2,35 HPMC\_PS + 36,1 Lac\*Lac + 0,000000 HPMC\_Visc\*HPMC\_Visc - 0,88 HPMC\_HP\*HPMC\_HP - 0,0167 HPMC\_PS\*HPMC\_PS - 0,00052 Lac\*HPMC\_Visc + 8,93 Lac\*HPMC\_HP + 1,08 Lac\*HPMC\_PS + 0,000231 HPMC\_Visc\*HPMC\_HP + 0,000063 HPMC\_Visc\*HPMC\_PS + 0,358 HPMC\_HP\*HPMC\_PS |

## Fits and Diagnostics for All Observations

| Obs | F\_SD\_6h(360min) | Fit | SE Fit | 95% CI | Resid | Std Resid | Del Resid | HI |
| --- | --- | --- | --- | --- | --- | --- | --- | --- |
| 1 | 3,951 | 3,653 | 1,059 | (1,346; 5,961) | 0,298 | 0,39 | 0,37 | 0,655994 |
| 2 | 3,293 | 3,472 | 1,059 | (1,165; 5,780) | -0,179 | -0,23 | -0,22 | 0,655994 |
| 3 | 1,837 | 2,461 | 1,117 | (0,027; 4,895) | -0,625 | -0,92 | -0,91 | 0,729945 |
| 4 | 0,881 | 0,848 | 1,117 | (-1,586; 3,282) | 0,033 | 0,05 | 0,05 | 0,729945 |
| 5 | 3,030 | 2,524 | 0,903 | (0,557; 4,492) | 0,506 | 0,53 | 0,52 | 0,476813 |
| 6 | 5,644 | 4,561 | 0,903 | (2,593; 6,528) | 1,084 | 1,15 | 1,16 | 0,476813 |
| 7 | 2,313 | 1,922 | 1,085 | (-0,442; 4,286) | 0,391 | 0,54 | 0,52 | 0,688750 |
| 8 | 2,883 | 3,453 | 1,085 | (1,089; 5,817) | -0,570 | -0,78 | -0,77 | 0,688750 |
| 9 | 1,548 | 1,355 | 0,997 | (-0,817; 3,526) | 0,193 | 0,23 | 0,22 | 0,580949 |
| 10 | 2,425 | 2,575 | 0,997 | (0,403; 4,746) | -0,150 | -0,18 | -0,17 | 0,580949 |
| 11 | 1,405 | 2,162 | 0,853 | (0,303; 4,021) | -0,757 | -0,76 | -0,75 | 0,425781 |
| 12 | 1,960 | 2,996 | 0,853 | (1,137; 4,855) | -1,036 | -1,05 | -1,05 | 0,425781 |
| 13 | 1,914 | 1,708 | 0,987 | (-0,441; 3,858) | 0,205 | 0,24 | 0,23 | 0,569258 |
| 14 | 5,889 | 5,609 | 0,987 | (3,459; 7,758) | 0,280 | 0,33 | 0,31 | 0,569258 |
| 15 | 2,064 | 2,756 | 0,861 | (0,881; 4,631) | -0,692 | -0,70 | -0,69 | 0,433227 |
| 16 | 5,014 | 5,125 | 0,861 | (3,250; 7,000) | -0,110 | -0,11 | -0,11 | 0,433227 |
| 17 | 3,590 | 3,491 | 1,011 | (1,288; 5,693) | 0,099 | 0,12 | 0,11 | 0,597775 |
| 18 | 6,655 | 6,471 | 1,011 | (4,269; 8,674) | 0,183 | 0,22 | 0,21 | 0,597775 |
| 19 | 1,519 | 3,272 | 0,834 | (1,454; 5,089) | -1,753 | -1,74 | -1,93 | 0,407038 |
| 20 | 3,915 | 2,723 | 1,052 | (0,431; 5,015) | 1,192 | 1,53 | 1,64 | 0,647412 |
| 21 | 2,319 | 1,400 | 0,835 | (-0,419; 3,220) | 0,918 | 0,91 | 0,91 | 0,407933 |
| 22 | 2,398 | 2,781 | 1,182 | (0,206; 5,356) | -0,383 | -0,68 | -0,67 | 0,817044 |
| 23 | 1,418 | 1,689 | 1,187 | (-0,896; 4,275) | -0,272 | -0,49 | -0,48 | 0,823434 |
| 24 | 2,052 | 1,630 | 1,105 | (-0,777; 4,037) | 0,422 | 0,60 | 0,59 | 0,713775 |
| 25 | 0,961 | 2,722 | 0,703 | (1,191; 4,253) | -1,761 | -1,60 | -1,72 | 0,288793 |
| 26 | 5,345 | 2,722 | 0,703 | (1,191; 4,253) | 2,623 | 2,38 | 3,13 | 0,288793 |
| 27 | 2,583 | 2,722 | 0,703 | (1,191; 4,253) | -0,139 | -0,13 | -0,12 | 0,288793 |

| Obs | Cook’s D | DFITS |  |
| --- | --- | --- | --- |
| 1 | 0,02 | 0,51637 |  |
| 2 | 0,01 | -0,30997 |  |
| 3 | 0,15 | -1,50055 |  |
| 4 | 0,00 | 0,07621 |  |
| 5 | 0,02 | 0,49468 |  |
| 6 | 0,08 | 1,10956 |  |
| 7 | 0,04 | 0,77181 |  |
| 8 | 0,09 | -1,14278 |  |
| 9 | 0,00 | 0,25808 |  |
| 10 | 0,00 | -0,19961 |  |
| 11 | 0,03 | -0,64621 |  |
| 12 | 0,05 | -0,90421 |  |
| 13 | 0,01 | 0,26407 |  |
| 14 | 0,01 | 0,36087 |  |
| 15 | 0,03 | -0,60121 |  |
| 16 | 0,00 | -0,09392 |  |
| 17 | 0,00 | 0,14007 |  |
| 18 | 0,00 | 0,25843 |  |
| 19 | 0,14 | -1,59720 |  |
| 20 | 0,29 | 2,22133 |  |
| 21 | 0,04 | 0,75170 |  |
| 22 | 0,14 | -1,41223 |  |
| 23 | 0,08 | -1,03250 |  |
| 24 | 0,06 | 0,92714 |  |
| 25 | 0,07 | -1,09807 |  |
| 26 | 0,15 | 1,99624 | R |
| 27 | 0,00 | -0,07691 |  |

R  Large residual

## Coded Coefficients

| Term | Coef | SE Coef | 95% CI | T-Value | P-Value | VIF |
| --- | --- | --- | --- | --- | --- | --- |
| Constant | 2,861 | 0,725 | (1,281; 4,440) | 3,95 | 0,002 |  |
| Lac | 1,592 | 0,600 | (0,286; 2,898) | 2,66 | 0,021 | 1,18 |
| HPMC\_Visc | -0,033 | 0,626 | (-1,398; 1,332) | -0,05 | 0,959 | 1,70 |
| HPMC\_HP | 0,925 | 0,613 | (-0,411; 2,260) | 1,51 | 0,157 | 1,26 |
| HPMC\_PS | 0,897 | 0,940 | (-1,151; 2,945) | 0,95 | 0,359 | 2,09 |
| Lac\*Lac | 2,30 | 1,19 | (-0,30; 4,90) | 1,93 | 0,077 | 1,30 |
| HPMC\_Visc\*HPMC\_Visc | 0,56 | 1,27 | (-2,20; 3,32) | 0,44 | 0,666 | 1,96 |
| HPMC\_HP\*HPMC\_HP | -0,97 | 1,20 | (-3,59; 1,64) | -0,81 | 0,434 | 1,81 |
| HPMC\_PS\*HPMC\_PS | -0,99 | 1,19 | (-3,59; 1,61) | -0,83 | 0,424 | 1,42 |
| Lac\*HPMC\_Visc | -0,19 | 1,31 | (-3,05; 2,67) | -0,14 | 0,887 | 1,49 |
| Lac\*HPMC\_HP | 2,39 | 1,38 | (-0,61; 5,40) | 1,74 | 0,108 | 1,17 |
| Lac\*HPMC\_PS | 2,46 | 2,13 | (-2,18; 7,09) | 1,15 | 0,271 | 1,42 |
| HPMC\_Visc\*HPMC\_HP | 1,21 | 1,57 | (-2,21; 4,62) | 0,77 | 0,456 | 2,73 |
| HPMC\_Visc\*HPMC\_PS | 1,67 | 2,22 | (-3,15; 6,50) | 0,76 | 0,465 | 2,74 |
| HPMC\_HP\*HPMC\_PS | 3,12 | 2,45 | (-2,21; 8,45) | 1,28 | 0,226 | 2,70 |

## Model Summary

| S | R-sq | R-sq(adj) | PRESS | R-sq(pred) | AICc | BIC |
| --- | --- | --- | --- | --- | --- | --- |
| 1,34973 | 68,68% | 32,15% | 85,8043 | 0,00% | 157,32 | 123,66 |

## Analysis of Variance

| Source | DF | Seq SS | Contribution | Adj SS | Adj MS | F-Value | P-Value |
| --- | --- | --- | --- | --- | --- | --- | --- |
| Model | 14 | 47,9449 | 68,68% | 47,9449 | 3,4246 | 1,88 | 0,140 |
| Linear | 4 | 16,5759 | 23,75% | 19,4364 | 4,8591 | 2,67 | 0,084 |
| Lac | 1 | 9,8049 | 14,05% | 12,8458 | 12,8458 | 7,05 | 0,021 |
| HPMC\_Visc | 1 | 1,8284 | 2,62% | 0,0051 | 0,0051 | 0,00 | 0,959 |
| HPMC\_HP | 1 | 4,9273 | 7,06% | 4,1462 | 4,1462 | 2,28 | 0,157 |
| HPMC\_PS | 1 | 0,0153 | 0,02% | 1,6599 | 1,6599 | 0,91 | 0,359 |
| Square | 4 | 15,5263 | 22,24% | 15,3447 | 3,8362 | 2,11 | 0,143 |
| Lac\*Lac | 1 | 11,5811 | 16,59% | 6,7926 | 6,7926 | 3,73 | 0,077 |
| HPMC\_Visc\*HPMC\_Visc | 1 | 0,2008 | 0,29% | 0,3566 | 0,3566 | 0,20 | 0,666 |
| HPMC\_HP\*HPMC\_HP | 1 | 0,6765 | 0,97% | 1,1954 | 1,1954 | 0,66 | 0,434 |
| HPMC\_PS\*HPMC\_PS | 1 | 3,0680 | 4,39% | 1,2468 | 1,2468 | 0,68 | 0,424 |
| 2-Way Interaction | 6 | 15,8426 | 22,70% | 15,8426 | 2,6404 | 1,45 | 0,275 |
| Lac\*HPMC\_Visc | 1 | 0,9883 | 1,42% | 0,0382 | 0,0382 | 0,02 | 0,887 |
| Lac\*HPMC\_HP | 1 | 7,8094 | 11,19% | 5,4879 | 5,4879 | 3,01 | 0,108 |
| Lac\*HPMC\_PS | 1 | 2,4261 | 3,48% | 2,4261 | 2,4261 | 1,33 | 0,271 |
| HPMC\_Visc\*HPMC\_HP | 1 | 0,0119 | 0,02% | 1,0789 | 1,0789 | 0,59 | 0,456 |
| HPMC\_Visc\*HPMC\_PS | 1 | 1,6428 | 2,35% | 1,0387 | 1,0387 | 0,57 | 0,465 |
| HPMC\_HP\*HPMC\_PS | 1 | 2,9641 | 4,25% | 2,9641 | 2,9641 | 1,63 | 0,226 |
| Error | 12 | 21,8613 | 31,32% | 21,8613 | 1,8218 |  |  |
| Lack-of-Fit | 10 | 11,8581 | 16,99% | 11,8581 | 1,1858 | 0,24 | 0,953 |
| Pure Error | 2 | 10,0032 | 14,33% | 10,0032 | 5,0016 |  |  |
| Total | 26 | 69,8062 | 100,00% |  |  |  |  |

## Regression Equation in Uncoded Units

|  |  |  |
| --- | --- | --- |
| F\_SD\_7h(420min) | = | 285 - 210,2 Lac - 0,00788 HPMC\_Visc - 19,1 HPMC\_HP - 2,75 HPMC\_PS + 36,8 Lac\*Lac + 0,000000 HPMC\_Visc\*HPMC\_Visc - 0,94 HPMC\_HP\*HPMC\_HP - 0,0183 HPMC\_PS\*HPMC\_PS - 0,00020 Lac\*HPMC\_Visc + 9,43 Lac\*HPMC\_HP + 1,34 Lac\*HPMC\_PS + 0,000306 HPMC\_Visc\*HPMC\_HP + 0,000058 HPMC\_Visc\*HPMC\_PS + 0,418 HPMC\_HP\*HPMC\_PS |

## Fits and Diagnostics for All Observations

| Obs | F\_SD\_7h(420min) | Fit | SE Fit | 95% CI | Resid | Std Resid | Del Resid | HI |
| --- | --- | --- | --- | --- | --- | --- | --- | --- |
| 1 | 4,518 | 4,062 | 1,093 | (1,680; 6,444) | 0,456 | 0,58 | 0,56 | 0,655994 |
| 2 | 3,309 | 3,456 | 1,093 | (1,074; 5,838) | -0,147 | -0,19 | -0,18 | 0,655994 |
| 3 | 1,805 | 2,528 | 1,153 | (0,016; 5,041) | -0,724 | -1,03 | -1,03 | 0,729945 |
| 4 | 0,785 | 0,768 | 1,153 | (-1,744; 3,281) | 0,017 | 0,02 | 0,02 | 0,729945 |
| 5 | 3,156 | 2,683 | 0,932 | (0,652; 4,713) | 0,473 | 0,48 | 0,47 | 0,476813 |
| 6 | 5,532 | 4,467 | 0,932 | (2,437; 6,498) | 1,065 | 1,09 | 1,10 | 0,476813 |
| 7 | 2,492 | 1,965 | 1,120 | (-0,476; 4,405) | 0,527 | 0,70 | 0,68 | 0,688750 |
| 8 | 3,044 | 3,600 | 1,120 | (1,160; 6,041) | -0,556 | -0,74 | -0,72 | 0,688750 |
| 9 | 1,507 | 1,501 | 1,029 | (-0,740; 3,743) | 0,006 | 0,01 | 0,01 | 0,580949 |
| 10 | 2,345 | 2,616 | 1,029 | (0,374; 4,857) | -0,270 | -0,31 | -0,30 | 0,580949 |
| 11 | 1,564 | 2,113 | 0,881 | (0,194; 4,032) | -0,548 | -0,54 | -0,52 | 0,425781 |
| 12 | 2,022 | 3,047 | 0,881 | (1,128; 4,966) | -1,025 | -1,00 | -1,00 | 0,425781 |
| 13 | 2,157 | 1,866 | 1,018 | (-0,353; 4,085) | 0,291 | 0,33 | 0,32 | 0,569258 |
| 14 | 6,144 | 5,846 | 1,018 | (3,627; 8,065) | 0,298 | 0,34 | 0,32 | 0,569258 |
| 15 | 1,815 | 2,796 | 0,888 | (0,860; 4,731) | -0,981 | -0,97 | -0,96 | 0,433227 |
| 16 | 5,112 | 5,402 | 0,888 | (3,467; 7,338) | -0,290 | -0,29 | -0,27 | 0,433227 |
| 17 | 3,792 | 3,719 | 1,044 | (1,445; 5,992) | 0,074 | 0,09 | 0,08 | 0,597775 |
| 18 | 6,822 | 6,544 | 1,044 | (4,270; 8,818) | 0,278 | 0,32 | 0,31 | 0,597775 |
| 19 | 1,585 | 3,368 | 0,861 | (1,491; 5,244) | -1,783 | -1,72 | -1,89 | 0,407038 |
| 20 | 3,927 | 2,687 | 1,086 | (0,321; 5,054) | 1,240 | 1,55 | 1,66 | 0,647412 |
| 21 | 2,408 | 1,459 | 0,862 | (-0,419; 3,337) | 0,949 | 0,91 | 0,91 | 0,407933 |
| 22 | 2,493 | 2,889 | 1,220 | (0,230; 5,547) | -0,395 | -0,68 | -0,67 | 0,817044 |
| 23 | 1,290 | 1,636 | 1,225 | (-1,032; 4,305) | -0,347 | -0,61 | -0,59 | 0,823434 |
| 24 | 2,248 | 1,698 | 1,140 | (-0,786; 4,183) | 0,550 | 0,76 | 0,75 | 0,713775 |
| 25 | 1,261 | 2,831 | 0,725 | (1,250; 4,411) | -1,569 | -1,38 | -1,44 | 0,288793 |
| 26 | 5,597 | 2,831 | 0,725 | (1,250; 4,411) | 2,766 | 2,43 | 3,26 | 0,288793 |
| 27 | 2,476 | 2,831 | 0,725 | (1,250; 4,411) | -0,354 | -0,31 | -0,30 | 0,288793 |

| Obs | Cook’s D | DFITS |  |
| --- | --- | --- | --- |
| 1 | 0,04 | 0,77234 |  |
| 2 | 0,00 | -0,24659 |  |
| 3 | 0,19 | -1,70105 |  |
| 4 | 0,00 | 0,03881 |  |
| 5 | 0,01 | 0,44716 |  |
| 6 | 0,07 | 1,05040 |  |
| 7 | 0,07 | 1,01795 |  |
| 8 | 0,08 | -1,07694 |  |
| 9 | 0,00 | 0,00756 |  |
| 10 | 0,01 | -0,34996 |  |
| 11 | 0,01 | -0,44726 |  |
| 12 | 0,05 | -0,86299 |  |
| 13 | 0,01 | 0,36328 |  |
| 14 | 0,01 | 0,37229 |  |
| 15 | 0,05 | -0,84158 |  |
| 16 | 0,00 | -0,23981 |  |
| 17 | 0,00 | 0,10083 |  |
| 18 | 0,01 | 0,38098 |  |
| 19 | 0,13 | -1,56641 |  |
| 20 | 0,29 | 2,24345 |  |
| 21 | 0,04 | 0,75260 |  |
| 22 | 0,14 | -1,41289 |  |
| 23 | 0,12 | -1,28395 |  |
| 24 | 0,10 | 1,18099 |  |
| 25 | 0,05 | -0,91694 |  |
| 26 | 0,16 | 2,08030 | R |
| 27 | 0,00 | -0,19072 |  |

R  Large residual

## Coded Coefficients

| Term | Coef | SE Coef | 95% CI | T-Value | P-Value | VIF |
| --- | --- | --- | --- | --- | --- | --- |
| Constant | 2,932 | 0,767 | (1,260; 4,604) | 3,82 | 0,002 |  |
| Lac | 1,467 | 0,635 | (0,084; 2,849) | 2,31 | 0,039 | 1,18 |
| HPMC\_Visc | -0,049 | 0,663 | (-1,494; 1,395) | -0,07 | 0,942 | 1,70 |
| HPMC\_HP | 1,094 | 0,649 | (-0,319; 2,508) | 1,69 | 0,117 | 1,26 |
| HPMC\_PS | 0,901 | 0,995 | (-1,267; 3,069) | 0,91 | 0,383 | 2,09 |
| Lac\*Lac | 2,38 | 1,26 | (-0,37; 5,13) | 1,89 | 0,084 | 1,30 |
| HPMC\_Visc\*HPMC\_Visc | 0,47 | 1,34 | (-2,45; 3,39) | 0,35 | 0,730 | 1,96 |
| HPMC\_HP\*HPMC\_HP | -0,94 | 1,27 | (-3,71; 1,83) | -0,74 | 0,474 | 1,81 |
| HPMC\_PS\*HPMC\_PS | -1,00 | 1,26 | (-3,76; 1,75) | -0,79 | 0,444 | 1,42 |
| Lac\*HPMC\_Visc | -0,18 | 1,39 | (-3,21; 2,85) | -0,13 | 0,900 | 1,49 |
| Lac\*HPMC\_HP | 2,25 | 1,46 | (-0,93; 5,43) | 1,54 | 0,149 | 1,17 |
| Lac\*HPMC\_PS | 2,52 | 2,25 | (-2,38; 7,43) | 1,12 | 0,284 | 1,42 |
| HPMC\_Visc\*HPMC\_HP | 1,31 | 1,66 | (-2,31; 4,93) | 0,79 | 0,444 | 2,73 |
| HPMC\_Visc\*HPMC\_PS | 1,44 | 2,35 | (-3,67; 6,55) | 0,61 | 0,552 | 2,74 |
| HPMC\_HP\*HPMC\_PS | 3,14 | 2,59 | (-2,50; 8,78) | 1,21 | 0,248 | 2,70 |

## Model Summary

| S | R-sq | R-sq(adj) | PRESS | R-sq(pred) | AICc | BIC |
| --- | --- | --- | --- | --- | --- | --- |
| 1,42882 | 66,16% | 26,68% | 99,1696 | 0,00% | 160,40 | 126,73 |

## Analysis of Variance

| Source | DF | Seq SS | Contribution | Adj SS | Adj MS | F-Value | P-Value |
| --- | --- | --- | --- | --- | --- | --- | --- |
| Model | 14 | 47,8996 | 66,16% | 47,8996 | 3,4214 | 1,68 | 0,188 |
| Linear | 4 | 16,8879 | 23,33% | 19,2478 | 4,8120 | 2,36 | 0,112 |
| Lac | 1 | 8,2053 | 11,33% | 10,9036 | 10,9036 | 5,34 | 0,039 |
| HPMC\_Visc | 1 | 1,8549 | 2,56% | 0,0113 | 0,0113 | 0,01 | 0,942 |
| HPMC\_HP | 1 | 6,7771 | 9,36% | 5,8101 | 5,8101 | 2,85 | 0,117 |
| HPMC\_PS | 1 | 0,0506 | 0,07% | 1,6745 | 1,6745 | 0,82 | 0,383 |
| Square | 4 | 15,9841 | 22,08% | 15,8070 | 3,9518 | 1,94 | 0,169 |
| Lac\*Lac | 1 | 12,3921 | 17,12% | 7,2621 | 7,2621 | 3,56 | 0,084 |
| HPMC\_Visc\*HPMC\_Visc | 1 | 0,1915 | 0,26% | 0,2540 | 0,2540 | 0,12 | 0,730 |
| HPMC\_HP\*HPMC\_HP | 1 | 0,4911 | 0,68% | 1,1154 | 1,1154 | 0,55 | 0,474 |
| HPMC\_PS\*HPMC\_PS | 1 | 2,9093 | 4,02% | 1,2806 | 1,2806 | 0,63 | 0,444 |
| 2-Way Interaction | 6 | 15,0276 | 20,76% | 15,0276 | 2,5046 | 1,23 | 0,358 |
| Lac\*HPMC\_Visc | 1 | 1,0315 | 1,42% | 0,0337 | 0,0337 | 0,02 | 0,900 |
| Lac\*HPMC\_HP | 1 | 7,0934 | 9,80% | 4,8615 | 4,8615 | 2,38 | 0,149 |
| Lac\*HPMC\_PS | 1 | 2,5631 | 3,54% | 2,5631 | 2,5631 | 1,26 | 0,284 |
| HPMC\_Visc\*HPMC\_HP | 1 | 0,0346 | 0,05% | 1,2763 | 1,2763 | 0,63 | 0,444 |
| HPMC\_Visc\*HPMC\_PS | 1 | 1,2955 | 1,79% | 0,7660 | 0,7660 | 0,38 | 0,552 |
| HPMC\_HP\*HPMC\_PS | 1 | 3,0095 | 4,16% | 3,0095 | 3,0095 | 1,47 | 0,248 |
| Error | 12 | 24,4984 | 33,84% | 24,4984 | 2,0415 |  |  |
| Lack-of-Fit | 10 | 13,2231 | 18,26% | 13,2231 | 1,3223 | 0,23 | 0,954 |
| Pure Error | 2 | 11,2753 | 15,57% | 11,2753 | 5,6377 |  |  |
| Total | 26 | 72,3980 | 100,00% |  |  |  |  |

## Regression Equation in Uncoded Units

|  |  |  |
| --- | --- | --- |
| F\_SD\_8h(480min) | = | 281 - 209 Lac - 0,00741 HPMC\_Visc - 19,9 HPMC\_HP - 2,65 HPMC\_PS + 38,1 Lac\*Lac + 0,000000 HPMC\_Visc\*HPMC\_Visc - 0,91 HPMC\_HP\*HPMC\_HP - 0,0185 HPMC\_PS\*HPMC\_PS - 0,00018 Lac\*HPMC\_Visc + 8,88 Lac\*HPMC\_HP + 1,37 Lac\*HPMC\_PS + 0,000333 HPMC\_Visc\*HPMC\_HP + 0,000050 HPMC\_Visc\*HPMC\_PS + 0,421 HPMC\_HP\*HPMC\_PS |

## Fits and Diagnostics for All Observations

| Obs | F\_SD\_8h(480min) | Fit | SE Fit | 95% CI | Resid | Std Resid | Del Resid | HI |
| --- | --- | --- | --- | --- | --- | --- | --- | --- |
| 1 | 4,53 | 4,05 | 1,16 | (1,53; 6,58) | 0,48 | 0,57 | 0,56 | 0,655994 |
| 2 | 3,29 | 3,40 | 1,16 | (0,88; 5,92) | -0,11 | -0,13 | -0,12 | 0,655994 |
| 3 | 1,77 | 2,53 | 1,22 | (-0,13; 5,19) | -0,75 | -1,02 | -1,02 | 0,729945 |
| 4 | 0,72 | 0,71 | 1,22 | (-1,95; 3,37) | 0,00 | 0,01 | 0,00 | 0,729945 |
| 5 | 3,38 | 2,83 | 0,99 | (0,68; 4,98) | 0,55 | 0,54 | 0,52 | 0,476813 |
| 6 | 5,53 | 4,44 | 0,99 | (2,29; 6,59) | 1,09 | 1,06 | 1,06 | 0,476813 |
| 7 | 2,94 | 2,29 | 1,19 | (-0,30; 4,87) | 0,65 | 0,82 | 0,80 | 0,688750 |
| 8 | 3,15 | 3,71 | 1,19 | (1,13; 6,29) | -0,56 | -0,70 | -0,68 | 0,688750 |
| 9 | 1,54 | 1,58 | 1,09 | (-0,80; 3,95) | -0,04 | -0,04 | -0,04 | 0,580949 |
| 10 | 2,24 | 2,68 | 1,09 | (0,31; 5,05) | -0,44 | -0,48 | -0,46 | 0,580949 |
| 11 | 1,58 | 2,12 | 0,93 | (0,09; 4,15) | -0,54 | -0,50 | -0,48 | 0,425781 |
| 12 | 1,99 | 3,02 | 0,93 | (0,98; 5,05) | -1,03 | -0,95 | -0,95 | 0,425781 |
| 13 | 2,43 | 2,14 | 1,08 | (-0,21; 4,49) | 0,29 | 0,31 | 0,30 | 0,569258 |
| 14 | 6,27 | 5,96 | 1,08 | (3,61; 8,31) | 0,31 | 0,33 | 0,31 | 0,569258 |
| 15 | 1,85 | 2,96 | 0,94 | (0,91; 5,01) | -1,11 | -1,03 | -1,03 | 0,433227 |
| 16 | 5,07 | 5,44 | 0,94 | (3,39; 7,49) | -0,36 | -0,34 | -0,32 | 0,433227 |
| 17 | 4,02 | 3,98 | 1,10 | (1,57; 6,39) | 0,04 | 0,04 | 0,04 | 0,597775 |
| 18 | 6,91 | 6,56 | 1,10 | (4,15; 8,97) | 0,35 | 0,39 | 0,37 | 0,597775 |
| 19 | 1,59 | 3,38 | 0,91 | (1,40; 5,37) | -1,79 | -1,63 | -1,76 | 0,407038 |
| 20 | 3,88 | 2,64 | 1,15 | (0,14; 5,15) | 1,24 | 1,46 | 1,54 | 0,647412 |
| 21 | 2,47 | 1,41 | 0,91 | (-0,58; 3,40) | 1,06 | 0,97 | 0,96 | 0,407933 |
| 22 | 2,71 | 3,15 | 1,29 | (0,34; 5,97) | -0,45 | -0,73 | -0,72 | 0,817044 |
| 23 | 1,18 | 1,62 | 1,30 | (-1,21; 4,44) | -0,44 | -0,73 | -0,71 | 0,823434 |
| 24 | 2,47 | 1,79 | 1,21 | (-0,84; 4,42) | 0,68 | 0,89 | 0,88 | 0,713775 |
| 25 | 1,36 | 2,89 | 0,77 | (1,22; 4,56) | -1,53 | -1,27 | -1,31 | 0,288793 |
| 26 | 5,87 | 2,89 | 0,77 | (1,22; 4,56) | 2,98 | 2,47 | 3,37 | 0,288793 |
| 27 | 2,32 | 2,89 | 0,77 | (1,22; 4,56) | -0,57 | -0,48 | -0,46 | 0,288793 |

| Obs | Cook’s D | DFITS |  |
| --- | --- | --- | --- |
| 1 | 0,04 | 0,76795 |  |
| 2 | 0,00 | -0,16806 |  |
| 3 | 0,19 | -1,67170 |  |
| 4 | 0,00 | 0,00809 |  |
| 5 | 0,02 | 0,49554 |  |
| 6 | 0,07 | 1,01475 |  |
| 7 | 0,10 | 1,19582 |  |
| 8 | 0,07 | -1,01533 |  |
| 9 | 0,00 | -0,04859 |  |
| 10 | 0,02 | -0,54589 |  |
| 11 | 0,01 | -0,41642 |  |
| 12 | 0,04 | -0,81579 |  |
| 13 | 0,01 | 0,33965 |  |
| 14 | 0,01 | 0,35967 |  |
| 15 | 0,05 | -0,90322 |  |
| 16 | 0,01 | -0,28298 |  |
| 17 | 0,00 | 0,05075 |  |
| 18 | 0,02 | 0,45704 |  |
| 19 | 0,12 | -1,46233 |  |
| 20 | 0,26 | 2,08661 |  |
| 21 | 0,04 | 0,79900 |  |
| 22 | 0,16 | -1,51215 |  |
| 23 | 0,16 | -1,53397 |  |
| 24 | 0,13 | 1,38627 |  |
| 25 | 0,04 | -0,83333 |  |
| 26 | 0,17 | 2,14875 | R |
| 27 | 0,01 | -0,29288 |  |

R  Large residual

## Coded Coefficients

| Term | Coef | SE Coef | 95% CI | T-Value | P-Value | VIF |
| --- | --- | --- | --- | --- | --- | --- |
| Constant | 2,984 | 0,786 | (1,271; 4,696) | 3,79 | 0,003 |  |
| Lac | 1,493 | 0,650 | (0,076; 2,909) | 2,30 | 0,040 | 1,18 |
| HPMC\_Visc | -0,145 | 0,679 | (-1,625; 1,335) | -0,21 | 0,834 | 1,70 |
| HPMC\_HP | 1,205 | 0,665 | (-0,243; 2,653) | 1,81 | 0,095 | 1,26 |
| HPMC\_PS | 0,88 | 1,02 | (-1,34; 3,10) | 0,86 | 0,405 | 2,09 |
| Lac\*Lac | 2,70 | 1,29 | (-0,11; 5,52) | 2,09 | 0,058 | 1,30 |
| HPMC\_Visc\*HPMC\_Visc | 0,15 | 1,37 | (-2,84; 3,14) | 0,11 | 0,915 | 1,96 |
| HPMC\_HP\*HPMC\_HP | -0,90 | 1,30 | (-3,74; 1,94) | -0,69 | 0,502 | 1,81 |
| HPMC\_PS\*HPMC\_PS | -1,13 | 1,30 | (-3,96; 1,69) | -0,88 | 0,399 | 1,42 |
| Lac\*HPMC\_Visc | -0,51 | 1,43 | (-3,61; 2,60) | -0,36 | 0,727 | 1,49 |
| Lac\*HPMC\_HP | 2,16 | 1,50 | (-1,10; 5,42) | 1,44 | 0,175 | 1,17 |
| Lac\*HPMC\_PS | 1,78 | 2,31 | (-3,25; 6,81) | 0,77 | 0,456 | 1,42 |
| HPMC\_Visc\*HPMC\_HP | 0,97 | 1,70 | (-2,74; 4,67) | 0,57 | 0,580 | 2,73 |
| HPMC\_Visc\*HPMC\_PS | 0,99 | 2,40 | (-4,25; 6,22) | 0,41 | 0,688 | 2,74 |
| HPMC\_HP\*HPMC\_PS | 2,84 | 2,65 | (-2,94; 8,62) | 1,07 | 0,305 | 2,70 |

## Model Summary

| S | R-sq | R-sq(adj) | PRESS | R-sq(pred) | AICc | BIC |
| --- | --- | --- | --- | --- | --- | --- |
| 1,46365 | 66,96% | 28,41% | 100,901 | 0,00% | 161,70 | 128,03 |

## Analysis of Variance

| Source | DF | Seq SS | Contribution | Adj SS | Adj MS | F-Value | P-Value |
| --- | --- | --- | --- | --- | --- | --- | --- |
| Model | 14 | 52,0945 | 66,96% | 52,0945 | 3,7210 | 1,74 | 0,172 |
| Linear | 4 | 20,7686 | 26,69% | 21,3305 | 5,3326 | 2,49 | 0,099 |
| Lac | 1 | 9,5266 | 12,24% | 11,2937 | 11,2937 | 5,27 | 0,040 |
| HPMC\_Visc | 1 | 1,8452 | 2,37% | 0,0980 | 0,0980 | 0,05 | 0,834 |
| HPMC\_HP | 1 | 9,1419 | 11,75% | 7,0389 | 7,0389 | 3,29 | 0,095 |
| HPMC\_PS | 1 | 0,2549 | 0,33% | 1,5962 | 1,5962 | 0,75 | 0,405 |
| Square | 4 | 19,5557 | 25,14% | 19,1634 | 4,7908 | 2,24 | 0,126 |
| Lac\*Lac | 1 | 16,2945 | 20,94% | 9,3673 | 9,3673 | 4,37 | 0,058 |
| HPMC\_Visc\*HPMC\_Visc | 1 | 0,0304 | 0,04% | 0,0257 | 0,0257 | 0,01 | 0,915 |
| HPMC\_HP\*HPMC\_HP | 1 | 0,3657 | 0,47% | 1,0283 | 1,0283 | 0,48 | 0,502 |
| HPMC\_PS\*HPMC\_PS | 1 | 2,8651 | 3,68% | 1,6407 | 1,6407 | 0,77 | 0,399 |
| 2-Way Interaction | 6 | 11,7701 | 15,13% | 11,7701 | 1,9617 | 0,92 | 0,516 |
| Lac\*HPMC\_Visc | 1 | 1,2968 | 1,67% | 0,2731 | 0,2731 | 0,13 | 0,727 |
| Lac\*HPMC\_HP | 1 | 6,0059 | 7,72% | 4,4563 | 4,4563 | 2,08 | 0,175 |
| Lac\*HPMC\_PS | 1 | 1,2735 | 1,64% | 1,2735 | 1,2735 | 0,59 | 0,456 |
| HPMC\_Visc\*HPMC\_HP | 1 | 0,0336 | 0,04% | 0,6917 | 0,6917 | 0,32 | 0,580 |
| HPMC\_Visc\*HPMC\_PS | 1 | 0,7018 | 0,90% | 0,3625 | 0,3625 | 0,17 | 0,688 |
| HPMC\_HP\*HPMC\_PS | 1 | 2,4585 | 3,16% | 2,4585 | 2,4585 | 1,15 | 0,305 |
| Error | 12 | 25,7071 | 33,04% | 25,7071 | 2,1423 |  |  |
| Lack-of-Fit | 10 | 14,0083 | 18,01% | 14,0083 | 1,4008 | 0,24 | 0,952 |
| Pure Error | 2 | 11,6989 | 15,04% | 11,6989 | 5,8494 |  |  |
| Total | 26 | 77,8016 | 100,00% |  |  |  |  |

## Regression Equation in Uncoded Units

|  |  |  |
| --- | --- | --- |
| F\_SD\_9h(540min) | = | 199 - 178 Lac - 0,00477 HPMC\_Visc - 16,3 HPMC\_HP - 1,52 HPMC\_PS + 43,2 Lac\*Lac + 0,000000 HPMC\_Visc\*HPMC\_Visc - 0,88 HPMC\_HP\*HPMC\_HP - 0,0210 HPMC\_PS\*HPMC\_PS - 0,00052 Lac\*HPMC\_Visc + 8,50 Lac\*HPMC\_HP + 0,97 Lac\*HPMC\_PS + 0,000245 HPMC\_Visc\*HPMC\_HP + 0,000035 HPMC\_Visc\*HPMC\_PS + 0,381 HPMC\_HP\*HPMC\_PS |

## Fits and Diagnostics for All Observations

| Obs | F\_SD\_9h(540min) | Fit | SE Fit | 95% CI | Resid | Std Resid | Del Resid | HI |
| --- | --- | --- | --- | --- | --- | --- | --- | --- |
| 1 | 3,66 | 3,46 | 1,19 | (0,88; 6,04) | 0,20 | 0,23 | 0,22 | 0,655994 |
| 2 | 3,34 | 3,32 | 1,19 | (0,74; 5,90) | 0,02 | 0,02 | 0,02 | 0,655994 |
| 3 | 1,76 | 2,42 | 1,25 | (-0,30; 5,15) | -0,66 | -0,87 | -0,86 | 0,729945 |
| 4 | 0,70 | 0,91 | 1,25 | (-1,81; 3,64) | -0,21 | -0,27 | -0,26 | 0,729945 |
| 5 | 3,46 | 2,74 | 1,01 | (0,54; 4,94) | 0,72 | 0,68 | 0,66 | 0,476813 |
| 6 | 5,48 | 4,70 | 1,01 | (2,50; 6,90) | 0,78 | 0,74 | 0,72 | 0,476813 |
| 7 | 2,83 | 2,28 | 1,21 | (-0,36; 4,93) | 0,54 | 0,67 | 0,65 | 0,688750 |
| 8 | 3,23 | 3,74 | 1,21 | (1,10; 6,39) | -0,51 | -0,63 | -0,61 | 0,688750 |
| 9 | 1,59 | 1,58 | 1,12 | (-0,85; 4,01) | 0,01 | 0,01 | 0,01 | 0,580949 |
| 10 | 2,14 | 2,69 | 1,12 | (0,26; 5,12) | -0,55 | -0,58 | -0,57 | 0,580949 |
| 11 | 1,63 | 2,29 | 0,96 | (0,21; 4,37) | -0,65 | -0,59 | -0,57 | 0,425781 |
| 12 | 1,86 | 3,03 | 0,96 | (0,95; 5,11) | -1,18 | -1,06 | -1,07 | 0,425781 |
| 13 | 2,78 | 2,47 | 1,10 | (0,06; 4,87) | 0,31 | 0,32 | 0,31 | 0,569258 |
| 14 | 6,39 | 6,12 | 1,10 | (3,72; 8,53) | 0,27 | 0,28 | 0,27 | 0,569258 |
| 15 | 1,94 | 3,12 | 0,96 | (1,02; 5,22) | -1,18 | -1,07 | -1,07 | 0,433227 |
| 16 | 4,94 | 5,32 | 0,96 | (3,22; 7,41) | -0,37 | -0,34 | -0,32 | 0,433227 |
| 17 | 4,28 | 4,23 | 1,13 | (1,76; 6,70) | 0,05 | 0,05 | 0,05 | 0,597775 |
| 18 | 7,62 | 7,05 | 1,13 | (4,58; 9,51) | 0,57 | 0,61 | 0,59 | 0,597775 |
| 19 | 1,52 | 3,22 | 0,93 | (1,18; 5,25) | -1,70 | -1,51 | -1,60 | 0,407038 |
| 20 | 3,77 | 2,37 | 1,18 | (-0,19; 4,94) | 1,40 | 1,61 | 1,74 | 0,647412 |
| 21 | 2,59 | 1,29 | 0,93 | (-0,74; 3,33) | 1,30 | 1,15 | 1,17 | 0,407933 |
| 22 | 2,95 | 3,39 | 1,32 | (0,50; 6,27) | -0,43 | -0,69 | -0,68 | 0,817044 |
| 23 | 1,02 | 1,41 | 1,33 | (-1,48; 4,30) | -0,39 | -0,64 | -0,62 | 0,823434 |
| 24 | 2,55 | 1,83 | 1,24 | (-0,86; 4,53) | 0,72 | 0,92 | 0,91 | 0,713775 |
| 25 | 1,50 | 2,94 | 0,79 | (1,22; 4,65) | -1,44 | -1,16 | -1,18 | 0,288793 |
| 26 | 6,01 | 2,94 | 0,79 | (1,22; 4,65) | 3,08 | 2,49 | 3,43 | 0,288793 |
| 27 | 2,25 | 2,94 | 0,79 | (1,22; 4,65) | -0,69 | -0,56 | -0,54 | 0,288793 |

| Obs | Cook’s D | DFITS |  |
| --- | --- | --- | --- |
| 1 | 0,01 | 0,30755 |  |
| 2 | 0,00 | 0,02511 |  |
| 3 | 0,14 | -1,41091 |  |
| 4 | 0,01 | -0,43010 |  |
| 5 | 0,03 | 0,63237 |  |
| 6 | 0,03 | 0,69052 |  |
| 7 | 0,07 | 0,96575 |  |
| 8 | 0,06 | -0,90933 |  |
| 9 | 0,00 | 0,01232 |  |
| 10 | 0,03 | -0,66561 |  |
| 11 | 0,02 | -0,49384 |  |
| 12 | 0,06 | -0,91748 |  |
| 13 | 0,01 | 0,35775 |  |
| 14 | 0,01 | 0,31333 |  |
| 15 | 0,06 | -0,93911 |  |
| 16 | 0,01 | -0,28333 |  |
| 17 | 0,00 | 0,05949 |  |
| 18 | 0,04 | 0,72389 |  |
| 19 | 0,10 | -1,32586 |  |
| 20 | 0,32 | 2,35384 |  |
| 21 | 0,06 | 0,97266 |  |
| 22 | 0,14 | -1,43255 |  |
| 23 | 0,13 | -1,33625 |  |
| 24 | 0,14 | 1,43789 |  |
| 25 | 0,04 | -0,75368 |  |
| 26 | 0,17 | 2,18835 | R |
| 27 | 0,01 | -0,34576 |  |

R  Large residual

## Coded Coefficients

| Term | Coef | SE Coef | 95% CI | T-Value | P-Value | VIF |
| --- | --- | --- | --- | --- | --- | --- |
| Constant | 2,854 | 0,897 | (0,899; 4,808) | 3,18 | 0,008 |  |
| Lac | 1,919 | 0,742 | (0,303; 3,535) | 2,59 | 0,024 | 1,18 |
| HPMC\_Visc | -0,275 | 0,775 | (-1,964; 1,414) | -0,35 | 0,729 | 1,70 |
| HPMC\_HP | 1,256 | 0,758 | (-0,396; 2,909) | 1,66 | 0,123 | 1,26 |
| HPMC\_PS | 0,91 | 1,16 | (-1,62; 3,44) | 0,78 | 0,449 | 2,09 |
| Lac\*Lac | 4,12 | 1,47 | (0,91; 7,34) | 2,80 | 0,016 | 1,30 |
| HPMC\_Visc\*HPMC\_Visc | -0,02 | 1,57 | (-3,44; 3,39) | -0,02 | 0,988 | 1,96 |
| HPMC\_HP\*HPMC\_HP | -0,91 | 1,49 | (-4,15; 2,33) | -0,61 | 0,552 | 1,81 |
| HPMC\_PS\*HPMC\_PS | -1,12 | 1,48 | (-4,33; 2,10) | -0,75 | 0,465 | 1,42 |
| Lac\*HPMC\_Visc | -0,50 | 1,63 | (-4,04; 3,04) | -0,31 | 0,764 | 1,49 |
| Lac\*HPMC\_HP | 2,46 | 1,71 | (-1,26; 6,18) | 1,44 | 0,175 | 1,17 |
| Lac\*HPMC\_PS | 1,66 | 2,63 | (-4,08; 7,40) | 0,63 | 0,540 | 1,42 |
| HPMC\_Visc\*HPMC\_HP | 1,15 | 1,94 | (-3,08; 5,38) | 0,59 | 0,564 | 2,73 |
| HPMC\_Visc\*HPMC\_PS | 1,04 | 2,74 | (-4,93; 7,02) | 0,38 | 0,710 | 2,74 |
| HPMC\_HP\*HPMC\_PS | 3,25 | 3,03 | (-3,34; 9,84) | 1,07 | 0,304 | 2,70 |

## Model Summary

| S | R-sq | R-sq(adj) | PRESS | R-sq(pred) | AICc | BIC |
| --- | --- | --- | --- | --- | --- | --- |
| 1,67004 | 70,74% | 36,59% | 145,989 | 0,00% | 168,82 | 135,15 |

## Analysis of Variance

| Source | DF | Seq SS | Contribution | Adj SS | Adj MS | F-Value | P-Value |
| --- | --- | --- | --- | --- | --- | --- | --- |
| Model | 14 | 80,896 | 70,74% | 80,8962 | 5,7783 | 2,07 | 0,107 |
| Linear | 4 | 29,053 | 25,40% | 30,5091 | 7,6273 | 2,73 | 0,079 |
| Lac | 1 | 16,269 | 14,23% | 18,6684 | 18,6684 | 6,69 | 0,024 |
| HPMC\_Visc | 1 | 3,213 | 2,81% | 0,3510 | 0,3510 | 0,13 | 0,729 |
| HPMC\_HP | 1 | 9,379 | 8,20% | 7,6564 | 7,6564 | 2,75 | 0,123 |
| HPMC\_PS | 1 | 0,192 | 0,17% | 1,7061 | 1,7061 | 0,61 | 0,449 |
| Square | 4 | 38,065 | 33,28% | 37,3843 | 9,3461 | 3,35 | 0,046 |
| Lac\*Lac | 1 | 34,930 | 30,54% | 21,8271 | 21,8271 | 7,83 | 0,016 |
| HPMC\_Visc\*HPMC\_Visc | 1 | 0,007 | 0,01% | 0,0007 | 0,0007 | 0,00 | 0,988 |
| HPMC\_HP\*HPMC\_HP | 1 | 0,292 | 0,26% | 1,0466 | 1,0466 | 0,38 | 0,552 |
| HPMC\_PS\*HPMC\_PS | 1 | 2,835 | 2,48% | 1,5883 | 1,5883 | 0,57 | 0,465 |
| 2-Way Interaction | 6 | 13,779 | 12,05% | 13,7786 | 2,2964 | 0,82 | 0,573 |
| Lac\*HPMC\_Visc | 1 | 1,127 | 0,99% | 0,2635 | 0,2635 | 0,09 | 0,764 |
| Lac\*HPMC\_HP | 1 | 7,481 | 6,54% | 5,7861 | 5,7861 | 2,07 | 0,175 |
| Lac\*HPMC\_PS | 1 | 1,108 | 0,97% | 1,1078 | 1,1078 | 0,40 | 0,540 |
| HPMC\_Visc\*HPMC\_HP | 1 | 0,028 | 0,02% | 0,9796 | 0,9796 | 0,35 | 0,564 |
| HPMC\_Visc\*HPMC\_PS | 1 | 0,818 | 0,72% | 0,4042 | 0,4042 | 0,14 | 0,710 |
| HPMC\_HP\*HPMC\_PS | 1 | 3,215 | 2,81% | 3,2152 | 3,2152 | 1,15 | 0,304 |
| Error | 12 | 33,468 | 29,26% | 33,4684 | 2,7890 |  |  |
| Lack-of-Fit | 10 | 20,742 | 18,14% | 20,7422 | 2,0742 | 0,33 | 0,909 |
| Pure Error | 2 | 12,726 | 11,13% | 12,7263 | 6,3631 |  |  |
| Total | 26 | 114,365 | 100,00% |  |  |  |  |

## Regression Equation in Uncoded Units

|  |  |  |
| --- | --- | --- |
| F\_SD\_10h(600min) | = | 249 - 206 Lac - 0,00507 HPMC\_Visc - 21,1 HPMC\_HP - 2,07 HPMC\_PS + 66,0 Lac\*Lac - 0,000000 HPMC\_Visc\*HPMC\_Visc - 0,88 HPMC\_HP\*HPMC\_HP - 0,0206 HPMC\_PS\*HPMC\_PS - 0,00051 Lac\*HPMC\_Visc + 9,69 Lac\*HPMC\_HP + 0,90 Lac\*HPMC\_PS + 0,000291 HPMC\_Visc\*HPMC\_HP + 0,000036 HPMC\_Visc\*HPMC\_PS + 0,435 HPMC\_HP\*HPMC\_PS |

## Fits and Diagnostics for All Observations

| Obs | F\_SD\_10h(600min) | Fit | SE Fit | 95% CI | Resid | Std Resid | Del Resid | HI |
| --- | --- | --- | --- | --- | --- | --- | --- | --- |
| 1 | 4,25 | 3,70 | 1,35 | (0,76; 6,65) | 0,55 | 0,56 | 0,54 | 0,655994 |
| 2 | 3,39 | 3,80 | 1,35 | (0,85; 6,74) | -0,40 | -0,41 | -0,40 | 0,655994 |
| 3 | 1,73 | 2,34 | 1,43 | (-0,77; 5,45) | -0,61 | -0,70 | -0,69 | 0,729945 |
| 4 | 0,69 | 1,11 | 1,43 | (-2,00; 4,22) | -0,41 | -0,48 | -0,46 | 0,729945 |
| 5 | 3,53 | 2,70 | 1,15 | (0,19; 5,21) | 0,83 | 0,69 | 0,67 | 0,476813 |
| 6 | 5,40 | 5,16 | 1,15 | (2,65; 7,68) | 0,23 | 0,19 | 0,18 | 0,476813 |
| 7 | 2,76 | 2,00 | 1,39 | (-1,02; 5,02) | 0,76 | 0,81 | 0,80 | 0,688750 |
| 8 | 3,23 | 4,09 | 1,39 | (1,07; 7,10) | -0,86 | -0,92 | -0,91 | 0,688750 |
| 9 | 1,61 | 1,66 | 1,27 | (-1,12; 4,43) | -0,04 | -0,04 | -0,04 | 0,580949 |
| 10 | 2,22 | 2,94 | 1,27 | (0,17; 5,71) | -0,72 | -0,66 | -0,65 | 0,580949 |
| 11 | 1,59 | 2,28 | 1,09 | (-0,09; 4,65) | -0,69 | -0,54 | -0,53 | 0,425781 |
| 12 | 1,84 | 3,28 | 1,09 | (0,91; 5,66) | -1,44 | -1,14 | -1,15 | 0,425781 |
| 13 | 3,04 | 2,51 | 1,26 | (-0,24; 5,26) | 0,53 | 0,48 | 0,47 | 0,569258 |
| 14 | 6,55 | 6,65 | 1,26 | (3,90; 9,39) | -0,09 | -0,08 | -0,08 | 0,569258 |
| 15 | 1,97 | 3,12 | 1,10 | (0,72; 5,51) | -1,15 | -0,91 | -0,91 | 0,433227 |
| 16 | 4,89 | 5,76 | 1,10 | (3,37; 8,16) | -0,88 | -0,70 | -0,68 | 0,433227 |
| 17 | 4,49 | 5,13 | 1,29 | (2,31; 7,94) | -0,63 | -0,60 | -0,58 | 0,597775 |
| 18 | 10,50 | 8,77 | 1,29 | (5,96; 11,59) | 1,73 | 1,64 | 1,78 | 0,597775 |
| 19 | 1,58 | 3,05 | 1,07 | (0,73; 5,37) | -1,46 | -1,14 | -1,15 | 0,407038 |
| 20 | 3,59 | 1,90 | 1,34 | (-1,03; 4,82) | 1,70 | 1,71 | 1,89 | 0,647412 |
| 21 | 2,69 | 1,20 | 1,07 | (-1,13; 3,52) | 1,49 | 1,16 | 1,18 | 0,407933 |
| 22 | 3,05 | 3,31 | 1,51 | (0,03; 6,60) | -0,26 | -0,37 | -0,35 | 0,817044 |
| 23 | 0,95 | 1,33 | 1,52 | (-1,97; 4,64) | -0,38 | -0,54 | -0,53 | 0,823434 |
| 24 | 2,60 | 1,69 | 1,41 | (-1,39; 4,76) | 0,91 | 1,02 | 1,02 | 0,713775 |
| 25 | 1,48 | 2,83 | 0,90 | (0,87; 4,78) | -1,35 | -0,96 | -0,95 | 0,288793 |
| 26 | 6,15 | 2,83 | 0,90 | (0,87; 4,78) | 3,32 | 2,36 | 3,08 | 0,288793 |
| 27 | 2,15 | 2,83 | 0,90 | (0,87; 4,78) | -0,67 | -0,48 | -0,46 | 0,288793 |

| Obs | Cook’s D | DFITS |  |
| --- | --- | --- | --- |
| 1 | 0,04 | 0,74627 |  |
| 2 | 0,02 | -0,54659 |  |
| 3 | 0,09 | -1,13120 |  |
| 4 | 0,04 | -0,75765 |  |
| 5 | 0,03 | 0,64092 |  |
| 6 | 0,00 | 0,17616 |  |
| 7 | 0,10 | 1,19105 |  |
| 8 | 0,12 | -1,35643 |  |
| 9 | 0,00 | -0,04483 |  |
| 10 | 0,04 | -0,76071 |  |
| 11 | 0,01 | -0,45330 |  |
| 12 | 0,06 | -0,99267 |  |
| 13 | 0,02 | 0,53612 |  |
| 14 | 0,00 | -0,09314 |  |
| 15 | 0,04 | -0,79352 |  |
| 16 | 0,02 | -0,59486 |  |
| 17 | 0,04 | -0,71002 |  |
| 18 | 0,27 | 2,16556 |  |
| 19 | 0,06 | -0,95600 |  |
| 20 | 0,36 | 2,55461 |  |
| 21 | 0,06 | 0,97756 |  |
| 22 | 0,04 | -0,74676 |  |
| 23 | 0,09 | -1,13653 |  |
| 24 | 0,17 | 1,61280 |  |
| 25 | 0,02 | -0,60634 |  |
| 26 | 0,15 | 1,96360 | R |
| 27 | 0,01 | -0,29449 |  |

R  Large residual

## Coded Coefficients

| Term | Coef | SE Coef | 95% CI | T-Value | P-Value | VIF |
| --- | --- | --- | --- | --- | --- | --- |
| Constant | 2,845 | 0,894 | (0,896; 4,793) | 3,18 | 0,008 |  |
| Lac | 2,012 | 0,739 | (0,401; 3,623) | 2,72 | 0,019 | 1,18 |
| HPMC\_Visc | -0,498 | 0,772 | (-2,181; 1,185) | -0,64 | 0,532 | 1,70 |
| HPMC\_HP | 1,417 | 0,756 | (-0,230; 3,063) | 1,87 | 0,085 | 1,26 |
| HPMC\_PS | 1,03 | 1,16 | (-1,50; 3,55) | 0,89 | 0,393 | 2,09 |
| Lac\*Lac | 4,29 | 1,47 | (1,09; 7,49) | 2,92 | 0,013 | 1,30 |
| HPMC\_Visc\*HPMC\_Visc | -0,09 | 1,56 | (-3,49; 3,31) | -0,06 | 0,956 | 1,96 |
| HPMC\_HP\*HPMC\_HP | -0,87 | 1,48 | (-4,10; 2,36) | -0,59 | 0,568 | 1,81 |
| HPMC\_PS\*HPMC\_PS | -1,19 | 1,47 | (-4,40; 2,02) | -0,81 | 0,435 | 1,42 |
| Lac\*HPMC\_Visc | -0,52 | 1,62 | (-4,05; 3,01) | -0,32 | 0,754 | 1,49 |
| Lac\*HPMC\_HP | 2,61 | 1,70 | (-1,10; 6,32) | 1,53 | 0,151 | 1,17 |
| Lac\*HPMC\_PS | 2,03 | 2,63 | (-3,69; 7,75) | 0,77 | 0,454 | 1,42 |
| HPMC\_Visc\*HPMC\_HP | 1,13 | 1,93 | (-3,08; 5,35) | 0,58 | 0,570 | 2,73 |
| HPMC\_Visc\*HPMC\_PS | 0,49 | 2,73 | (-5,46; 6,45) | 0,18 | 0,860 | 2,74 |
| HPMC\_HP\*HPMC\_PS | 3,76 | 3,02 | (-2,81; 10,33) | 1,25 | 0,236 | 2,70 |

## Model Summary

| S | R-sq | R-sq(adj) | PRESS | R-sq(pred) | AICc | BIC |
| --- | --- | --- | --- | --- | --- | --- |
| 1,66454 | 73,48% | 42,54% | 137,890 | 0,00% | 168,64 | 134,98 |

## Analysis of Variance

| Source | DF | Seq SS | Contribution | Adj SS | Adj MS | F-Value | P-Value |
| --- | --- | --- | --- | --- | --- | --- | --- |
| Model | 14 | 92,131 | 73,48% | 92,1309 | 6,5808 | 2,38 | 0,070 |
| Linear | 4 | 35,039 | 27,95% | 37,6717 | 9,4179 | 3,40 | 0,044 |
| Lac | 1 | 17,726 | 14,14% | 20,5250 | 20,5250 | 7,41 | 0,019 |
| HPMC\_Visc | 1 | 5,206 | 4,15% | 1,1500 | 1,1500 | 0,42 | 0,532 |
| HPMC\_HP | 1 | 11,584 | 9,24% | 9,7354 | 9,7354 | 3,51 | 0,085 |
| HPMC\_PS | 1 | 0,523 | 0,42% | 2,1756 | 2,1756 | 0,79 | 0,393 |
| Square | 4 | 40,240 | 32,09% | 40,2708 | 10,0677 | 3,63 | 0,037 |
| Lac\*Lac | 1 | 37,772 | 30,13% | 23,5934 | 23,5934 | 8,52 | 0,013 |
| HPMC\_Visc\*HPMC\_Visc | 1 | 0,014 | 0,01% | 0,0088 | 0,0088 | 0,00 | 0,956 |
| HPMC\_HP\*HPMC\_HP | 1 | 0,063 | 0,05% | 0,9549 | 0,9549 | 0,34 | 0,568 |
| HPMC\_PS\*HPMC\_PS | 1 | 2,391 | 1,91% | 1,8039 | 1,8039 | 0,65 | 0,435 |
| 2-Way Interaction | 6 | 16,852 | 13,44% | 16,8520 | 2,8087 | 1,01 | 0,461 |
| Lac\*HPMC\_Visc | 1 | 1,476 | 1,18% | 0,2846 | 0,2846 | 0,10 | 0,754 |
| Lac\*HPMC\_HP | 1 | 8,683 | 6,93% | 6,5273 | 6,5273 | 2,36 | 0,151 |
| Lac\*HPMC\_PS | 1 | 1,657 | 1,32% | 1,6571 | 1,6571 | 0,60 | 0,454 |
| HPMC\_Visc\*HPMC\_HP | 1 | 0,363 | 0,29% | 0,9462 | 0,9462 | 0,34 | 0,570 |
| HPMC\_Visc\*HPMC\_PS | 1 | 0,368 | 0,29% | 0,0898 | 0,0898 | 0,03 | 0,860 |
| HPMC\_HP\*HPMC\_PS | 1 | 4,305 | 3,43% | 4,3047 | 4,3047 | 1,55 | 0,236 |
| Error | 12 | 33,248 | 26,52% | 33,2484 | 2,7707 |  |  |
| Lack-of-Fit | 10 | 19,350 | 15,43% | 19,3497 | 1,9350 | 0,28 | 0,933 |
| Pure Error | 2 | 13,899 | 11,09% | 13,8987 | 6,9493 |  |  |
| Total | 26 | 125,379 | 100,00% |  |  |  |  |

## Regression Equation in Uncoded Units

|  |  |  |
| --- | --- | --- |
| F\_SD\_11h(660min) | = | 280 - 228 Lac - 0,00361 HPMC\_Visc - 26,7 HPMC\_HP - 2,36 HPMC\_PS + 68,6 Lac\*Lac - 0,000000 HPMC\_Visc\*HPMC\_Visc - 0,84 HPMC\_HP\*HPMC\_HP - 0,0220 HPMC\_PS\*HPMC\_PS - 0,00053 Lac\*HPMC\_Visc + 10,29 Lac\*HPMC\_HP + 1,10 Lac\*HPMC\_PS + 0,000286 HPMC\_Visc\*HPMC\_HP + 0,000017 HPMC\_Visc\*HPMC\_PS + 0,504 HPMC\_HP\*HPMC\_PS |

## Fits and Diagnostics for All Observations

| Obs | F\_SD\_11h(660min) | Fit | SE Fit | 95% CI | Resid | Std Resid | Del Resid | HI |
| --- | --- | --- | --- | --- | --- | --- | --- | --- |
| 1 | 4,21 | 3,76 | 1,35 | (0,83; 6,70) | 0,45 | 0,46 | 0,44 | 0,655994 |
| 2 | 3,43 | 3,76 | 1,35 | (0,82; 6,70) | -0,33 | -0,34 | -0,33 | 0,655994 |
| 3 | 1,78 | 2,58 | 1,42 | (-0,52; 5,68) | -0,80 | -0,92 | -0,92 | 0,729945 |
| 4 | 0,71 | 1,09 | 1,42 | (-2,00; 4,19) | -0,38 | -0,44 | -0,42 | 0,729945 |
| 5 | 3,63 | 2,75 | 1,15 | (0,24; 5,25) | 0,88 | 0,73 | 0,72 | 0,476813 |
| 6 | 5,32 | 5,28 | 1,15 | (2,78; 7,79) | 0,03 | 0,03 | 0,02 | 0,476813 |
| 7 | 2,59 | 1,92 | 1,38 | (-1,09; 4,93) | 0,67 | 0,72 | 0,70 | 0,688750 |
| 8 | 3,30 | 4,01 | 1,38 | (1,00; 7,02) | -0,71 | -0,77 | -0,75 | 0,688750 |
| 9 | 1,67 | 1,69 | 1,27 | (-1,08; 4,45) | -0,02 | -0,02 | -0,02 | 0,580949 |
| 10 | 2,40 | 3,12 | 1,27 | (0,36; 5,89) | -0,73 | -0,68 | -0,66 | 0,580949 |
| 11 | 1,54 | 2,10 | 1,09 | (-0,26; 4,47) | -0,56 | -0,45 | -0,43 | 0,425781 |
| 12 | 1,90 | 3,20 | 1,09 | (0,83; 5,57) | -1,30 | -1,03 | -1,04 | 0,425781 |
| 13 | 3,31 | 2,91 | 1,26 | (0,18; 5,65) | 0,39 | 0,36 | 0,35 | 0,569258 |
| 14 | 7,76 | 7,41 | 1,26 | (4,68; 10,15) | 0,35 | 0,32 | 0,31 | 0,569258 |
| 15 | 2,02 | 3,01 | 1,10 | (0,63; 5,40) | -0,99 | -0,79 | -0,78 | 0,433227 |
| 16 | 4,81 | 5,87 | 1,10 | (3,48; 8,26) | -1,06 | -0,84 | -0,83 | 0,433227 |
| 17 | 4,71 | 5,23 | 1,29 | (2,43; 8,03) | -0,53 | -0,50 | -0,48 | 0,597775 |
| 18 | 10,58 | 9,03 | 1,29 | (6,22; 11,83) | 1,55 | 1,47 | 1,56 | 0,597775 |
| 19 | 1,72 | 3,24 | 1,06 | (0,93; 5,56) | -1,52 | -1,19 | -1,21 | 0,407038 |
| 20 | 3,37 | 1,65 | 1,34 | (-1,27; 4,57) | 1,73 | 1,75 | 1,94 | 0,647412 |
| 21 | 2,69 | 1,13 | 1,06 | (-1,18; 3,45) | 1,56 | 1,22 | 1,24 | 0,407933 |
| 22 | 3,23 | 3,57 | 1,50 | (0,29; 6,85) | -0,34 | -0,48 | -0,47 | 0,817044 |
| 23 | 0,82 | 1,07 | 1,51 | (-2,22; 4,36) | -0,25 | -0,36 | -0,35 | 0,823434 |
| 24 | 2,55 | 1,81 | 1,41 | (-1,25; 4,88) | 0,74 | 0,83 | 0,82 | 0,713775 |
| 25 | 1,38 | 2,84 | 0,89 | (0,89; 4,79) | -1,46 | -1,04 | -1,04 | 0,288793 |
| 26 | 6,25 | 2,84 | 0,89 | (0,89; 4,79) | 3,41 | 2,43 | 3,27 | 0,288793 |
| 27 | 2,07 | 2,84 | 0,89 | (0,89; 4,79) | -0,77 | -0,55 | -0,53 | 0,288793 |

| Obs | Cook’s D | DFITS |  |
| --- | --- | --- | --- |
| 1 | 0,03 | 0,61043 |  |
| 2 | 0,01 | -0,45414 |  |
| 3 | 0,15 | -1,50859 |  |
| 4 | 0,03 | -0,69694 |  |
| 5 | 0,03 | 0,68695 |  |
| 6 | 0,00 | 0,02288 |  |
| 7 | 0,08 | 1,04492 |  |
| 8 | 0,09 | -1,12001 |  |
| 9 | 0,00 | -0,01823 |  |
| 10 | 0,04 | -0,77631 |  |
| 11 | 0,01 | -0,37010 |  |
| 12 | 0,05 | -0,89157 |  |
| 13 | 0,01 | 0,39804 |  |
| 14 | 0,01 | 0,35603 |  |
| 15 | 0,03 | -0,67917 |  |
| 16 | 0,04 | -0,72930 |  |
| 17 | 0,02 | -0,58660 |  |
| 18 | 0,21 | 1,89584 |  |
| 19 | 0,06 | -1,00364 |  |
| 20 | 0,37 | 2,62218 |  |
| 21 | 0,07 | 1,03209 |  |
| 22 | 0,07 | -0,98938 |  |
| 23 | 0,04 | -0,75115 |  |
| 24 | 0,11 | 1,28757 |  |
| 25 | 0,03 | -0,66345 |  |
| 26 | 0,16 | 2,08226 | R |
| 27 | 0,01 | -0,34016 |  |

R  Large residual

## Coded Coefficients

| Term | Coef | SE Coef | 95% CI | T-Value | P-Value | VIF |
| --- | --- | --- | --- | --- | --- | --- |
| Constant | 2,880 | 0,858 | (1,010; 4,751) | 3,36 | 0,006 |  |
| Lac | 1,754 | 0,710 | (0,207; 3,300) | 2,47 | 0,029 | 1,18 |
| HPMC\_Visc | -0,644 | 0,742 | (-2,260; 0,972) | -0,87 | 0,402 | 1,70 |
| HPMC\_HP | 1,498 | 0,726 | (-0,083; 3,080) | 2,06 | 0,061 | 1,26 |
| HPMC\_PS | 1,08 | 1,11 | (-1,35; 3,50) | 0,97 | 0,352 | 2,09 |
| Lac\*Lac | 3,67 | 1,41 | (0,60; 6,74) | 2,60 | 0,023 | 1,30 |
| HPMC\_Visc\*HPMC\_Visc | -0,09 | 1,50 | (-3,36; 3,18) | -0,06 | 0,954 | 1,96 |
| HPMC\_HP\*HPMC\_HP | -0,79 | 1,42 | (-3,89; 2,31) | -0,56 | 0,588 | 1,81 |
| HPMC\_PS\*HPMC\_PS | -1,22 | 1,41 | (-4,30; 1,86) | -0,86 | 0,405 | 1,42 |
| Lac\*HPMC\_Visc | -0,42 | 1,56 | (-3,81; 2,97) | -0,27 | 0,790 | 1,49 |
| Lac\*HPMC\_HP | 2,49 | 1,63 | (-1,07; 6,05) | 1,52 | 0,153 | 1,17 |
| Lac\*HPMC\_PS | 2,27 | 2,52 | (-3,22; 7,76) | 0,90 | 0,386 | 1,42 |
| HPMC\_Visc\*HPMC\_HP | 1,29 | 1,86 | (-2,76; 5,33) | 0,69 | 0,501 | 2,73 |
| HPMC\_Visc\*HPMC\_PS | -0,12 | 2,62 | (-5,83; 5,60) | -0,04 | 0,965 | 2,74 |
| HPMC\_HP\*HPMC\_PS | 4,29 | 2,90 | (-2,02; 10,59) | 1,48 | 0,165 | 2,70 |

## Model Summary

| S | R-sq | R-sq(adj) | PRESS | R-sq(pred) | AICc | BIC |
| --- | --- | --- | --- | --- | --- | --- |
| 1,59824 | 72,50% | 40,42% | 120,274 | 0,00% | 166,45 | 132,78 |

## Analysis of Variance

| Source | DF | Seq SS | Contribution | Adj SS | Adj MS | F-Value | P-Value |
| --- | --- | --- | --- | --- | --- | --- | --- |
| Model | 14 | 80,812 | 72,50% | 80,8119 | 5,7723 | 2,26 | 0,082 |
| Linear | 4 | 32,193 | 28,88% | 36,3246 | 9,0812 | 3,56 | 0,039 |
| Lac | 1 | 12,808 | 11,49% | 15,5908 | 15,5908 | 6,10 | 0,029 |
| HPMC\_Visc | 1 | 6,615 | 5,93% | 1,9274 | 1,9274 | 0,75 | 0,402 |
| HPMC\_HP | 1 | 11,991 | 10,76% | 10,8916 | 10,8916 | 4,26 | 0,061 |
| HPMC\_PS | 1 | 0,779 | 0,70% | 2,3947 | 2,3947 | 0,94 | 0,352 |
| Square | 4 | 30,571 | 27,43% | 30,9634 | 7,7408 | 3,03 | 0,061 |
| Lac\*Lac | 1 | 28,501 | 25,57% | 17,2844 | 17,2844 | 6,77 | 0,023 |
| HPMC\_Visc\*HPMC\_Visc | 1 | 0,189 | 0,17% | 0,0090 | 0,0090 | 0,00 | 0,954 |
| HPMC\_HP\*HPMC\_HP | 1 | 0,014 | 0,01% | 0,7913 | 0,7913 | 0,31 | 0,588 |
| HPMC\_PS\*HPMC\_PS | 1 | 1,867 | 1,67% | 1,9025 | 1,9025 | 0,74 | 0,405 |
| 2-Way Interaction | 6 | 18,047 | 16,19% | 18,0471 | 3,0078 | 1,18 | 0,380 |
| Lac\*HPMC\_Visc | 1 | 1,436 | 1,29% | 0,1896 | 0,1896 | 0,07 | 0,790 |
| Lac\*HPMC\_HP | 1 | 8,192 | 7,35% | 5,9377 | 5,9377 | 2,32 | 0,153 |
| Lac\*HPMC\_PS | 1 | 2,069 | 1,86% | 2,0686 | 2,0686 | 0,81 | 0,386 |
| HPMC\_Visc\*HPMC\_HP | 1 | 0,682 | 0,61% | 1,2281 | 1,2281 | 0,48 | 0,501 |
| HPMC\_Visc\*HPMC\_PS | 1 | 0,075 | 0,07% | 0,0050 | 0,0050 | 0,00 | 0,965 |
| HPMC\_HP\*HPMC\_PS | 1 | 5,594 | 5,02% | 5,5943 | 5,5943 | 2,19 | 0,165 |
| Error | 12 | 30,653 | 27,50% | 30,6526 | 2,5544 |  |  |
| Lack-of-Fit | 10 | 16,476 | 14,78% | 16,4757 | 1,6476 | 0,23 | 0,955 |
| Pure Error | 2 | 14,177 | 12,72% | 14,1768 | 7,0884 |  |  |
| Total | 26 | 111,464 | 100,00% |  |  |  |  |

## Regression Equation in Uncoded Units

|  |  |  |
| --- | --- | --- |
| F\_SD\_12h(720min) | = | 316 - 225 Lac - 0,00260 HPMC\_Visc - 33,3 HPMC\_HP - 2,71 HPMC\_PS + 58,7 Lac\*Lac - 0,000000 HPMC\_Visc\*HPMC\_Visc - 0,77 HPMC\_HP\*HPMC\_HP - 0,0226 HPMC\_PS\*HPMC\_PS - 0,00044 Lac\*HPMC\_Visc + 9,81 Lac\*HPMC\_HP + 1,23 Lac\*HPMC\_PS + 0,000326 HPMC\_Visc\*HPMC\_HP - 0,000004 HPMC\_Visc\*HPMC\_PS + 0,574 HPMC\_HP\*HPMC\_PS |

## Fits and Diagnostics for All Observations

| Obs | F\_SD\_12h(720min) | Fit | SE Fit | 95% CI | Resid | Std Resid | Del Resid | HI |
| --- | --- | --- | --- | --- | --- | --- | --- | --- |
| 1 | 4,31 | 3,93 | 1,29 | (1,11; 6,75) | 0,38 | 0,41 | 0,39 | 0,655994 |
| 2 | 3,41 | 3,63 | 1,29 | (0,81; 6,45) | -0,22 | -0,23 | -0,23 | 0,655994 |
| 3 | 1,83 | 2,81 | 1,37 | (-0,17; 5,78) | -0,97 | -1,17 | -1,19 | 0,729945 |
| 4 | 0,83 | 1,09 | 1,37 | (-1,89; 4,06) | -0,25 | -0,30 | -0,29 | 0,729945 |
| 5 | 3,62 | 2,82 | 1,10 | (0,42; 5,23) | 0,79 | 0,69 | 0,67 | 0,476813 |
| 6 | 5,24 | 4,97 | 1,10 | (2,57; 7,38) | 0,27 | 0,23 | 0,22 | 0,476813 |
| 7 | 2,46 | 2,02 | 1,33 | (-0,87; 4,91) | 0,44 | 0,50 | 0,48 | 0,688750 |
| 8 | 3,33 | 3,77 | 1,33 | (0,88; 6,66) | -0,44 | -0,50 | -0,48 | 0,688750 |
| 9 | 1,77 | 1,84 | 1,22 | (-0,82; 4,49) | -0,07 | -0,07 | -0,07 | 0,580949 |
| 10 | 2,62 | 3,13 | 1,22 | (0,48; 5,78) | -0,51 | -0,49 | -0,47 | 0,580949 |
| 11 | 1,40 | 1,92 | 1,04 | (-0,35; 4,19) | -0,52 | -0,43 | -0,41 | 0,425781 |
| 12 | 1,78 | 2,88 | 1,04 | (0,61; 5,16) | -1,10 | -0,91 | -0,90 | 0,425781 |
| 13 | 3,63 | 3,38 | 1,21 | (0,75; 6,00) | 0,25 | 0,24 | 0,23 | 0,569258 |
| 14 | 8,41 | 7,62 | 1,21 | (5,00; 10,25) | 0,79 | 0,75 | 0,74 | 0,569258 |
| 15 | 2,14 | 2,96 | 1,05 | (0,67; 5,25) | -0,82 | -0,68 | -0,66 | 0,433227 |
| 16 | 4,55 | 5,63 | 1,05 | (3,34; 7,92) | -1,08 | -0,89 | -0,89 | 0,433227 |
| 17 | 4,82 | 4,95 | 1,24 | (2,25; 7,64) | -0,13 | -0,13 | -0,12 | 0,597775 |
| 18 | 9,08 | 8,19 | 1,24 | (5,49; 10,88) | 0,89 | 0,88 | 0,87 | 0,597775 |
| 19 | 1,70 | 3,49 | 1,02 | (1,26; 5,71) | -1,79 | -1,45 | -1,53 | 0,407038 |
| 20 | 3,27 | 1,59 | 1,29 | (-1,21; 4,39) | 1,68 | 1,77 | 1,98 | 0,647412 |
| 21 | 2,74 | 1,26 | 1,02 | (-0,97; 3,48) | 1,49 | 1,21 | 1,24 | 0,407933 |
| 22 | 3,33 | 3,80 | 1,44 | (0,65; 6,95) | -0,47 | -0,68 | -0,67 | 0,817044 |
| 23 | 0,77 | 0,94 | 1,45 | (-2,22; 4,10) | -0,17 | -0,25 | -0,24 | 0,823434 |
| 24 | 2,51 | 1,98 | 1,35 | (-0,97; 4,92) | 0,53 | 0,62 | 0,60 | 0,713775 |
| 25 | 1,44 | 2,90 | 0,86 | (1,02; 4,77) | -1,46 | -1,08 | -1,09 | 0,288793 |
| 26 | 6,29 | 2,90 | 0,86 | (1,02; 4,77) | 3,40 | 2,52 | 3,52 | 0,288793 |
| 27 | 1,97 | 2,90 | 0,86 | (1,02; 4,77) | -0,93 | -0,69 | -0,67 | 0,288793 |

| Obs | Cook’s D | DFITS |  |
| --- | --- | --- | --- |
| 1 | 0,02 | 0,53972 |  |
| 2 | 0,01 | -0,31072 |  |
| 3 | 0,25 | -1,96392 |  |
| 4 | 0,02 | -0,47922 |  |
| 5 | 0,03 | 0,64108 |  |
| 6 | 0,00 | 0,21157 |  |
| 7 | 0,04 | 0,71451 |  |
| 8 | 0,04 | -0,71569 |  |
| 9 | 0,00 | -0,07686 |  |
| 10 | 0,02 | -0,55784 |  |
| 11 | 0,01 | -0,35343 |  |
| 12 | 0,04 | -0,77783 |  |
| 13 | 0,01 | 0,26751 |  |
| 14 | 0,05 | 0,84749 |  |
| 15 | 0,02 | -0,58128 |  |
| 16 | 0,04 | -0,77428 |  |
| 17 | 0,00 | -0,14686 |  |
| 18 | 0,08 | 1,06017 |  |
| 19 | 0,10 | -1,26848 |  |
| 20 | 0,39 | 2,68020 |  |
| 21 | 0,07 | 1,02532 |  |
| 22 | 0,14 | -1,40607 |  |
| 23 | 0,02 | -0,52788 |  |
| 24 | 0,06 | 0,95286 |  |
| 25 | 0,03 | -0,69404 |  |
| 26 | 0,17 | 2,24051 | R |
| 27 | 0,01 | -0,42840 |  |

R  Large residual

## Coded Coefficients

| Term | Coef | SE Coef | 95% CI | T-Value | P-Value | VIF |
| --- | --- | --- | --- | --- | --- | --- |
| Constant | 2,825 | 0,776 | (1,135; 4,515) | 3,64 | 0,003 |  |
| Lac | 1,442 | 0,641 | (0,044; 2,839) | 2,25 | 0,044 | 1,18 |
| HPMC\_Visc | -0,719 | 0,670 | (-2,179; 0,741) | -1,07 | 0,304 | 1,70 |
| HPMC\_HP | 1,416 | 0,656 | (-0,012; 2,845) | 2,16 | 0,052 | 1,26 |
| HPMC\_PS | 0,90 | 1,01 | (-1,29; 3,09) | 0,90 | 0,387 | 2,09 |
| Lac\*Lac | 3,08 | 1,27 | (0,31; 5,86) | 2,42 | 0,032 | 1,30 |
| HPMC\_Visc\*HPMC\_Visc | -0,08 | 1,35 | (-3,03; 2,87) | -0,06 | 0,954 | 1,96 |
| HPMC\_HP\*HPMC\_HP | -0,62 | 1,29 | (-3,42; 2,18) | -0,48 | 0,640 | 1,81 |
| HPMC\_PS\*HPMC\_PS | -1,12 | 1,28 | (-3,90; 1,66) | -0,88 | 0,398 | 1,42 |
| Lac\*HPMC\_Visc | -0,17 | 1,41 | (-3,23; 2,89) | -0,12 | 0,905 | 1,49 |
| Lac\*HPMC\_HP | 1,99 | 1,48 | (-1,23; 5,20) | 1,35 | 0,203 | 1,17 |
| Lac\*HPMC\_PS | 2,10 | 2,28 | (-2,86; 7,07) | 0,92 | 0,374 | 1,42 |
| HPMC\_Visc\*HPMC\_HP | 1,16 | 1,68 | (-2,49; 4,82) | 0,69 | 0,501 | 2,73 |
| HPMC\_Visc\*HPMC\_PS | -0,52 | 2,37 | (-5,68; 4,65) | -0,22 | 0,831 | 2,74 |
| HPMC\_HP\*HPMC\_PS | 3,85 | 2,62 | (-1,85; 9,55) | 1,47 | 0,167 | 2,70 |

## Model Summary

| S | R-sq | R-sq(adj) | PRESS | R-sq(pred) | AICc | BIC |
| --- | --- | --- | --- | --- | --- | --- |
| 1,44396 | 70,92% | 37,00% | 92,4865 | 0,00% | 160,97 | 127,30 |

## Analysis of Variance

| Source | DF | Seq SS | Contribution | Adj SS | Adj MS | F-Value | P-Value |
| --- | --- | --- | --- | --- | --- | --- | --- |
| Model | 14 | 61,0311 | 70,92% | 61,0311 | 4,3594 | 2,09 | 0,104 |
| Linear | 4 | 25,8739 | 30,07% | 29,5968 | 7,3992 | 3,55 | 0,039 |
| Lac | 1 | 8,3917 | 9,75% | 10,5381 | 10,5381 | 5,05 | 0,044 |
| HPMC\_Visc | 1 | 6,4681 | 7,52% | 2,3997 | 2,3997 | 1,15 | 0,304 |
| HPMC\_HP | 1 | 10,3575 | 12,04% | 9,7276 | 9,7276 | 4,67 | 0,052 |
| HPMC\_PS | 1 | 0,6566 | 0,76% | 1,6768 | 1,6768 | 0,80 | 0,387 |
| Square | 4 | 22,0010 | 25,57% | 22,2250 | 5,5563 | 2,66 | 0,084 |
| Lac\*Lac | 1 | 20,2842 | 23,57% | 12,2052 | 12,2052 | 5,85 | 0,032 |
| HPMC\_Visc\*HPMC\_Visc | 1 | 0,3397 | 0,39% | 0,0074 | 0,0074 | 0,00 | 0,954 |
| HPMC\_HP\*HPMC\_HP | 1 | 0,1176 | 0,14% | 0,4797 | 0,4797 | 0,23 | 0,640 |
| HPMC\_PS\*HPMC\_PS | 1 | 1,2595 | 1,46% | 1,6040 | 1,6040 | 0,77 | 0,398 |
| 2-Way Interaction | 6 | 13,1563 | 15,29% | 13,1563 | 2,1927 | 1,05 | 0,441 |
| Lac\*HPMC\_Visc | 1 | 0,7504 | 0,87% | 0,0311 | 0,0311 | 0,01 | 0,905 |
| Lac\*HPMC\_HP | 1 | 5,4340 | 6,31% | 3,7861 | 3,7861 | 1,82 | 0,203 |
| Lac\*HPMC\_PS | 1 | 1,7803 | 2,07% | 1,7803 | 1,7803 | 0,85 | 0,374 |
| HPMC\_Visc\*HPMC\_HP | 1 | 0,6697 | 0,78% | 1,0023 | 1,0023 | 0,48 | 0,501 |
| HPMC\_Visc\*HPMC\_PS | 1 | 0,0000 | 0,00% | 0,0987 | 0,0987 | 0,05 | 0,831 |
| HPMC\_HP\*HPMC\_PS | 1 | 4,5218 | 5,25% | 4,5218 | 4,5218 | 2,17 | 0,167 |
| Error | 12 | 25,0202 | 29,08% | 25,0202 | 2,0850 |  |  |
| Lack-of-Fit | 10 | 12,5249 | 14,56% | 12,5249 | 1,2525 | 0,20 | 0,969 |
| Pure Error | 2 | 12,4953 | 14,52% | 12,4953 | 6,2477 |  |  |
| Total | 26 | 86,0513 | 100,00% |  |  |  |  |

## Regression Equation in Uncoded Units

|  |  |  |
| --- | --- | --- |
| F\_SD\_13h(780min) | = | 275 - 195 Lac - 0,00149 HPMC\_Visc - 31,1 HPMC\_HP - 2,21 HPMC\_PS + 49,3 Lac\*Lac - 0,000000 HPMC\_Visc\*HPMC\_Visc - 0,60 HPMC\_HP\*HPMC\_HP - 0,0207 HPMC\_PS\*HPMC\_PS - 0,00018 Lac\*HPMC\_Visc + 7,84 Lac\*HPMC\_HP + 1,14 Lac\*HPMC\_PS + 0,000295 HPMC\_Visc\*HPMC\_HP - 0,000018 HPMC\_Visc\*HPMC\_PS + 0,516 HPMC\_HP\*HPMC\_PS |

## Fits and Diagnostics for All Observations

| Obs | F\_SD\_13h(780min) | Fit | SE Fit | 95% CI | Resid | Std Resid | Del Resid | HI |
| --- | --- | --- | --- | --- | --- | --- | --- | --- |
| 1 | 4,15 | 3,82 | 1,17 | (1,27; 6,37) | 0,33 | 0,39 | 0,38 | 0,655994 |
| 2 | 3,28 | 3,43 | 1,17 | (0,88; 5,98) | -0,15 | -0,18 | -0,17 | 0,655994 |
| 3 | 1,83 | 2,72 | 1,23 | (0,04; 5,41) | -0,90 | -1,19 | -1,22 | 0,729945 |
| 4 | 1,21 | 1,34 | 1,23 | (-1,35; 4,02) | -0,12 | -0,16 | -0,16 | 0,729945 |
| 5 | 3,58 | 2,96 | 1,00 | (0,78; 5,13) | 0,62 | 0,60 | 0,58 | 0,476813 |
| 6 | 5,12 | 4,56 | 1,00 | (2,38; 6,73) | 0,56 | 0,54 | 0,52 | 0,476813 |
| 7 | 2,21 | 2,16 | 1,20 | (-0,45; 4,77) | 0,05 | 0,07 | 0,06 | 0,688750 |
| 8 | 3,51 | 3,60 | 1,20 | (0,99; 6,21) | -0,09 | -0,12 | -0,11 | 0,688750 |
| 9 | 1,74 | 2,06 | 1,10 | (-0,34; 4,45) | -0,32 | -0,34 | -0,32 | 0,580949 |
| 10 | 3,12 | 3,14 | 1,10 | (0,74; 5,54) | -0,01 | -0,02 | -0,01 | 0,580949 |
| 11 | 1,38 | 1,82 | 0,94 | (-0,24; 3,87) | -0,43 | -0,39 | -0,38 | 0,425781 |
| 12 | 1,67 | 2,72 | 0,94 | (0,67; 4,78) | -1,06 | -0,96 | -0,96 | 0,425781 |
| 13 | 3,80 | 3,64 | 1,09 | (1,27; 6,01) | 0,16 | 0,17 | 0,16 | 0,569258 |
| 14 | 7,90 | 7,11 | 1,09 | (4,73; 9,48) | 0,79 | 0,83 | 0,82 | 0,569258 |
| 15 | 2,19 | 2,82 | 0,95 | (0,75; 4,89) | -0,63 | -0,58 | -0,56 | 0,433227 |
| 16 | 4,24 | 5,12 | 0,95 | (3,05; 7,19) | -0,87 | -0,80 | -0,79 | 0,433227 |
| 17 | 4,95 | 4,65 | 1,12 | (2,22; 7,08) | 0,29 | 0,32 | 0,31 | 0,597775 |
| 18 | 7,46 | 7,24 | 1,12 | (4,81; 9,67) | 0,22 | 0,24 | 0,23 | 0,597775 |
| 19 | 1,74 | 3,54 | 0,92 | (1,53; 5,54) | -1,80 | -1,61 | -1,75 | 0,407038 |
| 20 | 3,05 | 1,58 | 1,16 | (-0,95; 4,11) | 1,47 | 1,72 | 1,89 | 0,647412 |
| 21 | 2,63 | 1,42 | 0,92 | (-0,59; 3,43) | 1,22 | 1,10 | 1,11 | 0,407933 |
| 22 | 3,38 | 3,83 | 1,31 | (0,99; 6,67) | -0,45 | -0,73 | -0,71 | 0,817044 |
| 23 | 0,89 | 1,05 | 1,31 | (-1,81; 3,90) | -0,16 | -0,27 | -0,26 | 0,823434 |
| 24 | 2,47 | 2,06 | 1,22 | (-0,60; 4,72) | 0,41 | 0,53 | 0,51 | 0,713775 |
| 25 | 1,54 | 2,86 | 0,78 | (1,17; 4,55) | -1,33 | -1,09 | -1,10 | 0,288793 |
| 26 | 6,03 | 2,86 | 0,78 | (1,17; 4,55) | 3,16 | 2,60 | 3,76 | 0,288793 |
| 27 | 1,88 | 2,86 | 0,78 | (1,17; 4,55) | -0,98 | -0,81 | -0,80 | 0,288793 |

| Obs | Cook’s D | DFITS |  |
| --- | --- | --- | --- |
| 1 | 0,02 | 0,52169 |  |
| 2 | 0,00 | -0,23686 |  |
| 3 | 0,26 | -2,00387 |  |
| 4 | 0,00 | -0,25781 |  |
| 5 | 0,02 | 0,55463 |  |
| 6 | 0,02 | 0,49805 |  |
| 7 | 0,00 | 0,09490 |  |
| 8 | 0,00 | -0,16394 |  |
| 9 | 0,01 | -0,38171 |  |
| 10 | 0,00 | -0,01758 |  |
| 11 | 0,01 | -0,32760 |  |
| 12 | 0,05 | -0,82796 |  |
| 13 | 0,00 | 0,18448 |  |
| 14 | 0,06 | 0,94691 |  |
| 15 | 0,02 | -0,48833 |  |
| 16 | 0,03 | -0,69268 |  |
| 17 | 0,01 | 0,37482 |  |
| 18 | 0,01 | 0,28346 |  |
| 19 | 0,12 | -1,44751 |  |
| 20 | 0,36 | 2,56408 |  |
| 21 | 0,06 | 0,91829 |  |
| 22 | 0,16 | -1,50977 |  |
| 23 | 0,02 | -0,55807 |  |
| 24 | 0,05 | 0,81230 |  |
| 25 | 0,03 | -0,70011 |  |
| 26 | 0,18 | 2,39653 | R |
| 27 | 0,02 | -0,50707 |  |

R  Large residual

## Coded Coefficients

| Term | Coef | SE Coef | 95% CI | T-Value | P-Value | VIF |
| --- | --- | --- | --- | --- | --- | --- |
| Constant | 2,752 | 0,716 | (1,192; 4,312) | 3,84 | 0,002 |  |
| Lac | 1,163 | 0,592 | (-0,127; 2,453) | 1,96 | 0,073 | 1,18 |
| HPMC\_Visc | -0,643 | 0,619 | (-1,991; 0,705) | -1,04 | 0,319 | 1,70 |
| HPMC\_HP | 1,391 | 0,605 | (0,072; 2,709) | 2,30 | 0,040 | 1,26 |
| HPMC\_PS | 0,418 | 0,928 | (-1,605; 2,441) | 0,45 | 0,661 | 2,09 |
| Lac\*Lac | 2,69 | 1,18 | (0,12; 5,25) | 2,28 | 0,041 | 1,30 |
| HPMC\_Visc\*HPMC\_Visc | -0,09 | 1,25 | (-2,81; 2,64) | -0,07 | 0,947 | 1,96 |
| HPMC\_HP\*HPMC\_HP | -0,35 | 1,19 | (-2,93; 2,24) | -0,29 | 0,776 | 1,81 |
| HPMC\_PS\*HPMC\_PS | -1,01 | 1,18 | (-3,58; 1,56) | -0,85 | 0,410 | 1,42 |
| Lac\*HPMC\_Visc | 0,20 | 1,30 | (-2,63; 3,03) | 0,15 | 0,880 | 1,49 |
| Lac\*HPMC\_HP | 1,37 | 1,36 | (-1,59; 4,34) | 1,01 | 0,333 | 1,17 |
| Lac\*HPMC\_PS | 1,24 | 2,10 | (-3,34; 5,82) | 0,59 | 0,567 | 1,42 |
| HPMC\_Visc\*HPMC\_HP | 1,19 | 1,55 | (-2,18; 4,57) | 0,77 | 0,456 | 2,73 |
| HPMC\_Visc\*HPMC\_PS | -1,09 | 2,19 | (-5,85; 3,68) | -0,50 | 0,628 | 2,74 |
| HPMC\_HP\*HPMC\_PS | 2,97 | 2,42 | (-2,29; 8,23) | 1,23 | 0,242 | 2,70 |

## Model Summary

| S | R-sq | R-sq(adj) | PRESS | R-sq(pred) | AICc | BIC |
| --- | --- | --- | --- | --- | --- | --- |
| 1,33303 | 65,98% | 26,29% | 79,9845 | 0,00% | 156,65 | 122,98 |

## Analysis of Variance

| Source | DF | Seq SS | Contribution | Adj SS | Adj MS | F-Value | P-Value |
| --- | --- | --- | --- | --- | --- | --- | --- |
| Model | 14 | 41,3567 | 65,98% | 41,3567 | 2,95405 | 1,66 | 0,192 |
| Linear | 4 | 18,2697 | 29,15% | 21,1428 | 5,28571 | 2,97 | 0,064 |
| Lac | 1 | 5,3330 | 8,51% | 6,8596 | 6,85955 | 3,86 | 0,073 |
| HPMC\_Visc | 1 | 3,7346 | 5,96% | 1,9173 | 1,91734 | 1,08 | 0,319 |
| HPMC\_HP | 1 | 9,0887 | 14,50% | 9,3804 | 9,38040 | 5,28 | 0,040 |
| HPMC\_PS | 1 | 0,1133 | 0,18% | 0,3604 | 0,36035 | 0,20 | 0,661 |
| Square | 4 | 16,9432 | 27,03% | 16,5403 | 4,13508 | 2,33 | 0,116 |
| Lac\*Lac | 1 | 15,1437 | 24,16% | 9,2756 | 9,27556 | 5,22 | 0,041 |
| HPMC\_Visc\*HPMC\_Visc | 1 | 0,5878 | 0,94% | 0,0083 | 0,00830 | 0,00 | 0,947 |
| HPMC\_HP\*HPMC\_HP | 1 | 0,4589 | 0,73% | 0,1510 | 0,15096 | 0,08 | 0,776 |
| HPMC\_PS\*HPMC\_PS | 1 | 0,7528 | 1,20% | 1,2942 | 1,29419 | 0,73 | 0,410 |
| 2-Way Interaction | 6 | 6,1438 | 9,80% | 6,1438 | 1,02396 | 0,58 | 0,743 |
| Lac\*HPMC\_Visc | 1 | 0,0174 | 0,03% | 0,0422 | 0,04216 | 0,02 | 0,880 |
| Lac\*HPMC\_HP | 1 | 2,4896 | 3,97% | 1,8102 | 1,81016 | 1,02 | 0,333 |
| Lac\*HPMC\_PS | 1 | 0,6147 | 0,98% | 0,6147 | 0,61474 | 0,35 | 0,567 |
| HPMC\_Visc\*HPMC\_HP | 1 | 0,1460 | 0,23% | 1,0565 | 1,05646 | 0,59 | 0,456 |
| HPMC\_Visc\*HPMC\_PS | 1 | 0,1843 | 0,29% | 0,4384 | 0,43838 | 0,25 | 0,628 |
| HPMC\_HP\*HPMC\_PS | 1 | 2,6919 | 4,29% | 2,6919 | 2,69190 | 1,51 | 0,242 |
| Error | 12 | 21,3237 | 34,02% | 21,3237 | 1,77698 |  |  |
| Lack-of-Fit | 10 | 10,6240 | 16,95% | 10,6240 | 1,06240 | 0,20 | 0,969 |
| Pure Error | 2 | 10,6997 | 17,07% | 10,6997 | 5,34986 |  |  |
| Total | 26 | 62,6804 | 100,00% |  |  |  |  |

## Regression Equation in Uncoded Units

|  |  |  |
| --- | --- | --- |
| F\_SD\_14h(840min) | = | 191 - 139,3 Lac - 0,00034 HPMC\_Visc - 26,8 HPMC\_HP - 0,95 HPMC\_PS + 43,0 Lac\*Lac - 0,000000 HPMC\_Visc\*HPMC\_Visc - 0,34 HPMC\_HP\*HPMC\_HP - 0,0186 HPMC\_PS\*HPMC\_PS + 0,00021 Lac\*HPMC\_Visc + 5,42 Lac\*HPMC\_HP + 0,67 Lac\*HPMC\_PS + 0,000303 HPMC\_Visc\*HPMC\_HP - 0,000038 HPMC\_Visc\*HPMC\_PS + 0,398 HPMC\_HP\*HPMC\_PS |

## Fits and Diagnostics for All Observations

| Obs | F\_SD\_14h(840min) | Fit | SE Fit | 95% CI | Resid | Std Resid | Del Resid | HI |
| --- | --- | --- | --- | --- | --- | --- | --- | --- |
| 1 | 3,876 | 3,581 | 1,080 | (1,229; 5,933) | 0,295 | 0,38 | 0,36 | 0,655994 |
| 2 | 3,215 | 3,305 | 1,080 | (0,952; 5,657) | -0,089 | -0,11 | -0,11 | 0,655994 |
| 3 | 1,802 | 2,452 | 1,139 | (-0,029; 4,933) | -0,650 | -0,94 | -0,93 | 0,729945 |
| 4 | 1,979 | 2,004 | 1,139 | (-0,477; 4,486) | -0,025 | -0,04 | -0,03 | 0,729945 |
| 5 | 3,594 | 3,038 | 0,920 | (1,032; 5,043) | 0,556 | 0,58 | 0,56 | 0,476813 |
| 6 | 5,076 | 4,141 | 0,920 | (2,135; 6,147) | 0,935 | 0,97 | 0,97 | 0,476813 |
| 7 | 2,329 | 2,607 | 1,106 | (0,197; 5,017) | -0,278 | -0,37 | -0,36 | 0,688750 |
| 8 | 4,407 | 4,083 | 1,106 | (1,673; 6,493) | 0,324 | 0,44 | 0,42 | 0,688750 |
| 9 | 1,770 | 2,457 | 1,016 | (0,243; 4,671) | -0,687 | -0,80 | -0,78 | 0,580949 |
| 10 | 3,457 | 3,060 | 1,016 | (0,846; 5,273) | 0,397 | 0,46 | 0,44 | 0,580949 |
| 11 | 1,345 | 1,748 | 0,870 | (-0,147; 3,643) | -0,403 | -0,40 | -0,38 | 0,425781 |
| 12 | 1,503 | 2,556 | 0,870 | (0,660; 4,451) | -1,052 | -1,04 | -1,05 | 0,425781 |
| 13 | 3,929 | 3,860 | 1,006 | (1,668; 6,051) | 0,070 | 0,08 | 0,08 | 0,569258 |
| 14 | 6,612 | 6,087 | 1,006 | (3,896; 8,278) | 0,525 | 0,60 | 0,58 | 0,569258 |
| 15 | 2,288 | 2,725 | 0,877 | (0,813; 4,637) | -0,438 | -0,44 | -0,42 | 0,433227 |
| 16 | 3,886 | 4,512 | 0,877 | (2,600; 6,424) | -0,625 | -0,62 | -0,61 | 0,433227 |
| 17 | 5,093 | 4,469 | 1,031 | (2,223; 6,714) | 0,624 | 0,74 | 0,72 | 0,597775 |
| 18 | 6,147 | 6,485 | 1,031 | (4,240; 8,731) | -0,338 | -0,40 | -0,39 | 0,597775 |
| 19 | 1,792 | 3,404 | 0,850 | (1,551; 5,257) | -1,612 | -1,57 | -1,69 | 0,407038 |
| 20 | 2,800 | 1,714 | 1,073 | (-0,623; 4,051) | 1,086 | 1,37 | 1,43 | 0,647412 |
| 21 | 2,589 | 1,581 | 0,851 | (-0,274; 3,436) | 1,008 | 0,98 | 0,98 | 0,407933 |
| 22 | 3,471 | 3,920 | 1,205 | (1,295; 6,545) | -0,449 | -0,79 | -0,77 | 0,817044 |
| 23 | 1,031 | 1,344 | 1,210 | (-1,291; 3,980) | -0,314 | -0,56 | -0,54 | 0,823434 |
| 24 | 2,404 | 1,917 | 1,126 | (-0,537; 4,371) | 0,487 | 0,68 | 0,67 | 0,713775 |
| 25 | 1,549 | 2,788 | 0,716 | (1,227; 4,349) | -1,240 | -1,10 | -1,11 | 0,288793 |
| 26 | 5,673 | 2,788 | 0,716 | (1,227; 4,349) | 2,884 | 2,57 | 3,66 | 0,288793 |
| 27 | 1,796 | 2,788 | 0,716 | (1,227; 4,349) | -0,993 | -0,88 | -0,87 | 0,288793 |

| Obs | Cook’s D | DFITS |  |
| --- | --- | --- | --- |
| 1 | 0,02 | 0,50152 |  |
| 2 | 0,00 | -0,15129 |  |
| 3 | 0,16 | -1,53490 |  |
| 4 | 0,00 | -0,05739 |  |
| 5 | 0,02 | 0,53452 |  |
| 6 | 0,06 | 0,92374 |  |
| 7 | 0,02 | -0,53471 |  |
| 8 | 0,03 | 0,62574 |  |
| 9 | 0,06 | -0,92193 |  |
| 10 | 0,02 | 0,52353 |  |
| 11 | 0,01 | -0,33136 |  |
| 12 | 0,05 | -0,90072 |  |
| 13 | 0,00 | 0,08767 |  |
| 14 | 0,03 | 0,67083 |  |
| 15 | 0,01 | -0,36787 |  |
| 16 | 0,02 | -0,53037 |  |
| 17 | 0,05 | 0,88215 |  |
| 18 | 0,02 | -0,46958 |  |
| 19 | 0,11 | -1,39704 |  |
| 20 | 0,23 | 1,93884 |  |
| 21 | 0,04 | 0,81448 |  |
| 22 | 0,18 | -1,63717 |  |
| 23 | 0,10 | -1,17314 |  |
| 24 | 0,08 | 1,05363 |  |
| 25 | 0,03 | -0,70963 |  |
| 26 | 0,18 | 2,32989 | R |
| 27 | 0,02 | -0,55708 |  |

R  Large residual

## Coded Coefficients

| Term | Coef | SE Coef | 95% CI | T-Value | P-Value | VIF |
| --- | --- | --- | --- | --- | --- | --- |
| Constant | 2,600 | 0,711 | (1,051; 4,148) | 3,66 | 0,003 |  |
| Lac | 0,883 | 0,588 | (-0,397; 2,164) | 1,50 | 0,159 | 1,18 |
| HPMC\_Visc | -0,438 | 0,614 | (-1,776; 0,900) | -0,71 | 0,490 | 1,70 |
| HPMC\_HP | 1,382 | 0,601 | (0,073; 2,691) | 2,30 | 0,040 | 1,26 |
| HPMC\_PS | 0,028 | 0,921 | (-1,979; 2,036) | 0,03 | 0,976 | 2,09 |
| Lac\*Lac | 2,39 | 1,17 | (-0,16; 4,93) | 2,04 | 0,064 | 1,30 |
| HPMC\_Visc\*HPMC\_Visc | 0,09 | 1,24 | (-2,62; 2,80) | 0,07 | 0,943 | 1,96 |
| HPMC\_HP\*HPMC\_HP | 0,06 | 1,18 | (-2,51; 2,63) | 0,05 | 0,959 | 1,81 |
| HPMC\_PS\*HPMC\_PS | -0,82 | 1,17 | (-3,37; 1,73) | -0,70 | 0,499 | 1,42 |
| Lac\*HPMC\_Visc | 0,94 | 1,29 | (-1,87; 3,75) | 0,73 | 0,480 | 1,49 |
| Lac\*HPMC\_HP | 0,87 | 1,35 | (-2,07; 3,82) | 0,65 | 0,530 | 1,17 |
| Lac\*HPMC\_PS | 0,73 | 2,09 | (-3,82; 5,27) | 0,35 | 0,734 | 1,42 |
| HPMC\_Visc\*HPMC\_HP | 1,58 | 1,54 | (-1,77; 4,93) | 1,03 | 0,324 | 2,73 |
| HPMC\_Visc\*HPMC\_PS | -1,24 | 2,17 | (-5,97; 3,49) | -0,57 | 0,579 | 2,74 |
| HPMC\_HP\*HPMC\_PS | 2,83 | 2,40 | (-2,39; 8,05) | 1,18 | 0,261 | 2,70 |

## Model Summary

| S | R-sq | R-sq(adj) | PRESS | R-sq(pred) | AICc | BIC |
| --- | --- | --- | --- | --- | --- | --- |
| 1,32327 | 58,37% | 9,81% | 92,5468 | 0,00% | 156,25 | 122,59 |

## Analysis of Variance

| Source | DF | Seq SS | Contribution | Adj SS | Adj MS | F-Value | P-Value |
| --- | --- | --- | --- | --- | --- | --- | --- |
| Model | 14 | 29,4671 | 58,37% | 29,4671 | 2,10479 | 1,20 | 0,379 |
| Linear | 4 | 10,6910 | 21,18% | 14,8175 | 3,70437 | 2,12 | 0,142 |
| Lac | 1 | 2,4057 | 4,77% | 3,9554 | 3,95538 | 2,26 | 0,159 |
| HPMC\_Visc | 1 | 1,4642 | 2,90% | 0,8895 | 0,88953 | 0,51 | 0,490 |
| HPMC\_HP | 1 | 6,6630 | 13,20% | 9,2606 | 9,26064 | 5,29 | 0,040 |
| HPMC\_PS | 1 | 0,1583 | 0,31% | 0,0016 | 0,00165 | 0,00 | 0,976 |
| Square | 4 | 13,9722 | 27,68% | 11,9811 | 2,99528 | 1,71 | 0,212 |
| Lac\*Lac | 1 | 10,8993 | 21,59% | 7,3007 | 7,30065 | 4,17 | 0,064 |
| HPMC\_Visc\*HPMC\_Visc | 1 | 1,0737 | 2,13% | 0,0092 | 0,00919 | 0,01 | 0,943 |
| HPMC\_HP\*HPMC\_HP | 1 | 1,5749 | 3,12% | 0,0047 | 0,00471 | 0,00 | 0,959 |
| HPMC\_PS\*HPMC\_PS | 1 | 0,4243 | 0,84% | 0,8510 | 0,85099 | 0,49 | 0,499 |
| 2-Way Interaction | 6 | 4,8039 | 9,52% | 4,8039 | 0,80065 | 0,46 | 0,827 |
| Lac\*HPMC\_Visc | 1 | 0,8456 | 1,68% | 0,9312 | 0,93124 | 0,53 | 0,480 |
| Lac\*HPMC\_HP | 1 | 0,9879 | 1,96% | 0,7319 | 0,73187 | 0,42 | 0,530 |
| Lac\*HPMC\_PS | 1 | 0,2121 | 0,42% | 0,2121 | 0,21209 | 0,12 | 0,734 |
| HPMC\_Visc\*HPMC\_HP | 1 | 0,0312 | 0,06% | 1,8517 | 1,85168 | 1,06 | 0,324 |
| HPMC\_Visc\*HPMC\_PS | 1 | 0,2858 | 0,57% | 0,5700 | 0,57003 | 0,33 | 0,579 |
| HPMC\_HP\*HPMC\_PS | 1 | 2,4414 | 4,84% | 2,4414 | 2,44139 | 1,39 | 0,261 |
| Error | 12 | 21,0125 | 41,63% | 21,0125 | 1,75104 |  |  |
| Lack-of-Fit | 10 | 12,1338 | 24,04% | 12,1338 | 1,21338 | 0,27 | 0,936 |
| Pure Error | 2 | 8,8788 | 17,59% | 8,8788 | 4,43938 |  |  |
| Total | 26 | 50,4796 | 100,00% |  |  |  |  |

## Regression Equation in Uncoded Units

|  |  |  |
| --- | --- | --- |
| F\_SD\_15h(900min) | = | 229 - 107,8 Lac - 0,00153 HPMC\_Visc - 33,3 HPMC\_HP - 1,10 HPMC\_PS + 38,2 Lac\*Lac + 0,000000 HPMC\_Visc\*HPMC\_Visc + 0,06 HPMC\_HP\*HPMC\_HP - 0,0151 HPMC\_PS\*HPMC\_PS + 0,00097 Lac\*HPMC\_Visc + 3,45 Lac\*HPMC\_HP + 0,40 Lac\*HPMC\_PS + 0,000401 HPMC\_Visc\*HPMC\_HP - 0,000043 HPMC\_Visc\*HPMC\_PS + 0,379 HPMC\_HP\*HPMC\_PS |

## Fits and Diagnostics for All Observations

| Obs | F\_SD\_15h(900min) | Fit | SE Fit | 95% CI | Resid | Std Resid | Del Resid | HI |
| --- | --- | --- | --- | --- | --- | --- | --- | --- |
| 1 | 4,545 | 3,928 | 1,072 | (1,593; 6,263) | 0,617 | 0,79 | 0,78 | 0,655994 |
| 2 | 3,098 | 3,333 | 1,072 | (0,997; 5,668) | -0,235 | -0,30 | -0,29 | 0,655994 |
| 3 | 1,836 | 2,322 | 1,131 | (-0,141; 4,786) | -0,486 | -0,71 | -0,69 | 0,729945 |
| 4 | 2,994 | 2,776 | 1,131 | (0,313; 5,239) | 0,218 | 0,32 | 0,31 | 0,729945 |
| 5 | 3,497 | 3,163 | 0,914 | (1,172; 5,153) | 0,334 | 0,35 | 0,34 | 0,476813 |
| 6 | 4,903 | 3,490 | 0,914 | (1,500; 5,481) | 1,413 | 1,48 | 1,56 | 0,476813 |
| 7 | 2,512 | 2,977 | 1,098 | (0,584; 5,369) | -0,464 | -0,63 | -0,61 | 0,688750 |
| 8 | 5,260 | 4,659 | 1,098 | (2,266; 7,052) | 0,601 | 0,81 | 0,80 | 0,688750 |
| 9 | 1,653 | 2,834 | 1,009 | (0,636; 5,032) | -1,181 | -1,38 | -1,44 | 0,580949 |
| 10 | 3,621 | 2,774 | 1,009 | (0,576; 4,971) | 0,847 | 0,99 | 0,99 | 0,580949 |
| 11 | 1,294 | 1,565 | 0,863 | (-0,316; 3,446) | -0,271 | -0,27 | -0,26 | 0,425781 |
| 12 | 1,300 | 2,364 | 0,863 | (0,483; 4,246) | -1,065 | -1,06 | -1,07 | 0,425781 |
| 13 | 4,017 | 4,036 | 0,998 | (1,861; 6,211) | -0,019 | -0,02 | -0,02 | 0,569258 |
| 14 | 5,133 | 4,995 | 0,998 | (2,820; 7,171) | 0,138 | 0,16 | 0,15 | 0,569258 |
| 15 | 2,349 | 2,636 | 0,871 | (0,739; 4,534) | -0,288 | -0,29 | -0,28 | 0,433227 |
| 16 | 3,590 | 4,130 | 0,871 | (2,232; 6,027) | -0,539 | -0,54 | -0,52 | 0,433227 |
| 17 | 5,204 | 4,373 | 1,023 | (2,143; 6,602) | 0,831 | 0,99 | 0,99 | 0,597775 |
| 18 | 4,905 | 5,642 | 1,023 | (3,412; 7,871) | -0,736 | -0,88 | -0,87 | 0,597775 |
| 19 | 1,843 | 3,234 | 0,844 | (1,395; 5,073) | -1,392 | -1,37 | -1,42 | 0,407038 |
| 20 | 2,649 | 1,965 | 1,065 | (-0,355; 4,285) | 0,684 | 0,87 | 0,86 | 0,647412 |
| 21 | 2,543 | 1,908 | 0,845 | (0,067; 3,750) | 0,635 | 0,62 | 0,61 | 0,407933 |
| 22 | 3,716 | 4,072 | 1,196 | (1,466; 6,679) | -0,356 | -0,63 | -0,61 | 0,817044 |
| 23 | 1,152 | 1,691 | 1,201 | (-0,925; 4,307) | -0,539 | -0,97 | -0,97 | 0,823434 |
| 24 | 2,288 | 1,620 | 1,118 | (-0,816; 4,056) | 0,668 | 0,94 | 0,94 | 0,713775 |
| 25 | 1,533 | 2,622 | 0,711 | (1,072; 4,171) | -1,089 | -0,98 | -0,97 | 0,288793 |
| 26 | 5,248 | 2,622 | 0,711 | (1,072; 4,171) | 2,627 | 2,35 | 3,07 | 0,288793 |
| 27 | 1,669 | 2,622 | 0,711 | (1,072; 4,171) | -0,953 | -0,85 | -0,84 | 0,288793 |

| Obs | Cook’s D | DFITS |  |
| --- | --- | --- | --- |
| 1 | 0,08 | 1,07917 |  |
| 2 | 0,01 | -0,40178 |  |
| 3 | 0,09 | -1,13627 |  |
| 4 | 0,02 | 0,50193 |  |
| 5 | 0,01 | 0,32080 |  |
| 6 | 0,13 | 1,49113 |  |
| 7 | 0,06 | -0,91092 |  |
| 8 | 0,10 | 1,19242 |  |
| 9 | 0,18 | -1,69458 |  |
| 10 | 0,09 | 1,16307 |  |
| 11 | 0,00 | -0,22328 |  |
| 12 | 0,06 | -0,91968 |  |
| 13 | 0,00 | -0,02395 |  |
| 14 | 0,00 | 0,17531 |  |
| 15 | 0,00 | -0,24250 |  |
| 16 | 0,01 | -0,45884 |  |
| 17 | 0,10 | 1,20680 |  |
| 18 | 0,08 | -1,05882 |  |
| 19 | 0,09 | -1,17874 |  |
| 20 | 0,09 | 1,16641 |  |
| 21 | 0,02 | 0,50369 |  |
| 22 | 0,12 | -1,29452 |  |
| 23 | 0,29 | -2,08637 |  |
| 24 | 0,15 | 1,48260 |  |
| 25 | 0,03 | -0,62044 |  |
| 26 | 0,15 | 1,95710 | R |
| 27 | 0,02 | -0,53755 |  |

R  Large residual

## Coded Coefficients

| Term | Coef | SE Coef | 95% CI | T-Value | P-Value | VIF |
| --- | --- | --- | --- | --- | --- | --- |
| Constant | 2,442 | 0,668 | (0,986; 3,898) | 3,65 | 0,003 |  |
| Lac | 0,549 | 0,553 | (-0,654; 1,753) | 0,99 | 0,340 | 1,18 |
| HPMC\_Visc | -0,221 | 0,577 | (-1,479; 1,037) | -0,38 | 0,708 | 1,70 |
| HPMC\_HP | 1,442 | 0,565 | (0,211; 2,672) | 2,55 | 0,025 | 1,26 |
| HPMC\_PS | -0,229 | 0,866 | (-2,116; 1,659) | -0,26 | 0,796 | 2,09 |
| Lac\*Lac | 1,98 | 1,10 | (-0,41; 4,38) | 1,81 | 0,096 | 1,30 |
| HPMC\_Visc\*HPMC\_Visc | 0,20 | 1,17 | (-2,35; 2,74) | 0,17 | 0,868 | 1,96 |
| HPMC\_HP\*HPMC\_HP | 0,44 | 1,11 | (-1,97; 2,86) | 0,40 | 0,696 | 1,81 |
| HPMC\_PS\*HPMC\_PS | -0,61 | 1,10 | (-3,01; 1,79) | -0,55 | 0,589 | 1,42 |
| Lac\*HPMC\_Visc | 1,06 | 1,21 | (-1,58; 3,70) | 0,88 | 0,399 | 1,49 |
| Lac\*HPMC\_HP | 0,21 | 1,27 | (-2,56; 2,98) | 0,17 | 0,870 | 1,17 |
| Lac\*HPMC\_PS | -0,03 | 1,96 | (-4,31; 4,24) | -0,02 | 0,988 | 1,42 |
| HPMC\_Visc\*HPMC\_HP | 1,58 | 1,45 | (-1,57; 4,73) | 1,09 | 0,296 | 2,73 |
| HPMC\_Visc\*HPMC\_PS | -0,95 | 2,04 | (-5,40; 3,49) | -0,47 | 0,649 | 2,74 |
| HPMC\_HP\*HPMC\_PS | 2,45 | 2,25 | (-2,46; 7,36) | 1,09 | 0,299 | 2,70 |

## Model Summary

| S | R-sq | R-sq(adj) | PRESS | R-sq(pred) | AICc | BIC |
| --- | --- | --- | --- | --- | --- | --- |
| 1,24394 | 55,07% | 2,66% | 91,0098 | 0,00% | 152,92 | 119,25 |

## Analysis of Variance

| Source | DF | Seq SS | Contribution | Adj SS | Adj MS | F-Value | P-Value |
| --- | --- | --- | --- | --- | --- | --- | --- |
| Model | 14 | 22,7619 | 55,07% | 22,7619 | 1,6258 | 1,05 | 0,471 |
| Linear | 4 | 8,2916 | 20,06% | 12,0517 | 3,0129 | 1,95 | 0,167 |
| Lac | 1 | 0,8545 | 2,07% | 1,5296 | 1,5296 | 0,99 | 0,340 |
| HPMC\_Visc | 1 | 0,2538 | 0,61% | 0,2271 | 0,2271 | 0,15 | 0,708 |
| HPMC\_HP | 1 | 6,2820 | 15,20% | 10,0794 | 10,0794 | 6,51 | 0,025 |
| HPMC\_PS | 1 | 0,9013 | 2,18% | 0,1080 | 0,1080 | 0,07 | 0,796 |
| Square | 4 | 10,5366 | 25,49% | 7,8487 | 1,9622 | 1,27 | 0,336 |
| Lac\*Lac | 1 | 6,7489 | 16,33% | 5,0500 | 5,0500 | 3,26 | 0,096 |
| HPMC\_Visc\*HPMC\_Visc | 1 | 0,9475 | 2,29% | 0,0444 | 0,0444 | 0,03 | 0,868 |
| HPMC\_HP\*HPMC\_HP | 1 | 2,5730 | 6,23% | 0,2486 | 0,2486 | 0,16 | 0,696 |
| HPMC\_PS\*HPMC\_PS | 1 | 0,2672 | 0,65% | 0,4766 | 0,4766 | 0,31 | 0,589 |
| 2-Way Interaction | 6 | 3,9337 | 9,52% | 3,9337 | 0,6556 | 0,42 | 0,850 |
| Lac\*HPMC\_Visc | 1 | 1,7054 | 4,13% | 1,1852 | 1,1852 | 0,77 | 0,399 |
| Lac\*HPMC\_HP | 1 | 0,0438 | 0,11% | 0,0433 | 0,0433 | 0,03 | 0,870 |
| Lac\*HPMC\_PS | 1 | 0,0004 | 0,00% | 0,0004 | 0,0004 | 0,00 | 0,988 |
| HPMC\_Visc\*HPMC\_HP | 1 | 0,2068 | 0,50% | 1,8476 | 1,8476 | 1,19 | 0,296 |
| HPMC\_Visc\*HPMC\_PS | 1 | 0,1520 | 0,37% | 0,3378 | 0,3378 | 0,22 | 0,649 |
| HPMC\_HP\*HPMC\_PS | 1 | 1,8252 | 4,42% | 1,8252 | 1,8252 | 1,18 | 0,299 |
| Error | 12 | 18,5687 | 44,93% | 18,5687 | 1,5474 |  |  |
| Lack-of-Fit | 10 | 11,0138 | 26,65% | 11,0138 | 1,1014 | 0,29 | 0,927 |
| Pure Error | 2 | 7,5549 | 18,28% | 7,5549 | 3,7774 |  |  |
| Total | 26 | 41,3306 | 100,00% |  |  |  |  |

## Regression Equation in Uncoded Units

|  |  |  |
| --- | --- | --- |
| F\_SD\_16h(960min) | = | 232 - 51,0 Lac - 0,00242 HPMC\_Visc - 35,4 HPMC\_HP - 1,11 HPMC\_PS + 31,7 Lac\*Lac + 0,000000 HPMC\_Visc\*HPMC\_Visc + 0,43 HPMC\_HP\*HPMC\_HP - 0,0113 HPMC\_PS\*HPMC\_PS + 0,00109 Lac\*HPMC\_Visc + 0,84 Lac\*HPMC\_HP - 0,02 Lac\*HPMC\_PS + 0,000400 HPMC\_Visc\*HPMC\_HP - 0,000033 HPMC\_Visc\*HPMC\_PS + 0,328 HPMC\_HP\*HPMC\_PS |

## Fits and Diagnostics for All Observations

| Obs | F\_SD\_16h(960min) | Fit | SE Fit | 95% CI | Resid | Std Resid | Del Resid | HI |
| --- | --- | --- | --- | --- | --- | --- | --- | --- |
| 1 | 4,115 | 3,661 | 1,008 | (1,466; 5,856) | 0,454 | 0,62 | 0,61 | 0,655994 |
| 2 | 3,274 | 3,310 | 1,008 | (1,115; 5,505) | -0,036 | -0,05 | -0,05 | 0,655994 |
| 3 | 1,831 | 2,032 | 1,063 | (-0,284; 4,347) | -0,200 | -0,31 | -0,30 | 0,729945 |
| 4 | 3,457 | 3,170 | 1,063 | (0,854; 5,486) | 0,287 | 0,44 | 0,43 | 0,729945 |
| 5 | 3,358 | 3,142 | 0,859 | (1,271; 5,014) | 0,215 | 0,24 | 0,23 | 0,476813 |
| 6 | 4,508 | 3,052 | 0,859 | (1,181; 4,924) | 1,456 | 1,62 | 1,75 | 0,476813 |
| 7 | 2,764 | 3,297 | 1,032 | (1,048; 5,547) | -0,533 | -0,77 | -0,75 | 0,688750 |
| 8 | 5,430 | 4,713 | 1,032 | (2,463; 6,962) | 0,717 | 1,03 | 1,04 | 0,688750 |
| 9 | 1,468 | 2,709 | 0,948 | (0,643; 4,775) | -1,241 | -1,54 | -1,65 | 0,580949 |
| 10 | 3,186 | 2,367 | 0,948 | (0,301; 4,433) | 0,819 | 1,02 | 1,02 | 0,580949 |
| 11 | 1,246 | 1,493 | 0,812 | (-0,276; 3,262) | -0,247 | -0,26 | -0,25 | 0,425781 |
| 12 | 1,172 | 2,137 | 0,812 | (0,368; 3,905) | -0,965 | -1,02 | -1,03 | 0,425781 |
| 13 | 3,866 | 4,045 | 0,939 | (2,000; 6,090) | -0,179 | -0,22 | -0,21 | 0,569258 |
| 14 | 3,716 | 3,922 | 0,939 | (1,877; 5,967) | -0,207 | -0,25 | -0,24 | 0,569258 |
| 15 | 2,365 | 2,728 | 0,819 | (0,944; 4,512) | -0,363 | -0,39 | -0,37 | 0,433227 |
| 16 | 3,375 | 3,604 | 0,819 | (1,820; 5,388) | -0,230 | -0,25 | -0,24 | 0,433227 |
| 17 | 5,095 | 4,079 | 0,962 | (1,984; 6,175) | 1,016 | 1,29 | 1,33 | 0,597775 |
| 18 | 3,807 | 4,759 | 0,962 | (2,664; 6,855) | -0,952 | -1,21 | -1,23 | 0,597775 |
| 19 | 2,004 | 2,919 | 0,794 | (1,190; 4,648) | -0,915 | -0,96 | -0,95 | 0,407038 |
| 20 | 2,429 | 2,124 | 1,001 | (-0,057; 4,304) | 0,305 | 0,41 | 0,40 | 0,647412 |
| 21 | 2,386 | 2,032 | 0,795 | (0,301; 3,763) | 0,355 | 0,37 | 0,36 | 0,407933 |
| 22 | 4,091 | 4,298 | 1,124 | (1,848; 6,748) | -0,207 | -0,39 | -0,37 | 0,817044 |
| 23 | 1,311 | 1,990 | 1,129 | (-0,469; 4,449) | -0,679 | -1,30 | -1,34 | 0,823434 |
| 24 | 2,169 | 1,350 | 1,051 | (-0,940; 3,640) | 0,819 | 1,23 | 1,26 | 0,713775 |
| 25 | 1,404 | 2,435 | 0,668 | (0,979; 3,892) | -1,031 | -0,98 | -0,98 | 0,288793 |
| 26 | 4,848 | 2,435 | 0,668 | (0,979; 3,892) | 2,413 | 2,30 | 2,95 | 0,288793 |
| 27 | 1,566 | 2,435 | 0,668 | (0,979; 3,892) | -0,869 | -0,83 | -0,82 | 0,288793 |

| Obs | Cook’s D | DFITS |  |
| --- | --- | --- | --- |
| 1 | 0,05 | 0,83606 |  |
| 2 | 0,00 | -0,06612 |  |
| 3 | 0,02 | -0,49014 |  |
| 4 | 0,04 | 0,70473 |  |
| 5 | 0,00 | 0,21923 |  |
| 6 | 0,16 | 1,67291 |  |
| 7 | 0,09 | -1,12180 |  |
| 8 | 0,16 | 1,54179 |  |
| 9 | 0,22 | -1,94045 |  |
| 10 | 0,10 | 1,19926 |  |
| 11 | 0,00 | -0,21661 |  |
| 12 | 0,05 | -0,88314 |  |
| 13 | 0,00 | -0,24206 |  |
| 14 | 0,01 | -0,27932 |  |
| 15 | 0,01 | -0,32642 |  |
| 16 | 0,00 | -0,20583 |  |
| 17 | 0,16 | 1,61864 |  |
| 18 | 0,14 | -1,50336 |  |
| 19 | 0,04 | -0,78829 |  |
| 20 | 0,02 | 0,53988 |  |
| 21 | 0,01 | 0,29605 |  |
| 22 | 0,04 | -0,79145 |  |
| 23 | 0,53 | -2,89925 |  |
| 24 | 0,25 | 1,98913 |  |
| 25 | 0,03 | -0,62526 |  |
| 26 | 0,14 | 1,87710 | R |
| 27 | 0,02 | -0,52079 |  |

R  Large residual

## Coded Coefficients

| Term | Coef | SE Coef | 95% CI | T-Value | P-Value | VIF |
| --- | --- | --- | --- | --- | --- | --- |
| Constant | 2,255 | 0,667 | (0,802; 3,707) | 3,38 | 0,005 |  |
| Lac | 0,220 | 0,551 | (-0,981; 1,421) | 0,40 | 0,696 | 1,18 |
| HPMC\_Visc | -0,120 | 0,576 | (-1,375; 1,134) | -0,21 | 0,838 | 1,70 |
| HPMC\_HP | 1,253 | 0,563 | (0,025; 2,481) | 2,22 | 0,046 | 1,26 |
| HPMC\_PS | -0,225 | 0,864 | (-2,107; 1,658) | -0,26 | 0,799 | 2,09 |
| Lac\*Lac | 1,60 | 1,10 | (-0,79; 3,98) | 1,46 | 0,170 | 1,30 |
| HPMC\_Visc\*HPMC\_Visc | 0,44 | 1,16 | (-2,10; 2,98) | 0,38 | 0,713 | 1,96 |
| HPMC\_HP\*HPMC\_HP | 0,67 | 1,10 | (-1,73; 3,08) | 0,61 | 0,553 | 1,81 |
| HPMC\_PS\*HPMC\_PS | -0,43 | 1,10 | (-2,82; 1,96) | -0,39 | 0,701 | 1,42 |
| Lac\*HPMC\_Visc | 1,72 | 1,21 | (-0,92; 4,35) | 1,42 | 0,181 | 1,49 |
| Lac\*HPMC\_HP | 0,00 | 1,27 | (-2,76; 2,76) | 0,00 | 0,999 | 1,17 |
| Lac\*HPMC\_PS | 0,22 | 1,96 | (-4,04; 4,48) | 0,11 | 0,912 | 1,42 |
| HPMC\_Visc\*HPMC\_HP | 1,63 | 1,44 | (-1,51; 4,78) | 1,13 | 0,279 | 2,73 |
| HPMC\_Visc\*HPMC\_PS | 0,08 | 2,04 | (-4,36; 4,52) | 0,04 | 0,970 | 2,74 |
| HPMC\_HP\*HPMC\_PS | 2,56 | 2,25 | (-2,34; 7,46) | 1,14 | 0,278 | 2,70 |

## Model Summary

| S | R-sq | R-sq(adj) | PRESS | R-sq(pred) | AICc | BIC |
| --- | --- | --- | --- | --- | --- | --- |
| 1,24094 | 51,74% | 0,00% | 103,481 | 0,00% | 152,78 | 119,12 |

## Analysis of Variance

| Source | DF | Seq SS | Contribution | Adj SS | Adj MS | F-Value | P-Value |
| --- | --- | --- | --- | --- | --- | --- | --- |
| Model | 14 | 19,8149 | 51,74% | 19,8149 | 1,41535 | 0,92 | 0,565 |
| Linear | 4 | 5,8063 | 15,16% | 8,1065 | 2,02661 | 1,32 | 0,319 |
| Lac | 1 | 0,0014 | 0,00% | 0,2459 | 0,24586 | 0,16 | 0,696 |
| HPMC\_Visc | 1 | 0,2594 | 0,68% | 0,0673 | 0,06732 | 0,04 | 0,838 |
| HPMC\_HP | 1 | 3,4706 | 9,06% | 7,6164 | 7,61636 | 4,95 | 0,046 |
| HPMC\_PS | 1 | 2,0749 | 5,42% | 0,1041 | 0,10406 | 0,07 | 0,799 |
| Square | 4 | 7,5268 | 19,66% | 5,0704 | 1,26760 | 0,82 | 0,535 |
| Lac\*Lac | 1 | 3,7327 | 9,75% | 3,2772 | 3,27717 | 2,13 | 0,170 |
| HPMC\_Visc\*HPMC\_Visc | 1 | 0,6248 | 1,63% | 0,2186 | 0,21862 | 0,14 | 0,713 |
| HPMC\_HP\*HPMC\_HP | 1 | 2,7695 | 7,23% | 0,5739 | 0,57387 | 0,37 | 0,553 |
| HPMC\_PS\*HPMC\_PS | 1 | 0,3998 | 1,04% | 0,2387 | 0,23873 | 0,16 | 0,701 |
| 2-Way Interaction | 6 | 6,4819 | 16,93% | 6,4819 | 1,08031 | 0,70 | 0,654 |
| Lac\*HPMC\_Visc | 1 | 3,9534 | 10,32% | 3,1070 | 3,10698 | 2,02 | 0,181 |
| Lac\*HPMC\_HP | 1 | 0,0013 | 0,00% | 0,0000 | 0,00000 | 0,00 | 0,999 |
| Lac\*HPMC\_PS | 1 | 0,0195 | 0,05% | 0,0195 | 0,01946 | 0,01 | 0,912 |
| HPMC\_Visc\*HPMC\_HP | 1 | 0,4502 | 1,18% | 1,9757 | 1,97566 | 1,28 | 0,279 |
| HPMC\_Visc\*HPMC\_PS | 1 | 0,0648 | 0,17% | 0,0023 | 0,00227 | 0,00 | 0,970 |
| HPMC\_HP\*HPMC\_PS | 1 | 1,9927 | 5,20% | 1,9927 | 1,99269 | 1,29 | 0,278 |
| Error | 12 | 18,4791 | 48,26% | 18,4791 | 1,53993 |  |  |
| Lack-of-Fit | 10 | 12,0816 | 31,55% | 12,0816 | 1,20816 | 0,38 | 0,881 |
| Pure Error | 2 | 6,3975 | 16,71% | 6,3975 | 3,19877 |  |  |
| Total | 26 | 38,2940 | 100,00% |  |  |  |  |

## Regression Equation in Uncoded Units

|  |  |  |
| --- | --- | --- |
| F\_SD\_17h(1020min) | = | 322 - 56,9 Lac - 0,00581 HPMC\_Visc - 40,6 HPMC\_HP - 2,26 HPMC\_PS + 25,6 Lac\*Lac + 0,000000 HPMC\_Visc\*HPMC\_Visc + 0,65 HPMC\_HP\*HPMC\_HP - 0,0080 HPMC\_PS\*HPMC\_PS + 0,00176 Lac\*HPMC\_Visc + 0,01 Lac\*HPMC\_HP + 0,12 Lac\*HPMC\_PS + 0,000414 HPMC\_Visc\*HPMC\_HP + 0,000003 HPMC\_Visc\*HPMC\_PS + 0,343 HPMC\_HP\*HPMC\_PS |

## Fits and Diagnostics for All Observations

| Obs | F\_SD\_17h(1020min) | Fit | SE Fit | 95% CI | Resid | Std Resid | Del Resid |
| --- | --- | --- | --- | --- | --- | --- | --- |
| 1 | 5,036 | 4,275 | 1,005 | (2,086; 6,465) | 0,761 | 1,05 | 1,05 |
| 2 | 3,080 | 3,218 | 1,005 | (1,028; 5,408) | -0,138 | -0,19 | -0,18 |
| 3 | 1,802 | 1,801 | 1,060 | (-0,509; 4,111) | 0,001 | 0,00 | 0,00 |
| 4 | 3,395 | 3,076 | 1,060 | (0,766; 5,386) | 0,319 | 0,49 | 0,48 |
| 5 | 3,392 | 3,395 | 0,857 | (1,528; 5,262) | -0,003 | -0,00 | -0,00 |
| 6 | 4,087 | 2,464 | 0,857 | (0,597; 4,331) | 1,624 | 1,81 | 2,03 |
| 7 | 2,411 | 2,937 | 1,030 | (0,693; 5,181) | -0,527 | -0,76 | -0,75 |
| 8 | 5,066 | 4,292 | 1,030 | (2,048; 6,536) | 0,775 | 1,12 | 1,13 |
| 9 | 1,271 | 2,704 | 0,946 | (0,643; 4,765) | -1,433 | -1,78 | -1,99 |
| 10 | 2,708 | 1,826 | 0,946 | (-0,235; 3,887) | 0,881 | 1,10 | 1,11 |
| 11 | 1,171 | 1,439 | 0,810 | (-0,325; 3,203) | -0,268 | -0,28 | -0,27 |
| 12 | 1,334 | 2,032 | 0,810 | (0,268; 3,796) | -0,698 | -0,74 | -0,73 |
| 13 | 3,689 | 3,825 | 0,936 | (1,785; 5,865) | -0,136 | -0,17 | -0,16 |
| 14 | 2,400 | 2,958 | 0,936 | (0,918; 4,998) | -0,557 | -0,68 | -0,67 |
| 15 | 2,340 | 2,622 | 0,817 | (0,842; 4,401) | -0,282 | -0,30 | -0,29 |
| 16 | 2,990 | 3,385 | 0,817 | (1,606; 5,165) | -0,395 | -0,42 | -0,41 |
| 17 | 4,897 | 3,964 | 0,959 | (1,873; 6,054) | 0,934 | 1,19 | 1,21 |
| 18 | 2,830 | 3,745 | 0,959 | (1,654; 5,835) | -0,914 | -1,16 | -1,18 |
| 19 | 2,106 | 2,796 | 0,792 | (1,071; 4,521) | -0,690 | -0,72 | -0,71 |
| 20 | 2,241 | 2,169 | 0,998 | (-0,006; 4,345) | 0,072 | 0,10 | 0,09 |
| 21 | 2,322 | 2,296 | 0,793 | (0,569; 4,022) | 0,026 | 0,03 | 0,03 |
| 22 | 4,069 | 4,138 | 1,122 | (1,694; 6,582) | -0,069 | -0,13 | -0,13 |
| 23 | 1,438 | 2,259 | 1,126 | (-0,194; 4,713) | -0,821 | -1,57 | -1,69 |
| 24 | 2,048 | 1,080 | 1,048 | (-1,204; 3,364) | 0,968 | 1,46 | 1,54 |
| 25 | 1,391 | 2,256 | 0,667 | (0,803; 3,709) | -0,865 | -0,83 | -0,81 |
| 26 | 4,512 | 2,256 | 0,667 | (0,803; 3,709) | 2,256 | 2,16 | 2,64 |
| 27 | 1,437 | 2,256 | 0,667 | (0,803; 3,709) | -0,819 | -0,78 | -0,77 |

| Obs | HI | Cook’s D | DFITS |  |
| --- | --- | --- | --- | --- |
| 1 | 0,655994 | 0,14 | 1,44974 |  |
| 2 | 0,655994 | 0,00 | -0,25120 |  |
| 3 | 0,729945 | 0,00 | 0,00225 |  |
| 4 | 0,729945 | 0,04 | 0,78614 |  |
| 5 | 0,476813 | 0,00 | -0,00315 |  |
| 6 | 0,476813 | 0,20 | 1,93864 |  |
| 7 | 0,688750 | 0,09 | -1,11035 |  |
| 8 | 0,688750 | 0,18 | 1,68397 |  |
| 9 | 0,580949 | 0,29 | -2,34509 |  |
| 10 | 0,580949 | 0,11 | 1,30407 |  |
| 11 | 0,425781 | 0,00 | -0,23535 |  |
| 12 | 0,425781 | 0,03 | -0,62657 |  |
| 13 | 0,569258 | 0,00 | -0,18442 |  |
| 14 | 0,569258 | 0,04 | -0,76843 |  |
| 15 | 0,433227 | 0,00 | -0,25373 |  |
| 16 | 0,433227 | 0,01 | -0,35694 |  |
| 17 | 0,597775 | 0,14 | 1,47369 |  |
| 18 | 0,597775 | 0,13 | -1,43949 |  |
| 19 | 0,407038 | 0,02 | -0,58566 |  |
| 20 | 0,647412 | 0,00 | 0,12713 |  |
| 21 | 0,407933 | 0,00 | 0,02173 |  |
| 22 | 0,817044 | 0,01 | -0,26503 |  |
| 23 | 0,823434 | 0,77 | -3,65619 |  |
| 24 | 0,713775 | 0,35 | 2,42980 |  |
| 25 | 0,288793 | 0,02 | -0,51926 |  |
| 26 | 0,288793 | 0,13 | 1,67988 | R |
| 27 | 0,288793 | 0,02 | -0,48986 |  |

R  Large residual

## Coded Coefficients

| Term | Coef | SE Coef | 95% CI | T-Value | P-Value | VIF |
| --- | --- | --- | --- | --- | --- | --- |
| Constant | 2,108 | 0,626 | (0,743; 3,473) | 3,37 | 0,006 |  |
| Lac | -0,068 | 0,518 | (-1,197; 1,061) | -0,13 | 0,897 | 1,18 |
| HPMC\_Visc | -0,059 | 0,541 | (-1,238; 1,120) | -0,11 | 0,915 | 1,70 |
| HPMC\_HP | 1,040 | 0,530 | (-0,114; 2,194) | 1,96 | 0,073 | 1,26 |
| HPMC\_PS | -0,234 | 0,812 | (-2,004; 1,535) | -0,29 | 0,778 | 2,09 |
| Lac\*Lac | 1,19 | 1,03 | (-1,05; 3,44) | 1,16 | 0,270 | 1,30 |
| HPMC\_Visc\*HPMC\_Visc | 0,48 | 1,09 | (-1,91; 2,86) | 0,44 | 0,671 | 1,96 |
| HPMC\_HP\*HPMC\_HP | 0,69 | 1,04 | (-1,58; 2,95) | 0,66 | 0,522 | 1,81 |
| HPMC\_PS\*HPMC\_PS | -0,28 | 1,03 | (-2,53; 1,96) | -0,28 | 0,787 | 1,42 |
| Lac\*HPMC\_Visc | 1,72 | 1,14 | (-0,75; 4,19) | 1,51 | 0,156 | 1,49 |
| Lac\*HPMC\_HP | -0,54 | 1,19 | (-3,14; 2,05) | -0,46 | 0,657 | 1,17 |
| Lac\*HPMC\_PS | 0,36 | 1,84 | (-3,65; 4,37) | 0,20 | 0,848 | 1,42 |
| HPMC\_Visc\*HPMC\_HP | 1,20 | 1,36 | (-1,76; 4,15) | 0,88 | 0,394 | 2,73 |
| HPMC\_Visc\*HPMC\_PS | 0,76 | 1,91 | (-3,41; 4,93) | 0,40 | 0,697 | 2,74 |
| HPMC\_HP\*HPMC\_PS | 1,87 | 2,11 | (-2,74; 6,47) | 0,88 | 0,394 | 2,70 |

## Model Summary

| S | R-sq | R-sq(adj) | PRESS | R-sq(pred) | AICc | BIC |
| --- | --- | --- | --- | --- | --- | --- |
| 1,16635 | 48,00% | 0,00% | 94,8999 | 0,00% | 149,44 | 115,77 |

## Analysis of Variance

| Source | DF | Seq SS | Contribution | Adj SS | Adj MS | F-Value | P-Value |
| --- | --- | --- | --- | --- | --- | --- | --- |
| Model | 14 | 15,0659 | 48,00% | 15,0659 | 1,07614 | 0,79 | 0,666 |
| Linear | 4 | 5,1274 | 16,33% | 5,4741 | 1,36852 | 1,01 | 0,442 |
| Lac | 1 | 0,3281 | 1,05% | 0,0236 | 0,02360 | 0,02 | 0,897 |
| HPMC\_Visc | 1 | 0,1622 | 0,52% | 0,0162 | 0,01616 | 0,01 | 0,915 |
| HPMC\_HP | 1 | 2,3563 | 7,51% | 5,2485 | 5,24855 | 3,86 | 0,073 |
| HPMC\_PS | 1 | 2,2808 | 7,27% | 0,1134 | 0,11337 | 0,08 | 0,778 |
| Square | 4 | 4,1667 | 13,27% | 2,9444 | 0,73610 | 0,54 | 0,709 |
| Lac\*Lac | 1 | 1,8168 | 5,79% | 1,8221 | 1,82208 | 1,34 | 0,270 |
| HPMC\_Visc\*HPMC\_Visc | 1 | 0,2001 | 0,64% | 0,2581 | 0,25813 | 0,19 | 0,671 |
| HPMC\_HP\*HPMC\_HP | 1 | 1,7175 | 5,47% | 0,5920 | 0,59197 | 0,44 | 0,522 |
| HPMC\_PS\*HPMC\_PS | 1 | 0,4323 | 1,38% | 0,1034 | 0,10340 | 0,08 | 0,787 |
| 2-Way Interaction | 6 | 5,7718 | 18,39% | 5,7718 | 0,96196 | 0,71 | 0,650 |
| Lac\*HPMC\_Visc | 1 | 3,6209 | 11,54% | 3,1214 | 3,12138 | 2,29 | 0,156 |
| Lac\*HPMC\_HP | 1 | 0,2417 | 0,77% | 0,2828 | 0,28278 | 0,21 | 0,657 |
| Lac\*HPMC\_PS | 1 | 0,0519 | 0,17% | 0,0519 | 0,05188 | 0,04 | 0,848 |
| HPMC\_Visc\*HPMC\_HP | 1 | 0,4085 | 1,30% | 1,0625 | 1,06246 | 0,78 | 0,394 |
| HPMC\_Visc\*HPMC\_PS | 1 | 0,3850 | 1,23% | 0,2160 | 0,21599 | 0,16 | 0,697 |
| HPMC\_HP\*HPMC\_PS | 1 | 1,0638 | 3,39% | 1,0638 | 1,06380 | 0,78 | 0,394 |
| Error | 12 | 16,3244 | 52,00% | 16,3244 | 1,36037 |  |  |
| Lack-of-Fit | 10 | 10,9604 | 34,92% | 10,9604 | 1,09604 | 0,41 | 0,864 |
| Pure Error | 2 | 5,3640 | 17,09% | 5,3640 | 2,68199 |  |  |
| Total | 26 | 31,3903 | 100,00% |  |  |  |  |

## Regression Equation in Uncoded Units

|  |  |  |
| --- | --- | --- |
| F\_SD\_18h(1080min) | = | 277 - 36,6 Lac - 0,00648 HPMC\_Visc - 32,0 HPMC\_HP - 2,13 HPMC\_PS + 19,1 Lac\*Lac + 0,000000 HPMC\_Visc\*HPMC\_Visc + 0,66 HPMC\_HP\*HPMC\_HP - 0,0053 HPMC\_PS\*HPMC\_PS + 0,00177 Lac\*HPMC\_Visc - 2,14 Lac\*HPMC\_HP + 0,20 Lac\*HPMC\_PS + 0,000303 HPMC\_Visc\*HPMC\_HP + 0,000027 HPMC\_Visc\*HPMC\_PS + 0,250 HPMC\_HP\*HPMC\_PS |

## Fits and Diagnostics for All Observations

| Obs | F\_SD\_18h(1080min) | Fit | SE Fit | 95% CI | Resid | Std Resid | Del Resid |
| --- | --- | --- | --- | --- | --- | --- | --- |
| 1 | 4,498 | 3,914 | 0,945 | (1,856; 5,972) | 0,584 | 0,85 | 0,84 |
| 2 | 2,928 | 2,924 | 0,945 | (0,866; 4,982) | 0,004 | 0,01 | 0,01 |
| 3 | 1,640 | 1,466 | 0,996 | (-0,705; 3,637) | 0,174 | 0,29 | 0,28 |
| 4 | 2,919 | 2,784 | 0,996 | (0,613; 4,955) | 0,135 | 0,22 | 0,21 |
| 5 | 3,257 | 3,458 | 0,805 | (1,704; 5,213) | -0,202 | -0,24 | -0,23 |
| 6 | 3,655 | 2,100 | 0,805 | (0,345; 3,855) | 1,555 | 1,84 | 2,09 |
| 7 | 2,244 | 2,770 | 0,968 | (0,661; 4,879) | -0,526 | -0,81 | -0,80 |
| 8 | 4,397 | 3,526 | 0,968 | (1,417; 5,635) | 0,871 | 1,34 | 1,39 |
| 9 | 0,937 | 2,327 | 0,889 | (0,390; 4,264) | -1,389 | -1,84 | -2,08 |
| 10 | 2,278 | 1,586 | 0,889 | (-0,351; 3,523) | 0,692 | 0,92 | 0,91 |
| 11 | 1,090 | 1,448 | 0,761 | (-0,211; 3,106) | -0,358 | -0,40 | -0,39 |
| 12 | 1,714 | 2,042 | 0,761 | (0,384; 3,700) | -0,328 | -0,37 | -0,36 |
| 13 | 3,428 | 3,452 | 0,880 | (1,535; 5,370) | -0,024 | -0,03 | -0,03 |
| 14 | 1,485 | 2,143 | 0,880 | (0,226; 4,060) | -0,658 | -0,86 | -0,85 |
| 15 | 2,188 | 2,536 | 0,768 | (0,864; 4,209) | -0,349 | -0,40 | -0,38 |
| 16 | 2,609 | 2,956 | 0,768 | (1,283; 4,628) | -0,347 | -0,39 | -0,38 |
| 17 | 4,700 | 3,676 | 0,902 | (1,711; 5,641) | 1,024 | 1,38 | 1,45 |
| 18 | 1,945 | 2,928 | 0,902 | (0,963; 4,893) | -0,983 | -1,33 | -1,38 |
| 19 | 2,139 | 2,551 | 0,744 | (0,930; 4,172) | -0,412 | -0,46 | -0,44 |
| 20 | 2,064 | 2,126 | 0,938 | (0,081; 4,171) | -0,062 | -0,09 | -0,09 |
| 21 | 2,264 | 2,227 | 0,745 | (0,604; 3,850) | 0,036 | 0,04 | 0,04 |
| 22 | 3,731 | 3,791 | 1,054 | (1,494; 6,088) | -0,060 | -0,12 | -0,12 |
| 23 | 1,610 | 2,392 | 1,058 | (0,086; 4,698) | -0,782 | -1,59 | -1,72 |
| 24 | 1,946 | 1,008 | 0,985 | (-1,138; 3,155) | 0,938 | 1,50 | 1,60 |
| 25 | 1,342 | 2,110 | 0,627 | (0,744; 3,476) | -0,769 | -0,78 | -0,77 |
| 26 | 4,156 | 2,110 | 0,627 | (0,744; 3,476) | 2,046 | 2,08 | 2,49 |
| 27 | 1,298 | 2,110 | 0,627 | (0,744; 3,476) | -0,813 | -0,83 | -0,81 |

| Obs | HI | Cook’s D | DFITS |  |
| --- | --- | --- | --- | --- |
| 1 | 0,655994 | 0,09 | 1,16430 |  |
| 2 | 0,655994 | 0,00 | 0,00796 |  |
| 3 | 0,729945 | 0,01 | 0,45419 |  |
| 4 | 0,729945 | 0,01 | 0,35176 |  |
| 5 | 0,476813 | 0,00 | -0,21899 |  |
| 6 | 0,476813 | 0,21 | 1,99056 |  |
| 7 | 0,688750 | 0,10 | -1,18416 |  |
| 8 | 0,688750 | 0,26 | 2,06596 |  |
| 9 | 0,580949 | 0,31 | -2,44811 |  |
| 10 | 0,580949 | 0,08 | 1,07132 |  |
| 11 | 0,425781 | 0,01 | -0,33598 |  |
| 12 | 0,425781 | 0,01 | -0,30738 |  |
| 13 | 0,569258 | 0,00 | -0,03518 |  |
| 14 | 0,569258 | 0,07 | -0,97623 |  |
| 15 | 0,433227 | 0,01 | -0,33458 |  |
| 16 | 0,433227 | 0,01 | -0,33258 |  |
| 17 | 0,597775 | 0,19 | 1,76322 |  |
| 18 | 0,597775 | 0,18 | -1,68002 |  |
| 19 | 0,407038 | 0,01 | -0,36720 |  |
| 20 | 0,647412 | 0,00 | -0,11580 |  |
| 21 | 0,407933 | 0,00 | 0,03216 |  |
| 22 | 0,817044 | 0,00 | -0,24416 |  |
| 23 | 0,823434 | 0,79 | -3,71419 |  |
| 24 | 0,713775 | 0,38 | 2,52258 |  |
| 25 | 0,288793 | 0,02 | -0,48936 |  |
| 26 | 0,288793 | 0,12 | 1,58665 | R |
| 27 | 0,288793 | 0,02 | -0,51898 |  |

R  Large residual

## Coded Coefficients

| Term | Coef | SE Coef | 95% CI | T-Value | P-Value | VIF |
| --- | --- | --- | --- | --- | --- | --- |
| Constant | 1,982 | 0,587 | (0,703; 3,261) | 3,38 | 0,006 |  |
| Lac | -0,409 | 0,485 | (-1,466; 0,649) | -0,84 | 0,416 | 1,18 |
| HPMC\_Visc | 0,010 | 0,507 | (-1,095; 1,115) | 0,02 | 0,984 | 1,70 |
| HPMC\_HP | 0,908 | 0,496 | (-0,173; 1,989) | 1,83 | 0,092 | 1,26 |
| HPMC\_PS | -0,317 | 0,761 | (-1,975; 1,341) | -0,42 | 0,684 | 2,09 |
| Lac\*Lac | 0,778 | 0,965 | (-1,324; 2,880) | 0,81 | 0,435 | 1,30 |
| HPMC\_Visc\*HPMC\_Visc | 0,29 | 1,03 | (-1,94; 2,52) | 0,28 | 0,783 | 1,96 |
| HPMC\_HP\*HPMC\_HP | 0,452 | 0,973 | (-1,668; 2,572) | 0,46 | 0,650 | 1,81 |
| HPMC\_PS\*HPMC\_PS | -0,084 | 0,967 | (-2,191; 2,023) | -0,09 | 0,932 | 1,42 |
| Lac\*HPMC\_Visc | 1,19 | 1,06 | (-1,13; 3,50) | 1,11 | 0,287 | 1,49 |
| Lac\*HPMC\_HP | -0,79 | 1,12 | (-3,22; 1,64) | -0,71 | 0,493 | 1,17 |
| Lac\*HPMC\_PS | 0,02 | 1,72 | (-3,74; 3,77) | 0,01 | 0,993 | 1,42 |
| HPMC\_Visc\*HPMC\_HP | 0,95 | 1,27 | (-1,82; 3,72) | 0,75 | 0,468 | 2,73 |
| HPMC\_Visc\*HPMC\_PS | 0,60 | 1,79 | (-3,31; 4,51) | 0,34 | 0,743 | 2,74 |
| HPMC\_HP\*HPMC\_PS | 1,15 | 1,98 | (-3,16; 5,47) | 0,58 | 0,571 | 2,70 |

## Model Summary

| S | R-sq | R-sq(adj) | PRESS | R-sq(pred) | AICc | BIC |
| --- | --- | --- | --- | --- | --- | --- |
| 1,09277 | 42,17% | 0,00% | 76,9891 | 0,00% | 145,92 | 112,25 |

## Analysis of Variance

| Source | DF | Seq SS | Contribution | Adj SS | Adj MS | F-Value | P-Value |
| --- | --- | --- | --- | --- | --- | --- | --- |
| Model | 14 | 10,4493 | 42,17% | 10,4493 | 0,74638 | 0,63 | 0,801 |
| Linear | 4 | 5,2521 | 21,20% | 5,2247 | 1,30617 | 1,09 | 0,403 |
| Lac | 1 | 1,3019 | 5,25% | 0,8464 | 0,84636 | 0,71 | 0,416 |
| HPMC\_Visc | 1 | 0,0072 | 0,03% | 0,0005 | 0,00049 | 0,00 | 0,984 |
| HPMC\_HP | 1 | 2,2049 | 8,90% | 4,0002 | 4,00023 | 3,35 | 0,092 |
| HPMC\_PS | 1 | 1,7381 | 7,01% | 0,2073 | 0,20728 | 0,17 | 0,684 |
| Square | 4 | 1,5815 | 6,38% | 1,0995 | 0,27486 | 0,23 | 0,916 |
| Lac\*Lac | 1 | 0,7317 | 2,95% | 0,7774 | 0,77739 | 0,65 | 0,435 |
| HPMC\_Visc\*HPMC\_Visc | 1 | 0,0385 | 0,16% | 0,0952 | 0,09517 | 0,08 | 0,783 |
| HPMC\_HP\*HPMC\_HP | 1 | 0,6754 | 2,73% | 0,2580 | 0,25803 | 0,22 | 0,650 |
| HPMC\_PS\*HPMC\_PS | 1 | 0,1359 | 0,55% | 0,0091 | 0,00907 | 0,01 | 0,932 |
| 2-Way Interaction | 6 | 3,6157 | 14,59% | 3,6157 | 0,60261 | 0,50 | 0,794 |
| Lac\*HPMC\_Visc | 1 | 1,8789 | 7,58% | 1,4827 | 1,48274 | 1,24 | 0,287 |
| Lac\*HPMC\_HP | 1 | 0,6282 | 2,54% | 0,5961 | 0,59606 | 0,50 | 0,493 |
| Lac\*HPMC\_PS | 1 | 0,0001 | 0,00% | 0,0001 | 0,00010 | 0,00 | 0,993 |
| HPMC\_Visc\*HPMC\_HP | 1 | 0,4874 | 1,97% | 0,6693 | 0,66931 | 0,56 | 0,468 |
| HPMC\_Visc\*HPMC\_PS | 1 | 0,2155 | 0,87% | 0,1349 | 0,13486 | 0,11 | 0,743 |
| HPMC\_HP\*HPMC\_PS | 1 | 0,4055 | 1,64% | 0,4055 | 0,40552 | 0,34 | 0,571 |
| Error | 12 | 14,3298 | 57,83% | 14,3298 | 1,19415 |  |  |
| Lack-of-Fit | 10 | 9,8346 | 39,69% | 9,8346 | 0,98346 | 0,44 | 0,848 |
| Pure Error | 2 | 4,4951 | 18,14% | 4,4951 | 2,24757 |  |  |
| Total | 26 | 24,7791 | 100,00% |  |  |  |  |

## Regression Equation in Uncoded Units

|  |  |  |
| --- | --- | --- |
| F\_SD\_19h(1140min) | = | 182 - 1,7 Lac - 0,00487 HPMC\_Visc - 19,9 HPMC\_HP - 1,58 HPMC\_PS + 12,5 Lac\*Lac + 0,000000 HPMC\_Visc\*HPMC\_Visc + 0,439 HPMC\_HP\*HPMC\_HP - 0,0016 HPMC\_PS\*HPMC\_PS + 0,00122 Lac\*HPMC\_Visc - 3,11 Lac\*HPMC\_HP + 0,008 Lac\*HPMC\_PS + 0,000241 HPMC\_Visc\*HPMC\_HP + 0,000021 HPMC\_Visc\*HPMC\_PS + 0,155 HPMC\_HP\*HPMC\_PS |

## Fits and Diagnostics for All Observations

| Obs | F\_SD\_19h(1140min) | Fit | SE Fit | 95% CI | Resid | Std Resid | Del Resid |
| --- | --- | --- | --- | --- | --- | --- | --- |
| 1 | 3,547 | 3,113 | 0,885 | (1,185; 5,042) | 0,434 | 0,68 | 0,66 |
| 2 | 2,278 | 2,426 | 0,885 | (0,497; 4,354) | -0,148 | -0,23 | -0,22 |
| 3 | 1,435 | 1,322 | 0,934 | (-0,713; 3,356) | 0,113 | 0,20 | 0,19 |
| 4 | 2,320 | 2,314 | 0,934 | (0,280; 4,349) | 0,006 | 0,01 | 0,01 |
| 5 | 2,563 | 3,121 | 0,755 | (1,477; 4,765) | -0,558 | -0,71 | -0,69 |
| 6 | 3,236 | 1,778 | 0,755 | (0,134; 3,422) | 1,458 | 1,85 | 2,09 |
| 7 | 2,453 | 2,887 | 0,907 | (0,911; 4,863) | -0,435 | -0,71 | -0,70 |
| 8 | 3,705 | 2,940 | 0,907 | (0,964; 4,916) | 0,765 | 1,25 | 1,29 |
| 9 | 0,697 | 2,046 | 0,833 | (0,231; 3,861) | -1,350 | -1,91 | -2,19 |
| 10 | 1,948 | 1,360 | 0,833 | (-0,455; 3,175) | 0,588 | 0,83 | 0,82 |
| 11 | 1,089 | 1,513 | 0,713 | (-0,041; 3,067) | -0,424 | -0,51 | -0,50 |
| 12 | 1,459 | 1,709 | 0,713 | (0,156; 3,263) | -0,251 | -0,30 | -0,29 |
| 13 | 3,254 | 3,100 | 0,824 | (1,304; 4,897) | 0,154 | 0,21 | 0,21 |
| 14 | 0,853 | 1,547 | 0,824 | (-0,250; 3,343) | -0,693 | -0,97 | -0,96 |
| 15 | 2,088 | 2,530 | 0,719 | (0,963; 4,097) | -0,442 | -0,54 | -0,52 |
| 16 | 2,136 | 2,324 | 0,719 | (0,757; 3,891) | -0,188 | -0,23 | -0,22 |
| 17 | 4,468 | 3,336 | 0,845 | (1,495; 5,177) | 1,132 | 1,63 | 1,77 |
| 18 | 1,269 | 2,159 | 0,845 | (0,318; 3,999) | -0,890 | -1,28 | -1,32 |
| 19 | 2,076 | 2,173 | 0,697 | (0,654; 3,692) | -0,096 | -0,11 | -0,11 |
| 20 | 1,964 | 1,961 | 0,879 | (0,045; 3,877) | 0,003 | 0,00 | 0,00 |
| 21 | 2,220 | 1,870 | 0,698 | (0,350; 3,391) | 0,350 | 0,42 | 0,40 |
| 22 | 3,190 | 3,277 | 0,988 | (1,124; 5,429) | -0,086 | -0,18 | -0,18 |
| 23 | 1,834 | 2,427 | 0,992 | (0,267; 4,588) | -0,593 | -1,29 | -1,33 |
| 24 | 1,946 | 1,139 | 0,923 | (-0,873; 3,150) | 0,807 | 1,38 | 1,44 |
| 25 | 1,260 | 1,969 | 0,587 | (0,690; 3,249) | -0,710 | -0,77 | -0,76 |
| 26 | 3,814 | 1,969 | 0,587 | (0,690; 3,249) | 1,845 | 2,00 | 2,35 |
| 27 | 1,176 | 1,969 | 0,587 | (0,690; 3,249) | -0,793 | -0,86 | -0,85 |

| Obs | HI | Cook’s D | DFITS |  |
| --- | --- | --- | --- | --- |
| 1 | 0,655994 | 0,06 | 0,91280 |  |
| 2 | 0,655994 | 0,01 | -0,30630 |  |
| 3 | 0,729945 | 0,01 | 0,31496 |  |
| 4 | 0,729945 | 0,00 | 0,01620 |  |
| 5 | 0,476813 | 0,03 | -0,65862 |  |
| 6 | 0,476813 | 0,21 | 1,99257 |  |
| 7 | 0,688750 | 0,07 | -1,03765 |  |
| 8 | 0,688750 | 0,23 | 1,91748 |  |
| 9 | 0,580949 | 0,34 | -2,57662 |  |
| 10 | 0,580949 | 0,06 | 0,96481 |  |
| 11 | 0,425781 | 0,01 | -0,42697 |  |
| 12 | 0,425781 | 0,00 | -0,25039 |  |
| 13 | 0,569258 | 0,00 | 0,23696 |  |
| 14 | 0,569258 | 0,08 | -1,10789 |  |
| 15 | 0,433227 | 0,01 | -0,45507 |  |
| 16 | 0,433227 | 0,00 | -0,19142 |  |
| 17 | 0,597775 | 0,26 | 2,16194 |  |
| 18 | 0,597775 | 0,16 | -1,61347 |  |
| 19 | 0,407038 | 0,00 | -0,09061 |  |
| 20 | 0,647412 | 0,00 | 0,00621 |  |
| 21 | 0,407933 | 0,01 | 0,33299 |  |
| 22 | 0,817044 | 0,01 | -0,37372 |  |
| 23 | 0,823434 | 0,52 | -2,87993 |  |
| 24 | 0,713775 | 0,32 | 2,27644 |  |
| 25 | 0,288793 | 0,02 | -0,48179 |  |
| 26 | 0,288793 | 0,11 | 1,49613 | R |
| 27 | 0,288793 | 0,02 | -0,54180 |  |

R  Large residual

## Coded Coefficients

| Term | Coef | SE Coef | 95% CI | T-Value | P-Value | VIF |
| --- | --- | --- | --- | --- | --- | --- |
| Constant | 1,844 | 0,564 | (0,616; 3,072) | 3,27 | 0,007 |  |
| Lac | -0,794 | 0,466 | (-1,810; 0,221) | -1,70 | 0,114 | 1,18 |
| HPMC\_Visc | -0,064 | 0,487 | (-1,125; 0,996) | -0,13 | 0,897 | 1,70 |
| HPMC\_HP | 0,834 | 0,476 | (-0,204; 1,872) | 1,75 | 0,105 | 1,26 |
| HPMC\_PS | -0,423 | 0,731 | (-2,015; 1,169) | -0,58 | 0,573 | 2,09 |
| Lac\*Lac | 0,384 | 0,926 | (-1,634; 2,402) | 0,41 | 0,686 | 1,30 |
| HPMC\_Visc\*HPMC\_Visc | 0,142 | 0,984 | (-2,003; 2,286) | 0,14 | 0,888 | 1,96 |
| HPMC\_HP\*HPMC\_HP | 0,270 | 0,934 | (-1,765; 2,305) | 0,29 | 0,777 | 1,81 |
| HPMC\_PS\*HPMC\_PS | 0,090 | 0,928 | (-1,933; 2,112) | 0,10 | 0,925 | 1,42 |
| Lac\*HPMC\_Visc | 1,00 | 1,02 | (-1,23; 3,22) | 0,98 | 0,349 | 1,49 |
| Lac\*HPMC\_HP | -0,87 | 1,07 | (-3,21; 1,46) | -0,82 | 0,430 | 1,17 |
| Lac\*HPMC\_PS | 0,20 | 1,65 | (-3,40; 3,81) | 0,12 | 0,904 | 1,42 |
| HPMC\_Visc\*HPMC\_HP | 0,66 | 1,22 | (-2,00; 3,31) | 0,54 | 0,599 | 2,73 |
| HPMC\_Visc\*HPMC\_PS | 0,40 | 1,72 | (-3,35; 4,16) | 0,23 | 0,819 | 2,74 |
| HPMC\_HP\*HPMC\_PS | 0,65 | 1,90 | (-3,50; 4,79) | 0,34 | 0,740 | 2,70 |

## Model Summary

| S | R-sq | R-sq(adj) | PRESS | R-sq(pred) | AICc | BIC |
| --- | --- | --- | --- | --- | --- | --- |
| 1,04916 | 44,54% | 0,00% | 68,9089 | 0,00% | 143,72 | 110,05 |

## Analysis of Variance

| Source | DF | Seq SS | Contribution | Adj SS | Adj MS | F-Value | P-Value |
| --- | --- | --- | --- | --- | --- | --- | --- |
| Model | 14 | 10,6073 | 44,54% | 10,6073 | 0,75767 | 0,69 | 0,750 |
| Linear | 4 | 7,8467 | 32,95% | 7,0939 | 1,77347 | 1,61 | 0,235 |
| Lac | 1 | 3,9985 | 16,79% | 3,1995 | 3,19946 | 2,91 | 0,114 |
| HPMC\_Visc | 1 | 0,0090 | 0,04% | 0,0193 | 0,01926 | 0,02 | 0,897 |
| HPMC\_HP | 1 | 2,3149 | 9,72% | 3,3737 | 3,37371 | 3,06 | 0,105 |
| HPMC\_PS | 1 | 1,5244 | 6,40% | 0,3695 | 0,36953 | 0,34 | 0,573 |
| Square | 4 | 0,3477 | 1,46% | 0,2268 | 0,05671 | 0,05 | 0,994 |
| Lac\*Lac | 1 | 0,1386 | 0,58% | 0,1892 | 0,18921 | 0,17 | 0,686 |
| HPMC\_Visc\*HPMC\_Visc | 1 | 0,0009 | 0,00% | 0,0228 | 0,02282 | 0,02 | 0,888 |
| HPMC\_HP\*HPMC\_HP | 1 | 0,2026 | 0,85% | 0,0922 | 0,09218 | 0,08 | 0,777 |
| HPMC\_PS\*HPMC\_PS | 1 | 0,0056 | 0,02% | 0,0103 | 0,01026 | 0,01 | 0,925 |
| 2-Way Interaction | 6 | 2,4129 | 10,13% | 2,4129 | 0,40214 | 0,37 | 0,887 |
| Lac\*HPMC\_Visc | 1 | 1,1331 | 4,76% | 1,0464 | 1,04640 | 0,95 | 0,349 |
| Lac\*HPMC\_HP | 1 | 0,7218 | 3,03% | 0,7327 | 0,73272 | 0,67 | 0,430 |
| Lac\*HPMC\_PS | 1 | 0,0168 | 0,07% | 0,0168 | 0,01680 | 0,02 | 0,904 |
| HPMC\_Visc\*HPMC\_HP | 1 | 0,3238 | 1,36% | 0,3207 | 0,32072 | 0,29 | 0,599 |
| HPMC\_Visc\*HPMC\_PS | 1 | 0,0902 | 0,38% | 0,0603 | 0,06028 | 0,05 | 0,819 |
| HPMC\_HP\*HPMC\_PS | 1 | 0,1272 | 0,53% | 0,1272 | 0,12718 | 0,12 | 0,740 |
| Error | 12 | 13,2087 | 55,46% | 13,2087 | 1,10073 |  |  |
| Lack-of-Fit | 10 | 9,2106 | 38,67% | 9,2106 | 0,92106 | 0,46 | 0,835 |
| Pure Error | 2 | 3,9981 | 16,79% | 3,9981 | 1,99906 |  |  |
| Total | 26 | 23,8161 | 100,00% |  |  |  |  |

## Regression Equation in Uncoded Units

|  |  |  |
| --- | --- | --- |
| F\_SD\_20h(1200min) | = | 121 + 1,8 Lac - 0,00334 HPMC\_Visc - 10,7 HPMC\_HP - 1,35 HPMC\_PS + 6,1 Lac\*Lac + 0,000000 HPMC\_Visc\*HPMC\_Visc + 0,262 HPMC\_HP\*HPMC\_HP + 0,0017 HPMC\_PS\*HPMC\_PS + 0,00102 Lac\*HPMC\_Visc - 3,45 Lac\*HPMC\_HP + 0,111 Lac\*HPMC\_PS + 0,000167 HPMC\_Visc\*HPMC\_HP + 0,000014 HPMC\_Visc\*HPMC\_PS + 0,087 HPMC\_HP\*HPMC\_PS |

## Fits and Diagnostics for All Observations

| Obs | F\_SD\_20h(1200min) | Fit | SE Fit | 95% CI | Resid | Std Resid | Del Resid |
| --- | --- | --- | --- | --- | --- | --- | --- |
| 1 | 3,129 | 2,693 | 0,850 | (0,841; 4,544) | 0,437 | 0,71 | 0,69 |
| 2 | 1,458 | 1,769 | 0,850 | (-0,082; 3,621) | -0,311 | -0,50 | -0,49 |
| 3 | 1,199 | 1,327 | 0,896 | (-0,626; 3,280) | -0,127 | -0,23 | -0,22 |
| 4 | 1,760 | 1,758 | 0,896 | (-0,195; 3,711) | 0,002 | 0,00 | 0,00 |
| 5 | 2,589 | 3,062 | 0,724 | (1,483; 4,640) | -0,472 | -0,62 | -0,61 |
| 6 | 2,758 | 1,406 | 0,724 | (-0,173; 2,984) | 1,353 | 1,78 | 1,99 |
| 7 | 2,494 | 2,882 | 0,871 | (0,985; 4,779) | -0,388 | -0,66 | -0,65 |
| 8 | 3,046 | 2,301 | 0,871 | (0,404; 4,198) | 0,745 | 1,27 | 1,31 |
| 9 | 0,481 | 1,864 | 0,800 | (0,122; 3,606) | -1,383 | -2,04 | -2,41 |
| 10 | 1,653 | 1,060 | 0,800 | (-0,683; 2,802) | 0,593 | 0,87 | 0,86 |
| 11 | 1,111 | 1,476 | 0,685 | (-0,016; 2,968) | -0,365 | -0,46 | -0,44 |
| 12 | 0,921 | 1,331 | 0,685 | (-0,161; 2,822) | -0,410 | -0,52 | -0,50 |
| 13 | 3,098 | 2,918 | 0,792 | (1,193; 4,643) | 0,180 | 0,26 | 0,25 |
| 14 | 0,759 | 1,174 | 0,792 | (-0,550; 2,899) | -0,416 | -0,60 | -0,59 |
| 15 | 2,012 | 2,375 | 0,691 | (0,870; 3,880) | -0,363 | -0,46 | -0,44 |
| 16 | 1,460 | 1,765 | 0,691 | (0,261; 3,270) | -0,305 | -0,39 | -0,37 |
| 17 | 4,254 | 3,167 | 0,811 | (1,399; 4,934) | 1,087 | 1,63 | 1,77 |
| 18 | 0,505 | 1,285 | 0,811 | (-0,483; 3,052) | -0,780 | -1,17 | -1,19 |
| 19 | 1,743 | 1,958 | 0,669 | (0,499; 3,416) | -0,215 | -0,27 | -0,26 |
| 20 | 1,868 | 1,692 | 0,844 | (-0,147; 3,531) | 0,176 | 0,28 | 0,27 |
| 21 | 2,226 | 1,536 | 0,670 | (0,076; 2,996) | 0,690 | 0,85 | 0,84 |
| 22 | 2,665 | 2,891 | 0,948 | (0,825; 4,957) | -0,226 | -0,50 | -0,49 |
| 23 | 2,006 | 2,488 | 0,952 | (0,413; 4,562) | -0,482 | -1,09 | -1,10 |
| 24 | 1,921 | 1,212 | 0,886 | (-0,719; 3,144) | 0,708 | 1,26 | 1,30 |
| 25 | 1,170 | 1,842 | 0,564 | (0,613; 3,070) | -0,672 | -0,76 | -0,74 |
| 26 | 3,564 | 1,842 | 0,564 | (0,613; 3,070) | 1,722 | 1,95 | 2,25 |
| 27 | 1,063 | 1,842 | 0,564 | (0,613; 3,070) | -0,779 | -0,88 | -0,87 |

| Obs | HI | Cook’s D | DFITS |  |
| --- | --- | --- | --- | --- |
| 1 | 0,655994 | 0,06 | 0,95862 |  |
| 2 | 0,655994 | 0,03 | -0,67485 |  |
| 3 | 0,729945 | 0,01 | -0,36883 |  |
| 4 | 0,729945 | 0,00 | 0,00626 |  |
| 5 | 0,476813 | 0,02 | -0,57846 |  |
| 6 | 0,476813 | 0,19 | 1,89988 |  |
| 7 | 0,688750 | 0,06 | -0,96204 |  |
| 8 | 0,688750 | 0,24 | 1,95006 |  |
| 9 | 0,580949 | 0,38 | -2,83736 | R |
| 10 | 0,580949 | 0,07 | 1,01691 |  |
| 11 | 0,425781 | 0,01 | -0,38213 |  |
| 12 | 0,425781 | 0,01 | -0,42979 |  |
| 13 | 0,569258 | 0,01 | 0,28871 |  |
| 14 | 0,569258 | 0,03 | -0,67469 |  |
| 15 | 0,433227 | 0,01 | -0,38799 |  |
| 16 | 0,433227 | 0,01 | -0,32518 |  |
| 17 | 0,597775 | 0,26 | 2,16308 |  |
| 18 | 0,597775 | 0,14 | -1,45340 |  |
| 19 | 0,407038 | 0,00 | -0,21170 |  |
| 20 | 0,647412 | 0,01 | 0,36809 |  |
| 21 | 0,407933 | 0,03 | 0,70087 |  |
| 22 | 0,817044 | 0,08 | -1,03034 |  |
| 23 | 0,823434 | 0,37 | -2,38215 |  |
| 24 | 0,713775 | 0,26 | 2,04870 |  |
| 25 | 0,288793 | 0,02 | -0,47459 |  |
| 26 | 0,288793 | 0,10 | 1,43524 |  |
| 27 | 0,288793 | 0,02 | -0,55535 |  |

R  Large residual

## Coded Coefficients

| Term | Coef | SE Coef | 95% CI | T-Value | P-Value | VIF |
| --- | --- | --- | --- | --- | --- | --- |
| Constant | 1,671 | 0,572 | (0,425; 2,917) | 2,92 | 0,013 |  |
| Lac | -0,938 | 0,473 | (-1,968; 0,092) | -1,98 | 0,071 | 1,18 |
| HPMC\_Visc | -0,094 | 0,494 | (-1,170; 0,983) | -0,19 | 0,853 | 1,70 |
| HPMC\_HP | 0,760 | 0,483 | (-0,293; 1,813) | 1,57 | 0,142 | 1,26 |
| HPMC\_PS | -0,382 | 0,741 | (-1,998; 1,233) | -0,52 | 0,615 | 2,09 |
| Lac\*Lac | 0,396 | 0,940 | (-1,651; 2,444) | 0,42 | 0,681 | 1,30 |
| HPMC\_Visc\*HPMC\_Visc | -0,096 | 0,999 | (-2,273; 2,080) | -0,10 | 0,925 | 1,96 |
| HPMC\_HP\*HPMC\_HP | 0,122 | 0,948 | (-1,943; 2,187) | 0,13 | 0,900 | 1,81 |
| HPMC\_PS\*HPMC\_PS | 0,354 | 0,942 | (-1,699; 2,406) | 0,38 | 0,714 | 1,42 |
| Lac\*HPMC\_Visc | 0,46 | 1,04 | (-1,80; 2,71) | 0,44 | 0,668 | 1,49 |
| Lac\*HPMC\_HP | -0,74 | 1,09 | (-3,11; 1,63) | -0,68 | 0,509 | 1,17 |
| Lac\*HPMC\_PS | -0,16 | 1,68 | (-3,82; 3,50) | -0,10 | 0,925 | 1,42 |
| HPMC\_Visc\*HPMC\_HP | 0,55 | 1,24 | (-2,15; 3,24) | 0,44 | 0,666 | 2,73 |
| HPMC\_Visc\*HPMC\_PS | 0,31 | 1,75 | (-3,50; 4,11) | 0,18 | 0,864 | 2,74 |
| HPMC\_HP\*HPMC\_PS | 0,65 | 1,93 | (-3,55; 4,85) | 0,34 | 0,742 | 2,70 |

## Model Summary

| S | R-sq | R-sq(adj) | PRESS | R-sq(pred) | AICc | BIC |
| --- | --- | --- | --- | --- | --- | --- |
| 1,06460 | 41,82% | 0,00% | 68,6654 | 0,00% | 144,51 | 110,84 |

## Analysis of Variance

| Source | DF | Seq SS | Contribution | Adj SS | Adj MS | F-Value | P-Value |
| --- | --- | --- | --- | --- | --- | --- | --- |
| Model | 14 | 9,7752 | 41,82% | 9,7752 | 0,69823 | 0,62 | 0,808 |
| Linear | 4 | 8,1198 | 34,74% | 7,6646 | 1,91614 | 1,69 | 0,216 |
| Lac | 1 | 4,8671 | 20,82% | 4,4602 | 4,46024 | 3,94 | 0,071 |
| HPMC\_Visc | 1 | 0,0169 | 0,07% | 0,0406 | 0,04064 | 0,04 | 0,853 |
| HPMC\_HP | 1 | 2,1221 | 9,08% | 2,8003 | 2,80028 | 2,47 | 0,142 |
| HPMC\_PS | 1 | 1,1138 | 4,76% | 0,3014 | 0,30142 | 0,27 | 0,615 |
| Square | 4 | 0,3865 | 1,65% | 0,4021 | 0,10052 | 0,09 | 0,984 |
| Lac\*Lac | 1 | 0,1598 | 0,68% | 0,2016 | 0,20163 | 0,18 | 0,681 |
| HPMC\_Visc\*HPMC\_Visc | 1 | 0,0932 | 0,40% | 0,0106 | 0,01056 | 0,01 | 0,925 |
| HPMC\_HP\*HPMC\_HP | 1 | 0,0357 | 0,15% | 0,0187 | 0,01868 | 0,02 | 0,900 |
| HPMC\_PS\*HPMC\_PS | 1 | 0,0978 | 0,42% | 0,1597 | 0,15970 | 0,14 | 0,714 |
| 2-Way Interaction | 6 | 1,2688 | 5,43% | 1,2688 | 0,21147 | 0,19 | 0,975 |
| Lac\*HPMC\_Visc | 1 | 0,3138 | 1,34% | 0,2195 | 0,21949 | 0,19 | 0,668 |
| Lac\*HPMC\_HP | 1 | 0,5958 | 2,55% | 0,5261 | 0,52614 | 0,46 | 0,509 |
| Lac\*HPMC\_PS | 1 | 0,0103 | 0,04% | 0,0103 | 0,01034 | 0,01 | 0,925 |
| HPMC\_Visc\*HPMC\_HP | 1 | 0,1624 | 0,69% | 0,2223 | 0,22230 | 0,20 | 0,666 |
| HPMC\_Visc\*HPMC\_PS | 1 | 0,0581 | 0,25% | 0,0348 | 0,03482 | 0,03 | 0,864 |
| HPMC\_HP\*HPMC\_PS | 1 | 0,1285 | 0,55% | 0,1285 | 0,12852 | 0,11 | 0,742 |
| Error | 12 | 13,6004 | 58,18% | 13,6004 | 1,13337 |  |  |
| Lack-of-Fit | 10 | 9,6457 | 41,26% | 9,6457 | 0,96457 | 0,49 | 0,821 |
| Pure Error | 2 | 3,9548 | 16,92% | 3,9548 | 1,97738 |  |  |
| Total | 26 | 23,3756 | 100,00% |  |  |  |  |

## Regression Equation in Uncoded Units

|  |  |  |
| --- | --- | --- |
| F\_SD\_21h(1260min) | = | 115 + 17,3 Lac - 0,00215 HPMC\_Visc - 8,0 HPMC\_HP - 1,89 HPMC\_PS + 6,3 Lac\*Lac - 0,000000 HPMC\_Visc\*HPMC\_Visc + 0,118 HPMC\_HP\*HPMC\_HP + 0,0065 HPMC\_PS\*HPMC\_PS + 0,00047 Lac\*HPMC\_Visc - 2,92 Lac\*HPMC\_HP - 0,087 Lac\*HPMC\_PS + 0,000139 HPMC\_Visc\*HPMC\_HP + 0,000011 HPMC\_Visc\*HPMC\_PS + 0,087 HPMC\_HP\*HPMC\_PS |

## Fits and Diagnostics for All Observations

| Obs | F\_SD\_21h(1260min) | Fit | SE Fit | 95% CI | Resid | Std Resid | Del Resid |
| --- | --- | --- | --- | --- | --- | --- | --- |
| 1 | 2,746 | 2,215 | 0,862 | (0,336; 4,093) | 0,531 | 0,85 | 0,84 |
| 2 | 1,015 | 1,526 | 0,862 | (-0,353; 3,405) | -0,511 | -0,82 | -0,81 |
| 3 | 1,008 | 1,360 | 0,910 | (-0,622; 3,341) | -0,352 | -0,64 | -0,62 |
| 4 | 1,357 | 1,386 | 0,910 | (-0,596; 3,368) | -0,029 | -0,05 | -0,05 |
| 5 | 2,259 | 2,645 | 0,735 | (1,043; 4,246) | -0,385 | -0,50 | -0,48 |
| 6 | 2,507 | 1,286 | 0,735 | (-0,316; 2,888) | 1,221 | 1,59 | 1,71 |
| 7 | 2,232 | 2,677 | 0,884 | (0,752; 4,602) | -0,445 | -0,75 | -0,74 |
| 8 | 2,497 | 1,774 | 0,884 | (-0,152; 3,699) | 0,723 | 1,22 | 1,25 |
| 9 | 0,220 | 1,625 | 0,811 | (-0,143; 3,393) | -1,405 | -2,04 | -2,42 |
| 10 | 1,523 | 0,809 | 0,811 | (-0,959; 2,577) | 0,714 | 1,04 | 1,04 |
| 11 | 1,144 | 1,513 | 0,695 | (-0,000; 3,027) | -0,369 | -0,46 | -0,44 |
| 12 | 0,428 | 0,981 | 0,695 | (-0,533; 2,495) | -0,553 | -0,69 | -0,67 |
| 13 | 2,934 | 2,707 | 0,803 | (0,957; 4,457) | 0,227 | 0,32 | 0,31 |
| 14 | 0,928 | 1,063 | 0,803 | (-0,687; 2,813) | -0,134 | -0,19 | -0,18 |
| 15 | 2,175 | 2,363 | 0,701 | (0,836; 3,890) | -0,188 | -0,24 | -0,23 |
| 16 | 0,804 | 1,383 | 0,701 | (-0,144; 2,910) | -0,579 | -0,72 | -0,71 |
| 17 | 4,042 | 3,041 | 0,823 | (1,247; 4,834) | 1,001 | 1,48 | 1,57 |
| 18 | 0,468 | 1,085 | 0,823 | (-0,708; 2,878) | -0,617 | -0,91 | -0,91 |
| 19 | 1,118 | 1,587 | 0,679 | (0,108; 3,067) | -0,470 | -0,57 | -0,56 |
| 20 | 1,751 | 1,292 | 0,857 | (-0,574; 3,159) | 0,459 | 0,73 | 0,71 |
| 21 | 2,187 | 1,263 | 0,680 | (-0,218; 2,745) | 0,924 | 1,13 | 1,14 |
| 22 | 2,203 | 2,512 | 0,962 | (0,415; 4,609) | -0,309 | -0,68 | -0,66 |
| 23 | 2,164 | 2,507 | 0,966 | (0,402; 4,612) | -0,343 | -0,77 | -0,75 |
| 24 | 1,941 | 1,364 | 0,899 | (-0,596; 3,323) | 0,577 | 1,01 | 1,01 |
| 25 | 1,044 | 1,667 | 0,572 | (0,420; 2,913) | -0,622 | -0,69 | -0,68 |
| 26 | 3,392 | 1,667 | 0,572 | (0,420; 2,913) | 1,725 | 1,92 | 2,21 |
| 27 | 0,877 | 1,667 | 0,572 | (0,420; 2,913) | -0,789 | -0,88 | -0,87 |

| Obs | HI | Cook’s D | DFITS |  |
| --- | --- | --- | --- | --- |
| 1 | 0,655994 | 0,09 | 1,16061 |  |
| 2 | 0,655994 | 0,09 | -1,11443 |  |
| 3 | 0,729945 | 0,07 | -1,01772 |  |
| 4 | 0,729945 | 0,00 | -0,08232 |  |
| 5 | 0,476813 | 0,02 | -0,46237 |  |
| 6 | 0,476813 | 0,15 | 1,63021 |  |
| 7 | 0,688750 | 0,08 | -1,09347 |  |
| 8 | 0,688750 | 0,22 | 1,85260 |  |
| 9 | 0,580949 | 0,38 | -2,84418 | R |
| 10 | 0,580949 | 0,10 | 1,22348 |  |
| 11 | 0,425781 | 0,01 | -0,38012 |  |
| 12 | 0,425781 | 0,02 | -0,57688 |  |
| 13 | 0,569258 | 0,01 | 0,35917 |  |
| 14 | 0,569258 | 0,00 | -0,21213 |  |
| 15 | 0,433227 | 0,00 | -0,19731 |  |
| 16 | 0,433227 | 0,03 | -0,61872 |  |
| 17 | 0,597775 | 0,22 | 1,91535 |  |
| 18 | 0,597775 | 0,08 | -1,10638 |  |
| 19 | 0,407038 | 0,02 | -0,46081 |  |
| 20 | 0,647412 | 0,06 | 0,96376 |  |
| 21 | 0,407933 | 0,06 | 0,94815 |  |
| 22 | 0,817044 | 0,14 | -1,40156 |  |
| 23 | 0,823434 | 0,18 | -1,62798 |  |
| 24 | 0,713775 | 0,17 | 1,60268 |  |
| 25 | 0,288793 | 0,01 | -0,43149 |  |
| 26 | 0,288793 | 0,10 | 1,40934 |  |
| 27 | 0,288793 | 0,02 | -0,55464 |  |

R  Large residual

## Coded Coefficients

| Term | Coef | SE Coef | 95% CI | T-Value | P-Value | VIF |
| --- | --- | --- | --- | --- | --- | --- |
| Constant | 1,507 | 0,548 | (0,313; 2,702) | 2,75 | 0,018 |  |
| Lac | -0,911 | 0,453 | (-1,899; 0,076) | -2,01 | 0,067 | 1,18 |
| HPMC\_Visc | -0,103 | 0,474 | (-1,135; 0,929) | -0,22 | 0,832 | 1,70 |
| HPMC\_HP | 0,615 | 0,463 | (-0,395; 1,625) | 1,33 | 0,209 | 1,26 |
| HPMC\_PS | -0,309 | 0,711 | (-1,858; 1,240) | -0,43 | 0,671 | 2,09 |
| Lac\*Lac | 0,524 | 0,901 | (-1,439; 2,487) | 0,58 | 0,572 | 1,30 |
| HPMC\_Visc\*HPMC\_Visc | -0,201 | 0,958 | (-2,287; 1,886) | -0,21 | 0,838 | 1,96 |
| HPMC\_HP\*HPMC\_HP | 0,161 | 0,909 | (-1,819; 2,141) | 0,18 | 0,862 | 1,81 |
| HPMC\_PS\*HPMC\_PS | 0,628 | 0,903 | (-1,340; 2,596) | 0,70 | 0,500 | 1,42 |
| Lac\*HPMC\_Visc | 0,147 | 0,994 | (-2,018; 2,313) | 0,15 | 0,885 | 1,49 |
| Lac\*HPMC\_HP | -0,95 | 1,04 | (-3,22; 1,32) | -0,91 | 0,381 | 1,17 |
| Lac\*HPMC\_PS | 0,02 | 1,61 | (-3,48; 3,53) | 0,02 | 0,988 | 1,42 |
| HPMC\_Visc\*HPMC\_HP | 0,35 | 1,19 | (-2,24; 2,93) | 0,29 | 0,774 | 2,73 |
| HPMC\_Visc\*HPMC\_PS | 0,43 | 1,68 | (-3,22; 4,08) | 0,26 | 0,802 | 2,74 |
| HPMC\_HP\*HPMC\_PS | 0,35 | 1,85 | (-3,68; 4,38) | 0,19 | 0,852 | 2,70 |

## Model Summary

| S | R-sq | R-sq(adj) | PRESS | R-sq(pred) | AICc | BIC |
| --- | --- | --- | --- | --- | --- | --- |
| 1,02073 | 39,67% | 0,00% | 61,6565 | 0,00% | 142,24 | 108,57 |

## Analysis of Variance

| Source | DF | Seq SS | Contribution | Adj SS | Adj MS | F-Value | P-Value |
| --- | --- | --- | --- | --- | --- | --- | --- |
| Model | 14 | 8,2208 | 39,67% | 8,2208 | 0,58720 | 0,56 | 0,848 |
| Linear | 4 | 6,0795 | 29,34% | 6,3050 | 1,57625 | 1,51 | 0,260 |
| Lac | 1 | 3,9023 | 18,83% | 4,2109 | 4,21091 | 4,04 | 0,067 |
| HPMC\_Visc | 1 | 0,0369 | 0,18% | 0,0492 | 0,04923 | 0,05 | 0,832 |
| HPMC\_HP | 1 | 1,4090 | 6,80% | 1,8330 | 1,83304 | 1,76 | 0,209 |
| HPMC\_PS | 1 | 0,7312 | 3,53% | 0,1970 | 0,19705 | 0,19 | 0,671 |
| Square | 4 | 0,9869 | 4,76% | 1,0370 | 0,25925 | 0,25 | 0,905 |
| Lac\*Lac | 1 | 0,2430 | 1,17% | 0,3522 | 0,35219 | 0,34 | 0,572 |
| HPMC\_Visc\*HPMC\_Visc | 1 | 0,3418 | 1,65% | 0,0458 | 0,04575 | 0,04 | 0,838 |
| HPMC\_HP\*HPMC\_HP | 1 | 0,0053 | 0,03% | 0,0328 | 0,03279 | 0,03 | 0,862 |
| HPMC\_PS\*HPMC\_PS | 1 | 0,3968 | 1,91% | 0,5037 | 0,50373 | 0,48 | 0,500 |
| 2-Way Interaction | 6 | 1,1544 | 5,57% | 1,1544 | 0,19241 | 0,18 | 0,976 |
| Lac\*HPMC\_Visc | 1 | 0,0107 | 0,05% | 0,0229 | 0,02291 | 0,02 | 0,885 |
| Lac\*HPMC\_HP | 1 | 0,9057 | 4,37% | 0,8611 | 0,86111 | 0,83 | 0,381 |
| Lac\*HPMC\_PS | 1 | 0,0002 | 0,00% | 0,0002 | 0,00024 | 0,00 | 0,988 |
| HPMC\_Visc\*HPMC\_HP | 1 | 0,1144 | 0,55% | 0,0895 | 0,08953 | 0,09 | 0,774 |
| HPMC\_Visc\*HPMC\_PS | 1 | 0,0857 | 0,41% | 0,0684 | 0,06836 | 0,07 | 0,802 |
| HPMC\_HP\*HPMC\_PS | 1 | 0,0378 | 0,18% | 0,0378 | 0,03781 | 0,04 | 0,852 |
| Error | 12 | 12,5027 | 60,33% | 12,5027 | 1,04189 |  |  |
| Lack-of-Fit | 10 | 8,9870 | 43,37% | 8,9870 | 0,89870 | 0,51 | 0,808 |
| Pure Error | 2 | 3,5157 | 16,96% | 3,5157 | 1,75784 |  |  |
| Total | 26 | 20,7235 | 100,00% |  |  |  |  |

## Regression Equation in Uncoded Units

|  |  |  |
| --- | --- | --- |
| F\_SD\_22h(1320min) | = | 111 + 20,4 Lac - 0,00162 HPMC\_Visc - 5,0 HPMC\_HP - 2,32 HPMC\_PS + 8,4 Lac\*Lac - 0,000000 HPMC\_Visc\*HPMC\_Visc + 0,156 HPMC\_HP\*HPMC\_HP + 0,0116 HPMC\_PS\*HPMC\_PS + 0,00015 Lac\*HPMC\_Visc - 3,74 Lac\*HPMC\_HP + 0,013 Lac\*HPMC\_PS + 0,000088 HPMC\_Visc\*HPMC\_HP + 0,000015 HPMC\_Visc\*HPMC\_PS + 0,047 HPMC\_HP\*HPMC\_PS |

## Fits and Diagnostics for All Observations

| Obs | F\_SD\_22h(1320min) | Fit | SE Fit | 95% CI | Resid | Std Resid | Del Resid |
| --- | --- | --- | --- | --- | --- | --- | --- |
| 1 | 2,285 | 1,852 | 0,827 | (0,051; 3,653) | 0,434 | 0,72 | 0,71 |
| 2 | 0,989 | 1,513 | 0,827 | (-0,288; 3,314) | -0,524 | -0,88 | -0,87 |
| 3 | 0,982 | 1,347 | 0,872 | (-0,553; 3,248) | -0,365 | -0,69 | -0,67 |
| 4 | 1,133 | 1,236 | 0,872 | (-0,664; 3,136) | -0,103 | -0,19 | -0,19 |
| 5 | 2,072 | 2,417 | 0,705 | (0,882; 3,953) | -0,345 | -0,47 | -0,45 |
| 6 | 2,392 | 1,211 | 0,705 | (-0,324; 2,747) | 1,181 | 1,60 | 1,73 |
| 7 | 2,087 | 2,574 | 0,847 | (0,729; 4,420) | -0,487 | -0,85 | -0,84 |
| 8 | 1,987 | 1,308 | 0,847 | (-0,538; 3,154) | 0,679 | 1,19 | 1,22 |
| 9 | 0,128 | 1,370 | 0,778 | (-0,325; 3,065) | -1,242 | -1,88 | -2,14 |
| 10 | 1,836 | 1,012 | 0,778 | (-0,683; 2,707) | 0,824 | 1,25 | 1,28 |
| 11 | 1,131 | 1,482 | 0,666 | (0,031; 2,933) | -0,351 | -0,45 | -0,44 |
| 12 | 0,507 | 1,055 | 0,666 | (-0,396; 2,506) | -0,548 | -0,71 | -0,69 |
| 13 | 2,784 | 2,412 | 0,770 | (0,734; 4,090) | 0,371 | 0,55 | 0,54 |
| 14 | 1,004 | 1,023 | 0,770 | (-0,655; 2,701) | -0,019 | -0,03 | -0,03 |
| 15 | 2,151 | 2,245 | 0,672 | (0,781; 3,709) | -0,094 | -0,12 | -0,12 |
| 16 | 0,788 | 1,218 | 0,672 | (-0,245; 2,682) | -0,431 | -0,56 | -0,54 |
| 17 | 3,830 | 2,918 | 0,789 | (1,198; 4,637) | 0,912 | 1,41 | 1,48 |
| 18 | 0,484 | 1,141 | 0,789 | (-0,579; 2,860) | -0,657 | -1,01 | -1,02 |
| 19 | 0,663 | 1,325 | 0,651 | (-0,094; 2,744) | -0,662 | -0,84 | -0,83 |
| 20 | 1,577 | 1,047 | 0,821 | (-0,743; 2,836) | 0,530 | 0,87 | 0,87 |
| 21 | 2,163 | 1,213 | 0,652 | (-0,207; 2,634) | 0,950 | 1,21 | 1,24 |
| 22 | 1,869 | 2,254 | 0,923 | (0,244; 4,264) | -0,385 | -0,88 | -0,87 |
| 23 | 2,373 | 2,557 | 0,926 | (0,539; 4,576) | -0,185 | -0,43 | -0,42 |
| 24 | 1,911 | 1,578 | 0,862 | (-0,301; 3,457) | 0,333 | 0,61 | 0,59 |
| 25 | 0,917 | 1,505 | 0,549 | (0,310; 2,701) | -0,588 | -0,68 | -0,67 |
| 26 | 3,092 | 1,505 | 0,549 | (0,310; 2,701) | 1,587 | 1,84 | 2,08 |
| 27 | 0,691 | 1,505 | 0,549 | (0,310; 2,701) | -0,814 | -0,95 | -0,94 |

| Obs | HI | Cook’s D | DFITS |
| --- | --- | --- | --- |
| 1 | 0,655994 | 0,07 | 0,97899 |
| 2 | 0,655994 | 0,10 | -1,19613 |
| 3 | 0,729945 | 0,09 | -1,10621 |
| 4 | 0,729945 | 0,01 | -0,30545 |
| 5 | 0,476813 | 0,01 | -0,43092 |
| 6 | 0,476813 | 0,16 | 1,64760 |
| 7 | 0,688750 | 0,11 | -1,25645 |
| 8 | 0,688750 | 0,21 | 1,80910 |
| 9 | 0,580949 | 0,33 | -2,52383 |
| 10 | 0,580949 | 0,14 | 1,50669 |
| 11 | 0,425781 | 0,01 | -0,37733 |
| 12 | 0,425781 | 0,02 | -0,59674 |
| 13 | 0,569258 | 0,03 | 0,61778 |
| 14 | 0,569258 | 0,00 | -0,03165 |
| 15 | 0,433227 | 0,00 | -0,10248 |
| 16 | 0,433227 | 0,02 | -0,47561 |
| 17 | 0,597775 | 0,20 | 1,80053 |
| 18 | 0,597775 | 0,10 | -1,23891 |
| 19 | 0,407038 | 0,03 | -0,68883 |
| 20 | 0,647412 | 0,09 | 1,17289 |
| 21 | 0,407933 | 0,07 | 1,02609 |
| 22 | 0,817044 | 0,23 | -1,84639 |
| 23 | 0,823434 | 0,06 | -0,89714 |
| 24 | 0,713775 | 0,06 | 0,93566 |
| 25 | 0,288793 | 0,01 | -0,42510 |
| 26 | 0,288793 | 0,09 | 1,32850 |
| 27 | 0,288793 | 0,02 | -0,59985 |

## Coded Coefficients

| Term | Coef | SE Coef | 95% CI | T-Value | P-Value | VIF |
| --- | --- | --- | --- | --- | --- | --- |
| Constant | 1,334 | 0,497 | (0,251; 2,418) | 2,68 | 0,020 |  |
| Lac | -0,847 | 0,411 | (-1,743; 0,049) | -2,06 | 0,062 | 1,18 |
| HPMC\_Visc | -0,109 | 0,430 | (-1,045; 0,827) | -0,25 | 0,804 | 1,70 |
| HPMC\_HP | 0,403 | 0,420 | (-0,512; 1,319) | 0,96 | 0,356 | 1,26 |
| HPMC\_PS | -0,252 | 0,645 | (-1,656; 1,152) | -0,39 | 0,703 | 2,09 |
| Lac\*Lac | 0,688 | 0,817 | (-1,093; 2,468) | 0,84 | 0,416 | 1,30 |
| HPMC\_Visc\*HPMC\_Visc | -0,155 | 0,868 | (-2,047; 1,737) | -0,18 | 0,861 | 1,96 |
| HPMC\_HP\*HPMC\_HP | 0,285 | 0,824 | (-1,511; 2,080) | 0,35 | 0,736 | 1,81 |
| HPMC\_PS\*HPMC\_PS | 0,923 | 0,819 | (-0,862; 2,707) | 1,13 | 0,282 | 1,42 |
| Lac\*HPMC\_Visc | 0,087 | 0,901 | (-1,876; 2,051) | 0,10 | 0,924 | 1,49 |
| Lac\*HPMC\_HP | -1,015 | 0,946 | (-3,076; 1,046) | -1,07 | 0,304 | 1,17 |
| Lac\*HPMC\_PS | 0,64 | 1,46 | (-2,54; 3,82) | 0,44 | 0,671 | 1,42 |
| HPMC\_Visc\*HPMC\_HP | -0,02 | 1,08 | (-2,36; 2,32) | -0,02 | 0,986 | 2,73 |
| HPMC\_Visc\*HPMC\_PS | 0,71 | 1,52 | (-2,60; 4,02) | 0,47 | 0,647 | 2,74 |
| HPMC\_HP\*HPMC\_PS | -0,04 | 1,68 | (-3,69; 3,61) | -0,02 | 0,981 | 2,70 |

## Model Summary

| S | R-sq | R-sq(adj) | PRESS | R-sq(pred) | AICc | BIC |
| --- | --- | --- | --- | --- | --- | --- |
| 0,925640 | 41,89% | 0,00% | 49,1221 | 0,00% | 136,95 | 103,29 |

## Analysis of Variance

| Source | DF | Seq SS | Contribution | Adj SS | Adj MS | F-Value | P-Value |
| --- | --- | --- | --- | --- | --- | --- | --- |
| Model | 14 | 7,4112 | 41,89% | 7,4112 | 0,52937 | 0,62 | 0,806 |
| Linear | 4 | 4,3824 | 24,77% | 4,5928 | 1,14819 | 1,34 | 0,311 |
| Lac | 1 | 3,2024 | 18,10% | 3,6344 | 3,63443 | 4,24 | 0,062 |
| HPMC\_Visc | 1 | 0,0504 | 0,28% | 0,0554 | 0,05542 | 0,06 | 0,804 |
| HPMC\_HP | 1 | 0,5566 | 3,15% | 0,7888 | 0,78878 | 0,92 | 0,356 |
| HPMC\_PS | 1 | 0,5729 | 3,24% | 0,1309 | 0,13094 | 0,15 | 0,703 |
| Square | 4 | 1,7587 | 9,94% | 1,8201 | 0,45504 | 0,53 | 0,715 |
| Lac\*Lac | 1 | 0,3246 | 1,83% | 0,6070 | 0,60703 | 0,71 | 0,416 |
| HPMC\_Visc\*HPMC\_Visc | 1 | 0,5531 | 3,13% | 0,0273 | 0,02732 | 0,03 | 0,861 |
| HPMC\_HP\*HPMC\_HP | 1 | 0,0000 | 0,00% | 0,1024 | 0,10239 | 0,12 | 0,736 |
| HPMC\_PS\*HPMC\_PS | 1 | 0,8810 | 4,98% | 1,0871 | 1,08714 | 1,27 | 0,282 |
| 2-Way Interaction | 6 | 1,2701 | 7,18% | 1,2701 | 0,21168 | 0,25 | 0,951 |
| Lac\*HPMC\_Visc | 1 | 0,0421 | 0,24% | 0,0080 | 0,00804 | 0,01 | 0,924 |
| Lac\*HPMC\_HP | 1 | 0,8536 | 4,82% | 0,9869 | 0,98687 | 1,15 | 0,304 |
| Lac\*HPMC\_PS | 1 | 0,1627 | 0,92% | 0,1627 | 0,16269 | 0,19 | 0,671 |
| HPMC\_Visc\*HPMC\_HP | 1 | 0,0206 | 0,12% | 0,0003 | 0,00026 | 0,00 | 0,986 |
| HPMC\_Visc\*HPMC\_PS | 1 | 0,1905 | 1,08% | 0,1894 | 0,18936 | 0,22 | 0,647 |
| HPMC\_HP\*HPMC\_PS | 1 | 0,0005 | 0,00% | 0,0005 | 0,00050 | 0,00 | 0,981 |
| Error | 12 | 10,2817 | 58,11% | 10,2817 | 0,85681 |  |  |
| Lack-of-Fit | 10 | 7,3889 | 41,76% | 7,3889 | 0,73889 | 0,51 | 0,808 |
| Pure Error | 2 | 2,8928 | 16,35% | 2,8928 | 1,44641 |  |  |
| Total | 26 | 17,6929 | 100,00% |  |  |  |  |

## Regression Equation in Uncoded Units

|  |  |  |
| --- | --- | --- |
| F\_SD\_23h(1380min) | = | 123 - 1,8 Lac - 0,00149 HPMC\_Visc - 2,4 HPMC\_HP - 2,87 HPMC\_PS + 11,0 Lac\*Lac - 0,000000 HPMC\_Visc\*HPMC\_Visc + 0,277 HPMC\_HP\*HPMC\_HP + 0,0171 HPMC\_PS\*HPMC\_PS + 0,000090 Lac\*HPMC\_Visc - 4,00 Lac\*HPMC\_HP + 0,346 Lac\*HPMC\_PS - 0,000005 HPMC\_Visc\*HPMC\_HP + 0,000025 HPMC\_Visc\*HPMC\_PS - 0,005 HPMC\_HP\*HPMC\_PS |

## Fits and Diagnostics for All Observations

| Obs | F\_SD\_23h(1380min) | Fit | SE Fit | 95% CI | Resid | Std Resid | Del Resid |
| --- | --- | --- | --- | --- | --- | --- | --- |
| 1 | 2,044 | 1,758 | 0,750 | (0,124; 3,391) | 0,286 | 0,53 | 0,51 |
| 2 | 0,982 | 1,423 | 0,750 | (-0,210; 3,057) | -0,442 | -0,81 | -0,80 |
| 3 | 1,279 | 1,573 | 0,791 | (-0,150; 3,296) | -0,294 | -0,61 | -0,59 |
| 4 | 1,058 | 1,181 | 0,791 | (-0,542; 2,904) | -0,123 | -0,26 | -0,25 |
| 5 | 2,050 | 2,371 | 0,639 | (0,979; 3,764) | -0,321 | -0,48 | -0,46 |
| 6 | 2,357 | 1,150 | 0,639 | (-0,242; 2,543) | 1,207 | 1,80 | 2,02 |
| 7 | 2,012 | 2,405 | 0,768 | (0,731; 4,079) | -0,393 | -0,76 | -0,75 |
| 8 | 1,412 | 0,890 | 0,768 | (-0,783; 2,564) | 0,521 | 1,01 | 1,01 |
| 9 | 0,238 | 1,193 | 0,706 | (-0,344; 2,730) | -0,955 | -1,59 | -1,72 |
| 10 | 2,053 | 1,240 | 0,706 | (-0,297; 2,778) | 0,813 | 1,36 | 1,41 |
| 11 | 1,179 | 1,481 | 0,604 | (0,165; 2,797) | -0,302 | -0,43 | -0,42 |
| 12 | 0,755 | 1,268 | 0,604 | (-0,048; 2,584) | -0,513 | -0,73 | -0,72 |
| 13 | 2,569 | 2,070 | 0,698 | (0,548; 3,591) | 0,499 | 0,82 | 0,81 |
| 14 | 1,035 | 1,078 | 0,698 | (-0,444; 2,600) | -0,043 | -0,07 | -0,07 |
| 15 | 1,866 | 2,004 | 0,609 | (0,677; 3,332) | -0,139 | -0,20 | -0,19 |
| 16 | 1,138 | 1,177 | 0,609 | (-0,150; 2,504) | -0,039 | -0,06 | -0,05 |
| 17 | 3,641 | 2,861 | 0,716 | (1,301; 4,420) | 0,780 | 1,33 | 1,38 |
| 18 | 0,480 | 1,200 | 0,716 | (-0,359; 2,760) | -0,720 | -1,23 | -1,26 |
| 19 | 0,418 | 1,177 | 0,591 | (-0,109; 2,464) | -0,759 | -1,07 | -1,07 |
| 20 | 1,423 | 0,965 | 0,745 | (-0,658; 2,587) | 0,458 | 0,83 | 0,82 |
| 21 | 2,115 | 1,273 | 0,591 | (-0,015; 2,561) | 0,842 | 1,18 | 1,20 |
| 22 | 1,593 | 2,018 | 0,837 | (0,195; 3,841) | -0,424 | -1,07 | -1,08 |
| 23 | 2,596 | 2,681 | 0,840 | (0,851; 4,511) | -0,085 | -0,22 | -0,21 |
| 24 | 1,904 | 1,775 | 0,782 | (0,072; 3,479) | 0,128 | 0,26 | 0,25 |
| 25 | 0,766 | 1,343 | 0,497 | (0,259; 2,426) | -0,576 | -0,74 | -0,72 |
| 26 | 2,732 | 1,343 | 0,497 | (0,259; 2,426) | 1,390 | 1,78 | 1,99 |
| 27 | 0,549 | 1,343 | 0,497 | (0,259; 2,426) | -0,794 | -1,02 | -1,02 |

| Obs | HI | Cook’s D | DFITS |
| --- | --- | --- | --- |
| 1 | 0,655994 | 0,04 | 0,70441 |
| 2 | 0,655994 | 0,08 | -1,10637 |
| 3 | 0,729945 | 0,07 | -0,97713 |
| 4 | 0,729945 | 0,01 | -0,40412 |
| 5 | 0,476813 | 0,01 | -0,44251 |
| 6 | 0,476813 | 0,20 | 1,92906 |
| 7 | 0,688750 | 0,09 | -1,11162 |
| 8 | 0,688750 | 0,15 | 1,50284 |
| 9 | 0,580949 | 0,23 | -2,02444 |
| 10 | 0,580949 | 0,17 | 1,66119 |
| 11 | 0,425781 | 0,01 | -0,35803 |
| 12 | 0,425781 | 0,03 | -0,61741 |
| 13 | 0,569258 | 0,06 | 0,93033 |
| 14 | 0,569258 | 0,00 | -0,07770 |
| 15 | 0,433227 | 0,00 | -0,16693 |
| 16 | 0,433227 | 0,00 | -0,04711 |
| 17 | 0,597775 | 0,17 | 1,67905 |
| 18 | 0,597775 | 0,15 | -1,53081 |
| 19 | 0,407038 | 0,05 | -0,88796 |
| 20 | 0,647412 | 0,09 | 1,11498 |
| 21 | 0,407933 | 0,06 | 0,99918 |
| 22 | 0,817044 | 0,34 | -2,27994 |
| 23 | 0,823434 | 0,01 | -0,45204 |
| 24 | 0,713775 | 0,01 | 0,39218 |
| 25 | 0,288793 | 0,01 | -0,46112 |
| 26 | 0,288793 | 0,09 | 1,26600 |
| 27 | 0,288793 | 0,03 | -0,64875 |

## Coded Coefficients

| Term | Coef | SE Coef | 95% CI | T-Value | P-Value | VIF |
| --- | --- | --- | --- | --- | --- | --- |
| Constant | 1,167 | 0,461 | (0,163; 2,170) | 2,53 | 0,026 |  |
| Lac | -0,721 | 0,381 | (-1,551; 0,109) | -1,89 | 0,083 | 1,18 |
| HPMC\_Visc | -0,137 | 0,398 | (-1,004; 0,730) | -0,34 | 0,737 | 1,70 |
| HPMC\_HP | 0,235 | 0,389 | (-0,613; 1,084) | 0,60 | 0,557 | 1,26 |
| HPMC\_PS | -0,160 | 0,597 | (-1,461; 1,141) | -0,27 | 0,793 | 2,09 |
| Lac\*Lac | 0,841 | 0,757 | (-0,808; 2,490) | 1,11 | 0,288 | 1,30 |
| HPMC\_Visc\*HPMC\_Visc | -0,139 | 0,804 | (-1,892; 1,614) | -0,17 | 0,866 | 1,96 |
| HPMC\_HP\*HPMC\_HP | 0,437 | 0,763 | (-1,226; 2,100) | 0,57 | 0,577 | 1,81 |
| HPMC\_PS\*HPMC\_PS | 1,141 | 0,759 | (-0,512; 2,794) | 1,50 | 0,158 | 1,42 |
| Lac\*HPMC\_Visc | 0,031 | 0,835 | (-1,788; 1,850) | 0,04 | 0,971 | 1,49 |
| Lac\*HPMC\_HP | -0,982 | 0,876 | (-2,891; 0,927) | -1,12 | 0,284 | 1,17 |
| Lac\*HPMC\_PS | 1,06 | 1,35 | (-1,89; 4,01) | 0,78 | 0,449 | 1,42 |
| HPMC\_Visc\*HPMC\_HP | -0,299 | 0,996 | (-2,470; 1,872) | -0,30 | 0,769 | 2,73 |
| HPMC\_Visc\*HPMC\_PS | 0,89 | 1,41 | (-2,18; 3,96) | 0,63 | 0,539 | 2,74 |
| HPMC\_HP\*HPMC\_PS | -0,30 | 1,55 | (-3,68; 3,09) | -0,19 | 0,852 | 2,70 |

## Model Summary

| S | R-sq | R-sq(adj) | PRESS | R-sq(pred) | AICc | BIC |
| --- | --- | --- | --- | --- | --- | --- |
| 0,857365 | 44,30% | 0,00% | 42,5207 | 0,00% | 132,82 | 99,15 |

## Analysis of Variance

| Source | DF | Seq SS | Contribution | Adj SS | Adj MS | F-Value | P-Value |
| --- | --- | --- | --- | --- | --- | --- | --- |
| Model | 14 | 7,0147 | 44,30% | 7,01474 | 0,50105 | 0,68 | 0,755 |
| Linear | 4 | 2,7976 | 17,67% | 3,01584 | 0,75396 | 1,03 | 0,433 |
| Lac | 1 | 2,1998 | 13,89% | 2,63445 | 2,63445 | 3,58 | 0,083 |
| HPMC\_Visc | 1 | 0,1093 | 0,69% | 0,08665 | 0,08665 | 0,12 | 0,737 |
| HPMC\_HP | 1 | 0,1391 | 0,88% | 0,26880 | 0,26880 | 0,37 | 0,557 |
| HPMC\_PS | 1 | 0,3494 | 2,21% | 0,05290 | 0,05290 | 0,07 | 0,793 |
| Square | 4 | 2,5979 | 16,41% | 2,67137 | 0,66784 | 0,91 | 0,490 |
| Lac\*Lac | 1 | 0,4304 | 2,72% | 0,90822 | 0,90822 | 1,24 | 0,288 |
| HPMC\_Visc\*HPMC\_Visc | 1 | 0,7645 | 4,83% | 0,02200 | 0,02200 | 0,03 | 0,866 |
| HPMC\_HP\*HPMC\_HP | 1 | 0,0011 | 0,01% | 0,24099 | 0,24099 | 0,33 | 0,577 |
| HPMC\_PS\*HPMC\_PS | 1 | 1,4018 | 8,85% | 1,66325 | 1,66325 | 2,26 | 0,158 |
| 2-Way Interaction | 6 | 1,6192 | 10,23% | 1,61922 | 0,26987 | 0,37 | 0,886 |
| Lac\*HPMC\_Visc | 1 | 0,1858 | 1,17% | 0,00101 | 0,00101 | 0,00 | 0,971 |
| Lac\*HPMC\_HP | 1 | 0,6795 | 4,29% | 0,92287 | 0,92287 | 1,26 | 0,284 |
| Lac\*HPMC\_PS | 1 | 0,4510 | 2,85% | 0,45102 | 0,45102 | 0,61 | 0,449 |
| HPMC\_Visc\*HPMC\_HP | 1 | 0,0016 | 0,01% | 0,06612 | 0,06612 | 0,09 | 0,769 |
| HPMC\_Visc\*HPMC\_PS | 1 | 0,2748 | 1,74% | 0,29406 | 0,29406 | 0,40 | 0,539 |
| HPMC\_HP\*HPMC\_PS | 1 | 0,0266 | 0,17% | 0,02656 | 0,02656 | 0,04 | 0,852 |
| Error | 12 | 8,8209 | 55,70% | 8,82090 | 0,73508 |  |  |
| Lack-of-Fit | 10 | 6,7144 | 42,40% | 6,71439 | 0,67144 | 0,64 | 0,744 |
| Pure Error | 2 | 2,1065 | 13,30% | 2,10651 | 1,05326 |  |  |
| Total | 26 | 15,8356 | 100,00% |  |  |  |  |

## Regression Equation in Uncoded Units

|  |  |  |
| --- | --- | --- |
| F\_SD\_24h(1440min) | = | 140 - 20,2 Lac - 0,00125 HPMC\_Visc - 2,1 HPMC\_HP - 3,29 HPMC\_PS + 13,5 Lac\*Lac - 0,000000 HPMC\_Visc\*HPMC\_Visc + 0,424 HPMC\_HP\*HPMC\_HP + 0,0211 HPMC\_PS\*HPMC\_PS + 0,000032 Lac\*HPMC\_Visc - 3,87 Lac\*HPMC\_HP + 0,576 Lac\*HPMC\_PS - 0,000076 HPMC\_Visc\*HPMC\_HP + 0,000031 HPMC\_Visc\*HPMC\_PS - 0,040 HPMC\_HP\*HPMC\_PS |

## Fits and Diagnostics for All Observations

| Obs | F\_SD\_24h(1440min) | Fit | SE Fit | 95% CI | Resid | Std Resid | Del Resid |
| --- | --- | --- | --- | --- | --- | --- | --- |
| 1 | 1,825 | 1,670 | 0,694 | (0,157; 3,183) | 0,155 | 0,31 | 0,30 |
| 2 | 1,026 | 1,373 | 0,694 | (-0,140; 2,886) | -0,347 | -0,69 | -0,67 |
| 3 | 1,494 | 1,720 | 0,733 | (0,124; 3,316) | -0,226 | -0,51 | -0,49 |
| 4 | 1,025 | 1,146 | 0,733 | (-0,450; 2,742) | -0,120 | -0,27 | -0,26 |
| 5 | 1,906 | 2,247 | 0,592 | (0,957; 3,537) | -0,341 | -0,55 | -0,53 |
| 6 | 2,367 | 1,122 | 0,592 | (-0,168; 2,412) | 1,244 | 2,01 | 2,36 |
| 7 | 1,833 | 2,152 | 0,712 | (0,601; 3,702) | -0,319 | -0,67 | -0,65 |
| 8 | 0,989 | 0,574 | 0,712 | (-0,976; 2,125) | 0,415 | 0,87 | 0,86 |
| 9 | 0,346 | 1,082 | 0,653 | (-0,342; 2,505) | -0,735 | -1,32 | -1,37 |
| 10 | 2,241 | 1,446 | 0,653 | (0,022; 2,870) | 0,795 | 1,43 | 1,51 |
| 11 | 1,214 | 1,455 | 0,559 | (0,236; 2,674) | -0,241 | -0,37 | -0,36 |
| 12 | 0,987 | 1,430 | 0,559 | (0,211; 2,649) | -0,443 | -0,68 | -0,67 |
| 13 | 2,374 | 1,793 | 0,647 | (0,384; 3,203) | 0,580 | 1,03 | 1,03 |
| 14 | 1,133 | 1,201 | 0,647 | (-0,209; 2,610) | -0,068 | -0,12 | -0,12 |
| 15 | 1,566 | 1,758 | 0,564 | (0,528; 2,987) | -0,192 | -0,30 | -0,29 |
| 16 | 1,464 | 1,159 | 0,564 | (-0,070; 2,389) | 0,305 | 0,47 | 0,46 |
| 17 | 3,454 | 2,737 | 0,663 | (1,292; 4,181) | 0,717 | 1,32 | 1,37 |
| 18 | 0,483 | 1,316 | 0,663 | (-0,128; 2,760) | -0,833 | -1,53 | -1,64 |
| 19 | 0,254 | 1,041 | 0,547 | (-0,151; 2,233) | -0,788 | -1,19 | -1,22 |
| 20 | 1,178 | 0,828 | 0,690 | (-0,675; 2,331) | 0,350 | 0,69 | 0,67 |
| 21 | 2,065 | 1,350 | 0,548 | (0,157; 2,543) | 0,715 | 1,08 | 1,09 |
| 22 | 1,415 | 1,861 | 0,775 | (0,172; 3,549) | -0,446 | -1,22 | -1,24 |
| 23 | 2,669 | 2,683 | 0,778 | (0,988; 4,378) | -0,014 | -0,04 | -0,04 |
| 24 | 1,911 | 1,944 | 0,724 | (0,365; 3,522) | -0,033 | -0,07 | -0,07 |
| 25 | 0,649 | 1,185 | 0,461 | (0,181; 2,189) | -0,537 | -0,74 | -0,73 |
| 26 | 2,320 | 1,185 | 0,461 | (0,181; 2,189) | 1,135 | 1,57 | 1,69 |
| 27 | 0,453 | 1,185 | 0,461 | (0,181; 2,189) | -0,732 | -1,01 | -1,01 |

| Obs | HI | Cook’s D | DFITS |  |
| --- | --- | --- | --- | --- |
| 1 | 0,655994 | 0,01 | 0,40811 |  |
| 2 | 0,655994 | 0,06 | -0,93009 |  |
| 3 | 0,729945 | 0,05 | -0,80630 |  |
| 4 | 0,729945 | 0,01 | -0,42690 |  |
| 5 | 0,476813 | 0,02 | -0,50902 |  |
| 6 | 0,476813 | 0,24 | 2,24982 | R |
| 7 | 0,688750 | 0,07 | -0,96730 |  |
| 8 | 0,688750 | 0,11 | 1,27647 |  |
| 9 | 0,580949 | 0,16 | -1,61575 |  |
| 10 | 0,580949 | 0,19 | 1,77397 |  |
| 11 | 0,425781 | 0,01 | -0,30741 |  |
| 12 | 0,425781 | 0,02 | -0,57327 |  |
| 13 | 0,569258 | 0,09 | 1,18874 |  |
| 14 | 0,569258 | 0,00 | -0,13311 |  |
| 15 | 0,433227 | 0,00 | -0,24975 |  |
| 16 | 0,433227 | 0,01 | 0,39953 |  |
| 17 | 0,597775 | 0,17 | 1,66481 |  |
| 18 | 0,597775 | 0,23 | -1,99344 |  |
| 19 | 0,407038 | 0,07 | -1,00784 |  |
| 20 | 0,647412 | 0,06 | 0,91044 |  |
| 21 | 0,407933 | 0,05 | 0,90730 |  |
| 22 | 0,817044 | 0,44 | -2,62523 |  |
| 23 | 0,823434 | 0,00 | -0,07766 |  |
| 24 | 0,713775 | 0,00 | -0,10845 |  |
| 25 | 0,288793 | 0,01 | -0,46346 |  |
| 26 | 0,288793 | 0,07 | 1,07463 |  |
| 27 | 0,288793 | 0,03 | -0,64586 |  |

R  Large residual
