# Supplementary material for: Influence of Selected Hypromellose Functionality-Related Characteristics and Soluble/Insoluble Filler Ratio on Carvedilol Release from Matrix Tablets
Source: Pharmaceutics. 2025 Oct 21;17(10):1358. doi: 10.3390/pharmaceutics17101358 (PMC12566823; doi:10.3390/pharmaceutics17101358)
Supplement: Supplementary file 1 [file pharmaceutics-17-01358-s001.zip › Report_SD of Release Analysis_RSM(CCD)_Stepwise, Backward Elimination.htm]

# SD of Release Analysis, Response Surface Design (Central Composite Design), Stepwise Regression - Backward Elimination

## Backward Elimination of Terms

α to remove = 0,1

## Coded Coefficients

| Term | Coef | SE Coef | 95% CI | T-Value | P-Value | VIF |
| --- | --- | --- | --- | --- | --- | --- |
| Constant | 1,318 | 0,200 | (0,904; 1,732) | 6,60 | 0,000 |  |
| Lac | 1,034 | 0,323 | (0,364; 1,704) | 3,20 | 0,004 | 1,08 |
| HPMC\_Visc | -0,457 | 0,271 | (-1,019; 0,106) | -1,68 | 0,107 | 1,00 |
| Lac\*Lac | 1,166 | 0,591 | (-0,059; 2,391) | 1,97 | 0,061 | 1,00 |
| Lac\*HPMC\_Visc | -1,340 | 0,632 | (-2,650; -0,030) | -2,12 | 0,045 | 1,08 |

## Model Summary

| S | R-sq | R-sq(adj) | PRESS | R-sq(pred) | AICc | BIC |
| --- | --- | --- | --- | --- | --- | --- |
| 0,762443 | 54,69% | 46,45% | 19,1336 | 32,21% | 72,65 | 76,22 |

## Analysis of Variance

| Source | DF | Seq SS | Contribution | Adj SS | Adj MS | F-Value | P-Value |
| --- | --- | --- | --- | --- | --- | --- | --- |
| Model | 4 | 15,436 | 54,69% | 15,436 | 3,8590 | 6,64 | 0,001 |
| Linear | 2 | 10,554 | 37,39% | 7,604 | 3,8022 | 6,54 | 0,006 |
| Lac | 1 | 8,874 | 31,44% | 5,960 | 5,9595 | 10,25 | 0,004 |
| HPMC\_Visc | 1 | 1,680 | 5,95% | 1,645 | 1,6449 | 2,83 | 0,107 |
| Square | 1 | 2,267 | 8,03% | 2,267 | 2,2669 | 3,90 | 0,061 |
| Lac\*Lac | 1 | 2,267 | 8,03% | 2,267 | 2,2669 | 3,90 | 0,061 |
| 2-Way Interaction | 1 | 2,616 | 9,27% | 2,616 | 2,6155 | 4,50 | 0,045 |
| Lac\*HPMC\_Visc | 1 | 2,616 | 9,27% | 2,616 | 2,6155 | 4,50 | 0,045 |
| Error | 22 | 12,789 | 45,31% | 12,789 | 0,5813 |  |  |
| Lack-of-Fit | 20 | 10,190 | 36,10% | 10,190 | 0,5095 | 0,39 | 0,897 |
| Pure Error | 2 | 2,599 | 9,21% | 2,599 | 1,2993 |  |  |
| Total | 26 | 28,225 | 100,00% |  |  |  |  |

## Regression Equation in Uncoded Units

|  |  |  |
| --- | --- | --- |
| F\_SD\_0.17h(10min) | = | -3,81 + 4,1 Lac + 0,000571 HPMC\_Visc + 18,66 Lac\*Lac - 0,001378 Lac\*HPMC\_Visc |

## Fits and Diagnostics for All Observations

| Obs | F\_SD\_0.17h(10min) | Fit | SE Fit | 95% CI | Resid | Std Resid | Del Resid | HI |
| --- | --- | --- | --- | --- | --- | --- | --- | --- |
| 1 | 1,134 | 0,941 | 0,322 | (0,272; 1,609) | 0,194 | 0,28 | 0,27 | 0,178821 |
| 2 | 2,346 | 2,928 | 0,322 | (2,260; 3,597) | -0,583 | -0,84 | -0,84 | 0,178821 |
| 3 | 0,741 | 1,240 | 0,404 | (0,401; 2,079) | -0,499 | -0,77 | -0,76 | 0,281436 |
| 4 | 0,785 | 1,350 | 0,404 | (0,511; 2,189) | -0,565 | -0,87 | -0,87 | 0,281436 |
| 5 | 0,860 | 0,954 | 0,303 | (0,325; 1,583) | -0,094 | -0,13 | -0,13 | 0,158392 |
| 6 | 5,070 | 2,858 | 0,303 | (2,229; 3,487) | 2,212 | 3,16 | 4,18 | 0,158392 |
| 7 | 0,912 | 1,244 | 0,412 | (0,390; 2,098) | -0,332 | -0,52 | -0,51 | 0,291836 |
| 8 | 1,271 | 1,326 | 0,412 | (0,472; 2,180) | -0,056 | -0,09 | -0,08 | 0,291836 |
| 9 | 1,144 | 0,945 | 0,316 | (0,289; 1,601) | 0,199 | 0,29 | 0,28 | 0,172155 |
| 10 | 2,506 | 2,906 | 0,316 | (2,250; 3,562) | -0,401 | -0,58 | -0,57 | 0,172155 |
| 11 | 0,245 | 1,133 | 0,254 | (0,607; 1,659) | -0,888 | -1,24 | -1,25 | 0,110622 |
| 12 | 1,852 | 1,912 | 0,254 | (1,386; 2,438) | -0,060 | -0,08 | -0,08 | 0,110622 |
| 13 | 0,750 | 0,943 | 0,319 | (0,281; 1,605) | -0,193 | -0,28 | -0,27 | 0,175079 |
| 14 | 2,649 | 2,916 | 0,319 | (2,255; 3,578) | -0,268 | -0,39 | -0,38 | 0,175079 |
| 15 | 1,393 | 1,153 | 0,276 | (0,581; 1,725) | 0,240 | 0,34 | 0,33 | 0,130851 |
| 16 | 1,650 | 1,809 | 0,276 | (1,237; 2,380) | -0,158 | -0,22 | -0,22 | 0,130851 |
| 17 | 1,815 | 1,285 | 0,575 | (0,092; 2,477) | 0,530 | 1,06 | 1,06 | 0,568720 |
| 18 | 3,638 | 3,855 | 0,575 | (2,663; 5,048) | -0,217 | -0,43 | -0,43 | 0,568720 |
| 19 | 1,034 | 1,775 | 0,308 | (1,135; 2,414) | -0,741 | -1,06 | -1,07 | 0,163395 |
| 20 | 2,043 | 0,861 | 0,364 | (0,107; 1,615) | 1,182 | 1,76 | 1,86 | 0,227435 |
| 21 | 1,406 | 1,400 | 0,197 | (0,991; 1,810) | 0,005 | 0,01 | 0,01 | 0,067038 |
| 22 | 1,178 | 1,371 | 0,197 | (0,962; 1,779) | -0,192 | -0,26 | -0,26 | 0,066681 |
| 23 | 1,271 | 1,436 | 0,200 | (1,021; 1,852) | -0,166 | -0,23 | -0,22 | 0,068919 |
| 24 | 1,024 | 1,441 | 0,201 | (1,025; 1,857) | -0,417 | -0,57 | -0,56 | 0,069279 |
| 25 | 0,676 | 1,404 | 0,198 | (0,994; 1,813) | -0,727 | -0,99 | -0,99 | 0,067143 |
| 26 | 1,846 | 1,404 | 0,198 | (0,994; 1,813) | 0,442 | 0,60 | 0,59 | 0,067143 |
| 27 | 2,956 | 1,404 | 0,198 | (0,994; 1,813) | 1,552 | 2,11 | 2,31 | 0,067143 |

| Obs | Cook’s D | DFITS |  |  |
| --- | --- | --- | --- | --- |
| 1 | 0,00 | 0,12804 |  |  |
| 2 | 0,03 | -0,39079 |  |  |
| 3 | 0,05 | -0,47835 |  |  |
| 4 | 0,06 | -0,54404 |  |  |
| 5 | 0,00 | -0,05693 |  |  |
| 6 | 0,38 | 1,81479 | R |  |
| 7 | 0,02 | -0,32685 |  |  |
| 8 | 0,00 | -0,05429 |  |  |
| 9 | 0,00 | 0,12784 |  |  |
| 10 | 0,01 | -0,25925 |  |  |
| 11 | 0,04 | -0,44121 |  |  |
| 12 | 0,00 | -0,02880 |  |  |
| 13 | 0,00 | -0,12547 |  |  |
| 14 | 0,01 | -0,17451 |  |  |
| 15 | 0,00 | 0,12827 |  |  |
| 16 | 0,00 | -0,08460 |  |  |
| 17 | 0,30 | 1,21988 |  | X |
| 18 | 0,05 | -0,48940 |  | X |
| 19 | 0,04 | -0,47084 |  |  |
| 20 | 0,18 | 1,00875 |  |  |
| 21 | 0,00 | 0,00188 |  |  |
| 22 | 0,00 | -0,06834 |  |  |
| 23 | 0,00 | -0,05992 |  |  |
| 24 | 0,00 | -0,15212 |  |  |
| 25 | 0,01 | -0,26478 |  |  |
| 26 | 0,01 | 0,15869 |  |  |
| 27 | 0,06 | 0,61848 | R |  |

R  Large residual  
X  Unusual X

## Backward Elimination of Terms

α to remove = 0,1

## Coded Coefficients

| Term | Coef | SE Coef | 95% CI | T-Value | P-Value | VIF |
| --- | --- | --- | --- | --- | --- | --- |
| Constant | 1,524 | 0,206 | (1,097; 1,951) | 7,40 | 0,000 |  |
| Lac | 0,902 | 0,333 | (0,212; 1,592) | 2,71 | 0,013 | 1,08 |
| HPMC\_Visc | -0,545 | 0,280 | (-1,124; 0,035) | -1,95 | 0,064 | 1,00 |
| Lac\*Lac | 1,213 | 0,608 | (-0,049; 2,475) | 1,99 | 0,059 | 1,00 |
| Lac\*HPMC\_Visc | -1,424 | 0,651 | (-2,773; -0,074) | -2,19 | 0,040 | 1,08 |

## Model Summary

| S | R-sq | R-sq(adj) | PRESS | R-sq(pred) | AICc | BIC |
| --- | --- | --- | --- | --- | --- | --- |
| 0,785404 | 52,49% | 43,85% | 19,9130 | 30,28% | 74,25 | 77,82 |

## Analysis of Variance

| Source | DF | Seq SS | Contribution | Adj SS | Adj MS | F-Value | P-Value |
| --- | --- | --- | --- | --- | --- | --- | --- |
| Model | 4 | 14,992 | 52,49% | 14,992 | 3,7481 | 6,08 | 0,002 |
| Linear | 2 | 9,588 | 33,57% | 6,878 | 3,4389 | 5,57 | 0,011 |
| Lac | 1 | 7,204 | 25,22% | 4,537 | 4,5373 | 7,36 | 0,013 |
| HPMC\_Visc | 1 | 2,383 | 8,34% | 2,340 | 2,3404 | 3,79 | 0,064 |
| Square | 1 | 2,452 | 8,59% | 2,452 | 2,4524 | 3,98 | 0,059 |
| Lac\*Lac | 1 | 2,452 | 8,59% | 2,452 | 2,4524 | 3,98 | 0,059 |
| 2-Way Interaction | 1 | 2,952 | 10,34% | 2,952 | 2,9524 | 4,79 | 0,040 |
| Lac\*HPMC\_Visc | 1 | 2,952 | 10,34% | 2,952 | 2,9524 | 4,79 | 0,040 |
| Error | 22 | 13,571 | 47,51% | 13,571 | 0,6169 |  |  |
| Lack-of-Fit | 20 | 11,144 | 39,01% | 11,144 | 0,5572 | 0,46 | 0,861 |
| Pure Error | 2 | 2,427 | 8,50% | 2,427 | 1,2135 |  |  |
| Total | 26 | 28,563 | 100,00% |  |  |  |  |

## Regression Equation in Uncoded Units

|  |  |  |
| --- | --- | --- |
| F\_SD\_0.33h(20min) | = | -3,42 + 4,0 Lac + 0,000592 HPMC\_Visc + 19,41 Lac\*Lac - 0,001464 Lac\*HPMC\_Visc |

## Fits and Diagnostics for All Observations

| Obs | F\_SD\_0.33h(20min) | Fit | SE Fit | 95% CI | Resid | Std Resid | Del Resid | HI |
| --- | --- | --- | --- | --- | --- | --- | --- | --- |
| 1 | 1,310 | 1,257 | 0,332 | (0,568; 1,946) | 0,053 | 0,07 | 0,07 | 0,178821 |
| 2 | 2,624 | 3,172 | 0,332 | (2,484; 3,861) | -0,549 | -0,77 | -0,76 | 0,178821 |
| 3 | 0,977 | 1,491 | 0,417 | (0,627; 2,355) | -0,514 | -0,77 | -0,76 | 0,281436 |
| 4 | 0,852 | 1,412 | 0,417 | (0,548; 2,276) | -0,560 | -0,84 | -0,84 | 0,281436 |
| 5 | 1,255 | 1,267 | 0,313 | (0,619; 1,916) | -0,012 | -0,02 | -0,02 | 0,158392 |
| 6 | 5,302 | 3,094 | 0,313 | (2,445; 3,742) | 2,209 | 3,07 | 3,96 | 0,158392 |
| 7 | 1,054 | 1,495 | 0,424 | (0,615; 2,375) | -0,440 | -0,67 | -0,66 | 0,291836 |
| 8 | 1,174 | 1,386 | 0,424 | (0,506; 2,265) | -0,212 | -0,32 | -0,31 | 0,291836 |
| 9 | 1,561 | 1,260 | 0,326 | (0,584; 1,936) | 0,301 | 0,42 | 0,41 | 0,172155 |
| 10 | 2,614 | 3,148 | 0,326 | (2,472; 3,824) | -0,534 | -0,75 | -0,74 | 0,172155 |
| 11 | 0,419 | 1,408 | 0,261 | (0,866; 1,949) | -0,988 | -1,33 | -1,36 | 0,110622 |
| 12 | 2,099 | 2,038 | 0,261 | (1,497; 2,580) | 0,060 | 0,08 | 0,08 | 0,110622 |
| 13 | 1,208 | 1,259 | 0,329 | (0,577; 1,940) | -0,051 | -0,07 | -0,07 | 0,175079 |
| 14 | 2,965 | 3,159 | 0,329 | (2,477; 3,840) | -0,194 | -0,27 | -0,27 | 0,175079 |
| 15 | 1,951 | 1,423 | 0,284 | (0,834; 2,012) | 0,528 | 0,72 | 0,71 | 0,130851 |
| 16 | 1,714 | 1,923 | 0,284 | (1,334; 2,513) | -0,210 | -0,29 | -0,28 | 0,130851 |
| 17 | 2,092 | 1,670 | 0,592 | (0,441; 2,898) | 0,422 | 0,82 | 0,81 | 0,568720 |
| 18 | 3,864 | 4,008 | 0,592 | (2,780; 5,237) | -0,145 | -0,28 | -0,27 | 0,568720 |
| 19 | 1,408 | 2,068 | 0,317 | (1,410; 2,727) | -0,660 | -0,92 | -0,92 | 0,163395 |
| 20 | 2,367 | 0,979 | 0,375 | (0,202; 1,756) | 1,388 | 2,01 | 2,17 | 0,227435 |
| 21 | 1,467 | 1,622 | 0,203 | (1,200; 2,044) | -0,155 | -0,20 | -0,20 | 0,067038 |
| 22 | 1,483 | 1,587 | 0,203 | (1,166; 2,007) | -0,103 | -0,14 | -0,13 | 0,066681 |
| 23 | 1,413 | 1,665 | 0,206 | (1,237; 2,093) | -0,252 | -0,33 | -0,33 | 0,068919 |
| 24 | 1,115 | 1,671 | 0,207 | (1,242; 2,099) | -0,555 | -0,73 | -0,73 | 0,069279 |
| 25 | 0,866 | 1,626 | 0,204 | (1,204; 2,048) | -0,760 | -1,00 | -1,00 | 0,067143 |
| 26 | 2,122 | 1,626 | 0,204 | (1,204; 2,048) | 0,496 | 0,65 | 0,65 | 0,067143 |
| 27 | 3,062 | 1,626 | 0,204 | (1,204; 2,048) | 1,436 | 1,89 | 2,02 | 0,067143 |

| Obs | Cook’s D | DFITS |  |  |
| --- | --- | --- | --- | --- |
| 1 | 0,00 | 0,03411 |  |  |
| 2 | 0,03 | -0,35645 |  |  |
| 3 | 0,05 | -0,47831 |  |  |
| 4 | 0,06 | -0,52278 |  |  |
| 5 | 0,00 | -0,00719 |  |  |
| 6 | 0,35 | 1,71660 | R |  |
| 7 | 0,04 | -0,42206 |  |  |
| 8 | 0,01 | -0,20130 |  |  |
| 9 | 0,01 | 0,18846 |  |  |
| 10 | 0,02 | -0,33702 |  |  |
| 11 | 0,04 | -0,47966 |  |  |
| 12 | 0,00 | 0,02806 |  |  |
| 13 | 0,00 | -0,03206 |  |  |
| 14 | 0,00 | -0,12267 |  |  |
| 15 | 0,02 | 0,27677 |  |  |
| 16 | 0,00 | -0,10872 |  |  |
| 17 | 0,18 | 0,93335 |  | X |
| 18 | 0,02 | -0,31498 |  | X |
| 19 | 0,03 | -0,40462 |  |  |
| 20 | 0,24 | 1,17945 | R |  |
| 21 | 0,00 | -0,05341 |  |  |
| 22 | 0,00 | -0,03563 |  |  |
| 23 | 0,00 | -0,08876 |  |  |
| 24 | 0,01 | -0,19783 |  |  |
| 25 | 0,01 | -0,26872 |  |  |
| 26 | 0,01 | 0,17308 |  |  |
| 27 | 0,05 | 0,54226 |  |  |

R  Large residual  
X  Unusual X

## Backward Elimination of Terms

α to remove = 0,1

## Coded Coefficients

| Term | Coef | SE Coef | 95% CI | T-Value | P-Value | VIF |
| --- | --- | --- | --- | --- | --- | --- |
| Constant | 2,336 | 0,236 | (1,843; 2,829) | 9,91 | 0,000 |  |
| Lac | 0,893 | 0,343 | (0,175; 1,611) | 2,60 | 0,017 | 1,08 |
| HPMC\_Visc | -0,621 | 0,310 | (-1,269; 0,028) | -2,00 | 0,060 | 1,15 |
| HPMC\_HP | 0,116 | 0,351 | (-0,619; 0,850) | 0,33 | 0,745 | 1,15 |
| HPMC\_PS | -0,200 | 0,423 | (-1,086; 0,685) | -0,47 | 0,641 | 1,18 |
| HPMC\_HP\*HPMC\_HP | -1,023 | 0,576 | (-2,229; 0,182) | -1,78 | 0,092 | 1,15 |
| HPMC\_PS\*HPMC\_PS | -1,205 | 0,608 | (-2,478; 0,069) | -1,98 | 0,062 | 1,02 |
| Lac\*HPMC\_Visc | -1,432 | 0,671 | (-2,836; -0,027) | -2,13 | 0,046 | 1,08 |

## Model Summary

| S | R-sq | R-sq(adj) | PRESS | R-sq(pred) | AICc | BIC |
| --- | --- | --- | --- | --- | --- | --- |
| 0,810130 | 58,56% | 43,29% | 19,7295 | 34,43% | 84,35 | 85,43 |

## Analysis of Variance

| Source | DF | Seq SS | Contribution | Adj SS | Adj MS | F-Value | P-Value |
| --- | --- | --- | --- | --- | --- | --- | --- |
| Model | 7 | 17,6212 | 58,56% | 17,6212 | 2,51732 | 3,84 | 0,009 |
| Linear | 4 | 10,5204 | 34,96% | 7,1490 | 1,78725 | 2,72 | 0,060 |
| Lac | 1 | 7,1034 | 23,61% | 4,4494 | 4,44937 | 6,78 | 0,017 |
| HPMC\_Visc | 1 | 2,6774 | 8,90% | 2,6334 | 2,63337 | 4,01 | 0,060 |
| HPMC\_HP | 1 | 0,6764 | 2,25% | 0,0712 | 0,07120 | 0,11 | 0,745 |
| HPMC\_PS | 1 | 0,0632 | 0,21% | 0,1470 | 0,14704 | 0,22 | 0,641 |
| Square | 2 | 4,1151 | 13,68% | 4,1151 | 2,05753 | 3,13 | 0,067 |
| HPMC\_HP\*HPMC\_HP | 1 | 1,5415 | 5,12% | 2,0723 | 2,07229 | 3,16 | 0,092 |
| HPMC\_PS\*HPMC\_PS | 1 | 2,5735 | 8,55% | 2,5735 | 2,57351 | 3,92 | 0,062 |
| 2-Way Interaction | 1 | 2,9858 | 9,92% | 2,9858 | 2,98577 | 4,55 | 0,046 |
| Lac\*HPMC\_Visc | 1 | 2,9858 | 9,92% | 2,9858 | 2,98577 | 4,55 | 0,046 |
| Error | 19 | 12,4699 | 41,44% | 12,4699 | 0,65631 |  |  |
| Lack-of-Fit | 17 | 10,1337 | 33,68% | 10,1337 | 0,59610 | 0,51 | 0,829 |
| Pure Error | 2 | 2,3362 | 7,76% | 2,3362 | 1,16808 |  |  |
| Total | 26 | 30,0911 | 100,00% |  |  |  |  |

## Regression Equation in Uncoded Units

|  |  |  |
| --- | --- | --- |
| F\_SD\_0.5h(30min) | = | -203,6 + 23,46 Lac + 0,000576 HPMC\_Visc + 18,9 HPMC\_HP + 3,08 HPMC\_PS - 0,993 HPMC\_HP\*HPMC\_HP - 0,0223 HPMC\_PS\*HPMC\_PS - 0,001472 Lac\*HPMC\_Visc |

## Fits and Diagnostics for All Observations

| Obs | F\_SD\_0.5h(30min) | Fit | SE Fit | 95% CI | Resid | Std Resid | Del Resid | HI |
| --- | --- | --- | --- | --- | --- | --- | --- | --- |
| 1 | 1,373 | 1,182 | 0,426 | (0,290; 2,074) | 0,191 | 0,28 | 0,27 | 0,276595 |
| 2 | 2,735 | 3,094 | 0,426 | (2,203; 3,986) | -0,359 | -0,52 | -0,51 | 0,276595 |
| 3 | 1,066 | 1,002 | 0,514 | (-0,073; 2,077) | 0,064 | 0,10 | 0,10 | 0,402122 |
| 4 | 0,888 | 0,908 | 0,514 | (-0,167; 1,984) | -0,021 | -0,03 | -0,03 | 0,402122 |
| 5 | 1,512 | 1,807 | 0,389 | (0,992; 2,621) | -0,295 | -0,42 | -0,41 | 0,230817 |
| 6 | 5,374 | 3,630 | 0,389 | (2,815; 4,444) | 1,744 | 2,45 | 2,89 | 0,230817 |
| 7 | 1,170 | 1,676 | 0,487 | (0,657; 2,695) | -0,506 | -0,78 | -0,77 | 0,360983 |
| 8 | 1,178 | 1,553 | 0,487 | (0,534; 2,571) | -0,374 | -0,58 | -0,57 | 0,360983 |
| 9 | 1,738 | 0,981 | 0,412 | (0,119; 1,842) | 0,757 | 1,08 | 1,09 | 0,258085 |
| 10 | 2,574 | 2,865 | 0,412 | (2,004; 3,727) | -0,291 | -0,42 | -0,41 | 0,258085 |
| 11 | 0,359 | 1,525 | 0,340 | (0,814; 2,236) | -1,166 | -1,59 | -1,66 | 0,175870 |
| 12 | 2,121 | 2,145 | 0,340 | (1,434; 2,856) | -0,024 | -0,03 | -0,03 | 0,175870 |
| 13 | 1,384 | 1,269 | 0,418 | (0,395; 2,144) | 0,114 | 0,16 | 0,16 | 0,265836 |
| 14 | 3,249 | 3,166 | 0,418 | (2,292; 4,040) | 0,083 | 0,12 | 0,12 | 0,265836 |
| 15 | 2,159 | 1,766 | 0,355 | (1,023; 2,509) | 0,393 | 0,54 | 0,53 | 0,192197 |
| 16 | 1,865 | 2,255 | 0,355 | (1,512; 2,999) | -0,390 | -0,54 | -0,53 | 0,192197 |
| 17 | 2,180 | 1,286 | 0,407 | (0,435; 2,138) | 0,894 | 1,28 | 1,30 | 0,252354 |
| 18 | 4,096 | 3,610 | 0,407 | (2,759; 4,462) | 0,486 | 0,69 | 0,68 | 0,252354 |
| 19 | 1,429 | 2,913 | 0,343 | (2,195; 3,631) | -1,485 | -2,02 | -2,22 | 0,179243 |
| 20 | 2,438 | 1,669 | 0,408 | (0,816; 2,523) | 0,769 | 1,10 | 1,11 | 0,253359 |
| 21 | 1,537 | 1,317 | 0,469 | (0,336; 2,298) | 0,220 | 0,33 | 0,33 | 0,334695 |
| 22 | 1,468 | 1,485 | 0,690 | (0,041; 2,930) | -0,017 | -0,04 | -0,04 | 0,725396 |
| 23 | 1,412 | 1,478 | 0,695 | (0,024; 2,933) | -0,067 | -0,16 | -0,16 | 0,735668 |
| 24 | 1,115 | 1,048 | 0,673 | (-0,361; 2,457) | 0,067 | 0,15 | 0,15 | 0,690354 |
| 25 | 1,007 | 2,448 | 0,235 | (1,957; 2,939) | -1,441 | -1,86 | -2,00 | 0,083857 |
| 26 | 2,421 | 2,448 | 0,235 | (1,957; 2,939) | -0,027 | -0,04 | -0,03 | 0,083857 |
| 27 | 3,130 | 2,448 | 0,235 | (1,957; 2,939) | 0,681 | 0,88 | 0,87 | 0,083857 |

| Obs | Cook’s D | DFITS |  |
| --- | --- | --- | --- |
| 1 | 0,00 | 0,16715 |  |
| 2 | 0,01 | -0,31618 |  |
| 3 | 0,00 | 0,08119 |  |
| 4 | 0,00 | -0,02628 |  |
| 5 | 0,01 | -0,22238 |  |
| 6 | 0,23 | 1,58385 | R |
| 7 | 0,04 | -0,58094 |  |
| 8 | 0,02 | -0,42668 |  |
| 9 | 0,05 | 0,64283 |  |
| 10 | 0,01 | -0,24028 |  |
| 11 | 0,07 | -0,76522 |  |
| 12 | 0,00 | -0,01497 |  |
| 13 | 0,00 | 0,09655 |  |
| 14 | 0,00 | 0,06993 |  |
| 15 | 0,01 | 0,25829 |  |
| 16 | 0,01 | -0,25640 |  |
| 17 | 0,07 | 0,75441 |  |
| 18 | 0,02 | 0,39747 |  |
| 19 | 0,11 | -1,03861 | R |
| 20 | 0,05 | 0,64372 |  |
| 21 | 0,01 | 0,23082 |  |
| 22 | 0,00 | -0,06367 |  |
| 23 | 0,01 | -0,26070 |  |
| 24 | 0,01 | 0,21743 |  |
| 25 | 0,04 | -0,60517 |  |
| 26 | 0,00 | -0,01037 |  |
| 27 | 0,01 | 0,26422 |  |

R  Large residual

## Backward Elimination of Terms

α to remove = 0,1

## Coded Coefficients

| Term | Coef | SE Coef | 95% CI | T-Value | P-Value | VIF |
| --- | --- | --- | --- | --- | --- | --- |
| Constant | 2,483 | 0,251 | (1,958; 3,008) | 9,90 | 0,000 |  |
| Lac | 0,951 | 0,365 | (0,186; 1,716) | 2,60 | 0,017 | 1,08 |
| HPMC\_Visc | -0,605 | 0,330 | (-1,296; 0,086) | -1,83 | 0,082 | 1,15 |
| HPMC\_HP | 0,126 | 0,374 | (-0,656; 0,909) | 0,34 | 0,739 | 1,15 |
| HPMC\_PS | -0,202 | 0,451 | (-1,145; 0,742) | -0,45 | 0,660 | 1,18 |
| HPMC\_HP\*HPMC\_HP | -1,141 | 0,613 | (-2,425; 0,143) | -1,86 | 0,078 | 1,15 |
| HPMC\_PS\*HPMC\_PS | -1,333 | 0,648 | (-2,689; 0,023) | -2,06 | 0,054 | 1,02 |
| Lac\*HPMC\_Visc | -1,475 | 0,715 | (-2,971; 0,021) | -2,06 | 0,053 | 1,08 |

## Model Summary

| S | R-sq | R-sq(adj) | PRESS | R-sq(pred) | AICc | BIC |
| --- | --- | --- | --- | --- | --- | --- |
| 0,862823 | 58,14% | 42,72% | 22,3946 | 33,73% | 87,76 | 88,83 |

## Analysis of Variance

| Source | DF | Seq SS | Contribution | Adj SS | Adj MS | F-Value | P-Value |
| --- | --- | --- | --- | --- | --- | --- | --- |
| Model | 7 | 19,6467 | 58,14% | 19,6467 | 2,80667 | 3,77 | 0,010 |
| Linear | 4 | 11,4054 | 33,75% | 7,6145 | 1,90361 | 2,56 | 0,072 |
| Lac | 1 | 7,9624 | 23,56% | 5,0457 | 5,04567 | 6,78 | 0,017 |
| HPMC\_Visc | 1 | 2,5476 | 7,54% | 2,5020 | 2,50203 | 3,36 | 0,082 |
| HPMC\_HP | 1 | 0,8386 | 2,48% | 0,0850 | 0,08495 | 0,11 | 0,739 |
| HPMC\_PS | 1 | 0,0569 | 0,17% | 0,1489 | 0,14892 | 0,20 | 0,660 |
| Square | 2 | 5,0709 | 15,01% | 5,0709 | 2,53543 | 3,41 | 0,054 |
| HPMC\_HP\*HPMC\_HP | 1 | 1,9213 | 5,69% | 2,5761 | 2,57606 | 3,46 | 0,078 |
| HPMC\_PS\*HPMC\_PS | 1 | 3,1495 | 9,32% | 3,1495 | 3,14951 | 4,23 | 0,054 |
| 2-Way Interaction | 1 | 3,1704 | 9,38% | 3,1704 | 3,17039 | 4,26 | 0,053 |
| Lac\*HPMC\_Visc | 1 | 3,1704 | 9,38% | 3,1704 | 3,17039 | 4,26 | 0,053 |
| Error | 19 | 14,1448 | 41,86% | 14,1448 | 0,74446 |  |  |
| Lack-of-Fit | 17 | 11,2850 | 33,40% | 11,2850 | 0,66382 | 0,46 | 0,853 |
| Pure Error | 2 | 2,8598 | 8,46% | 2,8598 | 1,42991 |  |  |
| Total | 26 | 33,7915 | 100,00% |  |  |  |  |

## Regression Equation in Uncoded Units

|  |  |  |
| --- | --- | --- |
| F\_SD\_0.75h(45min) | = | -225,8 + 24,30 Lac + 0,000603 HPMC\_Visc + 21,1 HPMC\_HP + 3,40 HPMC\_PS - 1,107 HPMC\_HP\*HPMC\_HP - 0,0246 HPMC\_PS\*HPMC\_PS - 0,001517 Lac\*HPMC\_Visc |

## Fits and Diagnostics for All Observations

| Obs | F\_SD\_0.75h(45min) | Fit | SE Fit | 95% CI | Resid | Std Resid | Del Resid |
| --- | --- | --- | --- | --- | --- | --- | --- |
| 1 | 1,455 | 1,198 | 0,454 | (0,248; 2,147) | 0,258 | 0,35 | 0,34 |
| 2 | 2,942 | 3,199 | 0,454 | (2,249; 4,149) | -0,257 | -0,35 | -0,34 |
| 3 | 1,206 | 1,030 | 0,547 | (-0,115; 2,175) | 0,176 | 0,26 | 0,26 |
| 4 | 0,920 | 0,964 | 0,547 | (-0,181; 2,109) | -0,044 | -0,07 | -0,06 |
| 5 | 1,752 | 1,895 | 0,415 | (1,027; 2,762) | -0,142 | -0,19 | -0,18 |
| 6 | 5,464 | 3,804 | 0,415 | (2,936; 4,671) | 1,660 | 2,19 | 2,47 |
| 7 | 1,248 | 1,781 | 0,518 | (0,696; 2,866) | -0,533 | -0,77 | -0,76 |
| 8 | 1,255 | 1,685 | 0,518 | (0,599; 2,770) | -0,430 | -0,62 | -0,61 |
| 9 | 1,854 | 0,989 | 0,438 | (0,072; 1,907) | 0,865 | 1,16 | 1,18 |
| 10 | 2,459 | 2,962 | 0,438 | (2,044; 3,879) | -0,503 | -0,68 | -0,67 |
| 11 | 0,287 | 1,613 | 0,362 | (0,855; 2,370) | -1,326 | -1,69 | -1,79 |
| 12 | 2,231 | 2,283 | 0,362 | (1,525; 3,040) | -0,052 | -0,07 | -0,06 |
| 13 | 1,318 | 1,311 | 0,445 | (0,380; 2,242) | 0,006 | 0,01 | 0,01 |
| 14 | 3,625 | 3,296 | 0,445 | (2,365; 4,228) | 0,329 | 0,44 | 0,44 |
| 15 | 2,361 | 1,884 | 0,378 | (1,092; 2,676) | 0,477 | 0,61 | 0,60 |
| 16 | 2,163 | 2,419 | 0,378 | (1,627; 3,211) | -0,256 | -0,33 | -0,32 |
| 17 | 2,258 | 1,364 | 0,433 | (0,457; 2,271) | 0,894 | 1,20 | 1,21 |
| 18 | 4,381 | 3,820 | 0,433 | (2,913; 4,727) | 0,561 | 0,75 | 0,74 |
| 19 | 1,390 | 3,042 | 0,365 | (2,278; 3,807) | -1,652 | -2,11 | -2,35 |
| 20 | 2,566 | 1,825 | 0,434 | (0,916; 2,734) | 0,741 | 0,99 | 0,99 |
| 21 | 1,553 | 1,332 | 0,499 | (0,287; 2,377) | 0,222 | 0,31 | 0,31 |
| 22 | 1,446 | 1,524 | 0,735 | (-0,014; 3,062) | -0,078 | -0,17 | -0,17 |
| 23 | 1,379 | 1,494 | 0,740 | (-0,055; 3,043) | -0,115 | -0,26 | -0,25 |
| 24 | 1,118 | 1,057 | 0,717 | (-0,444; 2,557) | 0,061 | 0,13 | 0,12 |
| 25 | 0,949 | 2,592 | 0,250 | (2,069; 3,115) | -1,643 | -1,99 | -2,18 |
| 26 | 2,757 | 2,592 | 0,250 | (2,069; 3,115) | 0,165 | 0,20 | 0,19 |
| 27 | 3,208 | 2,592 | 0,250 | (2,069; 3,115) | 0,616 | 0,75 | 0,74 |

| Obs | HI | Cook’s D | DFITS |  |
| --- | --- | --- | --- | --- |
| 1 | 0,276595 | 0,01 | 0,21210 |  |
| 2 | 0,276595 | 0,01 | -0,21148 |  |
| 3 | 0,402122 | 0,01 | 0,21116 |  |
| 4 | 0,402122 | 0,00 | -0,05251 |  |
| 5 | 0,230817 | 0,00 | -0,10033 |  |
| 6 | 0,230817 | 0,18 | 1,35356 | R |
| 7 | 0,360983 | 0,04 | -0,57395 |  |
| 8 | 0,360983 | 0,03 | -0,46034 |  |
| 9 | 0,258085 | 0,06 | 0,69331 |  |
| 10 | 0,258085 | 0,02 | -0,39323 |  |
| 11 | 0,175870 | 0,08 | -0,82594 |  |
| 12 | 0,175870 | 0,00 | -0,02978 |  |
| 13 | 0,265836 | 0,00 | 0,00510 |  |
| 14 | 0,265836 | 0,01 | 0,26177 |  |
| 15 | 0,192197 | 0,01 | 0,29483 |  |
| 16 | 0,192197 | 0,00 | -0,15716 |  |
| 17 | 0,252354 | 0,06 | 0,70475 |  |
| 18 | 0,252354 | 0,02 | 0,43161 |  |
| 19 | 0,179243 | 0,12 | -1,09939 | R |
| 20 | 0,253359 | 0,04 | 0,57869 |  |
| 21 | 0,334695 | 0,01 | 0,21786 |  |
| 22 | 0,725396 | 0,01 | -0,27255 |  |
| 23 | 0,735668 | 0,02 | -0,42007 |  |
| 24 | 0,690354 | 0,00 | 0,18440 |  |
| 25 | 0,083857 | 0,05 | -0,65865 |  |
| 26 | 0,083857 | 0,00 | 0,05888 |  |
| 27 | 0,083857 | 0,01 | 0,22298 |  |

R  Large residual

## Backward Elimination of Terms

α to remove = 0,1

## Coded Coefficients

| Term | Coef | SE Coef | 95% CI | T-Value | P-Value | VIF |
| --- | --- | --- | --- | --- | --- | --- |
| Constant | 1,705 | 0,242 | (1,204; 2,207) | 7,05 | 0,000 |  |
| Lac | 1,003 | 0,391 | (0,193; 1,814) | 2,57 | 0,018 | 1,08 |
| HPMC\_Visc | -0,531 | 0,329 | (-1,212; 0,151) | -1,61 | 0,121 | 1,00 |
| Lac\*Lac | 1,655 | 0,715 | (0,172; 3,138) | 2,31 | 0,030 | 1,00 |
| Lac\*HPMC\_Visc | -1,448 | 0,765 | (-3,034; 0,138) | -1,89 | 0,072 | 1,08 |

## Model Summary

| S | R-sq | R-sq(adj) | PRESS | R-sq(pred) | AICc | BIC |
| --- | --- | --- | --- | --- | --- | --- |
| 0,923148 | 49,72% | 40,58% | 26,3725 | 29,27% | 82,98 | 86,55 |

## Analysis of Variance

| Source | DF | Seq SS | Contribution | Adj SS | Adj MS | F-Value | P-Value |
| --- | --- | --- | --- | --- | --- | --- | --- |
| Model | 4 | 18,540 | 49,72% | 18,540 | 4,6349 | 5,44 | 0,003 |
| Linear | 2 | 10,922 | 29,29% | 7,834 | 3,9169 | 4,60 | 0,021 |
| Lac | 1 | 8,644 | 23,18% | 5,612 | 5,6125 | 6,59 | 0,018 |
| HPMC\_Visc | 1 | 2,279 | 6,11% | 2,221 | 2,2213 | 2,61 | 0,121 |
| Square | 1 | 4,564 | 12,24% | 4,564 | 4,5637 | 5,36 | 0,030 |
| Lac\*Lac | 1 | 4,564 | 12,24% | 4,564 | 4,5637 | 5,36 | 0,030 |
| 2-Way Interaction | 1 | 3,054 | 8,19% | 3,054 | 3,0537 | 3,58 | 0,072 |
| Lac\*HPMC\_Visc | 1 | 3,054 | 8,19% | 3,054 | 3,0537 | 3,58 | 0,072 |
| Error | 22 | 18,748 | 50,28% | 18,748 | 0,8522 |  |  |
| Lack-of-Fit | 20 | 15,339 | 41,14% | 15,339 | 0,7670 | 0,45 | 0,866 |
| Pure Error | 2 | 3,409 | 9,14% | 3,409 | 1,7046 |  |  |
| Total | 26 | 37,288 | 100,00% |  |  |  |  |

## Regression Equation in Uncoded Units

|  |  |  |
| --- | --- | --- |
| F\_SD\_1h(60min) | = | -1,90 - 2,4 Lac + 0,000608 HPMC\_Visc + 26,5 Lac\*Lac - 0,001489 Lac\*HPMC\_Visc |

## Fits and Diagnostics for All Observations

| Obs | F\_SD\_1h(60min) | Fit | SE Fit | 95% CI | Resid | Std Resid | Del Resid | HI |
| --- | --- | --- | --- | --- | --- | --- | --- | --- |
| 1 | 1,575 | 1,480 | 0,390 | (0,670; 2,289) | 0,095 | 0,11 | 0,11 | 0,178821 |
| 2 | 3,009 | 3,514 | 0,390 | (2,704; 4,323) | -0,505 | -0,60 | -0,59 | 0,178821 |
| 3 | 1,284 | 1,751 | 0,490 | (0,735; 2,766) | -0,467 | -0,60 | -0,59 | 0,281436 |
| 4 | 0,992 | 1,756 | 0,490 | (0,740; 2,772) | -0,764 | -0,98 | -0,98 | 0,281436 |
| 5 | 1,922 | 1,492 | 0,367 | (0,730; 2,254) | 0,430 | 0,51 | 0,50 | 0,158392 |
| 6 | 5,554 | 3,435 | 0,367 | (2,673; 4,197) | 2,119 | 2,50 | 2,89 | 0,158392 |
| 7 | 1,297 | 1,755 | 0,499 | (0,720; 2,789) | -0,458 | -0,59 | -0,58 | 0,291836 |
| 8 | 1,379 | 1,730 | 0,499 | (0,695; 2,764) | -0,351 | -0,45 | -0,44 | 0,291836 |
| 9 | 1,905 | 1,484 | 0,383 | (0,689; 2,278) | 0,422 | 0,50 | 0,49 | 0,172155 |
| 10 | 2,436 | 3,489 | 0,383 | (2,695; 4,283) | -1,053 | -1,25 | -1,27 | 0,172155 |
| 11 | 0,289 | 1,654 | 0,307 | (1,018; 2,291) | -1,365 | -1,57 | -1,63 | 0,110622 |
| 12 | 2,220 | 2,381 | 0,307 | (1,745; 3,018) | -0,162 | -0,19 | -0,18 | 0,110622 |
| 13 | 1,323 | 1,482 | 0,386 | (0,681; 2,283) | -0,159 | -0,19 | -0,18 | 0,175079 |
| 14 | 3,913 | 3,500 | 0,386 | (2,699; 4,301) | 0,413 | 0,49 | 0,48 | 0,175079 |
| 15 | 2,468 | 1,672 | 0,334 | (0,979; 2,364) | 0,796 | 0,92 | 0,92 | 0,130851 |
| 16 | 2,474 | 2,267 | 0,334 | (1,574; 2,959) | 0,207 | 0,24 | 0,24 | 0,130851 |
| 17 | 2,438 | 2,185 | 0,696 | (0,741; 3,629) | 0,253 | 0,42 | 0,41 | 0,568720 |
| 18 | 4,683 | 4,735 | 0,696 | (3,291; 6,179) | -0,052 | -0,09 | -0,08 | 0,568720 |
| 19 | 1,281 | 2,236 | 0,373 | (1,462; 3,010) | -0,955 | -1,13 | -1,14 | 0,163395 |
| 20 | 2,722 | 1,175 | 0,440 | (0,262; 2,088) | 1,547 | 1,91 | 2,04 | 0,227435 |
| 21 | 1,632 | 1,801 | 0,239 | (1,305; 2,297) | -0,170 | -0,19 | -0,19 | 0,067038 |
| 22 | 1,475 | 1,767 | 0,238 | (1,272; 2,261) | -0,292 | -0,33 | -0,32 | 0,066681 |
| 23 | 1,360 | 1,843 | 0,242 | (1,341; 2,346) | -0,483 | -0,54 | -0,53 | 0,068919 |
| 24 | 1,112 | 1,849 | 0,243 | (1,345; 2,352) | -0,737 | -0,83 | -0,82 | 0,069279 |
| 25 | 0,864 | 1,805 | 0,239 | (1,309; 2,301) | -0,941 | -1,06 | -1,06 | 0,067143 |
| 26 | 3,042 | 1,805 | 0,239 | (1,309; 2,301) | 1,237 | 1,39 | 1,42 | 0,067143 |
| 27 | 3,200 | 1,805 | 0,239 | (1,309; 2,301) | 1,395 | 1,56 | 1,62 | 0,067143 |

| Obs | Cook’s D | DFITS |  |  |
| --- | --- | --- | --- | --- |
| 1 | 0,00 | 0,05180 |  |  |
| 2 | 0,02 | -0,27749 |  |  |
| 3 | 0,03 | -0,36802 |  |  |
| 4 | 0,07 | -0,61053 |  |  |
| 5 | 0,01 | 0,21632 |  |  |
| 6 | 0,24 | 1,25381 | R |  |
| 7 | 0,03 | -0,37287 |  |  |
| 8 | 0,02 | -0,28472 |  |  |
| 9 | 0,01 | 0,22491 |  |  |
| 10 | 0,07 | -0,57983 |  |  |
| 11 | 0,06 | -0,57337 |  |  |
| 12 | 0,00 | -0,06403 |  |  |
| 13 | 0,00 | -0,08516 |  |  |
| 14 | 0,01 | 0,22281 |  |  |
| 15 | 0,03 | 0,35767 |  |  |
| 16 | 0,00 | 0,09131 |  |  |
| 17 | 0,05 | 0,47010 |  | X |
| 18 | 0,00 | -0,09665 |  | X |
| 19 | 0,05 | -0,50311 |  |  |
| 20 | 0,21 | 1,10656 |  |  |
| 21 | 0,00 | -0,04984 |  |  |
| 22 | 0,00 | -0,08571 |  |  |
| 23 | 0,00 | -0,14502 |  |  |
| 24 | 0,01 | -0,22398 |  |  |
| 25 | 0,02 | -0,28399 |  |  |
| 26 | 0,03 | 0,38066 |  |  |
| 27 | 0,04 | 0,43502 |  |  |

R  Large residual  
X  Unusual X

## Backward Elimination of Terms

α to remove = 0,1

## Coded Coefficients

| Term | Coef | SE Coef | 95% CI | T-Value | P-Value | VIF |
| --- | --- | --- | --- | --- | --- | --- |
| Constant | 1,837 | 0,280 | (1,258; 2,416) | 6,55 | 0,000 |  |
| Lac | 1,278 | 0,443 | (0,363; 2,194) | 2,88 | 0,008 | 1,00 |
| Lac\*Lac | 1,939 | 0,841 | (0,203; 3,676) | 2,31 | 0,030 | 1,00 |

## Model Summary

| S | R-sq | R-sq(adj) | PRESS | R-sq(pred) | AICc | BIC |
| --- | --- | --- | --- | --- | --- | --- |
| 1,08612 | 36,21% | 30,90% | 33,2892 | 25,00% | 87,72 | 91,09 |

## Analysis of Variance

| Source | DF | Seq SS | Contribution | Adj SS | Adj MS | F-Value | P-Value |
| --- | --- | --- | --- | --- | --- | --- | --- |
| Model | 2 | 16,074 | 36,21% | 16,074 | 8,037 | 6,81 | 0,005 |
| Linear | 1 | 9,806 | 22,09% | 9,806 | 9,806 | 8,31 | 0,008 |
| Lac | 1 | 9,806 | 22,09% | 9,806 | 9,806 | 8,31 | 0,008 |
| Square | 1 | 6,268 | 14,12% | 6,268 | 6,268 | 5,31 | 0,030 |
| Lac\*Lac | 1 | 6,268 | 14,12% | 6,268 | 6,268 | 5,31 | 0,030 |
| Error | 24 | 28,312 | 63,79% | 28,312 | 1,180 |  |  |
| Lack-of-Fit | 22 | 23,087 | 52,02% | 23,087 | 1,049 | 0,40 | 0,894 |
| Pure Error | 2 | 5,224 | 11,77% | 5,224 | 2,612 |  |  |
| Total | 26 | 44,386 | 100,00% |  |  |  |  |

## Regression Equation in Uncoded Units

|  |  |  |
| --- | --- | --- |
| F\_SD\_1.5h(90min) | = | 7,04 - 25,9 Lac + 31,0 Lac\*Lac |

## Fits and Diagnostics for All Observations

| Obs | F\_SD\_1.5h(90min) | Fit | SE Fit | 95% CI | Resid | Std Resid | Del Resid | HI |
| --- | --- | --- | --- | --- | --- | --- | --- | --- |
| 1 | 1,783 | 1,683 | 0,306 | (1,052; 2,314) | 0,100 | 0,10 | 0,09 | 0,079167 |
| 2 | 3,215 | 2,961 | 0,306 | (2,331; 3,592) | 0,254 | 0,24 | 0,24 | 0,079167 |
| 3 | 1,392 | 1,683 | 0,306 | (1,052; 2,314) | -0,290 | -0,28 | -0,27 | 0,079167 |
| 4 | 1,054 | 2,961 | 0,306 | (2,331; 3,592) | -1,907 | -1,83 | -1,93 | 0,079167 |
| 5 | 2,184 | 1,683 | 0,306 | (1,052; 2,314) | 0,501 | 0,48 | 0,47 | 0,079167 |
| 6 | 5,687 | 2,961 | 0,306 | (2,331; 3,592) | 2,726 | 2,62 | 3,03 | 0,079167 |
| 7 | 1,391 | 1,683 | 0,306 | (1,052; 2,314) | -0,292 | -0,28 | -0,27 | 0,079167 |
| 8 | 1,603 | 2,961 | 0,306 | (2,331; 3,592) | -1,358 | -1,30 | -1,32 | 0,079167 |
| 9 | 1,901 | 1,683 | 0,306 | (1,052; 2,314) | 0,218 | 0,21 | 0,20 | 0,079167 |
| 10 | 2,432 | 2,961 | 0,306 | (2,331; 3,592) | -0,530 | -0,51 | -0,50 | 0,079167 |
| 11 | 0,302 | 1,683 | 0,306 | (1,052; 2,314) | -1,381 | -1,32 | -1,35 | 0,079167 |
| 12 | 2,133 | 2,961 | 0,306 | (2,331; 3,592) | -0,828 | -0,79 | -0,79 | 0,079167 |
| 13 | 1,297 | 1,683 | 0,306 | (1,052; 2,314) | -0,386 | -0,37 | -0,36 | 0,079167 |
| 14 | 4,384 | 2,961 | 0,306 | (2,331; 3,592) | 1,423 | 1,37 | 1,39 | 0,079167 |
| 15 | 2,605 | 1,683 | 0,306 | (1,052; 2,314) | 0,922 | 0,88 | 0,88 | 0,079167 |
| 16 | 3,017 | 2,961 | 0,306 | (2,331; 3,592) | 0,056 | 0,05 | 0,05 | 0,079167 |
| 17 | 2,706 | 2,498 | 0,818 | (0,811; 4,186) | 0,208 | 0,29 | 0,28 | 0,566667 |
| 18 | 5,041 | 5,055 | 0,818 | (3,367; 6,742) | -0,014 | -0,02 | -0,02 | 0,566667 |
| 19 | 1,124 | 1,837 | 0,280 | (1,258; 2,416) | -0,713 | -0,68 | -0,67 | 0,066667 |
| 20 | 2,952 | 1,837 | 0,280 | (1,258; 2,416) | 1,115 | 1,06 | 1,07 | 0,066667 |
| 21 | 1,733 | 1,837 | 0,280 | (1,258; 2,416) | -0,104 | -0,10 | -0,10 | 0,066667 |
| 22 | 1,503 | 1,837 | 0,280 | (1,258; 2,416) | -0,335 | -0,32 | -0,31 | 0,066667 |
| 23 | 1,348 | 1,837 | 0,280 | (1,258; 2,416) | -0,489 | -0,47 | -0,46 | 0,066667 |
| 24 | 1,137 | 1,837 | 0,280 | (1,258; 2,416) | -0,700 | -0,67 | -0,66 | 0,066667 |
| 25 | 0,582 | 1,837 | 0,280 | (1,258; 2,416) | -1,255 | -1,20 | -1,21 | 0,066667 |
| 26 | 3,530 | 1,837 | 0,280 | (1,258; 2,416) | 1,693 | 1,61 | 1,67 | 0,066667 |
| 27 | 3,205 | 1,837 | 0,280 | (1,258; 2,416) | 1,368 | 1,30 | 1,32 | 0,066667 |

| Obs | Cook’s D | DFITS |  |  |
| --- | --- | --- | --- | --- |
| 1 | 0,00 | 0,027507 |  |  |
| 2 | 0,00 | 0,069945 |  |  |
| 3 | 0,00 | -0,080120 |  |  |
| 4 | 0,10 | -0,566148 |  |  |
| 5 | 0,01 | 0,138766 |  |  |
| 6 | 0,20 | 0,887846 | R |  |
| 7 | 0,00 | -0,080608 |  |  |
| 8 | 0,05 | -0,387965 |  |  |
| 9 | 0,00 | 0,059999 |  |  |
| 10 | 0,01 | -0,146650 |  |  |
| 11 | 0,05 | -0,394972 |  |  |
| 12 | 0,02 | -0,231232 |  |  |
| 13 | 0,00 | -0,106612 |  |  |
| 14 | 0,05 | 0,408032 |  |  |
| 15 | 0,02 | 0,258069 |  |  |
| 16 | 0,00 | 0,015406 |  |  |
| 17 | 0,04 | 0,325800 |  | X |
| 18 | 0,00 | -0,022491 |  | X |
| 19 | 0,01 | -0,179547 |  |  |
| 20 | 0,03 | 0,284781 |  |  |
| 21 | 0,00 | -0,026002 |  |  |
| 22 | 0,00 | -0,083623 |  |  |
| 23 | 0,01 | -0,122463 |  |  |
| 24 | 0,01 | -0,176147 |  |  |
| 25 | 0,03 | -0,322643 |  |  |
| 26 | 0,06 | 0,447066 |  |  |
| 27 | 0,04 | 0,353810 |  |  |

R  Large residual  
X  Unusual X

## Backward Elimination of Terms

α to remove = 0,1

## Coded Coefficients

| Term | Coef | SE Coef | 95% CI | T-Value | P-Value | VIF |
| --- | --- | --- | --- | --- | --- | --- |
| Constant | 1,907 | 0,298 | (1,291; 2,522) | 6,40 | 0,000 |  |
| Lac | 1,322 | 0,471 | (0,349; 2,295) | 2,80 | 0,010 | 1,00 |
| Lac\*Lac | 2,102 | 0,894 | (0,256; 3,948) | 2,35 | 0,027 | 1,00 |

## Model Summary

| S | R-sq | R-sq(adj) | PRESS | R-sq(pred) | AICc | BIC |
| --- | --- | --- | --- | --- | --- | --- |
| 1,15468 | 35,81% | 30,46% | 37,4922 | 24,79% | 91,03 | 94,39 |

## Analysis of Variance

| Source | DF | Seq SS | Contribution | Adj SS | Adj MS | F-Value | P-Value |
| --- | --- | --- | --- | --- | --- | --- | --- |
| Model | 2 | 17,849 | 35,81% | 17,849 | 8,925 | 6,69 | 0,005 |
| Linear | 1 | 10,486 | 21,04% | 10,486 | 10,486 | 7,86 | 0,010 |
| Lac | 1 | 10,486 | 21,04% | 10,486 | 10,486 | 7,86 | 0,010 |
| Square | 1 | 7,364 | 14,77% | 7,364 | 7,364 | 5,52 | 0,027 |
| Lac\*Lac | 1 | 7,364 | 14,77% | 7,364 | 7,364 | 5,52 | 0,027 |
| Error | 24 | 31,999 | 64,19% | 31,999 | 1,333 |  |  |
| Lack-of-Fit | 22 | 24,800 | 49,75% | 24,800 | 1,127 | 0,31 | 0,939 |
| Pure Error | 2 | 7,199 | 14,44% | 7,199 | 3,600 |  |  |
| Total | 26 | 49,848 | 100,00% |  |  |  |  |

## Regression Equation in Uncoded Units

|  |  |  |
| --- | --- | --- |
| F\_SD\_2h(120min) | = | 7,67 - 28,3 Lac + 33,6 Lac\*Lac |

## Fits and Diagnostics for All Observations

| Obs | F\_SD\_2h(120min) | Fit | SE Fit | 95% CI | Resid | Std Resid | Del Resid | HI |
| --- | --- | --- | --- | --- | --- | --- | --- | --- |
| 1 | 2,004 | 1,771 | 0,325 | (1,101; 2,442) | 0,233 | 0,21 | 0,21 | 0,079167 |
| 2 | 3,271 | 3,093 | 0,325 | (2,423; 3,764) | 0,178 | 0,16 | 0,16 | 0,079167 |
| 3 | 1,456 | 1,771 | 0,325 | (1,101; 2,442) | -0,315 | -0,28 | -0,28 | 0,079167 |
| 4 | 1,045 | 3,093 | 0,325 | (2,423; 3,764) | -2,048 | -1,85 | -1,95 | 0,079167 |
| 5 | 2,398 | 1,771 | 0,325 | (1,101; 2,442) | 0,626 | 0,57 | 0,56 | 0,079167 |
| 6 | 5,712 | 3,093 | 0,325 | (2,423; 3,764) | 2,618 | 2,36 | 2,64 | 0,079167 |
| 7 | 1,491 | 1,771 | 0,325 | (1,101; 2,442) | -0,281 | -0,25 | -0,25 | 0,079167 |
| 8 | 1,746 | 3,093 | 0,325 | (2,423; 3,764) | -1,348 | -1,22 | -1,23 | 0,079167 |
| 9 | 1,819 | 1,771 | 0,325 | (1,101; 2,442) | 0,048 | 0,04 | 0,04 | 0,079167 |
| 10 | 2,487 | 3,093 | 0,325 | (2,423; 3,764) | -0,606 | -0,55 | -0,54 | 0,079167 |
| 11 | 0,460 | 1,771 | 0,325 | (1,101; 2,442) | -1,311 | -1,18 | -1,19 | 0,079167 |
| 12 | 2,050 | 3,093 | 0,325 | (2,423; 3,764) | -1,044 | -0,94 | -0,94 | 0,079167 |
| 13 | 1,302 | 1,771 | 0,325 | (1,101; 2,442) | -0,470 | -0,42 | -0,42 | 0,079167 |
| 14 | 4,644 | 3,093 | 0,325 | (2,423; 3,764) | 1,551 | 1,40 | 1,43 | 0,079167 |
| 15 | 2,712 | 1,771 | 0,325 | (1,101; 2,442) | 0,940 | 0,85 | 0,84 | 0,079167 |
| 16 | 3,524 | 3,093 | 0,325 | (2,423; 3,764) | 0,431 | 0,39 | 0,38 | 0,079167 |
| 17 | 2,852 | 2,687 | 0,869 | (0,893; 4,481) | 0,165 | 0,22 | 0,21 | 0,566667 |
| 18 | 5,365 | 5,331 | 0,869 | (3,537; 7,125) | 0,034 | 0,04 | 0,04 | 0,566667 |
| 19 | 1,098 | 1,907 | 0,298 | (1,291; 2,522) | -0,808 | -0,72 | -0,72 | 0,066667 |
| 20 | 3,212 | 1,907 | 0,298 | (1,291; 2,522) | 1,305 | 1,17 | 1,18 | 0,066667 |
| 21 | 1,875 | 1,907 | 0,298 | (1,291; 2,522) | -0,032 | -0,03 | -0,03 | 0,066667 |
| 22 | 1,577 | 1,907 | 0,298 | (1,291; 2,522) | -0,330 | -0,30 | -0,29 | 0,066667 |
| 23 | 1,359 | 1,907 | 0,298 | (1,291; 2,522) | -0,547 | -0,49 | -0,48 | 0,066667 |
| 24 | 1,152 | 1,907 | 0,298 | (1,291; 2,522) | -0,755 | -0,68 | -0,67 | 0,066667 |
| 25 | 0,358 | 1,907 | 0,298 | (1,291; 2,522) | -1,548 | -1,39 | -1,42 | 0,066667 |
| 26 | 3,982 | 1,907 | 0,298 | (1,291; 2,522) | 2,075 | 1,86 | 1,97 | 0,066667 |
| 27 | 3,145 | 1,907 | 0,298 | (1,291; 2,522) | 1,238 | 1,11 | 1,12 | 0,066667 |

| Obs | Cook’s D | DFITS |  |  |
| --- | --- | --- | --- | --- |
| 1 | 0,00 | 0,060377 |  |  |
| 2 | 0,00 | 0,046168 |  |  |
| 3 | 0,00 | -0,081862 |  |  |
| 4 | 0,10 | -0,572855 |  |  |
| 5 | 0,01 | 0,163352 |  |  |
| 6 | 0,16 | 0,774363 | R |  |
| 7 | 0,00 | -0,072802 |  |  |
| 8 | 0,04 | -0,360402 |  |  |
| 9 | 0,00 | 0,012408 |  |  |
| 10 | 0,01 | -0,158054 |  |  |
| 11 | 0,04 | -0,350011 |  |  |
| 12 | 0,03 | -0,275493 |  |  |
| 13 | 0,01 | -0,122098 |  |  |
| 14 | 0,06 | 0,419246 |  |  |
| 15 | 0,02 | 0,247323 |  |  |
| 16 | 0,00 | 0,111969 |  |  |
| 17 | 0,02 | 0,243434 |  | X |
| 18 | 0,00 | 0,050107 |  | X |
| 19 | 0,01 | -0,191686 |  |  |
| 20 | 0,03 | 0,315185 |  |  |
| 21 | 0,00 | -0,007467 |  |  |
| 22 | 0,00 | -0,077540 |  |  |
| 23 | 0,01 | -0,129006 |  |  |
| 24 | 0,01 | -0,178836 |  |  |
| 25 | 0,05 | -0,378686 |  |  |
| 26 | 0,08 | 0,526130 |  |  |
| 27 | 0,03 | 0,298180 |  |  |

R  Large residual  
X  Unusual X

## Backward Elimination of Terms

α to remove = 0,1

## Coded Coefficients

| Term | Coef | SE Coef | 95% CI | T-Value | P-Value | VIF |
| --- | --- | --- | --- | --- | --- | --- |
| Constant | 1,965 | 0,311 | (1,324; 2,606) | 6,33 | 0,000 |  |
| Lac | 1,332 | 0,491 | (0,319; 2,346) | 2,71 | 0,012 | 1,00 |
| Lac\*Lac | 2,222 | 0,932 | (0,299; 4,145) | 2,38 | 0,025 | 1,00 |

## Model Summary

| S | R-sq | R-sq(adj) | PRESS | R-sq(pred) | AICc | BIC |
| --- | --- | --- | --- | --- | --- | --- |
| 1,20301 | 35,21% | 29,81% | 40,6504 | 24,17% | 93,24 | 96,61 |

## Analysis of Variance

| Source | DF | Seq SS | Contribution | Adj SS | Adj MS | F-Value | P-Value |
| --- | --- | --- | --- | --- | --- | --- | --- |
| Model | 2 | 18,877 | 35,21% | 18,877 | 9,439 | 6,52 | 0,005 |
| Linear | 1 | 10,650 | 19,87% | 10,650 | 10,650 | 7,36 | 0,012 |
| Lac | 1 | 10,650 | 19,87% | 10,650 | 10,650 | 7,36 | 0,012 |
| Square | 1 | 8,227 | 15,35% | 8,227 | 8,227 | 5,68 | 0,025 |
| Lac\*Lac | 1 | 8,227 | 15,35% | 8,227 | 8,227 | 5,68 | 0,025 |
| Error | 24 | 34,733 | 64,79% | 34,733 | 1,447 |  |  |
| Lack-of-Fit | 22 | 26,126 | 48,73% | 26,126 | 1,188 | 0,28 | 0,956 |
| Pure Error | 2 | 8,608 | 16,06% | 8,608 | 4,304 |  |  |
| Total | 26 | 53,611 | 100,00% |  |  |  |  |

## Regression Equation in Uncoded Units

|  |  |  |
| --- | --- | --- |
| F\_SD\_2.5h(150min) | = | 8,19 - 30,2 Lac + 35,5 Lac\*Lac |

## Fits and Diagnostics for All Observations

| Obs | F\_SD\_2.5h(150min) | Fit | SE Fit | 95% CI | Resid | Std Resid | Del Resid | HI |
| --- | --- | --- | --- | --- | --- | --- | --- | --- |
| 1 | 2,206 | 1,854 | 0,338 | (1,156; 2,553) | 0,352 | 0,30 | 0,30 | 0,079167 |
| 2 | 3,259 | 3,187 | 0,338 | (2,488; 3,885) | 0,073 | 0,06 | 0,06 | 0,079167 |
| 3 | 1,540 | 1,854 | 0,338 | (1,156; 2,553) | -0,314 | -0,27 | -0,27 | 0,079167 |
| 4 | 1,023 | 3,187 | 0,338 | (2,488; 3,885) | -2,164 | -1,87 | -1,99 | 0,079167 |
| 5 | 2,526 | 1,854 | 0,338 | (1,156; 2,553) | 0,672 | 0,58 | 0,57 | 0,079167 |
| 6 | 5,694 | 3,187 | 0,338 | (2,488; 3,885) | 2,507 | 2,17 | 2,37 | 0,079167 |
| 7 | 1,584 | 1,854 | 0,338 | (1,156; 2,553) | -0,271 | -0,23 | -0,23 | 0,079167 |
| 8 | 1,938 | 3,187 | 0,338 | (2,488; 3,885) | -1,249 | -1,08 | -1,09 | 0,079167 |
| 9 | 1,796 | 1,854 | 0,338 | (1,156; 2,553) | -0,059 | -0,05 | -0,05 | 0,079167 |
| 10 | 2,518 | 3,187 | 0,338 | (2,488; 3,885) | -0,668 | -0,58 | -0,57 | 0,079167 |
| 11 | 0,651 | 1,854 | 0,338 | (1,156; 2,553) | -1,203 | -1,04 | -1,04 | 0,079167 |
| 12 | 1,918 | 3,187 | 0,338 | (2,488; 3,885) | -1,269 | -1,10 | -1,10 | 0,079167 |
| 13 | 1,302 | 1,854 | 0,338 | (1,156; 2,553) | -0,552 | -0,48 | -0,47 | 0,079167 |
| 14 | 4,896 | 3,187 | 0,338 | (2,488; 3,885) | 1,709 | 1,48 | 1,52 | 0,079167 |
| 15 | 2,719 | 1,854 | 0,338 | (1,156; 2,553) | 0,865 | 0,75 | 0,74 | 0,079167 |
| 16 | 3,911 | 3,187 | 0,338 | (2,488; 3,885) | 0,724 | 0,63 | 0,62 | 0,079167 |
| 17 | 3,004 | 2,855 | 0,906 | (0,986; 4,724) | 0,150 | 0,19 | 0,19 | 0,566667 |
| 18 | 5,581 | 5,519 | 0,906 | (3,650; 7,388) | 0,062 | 0,08 | 0,08 | 0,566667 |
| 19 | 1,072 | 1,965 | 0,311 | (1,324; 2,606) | -0,893 | -0,77 | -0,76 | 0,066667 |
| 20 | 3,414 | 1,965 | 0,311 | (1,324; 2,606) | 1,449 | 1,25 | 1,26 | 0,066667 |
| 21 | 1,961 | 1,965 | 0,311 | (1,324; 2,606) | -0,004 | -0,00 | -0,00 | 0,066667 |
| 22 | 1,672 | 1,965 | 0,311 | (1,324; 2,606) | -0,293 | -0,25 | -0,25 | 0,066667 |
| 23 | 1,382 | 1,965 | 0,311 | (1,324; 2,606) | -0,583 | -0,50 | -0,49 | 0,066667 |
| 24 | 1,233 | 1,965 | 0,311 | (1,324; 2,606) | -0,733 | -0,63 | -0,62 | 0,066667 |
| 25 | 0,242 | 1,965 | 0,311 | (1,324; 2,606) | -1,723 | -1,48 | -1,52 | 0,066667 |
| 26 | 4,290 | 1,965 | 0,311 | (1,324; 2,606) | 2,325 | 2,00 | 2,15 | 0,066667 |
| 27 | 3,056 | 1,965 | 0,311 | (1,324; 2,606) | 1,091 | 0,94 | 0,94 | 0,066667 |

| Obs | Cook’s D | DFITS |  |  |
| --- | --- | --- | --- | --- |
| 1 | 0,00 | 0,087696 |  |  |
| 2 | 0,00 | 0,018072 |  |  |
| 3 | 0,00 | -0,078292 |  |  |
| 4 | 0,10 | -0,582292 |  |  |
| 5 | 0,01 | 0,168189 |  |  |
| 6 | 0,14 | 0,695388 | R |  |
| 7 | 0,00 | -0,067442 |  |  |
| 8 | 0,03 | -0,318300 |  |  |
| 9 | 0,00 | -0,014635 |  |  |
| 10 | 0,01 | -0,167349 |  |  |
| 11 | 0,03 | -0,306244 |  |  |
| 12 | 0,03 | -0,323800 |  |  |
| 13 | 0,01 | -0,137941 |  |  |
| 14 | 0,06 | 0,445820 |  |  |
| 15 | 0,02 | 0,217515 |  |  |
| 16 | 0,01 | 0,181604 |  |  |
| 17 | 0,02 | 0,211882 |  | X |
| 18 | 0,00 | 0,088000 |  | X |
| 19 | 0,01 | -0,203509 |  |  |
| 20 | 0,04 | 0,337294 |  |  |
| 21 | 0,00 | -0,000913 |  |  |
| 22 | 0,00 | -0,066006 |  |  |
| 23 | 0,01 | -0,132046 |  |  |
| 24 | 0,01 | -0,166300 |  |  |
| 25 | 0,05 | -0,406928 |  |  |
| 26 | 0,10 | 0,573288 | R |  |
| 27 | 0,02 | 0,250251 |  |  |

R  Large residual  
X  Unusual X

## Backward Elimination of Terms

α to remove = 0,1

## Coded Coefficients

| Term | Coef | SE Coef | 95% CI | T-Value | P-Value | VIF |
| --- | --- | --- | --- | --- | --- | --- |
| Constant | 2,019 | 0,322 | (1,355; 2,683) | 6,27 | 0,000 |  |
| Lac | 1,355 | 0,509 | (0,305; 2,405) | 2,66 | 0,014 | 1,00 |
| Lac\*Lac | 2,304 | 0,965 | (0,311; 4,296) | 2,39 | 0,025 | 1,00 |

## Model Summary

| S | R-sq | R-sq(adj) | PRESS | R-sq(pred) | AICc | BIC |
| --- | --- | --- | --- | --- | --- | --- |
| 1,24635 | 34,76% | 29,32% | 43,5942 | 23,71% | 95,15 | 98,52 |

## Analysis of Variance

| Source | DF | Seq SS | Contribution | Adj SS | Adj MS | F-Value | P-Value |
| --- | --- | --- | --- | --- | --- | --- | --- |
| Model | 2 | 19,861 | 34,76% | 19,861 | 9,930 | 6,39 | 0,006 |
| Linear | 1 | 11,014 | 19,27% | 11,014 | 11,014 | 7,09 | 0,014 |
| Lac | 1 | 11,014 | 19,27% | 11,014 | 11,014 | 7,09 | 0,014 |
| Square | 1 | 8,847 | 15,48% | 8,847 | 8,847 | 5,70 | 0,025 |
| Lac\*Lac | 1 | 8,847 | 15,48% | 8,847 | 8,847 | 5,70 | 0,025 |
| Error | 24 | 37,282 | 65,24% | 37,282 | 1,553 |  |  |
| Lack-of-Fit | 22 | 27,356 | 47,87% | 27,356 | 1,243 | 0,25 | 0,967 |
| Pure Error | 2 | 9,925 | 17,37% | 9,925 | 4,963 |  |  |
| Total | 26 | 57,142 | 100,00% |  |  |  |  |

## Regression Equation in Uncoded Units

|  |  |  |
| --- | --- | --- |
| F\_SD\_3h(180min) | = | 8,52 - 31,4 Lac + 36,9 Lac\*Lac |

## Fits and Diagnostics for All Observations

| Obs | F\_SD\_3h(180min) | Fit | SE Fit | 95% CI | Resid | Std Resid | Del Resid | HI |
| --- | --- | --- | --- | --- | --- | --- | --- | --- |
| 1 | 2,375 | 1,917 | 0,351 | (1,194; 2,641) | 0,458 | 0,38 | 0,38 | 0,079167 |
| 2 | 3,270 | 3,272 | 0,351 | (2,548; 3,996) | -0,002 | -0,00 | -0,00 | 0,079167 |
| 3 | 1,617 | 1,917 | 0,351 | (1,194; 2,641) | -0,300 | -0,25 | -0,25 | 0,079167 |
| 4 | 0,976 | 3,272 | 0,351 | (2,548; 3,996) | -2,296 | -1,92 | -2,04 | 0,079167 |
| 5 | 2,604 | 1,917 | 0,351 | (1,194; 2,641) | 0,687 | 0,57 | 0,57 | 0,079167 |
| 6 | 5,741 | 3,272 | 0,351 | (2,548; 3,996) | 2,469 | 2,06 | 2,23 | 0,079167 |
| 7 | 1,658 | 1,917 | 0,351 | (1,194; 2,641) | -0,259 | -0,22 | -0,21 | 0,079167 |
| 8 | 2,097 | 3,272 | 0,351 | (2,548; 3,996) | -1,176 | -0,98 | -0,98 | 0,079167 |
| 9 | 1,707 | 1,917 | 0,351 | (1,194; 2,641) | -0,210 | -0,18 | -0,17 | 0,079167 |
| 10 | 2,506 | 3,272 | 0,351 | (2,548; 3,996) | -0,767 | -0,64 | -0,63 | 0,079167 |
| 11 | 0,812 | 1,917 | 0,351 | (1,194; 2,641) | -1,106 | -0,92 | -0,92 | 0,079167 |
| 12 | 1,867 | 3,272 | 0,351 | (2,548; 3,996) | -1,406 | -1,18 | -1,19 | 0,079167 |
| 13 | 1,422 | 1,917 | 0,351 | (1,194; 2,641) | -0,496 | -0,41 | -0,41 | 0,079167 |
| 14 | 5,046 | 3,272 | 0,351 | (2,548; 3,996) | 1,773 | 1,48 | 1,52 | 0,079167 |
| 15 | 2,717 | 1,917 | 0,351 | (1,194; 2,641) | 0,800 | 0,67 | 0,66 | 0,079167 |
| 16 | 4,260 | 3,272 | 0,351 | (2,548; 3,996) | 0,988 | 0,83 | 0,82 | 0,079167 |
| 17 | 3,076 | 2,968 | 0,938 | (1,032; 4,904) | 0,108 | 0,13 | 0,13 | 0,566667 |
| 18 | 5,780 | 5,678 | 0,938 | (3,741; 7,614) | 0,103 | 0,12 | 0,12 | 0,566667 |
| 19 | 1,085 | 2,019 | 0,322 | (1,355; 2,683) | -0,934 | -0,78 | -0,77 | 0,066667 |
| 20 | 3,525 | 2,019 | 0,322 | (1,355; 2,683) | 1,506 | 1,25 | 1,27 | 0,066667 |
| 21 | 2,072 | 2,019 | 0,322 | (1,355; 2,683) | 0,053 | 0,04 | 0,04 | 0,066667 |
| 22 | 1,762 | 2,019 | 0,322 | (1,355; 2,683) | -0,257 | -0,21 | -0,21 | 0,066667 |
| 23 | 1,412 | 2,019 | 0,322 | (1,355; 2,683) | -0,607 | -0,50 | -0,50 | 0,066667 |
| 24 | 1,311 | 2,019 | 0,322 | (1,355; 2,683) | -0,707 | -0,59 | -0,58 | 0,066667 |
| 25 | 0,138 | 2,019 | 0,322 | (1,355; 2,683) | -1,881 | -1,56 | -1,61 | 0,066667 |
| 26 | 4,535 | 2,019 | 0,322 | (1,355; 2,683) | 2,516 | 2,09 | 2,26 | 0,066667 |
| 27 | 2,962 | 2,019 | 0,322 | (1,355; 2,683) | 0,943 | 0,78 | 0,78 | 0,066667 |

| Obs | Cook’s D | DFITS |  |  |
| --- | --- | --- | --- | --- |
| 1 | 0,00 | 0,110271 |  |  |
| 2 | 0,00 | -0,000461 |  |  |
| 3 | 0,00 | -0,072200 |  |  |
| 4 | 0,11 | -0,598934 |  |  |
| 5 | 0,01 | 0,165904 |  |  |
| 6 | 0,12 | 0,653469 | R |  |
| 7 | 0,00 | -0,062328 |  |  |
| 8 | 0,03 | -0,288018 |  |  |
| 9 | 0,00 | -0,050543 |  |  |
| 10 | 0,01 | -0,185614 |  |  |
| 11 | 0,02 | -0,270248 |  |  |
| 12 | 0,04 | -0,347475 |  |  |
| 13 | 0,00 | -0,119355 |  |  |
| 14 | 0,06 | 0,446522 |  |  |
| 15 | 0,01 | 0,193763 |  |  |
| 16 | 0,02 | 0,240467 |  |  |
| 17 | 0,01 | 0,147791 |  | X |
| 18 | 0,01 | 0,139905 |  | X |
| 19 | 0,01 | -0,205529 |  |  |
| 20 | 0,04 | 0,338544 |  |  |
| 21 | 0,00 | 0,011507 |  |  |
| 22 | 0,00 | -0,055800 |  |  |
| 23 | 0,01 | -0,132572 |  |  |
| 24 | 0,01 | -0,154824 |  |  |
| 25 | 0,06 | -0,431185 |  |  |
| 26 | 0,10 | 0,604343 | R |  |
| 27 | 0,01 | 0,207551 |  |  |

R  Large residual  
X  Unusual X

## Backward Elimination of Terms

α to remove = 0,1

## Coded Coefficients

| Term | Coef | SE Coef | 95% CI | T-Value | P-Value | VIF |
| --- | --- | --- | --- | --- | --- | --- |
| Constant | 2,084 | 0,329 | (1,404; 2,764) | 6,33 | 0,000 |  |
| Lac | 1,390 | 0,521 | (0,315; 2,464) | 2,67 | 0,013 | 1,00 |
| Lac\*Lac | 2,346 | 0,988 | (0,307; 4,386) | 2,37 | 0,026 | 1,00 |

## Model Summary

| S | R-sq | R-sq(adj) | PRESS | R-sq(pred) | AICc | BIC |
| --- | --- | --- | --- | --- | --- | --- |
| 1,27567 | 34,71% | 29,27% | 45,7120 | 23,58% | 96,41 | 99,77 |

## Analysis of Variance

| Source | DF | Seq SS | Contribution | Adj SS | Adj MS | F-Value | P-Value |
| --- | --- | --- | --- | --- | --- | --- | --- |
| Model | 2 | 20,761 | 34,71% | 20,761 | 10,381 | 6,38 | 0,006 |
| Linear | 1 | 11,586 | 19,37% | 11,586 | 11,586 | 7,12 | 0,013 |
| Lac | 1 | 11,586 | 19,37% | 11,586 | 11,586 | 7,12 | 0,013 |
| Square | 1 | 9,175 | 15,34% | 9,175 | 9,175 | 5,64 | 0,026 |
| Lac\*Lac | 1 | 9,175 | 15,34% | 9,175 | 9,175 | 5,64 | 0,026 |
| Error | 24 | 39,056 | 65,29% | 39,056 | 1,627 |  |  |
| Lack-of-Fit | 22 | 28,605 | 47,82% | 28,605 | 1,300 | 0,25 | 0,967 |
| Pure Error | 2 | 10,451 | 17,47% | 10,451 | 5,225 |  |  |
| Total | 26 | 59,817 | 100,00% |  |  |  |  |

## Regression Equation in Uncoded Units

|  |  |  |
| --- | --- | --- |
| F\_SD\_3.5h(210min) | = | 8,69 - 32,0 Lac + 37,5 Lac\*Lac |

## Fits and Diagnostics for All Observations

| Obs | F\_SD\_3.5h(210min) | Fit | SE Fit | 95% CI | Resid | Std Resid | Del Resid | HI |
| --- | --- | --- | --- | --- | --- | --- | --- | --- |
| 1 | 2,522 | 1,976 | 0,359 | (1,235; 2,716) | 0,547 | 0,45 | 0,44 | 0,079167 |
| 2 | 3,262 | 3,365 | 0,359 | (2,624; 4,106) | -0,104 | -0,08 | -0,08 | 0,079167 |
| 3 | 1,694 | 1,976 | 0,359 | (1,235; 2,716) | -0,282 | -0,23 | -0,23 | 0,079167 |
| 4 | 0,947 | 3,365 | 0,359 | (2,624; 4,106) | -2,418 | -1,98 | -2,11 | 0,079167 |
| 5 | 2,736 | 1,976 | 0,359 | (1,235; 2,716) | 0,761 | 0,62 | 0,61 | 0,079167 |
| 6 | 5,756 | 3,365 | 0,359 | (2,624; 4,106) | 2,391 | 1,95 | 2,08 | 0,079167 |
| 7 | 1,729 | 1,976 | 0,359 | (1,235; 2,716) | -0,247 | -0,20 | -0,20 | 0,079167 |
| 8 | 2,304 | 3,365 | 0,359 | (2,624; 4,106) | -1,061 | -0,87 | -0,86 | 0,079167 |
| 9 | 1,695 | 1,976 | 0,359 | (1,235; 2,716) | -0,280 | -0,23 | -0,22 | 0,079167 |
| 10 | 2,498 | 3,365 | 0,359 | (2,624; 4,106) | -0,867 | -0,71 | -0,70 | 0,079167 |
| 11 | 0,934 | 1,976 | 0,359 | (1,235; 2,716) | -1,042 | -0,85 | -0,85 | 0,079167 |
| 12 | 1,818 | 3,365 | 0,359 | (2,624; 4,106) | -1,547 | -1,26 | -1,28 | 0,079167 |
| 13 | 1,470 | 1,976 | 0,359 | (1,235; 2,716) | -0,506 | -0,41 | -0,41 | 0,079167 |
| 14 | 5,236 | 3,365 | 0,359 | (2,624; 4,106) | 1,871 | 1,53 | 1,57 | 0,079167 |
| 15 | 2,553 | 1,976 | 0,359 | (1,235; 2,716) | 0,578 | 0,47 | 0,46 | 0,079167 |
| 16 | 4,551 | 3,365 | 0,359 | (2,624; 4,106) | 1,185 | 0,97 | 0,97 | 0,079167 |
| 17 | 3,148 | 3,040 | 0,960 | (1,058; 5,022) | 0,108 | 0,13 | 0,13 | 0,566667 |
| 18 | 5,967 | 5,820 | 0,960 | (3,838; 7,802) | 0,147 | 0,18 | 0,17 | 0,566667 |
| 19 | 1,110 | 2,084 | 0,329 | (1,404; 2,764) | -0,973 | -0,79 | -0,78 | 0,066667 |
| 20 | 3,635 | 2,084 | 0,329 | (1,404; 2,764) | 1,551 | 1,26 | 1,27 | 0,066667 |
| 21 | 2,104 | 2,084 | 0,329 | (1,404; 2,764) | 0,020 | 0,02 | 0,02 | 0,066667 |
| 22 | 1,898 | 2,084 | 0,329 | (1,404; 2,764) | -0,185 | -0,15 | -0,15 | 0,066667 |
| 23 | 1,445 | 2,084 | 0,329 | (1,404; 2,764) | -0,639 | -0,52 | -0,51 | 0,066667 |
| 24 | 1,463 | 2,084 | 0,329 | (1,404; 2,764) | -0,621 | -0,50 | -0,50 | 0,066667 |
| 25 | 0,199 | 2,084 | 0,329 | (1,404; 2,764) | -1,885 | -1,53 | -1,58 | 0,066667 |
| 26 | 4,741 | 2,084 | 0,329 | (1,404; 2,764) | 2,657 | 2,16 | 2,35 | 0,066667 |
| 27 | 2,925 | 2,084 | 0,329 | (1,404; 2,764) | 0,841 | 0,68 | 0,67 | 0,066667 |

| Obs | Cook’s D | DFITS |  |  |
| --- | --- | --- | --- | --- |
| 1 | 0,01 | 0,128796 |  |  |
| 2 | 0,00 | -0,024297 |  |  |
| 3 | 0,00 | -0,066106 |  |  |
| 4 | 0,11 | -0,619717 |  |  |
| 5 | 0,01 | 0,179804 |  |  |
| 6 | 0,11 | 0,611205 |  |  |
| 7 | 0,00 | -0,057963 |  |  |
| 8 | 0,02 | -0,252801 |  |  |
| 9 | 0,00 | -0,065775 |  |  |
| 10 | 0,01 | -0,205404 |  |  |
| 11 | 0,02 | -0,248044 |  |  |
| 12 | 0,05 | -0,375430 |  |  |
| 13 | 0,00 | -0,119073 |  |  |
| 14 | 0,07 | 0,461751 |  |  |
| 15 | 0,01 | 0,136135 |  |  |
| 16 | 0,03 | 0,283551 |  |  |
| 17 | 0,01 | 0,143897 |  | X |
| 18 | 0,01 | 0,196570 |  | X |
| 19 | 0,01 | -0,209401 |  |  |
| 20 | 0,04 | 0,340692 |  |  |
| 21 | 0,00 | 0,004285 |  |  |
| 22 | 0,00 | -0,039373 |  |  |
| 23 | 0,01 | -0,136448 |  |  |
| 24 | 0,01 | -0,132533 |  |  |
| 25 | 0,06 | -0,421146 |  |  |
| 26 | 0,11 | 0,628098 | R |  |
| 27 | 0,01 | 0,180399 |  |  |

R  Large residual  
X  Unusual X

## Backward Elimination of Terms

α to remove = 0,1

## Coded Coefficients

| Term | Coef | SE Coef | 95% CI | T-Value | P-Value | VIF |
| --- | --- | --- | --- | --- | --- | --- |
| Constant | 2,257 | 0,325 | (1,583; 2,931) | 6,94 | 0,000 |  |
| Lac | 1,613 | 0,529 | (0,515; 2,711) | 3,05 | 0,006 | 1,10 |
| HPMC\_HP | 0,645 | 0,500 | (-0,391; 1,682) | 1,29 | 0,210 | 1,00 |
| Lac\*Lac | 2,397 | 0,956 | (0,414; 4,381) | 2,51 | 0,020 | 1,00 |
| Lac\*HPMC\_HP | 2,19 | 1,22 | (-0,35; 4,72) | 1,79 | 0,087 | 1,10 |

## Model Summary

| S | R-sq | R-sq(adj) | PRESS | R-sq(pred) | AICc | BIC |
| --- | --- | --- | --- | --- | --- | --- |
| 1,23480 | 45,03% | 35,03% | 45,7641 | 25,00% | 98,68 | 102,26 |

## Analysis of Variance

| Source | DF | Seq SS | Contribution | Adj SS | Adj MS | F-Value | P-Value |
| --- | --- | --- | --- | --- | --- | --- | --- |
| Model | 4 | 27,476 | 45,03% | 27,476 | 6,869 | 4,51 | 0,008 |
| Linear | 2 | 13,011 | 21,32% | 16,698 | 8,349 | 5,48 | 0,012 |
| Lac | 1 | 10,513 | 17,23% | 14,155 | 14,155 | 9,28 | 0,006 |
| HPMC\_HP | 1 | 2,497 | 4,09% | 2,543 | 2,543 | 1,67 | 0,210 |
| Square | 1 | 9,577 | 15,70% | 9,577 | 9,577 | 6,28 | 0,020 |
| Lac\*Lac | 1 | 9,577 | 15,70% | 9,577 | 9,577 | 6,28 | 0,020 |
| 2-Way Interaction | 1 | 4,888 | 8,01% | 4,888 | 4,888 | 3,21 | 0,087 |
| Lac\*HPMC\_HP | 1 | 4,888 | 8,01% | 4,888 | 4,888 | 3,21 | 0,087 |
| Error | 22 | 33,544 | 54,97% | 33,544 | 1,525 |  |  |
| Lack-of-Fit | 20 | 23,328 | 38,23% | 23,328 | 1,166 | 0,23 | 0,974 |
| Pure Error | 2 | 10,216 | 16,74% | 10,216 | 5,108 |  |  |
| Total | 26 | 61,020 | 100,00% |  |  |  |  |

## Regression Equation in Uncoded Units

|  |  |  |
| --- | --- | --- |
| F\_SD\_4h(240min) | = | 43,5 - 113,6 Lac - 3,68 HPMC\_HP + 38,4 Lac\*Lac + 8,63 Lac\*HPMC\_HP |

## Fits and Diagnostics for All Observations

| Obs | F\_SD\_4h(240min) | Fit | SE Fit | 95% CI | Resid | Std Resid | Del Resid | HI |
| --- | --- | --- | --- | --- | --- | --- | --- | --- |
| 1 | 3,590 | 2,374 | 0,581 | (1,169; 3,579) | 1,216 | 1,12 | 1,12 | 0,221366 |
| 2 | 3,304 | 2,408 | 0,581 | (1,204; 3,613) | 0,895 | 0,82 | 0,82 | 0,221366 |
| 3 | 1,736 | 2,388 | 0,601 | (1,142; 3,633) | -0,651 | -0,60 | -0,59 | 0,236586 |
| 4 | 0,962 | 2,355 | 0,601 | (1,109; 3,601) | -1,393 | -1,29 | -1,31 | 0,236586 |
| 5 | 2,780 | 1,958 | 0,437 | (1,051; 2,865) | 0,822 | 0,71 | 0,70 | 0,125431 |
| 6 | 5,733 | 4,020 | 0,437 | (3,113; 4,927) | 1,714 | 1,48 | 1,53 | 0,125431 |
| 7 | 1,835 | 1,837 | 0,591 | (0,611; 3,063) | -0,001 | -0,00 | -0,00 | 0,229169 |
| 8 | 2,436 | 4,488 | 0,591 | (3,262; 5,714) | -2,052 | -1,89 | -2,02 | 0,229169 |
| 9 | 1,672 | 2,356 | 0,556 | (1,203; 3,508) | -0,684 | -0,62 | -0,61 | 0,202403 |
| 10 | 2,478 | 2,479 | 0,556 | (1,327; 3,631) | -0,001 | -0,00 | -0,00 | 0,202403 |
| 11 | 1,058 | 2,264 | 0,441 | (1,348; 3,179) | -1,205 | -1,05 | -1,05 | 0,127732 |
| 12 | 1,842 | 2,835 | 0,441 | (1,920; 3,750) | -0,993 | -0,86 | -0,86 | 0,127732 |
| 13 | 1,556 | 1,866 | 0,550 | (0,726; 3,006) | -0,310 | -0,28 | -0,27 | 0,198178 |
| 14 | 5,364 | 4,373 | 0,550 | (3,233; 5,513) | 0,991 | 0,90 | 0,89 | 0,198178 |
| 15 | 2,538 | 1,972 | 0,422 | (1,097; 2,848) | 0,565 | 0,49 | 0,48 | 0,116856 |
| 16 | 4,690 | 3,963 | 0,422 | (3,087; 4,838) | 0,728 | 0,63 | 0,62 | 0,116856 |
| 17 | 3,204 | 3,125 | 0,935 | (1,185; 5,065) | 0,079 | 0,10 | 0,10 | 0,573726 |
| 18 | 6,124 | 6,114 | 0,935 | (4,174; 8,053) | 0,011 | 0,01 | 0,01 | 0,573726 |
| 19 | 1,161 | 2,209 | 0,320 | (1,546; 2,873) | -1,048 | -0,88 | -0,87 | 0,067140 |
| 20 | 3,679 | 2,146 | 0,320 | (1,483; 2,809) | 1,533 | 1,29 | 1,31 | 0,066994 |
| 21 | 2,159 | 1,612 | 0,540 | (0,492; 2,732) | 0,547 | 0,49 | 0,48 | 0,191276 |
| 22 | 1,999 | 2,902 | 0,647 | (1,560; 4,245) | -0,903 | -0,86 | -0,85 | 0,274906 |
| 23 | 1,482 | 2,209 | 0,320 | (1,546; 2,873) | -0,727 | -0,61 | -0,60 | 0,067140 |
| 24 | 1,619 | 2,146 | 0,320 | (1,483; 2,809) | -0,527 | -0,44 | -0,43 | 0,066994 |
| 25 | 0,362 | 2,222 | 0,321 | (1,557; 2,888) | -1,860 | -1,56 | -1,62 | 0,067551 |
| 26 | 4,876 | 2,222 | 0,321 | (1,557; 2,888) | 2,654 | 2,23 | 2,47 | 0,067551 |
| 27 | 2,824 | 2,222 | 0,321 | (1,557; 2,888) | 0,602 | 0,51 | 0,50 | 0,067551 |

| Obs | Cook’s D | DFITS |  |  |
| --- | --- | --- | --- | --- |
| 1 | 0,07 | 0,59856 |  |  |
| 2 | 0,04 | 0,43468 |  |  |
| 3 | 0,02 | -0,33102 |  |  |
| 4 | 0,10 | -0,73037 |  |  |
| 5 | 0,01 | 0,26642 |  |  |
| 6 | 0,06 | 0,57881 |  |  |
| 7 | 0,00 | -0,00069 |  |  |
| 8 | 0,21 | -1,10218 |  |  |
| 9 | 0,02 | -0,30781 |  |  |
| 10 | 0,00 | -0,00041 |  |  |
| 11 | 0,03 | -0,40088 |  |  |
| 12 | 0,02 | -0,32751 |  |  |
| 13 | 0,00 | -0,13650 |  |  |
| 14 | 0,04 | 0,44358 |  |  |
| 15 | 0,01 | 0,17409 |  |  |
| 16 | 0,01 | 0,22486 |  |  |
| 17 | 0,00 | 0,11153 |  | X |
| 18 | 0,00 | 0,01508 |  | X |
| 19 | 0,01 | -0,23452 |  |  |
| 20 | 0,02 | 0,34986 |  |  |
| 21 | 0,01 | 0,23547 |  |  |
| 22 | 0,06 | -0,52559 |  |  |
| 23 | 0,01 | -0,16123 |  |  |
| 24 | 0,00 | -0,11619 |  |  |
| 25 | 0,04 | -0,43503 |  |  |
| 26 | 0,07 | 0,66487 | R |  |
| 27 | 0,00 | 0,13358 |  |  |

R  Large residual  
X  Unusual X

## Backward Elimination of Terms

α to remove = 0,1

## Coded Coefficients

| Term | Coef | SE Coef | 95% CI | T-Value | P-Value | VIF |
| --- | --- | --- | --- | --- | --- | --- |
| Constant | 2,317 | 0,324 | (1,646; 2,989) | 7,16 | 0,000 |  |
| Lac | 1,615 | 0,527 | (0,522; 2,708) | 3,06 | 0,006 | 1,10 |
| HPMC\_HP | 0,703 | 0,498 | (-0,329; 1,735) | 1,41 | 0,172 | 1,00 |
| Lac\*Lac | 2,465 | 0,952 | (0,490; 4,440) | 2,59 | 0,017 | 1,00 |
| Lac\*HPMC\_HP | 2,25 | 1,22 | (-0,27; 4,78) | 1,85 | 0,078 | 1,10 |

## Model Summary

| S | R-sq | R-sq(adj) | PRESS | R-sq(pred) | AICc | BIC |
| --- | --- | --- | --- | --- | --- | --- |
| 1,22955 | 46,30% | 36,54% | 45,5531 | 26,45% | 98,45 | 102,03 |

## Analysis of Variance

| Source | DF | Seq SS | Contribution | Adj SS | Adj MS | F-Value | P-Value |
| --- | --- | --- | --- | --- | --- | --- | --- |
| Model | 4 | 28,679 | 46,30% | 28,679 | 7,170 | 4,74 | 0,007 |
| Linear | 2 | 13,379 | 21,60% | 17,206 | 8,603 | 5,69 | 0,010 |
| Lac | 1 | 10,412 | 16,81% | 14,189 | 14,189 | 9,39 | 0,006 |
| HPMC\_HP | 1 | 2,967 | 4,79% | 3,017 | 3,017 | 2,00 | 0,172 |
| Square | 1 | 10,128 | 16,35% | 10,128 | 10,128 | 6,70 | 0,017 |
| Lac\*Lac | 1 | 10,128 | 16,35% | 10,128 | 10,128 | 6,70 | 0,017 |
| 2-Way Interaction | 1 | 5,173 | 8,35% | 5,173 | 5,173 | 3,42 | 0,078 |
| Lac\*HPMC\_HP | 1 | 5,173 | 8,35% | 5,173 | 5,173 | 3,42 | 0,078 |
| Error | 22 | 33,259 | 53,70% | 33,259 | 1,512 |  |  |
| Lack-of-Fit | 20 | 23,088 | 37,28% | 23,088 | 1,154 | 0,23 | 0,974 |
| Pure Error | 2 | 10,171 | 16,42% | 10,171 | 5,086 |  |  |
| Total | 26 | 61,939 | 100,00% |  |  |  |  |

## Regression Equation in Uncoded Units

|  |  |  |
| --- | --- | --- |
| F\_SD\_4.5h(270min) | = | 44,4 - 117,1 Lac - 3,74 HPMC\_HP + 39,4 Lac\*Lac + 8,87 Lac\*HPMC\_HP |

## Fits and Diagnostics for All Observations

| Obs | F\_SD\_4.5h(270min) | Fit | SE Fit | 95% CI | Resid | Std Resid | Del Resid | HI |
| --- | --- | --- | --- | --- | --- | --- | --- | --- |
| 1 | 3,719 | 2,431 | 0,578 | (1,231; 3,631) | 1,288 | 1,19 | 1,20 | 0,221366 |
| 2 | 3,300 | 2,422 | 0,578 | (1,223; 3,622) | 0,878 | 0,81 | 0,80 | 0,221366 |
| 3 | 1,817 | 2,444 | 0,598 | (1,204; 3,684) | -0,627 | -0,58 | -0,58 | 0,236586 |
| 4 | 0,927 | 2,366 | 0,598 | (1,126; 3,606) | -1,439 | -1,34 | -1,37 | 0,236586 |
| 5 | 2,851 | 2,039 | 0,435 | (1,136; 2,942) | 0,811 | 0,71 | 0,70 | 0,125431 |
| 6 | 5,701 | 4,116 | 0,435 | (3,213; 5,019) | 1,585 | 1,38 | 1,41 | 0,125431 |
| 7 | 2,055 | 1,925 | 0,589 | (0,705; 3,146) | 0,130 | 0,12 | 0,12 | 0,229169 |
| 8 | 2,561 | 4,609 | 0,589 | (3,388; 5,829) | -2,048 | -1,90 | -2,03 | 0,229169 |
| 9 | 1,609 | 2,414 | 0,553 | (1,267; 3,561) | -0,804 | -0,73 | -0,72 | 0,202403 |
| 10 | 2,514 | 2,497 | 0,553 | (1,350; 3,644) | 0,017 | 0,02 | 0,02 | 0,202403 |
| 11 | 1,195 | 2,327 | 0,439 | (1,416; 3,239) | -1,133 | -0,99 | -0,99 | 0,127732 |
| 12 | 1,899 | 2,871 | 0,439 | (1,959; 3,782) | -0,971 | -0,85 | -0,84 | 0,127732 |
| 13 | 1,622 | 1,953 | 0,547 | (0,818; 3,088) | -0,332 | -0,30 | -0,29 | 0,198178 |
| 14 | 5,475 | 4,488 | 0,547 | (3,352; 5,623) | 0,987 | 0,90 | 0,89 | 0,198178 |
| 15 | 2,481 | 2,053 | 0,420 | (1,181; 2,925) | 0,428 | 0,37 | 0,36 | 0,116856 |
| 16 | 4,851 | 4,056 | 0,420 | (3,184; 4,928) | 0,795 | 0,69 | 0,68 | 0,116856 |
| 17 | 3,317 | 3,251 | 0,931 | (1,320; 5,183) | 0,065 | 0,08 | 0,08 | 0,573726 |
| 18 | 6,281 | 6,237 | 0,931 | (4,306; 8,168) | 0,044 | 0,05 | 0,05 | 0,573726 |
| 19 | 1,209 | 2,265 | 0,319 | (1,604; 2,926) | -1,057 | -0,89 | -0,89 | 0,067140 |
| 20 | 3,713 | 2,196 | 0,318 | (1,536; 2,856) | 1,517 | 1,28 | 1,30 | 0,066994 |
| 21 | 2,151 | 1,614 | 0,538 | (0,499; 2,729) | 0,537 | 0,49 | 0,48 | 0,191276 |
| 22 | 2,102 | 3,020 | 0,645 | (1,683; 4,357) | -0,918 | -0,88 | -0,87 | 0,274906 |
| 23 | 1,513 | 2,265 | 0,319 | (1,604; 2,926) | -0,752 | -0,63 | -0,62 | 0,067140 |
| 24 | 1,738 | 2,196 | 0,318 | (1,536; 2,856) | -0,458 | -0,39 | -0,38 | 0,066994 |
| 25 | 0,501 | 2,279 | 0,320 | (1,616; 2,942) | -1,778 | -1,50 | -1,54 | 0,067551 |
| 26 | 5,011 | 2,279 | 0,320 | (1,616; 2,942) | 2,732 | 2,30 | 2,58 | 0,067551 |
| 27 | 2,782 | 2,279 | 0,320 | (1,616; 2,942) | 0,503 | 0,42 | 0,42 | 0,067551 |

| Obs | Cook’s D | DFITS |  |  |
| --- | --- | --- | --- | --- |
| 1 | 0,08 | 0,63937 |  |  |
| 2 | 0,04 | 0,42790 |  |  |
| 3 | 0,02 | -0,32015 |  |  |
| 4 | 0,11 | -0,76044 |  |  |
| 5 | 0,01 | 0,26408 |  |  |
| 6 | 0,05 | 0,53370 |  |  |
| 7 | 0,00 | 0,06400 |  |  |
| 8 | 0,21 | -1,10484 |  |  |
| 9 | 0,03 | -0,36506 |  |  |
| 10 | 0,00 | 0,00773 |  |  |
| 11 | 0,03 | -0,37724 |  |  |
| 12 | 0,02 | -0,32145 |  |  |
| 13 | 0,00 | -0,14666 |  |  |
| 14 | 0,04 | 0,44359 |  |  |
| 15 | 0,00 | 0,13215 |  |  |
| 16 | 0,01 | 0,24707 |  |  |
| 17 | 0,00 | 0,09189 |  | X |
| 18 | 0,00 | 0,06174 |  | X |
| 19 | 0,01 | -0,23750 |  |  |
| 20 | 0,02 | 0,34753 |  |  |
| 21 | 0,01 | 0,23188 |  |  |
| 22 | 0,06 | -0,53710 |  |  |
| 23 | 0,01 | -0,16752 |  |  |
| 24 | 0,00 | -0,10134 |  |  |
| 25 | 0,03 | -0,41548 |  |  |
| 26 | 0,08 | 0,69453 | R |  |
| 27 | 0,00 | 0,11188 |  |  |

R  Large residual  
X  Unusual X

## Backward Elimination of Terms

α to remove = 0,1

## Coded Coefficients

| Term | Coef | SE Coef | 95% CI | T-Value | P-Value | VIF |
| --- | --- | --- | --- | --- | --- | --- |
| Constant | 2,355 | 0,318 | (1,695; 3,015) | 7,40 | 0,000 |  |
| Lac | 1,690 | 0,518 | (0,616; 2,764) | 3,26 | 0,004 | 1,10 |
| HPMC\_HP | 0,760 | 0,489 | (-0,254; 1,773) | 1,55 | 0,134 | 1,00 |
| Lac\*Lac | 2,488 | 0,936 | (0,547; 4,428) | 2,66 | 0,014 | 1,00 |
| Lac\*HPMC\_HP | 2,26 | 1,20 | (-0,22; 4,75) | 1,89 | 0,072 | 1,10 |

## Model Summary

| S | R-sq | R-sq(adj) | PRESS | R-sq(pred) | AICc | BIC |
| --- | --- | --- | --- | --- | --- | --- |
| 1,20802 | 48,81% | 39,51% | 43,9632 | 29,91% | 97,50 | 101,07 |

## Analysis of Variance

| Source | DF | Seq SS | Contribution | Adj SS | Adj MS | F-Value | P-Value |
| --- | --- | --- | --- | --- | --- | --- | --- |
| Model | 4 | 30,616 | 48,81% | 30,616 | 7,654 | 5,24 | 0,004 |
| Linear | 2 | 15,072 | 24,03% | 19,061 | 9,531 | 6,53 | 0,006 |
| Lac | 1 | 11,603 | 18,50% | 15,537 | 15,537 | 10,65 | 0,004 |
| HPMC\_HP | 1 | 3,469 | 5,53% | 3,524 | 3,524 | 2,41 | 0,134 |
| Square | 1 | 10,313 | 16,44% | 10,313 | 10,313 | 7,07 | 0,014 |
| Lac\*Lac | 1 | 10,313 | 16,44% | 10,313 | 10,313 | 7,07 | 0,014 |
| 2-Way Interaction | 1 | 5,231 | 8,34% | 5,231 | 5,231 | 3,58 | 0,072 |
| Lac\*HPMC\_HP | 1 | 5,231 | 8,34% | 5,231 | 5,231 | 3,58 | 0,072 |
| Error | 22 | 32,105 | 51,19% | 32,105 | 1,459 |  |  |
| Lack-of-Fit | 20 | 22,273 | 35,51% | 22,273 | 1,114 | 0,23 | 0,974 |
| Pure Error | 2 | 9,832 | 15,68% | 9,832 | 4,916 |  |  |
| Total | 26 | 62,721 | 100,00% |  |  |  |  |

## Regression Equation in Uncoded Units

|  |  |  |
| --- | --- | --- |
| F\_SD\_5h(300min) | = | 44,1 - 117,6 Lac - 3,71 HPMC\_HP + 39,8 Lac\*Lac + 8,92 Lac\*HPMC\_HP |

## Fits and Diagnostics for All Observations

| Obs | F\_SD\_5h(300min) | Fit | SE Fit | 95% CI | Resid | Std Resid | Del Resid | HI |
| --- | --- | --- | --- | --- | --- | --- | --- | --- |
| 1 | 3,441 | 2,401 | 0,568 | (1,222; 3,580) | 1,040 | 0,98 | 0,97 | 0,221366 |
| 2 | 3,335 | 2,458 | 0,568 | (1,279; 3,637) | 0,877 | 0,82 | 0,82 | 0,221366 |
| 3 | 1,801 | 2,412 | 0,588 | (1,194; 3,631) | -0,611 | -0,58 | -0,57 | 0,236586 |
| 4 | 0,963 | 2,400 | 0,588 | (1,181; 3,618) | -1,437 | -1,36 | -1,39 | 0,236586 |
| 5 | 2,870 | 2,056 | 0,428 | (1,168; 2,943) | 0,814 | 0,72 | 0,71 | 0,125431 |
| 6 | 5,707 | 4,210 | 0,428 | (3,323; 5,097) | 1,497 | 1,33 | 1,35 | 0,125431 |
| 7 | 2,089 | 1,955 | 0,578 | (0,756; 3,155) | 0,134 | 0,13 | 0,12 | 0,229169 |
| 8 | 2,667 | 4,720 | 0,578 | (3,520; 5,919) | -2,052 | -1,93 | -2,08 | 0,229169 |
| 9 | 1,635 | 2,386 | 0,543 | (1,258; 3,513) | -0,751 | -0,70 | -0,69 | 0,202403 |
| 10 | 2,462 | 2,535 | 0,543 | (1,408; 3,662) | -0,073 | -0,07 | -0,07 | 0,202403 |
| 11 | 1,282 | 2,309 | 0,432 | (1,414; 3,205) | -1,027 | -0,91 | -0,91 | 0,127732 |
| 12 | 1,933 | 2,922 | 0,432 | (2,026; 3,817) | -0,989 | -0,88 | -0,87 | 0,127732 |
| 13 | 1,739 | 1,980 | 0,538 | (0,865; 3,095) | -0,241 | -0,22 | -0,22 | 0,198178 |
| 14 | 5,625 | 4,594 | 0,538 | (3,479; 5,710) | 1,030 | 0,95 | 0,95 | 0,198178 |
| 15 | 2,218 | 2,068 | 0,413 | (1,211; 2,924) | 0,150 | 0,13 | 0,13 | 0,116856 |
| 16 | 4,946 | 4,148 | 0,413 | (3,292; 5,004) | 0,798 | 0,70 | 0,69 | 0,116856 |
| 17 | 3,375 | 3,234 | 0,915 | (1,337; 5,132) | 0,141 | 0,18 | 0,17 | 0,573726 |
| 18 | 6,437 | 6,369 | 0,915 | (4,471; 8,266) | 0,069 | 0,09 | 0,09 | 0,573726 |
| 19 | 1,280 | 2,299 | 0,313 | (1,650; 2,948) | -1,018 | -0,87 | -0,87 | 0,067140 |
| 20 | 3,821 | 2,224 | 0,313 | (1,576; 2,873) | 1,597 | 1,37 | 1,40 | 0,066994 |
| 21 | 2,214 | 1,595 | 0,528 | (0,500; 2,691) | 0,618 | 0,57 | 0,56 | 0,191276 |
| 22 | 2,176 | 3,115 | 0,633 | (1,801; 4,428) | -0,939 | -0,91 | -0,91 | 0,274906 |
| 23 | 1,516 | 2,299 | 0,313 | (1,650; 2,948) | -0,783 | -0,67 | -0,66 | 0,067140 |
| 24 | 1,817 | 2,224 | 0,313 | (1,576; 2,873) | -0,408 | -0,35 | -0,34 | 0,066994 |
| 25 | 0,679 | 2,314 | 0,314 | (1,663; 2,965) | -1,635 | -1,40 | -1,43 | 0,067551 |
| 26 | 5,109 | 2,314 | 0,314 | (1,663; 2,965) | 2,795 | 2,40 | 2,72 | 0,067551 |
| 27 | 2,715 | 2,314 | 0,314 | (1,663; 2,965) | 0,401 | 0,34 | 0,34 | 0,067551 |

| Obs | Cook’s D | DFITS |  |  |
| --- | --- | --- | --- | --- |
| 1 | 0,05 | 0,51982 |  |  |
| 2 | 0,04 | 0,43556 |  |  |
| 3 | 0,02 | -0,31730 |  |  |
| 4 | 0,11 | -0,77351 |  |  |
| 5 | 0,01 | 0,26994 |  |  |
| 6 | 0,05 | 0,51124 |  |  |
| 7 | 0,00 | 0,06728 |  |  |
| 8 | 0,22 | -1,13155 |  |  |
| 9 | 0,02 | -0,34626 |  |  |
| 10 | 0,00 | -0,03321 |  |  |
| 11 | 0,02 | -0,34696 |  |  |
| 12 | 0,02 | -0,33354 |  |  |
| 13 | 0,00 | -0,10826 |  |  |
| 14 | 0,04 | 0,47243 |  |  |
| 15 | 0,00 | 0,04690 |  |  |
| 16 | 0,01 | 0,25264 |  |  |
| 17 | 0,01 | 0,20241 |  | X |
| 18 | 0,00 | 0,09903 |  | X |
| 19 | 0,01 | -0,23287 |  |  |
| 20 | 0,03 | 0,37465 |  |  |
| 21 | 0,02 | 0,27252 |  |  |
| 22 | 0,06 | -0,56001 |  |  |
| 23 | 0,01 | -0,17766 |  |  |
| 24 | 0,00 | -0,09170 |  |  |
| 25 | 0,03 | -0,38616 |  |  |
| 26 | 0,08 | 0,73290 | R |  |
| 27 | 0,00 | 0,09069 |  |  |

R  Large residual  
X  Unusual X

## Backward Elimination of Terms

α to remove = 0,1

## Coded Coefficients

| Term | Coef | SE Coef | 95% CI | T-Value | P-Value | VIF |
| --- | --- | --- | --- | --- | --- | --- |
| Constant | 2,406 | 0,317 | (1,749; 3,063) | 7,59 | 0,000 |  |
| Lac | 1,677 | 0,516 | (0,607; 2,747) | 3,25 | 0,004 | 1,10 |
| HPMC\_HP | 0,804 | 0,487 | (-0,206; 1,814) | 1,65 | 0,113 | 1,00 |
| Lac\*Lac | 2,523 | 0,932 | (0,590; 4,456) | 2,71 | 0,013 | 1,00 |
| Lac\*HPMC\_HP | 2,31 | 1,19 | (-0,16; 4,78) | 1,94 | 0,065 | 1,10 |

## Model Summary

| S | R-sq | R-sq(adj) | PRESS | R-sq(pred) | AICc | BIC |
| --- | --- | --- | --- | --- | --- | --- |
| 1,20330 | 49,51% | 40,33% | 43,8493 | 30,50% | 97,29 | 100,86 |

## Analysis of Variance

| Source | DF | Seq SS | Contribution | Adj SS | Adj MS | F-Value | P-Value |
| --- | --- | --- | --- | --- | --- | --- | --- |
| Model | 4 | 31,240 | 49,51% | 31,240 | 7,810 | 5,39 | 0,004 |
| Linear | 2 | 15,173 | 24,05% | 19,252 | 9,626 | 6,65 | 0,006 |
| Lac | 1 | 11,288 | 17,89% | 15,307 | 15,307 | 10,57 | 0,004 |
| HPMC\_HP | 1 | 3,885 | 6,16% | 3,944 | 3,944 | 2,72 | 0,113 |
| Square | 1 | 10,607 | 16,81% | 10,607 | 10,607 | 7,33 | 0,013 |
| Lac\*Lac | 1 | 10,607 | 16,81% | 10,607 | 10,607 | 7,33 | 0,013 |
| 2-Way Interaction | 1 | 5,460 | 8,65% | 5,460 | 5,460 | 3,77 | 0,065 |
| Lac\*HPMC\_HP | 1 | 5,460 | 8,65% | 5,460 | 5,460 | 3,77 | 0,065 |
| Error | 22 | 31,854 | 50,49% | 31,854 | 1,448 |  |  |
| Lack-of-Fit | 20 | 22,216 | 35,21% | 22,216 | 1,111 | 0,23 | 0,973 |
| Pure Error | 2 | 9,638 | 15,28% | 9,638 | 4,819 |  |  |
| Total | 26 | 63,094 | 100,00% |  |  |  |  |

## Regression Equation in Uncoded Units

|  |  |  |
| --- | --- | --- |
| F\_SD\_5.5h(330min) | = | 44,8 - 120,0 Lac - 3,77 HPMC\_HP + 40,4 Lac\*Lac + 9,12 Lac\*HPMC\_HP |

## Fits and Diagnostics for All Observations

| Obs | F\_SD\_5.5h(330min) | Fit | SE Fit | 95% CI | Resid | Std Resid | Del Resid | HI |
| --- | --- | --- | --- | --- | --- | --- | --- | --- |
| 1 | 3,526 | 2,453 | 0,566 | (1,279; 3,627) | 1,073 | 1,01 | 1,01 | 0,221366 |
| 2 | 3,308 | 2,462 | 0,566 | (1,288; 3,636) | 0,846 | 0,80 | 0,79 | 0,221366 |
| 3 | 1,841 | 2,464 | 0,585 | (1,250; 3,677) | -0,623 | -0,59 | -0,58 | 0,236586 |
| 4 | 0,944 | 2,402 | 0,585 | (1,188; 3,615) | -1,457 | -1,39 | -1,42 | 0,236586 |
| 5 | 2,963 | 2,126 | 0,426 | (1,242; 3,009) | 0,838 | 0,74 | 0,74 | 0,125431 |
| 6 | 5,682 | 4,278 | 0,426 | (3,394; 5,161) | 1,405 | 1,25 | 1,27 | 0,125431 |
| 7 | 2,209 | 2,031 | 0,576 | (0,836; 3,225) | 0,178 | 0,17 | 0,16 | 0,229169 |
| 8 | 2,783 | 4,806 | 0,576 | (3,611; 6,000) | -2,023 | -1,91 | -2,05 | 0,229169 |
| 9 | 1,594 | 2,438 | 0,541 | (1,316; 3,561) | -0,845 | -0,79 | -0,78 | 0,202403 |
| 10 | 2,466 | 2,542 | 0,541 | (1,419; 3,664) | -0,075 | -0,07 | -0,07 | 0,202403 |
| 11 | 1,341 | 2,366 | 0,430 | (1,474; 3,258) | -1,025 | -0,91 | -0,91 | 0,127732 |
| 12 | 1,952 | 2,942 | 0,430 | (2,050; 3,834) | -0,990 | -0,88 | -0,88 | 0,127732 |
| 13 | 1,815 | 2,054 | 0,536 | (0,943; 3,165) | -0,239 | -0,22 | -0,22 | 0,198178 |
| 14 | 5,753 | 4,676 | 0,536 | (3,565; 5,787) | 1,077 | 1,00 | 1,00 | 0,198178 |
| 15 | 2,137 | 2,137 | 0,411 | (1,284; 2,990) | -0,000 | -0,00 | -0,00 | 0,116856 |
| 16 | 4,968 | 4,213 | 0,411 | (3,360; 5,066) | 0,755 | 0,67 | 0,66 | 0,116856 |
| 17 | 3,517 | 3,333 | 0,911 | (1,443; 5,224) | 0,183 | 0,23 | 0,23 | 0,573726 |
| 18 | 6,530 | 6,437 | 0,911 | (4,547; 8,327) | 0,093 | 0,12 | 0,12 | 0,573726 |
| 19 | 1,439 | 2,347 | 0,312 | (1,700; 2,993) | -0,908 | -0,78 | -0,77 | 0,067140 |
| 20 | 3,843 | 2,267 | 0,311 | (1,622; 2,913) | 1,575 | 1,36 | 1,38 | 0,066994 |
| 21 | 2,282 | 1,602 | 0,526 | (0,511; 2,694) | 0,680 | 0,63 | 0,62 | 0,191276 |
| 22 | 2,258 | 3,210 | 0,631 | (1,901; 4,518) | -0,952 | -0,93 | -0,93 | 0,274906 |
| 23 | 1,448 | 2,347 | 0,312 | (1,700; 2,993) | -0,899 | -0,77 | -0,77 | 0,067140 |
| 24 | 1,969 | 2,267 | 0,311 | (1,622; 2,913) | -0,299 | -0,26 | -0,25 | 0,066994 |
| 25 | 0,837 | 2,362 | 0,313 | (1,714; 3,011) | -1,525 | -1,31 | -1,34 | 0,067551 |
| 26 | 5,209 | 2,362 | 0,313 | (1,714; 3,011) | 2,846 | 2,45 | 2,81 | 0,067551 |
| 27 | 2,672 | 2,362 | 0,313 | (1,714; 3,011) | 0,309 | 0,27 | 0,26 | 0,067551 |

| Obs | Cook’s D | DFITS |  |  |
| --- | --- | --- | --- | --- |
| 1 | 0,06 | 0,53922 |  |  |
| 2 | 0,04 | 0,42144 |  |  |
| 3 | 0,02 | -0,32471 |  |  |
| 4 | 0,12 | -0,78910 |  |  |
| 5 | 0,02 | 0,27895 |  |  |
| 6 | 0,04 | 0,47921 |  |  |
| 7 | 0,00 | 0,08986 |  |  |
| 8 | 0,22 | -1,11749 |  |  |
| 9 | 0,03 | -0,39240 |  |  |
| 10 | 0,00 | -0,03451 |  |  |
| 11 | 0,02 | -0,34776 |  |  |
| 12 | 0,02 | -0,33533 |  |  |
| 13 | 0,00 | -0,10771 |  |  |
| 14 | 0,05 | 0,49704 |  |  |
| 15 | 0,00 | -0,00011 |  |  |
| 16 | 0,01 | 0,23976 |  |  |
| 17 | 0,01 | 0,26478 |  | X |
| 18 | 0,00 | 0,13403 |  | X |
| 19 | 0,01 | -0,20759 |  |  |
| 20 | 0,03 | 0,37063 |  |  |
| 21 | 0,02 | 0,30119 |  |  |
| 22 | 0,07 | -0,57016 |  |  |
| 23 | 0,01 | -0,20550 |  |  |
| 24 | 0,00 | -0,06742 |  |  |
| 25 | 0,02 | -0,35955 |  |  |
| 26 | 0,09 | 0,75541 | R |  |
| 27 | 0,00 | 0,07013 |  |  |

R  Large residual  
X  Unusual X

## Backward Elimination of Terms

α to remove = 0,1

## Coded Coefficients

| Term | Coef | SE Coef | 95% CI | T-Value | P-Value | VIF |
| --- | --- | --- | --- | --- | --- | --- |
| Constant | 2,460 | 0,324 | (1,788; 3,132) | 7,59 | 0,000 |  |
| Lac | 1,666 | 0,527 | (0,572; 2,760) | 3,16 | 0,005 | 1,10 |
| HPMC\_HP | 0,840 | 0,498 | (-0,192; 1,873) | 1,69 | 0,106 | 1,00 |
| Lac\*Lac | 2,555 | 0,953 | (0,578; 4,531) | 2,68 | 0,014 | 1,00 |
| Lac\*HPMC\_HP | 2,48 | 1,22 | (-0,05; 5,01) | 2,04 | 0,054 | 1,10 |

## Model Summary

| S | R-sq | R-sq(adj) | PRESS | R-sq(pred) | AICc | BIC |
| --- | --- | --- | --- | --- | --- | --- |
| 1,23039 | 49,11% | 39,86% | 46,1478 | 29,49% | 98,49 | 102,06 |

## Analysis of Variance

| Source | DF | Seq SS | Contribution | Adj SS | Adj MS | F-Value | P-Value |
| --- | --- | --- | --- | --- | --- | --- | --- |
| Model | 4 | 32,146 | 49,11% | 32,146 | 8,037 | 5,31 | 0,004 |
| Linear | 2 | 14,995 | 22,91% | 19,412 | 9,706 | 6,41 | 0,006 |
| Lac | 1 | 10,743 | 16,41% | 15,099 | 15,099 | 9,97 | 0,005 |
| HPMC\_HP | 1 | 4,251 | 6,50% | 4,314 | 4,314 | 2,85 | 0,106 |
| Square | 1 | 10,877 | 16,62% | 10,877 | 10,877 | 7,18 | 0,014 |
| Lac\*Lac | 1 | 10,877 | 16,62% | 10,877 | 10,877 | 7,18 | 0,014 |
| 2-Way Interaction | 1 | 6,275 | 9,59% | 6,275 | 6,275 | 4,14 | 0,054 |
| Lac\*HPMC\_HP | 1 | 6,275 | 9,59% | 6,275 | 6,275 | 4,14 | 0,054 |
| Error | 22 | 33,305 | 50,89% | 33,305 | 1,514 |  |  |
| Lack-of-Fit | 20 | 23,478 | 35,87% | 23,478 | 1,174 | 0,24 | 0,970 |
| Pure Error | 2 | 9,827 | 15,01% | 9,827 | 4,913 |  |  |
| Total | 26 | 65,451 | 100,00% |  |  |  |  |

## Regression Equation in Uncoded Units

|  |  |  |
| --- | --- | --- |
| F\_SD\_6h(360min) | = | 47,8 - 126,8 Lac - 4,06 HPMC\_HP + 40,9 Lac\*Lac + 9,77 Lac\*HPMC\_HP |

## Fits and Diagnostics for All Observations

| Obs | F\_SD\_6h(360min) | Fit | SE Fit | 95% CI | Resid | Std Resid | Del Resid | HI |
| --- | --- | --- | --- | --- | --- | --- | --- | --- |
| 1 | 3,951 | 2,554 | 0,579 | (1,353; 3,754) | 1,397 | 1,29 | 1,31 | 0,221366 |
| 2 | 3,293 | 2,431 | 0,579 | (1,231; 3,632) | 0,861 | 0,79 | 0,79 | 0,221366 |
| 3 | 1,837 | 2,566 | 0,598 | (1,325; 3,807) | -0,729 | -0,68 | -0,67 | 0,236586 |
| 4 | 0,881 | 2,367 | 0,598 | (1,126; 3,609) | -1,486 | -1,38 | -1,41 | 0,236586 |
| 5 | 3,030 | 2,184 | 0,436 | (1,280; 3,087) | 0,846 | 0,74 | 0,73 | 0,125431 |
| 6 | 5,644 | 4,358 | 0,436 | (3,455; 5,262) | 1,286 | 1,12 | 1,12 | 0,125431 |
| 7 | 2,313 | 2,076 | 0,589 | (0,855; 3,298) | 0,237 | 0,22 | 0,21 | 0,229169 |
| 8 | 2,883 | 4,919 | 0,589 | (3,697; 6,140) | -2,036 | -1,88 | -2,01 | 0,229169 |
| 9 | 1,548 | 2,538 | 0,554 | (1,390; 3,685) | -0,990 | -0,90 | -0,90 | 0,202403 |
| 10 | 2,425 | 2,516 | 0,554 | (1,368; 3,664) | -0,091 | -0,08 | -0,08 | 0,202403 |
| 11 | 1,405 | 2,456 | 0,440 | (1,544; 3,368) | -1,051 | -0,91 | -0,91 | 0,127732 |
| 12 | 1,960 | 2,941 | 0,440 | (2,029; 3,853) | -0,981 | -0,85 | -0,85 | 0,127732 |
| 13 | 1,914 | 2,103 | 0,548 | (0,967; 3,238) | -0,189 | -0,17 | -0,17 | 0,198178 |
| 14 | 5,889 | 4,781 | 0,548 | (3,645; 5,917) | 1,108 | 1,01 | 1,01 | 0,198178 |
| 15 | 2,064 | 2,197 | 0,421 | (1,325; 3,069) | -0,133 | -0,12 | -0,11 | 0,116856 |
| 16 | 5,014 | 4,290 | 0,421 | (3,418; 5,162) | 0,724 | 0,63 | 0,62 | 0,116856 |
| 17 | 3,590 | 3,438 | 0,932 | (1,505; 5,370) | 0,152 | 0,19 | 0,19 | 0,573726 |
| 18 | 6,655 | 6,500 | 0,932 | (4,568; 8,433) | 0,154 | 0,19 | 0,19 | 0,573726 |
| 19 | 1,519 | 2,398 | 0,319 | (1,737; 3,059) | -0,879 | -0,74 | -0,73 | 0,067140 |
| 20 | 3,915 | 2,315 | 0,318 | (1,655; 2,975) | 1,600 | 1,35 | 1,37 | 0,066994 |
| 21 | 2,319 | 1,619 | 0,538 | (0,503; 2,735) | 0,699 | 0,63 | 0,62 | 0,191276 |
| 22 | 2,398 | 3,300 | 0,645 | (1,963; 4,638) | -0,902 | -0,86 | -0,86 | 0,274906 |
| 23 | 1,418 | 2,398 | 0,319 | (1,737; 3,059) | -0,980 | -0,82 | -0,82 | 0,067140 |
| 24 | 2,052 | 2,315 | 0,318 | (1,655; 2,975) | -0,263 | -0,22 | -0,22 | 0,066994 |
| 25 | 0,961 | 2,414 | 0,320 | (1,751; 3,078) | -1,454 | -1,22 | -1,24 | 0,067551 |
| 26 | 5,345 | 2,414 | 0,320 | (1,751; 3,078) | 2,930 | 2,47 | 2,83 | 0,067551 |
| 27 | 2,583 | 2,414 | 0,320 | (1,751; 3,078) | 0,168 | 0,14 | 0,14 | 0,067551 |

| Obs | Cook’s D | DFITS |  |  |
| --- | --- | --- | --- | --- |
| 1 | 0,09 | 0,69710 |  |  |
| 2 | 0,04 | 0,41929 |  |  |
| 3 | 0,03 | -0,37288 |  |  |
| 4 | 0,12 | -0,78703 |  |  |
| 5 | 0,02 | 0,27557 |  |  |
| 6 | 0,04 | 0,42572 |  |  |
| 7 | 0,00 | 0,11679 |  |  |
| 8 | 0,21 | -1,09637 |  |  |
| 9 | 0,04 | -0,45165 |  |  |
| 10 | 0,00 | -0,04082 |  |  |
| 11 | 0,02 | -0,34859 |  |  |
| 12 | 0,02 | -0,32456 |  |  |
| 13 | 0,00 | -0,08327 |  |  |
| 14 | 0,05 | 0,50005 |  |  |
| 15 | 0,00 | -0,04094 |  |  |
| 16 | 0,01 | 0,22461 |  |  |
| 17 | 0,01 | 0,21529 |  | X |
| 18 | 0,01 | 0,21783 |  | X |
| 19 | 0,01 | -0,19633 |  |  |
| 20 | 0,03 | 0,36788 |  |  |
| 21 | 0,02 | 0,30298 |  |  |
| 22 | 0,06 | -0,52703 |  |  |
| 23 | 0,01 | -0,21957 |  |  |
| 24 | 0,00 | -0,05790 |  |  |
| 25 | 0,02 | -0,33332 |  |  |
| 26 | 0,09 | 0,76253 | R |  |
| 27 | 0,00 | 0,03730 |  |  |

R  Large residual  
X  Unusual X

## Backward Elimination of Terms

α to remove = 0,1

## Coded Coefficients

| Term | Coef | SE Coef | 95% CI | T-Value | P-Value | VIF |
| --- | --- | --- | --- | --- | --- | --- |
| Constant | 2,534 | 0,338 | (1,833; 3,235) | 7,49 | 0,000 |  |
| Lac | 1,634 | 0,551 | (0,492; 2,776) | 2,97 | 0,007 | 1,10 |
| HPMC\_HP | 0,879 | 0,520 | (-0,198; 1,957) | 1,69 | 0,105 | 1,00 |
| Lac\*Lac | 2,645 | 0,995 | (0,582; 4,708) | 2,66 | 0,014 | 1,00 |
| Lac\*HPMC\_HP | 2,69 | 1,27 | (0,06; 5,33) | 2,12 | 0,046 | 1,10 |

## Model Summary

| S | R-sq | R-sq(adj) | PRESS | R-sq(pred) | AICc | BIC |
| --- | --- | --- | --- | --- | --- | --- |
| 1,28428 | 48,02% | 38,57% | 50,9054 | 27,08% | 100,80 | 104,38 |

## Analysis of Variance

| Source | DF | Seq SS | Contribution | Adj SS | Adj MS | F-Value | P-Value |
| --- | --- | --- | --- | --- | --- | --- | --- |
| Model | 4 | 33,520 | 48,02% | 33,520 | 8,380 | 5,08 | 0,005 |
| Linear | 2 | 14,460 | 20,71% | 19,253 | 9,627 | 5,84 | 0,009 |
| Lac | 1 | 9,805 | 14,05% | 14,530 | 14,530 | 8,81 | 0,007 |
| HPMC\_HP | 1 | 4,655 | 6,67% | 4,723 | 4,723 | 2,86 | 0,105 |
| Square | 1 | 11,661 | 16,70% | 11,661 | 11,661 | 7,07 | 0,014 |
| Lac\*Lac | 1 | 11,661 | 16,70% | 11,661 | 11,661 | 7,07 | 0,014 |
| 2-Way Interaction | 1 | 7,399 | 10,60% | 7,399 | 7,399 | 4,49 | 0,046 |
| Lac\*HPMC\_HP | 1 | 7,399 | 10,60% | 7,399 | 7,399 | 4,49 | 0,046 |
| Error | 22 | 36,287 | 51,98% | 36,287 | 1,649 |  |  |
| Lack-of-Fit | 20 | 26,283 | 37,65% | 26,283 | 1,314 | 0,26 | 0,960 |
| Pure Error | 2 | 10,003 | 14,33% | 10,003 | 5,002 |  |  |
| Total | 26 | 69,806 | 100,00% |  |  |  |  |

## Regression Equation in Uncoded Units

|  |  |  |
| --- | --- | --- |
| F\_SD\_7h(420min) | = | 51,9 - 136,4 Lac - 4,44 HPMC\_HP + 42,3 Lac\*Lac + 10,61 Lac\*HPMC\_HP |

## Fits and Diagnostics for All Observations

| Obs | F\_SD\_7h(420min) | Fit | SE Fit | 95% CI | Resid | Std Resid | Del Resid | HI |
| --- | --- | --- | --- | --- | --- | --- | --- | --- |
| 1 | 4,518 | 2,715 | 0,604 | (1,462; 3,968) | 1,803 | 1,59 | 1,65 | 0,221366 |
| 2 | 3,309 | 2,407 | 0,604 | (1,154; 3,660) | 0,901 | 0,80 | 0,79 | 0,221366 |
| 3 | 1,805 | 2,729 | 0,625 | (1,434; 4,025) | -0,925 | -0,82 | -0,82 | 0,236586 |
| 4 | 0,785 | 2,339 | 0,625 | (1,043; 3,634) | -1,553 | -1,38 | -1,42 | 0,236586 |
| 5 | 3,156 | 2,282 | 0,455 | (1,339; 3,226) | 0,873 | 0,73 | 0,72 | 0,125431 |
| 6 | 5,532 | 4,469 | 0,455 | (3,526; 5,412) | 1,063 | 0,89 | 0,88 | 0,125431 |
| 7 | 2,492 | 2,156 | 0,615 | (0,881; 3,432) | 0,335 | 0,30 | 0,29 | 0,229169 |
| 8 | 3,044 | 5,069 | 0,615 | (3,794; 6,344) | -2,024 | -1,80 | -1,90 | 0,229169 |
| 9 | 1,507 | 2,696 | 0,578 | (1,498; 3,894) | -1,189 | -1,04 | -1,04 | 0,202403 |
| 10 | 2,345 | 2,498 | 0,578 | (1,300; 3,696) | -0,153 | -0,13 | -0,13 | 0,202403 |
| 11 | 1,564 | 2,600 | 0,459 | (1,649; 3,552) | -1,036 | -0,86 | -0,86 | 0,127732 |
| 12 | 2,022 | 2,953 | 0,459 | (2,001; 3,905) | -0,931 | -0,78 | -0,77 | 0,127732 |
| 13 | 2,157 | 2,187 | 0,572 | (1,002; 3,373) | -0,030 | -0,03 | -0,03 | 0,198178 |
| 14 | 6,144 | 4,921 | 0,572 | (3,736; 6,107) | 1,223 | 1,06 | 1,07 | 0,198178 |
| 15 | 1,815 | 2,298 | 0,439 | (1,387; 3,208) | -0,483 | -0,40 | -0,39 | 0,116856 |
| 16 | 5,112 | 4,396 | 0,439 | (3,486; 5,307) | 0,716 | 0,59 | 0,58 | 0,116856 |
| 17 | 3,792 | 3,643 | 0,973 | (1,626; 5,661) | 0,149 | 0,18 | 0,17 | 0,573726 |
| 18 | 6,822 | 6,620 | 0,973 | (4,602; 8,637) | 0,203 | 0,24 | 0,24 | 0,573726 |
| 19 | 1,585 | 2,469 | 0,333 | (1,779; 3,159) | -0,884 | -0,71 | -0,70 | 0,067140 |
| 20 | 3,927 | 2,382 | 0,332 | (1,693; 3,072) | 1,545 | 1,25 | 1,26 | 0,066994 |
| 21 | 2,408 | 1,654 | 0,562 | (0,490; 2,819) | 0,753 | 0,65 | 0,64 | 0,191276 |
| 22 | 2,493 | 3,413 | 0,673 | (2,017; 4,810) | -0,920 | -0,84 | -0,84 | 0,274906 |
| 23 | 1,290 | 2,469 | 0,333 | (1,779; 3,159) | -1,179 | -0,95 | -0,95 | 0,067140 |
| 24 | 2,248 | 2,382 | 0,332 | (1,693; 3,072) | -0,134 | -0,11 | -0,11 | 0,066994 |
| 25 | 1,261 | 2,486 | 0,334 | (1,794; 3,179) | -1,225 | -0,99 | -0,99 | 0,067551 |
| 26 | 5,597 | 2,486 | 0,334 | (1,794; 3,179) | 3,110 | 2,51 | 2,90 | 0,067551 |
| 27 | 2,476 | 2,486 | 0,334 | (1,794; 3,179) | -0,010 | -0,01 | -0,01 | 0,067551 |

| Obs | Cook’s D | DFITS |  |  |
| --- | --- | --- | --- | --- |
| 1 | 0,14 | 0,88122 |  |  |
| 2 | 0,04 | 0,42046 |  |  |
| 3 | 0,04 | -0,45529 |  |  |
| 4 | 0,12 | -0,78803 |  |  |
| 5 | 0,02 | 0,27235 |  |  |
| 6 | 0,02 | 0,33353 |  |  |
| 7 | 0,01 | 0,15875 |  |  |
| 8 | 0,19 | -1,03530 |  |  |
| 9 | 0,05 | -0,52301 |  |  |
| 10 | 0,00 | -0,06553 |  |  |
| 11 | 0,02 | -0,32855 |  |  |
| 12 | 0,02 | -0,29427 |  |  |
| 13 | 0,00 | -0,01271 |  |  |
| 14 | 0,06 | 0,53048 |  |  |
| 15 | 0,00 | -0,14272 |  |  |
| 16 | 0,01 | 0,21260 |  |  |
| 17 | 0,01 | 0,20188 |  | X |
| 18 | 0,02 | 0,27443 |  | X |
| 19 | 0,01 | -0,18906 |  |  |
| 20 | 0,02 | 0,33822 |  |  |
| 21 | 0,02 | 0,31290 |  |  |
| 22 | 0,05 | -0,51439 |  |  |
| 23 | 0,01 | -0,25447 |  |  |
| 24 | 0,00 | -0,02826 |  |  |
| 25 | 0,01 | -0,26574 |  |  |
| 26 | 0,09 | 0,78048 | R |  |
| 27 | 0,00 | -0,00214 |  |  |

R  Large residual  
X  Unusual X

## Backward Elimination of Terms

α to remove = 0,1

## Coded Coefficients

| Term | Coef | SE Coef | 95% CI | T-Value | P-Value | VIF |
| --- | --- | --- | --- | --- | --- | --- |
| Constant | 2,603 | 0,349 | (1,880; 3,326) | 7,46 | 0,000 |  |
| Lac | 1,508 | 0,568 | (0,331; 2,685) | 2,66 | 0,014 | 1,10 |
| HPMC\_HP | 1,035 | 0,536 | (-0,077; 2,146) | 1,93 | 0,067 | 1,00 |
| Lac\*Lac | 2,74 | 1,03 | (0,61; 4,86) | 2,67 | 0,014 | 1,00 |
| Lac\*HPMC\_HP | 2,56 | 1,31 | (-0,16; 5,28) | 1,95 | 0,063 | 1,10 |

## Model Summary

| S | R-sq | R-sq(adj) | PRESS | R-sq(pred) | AICc | BIC |
| --- | --- | --- | --- | --- | --- | --- |
| 1,32410 | 46,72% | 37,04% | 53,9068 | 25,54% | 102,45 | 106,03 |

## Analysis of Variance

| Source | DF | Seq SS | Contribution | Adj SS | Adj MS | F-Value | P-Value |
| --- | --- | --- | --- | --- | --- | --- | --- |
| Model | 4 | 33,827 | 46,72% | 33,827 | 8,457 | 4,82 | 0,006 |
| Linear | 2 | 14,658 | 20,25% | 18,908 | 9,454 | 5,39 | 0,012 |
| Lac | 1 | 8,205 | 11,33% | 12,372 | 12,372 | 7,06 | 0,014 |
| HPMC\_HP | 1 | 6,453 | 8,91% | 6,536 | 6,536 | 3,73 | 0,067 |
| Square | 1 | 12,472 | 17,23% | 12,472 | 12,472 | 7,11 | 0,014 |
| Lac\*Lac | 1 | 12,472 | 17,23% | 12,472 | 12,472 | 7,11 | 0,014 |
| 2-Way Interaction | 1 | 6,697 | 9,25% | 6,697 | 6,697 | 3,82 | 0,063 |
| Lac\*HPMC\_HP | 1 | 6,697 | 9,25% | 6,697 | 6,697 | 3,82 | 0,063 |
| Error | 22 | 38,571 | 53,28% | 38,571 | 1,753 |  |  |
| Lack-of-Fit | 20 | 27,296 | 37,70% | 27,296 | 1,365 | 0,24 | 0,968 |
| Pure Error | 2 | 11,275 | 15,57% | 11,275 | 5,638 |  |  |
| Total | 26 | 72,398 | 100,00% |  |  |  |  |

## Regression Equation in Uncoded Units

|  |  |  |
| --- | --- | --- |
| F\_SD\_8h(480min) | = | 48,7 - 133,4 Lac - 4,03 HPMC\_HP + 43,8 Lac\*Lac + 10,10 Lac\*HPMC\_HP |

## Fits and Diagnostics for All Observations

| Obs | F\_SD\_8h(480min) | Fit | SE Fit | 95% CI | Resid | Std Resid | Del Resid | HI |
| --- | --- | --- | --- | --- | --- | --- | --- | --- |
| 1 | 4,534 | 2,710 | 0,623 | (1,418; 4,002) | 1,824 | 1,56 | 1,62 | 0,221366 |
| 2 | 3,293 | 2,371 | 0,623 | (1,079; 3,663) | 0,922 | 0,79 | 0,78 | 0,221366 |
| 3 | 1,772 | 2,718 | 0,644 | (1,382; 4,054) | -0,946 | -0,82 | -0,81 | 0,236586 |
| 4 | 0,718 | 2,300 | 0,644 | (0,964; 3,635) | -1,581 | -1,37 | -1,40 | 0,236586 |
| 5 | 3,381 | 2,482 | 0,469 | (1,509; 3,454) | 0,899 | 0,73 | 0,72 | 0,125431 |
| 6 | 5,531 | 4,516 | 0,469 | (3,543; 5,488) | 1,016 | 0,82 | 0,81 | 0,125431 |
| 7 | 2,936 | 2,416 | 0,634 | (1,101; 3,730) | 0,521 | 0,45 | 0,44 | 0,229169 |
| 8 | 3,155 | 5,139 | 0,634 | (3,825; 6,454) | -1,985 | -1,71 | -1,79 | 0,229169 |
| 9 | 1,536 | 2,700 | 0,596 | (1,465; 3,936) | -1,164 | -0,98 | -0,98 | 0,202403 |
| 10 | 2,237 | 2,465 | 0,596 | (1,230; 3,701) | -0,228 | -0,19 | -0,19 | 0,202403 |
| 11 | 1,582 | 2,650 | 0,473 | (1,668; 3,631) | -1,068 | -0,86 | -0,86 | 0,127732 |
| 12 | 1,985 | 2,938 | 0,473 | (1,957; 3,920) | -0,953 | -0,77 | -0,76 | 0,127732 |
| 13 | 2,425 | 2,432 | 0,589 | (1,209; 3,654) | -0,007 | -0,01 | -0,01 | 0,198178 |
| 14 | 6,267 | 4,986 | 0,589 | (3,763; 6,208) | 1,281 | 1,08 | 1,08 | 0,198178 |
| 15 | 1,849 | 2,490 | 0,453 | (1,551; 3,429) | -0,641 | -0,51 | -0,51 | 0,116856 |
| 16 | 5,074 | 4,440 | 0,453 | (3,501; 5,378) | 0,635 | 0,51 | 0,50 | 0,116856 |
| 17 | 4,020 | 3,913 | 1,003 | (1,833; 5,993) | 0,106 | 0,12 | 0,12 | 0,573726 |
| 18 | 6,914 | 6,651 | 1,003 | (4,571; 8,731) | 0,263 | 0,30 | 0,30 | 0,573726 |
| 19 | 1,594 | 2,526 | 0,343 | (1,815; 3,238) | -0,932 | -0,73 | -0,72 | 0,067140 |
| 20 | 3,879 | 2,424 | 0,343 | (1,713; 3,135) | 1,455 | 1,14 | 1,15 | 0,066994 |
| 21 | 2,471 | 1,568 | 0,579 | (0,367; 2,769) | 0,903 | 0,76 | 0,75 | 0,191276 |
| 22 | 2,708 | 3,637 | 0,694 | (2,197; 5,077) | -0,930 | -0,82 | -0,82 | 0,274906 |
| 23 | 1,183 | 2,526 | 0,343 | (1,815; 3,238) | -1,343 | -1,05 | -1,05 | 0,067140 |
| 24 | 2,472 | 2,424 | 0,343 | (1,713; 3,135) | 0,047 | 0,04 | 0,04 | 0,066994 |
| 25 | 1,361 | 2,547 | 0,344 | (1,833; 3,260) | -1,186 | -0,93 | -0,92 | 0,067551 |
| 26 | 5,868 | 2,547 | 0,344 | (1,833; 3,260) | 3,321 | 2,60 | 3,05 | 0,067551 |
| 27 | 2,319 | 2,547 | 0,344 | (1,833; 3,260) | -0,228 | -0,18 | -0,17 | 0,067551 |

| Obs | Cook’s D | DFITS |  |  |
| --- | --- | --- | --- | --- |
| 1 | 0,14 | 0,862427 |  |  |
| 2 | 0,04 | 0,416925 |  |  |
| 3 | 0,04 | -0,451765 |  |  |
| 4 | 0,12 | -0,777147 |  |  |
| 5 | 0,02 | 0,271911 |  |  |
| 6 | 0,02 | 0,308211 |  |  |
| 7 | 0,01 | 0,239807 |  |  |
| 8 | 0,17 | -0,976374 |  |  |
| 9 | 0,05 | -0,495693 |  |  |
| 10 | 0,00 | -0,095064 |  |  |
| 11 | 0,02 | -0,328491 |  |  |
| 12 | 0,02 | -0,292118 |  |  |
| 13 | 0,00 | -0,002759 |  |  |
| 14 | 0,06 | 0,539126 |  |  |
| 15 | 0,01 | -0,184081 |  |  |
| 16 | 0,01 | 0,182322 |  |  |
| 17 | 0,00 | 0,139606 |  | X |
| 18 | 0,02 | 0,345177 |  | X |
| 19 | 0,01 | -0,193351 |  |  |
| 20 | 0,02 | 0,306892 |  |  |
| 21 | 0,03 | 0,365210 |  |  |
| 22 | 0,05 | -0,503834 |  |  |
| 23 | 0,02 | -0,282489 |  |  |
| 24 | 0,00 | 0,009682 |  |  |
| 25 | 0,01 | -0,248828 |  |  |
| 26 | 0,10 | 0,820307 | R |  |
| 27 | 0,00 | -0,046885 |  |  |

R  Large residual  
X  Unusual X

## Backward Elimination of Terms

α to remove = 0,1

## Coded Coefficients

| Term | Coef | SE Coef | 95% CI | T-Value | P-Value | VIF |
| --- | --- | --- | --- | --- | --- | --- |
| Constant | 2,563 | 0,344 | (1,850; 3,277) | 7,45 | 0,000 |  |
| Lac | 1,570 | 0,560 | (0,408; 2,731) | 2,80 | 0,010 | 1,10 |
| HPMC\_HP | 1,205 | 0,529 | (0,109; 2,302) | 2,28 | 0,033 | 1,00 |
| Lac\*Lac | 3,13 | 1,01 | (1,04; 5,23) | 3,10 | 0,005 | 1,00 |
| Lac\*HPMC\_HP | 2,34 | 1,29 | (-0,34; 5,03) | 1,81 | 0,084 | 1,10 |

## Model Summary

| S | R-sq | R-sq(adj) | PRESS | R-sq(pred) | AICc | BIC |
| --- | --- | --- | --- | --- | --- | --- |
| 1,30619 | 51,76% | 42,98% | 52,4918 | 32,53% | 101,72 | 105,29 |

## Analysis of Variance

| Source | DF | Seq SS | Contribution | Adj SS | Adj MS | F-Value | P-Value |
| --- | --- | --- | --- | --- | --- | --- | --- |
| Model | 4 | 40,267 | 51,76% | 40,267 | 10,067 | 5,90 | 0,002 |
| Linear | 2 | 18,290 | 23,51% | 22,281 | 11,140 | 6,53 | 0,006 |
| Lac | 1 | 9,527 | 12,24% | 13,407 | 13,407 | 7,86 | 0,010 |
| HPMC\_HP | 1 | 8,763 | 11,26% | 8,874 | 8,874 | 5,20 | 0,033 |
| Square | 1 | 16,373 | 21,05% | 16,373 | 16,373 | 9,60 | 0,005 |
| Lac\*Lac | 1 | 16,373 | 21,05% | 16,373 | 16,373 | 9,60 | 0,005 |
| 2-Way Interaction | 1 | 5,604 | 7,20% | 5,604 | 5,604 | 3,28 | 0,084 |
| Lac\*HPMC\_HP | 1 | 5,604 | 7,20% | 5,604 | 5,604 | 3,28 | 0,084 |
| Error | 22 | 37,535 | 48,24% | 37,535 | 1,706 |  |  |
| Lack-of-Fit | 20 | 25,836 | 33,21% | 25,836 | 1,292 | 0,22 | 0,976 |
| Pure Error | 2 | 11,699 | 15,04% | 11,699 | 5,849 |  |  |
| Total | 26 | 77,802 | 100,00% |  |  |  |  |

## Regression Equation in Uncoded Units

|  |  |  |
| --- | --- | --- |
| F\_SD\_9h(540min) | = | 44,5 - 131,4 Lac - 3,43 HPMC\_HP + 50,1 Lac\*Lac + 9,24 Lac\*HPMC\_HP |

## Fits and Diagnostics for All Observations

| Obs | F\_SD\_9h(540min) | Fit | SE Fit | 95% CI | Resid | Std Resid | Del Resid | HI |
| --- | --- | --- | --- | --- | --- | --- | --- | --- |
| 1 | 3,660 | 2,538 | 0,615 | (1,264; 3,813) | 1,122 | 0,97 | 0,97 | 0,221366 |
| 2 | 3,337 | 2,418 | 0,615 | (1,143; 3,692) | 0,919 | 0,80 | 0,79 | 0,221366 |
| 3 | 1,761 | 2,537 | 0,635 | (1,219; 3,855) | -0,776 | -0,68 | -0,67 | 0,236586 |
| 4 | 0,705 | 2,345 | 0,635 | (1,027; 3,662) | -1,640 | -1,44 | -1,48 | 0,236586 |
| 5 | 3,461 | 2,569 | 0,463 | (1,610; 3,529) | 0,892 | 0,73 | 0,72 | 0,125431 |
| 6 | 5,484 | 4,620 | 0,463 | (3,660; 5,579) | 0,864 | 0,71 | 0,70 | 0,125431 |
| 7 | 2,825 | 2,578 | 0,625 | (1,281; 3,875) | 0,247 | 0,22 | 0,21 | 0,229169 |
| 8 | 3,231 | 5,260 | 0,625 | (3,963; 6,557) | -2,029 | -1,77 | -1,87 | 0,229169 |
| 9 | 1,587 | 2,539 | 0,588 | (1,321; 3,758) | -0,953 | -0,82 | -0,81 | 0,202403 |
| 10 | 2,141 | 2,515 | 0,588 | (1,296; 3,733) | -0,374 | -0,32 | -0,31 | 0,202403 |
| 11 | 1,635 | 2,546 | 0,467 | (1,578; 3,514) | -0,912 | -0,75 | -0,74 | 0,127732 |
| 12 | 1,858 | 3,001 | 0,467 | (2,032; 3,969) | -1,142 | -0,94 | -0,93 | 0,127732 |
| 13 | 2,777 | 2,576 | 0,581 | (1,370; 3,782) | 0,201 | 0,17 | 0,17 | 0,198178 |
| 14 | 6,394 | 5,103 | 0,581 | (3,897; 6,309) | 1,291 | 1,10 | 1,11 | 0,198178 |
| 15 | 1,943 | 2,568 | 0,447 | (1,642; 3,494) | -0,625 | -0,51 | -0,50 | 0,116856 |
| 16 | 4,944 | 4,542 | 0,447 | (3,616; 5,468) | 0,402 | 0,33 | 0,32 | 0,116856 |
| 17 | 4,278 | 4,190 | 0,989 | (2,138; 6,242) | 0,088 | 0,10 | 0,10 | 0,573726 |
| 18 | 7,616 | 7,075 | 0,989 | (5,023; 9,127) | 0,541 | 0,63 | 0,63 | 0,573726 |
| 19 | 1,521 | 2,474 | 0,338 | (1,773; 3,176) | -0,954 | -0,76 | -0,75 | 0,067140 |
| 20 | 3,769 | 2,356 | 0,338 | (1,655; 3,057) | 1,413 | 1,12 | 1,13 | 0,066994 |
| 21 | 2,593 | 1,358 | 0,571 | (0,173; 2,543) | 1,235 | 1,05 | 1,05 | 0,191276 |
| 22 | 2,951 | 3,769 | 0,685 | (2,349; 5,189) | -0,818 | -0,74 | -0,73 | 0,274906 |
| 23 | 1,019 | 2,474 | 0,338 | (1,773; 3,176) | -1,456 | -1,15 | -1,16 | 0,067140 |
| 24 | 2,552 | 2,356 | 0,338 | (1,655; 3,057) | 0,196 | 0,16 | 0,15 | 0,066994 |
| 25 | 1,502 | 2,498 | 0,339 | (1,794; 3,202) | -0,997 | -0,79 | -0,78 | 0,067551 |
| 26 | 6,014 | 2,498 | 0,339 | (1,794; 3,202) | 3,515 | 2,79 | 3,39 | 0,067551 |
| 27 | 2,248 | 2,498 | 0,339 | (1,794; 3,202) | -0,251 | -0,20 | -0,19 | 0,067551 |

| Obs | Cook’s D | DFITS |  |  |
| --- | --- | --- | --- | --- |
| 1 | 0,05 | 0,51847 |  |  |
| 2 | 0,04 | 0,42137 |  |  |
| 3 | 0,03 | -0,37366 |  |  |
| 4 | 0,13 | -0,82112 |  |  |
| 5 | 0,02 | 0,27346 |  |  |
| 6 | 0,01 | 0,26479 |  |  |
| 7 | 0,00 | 0,11482 |  |  |
| 8 | 0,19 | -1,01799 |  |  |
| 9 | 0,03 | -0,40816 |  |  |
| 10 | 0,01 | -0,15822 |  |  |
| 11 | 0,02 | -0,28304 |  |  |
| 12 | 0,03 | -0,35732 |  |  |
| 13 | 0,00 | 0,08335 |  |  |
| 14 | 0,06 | 0,55162 |  |  |
| 15 | 0,01 | -0,18203 |  |  |
| 16 | 0,00 | 0,11671 |  |  |
| 17 | 0,00 | 0,11652 |  | X |
| 18 | 0,11 | 0,72548 |  | X |
| 19 | 0,01 | -0,20081 |  |  |
| 20 | 0,02 | 0,30202 |  |  |
| 21 | 0,05 | 0,51272 |  |  |
| 22 | 0,04 | -0,44802 |  |  |
| 23 | 0,02 | -0,31207 |  |  |
| 24 | 0,00 | 0,04078 |  |  |
| 25 | 0,01 | -0,21077 |  |  |
| 26 | 0,11 | 0,91125 | R |  |
| 27 | 0,00 | -0,05230 |  |  |

R  Large residual  
X  Unusual X

## Backward Elimination of Terms

α to remove = 0,1

## Coded Coefficients

| Term | Coef | SE Coef | 95% CI | T-Value | P-Value | VIF |
| --- | --- | --- | --- | --- | --- | --- |
| Constant | 2,395 | 0,385 | (1,597; 3,194) | 6,22 | 0,000 |  |
| Lac | 1,994 | 0,627 | (0,694; 3,294) | 3,18 | 0,004 | 1,10 |
| HPMC\_HP | 1,217 | 0,592 | (-0,011; 2,444) | 2,06 | 0,052 | 1,00 |
| Lac\*Lac | 4,59 | 1,13 | (2,24; 6,94) | 4,05 | 0,001 | 1,00 |
| Lac\*HPMC\_HP | 2,63 | 1,45 | (-0,37; 5,63) | 1,82 | 0,083 | 1,10 |

## Model Summary

| S | R-sq | R-sq(adj) | PRESS | R-sq(pred) | AICc | BIC |
| --- | --- | --- | --- | --- | --- | --- |
| 1,46265 | 58,85% | 51,36% | 79,1098 | 30,83% | 107,83 | 111,40 |

## Analysis of Variance

| Source | DF | Seq SS | Contribution | Adj SS | Adj MS | F-Value | P-Value |
| --- | --- | --- | --- | --- | --- | --- | --- |
| Model | 4 | 67,299 | 58,85% | 67,299 | 16,825 | 7,86 | 0,000 |
| Linear | 2 | 25,149 | 21,99% | 30,680 | 15,340 | 7,17 | 0,004 |
| Lac | 1 | 16,269 | 14,23% | 21,637 | 21,637 | 10,11 | 0,004 |
| HPMC\_HP | 1 | 8,880 | 7,76% | 9,043 | 9,043 | 4,23 | 0,052 |
| Square | 1 | 35,094 | 30,69% | 35,094 | 35,094 | 16,40 | 0,001 |
| Lac\*Lac | 1 | 35,094 | 30,69% | 35,094 | 35,094 | 16,40 | 0,001 |
| 2-Way Interaction | 1 | 7,056 | 6,17% | 7,056 | 7,056 | 3,30 | 0,083 |
| Lac\*HPMC\_HP | 1 | 7,056 | 6,17% | 7,056 | 7,056 | 3,30 | 0,083 |
| Error | 22 | 47,066 | 41,15% | 47,066 | 2,139 |  |  |
| Lack-of-Fit | 20 | 34,339 | 30,03% | 34,339 | 1,717 | 0,27 | 0,957 |
| Pure Error | 2 | 12,726 | 11,13% | 12,726 | 6,363 |  |  |
| Total | 26 | 114,365 | 100,00% |  |  |  |  |

## Regression Equation in Uncoded Units

|  |  |  |
| --- | --- | --- |
| F\_SD\_10h(600min) | = | 54,5 - 163,7 Lac - 3,98 HPMC\_HP + 73,4 Lac\*Lac + 10,36 Lac\*HPMC\_HP |

## Fits and Diagnostics for All Observations

| Obs | F\_SD\_10h(600min) | Fit | SE Fit | 95% CI | Resid | Std Resid | Del Resid |
| --- | --- | --- | --- | --- | --- | --- | --- |
| 1 | 4,249 | 2,616 | 0,688 | (1,189; 4,044) | 1,632 | 1,26 | 1,28 |
| 2 | 3,393 | 2,714 | 0,688 | (1,287; 4,141) | 0,679 | 0,53 | 0,52 |
| 3 | 1,732 | 2,619 | 0,711 | (1,144; 4,095) | -0,888 | -0,69 | -0,69 |
| 4 | 0,695 | 2,636 | 0,711 | (1,161; 4,112) | -1,941 | -1,52 | -1,57 |
| 5 | 3,531 | 2,525 | 0,518 | (1,451; 3,600) | 1,005 | 0,73 | 0,73 |
| 6 | 5,396 | 5,059 | 0,518 | (3,985; 6,134) | 0,336 | 0,25 | 0,24 |
| 7 | 2,759 | 2,499 | 0,700 | (1,047; 3,951) | 0,260 | 0,20 | 0,20 |
| 8 | 3,229 | 5,741 | 0,700 | (4,289; 7,193) | -2,512 | -1,96 | -2,10 |
| 9 | 1,614 | 2,612 | 0,658 | (1,248; 3,977) | -0,998 | -0,76 | -0,76 |
| 10 | 2,223 | 2,817 | 0,658 | (1,453; 4,182) | -0,595 | -0,46 | -0,45 |
| 11 | 1,592 | 2,592 | 0,523 | (1,508; 3,676) | -1,000 | -0,73 | -0,72 |
| 12 | 1,844 | 3,335 | 0,523 | (2,251; 4,419) | -1,491 | -1,09 | -1,10 |
| 13 | 3,039 | 2,506 | 0,651 | (1,155; 3,856) | 0,533 | 0,41 | 0,40 |
| 14 | 6,554 | 5,574 | 0,651 | (4,223; 6,924) | 0,980 | 0,75 | 0,74 |
| 15 | 1,970 | 2,529 | 0,500 | (1,492; 3,566) | -0,558 | -0,41 | -0,40 |
| 16 | 4,890 | 4,976 | 0,500 | (3,939; 6,013) | -0,087 | -0,06 | -0,06 |
| 17 | 4,493 | 5,067 | 1,108 | (2,769; 7,364) | -0,574 | -0,60 | -0,59 |
| 18 | 10,504 | 8,770 | 1,108 | (6,472; 11,067) | 1,734 | 1,82 | 1,92 |
| 19 | 1,583 | 2,306 | 0,379 | (1,520; 3,092) | -0,723 | -0,51 | -0,50 |
| 20 | 3,593 | 2,186 | 0,379 | (1,401; 2,971) | 1,407 | 1,00 | 1,00 |
| 21 | 2,686 | 1,179 | 0,640 | (-0,148; 2,505) | 1,507 | 1,15 | 1,15 |
| 22 | 3,052 | 3,612 | 0,767 | (2,022; 5,203) | -0,560 | -0,45 | -0,44 |
| 23 | 0,953 | 2,306 | 0,379 | (1,520; 3,092) | -1,352 | -0,96 | -0,96 |
| 24 | 2,600 | 2,186 | 0,379 | (1,401; 2,971) | 0,414 | 0,29 | 0,29 |
| 25 | 1,480 | 2,330 | 0,380 | (1,541; 3,118) | -0,849 | -0,60 | -0,59 |
| 26 | 6,146 | 2,330 | 0,380 | (1,541; 3,118) | 3,817 | 2,70 | 3,23 |
| 27 | 2,152 | 2,330 | 0,380 | (1,541; 3,118) | -0,177 | -0,13 | -0,12 |

| Obs | HI | Cook’s D | DFITS |  |  |
| --- | --- | --- | --- | --- | --- |
| 1 | 0,221366 | 0,09 | 0,68425 |  |  |
| 2 | 0,221366 | 0,02 | 0,27576 |  |  |
| 3 | 0,236586 | 0,03 | -0,38206 |  |  |
| 4 | 0,236586 | 0,14 | -0,87335 |  |  |
| 5 | 0,125431 | 0,02 | 0,27531 |  |  |
| 6 | 0,125431 | 0,00 | 0,09113 |  |  |
| 7 | 0,229169 | 0,00 | 0,10776 |  |  |
| 8 | 0,229169 | 0,23 | -1,14640 |  |  |
| 9 | 0,202403 | 0,03 | -0,38107 |  |  |
| 10 | 0,202403 | 0,01 | -0,22506 |  |  |
| 11 | 0,127732 | 0,02 | -0,27710 |  |  |
| 12 | 0,127732 | 0,03 | -0,41952 |  |  |
| 13 | 0,198178 | 0,01 | 0,19852 |  |  |
| 14 | 0,198178 | 0,03 | 0,36816 |  |  |
| 15 | 0,116856 | 0,00 | -0,14492 |  |  |
| 16 | 0,116856 | 0,00 | -0,02242 |  |  |
| 17 | 0,573726 | 0,10 | -0,68646 |  | X |
| 18 | 0,573726 | 0,89 | 2,23291 |  | X |
| 19 | 0,067140 | 0,00 | -0,13488 |  |  |
| 20 | 0,066994 | 0,01 | 0,26683 |  |  |
| 21 | 0,191276 | 0,06 | 0,56140 |  |  |
| 22 | 0,274906 | 0,02 | -0,27194 |  |  |
| 23 | 0,067140 | 0,01 | -0,25632 |  |  |
| 24 | 0,066994 | 0,00 | 0,07685 |  |  |
| 25 | 0,067551 | 0,01 | -0,15946 |  |  |
| 26 | 0,067551 | 0,11 | 0,86946 | R |  |
| 27 | 0,067551 | 0,00 | -0,03302 |  |  |

R  Large residual  
X  Unusual X

## Backward Elimination of Terms

α to remove = 0,1

## Coded Coefficients

| Term | Coef | SE Coef | 95% CI | T-Value | P-Value | VIF |
| --- | --- | --- | --- | --- | --- | --- |
| Constant | 2,437 | 0,399 | (1,609; 3,266) | 6,10 | 0,000 |  |
| Lac | 2,092 | 0,650 | (0,744; 3,441) | 3,22 | 0,004 | 1,10 |
| HPMC\_HP | 1,346 | 0,614 | (0,073; 2,620) | 2,19 | 0,039 | 1,00 |
| Lac\*Lac | 4,77 | 1,18 | (2,34; 7,21) | 4,06 | 0,001 | 1,00 |
| Lac\*HPMC\_HP | 2,83 | 1,50 | (-0,29; 5,94) | 1,88 | 0,073 | 1,10 |

## Model Summary

| S | R-sq | R-sq(adj) | PRESS | R-sq(pred) | AICc | BIC |
| --- | --- | --- | --- | --- | --- | --- |
| 1,51709 | 59,62% | 52,27% | 81,1308 | 35,29% | 109,80 | 113,37 |

## Analysis of Variance

| Source | DF | Seq SS | Contribution | Adj SS | Adj MS | F-Value | P-Value |
| --- | --- | --- | --- | --- | --- | --- | --- |
| Model | 4 | 74,745 | 59,62% | 74,745 | 18,686 | 8,12 | 0,000 |
| Linear | 2 | 28,608 | 22,82% | 34,895 | 17,448 | 7,58 | 0,003 |
| Lac | 1 | 17,726 | 14,14% | 23,825 | 23,825 | 10,35 | 0,004 |
| HPMC\_HP | 1 | 10,882 | 8,68% | 11,070 | 11,070 | 4,81 | 0,039 |
| Square | 1 | 37,975 | 30,29% | 37,975 | 37,975 | 16,50 | 0,001 |
| Lac\*Lac | 1 | 37,975 | 30,29% | 37,975 | 37,975 | 16,50 | 0,001 |
| 2-Way Interaction | 1 | 8,162 | 6,51% | 8,162 | 8,162 | 3,55 | 0,073 |
| Lac\*HPMC\_HP | 1 | 8,162 | 6,51% | 8,162 | 8,162 | 3,55 | 0,073 |
| Error | 22 | 50,634 | 40,38% | 50,634 | 2,302 |  |  |
| Lack-of-Fit | 20 | 36,735 | 29,30% | 36,735 | 1,837 | 0,26 | 0,960 |
| Pure Error | 2 | 13,899 | 11,09% | 13,899 | 6,949 |  |  |
| Total | 26 | 125,379 | 100,00% |  |  |  |  |

## Regression Equation in Uncoded Units

|  |  |  |
| --- | --- | --- |
| F\_SD\_11h(660min) | = | 57,6 - 173,6 Lac - 4,25 HPMC\_HP + 76,4 Lac\*Lac + 11,15 Lac\*HPMC\_HP |

## Fits and Diagnostics for All Observations

| Obs | F\_SD\_11h(660min) | Fit | SE Fit | 95% CI | Resid | Std Resid | Del Resid |
| --- | --- | --- | --- | --- | --- | --- | --- |
| 1 | 4,211 | 2,633 | 0,714 | (1,153; 4,114) | 1,578 | 1,18 | 1,19 |
| 2 | 3,428 | 2,687 | 0,714 | (1,206; 4,167) | 0,742 | 0,55 | 0,55 |
| 3 | 1,778 | 2,635 | 0,738 | (1,105; 4,166) | -0,857 | -0,65 | -0,64 |
| 4 | 0,714 | 2,602 | 0,738 | (1,071; 4,132) | -1,887 | -1,42 | -1,46 |
| 5 | 3,633 | 2,571 | 0,537 | (1,456; 3,685) | 1,062 | 0,75 | 0,74 |
| 6 | 5,315 | 5,243 | 0,537 | (4,129; 6,358) | 0,072 | 0,05 | 0,05 |
| 7 | 2,585 | 2,552 | 0,726 | (1,046; 4,058) | 0,033 | 0,02 | 0,02 |
| 8 | 3,298 | 5,987 | 0,726 | (4,481; 7,493) | -2,689 | -2,02 | -2,19 |
| 9 | 1,668 | 2,631 | 0,683 | (1,215; 4,046) | -0,962 | -0,71 | -0,70 |
| 10 | 2,396 | 2,799 | 0,683 | (1,383; 4,214) | -0,403 | -0,30 | -0,29 |
| 11 | 1,540 | 2,617 | 0,542 | (1,492; 3,741) | -1,077 | -0,76 | -0,75 |
| 12 | 1,898 | 3,363 | 0,542 | (2,239; 4,488) | -1,465 | -1,03 | -1,04 |
| 13 | 3,308 | 2,557 | 0,675 | (1,156; 3,957) | 0,751 | 0,55 | 0,54 |
| 14 | 7,764 | 5,804 | 0,675 | (4,404; 7,205) | 1,960 | 1,44 | 1,48 |
| 15 | 2,023 | 2,573 | 0,519 | (1,497; 3,648) | -0,550 | -0,39 | -0,38 |
| 16 | 4,812 | 5,153 | 0,519 | (4,077; 6,228) | -0,341 | -0,24 | -0,23 |
| 17 | 4,705 | 5,199 | 1,149 | (2,816; 7,582) | -0,493 | -0,50 | -0,49 |
| 18 | 10,579 | 9,077 | 1,149 | (6,694; 11,460) | 1,502 | 1,52 | 1,57 |
| 19 | 1,719 | 2,338 | 0,393 | (1,523; 3,153) | -0,619 | -0,42 | -0,41 |
| 20 | 3,374 | 2,205 | 0,393 | (1,391; 3,020) | 1,169 | 0,80 | 0,79 |
| 21 | 2,689 | 1,091 | 0,663 | (-0,285; 2,467) | 1,598 | 1,17 | 1,18 |
| 22 | 3,226 | 3,784 | 0,795 | (2,134; 5,433) | -0,558 | -0,43 | -0,42 |
| 23 | 0,817 | 2,338 | 0,393 | (1,523; 3,153) | -1,521 | -1,04 | -1,04 |
| 24 | 2,549 | 2,205 | 0,393 | (1,391; 3,020) | 0,344 | 0,23 | 0,23 |
| 25 | 1,384 | 2,364 | 0,394 | (1,547; 3,182) | -0,981 | -0,67 | -0,66 |
| 26 | 6,253 | 2,364 | 0,394 | (1,547; 3,182) | 3,889 | 2,65 | 3,15 |
| 27 | 2,068 | 2,364 | 0,394 | (1,547; 3,182) | -0,297 | -0,20 | -0,20 |

| Obs | HI | Cook’s D | DFITS |  |  |
| --- | --- | --- | --- | --- | --- |
| 1 | 0,221366 | 0,08 | 0,63431 |  |  |
| 2 | 0,221366 | 0,02 | 0,29064 |  |  |
| 3 | 0,236586 | 0,03 | -0,35519 |  |  |
| 4 | 0,236586 | 0,13 | -0,81275 |  |  |
| 5 | 0,125431 | 0,02 | 0,28067 |  |  |
| 6 | 0,125431 | 0,00 | 0,01869 |  |  |
| 7 | 0,229169 | 0,00 | 0,01328 |  |  |
| 8 | 0,229169 | 0,24 | -1,19141 | R |  |
| 9 | 0,202403 | 0,03 | -0,35363 |  |  |
| 10 | 0,202403 | 0,00 | -0,14671 |  |  |
| 11 | 0,127732 | 0,02 | -0,28786 |  |  |
| 12 | 0,127732 | 0,03 | -0,39627 |  |  |
| 13 | 0,198178 | 0,02 | 0,27033 |  |  |
| 14 | 0,198178 | 0,10 | 0,73656 |  |  |
| 15 | 0,116856 | 0,00 | -0,13745 |  |  |
| 16 | 0,116856 | 0,00 | -0,08509 |  |  |
| 17 | 0,573726 | 0,07 | -0,56776 |  | X |
| 18 | 0,573726 | 0,62 | 1,81583 |  | X |
| 19 | 0,067140 | 0,00 | -0,11120 |  |  |
| 20 | 0,066994 | 0,01 | 0,21192 |  |  |
| 21 | 0,191276 | 0,06 | 0,57487 |  |  |
| 22 | 0,274906 | 0,01 | -0,26092 |  |  |
| 23 | 0,067140 | 0,02 | -0,27892 |  |  |
| 24 | 0,066994 | 0,00 | 0,06153 |  |  |
| 25 | 0,067551 | 0,01 | -0,17784 |  |  |
| 26 | 0,067551 | 0,10 | 0,84669 | R |  |
| 27 | 0,067551 | 0,00 | -0,05333 |  |  |

R  Large residual  
X  Unusual X

## Backward Elimination of Terms

α to remove = 0,1

## Coded Coefficients

| Term | Coef | SE Coef | 95% CI | T-Value | P-Value | VIF |
| --- | --- | --- | --- | --- | --- | --- |
| Constant | 2,447 | 0,386 | (1,644; 3,249) | 6,34 | 0,000 |  |
| Lac | 1,824 | 0,620 | (0,535; 3,113) | 2,94 | 0,008 | 1,10 |
| HPMC\_Visc | -0,954 | 0,515 | (-2,025; 0,118) | -1,85 | 0,078 | 1,00 |
| HPMC\_HP | 1,412 | 0,586 | (0,194; 2,630) | 2,41 | 0,025 | 1,00 |
| Lac\*Lac | 4,13 | 1,12 | (1,80; 6,46) | 3,69 | 0,001 | 1,00 |
| Lac\*HPMC\_HP | 2,75 | 1,43 | (-0,23; 5,72) | 1,92 | 0,069 | 1,10 |

## Model Summary

| S | R-sq | R-sq(adj) | PRESS | R-sq(pred) | AICc | BIC |
| --- | --- | --- | --- | --- | --- | --- |
| 1,44607 | 60,60% | 51,22% | 66,6498 | 40,21% | 109,65 | 112,83 |

## Analysis of Variance

| Source | DF | Seq SS | Contribution | Adj SS | Adj MS | F-Value | P-Value |
| --- | --- | --- | --- | --- | --- | --- | --- |
| Model | 5 | 67,551 | 60,60% | 67,551 | 13,510 | 6,46 | 0,001 |
| Linear | 3 | 31,414 | 28,18% | 36,620 | 12,207 | 5,84 | 0,005 |
| Lac | 1 | 12,808 | 11,49% | 18,100 | 18,100 | 8,66 | 0,008 |
| HPMC\_Visc | 1 | 6,615 | 5,93% | 7,165 | 7,165 | 3,43 | 0,078 |
| HPMC\_HP | 1 | 11,991 | 10,76% | 12,147 | 12,147 | 5,81 | 0,025 |
| Square | 1 | 28,444 | 25,52% | 28,444 | 28,444 | 13,60 | 0,001 |
| Lac\*Lac | 1 | 28,444 | 25,52% | 28,444 | 28,444 | 13,60 | 0,001 |
| 2-Way Interaction | 1 | 7,693 | 6,90% | 7,693 | 7,693 | 3,68 | 0,069 |
| Lac\*HPMC\_HP | 1 | 7,693 | 6,90% | 7,693 | 7,693 | 3,68 | 0,069 |
| Error | 21 | 43,913 | 39,40% | 43,913 | 2,091 |  |  |
| Lack-of-Fit | 19 | 29,737 | 26,68% | 29,737 | 1,565 | 0,22 | 0,975 |
| Pure Error | 2 | 14,177 | 12,72% | 14,177 | 7,088 |  |  |
| Total | 26 | 111,464 | 100,00% |  |  |  |  |

## Regression Equation in Uncoded Units

|  |  |  |
| --- | --- | --- |
| F\_SD\_12h(720min) | = | 56,7 - 161,4 Lac - 0,000245 HPMC\_Visc - 4,02 HPMC\_HP + 66,1 Lac\*Lac + 10,82 Lac\*HPMC\_HP |

## Fits and Diagnostics for All Observations

| Obs | F\_SD\_12h(720min) | Fit | SE Fit | 95% CI | Resid | Std Resid | Del Resid |
| --- | --- | --- | --- | --- | --- | --- | --- |
| 1 | 4,313 | 3,219 | 0,737 | (1,686; 4,752) | 1,094 | 0,88 | 0,87 |
| 2 | 3,413 | 3,063 | 0,737 | (1,529; 4,596) | 0,350 | 0,28 | 0,28 |
| 3 | 1,834 | 1,881 | 0,829 | (0,157; 3,605) | -0,047 | -0,04 | -0,04 |
| 4 | 0,835 | 1,640 | 0,829 | (-0,084; 3,365) | -0,806 | -0,68 | -0,67 |
| 5 | 3,615 | 3,195 | 0,582 | (1,985; 4,404) | 0,421 | 0,32 | 0,31 |
| 6 | 5,238 | 5,582 | 0,582 | (4,372; 6,792) | -0,344 | -0,26 | -0,25 |
| 7 | 2,464 | 1,908 | 0,809 | (0,227; 3,590) | 0,556 | 0,46 | 0,45 |
| 8 | 3,329 | 5,035 | 0,809 | (3,354; 6,717) | -1,706 | -1,42 | -1,46 |
| 9 | 1,765 | 3,202 | 0,706 | (1,733; 4,670) | -1,436 | -1,14 | -1,15 |
| 10 | 2,623 | 3,157 | 0,706 | (1,689; 4,626) | -0,534 | -0,42 | -0,41 |
| 11 | 1,403 | 2,367 | 0,546 | (1,233; 3,502) | -0,965 | -0,72 | -0,71 |
| 12 | 1,781 | 2,884 | 0,546 | (1,750; 4,019) | -1,103 | -0,82 | -0,82 |
| 13 | 3,630 | 3,252 | 0,713 | (1,768; 4,736) | 0,378 | 0,30 | 0,29 |
| 14 | 8,411 | 6,197 | 0,713 | (4,713; 7,681) | 2,214 | 1,76 | 1,86 |
| 15 | 2,142 | 2,305 | 0,535 | (1,192; 3,418) | -0,163 | -0,12 | -0,12 |
| 16 | 4,555 | 4,602 | 0,535 | (3,489; 5,715) | -0,048 | -0,04 | -0,03 |
| 17 | 4,819 | 5,005 | 1,096 | (2,727; 7,284) | -0,186 | -0,20 | -0,19 |
| 18 | 9,077 | 8,355 | 1,096 | (6,077; 10,634) | 0,721 | 0,76 | 0,76 |
| 19 | 1,700 | 3,296 | 0,587 | (2,076; 4,516) | -1,596 | -1,21 | -1,22 |
| 20 | 3,271 | 1,249 | 0,692 | (-0,189; 2,688) | 2,022 | 1,59 | 1,66 |
| 21 | 2,743 | 1,207 | 0,632 | (-0,108; 2,522) | 1,536 | 1,18 | 1,19 |
| 22 | 3,334 | 3,969 | 0,759 | (2,391; 5,546) | -0,635 | -0,52 | -0,51 |
| 23 | 0,768 | 2,590 | 0,381 | (1,797; 3,383) | -1,821 | -1,31 | -1,33 |
| 24 | 2,505 | 2,461 | 0,381 | (1,668; 3,254) | 0,045 | 0,03 | 0,03 |
| 25 | 1,439 | 2,549 | 0,377 | (1,764; 3,334) | -1,110 | -0,80 | -0,79 |
| 26 | 6,292 | 2,549 | 0,377 | (1,764; 3,334) | 3,743 | 2,68 | 3,23 |
| 27 | 1,968 | 2,549 | 0,377 | (1,764; 3,334) | -0,581 | -0,42 | -0,41 |

| Obs | HI | Cook’s D | DFITS |  |
| --- | --- | --- | --- | --- |
| 1 | 0,259907 | 0,05 | 0,51844 |  |
| 2 | 0,259907 | 0,00 | 0,16303 |  |
| 3 | 0,328762 | 0,00 | -0,02695 |  |
| 4 | 0,328762 | 0,04 | -0,46966 |  |
| 5 | 0,161779 | 0,00 | 0,13651 |  |
| 6 | 0,161779 | 0,00 | -0,11168 |  |
| 7 | 0,312694 | 0,02 | 0,30687 |  |
| 8 | 0,312694 | 0,15 | -0,98569 |  |
| 9 | 0,238520 | 0,07 | -0,64169 |  |
| 10 | 0,238520 | 0,01 | -0,23209 |  |
| 11 | 0,142357 | 0,01 | -0,29001 |  |
| 12 | 0,142357 | 0,02 | -0,33285 |  |
| 13 | 0,243427 | 0,00 | 0,16675 |  |
| 14 | 0,243427 | 0,17 | 1,05533 |  |
| 15 | 0,136989 | 0,00 | -0,04726 |  |
| 16 | 0,136989 | 0,00 | -0,01381 |  |
| 17 | 0,573996 | 0,01 | -0,22403 |  |
| 18 | 0,573996 | 0,13 | 0,87780 |  |
| 19 | 0,164663 | 0,05 | -0,54255 |  |
| 20 | 0,228727 | 0,13 | 0,90244 |  |
| 21 | 0,191289 | 0,06 | 0,58040 |  |
| 22 | 0,275177 | 0,02 | -0,31193 |  |
| 23 | 0,069490 | 0,02 | -0,36329 |  |
| 24 | 0,069531 | 0,00 | 0,00857 |  |
| 25 | 0,068088 | 0,01 | -0,21294 |  |
| 26 | 0,068088 | 0,09 | 0,87230 | R |
| 27 | 0,068088 | 0,00 | -0,11017 |  |

R  Large residual

## Backward Elimination of Terms

α to remove = 0,1

## Coded Coefficients

| Term | Coef | SE Coef | 95% CI | T-Value | P-Value | VIF |
| --- | --- | --- | --- | --- | --- | --- |
| Constant | 2,493 | 0,347 | (1,772; 3,214) | 7,19 | 0,000 |  |
| Lac | 1,479 | 0,557 | (0,321; 2,637) | 2,66 | 0,015 | 1,10 |
| HPMC\_Visc | -0,942 | 0,463 | (-1,905; 0,021) | -2,04 | 0,055 | 1,00 |
| HPMC\_HP | 1,311 | 0,526 | (0,217; 2,406) | 2,49 | 0,021 | 1,00 |
| Lac\*Lac | 3,48 | 1,01 | (1,39; 5,58) | 3,46 | 0,002 | 1,00 |
| Lac\*HPMC\_HP | 2,24 | 1,29 | (-0,43; 4,92) | 1,74 | 0,096 | 1,10 |

## Model Summary

| S | R-sq | R-sq(adj) | PRESS | R-sq(pred) | AICc | BIC |
| --- | --- | --- | --- | --- | --- | --- |
| 1,29940 | 58,80% | 48,98% | 50,6920 | 41,09% | 103,87 | 107,05 |

## Analysis of Variance

| Source | DF | Seq SS | Contribution | Adj SS | Adj MS | F-Value | P-Value |
| --- | --- | --- | --- | --- | --- | --- | --- |
| Model | 5 | 50,594 | 58,80% | 50,594 | 10,119 | 5,99 | 0,001 |
| Linear | 3 | 25,217 | 29,30% | 28,650 | 9,550 | 5,66 | 0,005 |
| Lac | 1 | 8,392 | 9,75% | 11,904 | 11,904 | 7,05 | 0,015 |
| HPMC\_Visc | 1 | 6,468 | 7,52% | 6,993 | 6,993 | 4,14 | 0,055 |
| HPMC\_HP | 1 | 10,358 | 12,04% | 10,480 | 10,480 | 6,21 | 0,021 |
| Square | 1 | 20,240 | 23,52% | 20,240 | 20,240 | 11,99 | 0,002 |
| Lac\*Lac | 1 | 20,240 | 23,52% | 20,240 | 20,240 | 11,99 | 0,002 |
| 2-Way Interaction | 1 | 5,137 | 5,97% | 5,137 | 5,137 | 3,04 | 0,096 |
| Lac\*HPMC\_HP | 1 | 5,137 | 5,97% | 5,137 | 5,137 | 3,04 | 0,096 |
| Error | 21 | 35,457 | 41,20% | 35,457 | 1,688 |  |  |
| Lack-of-Fit | 19 | 22,962 | 26,68% | 22,962 | 1,209 | 0,19 | 0,984 |
| Pure Error | 2 | 12,495 | 14,52% | 12,495 | 6,248 |  |  |
| Total | 26 | 86,051 | 100,00% |  |  |  |  |

## Regression Equation in Uncoded Units

|  |  |  |
| --- | --- | --- |
| F\_SD\_13h(780min) | = | 46,4 - 133,6 Lac - 0,000242 HPMC\_Visc - 3,13 HPMC\_HP + 55,8 Lac\*Lac + 8,84 Lac\*HPMC\_HP |

## Fits and Diagnostics for All Observations

| Obs | F\_SD\_13h(780min) | Fit | SE Fit | 95% CI | Resid | Std Resid | Del Resid | HI |
| --- | --- | --- | --- | --- | --- | --- | --- | --- |
| 1 | 4,154 | 3,159 | 0,662 | (1,782; 4,537) | 0,995 | 0,89 | 0,89 | 0,259907 |
| 2 | 3,281 | 3,020 | 0,662 | (1,643; 4,398) | 0,260 | 0,23 | 0,23 | 0,259907 |
| 3 | 1,828 | 1,833 | 0,745 | (0,283; 3,382) | -0,005 | -0,00 | -0,00 | 0,328762 |
| 4 | 1,213 | 1,625 | 0,745 | (0,076; 3,174) | -0,412 | -0,39 | -0,38 | 0,328762 |
| 5 | 3,581 | 3,275 | 0,523 | (2,189; 4,362) | 0,306 | 0,26 | 0,25 | 0,161779 |
| 6 | 5,119 | 5,215 | 0,523 | (4,128; 6,302) | -0,096 | -0,08 | -0,08 | 0,161779 |
| 7 | 2,211 | 2,045 | 0,727 | (0,534; 3,556) | 0,166 | 0,15 | 0,15 | 0,312694 |
| 8 | 3,508 | 4,589 | 0,727 | (3,078; 6,100) | -1,081 | -1,00 | -1,00 | 0,312694 |
| 9 | 1,741 | 3,148 | 0,635 | (1,829; 4,468) | -1,407 | -1,24 | -1,26 | 0,238520 |
| 10 | 3,123 | 3,101 | 0,635 | (1,781; 4,421) | 0,022 | 0,02 | 0,02 | 0,238520 |
| 11 | 1,385 | 2,355 | 0,490 | (1,335; 3,375) | -0,970 | -0,81 | -0,80 | 0,142357 |
| 12 | 1,668 | 2,766 | 0,490 | (1,747; 3,786) | -1,098 | -0,91 | -0,91 | 0,142357 |
| 13 | 3,799 | 3,363 | 0,641 | (2,029; 4,696) | 0,436 | 0,39 | 0,38 | 0,243427 |
| 14 | 7,900 | 5,758 | 0,641 | (4,425; 7,091) | 2,142 | 1,90 | 2,03 | 0,243427 |
| 15 | 2,194 | 2,391 | 0,481 | (1,391; 3,392) | -0,197 | -0,16 | -0,16 | 0,136989 |
| 16 | 4,242 | 4,257 | 0,481 | (3,257; 5,258) | -0,015 | -0,01 | -0,01 | 0,136989 |
| 17 | 4,945 | 4,726 | 0,984 | (2,679; 6,774) | 0,219 | 0,26 | 0,25 | 0,573996 |
| 18 | 7,461 | 7,441 | 0,984 | (5,394; 9,489) | 0,020 | 0,02 | 0,02 | 0,573996 |
| 19 | 1,742 | 3,338 | 0,527 | (2,242; 4,435) | -1,597 | -1,34 | -1,37 | 0,164663 |
| 20 | 3,051 | 1,325 | 0,621 | (0,032; 2,617) | 1,727 | 1,51 | 1,56 | 0,228727 |
| 21 | 2,635 | 1,352 | 0,568 | (0,170; 2,534) | 1,283 | 1,10 | 1,10 | 0,191289 |
| 22 | 3,380 | 3,913 | 0,682 | (2,496; 5,331) | -0,533 | -0,48 | -0,47 | 0,275177 |
| 23 | 0,886 | 2,641 | 0,343 | (1,928; 3,353) | -1,755 | -1,40 | -1,44 | 0,069490 |
| 24 | 2,471 | 2,521 | 0,343 | (1,809; 3,234) | -0,050 | -0,04 | -0,04 | 0,069531 |
| 25 | 1,535 | 2,599 | 0,339 | (1,894; 3,304) | -1,064 | -0,85 | -0,84 | 0,068088 |
| 26 | 6,025 | 2,599 | 0,339 | (1,894; 3,304) | 3,427 | 2,73 | 3,32 | 0,068088 |
| 27 | 1,877 | 2,599 | 0,339 | (1,894; 3,304) | -0,721 | -0,58 | -0,57 | 0,068088 |

| Obs | Cook’s D | DFITS |  |
| --- | --- | --- | --- |
| 1 | 0,05 | 0,52483 |  |
| 2 | 0,00 | 0,13491 |  |
| 3 | 0,00 | -0,00300 |  |
| 4 | 0,01 | -0,26496 |  |
| 5 | 0,00 | 0,11042 |  |
| 6 | 0,00 | -0,03460 |  |
| 7 | 0,00 | 0,10136 |  |
| 8 | 0,08 | -0,67683 |  |
| 9 | 0,08 | -0,70413 |  |
| 10 | 0,00 | 0,01042 |  |
| 11 | 0,02 | -0,32563 |  |
| 12 | 0,02 | -0,37033 |  |
| 13 | 0,01 | 0,21455 |  |
| 14 | 0,19 | 1,15216 |  |
| 15 | 0,00 | -0,06361 |  |
| 16 | 0,00 | -0,00484 |  |
| 17 | 0,01 | 0,29270 |  |
| 18 | 0,00 | 0,02628 |  |
| 19 | 0,06 | -0,60930 |  |
| 20 | 0,11 | 0,85184 |  |
| 21 | 0,05 | 0,53674 |  |
| 22 | 0,01 | -0,29156 |  |
| 23 | 0,02 | -0,39222 |  |
| 24 | 0,00 | -0,01072 |  |
| 25 | 0,01 | -0,22763 |  |
| 26 | 0,09 | 0,89748 | R |
| 27 | 0,00 | -0,15291 |  |

R  Large residual

## Backward Elimination of Terms

α to remove = 0,1

## Coded Coefficients

| Term | Coef | SE Coef | 95% CI | T-Value | P-Value | VIF |
| --- | --- | --- | --- | --- | --- | --- |
| Constant | 2,559 | 0,308 | (1,919; 3,198) | 8,30 | 0,000 |  |
| Lac | 0,943 | 0,472 | (-0,036; 1,921) | 2,00 | 0,058 | 1,00 |
| HPMC\_Visc | -0,724 | 0,412 | (-1,578; 0,131) | -1,76 | 0,093 | 1,00 |
| HPMC\_HP | 1,228 | 0,468 | (0,257; 2,199) | 2,62 | 0,016 | 1,00 |
| Lac\*Lac | 3,013 | 0,895 | (1,156; 4,870) | 3,36 | 0,003 | 1,00 |

## Model Summary

| S | R-sq | R-sq(adj) | PRESS | R-sq(pred) | AICc | BIC |
| --- | --- | --- | --- | --- | --- | --- |
| 1,15594 | 53,10% | 44,57% | 39,9604 | 36,25% | 95,12 | 98,69 |

## Analysis of Variance

| Source | DF | Seq SS | Contribution | Adj SS | Adj MS | F-Value | P-Value |
| --- | --- | --- | --- | --- | --- | --- | --- |
| Model | 4 | 33,284 | 53,10% | 33,284 | 8,3210 | 6,23 | 0,002 |
| Linear | 3 | 18,156 | 28,97% | 18,123 | 6,0410 | 4,52 | 0,013 |
| Lac | 1 | 5,333 | 8,51% | 5,333 | 5,3330 | 3,99 | 0,058 |
| HPMC\_Visc | 1 | 3,735 | 5,96% | 4,123 | 4,1233 | 3,09 | 0,093 |
| HPMC\_HP | 1 | 9,089 | 14,50% | 9,188 | 9,1878 | 6,88 | 0,016 |
| Square | 1 | 15,128 | 24,13% | 15,128 | 15,1276 | 11,32 | 0,003 |
| Lac\*Lac | 1 | 15,128 | 24,13% | 15,128 | 15,1276 | 11,32 | 0,003 |
| Error | 22 | 29,396 | 46,90% | 29,396 | 1,3362 |  |  |
| Lack-of-Fit | 20 | 18,697 | 29,83% | 18,697 | 0,9348 | 0,17 | 0,989 |
| Pure Error | 2 | 10,700 | 17,07% | 10,700 | 5,3499 |  |  |
| Total | 26 | 62,680 | 100,00% |  |  |  |  |

## Regression Equation in Uncoded Units

|  |  |  |
| --- | --- | --- |
| F\_SD\_14h(840min) | = | 3,78 - 44,4 Lac - 0,000186 HPMC\_Visc + 1,210 HPMC\_HP + 48,2 Lac\*Lac |

## Fits and Diagnostics for All Observations

| Obs | F\_SD\_14h(840min) | Fit | SE Fit | 95% CI | Resid | Std Resid | Del Resid | HI |
| --- | --- | --- | --- | --- | --- | --- | --- | --- |
| 1 | 3,876 | 2,470 | 0,483 | (1,468; 3,473) | 1,405 | 1,34 | 1,36 | 0,174920 |
| 2 | 3,215 | 3,413 | 0,483 | (2,410; 4,416) | -0,198 | -0,19 | -0,18 | 0,174920 |
| 3 | 1,802 | 1,419 | 0,560 | (0,257; 2,580) | 0,383 | 0,38 | 0,37 | 0,234664 |
| 4 | 1,979 | 2,361 | 0,560 | (1,200; 3,523) | -0,382 | -0,38 | -0,37 | 0,234664 |
| 5 | 3,594 | 3,562 | 0,423 | (2,685; 4,439) | 0,032 | 0,03 | 0,03 | 0,133899 |
| 6 | 5,076 | 4,505 | 0,423 | (3,628; 5,382) | 0,572 | 0,53 | 0,52 | 0,133899 |
| 7 | 2,329 | 2,909 | 0,545 | (1,778; 4,040) | -0,580 | -0,57 | -0,56 | 0,222511 |
| 8 | 4,407 | 3,852 | 0,545 | (2,721; 4,983) | 0,555 | 0,54 | 0,54 | 0,222511 |
| 9 | 1,770 | 2,506 | 0,469 | (1,533; 3,480) | -0,736 | -0,70 | -0,69 | 0,164882 |
| 10 | 3,457 | 3,449 | 0,469 | (2,475; 4,422) | 0,008 | 0,01 | 0,01 | 0,164882 |
| 11 | 1,345 | 2,118 | 0,389 | (1,311; 2,925) | -0,774 | -0,71 | -0,70 | 0,113393 |
| 12 | 1,503 | 3,061 | 0,389 | (2,254; 3,868) | -1,558 | -1,43 | -1,47 | 0,113393 |
| 13 | 3,929 | 3,849 | 0,479 | (2,855; 4,843) | 0,080 | 0,08 | 0,07 | 0,171851 |
| 14 | 6,612 | 4,792 | 0,479 | (3,798; 5,786) | 1,820 | 1,73 | 1,82 | 0,171851 |
| 15 | 2,288 | 2,848 | 0,391 | (2,037; 3,658) | -0,560 | -0,52 | -0,51 | 0,114264 |
| 16 | 3,886 | 3,791 | 0,391 | (2,980; 4,601) | 0,096 | 0,09 | 0,09 | 0,114264 |
| 17 | 5,093 | 4,698 | 0,871 | (2,891; 6,505) | 0,395 | 0,52 | 0,51 | 0,568046 |
| 18 | 6,147 | 6,583 | 0,871 | (4,777; 8,390) | -0,436 | -0,57 | -0,56 | 0,568046 |
| 19 | 1,792 | 3,191 | 0,469 | (2,219; 4,164) | -1,399 | -1,32 | -1,35 | 0,164663 |
| 20 | 2,800 | 1,623 | 0,553 | (0,477; 2,770) | 1,177 | 1,16 | 1,17 | 0,228727 |
| 21 | 2,589 | 1,461 | 0,506 | (0,413; 2,510) | 1,127 | 1,08 | 1,09 | 0,191289 |
| 22 | 3,471 | 3,870 | 0,606 | (2,613; 5,128) | -0,399 | -0,41 | -0,40 | 0,275177 |
| 23 | 1,031 | 2,656 | 0,305 | (2,024; 3,288) | -1,625 | -1,46 | -1,50 | 0,069490 |
| 24 | 2,404 | 2,542 | 0,305 | (1,910; 3,174) | -0,138 | -0,12 | -0,12 | 0,069531 |
| 25 | 1,549 | 2,628 | 0,302 | (2,002; 3,253) | -1,079 | -0,97 | -0,97 | 0,068088 |
| 26 | 5,673 | 2,628 | 0,302 | (2,002; 3,253) | 3,045 | 2,73 | 3,28 | 0,068088 |
| 27 | 1,796 | 2,628 | 0,302 | (2,002; 3,253) | -0,832 | -0,75 | -0,74 | 0,068088 |

| Obs | Cook’s D | DFITS |  |  |
| --- | --- | --- | --- | --- |
| 1 | 0,08 | 0,628283 |  |  |
| 2 | 0,00 | -0,084757 |  |  |
| 3 | 0,01 | 0,205712 |  |  |
| 4 | 0,01 | -0,205198 |  |  |
| 5 | 0,00 | 0,011344 |  |  |
| 6 | 0,01 | 0,205416 |  |  |
| 7 | 0,02 | -0,299444 |  |  |
| 8 | 0,02 | 0,286705 |  |  |
| 9 | 0,02 | -0,305837 |  |  |
| 10 | 0,00 | 0,003221 |  |  |
| 11 | 0,01 | -0,251236 |  |  |
| 12 | 0,05 | -0,525126 |  |  |
| 13 | 0,00 | 0,033999 |  |  |
| 14 | 0,12 | 0,828693 |  |  |
| 15 | 0,01 | -0,181828 |  |  |
| 16 | 0,00 | 0,030895 |  |  |
| 17 | 0,07 | 0,585941 |  | X |
| 18 | 0,09 | -0,647864 |  | X |
| 19 | 0,07 | -0,598796 |  |  |
| 20 | 0,08 | 0,636369 |  |  |
| 21 | 0,06 | 0,529735 |  |  |
| 22 | 0,01 | -0,245115 |  |  |
| 23 | 0,03 | -0,409368 |  |  |
| 24 | 0,00 | -0,033027 |  |  |
| 25 | 0,01 | -0,260981 |  |  |
| 26 | 0,11 | 0,885948 | R |  |
| 27 | 0,01 | -0,199459 |  |  |

R  Large residual  
X  Unusual X

## Backward Elimination of Terms

α to remove = 0,1

## Coded Coefficients

| Term | Coef | SE Coef | 95% CI | T-Value | P-Value | VIF |
| --- | --- | --- | --- | --- | --- | --- |
| Constant | 2,687 | 0,304 | (2,057; 3,316) | 8,83 | 0,000 |  |
| Lac | 0,633 | 0,472 | (-0,343; 1,609) | 1,34 | 0,193 | 1,00 |
| HPMC\_HP | 1,028 | 0,468 | (0,061; 1,995) | 2,20 | 0,038 | 1,00 |
| Lac\*Lac | 2,568 | 0,895 | (0,716; 4,419) | 2,87 | 0,009 | 1,00 |

## Model Summary

| S | R-sq | R-sq(adj) | PRESS | R-sq(pred) | AICc | BIC |
| --- | --- | --- | --- | --- | --- | --- |
| 1,15549 | 39,17% | 31,23% | 43,3451 | 14,13% | 92,95 | 96,58 |

## Analysis of Variance

| Source | DF | Seq SS | Contribution | Adj SS | Adj MS | F-Value | P-Value |
| --- | --- | --- | --- | --- | --- | --- | --- |
| Model | 3 | 19,771 | 39,17% | 19,771 | 6,590 | 4,94 | 0,009 |
| Linear | 2 | 8,781 | 17,40% | 8,858 | 4,429 | 3,32 | 0,054 |
| Lac | 1 | 2,406 | 4,77% | 2,406 | 2,406 | 1,80 | 0,193 |
| HPMC\_HP | 1 | 6,375 | 12,63% | 6,453 | 6,453 | 4,83 | 0,038 |
| Square | 1 | 10,990 | 21,77% | 10,990 | 10,990 | 8,23 | 0,009 |
| Lac\*Lac | 1 | 10,990 | 21,77% | 10,990 | 10,990 | 8,23 | 0,009 |
| Error | 23 | 30,708 | 60,83% | 30,708 | 1,335 |  |  |
| Lack-of-Fit | 21 | 21,830 | 43,24% | 21,830 | 1,040 | 0,23 | 0,972 |
| Pure Error | 2 | 8,879 | 17,59% | 8,879 | 4,439 |  |  |
| Total | 26 | 50,480 | 100,00% |  |  |  |  |

## Regression Equation in Uncoded Units

|  |  |  |
| --- | --- | --- |
| F\_SD\_15h(900min) | = | 2,10 - 38,6 Lac + 1,013 HPMC\_HP + 41,1 Lac\*Lac |

## Fits and Diagnostics for All Observations

| Obs | F\_SD\_15h(900min) | Fit | SE Fit | 95% CI | Resid | Std Resid | Del Resid | HI |
| --- | --- | --- | --- | --- | --- | --- | --- | --- |
| 1 | 4,545 | 2,271 | 0,427 | (1,388; 3,154) | 2,274 | 2,12 | 2,31 | 0,136379 |
| 2 | 3,098 | 2,904 | 0,427 | (2,022; 3,787) | 0,193 | 0,18 | 0,18 | 0,136379 |
| 3 | 1,836 | 2,239 | 0,436 | (1,337; 3,142) | -0,403 | -0,38 | -0,37 | 0,142488 |
| 4 | 2,994 | 2,873 | 0,436 | (1,970; 3,775) | 0,122 | 0,11 | 0,11 | 0,142488 |
| 5 | 3,497 | 3,223 | 0,361 | (2,476; 3,970) | 0,274 | 0,25 | 0,24 | 0,097552 |
| 6 | 4,903 | 3,856 | 0,361 | (3,110; 4,603) | 1,047 | 0,95 | 0,95 | 0,097552 |
| 7 | 2,512 | 3,500 | 0,431 | (2,609; 4,391) | -0,988 | -0,92 | -0,92 | 0,138986 |
| 8 | 5,260 | 4,133 | 0,431 | (3,242; 5,024) | 1,127 | 1,05 | 1,05 | 0,138986 |
| 9 | 1,653 | 2,313 | 0,415 | (1,455; 3,171) | -0,660 | -0,61 | -0,60 | 0,128766 |
| 10 | 3,621 | 2,946 | 0,415 | (2,088; 3,804) | 0,674 | 0,63 | 0,62 | 0,128766 |
| 11 | 1,294 | 2,523 | 0,363 | (1,772; 3,274) | -1,229 | -1,12 | -1,13 | 0,098768 |
| 12 | 1,300 | 3,156 | 0,363 | (2,405; 3,907) | -1,856 | -1,69 | -1,77 | 0,098768 |
| 13 | 4,017 | 3,432 | 0,411 | (2,581; 4,282) | 0,585 | 0,54 | 0,53 | 0,126602 |
| 14 | 5,133 | 4,065 | 0,411 | (3,215; 4,916) | 1,068 | 0,99 | 0,99 | 0,126602 |
| 15 | 2,349 | 3,189 | 0,355 | (2,456; 3,923) | -0,840 | -0,76 | -0,76 | 0,094131 |
| 16 | 3,590 | 3,823 | 0,355 | (3,089; 4,556) | -0,232 | -0,21 | -0,21 | 0,094131 |
| 17 | 5,204 | 4,566 | 0,871 | (2,765; 6,367) | 0,638 | 0,84 | 0,83 | 0,567776 |
| 18 | 4,905 | 5,832 | 0,871 | (4,031; 7,633) | -0,927 | -1,22 | -1,23 | 0,567776 |
| 19 | 1,843 | 2,611 | 0,299 | (1,991; 3,230) | -0,768 | -0,69 | -0,68 | 0,067140 |
| 20 | 2,649 | 2,510 | 0,299 | (1,891; 3,128) | 0,139 | 0,12 | 0,12 | 0,066994 |
| 21 | 2,543 | 1,659 | 0,505 | (0,613; 2,704) | 0,884 | 0,85 | 0,85 | 0,191276 |
| 22 | 3,716 | 3,715 | 0,606 | (2,461; 4,968) | 0,002 | 0,00 | 0,00 | 0,274906 |
| 23 | 1,152 | 2,611 | 0,299 | (1,991; 3,230) | -1,459 | -1,31 | -1,33 | 0,067140 |
| 24 | 2,288 | 2,510 | 0,299 | (1,891; 3,128) | -0,221 | -0,20 | -0,19 | 0,066994 |
| 25 | 1,533 | 2,631 | 0,300 | (2,010; 3,252) | -1,098 | -0,98 | -0,98 | 0,067551 |
| 26 | 5,248 | 2,631 | 0,300 | (2,010; 3,252) | 2,617 | 2,35 | 2,63 | 0,067551 |
| 27 | 1,669 | 2,631 | 0,300 | (2,010; 3,252) | -0,962 | -0,86 | -0,86 | 0,067551 |

| Obs | Cook’s D | DFITS |  |  |
| --- | --- | --- | --- | --- |
| 1 | 0,18 | 0,91714 | R |  |
| 2 | 0,00 | 0,07004 |  |  |
| 3 | 0,01 | -0,15063 |  |  |
| 4 | 0,00 | 0,04542 |  |  |
| 5 | 0,00 | 0,08031 |  |  |
| 6 | 0,02 | 0,31293 |  |  |
| 7 | 0,03 | -0,36886 |  |  |
| 8 | 0,04 | 0,42326 |  |  |
| 9 | 0,01 | -0,23203 |  |  |
| 10 | 0,01 | 0,23715 |  |  |
| 11 | 0,03 | -0,37303 |  |  |
| 12 | 0,08 | -0,58562 |  |  |
| 13 | 0,01 | 0,20306 |  |  |
| 14 | 0,04 | 0,37647 |  |  |
| 15 | 0,02 | -0,24406 |  |  |
| 16 | 0,00 | -0,06666 |  |  |
| 17 | 0,23 | 0,95662 |  | X |
| 18 | 0,49 | -1,41457 |  | X |
| 19 | 0,01 | -0,18252 |  |  |
| 20 | 0,00 | 0,03269 |  |  |
| 21 | 0,04 | 0,41123 |  |  |
| 22 | 0,00 | 0,00098 |  |  |
| 23 | 0,03 | -0,35640 |  |  |
| 24 | 0,00 | -0,05205 |  |  |
| 25 | 0,02 | -0,26472 |  |  |
| 26 | 0,10 | 0,70795 | R |  |
| 27 | 0,01 | -0,23076 |  |  |

R  Large residual  
X  Unusual X

## Backward Elimination of Terms

α to remove = 0,1

## Coded Coefficients

| Term | Coef | SE Coef | 95% CI | T-Value | P-Value | VIF |
| --- | --- | --- | --- | --- | --- | --- |
| Constant | 2,654 | 0,288 | (2,058; 3,249) | 9,21 | 0,000 |  |
| Lac | 0,377 | 0,447 | (-0,546; 1,301) | 0,85 | 0,407 | 1,00 |
| HPMC\_HP | 1,009 | 0,443 | (0,093; 1,925) | 2,28 | 0,032 | 1,00 |
| Lac\*Lac | 2,021 | 0,847 | (0,268; 3,773) | 2,39 | 0,026 | 1,00 |

## Model Summary

| S | R-sq | R-sq(adj) | PRESS | R-sq(pred) | AICc | BIC |
| --- | --- | --- | --- | --- | --- | --- |
| 1,09371 | 33,43% | 24,75% | 43,2100 | 0,00% | 89,99 | 93,61 |

## Analysis of Variance

| Source | DF | Seq SS | Contribution | Adj SS | Adj MS | F-Value | P-Value |
| --- | --- | --- | --- | --- | --- | --- | --- |
| Model | 3 | 13,8179 | 33,43% | 13,8179 | 4,6060 | 3,85 | 0,023 |
| Linear | 2 | 7,0125 | 16,97% | 7,0722 | 3,5361 | 2,96 | 0,072 |
| Lac | 1 | 0,8545 | 2,07% | 0,8545 | 0,8545 | 0,71 | 0,407 |
| HPMC\_HP | 1 | 6,1580 | 14,90% | 6,2178 | 6,2178 | 5,20 | 0,032 |
| Square | 1 | 6,8054 | 16,47% | 6,8054 | 6,8054 | 5,69 | 0,026 |
| Lac\*Lac | 1 | 6,8054 | 16,47% | 6,8054 | 6,8054 | 5,69 | 0,026 |
| Error | 23 | 27,5127 | 66,57% | 27,5127 | 1,1962 |  |  |
| Lack-of-Fit | 21 | 19,9578 | 48,29% | 19,9578 | 0,9504 | 0,25 | 0,966 |
| Pure Error | 2 | 7,5549 | 18,28% | 7,5549 | 3,7774 |  |  |
| Total | 26 | 41,3306 | 100,00% |  |  |  |  |

## Regression Equation in Uncoded Units

|  |  |  |
| --- | --- | --- |
| F\_SD\_16h(960min) | = | 0,56 - 30,8 Lac + 0,994 HPMC\_HP + 32,3 Lac\*Lac |

## Fits and Diagnostics for All Observations

| Obs | F\_SD\_16h(960min) | Fit | SE Fit | 95% CI | Resid | Std Resid | Del Resid | HI |
| --- | --- | --- | --- | --- | --- | --- | --- | --- |
| 1 | 4,115 | 2,243 | 0,404 | (1,407; 3,078) | 1,872 | 1,84 | 1,95 | 0,136379 |
| 2 | 3,274 | 2,620 | 0,404 | (1,784; 3,455) | 0,654 | 0,64 | 0,63 | 0,136379 |
| 3 | 1,831 | 2,212 | 0,413 | (1,357; 3,066) | -0,380 | -0,38 | -0,37 | 0,142488 |
| 4 | 3,457 | 2,589 | 0,413 | (1,735; 3,443) | 0,868 | 0,86 | 0,85 | 0,142488 |
| 5 | 3,358 | 3,177 | 0,342 | (2,470; 3,884) | 0,180 | 0,17 | 0,17 | 0,097552 |
| 6 | 4,508 | 3,554 | 0,342 | (2,848; 4,261) | 0,954 | 0,92 | 0,91 | 0,097552 |
| 7 | 2,764 | 3,449 | 0,408 | (2,605; 4,292) | -0,685 | -0,67 | -0,67 | 0,138986 |
| 8 | 5,430 | 3,826 | 0,408 | (2,983; 4,670) | 1,604 | 1,58 | 1,64 | 0,138986 |
| 9 | 1,468 | 2,284 | 0,392 | (1,472; 3,096) | -0,816 | -0,80 | -0,79 | 0,128766 |
| 10 | 3,186 | 2,661 | 0,392 | (1,849; 3,473) | 0,525 | 0,51 | 0,51 | 0,128766 |
| 11 | 1,246 | 2,490 | 0,344 | (1,779; 3,201) | -1,244 | -1,20 | -1,21 | 0,098768 |
| 12 | 1,172 | 2,867 | 0,344 | (2,156; 3,578) | -1,695 | -1,63 | -1,70 | 0,098768 |
| 13 | 3,866 | 3,382 | 0,389 | (2,577; 4,187) | 0,484 | 0,47 | 0,47 | 0,126602 |
| 14 | 3,716 | 3,759 | 0,389 | (2,954; 4,564) | -0,044 | -0,04 | -0,04 | 0,126602 |
| 15 | 2,365 | 3,144 | 0,336 | (2,450; 3,838) | -0,779 | -0,75 | -0,74 | 0,094131 |
| 16 | 3,375 | 3,521 | 0,336 | (2,827; 4,216) | -0,147 | -0,14 | -0,14 | 0,094131 |
| 17 | 5,095 | 4,242 | 0,824 | (2,537; 5,947) | 0,853 | 1,19 | 1,20 | 0,567776 |
| 18 | 3,807 | 4,997 | 0,824 | (3,292; 6,702) | -1,190 | -1,66 | -1,73 | 0,567776 |
| 19 | 2,004 | 2,579 | 0,283 | (1,993; 3,165) | -0,575 | -0,54 | -0,54 | 0,067140 |
| 20 | 2,429 | 2,480 | 0,283 | (1,894; 3,065) | -0,051 | -0,05 | -0,05 | 0,066994 |
| 21 | 2,386 | 1,644 | 0,478 | (0,655; 2,634) | 0,742 | 0,75 | 0,75 | 0,191276 |
| 22 | 4,091 | 3,663 | 0,573 | (2,476; 4,849) | 0,428 | 0,46 | 0,45 | 0,274906 |
| 23 | 1,311 | 2,579 | 0,283 | (1,993; 3,165) | -1,268 | -1,20 | -1,21 | 0,067140 |
| 24 | 2,169 | 2,480 | 0,283 | (1,894; 3,065) | -0,311 | -0,29 | -0,29 | 0,066994 |
| 25 | 1,404 | 2,599 | 0,284 | (2,011; 3,187) | -1,195 | -1,13 | -1,14 | 0,067551 |
| 26 | 4,848 | 2,599 | 0,284 | (2,011; 3,187) | 2,250 | 2,13 | 2,33 | 0,067551 |
| 27 | 1,566 | 2,599 | 0,284 | (2,011; 3,187) | -1,033 | -0,98 | -0,98 | 0,067551 |

| Obs | Cook’s D | DFITS |  |  |
| --- | --- | --- | --- | --- |
| 1 | 0,13 | 0,77539 |  |  |
| 2 | 0,02 | 0,25227 |  |  |
| 3 | 0,01 | -0,15020 |  |  |
| 4 | 0,03 | 0,34736 |  |  |
| 5 | 0,00 | 0,05589 |  |  |
| 6 | 0,02 | 0,30082 |  |  |
| 7 | 0,02 | -0,26771 |  |  |
| 8 | 0,10 | 0,65758 |  |  |
| 9 | 0,02 | -0,30485 |  |  |
| 10 | 0,01 | 0,19436 |  |  |
| 11 | 0,04 | -0,40055 |  |  |
| 12 | 0,07 | -0,56217 |  |  |
| 13 | 0,01 | 0,17722 |  |  |
| 14 | 0,00 | -0,01600 |  |  |
| 15 | 0,01 | -0,23895 |  |  |
| 16 | 0,00 | -0,04444 |  |  |
| 17 | 0,46 | 1,37166 |  | X |
| 18 | 0,90 | -1,97719 |  | X |
| 19 | 0,01 | -0,14380 |  |  |
| 20 | 0,00 | -0,01262 |  |  |
| 21 | 0,03 | 0,36330 |  |  |
| 22 | 0,02 | 0,27825 |  |  |
| 23 | 0,03 | -0,32542 |  |  |
| 24 | 0,00 | -0,07731 |  |  |
| 25 | 0,02 | -0,30639 |  |  |
| 26 | 0,08 | 0,62583 | R |  |
| 27 | 0,02 | -0,26303 |  |  |

R  Large residual  
X  Unusual X

## Backward Elimination of Terms

α to remove = 0,1

## Coded Coefficients

| Term | Coef | SE Coef | 95% CI | T-Value | P-Value | VIF |
| --- | --- | --- | --- | --- | --- | --- |
| Constant | 2,473 | 0,283 | (1,879; 3,067) | 8,74 | 0,000 |  |
| Lac | 0,209 | 0,447 | (-0,730; 1,147) | 0,47 | 0,646 | 1,08 |
| HPMC\_Visc | -0,082 | 0,437 | (-1,001; 0,836) | -0,19 | 0,853 | 1,35 |
| HPMC\_HP | 1,200 | 0,465 | (0,222; 2,178) | 2,58 | 0,019 | 1,19 |
| HPMC\_PS | -0,212 | 0,617 | (-1,508; 1,084) | -0,34 | 0,735 | 1,48 |
| Lac\*Lac | 1,484 | 0,817 | (-0,234; 3,201) | 1,82 | 0,086 | 1,00 |
| Lac\*HPMC\_Visc | 1,647 | 0,874 | (-0,189; 3,484) | 1,88 | 0,076 | 1,08 |
| HPMC\_Visc\*HPMC\_HP | 2,09 | 1,09 | (-0,20; 4,38) | 1,92 | 0,071 | 2,16 |
| HPMC\_HP\*HPMC\_PS | 3,28 | 1,74 | (-0,39; 6,94) | 1,88 | 0,077 | 2,25 |

## Model Summary

| S | R-sq | R-sq(adj) | PRESS | R-sq(pred) | AICc | BIC |
| --- | --- | --- | --- | --- | --- | --- |
| 1,05512 | 47,67% | 24,41% | 46,6350 | 0,00% | 102,32 | 101,53 |

## Analysis of Variance

| Source | DF | Seq SS | Contribution | Adj SS | Adj MS | F-Value | P-Value |
| --- | --- | --- | --- | --- | --- | --- | --- |
| Model | 8 | 18,2549 | 47,67% | 18,2549 | 2,28186 | 2,05 | 0,098 |
| Linear | 4 | 5,8063 | 15,16% | 7,8889 | 1,97222 | 1,77 | 0,179 |
| Lac | 1 | 0,0014 | 0,00% | 0,2425 | 0,24253 | 0,22 | 0,646 |
| HPMC\_Visc | 1 | 0,2594 | 0,68% | 0,0396 | 0,03959 | 0,04 | 0,853 |
| HPMC\_HP | 1 | 3,4706 | 9,06% | 7,4028 | 7,40281 | 6,65 | 0,019 |
| HPMC\_PS | 1 | 2,0749 | 5,42% | 0,1314 | 0,13137 | 0,12 | 0,735 |
| Square | 1 | 3,7327 | 9,75% | 3,6684 | 3,66835 | 3,30 | 0,086 |
| Lac\*Lac | 1 | 3,7327 | 9,75% | 3,6684 | 3,66835 | 3,30 | 0,086 |
| 2-Way Interaction | 3 | 8,7160 | 22,76% | 8,7160 | 2,90534 | 2,61 | 0,083 |
| Lac\*HPMC\_Visc | 1 | 3,9534 | 10,32% | 3,9534 | 3,95345 | 3,55 | 0,076 |
| HPMC\_Visc\*HPMC\_HP | 1 | 0,8330 | 2,18% | 4,0897 | 4,08968 | 3,67 | 0,071 |
| HPMC\_HP\*HPMC\_PS | 1 | 3,9296 | 10,26% | 3,9296 | 3,92961 | 3,53 | 0,077 |
| Error | 18 | 20,0391 | 52,33% | 20,0391 | 1,11328 |  |  |
| Lack-of-Fit | 16 | 13,6416 | 35,62% | 13,6416 | 0,85260 | 0,27 | 0,954 |
| Pure Error | 2 | 6,3975 | 16,71% | 6,3975 | 3,19877 |  |  |
| Total | 26 | 38,2940 | 100,00% |  |  |  |  |

## Regression Equation in Uncoded Units

|  |  |  |
| --- | --- | --- |
| F\_SD\_17h(1020min) | = | 368 - 45,8 Lac - 0,00588 HPMC\_Visc - 36,5 HPMC\_HP - 4,19 HPMC\_PS + 23,7 Lac\*Lac + 0,001694 Lac\*HPMC\_Visc + 0,000529 HPMC\_Visc\*HPMC\_HP + 0,439 HPMC\_HP\*HPMC\_PS |

## Fits and Diagnostics for All Observations

| Obs | F\_SD\_17h(1020min) | Fit | SE Fit | 95% CI | Resid | Std Resid | Del Resid | HI |
| --- | --- | --- | --- | --- | --- | --- | --- | --- |
| 1 | 5,036 | 4,229 | 0,726 | (2,703; 5,755) | 0,807 | 1,05 | 1,06 | 0,473703 |
| 2 | 3,080 | 3,265 | 0,726 | (1,739; 4,791) | -0,185 | -0,24 | -0,23 | 0,473703 |
| 3 | 1,802 | 1,678 | 0,764 | (0,071; 3,284) | 0,124 | 0,17 | 0,17 | 0,524922 |
| 4 | 3,395 | 3,022 | 0,764 | (1,416; 4,628) | 0,373 | 0,51 | 0,50 | 0,524922 |
| 5 | 3,392 | 3,217 | 0,581 | (1,995; 4,438) | 0,175 | 0,20 | 0,19 | 0,303603 |
| 6 | 4,087 | 2,356 | 0,581 | (1,134; 3,577) | 1,732 | 1,97 | 2,16 | 0,303603 |
| 7 | 2,411 | 2,847 | 0,747 | (1,278; 4,416) | -0,436 | -0,59 | -0,57 | 0,501257 |
| 8 | 5,066 | 4,226 | 0,747 | (2,656; 5,795) | 0,841 | 1,13 | 1,14 | 0,501257 |
| 9 | 1,271 | 2,522 | 0,623 | (1,213; 3,832) | -1,251 | -1,47 | -1,52 | 0,348958 |
| 10 | 2,708 | 1,590 | 0,623 | (0,281; 2,900) | 1,117 | 1,31 | 1,34 | 0,348958 |
| 11 | 1,171 | 1,433 | 0,506 | (0,369; 2,497) | -0,261 | -0,28 | -0,28 | 0,230323 |
| 12 | 1,334 | 1,956 | 0,506 | (0,892; 3,019) | -0,622 | -0,67 | -0,66 | 0,230323 |
| 13 | 3,689 | 3,852 | 0,654 | (2,478; 5,226) | -0,163 | -0,20 | -0,19 | 0,384175 |
| 14 | 2,400 | 2,906 | 0,654 | (1,532; 4,280) | -0,506 | -0,61 | -0,60 | 0,384175 |
| 15 | 2,340 | 2,887 | 0,508 | (1,821; 3,953) | -0,547 | -0,59 | -0,58 | 0,231377 |
| 16 | 2,990 | 3,561 | 0,508 | (2,494; 4,627) | -0,571 | -0,62 | -0,61 | 0,231377 |
| 17 | 4,897 | 4,043 | 0,799 | (2,364; 5,721) | 0,855 | 1,24 | 1,26 | 0,573577 |
| 18 | 2,830 | 3,842 | 0,799 | (2,163; 5,520) | -1,011 | -1,47 | -1,52 | 0,573577 |
| 19 | 2,106 | 2,579 | 0,447 | (1,640; 3,518) | -0,473 | -0,49 | -0,48 | 0,179361 |
| 20 | 2,241 | 1,932 | 0,518 | (0,845; 3,020) | 0,309 | 0,34 | 0,33 | 0,240750 |
| 21 | 2,322 | 2,031 | 0,472 | (1,039; 3,024) | 0,291 | 0,31 | 0,30 | 0,200498 |
| 22 | 4,069 | 3,608 | 0,573 | (2,404; 4,812) | 0,461 | 0,52 | 0,51 | 0,294920 |
| 23 | 1,438 | 2,900 | 0,664 | (1,504; 4,296) | -1,462 | -1,78 | -1,91 | 0,396401 |
| 24 | 2,048 | 1,609 | 0,606 | (0,335; 2,882) | 0,439 | 0,51 | 0,50 | 0,329954 |
| 25 | 1,391 | 2,458 | 0,282 | (1,866; 3,051) | -1,067 | -1,05 | -1,05 | 0,071443 |
| 26 | 4,512 | 2,458 | 0,282 | (1,866; 3,051) | 2,053 | 2,02 | 2,23 | 0,071443 |
| 27 | 1,437 | 2,458 | 0,282 | (1,866; 3,051) | -1,021 | -1,00 | -1,00 | 0,071443 |

| Obs | Cook’s D | DFITS |  |
| --- | --- | --- | --- |
| 1 | 0,11 | 1,00373 |  |
| 2 | 0,01 | -0,22286 |  |
| 3 | 0,00 | 0,17483 |  |
| 4 | 0,03 | 0,52766 |  |
| 5 | 0,00 | 0,12797 |  |
| 6 | 0,19 | 1,42416 |  |
| 7 | 0,04 | -0,57575 |  |
| 8 | 0,14 | 1,14035 |  |
| 9 | 0,13 | -1,11430 |  |
| 10 | 0,10 | 0,98198 |  |
| 11 | 0,00 | -0,15048 |  |
| 12 | 0,01 | -0,36152 |  |
| 13 | 0,00 | -0,15168 |  |
| 14 | 0,03 | -0,47415 |  |
| 15 | 0,01 | -0,31861 |  |
| 16 | 0,01 | -0,33240 |  |
| 17 | 0,23 | 1,46178 |  |
| 18 | 0,32 | -1,76341 |  |
| 19 | 0,01 | -0,22641 |  |
| 20 | 0,00 | 0,18448 |  |
| 21 | 0,00 | 0,15027 |  |
| 22 | 0,01 | 0,32925 |  |
| 23 | 0,23 | -1,54780 |  |
| 24 | 0,01 | 0,34930 |  |
| 25 | 0,01 | -0,29207 |  |
| 26 | 0,03 | 0,61904 | R |
| 27 | 0,01 | -0,27860 |  |

R  Large residual

## Backward Elimination of Terms

α to remove = 0,1

## Coded Coefficients

| Term | Coef | SE Coef | 95% CI | T-Value | P-Value | VIF |
| --- | --- | --- | --- | --- | --- | --- |
| Constant | 2,517 | 0,216 | (2,071; 2,963) | 11,67 | 0,000 |  |
| Lac | -0,019 | 0,461 | (-0,974; 0,935) | -0,04 | 0,967 | 1,08 |
| HPMC\_Visc | -0,143 | 0,388 | (-0,945; 0,659) | -0,37 | 0,715 | 1,00 |
| Lac\*HPMC\_Visc | 1,576 | 0,902 | (-0,290; 3,443) | 1,75 | 0,094 | 1,08 |

## Model Summary

| S | R-sq | R-sq(adj) | PRESS | R-sq(pred) | AICc | BIC |
| --- | --- | --- | --- | --- | --- | --- |
| 1,08906 | 13,10% | 1,76% | 37,6915 | 0,00% | 89,76 | 93,38 |

## Analysis of Variance

| Source | DF | Seq SS | Contribution | Adj SS | Adj MS | F-Value | P-Value |
| --- | --- | --- | --- | --- | --- | --- | --- |
| Model | 3 | 4,1112 | 13,10% | 4,1112 | 1,37040 | 1,16 | 0,348 |
| Linear | 2 | 0,4903 | 1,56% | 0,1643 | 0,08217 | 0,07 | 0,933 |
| Lac | 1 | 0,3281 | 1,05% | 0,0021 | 0,00210 | 0,00 | 0,967 |
| HPMC\_Visc | 1 | 0,1622 | 0,52% | 0,1622 | 0,16224 | 0,14 | 0,715 |
| 2-Way Interaction | 1 | 3,6209 | 11,54% | 3,6209 | 3,62089 | 3,05 | 0,094 |
| Lac\*HPMC\_Visc | 1 | 3,6209 | 11,54% | 3,6209 | 3,62089 | 3,05 | 0,094 |
| Error | 23 | 27,2791 | 86,90% | 27,2791 | 1,18605 |  |  |
| Lack-of-Fit | 21 | 21,9151 | 69,81% | 21,9151 | 1,04358 | 0,39 | 0,900 |
| Pure Error | 2 | 5,3640 | 17,09% | 5,3640 | 2,68199 |  |  |
| Total | 26 | 31,3903 | 100,00% |  |  |  |  |

## Regression Equation in Uncoded Units

|  |  |  |
| --- | --- | --- |
| F\_SD\_18h(1080min) | = | 14,00 - 22,0 Lac - 0,000847 HPMC\_Visc + 0,001621 Lac\*HPMC\_Visc |

## Fits and Diagnostics for All Observations

| Obs | F\_SD\_18h(1080min) | Fit | SE Fit | 95% CI | Resid | Std Resid | Del Resid | HI |
| --- | --- | --- | --- | --- | --- | --- | --- | --- |
| 1 | 4,498 | 3,190 | 0,460 | (2,238; 4,141) | 1,308 | 1,32 | 1,35 | 0,178433 |
| 2 | 2,928 | 2,048 | 0,460 | (1,096; 3,000) | 0,880 | 0,89 | 0,89 | 0,178433 |
| 3 | 1,640 | 1,884 | 0,577 | (0,690; 3,078) | -0,244 | -0,26 | -0,26 | 0,280855 |
| 4 | 2,919 | 2,952 | 0,577 | (1,758; 4,146) | -0,032 | -0,04 | -0,03 | 0,280855 |
| 5 | 3,257 | 3,131 | 0,433 | (2,236; 4,027) | 0,125 | 0,13 | 0,12 | 0,157997 |
| 6 | 3,655 | 2,088 | 0,433 | (1,193; 2,984) | 1,567 | 1,57 | 1,62 | 0,157997 |
| 7 | 2,244 | 1,865 | 0,588 | (0,649; 3,081) | 0,379 | 0,41 | 0,41 | 0,291251 |
| 8 | 4,397 | 2,965 | 0,588 | (1,749; 4,181) | 1,431 | 1,56 | 1,61 | 0,291251 |
| 9 | 0,937 | 3,172 | 0,451 | (2,238; 4,105) | -2,234 | -2,25 | -2,50 | 0,171765 |
| 10 | 2,278 | 2,061 | 0,451 | (1,127; 2,994) | 0,217 | 0,22 | 0,21 | 0,171765 |
| 11 | 1,090 | 2,349 | 0,361 | (1,601; 3,096) | -1,259 | -1,23 | -1,24 | 0,110114 |
| 12 | 1,714 | 2,630 | 0,361 | (1,883; 3,378) | -0,916 | -0,89 | -0,89 | 0,110114 |
| 13 | 3,428 | 3,180 | 0,455 | (2,238; 4,121) | 0,248 | 0,25 | 0,25 | 0,174690 |
| 14 | 1,485 | 2,055 | 0,455 | (1,114; 2,997) | -0,570 | -0,58 | -0,57 | 0,174690 |
| 15 | 2,188 | 2,264 | 0,393 | (1,450; 3,077) | -0,076 | -0,07 | -0,07 | 0,130330 |
| 16 | 2,609 | 2,689 | 0,393 | (1,876; 3,503) | -0,080 | -0,08 | -0,08 | 0,130330 |
| 17 | 4,700 | 2,859 | 0,494 | (1,837; 3,881) | 1,841 | 1,90 | 2,02 | 0,205946 |
| 18 | 1,945 | 2,229 | 0,494 | (1,206; 3,251) | -0,284 | -0,29 | -0,29 | 0,205946 |
| 19 | 2,139 | 2,660 | 0,397 | (1,839; 3,481) | -0,521 | -0,51 | -0,51 | 0,132807 |
| 20 | 2,064 | 2,374 | 0,486 | (1,368; 3,379) | -0,309 | -0,32 | -0,31 | 0,199025 |
| 21 | 2,264 | 2,543 | 0,210 | (2,107; 2,978) | -0,279 | -0,26 | -0,26 | 0,037352 |
| 22 | 3,731 | 2,533 | 0,210 | (2,100; 2,967) | 1,198 | 1,12 | 1,13 | 0,037065 |
| 23 | 1,610 | 2,554 | 0,215 | (2,108; 3,000) | -0,944 | -0,88 | -0,88 | 0,039146 |
| 24 | 1,946 | 2,556 | 0,216 | (2,108; 3,003) | -0,609 | -0,57 | -0,56 | 0,039495 |
| 25 | 1,342 | 2,544 | 0,211 | (2,108; 2,980) | -1,202 | -1,13 | -1,13 | 0,037449 |
| 26 | 4,156 | 2,544 | 0,211 | (2,108; 2,980) | 1,612 | 1,51 | 1,55 | 0,037449 |
| 27 | 1,298 | 2,544 | 0,211 | (2,108; 2,980) | -1,246 | -1,17 | -1,18 | 0,037449 |

| Obs | Cook’s D | DFITS |  |
| --- | --- | --- | --- |
| 1 | 0,10 | 0,62832 |  |
| 2 | 0,04 | 0,41350 |  |
| 3 | 0,01 | -0,16178 |  |
| 4 | 0,00 | -0,02139 |  |
| 5 | 0,00 | 0,05310 |  |
| 6 | 0,12 | 0,70294 |  |
| 7 | 0,02 | 0,26026 |  |
| 8 | 0,25 | 1,03512 |  |
| 9 | 0,26 | -1,13744 | R |
| 10 | 0,00 | 0,09780 |  |
| 11 | 0,05 | -0,43605 |  |
| 12 | 0,02 | -0,31217 |  |
| 13 | 0,00 | 0,11300 |  |
| 14 | 0,02 | -0,26103 |  |
| 15 | 0,00 | -0,02832 |  |
| 16 | 0,00 | -0,02984 |  |
| 17 | 0,23 | 1,02883 |  |
| 18 | 0,01 | -0,14606 |  |
| 19 | 0,01 | -0,19787 |  |
| 20 | 0,01 | -0,15510 |  |
| 21 | 0,00 | -0,05039 |  |
| 22 | 0,01 | 0,22116 |  |
| 23 | 0,01 | -0,17763 |  |
| 24 | 0,00 | -0,11399 |  |
| 25 | 0,01 | -0,22330 |  |
| 26 | 0,02 | 0,30662 |  |
| 27 | 0,01 | -0,23197 |  |

R  Large residual

## Backward Elimination of Terms

α to remove = 0,1  
Backward elimination removed all terms from the model.

## Backward Elimination of Terms

α to remove = 0,1

## Coded Coefficients

| Term | Coef | SE Coef | 95% CI | T-Value | P-Value | VIF |
| --- | --- | --- | --- | --- | --- | --- |
| Constant | 2,039 | 0,170 | (1,688; 2,391) | 11,97 | 0,000 |  |
| Lac | -0,816 | 0,349 | (-1,536; -0,096) | -2,34 | 0,028 | 1,00 |
| HPMC\_HP | 0,613 | 0,346 | (-0,100; 1,327) | 1,77 | 0,089 | 1,00 |

## Model Summary

| S | R-sq | R-sq(adj) | PRESS | R-sq(pred) | AICc | BIC |
| --- | --- | --- | --- | --- | --- | --- |
| 0,854402 | 26,44% | 20,31% | 22,6990 | 4,69% | 74,76 | 78,13 |

## Analysis of Variance

| Source | DF | Seq SS | Contribution | Adj SS | Adj MS | F-Value | P-Value |
| --- | --- | --- | --- | --- | --- | --- | --- |
| Model | 2 | 6,296 | 26,44% | 6,296 | 3,1480 | 4,31 | 0,025 |
| Linear | 2 | 6,296 | 26,44% | 6,296 | 3,1480 | 4,31 | 0,025 |
| Lac | 1 | 3,999 | 16,79% | 3,999 | 3,9985 | 5,48 | 0,028 |
| HPMC\_HP | 1 | 2,297 | 9,65% | 2,297 | 2,2975 | 3,15 | 0,089 |
| Error | 24 | 17,520 | 73,56% | 17,520 | 0,7300 |  |  |
| Lack-of-Fit | 22 | 13,522 | 56,78% | 13,522 | 0,6146 | 0,31 | 0,942 |
| Pure Error | 2 | 3,998 | 16,79% | 3,998 | 1,9991 |  |  |
| Total | 26 | 23,816 | 100,00% |  |  |  |  |

## Regression Equation in Uncoded Units

|  |  |  |
| --- | --- | --- |
| F\_SD\_20h(1200min) | = | -2,05 - 3,27 Lac + 0,604 HPMC\_HP |

## Fits and Diagnostics for All Observations

| Obs | F\_SD\_20h(1200min) | Fit | SE Fit | 95% CI | Resid | Std Resid | Del Resid | HI |
| --- | --- | --- | --- | --- | --- | --- | --- | --- |
| 1 | 3,129 | 2,005 | 0,315 | (1,355; 2,655) | 1,124 | 1,42 | 1,45 | 0,135962 |
| 2 | 1,458 | 1,189 | 0,315 | (0,539; 1,839) | 0,270 | 0,34 | 0,33 | 0,135962 |
| 3 | 1,199 | 1,986 | 0,322 | (1,322; 2,651) | -0,787 | -0,99 | -0,99 | 0,142074 |
| 4 | 1,760 | 1,170 | 0,322 | (0,505; 1,835) | 0,590 | 0,75 | 0,74 | 0,142074 |
| 5 | 2,589 | 2,573 | 0,266 | (2,024; 3,123) | 0,016 | 0,02 | 0,02 | 0,097061 |
| 6 | 2,758 | 1,757 | 0,266 | (1,208; 2,306) | 1,001 | 1,23 | 1,25 | 0,097061 |
| 7 | 2,494 | 2,739 | 0,318 | (2,082; 3,395) | -0,244 | -0,31 | -0,30 | 0,138473 |
| 8 | 3,046 | 1,922 | 0,318 | (1,266; 2,578) | 1,124 | 1,42 | 1,45 | 0,138473 |
| 9 | 0,481 | 2,030 | 0,306 | (1,398; 2,662) | -1,549 | -1,94 | -2,07 | 0,128346 |
| 10 | 1,653 | 1,214 | 0,306 | (0,582; 1,846) | 0,439 | 0,55 | 0,54 | 0,128346 |
| 11 | 1,111 | 2,156 | 0,268 | (1,603; 2,709) | -1,045 | -1,29 | -1,31 | 0,098333 |
| 12 | 0,921 | 1,339 | 0,268 | (0,786; 1,892) | -0,418 | -0,52 | -0,51 | 0,098333 |
| 13 | 3,098 | 2,698 | 0,303 | (2,072; 3,324) | 0,400 | 0,50 | 0,49 | 0,126094 |
| 14 | 0,759 | 1,882 | 0,303 | (1,255; 2,508) | -1,123 | -1,41 | -1,44 | 0,126094 |
| 15 | 2,012 | 2,553 | 0,261 | (2,014; 3,093) | -0,541 | -0,67 | -0,66 | 0,093644 |
| 16 | 1,460 | 1,737 | 0,261 | (1,197; 2,277) | -0,276 | -0,34 | -0,33 | 0,093644 |
| 17 | 4,254 | 2,822 | 0,387 | (2,025; 3,620) | 1,432 | 1,88 | 1,99 | 0,204636 |
| 18 | 0,505 | 1,190 | 0,387 | (0,392; 1,987) | -0,685 | -0,90 | -0,89 | 0,204636 |
| 19 | 1,743 | 1,994 | 0,166 | (1,652; 2,336) | -0,251 | -0,30 | -0,29 | 0,037546 |
| 20 | 1,868 | 1,934 | 0,165 | (1,593; 2,274) | -0,065 | -0,08 | -0,08 | 0,037337 |
| 21 | 2,226 | 1,426 | 0,343 | (0,718; 2,134) | 0,800 | 1,02 | 1,02 | 0,161083 |
| 22 | 2,665 | 2,653 | 0,424 | (1,778; 3,527) | 0,012 | 0,02 | 0,02 | 0,245997 |
| 23 | 2,006 | 1,994 | 0,166 | (1,652; 2,336) | 0,012 | 0,01 | 0,01 | 0,037546 |
| 24 | 1,921 | 1,934 | 0,165 | (1,593; 2,274) | -0,013 | -0,02 | -0,01 | 0,037337 |
| 25 | 1,170 | 2,006 | 0,166 | (1,662; 2,350) | -0,836 | -1,00 | -1,00 | 0,037969 |
| 26 | 3,564 | 2,006 | 0,166 | (1,662; 2,350) | 1,558 | 1,86 | 1,97 | 0,037969 |
| 27 | 1,063 | 2,006 | 0,166 | (1,662; 2,350) | -0,943 | -1,13 | -1,13 | 0,037969 |

| Obs | Cook’s D | DFITS |
| --- | --- | --- |
| 1 | 0,11 | 0,57408 |
| 2 | 0,01 | 0,13209 |
| 3 | 0,05 | -0,40462 |
| 4 | 0,03 | 0,30071 |
| 5 | 0,00 | 0,00625 |
| 6 | 0,05 | 0,40908 |
| 7 | 0,01 | -0,12110 |
| 8 | 0,11 | 0,58099 |
| 9 | 0,19 | -0,79471 |
| 10 | 0,01 | 0,20791 |
| 11 | 0,06 | -0,43153 |
| 12 | 0,01 | -0,16761 |
| 13 | 0,01 | 0,18728 |
| 14 | 0,10 | -0,54567 |
| 15 | 0,02 | -0,21127 |
| 16 | 0,00 | -0,10719 |
| 17 | 0,30 | 1,01023 |
| 18 | 0,07 | -0,45393 |
| 19 | 0,00 | -0,05806 |
| 20 | 0,00 | -0,01503 |
| 21 | 0,07 | 0,44836 |
| 22 | 0,00 | 0,00930 |
| 23 | 0,00 | 0,00273 |
| 24 | 0,00 | -0,00294 |
| 25 | 0,01 | -0,19814 |
| 26 | 0,05 | 0,39068 |
| 27 | 0,02 | -0,22493 |

## Backward Elimination of Terms

α to remove = 0,1

## Coded Coefficients

| Term | Coef | SE Coef | 95% CI | T-Value | P-Value | VIF |
| --- | --- | --- | --- | --- | --- | --- |
| Constant | 1,815 | 0,165 | (1,475; 2,156) | 11,01 | 0,000 |  |
| Lac | -0,901 | 0,338 | (-1,597; -0,204) | -2,67 | 0,013 | 1,00 |
| HPMC\_HP | 0,587 | 0,335 | (-0,104; 1,277) | 1,75 | 0,092 | 1,00 |

## Model Summary

| S | R-sq | R-sq(adj) | PRESS | R-sq(pred) | AICc | BIC |
| --- | --- | --- | --- | --- | --- | --- |
| 0,826825 | 29,81% | 23,96% | 20,9962 | 10,18% | 72,99 | 76,36 |

## Analysis of Variance

| Source | DF | Seq SS | Contribution | Adj SS | Adj MS | F-Value | P-Value |
| --- | --- | --- | --- | --- | --- | --- | --- |
| Model | 2 | 6,968 | 29,81% | 6,968 | 3,4841 | 5,10 | 0,014 |
| Linear | 2 | 6,968 | 29,81% | 6,968 | 3,4841 | 5,10 | 0,014 |
| Lac | 1 | 4,867 | 20,82% | 4,867 | 4,8671 | 7,12 | 0,013 |
| HPMC\_HP | 1 | 2,101 | 8,99% | 2,101 | 2,1011 | 3,07 | 0,092 |
| Error | 24 | 16,407 | 70,19% | 16,407 | 0,6836 |  |  |
| Lack-of-Fit | 22 | 12,453 | 53,27% | 12,453 | 0,5660 | 0,29 | 0,952 |
| Pure Error | 2 | 3,955 | 16,92% | 3,955 | 1,9774 |  |  |
| Total | 26 | 23,376 | 100,00% |  |  |  |  |

## Regression Equation in Uncoded Units

|  |  |  |
| --- | --- | --- |
| F\_SD\_21h(1260min) | = | -1,86 - 3,60 Lac + 0,578 HPMC\_HP |

## Fits and Diagnostics for All Observations

| Obs | F\_SD\_21h(1260min) | Fit | SE Fit | 95% CI | Resid | Std Resid | Del Resid | HI |
| --- | --- | --- | --- | --- | --- | --- | --- | --- |
| 1 | 2,746 | 1,843 | 0,305 | (1,214; 2,472) | 0,903 | 1,18 | 1,18 | 0,135962 |
| 2 | 1,015 | 0,942 | 0,305 | (0,313; 1,571) | 0,072 | 0,09 | 0,09 | 0,135962 |
| 3 | 1,008 | 1,825 | 0,312 | (1,182; 2,468) | -0,817 | -1,07 | -1,07 | 0,142074 |
| 4 | 1,357 | 0,924 | 0,312 | (0,281; 1,567) | 0,433 | 0,57 | 0,56 | 0,142074 |
| 5 | 2,259 | 2,386 | 0,258 | (1,854; 2,918) | -0,127 | -0,16 | -0,16 | 0,097061 |
| 6 | 2,507 | 1,485 | 0,258 | (0,954; 2,017) | 1,022 | 1,30 | 1,32 | 0,097061 |
| 7 | 2,232 | 2,544 | 0,308 | (1,909; 3,179) | -0,312 | -0,41 | -0,40 | 0,138473 |
| 8 | 2,497 | 1,643 | 0,308 | (1,008; 2,278) | 0,853 | 1,11 | 1,12 | 0,138473 |
| 9 | 0,220 | 1,867 | 0,296 | (1,255; 2,478) | -1,647 | -2,13 | -2,32 | 0,128346 |
| 10 | 1,523 | 0,966 | 0,296 | (0,355; 1,577) | 0,557 | 0,72 | 0,71 | 0,128346 |
| 11 | 1,144 | 1,987 | 0,259 | (1,451; 2,522) | -0,842 | -1,07 | -1,08 | 0,098333 |
| 12 | 0,428 | 1,086 | 0,259 | (0,551; 1,621) | -0,658 | -0,84 | -0,83 | 0,098333 |
| 13 | 2,934 | 2,505 | 0,294 | (1,899; 3,111) | 0,429 | 0,55 | 0,55 | 0,126094 |
| 14 | 0,928 | 1,605 | 0,294 | (0,999; 2,211) | -0,676 | -0,87 | -0,87 | 0,126094 |
| 15 | 2,175 | 2,367 | 0,253 | (1,845; 2,889) | -0,192 | -0,24 | -0,24 | 0,093644 |
| 16 | 0,804 | 1,466 | 0,253 | (0,944; 1,988) | -0,662 | -0,84 | -0,84 | 0,093644 |
| 17 | 4,042 | 2,684 | 0,374 | (1,912; 3,456) | 1,358 | 1,84 | 1,95 | 0,204636 |
| 18 | 0,468 | 0,883 | 0,374 | (0,111; 1,655) | -0,415 | -0,56 | -0,55 | 0,204636 |
| 19 | 1,118 | 1,772 | 0,160 | (1,441; 2,103) | -0,654 | -0,81 | -0,80 | 0,037546 |
| 20 | 1,751 | 1,714 | 0,160 | (1,385; 2,044) | 0,037 | 0,05 | 0,04 | 0,037337 |
| 21 | 2,187 | 1,229 | 0,332 | (0,544; 1,914) | 0,958 | 1,27 | 1,28 | 0,161083 |
| 22 | 2,203 | 2,402 | 0,410 | (1,556; 3,248) | -0,199 | -0,28 | -0,27 | 0,245997 |
| 23 | 2,164 | 1,772 | 0,160 | (1,441; 2,103) | 0,392 | 0,48 | 0,47 | 0,037546 |
| 24 | 1,941 | 1,714 | 0,160 | (1,385; 2,044) | 0,227 | 0,28 | 0,27 | 0,037337 |
| 25 | 1,044 | 1,784 | 0,161 | (1,451; 2,116) | -0,739 | -0,91 | -0,91 | 0,037969 |
| 26 | 3,392 | 1,784 | 0,161 | (1,451; 2,116) | 1,608 | 1,98 | 2,12 | 0,037969 |
| 27 | 0,877 | 1,784 | 0,161 | (1,451; 2,116) | -0,907 | -1,12 | -1,12 | 0,037969 |

| Obs | Cook’s D | DFITS |  |
| --- | --- | --- | --- |
| 1 | 0,07 | 0,470037 |  |
| 2 | 0,00 | 0,036557 |  |
| 3 | 0,06 | -0,435267 |  |
| 4 | 0,02 | 0,226794 |  |
| 5 | 0,00 | -0,051812 |  |
| 6 | 0,06 | 0,432907 |  |
| 7 | 0,01 | -0,160354 |  |
| 8 | 0,07 | 0,448109 |  |
| 9 | 0,22 | -0,890463 | R |
| 10 | 0,03 | 0,273826 |  |
| 11 | 0,04 | -0,355411 |  |
| 12 | 0,03 | -0,275104 |  |
| 13 | 0,01 | 0,207481 |  |
| 14 | 0,04 | -0,330587 |  |
| 15 | 0,00 | -0,076942 |  |
| 16 | 0,02 | -0,268772 |  |
| 17 | 0,29 | 0,986773 |  |
| 18 | 0,03 | -0,281477 |  |
| 19 | 0,01 | -0,158134 |  |
| 20 | 0,00 | 0,008812 |  |
| 21 | 0,10 | 0,561974 |  |
| 22 | 0,01 | -0,155429 |  |
| 23 | 0,00 | 0,093779 |  |
| 24 | 0,00 | 0,053981 |  |
| 25 | 0,01 | -0,180416 |  |
| 26 | 0,05 | 0,421837 |  |
| 27 | 0,02 | -0,223283 |  |

R  Large residual

## Backward Elimination of Terms

α to remove = 0,1

## Coded Coefficients

| Term | Coef | SE Coef | 95% CI | T-Value | P-Value | VIF |
| --- | --- | --- | --- | --- | --- | --- |
| Constant | 1,623 | 0,158 | (1,298; 1,948) | 10,28 | 0,000 |  |
| Lac | -0,806 | 0,335 | (-1,496; -0,117) | -2,41 | 0,024 | 1,00 |

## Model Summary

| S | R-sq | R-sq(adj) | PRESS | R-sq(pred) | AICc | BIC |
| --- | --- | --- | --- | --- | --- | --- |
| 0,820273 | 18,83% | 15,58% | 20,1013 | 3,00% | 70,89 | 73,73 |

## Analysis of Variance

| Source | DF | Seq SS | Contribution | Adj SS | Adj MS | F-Value | P-Value |
| --- | --- | --- | --- | --- | --- | --- | --- |
| Model | 1 | 3,902 | 18,83% | 3,902 | 3,9023 | 5,80 | 0,024 |
| Linear | 1 | 3,902 | 18,83% | 3,902 | 3,9023 | 5,80 | 0,024 |
| Lac | 1 | 3,902 | 18,83% | 3,902 | 3,9023 | 5,80 | 0,024 |
| Error | 25 | 16,821 | 81,17% | 16,821 | 0,6728 |  |  |
| Lack-of-Fit | 23 | 13,305 | 64,20% | 13,305 | 0,5785 | 0,33 | 0,933 |
| Pure Error | 2 | 3,516 | 16,96% | 3,516 | 1,7578 |  |  |
| Total | 26 | 20,724 | 100,00% |  |  |  |  |

## Regression Equation in Uncoded Units

|  |  |  |
| --- | --- | --- |
| F\_SD\_22h(1320min) | = | 3,236 - 3,23 Lac |

## Fits and Diagnostics for All Observations

| Obs | F\_SD\_22h(1320min) | Fit | SE Fit | 95% CI | Resid | Std Resid | Del Resid | HI |
| --- | --- | --- | --- | --- | --- | --- | --- | --- |
| 1 | 2,285 | 2,026 | 0,230 | (1,553; 2,500) | 0,259 | 0,33 | 0,32 | 0,078704 |
| 2 | 0,989 | 1,220 | 0,230 | (0,746; 1,694) | -0,231 | -0,29 | -0,29 | 0,078704 |
| 3 | 0,982 | 2,026 | 0,230 | (1,553; 2,500) | -1,044 | -1,33 | -1,35 | 0,078704 |
| 4 | 1,133 | 1,220 | 0,230 | (0,746; 1,694) | -0,086 | -0,11 | -0,11 | 0,078704 |
| 5 | 2,072 | 2,026 | 0,230 | (1,553; 2,500) | 0,046 | 0,06 | 0,06 | 0,078704 |
| 6 | 2,392 | 1,220 | 0,230 | (0,746; 1,694) | 1,172 | 1,49 | 1,53 | 0,078704 |
| 7 | 2,087 | 2,026 | 0,230 | (1,553; 2,500) | 0,061 | 0,08 | 0,08 | 0,078704 |
| 8 | 1,987 | 1,220 | 0,230 | (0,746; 1,694) | 0,767 | 0,97 | 0,97 | 0,078704 |
| 9 | 0,128 | 2,026 | 0,230 | (1,553; 2,500) | -1,899 | -2,41 | -2,70 | 0,078704 |
| 10 | 1,836 | 1,220 | 0,230 | (0,746; 1,694) | 0,616 | 0,78 | 0,78 | 0,078704 |
| 11 | 1,131 | 2,026 | 0,230 | (1,553; 2,500) | -0,895 | -1,14 | -1,14 | 0,078704 |
| 12 | 0,507 | 1,220 | 0,230 | (0,746; 1,694) | -0,713 | -0,91 | -0,90 | 0,078704 |
| 13 | 2,784 | 2,026 | 0,230 | (1,553; 2,500) | 0,757 | 0,96 | 0,96 | 0,078704 |
| 14 | 1,004 | 1,220 | 0,230 | (0,746; 1,694) | -0,216 | -0,27 | -0,27 | 0,078704 |
| 15 | 2,151 | 2,026 | 0,230 | (1,553; 2,500) | 0,125 | 0,16 | 0,16 | 0,078704 |
| 16 | 0,788 | 1,220 | 0,230 | (0,746; 1,694) | -0,432 | -0,55 | -0,54 | 0,078704 |
| 17 | 3,830 | 2,430 | 0,370 | (1,667; 3,192) | 1,400 | 1,91 | 2,03 | 0,203704 |
| 18 | 0,484 | 0,817 | 0,370 | (0,054; 1,579) | -0,333 | -0,46 | -0,45 | 0,203704 |
| 19 | 0,663 | 1,623 | 0,158 | (1,298; 1,948) | -0,960 | -1,19 | -1,20 | 0,037037 |
| 20 | 1,577 | 1,623 | 0,158 | (1,298; 1,948) | -0,046 | -0,06 | -0,06 | 0,037037 |
| 21 | 2,163 | 1,623 | 0,158 | (1,298; 1,948) | 0,540 | 0,67 | 0,66 | 0,037037 |
| 22 | 1,869 | 1,623 | 0,158 | (1,298; 1,948) | 0,245 | 0,30 | 0,30 | 0,037037 |
| 23 | 2,373 | 1,623 | 0,158 | (1,298; 1,948) | 0,750 | 0,93 | 0,93 | 0,037037 |
| 24 | 1,911 | 1,623 | 0,158 | (1,298; 1,948) | 0,287 | 0,36 | 0,35 | 0,037037 |
| 25 | 0,917 | 1,623 | 0,158 | (1,298; 1,948) | -0,706 | -0,88 | -0,87 | 0,037037 |
| 26 | 3,092 | 1,623 | 0,158 | (1,298; 1,948) | 1,469 | 1,83 | 1,92 | 0,037037 |
| 27 | 0,691 | 1,623 | 0,158 | (1,298; 1,948) | -0,932 | -1,16 | -1,17 | 0,037037 |

| Obs | Cook’s D | DFITS |  |
| --- | --- | --- | --- |
| 1 | 0,00 | 0,09435 |  |
| 2 | 0,00 | -0,08417 |  |
| 3 | 0,08 | -0,39397 |  |
| 4 | 0,00 | -0,03147 |  |
| 5 | 0,00 | 0,01675 |  |
| 6 | 0,09 | 0,44658 |  |
| 7 | 0,00 | 0,02217 |  |
| 8 | 0,04 | 0,28446 |  |
| 9 | 0,25 | -0,78843 | R |
| 10 | 0,03 | 0,22677 |  |
| 11 | 0,06 | -0,33443 |  |
| 12 | 0,04 | -0,26365 |  |
| 13 | 0,04 | 0,28062 |  |
| 14 | 0,00 | -0,07870 |  |
| 15 | 0,00 | 0,04542 |  |
| 16 | 0,01 | -0,15822 |  |
| 17 | 0,47 | 1,02620 |  |
| 18 | 0,03 | -0,22643 |  |
| 19 | 0,03 | -0,23601 |  |
| 20 | 0,00 | -0,01107 |  |
| 21 | 0,01 | 0,13013 |  |
| 22 | 0,00 | 0,05870 |  |
| 23 | 0,02 | 0,18211 |  |
| 24 | 0,00 | 0,06879 |  |
| 25 | 0,01 | -0,17116 |  |
| 26 | 0,06 | 0,37669 |  |
| 27 | 0,03 | -0,22872 |  |

R  Large residual

## Backward Elimination of Terms

α to remove = 0,1

## Coded Coefficients

| Term | Coef | SE Coef | 95% CI | T-Value | P-Value | VIF |
| --- | --- | --- | --- | --- | --- | --- |
| Constant | 1,565 | 0,147 | (1,263; 1,866) | 10,68 | 0,000 |  |
| Lac | -0,731 | 0,311 | (-1,371; -0,090) | -2,35 | 0,027 | 1,00 |

## Model Summary

| S | R-sq | R-sq(adj) | PRESS | R-sq(pred) | AICc | BIC |
| --- | --- | --- | --- | --- | --- | --- |
| 0,761327 | 18,10% | 14,82% | 17,2606 | 2,44% | 66,86 | 69,71 |

## Analysis of Variance

| Source | DF | Seq SS | Contribution | Adj SS | Adj MS | F-Value | P-Value |
| --- | --- | --- | --- | --- | --- | --- | --- |
| Model | 1 | 3,202 | 18,10% | 3,202 | 3,2024 | 5,53 | 0,027 |
| Linear | 1 | 3,202 | 18,10% | 3,202 | 3,2024 | 5,53 | 0,027 |
| Lac | 1 | 3,202 | 18,10% | 3,202 | 3,2024 | 5,53 | 0,027 |
| Error | 25 | 14,490 | 81,90% | 14,490 | 0,5796 |  |  |
| Lack-of-Fit | 23 | 11,598 | 65,55% | 11,598 | 0,5042 | 0,35 | 0,923 |
| Pure Error | 2 | 2,893 | 16,35% | 2,893 | 1,4464 |  |  |
| Total | 26 | 17,693 | 100,00% |  |  |  |  |

## Regression Equation in Uncoded Units

|  |  |  |
| --- | --- | --- |
| F\_SD\_23h(1380min) | = | 3,026 - 2,92 Lac |

## Fits and Diagnostics for All Observations

| Obs | F\_SD\_23h(1380min) | Fit | SE Fit | 95% CI | Resid | Std Resid | Del Resid | HI |
| --- | --- | --- | --- | --- | --- | --- | --- | --- |
| 1 | 2,044 | 1,930 | 0,214 | (1,490; 2,370) | 0,114 | 0,16 | 0,15 | 0,078704 |
| 2 | 0,982 | 1,199 | 0,214 | (0,759; 1,639) | -0,218 | -0,30 | -0,29 | 0,078704 |
| 3 | 1,279 | 1,930 | 0,214 | (1,490; 2,370) | -0,651 | -0,89 | -0,89 | 0,078704 |
| 4 | 1,058 | 1,199 | 0,214 | (0,759; 1,639) | -0,141 | -0,19 | -0,19 | 0,078704 |
| 5 | 2,050 | 1,930 | 0,214 | (1,490; 2,370) | 0,121 | 0,16 | 0,16 | 0,078704 |
| 6 | 2,357 | 1,199 | 0,214 | (0,759; 1,639) | 1,158 | 1,58 | 1,64 | 0,078704 |
| 7 | 2,012 | 1,930 | 0,214 | (1,490; 2,370) | 0,082 | 0,11 | 0,11 | 0,078704 |
| 8 | 1,412 | 1,199 | 0,214 | (0,759; 1,639) | 0,213 | 0,29 | 0,29 | 0,078704 |
| 9 | 0,238 | 1,930 | 0,214 | (1,490; 2,370) | -1,692 | -2,32 | -2,56 | 0,078704 |
| 10 | 2,053 | 1,199 | 0,214 | (0,759; 1,639) | 0,854 | 1,17 | 1,18 | 0,078704 |
| 11 | 1,179 | 1,930 | 0,214 | (1,490; 2,370) | -0,751 | -1,03 | -1,03 | 0,078704 |
| 12 | 0,755 | 1,199 | 0,214 | (0,759; 1,639) | -0,444 | -0,61 | -0,60 | 0,078704 |
| 13 | 2,569 | 1,930 | 0,214 | (1,490; 2,370) | 0,639 | 0,87 | 0,87 | 0,078704 |
| 14 | 1,035 | 1,199 | 0,214 | (0,759; 1,639) | -0,164 | -0,22 | -0,22 | 0,078704 |
| 15 | 1,866 | 1,930 | 0,214 | (1,490; 2,370) | -0,064 | -0,09 | -0,09 | 0,078704 |
| 16 | 1,138 | 1,199 | 0,214 | (0,759; 1,639) | -0,061 | -0,08 | -0,08 | 0,078704 |
| 17 | 3,641 | 2,295 | 0,344 | (1,587; 3,003) | 1,345 | 1,98 | 2,11 | 0,203704 |
| 18 | 0,480 | 0,834 | 0,344 | (0,126; 1,542) | -0,354 | -0,52 | -0,51 | 0,203704 |
| 19 | 0,418 | 1,565 | 0,147 | (1,263; 1,866) | -1,147 | -1,53 | -1,58 | 0,037037 |
| 20 | 1,423 | 1,565 | 0,147 | (1,263; 1,866) | -0,141 | -0,19 | -0,19 | 0,037037 |
| 21 | 2,115 | 1,565 | 0,147 | (1,263; 1,866) | 0,550 | 0,74 | 0,73 | 0,037037 |
| 22 | 1,593 | 1,565 | 0,147 | (1,263; 1,866) | 0,029 | 0,04 | 0,04 | 0,037037 |
| 23 | 2,596 | 1,565 | 0,147 | (1,263; 1,866) | 1,031 | 1,38 | 1,41 | 0,037037 |
| 24 | 1,904 | 1,565 | 0,147 | (1,263; 1,866) | 0,339 | 0,45 | 0,45 | 0,037037 |
| 25 | 0,766 | 1,565 | 0,147 | (1,263; 1,866) | -0,798 | -1,07 | -1,07 | 0,037037 |
| 26 | 2,732 | 1,565 | 0,147 | (1,263; 1,866) | 1,168 | 1,56 | 1,61 | 0,037037 |
| 27 | 0,549 | 1,565 | 0,147 | (1,263; 1,866) | -1,015 | -1,36 | -1,38 | 0,037037 |

| Obs | Cook’s D | DFITS |  |
| --- | --- | --- | --- |
| 1 | 0,00 | 0,04462 |  |
| 2 | 0,00 | -0,08539 |  |
| 3 | 0,03 | -0,25926 |  |
| 4 | 0,00 | -0,05534 |  |
| 5 | 0,00 | 0,04728 |  |
| 6 | 0,11 | 0,47840 |  |
| 7 | 0,00 | 0,03204 |  |
| 8 | 0,00 | 0,08342 |  |
| 9 | 0,23 | -0,74808 | R |
| 10 | 0,06 | 0,34407 |  |
| 11 | 0,05 | -0,30061 |  |
| 12 | 0,02 | -0,17541 |  |
| 13 | 0,03 | 0,25422 |  |
| 14 | 0,00 | -0,06442 |  |
| 15 | 0,00 | -0,02516 |  |
| 16 | 0,00 | -0,02409 |  |
| 17 | 0,50 | 1,06888 |  |
| 18 | 0,03 | -0,25944 |  |
| 19 | 0,05 | -0,30984 |  |
| 20 | 0,00 | -0,03639 |  |
| 21 | 0,01 | 0,14313 |  |
| 22 | 0,00 | 0,00742 |  |
| 23 | 0,04 | 0,27601 |  |
| 24 | 0,00 | 0,08756 |  |
| 25 | 0,02 | -0,21018 |  |
| 26 | 0,05 | 0,31619 |  |
| 27 | 0,04 | -0,27138 |  |

R  Large residual

## Backward Elimination of Terms

α to remove = 0,1

## Coded Coefficients

| Term | Coef | SE Coef | 95% CI | T-Value | P-Value | VIF |
| --- | --- | --- | --- | --- | --- | --- |
| Constant | 1,505 | 0,142 | (1,212; 1,798) | 10,59 | 0,000 |  |
| Lac | -0,606 | 0,302 | (-1,226; 0,015) | -2,01 | 0,056 | 1,00 |

## Model Summary

| S | R-sq | R-sq(adj) | PRESS | R-sq(pred) | AICc | BIC |
| --- | --- | --- | --- | --- | --- | --- |
| 0,738535 | 13,89% | 10,45% | 16,2535 | 0,00% | 65,22 | 68,07 |

## Analysis of Variance

| Source | DF | Seq SS | Contribution | Adj SS | Adj MS | F-Value | P-Value |
| --- | --- | --- | --- | --- | --- | --- | --- |
| Model | 1 | 2,200 | 13,89% | 2,200 | 2,1998 | 4,03 | 0,056 |
| Linear | 1 | 2,200 | 13,89% | 2,200 | 2,1998 | 4,03 | 0,056 |
| Lac | 1 | 2,200 | 13,89% | 2,200 | 2,1998 | 4,03 | 0,056 |
| Error | 25 | 13,636 | 86,11% | 13,636 | 0,5454 |  |  |
| Lack-of-Fit | 23 | 11,529 | 72,81% | 11,529 | 0,5013 | 0,48 | 0,855 |
| Pure Error | 2 | 2,107 | 13,30% | 2,107 | 1,0533 |  |  |
| Total | 26 | 15,836 | 100,00% |  |  |  |  |

## Regression Equation in Uncoded Units

|  |  |  |
| --- | --- | --- |
| F\_SD\_24h(1440min) | = | 2,716 - 2,42 Lac |

## Fits and Diagnostics for All Observations

| Obs | F\_SD\_24h(1440min) | Fit | SE Fit | 95% CI | Resid | Std Resid | Del Resid | HI |
| --- | --- | --- | --- | --- | --- | --- | --- | --- |
| 1 | 1,825 | 1,808 | 0,207 | (1,381; 2,235) | 0,017 | 0,02 | 0,02 | 0,078704 |
| 2 | 1,026 | 1,202 | 0,207 | (0,776; 1,629) | -0,176 | -0,25 | -0,24 | 0,078704 |
| 3 | 1,494 | 1,808 | 0,207 | (1,381; 2,235) | -0,314 | -0,44 | -0,44 | 0,078704 |
| 4 | 1,025 | 1,202 | 0,207 | (0,776; 1,629) | -0,177 | -0,25 | -0,25 | 0,078704 |
| 5 | 1,906 | 1,808 | 0,207 | (1,381; 2,235) | 0,098 | 0,14 | 0,14 | 0,078704 |
| 6 | 2,367 | 1,202 | 0,207 | (0,776; 1,629) | 1,164 | 1,64 | 1,70 | 0,078704 |
| 7 | 1,833 | 1,808 | 0,207 | (1,381; 2,235) | 0,025 | 0,04 | 0,03 | 0,078704 |
| 8 | 0,989 | 1,202 | 0,207 | (0,776; 1,629) | -0,213 | -0,30 | -0,30 | 0,078704 |
| 9 | 0,346 | 1,808 | 0,207 | (1,381; 2,235) | -1,461 | -2,06 | -2,22 | 0,078704 |
| 10 | 2,241 | 1,202 | 0,207 | (0,776; 1,629) | 1,039 | 1,47 | 1,50 | 0,078704 |
| 11 | 1,214 | 1,808 | 0,207 | (1,381; 2,235) | -0,594 | -0,84 | -0,83 | 0,078704 |
| 12 | 0,987 | 1,202 | 0,207 | (0,776; 1,629) | -0,215 | -0,30 | -0,30 | 0,078704 |
| 13 | 2,374 | 1,808 | 0,207 | (1,381; 2,235) | 0,566 | 0,80 | 0,79 | 0,078704 |
| 14 | 1,133 | 1,202 | 0,207 | (0,776; 1,629) | -0,070 | -0,10 | -0,10 | 0,078704 |
| 15 | 1,566 | 1,808 | 0,207 | (1,381; 2,235) | -0,242 | -0,34 | -0,34 | 0,078704 |
| 16 | 1,464 | 1,202 | 0,207 | (0,776; 1,629) | 0,262 | 0,37 | 0,36 | 0,078704 |
| 17 | 3,454 | 2,111 | 0,333 | (1,424; 2,797) | 1,343 | 2,04 | 2,19 | 0,203704 |
| 18 | 0,483 | 0,900 | 0,333 | (0,213; 1,586) | -0,417 | -0,63 | -0,62 | 0,203704 |
| 19 | 0,254 | 1,505 | 0,142 | (1,212; 1,798) | -1,252 | -1,73 | -1,80 | 0,037037 |
| 20 | 1,178 | 1,505 | 0,142 | (1,212; 1,798) | -0,327 | -0,45 | -0,44 | 0,037037 |
| 21 | 2,065 | 1,505 | 0,142 | (1,212; 1,798) | 0,560 | 0,77 | 0,77 | 0,037037 |
| 22 | 1,415 | 1,505 | 0,142 | (1,212; 1,798) | -0,090 | -0,12 | -0,12 | 0,037037 |
| 23 | 2,669 | 1,505 | 0,142 | (1,212; 1,798) | 1,164 | 1,61 | 1,66 | 0,037037 |
| 24 | 1,911 | 1,505 | 0,142 | (1,212; 1,798) | 0,406 | 0,56 | 0,55 | 0,037037 |
| 25 | 0,649 | 1,505 | 0,142 | (1,212; 1,798) | -0,857 | -1,18 | -1,19 | 0,037037 |
| 26 | 2,320 | 1,505 | 0,142 | (1,212; 1,798) | 0,815 | 1,12 | 1,13 | 0,037037 |
| 27 | 0,453 | 1,505 | 0,142 | (1,212; 1,798) | -1,052 | -1,45 | -1,49 | 0,037037 |

| Obs | Cook’s D | DFITS |  |
| --- | --- | --- | --- |
| 1 | 0,00 | 0,00671 |  |
| 2 | 0,00 | -0,07134 |  |
| 3 | 0,01 | -0,12728 |  |
| 4 | 0,00 | -0,07174 |  |
| 5 | 0,00 | 0,03955 |  |
| 6 | 0,12 | 0,49794 |  |
| 7 | 0,00 | 0,01006 |  |
| 8 | 0,00 | -0,08625 |  |
| 9 | 0,18 | -0,64806 | R |
| 10 | 0,09 | 0,43901 |  |
| 11 | 0,03 | -0,24349 |  |
| 12 | 0,00 | -0,08718 |  |
| 13 | 0,03 | 0,23151 |  |
| 14 | 0,00 | -0,02822 |  |
| 15 | 0,00 | -0,09800 |  |
| 16 | 0,01 | 0,10605 |  |
| 17 | 0,53 | 1,10600 | R |
| 18 | 0,05 | -0,31573 |  |
| 19 | 0,06 | -0,35359 |  |
| 20 | 0,00 | -0,08710 |  |
| 21 | 0,01 | 0,15026 |  |
| 22 | 0,00 | -0,02390 |  |
| 23 | 0,05 | 0,32588 |  |
| 24 | 0,01 | 0,10819 |  |
| 25 | 0,03 | -0,23375 |  |
| 26 | 0,02 | 0,22180 |  |
| 27 | 0,04 | -0,29152 |  |

R  Large residual
