# Supplementary material for: Influence of Selected Hypromellose Functionality-Related Characteristics and Soluble/Insoluble Filler Ratio on Carvedilol Release from Matrix Tablets
Source: Pharmaceutics. 2025 Oct 21;17(10):1358. doi: 10.3390/pharmaceutics17101358 (PMC12566823; doi:10.3390/pharmaceutics17101358)
Supplement: Supplementary file 1 [file pharmaceutics-17-01358-s001.zip › Report_SD of Release Analysis_RSM(CCD)_Stepwise, Forward Selection.htm]

# SD of Release Analysis, Response Surface Design (Central Composite Design), Stepwise Regression - Forward Selection

## Forward Selection of Terms

α to enter = 0,25

## Coded Coefficients

| Term | Coef | SE Coef | 95% CI | T-Value | P-Value | VIF |
| --- | --- | --- | --- | --- | --- | --- |
| Constant | 1,318 | 0,200 | (0,904; 1,732) | 6,60 | 0,000 |  |
| Lac | 1,034 | 0,323 | (0,364; 1,704) | 3,20 | 0,004 | 1,08 |
| HPMC\_Visc | -0,457 | 0,271 | (-1,019; 0,106) | -1,68 | 0,107 | 1,00 |
| Lac\*Lac | 1,166 | 0,591 | (-0,059; 2,391) | 1,97 | 0,061 | 1,00 |
| Lac\*HPMC\_Visc | -1,340 | 0,632 | (-2,650; -0,030) | -2,12 | 0,045 | 1,08 |

## Model Summary

| S | R-sq | R-sq(adj) | PRESS | R-sq(pred) | AICc | BIC |
| --- | --- | --- | --- | --- | --- | --- |
| 0,762443 | 54,69% | 46,45% | 19,1336 | 32,21% | 72,65 | 76,22 |

## Analysis of Variance

| Source | DF | Seq SS | Contribution | Adj SS | Adj MS | F-Value | P-Value |
| --- | --- | --- | --- | --- | --- | --- | --- |
| Model | 4 | 15,436 | 54,69% | 15,436 | 3,8590 | 6,64 | 0,001 |
| Linear | 2 | 10,554 | 37,39% | 7,604 | 3,8022 | 6,54 | 0,006 |
| Lac | 1 | 8,874 | 31,44% | 5,960 | 5,9595 | 10,25 | 0,004 |
| HPMC\_Visc | 1 | 1,680 | 5,95% | 1,645 | 1,6449 | 2,83 | 0,107 |
| Square | 1 | 2,267 | 8,03% | 2,267 | 2,2669 | 3,90 | 0,061 |
| Lac\*Lac | 1 | 2,267 | 8,03% | 2,267 | 2,2669 | 3,90 | 0,061 |
| 2-Way Interaction | 1 | 2,616 | 9,27% | 2,616 | 2,6155 | 4,50 | 0,045 |
| Lac\*HPMC\_Visc | 1 | 2,616 | 9,27% | 2,616 | 2,6155 | 4,50 | 0,045 |
| Error | 22 | 12,789 | 45,31% | 12,789 | 0,5813 |  |  |
| Lack-of-Fit | 20 | 10,190 | 36,10% | 10,190 | 0,5095 | 0,39 | 0,897 |
| Pure Error | 2 | 2,599 | 9,21% | 2,599 | 1,2993 |  |  |
| Total | 26 | 28,225 | 100,00% |  |  |  |  |

## Regression Equation in Uncoded Units

|  |  |  |
| --- | --- | --- |
| F\_SD\_0.17h(10min) | = | -3,81 + 4,1 Lac + 0,000571 HPMC\_Visc + 18,66 Lac\*Lac - 0,001378 Lac\*HPMC\_Visc |

## Fits and Diagnostics for All Observations

| Obs | F\_SD\_0.17h(10min) | Fit | SE Fit | 95% CI | Resid | Std Resid | Del Resid | HI |
| --- | --- | --- | --- | --- | --- | --- | --- | --- |
| 1 | 1,134 | 0,941 | 0,322 | (0,272; 1,609) | 0,194 | 0,28 | 0,27 | 0,178821 |
| 2 | 2,346 | 2,928 | 0,322 | (2,260; 3,597) | -0,583 | -0,84 | -0,84 | 0,178821 |
| 3 | 0,741 | 1,240 | 0,404 | (0,401; 2,079) | -0,499 | -0,77 | -0,76 | 0,281436 |
| 4 | 0,785 | 1,350 | 0,404 | (0,511; 2,189) | -0,565 | -0,87 | -0,87 | 0,281436 |
| 5 | 0,860 | 0,954 | 0,303 | (0,325; 1,583) | -0,094 | -0,13 | -0,13 | 0,158392 |
| 6 | 5,070 | 2,858 | 0,303 | (2,229; 3,487) | 2,212 | 3,16 | 4,18 | 0,158392 |
| 7 | 0,912 | 1,244 | 0,412 | (0,390; 2,098) | -0,332 | -0,52 | -0,51 | 0,291836 |
| 8 | 1,271 | 1,326 | 0,412 | (0,472; 2,180) | -0,056 | -0,09 | -0,08 | 0,291836 |
| 9 | 1,144 | 0,945 | 0,316 | (0,289; 1,601) | 0,199 | 0,29 | 0,28 | 0,172155 |
| 10 | 2,506 | 2,906 | 0,316 | (2,250; 3,562) | -0,401 | -0,58 | -0,57 | 0,172155 |
| 11 | 0,245 | 1,133 | 0,254 | (0,607; 1,659) | -0,888 | -1,24 | -1,25 | 0,110622 |
| 12 | 1,852 | 1,912 | 0,254 | (1,386; 2,438) | -0,060 | -0,08 | -0,08 | 0,110622 |
| 13 | 0,750 | 0,943 | 0,319 | (0,281; 1,605) | -0,193 | -0,28 | -0,27 | 0,175079 |
| 14 | 2,649 | 2,916 | 0,319 | (2,255; 3,578) | -0,268 | -0,39 | -0,38 | 0,175079 |
| 15 | 1,393 | 1,153 | 0,276 | (0,581; 1,725) | 0,240 | 0,34 | 0,33 | 0,130851 |
| 16 | 1,650 | 1,809 | 0,276 | (1,237; 2,380) | -0,158 | -0,22 | -0,22 | 0,130851 |
| 17 | 1,815 | 1,285 | 0,575 | (0,092; 2,477) | 0,530 | 1,06 | 1,06 | 0,568720 |
| 18 | 3,638 | 3,855 | 0,575 | (2,663; 5,048) | -0,217 | -0,43 | -0,43 | 0,568720 |
| 19 | 1,034 | 1,775 | 0,308 | (1,135; 2,414) | -0,741 | -1,06 | -1,07 | 0,163395 |
| 20 | 2,043 | 0,861 | 0,364 | (0,107; 1,615) | 1,182 | 1,76 | 1,86 | 0,227435 |
| 21 | 1,406 | 1,400 | 0,197 | (0,991; 1,810) | 0,005 | 0,01 | 0,01 | 0,067038 |
| 22 | 1,178 | 1,371 | 0,197 | (0,962; 1,779) | -0,192 | -0,26 | -0,26 | 0,066681 |
| 23 | 1,271 | 1,436 | 0,200 | (1,021; 1,852) | -0,166 | -0,23 | -0,22 | 0,068919 |
| 24 | 1,024 | 1,441 | 0,201 | (1,025; 1,857) | -0,417 | -0,57 | -0,56 | 0,069279 |
| 25 | 0,676 | 1,404 | 0,198 | (0,994; 1,813) | -0,727 | -0,99 | -0,99 | 0,067143 |
| 26 | 1,846 | 1,404 | 0,198 | (0,994; 1,813) | 0,442 | 0,60 | 0,59 | 0,067143 |
| 27 | 2,956 | 1,404 | 0,198 | (0,994; 1,813) | 1,552 | 2,11 | 2,31 | 0,067143 |

| Obs | Cook’s D | DFITS |  |  |
| --- | --- | --- | --- | --- |
| 1 | 0,00 | 0,12804 |  |  |
| 2 | 0,03 | -0,39079 |  |  |
| 3 | 0,05 | -0,47835 |  |  |
| 4 | 0,06 | -0,54404 |  |  |
| 5 | 0,00 | -0,05693 |  |  |
| 6 | 0,38 | 1,81479 | R |  |
| 7 | 0,02 | -0,32685 |  |  |
| 8 | 0,00 | -0,05429 |  |  |
| 9 | 0,00 | 0,12784 |  |  |
| 10 | 0,01 | -0,25925 |  |  |
| 11 | 0,04 | -0,44121 |  |  |
| 12 | 0,00 | -0,02880 |  |  |
| 13 | 0,00 | -0,12547 |  |  |
| 14 | 0,01 | -0,17451 |  |  |
| 15 | 0,00 | 0,12827 |  |  |
| 16 | 0,00 | -0,08460 |  |  |
| 17 | 0,30 | 1,21988 |  | X |
| 18 | 0,05 | -0,48940 |  | X |
| 19 | 0,04 | -0,47084 |  |  |
| 20 | 0,18 | 1,00875 |  |  |
| 21 | 0,00 | 0,00188 |  |  |
| 22 | 0,00 | -0,06834 |  |  |
| 23 | 0,00 | -0,05992 |  |  |
| 24 | 0,00 | -0,15212 |  |  |
| 25 | 0,01 | -0,26478 |  |  |
| 26 | 0,01 | 0,15869 |  |  |
| 27 | 0,06 | 0,61848 | R |  |

R  Large residual  
X  Unusual X

## Forward Selection of Terms

α to enter = 0,25

## Coded Coefficients

| Term | Coef | SE Coef | 95% CI | T-Value | P-Value | VIF |
| --- | --- | --- | --- | --- | --- | --- |
| Constant | 1,524 | 0,206 | (1,097; 1,951) | 7,40 | 0,000 |  |
| Lac | 0,902 | 0,333 | (0,212; 1,592) | 2,71 | 0,013 | 1,08 |
| HPMC\_Visc | -0,545 | 0,280 | (-1,124; 0,035) | -1,95 | 0,064 | 1,00 |
| Lac\*Lac | 1,213 | 0,608 | (-0,049; 2,475) | 1,99 | 0,059 | 1,00 |
| Lac\*HPMC\_Visc | -1,424 | 0,651 | (-2,773; -0,074) | -2,19 | 0,040 | 1,08 |

## Model Summary

| S | R-sq | R-sq(adj) | PRESS | R-sq(pred) | AICc | BIC |
| --- | --- | --- | --- | --- | --- | --- |
| 0,785404 | 52,49% | 43,85% | 19,9130 | 30,28% | 74,25 | 77,82 |

## Analysis of Variance

| Source | DF | Seq SS | Contribution | Adj SS | Adj MS | F-Value | P-Value |
| --- | --- | --- | --- | --- | --- | --- | --- |
| Model | 4 | 14,992 | 52,49% | 14,992 | 3,7481 | 6,08 | 0,002 |
| Linear | 2 | 9,588 | 33,57% | 6,878 | 3,4389 | 5,57 | 0,011 |
| Lac | 1 | 7,204 | 25,22% | 4,537 | 4,5373 | 7,36 | 0,013 |
| HPMC\_Visc | 1 | 2,383 | 8,34% | 2,340 | 2,3404 | 3,79 | 0,064 |
| Square | 1 | 2,452 | 8,59% | 2,452 | 2,4524 | 3,98 | 0,059 |
| Lac\*Lac | 1 | 2,452 | 8,59% | 2,452 | 2,4524 | 3,98 | 0,059 |
| 2-Way Interaction | 1 | 2,952 | 10,34% | 2,952 | 2,9524 | 4,79 | 0,040 |
| Lac\*HPMC\_Visc | 1 | 2,952 | 10,34% | 2,952 | 2,9524 | 4,79 | 0,040 |
| Error | 22 | 13,571 | 47,51% | 13,571 | 0,6169 |  |  |
| Lack-of-Fit | 20 | 11,144 | 39,01% | 11,144 | 0,5572 | 0,46 | 0,861 |
| Pure Error | 2 | 2,427 | 8,50% | 2,427 | 1,2135 |  |  |
| Total | 26 | 28,563 | 100,00% |  |  |  |  |

## Regression Equation in Uncoded Units

|  |  |  |
| --- | --- | --- |
| F\_SD\_0.33h(20min) | = | -3,42 + 4,0 Lac + 0,000592 HPMC\_Visc + 19,41 Lac\*Lac - 0,001464 Lac\*HPMC\_Visc |

## Fits and Diagnostics for All Observations

| Obs | F\_SD\_0.33h(20min) | Fit | SE Fit | 95% CI | Resid | Std Resid | Del Resid | HI |
| --- | --- | --- | --- | --- | --- | --- | --- | --- |
| 1 | 1,310 | 1,257 | 0,332 | (0,568; 1,946) | 0,053 | 0,07 | 0,07 | 0,178821 |
| 2 | 2,624 | 3,172 | 0,332 | (2,484; 3,861) | -0,549 | -0,77 | -0,76 | 0,178821 |
| 3 | 0,977 | 1,491 | 0,417 | (0,627; 2,355) | -0,514 | -0,77 | -0,76 | 0,281436 |
| 4 | 0,852 | 1,412 | 0,417 | (0,548; 2,276) | -0,560 | -0,84 | -0,84 | 0,281436 |
| 5 | 1,255 | 1,267 | 0,313 | (0,619; 1,916) | -0,012 | -0,02 | -0,02 | 0,158392 |
| 6 | 5,302 | 3,094 | 0,313 | (2,445; 3,742) | 2,209 | 3,07 | 3,96 | 0,158392 |
| 7 | 1,054 | 1,495 | 0,424 | (0,615; 2,375) | -0,440 | -0,67 | -0,66 | 0,291836 |
| 8 | 1,174 | 1,386 | 0,424 | (0,506; 2,265) | -0,212 | -0,32 | -0,31 | 0,291836 |
| 9 | 1,561 | 1,260 | 0,326 | (0,584; 1,936) | 0,301 | 0,42 | 0,41 | 0,172155 |
| 10 | 2,614 | 3,148 | 0,326 | (2,472; 3,824) | -0,534 | -0,75 | -0,74 | 0,172155 |
| 11 | 0,419 | 1,408 | 0,261 | (0,866; 1,949) | -0,988 | -1,33 | -1,36 | 0,110622 |
| 12 | 2,099 | 2,038 | 0,261 | (1,497; 2,580) | 0,060 | 0,08 | 0,08 | 0,110622 |
| 13 | 1,208 | 1,259 | 0,329 | (0,577; 1,940) | -0,051 | -0,07 | -0,07 | 0,175079 |
| 14 | 2,965 | 3,159 | 0,329 | (2,477; 3,840) | -0,194 | -0,27 | -0,27 | 0,175079 |
| 15 | 1,951 | 1,423 | 0,284 | (0,834; 2,012) | 0,528 | 0,72 | 0,71 | 0,130851 |
| 16 | 1,714 | 1,923 | 0,284 | (1,334; 2,513) | -0,210 | -0,29 | -0,28 | 0,130851 |
| 17 | 2,092 | 1,670 | 0,592 | (0,441; 2,898) | 0,422 | 0,82 | 0,81 | 0,568720 |
| 18 | 3,864 | 4,008 | 0,592 | (2,780; 5,237) | -0,145 | -0,28 | -0,27 | 0,568720 |
| 19 | 1,408 | 2,068 | 0,317 | (1,410; 2,727) | -0,660 | -0,92 | -0,92 | 0,163395 |
| 20 | 2,367 | 0,979 | 0,375 | (0,202; 1,756) | 1,388 | 2,01 | 2,17 | 0,227435 |
| 21 | 1,467 | 1,622 | 0,203 | (1,200; 2,044) | -0,155 | -0,20 | -0,20 | 0,067038 |
| 22 | 1,483 | 1,587 | 0,203 | (1,166; 2,007) | -0,103 | -0,14 | -0,13 | 0,066681 |
| 23 | 1,413 | 1,665 | 0,206 | (1,237; 2,093) | -0,252 | -0,33 | -0,33 | 0,068919 |
| 24 | 1,115 | 1,671 | 0,207 | (1,242; 2,099) | -0,555 | -0,73 | -0,73 | 0,069279 |
| 25 | 0,866 | 1,626 | 0,204 | (1,204; 2,048) | -0,760 | -1,00 | -1,00 | 0,067143 |
| 26 | 2,122 | 1,626 | 0,204 | (1,204; 2,048) | 0,496 | 0,65 | 0,65 | 0,067143 |
| 27 | 3,062 | 1,626 | 0,204 | (1,204; 2,048) | 1,436 | 1,89 | 2,02 | 0,067143 |

| Obs | Cook’s D | DFITS |  |  |
| --- | --- | --- | --- | --- |
| 1 | 0,00 | 0,03411 |  |  |
| 2 | 0,03 | -0,35645 |  |  |
| 3 | 0,05 | -0,47831 |  |  |
| 4 | 0,06 | -0,52278 |  |  |
| 5 | 0,00 | -0,00719 |  |  |
| 6 | 0,35 | 1,71660 | R |  |
| 7 | 0,04 | -0,42206 |  |  |
| 8 | 0,01 | -0,20130 |  |  |
| 9 | 0,01 | 0,18846 |  |  |
| 10 | 0,02 | -0,33702 |  |  |
| 11 | 0,04 | -0,47966 |  |  |
| 12 | 0,00 | 0,02806 |  |  |
| 13 | 0,00 | -0,03206 |  |  |
| 14 | 0,00 | -0,12267 |  |  |
| 15 | 0,02 | 0,27677 |  |  |
| 16 | 0,00 | -0,10872 |  |  |
| 17 | 0,18 | 0,93335 |  | X |
| 18 | 0,02 | -0,31498 |  | X |
| 19 | 0,03 | -0,40462 |  |  |
| 20 | 0,24 | 1,17945 | R |  |
| 21 | 0,00 | -0,05341 |  |  |
| 22 | 0,00 | -0,03563 |  |  |
| 23 | 0,00 | -0,08876 |  |  |
| 24 | 0,01 | -0,19783 |  |  |
| 25 | 0,01 | -0,26872 |  |  |
| 26 | 0,01 | 0,17308 |  |  |
| 27 | 0,05 | 0,54226 |  |  |

R  Large residual  
X  Unusual X

## Forward Selection of Terms

α to enter = 0,25

## Coded Coefficients

| Term | Coef | SE Coef | 95% CI | T-Value | P-Value | VIF |
| --- | --- | --- | --- | --- | --- | --- |
| Constant | 1,597 | 0,213 | (1,156; 2,038) | 7,51 | 0,000 |  |
| Lac | 0,893 | 0,344 | (0,180; 1,606) | 2,60 | 0,016 | 1,08 |
| HPMC\_Visc | -0,577 | 0,289 | (-1,176; 0,022) | -2,00 | 0,058 | 1,00 |
| Lac\*Lac | 1,303 | 0,629 | (-0,001; 2,607) | 2,07 | 0,050 | 1,00 |
| Lac\*HPMC\_Visc | -1,432 | 0,673 | (-2,826; -0,037) | -2,13 | 0,045 | 1,08 |

## Model Summary

| S | R-sq | R-sq(adj) | PRESS | R-sq(pred) | AICc | BIC |
| --- | --- | --- | --- | --- | --- | --- |
| 0,811745 | 51,82% | 43,07% | 20,7978 | 30,88% | 76,03 | 79,61 |

## Analysis of Variance

| Source | DF | Seq SS | Contribution | Adj SS | Adj MS | F-Value | P-Value |
| --- | --- | --- | --- | --- | --- | --- | --- |
| Model | 4 | 15,595 | 51,82% | 15,595 | 3,8987 | 5,92 | 0,002 |
| Linear | 2 | 9,781 | 32,50% | 7,078 | 3,5389 | 5,37 | 0,013 |
| Lac | 1 | 7,103 | 23,61% | 4,449 | 4,4494 | 6,75 | 0,016 |
| HPMC\_Visc | 1 | 2,677 | 8,90% | 2,628 | 2,6284 | 3,99 | 0,058 |
| Square | 1 | 2,828 | 9,40% | 2,828 | 2,8280 | 4,29 | 0,050 |
| Lac\*Lac | 1 | 2,828 | 9,40% | 2,828 | 2,8280 | 4,29 | 0,050 |
| 2-Way Interaction | 1 | 2,986 | 9,92% | 2,986 | 2,9858 | 4,53 | 0,045 |
| Lac\*HPMC\_Visc | 1 | 2,986 | 9,92% | 2,986 | 2,9858 | 4,53 | 0,045 |
| Error | 22 | 14,496 | 48,18% | 14,496 | 0,6589 |  |  |
| Lack-of-Fit | 20 | 12,160 | 40,41% | 12,160 | 0,6080 | 0,52 | 0,827 |
| Pure Error | 2 | 2,336 | 7,76% | 2,336 | 1,1681 |  |  |
| Total | 26 | 30,091 | 100,00% |  |  |  |  |

## Regression Equation in Uncoded Units

|  |  |  |
| --- | --- | --- |
| F\_SD\_0.5h(30min) | = | -2,92 + 2,6 Lac + 0,000588 HPMC\_Visc + 20,8 Lac\*Lac - 0,001472 Lac\*HPMC\_Visc |

## Fits and Diagnostics for All Observations

| Obs | F\_SD\_0.5h(30min) | Fit | SE Fit | 95% CI | Resid | Std Resid | Del Resid | HI |
| --- | --- | --- | --- | --- | --- | --- | --- | --- |
| 1 | 1,373 | 1,378 | 0,343 | (0,666; 2,089) | -0,005 | -0,01 | -0,01 | 0,178821 |
| 2 | 2,735 | 3,290 | 0,343 | (2,578; 4,002) | -0,555 | -0,75 | -0,75 | 0,178821 |
| 3 | 1,066 | 1,572 | 0,431 | (0,679; 2,465) | -0,506 | -0,74 | -0,73 | 0,281436 |
| 4 | 0,888 | 1,478 | 0,431 | (0,585; 2,371) | -0,590 | -0,86 | -0,85 | 0,281436 |
| 5 | 1,512 | 1,386 | 0,323 | (0,716; 2,056) | 0,126 | 0,17 | 0,16 | 0,158392 |
| 6 | 5,374 | 3,209 | 0,323 | (2,539; 3,879) | 2,165 | 2,91 | 3,62 | 0,158392 |
| 7 | 1,170 | 1,575 | 0,439 | (0,665; 2,484) | -0,404 | -0,59 | -0,58 | 0,291836 |
| 8 | 1,178 | 1,451 | 0,439 | (0,542; 2,361) | -0,273 | -0,40 | -0,39 | 0,291836 |
| 9 | 1,738 | 1,380 | 0,337 | (0,682; 2,079) | 0,357 | 0,48 | 0,48 | 0,172155 |
| 10 | 2,574 | 3,265 | 0,337 | (2,566; 3,963) | -0,690 | -0,93 | -0,93 | 0,172155 |
| 11 | 0,359 | 1,503 | 0,270 | (0,943; 2,063) | -1,144 | -1,49 | -1,54 | 0,110622 |
| 12 | 2,121 | 2,123 | 0,270 | (1,563; 2,683) | -0,002 | -0,00 | -0,00 | 0,110622 |
| 13 | 1,384 | 1,379 | 0,340 | (0,675; 2,083) | 0,005 | 0,01 | 0,01 | 0,175079 |
| 14 | 3,249 | 3,276 | 0,340 | (2,571; 3,980) | -0,027 | -0,04 | -0,04 | 0,175079 |
| 15 | 2,159 | 1,515 | 0,294 | (0,906; 2,124) | 0,644 | 0,85 | 0,85 | 0,130851 |
| 16 | 1,865 | 2,005 | 0,294 | (1,396; 2,614) | -0,140 | -0,18 | -0,18 | 0,130851 |
| 17 | 2,180 | 1,846 | 0,612 | (0,577; 3,116) | 0,334 | 0,63 | 0,62 | 0,568720 |
| 18 | 4,096 | 4,170 | 0,612 | (2,901; 5,440) | -0,074 | -0,14 | -0,14 | 0,568720 |
| 19 | 1,429 | 2,174 | 0,328 | (1,494; 2,855) | -0,746 | -1,00 | -1,00 | 0,163395 |
| 20 | 2,438 | 1,020 | 0,387 | (0,217; 1,823) | 1,418 | 1,99 | 2,14 | 0,227435 |
| 21 | 1,537 | 1,701 | 0,210 | (1,266; 2,137) | -0,165 | -0,21 | -0,21 | 0,067038 |
| 22 | 1,468 | 1,664 | 0,210 | (1,229; 2,099) | -0,196 | -0,25 | -0,24 | 0,066681 |
| 23 | 1,412 | 1,747 | 0,213 | (1,305; 2,189) | -0,336 | -0,43 | -0,42 | 0,068919 |
| 24 | 1,115 | 1,753 | 0,214 | (1,310; 2,196) | -0,638 | -0,81 | -0,81 | 0,069279 |
| 25 | 1,007 | 1,706 | 0,210 | (1,269; 2,142) | -0,699 | -0,89 | -0,89 | 0,067143 |
| 26 | 2,421 | 1,706 | 0,210 | (1,269; 2,142) | 0,716 | 0,91 | 0,91 | 0,067143 |
| 27 | 3,130 | 1,706 | 0,210 | (1,269; 2,142) | 1,424 | 1,82 | 1,93 | 0,067143 |

| Obs | Cook’s D | DFITS |  |  |
| --- | --- | --- | --- | --- |
| 1 | 0,00 | -0,00286 |  |  |
| 2 | 0,02 | -0,34850 |  |  |
| 3 | 0,04 | -0,45529 |  |  |
| 4 | 0,06 | -0,53359 |  |  |
| 5 | 0,00 | 0,07152 |  |  |
| 6 | 0,32 | 1,56987 | R |  |
| 7 | 0,03 | -0,37435 |  |  |
| 8 | 0,01 | -0,25147 |  |  |
| 9 | 0,01 | 0,21673 |  |  |
| 10 | 0,04 | -0,42487 |  |  |
| 11 | 0,06 | -0,54317 |  |  |
| 12 | 0,00 | -0,00111 |  |  |
| 13 | 0,00 | 0,00280 |  |  |
| 14 | 0,00 | -0,01643 |  |  |
| 15 | 0,02 | 0,32794 |  |  |
| 16 | 0,00 | -0,06994 |  |  |
| 17 | 0,10 | 0,70886 |  | X |
| 18 | 0,01 | -0,15523 |  | X |
| 19 | 0,04 | -0,44396 |  |  |
| 20 | 0,23 | 1,16313 |  |  |
| 21 | 0,00 | -0,05501 |  |  |
| 22 | 0,00 | -0,06526 |  |  |
| 23 | 0,00 | -0,11435 |  |  |
| 24 | 0,01 | -0,22039 |  |  |
| 25 | 0,01 | -0,23786 |  |  |
| 26 | 0,01 | 0,24389 |  |  |
| 27 | 0,05 | 0,51650 |  |  |

R  Large residual  
X  Unusual X

## Forward Selection of Terms

α to enter = 0,25

## Coded Coefficients

| Term | Coef | SE Coef | 95% CI | T-Value | P-Value | VIF |
| --- | --- | --- | --- | --- | --- | --- |
| Constant | 1,662 | 0,228 | (1,190; 2,134) | 7,30 | 0,000 |  |
| Lac | 0,951 | 0,368 | (0,188; 1,715) | 2,58 | 0,017 | 1,08 |
| HPMC\_Visc | -0,562 | 0,309 | (-1,204; 0,079) | -1,82 | 0,083 | 1,00 |
| Lac\*Lac | 1,448 | 0,673 | (0,051; 2,844) | 2,15 | 0,043 | 1,00 |
| Lac\*HPMC\_Visc | -1,475 | 0,720 | (-2,968; 0,018) | -2,05 | 0,053 | 1,08 |

## Model Summary

| S | R-sq | R-sq(adj) | PRESS | R-sq(pred) | AICc | BIC |
| --- | --- | --- | --- | --- | --- | --- |
| 0,869142 | 50,82% | 41,88% | 23,4582 | 30,58% | 79,72 | 83,29 |

## Analysis of Variance

| Source | DF | Seq SS | Contribution | Adj SS | Adj MS | F-Value | P-Value |
| --- | --- | --- | --- | --- | --- | --- | --- |
| Model | 4 | 17,173 | 50,82% | 17,173 | 4,2931 | 5,68 | 0,003 |
| Linear | 2 | 10,510 | 31,10% | 7,540 | 3,7701 | 4,99 | 0,016 |
| Lac | 1 | 7,962 | 23,56% | 5,046 | 5,0457 | 6,68 | 0,017 |
| HPMC\_Visc | 1 | 2,548 | 7,54% | 2,495 | 2,4945 | 3,30 | 0,083 |
| Square | 1 | 3,492 | 10,33% | 3,492 | 3,4922 | 4,62 | 0,043 |
| Lac\*Lac | 1 | 3,492 | 10,33% | 3,492 | 3,4922 | 4,62 | 0,043 |
| 2-Way Interaction | 1 | 3,170 | 9,38% | 3,170 | 3,1704 | 4,20 | 0,053 |
| Lac\*HPMC\_Visc | 1 | 3,170 | 9,38% | 3,170 | 3,1704 | 4,20 | 0,053 |
| Error | 22 | 16,619 | 49,18% | 16,619 | 0,7554 |  |  |
| Lack-of-Fit | 20 | 13,759 | 40,72% | 13,759 | 0,6880 | 0,48 | 0,849 |
| Pure Error | 2 | 2,860 | 8,46% | 2,860 | 1,4299 |  |  |
| Total | 26 | 33,791 | 100,00% |  |  |  |  |

## Regression Equation in Uncoded Units

|  |  |  |
| --- | --- | --- |
| F\_SD\_0.75h(45min) | = | -2,74 + 1,1 Lac + 0,000614 HPMC\_Visc + 23,2 Lac\*Lac - 0,001517 Lac\*HPMC\_Visc |

## Fits and Diagnostics for All Observations

| Obs | F\_SD\_0.75h(45min) | Fit | SE Fit | 95% CI | Resid | Std Resid | Del Resid | HI |
| --- | --- | --- | --- | --- | --- | --- | --- | --- |
| 1 | 1,455 | 1,423 | 0,368 | (0,661; 2,186) | 0,032 | 0,04 | 0,04 | 0,178821 |
| 2 | 2,942 | 3,425 | 0,368 | (2,663; 4,187) | -0,483 | -0,61 | -0,60 | 0,178821 |
| 3 | 1,206 | 1,669 | 0,461 | (0,713; 2,625) | -0,463 | -0,63 | -0,62 | 0,281436 |
| 4 | 0,920 | 1,603 | 0,461 | (0,647; 2,560) | -0,683 | -0,93 | -0,92 | 0,281436 |
| 5 | 1,752 | 1,434 | 0,346 | (0,717; 2,152) | 0,318 | 0,40 | 0,39 | 0,158392 |
| 6 | 5,464 | 3,344 | 0,346 | (2,626; 4,061) | 2,120 | 2,66 | 3,15 | 0,158392 |
| 7 | 1,248 | 1,673 | 0,470 | (0,699; 2,647) | -0,424 | -0,58 | -0,57 | 0,291836 |
| 8 | 1,255 | 1,576 | 0,470 | (0,603; 2,550) | -0,321 | -0,44 | -0,43 | 0,291836 |
| 9 | 1,854 | 1,427 | 0,361 | (0,679; 2,175) | 0,427 | 0,54 | 0,53 | 0,172155 |
| 10 | 2,459 | 3,399 | 0,361 | (2,652; 4,147) | -0,941 | -1,19 | -1,20 | 0,172155 |
| 11 | 0,287 | 1,582 | 0,289 | (0,982; 2,181) | -1,295 | -1,58 | -1,64 | 0,110622 |
| 12 | 2,231 | 2,252 | 0,289 | (1,652; 2,851) | -0,021 | -0,03 | -0,02 | 0,110622 |
| 13 | 1,318 | 1,425 | 0,364 | (0,671; 2,180) | -0,108 | -0,14 | -0,13 | 0,175079 |
| 14 | 3,625 | 3,411 | 0,364 | (2,657; 4,165) | 0,214 | 0,27 | 0,27 | 0,175079 |
| 15 | 2,361 | 1,598 | 0,314 | (0,946; 2,250) | 0,763 | 0,94 | 0,94 | 0,130851 |
| 16 | 2,163 | 2,133 | 0,314 | (1,481; 2,785) | 0,030 | 0,04 | 0,04 | 0,130851 |
| 17 | 2,258 | 1,987 | 0,655 | (0,628; 3,346) | 0,271 | 0,47 | 0,47 | 0,568720 |
| 18 | 4,381 | 4,443 | 0,655 | (3,084; 5,803) | -0,062 | -0,11 | -0,11 | 0,568720 |
| 19 | 1,390 | 2,224 | 0,351 | (1,496; 2,953) | -0,834 | -1,05 | -1,05 | 0,163395 |
| 20 | 2,566 | 1,100 | 0,414 | (0,240; 1,959) | 1,466 | 1,92 | 2,05 | 0,227435 |
| 21 | 1,553 | 1,764 | 0,225 | (1,297; 2,230) | -0,210 | -0,25 | -0,25 | 0,067038 |
| 22 | 1,446 | 1,727 | 0,224 | (1,262; 2,193) | -0,281 | -0,33 | -0,33 | 0,066681 |
| 23 | 1,379 | 1,808 | 0,228 | (1,335; 2,281) | -0,429 | -0,51 | -0,50 | 0,068919 |
| 24 | 1,118 | 1,814 | 0,229 | (1,339; 2,288) | -0,696 | -0,83 | -0,82 | 0,069279 |
| 25 | 0,949 | 1,768 | 0,225 | (1,301; 2,235) | -0,819 | -0,98 | -0,97 | 0,067143 |
| 26 | 2,757 | 1,768 | 0,225 | (1,301; 2,235) | 0,989 | 1,18 | 1,19 | 0,067143 |
| 27 | 3,208 | 1,768 | 0,225 | (1,301; 2,235) | 1,441 | 1,72 | 1,80 | 0,067143 |

| Obs | Cook’s D | DFITS |  |  |
| --- | --- | --- | --- | --- |
| 1 | 0,00 | 0,01840 |  |  |
| 2 | 0,02 | -0,28202 |  |  |
| 3 | 0,03 | -0,38784 |  |  |
| 4 | 0,07 | -0,57838 |  |  |
| 5 | 0,01 | 0,16965 |  |  |
| 6 | 0,27 | 1,36821 | R |  |
| 7 | 0,03 | -0,36673 |  |  |
| 8 | 0,02 | -0,27679 |  |  |
| 9 | 0,01 | 0,24233 |  |  |
| 10 | 0,06 | -0,54786 |  |  |
| 11 | 0,06 | -0,57813 |  |  |
| 12 | 0,00 | -0,00877 |  |  |
| 13 | 0,00 | -0,06154 |  |  |
| 14 | 0,00 | 0,12244 |  |  |
| 15 | 0,03 | 0,36443 |  |  |
| 16 | 0,00 | 0,01421 |  |  |
| 17 | 0,06 | 0,53504 |  | X |
| 18 | 0,00 | -0,12243 |  | X |
| 19 | 0,04 | -0,46487 |  |  |
| 20 | 0,22 | 1,11455 |  |  |
| 21 | 0,00 | -0,06569 |  |  |
| 22 | 0,00 | -0,08768 |  |  |
| 23 | 0,00 | -0,13676 |  |  |
| 24 | 0,01 | -0,22490 |  |  |
| 25 | 0,01 | -0,26146 |  |  |
| 26 | 0,02 | 0,31916 |  |  |
| 27 | 0,04 | 0,48330 |  |  |

R  Large residual  
X  Unusual X

## Forward Selection of Terms

α to enter = 0,25

## Coded Coefficients

| Term | Coef | SE Coef | 95% CI | T-Value | P-Value | VIF |
| --- | --- | --- | --- | --- | --- | --- |
| Constant | 1,705 | 0,242 | (1,204; 2,207) | 7,05 | 0,000 |  |
| Lac | 1,003 | 0,391 | (0,193; 1,814) | 2,57 | 0,018 | 1,08 |
| HPMC\_Visc | -0,531 | 0,329 | (-1,212; 0,151) | -1,61 | 0,121 | 1,00 |
| Lac\*Lac | 1,655 | 0,715 | (0,172; 3,138) | 2,31 | 0,030 | 1,00 |
| Lac\*HPMC\_Visc | -1,448 | 0,765 | (-3,034; 0,138) | -1,89 | 0,072 | 1,08 |

## Model Summary

| S | R-sq | R-sq(adj) | PRESS | R-sq(pred) | AICc | BIC |
| --- | --- | --- | --- | --- | --- | --- |
| 0,923148 | 49,72% | 40,58% | 26,3725 | 29,27% | 82,98 | 86,55 |

## Analysis of Variance

| Source | DF | Seq SS | Contribution | Adj SS | Adj MS | F-Value | P-Value |
| --- | --- | --- | --- | --- | --- | --- | --- |
| Model | 4 | 18,540 | 49,72% | 18,540 | 4,6349 | 5,44 | 0,003 |
| Linear | 2 | 10,922 | 29,29% | 7,834 | 3,9169 | 4,60 | 0,021 |
| Lac | 1 | 8,644 | 23,18% | 5,612 | 5,6125 | 6,59 | 0,018 |
| HPMC\_Visc | 1 | 2,279 | 6,11% | 2,221 | 2,2213 | 2,61 | 0,121 |
| Square | 1 | 4,564 | 12,24% | 4,564 | 4,5637 | 5,36 | 0,030 |
| Lac\*Lac | 1 | 4,564 | 12,24% | 4,564 | 4,5637 | 5,36 | 0,030 |
| 2-Way Interaction | 1 | 3,054 | 8,19% | 3,054 | 3,0537 | 3,58 | 0,072 |
| Lac\*HPMC\_Visc | 1 | 3,054 | 8,19% | 3,054 | 3,0537 | 3,58 | 0,072 |
| Error | 22 | 18,748 | 50,28% | 18,748 | 0,8522 |  |  |
| Lack-of-Fit | 20 | 15,339 | 41,14% | 15,339 | 0,7670 | 0,45 | 0,866 |
| Pure Error | 2 | 3,409 | 9,14% | 3,409 | 1,7046 |  |  |
| Total | 26 | 37,288 | 100,00% |  |  |  |  |

## Regression Equation in Uncoded Units

|  |  |  |
| --- | --- | --- |
| F\_SD\_1h(60min) | = | -1,90 - 2,4 Lac + 0,000608 HPMC\_Visc + 26,5 Lac\*Lac - 0,001489 Lac\*HPMC\_Visc |

## Fits and Diagnostics for All Observations

| Obs | F\_SD\_1h(60min) | Fit | SE Fit | 95% CI | Resid | Std Resid | Del Resid | HI |
| --- | --- | --- | --- | --- | --- | --- | --- | --- |
| 1 | 1,575 | 1,480 | 0,390 | (0,670; 2,289) | 0,095 | 0,11 | 0,11 | 0,178821 |
| 2 | 3,009 | 3,514 | 0,390 | (2,704; 4,323) | -0,505 | -0,60 | -0,59 | 0,178821 |
| 3 | 1,284 | 1,751 | 0,490 | (0,735; 2,766) | -0,467 | -0,60 | -0,59 | 0,281436 |
| 4 | 0,992 | 1,756 | 0,490 | (0,740; 2,772) | -0,764 | -0,98 | -0,98 | 0,281436 |
| 5 | 1,922 | 1,492 | 0,367 | (0,730; 2,254) | 0,430 | 0,51 | 0,50 | 0,158392 |
| 6 | 5,554 | 3,435 | 0,367 | (2,673; 4,197) | 2,119 | 2,50 | 2,89 | 0,158392 |
| 7 | 1,297 | 1,755 | 0,499 | (0,720; 2,789) | -0,458 | -0,59 | -0,58 | 0,291836 |
| 8 | 1,379 | 1,730 | 0,499 | (0,695; 2,764) | -0,351 | -0,45 | -0,44 | 0,291836 |
| 9 | 1,905 | 1,484 | 0,383 | (0,689; 2,278) | 0,422 | 0,50 | 0,49 | 0,172155 |
| 10 | 2,436 | 3,489 | 0,383 | (2,695; 4,283) | -1,053 | -1,25 | -1,27 | 0,172155 |
| 11 | 0,289 | 1,654 | 0,307 | (1,018; 2,291) | -1,365 | -1,57 | -1,63 | 0,110622 |
| 12 | 2,220 | 2,381 | 0,307 | (1,745; 3,018) | -0,162 | -0,19 | -0,18 | 0,110622 |
| 13 | 1,323 | 1,482 | 0,386 | (0,681; 2,283) | -0,159 | -0,19 | -0,18 | 0,175079 |
| 14 | 3,913 | 3,500 | 0,386 | (2,699; 4,301) | 0,413 | 0,49 | 0,48 | 0,175079 |
| 15 | 2,468 | 1,672 | 0,334 | (0,979; 2,364) | 0,796 | 0,92 | 0,92 | 0,130851 |
| 16 | 2,474 | 2,267 | 0,334 | (1,574; 2,959) | 0,207 | 0,24 | 0,24 | 0,130851 |
| 17 | 2,438 | 2,185 | 0,696 | (0,741; 3,629) | 0,253 | 0,42 | 0,41 | 0,568720 |
| 18 | 4,683 | 4,735 | 0,696 | (3,291; 6,179) | -0,052 | -0,09 | -0,08 | 0,568720 |
| 19 | 1,281 | 2,236 | 0,373 | (1,462; 3,010) | -0,955 | -1,13 | -1,14 | 0,163395 |
| 20 | 2,722 | 1,175 | 0,440 | (0,262; 2,088) | 1,547 | 1,91 | 2,04 | 0,227435 |
| 21 | 1,632 | 1,801 | 0,239 | (1,305; 2,297) | -0,170 | -0,19 | -0,19 | 0,067038 |
| 22 | 1,475 | 1,767 | 0,238 | (1,272; 2,261) | -0,292 | -0,33 | -0,32 | 0,066681 |
| 23 | 1,360 | 1,843 | 0,242 | (1,341; 2,346) | -0,483 | -0,54 | -0,53 | 0,068919 |
| 24 | 1,112 | 1,849 | 0,243 | (1,345; 2,352) | -0,737 | -0,83 | -0,82 | 0,069279 |
| 25 | 0,864 | 1,805 | 0,239 | (1,309; 2,301) | -0,941 | -1,06 | -1,06 | 0,067143 |
| 26 | 3,042 | 1,805 | 0,239 | (1,309; 2,301) | 1,237 | 1,39 | 1,42 | 0,067143 |
| 27 | 3,200 | 1,805 | 0,239 | (1,309; 2,301) | 1,395 | 1,56 | 1,62 | 0,067143 |

| Obs | Cook’s D | DFITS |  |  |
| --- | --- | --- | --- | --- |
| 1 | 0,00 | 0,05180 |  |  |
| 2 | 0,02 | -0,27749 |  |  |
| 3 | 0,03 | -0,36802 |  |  |
| 4 | 0,07 | -0,61053 |  |  |
| 5 | 0,01 | 0,21632 |  |  |
| 6 | 0,24 | 1,25381 | R |  |
| 7 | 0,03 | -0,37287 |  |  |
| 8 | 0,02 | -0,28472 |  |  |
| 9 | 0,01 | 0,22491 |  |  |
| 10 | 0,07 | -0,57983 |  |  |
| 11 | 0,06 | -0,57337 |  |  |
| 12 | 0,00 | -0,06403 |  |  |
| 13 | 0,00 | -0,08516 |  |  |
| 14 | 0,01 | 0,22281 |  |  |
| 15 | 0,03 | 0,35767 |  |  |
| 16 | 0,00 | 0,09131 |  |  |
| 17 | 0,05 | 0,47010 |  | X |
| 18 | 0,00 | -0,09665 |  | X |
| 19 | 0,05 | -0,50311 |  |  |
| 20 | 0,21 | 1,10656 |  |  |
| 21 | 0,00 | -0,04984 |  |  |
| 22 | 0,00 | -0,08571 |  |  |
| 23 | 0,00 | -0,14502 |  |  |
| 24 | 0,01 | -0,22398 |  |  |
| 25 | 0,02 | -0,28399 |  |  |
| 26 | 0,03 | 0,38066 |  |  |
| 27 | 0,04 | 0,43502 |  |  |

R  Large residual  
X  Unusual X

## Forward Selection of Terms

α to enter = 0,25

## Coded Coefficients

| Term | Coef | SE Coef | 95% CI | T-Value | P-Value | VIF |
| --- | --- | --- | --- | --- | --- | --- |
| Constant | 1,775 | 0,270 | (1,215; 2,335) | 6,58 | 0,000 |  |
| Lac | 1,081 | 0,436 | (0,176; 1,986) | 2,48 | 0,021 | 1,08 |
| HPMC\_Visc | -0,490 | 0,367 | (-1,250; 0,271) | -1,34 | 0,195 | 1,00 |
| Lac\*Lac | 1,930 | 0,798 | (0,275; 3,585) | 2,42 | 0,024 | 1,00 |
| Lac\*HPMC\_Visc | -1,452 | 0,853 | (-3,222; 0,318) | -1,70 | 0,103 | 1,08 |

## Model Summary

| S | R-sq | R-sq(adj) | PRESS | R-sq(pred) | AICc | BIC |
| --- | --- | --- | --- | --- | --- | --- |
| 1,03014 | 47,40% | 37,84% | 32,6002 | 26,55% | 88,90 | 92,47 |

## Analysis of Variance

| Source | DF | Seq SS | Contribution | Adj SS | Adj MS | F-Value | P-Value |
| --- | --- | --- | --- | --- | --- | --- | --- |
| Model | 4 | 21,039 | 47,40% | 21,039 | 5,2598 | 4,96 | 0,005 |
| Linear | 2 | 11,760 | 26,50% | 8,406 | 4,2028 | 3,96 | 0,034 |
| Lac | 1 | 9,806 | 22,09% | 6,513 | 6,5131 | 6,14 | 0,021 |
| HPMC\_Visc | 1 | 1,954 | 4,40% | 1,893 | 1,8926 | 1,78 | 0,195 |
| Square | 1 | 6,206 | 13,98% | 6,206 | 6,2064 | 5,85 | 0,024 |
| Lac\*Lac | 1 | 6,206 | 13,98% | 6,206 | 6,2064 | 5,85 | 0,024 |
| 2-Way Interaction | 1 | 3,073 | 6,92% | 3,073 | 3,0729 | 2,90 | 0,103 |
| Lac\*HPMC\_Visc | 1 | 3,073 | 6,92% | 3,073 | 3,0729 | 2,90 | 0,103 |
| Error | 22 | 23,346 | 52,60% | 23,346 | 1,0612 |  |  |
| Lack-of-Fit | 20 | 18,122 | 40,83% | 18,122 | 0,9061 | 0,35 | 0,921 |
| Pure Error | 2 | 5,224 | 11,77% | 5,224 | 2,6122 |  |  |
| Total | 26 | 44,386 | 100,00% |  |  |  |  |

## Regression Equation in Uncoded Units

|  |  |  |
| --- | --- | --- |
| F\_SD\_1.5h(90min) | = | -1,05 - 6,4 Lac + 0,000621 HPMC\_Visc + 30,9 Lac\*Lac - 0,001493 Lac\*HPMC\_Visc |

## Fits and Diagnostics for All Observations

| Obs | F\_SD\_1.5h(90min) | Fit | SE Fit | 95% CI | Resid | Std Resid | Del Resid | HI |
| --- | --- | --- | --- | --- | --- | --- | --- | --- |
| 1 | 1,783 | 1,549 | 0,436 | (0,646; 2,452) | 0,234 | 0,25 | 0,24 | 0,178821 |
| 2 | 3,215 | 3,664 | 0,436 | (2,760; 4,567) | -0,449 | -0,48 | -0,47 | 0,178821 |
| 3 | 1,392 | 1,880 | 0,546 | (0,747; 3,014) | -0,488 | -0,56 | -0,55 | 0,281436 |
| 4 | 1,054 | 1,960 | 0,546 | (0,827; 3,093) | -0,906 | -1,04 | -1,04 | 0,281436 |
| 5 | 2,184 | 1,564 | 0,410 | (0,714; 2,414) | 0,620 | 0,66 | 0,65 | 0,158392 |
| 6 | 5,687 | 3,588 | 0,410 | (2,737; 4,438) | 2,099 | 2,22 | 2,46 | 0,158392 |
| 7 | 1,391 | 1,885 | 0,557 | (0,731; 3,039) | -0,495 | -0,57 | -0,56 | 0,291836 |
| 8 | 1,603 | 1,935 | 0,557 | (0,780; 3,089) | -0,331 | -0,38 | -0,37 | 0,291836 |
| 9 | 1,901 | 1,554 | 0,427 | (0,667; 2,440) | 0,347 | 0,37 | 0,36 | 0,172155 |
| 10 | 2,432 | 3,640 | 0,427 | (2,754; 4,526) | -1,208 | -1,29 | -1,31 | 0,172155 |
| 11 | 0,302 | 1,762 | 0,343 | (1,052; 2,473) | -1,460 | -1,50 | -1,55 | 0,110622 |
| 12 | 2,133 | 2,566 | 0,343 | (1,856; 3,277) | -0,434 | -0,45 | -0,44 | 0,110622 |
| 13 | 1,297 | 1,552 | 0,431 | (0,658; 2,446) | -0,255 | -0,27 | -0,27 | 0,175079 |
| 14 | 4,384 | 3,651 | 0,431 | (2,757; 4,544) | 0,734 | 0,78 | 0,78 | 0,175079 |
| 15 | 2,605 | 1,784 | 0,373 | (1,011; 2,557) | 0,820 | 0,85 | 0,85 | 0,130851 |
| 16 | 3,017 | 2,455 | 0,373 | (1,682; 3,228) | 0,562 | 0,59 | 0,58 | 0,130851 |
| 17 | 2,706 | 2,444 | 0,777 | (0,833; 4,055) | 0,262 | 0,39 | 0,38 | 0,568720 |
| 18 | 5,041 | 5,150 | 0,777 | (3,539; 6,762) | -0,110 | -0,16 | -0,16 | 0,568720 |
| 19 | 1,124 | 2,265 | 0,416 | (1,402; 3,129) | -1,141 | -1,21 | -1,22 | 0,163395 |
| 20 | 2,952 | 1,286 | 0,491 | (0,267; 2,304) | 1,667 | 1,84 | 1,96 | 0,227435 |
| 21 | 1,733 | 1,864 | 0,267 | (1,311; 2,417) | -0,131 | -0,13 | -0,13 | 0,067038 |
| 22 | 1,503 | 1,832 | 0,266 | (1,280; 2,384) | -0,329 | -0,33 | -0,32 | 0,066681 |
| 23 | 1,348 | 1,903 | 0,270 | (1,342; 2,463) | -0,554 | -0,56 | -0,55 | 0,068919 |
| 24 | 1,137 | 1,908 | 0,271 | (1,345; 2,470) | -0,770 | -0,77 | -0,77 | 0,069279 |
| 25 | 0,582 | 1,867 | 0,267 | (1,314; 2,421) | -1,285 | -1,29 | -1,31 | 0,067143 |
| 26 | 3,530 | 1,867 | 0,267 | (1,314; 2,421) | 1,663 | 1,67 | 1,75 | 0,067143 |
| 27 | 3,205 | 1,867 | 0,267 | (1,314; 2,421) | 1,338 | 1,34 | 1,37 | 0,067143 |

| Obs | Cook’s D | DFITS |  |  |
| --- | --- | --- | --- | --- |
| 1 | 0,00 | 0,11427 |  |  |
| 2 | 0,01 | -0,22043 |  |  |
| 3 | 0,02 | -0,34413 |  |  |
| 4 | 0,08 | -0,65022 |  |  |
| 5 | 0,02 | 0,28101 |  |  |
| 6 | 0,19 | 1,06913 | R |  |
| 7 | 0,03 | -0,36057 |  |  |
| 8 | 0,01 | -0,24045 |  |  |
| 9 | 0,01 | 0,16536 |  |  |
| 10 | 0,07 | -0,59738 |  |  |
| 11 | 0,06 | -0,54679 |  |  |
| 12 | 0,00 | -0,15447 |  |  |
| 13 | 0,00 | -0,12279 |  |  |
| 14 | 0,03 | 0,35794 |  |  |
| 15 | 0,02 | 0,32934 |  |  |
| 16 | 0,01 | 0,22364 |  |  |
| 17 | 0,04 | 0,43626 |  | X |
| 18 | 0,01 | -0,18236 |  | X |
| 19 | 0,06 | -0,54120 |  |  |
| 20 | 0,20 | 1,06077 |  |  |
| 21 | 0,00 | -0,03443 |  |  |
| 22 | 0,00 | -0,08666 |  |  |
| 23 | 0,00 | -0,14926 |  |  |
| 24 | 0,01 | -0,20945 |  |  |
| 25 | 0,02 | -0,35210 |  |  |
| 26 | 0,04 | 0,46886 |  |  |
| 27 | 0,03 | 0,36787 |  |  |

R  Large residual  
X  Unusual X

## Forward Selection of Terms

α to enter = 0,25

## Coded Coefficients

| Term | Coef | SE Coef | 95% CI | T-Value | P-Value | VIF |
| --- | --- | --- | --- | --- | --- | --- |
| Constant | 1,907 | 0,298 | (1,291; 2,522) | 6,40 | 0,000 |  |
| Lac | 1,322 | 0,471 | (0,349; 2,295) | 2,80 | 0,010 | 1,00 |
| Lac\*Lac | 2,102 | 0,894 | (0,256; 3,948) | 2,35 | 0,027 | 1,00 |

## Model Summary

| S | R-sq | R-sq(adj) | PRESS | R-sq(pred) | AICc | BIC |
| --- | --- | --- | --- | --- | --- | --- |
| 1,15468 | 35,81% | 30,46% | 37,4922 | 24,79% | 91,03 | 94,39 |

## Analysis of Variance

| Source | DF | Seq SS | Contribution | Adj SS | Adj MS | F-Value | P-Value |
| --- | --- | --- | --- | --- | --- | --- | --- |
| Model | 2 | 17,849 | 35,81% | 17,849 | 8,925 | 6,69 | 0,005 |
| Linear | 1 | 10,486 | 21,04% | 10,486 | 10,486 | 7,86 | 0,010 |
| Lac | 1 | 10,486 | 21,04% | 10,486 | 10,486 | 7,86 | 0,010 |
| Square | 1 | 7,364 | 14,77% | 7,364 | 7,364 | 5,52 | 0,027 |
| Lac\*Lac | 1 | 7,364 | 14,77% | 7,364 | 7,364 | 5,52 | 0,027 |
| Error | 24 | 31,999 | 64,19% | 31,999 | 1,333 |  |  |
| Lack-of-Fit | 22 | 24,800 | 49,75% | 24,800 | 1,127 | 0,31 | 0,939 |
| Pure Error | 2 | 7,199 | 14,44% | 7,199 | 3,600 |  |  |
| Total | 26 | 49,848 | 100,00% |  |  |  |  |

## Regression Equation in Uncoded Units

|  |  |  |
| --- | --- | --- |
| F\_SD\_2h(120min) | = | 7,67 - 28,3 Lac + 33,6 Lac\*Lac |

## Fits and Diagnostics for All Observations

| Obs | F\_SD\_2h(120min) | Fit | SE Fit | 95% CI | Resid | Std Resid | Del Resid | HI |
| --- | --- | --- | --- | --- | --- | --- | --- | --- |
| 1 | 2,004 | 1,771 | 0,325 | (1,101; 2,442) | 0,233 | 0,21 | 0,21 | 0,079167 |
| 2 | 3,271 | 3,093 | 0,325 | (2,423; 3,764) | 0,178 | 0,16 | 0,16 | 0,079167 |
| 3 | 1,456 | 1,771 | 0,325 | (1,101; 2,442) | -0,315 | -0,28 | -0,28 | 0,079167 |
| 4 | 1,045 | 3,093 | 0,325 | (2,423; 3,764) | -2,048 | -1,85 | -1,95 | 0,079167 |
| 5 | 2,398 | 1,771 | 0,325 | (1,101; 2,442) | 0,626 | 0,57 | 0,56 | 0,079167 |
| 6 | 5,712 | 3,093 | 0,325 | (2,423; 3,764) | 2,618 | 2,36 | 2,64 | 0,079167 |
| 7 | 1,491 | 1,771 | 0,325 | (1,101; 2,442) | -0,281 | -0,25 | -0,25 | 0,079167 |
| 8 | 1,746 | 3,093 | 0,325 | (2,423; 3,764) | -1,348 | -1,22 | -1,23 | 0,079167 |
| 9 | 1,819 | 1,771 | 0,325 | (1,101; 2,442) | 0,048 | 0,04 | 0,04 | 0,079167 |
| 10 | 2,487 | 3,093 | 0,325 | (2,423; 3,764) | -0,606 | -0,55 | -0,54 | 0,079167 |
| 11 | 0,460 | 1,771 | 0,325 | (1,101; 2,442) | -1,311 | -1,18 | -1,19 | 0,079167 |
| 12 | 2,050 | 3,093 | 0,325 | (2,423; 3,764) | -1,044 | -0,94 | -0,94 | 0,079167 |
| 13 | 1,302 | 1,771 | 0,325 | (1,101; 2,442) | -0,470 | -0,42 | -0,42 | 0,079167 |
| 14 | 4,644 | 3,093 | 0,325 | (2,423; 3,764) | 1,551 | 1,40 | 1,43 | 0,079167 |
| 15 | 2,712 | 1,771 | 0,325 | (1,101; 2,442) | 0,940 | 0,85 | 0,84 | 0,079167 |
| 16 | 3,524 | 3,093 | 0,325 | (2,423; 3,764) | 0,431 | 0,39 | 0,38 | 0,079167 |
| 17 | 2,852 | 2,687 | 0,869 | (0,893; 4,481) | 0,165 | 0,22 | 0,21 | 0,566667 |
| 18 | 5,365 | 5,331 | 0,869 | (3,537; 7,125) | 0,034 | 0,04 | 0,04 | 0,566667 |
| 19 | 1,098 | 1,907 | 0,298 | (1,291; 2,522) | -0,808 | -0,72 | -0,72 | 0,066667 |
| 20 | 3,212 | 1,907 | 0,298 | (1,291; 2,522) | 1,305 | 1,17 | 1,18 | 0,066667 |
| 21 | 1,875 | 1,907 | 0,298 | (1,291; 2,522) | -0,032 | -0,03 | -0,03 | 0,066667 |
| 22 | 1,577 | 1,907 | 0,298 | (1,291; 2,522) | -0,330 | -0,30 | -0,29 | 0,066667 |
| 23 | 1,359 | 1,907 | 0,298 | (1,291; 2,522) | -0,547 | -0,49 | -0,48 | 0,066667 |
| 24 | 1,152 | 1,907 | 0,298 | (1,291; 2,522) | -0,755 | -0,68 | -0,67 | 0,066667 |
| 25 | 0,358 | 1,907 | 0,298 | (1,291; 2,522) | -1,548 | -1,39 | -1,42 | 0,066667 |
| 26 | 3,982 | 1,907 | 0,298 | (1,291; 2,522) | 2,075 | 1,86 | 1,97 | 0,066667 |
| 27 | 3,145 | 1,907 | 0,298 | (1,291; 2,522) | 1,238 | 1,11 | 1,12 | 0,066667 |

| Obs | Cook’s D | DFITS |  |  |
| --- | --- | --- | --- | --- |
| 1 | 0,00 | 0,060377 |  |  |
| 2 | 0,00 | 0,046168 |  |  |
| 3 | 0,00 | -0,081862 |  |  |
| 4 | 0,10 | -0,572855 |  |  |
| 5 | 0,01 | 0,163352 |  |  |
| 6 | 0,16 | 0,774363 | R |  |
| 7 | 0,00 | -0,072802 |  |  |
| 8 | 0,04 | -0,360402 |  |  |
| 9 | 0,00 | 0,012408 |  |  |
| 10 | 0,01 | -0,158054 |  |  |
| 11 | 0,04 | -0,350011 |  |  |
| 12 | 0,03 | -0,275493 |  |  |
| 13 | 0,01 | -0,122098 |  |  |
| 14 | 0,06 | 0,419246 |  |  |
| 15 | 0,02 | 0,247323 |  |  |
| 16 | 0,00 | 0,111969 |  |  |
| 17 | 0,02 | 0,243434 |  | X |
| 18 | 0,00 | 0,050107 |  | X |
| 19 | 0,01 | -0,191686 |  |  |
| 20 | 0,03 | 0,315185 |  |  |
| 21 | 0,00 | -0,007467 |  |  |
| 22 | 0,00 | -0,077540 |  |  |
| 23 | 0,01 | -0,129006 |  |  |
| 24 | 0,01 | -0,178836 |  |  |
| 25 | 0,05 | -0,378686 |  |  |
| 26 | 0,08 | 0,526130 |  |  |
| 27 | 0,03 | 0,298180 |  |  |

R  Large residual  
X  Unusual X

## Forward Selection of Terms

α to enter = 0,25

## Coded Coefficients

| Term | Coef | SE Coef | 95% CI | T-Value | P-Value | VIF |
| --- | --- | --- | --- | --- | --- | --- |
| Constant | 2,038 | 0,311 | (1,393; 2,684) | 6,55 | 0,000 |  |
| Lac | 1,517 | 0,507 | (0,467; 2,568) | 3,00 | 0,007 | 1,10 |
| HPMC\_HP | 0,573 | 0,478 | (-0,419; 1,565) | 1,20 | 0,244 | 1,00 |
| Lac\*Lac | 2,227 | 0,915 | (0,328; 4,125) | 2,43 | 0,024 | 1,00 |
| Lac\*HPMC\_HP | 1,40 | 1,17 | (-1,03; 3,83) | 1,20 | 0,244 | 1,10 |

## Model Summary

| S | R-sq | R-sq(adj) | PRESS | R-sq(pred) | AICc | BIC |
| --- | --- | --- | --- | --- | --- | --- |
| 1,18179 | 42,69% | 32,27% | 41,8248 | 21,98% | 96,31 | 99,89 |

## Analysis of Variance

| Source | DF | Seq SS | Contribution | Adj SS | Adj MS | F-Value | P-Value |
| --- | --- | --- | --- | --- | --- | --- | --- |
| Model | 4 | 22,885 | 42,69% | 22,885 | 5,721 | 4,10 | 0,012 |
| Linear | 2 | 12,618 | 23,54% | 14,534 | 7,267 | 5,20 | 0,014 |
| Lac | 1 | 10,650 | 19,87% | 12,529 | 12,529 | 8,97 | 0,007 |
| HPMC\_HP | 1 | 1,968 | 3,67% | 2,005 | 2,005 | 1,44 | 0,244 |
| Square | 1 | 8,265 | 15,42% | 8,265 | 8,265 | 5,92 | 0,024 |
| Lac\*Lac | 1 | 8,265 | 15,42% | 8,265 | 8,265 | 5,92 | 0,024 |
| 2-Way Interaction | 1 | 2,003 | 3,74% | 2,003 | 2,003 | 1,43 | 0,244 |
| Lac\*HPMC\_HP | 1 | 2,003 | 3,74% | 2,003 | 2,003 | 1,43 | 0,244 |
| Error | 22 | 30,726 | 57,31% | 30,726 | 1,397 |  |  |
| Lack-of-Fit | 20 | 22,118 | 41,26% | 22,118 | 1,106 | 0,26 | 0,963 |
| Pure Error | 2 | 8,608 | 16,06% | 8,608 | 4,304 |  |  |
| Total | 26 | 53,611 | 100,00% |  |  |  |  |

## Regression Equation in Uncoded Units

|  |  |  |
| --- | --- | --- |
| F\_SD\_2.5h(150min) | = | 28,7 - 81,9 Lac - 2,20 HPMC\_HP + 35,6 Lac\*Lac + 5,52 Lac\*HPMC\_HP |

## Fits and Diagnostics for All Observations

| Obs | F\_SD\_2.5h(150min) | Fit | SE Fit | 95% CI | Resid | Std Resid | Del Resid | HI |
| --- | --- | --- | --- | --- | --- | --- | --- | --- |
| 1 | 2,206 | 1,928 | 0,556 | (0,775; 3,081) | 0,278 | 0,27 | 0,26 | 0,221366 |
| 2 | 3,259 | 2,435 | 0,556 | (1,282; 3,589) | 0,824 | 0,79 | 0,78 | 0,221366 |
| 3 | 1,540 | 1,932 | 0,575 | (0,740; 3,124) | -0,392 | -0,38 | -0,37 | 0,236586 |
| 4 | 1,023 | 2,396 | 0,575 | (1,204; 3,588) | -1,373 | -1,33 | -1,35 | 0,236586 |
| 5 | 2,526 | 1,810 | 0,419 | (0,942; 2,678) | 0,716 | 0,65 | 0,64 | 0,125431 |
| 6 | 5,694 | 3,615 | 0,419 | (2,747; 4,483) | 2,079 | 1,88 | 2,01 | 0,125431 |
| 7 | 1,584 | 1,776 | 0,566 | (0,602; 2,949) | -0,192 | -0,19 | -0,18 | 0,229169 |
| 8 | 1,938 | 3,958 | 0,566 | (2,785; 5,131) | -2,020 | -1,95 | -2,09 | 0,229169 |
| 9 | 1,796 | 1,923 | 0,532 | (0,821; 3,026) | -0,128 | -0,12 | -0,12 | 0,202403 |
| 10 | 2,518 | 2,487 | 0,532 | (1,385; 3,590) | 0,031 | 0,03 | 0,03 | 0,202403 |
| 11 | 0,651 | 1,897 | 0,422 | (1,021; 2,773) | -1,246 | -1,13 | -1,14 | 0,127732 |
| 12 | 1,918 | 2,748 | 0,422 | (1,872; 3,624) | -0,830 | -0,75 | -0,74 | 0,127732 |
| 13 | 1,302 | 1,784 | 0,526 | (0,693; 2,875) | -0,482 | -0,46 | -0,45 | 0,198178 |
| 14 | 4,896 | 3,874 | 0,526 | (2,783; 4,965) | 1,022 | 0,97 | 0,96 | 0,198178 |
| 15 | 2,719 | 1,814 | 0,404 | (0,976; 2,652) | 0,905 | 0,81 | 0,81 | 0,116856 |
| 16 | 3,911 | 3,573 | 0,404 | (2,735; 4,411) | 0,338 | 0,30 | 0,30 | 0,116856 |
| 17 | 3,004 | 2,793 | 0,895 | (0,936; 4,649) | 0,212 | 0,27 | 0,27 | 0,573726 |
| 18 | 5,581 | 5,676 | 0,895 | (3,819; 7,532) | -0,094 | -0,12 | -0,12 | 0,573726 |
| 19 | 1,072 | 1,996 | 0,306 | (1,361; 2,631) | -0,924 | -0,81 | -0,80 | 0,067140 |
| 20 | 3,414 | 1,940 | 0,306 | (1,305; 2,574) | 1,475 | 1,29 | 1,31 | 0,066994 |
| 21 | 1,961 | 1,465 | 0,517 | (0,393; 2,537) | 0,496 | 0,47 | 0,46 | 0,191276 |
| 22 | 1,672 | 2,611 | 0,620 | (1,326; 3,896) | -0,939 | -0,93 | -0,93 | 0,274906 |
| 23 | 1,382 | 1,996 | 0,306 | (1,361; 2,631) | -0,614 | -0,54 | -0,53 | 0,067140 |
| 24 | 1,233 | 1,940 | 0,306 | (1,305; 2,574) | -0,707 | -0,62 | -0,61 | 0,066994 |
| 25 | 0,242 | 2,007 | 0,307 | (1,370; 2,644) | -1,765 | -1,55 | -1,60 | 0,067551 |
| 26 | 4,290 | 2,007 | 0,307 | (1,370; 2,644) | 2,283 | 2,00 | 2,16 | 0,067551 |
| 27 | 3,056 | 2,007 | 0,307 | (1,370; 2,644) | 1,049 | 0,92 | 0,92 | 0,067551 |

| Obs | Cook’s D | DFITS |  |  |
| --- | --- | --- | --- | --- |
| 1 | 0,00 | 0,13916 |  |  |
| 2 | 0,04 | 0,41756 |  |  |
| 3 | 0,01 | -0,20727 |  |  |
| 4 | 0,11 | -0,75430 |  |  |
| 5 | 0,01 | 0,24201 |  |  |
| 6 | 0,10 | 0,75964 |  |  |
| 7 | 0,00 | -0,09878 |  |  |
| 8 | 0,23 | -1,13986 |  |  |
| 9 | 0,00 | -0,05950 |  |  |
| 10 | 0,00 | 0,01448 |  |  |
| 11 | 0,04 | -0,43486 |  |  |
| 12 | 0,02 | -0,28482 |  |  |
| 13 | 0,01 | -0,22223 |  |  |
| 14 | 0,05 | 0,47944 |  |  |
| 15 | 0,02 | 0,29396 |  |  |
| 16 | 0,00 | 0,10833 |  |  |
| 17 | 0,02 | 0,31162 |  | X |
| 18 | 0,00 | -0,13826 |  | X |
| 19 | 0,01 | -0,21533 |  |  |
| 20 | 0,02 | 0,35180 |  |  |
| 21 | 0,01 | 0,22275 |  |  |
| 22 | 0,07 | -0,57276 |  |  |
| 23 | 0,00 | -0,14200 |  |  |
| 24 | 0,01 | -0,16357 |  |  |
| 25 | 0,03 | -0,43081 |  |  |
| 26 | 0,06 | 0,58152 | R |  |
| 27 | 0,01 | 0,24649 |  |  |

R  Large residual  
X  Unusual X

## Forward Selection of Terms

α to enter = 0,25

## Coded Coefficients

| Term | Coef | SE Coef | 95% CI | T-Value | P-Value | VIF |
| --- | --- | --- | --- | --- | --- | --- |
| Constant | 2,099 | 0,319 | (1,438; 2,761) | 6,58 | 0,000 |  |
| Lac | 1,565 | 0,519 | (0,488; 2,642) | 3,01 | 0,006 | 1,10 |
| HPMC\_HP | 0,630 | 0,490 | (-0,386; 1,647) | 1,29 | 0,212 | 1,00 |
| Lac\*Lac | 2,309 | 0,938 | (0,364; 4,255) | 2,46 | 0,022 | 1,00 |
| Lac\*HPMC\_HP | 1,59 | 1,20 | (-0,90; 4,08) | 1,33 | 0,199 | 1,10 |

## Model Summary

| S | R-sq | R-sq(adj) | PRESS | R-sq(pred) | AICc | BIC |
| --- | --- | --- | --- | --- | --- | --- |
| 1,21127 | 43,51% | 33,24% | 43,7057 | 23,51% | 97,64 | 101,22 |

## Analysis of Variance

| Source | DF | Seq SS | Contribution | Adj SS | Adj MS | F-Value | P-Value |
| --- | --- | --- | --- | --- | --- | --- | --- |
| Model | 4 | 24,864 | 43,51% | 24,864 | 6,216 | 4,24 | 0,011 |
| Linear | 2 | 13,397 | 23,45% | 15,751 | 7,875 | 5,37 | 0,013 |
| Lac | 1 | 11,014 | 19,27% | 13,325 | 13,325 | 9,08 | 0,006 |
| HPMC\_HP | 1 | 2,383 | 4,17% | 2,426 | 2,426 | 1,65 | 0,212 |
| Square | 1 | 8,889 | 15,56% | 8,889 | 8,889 | 6,06 | 0,022 |
| Lac\*Lac | 1 | 8,889 | 15,56% | 8,889 | 8,889 | 6,06 | 0,022 |
| 2-Way Interaction | 1 | 2,578 | 4,51% | 2,578 | 2,578 | 1,76 | 0,199 |
| Lac\*HPMC\_HP | 1 | 2,578 | 4,51% | 2,578 | 2,578 | 1,76 | 0,199 |
| Error | 22 | 32,278 | 56,49% | 32,278 | 1,467 |  |  |
| Lack-of-Fit | 20 | 22,352 | 39,12% | 22,352 | 1,118 | 0,23 | 0,975 |
| Pure Error | 2 | 9,925 | 17,37% | 9,925 | 4,963 |  |  |
| Total | 26 | 57,142 | 100,00% |  |  |  |  |

## Regression Equation in Uncoded Units

|  |  |  |
| --- | --- | --- |
| F\_SD\_3h(180min) | = | 32,0 - 90,1 Lac - 2,51 HPMC\_HP + 37,0 Lac\*Lac + 6,26 Lac\*HPMC\_HP |

## Fits and Diagnostics for All Observations

| Obs | F\_SD\_3h(180min) | Fit | SE Fit | 95% CI | Resid | Std Resid | Del Resid | HI |
| --- | --- | --- | --- | --- | --- | --- | --- | --- |
| 1 | 2,375 | 2,013 | 0,570 | (0,831; 3,195) | 0,363 | 0,34 | 0,33 | 0,221366 |
| 2 | 3,270 | 2,432 | 0,570 | (1,250; 3,614) | 0,839 | 0,78 | 0,78 | 0,221366 |
| 3 | 1,617 | 2,018 | 0,589 | (0,796; 3,240) | -0,401 | -0,38 | -0,37 | 0,236586 |
| 4 | 0,976 | 2,388 | 0,589 | (1,166; 3,610) | -1,412 | -1,33 | -1,36 | 0,236586 |
| 5 | 2,604 | 1,860 | 0,429 | (0,971; 2,750) | 0,743 | 0,66 | 0,65 | 0,125431 |
| 6 | 5,741 | 3,752 | 0,429 | (2,862; 4,641) | 1,990 | 1,76 | 1,85 | 0,125431 |
| 7 | 1,658 | 1,816 | 0,580 | (0,614; 3,019) | -0,158 | -0,15 | -0,15 | 0,229169 |
| 8 | 2,097 | 4,135 | 0,580 | (2,933; 5,338) | -2,039 | -1,92 | -2,05 | 0,229169 |
| 9 | 1,707 | 2,006 | 0,545 | (0,876; 3,136) | -0,299 | -0,28 | -0,27 | 0,202403 |
| 10 | 2,506 | 2,490 | 0,545 | (1,360; 3,620) | 0,016 | 0,01 | 0,01 | 0,202403 |
| 11 | 0,812 | 1,973 | 0,433 | (1,075; 2,870) | -1,161 | -1,03 | -1,03 | 0,127732 |
| 12 | 1,867 | 2,781 | 0,433 | (1,883; 3,679) | -0,914 | -0,81 | -0,80 | 0,127732 |
| 13 | 1,422 | 1,827 | 0,539 | (0,709; 2,945) | -0,405 | -0,37 | -0,37 | 0,198178 |
| 14 | 5,046 | 4,041 | 0,539 | (2,923; 5,159) | 1,005 | 0,93 | 0,92 | 0,198178 |
| 15 | 2,717 | 1,866 | 0,414 | (1,007; 2,725) | 0,851 | 0,75 | 0,74 | 0,116856 |
| 16 | 4,260 | 3,705 | 0,414 | (2,846; 4,564) | 0,555 | 0,49 | 0,48 | 0,116856 |
| 17 | 3,076 | 2,896 | 0,917 | (0,993; 4,799) | 0,180 | 0,23 | 0,22 | 0,573726 |
| 18 | 5,780 | 5,853 | 0,917 | (3,951; 7,756) | -0,073 | -0,09 | -0,09 | 0,573726 |
| 19 | 1,085 | 2,053 | 0,314 | (1,402; 2,704) | -0,968 | -0,83 | -0,82 | 0,067140 |
| 20 | 3,525 | 1,991 | 0,314 | (1,340; 2,641) | 1,535 | 1,31 | 1,33 | 0,066994 |
| 21 | 2,072 | 1,469 | 0,530 | (0,370; 2,568) | 0,603 | 0,55 | 0,54 | 0,191276 |
| 22 | 1,762 | 2,730 | 0,635 | (1,412; 4,047) | -0,967 | -0,94 | -0,94 | 0,274906 |
| 23 | 1,412 | 2,053 | 0,314 | (1,402; 2,704) | -0,641 | -0,55 | -0,54 | 0,067140 |
| 24 | 1,311 | 1,991 | 0,314 | (1,340; 2,641) | -0,679 | -0,58 | -0,57 | 0,066994 |
| 25 | 0,138 | 2,065 | 0,315 | (1,412; 2,718) | -1,927 | -1,65 | -1,72 | 0,067551 |
| 26 | 4,535 | 2,065 | 0,315 | (1,412; 2,718) | 2,469 | 2,11 | 2,31 | 0,067551 |
| 27 | 2,962 | 2,065 | 0,315 | (1,412; 2,718) | 0,897 | 0,77 | 0,76 | 0,067551 |

| Obs | Cook’s D | DFITS |  |  |
| --- | --- | --- | --- | --- |
| 1 | 0,01 | 0,17716 |  |  |
| 2 | 0,04 | 0,41461 |  |  |
| 3 | 0,01 | -0,20677 |  |  |
| 4 | 0,11 | -0,75666 |  |  |
| 5 | 0,01 | 0,24524 |  |  |
| 6 | 0,09 | 0,70102 |  |  |
| 7 | 0,00 | -0,07929 |  |  |
| 8 | 0,22 | -1,11902 |  |  |
| 9 | 0,00 | -0,13641 |  |  |
| 10 | 0,00 | 0,00719 |  |  |
| 11 | 0,03 | -0,39322 |  |  |
| 12 | 0,02 | -0,30673 |  |  |
| 13 | 0,01 | -0,18203 |  |  |
| 14 | 0,04 | 0,45887 |  |  |
| 15 | 0,01 | 0,26923 |  |  |
| 16 | 0,01 | 0,17424 |  |  |
| 17 | 0,01 | 0,25863 |  | X |
| 18 | 0,00 | -0,10497 |  | X |
| 19 | 0,01 | -0,22029 |  |  |
| 20 | 0,02 | 0,35765 |  |  |
| 21 | 0,01 | 0,26477 |  |  |
| 22 | 0,07 | -0,57580 |  |  |
| 23 | 0,00 | -0,14455 |  |  |
| 24 | 0,00 | -0,15316 |  |  |
| 25 | 0,04 | -0,46276 |  |  |
| 26 | 0,06 | 0,62172 | R |  |
| 27 | 0,01 | 0,20433 |  |  |

R  Large residual  
X  Unusual X

## Forward Selection of Terms

α to enter = 0,25

## Coded Coefficients

| Term | Coef | SE Coef | 95% CI | T-Value | P-Value | VIF |
| --- | --- | --- | --- | --- | --- | --- |
| Constant | 2,173 | 0,321 | (1,507; 2,838) | 6,77 | 0,000 |  |
| Lac | 1,631 | 0,522 | (0,548; 2,715) | 3,12 | 0,005 | 1,10 |
| HPMC\_HP | 0,698 | 0,493 | (-0,325; 1,720) | 1,42 | 0,171 | 1,00 |
| Lac\*Lac | 2,352 | 0,944 | (0,395; 4,310) | 2,49 | 0,021 | 1,00 |
| Lac\*HPMC\_HP | 1,83 | 1,21 | (-0,67; 4,33) | 1,52 | 0,143 | 1,10 |

## Model Summary

| S | R-sq | R-sq(adj) | PRESS | R-sq(pred) | AICc | BIC |
| --- | --- | --- | --- | --- | --- | --- |
| 1,21852 | 45,39% | 35,46% | 44,1649 | 26,17% | 97,97 | 101,54 |

## Analysis of Variance

| Source | DF | Seq SS | Contribution | Adj SS | Adj MS | F-Value | P-Value |
| --- | --- | --- | --- | --- | --- | --- | --- |
| Model | 4 | 27,152 | 45,39% | 27,152 | 6,788 | 4,57 | 0,008 |
| Linear | 2 | 14,512 | 24,26% | 17,456 | 8,728 | 5,88 | 0,009 |
| Lac | 1 | 11,586 | 19,37% | 14,482 | 14,482 | 9,75 | 0,005 |
| HPMC\_HP | 1 | 2,926 | 4,89% | 2,974 | 2,974 | 2,00 | 0,171 |
| Square | 1 | 9,223 | 15,42% | 9,223 | 9,223 | 6,21 | 0,021 |
| Lac\*Lac | 1 | 9,223 | 15,42% | 9,223 | 9,223 | 6,21 | 0,021 |
| 2-Way Interaction | 1 | 3,417 | 5,71% | 3,417 | 3,417 | 2,30 | 0,143 |
| Lac\*HPMC\_HP | 1 | 3,417 | 5,71% | 3,417 | 3,417 | 2,30 | 0,143 |
| Error | 22 | 32,665 | 54,61% | 32,665 | 1,485 |  |  |
| Lack-of-Fit | 20 | 22,215 | 37,14% | 22,215 | 1,111 | 0,21 | 0,979 |
| Pure Error | 2 | 10,451 | 17,47% | 10,451 | 5,225 |  |  |
| Total | 26 | 59,817 | 100,00% |  |  |  |  |

## Regression Equation in Uncoded Units

|  |  |  |
| --- | --- | --- |
| F\_SD\_3.5h(210min) | = | 36,0 - 99,5 Lac - 2,92 HPMC\_HP + 37,6 Lac\*Lac + 7,21 Lac\*HPMC\_HP |

## Fits and Diagnostics for All Observations

| Obs | F\_SD\_3.5h(210min) | Fit | SE Fit | 95% CI | Resid | Std Resid | Del Resid | HI |
| --- | --- | --- | --- | --- | --- | --- | --- | --- |
| 1 | 2,522 | 2,102 | 0,573 | (0,913; 3,291) | 0,421 | 0,39 | 0,38 | 0,221366 |
| 2 | 3,262 | 2,414 | 0,573 | (1,225; 3,603) | 0,848 | 0,79 | 0,78 | 0,221366 |
| 3 | 1,694 | 2,109 | 0,593 | (0,879; 3,338) | -0,415 | -0,39 | -0,38 | 0,236586 |
| 4 | 0,947 | 2,364 | 0,593 | (1,135; 3,593) | -1,417 | -1,33 | -1,36 | 0,236586 |
| 5 | 2,736 | 1,901 | 0,432 | (1,006; 2,796) | 0,835 | 0,73 | 0,73 | 0,125431 |
| 6 | 5,756 | 3,908 | 0,432 | (3,013; 4,803) | 1,848 | 1,62 | 1,69 | 0,125431 |
| 7 | 1,729 | 1,842 | 0,583 | (0,632; 3,052) | -0,114 | -0,11 | -0,10 | 0,229169 |
| 8 | 2,304 | 4,342 | 0,583 | (3,132; 5,552) | -2,038 | -1,90 | -2,04 | 0,229169 |
| 9 | 1,695 | 2,093 | 0,548 | (0,956; 3,230) | -0,398 | -0,37 | -0,36 | 0,202403 |
| 10 | 2,498 | 2,479 | 0,548 | (1,343; 3,616) | 0,019 | 0,02 | 0,02 | 0,202403 |
| 11 | 0,934 | 2,049 | 0,435 | (1,146; 2,952) | -1,115 | -0,98 | -0,98 | 0,127732 |
| 12 | 1,818 | 2,809 | 0,435 | (1,906; 3,712) | -0,991 | -0,87 | -0,87 | 0,127732 |
| 13 | 1,470 | 1,857 | 0,542 | (0,732; 2,981) | -0,387 | -0,35 | -0,35 | 0,198178 |
| 14 | 5,236 | 4,235 | 0,542 | (3,110; 5,360) | 1,001 | 0,92 | 0,91 | 0,198178 |
| 15 | 2,553 | 1,908 | 0,417 | (1,044; 2,772) | 0,646 | 0,56 | 0,55 | 0,116856 |
| 16 | 4,551 | 3,855 | 0,417 | (2,991; 4,719) | 0,696 | 0,61 | 0,60 | 0,116856 |
| 17 | 3,148 | 2,955 | 0,923 | (1,041; 4,869) | 0,193 | 0,24 | 0,24 | 0,573726 |
| 18 | 5,967 | 6,020 | 0,923 | (4,106; 7,934) | -0,053 | -0,07 | -0,06 | 0,573726 |
| 19 | 1,110 | 2,121 | 0,316 | (1,466; 2,776) | -1,011 | -0,86 | -0,85 | 0,067140 |
| 20 | 3,635 | 2,053 | 0,315 | (1,398; 2,707) | 1,582 | 1,34 | 1,37 | 0,066994 |
| 21 | 2,104 | 1,475 | 0,533 | (0,370; 2,580) | 0,629 | 0,57 | 0,56 | 0,191276 |
| 22 | 1,898 | 2,871 | 0,639 | (1,546; 4,196) | -0,972 | -0,94 | -0,93 | 0,274906 |
| 23 | 1,445 | 2,121 | 0,316 | (1,466; 2,776) | -0,677 | -0,57 | -0,57 | 0,067140 |
| 24 | 1,463 | 2,053 | 0,315 | (1,398; 2,707) | -0,590 | -0,50 | -0,49 | 0,066994 |
| 25 | 0,199 | 2,135 | 0,317 | (1,478; 2,792) | -1,936 | -1,65 | -1,72 | 0,067551 |
| 26 | 4,741 | 2,135 | 0,317 | (1,478; 2,792) | 2,605 | 2,21 | 2,45 | 0,067551 |
| 27 | 2,925 | 2,135 | 0,317 | (1,478; 2,792) | 0,790 | 0,67 | 0,66 | 0,067551 |

| Obs | Cook’s D | DFITS |  |  |
| --- | --- | --- | --- | --- |
| 1 | 0,01 | 0,20447 |  |  |
| 2 | 0,04 | 0,41666 |  |  |
| 3 | 0,01 | -0,21260 |  |  |
| 4 | 0,11 | -0,75521 |  |  |
| 5 | 0,02 | 0,27464 |  |  |
| 6 | 0,08 | 0,63951 |  |  |
| 7 | 0,00 | -0,05659 |  |  |
| 8 | 0,22 | -1,11054 |  |  |
| 9 | 0,01 | -0,18045 |  |  |
| 10 | 0,00 | 0,00853 |  |  |
| 11 | 0,03 | -0,37454 |  |  |
| 12 | 0,02 | -0,33129 |  |  |
| 13 | 0,01 | -0,17278 |  |  |
| 14 | 0,04 | 0,45430 |  |  |
| 15 | 0,01 | 0,20183 |  |  |
| 16 | 0,01 | 0,21778 |  |  |
| 17 | 0,02 | 0,27540 |  | X |
| 18 | 0,00 | -0,07504 |  | X |
| 19 | 0,01 | -0,22903 |  |  |
| 20 | 0,03 | 0,36734 |  |  |
| 21 | 0,02 | 0,27476 |  |  |
| 22 | 0,07 | -0,57531 |  |  |
| 23 | 0,00 | -0,15184 |  |  |
| 24 | 0,00 | -0,13194 |  |  |
| 25 | 0,04 | -0,46202 |  |  |
| 26 | 0,07 | 0,66055 | R |  |
| 27 | 0,01 | 0,17844 |  |  |

R  Large residual  
X  Unusual X

## Forward Selection of Terms

α to enter = 0,25

## Coded Coefficients

| Term | Coef | SE Coef | 95% CI | T-Value | P-Value | VIF |
| --- | --- | --- | --- | --- | --- | --- |
| Constant | 2,257 | 0,325 | (1,583; 2,931) | 6,94 | 0,000 |  |
| Lac | 1,613 | 0,529 | (0,515; 2,711) | 3,05 | 0,006 | 1,10 |
| HPMC\_HP | 0,645 | 0,500 | (-0,391; 1,682) | 1,29 | 0,210 | 1,00 |
| Lac\*Lac | 2,397 | 0,956 | (0,414; 4,381) | 2,51 | 0,020 | 1,00 |
| Lac\*HPMC\_HP | 2,19 | 1,22 | (-0,35; 4,72) | 1,79 | 0,087 | 1,10 |

## Model Summary

| S | R-sq | R-sq(adj) | PRESS | R-sq(pred) | AICc | BIC |
| --- | --- | --- | --- | --- | --- | --- |
| 1,23480 | 45,03% | 35,03% | 45,7641 | 25,00% | 98,68 | 102,26 |

## Analysis of Variance

| Source | DF | Seq SS | Contribution | Adj SS | Adj MS | F-Value | P-Value |
| --- | --- | --- | --- | --- | --- | --- | --- |
| Model | 4 | 27,476 | 45,03% | 27,476 | 6,869 | 4,51 | 0,008 |
| Linear | 2 | 13,011 | 21,32% | 16,698 | 8,349 | 5,48 | 0,012 |
| Lac | 1 | 10,513 | 17,23% | 14,155 | 14,155 | 9,28 | 0,006 |
| HPMC\_HP | 1 | 2,497 | 4,09% | 2,543 | 2,543 | 1,67 | 0,210 |
| Square | 1 | 9,577 | 15,70% | 9,577 | 9,577 | 6,28 | 0,020 |
| Lac\*Lac | 1 | 9,577 | 15,70% | 9,577 | 9,577 | 6,28 | 0,020 |
| 2-Way Interaction | 1 | 4,888 | 8,01% | 4,888 | 4,888 | 3,21 | 0,087 |
| Lac\*HPMC\_HP | 1 | 4,888 | 8,01% | 4,888 | 4,888 | 3,21 | 0,087 |
| Error | 22 | 33,544 | 54,97% | 33,544 | 1,525 |  |  |
| Lack-of-Fit | 20 | 23,328 | 38,23% | 23,328 | 1,166 | 0,23 | 0,974 |
| Pure Error | 2 | 10,216 | 16,74% | 10,216 | 5,108 |  |  |
| Total | 26 | 61,020 | 100,00% |  |  |  |  |

## Regression Equation in Uncoded Units

|  |  |  |
| --- | --- | --- |
| F\_SD\_4h(240min) | = | 43,5 - 113,6 Lac - 3,68 HPMC\_HP + 38,4 Lac\*Lac + 8,63 Lac\*HPMC\_HP |

## Fits and Diagnostics for All Observations

| Obs | F\_SD\_4h(240min) | Fit | SE Fit | 95% CI | Resid | Std Resid | Del Resid | HI |
| --- | --- | --- | --- | --- | --- | --- | --- | --- |
| 1 | 3,590 | 2,374 | 0,581 | (1,169; 3,579) | 1,216 | 1,12 | 1,12 | 0,221366 |
| 2 | 3,304 | 2,408 | 0,581 | (1,204; 3,613) | 0,895 | 0,82 | 0,82 | 0,221366 |
| 3 | 1,736 | 2,388 | 0,601 | (1,142; 3,633) | -0,651 | -0,60 | -0,59 | 0,236586 |
| 4 | 0,962 | 2,355 | 0,601 | (1,109; 3,601) | -1,393 | -1,29 | -1,31 | 0,236586 |
| 5 | 2,780 | 1,958 | 0,437 | (1,051; 2,865) | 0,822 | 0,71 | 0,70 | 0,125431 |
| 6 | 5,733 | 4,020 | 0,437 | (3,113; 4,927) | 1,714 | 1,48 | 1,53 | 0,125431 |
| 7 | 1,835 | 1,837 | 0,591 | (0,611; 3,063) | -0,001 | -0,00 | -0,00 | 0,229169 |
| 8 | 2,436 | 4,488 | 0,591 | (3,262; 5,714) | -2,052 | -1,89 | -2,02 | 0,229169 |
| 9 | 1,672 | 2,356 | 0,556 | (1,203; 3,508) | -0,684 | -0,62 | -0,61 | 0,202403 |
| 10 | 2,478 | 2,479 | 0,556 | (1,327; 3,631) | -0,001 | -0,00 | -0,00 | 0,202403 |
| 11 | 1,058 | 2,264 | 0,441 | (1,348; 3,179) | -1,205 | -1,05 | -1,05 | 0,127732 |
| 12 | 1,842 | 2,835 | 0,441 | (1,920; 3,750) | -0,993 | -0,86 | -0,86 | 0,127732 |
| 13 | 1,556 | 1,866 | 0,550 | (0,726; 3,006) | -0,310 | -0,28 | -0,27 | 0,198178 |
| 14 | 5,364 | 4,373 | 0,550 | (3,233; 5,513) | 0,991 | 0,90 | 0,89 | 0,198178 |
| 15 | 2,538 | 1,972 | 0,422 | (1,097; 2,848) | 0,565 | 0,49 | 0,48 | 0,116856 |
| 16 | 4,690 | 3,963 | 0,422 | (3,087; 4,838) | 0,728 | 0,63 | 0,62 | 0,116856 |
| 17 | 3,204 | 3,125 | 0,935 | (1,185; 5,065) | 0,079 | 0,10 | 0,10 | 0,573726 |
| 18 | 6,124 | 6,114 | 0,935 | (4,174; 8,053) | 0,011 | 0,01 | 0,01 | 0,573726 |
| 19 | 1,161 | 2,209 | 0,320 | (1,546; 2,873) | -1,048 | -0,88 | -0,87 | 0,067140 |
| 20 | 3,679 | 2,146 | 0,320 | (1,483; 2,809) | 1,533 | 1,29 | 1,31 | 0,066994 |
| 21 | 2,159 | 1,612 | 0,540 | (0,492; 2,732) | 0,547 | 0,49 | 0,48 | 0,191276 |
| 22 | 1,999 | 2,902 | 0,647 | (1,560; 4,245) | -0,903 | -0,86 | -0,85 | 0,274906 |
| 23 | 1,482 | 2,209 | 0,320 | (1,546; 2,873) | -0,727 | -0,61 | -0,60 | 0,067140 |
| 24 | 1,619 | 2,146 | 0,320 | (1,483; 2,809) | -0,527 | -0,44 | -0,43 | 0,066994 |
| 25 | 0,362 | 2,222 | 0,321 | (1,557; 2,888) | -1,860 | -1,56 | -1,62 | 0,067551 |
| 26 | 4,876 | 2,222 | 0,321 | (1,557; 2,888) | 2,654 | 2,23 | 2,47 | 0,067551 |
| 27 | 2,824 | 2,222 | 0,321 | (1,557; 2,888) | 0,602 | 0,51 | 0,50 | 0,067551 |

| Obs | Cook’s D | DFITS |  |  |
| --- | --- | --- | --- | --- |
| 1 | 0,07 | 0,59856 |  |  |
| 2 | 0,04 | 0,43468 |  |  |
| 3 | 0,02 | -0,33102 |  |  |
| 4 | 0,10 | -0,73037 |  |  |
| 5 | 0,01 | 0,26642 |  |  |
| 6 | 0,06 | 0,57881 |  |  |
| 7 | 0,00 | -0,00069 |  |  |
| 8 | 0,21 | -1,10218 |  |  |
| 9 | 0,02 | -0,30781 |  |  |
| 10 | 0,00 | -0,00041 |  |  |
| 11 | 0,03 | -0,40088 |  |  |
| 12 | 0,02 | -0,32751 |  |  |
| 13 | 0,00 | -0,13650 |  |  |
| 14 | 0,04 | 0,44358 |  |  |
| 15 | 0,01 | 0,17409 |  |  |
| 16 | 0,01 | 0,22486 |  |  |
| 17 | 0,00 | 0,11153 |  | X |
| 18 | 0,00 | 0,01508 |  | X |
| 19 | 0,01 | -0,23452 |  |  |
| 20 | 0,02 | 0,34986 |  |  |
| 21 | 0,01 | 0,23547 |  |  |
| 22 | 0,06 | -0,52559 |  |  |
| 23 | 0,01 | -0,16123 |  |  |
| 24 | 0,00 | -0,11619 |  |  |
| 25 | 0,04 | -0,43503 |  |  |
| 26 | 0,07 | 0,66487 | R |  |
| 27 | 0,00 | 0,13358 |  |  |

R  Large residual  
X  Unusual X

## Forward Selection of Terms

α to enter = 0,25

## Coded Coefficients

| Term | Coef | SE Coef | 95% CI | T-Value | P-Value | VIF |
| --- | --- | --- | --- | --- | --- | --- |
| Constant | 2,317 | 0,324 | (1,646; 2,989) | 7,16 | 0,000 |  |
| Lac | 1,615 | 0,527 | (0,522; 2,708) | 3,06 | 0,006 | 1,10 |
| HPMC\_HP | 0,703 | 0,498 | (-0,329; 1,735) | 1,41 | 0,172 | 1,00 |
| Lac\*Lac | 2,465 | 0,952 | (0,490; 4,440) | 2,59 | 0,017 | 1,00 |
| Lac\*HPMC\_HP | 2,25 | 1,22 | (-0,27; 4,78) | 1,85 | 0,078 | 1,10 |

## Model Summary

| S | R-sq | R-sq(adj) | PRESS | R-sq(pred) | AICc | BIC |
| --- | --- | --- | --- | --- | --- | --- |
| 1,22955 | 46,30% | 36,54% | 45,5531 | 26,45% | 98,45 | 102,03 |

## Analysis of Variance

| Source | DF | Seq SS | Contribution | Adj SS | Adj MS | F-Value | P-Value |
| --- | --- | --- | --- | --- | --- | --- | --- |
| Model | 4 | 28,679 | 46,30% | 28,679 | 7,170 | 4,74 | 0,007 |
| Linear | 2 | 13,379 | 21,60% | 17,206 | 8,603 | 5,69 | 0,010 |
| Lac | 1 | 10,412 | 16,81% | 14,189 | 14,189 | 9,39 | 0,006 |
| HPMC\_HP | 1 | 2,967 | 4,79% | 3,017 | 3,017 | 2,00 | 0,172 |
| Square | 1 | 10,128 | 16,35% | 10,128 | 10,128 | 6,70 | 0,017 |
| Lac\*Lac | 1 | 10,128 | 16,35% | 10,128 | 10,128 | 6,70 | 0,017 |
| 2-Way Interaction | 1 | 5,173 | 8,35% | 5,173 | 5,173 | 3,42 | 0,078 |
| Lac\*HPMC\_HP | 1 | 5,173 | 8,35% | 5,173 | 5,173 | 3,42 | 0,078 |
| Error | 22 | 33,259 | 53,70% | 33,259 | 1,512 |  |  |
| Lack-of-Fit | 20 | 23,088 | 37,28% | 23,088 | 1,154 | 0,23 | 0,974 |
| Pure Error | 2 | 10,171 | 16,42% | 10,171 | 5,086 |  |  |
| Total | 26 | 61,939 | 100,00% |  |  |  |  |

## Regression Equation in Uncoded Units

|  |  |  |
| --- | --- | --- |
| F\_SD\_4.5h(270min) | = | 44,4 - 117,1 Lac - 3,74 HPMC\_HP + 39,4 Lac\*Lac + 8,87 Lac\*HPMC\_HP |

## Fits and Diagnostics for All Observations

| Obs | F\_SD\_4.5h(270min) | Fit | SE Fit | 95% CI | Resid | Std Resid | Del Resid | HI |
| --- | --- | --- | --- | --- | --- | --- | --- | --- |
| 1 | 3,719 | 2,431 | 0,578 | (1,231; 3,631) | 1,288 | 1,19 | 1,20 | 0,221366 |
| 2 | 3,300 | 2,422 | 0,578 | (1,223; 3,622) | 0,878 | 0,81 | 0,80 | 0,221366 |
| 3 | 1,817 | 2,444 | 0,598 | (1,204; 3,684) | -0,627 | -0,58 | -0,58 | 0,236586 |
| 4 | 0,927 | 2,366 | 0,598 | (1,126; 3,606) | -1,439 | -1,34 | -1,37 | 0,236586 |
| 5 | 2,851 | 2,039 | 0,435 | (1,136; 2,942) | 0,811 | 0,71 | 0,70 | 0,125431 |
| 6 | 5,701 | 4,116 | 0,435 | (3,213; 5,019) | 1,585 | 1,38 | 1,41 | 0,125431 |
| 7 | 2,055 | 1,925 | 0,589 | (0,705; 3,146) | 0,130 | 0,12 | 0,12 | 0,229169 |
| 8 | 2,561 | 4,609 | 0,589 | (3,388; 5,829) | -2,048 | -1,90 | -2,03 | 0,229169 |
| 9 | 1,609 | 2,414 | 0,553 | (1,267; 3,561) | -0,804 | -0,73 | -0,72 | 0,202403 |
| 10 | 2,514 | 2,497 | 0,553 | (1,350; 3,644) | 0,017 | 0,02 | 0,02 | 0,202403 |
| 11 | 1,195 | 2,327 | 0,439 | (1,416; 3,239) | -1,133 | -0,99 | -0,99 | 0,127732 |
| 12 | 1,899 | 2,871 | 0,439 | (1,959; 3,782) | -0,971 | -0,85 | -0,84 | 0,127732 |
| 13 | 1,622 | 1,953 | 0,547 | (0,818; 3,088) | -0,332 | -0,30 | -0,29 | 0,198178 |
| 14 | 5,475 | 4,488 | 0,547 | (3,352; 5,623) | 0,987 | 0,90 | 0,89 | 0,198178 |
| 15 | 2,481 | 2,053 | 0,420 | (1,181; 2,925) | 0,428 | 0,37 | 0,36 | 0,116856 |
| 16 | 4,851 | 4,056 | 0,420 | (3,184; 4,928) | 0,795 | 0,69 | 0,68 | 0,116856 |
| 17 | 3,317 | 3,251 | 0,931 | (1,320; 5,183) | 0,065 | 0,08 | 0,08 | 0,573726 |
| 18 | 6,281 | 6,237 | 0,931 | (4,306; 8,168) | 0,044 | 0,05 | 0,05 | 0,573726 |
| 19 | 1,209 | 2,265 | 0,319 | (1,604; 2,926) | -1,057 | -0,89 | -0,89 | 0,067140 |
| 20 | 3,713 | 2,196 | 0,318 | (1,536; 2,856) | 1,517 | 1,28 | 1,30 | 0,066994 |
| 21 | 2,151 | 1,614 | 0,538 | (0,499; 2,729) | 0,537 | 0,49 | 0,48 | 0,191276 |
| 22 | 2,102 | 3,020 | 0,645 | (1,683; 4,357) | -0,918 | -0,88 | -0,87 | 0,274906 |
| 23 | 1,513 | 2,265 | 0,319 | (1,604; 2,926) | -0,752 | -0,63 | -0,62 | 0,067140 |
| 24 | 1,738 | 2,196 | 0,318 | (1,536; 2,856) | -0,458 | -0,39 | -0,38 | 0,066994 |
| 25 | 0,501 | 2,279 | 0,320 | (1,616; 2,942) | -1,778 | -1,50 | -1,54 | 0,067551 |
| 26 | 5,011 | 2,279 | 0,320 | (1,616; 2,942) | 2,732 | 2,30 | 2,58 | 0,067551 |
| 27 | 2,782 | 2,279 | 0,320 | (1,616; 2,942) | 0,503 | 0,42 | 0,42 | 0,067551 |

| Obs | Cook’s D | DFITS |  |  |
| --- | --- | --- | --- | --- |
| 1 | 0,08 | 0,63937 |  |  |
| 2 | 0,04 | 0,42790 |  |  |
| 3 | 0,02 | -0,32015 |  |  |
| 4 | 0,11 | -0,76044 |  |  |
| 5 | 0,01 | 0,26408 |  |  |
| 6 | 0,05 | 0,53370 |  |  |
| 7 | 0,00 | 0,06400 |  |  |
| 8 | 0,21 | -1,10484 |  |  |
| 9 | 0,03 | -0,36506 |  |  |
| 10 | 0,00 | 0,00773 |  |  |
| 11 | 0,03 | -0,37724 |  |  |
| 12 | 0,02 | -0,32145 |  |  |
| 13 | 0,00 | -0,14666 |  |  |
| 14 | 0,04 | 0,44359 |  |  |
| 15 | 0,00 | 0,13215 |  |  |
| 16 | 0,01 | 0,24707 |  |  |
| 17 | 0,00 | 0,09189 |  | X |
| 18 | 0,00 | 0,06174 |  | X |
| 19 | 0,01 | -0,23750 |  |  |
| 20 | 0,02 | 0,34753 |  |  |
| 21 | 0,01 | 0,23188 |  |  |
| 22 | 0,06 | -0,53710 |  |  |
| 23 | 0,01 | -0,16752 |  |  |
| 24 | 0,00 | -0,10134 |  |  |
| 25 | 0,03 | -0,41548 |  |  |
| 26 | 0,08 | 0,69453 | R |  |
| 27 | 0,00 | 0,11188 |  |  |

R  Large residual  
X  Unusual X

## Forward Selection of Terms

α to enter = 0,25

## Coded Coefficients

| Term | Coef | SE Coef | 95% CI | T-Value | P-Value | VIF |
| --- | --- | --- | --- | --- | --- | --- |
| Constant | 2,355 | 0,318 | (1,695; 3,015) | 7,40 | 0,000 |  |
| Lac | 1,690 | 0,518 | (0,616; 2,764) | 3,26 | 0,004 | 1,10 |
| HPMC\_HP | 0,760 | 0,489 | (-0,254; 1,773) | 1,55 | 0,134 | 1,00 |
| Lac\*Lac | 2,488 | 0,936 | (0,547; 4,428) | 2,66 | 0,014 | 1,00 |
| Lac\*HPMC\_HP | 2,26 | 1,20 | (-0,22; 4,75) | 1,89 | 0,072 | 1,10 |

## Model Summary

| S | R-sq | R-sq(adj) | PRESS | R-sq(pred) | AICc | BIC |
| --- | --- | --- | --- | --- | --- | --- |
| 1,20802 | 48,81% | 39,51% | 43,9632 | 29,91% | 97,50 | 101,07 |

## Analysis of Variance

| Source | DF | Seq SS | Contribution | Adj SS | Adj MS | F-Value | P-Value |
| --- | --- | --- | --- | --- | --- | --- | --- |
| Model | 4 | 30,616 | 48,81% | 30,616 | 7,654 | 5,24 | 0,004 |
| Linear | 2 | 15,072 | 24,03% | 19,061 | 9,531 | 6,53 | 0,006 |
| Lac | 1 | 11,603 | 18,50% | 15,537 | 15,537 | 10,65 | 0,004 |
| HPMC\_HP | 1 | 3,469 | 5,53% | 3,524 | 3,524 | 2,41 | 0,134 |
| Square | 1 | 10,313 | 16,44% | 10,313 | 10,313 | 7,07 | 0,014 |
| Lac\*Lac | 1 | 10,313 | 16,44% | 10,313 | 10,313 | 7,07 | 0,014 |
| 2-Way Interaction | 1 | 5,231 | 8,34% | 5,231 | 5,231 | 3,58 | 0,072 |
| Lac\*HPMC\_HP | 1 | 5,231 | 8,34% | 5,231 | 5,231 | 3,58 | 0,072 |
| Error | 22 | 32,105 | 51,19% | 32,105 | 1,459 |  |  |
| Lack-of-Fit | 20 | 22,273 | 35,51% | 22,273 | 1,114 | 0,23 | 0,974 |
| Pure Error | 2 | 9,832 | 15,68% | 9,832 | 4,916 |  |  |
| Total | 26 | 62,721 | 100,00% |  |  |  |  |

## Regression Equation in Uncoded Units

|  |  |  |
| --- | --- | --- |
| F\_SD\_5h(300min) | = | 44,1 - 117,6 Lac - 3,71 HPMC\_HP + 39,8 Lac\*Lac + 8,92 Lac\*HPMC\_HP |

## Fits and Diagnostics for All Observations

| Obs | F\_SD\_5h(300min) | Fit | SE Fit | 95% CI | Resid | Std Resid | Del Resid | HI |
| --- | --- | --- | --- | --- | --- | --- | --- | --- |
| 1 | 3,441 | 2,401 | 0,568 | (1,222; 3,580) | 1,040 | 0,98 | 0,97 | 0,221366 |
| 2 | 3,335 | 2,458 | 0,568 | (1,279; 3,637) | 0,877 | 0,82 | 0,82 | 0,221366 |
| 3 | 1,801 | 2,412 | 0,588 | (1,194; 3,631) | -0,611 | -0,58 | -0,57 | 0,236586 |
| 4 | 0,963 | 2,400 | 0,588 | (1,181; 3,618) | -1,437 | -1,36 | -1,39 | 0,236586 |
| 5 | 2,870 | 2,056 | 0,428 | (1,168; 2,943) | 0,814 | 0,72 | 0,71 | 0,125431 |
| 6 | 5,707 | 4,210 | 0,428 | (3,323; 5,097) | 1,497 | 1,33 | 1,35 | 0,125431 |
| 7 | 2,089 | 1,955 | 0,578 | (0,756; 3,155) | 0,134 | 0,13 | 0,12 | 0,229169 |
| 8 | 2,667 | 4,720 | 0,578 | (3,520; 5,919) | -2,052 | -1,93 | -2,08 | 0,229169 |
| 9 | 1,635 | 2,386 | 0,543 | (1,258; 3,513) | -0,751 | -0,70 | -0,69 | 0,202403 |
| 10 | 2,462 | 2,535 | 0,543 | (1,408; 3,662) | -0,073 | -0,07 | -0,07 | 0,202403 |
| 11 | 1,282 | 2,309 | 0,432 | (1,414; 3,205) | -1,027 | -0,91 | -0,91 | 0,127732 |
| 12 | 1,933 | 2,922 | 0,432 | (2,026; 3,817) | -0,989 | -0,88 | -0,87 | 0,127732 |
| 13 | 1,739 | 1,980 | 0,538 | (0,865; 3,095) | -0,241 | -0,22 | -0,22 | 0,198178 |
| 14 | 5,625 | 4,594 | 0,538 | (3,479; 5,710) | 1,030 | 0,95 | 0,95 | 0,198178 |
| 15 | 2,218 | 2,068 | 0,413 | (1,211; 2,924) | 0,150 | 0,13 | 0,13 | 0,116856 |
| 16 | 4,946 | 4,148 | 0,413 | (3,292; 5,004) | 0,798 | 0,70 | 0,69 | 0,116856 |
| 17 | 3,375 | 3,234 | 0,915 | (1,337; 5,132) | 0,141 | 0,18 | 0,17 | 0,573726 |
| 18 | 6,437 | 6,369 | 0,915 | (4,471; 8,266) | 0,069 | 0,09 | 0,09 | 0,573726 |
| 19 | 1,280 | 2,299 | 0,313 | (1,650; 2,948) | -1,018 | -0,87 | -0,87 | 0,067140 |
| 20 | 3,821 | 2,224 | 0,313 | (1,576; 2,873) | 1,597 | 1,37 | 1,40 | 0,066994 |
| 21 | 2,214 | 1,595 | 0,528 | (0,500; 2,691) | 0,618 | 0,57 | 0,56 | 0,191276 |
| 22 | 2,176 | 3,115 | 0,633 | (1,801; 4,428) | -0,939 | -0,91 | -0,91 | 0,274906 |
| 23 | 1,516 | 2,299 | 0,313 | (1,650; 2,948) | -0,783 | -0,67 | -0,66 | 0,067140 |
| 24 | 1,817 | 2,224 | 0,313 | (1,576; 2,873) | -0,408 | -0,35 | -0,34 | 0,066994 |
| 25 | 0,679 | 2,314 | 0,314 | (1,663; 2,965) | -1,635 | -1,40 | -1,43 | 0,067551 |
| 26 | 5,109 | 2,314 | 0,314 | (1,663; 2,965) | 2,795 | 2,40 | 2,72 | 0,067551 |
| 27 | 2,715 | 2,314 | 0,314 | (1,663; 2,965) | 0,401 | 0,34 | 0,34 | 0,067551 |

| Obs | Cook’s D | DFITS |  |  |
| --- | --- | --- | --- | --- |
| 1 | 0,05 | 0,51982 |  |  |
| 2 | 0,04 | 0,43556 |  |  |
| 3 | 0,02 | -0,31730 |  |  |
| 4 | 0,11 | -0,77351 |  |  |
| 5 | 0,01 | 0,26994 |  |  |
| 6 | 0,05 | 0,51124 |  |  |
| 7 | 0,00 | 0,06728 |  |  |
| 8 | 0,22 | -1,13155 |  |  |
| 9 | 0,02 | -0,34626 |  |  |
| 10 | 0,00 | -0,03321 |  |  |
| 11 | 0,02 | -0,34696 |  |  |
| 12 | 0,02 | -0,33354 |  |  |
| 13 | 0,00 | -0,10826 |  |  |
| 14 | 0,04 | 0,47243 |  |  |
| 15 | 0,00 | 0,04690 |  |  |
| 16 | 0,01 | 0,25264 |  |  |
| 17 | 0,01 | 0,20241 |  | X |
| 18 | 0,00 | 0,09903 |  | X |
| 19 | 0,01 | -0,23287 |  |  |
| 20 | 0,03 | 0,37465 |  |  |
| 21 | 0,02 | 0,27252 |  |  |
| 22 | 0,06 | -0,56001 |  |  |
| 23 | 0,01 | -0,17766 |  |  |
| 24 | 0,00 | -0,09170 |  |  |
| 25 | 0,03 | -0,38616 |  |  |
| 26 | 0,08 | 0,73290 | R |  |
| 27 | 0,00 | 0,09069 |  |  |

R  Large residual  
X  Unusual X

## Forward Selection of Terms

α to enter = 0,25

## Coded Coefficients

| Term | Coef | SE Coef | 95% CI | T-Value | P-Value | VIF |
| --- | --- | --- | --- | --- | --- | --- |
| Constant | 2,406 | 0,317 | (1,749; 3,063) | 7,59 | 0,000 |  |
| Lac | 1,677 | 0,516 | (0,607; 2,747) | 3,25 | 0,004 | 1,10 |
| HPMC\_HP | 0,804 | 0,487 | (-0,206; 1,814) | 1,65 | 0,113 | 1,00 |
| Lac\*Lac | 2,523 | 0,932 | (0,590; 4,456) | 2,71 | 0,013 | 1,00 |
| Lac\*HPMC\_HP | 2,31 | 1,19 | (-0,16; 4,78) | 1,94 | 0,065 | 1,10 |

## Model Summary

| S | R-sq | R-sq(adj) | PRESS | R-sq(pred) | AICc | BIC |
| --- | --- | --- | --- | --- | --- | --- |
| 1,20330 | 49,51% | 40,33% | 43,8493 | 30,50% | 97,29 | 100,86 |

## Analysis of Variance

| Source | DF | Seq SS | Contribution | Adj SS | Adj MS | F-Value | P-Value |
| --- | --- | --- | --- | --- | --- | --- | --- |
| Model | 4 | 31,240 | 49,51% | 31,240 | 7,810 | 5,39 | 0,004 |
| Linear | 2 | 15,173 | 24,05% | 19,252 | 9,626 | 6,65 | 0,006 |
| Lac | 1 | 11,288 | 17,89% | 15,307 | 15,307 | 10,57 | 0,004 |
| HPMC\_HP | 1 | 3,885 | 6,16% | 3,944 | 3,944 | 2,72 | 0,113 |
| Square | 1 | 10,607 | 16,81% | 10,607 | 10,607 | 7,33 | 0,013 |
| Lac\*Lac | 1 | 10,607 | 16,81% | 10,607 | 10,607 | 7,33 | 0,013 |
| 2-Way Interaction | 1 | 5,460 | 8,65% | 5,460 | 5,460 | 3,77 | 0,065 |
| Lac\*HPMC\_HP | 1 | 5,460 | 8,65% | 5,460 | 5,460 | 3,77 | 0,065 |
| Error | 22 | 31,854 | 50,49% | 31,854 | 1,448 |  |  |
| Lack-of-Fit | 20 | 22,216 | 35,21% | 22,216 | 1,111 | 0,23 | 0,973 |
| Pure Error | 2 | 9,638 | 15,28% | 9,638 | 4,819 |  |  |
| Total | 26 | 63,094 | 100,00% |  |  |  |  |

## Regression Equation in Uncoded Units

|  |  |  |
| --- | --- | --- |
| F\_SD\_5.5h(330min) | = | 44,8 - 120,0 Lac - 3,77 HPMC\_HP + 40,4 Lac\*Lac + 9,12 Lac\*HPMC\_HP |

## Fits and Diagnostics for All Observations

| Obs | F\_SD\_5.5h(330min) | Fit | SE Fit | 95% CI | Resid | Std Resid | Del Resid | HI |
| --- | --- | --- | --- | --- | --- | --- | --- | --- |
| 1 | 3,526 | 2,453 | 0,566 | (1,279; 3,627) | 1,073 | 1,01 | 1,01 | 0,221366 |
| 2 | 3,308 | 2,462 | 0,566 | (1,288; 3,636) | 0,846 | 0,80 | 0,79 | 0,221366 |
| 3 | 1,841 | 2,464 | 0,585 | (1,250; 3,677) | -0,623 | -0,59 | -0,58 | 0,236586 |
| 4 | 0,944 | 2,402 | 0,585 | (1,188; 3,615) | -1,457 | -1,39 | -1,42 | 0,236586 |
| 5 | 2,963 | 2,126 | 0,426 | (1,242; 3,009) | 0,838 | 0,74 | 0,74 | 0,125431 |
| 6 | 5,682 | 4,278 | 0,426 | (3,394; 5,161) | 1,405 | 1,25 | 1,27 | 0,125431 |
| 7 | 2,209 | 2,031 | 0,576 | (0,836; 3,225) | 0,178 | 0,17 | 0,16 | 0,229169 |
| 8 | 2,783 | 4,806 | 0,576 | (3,611; 6,000) | -2,023 | -1,91 | -2,05 | 0,229169 |
| 9 | 1,594 | 2,438 | 0,541 | (1,316; 3,561) | -0,845 | -0,79 | -0,78 | 0,202403 |
| 10 | 2,466 | 2,542 | 0,541 | (1,419; 3,664) | -0,075 | -0,07 | -0,07 | 0,202403 |
| 11 | 1,341 | 2,366 | 0,430 | (1,474; 3,258) | -1,025 | -0,91 | -0,91 | 0,127732 |
| 12 | 1,952 | 2,942 | 0,430 | (2,050; 3,834) | -0,990 | -0,88 | -0,88 | 0,127732 |
| 13 | 1,815 | 2,054 | 0,536 | (0,943; 3,165) | -0,239 | -0,22 | -0,22 | 0,198178 |
| 14 | 5,753 | 4,676 | 0,536 | (3,565; 5,787) | 1,077 | 1,00 | 1,00 | 0,198178 |
| 15 | 2,137 | 2,137 | 0,411 | (1,284; 2,990) | -0,000 | -0,00 | -0,00 | 0,116856 |
| 16 | 4,968 | 4,213 | 0,411 | (3,360; 5,066) | 0,755 | 0,67 | 0,66 | 0,116856 |
| 17 | 3,517 | 3,333 | 0,911 | (1,443; 5,224) | 0,183 | 0,23 | 0,23 | 0,573726 |
| 18 | 6,530 | 6,437 | 0,911 | (4,547; 8,327) | 0,093 | 0,12 | 0,12 | 0,573726 |
| 19 | 1,439 | 2,347 | 0,312 | (1,700; 2,993) | -0,908 | -0,78 | -0,77 | 0,067140 |
| 20 | 3,843 | 2,267 | 0,311 | (1,622; 2,913) | 1,575 | 1,36 | 1,38 | 0,066994 |
| 21 | 2,282 | 1,602 | 0,526 | (0,511; 2,694) | 0,680 | 0,63 | 0,62 | 0,191276 |
| 22 | 2,258 | 3,210 | 0,631 | (1,901; 4,518) | -0,952 | -0,93 | -0,93 | 0,274906 |
| 23 | 1,448 | 2,347 | 0,312 | (1,700; 2,993) | -0,899 | -0,77 | -0,77 | 0,067140 |
| 24 | 1,969 | 2,267 | 0,311 | (1,622; 2,913) | -0,299 | -0,26 | -0,25 | 0,066994 |
| 25 | 0,837 | 2,362 | 0,313 | (1,714; 3,011) | -1,525 | -1,31 | -1,34 | 0,067551 |
| 26 | 5,209 | 2,362 | 0,313 | (1,714; 3,011) | 2,846 | 2,45 | 2,81 | 0,067551 |
| 27 | 2,672 | 2,362 | 0,313 | (1,714; 3,011) | 0,309 | 0,27 | 0,26 | 0,067551 |

| Obs | Cook’s D | DFITS |  |  |
| --- | --- | --- | --- | --- |
| 1 | 0,06 | 0,53922 |  |  |
| 2 | 0,04 | 0,42144 |  |  |
| 3 | 0,02 | -0,32471 |  |  |
| 4 | 0,12 | -0,78910 |  |  |
| 5 | 0,02 | 0,27895 |  |  |
| 6 | 0,04 | 0,47921 |  |  |
| 7 | 0,00 | 0,08986 |  |  |
| 8 | 0,22 | -1,11749 |  |  |
| 9 | 0,03 | -0,39240 |  |  |
| 10 | 0,00 | -0,03451 |  |  |
| 11 | 0,02 | -0,34776 |  |  |
| 12 | 0,02 | -0,33533 |  |  |
| 13 | 0,00 | -0,10771 |  |  |
| 14 | 0,05 | 0,49704 |  |  |
| 15 | 0,00 | -0,00011 |  |  |
| 16 | 0,01 | 0,23976 |  |  |
| 17 | 0,01 | 0,26478 |  | X |
| 18 | 0,00 | 0,13403 |  | X |
| 19 | 0,01 | -0,20759 |  |  |
| 20 | 0,03 | 0,37063 |  |  |
| 21 | 0,02 | 0,30119 |  |  |
| 22 | 0,07 | -0,57016 |  |  |
| 23 | 0,01 | -0,20550 |  |  |
| 24 | 0,00 | -0,06742 |  |  |
| 25 | 0,02 | -0,35955 |  |  |
| 26 | 0,09 | 0,75541 | R |  |
| 27 | 0,00 | 0,07013 |  |  |

R  Large residual  
X  Unusual X

## Forward Selection of Terms

α to enter = 0,25

## Coded Coefficients

| Term | Coef | SE Coef | 95% CI | T-Value | P-Value | VIF |
| --- | --- | --- | --- | --- | --- | --- |
| Constant | 2,460 | 0,324 | (1,788; 3,132) | 7,59 | 0,000 |  |
| Lac | 1,666 | 0,527 | (0,572; 2,760) | 3,16 | 0,005 | 1,10 |
| HPMC\_HP | 0,840 | 0,498 | (-0,192; 1,873) | 1,69 | 0,106 | 1,00 |
| Lac\*Lac | 2,555 | 0,953 | (0,578; 4,531) | 2,68 | 0,014 | 1,00 |
| Lac\*HPMC\_HP | 2,48 | 1,22 | (-0,05; 5,01) | 2,04 | 0,054 | 1,10 |

## Model Summary

| S | R-sq | R-sq(adj) | PRESS | R-sq(pred) | AICc | BIC |
| --- | --- | --- | --- | --- | --- | --- |
| 1,23039 | 49,11% | 39,86% | 46,1478 | 29,49% | 98,49 | 102,06 |

## Analysis of Variance

| Source | DF | Seq SS | Contribution | Adj SS | Adj MS | F-Value | P-Value |
| --- | --- | --- | --- | --- | --- | --- | --- |
| Model | 4 | 32,146 | 49,11% | 32,146 | 8,037 | 5,31 | 0,004 |
| Linear | 2 | 14,995 | 22,91% | 19,412 | 9,706 | 6,41 | 0,006 |
| Lac | 1 | 10,743 | 16,41% | 15,099 | 15,099 | 9,97 | 0,005 |
| HPMC\_HP | 1 | 4,251 | 6,50% | 4,314 | 4,314 | 2,85 | 0,106 |
| Square | 1 | 10,877 | 16,62% | 10,877 | 10,877 | 7,18 | 0,014 |
| Lac\*Lac | 1 | 10,877 | 16,62% | 10,877 | 10,877 | 7,18 | 0,014 |
| 2-Way Interaction | 1 | 6,275 | 9,59% | 6,275 | 6,275 | 4,14 | 0,054 |
| Lac\*HPMC\_HP | 1 | 6,275 | 9,59% | 6,275 | 6,275 | 4,14 | 0,054 |
| Error | 22 | 33,305 | 50,89% | 33,305 | 1,514 |  |  |
| Lack-of-Fit | 20 | 23,478 | 35,87% | 23,478 | 1,174 | 0,24 | 0,970 |
| Pure Error | 2 | 9,827 | 15,01% | 9,827 | 4,913 |  |  |
| Total | 26 | 65,451 | 100,00% |  |  |  |  |

## Regression Equation in Uncoded Units

|  |  |  |
| --- | --- | --- |
| F\_SD\_6h(360min) | = | 47,8 - 126,8 Lac - 4,06 HPMC\_HP + 40,9 Lac\*Lac + 9,77 Lac\*HPMC\_HP |

## Fits and Diagnostics for All Observations

| Obs | F\_SD\_6h(360min) | Fit | SE Fit | 95% CI | Resid | Std Resid | Del Resid | HI |
| --- | --- | --- | --- | --- | --- | --- | --- | --- |
| 1 | 3,951 | 2,554 | 0,579 | (1,353; 3,754) | 1,397 | 1,29 | 1,31 | 0,221366 |
| 2 | 3,293 | 2,431 | 0,579 | (1,231; 3,632) | 0,861 | 0,79 | 0,79 | 0,221366 |
| 3 | 1,837 | 2,566 | 0,598 | (1,325; 3,807) | -0,729 | -0,68 | -0,67 | 0,236586 |
| 4 | 0,881 | 2,367 | 0,598 | (1,126; 3,609) | -1,486 | -1,38 | -1,41 | 0,236586 |
| 5 | 3,030 | 2,184 | 0,436 | (1,280; 3,087) | 0,846 | 0,74 | 0,73 | 0,125431 |
| 6 | 5,644 | 4,358 | 0,436 | (3,455; 5,262) | 1,286 | 1,12 | 1,12 | 0,125431 |
| 7 | 2,313 | 2,076 | 0,589 | (0,855; 3,298) | 0,237 | 0,22 | 0,21 | 0,229169 |
| 8 | 2,883 | 4,919 | 0,589 | (3,697; 6,140) | -2,036 | -1,88 | -2,01 | 0,229169 |
| 9 | 1,548 | 2,538 | 0,554 | (1,390; 3,685) | -0,990 | -0,90 | -0,90 | 0,202403 |
| 10 | 2,425 | 2,516 | 0,554 | (1,368; 3,664) | -0,091 | -0,08 | -0,08 | 0,202403 |
| 11 | 1,405 | 2,456 | 0,440 | (1,544; 3,368) | -1,051 | -0,91 | -0,91 | 0,127732 |
| 12 | 1,960 | 2,941 | 0,440 | (2,029; 3,853) | -0,981 | -0,85 | -0,85 | 0,127732 |
| 13 | 1,914 | 2,103 | 0,548 | (0,967; 3,238) | -0,189 | -0,17 | -0,17 | 0,198178 |
| 14 | 5,889 | 4,781 | 0,548 | (3,645; 5,917) | 1,108 | 1,01 | 1,01 | 0,198178 |
| 15 | 2,064 | 2,197 | 0,421 | (1,325; 3,069) | -0,133 | -0,12 | -0,11 | 0,116856 |
| 16 | 5,014 | 4,290 | 0,421 | (3,418; 5,162) | 0,724 | 0,63 | 0,62 | 0,116856 |
| 17 | 3,590 | 3,438 | 0,932 | (1,505; 5,370) | 0,152 | 0,19 | 0,19 | 0,573726 |
| 18 | 6,655 | 6,500 | 0,932 | (4,568; 8,433) | 0,154 | 0,19 | 0,19 | 0,573726 |
| 19 | 1,519 | 2,398 | 0,319 | (1,737; 3,059) | -0,879 | -0,74 | -0,73 | 0,067140 |
| 20 | 3,915 | 2,315 | 0,318 | (1,655; 2,975) | 1,600 | 1,35 | 1,37 | 0,066994 |
| 21 | 2,319 | 1,619 | 0,538 | (0,503; 2,735) | 0,699 | 0,63 | 0,62 | 0,191276 |
| 22 | 2,398 | 3,300 | 0,645 | (1,963; 4,638) | -0,902 | -0,86 | -0,86 | 0,274906 |
| 23 | 1,418 | 2,398 | 0,319 | (1,737; 3,059) | -0,980 | -0,82 | -0,82 | 0,067140 |
| 24 | 2,052 | 2,315 | 0,318 | (1,655; 2,975) | -0,263 | -0,22 | -0,22 | 0,066994 |
| 25 | 0,961 | 2,414 | 0,320 | (1,751; 3,078) | -1,454 | -1,22 | -1,24 | 0,067551 |
| 26 | 5,345 | 2,414 | 0,320 | (1,751; 3,078) | 2,930 | 2,47 | 2,83 | 0,067551 |
| 27 | 2,583 | 2,414 | 0,320 | (1,751; 3,078) | 0,168 | 0,14 | 0,14 | 0,067551 |

| Obs | Cook’s D | DFITS |  |  |
| --- | --- | --- | --- | --- |
| 1 | 0,09 | 0,69710 |  |  |
| 2 | 0,04 | 0,41929 |  |  |
| 3 | 0,03 | -0,37288 |  |  |
| 4 | 0,12 | -0,78703 |  |  |
| 5 | 0,02 | 0,27557 |  |  |
| 6 | 0,04 | 0,42572 |  |  |
| 7 | 0,00 | 0,11679 |  |  |
| 8 | 0,21 | -1,09637 |  |  |
| 9 | 0,04 | -0,45165 |  |  |
| 10 | 0,00 | -0,04082 |  |  |
| 11 | 0,02 | -0,34859 |  |  |
| 12 | 0,02 | -0,32456 |  |  |
| 13 | 0,00 | -0,08327 |  |  |
| 14 | 0,05 | 0,50005 |  |  |
| 15 | 0,00 | -0,04094 |  |  |
| 16 | 0,01 | 0,22461 |  |  |
| 17 | 0,01 | 0,21529 |  | X |
| 18 | 0,01 | 0,21783 |  | X |
| 19 | 0,01 | -0,19633 |  |  |
| 20 | 0,03 | 0,36788 |  |  |
| 21 | 0,02 | 0,30298 |  |  |
| 22 | 0,06 | -0,52703 |  |  |
| 23 | 0,01 | -0,21957 |  |  |
| 24 | 0,00 | -0,05790 |  |  |
| 25 | 0,02 | -0,33332 |  |  |
| 26 | 0,09 | 0,76253 | R |  |
| 27 | 0,00 | 0,03730 |  |  |

R  Large residual  
X  Unusual X

## Forward Selection of Terms

α to enter = 0,25

## Coded Coefficients

| Term | Coef | SE Coef | 95% CI | T-Value | P-Value | VIF |
| --- | --- | --- | --- | --- | --- | --- |
| Constant | 2,534 | 0,338 | (1,833; 3,235) | 7,49 | 0,000 |  |
| Lac | 1,634 | 0,551 | (0,492; 2,776) | 2,97 | 0,007 | 1,10 |
| HPMC\_HP | 0,879 | 0,520 | (-0,198; 1,957) | 1,69 | 0,105 | 1,00 |
| Lac\*Lac | 2,645 | 0,995 | (0,582; 4,708) | 2,66 | 0,014 | 1,00 |
| Lac\*HPMC\_HP | 2,69 | 1,27 | (0,06; 5,33) | 2,12 | 0,046 | 1,10 |

## Model Summary

| S | R-sq | R-sq(adj) | PRESS | R-sq(pred) | AICc | BIC |
| --- | --- | --- | --- | --- | --- | --- |
| 1,28428 | 48,02% | 38,57% | 50,9054 | 27,08% | 100,80 | 104,38 |

## Analysis of Variance

| Source | DF | Seq SS | Contribution | Adj SS | Adj MS | F-Value | P-Value |
| --- | --- | --- | --- | --- | --- | --- | --- |
| Model | 4 | 33,520 | 48,02% | 33,520 | 8,380 | 5,08 | 0,005 |
| Linear | 2 | 14,460 | 20,71% | 19,253 | 9,627 | 5,84 | 0,009 |
| Lac | 1 | 9,805 | 14,05% | 14,530 | 14,530 | 8,81 | 0,007 |
| HPMC\_HP | 1 | 4,655 | 6,67% | 4,723 | 4,723 | 2,86 | 0,105 |
| Square | 1 | 11,661 | 16,70% | 11,661 | 11,661 | 7,07 | 0,014 |
| Lac\*Lac | 1 | 11,661 | 16,70% | 11,661 | 11,661 | 7,07 | 0,014 |
| 2-Way Interaction | 1 | 7,399 | 10,60% | 7,399 | 7,399 | 4,49 | 0,046 |
| Lac\*HPMC\_HP | 1 | 7,399 | 10,60% | 7,399 | 7,399 | 4,49 | 0,046 |
| Error | 22 | 36,287 | 51,98% | 36,287 | 1,649 |  |  |
| Lack-of-Fit | 20 | 26,283 | 37,65% | 26,283 | 1,314 | 0,26 | 0,960 |
| Pure Error | 2 | 10,003 | 14,33% | 10,003 | 5,002 |  |  |
| Total | 26 | 69,806 | 100,00% |  |  |  |  |

## Regression Equation in Uncoded Units

|  |  |  |
| --- | --- | --- |
| F\_SD\_7h(420min) | = | 51,9 - 136,4 Lac - 4,44 HPMC\_HP + 42,3 Lac\*Lac + 10,61 Lac\*HPMC\_HP |

## Fits and Diagnostics for All Observations

| Obs | F\_SD\_7h(420min) | Fit | SE Fit | 95% CI | Resid | Std Resid | Del Resid | HI |
| --- | --- | --- | --- | --- | --- | --- | --- | --- |
| 1 | 4,518 | 2,715 | 0,604 | (1,462; 3,968) | 1,803 | 1,59 | 1,65 | 0,221366 |
| 2 | 3,309 | 2,407 | 0,604 | (1,154; 3,660) | 0,901 | 0,80 | 0,79 | 0,221366 |
| 3 | 1,805 | 2,729 | 0,625 | (1,434; 4,025) | -0,925 | -0,82 | -0,82 | 0,236586 |
| 4 | 0,785 | 2,339 | 0,625 | (1,043; 3,634) | -1,553 | -1,38 | -1,42 | 0,236586 |
| 5 | 3,156 | 2,282 | 0,455 | (1,339; 3,226) | 0,873 | 0,73 | 0,72 | 0,125431 |
| 6 | 5,532 | 4,469 | 0,455 | (3,526; 5,412) | 1,063 | 0,89 | 0,88 | 0,125431 |
| 7 | 2,492 | 2,156 | 0,615 | (0,881; 3,432) | 0,335 | 0,30 | 0,29 | 0,229169 |
| 8 | 3,044 | 5,069 | 0,615 | (3,794; 6,344) | -2,024 | -1,80 | -1,90 | 0,229169 |
| 9 | 1,507 | 2,696 | 0,578 | (1,498; 3,894) | -1,189 | -1,04 | -1,04 | 0,202403 |
| 10 | 2,345 | 2,498 | 0,578 | (1,300; 3,696) | -0,153 | -0,13 | -0,13 | 0,202403 |
| 11 | 1,564 | 2,600 | 0,459 | (1,649; 3,552) | -1,036 | -0,86 | -0,86 | 0,127732 |
| 12 | 2,022 | 2,953 | 0,459 | (2,001; 3,905) | -0,931 | -0,78 | -0,77 | 0,127732 |
| 13 | 2,157 | 2,187 | 0,572 | (1,002; 3,373) | -0,030 | -0,03 | -0,03 | 0,198178 |
| 14 | 6,144 | 4,921 | 0,572 | (3,736; 6,107) | 1,223 | 1,06 | 1,07 | 0,198178 |
| 15 | 1,815 | 2,298 | 0,439 | (1,387; 3,208) | -0,483 | -0,40 | -0,39 | 0,116856 |
| 16 | 5,112 | 4,396 | 0,439 | (3,486; 5,307) | 0,716 | 0,59 | 0,58 | 0,116856 |
| 17 | 3,792 | 3,643 | 0,973 | (1,626; 5,661) | 0,149 | 0,18 | 0,17 | 0,573726 |
| 18 | 6,822 | 6,620 | 0,973 | (4,602; 8,637) | 0,203 | 0,24 | 0,24 | 0,573726 |
| 19 | 1,585 | 2,469 | 0,333 | (1,779; 3,159) | -0,884 | -0,71 | -0,70 | 0,067140 |
| 20 | 3,927 | 2,382 | 0,332 | (1,693; 3,072) | 1,545 | 1,25 | 1,26 | 0,066994 |
| 21 | 2,408 | 1,654 | 0,562 | (0,490; 2,819) | 0,753 | 0,65 | 0,64 | 0,191276 |
| 22 | 2,493 | 3,413 | 0,673 | (2,017; 4,810) | -0,920 | -0,84 | -0,84 | 0,274906 |
| 23 | 1,290 | 2,469 | 0,333 | (1,779; 3,159) | -1,179 | -0,95 | -0,95 | 0,067140 |
| 24 | 2,248 | 2,382 | 0,332 | (1,693; 3,072) | -0,134 | -0,11 | -0,11 | 0,066994 |
| 25 | 1,261 | 2,486 | 0,334 | (1,794; 3,179) | -1,225 | -0,99 | -0,99 | 0,067551 |
| 26 | 5,597 | 2,486 | 0,334 | (1,794; 3,179) | 3,110 | 2,51 | 2,90 | 0,067551 |
| 27 | 2,476 | 2,486 | 0,334 | (1,794; 3,179) | -0,010 | -0,01 | -0,01 | 0,067551 |

| Obs | Cook’s D | DFITS |  |  |
| --- | --- | --- | --- | --- |
| 1 | 0,14 | 0,88122 |  |  |
| 2 | 0,04 | 0,42046 |  |  |
| 3 | 0,04 | -0,45529 |  |  |
| 4 | 0,12 | -0,78803 |  |  |
| 5 | 0,02 | 0,27235 |  |  |
| 6 | 0,02 | 0,33353 |  |  |
| 7 | 0,01 | 0,15875 |  |  |
| 8 | 0,19 | -1,03530 |  |  |
| 9 | 0,05 | -0,52301 |  |  |
| 10 | 0,00 | -0,06553 |  |  |
| 11 | 0,02 | -0,32855 |  |  |
| 12 | 0,02 | -0,29427 |  |  |
| 13 | 0,00 | -0,01271 |  |  |
| 14 | 0,06 | 0,53048 |  |  |
| 15 | 0,00 | -0,14272 |  |  |
| 16 | 0,01 | 0,21260 |  |  |
| 17 | 0,01 | 0,20188 |  | X |
| 18 | 0,02 | 0,27443 |  | X |
| 19 | 0,01 | -0,18906 |  |  |
| 20 | 0,02 | 0,33822 |  |  |
| 21 | 0,02 | 0,31290 |  |  |
| 22 | 0,05 | -0,51439 |  |  |
| 23 | 0,01 | -0,25447 |  |  |
| 24 | 0,00 | -0,02826 |  |  |
| 25 | 0,01 | -0,26574 |  |  |
| 26 | 0,09 | 0,78048 | R |  |
| 27 | 0,00 | -0,00214 |  |  |

R  Large residual  
X  Unusual X

## Forward Selection of Terms

α to enter = 0,25

## Coded Coefficients

| Term | Coef | SE Coef | 95% CI | T-Value | P-Value | VIF |
| --- | --- | --- | --- | --- | --- | --- |
| Constant | 2,603 | 0,349 | (1,880; 3,326) | 7,46 | 0,000 |  |
| Lac | 1,508 | 0,568 | (0,331; 2,685) | 2,66 | 0,014 | 1,10 |
| HPMC\_HP | 1,035 | 0,536 | (-0,077; 2,146) | 1,93 | 0,067 | 1,00 |
| Lac\*Lac | 2,74 | 1,03 | (0,61; 4,86) | 2,67 | 0,014 | 1,00 |
| Lac\*HPMC\_HP | 2,56 | 1,31 | (-0,16; 5,28) | 1,95 | 0,063 | 1,10 |

## Model Summary

| S | R-sq | R-sq(adj) | PRESS | R-sq(pred) | AICc | BIC |
| --- | --- | --- | --- | --- | --- | --- |
| 1,32410 | 46,72% | 37,04% | 53,9068 | 25,54% | 102,45 | 106,03 |

## Analysis of Variance

| Source | DF | Seq SS | Contribution | Adj SS | Adj MS | F-Value | P-Value |
| --- | --- | --- | --- | --- | --- | --- | --- |
| Model | 4 | 33,827 | 46,72% | 33,827 | 8,457 | 4,82 | 0,006 |
| Linear | 2 | 14,658 | 20,25% | 18,908 | 9,454 | 5,39 | 0,012 |
| Lac | 1 | 8,205 | 11,33% | 12,372 | 12,372 | 7,06 | 0,014 |
| HPMC\_HP | 1 | 6,453 | 8,91% | 6,536 | 6,536 | 3,73 | 0,067 |
| Square | 1 | 12,472 | 17,23% | 12,472 | 12,472 | 7,11 | 0,014 |
| Lac\*Lac | 1 | 12,472 | 17,23% | 12,472 | 12,472 | 7,11 | 0,014 |
| 2-Way Interaction | 1 | 6,697 | 9,25% | 6,697 | 6,697 | 3,82 | 0,063 |
| Lac\*HPMC\_HP | 1 | 6,697 | 9,25% | 6,697 | 6,697 | 3,82 | 0,063 |
| Error | 22 | 38,571 | 53,28% | 38,571 | 1,753 |  |  |
| Lack-of-Fit | 20 | 27,296 | 37,70% | 27,296 | 1,365 | 0,24 | 0,968 |
| Pure Error | 2 | 11,275 | 15,57% | 11,275 | 5,638 |  |  |
| Total | 26 | 72,398 | 100,00% |  |  |  |  |

## Regression Equation in Uncoded Units

|  |  |  |
| --- | --- | --- |
| F\_SD\_8h(480min) | = | 48,7 - 133,4 Lac - 4,03 HPMC\_HP + 43,8 Lac\*Lac + 10,10 Lac\*HPMC\_HP |

## Fits and Diagnostics for All Observations

| Obs | F\_SD\_8h(480min) | Fit | SE Fit | 95% CI | Resid | Std Resid | Del Resid | HI |
| --- | --- | --- | --- | --- | --- | --- | --- | --- |
| 1 | 4,534 | 2,710 | 0,623 | (1,418; 4,002) | 1,824 | 1,56 | 1,62 | 0,221366 |
| 2 | 3,293 | 2,371 | 0,623 | (1,079; 3,663) | 0,922 | 0,79 | 0,78 | 0,221366 |
| 3 | 1,772 | 2,718 | 0,644 | (1,382; 4,054) | -0,946 | -0,82 | -0,81 | 0,236586 |
| 4 | 0,718 | 2,300 | 0,644 | (0,964; 3,635) | -1,581 | -1,37 | -1,40 | 0,236586 |
| 5 | 3,381 | 2,482 | 0,469 | (1,509; 3,454) | 0,899 | 0,73 | 0,72 | 0,125431 |
| 6 | 5,531 | 4,516 | 0,469 | (3,543; 5,488) | 1,016 | 0,82 | 0,81 | 0,125431 |
| 7 | 2,936 | 2,416 | 0,634 | (1,101; 3,730) | 0,521 | 0,45 | 0,44 | 0,229169 |
| 8 | 3,155 | 5,139 | 0,634 | (3,825; 6,454) | -1,985 | -1,71 | -1,79 | 0,229169 |
| 9 | 1,536 | 2,700 | 0,596 | (1,465; 3,936) | -1,164 | -0,98 | -0,98 | 0,202403 |
| 10 | 2,237 | 2,465 | 0,596 | (1,230; 3,701) | -0,228 | -0,19 | -0,19 | 0,202403 |
| 11 | 1,582 | 2,650 | 0,473 | (1,668; 3,631) | -1,068 | -0,86 | -0,86 | 0,127732 |
| 12 | 1,985 | 2,938 | 0,473 | (1,957; 3,920) | -0,953 | -0,77 | -0,76 | 0,127732 |
| 13 | 2,425 | 2,432 | 0,589 | (1,209; 3,654) | -0,007 | -0,01 | -0,01 | 0,198178 |
| 14 | 6,267 | 4,986 | 0,589 | (3,763; 6,208) | 1,281 | 1,08 | 1,08 | 0,198178 |
| 15 | 1,849 | 2,490 | 0,453 | (1,551; 3,429) | -0,641 | -0,51 | -0,51 | 0,116856 |
| 16 | 5,074 | 4,440 | 0,453 | (3,501; 5,378) | 0,635 | 0,51 | 0,50 | 0,116856 |
| 17 | 4,020 | 3,913 | 1,003 | (1,833; 5,993) | 0,106 | 0,12 | 0,12 | 0,573726 |
| 18 | 6,914 | 6,651 | 1,003 | (4,571; 8,731) | 0,263 | 0,30 | 0,30 | 0,573726 |
| 19 | 1,594 | 2,526 | 0,343 | (1,815; 3,238) | -0,932 | -0,73 | -0,72 | 0,067140 |
| 20 | 3,879 | 2,424 | 0,343 | (1,713; 3,135) | 1,455 | 1,14 | 1,15 | 0,066994 |
| 21 | 2,471 | 1,568 | 0,579 | (0,367; 2,769) | 0,903 | 0,76 | 0,75 | 0,191276 |
| 22 | 2,708 | 3,637 | 0,694 | (2,197; 5,077) | -0,930 | -0,82 | -0,82 | 0,274906 |
| 23 | 1,183 | 2,526 | 0,343 | (1,815; 3,238) | -1,343 | -1,05 | -1,05 | 0,067140 |
| 24 | 2,472 | 2,424 | 0,343 | (1,713; 3,135) | 0,047 | 0,04 | 0,04 | 0,066994 |
| 25 | 1,361 | 2,547 | 0,344 | (1,833; 3,260) | -1,186 | -0,93 | -0,92 | 0,067551 |
| 26 | 5,868 | 2,547 | 0,344 | (1,833; 3,260) | 3,321 | 2,60 | 3,05 | 0,067551 |
| 27 | 2,319 | 2,547 | 0,344 | (1,833; 3,260) | -0,228 | -0,18 | -0,17 | 0,067551 |

| Obs | Cook’s D | DFITS |  |  |
| --- | --- | --- | --- | --- |
| 1 | 0,14 | 0,862427 |  |  |
| 2 | 0,04 | 0,416925 |  |  |
| 3 | 0,04 | -0,451765 |  |  |
| 4 | 0,12 | -0,777147 |  |  |
| 5 | 0,02 | 0,271911 |  |  |
| 6 | 0,02 | 0,308211 |  |  |
| 7 | 0,01 | 0,239807 |  |  |
| 8 | 0,17 | -0,976374 |  |  |
| 9 | 0,05 | -0,495693 |  |  |
| 10 | 0,00 | -0,095064 |  |  |
| 11 | 0,02 | -0,328491 |  |  |
| 12 | 0,02 | -0,292118 |  |  |
| 13 | 0,00 | -0,002759 |  |  |
| 14 | 0,06 | 0,539126 |  |  |
| 15 | 0,01 | -0,184081 |  |  |
| 16 | 0,01 | 0,182322 |  |  |
| 17 | 0,00 | 0,139606 |  | X |
| 18 | 0,02 | 0,345177 |  | X |
| 19 | 0,01 | -0,193351 |  |  |
| 20 | 0,02 | 0,306892 |  |  |
| 21 | 0,03 | 0,365210 |  |  |
| 22 | 0,05 | -0,503834 |  |  |
| 23 | 0,02 | -0,282489 |  |  |
| 24 | 0,00 | 0,009682 |  |  |
| 25 | 0,01 | -0,248828 |  |  |
| 26 | 0,10 | 0,820307 | R |  |
| 27 | 0,00 | -0,046885 |  |  |

R  Large residual  
X  Unusual X

## Forward Selection of Terms

α to enter = 0,25

## Coded Coefficients

| Term | Coef | SE Coef | 95% CI | T-Value | P-Value | VIF |
| --- | --- | --- | --- | --- | --- | --- |
| Constant | 2,563 | 0,344 | (1,850; 3,277) | 7,45 | 0,000 |  |
| Lac | 1,570 | 0,560 | (0,408; 2,731) | 2,80 | 0,010 | 1,10 |
| HPMC\_HP | 1,205 | 0,529 | (0,109; 2,302) | 2,28 | 0,033 | 1,00 |
| Lac\*Lac | 3,13 | 1,01 | (1,04; 5,23) | 3,10 | 0,005 | 1,00 |
| Lac\*HPMC\_HP | 2,34 | 1,29 | (-0,34; 5,03) | 1,81 | 0,084 | 1,10 |

## Model Summary

| S | R-sq | R-sq(adj) | PRESS | R-sq(pred) | AICc | BIC |
| --- | --- | --- | --- | --- | --- | --- |
| 1,30619 | 51,76% | 42,98% | 52,4918 | 32,53% | 101,72 | 105,29 |

## Analysis of Variance

| Source | DF | Seq SS | Contribution | Adj SS | Adj MS | F-Value | P-Value |
| --- | --- | --- | --- | --- | --- | --- | --- |
| Model | 4 | 40,267 | 51,76% | 40,267 | 10,067 | 5,90 | 0,002 |
| Linear | 2 | 18,290 | 23,51% | 22,281 | 11,140 | 6,53 | 0,006 |
| Lac | 1 | 9,527 | 12,24% | 13,407 | 13,407 | 7,86 | 0,010 |
| HPMC\_HP | 1 | 8,763 | 11,26% | 8,874 | 8,874 | 5,20 | 0,033 |
| Square | 1 | 16,373 | 21,05% | 16,373 | 16,373 | 9,60 | 0,005 |
| Lac\*Lac | 1 | 16,373 | 21,05% | 16,373 | 16,373 | 9,60 | 0,005 |
| 2-Way Interaction | 1 | 5,604 | 7,20% | 5,604 | 5,604 | 3,28 | 0,084 |
| Lac\*HPMC\_HP | 1 | 5,604 | 7,20% | 5,604 | 5,604 | 3,28 | 0,084 |
| Error | 22 | 37,535 | 48,24% | 37,535 | 1,706 |  |  |
| Lack-of-Fit | 20 | 25,836 | 33,21% | 25,836 | 1,292 | 0,22 | 0,976 |
| Pure Error | 2 | 11,699 | 15,04% | 11,699 | 5,849 |  |  |
| Total | 26 | 77,802 | 100,00% |  |  |  |  |

## Regression Equation in Uncoded Units

|  |  |  |
| --- | --- | --- |
| F\_SD\_9h(540min) | = | 44,5 - 131,4 Lac - 3,43 HPMC\_HP + 50,1 Lac\*Lac + 9,24 Lac\*HPMC\_HP |

## Fits and Diagnostics for All Observations

| Obs | F\_SD\_9h(540min) | Fit | SE Fit | 95% CI | Resid | Std Resid | Del Resid | HI |
| --- | --- | --- | --- | --- | --- | --- | --- | --- |
| 1 | 3,660 | 2,538 | 0,615 | (1,264; 3,813) | 1,122 | 0,97 | 0,97 | 0,221366 |
| 2 | 3,337 | 2,418 | 0,615 | (1,143; 3,692) | 0,919 | 0,80 | 0,79 | 0,221366 |
| 3 | 1,761 | 2,537 | 0,635 | (1,219; 3,855) | -0,776 | -0,68 | -0,67 | 0,236586 |
| 4 | 0,705 | 2,345 | 0,635 | (1,027; 3,662) | -1,640 | -1,44 | -1,48 | 0,236586 |
| 5 | 3,461 | 2,569 | 0,463 | (1,610; 3,529) | 0,892 | 0,73 | 0,72 | 0,125431 |
| 6 | 5,484 | 4,620 | 0,463 | (3,660; 5,579) | 0,864 | 0,71 | 0,70 | 0,125431 |
| 7 | 2,825 | 2,578 | 0,625 | (1,281; 3,875) | 0,247 | 0,22 | 0,21 | 0,229169 |
| 8 | 3,231 | 5,260 | 0,625 | (3,963; 6,557) | -2,029 | -1,77 | -1,87 | 0,229169 |
| 9 | 1,587 | 2,539 | 0,588 | (1,321; 3,758) | -0,953 | -0,82 | -0,81 | 0,202403 |
| 10 | 2,141 | 2,515 | 0,588 | (1,296; 3,733) | -0,374 | -0,32 | -0,31 | 0,202403 |
| 11 | 1,635 | 2,546 | 0,467 | (1,578; 3,514) | -0,912 | -0,75 | -0,74 | 0,127732 |
| 12 | 1,858 | 3,001 | 0,467 | (2,032; 3,969) | -1,142 | -0,94 | -0,93 | 0,127732 |
| 13 | 2,777 | 2,576 | 0,581 | (1,370; 3,782) | 0,201 | 0,17 | 0,17 | 0,198178 |
| 14 | 6,394 | 5,103 | 0,581 | (3,897; 6,309) | 1,291 | 1,10 | 1,11 | 0,198178 |
| 15 | 1,943 | 2,568 | 0,447 | (1,642; 3,494) | -0,625 | -0,51 | -0,50 | 0,116856 |
| 16 | 4,944 | 4,542 | 0,447 | (3,616; 5,468) | 0,402 | 0,33 | 0,32 | 0,116856 |
| 17 | 4,278 | 4,190 | 0,989 | (2,138; 6,242) | 0,088 | 0,10 | 0,10 | 0,573726 |
| 18 | 7,616 | 7,075 | 0,989 | (5,023; 9,127) | 0,541 | 0,63 | 0,63 | 0,573726 |
| 19 | 1,521 | 2,474 | 0,338 | (1,773; 3,176) | -0,954 | -0,76 | -0,75 | 0,067140 |
| 20 | 3,769 | 2,356 | 0,338 | (1,655; 3,057) | 1,413 | 1,12 | 1,13 | 0,066994 |
| 21 | 2,593 | 1,358 | 0,571 | (0,173; 2,543) | 1,235 | 1,05 | 1,05 | 0,191276 |
| 22 | 2,951 | 3,769 | 0,685 | (2,349; 5,189) | -0,818 | -0,74 | -0,73 | 0,274906 |
| 23 | 1,019 | 2,474 | 0,338 | (1,773; 3,176) | -1,456 | -1,15 | -1,16 | 0,067140 |
| 24 | 2,552 | 2,356 | 0,338 | (1,655; 3,057) | 0,196 | 0,16 | 0,15 | 0,066994 |
| 25 | 1,502 | 2,498 | 0,339 | (1,794; 3,202) | -0,997 | -0,79 | -0,78 | 0,067551 |
| 26 | 6,014 | 2,498 | 0,339 | (1,794; 3,202) | 3,515 | 2,79 | 3,39 | 0,067551 |
| 27 | 2,248 | 2,498 | 0,339 | (1,794; 3,202) | -0,251 | -0,20 | -0,19 | 0,067551 |

| Obs | Cook’s D | DFITS |  |  |
| --- | --- | --- | --- | --- |
| 1 | 0,05 | 0,51847 |  |  |
| 2 | 0,04 | 0,42137 |  |  |
| 3 | 0,03 | -0,37366 |  |  |
| 4 | 0,13 | -0,82112 |  |  |
| 5 | 0,02 | 0,27346 |  |  |
| 6 | 0,01 | 0,26479 |  |  |
| 7 | 0,00 | 0,11482 |  |  |
| 8 | 0,19 | -1,01799 |  |  |
| 9 | 0,03 | -0,40816 |  |  |
| 10 | 0,01 | -0,15822 |  |  |
| 11 | 0,02 | -0,28304 |  |  |
| 12 | 0,03 | -0,35732 |  |  |
| 13 | 0,00 | 0,08335 |  |  |
| 14 | 0,06 | 0,55162 |  |  |
| 15 | 0,01 | -0,18203 |  |  |
| 16 | 0,00 | 0,11671 |  |  |
| 17 | 0,00 | 0,11652 |  | X |
| 18 | 0,11 | 0,72548 |  | X |
| 19 | 0,01 | -0,20081 |  |  |
| 20 | 0,02 | 0,30202 |  |  |
| 21 | 0,05 | 0,51272 |  |  |
| 22 | 0,04 | -0,44802 |  |  |
| 23 | 0,02 | -0,31207 |  |  |
| 24 | 0,00 | 0,04078 |  |  |
| 25 | 0,01 | -0,21077 |  |  |
| 26 | 0,11 | 0,91125 | R |  |
| 27 | 0,00 | -0,05230 |  |  |

R  Large residual  
X  Unusual X

## Forward Selection of Terms

α to enter = 0,25

## Coded Coefficients

| Term | Coef | SE Coef | 95% CI | T-Value | P-Value | VIF |
| --- | --- | --- | --- | --- | --- | --- |
| Constant | 2,315 | 0,384 | (1,516; 3,114) | 6,03 | 0,000 |  |
| Lac | 1,994 | 0,617 | (0,710; 3,278) | 3,23 | 0,004 | 1,10 |
| HPMC\_Visc | -0,668 | 0,513 | (-1,735; 0,399) | -1,30 | 0,207 | 1,00 |
| HPMC\_HP | 1,251 | 0,583 | (0,038; 2,464) | 2,14 | 0,044 | 1,00 |
| Lac\*Lac | 4,58 | 1,12 | (2,26; 6,90) | 4,10 | 0,001 | 1,00 |
| Lac\*HPMC\_HP | 2,63 | 1,43 | (-0,34; 5,60) | 1,84 | 0,079 | 1,10 |

## Model Summary

| S | R-sq | R-sq(adj) | PRESS | R-sq(pred) | AICc | BIC |
| --- | --- | --- | --- | --- | --- | --- |
| 1,44006 | 61,92% | 52,85% | 78,4683 | 31,39% | 109,43 | 112,60 |

## Analysis of Variance

| Source | DF | Seq SS | Contribution | Adj SS | Adj MS | F-Value | P-Value |
| --- | --- | --- | --- | --- | --- | --- | --- |
| Model | 5 | 70,815 | 61,92% | 70,815 | 14,163 | 6,83 | 0,001 |
| Linear | 3 | 28,861 | 25,24% | 34,197 | 11,399 | 5,50 | 0,006 |
| Lac | 1 | 16,269 | 14,23% | 21,637 | 21,637 | 10,43 | 0,004 |
| HPMC\_Visc | 1 | 3,213 | 2,81% | 3,516 | 3,516 | 1,70 | 0,207 |
| HPMC\_HP | 1 | 9,379 | 8,20% | 9,532 | 9,532 | 4,60 | 0,044 |
| Square | 1 | 34,898 | 30,52% | 34,898 | 34,898 | 16,83 | 0,001 |
| Lac\*Lac | 1 | 34,898 | 30,52% | 34,898 | 34,898 | 16,83 | 0,001 |
| 2-Way Interaction | 1 | 7,056 | 6,17% | 7,056 | 7,056 | 3,40 | 0,079 |
| Lac\*HPMC\_HP | 1 | 7,056 | 6,17% | 7,056 | 7,056 | 3,40 | 0,079 |
| Error | 21 | 43,549 | 38,08% | 43,549 | 2,074 |  |  |
| Lack-of-Fit | 19 | 30,823 | 26,95% | 30,823 | 1,622 | 0,25 | 0,963 |
| Pure Error | 2 | 12,726 | 11,13% | 12,726 | 6,363 |  |  |
| Total | 26 | 114,365 | 100,00% |  |  |  |  |

## Regression Equation in Uncoded Units

|  |  |  |
| --- | --- | --- |
| F\_SD\_10h(600min) | = | 56,4 - 163,4 Lac - 0,000172 HPMC\_Visc - 3,95 HPMC\_HP + 73,2 Lac\*Lac + 10,36 Lac\*HPMC\_HP |

## Fits and Diagnostics for All Observations

| Obs | F\_SD\_10h(600min) | Fit | SE Fit | 95% CI | Resid | Std Resid | Del Resid |
| --- | --- | --- | --- | --- | --- | --- | --- |
| 1 | 4,249 | 2,985 | 0,734 | (1,458; 4,511) | 1,264 | 1,02 | 1,02 |
| 2 | 3,393 | 3,082 | 0,734 | (1,556; 4,609) | 0,311 | 0,25 | 0,25 |
| 3 | 1,732 | 2,050 | 0,826 | (0,333; 3,767) | -0,319 | -0,27 | -0,26 |
| 4 | 0,695 | 2,067 | 0,826 | (0,350; 3,784) | -1,372 | -1,16 | -1,17 |
| 5 | 3,531 | 2,883 | 0,579 | (1,678; 4,088) | 0,648 | 0,49 | 0,48 |
| 6 | 5,396 | 5,417 | 0,579 | (4,212; 6,621) | -0,021 | -0,02 | -0,02 |
| 7 | 2,759 | 1,957 | 0,805 | (0,282; 3,632) | 0,801 | 0,67 | 0,66 |
| 8 | 3,229 | 5,199 | 0,805 | (3,525; 6,874) | -1,970 | -1,65 | -1,73 |
| 9 | 1,614 | 2,969 | 0,703 | (1,506; 4,431) | -1,354 | -1,08 | -1,08 |
| 10 | 2,223 | 3,174 | 0,703 | (1,711; 4,636) | -0,951 | -0,76 | -0,75 |
| 11 | 1,592 | 2,366 | 0,543 | (1,236; 3,495) | -0,773 | -0,58 | -0,57 |
| 12 | 1,844 | 3,108 | 0,543 | (1,978; 4,238) | -1,264 | -0,95 | -0,95 |
| 13 | 3,039 | 2,904 | 0,711 | (1,427; 4,382) | 0,134 | 0,11 | 0,10 |
| 14 | 6,554 | 5,972 | 0,711 | (4,495; 7,450) | 0,581 | 0,46 | 0,46 |
| 15 | 1,970 | 2,263 | 0,533 | (1,154; 3,371) | -0,292 | -0,22 | -0,21 |
| 16 | 4,890 | 4,710 | 0,533 | (3,602; 5,819) | 0,179 | 0,13 | 0,13 |
| 17 | 4,493 | 5,098 | 1,091 | (2,829; 7,366) | -0,604 | -0,64 | -0,63 |
| 18 | 10,504 | 8,801 | 1,091 | (6,532; 11,070) | 1,704 | 1,81 | 1,93 |
| 19 | 1,583 | 2,891 | 0,584 | (1,676; 4,106) | -1,308 | -0,99 | -0,99 |
| 20 | 3,593 | 1,432 | 0,689 | (-0,001; 2,864) | 2,161 | 1,71 | 1,80 |
| 21 | 2,686 | 1,185 | 0,630 | (-0,124; 2,495) | 1,500 | 1,16 | 1,17 |
| 22 | 3,052 | 3,643 | 0,755 | (2,072; 5,214) | -0,591 | -0,48 | -0,47 |
| 23 | 0,953 | 2,396 | 0,380 | (1,607; 3,186) | -1,443 | -1,04 | -1,04 |
| 24 | 2,600 | 2,280 | 0,380 | (1,490; 3,070) | 0,319 | 0,23 | 0,22 |
| 25 | 1,480 | 2,373 | 0,376 | (1,592; 3,154) | -0,893 | -0,64 | -0,63 |
| 26 | 6,146 | 2,373 | 0,376 | (1,592; 3,154) | 3,773 | 2,71 | 3,29 |
| 27 | 2,152 | 2,373 | 0,376 | (1,592; 3,154) | -0,221 | -0,16 | -0,16 |

| Obs | HI | Cook’s D | DFITS |  |
| --- | --- | --- | --- | --- |
| 1 | 0,259907 | 0,06 | 0,60542 |  |
| 2 | 0,259907 | 0,00 | 0,14529 |  |
| 3 | 0,328762 | 0,01 | -0,18470 |  |
| 4 | 0,328762 | 0,11 | -0,82121 |  |
| 5 | 0,161779 | 0,01 | 0,21185 |  |
| 6 | 0,161779 | 0,00 | -0,00685 |  |
| 7 | 0,312694 | 0,03 | 0,44671 |  |
| 8 | 0,312694 | 0,21 | -1,16415 |  |
| 9 | 0,238520 | 0,06 | -0,60560 |  |
| 10 | 0,238520 | 0,03 | -0,41903 |  |
| 11 | 0,142357 | 0,01 | -0,23241 |  |
| 12 | 0,142357 | 0,02 | -0,38517 |  |
| 13 | 0,243427 | 0,00 | 0,05941 |  |
| 14 | 0,243427 | 0,01 | 0,25815 |  |
| 15 | 0,136989 | 0,00 | -0,08506 |  |
| 16 | 0,136989 | 0,00 | 0,05215 |  |
| 17 | 0,573996 | 0,09 | -0,73570 |  |
| 18 | 0,573996 | 0,74 | 2,23563 |  |
| 19 | 0,164663 | 0,03 | -0,44118 |  |
| 20 | 0,228727 | 0,14 | 0,97877 |  |
| 21 | 0,191289 | 0,05 | 0,56832 |  |
| 22 | 0,275177 | 0,01 | -0,29163 |  |
| 23 | 0,069490 | 0,01 | -0,28450 |  |
| 24 | 0,069531 | 0,00 | 0,06143 |  |
| 25 | 0,068088 | 0,01 | -0,17111 |  |
| 26 | 0,068088 | 0,09 | 0,88867 | R |
| 27 | 0,068088 | 0,00 | -0,04191 |  |

R  Large residual

## Forward Selection of Terms

α to enter = 0,25

## Coded Coefficients

| Term | Coef | SE Coef | 95% CI | T-Value | P-Value | VIF |
| --- | --- | --- | --- | --- | --- | --- |
| Constant | 2,336 | 0,390 | (1,524; 3,148) | 5,98 | 0,000 |  |
| Lac | 2,092 | 0,627 | (0,788; 3,397) | 3,34 | 0,003 | 1,10 |
| HPMC\_Visc | -0,847 | 0,522 | (-1,932; 0,238) | -1,62 | 0,119 | 1,00 |
| HPMC\_HP | 1,389 | 0,593 | (0,156; 2,622) | 2,34 | 0,029 | 1,00 |
| Lac\*Lac | 4,76 | 1,13 | (2,40; 7,12) | 4,20 | 0,000 | 1,00 |
| Lac\*HPMC\_HP | 2,83 | 1,45 | (-0,18; 5,84) | 1,95 | 0,064 | 1,10 |

## Model Summary

| S | R-sq | R-sq(adj) | PRESS | R-sq(pred) | AICc | BIC |
| --- | --- | --- | --- | --- | --- | --- |
| 1,46359 | 64,12% | 55,58% | 76,6527 | 38,86% | 110,30 | 113,48 |

## Analysis of Variance

| Source | DF | Seq SS | Contribution | Adj SS | Adj MS | F-Value | P-Value |
| --- | --- | --- | --- | --- | --- | --- | --- |
| Model | 5 | 80,396 | 64,12% | 80,396 | 16,079 | 7,51 | 0,000 |
| Linear | 3 | 34,516 | 27,53% | 40,546 | 13,515 | 6,31 | 0,003 |
| Lac | 1 | 17,726 | 14,14% | 23,825 | 23,825 | 11,12 | 0,003 |
| HPMC\_Visc | 1 | 5,206 | 4,15% | 5,650 | 5,650 | 2,64 | 0,119 |
| HPMC\_HP | 1 | 11,584 | 9,24% | 11,761 | 11,761 | 5,49 | 0,029 |
| Square | 1 | 37,718 | 30,08% | 37,718 | 37,718 | 17,61 | 0,000 |
| Lac\*Lac | 1 | 37,718 | 30,08% | 37,718 | 37,718 | 17,61 | 0,000 |
| 2-Way Interaction | 1 | 8,162 | 6,51% | 8,162 | 8,162 | 3,81 | 0,064 |
| Lac\*HPMC\_HP | 1 | 8,162 | 6,51% | 8,162 | 8,162 | 3,81 | 0,064 |
| Error | 21 | 44,984 | 35,88% | 44,984 | 2,142 |  |  |
| Lack-of-Fit | 19 | 31,085 | 24,79% | 31,085 | 1,636 | 0,24 | 0,970 |
| Pure Error | 2 | 13,899 | 11,09% | 13,899 | 6,949 |  |  |
| Total | 26 | 125,379 | 100,00% |  |  |  |  |

## Regression Equation in Uncoded Units

|  |  |  |
| --- | --- | --- |
| F\_SD\_11h(660min) | = | 60,0 - 173,4 Lac - 0,000218 HPMC\_Visc - 4,21 HPMC\_HP + 76,1 Lac\*Lac + 11,15 Lac\*HPMC\_HP |

## Fits and Diagnostics for All Observations

| Obs | F\_SD\_11h(660min) | Fit | SE Fit | 95% CI | Resid | Std Resid | Del Resid |
| --- | --- | --- | --- | --- | --- | --- | --- |
| 1 | 4,211 | 3,100 | 0,746 | (1,548; 4,652) | 1,111 | 0,88 | 0,88 |
| 2 | 3,428 | 3,153 | 0,746 | (1,601; 4,705) | 0,275 | 0,22 | 0,21 |
| 3 | 1,778 | 1,914 | 0,839 | (0,169; 3,659) | -0,136 | -0,11 | -0,11 |
| 4 | 0,714 | 1,880 | 0,839 | (0,135; 3,625) | -1,166 | -0,97 | -0,97 |
| 5 | 3,633 | 3,024 | 0,589 | (1,799; 4,248) | 0,609 | 0,45 | 0,45 |
| 6 | 5,315 | 5,697 | 0,589 | (4,472; 6,921) | -0,382 | -0,28 | -0,28 |
| 7 | 2,585 | 1,865 | 0,818 | (0,163; 3,567) | 0,720 | 0,59 | 0,58 |
| 8 | 3,298 | 5,300 | 0,818 | (3,598; 7,002) | -2,002 | -1,65 | -1,73 |
| 9 | 1,668 | 3,082 | 0,715 | (1,596; 4,569) | -1,414 | -1,11 | -1,11 |
| 10 | 2,396 | 3,251 | 0,715 | (1,764; 4,737) | -0,855 | -0,67 | -0,66 |
| 11 | 1,540 | 2,329 | 0,552 | (1,181; 3,478) | -0,789 | -0,58 | -0,57 |
| 12 | 1,898 | 3,076 | 0,552 | (1,927; 4,224) | -1,177 | -0,87 | -0,86 |
| 13 | 3,308 | 3,062 | 0,722 | (1,561; 4,564) | 0,245 | 0,19 | 0,19 |
| 14 | 7,764 | 6,310 | 0,722 | (4,808; 7,812) | 1,454 | 1,14 | 1,15 |
| 15 | 2,023 | 2,235 | 0,542 | (1,109; 3,362) | -0,212 | -0,16 | -0,15 |
| 16 | 4,812 | 4,816 | 0,542 | (3,689; 5,942) | -0,004 | -0,00 | -0,00 |
| 17 | 4,705 | 5,238 | 1,109 | (2,932; 7,544) | -0,532 | -0,56 | -0,55 |
| 18 | 10,579 | 9,116 | 1,109 | (6,810; 11,422) | 1,463 | 1,53 | 1,59 |
| 19 | 1,719 | 3,080 | 0,594 | (1,845; 4,315) | -1,361 | -1,02 | -1,02 |
| 20 | 3,374 | 1,249 | 0,700 | (-0,206; 2,705) | 2,125 | 1,65 | 1,73 |
| 21 | 2,689 | 1,099 | 0,640 | (-0,232; 2,431) | 1,590 | 1,21 | 1,22 |
| 22 | 3,226 | 3,823 | 0,768 | (2,226; 5,420) | -0,597 | -0,48 | -0,47 |
| 23 | 0,817 | 2,453 | 0,386 | (1,651; 3,255) | -1,636 | -1,16 | -1,17 |
| 24 | 2,549 | 2,325 | 0,386 | (1,522; 3,127) | 0,224 | 0,16 | 0,16 |
| 25 | 1,384 | 2,419 | 0,382 | (1,625; 3,214) | -1,036 | -0,73 | -0,72 |
| 26 | 6,253 | 2,419 | 0,382 | (1,625; 3,214) | 3,834 | 2,71 | 3,29 |
| 27 | 2,068 | 2,419 | 0,382 | (1,625; 3,214) | -0,352 | -0,25 | -0,24 |

| Obs | HI | Cook’s D | DFITS |  |
| --- | --- | --- | --- | --- |
| 1 | 0,259907 | 0,05 | 0,52006 |  |
| 2 | 0,259907 | 0,00 | 0,12645 |  |
| 3 | 0,328762 | 0,00 | -0,07730 |  |
| 4 | 0,328762 | 0,08 | -0,67937 |  |
| 5 | 0,161779 | 0,01 | 0,19589 |  |
| 6 | 0,161779 | 0,00 | -0,12231 |  |
| 7 | 0,312694 | 0,03 | 0,39402 |  |
| 8 | 0,312694 | 0,21 | -1,16407 |  |
| 9 | 0,238520 | 0,06 | -0,62317 |  |
| 10 | 0,238520 | 0,02 | -0,36952 |  |
| 11 | 0,142357 | 0,01 | -0,23335 |  |
| 12 | 0,142357 | 0,02 | -0,35174 |  |
| 13 | 0,243427 | 0,00 | 0,10669 |  |
| 14 | 0,243427 | 0,07 | 0,65307 |  |
| 15 | 0,136989 | 0,00 | -0,06074 |  |
| 16 | 0,136989 | 0,00 | -0,00104 |  |
| 17 | 0,573996 | 0,07 | -0,63606 |  |
| 18 | 0,573996 | 0,53 | 1,84014 |  |
| 19 | 0,164663 | 0,03 | -0,45228 |  |
| 20 | 0,228727 | 0,14 | 0,94197 |  |
| 21 | 0,191289 | 0,06 | 0,59430 |  |
| 22 | 0,275177 | 0,01 | -0,28972 |  |
| 23 | 0,069490 | 0,02 | -0,31938 |  |
| 24 | 0,069531 | 0,00 | 0,04240 |  |
| 25 | 0,068088 | 0,01 | -0,19588 |  |
| 26 | 0,068088 | 0,09 | 0,88817 | R |
| 27 | 0,068088 | 0,00 | -0,06580 |  |

R  Large residual

## Forward Selection of Terms

α to enter = 0,25

## Coded Coefficients

| Term | Coef | SE Coef | 95% CI | T-Value | P-Value | VIF |
| --- | --- | --- | --- | --- | --- | --- |
| Constant | 2,447 | 0,386 | (1,644; 3,249) | 6,34 | 0,000 |  |
| Lac | 1,824 | 0,620 | (0,535; 3,113) | 2,94 | 0,008 | 1,10 |
| HPMC\_Visc | -0,954 | 0,515 | (-2,025; 0,118) | -1,85 | 0,078 | 1,00 |
| HPMC\_HP | 1,412 | 0,586 | (0,194; 2,630) | 2,41 | 0,025 | 1,00 |
| Lac\*Lac | 4,13 | 1,12 | (1,80; 6,46) | 3,69 | 0,001 | 1,00 |
| Lac\*HPMC\_HP | 2,75 | 1,43 | (-0,23; 5,72) | 1,92 | 0,069 | 1,10 |

## Model Summary

| S | R-sq | R-sq(adj) | PRESS | R-sq(pred) | AICc | BIC |
| --- | --- | --- | --- | --- | --- | --- |
| 1,44607 | 60,60% | 51,22% | 66,6498 | 40,21% | 109,65 | 112,83 |

## Analysis of Variance

| Source | DF | Seq SS | Contribution | Adj SS | Adj MS | F-Value | P-Value |
| --- | --- | --- | --- | --- | --- | --- | --- |
| Model | 5 | 67,551 | 60,60% | 67,551 | 13,510 | 6,46 | 0,001 |
| Linear | 3 | 31,414 | 28,18% | 36,620 | 12,207 | 5,84 | 0,005 |
| Lac | 1 | 12,808 | 11,49% | 18,100 | 18,100 | 8,66 | 0,008 |
| HPMC\_Visc | 1 | 6,615 | 5,93% | 7,165 | 7,165 | 3,43 | 0,078 |
| HPMC\_HP | 1 | 11,991 | 10,76% | 12,147 | 12,147 | 5,81 | 0,025 |
| Square | 1 | 28,444 | 25,52% | 28,444 | 28,444 | 13,60 | 0,001 |
| Lac\*Lac | 1 | 28,444 | 25,52% | 28,444 | 28,444 | 13,60 | 0,001 |
| 2-Way Interaction | 1 | 7,693 | 6,90% | 7,693 | 7,693 | 3,68 | 0,069 |
| Lac\*HPMC\_HP | 1 | 7,693 | 6,90% | 7,693 | 7,693 | 3,68 | 0,069 |
| Error | 21 | 43,913 | 39,40% | 43,913 | 2,091 |  |  |
| Lack-of-Fit | 19 | 29,737 | 26,68% | 29,737 | 1,565 | 0,22 | 0,975 |
| Pure Error | 2 | 14,177 | 12,72% | 14,177 | 7,088 |  |  |
| Total | 26 | 111,464 | 100,00% |  |  |  |  |

## Regression Equation in Uncoded Units

|  |  |  |
| --- | --- | --- |
| F\_SD\_12h(720min) | = | 56,7 - 161,4 Lac - 0,000245 HPMC\_Visc - 4,02 HPMC\_HP + 66,1 Lac\*Lac + 10,82 Lac\*HPMC\_HP |

## Fits and Diagnostics for All Observations

| Obs | F\_SD\_12h(720min) | Fit | SE Fit | 95% CI | Resid | Std Resid | Del Resid |
| --- | --- | --- | --- | --- | --- | --- | --- |
| 1 | 4,313 | 3,219 | 0,737 | (1,686; 4,752) | 1,094 | 0,88 | 0,87 |
| 2 | 3,413 | 3,063 | 0,737 | (1,529; 4,596) | 0,350 | 0,28 | 0,28 |
| 3 | 1,834 | 1,881 | 0,829 | (0,157; 3,605) | -0,047 | -0,04 | -0,04 |
| 4 | 0,835 | 1,640 | 0,829 | (-0,084; 3,365) | -0,806 | -0,68 | -0,67 |
| 5 | 3,615 | 3,195 | 0,582 | (1,985; 4,404) | 0,421 | 0,32 | 0,31 |
| 6 | 5,238 | 5,582 | 0,582 | (4,372; 6,792) | -0,344 | -0,26 | -0,25 |
| 7 | 2,464 | 1,908 | 0,809 | (0,227; 3,590) | 0,556 | 0,46 | 0,45 |
| 8 | 3,329 | 5,035 | 0,809 | (3,354; 6,717) | -1,706 | -1,42 | -1,46 |
| 9 | 1,765 | 3,202 | 0,706 | (1,733; 4,670) | -1,436 | -1,14 | -1,15 |
| 10 | 2,623 | 3,157 | 0,706 | (1,689; 4,626) | -0,534 | -0,42 | -0,41 |
| 11 | 1,403 | 2,367 | 0,546 | (1,233; 3,502) | -0,965 | -0,72 | -0,71 |
| 12 | 1,781 | 2,884 | 0,546 | (1,750; 4,019) | -1,103 | -0,82 | -0,82 |
| 13 | 3,630 | 3,252 | 0,713 | (1,768; 4,736) | 0,378 | 0,30 | 0,29 |
| 14 | 8,411 | 6,197 | 0,713 | (4,713; 7,681) | 2,214 | 1,76 | 1,86 |
| 15 | 2,142 | 2,305 | 0,535 | (1,192; 3,418) | -0,163 | -0,12 | -0,12 |
| 16 | 4,555 | 4,602 | 0,535 | (3,489; 5,715) | -0,048 | -0,04 | -0,03 |
| 17 | 4,819 | 5,005 | 1,096 | (2,727; 7,284) | -0,186 | -0,20 | -0,19 |
| 18 | 9,077 | 8,355 | 1,096 | (6,077; 10,634) | 0,721 | 0,76 | 0,76 |
| 19 | 1,700 | 3,296 | 0,587 | (2,076; 4,516) | -1,596 | -1,21 | -1,22 |
| 20 | 3,271 | 1,249 | 0,692 | (-0,189; 2,688) | 2,022 | 1,59 | 1,66 |
| 21 | 2,743 | 1,207 | 0,632 | (-0,108; 2,522) | 1,536 | 1,18 | 1,19 |
| 22 | 3,334 | 3,969 | 0,759 | (2,391; 5,546) | -0,635 | -0,52 | -0,51 |
| 23 | 0,768 | 2,590 | 0,381 | (1,797; 3,383) | -1,821 | -1,31 | -1,33 |
| 24 | 2,505 | 2,461 | 0,381 | (1,668; 3,254) | 0,045 | 0,03 | 0,03 |
| 25 | 1,439 | 2,549 | 0,377 | (1,764; 3,334) | -1,110 | -0,80 | -0,79 |
| 26 | 6,292 | 2,549 | 0,377 | (1,764; 3,334) | 3,743 | 2,68 | 3,23 |
| 27 | 1,968 | 2,549 | 0,377 | (1,764; 3,334) | -0,581 | -0,42 | -0,41 |

| Obs | HI | Cook’s D | DFITS |  |
| --- | --- | --- | --- | --- |
| 1 | 0,259907 | 0,05 | 0,51844 |  |
| 2 | 0,259907 | 0,00 | 0,16303 |  |
| 3 | 0,328762 | 0,00 | -0,02695 |  |
| 4 | 0,328762 | 0,04 | -0,46966 |  |
| 5 | 0,161779 | 0,00 | 0,13651 |  |
| 6 | 0,161779 | 0,00 | -0,11168 |  |
| 7 | 0,312694 | 0,02 | 0,30687 |  |
| 8 | 0,312694 | 0,15 | -0,98569 |  |
| 9 | 0,238520 | 0,07 | -0,64169 |  |
| 10 | 0,238520 | 0,01 | -0,23209 |  |
| 11 | 0,142357 | 0,01 | -0,29001 |  |
| 12 | 0,142357 | 0,02 | -0,33285 |  |
| 13 | 0,243427 | 0,00 | 0,16675 |  |
| 14 | 0,243427 | 0,17 | 1,05533 |  |
| 15 | 0,136989 | 0,00 | -0,04726 |  |
| 16 | 0,136989 | 0,00 | -0,01381 |  |
| 17 | 0,573996 | 0,01 | -0,22403 |  |
| 18 | 0,573996 | 0,13 | 0,87780 |  |
| 19 | 0,164663 | 0,05 | -0,54255 |  |
| 20 | 0,228727 | 0,13 | 0,90244 |  |
| 21 | 0,191289 | 0,06 | 0,58040 |  |
| 22 | 0,275177 | 0,02 | -0,31193 |  |
| 23 | 0,069490 | 0,02 | -0,36329 |  |
| 24 | 0,069531 | 0,00 | 0,00857 |  |
| 25 | 0,068088 | 0,01 | -0,21294 |  |
| 26 | 0,068088 | 0,09 | 0,87230 | R |
| 27 | 0,068088 | 0,00 | -0,11017 |  |

R  Large residual

## Forward Selection of Terms

α to enter = 0,25

## Coded Coefficients

| Term | Coef | SE Coef | 95% CI | T-Value | P-Value | VIF |
| --- | --- | --- | --- | --- | --- | --- |
| Constant | 2,493 | 0,347 | (1,772; 3,214) | 7,19 | 0,000 |  |
| Lac | 1,479 | 0,557 | (0,321; 2,637) | 2,66 | 0,015 | 1,10 |
| HPMC\_Visc | -0,942 | 0,463 | (-1,905; 0,021) | -2,04 | 0,055 | 1,00 |
| HPMC\_HP | 1,311 | 0,526 | (0,217; 2,406) | 2,49 | 0,021 | 1,00 |
| Lac\*Lac | 3,48 | 1,01 | (1,39; 5,58) | 3,46 | 0,002 | 1,00 |
| Lac\*HPMC\_HP | 2,24 | 1,29 | (-0,43; 4,92) | 1,74 | 0,096 | 1,10 |

## Model Summary

| S | R-sq | R-sq(adj) | PRESS | R-sq(pred) | AICc | BIC |
| --- | --- | --- | --- | --- | --- | --- |
| 1,29940 | 58,80% | 48,98% | 50,6920 | 41,09% | 103,87 | 107,05 |

## Analysis of Variance

| Source | DF | Seq SS | Contribution | Adj SS | Adj MS | F-Value | P-Value |
| --- | --- | --- | --- | --- | --- | --- | --- |
| Model | 5 | 50,594 | 58,80% | 50,594 | 10,119 | 5,99 | 0,001 |
| Linear | 3 | 25,217 | 29,30% | 28,650 | 9,550 | 5,66 | 0,005 |
| Lac | 1 | 8,392 | 9,75% | 11,904 | 11,904 | 7,05 | 0,015 |
| HPMC\_Visc | 1 | 6,468 | 7,52% | 6,993 | 6,993 | 4,14 | 0,055 |
| HPMC\_HP | 1 | 10,358 | 12,04% | 10,480 | 10,480 | 6,21 | 0,021 |
| Square | 1 | 20,240 | 23,52% | 20,240 | 20,240 | 11,99 | 0,002 |
| Lac\*Lac | 1 | 20,240 | 23,52% | 20,240 | 20,240 | 11,99 | 0,002 |
| 2-Way Interaction | 1 | 5,137 | 5,97% | 5,137 | 5,137 | 3,04 | 0,096 |
| Lac\*HPMC\_HP | 1 | 5,137 | 5,97% | 5,137 | 5,137 | 3,04 | 0,096 |
| Error | 21 | 35,457 | 41,20% | 35,457 | 1,688 |  |  |
| Lack-of-Fit | 19 | 22,962 | 26,68% | 22,962 | 1,209 | 0,19 | 0,984 |
| Pure Error | 2 | 12,495 | 14,52% | 12,495 | 6,248 |  |  |
| Total | 26 | 86,051 | 100,00% |  |  |  |  |

## Regression Equation in Uncoded Units

|  |  |  |
| --- | --- | --- |
| F\_SD\_13h(780min) | = | 46,4 - 133,6 Lac - 0,000242 HPMC\_Visc - 3,13 HPMC\_HP + 55,8 Lac\*Lac + 8,84 Lac\*HPMC\_HP |

## Fits and Diagnostics for All Observations

| Obs | F\_SD\_13h(780min) | Fit | SE Fit | 95% CI | Resid | Std Resid | Del Resid | HI |
| --- | --- | --- | --- | --- | --- | --- | --- | --- |
| 1 | 4,154 | 3,159 | 0,662 | (1,782; 4,537) | 0,995 | 0,89 | 0,89 | 0,259907 |
| 2 | 3,281 | 3,020 | 0,662 | (1,643; 4,398) | 0,260 | 0,23 | 0,23 | 0,259907 |
| 3 | 1,828 | 1,833 | 0,745 | (0,283; 3,382) | -0,005 | -0,00 | -0,00 | 0,328762 |
| 4 | 1,213 | 1,625 | 0,745 | (0,076; 3,174) | -0,412 | -0,39 | -0,38 | 0,328762 |
| 5 | 3,581 | 3,275 | 0,523 | (2,189; 4,362) | 0,306 | 0,26 | 0,25 | 0,161779 |
| 6 | 5,119 | 5,215 | 0,523 | (4,128; 6,302) | -0,096 | -0,08 | -0,08 | 0,161779 |
| 7 | 2,211 | 2,045 | 0,727 | (0,534; 3,556) | 0,166 | 0,15 | 0,15 | 0,312694 |
| 8 | 3,508 | 4,589 | 0,727 | (3,078; 6,100) | -1,081 | -1,00 | -1,00 | 0,312694 |
| 9 | 1,741 | 3,148 | 0,635 | (1,829; 4,468) | -1,407 | -1,24 | -1,26 | 0,238520 |
| 10 | 3,123 | 3,101 | 0,635 | (1,781; 4,421) | 0,022 | 0,02 | 0,02 | 0,238520 |
| 11 | 1,385 | 2,355 | 0,490 | (1,335; 3,375) | -0,970 | -0,81 | -0,80 | 0,142357 |
| 12 | 1,668 | 2,766 | 0,490 | (1,747; 3,786) | -1,098 | -0,91 | -0,91 | 0,142357 |
| 13 | 3,799 | 3,363 | 0,641 | (2,029; 4,696) | 0,436 | 0,39 | 0,38 | 0,243427 |
| 14 | 7,900 | 5,758 | 0,641 | (4,425; 7,091) | 2,142 | 1,90 | 2,03 | 0,243427 |
| 15 | 2,194 | 2,391 | 0,481 | (1,391; 3,392) | -0,197 | -0,16 | -0,16 | 0,136989 |
| 16 | 4,242 | 4,257 | 0,481 | (3,257; 5,258) | -0,015 | -0,01 | -0,01 | 0,136989 |
| 17 | 4,945 | 4,726 | 0,984 | (2,679; 6,774) | 0,219 | 0,26 | 0,25 | 0,573996 |
| 18 | 7,461 | 7,441 | 0,984 | (5,394; 9,489) | 0,020 | 0,02 | 0,02 | 0,573996 |
| 19 | 1,742 | 3,338 | 0,527 | (2,242; 4,435) | -1,597 | -1,34 | -1,37 | 0,164663 |
| 20 | 3,051 | 1,325 | 0,621 | (0,032; 2,617) | 1,727 | 1,51 | 1,56 | 0,228727 |
| 21 | 2,635 | 1,352 | 0,568 | (0,170; 2,534) | 1,283 | 1,10 | 1,10 | 0,191289 |
| 22 | 3,380 | 3,913 | 0,682 | (2,496; 5,331) | -0,533 | -0,48 | -0,47 | 0,275177 |
| 23 | 0,886 | 2,641 | 0,343 | (1,928; 3,353) | -1,755 | -1,40 | -1,44 | 0,069490 |
| 24 | 2,471 | 2,521 | 0,343 | (1,809; 3,234) | -0,050 | -0,04 | -0,04 | 0,069531 |
| 25 | 1,535 | 2,599 | 0,339 | (1,894; 3,304) | -1,064 | -0,85 | -0,84 | 0,068088 |
| 26 | 6,025 | 2,599 | 0,339 | (1,894; 3,304) | 3,427 | 2,73 | 3,32 | 0,068088 |
| 27 | 1,877 | 2,599 | 0,339 | (1,894; 3,304) | -0,721 | -0,58 | -0,57 | 0,068088 |

| Obs | Cook’s D | DFITS |  |
| --- | --- | --- | --- |
| 1 | 0,05 | 0,52483 |  |
| 2 | 0,00 | 0,13491 |  |
| 3 | 0,00 | -0,00300 |  |
| 4 | 0,01 | -0,26496 |  |
| 5 | 0,00 | 0,11042 |  |
| 6 | 0,00 | -0,03460 |  |
| 7 | 0,00 | 0,10136 |  |
| 8 | 0,08 | -0,67683 |  |
| 9 | 0,08 | -0,70413 |  |
| 10 | 0,00 | 0,01042 |  |
| 11 | 0,02 | -0,32563 |  |
| 12 | 0,02 | -0,37033 |  |
| 13 | 0,01 | 0,21455 |  |
| 14 | 0,19 | 1,15216 |  |
| 15 | 0,00 | -0,06361 |  |
| 16 | 0,00 | -0,00484 |  |
| 17 | 0,01 | 0,29270 |  |
| 18 | 0,00 | 0,02628 |  |
| 19 | 0,06 | -0,60930 |  |
| 20 | 0,11 | 0,85184 |  |
| 21 | 0,05 | 0,53674 |  |
| 22 | 0,01 | -0,29156 |  |
| 23 | 0,02 | -0,39222 |  |
| 24 | 0,00 | -0,01072 |  |
| 25 | 0,01 | -0,22763 |  |
| 26 | 0,09 | 0,89748 | R |
| 27 | 0,00 | -0,15291 |  |

R  Large residual

## Forward Selection of Terms

α to enter = 0,25

## Coded Coefficients

| Term | Coef | SE Coef | 95% CI | T-Value | P-Value | VIF |
| --- | --- | --- | --- | --- | --- | --- |
| Constant | 2,559 | 0,302 | (1,930; 3,187) | 8,47 | 0,000 |  |
| Lac | 1,147 | 0,486 | (0,138; 2,157) | 2,36 | 0,028 | 1,10 |
| HPMC\_Visc | -0,724 | 0,404 | (-1,563; 0,116) | -1,79 | 0,087 | 1,00 |
| HPMC\_HP | 1,228 | 0,459 | (0,274; 2,182) | 2,68 | 0,014 | 1,00 |
| Lac\*Lac | 3,013 | 0,877 | (1,188; 4,838) | 3,43 | 0,002 | 1,00 |
| Lac\*HPMC\_HP | 1,55 | 1,12 | (-0,78; 3,88) | 1,38 | 0,182 | 1,10 |

## Model Summary

| S | R-sq | R-sq(adj) | PRESS | R-sq(pred) | AICc | BIC |
| --- | --- | --- | --- | --- | --- | --- |
| 1,13277 | 57,01% | 46,77% | 38,9660 | 37,83% | 96,46 | 99,64 |

## Analysis of Variance

| Source | DF | Seq SS | Contribution | Adj SS | Adj MS | F-Value | P-Value |
| --- | --- | --- | --- | --- | --- | --- | --- |
| Model | 5 | 35,734 | 57,01% | 35,734 | 7,1467 | 5,57 | 0,002 |
| Linear | 3 | 18,156 | 28,97% | 19,955 | 6,6516 | 5,18 | 0,008 |
| Lac | 1 | 5,333 | 8,51% | 7,165 | 7,1650 | 5,58 | 0,028 |
| HPMC\_Visc | 1 | 3,735 | 5,96% | 4,123 | 4,1233 | 3,21 | 0,087 |
| HPMC\_HP | 1 | 9,089 | 14,50% | 9,188 | 9,1878 | 7,16 | 0,014 |
| Square | 1 | 15,128 | 24,13% | 15,128 | 15,1276 | 11,79 | 0,002 |
| Lac\*Lac | 1 | 15,128 | 24,13% | 15,128 | 15,1276 | 11,79 | 0,002 |
| 2-Way Interaction | 1 | 2,450 | 3,91% | 2,450 | 2,4497 | 1,91 | 0,182 |
| Lac\*HPMC\_HP | 1 | 2,450 | 3,91% | 2,450 | 2,4497 | 1,91 | 0,182 |
| Error | 21 | 26,947 | 42,99% | 26,947 | 1,2832 |  |  |
| Lack-of-Fit | 19 | 16,247 | 25,92% | 16,247 | 0,8551 | 0,16 | 0,992 |
| Pure Error | 2 | 10,700 | 17,07% | 10,700 | 5,3499 |  |  |
| Total | 26 | 62,680 | 100,00% |  |  |  |  |

## Regression Equation in Uncoded Units

|  |  |  |
| --- | --- | --- |
| F\_SD\_14h(840min) | = | 32,3 - 101,5 Lac - 0,000186 HPMC\_Visc - 1,84 HPMC\_HP + 48,2 Lac\*Lac + 6,11 Lac\*HPMC\_HP |

## Fits and Diagnostics for All Observations

| Obs | F\_SD\_14h(840min) | Fit | SE Fit | 95% CI | Resid | Std Resid | Del Resid | HI |
| --- | --- | --- | --- | --- | --- | --- | --- | --- |
| 1 | 3,876 | 2,927 | 0,578 | (1,726; 4,128) | 0,949 | 0,97 | 0,97 | 0,259907 |
| 2 | 3,215 | 2,957 | 0,578 | (1,756; 4,158) | 0,259 | 0,27 | 0,26 | 0,259907 |
| 3 | 1,802 | 1,899 | 0,650 | (0,548; 3,249) | -0,097 | -0,10 | -0,10 | 0,328762 |
| 4 | 1,979 | 1,881 | 0,650 | (0,530; 3,232) | 0,098 | 0,11 | 0,10 | 0,328762 |
| 5 | 3,594 | 3,301 | 0,456 | (2,353; 4,248) | 0,293 | 0,28 | 0,28 | 0,161779 |
| 6 | 5,076 | 4,766 | 0,456 | (3,819; 5,714) | 0,310 | 0,30 | 0,29 | 0,161779 |
| 7 | 2,329 | 2,439 | 0,633 | (1,122; 3,756) | -0,110 | -0,12 | -0,11 | 0,312694 |
| 8 | 4,407 | 4,322 | 0,633 | (3,005; 5,639) | 0,085 | 0,09 | 0,09 | 0,312694 |
| 9 | 1,770 | 2,931 | 0,553 | (1,780; 4,081) | -1,161 | -1,17 | -1,19 | 0,238520 |
| 10 | 3,457 | 3,024 | 0,553 | (1,874; 4,175) | 0,433 | 0,44 | 0,43 | 0,238520 |
| 11 | 1,345 | 2,384 | 0,427 | (1,496; 3,273) | -1,040 | -0,99 | -0,99 | 0,142357 |
| 12 | 1,503 | 2,795 | 0,427 | (1,906; 3,683) | -1,291 | -1,23 | -1,25 | 0,142357 |
| 13 | 3,929 | 3,430 | 0,559 | (2,268; 4,592) | 0,499 | 0,51 | 0,50 | 0,243427 |
| 14 | 6,612 | 5,210 | 0,559 | (4,048; 6,373) | 1,402 | 1,42 | 1,46 | 0,243427 |
| 15 | 2,288 | 2,612 | 0,419 | (1,740; 3,484) | -0,324 | -0,31 | -0,30 | 0,136989 |
| 16 | 3,886 | 4,027 | 0,419 | (3,155; 4,898) | -0,140 | -0,13 | -0,13 | 0,136989 |
| 17 | 5,093 | 4,577 | 0,858 | (2,792; 6,362) | 0,516 | 0,70 | 0,69 | 0,573996 |
| 18 | 6,147 | 6,704 | 0,858 | (4,919; 8,489) | -0,557 | -0,75 | -0,74 | 0,573996 |
| 19 | 1,792 | 3,191 | 0,460 | (2,235; 4,147) | -1,399 | -1,35 | -1,38 | 0,164663 |
| 20 | 2,800 | 1,623 | 0,542 | (0,497; 2,750) | 1,177 | 1,18 | 1,19 | 0,228727 |
| 21 | 2,589 | 1,461 | 0,495 | (0,431; 2,492) | 1,127 | 1,11 | 1,11 | 0,191289 |
| 22 | 3,471 | 3,870 | 0,594 | (2,634; 5,106) | -0,399 | -0,41 | -0,41 | 0,275177 |
| 23 | 1,031 | 2,656 | 0,299 | (2,035; 3,277) | -1,625 | -1,49 | -1,53 | 0,069490 |
| 24 | 2,404 | 2,542 | 0,299 | (1,921; 3,163) | -0,138 | -0,13 | -0,12 | 0,069531 |
| 25 | 1,549 | 2,628 | 0,296 | (2,013; 3,243) | -1,079 | -0,99 | -0,99 | 0,068088 |
| 26 | 5,673 | 2,628 | 0,296 | (2,013; 3,243) | 3,045 | 2,78 | 3,42 | 0,068088 |
| 27 | 1,796 | 2,628 | 0,296 | (2,013; 3,243) | -0,832 | -0,76 | -0,75 | 0,068088 |

| Obs | Cook’s D | DFITS |  |
| --- | --- | --- | --- |
| 1 | 0,06 | 0,576469 |  |
| 2 | 0,00 | 0,153733 |  |
| 3 | 0,00 | -0,071291 |  |
| 4 | 0,00 | 0,071992 |  |
| 5 | 0,00 | 0,121398 |  |
| 6 | 0,00 | 0,128510 |  |
| 7 | 0,00 | -0,076855 |  |
| 8 | 0,00 | 0,059796 |  |
| 9 | 0,07 | -0,663467 |  |
| 10 | 0,01 | 0,240106 |  |
| 11 | 0,03 | -0,403710 |  |
| 12 | 0,04 | -0,508143 |  |
| 13 | 0,01 | 0,282126 |  |
| 14 | 0,11 | 0,828462 |  |
| 15 | 0,00 | -0,120112 |  |
| 16 | 0,00 | -0,051815 |  |
| 17 | 0,11 | 0,799282 |  |
| 18 | 0,13 | -0,864752 |  |
| 19 | 0,06 | -0,612767 |  |
| 20 | 0,07 | 0,650574 |  |
| 21 | 0,05 | 0,541348 |  |
| 22 | 0,01 | -0,249930 |  |
| 23 | 0,03 | -0,419308 |  |
| 24 | 0,00 | -0,033665 |  |
| 25 | 0,01 | -0,266555 |  |
| 26 | 0,09 | 0,924802 | R |
| 27 | 0,01 | -0,203547 |  |

R  Large residual

## Forward Selection of Terms

α to enter = 0,25

## Coded Coefficients

| Term | Coef | SE Coef | 95% CI | T-Value | P-Value | VIF |
| --- | --- | --- | --- | --- | --- | --- |
| Constant | 3,257 | 0,265 | (2,711; 3,802) | 12,29 | 0,000 |  |
| HPMC\_HP | 1,022 | 0,537 | (-0,085; 2,129) | 1,90 | 0,069 | 1,00 |

## Model Summary

| S | R-sq | R-sq(adj) | PRESS | R-sq(pred) | AICc | BIC |
| --- | --- | --- | --- | --- | --- | --- |
| 1,32822 | 12,63% | 9,13% | 49,9617 | 1,03% | 96,92 | 99,76 |

## Analysis of Variance

| Source | DF | Seq SS | Contribution | Adj SS | Adj MS | F-Value | P-Value |
| --- | --- | --- | --- | --- | --- | --- | --- |
| Model | 1 | 6,375 | 12,63% | 6,375 | 6,375 | 3,61 | 0,069 |
| Linear | 1 | 6,375 | 12,63% | 6,375 | 6,375 | 3,61 | 0,069 |
| HPMC\_HP | 1 | 6,375 | 12,63% | 6,375 | 6,375 | 3,61 | 0,069 |
| Error | 25 | 44,104 | 87,37% | 44,104 | 1,764 |  |  |
| Lack-of-Fit | 23 | 35,225 | 69,78% | 35,225 | 1,532 | 0,34 | 0,925 |
| Pure Error | 2 | 8,879 | 17,59% | 8,879 | 4,439 |  |  |
| Total | 26 | 50,480 | 100,00% |  |  |  |  |

## Regression Equation in Uncoded Units

|  |  |  |
| --- | --- | --- |
| F\_SD\_15h(900min) | = | -6,28 + 1,007 HPMC\_HP |

## Fits and Diagnostics for All Observations

| Obs | F\_SD\_15h(900min) | Fit | SE Fit | 95% CI | Resid | Std Resid | Del Resid | HI |
| --- | --- | --- | --- | --- | --- | --- | --- | --- |
| 1 | 4,545 | 2,520 | 0,408 | (1,680; 3,360) | 2,025 | 1,60 | 1,66 | 0,094295 |
| 2 | 3,098 | 2,520 | 0,408 | (1,680; 3,360) | 0,578 | 0,46 | 0,45 | 0,094295 |
| 3 | 1,836 | 2,489 | 0,421 | (1,622; 3,355) | -0,652 | -0,52 | -0,51 | 0,100407 |
| 4 | 2,994 | 2,489 | 0,421 | (1,622; 3,355) | 0,506 | 0,40 | 0,39 | 0,100407 |
| 5 | 3,497 | 3,466 | 0,313 | (2,822; 4,110) | 0,031 | 0,02 | 0,02 | 0,055395 |
| 6 | 4,903 | 3,466 | 0,313 | (2,822; 4,110) | 1,437 | 1,11 | 1,12 | 0,055395 |
| 7 | 2,512 | 3,741 | 0,413 | (2,890; 4,593) | -1,229 | -0,97 | -0,97 | 0,096807 |
| 8 | 5,260 | 3,741 | 0,413 | (2,890; 4,593) | 1,519 | 1,20 | 1,21 | 0,096807 |
| 9 | 1,653 | 2,562 | 0,391 | (1,756; 3,367) | -0,909 | -0,72 | -0,71 | 0,086679 |
| 10 | 3,621 | 2,562 | 0,391 | (1,756; 3,367) | 1,059 | 0,83 | 0,83 | 0,086679 |
| 11 | 1,294 | 2,770 | 0,316 | (2,119; 3,422) | -1,476 | -1,14 | -1,15 | 0,056666 |
| 12 | 1,300 | 2,770 | 0,316 | (2,119; 3,422) | -1,471 | -1,14 | -1,15 | 0,056666 |
| 13 | 4,017 | 3,674 | 0,386 | (2,879; 4,469) | 0,343 | 0,27 | 0,26 | 0,084428 |
| 14 | 5,133 | 3,674 | 0,386 | (2,879; 4,469) | 1,460 | 1,15 | 1,16 | 0,084428 |
| 15 | 2,349 | 3,433 | 0,303 | (2,809; 4,056) | -1,084 | -0,84 | -0,83 | 0,051977 |
| 16 | 3,590 | 3,433 | 0,303 | (2,809; 4,056) | 0,157 | 0,12 | 0,12 | 0,051977 |
| 17 | 5,204 | 3,201 | 0,259 | (2,668; 3,734) | 2,003 | 1,54 | 1,58 | 0,037969 |
| 18 | 4,905 | 3,201 | 0,259 | (2,668; 3,734) | 1,704 | 1,31 | 1,33 | 0,037969 |
| 19 | 1,843 | 3,181 | 0,257 | (2,651; 3,711) | -1,339 | -1,03 | -1,03 | 0,037546 |
| 20 | 2,649 | 3,080 | 0,257 | (2,552; 3,609) | -0,432 | -0,33 | -0,33 | 0,037337 |
| 21 | 2,543 | 2,235 | 0,533 | (1,137; 3,333) | 0,308 | 0,25 | 0,25 | 0,161083 |
| 22 | 3,716 | 4,278 | 0,659 | (2,922; 5,635) | -0,562 | -0,49 | -0,48 | 0,245997 |
| 23 | 1,152 | 3,181 | 0,257 | (2,651; 3,711) | -2,029 | -1,56 | -1,61 | 0,037546 |
| 24 | 2,288 | 3,080 | 0,257 | (2,552; 3,609) | -0,792 | -0,61 | -0,60 | 0,037337 |
| 25 | 1,533 | 3,201 | 0,259 | (2,668; 3,734) | -1,668 | -1,28 | -1,30 | 0,037969 |
| 26 | 5,248 | 3,201 | 0,259 | (2,668; 3,734) | 2,047 | 1,57 | 1,62 | 0,037969 |
| 27 | 1,669 | 3,201 | 0,259 | (2,668; 3,734) | -1,532 | -1,18 | -1,19 | 0,037969 |

| Obs | Cook’s D | DFITS |  |
| --- | --- | --- | --- |
| 1 | 0,13 | 0,534563 |  |
| 2 | 0,01 | 0,145084 |  |
| 3 | 0,01 | -0,170366 |  |
| 4 | 0,01 | 0,131888 |  |
| 5 | 0,00 | 0,005621 |  |
| 6 | 0,04 | 0,270904 |  |
| 7 | 0,05 | -0,318479 |  |
| 8 | 0,08 | 0,397563 |  |
| 9 | 0,02 | -0,218359 |  |
| 10 | 0,03 | 0,255396 |  |
| 11 | 0,04 | -0,282304 |  |
| 12 | 0,04 | -0,281166 |  |
| 13 | 0,00 | 0,080455 |  |
| 14 | 0,06 | 0,351086 |  |
| 15 | 0,02 | -0,195042 |  |
| 16 | 0,00 | 0,027945 |  |
| 17 | 0,05 | 0,314483 |  |
| 18 | 0,03 | 0,263762 |  |
| 19 | 0,02 | -0,203132 |  |
| 20 | 0,00 | -0,064068 |  |
| 21 | 0,01 | 0,108879 |  |
| 22 | 0,04 | -0,274038 | X |
| 23 | 0,05 | -0,317078 |  |
| 24 | 0,01 | -0,118205 |  |
| 25 | 0,03 | -0,257881 |  |
| 26 | 0,05 | 0,322197 |  |
| 27 | 0,03 | -0,235568 |  |

X  Unusual X

## Forward Selection of Terms

α to enter = 0,25

## Coded Coefficients

| Term | Coef | SE Coef | 95% CI | T-Value | P-Value | VIF |
| --- | --- | --- | --- | --- | --- | --- |
| Constant | 3,102 | 0,237 | (2,615; 3,589) | 13,11 | 0,000 |  |
| HPMC\_HP | 1,004 | 0,480 | (0,016; 1,993) | 2,09 | 0,047 | 1,00 |

## Model Summary

| S | R-sq | R-sq(adj) | PRESS | R-sq(pred) | AICc | BIC |
| --- | --- | --- | --- | --- | --- | --- |
| 1,18613 | 14,90% | 11,50% | 39,8374 | 3,61% | 90,81 | 93,65 |

## Analysis of Variance

| Source | DF | Seq SS | Contribution | Adj SS | Adj MS | F-Value | P-Value |
| --- | --- | --- | --- | --- | --- | --- | --- |
| Model | 1 | 6,158 | 14,90% | 6,158 | 6,158 | 4,38 | 0,047 |
| Linear | 1 | 6,158 | 14,90% | 6,158 | 6,158 | 4,38 | 0,047 |
| HPMC\_HP | 1 | 6,158 | 14,90% | 6,158 | 6,158 | 4,38 | 0,047 |
| Error | 25 | 35,173 | 85,10% | 35,173 | 1,407 |  |  |
| Lack-of-Fit | 23 | 27,618 | 66,82% | 27,618 | 1,201 | 0,32 | 0,938 |
| Pure Error | 2 | 7,555 | 18,28% | 7,555 | 3,777 |  |  |
| Total | 26 | 41,331 | 100,00% |  |  |  |  |

## Regression Equation in Uncoded Units

|  |  |  |
| --- | --- | --- |
| F\_SD\_16h(960min) | = | -6,27 + 0,989 HPMC\_HP |

## Fits and Diagnostics for All Observations

| Obs | F\_SD\_16h(960min) | Fit | SE Fit | 95% CI | Resid | Std Resid | Del Resid | HI |
| --- | --- | --- | --- | --- | --- | --- | --- | --- |
| 1 | 4,115 | 2,378 | 0,364 | (1,628; 3,128) | 1,737 | 1,54 | 1,58 | 0,094295 |
| 2 | 3,274 | 2,378 | 0,364 | (1,628; 3,128) | 0,896 | 0,79 | 0,79 | 0,094295 |
| 3 | 1,831 | 2,347 | 0,376 | (1,573; 3,121) | -0,516 | -0,46 | -0,45 | 0,100407 |
| 4 | 3,457 | 2,347 | 0,376 | (1,573; 3,121) | 1,110 | 0,99 | 0,99 | 0,100407 |
| 5 | 3,358 | 3,308 | 0,279 | (2,733; 3,883) | 0,050 | 0,04 | 0,04 | 0,055395 |
| 6 | 4,508 | 3,308 | 0,279 | (2,733; 3,883) | 1,200 | 1,04 | 1,04 | 0,055395 |
| 7 | 2,764 | 3,578 | 0,369 | (2,818; 4,339) | -0,814 | -0,72 | -0,72 | 0,096807 |
| 8 | 5,430 | 3,578 | 0,369 | (2,818; 4,339) | 1,851 | 1,64 | 1,70 | 0,096807 |
| 9 | 1,468 | 2,419 | 0,349 | (1,700; 3,138) | -0,951 | -0,84 | -0,83 | 0,086679 |
| 10 | 3,186 | 2,419 | 0,349 | (1,700; 3,138) | 0,767 | 0,68 | 0,67 | 0,086679 |
| 11 | 1,246 | 2,624 | 0,282 | (2,043; 3,206) | -1,378 | -1,20 | -1,21 | 0,056666 |
| 12 | 1,172 | 2,624 | 0,282 | (2,043; 3,206) | -1,452 | -1,26 | -1,28 | 0,056666 |
| 13 | 3,866 | 3,512 | 0,345 | (2,802; 4,222) | 0,354 | 0,31 | 0,31 | 0,084428 |
| 14 | 3,716 | 3,512 | 0,345 | (2,802; 4,222) | 0,204 | 0,18 | 0,18 | 0,084428 |
| 15 | 2,365 | 3,275 | 0,270 | (2,718; 3,832) | -0,910 | -0,79 | -0,78 | 0,051977 |
| 16 | 3,375 | 3,275 | 0,270 | (2,718; 3,832) | 0,100 | 0,09 | 0,08 | 0,051977 |
| 17 | 5,095 | 3,048 | 0,231 | (2,572; 3,524) | 2,047 | 1,76 | 1,84 | 0,037969 |
| 18 | 3,807 | 3,048 | 0,231 | (2,572; 3,524) | 0,759 | 0,65 | 0,64 | 0,037969 |
| 19 | 2,004 | 3,028 | 0,230 | (2,554; 3,501) | -1,024 | -0,88 | -0,88 | 0,037546 |
| 20 | 2,429 | 2,929 | 0,229 | (2,457; 3,401) | -0,500 | -0,43 | -0,42 | 0,037337 |
| 21 | 2,386 | 2,098 | 0,476 | (1,117; 3,078) | 0,289 | 0,27 | 0,26 | 0,161083 |
| 22 | 4,091 | 4,106 | 0,588 | (2,895; 5,318) | -0,015 | -0,01 | -0,01 | 0,245997 |
| 23 | 1,311 | 3,028 | 0,230 | (2,554; 3,501) | -1,717 | -1,48 | -1,51 | 0,037546 |
| 24 | 2,169 | 2,929 | 0,229 | (2,457; 3,401) | -0,760 | -0,65 | -0,65 | 0,037337 |
| 25 | 1,404 | 3,048 | 0,231 | (2,572; 3,524) | -1,643 | -1,41 | -1,44 | 0,037969 |
| 26 | 4,848 | 3,048 | 0,231 | (2,572; 3,524) | 1,801 | 1,55 | 1,60 | 0,037969 |
| 27 | 1,566 | 3,048 | 0,231 | (2,572; 3,524) | -1,482 | -1,27 | -1,29 | 0,037969 |

| Obs | Cook’s D | DFITS |  |
| --- | --- | --- | --- |
| 1 | 0,12 | 0,511234 |  |
| 2 | 0,03 | 0,254079 |  |
| 3 | 0,01 | -0,150770 |  |
| 4 | 0,05 | 0,329453 |  |
| 5 | 0,00 | 0,010200 |  |
| 6 | 0,03 | 0,252619 |  |
| 7 | 0,03 | -0,234139 |  |
| 8 | 0,14 | 0,557750 |  |
| 9 | 0,03 | -0,256973 |  |
| 10 | 0,02 | 0,206082 |  |
| 11 | 0,04 | -0,295843 |  |
| 12 | 0,05 | -0,312770 |  |
| 13 | 0,00 | 0,093024 |  |
| 14 | 0,00 | 0,053390 |  |
| 15 | 0,02 | -0,183136 |  |
| 16 | 0,00 | 0,019792 |  |
| 17 | 0,06 | 0,365939 |  |
| 18 | 0,01 | 0,128099 |  |
| 19 | 0,02 | -0,172991 |  |
| 20 | 0,00 | -0,083229 |  |
| 21 | 0,01 | 0,114226 |  |
| 22 | 0,00 | -0,008263 | X |
| 23 | 0,04 | -0,298891 |  |
| 24 | 0,01 | -0,127155 |  |
| 25 | 0,04 | -0,286615 |  |
| 26 | 0,05 | 0,316886 |  |
| 27 | 0,03 | -0,256382 |  |

X  Unusual X

## Forward Selection of Terms

α to enter = 0,25

## Coded Coefficients

| Term | Coef | SE Coef | 95% CI | T-Value | P-Value | VIF |
| --- | --- | --- | --- | --- | --- | --- |
| Constant | 2,891 | 0,236 | (2,406; 3,377) | 12,27 | 0,000 |  |
| HPMC\_HP | 0,744 | 0,478 | (-0,241; 1,729) | 1,56 | 0,132 | 1,00 |

## Model Summary

| S | R-sq | R-sq(adj) | PRESS | R-sq(pred) | AICc | BIC |
| --- | --- | --- | --- | --- | --- | --- |
| 1,18176 | 8,83% | 5,18% | 40,1946 | 0,00% | 90,61 | 93,45 |

## Analysis of Variance

| Source | DF | Seq SS | Contribution | Adj SS | Adj MS | F-Value | P-Value |
| --- | --- | --- | --- | --- | --- | --- | --- |
| Model | 1 | 3,380 | 8,83% | 3,380 | 3,380 | 2,42 | 0,132 |
| Linear | 1 | 3,380 | 8,83% | 3,380 | 3,380 | 2,42 | 0,132 |
| HPMC\_HP | 1 | 3,380 | 8,83% | 3,380 | 3,380 | 2,42 | 0,132 |
| Error | 25 | 34,914 | 91,17% | 34,914 | 1,397 |  |  |
| Lack-of-Fit | 23 | 28,516 | 74,47% | 28,516 | 1,240 | 0,39 | 0,902 |
| Pure Error | 2 | 6,398 | 16,71% | 6,398 | 3,199 |  |  |
| Total | 26 | 38,294 | 100,00% |  |  |  |  |

## Regression Equation in Uncoded Units

|  |  |  |
| --- | --- | --- |
| F\_SD\_17h(1020min) | = | -4,05 + 0,733 HPMC\_HP |

## Fits and Diagnostics for All Observations

| Obs | F\_SD\_17h(1020min) | Fit | SE Fit | 95% CI | Resid | Std Resid | Del Resid | HI |
| --- | --- | --- | --- | --- | --- | --- | --- | --- |
| 1 | 5,036 | 2,355 | 0,363 | (1,608; 3,102) | 2,681 | 2,38 | 2,66 | 0,094295 |
| 2 | 3,080 | 2,355 | 0,363 | (1,608; 3,102) | 0,725 | 0,64 | 0,64 | 0,094295 |
| 3 | 1,802 | 2,332 | 0,374 | (1,561; 3,103) | -0,530 | -0,47 | -0,47 | 0,100407 |
| 4 | 3,395 | 2,332 | 0,374 | (1,561; 3,103) | 1,063 | 0,95 | 0,95 | 0,100407 |
| 5 | 3,392 | 3,044 | 0,278 | (2,471; 3,617) | 0,348 | 0,30 | 0,30 | 0,055395 |
| 6 | 4,087 | 3,044 | 0,278 | (2,471; 3,617) | 1,043 | 0,91 | 0,91 | 0,055395 |
| 7 | 2,411 | 3,244 | 0,368 | (2,487; 4,002) | -0,834 | -0,74 | -0,74 | 0,096807 |
| 8 | 5,066 | 3,244 | 0,368 | (2,487; 4,002) | 1,822 | 1,62 | 1,68 | 0,096807 |
| 9 | 1,271 | 2,385 | 0,348 | (1,669; 3,102) | -1,114 | -0,99 | -0,99 | 0,086679 |
| 10 | 2,708 | 2,385 | 0,348 | (1,669; 3,102) | 0,322 | 0,29 | 0,28 | 0,086679 |
| 11 | 1,171 | 2,537 | 0,281 | (1,958; 3,117) | -1,366 | -1,19 | -1,20 | 0,056666 |
| 12 | 1,334 | 2,537 | 0,281 | (1,958; 3,117) | -1,203 | -1,05 | -1,05 | 0,056666 |
| 13 | 3,689 | 3,195 | 0,343 | (2,488; 3,902) | 0,494 | 0,44 | 0,43 | 0,084428 |
| 14 | 2,400 | 3,195 | 0,343 | (2,488; 3,902) | -0,795 | -0,70 | -0,70 | 0,084428 |
| 15 | 2,340 | 3,020 | 0,269 | (2,465; 3,575) | -0,680 | -0,59 | -0,58 | 0,051977 |
| 16 | 2,990 | 3,020 | 0,269 | (2,465; 3,575) | -0,030 | -0,03 | -0,03 | 0,051977 |
| 17 | 4,897 | 2,851 | 0,230 | (2,377; 3,325) | 2,046 | 1,77 | 1,85 | 0,037969 |
| 18 | 2,830 | 2,851 | 0,230 | (2,377; 3,325) | -0,021 | -0,02 | -0,02 | 0,037969 |
| 19 | 2,106 | 2,836 | 0,229 | (2,365; 3,308) | -0,730 | -0,63 | -0,62 | 0,037546 |
| 20 | 2,241 | 2,763 | 0,228 | (2,293; 3,233) | -0,522 | -0,45 | -0,44 | 0,037337 |
| 21 | 2,322 | 2,147 | 0,474 | (1,171; 3,124) | 0,174 | 0,16 | 0,16 | 0,161083 |
| 22 | 4,069 | 3,635 | 0,586 | (2,428; 4,843) | 0,433 | 0,42 | 0,42 | 0,245997 |
| 23 | 1,438 | 2,836 | 0,229 | (2,365; 3,308) | -1,398 | -1,21 | -1,22 | 0,037546 |
| 24 | 2,048 | 2,763 | 0,228 | (2,293; 3,233) | -0,715 | -0,62 | -0,61 | 0,037337 |
| 25 | 1,391 | 2,851 | 0,230 | (2,377; 3,325) | -1,460 | -1,26 | -1,28 | 0,037969 |
| 26 | 4,512 | 2,851 | 0,230 | (2,377; 3,325) | 1,661 | 1,43 | 1,47 | 0,037969 |
| 27 | 1,437 | 2,851 | 0,230 | (2,377; 3,325) | -1,414 | -1,22 | -1,23 | 0,037969 |

| Obs | Cook’s D | DFITS |  |  |
| --- | --- | --- | --- | --- |
| 1 | 0,30 | 0,857443 | R |  |
| 2 | 0,02 | 0,205563 |  |  |
| 3 | 0,01 | -0,155547 |  |  |
| 4 | 0,05 | 0,316054 |  |  |
| 5 | 0,00 | 0,072055 |  |  |
| 6 | 0,02 | 0,219186 |  |  |
| 7 | 0,03 | -0,240764 |  |  |
| 8 | 0,14 | 0,550147 |  |  |
| 9 | 0,05 | -0,303714 |  |  |
| 10 | 0,00 | 0,086297 |  |  |
| 11 | 0,04 | -0,294270 |  |  |
| 12 | 0,03 | -0,257506 |  |  |
| 13 | 0,01 | 0,130434 |  |  |
| 14 | 0,02 | -0,211279 |  |  |
| 15 | 0,01 | -0,136547 |  |  |
| 16 | 0,00 | -0,005924 |  |  |
| 17 | 0,06 | 0,367255 |  |  |
| 18 | 0,00 | -0,003522 |  |  |
| 19 | 0,01 | -0,122910 |  |  |
| 20 | 0,00 | -0,087212 |  |  |
| 21 | 0,00 | 0,069133 |  |  |
| 22 | 0,03 | 0,237131 |  | X |
| 23 | 0,03 | -0,240490 |  |  |
| 24 | 0,01 | -0,119946 |  |  |
| 25 | 0,03 | -0,253375 |  |  |
| 26 | 0,04 | 0,291073 |  |  |
| 27 | 0,03 | -0,244804 |  |  |

R  Large residual  
X  Unusual X

## Forward Selection of Terms

α to enter = 0,25

## Coded Coefficients

| Term | Coef | SE Coef | 95% CI | T-Value | P-Value | VIF |
| --- | --- | --- | --- | --- | --- | --- |
| Constant | 2,615 | 0,215 | (2,172; 3,058) | 12,15 | 0,000 |  |
| HPMC\_HP | 0,613 | 0,437 | (-0,286; 1,512) | 1,40 | 0,172 | 1,00 |

## Model Summary

| S | R-sq | R-sq(adj) | PRESS | R-sq(pred) | AICc | BIC |
| --- | --- | --- | --- | --- | --- | --- |
| 1,07876 | 7,32% | 3,61% | 33,5486 | 0,00% | 85,68 | 88,53 |

## Analysis of Variance

| Source | DF | Seq SS | Contribution | Adj SS | Adj MS | F-Value | P-Value |
| --- | --- | --- | --- | --- | --- | --- | --- |
| Model | 1 | 2,297 | 7,32% | 2,297 | 2,297 | 1,97 | 0,172 |
| Linear | 1 | 2,297 | 7,32% | 2,297 | 2,297 | 1,97 | 0,172 |
| HPMC\_HP | 1 | 2,297 | 7,32% | 2,297 | 2,297 | 1,97 | 0,172 |
| Error | 25 | 29,093 | 92,68% | 29,093 | 1,164 |  |  |
| Lack-of-Fit | 23 | 23,729 | 75,59% | 23,729 | 1,032 | 0,38 | 0,904 |
| Pure Error | 2 | 5,364 | 17,09% | 5,364 | 2,682 |  |  |
| Total | 26 | 31,390 | 100,00% |  |  |  |  |

## Regression Equation in Uncoded Units

|  |  |  |
| --- | --- | --- |
| F\_SD\_18h(1080min) | = | -3,11 + 0,604 HPMC\_HP |

## Fits and Diagnostics for All Observations

| Obs | F\_SD\_18h(1080min) | Fit | SE Fit | 95% CI | Resid | Std Resid | Del Resid | HI |
| --- | --- | --- | --- | --- | --- | --- | --- | --- |
| 1 | 4,498 | 2,173 | 0,331 | (1,491; 2,855) | 2,325 | 2,26 | 2,49 | 0,094295 |
| 2 | 2,928 | 2,173 | 0,331 | (1,491; 2,855) | 0,755 | 0,74 | 0,73 | 0,094295 |
| 3 | 1,640 | 2,154 | 0,342 | (1,450; 2,858) | -0,514 | -0,50 | -0,49 | 0,100407 |
| 4 | 2,919 | 2,154 | 0,342 | (1,450; 2,858) | 0,765 | 0,75 | 0,74 | 0,100407 |
| 5 | 3,257 | 2,741 | 0,254 | (2,218; 3,264) | 0,516 | 0,49 | 0,48 | 0,055395 |
| 6 | 3,655 | 2,741 | 0,254 | (2,218; 3,264) | 0,914 | 0,87 | 0,87 | 0,055395 |
| 7 | 2,244 | 2,906 | 0,336 | (2,215; 3,597) | -0,662 | -0,65 | -0,64 | 0,096807 |
| 8 | 4,397 | 2,906 | 0,336 | (2,215; 3,597) | 1,490 | 1,45 | 1,49 | 0,096807 |
| 9 | 0,937 | 2,198 | 0,318 | (1,544; 2,852) | -1,261 | -1,22 | -1,24 | 0,086679 |
| 10 | 2,278 | 2,198 | 0,318 | (1,544; 2,852) | 0,080 | 0,08 | 0,08 | 0,086679 |
| 11 | 1,090 | 2,323 | 0,257 | (1,794; 2,852) | -1,233 | -1,18 | -1,19 | 0,056666 |
| 12 | 1,714 | 2,323 | 0,257 | (1,794; 2,852) | -0,609 | -0,58 | -0,57 | 0,056666 |
| 13 | 3,428 | 2,866 | 0,313 | (2,220; 3,511) | 0,562 | 0,54 | 0,54 | 0,084428 |
| 14 | 1,485 | 2,866 | 0,313 | (2,220; 3,511) | -1,380 | -1,34 | -1,36 | 0,084428 |
| 15 | 2,188 | 2,721 | 0,246 | (2,214; 3,227) | -0,533 | -0,51 | -0,50 | 0,051977 |
| 16 | 2,609 | 2,721 | 0,246 | (2,214; 3,227) | -0,112 | -0,11 | -0,10 | 0,051977 |
| 17 | 4,700 | 2,582 | 0,210 | (2,149; 3,015) | 2,118 | 2,00 | 2,14 | 0,037969 |
| 18 | 1,945 | 2,582 | 0,210 | (2,149; 3,015) | -0,637 | -0,60 | -0,59 | 0,037969 |
| 19 | 2,139 | 2,570 | 0,209 | (2,139; 3,000) | -0,431 | -0,41 | -0,40 | 0,037546 |
| 20 | 2,064 | 2,509 | 0,208 | (2,080; 2,939) | -0,445 | -0,42 | -0,41 | 0,037337 |
| 21 | 2,264 | 2,002 | 0,433 | (1,110; 2,894) | 0,262 | 0,27 | 0,26 | 0,161083 |
| 22 | 3,731 | 3,228 | 0,535 | (2,126; 4,330) | 0,503 | 0,54 | 0,53 | 0,245997 |
| 23 | 1,610 | 2,570 | 0,209 | (2,139; 3,000) | -0,960 | -0,91 | -0,90 | 0,037546 |
| 24 | 1,946 | 2,509 | 0,208 | (2,080; 2,939) | -0,563 | -0,53 | -0,52 | 0,037337 |
| 25 | 1,342 | 2,582 | 0,210 | (2,149; 3,015) | -1,240 | -1,17 | -1,18 | 0,037969 |
| 26 | 4,156 | 2,582 | 0,210 | (2,149; 3,015) | 1,574 | 1,49 | 1,53 | 0,037969 |
| 27 | 1,298 | 2,582 | 0,210 | (2,149; 3,015) | -1,284 | -1,21 | -1,23 | 0,037969 |

| Obs | Cook’s D | DFITS |  |  |
| --- | --- | --- | --- | --- |
| 1 | 0,27 | 0,802939 | R |  |
| 2 | 0,03 | 0,235077 |  |  |
| 3 | 0,01 | -0,165246 |  |  |
| 4 | 0,03 | 0,247651 |  |  |
| 5 | 0,01 | 0,117280 |  |  |
| 6 | 0,02 | 0,210176 |  |  |
| 7 | 0,02 | -0,208937 |  |  |
| 8 | 0,11 | 0,487382 |  |  |
| 9 | 0,07 | -0,380622 |  |  |
| 10 | 0,00 | 0,023504 |  |  |
| 11 | 0,04 | -0,290838 |  |  |
| 12 | 0,01 | -0,140541 |  |  |
| 13 | 0,01 | 0,163021 |  |  |
| 14 | 0,08 | -0,412872 |  |  |
| 15 | 0,01 | -0,117072 |  |  |
| 16 | 0,00 | -0,024400 |  |  |
| 17 | 0,08 | 0,425249 | R |  |
| 18 | 0,01 | -0,118117 |  |  |
| 19 | 0,00 | -0,079045 |  |  |
| 20 | 0,00 | -0,081467 |  |  |
| 21 | 0,01 | 0,113961 |  |  |
| 22 | 0,05 | 0,302066 |  | X |
| 23 | 0,02 | -0,178464 |  |  |
| 24 | 0,01 | -0,103209 |  |  |
| 25 | 0,03 | -0,234730 |  |  |
| 26 | 0,04 | 0,303283 |  |  |
| 27 | 0,03 | -0,243558 |  |  |

R  Large residual  
X  Unusual X

## Forward Selection of Terms

α to enter = 0,25

## Coded Coefficients

| Term | Coef | SE Coef | 95% CI | T-Value | P-Value | VIF |
| --- | --- | --- | --- | --- | --- | --- |
| Constant | 2,313 | 0,186 | (1,929; 2,697) | 12,46 | 0,000 |  |
| Lac | -0,466 | 0,380 | (-1,252; 0,320) | -1,23 | 0,232 | 1,00 |
| HPMC\_HP | 0,654 | 0,379 | (-0,130; 1,439) | 1,73 | 0,098 | 1,01 |
| HPMC\_PS | -0,569 | 0,451 | (-1,501; 0,364) | -1,26 | 0,220 | 1,01 |

## Model Summary

| S | R-sq | R-sq(adj) | PRESS | R-sq(pred) | AICc | BIC |
| --- | --- | --- | --- | --- | --- | --- |
| 0,930430 | 19,65% | 9,16% | 27,8047 | 0,00% | 81,26 | 84,88 |

## Analysis of Variance

| Source | DF | Seq SS | Contribution | Adj SS | Adj MS | F-Value | P-Value |
| --- | --- | --- | --- | --- | --- | --- | --- |
| Model | 3 | 4,868 | 19,65% | 4,868 | 1,6227 | 1,87 | 0,162 |
| Linear | 3 | 4,868 | 19,65% | 4,868 | 1,6227 | 1,87 | 0,162 |
| Lac | 1 | 1,302 | 5,25% | 1,302 | 1,3019 | 1,50 | 0,232 |
| HPMC\_HP | 1 | 2,189 | 8,84% | 2,579 | 2,5792 | 2,98 | 0,098 |
| HPMC\_PS | 1 | 1,377 | 5,56% | 1,377 | 1,3768 | 1,59 | 0,220 |
| Error | 23 | 19,911 | 80,35% | 19,911 | 0,8657 |  |  |
| Lack-of-Fit | 21 | 15,416 | 62,21% | 15,416 | 0,7341 | 0,33 | 0,932 |
| Pure Error | 2 | 4,495 | 18,14% | 4,495 | 2,2476 |  |  |
| Total | 26 | 24,779 | 100,00% |  |  |  |  |

## Regression Equation in Uncoded Units

|  |  |  |
| --- | --- | --- |
| F\_SD\_19h(1140min) | = | 2,52 - 1,86 Lac + 0,645 HPMC\_HP - 0,0773 HPMC\_PS |

## Fits and Diagnostics for All Observations

| Obs | F\_SD\_19h(1140min) | Fit | SE Fit | 95% CI | Resid | Std Resid | Del Resid | HI |
| --- | --- | --- | --- | --- | --- | --- | --- | --- |
| 1 | 3,547 | 2,215 | 0,353 | (1,485; 2,944) | 1,333 | 1,55 | 1,60 | 0,143688 |
| 2 | 2,278 | 1,749 | 0,353 | (1,019; 2,478) | 0,529 | 0,61 | 0,61 | 0,143688 |
| 3 | 1,435 | 2,383 | 0,419 | (1,515; 3,250) | -0,948 | -1,14 | -1,15 | 0,203044 |
| 4 | 2,320 | 1,917 | 0,419 | (1,050; 2,784) | 0,403 | 0,49 | 0,48 | 0,203044 |
| 5 | 2,563 | 2,778 | 0,303 | (2,151; 3,405) | -0,214 | -0,24 | -0,24 | 0,106120 |
| 6 | 3,236 | 2,312 | 0,303 | (1,685; 2,939) | 0,924 | 1,05 | 1,05 | 0,106120 |
| 7 | 2,453 | 3,078 | 0,399 | (2,252; 3,903) | -0,625 | -0,74 | -0,74 | 0,184023 |
| 8 | 3,705 | 2,612 | 0,399 | (1,786; 3,437) | 1,094 | 1,30 | 1,32 | 0,184023 |
| 9 | 0,697 | 1,865 | 0,397 | (1,044; 2,685) | -1,168 | -1,39 | -1,42 | 0,181719 |
| 10 | 1,948 | 1,399 | 0,397 | (0,578; 2,219) | 0,549 | 0,65 | 0,64 | 0,181719 |
| 11 | 1,089 | 2,114 | 0,313 | (1,466; 2,762) | -1,026 | -1,17 | -1,18 | 0,113336 |
| 12 | 1,459 | 1,649 | 0,313 | (1,001; 2,297) | -0,190 | -0,22 | -0,21 | 0,113336 |
| 13 | 3,254 | 2,517 | 0,394 | (1,702; 3,332) | 0,737 | 0,87 | 0,87 | 0,179186 |
| 14 | 0,853 | 2,051 | 0,394 | (1,237; 2,866) | -1,198 | -1,42 | -1,46 | 0,179186 |
| 15 | 2,088 | 2,507 | 0,305 | (1,875; 3,139) | -0,419 | -0,48 | -0,47 | 0,107803 |
| 16 | 2,136 | 2,041 | 0,305 | (1,409; 2,673) | 0,095 | 0,11 | 0,11 | 0,107803 |
| 17 | 4,468 | 2,763 | 0,421 | (1,892; 3,634) | 1,706 | 2,06 | 2,23 | 0,204905 |
| 18 | 1,269 | 1,831 | 0,421 | (0,960; 2,702) | -0,562 | -0,68 | -0,67 | 0,204905 |
| 19 | 2,076 | 2,211 | 0,185 | (1,828; 2,595) | -0,135 | -0,15 | -0,14 | 0,039704 |
| 20 | 1,964 | 2,280 | 0,189 | (1,889; 2,670) | -0,316 | -0,35 | -0,34 | 0,041165 |
| 21 | 2,220 | 1,718 | 0,373 | (0,945; 2,491) | 0,502 | 0,59 | 0,58 | 0,161116 |
| 22 | 3,190 | 2,936 | 0,462 | (1,980; 3,892) | 0,254 | 0,31 | 0,31 | 0,246534 |
| 23 | 1,834 | 2,833 | 0,484 | (1,831; 3,835) | -0,999 | -1,26 | -1,27 | 0,271099 |
| 24 | 1,946 | 1,631 | 0,491 | (0,616; 2,646) | 0,315 | 0,40 | 0,39 | 0,278014 |
| 25 | 1,260 | 2,297 | 0,182 | (1,921; 2,673) | -1,037 | -1,14 | -1,14 | 0,038239 |
| 26 | 3,814 | 2,297 | 0,182 | (1,921; 2,673) | 1,517 | 1,66 | 1,73 | 0,038239 |
| 27 | 1,176 | 2,297 | 0,182 | (1,921; 2,673) | -1,121 | -1,23 | -1,24 | 0,038239 |

| Obs | Cook’s D | DFITS |  |
| --- | --- | --- | --- |
| 1 | 0,10 | 0,65529 |  |
| 2 | 0,02 | 0,24816 |  |
| 3 | 0,08 | -0,57991 |  |
| 4 | 0,02 | 0,24099 |  |
| 5 | 0,00 | -0,08225 |  |
| 6 | 0,03 | 0,36296 |  |
| 7 | 0,03 | -0,34959 |  |
| 8 | 0,10 | 0,62797 |  |
| 9 | 0,11 | -0,66828 |  |
| 10 | 0,02 | 0,30329 |  |
| 11 | 0,04 | -0,42208 |  |
| 12 | 0,00 | -0,07589 |  |
| 13 | 0,04 | 0,40647 |  |
| 14 | 0,11 | -0,68000 |  |
| 15 | 0,01 | -0,16280 |  |
| 16 | 0,00 | 0,03686 |  |
| 17 | 0,27 | 1,12977 | R |
| 18 | 0,03 | -0,33996 |  |
| 19 | 0,00 | -0,02944 |  |
| 20 | 0,00 | -0,07036 |  |
| 21 | 0,02 | 0,25437 |  |
| 22 | 0,01 | 0,17653 |  |
| 23 | 0,15 | -0,77755 |  |
| 24 | 0,02 | 0,24233 |  |
| 25 | 0,01 | -0,22822 |  |
| 26 | 0,03 | 0,34556 |  |
| 27 | 0,01 | -0,24776 |  |

R  Large residual

## Forward Selection of Terms

α to enter = 0,25

## Coded Coefficients

| Term | Coef | SE Coef | 95% CI | T-Value | P-Value | VIF |
| --- | --- | --- | --- | --- | --- | --- |
| Constant | 2,042 | 0,168 | (1,694; 2,389) | 12,15 | 0,000 |  |
| Lac | -0,816 | 0,344 | (-1,528; -0,105) | -2,37 | 0,026 | 1,00 |
| HPMC\_HP | 0,665 | 0,343 | (-0,045; 1,375) | 1,94 | 0,065 | 1,01 |
| HPMC\_PS | -0,529 | 0,408 | (-1,373; 0,316) | -1,29 | 0,208 | 1,01 |

## Model Summary

| S | R-sq | R-sq(adj) | PRESS | R-sq(pred) | AICc | BIC |
| --- | --- | --- | --- | --- | --- | --- |
| 0,842602 | 31,43% | 22,49% | 22,6104 | 5,06% | 75,90 | 79,52 |

## Analysis of Variance

| Source | DF | Seq SS | Contribution | Adj SS | Adj MS | F-Value | P-Value |
| --- | --- | --- | --- | --- | --- | --- | --- |
| Model | 3 | 7,487 | 31,43% | 7,487 | 2,4955 | 3,51 | 0,031 |
| Linear | 3 | 7,487 | 31,43% | 7,487 | 2,4955 | 3,51 | 0,031 |
| Lac | 1 | 3,999 | 16,79% | 3,999 | 3,9985 | 5,63 | 0,026 |
| HPMC\_HP | 1 | 2,297 | 9,65% | 2,664 | 2,6644 | 3,75 | 0,065 |
| HPMC\_PS | 1 | 1,191 | 5,00% | 1,191 | 1,1906 | 1,68 | 0,208 |
| Error | 23 | 16,329 | 68,57% | 16,329 | 0,7100 |  |  |
| Lack-of-Fit | 21 | 12,331 | 51,78% | 12,331 | 0,5872 | 0,29 | 0,948 |
| Pure Error | 2 | 3,998 | 16,79% | 3,998 | 1,9991 |  |  |
| Total | 26 | 23,816 | 100,00% |  |  |  |  |

## Regression Equation in Uncoded Units

|  |  |  |
| --- | --- | --- |
| F\_SD\_20h(1200min) | = | 2,47 - 3,27 Lac + 0,655 HPMC\_HP - 0,0719 HPMC\_PS |

## Fits and Diagnostics for All Observations

| Obs | F\_SD\_20h(1200min) | Fit | SE Fit | 95% CI | Resid | Std Resid | Del Resid | HI |
| --- | --- | --- | --- | --- | --- | --- | --- | --- |
| 1 | 3,129 | 2,101 | 0,319 | (1,440; 2,762) | 1,028 | 1,32 | 1,34 | 0,143688 |
| 2 | 1,458 | 1,285 | 0,319 | (0,624; 1,946) | 0,174 | 0,22 | 0,22 | 0,143688 |
| 3 | 1,199 | 2,256 | 0,380 | (1,470; 3,041) | -1,056 | -1,40 | -1,44 | 0,203044 |
| 4 | 1,760 | 1,439 | 0,380 | (0,654; 2,225) | 0,321 | 0,43 | 0,42 | 0,203044 |
| 5 | 2,589 | 2,677 | 0,274 | (2,109; 3,245) | -0,088 | -0,11 | -0,11 | 0,106120 |
| 6 | 2,758 | 1,861 | 0,274 | (1,293; 2,429) | 0,898 | 1,13 | 1,13 | 0,106120 |
| 7 | 2,494 | 2,971 | 0,361 | (2,224; 3,719) | -0,477 | -0,63 | -0,62 | 0,184023 |
| 8 | 3,046 | 2,155 | 0,361 | (1,407; 2,903) | 0,891 | 1,17 | 1,18 | 0,184023 |
| 9 | 0,481 | 1,778 | 0,359 | (1,035; 2,521) | -1,297 | -1,70 | -1,78 | 0,181719 |
| 10 | 1,653 | 0,962 | 0,359 | (0,219; 1,705) | 0,691 | 0,91 | 0,90 | 0,181719 |
| 11 | 1,111 | 2,022 | 0,284 | (1,435; 2,609) | -0,911 | -1,15 | -1,16 | 0,113336 |
| 12 | 0,921 | 1,206 | 0,284 | (0,619; 1,792) | -0,285 | -0,36 | -0,35 | 0,113336 |
| 13 | 3,098 | 2,447 | 0,357 | (1,709; 3,184) | 0,652 | 0,85 | 0,85 | 0,179186 |
| 14 | 0,759 | 1,630 | 0,357 | (0,892; 2,368) | -0,871 | -1,14 | -1,15 | 0,179186 |
| 15 | 2,012 | 2,423 | 0,277 | (1,851; 2,996) | -0,411 | -0,52 | -0,51 | 0,107803 |
| 16 | 1,460 | 1,607 | 0,277 | (1,035; 2,179) | -0,147 | -0,18 | -0,18 | 0,107803 |
| 17 | 4,254 | 2,840 | 0,381 | (2,051; 3,629) | 1,414 | 1,88 | 2,00 | 0,204905 |
| 18 | 0,505 | 1,208 | 0,381 | (0,419; 1,997) | -0,703 | -0,94 | -0,93 | 0,204905 |
| 19 | 1,743 | 1,943 | 0,168 | (1,596; 2,291) | -0,201 | -0,24 | -0,24 | 0,039704 |
| 20 | 1,868 | 2,001 | 0,171 | (1,647; 2,355) | -0,133 | -0,16 | -0,16 | 0,041165 |
| 21 | 2,226 | 1,432 | 0,338 | (0,732; 2,132) | 0,794 | 1,03 | 1,03 | 0,161116 |
| 22 | 2,665 | 2,678 | 0,418 | (1,812; 3,543) | -0,013 | -0,02 | -0,02 | 0,246534 |
| 23 | 2,006 | 2,521 | 0,439 | (1,614; 3,429) | -0,515 | -0,72 | -0,71 | 0,271099 |
| 24 | 1,921 | 1,398 | 0,444 | (0,479; 2,317) | 0,523 | 0,73 | 0,72 | 0,278014 |
| 25 | 1,170 | 2,024 | 0,165 | (1,683; 2,365) | -0,854 | -1,03 | -1,03 | 0,038239 |
| 26 | 3,564 | 2,024 | 0,165 | (1,683; 2,365) | 1,540 | 1,86 | 1,98 | 0,038239 |
| 27 | 1,063 | 2,024 | 0,165 | (1,683; 2,365) | -0,961 | -1,16 | -1,17 | 0,038239 |

| Obs | Cook’s D | DFITS |
| --- | --- | --- |
| 1 | 0,07 | 0,54944 |
| 2 | 0,00 | 0,08929 |
| 3 | 0,13 | -0,72513 |
| 4 | 0,01 | 0,21150 |
| 5 | 0,00 | -0,03725 |
| 6 | 0,04 | 0,39062 |
| 7 | 0,02 | -0,29365 |
| 8 | 0,08 | 0,56061 |
| 9 | 0,16 | -0,83900 |
| 10 | 0,05 | 0,42537 |
| 11 | 0,04 | -0,41361 |
| 12 | 0,00 | -0,12580 |
| 13 | 0,04 | 0,39635 |
| 14 | 0,07 | -0,53702 |
| 15 | 0,01 | -0,17669 |
| 16 | 0,00 | -0,06266 |
| 17 | 0,23 | 1,01571 |
| 18 | 0,06 | -0,47342 |
| 19 | 0,00 | -0,04836 |
| 20 | 0,00 | -0,03265 |
| 21 | 0,05 | 0,45134 |
| 22 | 0,00 | -0,00991 |
| 23 | 0,05 | -0,43224 |
| 24 | 0,05 | 0,44813 |
| 25 | 0,01 | -0,20634 |
| 26 | 0,03 | 0,39434 |
| 27 | 0,01 | -0,23382 |

## Forward Selection of Terms

α to enter = 0,25

## Coded Coefficients

| Term | Coef | SE Coef | 95% CI | T-Value | P-Value | VIF |
| --- | --- | --- | --- | --- | --- | --- |
| Constant | 1,815 | 0,165 | (1,475; 2,156) | 11,01 | 0,000 |  |
| Lac | -0,901 | 0,338 | (-1,597; -0,204) | -2,67 | 0,013 | 1,00 |
| HPMC\_HP | 0,587 | 0,335 | (-0,104; 1,277) | 1,75 | 0,092 | 1,00 |

## Model Summary

| S | R-sq | R-sq(adj) | PRESS | R-sq(pred) | AICc | BIC |
| --- | --- | --- | --- | --- | --- | --- |
| 0,826825 | 29,81% | 23,96% | 20,9962 | 10,18% | 72,99 | 76,36 |

## Analysis of Variance

| Source | DF | Seq SS | Contribution | Adj SS | Adj MS | F-Value | P-Value |
| --- | --- | --- | --- | --- | --- | --- | --- |
| Model | 2 | 6,968 | 29,81% | 6,968 | 3,4841 | 5,10 | 0,014 |
| Linear | 2 | 6,968 | 29,81% | 6,968 | 3,4841 | 5,10 | 0,014 |
| Lac | 1 | 4,867 | 20,82% | 4,867 | 4,8671 | 7,12 | 0,013 |
| HPMC\_HP | 1 | 2,101 | 8,99% | 2,101 | 2,1011 | 3,07 | 0,092 |
| Error | 24 | 16,407 | 70,19% | 16,407 | 0,6836 |  |  |
| Lack-of-Fit | 22 | 12,453 | 53,27% | 12,453 | 0,5660 | 0,29 | 0,952 |
| Pure Error | 2 | 3,955 | 16,92% | 3,955 | 1,9774 |  |  |
| Total | 26 | 23,376 | 100,00% |  |  |  |  |

## Regression Equation in Uncoded Units

|  |  |  |
| --- | --- | --- |
| F\_SD\_21h(1260min) | = | -1,86 - 3,60 Lac + 0,578 HPMC\_HP |

## Fits and Diagnostics for All Observations

| Obs | F\_SD\_21h(1260min) | Fit | SE Fit | 95% CI | Resid | Std Resid | Del Resid | HI |
| --- | --- | --- | --- | --- | --- | --- | --- | --- |
| 1 | 2,746 | 1,843 | 0,305 | (1,214; 2,472) | 0,903 | 1,18 | 1,18 | 0,135962 |
| 2 | 1,015 | 0,942 | 0,305 | (0,313; 1,571) | 0,072 | 0,09 | 0,09 | 0,135962 |
| 3 | 1,008 | 1,825 | 0,312 | (1,182; 2,468) | -0,817 | -1,07 | -1,07 | 0,142074 |
| 4 | 1,357 | 0,924 | 0,312 | (0,281; 1,567) | 0,433 | 0,57 | 0,56 | 0,142074 |
| 5 | 2,259 | 2,386 | 0,258 | (1,854; 2,918) | -0,127 | -0,16 | -0,16 | 0,097061 |
| 6 | 2,507 | 1,485 | 0,258 | (0,954; 2,017) | 1,022 | 1,30 | 1,32 | 0,097061 |
| 7 | 2,232 | 2,544 | 0,308 | (1,909; 3,179) | -0,312 | -0,41 | -0,40 | 0,138473 |
| 8 | 2,497 | 1,643 | 0,308 | (1,008; 2,278) | 0,853 | 1,11 | 1,12 | 0,138473 |
| 9 | 0,220 | 1,867 | 0,296 | (1,255; 2,478) | -1,647 | -2,13 | -2,32 | 0,128346 |
| 10 | 1,523 | 0,966 | 0,296 | (0,355; 1,577) | 0,557 | 0,72 | 0,71 | 0,128346 |
| 11 | 1,144 | 1,987 | 0,259 | (1,451; 2,522) | -0,842 | -1,07 | -1,08 | 0,098333 |
| 12 | 0,428 | 1,086 | 0,259 | (0,551; 1,621) | -0,658 | -0,84 | -0,83 | 0,098333 |
| 13 | 2,934 | 2,505 | 0,294 | (1,899; 3,111) | 0,429 | 0,55 | 0,55 | 0,126094 |
| 14 | 0,928 | 1,605 | 0,294 | (0,999; 2,211) | -0,676 | -0,87 | -0,87 | 0,126094 |
| 15 | 2,175 | 2,367 | 0,253 | (1,845; 2,889) | -0,192 | -0,24 | -0,24 | 0,093644 |
| 16 | 0,804 | 1,466 | 0,253 | (0,944; 1,988) | -0,662 | -0,84 | -0,84 | 0,093644 |
| 17 | 4,042 | 2,684 | 0,374 | (1,912; 3,456) | 1,358 | 1,84 | 1,95 | 0,204636 |
| 18 | 0,468 | 0,883 | 0,374 | (0,111; 1,655) | -0,415 | -0,56 | -0,55 | 0,204636 |
| 19 | 1,118 | 1,772 | 0,160 | (1,441; 2,103) | -0,654 | -0,81 | -0,80 | 0,037546 |
| 20 | 1,751 | 1,714 | 0,160 | (1,385; 2,044) | 0,037 | 0,05 | 0,04 | 0,037337 |
| 21 | 2,187 | 1,229 | 0,332 | (0,544; 1,914) | 0,958 | 1,27 | 1,28 | 0,161083 |
| 22 | 2,203 | 2,402 | 0,410 | (1,556; 3,248) | -0,199 | -0,28 | -0,27 | 0,245997 |
| 23 | 2,164 | 1,772 | 0,160 | (1,441; 2,103) | 0,392 | 0,48 | 0,47 | 0,037546 |
| 24 | 1,941 | 1,714 | 0,160 | (1,385; 2,044) | 0,227 | 0,28 | 0,27 | 0,037337 |
| 25 | 1,044 | 1,784 | 0,161 | (1,451; 2,116) | -0,739 | -0,91 | -0,91 | 0,037969 |
| 26 | 3,392 | 1,784 | 0,161 | (1,451; 2,116) | 1,608 | 1,98 | 2,12 | 0,037969 |
| 27 | 0,877 | 1,784 | 0,161 | (1,451; 2,116) | -0,907 | -1,12 | -1,12 | 0,037969 |

| Obs | Cook’s D | DFITS |  |
| --- | --- | --- | --- |
| 1 | 0,07 | 0,470037 |  |
| 2 | 0,00 | 0,036557 |  |
| 3 | 0,06 | -0,435267 |  |
| 4 | 0,02 | 0,226794 |  |
| 5 | 0,00 | -0,051812 |  |
| 6 | 0,06 | 0,432907 |  |
| 7 | 0,01 | -0,160354 |  |
| 8 | 0,07 | 0,448109 |  |
| 9 | 0,22 | -0,890463 | R |
| 10 | 0,03 | 0,273826 |  |
| 11 | 0,04 | -0,355411 |  |
| 12 | 0,03 | -0,275104 |  |
| 13 | 0,01 | 0,207481 |  |
| 14 | 0,04 | -0,330587 |  |
| 15 | 0,00 | -0,076942 |  |
| 16 | 0,02 | -0,268772 |  |
| 17 | 0,29 | 0,986773 |  |
| 18 | 0,03 | -0,281477 |  |
| 19 | 0,01 | -0,158134 |  |
| 20 | 0,00 | 0,008812 |  |
| 21 | 0,10 | 0,561974 |  |
| 22 | 0,01 | -0,155429 |  |
| 23 | 0,00 | 0,093779 |  |
| 24 | 0,00 | 0,053981 |  |
| 25 | 0,01 | -0,180416 |  |
| 26 | 0,05 | 0,421837 |  |
| 27 | 0,02 | -0,223283 |  |

R  Large residual

## Forward Selection of Terms

α to enter = 0,25

## Coded Coefficients

| Term | Coef | SE Coef | 95% CI | T-Value | P-Value | VIF |
| --- | --- | --- | --- | --- | --- | --- |
| Constant | 1,685 | 0,159 | (1,357; 2,013) | 10,62 | 0,000 |  |
| Lac | -0,930 | 0,341 | (-1,635; -0,224) | -2,73 | 0,012 | 1,10 |
| HPMC\_HP | 0,476 | 0,322 | (-0,189; 1,142) | 1,48 | 0,152 | 1,00 |
| Lac\*HPMC\_HP | -0,933 | 0,787 | (-2,562; 0,696) | -1,19 | 0,248 | 1,10 |

## Model Summary

| S | R-sq | R-sq(adj) | PRESS | R-sq(pred) | AICc | BIC |
| --- | --- | --- | --- | --- | --- | --- |
| 0,795284 | 29,80% | 20,65% | 19,7892 | 4,51% | 72,78 | 76,40 |

## Analysis of Variance

| Source | DF | Seq SS | Contribution | Adj SS | Adj MS | F-Value | P-Value |
| --- | --- | --- | --- | --- | --- | --- | --- |
| Model | 3 | 6,1766 | 29,80% | 6,1766 | 2,0589 | 3,26 | 0,040 |
| Linear | 2 | 5,2884 | 25,52% | 6,0897 | 3,0448 | 4,81 | 0,018 |
| Lac | 1 | 3,9023 | 18,83% | 4,7036 | 4,7036 | 7,44 | 0,012 |
| HPMC\_HP | 1 | 1,3861 | 6,69% | 1,3861 | 1,3861 | 2,19 | 0,152 |
| 2-Way Interaction | 1 | 0,8882 | 4,29% | 0,8882 | 0,8882 | 1,40 | 0,248 |
| Lac\*HPMC\_HP | 1 | 0,8882 | 4,29% | 0,8882 | 0,8882 | 1,40 | 0,248 |
| Error | 23 | 14,5470 | 70,20% | 14,5470 | 0,6325 |  |  |
| Lack-of-Fit | 21 | 11,0313 | 53,23% | 11,0313 | 0,5253 | 0,30 | 0,945 |
| Pure Error | 2 | 3,5157 | 16,96% | 3,5157 | 1,7578 |  |  |
| Total | 26 | 20,7235 | 100,00% |  |  |  |  |

## Regression Equation in Uncoded Units

|  |  |  |
| --- | --- | --- |
| F\_SD\_22h(1320min) | = | -18,3 + 31,1 Lac + 2,31 HPMC\_HP - 3,68 Lac\*HPMC\_HP |

## Fits and Diagnostics for All Observations

| Obs | F\_SD\_22h(1320min) | Fit | SE Fit | 95% CI | Resid | Std Resid | Del Resid | HI |
| --- | --- | --- | --- | --- | --- | --- | --- | --- |
| 1 | 2,285 | 1,470 | 0,374 | (0,697; 2,243) | 0,815 | 1,16 | 1,17 | 0,220950 |
| 2 | 0,989 | 1,213 | 0,374 | (0,440; 1,986) | -0,224 | -0,32 | -0,31 | 0,220950 |
| 3 | 0,982 | 1,441 | 0,386 | (0,641; 2,241) | -0,459 | -0,66 | -0,65 | 0,236172 |
| 4 | 1,133 | 1,213 | 0,386 | (0,413; 2,012) | -0,079 | -0,11 | -0,11 | 0,236172 |
| 5 | 2,072 | 2,343 | 0,281 | (1,762; 2,925) | -0,271 | -0,36 | -0,36 | 0,124941 |
| 6 | 2,392 | 1,222 | 0,281 | (0,641; 1,804) | 1,170 | 1,57 | 1,63 | 0,124941 |
| 7 | 2,087 | 2,597 | 0,380 | (1,811; 3,384) | -0,510 | -0,73 | -0,72 | 0,228657 |
| 8 | 1,987 | 1,225 | 0,380 | (0,438; 2,011) | 0,762 | 1,09 | 1,10 | 0,228657 |
| 9 | 0,128 | 1,508 | 0,357 | (0,769; 2,248) | -1,381 | -1,94 | -2,08 | 0,201983 |
| 10 | 1,836 | 1,213 | 0,357 | (0,474; 1,953) | 0,622 | 0,88 | 0,87 | 0,201983 |
| 11 | 1,131 | 1,701 | 0,284 | (1,114; 2,288) | -0,570 | -0,77 | -0,76 | 0,127296 |
| 12 | 0,507 | 1,215 | 0,284 | (0,628; 1,802) | -0,708 | -0,95 | -0,95 | 0,127296 |
| 13 | 2,784 | 2,535 | 0,354 | (1,803; 3,266) | 0,249 | 0,35 | 0,34 | 0,197671 |
| 14 | 1,004 | 1,224 | 0,354 | (0,493; 1,956) | -0,220 | -0,31 | -0,30 | 0,197671 |
| 15 | 2,151 | 2,312 | 0,271 | (1,751; 2,874) | -0,161 | -0,22 | -0,21 | 0,116368 |
| 16 | 0,788 | 1,222 | 0,271 | (0,661; 1,783) | -0,434 | -0,58 | -0,57 | 0,116368 |
| 17 | 3,830 | 2,538 | 0,365 | (1,783; 3,293) | 1,292 | 1,83 | 1,93 | 0,210585 |
| 18 | 0,484 | 0,780 | 0,365 | (0,025; 1,535) | -0,296 | -0,42 | -0,41 | 0,210585 |
| 19 | 0,663 | 1,650 | 0,154 | (1,331; 1,969) | -0,987 | -1,26 | -1,28 | 0,037546 |
| 20 | 1,577 | 1,603 | 0,154 | (1,285; 1,921) | -0,026 | -0,03 | -0,03 | 0,037337 |
| 21 | 2,163 | 1,209 | 0,319 | (0,548; 1,869) | 0,955 | 1,31 | 1,33 | 0,161083 |
| 22 | 1,869 | 2,161 | 0,394 | (1,345; 2,977) | -0,293 | -0,42 | -0,42 | 0,245997 |
| 23 | 2,373 | 1,650 | 0,154 | (1,331; 1,969) | 0,723 | 0,93 | 0,92 | 0,037546 |
| 24 | 1,911 | 1,603 | 0,154 | (1,285; 1,921) | 0,308 | 0,39 | 0,39 | 0,037337 |
| 25 | 0,917 | 1,659 | 0,155 | (1,339; 1,980) | -0,742 | -0,95 | -0,95 | 0,037969 |
| 26 | 3,092 | 1,659 | 0,155 | (1,339; 1,980) | 1,433 | 1,84 | 1,95 | 0,037969 |
| 27 | 0,691 | 1,659 | 0,155 | (1,339; 1,980) | -0,968 | -1,24 | -1,26 | 0,037969 |

| Obs | Cook’s D | DFITS |
| --- | --- | --- |
| 1 | 0,10 | 0,62352 |
| 2 | 0,01 | -0,16660 |
| 3 | 0,03 | -0,36249 |
| 4 | 0,00 | -0,06200 |
| 5 | 0,00 | -0,13492 |
| 6 | 0,09 | 0,61518 |
| 7 | 0,04 | -0,39332 |
| 8 | 0,09 | 0,59680 |
| 9 | 0,24 | -1,04604 |
| 10 | 0,05 | 0,43840 |
| 11 | 0,02 | -0,29033 |
| 12 | 0,03 | -0,36337 |
| 13 | 0,01 | 0,16993 |
| 14 | 0,01 | -0,15038 |
| 15 | 0,00 | -0,07659 |
| 16 | 0,01 | -0,20767 |
| 17 | 0,22 | 0,99888 |
| 18 | 0,01 | -0,21264 |
| 19 | 0,02 | -0,25324 |
| 20 | 0,00 | -0,00641 |
| 21 | 0,08 | 0,58403 |
| 22 | 0,01 | -0,23775 |
| 23 | 0,01 | 0,18243 |
| 24 | 0,00 | 0,07624 |
| 25 | 0,01 | -0,18852 |
| 26 | 0,03 | 0,38646 |
| 27 | 0,02 | -0,24962 |

## Forward Selection of Terms

α to enter = 0,25

## Coded Coefficients

| Term | Coef | SE Coef | 95% CI | T-Value | P-Value | VIF |
| --- | --- | --- | --- | --- | --- | --- |
| Constant | 1,565 | 0,147 | (1,263; 1,866) | 10,68 | 0,000 |  |
| Lac | -0,731 | 0,311 | (-1,371; -0,090) | -2,35 | 0,027 | 1,00 |

## Model Summary

| S | R-sq | R-sq(adj) | PRESS | R-sq(pred) | AICc | BIC |
| --- | --- | --- | --- | --- | --- | --- |
| 0,761327 | 18,10% | 14,82% | 17,2606 | 2,44% | 66,86 | 69,71 |

## Analysis of Variance

| Source | DF | Seq SS | Contribution | Adj SS | Adj MS | F-Value | P-Value |
| --- | --- | --- | --- | --- | --- | --- | --- |
| Model | 1 | 3,202 | 18,10% | 3,202 | 3,2024 | 5,53 | 0,027 |
| Linear | 1 | 3,202 | 18,10% | 3,202 | 3,2024 | 5,53 | 0,027 |
| Lac | 1 | 3,202 | 18,10% | 3,202 | 3,2024 | 5,53 | 0,027 |
| Error | 25 | 14,490 | 81,90% | 14,490 | 0,5796 |  |  |
| Lack-of-Fit | 23 | 11,598 | 65,55% | 11,598 | 0,5042 | 0,35 | 0,923 |
| Pure Error | 2 | 2,893 | 16,35% | 2,893 | 1,4464 |  |  |
| Total | 26 | 17,693 | 100,00% |  |  |  |  |

## Regression Equation in Uncoded Units

|  |  |  |
| --- | --- | --- |
| F\_SD\_23h(1380min) | = | 3,026 - 2,92 Lac |

## Fits and Diagnostics for All Observations

| Obs | F\_SD\_23h(1380min) | Fit | SE Fit | 95% CI | Resid | Std Resid | Del Resid | HI |
| --- | --- | --- | --- | --- | --- | --- | --- | --- |
| 1 | 2,044 | 1,930 | 0,214 | (1,490; 2,370) | 0,114 | 0,16 | 0,15 | 0,078704 |
| 2 | 0,982 | 1,199 | 0,214 | (0,759; 1,639) | -0,218 | -0,30 | -0,29 | 0,078704 |
| 3 | 1,279 | 1,930 | 0,214 | (1,490; 2,370) | -0,651 | -0,89 | -0,89 | 0,078704 |
| 4 | 1,058 | 1,199 | 0,214 | (0,759; 1,639) | -0,141 | -0,19 | -0,19 | 0,078704 |
| 5 | 2,050 | 1,930 | 0,214 | (1,490; 2,370) | 0,121 | 0,16 | 0,16 | 0,078704 |
| 6 | 2,357 | 1,199 | 0,214 | (0,759; 1,639) | 1,158 | 1,58 | 1,64 | 0,078704 |
| 7 | 2,012 | 1,930 | 0,214 | (1,490; 2,370) | 0,082 | 0,11 | 0,11 | 0,078704 |
| 8 | 1,412 | 1,199 | 0,214 | (0,759; 1,639) | 0,213 | 0,29 | 0,29 | 0,078704 |
| 9 | 0,238 | 1,930 | 0,214 | (1,490; 2,370) | -1,692 | -2,32 | -2,56 | 0,078704 |
| 10 | 2,053 | 1,199 | 0,214 | (0,759; 1,639) | 0,854 | 1,17 | 1,18 | 0,078704 |
| 11 | 1,179 | 1,930 | 0,214 | (1,490; 2,370) | -0,751 | -1,03 | -1,03 | 0,078704 |
| 12 | 0,755 | 1,199 | 0,214 | (0,759; 1,639) | -0,444 | -0,61 | -0,60 | 0,078704 |
| 13 | 2,569 | 1,930 | 0,214 | (1,490; 2,370) | 0,639 | 0,87 | 0,87 | 0,078704 |
| 14 | 1,035 | 1,199 | 0,214 | (0,759; 1,639) | -0,164 | -0,22 | -0,22 | 0,078704 |
| 15 | 1,866 | 1,930 | 0,214 | (1,490; 2,370) | -0,064 | -0,09 | -0,09 | 0,078704 |
| 16 | 1,138 | 1,199 | 0,214 | (0,759; 1,639) | -0,061 | -0,08 | -0,08 | 0,078704 |
| 17 | 3,641 | 2,295 | 0,344 | (1,587; 3,003) | 1,345 | 1,98 | 2,11 | 0,203704 |
| 18 | 0,480 | 0,834 | 0,344 | (0,126; 1,542) | -0,354 | -0,52 | -0,51 | 0,203704 |
| 19 | 0,418 | 1,565 | 0,147 | (1,263; 1,866) | -1,147 | -1,53 | -1,58 | 0,037037 |
| 20 | 1,423 | 1,565 | 0,147 | (1,263; 1,866) | -0,141 | -0,19 | -0,19 | 0,037037 |
| 21 | 2,115 | 1,565 | 0,147 | (1,263; 1,866) | 0,550 | 0,74 | 0,73 | 0,037037 |
| 22 | 1,593 | 1,565 | 0,147 | (1,263; 1,866) | 0,029 | 0,04 | 0,04 | 0,037037 |
| 23 | 2,596 | 1,565 | 0,147 | (1,263; 1,866) | 1,031 | 1,38 | 1,41 | 0,037037 |
| 24 | 1,904 | 1,565 | 0,147 | (1,263; 1,866) | 0,339 | 0,45 | 0,45 | 0,037037 |
| 25 | 0,766 | 1,565 | 0,147 | (1,263; 1,866) | -0,798 | -1,07 | -1,07 | 0,037037 |
| 26 | 2,732 | 1,565 | 0,147 | (1,263; 1,866) | 1,168 | 1,56 | 1,61 | 0,037037 |
| 27 | 0,549 | 1,565 | 0,147 | (1,263; 1,866) | -1,015 | -1,36 | -1,38 | 0,037037 |

| Obs | Cook’s D | DFITS |  |
| --- | --- | --- | --- |
| 1 | 0,00 | 0,04462 |  |
| 2 | 0,00 | -0,08539 |  |
| 3 | 0,03 | -0,25926 |  |
| 4 | 0,00 | -0,05534 |  |
| 5 | 0,00 | 0,04728 |  |
| 6 | 0,11 | 0,47840 |  |
| 7 | 0,00 | 0,03204 |  |
| 8 | 0,00 | 0,08342 |  |
| 9 | 0,23 | -0,74808 | R |
| 10 | 0,06 | 0,34407 |  |
| 11 | 0,05 | -0,30061 |  |
| 12 | 0,02 | -0,17541 |  |
| 13 | 0,03 | 0,25422 |  |
| 14 | 0,00 | -0,06442 |  |
| 15 | 0,00 | -0,02516 |  |
| 16 | 0,00 | -0,02409 |  |
| 17 | 0,50 | 1,06888 |  |
| 18 | 0,03 | -0,25944 |  |
| 19 | 0,05 | -0,30984 |  |
| 20 | 0,00 | -0,03639 |  |
| 21 | 0,01 | 0,14313 |  |
| 22 | 0,00 | 0,00742 |  |
| 23 | 0,04 | 0,27601 |  |
| 24 | 0,00 | 0,08756 |  |
| 25 | 0,02 | -0,21018 |  |
| 26 | 0,05 | 0,31619 |  |
| 27 | 0,04 | -0,27138 |  |

R  Large residual

## Forward Selection of Terms

α to enter = 0,25

## Coded Coefficients

| Term | Coef | SE Coef | 95% CI | T-Value | P-Value | VIF |
| --- | --- | --- | --- | --- | --- | --- |
| Constant | 1,505 | 0,142 | (1,212; 1,798) | 10,59 | 0,000 |  |
| Lac | -0,606 | 0,302 | (-1,226; 0,015) | -2,01 | 0,056 | 1,00 |

## Model Summary

| S | R-sq | R-sq(adj) | PRESS | R-sq(pred) | AICc | BIC |
| --- | --- | --- | --- | --- | --- | --- |
| 0,738535 | 13,89% | 10,45% | 16,2535 | 0,00% | 65,22 | 68,07 |

## Analysis of Variance

| Source | DF | Seq SS | Contribution | Adj SS | Adj MS | F-Value | P-Value |
| --- | --- | --- | --- | --- | --- | --- | --- |
| Model | 1 | 2,200 | 13,89% | 2,200 | 2,1998 | 4,03 | 0,056 |
| Linear | 1 | 2,200 | 13,89% | 2,200 | 2,1998 | 4,03 | 0,056 |
| Lac | 1 | 2,200 | 13,89% | 2,200 | 2,1998 | 4,03 | 0,056 |
| Error | 25 | 13,636 | 86,11% | 13,636 | 0,5454 |  |  |
| Lack-of-Fit | 23 | 11,529 | 72,81% | 11,529 | 0,5013 | 0,48 | 0,855 |
| Pure Error | 2 | 2,107 | 13,30% | 2,107 | 1,0533 |  |  |
| Total | 26 | 15,836 | 100,00% |  |  |  |  |

## Regression Equation in Uncoded Units

|  |  |  |
| --- | --- | --- |
| F\_SD\_24h(1440min) | = | 2,716 - 2,42 Lac |

## Fits and Diagnostics for All Observations

| Obs | F\_SD\_24h(1440min) | Fit | SE Fit | 95% CI | Resid | Std Resid | Del Resid | HI |
| --- | --- | --- | --- | --- | --- | --- | --- | --- |
| 1 | 1,825 | 1,808 | 0,207 | (1,381; 2,235) | 0,017 | 0,02 | 0,02 | 0,078704 |
| 2 | 1,026 | 1,202 | 0,207 | (0,776; 1,629) | -0,176 | -0,25 | -0,24 | 0,078704 |
| 3 | 1,494 | 1,808 | 0,207 | (1,381; 2,235) | -0,314 | -0,44 | -0,44 | 0,078704 |
| 4 | 1,025 | 1,202 | 0,207 | (0,776; 1,629) | -0,177 | -0,25 | -0,25 | 0,078704 |
| 5 | 1,906 | 1,808 | 0,207 | (1,381; 2,235) | 0,098 | 0,14 | 0,14 | 0,078704 |
| 6 | 2,367 | 1,202 | 0,207 | (0,776; 1,629) | 1,164 | 1,64 | 1,70 | 0,078704 |
| 7 | 1,833 | 1,808 | 0,207 | (1,381; 2,235) | 0,025 | 0,04 | 0,03 | 0,078704 |
| 8 | 0,989 | 1,202 | 0,207 | (0,776; 1,629) | -0,213 | -0,30 | -0,30 | 0,078704 |
| 9 | 0,346 | 1,808 | 0,207 | (1,381; 2,235) | -1,461 | -2,06 | -2,22 | 0,078704 |
| 10 | 2,241 | 1,202 | 0,207 | (0,776; 1,629) | 1,039 | 1,47 | 1,50 | 0,078704 |
| 11 | 1,214 | 1,808 | 0,207 | (1,381; 2,235) | -0,594 | -0,84 | -0,83 | 0,078704 |
| 12 | 0,987 | 1,202 | 0,207 | (0,776; 1,629) | -0,215 | -0,30 | -0,30 | 0,078704 |
| 13 | 2,374 | 1,808 | 0,207 | (1,381; 2,235) | 0,566 | 0,80 | 0,79 | 0,078704 |
| 14 | 1,133 | 1,202 | 0,207 | (0,776; 1,629) | -0,070 | -0,10 | -0,10 | 0,078704 |
| 15 | 1,566 | 1,808 | 0,207 | (1,381; 2,235) | -0,242 | -0,34 | -0,34 | 0,078704 |
| 16 | 1,464 | 1,202 | 0,207 | (0,776; 1,629) | 0,262 | 0,37 | 0,36 | 0,078704 |
| 17 | 3,454 | 2,111 | 0,333 | (1,424; 2,797) | 1,343 | 2,04 | 2,19 | 0,203704 |
| 18 | 0,483 | 0,900 | 0,333 | (0,213; 1,586) | -0,417 | -0,63 | -0,62 | 0,203704 |
| 19 | 0,254 | 1,505 | 0,142 | (1,212; 1,798) | -1,252 | -1,73 | -1,80 | 0,037037 |
| 20 | 1,178 | 1,505 | 0,142 | (1,212; 1,798) | -0,327 | -0,45 | -0,44 | 0,037037 |
| 21 | 2,065 | 1,505 | 0,142 | (1,212; 1,798) | 0,560 | 0,77 | 0,77 | 0,037037 |
| 22 | 1,415 | 1,505 | 0,142 | (1,212; 1,798) | -0,090 | -0,12 | -0,12 | 0,037037 |
| 23 | 2,669 | 1,505 | 0,142 | (1,212; 1,798) | 1,164 | 1,61 | 1,66 | 0,037037 |
| 24 | 1,911 | 1,505 | 0,142 | (1,212; 1,798) | 0,406 | 0,56 | 0,55 | 0,037037 |
| 25 | 0,649 | 1,505 | 0,142 | (1,212; 1,798) | -0,857 | -1,18 | -1,19 | 0,037037 |
| 26 | 2,320 | 1,505 | 0,142 | (1,212; 1,798) | 0,815 | 1,12 | 1,13 | 0,037037 |
| 27 | 0,453 | 1,505 | 0,142 | (1,212; 1,798) | -1,052 | -1,45 | -1,49 | 0,037037 |

| Obs | Cook’s D | DFITS |  |
| --- | --- | --- | --- |
| 1 | 0,00 | 0,00671 |  |
| 2 | 0,00 | -0,07134 |  |
| 3 | 0,01 | -0,12728 |  |
| 4 | 0,00 | -0,07174 |  |
| 5 | 0,00 | 0,03955 |  |
| 6 | 0,12 | 0,49794 |  |
| 7 | 0,00 | 0,01006 |  |
| 8 | 0,00 | -0,08625 |  |
| 9 | 0,18 | -0,64806 | R |
| 10 | 0,09 | 0,43901 |  |
| 11 | 0,03 | -0,24349 |  |
| 12 | 0,00 | -0,08718 |  |
| 13 | 0,03 | 0,23151 |  |
| 14 | 0,00 | -0,02822 |  |
| 15 | 0,00 | -0,09800 |  |
| 16 | 0,01 | 0,10605 |  |
| 17 | 0,53 | 1,10600 | R |
| 18 | 0,05 | -0,31573 |  |
| 19 | 0,06 | -0,35359 |  |
| 20 | 0,00 | -0,08710 |  |
| 21 | 0,01 | 0,15026 |  |
| 22 | 0,00 | -0,02390 |  |
| 23 | 0,05 | 0,32588 |  |
| 24 | 0,01 | 0,10819 |  |
| 25 | 0,03 | -0,23375 |  |
| 26 | 0,02 | 0,22180 |  |
| 27 | 0,04 | -0,29152 |  |

R  Large residual
